# Gene-expression patterns in peripheral blood classify familial breast cancer susceptibility

**DOI:** 10.1186/s12920-015-0145-6

**Published:** 2015-11-04

**Authors:** Stephen R. Piccolo, Irene L. Andrulis, Adam L. Cohen, Thomas Conner, Philip J. Moos, Avrum E. Spira, Saundra S. Buys, W. Evan Johnson, Andrea H. Bild

**Affiliations:** Department of Pharmacology and Toxicology, University of Utah, Salt Lake City, UT USA; Division of Computational Biomedicine, Boston University School of Medicine, Boston, MA USA; Department of Biology, Brigham Young University, Provo, UT USA; Lunenfeld-Tanenbaum Research Institute, Mount Sinai Hospital, Toronto, Ontario Canada; Huntsman Cancer Institute, Salt Lake City, UT USA; Department of Medicine, University of Utah, Salt Lake City, UT USA; Department of Oncological Sciences, University of Utah, Salt Lake City, UT USA

**Keywords:** Breast cancer, Disease risk, Biomarker

## Abstract

**Background:**

Women with a family history of breast cancer face considerable uncertainty about whether to pursue standard screening, intensive screening, or prophylactic surgery. Accurate and individualized risk-estimation approaches may help these women make more informed decisions. Although highly penetrant genetic variants have been associated with familial breast cancer (FBC) risk, many individuals do not carry these variants, and many carriers never develop breast cancer. Common risk variants have a relatively modest effect on risk and show limited potential for predicting FBC development. As an alternative, we hypothesized that additional genomic data types, such as gene-expression levels, which can reflect genetic and epigenetic variation, could contribute to classifying a person’s risk status. Specifically, we aimed to identify common patterns in gene-expression levels across individuals who develop FBC.

**Methods:**

We profiled peripheral blood mononuclear cells from women with a family history of breast cancer (with or without a germline BRCA1/2 variant) and from controls. We used the support vector machines algorithm to differentiate between patients who developed FBC and those who did not. Our study used two independent datasets, a training set of 124 women from Utah (USA) and an external validation (test) set from Ontario (Canada) of 73 women (197 total). We controlled for expression variation associated with clinical, demographic, and treatment variables as well as lymphocyte markers.

**Results:**

Our multigene biomarker provided accurate, individual-level estimates of FBC occurrence for the Utah cohort (AUC = 0.76 [0.67-84]) . Even at their lower confidence bounds, these accuracy estimates meet or exceed estimates from alternative approaches. Our Ontario cohort resulted in similarly high levels of accuracy (AUC = 0.73 [0.59-0.86]), thus providing external validation of our findings. Individuals deemed to have “high” risk by our model would have an estimated 2.4 times greater odds of developing familial breast cancer than individuals deemed to have “low” risk.

**Conclusions:**

Together, these findings suggest that gene-expression levels in peripheral blood cells reflect genomic variation associated with breast cancer risk and that such data have potential to be used as a non-invasive biomarker for familial breast cancer risk.

**Electronic supplementary material:**

The online version of this article (doi:10.1186/s12920-015-0145-6) contains supplementary material, which is available to authorized users.

## Background

Current clinical standards define a woman's breast cancer risk based on population averages. For individuals deemed to have a lifetime risk over 20 %, based primarily on family history, a strict surveillance regimen is recommended; this regimen typically includes twice-yearly clinical breast exams, yearly mammograms, and yearly breast MRI. For the five to ten percent of women who have a strong inherited predisposition to breast cancer [1], more aggressive prevention strategies—such as chemoprevention or prophylactic mastectomy/oophorectomy—may be recommended in addition to or instead of surveillance. However, many women with a family history of breast cancer, including many who carry *BRCA1* or *BRCA2* mutations, never develop breast cancer. Indeed, 40-50 % of women with a *BRCA1* or *BRCA2* mutation do not develop breast cancer by 70 years of age [2]. This situation leads to uncertainty for both patient and physician regarding whether to pursue these aggressive prevention strategies, which can cause health and lifestyle effects that many women consider to be severe. For example, side effects of chemoprevention can include osteoporosis, blood clots, endometrial cancer, hot flushes, joint pain, and depression [3, 4]. Thus there is a critical need to accurately differentiate individuals from high-risk families who will develop breast cancer from those who will not develop breast cancer. Screening and prevention resources could then be focused on those women who carry the highest risk for familial breast cancer (FBC), while women with a lower risk could be spared the risks and inconveniences of prophylaxis or intensive screening. Optimally, such an approach would be non-invasive and provide estimates of risk that are tailored to each individual.

One existing method for estimating breast-cancer risk is based on personal health history, family health history, and demographic variables [5]; however, this approach is designed as a population-wide screening tool—not specifically for individuals from high-risk families—and the predictive accuracy of this method is limited [6]. Others have examined the potential to predict breast-cancer risk based on genetic or epigenetic variation, and these approaches have improved prediction accuracy [7–9]. We evaluated an alternative approach, hypothesizing that by quantifying gene-expression activity in peripheral-blood cells, we would be able to identify patterns that indicate a woman’s risk for developing breast cancer, as gene-expression profiling of *normal* cells has previously provided information about disease development [10–13]. This approach aims to overcome limitations of using genetic variants to predict risk. For example, due to genetic heterogeneity, individuals who develop breast cancer differ considerably in the risk variants that they carry, and most such variants are believed to have a subtle effect on risk. Gene-expression levels reflect biological activity within cells and serve as proxies for genetic (and epigenetic) variation within normal cells as well as tumor cells [10, 14, 15]. Indeed, it has been shown that global expression levels in lymphoblastoid cells reflect a person’s *BRCA1* or *BRCA2* mutation status, even when the mutations lie at different genomic loci within these genes [16]. Aberrant expression resulting from *BRCA1* and *BRCA2* mutations may not be reflected phenotypically in peripheral-blood cells; however expression levels in these cells may indicate a propensity for normal cellular activity within breast cells to become disrupted. Gene-expression levels in breast tumors have been shown to reflect functional germline variation. For example, expression patterns in tumors from patients with germline *BRCA1* and *BRCA2* variants exhibit distinctive patterns compared to tumors from individuals who do not carry these mutations [14–26]. Accordingly, we hypothesized that gene-expression-levels in normal cells should be similar across many women who develop FBC and thus indicative of disease risk, even though the underlying genetic and epigenetic variation may vary considerably across these women.

For this study, we examined the potential to use peripheral-blood gene-expression levels to identify women who possess the highest risk for developing FBC. We obtained peripheral blood mononuclear cells (PBMCs) for two independent patient cohorts and evaluated how well this gene-expression data could be used to differentiate between women who have developed FBC and women who have not, independent of *BRCA1/BRCA2* mutation status. Our findings indicate that this approach has potential to provide women from breast cancer families with individualized estimates of breast cancer risk and therefore to guide patient decisions regarding medical management.

## Methods

### Description of patient cohorts and data sets and consent to publish

#### Utah

We recruited participants via the High Risk Breast Cancer Clinic at the Huntsman Cancer Institute (Utah, USA) under Institutional Review Board approved protocols (#00022886 and #00004965). We have obtained consent from these patients to report individual patient data. We collected blood samples after breast cancer occurrence and after participants had been in remission for at least six months. In general, we considered participants to have a family history of breast cancer if two or more first-degree relatives (mother, sister, daughter) had been diagnosed with breast cancer. Among eligible participants who met these criteria, we included all those from whom we could obtain fresh mononuclear cells at the time of the study. Among 83 individuals in the Utah cohort who had a family history of breast cancer, 39 had been diagnosed with breast cancer, whereas 44 women had never been diagnosed with breast cancer and were at least 55 years of age (Table 1). Of the participants with a breast cancer family history, 38 carried a known pathogenic mutation (identified via commercial testing) in *BRCA1* or *BRCA2*. We excluded women who carried a *BRCA1/BRCA2* mutation but lacked a family history of breast cancer. To ensure that our findings were specific to familial breast cancer, we recruited 41 individuals with no known family history of breast cancer; 22 of these women had developed sporadic (non-familial) breast cancer. We matched the patients by age. The median age of blood draw was consistent across all patient subgroups in the Utah cohort (Table 2). We used an analysis-of-variance test to ensure that the difference in age across the subgroups was not statistically significant (*p* = 0.064). In addition, because our goal was to demonstrate an ability to differentiate between individuals who developed familial breast cancer and individuals who did not, we also verified that there was not a significant difference in ages between these two groups (t-test *p*-value = 0.28).

**Table 1:** Summary of patient subgroups in the Utah and Ontario populations

Category	Utah	Ontario
Family history, BRCA1/2, Cancer	16	11
Family history, BRCAX, Cancer	23	17
Family history, BRCA1/2, No Cancer	18	14
Family history, BRCAX, No Cancer	26	18
No family history, Cancer	22	8
No family history, No Cancer	19	5
Total	124	73

Patients fell into one of six groups, depending on whether 1) they had a family history of breast cancer, 2) had been diagnosed with breast cancer previously, or 3) were known to carry a pathogenic variant in *BRCA1* or *BRCA2*. The number of patients in each group is listed for each cohort

**Table 2:** Summary of ages at which blood samples were acquired

Description	Minimum	Median	Maximum
Family history, BRCA1/2, Cancer	45	59.0	77
Family history, BRCAX, Cancer	56	58.5	78
Family history, BRCA1/2, No Cancer	46	60.0	78
Family history, BRCAX, No Cancer	55	63.0	83
No family history, Cancer	53	65.5	79
No family history, No Cancer	51	58.0	86

For participants within each group, this table indicates the minimum, average, and maximum age at which blood samples were drawn. These data represent 117 participants from the Utah cohort. The remaining 8 Utah participants were at least 55 years old; however, it was not feasible to collect their exact ages in retrospect. The median age at which blood samples were acquired was consistent across the groups (*p* = 0.064)

#### Ontario

We obtained de-identified samples via the Ontario, Canada site of the Breast Cancer Family Registry (BCFR). We also obtained consent from these patients to report individual patient data. This cohort included 28 samples from women with a family history of breast cancer who had developed breast cancer, 32 from women who had a family history of breast cancer but had not developed breast cancer, and 13 from women with no family history of breast cancer, 8 of whom had developed breast cancer. Of these 73 women, approximately half were post-menopausal. We obtained, isolated, and stored blood samples for these women when they first enrolled in the BCFR.

### Genomic data acquisition and deposition

We used the RNeasy Kit (Qiagen, Hilden, Germany) to isolate PBMCs from whole blood in cell-preparation tubes following manufacturer (Becton-Dickinson) protocol. Within two hours, we extracted RNA using the RiboPure RNA Isolation Kit, and hybridized the RNA to Affymetrix Genechip Human Exon 1.0 ST microarrays. Hybridization and scanning were performed at Duke University (for Utah samples and for half of the Ontario samples) and at Boston University School of Medicine (for the remaining Ontario samples).

### Microarray data normalization

To correct for array- and probe-level background noise, we applied our Single-channel Array Normalization algorithm [27] to the Affymetrix microarray data. We subsequently used the *PLANdbAffy* database [28] to limit our analysis to microarray probes that were classified as high quality ("green") and that did not map to a single-nucleotide variant. For each microarray, we summarized the remaining 2,201,005 probes into gene-level values using a 10 % trimmed mean. In addition, we excluded genes that contained fewer than five probes.

Because the microarrays had been processed at different facilities and at different times, we used the *ComBat* tool [29] to adjust for confounding effects that might arise due to these differences. We used cohort and processing facility to define the batches. We applied *ComBat* two separate times: 1) to the Utah samples and Ontario samples that were processed at Duke University and 2) to the same Utah samples (processed at Duke University) and to the Ontario samples that were processed at Boston University. Strong batch effects existed before adjustment, but after *ComBat* adjustment, no clear pattern remained between batches and gene-expression levels (Additional file 1: Figure S1). We have deposited the raw and processed data files in Gene Expression Omnibus (GSE47862).

### Gene-expression data filtering

To identify blood markers that could influence mRNA expression but that may cause confounding effects, we used a total lymphocyte enumeration test to evaluate the blood cells. This test provided total counts of CD4-positive T cells, CD8-positive T-cells, CD3-positive T-cells, B-cells and NK-cells. These counts were available for 22 samples from the Utah cohort. Furthermore, we obtained epidemiological and demographic data via a health-assessment survey for 63 patients from the Utah cohort. The health survey variables we collected were age, education level, marital status, religious preference, health status, physical activity, age at menarche, contraceptive use, total number of pregnancies, total number of live births, age at first live birth, age at last live birth, breastfeeding status, time since last menstrual period, age of menopause, chemopreventive drug use (selective estrogen receptor modulators), alcohol use, tobacco use, occupational history, immunological disorder history, hypertension drug use, and anti-inflammatory drug use. Additional files 2, 3 and 4 list these values. Additional file 5 indicates cancer, family-history, and BRCA1/2 status for these patients. Using a multifactor analysis of covariance model, we excluded genes whose expression patterns correlated with any of these variables at a 0.01 significance level. Additional file 6 indicates which genes were excluded.

### Evaluation of normal breast cell expression

We downloaded data from Gene Expression Omnibus (GSE17072), which had been produced by Lim, et al. [30]. These data contained gene-expression levels for normal breast cells—acquired via prophylactic mastectomy or reduction mammoplasty—for women who had a strong family history of breast cancer and for controls, respectively. Then using the top 250 genes that were expressed more highly (according to average fold change rank) in our PBMC cells from women who had developed FBC, we used the Gene Set Enrichment Analysis analytical technique [31] to assess whether these genes were up-regulated in the Lim, et al. samples. For this tool, we used default settings, except that we did not collapse genes, and we used gene-based permutation to estimate significance.

### Software

To identify genes whose expression differed most between FBC patients and controls, we used the Support Vector Machines-Recursive Feature Elimination (SVM-RFE) algorithm. We used the *SVMAttributeEval* module within the *Weka* software package [32] and configured the algorithm to remove 10 % of genes in each iteration.

We used the Support Vector Machines (SVM) classification algorithm to predict whether each participant had or had not developed FBC. To facilitate this analysis, we used the *e1071* R package (http://cran.r-project.org/package=e1071) and the *LIBSVM* library [33]. We used the radial-basis-function SVM kernel and tuned the “C” parameter via nested cross validation. Additionally, we used the ML-Flex software package [34] to execute the analysis on a high-performance computing cluster.

To plot receiver operating characteristic curves, we used the *ROCR* package [35]. We used a bootstrapping procedure (10,000 iterations) to calculate 95 % confidence intervals [36].

Software scripts used for this study are available from https://github.com/srp33/BCSP.

## Results

### Multigene predictions perform well for both a Utah cohort and an external validation cohort from Ontario

**Fig. 1:**
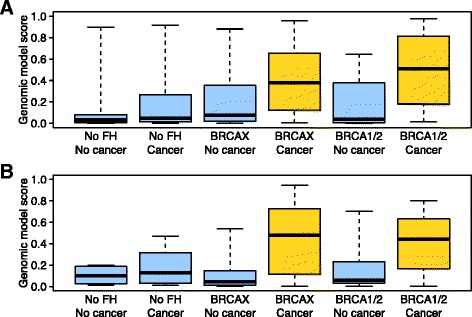
Predictions of familial breast cancer status in two independent cohorts. **a** In a cross-validated design, we predicted familial breast cancer status for 124 women from Utah. This cohort included women who did or did not have a family history (FH) of breast cancer, who did or did not carry a *BRCA1* or *BRCA2* mutation (*BRCAX* if not), and who had or had not developed breast cancer. The “Genomic model score” values represent probabilistic predictions made by the support vector machines algorithm. Higher values indicate a higher probability that a given individual had developed familial breast cancer. These scores were much higher for individuals who had a family history of breast cancer and developed a breast tumor, irrespective of *BRCA1/BRCA2* mutation status. **b** In a training/testing design, we predicted whether individuals in the independent Ontario cohort had developed familial breast cancer. The support vector machines algorithm was trained on the full Utah data set. Again, the scores were considerably higher for women with a family history of breast cancer who had developed a breast tumor

**Fig. 2:**
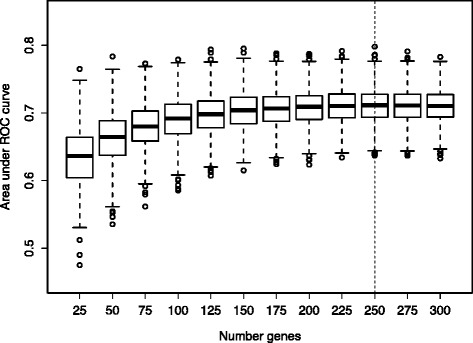
Cross-validation performance of gene-expression biomarker with different quantities of genes. For the gene-expression biomarker, we used the SVM-RFE method to identify genes whose expression differed most consistently between individuals who developed familial breast cancer and individuals who did not. The sizes of these gene subsets ranged in size between 25 and 300 genes. In repeated cross-validation (1,000 iterations), predictive accuracy peaked at 250 genes and was consistent when the number of genes was 150 or higher

We filtered the genome-wide PBMC gene-expression data by identifying genes whose expression best differentiated individuals who developed FBC from controls (see Methods). Controls were of three types: individuals with a family history of breast cancer who themselves did not develop breast cancer by age 55 or greater; individuals with no family history of breast cancer who also did not develop breast cancer; and individuals with no family history of breast cancer who did develop (sporadic) breast cancer. We then used expression values for those genes to predict FBC status for each individual using the SVM algorithm [37]. Two cohorts of samples were used for this study: a cohort from Utah and an independent cohort from Ontario; both included high-risk unaffected and affected women (see Methods for cohort details). Initially, we evaluated this approach in the Utah cohort via ten-fold cross validation. Our gene expression-based estimates of FBC development were consistently higher for women from FBC families who had developed cancer than for any subset of controls (Fig. 1a), attaining an AUC value of 0.76 (95 % CI = 0.67-0.85). Similar levels of accuracy were attained for women who carried a BRCA1/2 mutation as for women with a family history of breast cancer but with no known BRCA1/2 mutation (termed BRCAX) (Fig. 1a; Additional file 1: Table S1). Even at the lower confidence bounds, these AUC values are competitive with results observed in previous studies that used alternative approaches [7–9]. To further evaluate this result, we randomly permuted the class labels and observed that the biomarker’s accuracy was highly significant (*p* = 0.001). We also repeated cross-validation 1,000 times on the Utah data and observed that on average the best prediction results were attained using 250 genes; however accuracy was consistently high, independent of gene number (Fig. 2).

**Fig. 3:**
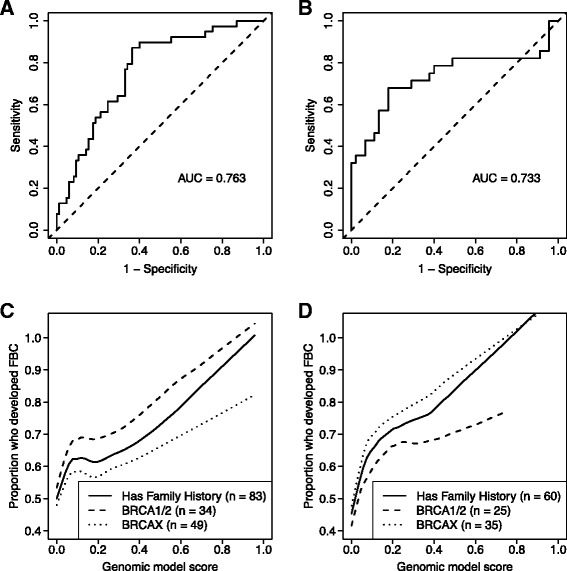
Sensitivity and specificity of biomarker predictions. Because the support vector machines predictions (genomic model score) are probabilistic, we evaluated various cutoff thresholds at which patients could be considered to have had a “high” probability of developing familial breast cancer. **a**-**b** Receiver operating characteristic curves illustrate the balance between sensitivity and specificity across many probability thresholds for the Utah and Ontario cohorts. **c**-**d** As the genomic model scores increase, a larger proportion of patients who fell above the threshold would have been predicted accurately to develop familial breast cancer. As the threshold approaches its maximum, the predictive accuracy for patients above the threshold is nearly perfect; however, such high thresholds would result in low sensitivity levels. A threshold near 0.2 may be optimal. Panel C represents predictions for Utah participants who had a family history of breast cancer; Panel D represents the Ontario cohort. The dashed lines represent predictions for individuals who carried a *BRCA1* or *BRCA2* mutation. The dotted lines represent predictions for BRCAX individuals. (Plotted lines were fitted using a LOESS model [span = 0.5] for smoothing)

To test whether this biomarker approach could be applied more generally via external validation, we derived an SVM model from the full Utah data set alone, and then used this model to predict FBC development in the external and independent Ontario data set. In accordance with Institute of Medicine recommendations [38], model derivation was performed solely on the Utah data before it was tested on the Ontario samples. These predictions attained an AUC of 0.73 (95 % CI = 0.59-0.86; permutation *p*-value = 0.002), showing a consistently high level of accuracy between the cohorts (Figs. 1 and 3; Additional file 1: Table S2).

### Risk prediction accuracy is independent of treatment effect

Blood samples for these patients were collected retrospectively—at least six months after treatment had been administered to individuals who developed breast cancer. To alleviate the concern that gene-expression changes in women who developed FBC were caused by lingering treatment effects, we also collected PBMC samples for women who had developed sporadic (non-familial) breast cancer and had received treatment. In our initial analysis, we grouped these women with the participants who had no history of breast cancer and broadly classified this group as “controls”. To further assess whether the predictive gene-expression patterns we identified are specific to women who develop FBC and thus have potential to predict disease risk, we assessed how well the SVM algorithm could distinguish between individuals who developed FBC and those who developed a sporadic tumor. This comparison was identical to the initial assessment, except that the control group excluded individuals who did not develop breast cancer. In this setting, the predictions attained similar levels of accuracy (Utah AUC = 0.77 [0.64-0.88]; Ontario AUC = 0.69 [0.49-0.89]) as the initial analysis, although the confidence intervals were wider due to the smaller sample sizes. These findings indicate that PBMC gene-expression patterns may be useful to predict FBC risk.

In addition, we tested whether similar genes were dysregulated in our predictive model if the sporadics-only control group was included or not. The SVM-RFE algorithm ranks each gene according to how strongly the gene-expression values differ between the patient groups. We found that the rankings were highly similar (Spearman's rank correlation rho statistic = 0.43), whether the control group contained sporadic patients only or the full control set. This finding suggests that individuals who develop familial breast cancer exhibit different gene-expression patterns than individuals who do not develop familial breast cancer, even when compared solely against individuals who had received prior diagnosis/treatments.

To affirm that the expression differences in the FBC women were not confounded by treatment with estrogen receptor pathway inhibitors, we independently evaluated a publicly available data set that profiled PBMC gene-expression levels for post-menopausal women who had or had not been treated with tamoxifen or aromatase inhibitors, respectively (http://www.ncbi.nlm.nih.gov/geo/query/acc.cgi?acc=GSE12517). We applied the Support Vector Machines algorithm to rank the genes according to differences in expression between women who had received a given treatment and post-menopausal women who had not received either treatment. We compared these gene rankings from this study to the original analysis that compared women who had developed familial breast cancer against controls. The gene rankings were not correlated for either treatment (Spearman’s rho for taxomifen = 0.030, aromatase inhibitors = 0.029). This result suggests that the genes perturbed by hormone treatments are different from those that discriminate between women who develop familial breast cancer and those who do not.

### Evaluating the balance between sensitivity and specificity of predictions

The SVM prediction scores are probabilistic values ranging between zero and one. Higher values suggest a relatively high risk of familial breast cancer, and lower values suggest a relatively low risk. In clinical settings, it is often desirable to identify a single cutoff threshold above which individuals are considered to have “high” risk. We used receiver operating characteristic curves to confirm that the sensitivity and specificity of our predictions remain strong across a broad range of thresholds (Fig. 3a-b). Then to identify a single threshold that may be best in clinical settings, we plotted the proportion of patients who were predicted to have “high” risk and who actually developed FBC, against a range of thresholds (Fig. 3c-d). As expected, this value increased as the threshold increased. Accordingly, at higher thresholds, a large proportion of patients predicted to have “high” risk would received accurate predictions. However, a tradeoff would be lower sensitivity (fewer individuals who actually developed FBC would be predicted to carry a high risk). Visual inspection of Fig. 3c-d suggests that a cutoff threshold near 0.2 may be optimal because the slope begins to level off (or drop temporarily). If a threshold of 0.2 were used to identify individuals at the highest risk of breast cancer in the Ontario cohort, the sensitivity would be 0.68 and the specificity would be 0.71, equating to a positive likelihood ratio of 2.35 and a negative likelihood ratio of 0.45. Put another way, for a woman with a 50 % chance of breast cancer based on family history and BRCA status, a prediction greater than 0.2 would suggest a 70 % chance of developing breast cancer, and a score less than 0.2 would indicate a 31 % chance of developing breast cancer.

### Biological interpretation

Interestingly, many genes—for example, *DSC1, FN1, ST6GALNAC5, TP63, SHB*, and *WNT3*—used in the above biomarker are known to play important roles in regulating cell–cell adhesion and cell–ECM interactions (see Additional files 7 and 8 for complete lists). To evaluate these genes at the biological pathway level, we applied the GATHER algorithm [39] to the 250 genes that best distinguished affected FBC women from controls in the Utah and Ontario data (Additional files 9 and 10); this approach indicated a significant association between FBC development and pathways that play a role in cell adhesion, including KEGG *Adherens Junctions* and E*xtracellular Matrix-receptor Interaction* (*p*-values < 0.05, Table 3). This finding suggests that these pathways may be fundamentally dysregulated in multiple cell types, potentially including asymptomatic breast tissue, which may be a mechanism that leads to increased risk of familial breast cancer. To assess whether the gene-expression patterns associated with FBC status in our PBMC samples also occur in normal breast cells, we examined publicly available data from Lim et al. [30] and found that patients with a strong family history of breast cancer have significantly higher overall expression (p = 0.001, see Methods) for genes that were overexpressed in our FBC samples.

**Table 3:** Top pathway results from GATHER analysis

Term ID	Term	*p*-value
hsa04520	Adherens junction	0.00149
hsa00590	Prostaglandin and leukotriene metabolism	0.00775
hsa04350	TGF-beta signaling pathway	0.0132
hsa04510	Focal adhesion	0.014

For genes that exhibited consistent fold-change directions in the Utah and Ontario gene-expression data (Additional files 7 and 8), we sorted the genes by average rank of fold change and t-test *p*-values. The 250 top-ranked genes were used to query GATHER [39] for KEGG pathways most strongly associated with this gene list. Pathways that attained a *p*-value less than 0.05 are shown

## Discussion

Women from FBC families face greater uncertainty regarding their personal risk of breast cancer than the general population [40]. Accurate risk prediction is important in part because 54 % of high-risk women fail to follow appropriate screening procedures for breast-cancer prevention, even when they have health insurance, receive reminders, and have a positive attitude toward screening [41]; however, increased *perceived* risk translates into increased willingness to consider effective prevention strategies such as tamoxifen [42]. Various risk-prediction models based on clinical and/or genomic data have been proposed, yet the discriminatory accuracy of these models has been modest (AUC 0.56-0.70) [6–9, 43]. Multiple studies have shown that higher accuracy levels can be obtained using gene-expression profiles of peripheral blood cells in the context of early detection [44, 45]. However, these studies have focused on general breast-cancer risk, and their approaches were tested in single cohorts. Our goal was to develop a classification approach specific to women from high-risk families—based on PBMC gene expression—and to validate this approach in an external cohort consisting of women from a different geographical location.

Researchers have placed much emphasis on identifying additional susceptibility variants [46]; however, known susceptibility variants account for at most ~30 % of familial breast cancer risk [47], and common variants currently show only moderate predictive capabilities for risk [8]. Here, we identify expression-based changes reflective of a person's risk to develop breast cancer. We emphasize the importance of additional, prospective studies with larger sample sizes to further evaluate the clinical potential of our approach (and alternative approaches); however, the confidence intervals for our results demonstrate that our sample size was large enough to obtain statistically meaningful results. Furthermore, previous studies that have used transcriptomic predictors for prognostic and diagnostic purposes have been deemed informative using similar or smaller cohort sizes [44, 45, 48].

We propose that additional approaches could be used to inform women about their personal breast cancer risk. In this study, we identified multigene expression patterns in peripheral blood cells that differ between individuals who have developed familial breast cancer and those who have not. Importantly, the peripheral-blood expression patterns were predictive of familial breast cancer, independent of *BRCA1/BRCA2* mutation status. In addition, our approach distinguished between individuals who developed familial breast cancer and those who developed sporadic breast cancer, suggesting that our approach’s predictive ability was not the result of prior cancer or its treatment and that the gene-expression patterns may be driven by inherited risk factors common to many women from high-risk families. [49]. Additional studies are critical to prospectively evaluate the risk-predictive utility of our approach in different clinical settings.

## Conclusion

Our approach has the possibility to alter how women with a family history of breast cancer make decisions regarding their health. Indeed, the risk-estimation approach we present here has the ability to provide a risk assessment for each individual woman. For example, each woman within a given family or multiple women who carry a known susceptibility variant could be assigned different individual risks based on their gene-expression profile, leading to more personalized prevention decisions. These risk assessments could provide reassurance for women who are not as highly predisposed and thus may opt for monitoring and/or chemoprevention rather than prophylactic mastectomy. Alternatively, a high predicted risk could provide evidence to support prophylactic surgeries or chemopreventive intervention.

Further studies will be needed to develop multi-data risk models that incorporate gene-expression based models with other informative data such as family history, clinical and demographic characteristics, and germline variants. Additionally, it will be helpful in the future to evaluate whether our findings generalize to women who have only one known first-degree relative with breast cancer (in this study, we focused on women with multiple affected first-degree relatives). However, the accuracy of our results indicates that gene expression based biomarkers hold promise for assessing individual breast cancer risk in a minimally invasive manner and that they can be applied broadly to women from high-risk families.

### Ethical approval

The institutional/ethical review boards at the University of Utah and Mount Sinai Hospital approved collection and dissemination of data for this study.

## Additional files

Additional file 1:
**Supplementary Tables and figures.** (DOC 313 kb) Additional file 2:
**Summarized clinical, demographic, and treatment data.** Summary of clinical, demographic, and prior treatment data for 61 individuals who responded to the health-assessment survey. (PDF 36 kb) Additional file 3:
**Raw clinical, demographic, and treatment data.** Raw clinical, demographic, and prior treatment data for 61 individuals who responded to the health-assessment survey. (PDF 50 kb) Additional file 4:
**Descriptions of variables used in health-assessment survey.** Descriptions of variables that were used in the health-assessment survey. (PDF 41 kb) Additional file 5:
**Cancer, family-history, and BRCA1/2 status for all patients.** This file indicates cancer, family history, and BRCA1/2 status for patients in the Utah and Ontario cohorts. (PDF 51 kb) Additional file 6:
**Genes filtered based on correlation with potential confounders.** Genes filtered based on association between gene-expression levels and clinical, demographic, prior treatment, or lymphocyte enumeration data. (PDF 82 kb) Additional file 7:
**Genes selected for the first half of the Ontario cohort.** Genes selected via SVM-RFE from the Utah cohort for the Ontario (36 samples) biomarker predictions. (PDF 53 kb) Additional file 8:
**Genes selected for the second half of the Ontario cohort.** Genes selected via SVM-RFE from the Utah cohort for the Ontario (remaining samples) biomarker predictions. (PDF 67 kb) Additional file 9:
**Gene-level summary of expression data for Utah and Ontario cohorts.** Fold change values represent the ratio of average gene expression for FBC individuals who developed cancer relative to expression levels for those who did not. (PDF 4554 kb) Additional file 10:
**Summary of differentially expressed genes.** Genes for which the average expression was consistently either higher or lower in FBC individuals relative to controls for the Utah and Ontario cohorts. (PDF 987 kb)

## Abbreviations

MRI: Magnetic resonance imaging
FBC: Familial breast cancer
PMBC: Peripheral blood mononuclear cells
BCFR: Breast Cancer Family Registry
SVM-RFE: Support Vector Machines-Recursive Feature Elimination
SVM: Support Vector Machines
AUC: Area under receiver operating characteristic curve
KEGG: Kyoto Encyclopedia of Genes and Genomes
