# Supplementary material for: Gene-expression patterns in peripheral blood classify familial breast cancer susceptibility
Source: BMC Med Genomics. 2015 Nov 4;8:72. doi: 10.1186/s12920-015-0145-6 (PMC4634735; doi:10.1186/s12920-015-0145-6)
Supplement: Additional file 1: — Supplementary Tables and figures. (DOC 313 kb) [file 12920_2015_145_MOESM1_ESM.doc]

**Gene-expression patterns in peripheral blood**

**classify familial breast cancer susceptibility**

Supplementary Materials

Supplementary Tables

**Table S1: Comparisons of gene-expression biomarker predictions among Utah patient subgroups**. We used an analysis-of-variance test to compare the support vector machines predictions across the patient subgroups (n = 124 patients) in the Utah cohort. To correct for multiple comparisons, we used Tukey’s “Honest Significant Difference” method. Asterisks indicate which comparisons resulted in significant (p < 0.05) differences.

| **Comparison Group 1** | **Comparison Group 2** | **Adjusted p-value** |
| --- | --- | --- |
| BRCA1/2, no cancer | BRCA1/2, cancer | 0.007* |
| BRCAX, cancer | BRCA1/2, cancer | 0.81 |
| BRCAX, no cancer | BRCA1/2, cancer | 0.03* |
| No family history, cancer | BRCA1/2, cancer | 0.01* |
| No family history, no cancer | BRCA1/2, cancer | 0.004* |
| BRCAX, cancer | BRCA1/2, no cancer | 0.11 |
| BRCAX, no cancer | BRCA1/2, no cancer | 0.96 |
| No family history, cancer | BRCA1/2, no cancer | 0.99 |
| No family history, no cancer | BRCA1/2, no cancer | 0.99 |
| BRCAX, no cancer | BRCAX, cancer | 0.39 |
| No family history, cancer | BRCAX, cancer | 0.20 |
| No family history, no cancer | BRCAX, cancer | 0.08 |
| No family history, cancer | BRCAX, no cancer | 0.99 |
| No family history, no cancer | BRCAX, no cancer | 0.92 |
| No family history, no cancer | No family history, cancer | 0.99 |

**Table S2: Comparisons of gene-expression biomarker predictions among Ontario patient subgroups**. We used an analysis-of-variance test to compare the support vector machines predictions across the patient subgroups (n = 73 patients) in the Ontario cohort. To correct for multiple comparisons, we used Tukey’s “Honest Significant Difference” method. Asterisks indicate which comparisons resulted in significant (p < 0.05) differences.

| **Comparison Group 1** | **Comparison Group 2** | **Adjusted p-value** |
| --- | --- | --- |
| BRCA1/2, no cancer | BRCA1/2, cancer | 0.32 |
| BRCAX, cancer | BRCA1/2, cancer | 0.99 |
| BRCAX, no cancer | BRCA1/2, cancer | 0.06 |
| No family history, cancer | BRCA1/2, cancer | 0.44 |
| No family history, no cancer | BRCA1/2, cancer | 0.28 |
| BRCAX, cancer | BRCA1/2, no cancer | 0.046* |
| BRCAX, no cancer | BRCA1/2, no cancer | 0.98 |
| No family history, cancer | BRCA1/2, no cancer | 0.99 |
| No family history, no cancer | BRCA1/2, no cancer | 0.99 |
| BRCAX, no cancer | BRCAX, cancer | 0.003* |
| No family history, cancer | BRCAX, cancer | 0.12 |
| No family history, no cancer | BRCAX, cancer | 0.08 |
| No family history, cancer | BRCAX, no cancer | 0.99 |
| No family history, no cancer | BRCAX, no cancer | 0.99 |
| No family history, no cancer | No family history, cancer | 0.99 |

Supplementary Figures

#### Figure S1: Principal component analysis shows clear need for batch adjustment. Microarray samples were processed at two different facilities, so we adjusted for batch effects (see Methods). A-B) The first two principal components of the gene-expression values before and after batch adjustment for Utah and Ontario samples processed (separately) at Duke University. C-D) The first two principal components of the gene-expression values before and after batch adjustment for Utah samples processed at Duke University and Ontario samples processed at Boston University.

#### 
