# Supplementary material for: Gene-expression patterns in peripheral blood classify familial breast cancer susceptibility
Source: BMC Med Genomics. 2015 Nov 4;8:72. doi: 10.1186/s12920-015-0145-6 (PMC4634735; doi:10.1186/s12920-015-0145-6)
Supplement: Additional file 2: — Summarized clinical, demographic, and treatment data. Summary of clinical, demographic, and prior treatment data for 61 individuals who responded to the health-assessment survey. (PDF 36 kb) [file 12920_2015_145_MOESM2_ESM.pdf]

Health Survey Variable	Min	Max	Average	Counts
Age	35	76	56.65	
Edu				2=2; 3=14; 4=4; 5=20; 6=18; 7=5
Marital				1=1; 2=53; 3=0; 4=5; 5=4
RelPref				1=6; 5=4; 6=2; 34=1; 37=49; 70=1
Health				1=15; 2=25; 3=19; 4=3; 5=1
Physical				1=3; 2=38; 3=14; 4=6; 5=2
MenstrAge	9	16	12.67	
Contr				1=45; 2=18
ContrAge1st	17	48	24.38	
Pregnant				1=62; 2=1
PregnantNo	1	10	4.44	
TtlLiveBirth	0	7	3.73	
FirstBirthAge	18	35	23.33	
LastBirthAge	18	40	31.61	
BrFeed				1=43; 2=18
UnableChild				1=44; 2=18; 9=1
Period				1=7; 2=3; 3=1; 4=52
PeriodStop				1=48; 2=15
PeriodAge	29	58	45.25	
Tamoxifen				1=11; 2=52
Alcohol				1=13; 2=50
CigSmoke				1=9; 2=54
Employment				1=18; 2=14; 3=31
Polyps				1=10; 2=23
ImmunoDisorder				1=25; 2=38
Hypertension				1=16; 2=47
AntiInfDrug				1=24; 2=39

This table summarizes health-survey responses that were provided by research participants from the Utah cohort. The first column indicates the data points that were collected. The Min and Max columns indicate the minimum and maximum values across all participants. The Average column indicates the average value across all responses. For discrete variables, the Counts column indicates the number of people who provided each response. Descriptions of these variables are provided in Additional file 3.
