# Supplementary material for: Gene-expression patterns in peripheral blood classify familial breast cancer susceptibility
Source: BMC Med Genomics. 2015 Nov 4;8:72. doi: 10.1186/s12920-015-0145-6 (PMC4634735; doi:10.1186/s12920-015-0145-6)
Supplement: Additional file 3: — Raw clinical, demographic, and treatment data. Raw clinical, demographic, and prior treatment data for 61 individuals who responded to the health-assessment survey. (PDF 50 kb) [file 12920_2015_145_MOESM3_ESM.pdf]

Patient	Age	Edu	Marital	RelPref	Health	Physical	MenstrAge	Contr	ContrAge1st	Pregnant	PregnantNo	TtlLiveBirth	FirstBirthAge	LastBirthAge	BrFeed	UnableChild	Period	PeriodStop	PeriodAge	Tamoxifen	Alcohol	CigSmoke	Employment	Polyps	ImmunoDisorder	Hypertension	AntiInfDrug
Utah__C06	50	5	2	37	2	4	13	1	25	1	7	7	20	34	1	1	4	1	48	1	2	2	2	NA	2	2	2
Utah__I06	55	6	2	34	1	5	14	1	22	1	1	1	27	27	2	2	4	1	45	2	2	1	1	NA	2	2	2
Utah__G12*	47	6	4	37	2	2	12	2	NA	1	1	0	NA	NA	NA	1	4	1	41	2	2	2	1	NA	1	1	1
Utah__H08	67	6	2	37	3	3	12	1	34	1	2	2	34	36	2	2	4	1	58	2	2	2	3	NA	1	2	2
Utah__D12	49	3	2	37	3	3	14	1	19	1	6	6	20	34	1	1	4	1	47	2	2	2	2	NA	2	2	2
Utah__D08	76	4	2	37	4	3	15	2	NA	1	4	4	21	31	2	2	4	1	32	2	2	2	1	2	1	1	1
Utah__E11	54	5	2	37	3	2	9	1	21	1	10	5	18	28	1	1	4	1	29	2	1	1	3	2	1	1	1
Utah__H05	45	5	2	37	2	1	13	1	18	1	3	2	31	35	1	1	1	2	NA	2	2	2	3	NA	2	2	2
Utah__C07	52	6	2	37	3	3	11	1	23	1	6	5	26	39	1	1	4	1	51	2	2	2	3	NA	1	2	1
Utah__K02	53	5	2	5	3	3	14	1	21	1	3	2	35	38	2	1	2	2	NA	2	2	2	3	2	2	2	2
Utah__E08	64	5	2	1	1	2	12	1	30	1	4	3	19	29	2	1	4	1	55	2	1	1	3	NA	1	1	1
Utah__E02	46	7	4	37	2	2	14	1	22	1	4	4	24	38	1	1	3	1	45	2	2	2	2	NA	2	2	1
Utah__H10	56	7	2	1	2	2	11	1	21	1	3	2	24	27	2	1	4	1	52	1	2	2	1	NA	2	2	2
Utah__D10	55	3	2	37	2	2	12	1	20	1	3	3	19	26	2	1	4	1	46	2	2	2	2	NA	2	2	2
Utah__D11	59	7	4	37	2	5	12	1	23	1	3	3	24	29	1	2	4	2	NA	2	1	2	3	NA	2	2	2
Utah__I05	62	5	2	37	2	2	14	2	NA	1	5	5	21	36	1	1	4	1	54	2	2	2	3	2	1	2	2
Utah__I07	51	3	2	37	3	2	13	1	17	1	6	5	20	28	1	2	1	2	NA	2	2	2	1	2	1	1	2
Utah__I08	51	5	2	37	1	2	13	1	20	1	6	6	19	29	1	1	4	1	43	2	2	2	1	NA	2	2	2
Utah__B01	56	5	2	6	1	2	14	1	22	1	3	2	29	31	1	1	4	1	37	2	1	1	3	2	2	2	2
Utah__I10	67	4	5	37	2	4	13	2	NA	1	5	5	18	32	1	1	4	1	33	2	2	2	1	1	1	1	1
Utah__C09	54	5	2	37	3	2	12	2	NA	1	3	1	18	18	1	2	4	1	48	2	2	2	1	NA	2	1	2
Utah__H01	55	3	2	5	3	4	10	1	19	1	3	1	28	29	1	2	4	1	46	2	2	2	2	NA	1	2	1
Utah__B04	50	3	2	37	2	2	12	1	19	1	4	4	21	29	1	1	4	1	37	1	2	2	2	NA	2	2	2
Utah__G11	53	6	2	37	4	2	12	2	NA	1	4	4	24	35	2	1	4	1	51	1	2	2	3	1	2	2	1
Utah__E12	63	5	2	37	2	1	13	1	30	1	3	3	25	28	2	1	4	1	54	1	1	2	3	2	1	1	1
Utah__C08	56	7	4	1	1	3	12	1	24	1	2	2	31	33	1	1	4	1	41	2	1	1	1	2	2	2	2
Utah__C03	72	2	2	37	1	3	15	2	NA	1	4	4	27	37	1	2	4	1	54	1	2	2	3	2	1	2	2
Utah__I12	54	6	2	37	1	2	13	1	48	1	7	7	25	39	1	2	4	1	49	2	2	2	3	NA	1	2	1
Utah__C02	51	5	2	37	1	2	13	1	31	1	5	5	20	31	1	1	1	2	NA	2	2	2	1	NA	2	2	2
Utah__E06	57	3	2	37	3	2	15	2	NA	1	4	4	20	31	1	2	4	1	36	2	2	2	3	2	1	2	2
Utah__H02	54	3	2	37	5	2	14	1	25	1	3	3	20	27	1	2	4	1	30	2	1	2	2	1	1	1	2
Utah__E05	58	3	2	37	2	4	12	1	19	1	6	4	22	28	1	1	4	1	45	2	2	2	2	2	2	1	2
Utah__E10	75	3	2	37	2	2	13	1	35	1	6	6	20	29	2	2	4	1	52	2	2	2	3	1	1	1	2
Utah__E01	58	6	2	37	3	3	12	2	NA	1	7	4	23	34	1	2	4	1	48	2	2	2	3	2	2	2	2
Utah__G08	57	5	2	37	1	2	12	1	21	1	4	4	23	29	1	1	4	1	38	2	2	2	2	2	2	2	2
Utah__E07	62	5	2	37	3	2	13	1	32	1	3	4	31	38	2	1	4	2	NA	1	2	2	3	2	2	2	2
Utah__G07	56	6	2	1	2	2	16	1	21	1	5	3	28	37	1	1	4	2	NA	2	2	2	1	2	2	2	1
Utah__I11	65	5	5	37	3	3	14	1	20	1	4	4	20	36	2	1	4	1	50	2	2	2	3	2	2	1	1
Utah__K03	50	4	2	37	2	4	12	1	21	1	5	4	23	29	1	1	4	1	43	2	2	2	2	2	2	2	2
Utah__K04	76	3	5	37	3	3	11	1	32	1	4	4	19	35	2	2	4	2	NA	2	2	2	3	NA	1	1	1
Utah__K05	56	5	5	37	3	2	12	1	24	1	4	3	25	32	1	1	4	1	38	2	2	2	1	1	1	2	1
Utah__C05	65	6	2	70	1	2	12	1	40	1	3	3	23	30	1	1	4	1	55	1	1	1	3	1	2	1	2
Utah__G06	69	4	2	37	2	2	14	2	NA	1	4	4	21	30	1	2	4	1	55	2	2	2	3	2	1	2	1
Utah__E04	53	6	4	37	3	2	15	1	23	1	3	3	24	31	2	9	4	1	50	2	2	2	1	NA	2	2	2
Utah__G02	53	7	2	5	1	2	12	2	NA	1	3	3	28	36	1	1	1	2	NA	2	1	2	1	NA	2	2	2
Utah__K01	35	5	2	37	3	3	10	1	19	1	5	4	20	27	1	1	1	2	NA	2	2	2	3	NA	2	2	2
Utah__E09	43	6	2	37	2	2	13	1	27	1	2	2	32	35	1	1	1	2	NA	2	2	2	2	NA	2	2	1
Utah__D07	68	3	2	37	3	3	15	2	NA	1	3	3	18	30	2	1	4	2	NA	2	2	2	3	1	2	2	1
Utah__H03	51	3	2	37	3	2	13	1	20	1	7	3	19	30	1	1	4	1	29	2	1	1	1	1	1	2	1
Utah__G10	64	2	2	37	2	4	11	2	NA	1	6	6	18	25	1	1	2	1	40	1	2	2	3	2	1	2	1
Utah__B08	57	6	2	6	1	2	11	1	23	1	4	2	27	28	1	1	4	1	51	2	2	2	2	NA	1	1	2
Utah__H11	69	6	2	37	2	2	14	2	NA	1	5	4	28	35	1	2	4	1	54	1	2	2	3	2	2	2	2
Utah__G03	58	6	2	5	2	2	13	1	32	1	3	3	24	27	1	1	4	2	NA	2	1	1	3	NA	2	2	2
Utah__C10*	51	5	1	1	2	3	11	1	18	2	NA	NA	NA	NA	NA	2	4	1	45	2	1	1	1	2	2	2	1
Utah__B07	65	3	2	37	4	2	11	2	NA	1	9	7	18	40	1	1	4	1	50	2	2	2	3	2	1	2	1
Utah__B06	47	5	2	37	2	2	12	1	19	1	4	4	22	28	1	1	2	2	NA	2	2	2	2	NA	2	2	2
Utah__I09	48	6	2	37	3	2	12	2	NA	1	7	6	25	36	1	1	4	1	37	2	2	2	3	1	1	1	1
Utah__G09	47	6	2	37	1	2	13	1	22	1	9	5	24	36	1	1	4	1	44	2	2	2	2	NA	2	2	1
Utah__B05	50	3	2	1	1	2	13	1	20	1	2	2	21	25	2	1	4	1	42	2	1	2	1	NA	2	2	2
Utah__C11	51	6	2	37	1	2	13	1	19	1	4	4	20	30	2	1	4	1	45	2	2	2	1	NA	2	2	2
Utah__F03	66	5	2	37	2	2	11	1	36	1	3	3	20	28	1	1	4	1	51	1	2	2	3	1	1	2	2
Utah__G05	65	5	2	37	2	3	13	2	NA	1	7	5	22	31	2	1	4	1	48	2	2	2	3	2	2	2	2
Utah__C01	47	6	2	37	2	1	13	2	NA	1	7	5	27	39	1	2	1	2	NA	2	2	2	3	NA	2	2	2

This table contains health-survey responses that were provided by research participants from the Utah cohort. Descriptions of the variables are provided in Additional file 3.
