# Supplementary material for: Gene-expression patterns in peripheral blood classify familial breast cancer susceptibility
Source: BMC Med Genomics. 2015 Nov 4;8:72. doi: 10.1186/s12920-015-0145-6 (PMC4634735; doi:10.1186/s12920-015-0145-6)
Supplement: Additional file 4: — Descriptions of variables used in health-assessment survey. Descriptions of variables that were used in the health-assessment survey. (PDF 41 kb) [file 12920_2015_145_MOESM4_ESM.pdf]

Variable	Description	Options
Age	Age of diagnosis or age at which survey was completed	
Edu	Education	1. Less than 8 years
Edu	Education	2. 8 - 11 years (without graduation)
Edu	Education	3. High school graduation
Edu	Education	4. Vocational or technical school
Edu	Education	5. Some college or university
Edu	Education	6. Bachelor's degree
Edu	Education	7. Graduate degree
Marital	Marriage Status	1. Never married
Marital	Marriage Status	2. Married or living as married
Marital	Marriage Status	3. Separated
Marital	Marriage Status	4. Divorced
Marital	Marriage Status	5. Widowed
Marital	Marriage Status	9. Unknown
RelPref	Religious Preference	01. None
RelPref	Religious Preference	05. Roman Catholic
RelPref	Religious Preference	06. Christian or Protestant, NOS
RelPref	Religious Preference	34. Orthodox, Christian, NOS or Other (Lebanese Maronite)
RelPref	Religious Preference	37. Latter Day Saints (Mormon)
RelPref	Religious Preference	70. Jewish
Health	In General, your health is:	1. Excellent
Health	In General, your health is:	2. Very good
Health	In General, your health is:	3. Good
Health	In General, your health is:	4. Fair
Health	In General, your health is:	5. Poor
Physical	Last Complete Physical Exam	1. Never
Physical	Last Complete Physical Exam	2. Within the past year (0 to 12 months ago)
Physical	Last Complete Physical Exam	3. One to two years ago (13 to 24 months ago)
Physical	Last Complete Physical Exam	4. Two to five years ago (25 to 60 months ago)
Physical	Last Complete Physical Exam	5. More than five years ago (61 months or more)
MenstrAge	Age at menarche	
Contr	Ever used hormonal Contraceptives?	1. Yes
Contr	Ever used hormonal Contraceptives?	2. No
Contr	Ever used hormonal Contraceptives?	9. Unknown
ContrAge1st	Age at first contraceptive use	
Pregnant	Ever been Pregnant?	1. Yes
Pregnant	Ever been Pregnant?	2. No

Pregnant	Ever been Pregnant?	9. Unknown
PregnantNo	Number of pregnancies	
TtlLiveBirth	Total live births	
FirstBirthAge	Age at first birth	
LastBirthAge	Age at last birth	
BrFeed	Ever Breast Feed for one month or more?	1. Yes
BrFeed	Ever Breast Feed for one month or more?	2. No
BrFeed	Ever Breast Feed for one month or more?	9. Unknown
UnableChild	Unable to have children	1. Yes
UnableChild	Unable to have children	2. No
UnableChild	Unable to have children	9. Unknown
Period	Last Period	1. Less than 1 month
Period	Last Period	2. 1 to 6 months
Period	Last Period	3. 7 months to less than 1 year
Period	Last Period	4. 1 year or more
Period	Last Period	5. Never had a menstrual period
Period	Last Period	9. Unknown
PeriodStop	Period Stop	1. Yes
PeriodStop	Period Stop	2. No
PeriodStop	Period Stop	9. Unknown
PeriodAge	Age when period stopped	
Tamoxifen	Tamoxifen	1. Yes
Tamoxifen	Tamoxifen	2. No
Tamoxifen	Tamoxifen	9. Unknown
Alcohol	Consumed alcohol for 6 months or longer?	1. Yes
Alcohol	Consumed alcohol for 6 months or longer?	2. No
Alcohol	Consumed alcohol for 6 months or longer?	9. Unknown
CigSmoke	Smoked at least one cigarette a day	1. Yes
CigSmoke	Smoked at least one cigarette a day	2. No
CigSmoke	Smoked at least one cigarette a day	9. Unknown
Employment	Do you currently have a job?	1. Employed full time
Employment	Do you currently have a job?	2. Employed part time
Employment	Do you currently have a job?	3. Currently not employed
Polyps	Polyps	1. Yes
Polyps	Polyps	2. No
Polyps	Polyps	9. Unknown.
ImmunoDisorder	Immunologic Disorder	1. Yes
ImmunoDisorder	Immunologic Disorder	2. No
ImmunoDisorder	Immunologic Disorder	9. Unknown
Hypertension	Hypertension	1. Yes
Hypertension	Hypertension	2. No
Hypertension	Hypertension	9. Unknown

AntiInfDrug	Anti inflammatory	1. Yes
AntiInfDrug	Anti inflammatory	2. No
AntiInfDrug	Anti inflammatory	9. Unknown
