# Supplementary material for: Gene-expression patterns in peripheral blood classify familial breast cancer susceptibility
Source: BMC Med Genomics. 2015 Nov 4;8:72. doi: 10.1186/s12920-015-0145-6 (PMC4634735; doi:10.1186/s12920-015-0145-6)
Supplement: Additional file 5: — Cancer, family-history, and BRCA1/2 status for all patients. This file indicates cancer, family history, and BRCA1/2 status for patients in the Utah and Ontario cohorts. (PDF 51 kb) [file 12920_2015_145_MOESM5_ESM.pdf]

Patient	Had Cancer	Had Family History	Had BRCA1/2 Mutation	Had BRCA1 mutation	Had BRCA2 mutation
Utah__A01	No	No	No	No	No
Utah__A02	Yes	No	No	No	No
Utah__A03	Yes	No	No	No	No
Utah__A04	Yes	No	No	No	No
Utah__A05	Yes	No	No	No	No
Utah__A06	Yes	No	No	No	No
Utah__A07	No	No	No	No	No
Utah__A08	No	No	No	No	No
Utah__A09	No	No	No	No	No
Utah__A10	No	No	No	No	No
Utah__A11	No	No	No	No	No
Utah__A12	No	No	No	No	No
Utah__B01	No	Yes	No	No	No
Utah__B02	No	Yes	No	No	No
Utah__B03	Yes	Yes	No	No	No
Utah__B04	Yes	Yes	No	No	No
Utah__B05	Yes	Yes	No	No	No
Utah__B06	Yes	Yes	No	No	No
Utah__B07	Yes	Yes	No	No	No
Utah__B08	No	Yes	No	No	No
Utah__B09	No	Yes	No	No	No
Utah__B10	No	Yes	No	No	No
Utah__B11	No	Yes	No	No	No
Utah__B12	No	No	No	No	No
Utah__C01	Yes	Yes	Yes	No	Yes
Utah__C02	Yes	Yes	Yes	No	Yes
Utah__C03	Yes	Yes	Yes	No	Yes
Utah__C04	No	Yes	Yes	Yes	No
Utah__C05	Yes	Yes	Yes	No	Yes

Utah__C06	No	Yes	Yes	No	Yes
Utah__C07	No	Yes	Yes	No	Yes
Utah__C08	No	Yes	Yes	No	Yes
Utah__C09	No	Yes	Yes	Yes	No
Utah__C10	Yes	Yes	Yes	Yes	No
Utah__C11	No	Yes	Yes	Yes	No
Utah__C12	Yes	Yes	Yes	No	Yes
Utah__D01	Yes	Yes	No	No	No
Utah__D02	Yes	Yes	No	No	No
Utah__D03	Yes	Yes	No	No	No
Utah__D04	Yes	Yes	No	No	No
Utah__D05	Yes	Yes	No	No	No
Utah__D06	Yes	Yes	No	No	No
Utah__D07	No	Yes	No	No	No
Utah__D08	No	Yes	No	No	No
Utah__D09	No	Yes	No	No	No
Utah__D10	No	Yes	No	No	No
Utah__D11	No	Yes	No	No	No
Utah__D12	No	Yes	No	No	No
Utah__E01	Yes	Yes	Yes	No	Yes
Utah__E02	No	Yes	Yes	Yes	No
Utah__E03	No	Yes	Yes	Yes	No
Utah__E04	No	Yes	Yes	Yes	No
Utah__E05	No	Yes	Yes	No	Yes
Utah__E06	Yes	Yes	Yes	No	Yes
Utah__E07	Yes	Yes	Yes	No	Yes
Utah__E08	No	Yes	No	No	No
Utah__E09	Yes	Yes	Yes	Yes	No
Utah__E10	Yes	Yes	Yes	No	Yes
Utah__E11	No	Yes	No	No	No

Utah__E12	Yes	Yes	No	No	No
Utah__F01	Yes	No	No	No	No
Utah__F02	Yes	No	No	No	No
Utah__F03	Yes	No	No	No	No
Utah__F04	Yes	No	No	No	No
Utah__F05	Yes	No	No	No	No
Utah__F06	Yes	No	No	No	No
Utah__F07	No	No	No	No	No
Utah__F09	No	No	No	No	No
Utah__F10	No	No	No	No	No
Utah__F11	No	No	No	No	No
Utah__F12	No	No	No	No	No
Utah__G01	No	Yes	No	No	No
Utah__G02	No	Yes	No	No	No
Utah__G03	No	Yes	No	No	No
Utah__G04	Yes	Yes	No	No	No
Utah__G05	Yes	Yes	No	No	No
Utah__G06	Yes	Yes	No	No	No
Utah__G07	No	Yes	Yes	No	Yes
Utah__G08	No	Yes	Yes	No	Yes
Utah__G09	No	Yes	Yes	Yes	No
Utah__G10	Yes	Yes	Yes	Yes	No
Utah__G11	Yes	Yes	Yes	Yes	No
Utah__G12	Yes	Yes	Yes	No	Yes
Utah__H01	No	Yes	No	No	No
Utah__H02	No	Yes	No	No	No
Utah__H03	No	Yes	No	No	No
Utah__H04	No	Yes	No	No	No
Utah__H06	No	Yes	No	No	No
Utah__H07	Yes	Yes	No	No	No

Utah__H08	Yes	Yes	No	No	No
Utah__H09	Yes	Yes	No	No	No
Utah__H10	Yes	Yes	No	No	No
Utah__H11	Yes	Yes	No	No	No
Utah__H12	Yes	Yes	No	No	No
Utah__I01	Yes	No	No	No	No
Utah__I02	Yes	No	No	No	No
Utah__I03	Yes	No	No	No	No
Utah__I04	Yes	No	No	No	No
Utah__I05	No	Yes	No	No	No
Utah__I06	No	Yes	No	No	No
Utah__I07	No	Yes	No	No	No
Utah__I08	No	Yes	Yes	No	Yes
Utah__I09	No	Yes	Yes	Yes	No
Utah__I10	No	Yes	Yes	Yes	No
Utah__I11	No	Yes	No	No	No
Utah__I12	No	Yes	Yes	No	Yes
Utah__I13	Yes	No	No	No	No
Utah__J01	No	No	No	No	No
Utah__J02	No	No	No	No	No
Utah__J03	Yes	No	No	No	No
Utah__J04	Yes	No	No	No	No
Utah__J05	Yes	No	No	No	No
Utah__J06	Yes	No	No	No	No
Utah__J07	Yes	No	No	No	No
Utah__J08	Yes	No	No	No	No
Utah__J09	No	No	No	No	No
Utah__J10	No	No	No	No	No
Utah__J11	No	No	No	No	No
Utah__J12	No	No	No	No	No

Utah__K01	Yes	Yes	Yes	Yes	No
Utah__K02	Yes	Yes	No	No	No
Utah__K03	Yes	Yes	Yes	Yes	No
Utah__K04	Yes	Yes	No	No	No
Utah__K05	No	Yes	Yes	Yes	No
Ontario__K1162	Yes	Yes	Yes	N/A	N/A
Ontario__P1075	Yes	Yes	No	No	No
Ontario__Q1080	Yes	Yes	No	No	No
Ontario__R1104	Yes	Yes	No	No	No
Ontario__S1133	Yes	Yes	No	No	No
Ontario__Z3225	No	Yes	Yes	N/A	N/A
Ontario__AE1543	No	Yes	No	No	No
Ontario__AF3229	No	Yes	No	No	No
Ontario__AG3230	No	Yes	No	No	No
Ontario__AH3232	No	Yes	No	No	No
Ontario__C474	Yes	No	No	No	No
Ontario__D480	Yes	No	No	No	No
Ontario__L1176	Yes	Yes	Yes	N/A	N/A
Ontario__M1263	Yes	Yes	Yes	N/A	N/A
Ontario__T1463	Yes	Yes	No	No	No
Ontario__U2017	Yes	Yes	No	No	No
Ontario__V432	Yes	Yes	No	No	No
Ontario__AA3226	No	Yes	Yes	N/A	N/A
Ontario__AB3227	No	Yes	Yes	N/A	N/A
Ontario__AI3233	No	Yes	No	No	No
Ontario__AJ3234	No	Yes	No	No	No
Ontario__AK3235	No	Yes	No	No	No
Ontario__E519	Yes	No	No	No	No
Ontario__F548	Yes	No	No	No	No
Ontario__W433	Yes	Yes	No	No	No

Ontario__X524	Yes	Yes	No	No	No
Ontario__Y546	Yes	Yes	No	No	No
Ontario__AC3228	No	Yes	Yes	N/A	N/A
Ontario__AD3231	No	Yes	Yes	N/A	N/A
Ontario__AL3236	No	Yes	No	No	No
Ontario__AM3237	No	Yes	No	No	No
Ontario__AN757	No	Yes	No	No	No
Ontario__G660	Yes	No	No	No	No
Ontario__H663	Yes	No	No	No	No
Ontario__I782	Yes	No	No	No	No
Ontario__J787	Yes	No	No	No	No
Ontario__AA_RR-849	Yes	Yes	No	No	No
Ontario__AE_RR-3300	No	Yes	No	No	No
Ontario__AF_RR-3276	No	Yes	No	No	No
Ontario__AG_RR-3298	No	Yes	No	No	No
Ontario__AH_RR-3295	No	Yes	No	No	No
Ontario__AI_RR-3291	No	Yes	No	No	No
Ontario__AJ_RR-3296	No	Yes	No	No	No
Ontario__AM_RR-1949	No	Yes	No	No	No
Ontario__AN_RR-3297	No	Yes	No	No	No
Ontario__AO_RR-3288	No	No	No	No	No
Ontario__AP_RR-3280	No	No	No	No	No
Ontario__AR_RR-3278	No	No	No	No	No
Ontario__AS_RR-3277	No	No	No	No	No
Ontario__A_RR96-12	Yes	Yes	Yes	N/A	N/A
Ontario__B_RR-1519	Yes	Yes	Yes	N/A	N/A
Ontario__C_RR-3293	Yes	Yes	Yes	N/A	N/A
Ontario__D_RR-3301	Yes	Yes	Yes	N/A	N/A
Ontario__E_RR-2070	Yes	Yes	Yes	N/A	N/A
Ontario__J_RR-1226	Yes	Yes	Yes	N/A	N/A

Ontario__K_RR-3290	No	Yes	Yes	N/A	N/A
Ontario__L_RR-3286	No	Yes	Yes	N/A	N/A
Ontario__N_RR-3294	No	Yes	Yes	N/A	N/A
Ontario__O_RR-3285	No	Yes	Yes	N/A	N/A
Ontario__P_RR-3274	No	Yes	Yes	N/A	N/A
Ontario__Q_RR-3283	No	Yes	Yes	N/A	N/A
Ontario__RR-3275	No	Yes	Yes	N/A	N/A
Ontario__RR-3281	No	No	No	No	No
Ontario__RR-3287	Yes	Yes	Yes	N/A	N/A
Ontario__RR-3289	No	Yes	Yes	N/A	N/A
Ontario__RR-3299	Yes	Yes	Yes	N/A	N/A
Ontario__T_RR-3292	No	Yes	Yes	N/A	N/A
Ontario__U_RR-2236	Yes	Yes	No	No	No
Ontario__V_RR-1455	Yes	Yes	No	No	No
Ontario__W_RR-922	Yes	Yes	No	No	No
Ontario__X_RR-1083	Yes	Yes	No	No	No
Ontario__Y_RR-841	Yes	Yes	No	No	No
Ontario__Z_RR-778	Yes	Yes	No	No	No

N/A = Not available
