# Supplementary material for: Gene-expression patterns in peripheral blood classify familial breast cancer susceptibility
Source: BMC Med Genomics. 2015 Nov 4;8:72. doi: 10.1186/s12920-015-0145-6 (PMC4634735; doi:10.1186/s12920-015-0145-6)
Supplement: Additional file 6: — Genes filtered based on correlation with potential confounders. Genes filtered based on association between gene-expression levels and clinical, demographic, prior treatment, or lymphocyte enumeration data. (PDF 82 kb) [file 12920_2015_145_MOESM6_ESM.pdf]

Entrez ID	Symbol	Name
51146	A4GNT	alpha-1,4-N-acetylglucosaminyltransferase
15	AANAT	aralkylamine N-acetyltransferase
5244	ABCB4	ATP-binding cassette, sub-family B (MDR/TAP), member 4
125981	ACER1	alkaline ceramidase 1
653857	ACTR3C	ARP3 actin-related protein 3 homolog C (yeast)
94	ACVRL1	activin A receptor type II-like 1
8728	ADAM19	ADAM metallopeptidase domain 19
10863	ADAM28	ADAM metallopeptidase domain 28
9510	ADAMTS1	ADAM metallopeptidase with thrombospondin type 1 motif, 1
124	ADH1A	alcohol dehydrogenase 1A (class I), alpha polypeptide
84883	AIFM2	apoptosis-inducing factor, mitochondrion-associated, 2
8644	AKR1C3	aldo-keto reductase family 1, member C3 (3-alpha hydroxysteroid dehydrogenase, type II)
217	ALDH2	aldehyde dehydrogenase 2 family (mitochondrial)
266	AMELY	amelogenin, Y-linked
10218	ANGPTL7	angiopoietin-like 7
26287	ANKRD2	ankyrin repeat domain 2 (stretch responsive muscle)
151516	ASPRV1	aspartic peptidase, retroviral-like 1
10079	ATP9A	ATPase, class II, type 9A
283358	B4GALNT3	beta-1,4-N-acetyl-galactosaminyl transferase 3
55024	BANK1	B-cell scaffold protein with ankyrin repeats 1
640	BLK	B lymphoid tyrosine kinase
168667	BMPER	BMP binding endothelial regulator
140707	BRI3BP	BRI3 binding protein
283025	C10orf40	chromosome 10 open reading frame 40
56673	C11orf16	chromosome 11 open reading frame 16
256369	C14orf48	chromosome 14 open reading frame 48
199675	C19orf59	chromosome 19 open reading frame 59
128346	C1orf162	chromosome 1 open reading frame 162
391269	C21orf126	ankyrin repeat domain 20 family, member A3 pseudogene

348738	C2orf48	chromosome 2 open reading frame 48
152065	C3orf22	chromosome 3 open reading frame 22
152816	C4orf26	chromosome 4 open reading frame 26
116328	C8orf34	chromosome 8 open reading frame 34
55472	C8orf39	chromosome 8 open reading frame 39
56344	CABP5	calcium binding protein 5
776	CACNA1D	calcium channel, voltage-dependent, L type, alpha 1D subunit
117144	CATSPER1	cation channel, sperm associated 1
164592	CCDC116	coiled-coil domain containing 116
120935	CCDC38	coiled-coil domain containing 38
6361	CCL17	chemokine (C-C motif) ligand 17
1235	CCR6	chemokine (C-C motif) receptor 6
930	CD19	CD19 molecule
4345	CD200	CD200 molecule
933	CD22	CD22 molecule
972	CD74	CD74 molecule, major histocompatibility complex, class II invariant chain
973	CD79A	CD79a molecule, immunoglobulin-associated alpha
926	CD8B	CD8b molecule
22918	CD93	CD93 molecule
1675	CFD	complement factor D (adipsin)
10970	CKAP4	cytoskeleton-associated protein 4
161198	CLEC14A	C-type lectin domain family 14, member A
388512	CLEC17A	C-type lectin domain family 17, member A
157807	CLVS1	clavesin 1
146225	CMTM2	CKLF-like MARVEL transmembrane domain containing 2
53942	CNTN5	contactin 5
1310	COL19A1	collagen, type XIX, alpha 1
344875	COL6A4P1	collagen, type VI, alpha 4 pseudogene 1
10699	CORIN	corin, serine peptidase
1356	CP	ceruloplasmin (ferroxidase)

1369	CPN1	carboxypeptidase N, polypeptide 1
221184	CPNE2	copine II
53336	CPXCR1	CPX chromosome region, candidate 1
8804	CREG1	cellular repressor of E1A-stimulated genes 1
10491	CRTAP	cartilage associated protein
140690	CTCFL	CCCTC-binding factor (zinc finger protein)-like
139212	CXorf41	chromosome X open reading frame 41
79901	CYBRD1	cytochrome b reductase 1
163007	CYP2T3P	cytochrome P450, family 2, subfamily T, polypeptide 3 pseudogene
58511	DNASE2B	deoxyribonuclease II beta
1808	DPYSL2	dihydropyrimidinase-like 2
100131899	E2F4P1	E2F transcription factor 4, p107/p130-binding pseudogene 1
1879	EBF1	early B-cell factor 1
1842	ECM2	extracellular matrix protein 2, female organ and adipocyte specific
80258	EFHC2	EF-hand domain (C-terminal) containing 2
79852	EPHX3	epoxide hydrolase 3
2184	FAH	fumarylacetoacetate hydrolase (fumarylacetoacetase)
199786	FAM129C	family with sequence similarity 129, member C
84182	FAM188B	family with sequence similarity 188, member B
63895	FAM38B	family with sequence similarity 38, member B
2204	FCAR	Fc fragment of IgA, receptor for
2208	FCER2	Fc fragment of IgE, low affinity II, receptor for (CD23)
115350	FCRL1	Fc receptor-like 1
79368	FCRL2	Fc receptor-like 2
84824	FCRLA	Fc receptor-like A
2258	FGF13	fibroblast growth factor 13
100128181	FMO10P	flavin containing monooxygenase 10, pseudogene
2328	FMO3	flavin containing monooxygenase 3
388714	FMO6P	flavin containing monooxygenase 6 pseudogene
2355	FOSL2	FOS-like antigen 2

283150	FOXR1	forkhead box R1
80020	FOXRED2	FAD-dependent oxidoreductase domain containing 2
53826	FXYD6	FXYD domain containing ion transport regulator 6
8327	GABPAP	GA binding protein transcription factor, alpha subunit pseudogene
2568	GABRP	gamma-aminobutyric acid (GABA) A receptor, pi
79623	GALNT14	UDP-N-acetyl-alpha-D-galactosamine:polypeptide N-acetylgalactosaminyltransferase 14 (GalNAc-T14)
449520	GGNBP1	gametogenetin binding protein 1
2681	GGTA1P	glycoprotein, alpha-galactosyltransferase 1 pseudogene
11010	GLIPR1	GLI pathogenesis-related 1
2774	GNAL	guanine nucleotide binding protein (G protein), alpha activating activity polypeptide, olfactory type
2788	GNG7	guanine nucleotide binding protein (G protein), gamma 7
51280	GOLM1	golgi membrane protein 1
353345	GPR141	G protein-coupled receptor 141
344561	GPR148	G protein-coupled receptor 148
2893	GRIA4	glutamate receptor, ionotrophic, AMPA 4
2998	GYS2	glycogen synthase 2 (liver)
3002	GZMB	granzyme B (granzyme 2, cytotoxic T-lymphocyte-associated serine esterase 1)
3005	H1F0	H1 histone family, member 0
727957	HEATR7A	HEAT repeat containing 7A
28996	HIPK2	homeodomain interacting protein kinase 2
8350	HIST1H3A	histone cluster 1, H3a
8351	HIST1H3D	histone cluster 1, H3d
8357	HIST1H3H	histone cluster 1, H3h
3112	HLA-DOB	major histocompatibility complex, class II, DO beta
83872	HMCN1	hemicentin 1
9455	HOMER2	homer homolog 2 (Drosophila)
3273	HRG	histidine-rich glycoprotein
203100	HTRA4	HtrA serine peptidase 4
84329	HVCN1	hydrogen voltage-gated channel 1
8870	IER3	immediate early response 3

387733	IFITM5	interferon induced transmembrane protein 5
3495	IGHD	immunoglobulin heavy constant delta
28373	IGHVII-26-2	immunoglobulin heavy variable (II)-26-2 (pseudogene)
28354	IGHVIII-5-1	immunoglobulin heavy variable (III)-5-1 (pseudogene)
28908	IGKV4-1	immunoglobulin kappa variable 4-1
152404	IGSF11	immunoglobulin superfamily, member 11
3598	IL13RA2	interleukin 13 receptor, alpha 2
3570	IL6R	interleukin 6 receptor
650747	IMPA1P	inositol(myo)-1(or 4)-monophosphatase 1 pseudogene
128239	IQGAP3	IQ motif containing GTPase activating protein 3
3667	IRS1	insulin receptor substrate 1
23254	KAZN	kazrin, periplakin interacting protein
30819	KCNIP2	Kv channel interacting protein 2
9424	KCNK6	potassium channel, subfamily K, member 6
3782	KCNN3	potassium intermediate/small conductance calcium-activated channel, subfamily N, member 3
3792	KEL	Kell blood group, metallo-endopeptidase
9834	KIAA0125	KIAA0125
57565	KLHL14	kelch-like 14 (Drosophila)
11202	KLK8	kallikrein-related peptidase 8
3821	KLRC1	killer cell lectin-like receptor subfamily C, member 1
22914	KLRK1	killer cell lectin-like receptor subfamily K, member 1
729682	KRT17P3	keratin 17 pseudogene 3
391584	KRT18P35	keratin 18 pseudogene 35
442114	KRT19P3	keratin 19 pseudogene 3
3912	LAMB1	laminin, beta 1
3915	LAMC1	laminin, gamma 1 (formerly LAMB2)
55323	LARP6	La ribonucleoprotein domain family, member 6
3948	LDHC	lactate dehydrogenase C
388633	LDLRAD1	low density lipoprotein receptor class A domain containing 1
342900	LEUTX	leucine twenty homeobox

100127904	LOC100127904	hypothetical LOC100127904
100128523	LOC100128523	mitochondrial carrier homolog 2 pseudogene
100128568	LOC100128568	similar to hCG2045263
100128655	LOC100128655	similar to hCG1645245
100128712	LOC100128712	slowmo homolog 2 (Drosophila) pseudogene
100129672	LOC100129672	Cdon homolog (mouse) pseudogene
100129878	LOC100129878	hypothetical protein LOC100129878
100129972	LOC100129972	nucleophosmin (nucleolar phosphoprotein B23, numatrin) pseudogene
100130100	LOC100130100	ig kappa chain V-I region Walker-like
100130660	LOC100130660	heterogeneous nuclear ribonucleoprotein A1 pseudogene
100131072	LOC100131072	karyopherin (importin) beta 1 pseudogene
100131131	LOC100131131	AHPA9419
100131340	LOC100131340	5'-nucleotidase domain containing 1 pseudogene
100132609	LOC100132609	programmed cell death 2 pseudogene
100132807	LOC100132807	similar to hCG2040244
139542	LOC139542	hCG1660138
255187	LOC255187	hCG1980447
283663	LOC283663	hypothetical LOC283663
392426	LOC392426	nucleolar and coiled-body phosphoprotein 1 pseudogene
393076	LOC393076	hypothetical LOC393076
441268	LOC441268	hypothetical LOC441268
441644	LOC441644	REST corepressor 2 pseudogene
441914	LOC441914	dynactin 5 (p25) pseudogene
442075	LOC442075	hypothetical LOC442075
442446	LOC442446	upstream binding transcription factor, RNA polymerase I pseudogene
644100	LOC644100	hypothetical LOC644100
644387	LOC644387	myelin protein zero-like 1 pseudogene
646701	LOC646701	developmental pluripotency associated 5 pseudogene
647145	LOC647145	splicing factor, arginine/serine-rich 2B pseudogene
649395	LOC649395	tyrosine 3-monooxygenase/tryptophan 5-monooxygenase activation protein, epsilon polypeptide pseudogene

139081	MAGEC3	melanoma antigen family C, 3
7867	MAPKAPK3	mitogen-activated protein kinase-activated protein kinase 3
83742	MARVELD1	MARVEL domain containing 1
4239	MFAP4	microfibrillar-associated protein 4
8972	MGAM	maltase-glucoamylase (alpha-glucosidase)
4245	MGAT1	mannosyl (alpha-1,3-)-glycoprotein beta-1,2-N-acetylglucosaminyltransferase
619555	MIR487A	microRNA 487a
406883	MIRLET7A3	microRNA let-7a-3
10335	MRVI1	murine retrovirus integration site 1 homolog
931	MS4A1	membrane-spanning 4-domains, subfamily A, member 1
4610	MYCL1	v-myc myelocytomatosis viral oncogene homolog 1, lung carcinoma derived (avian)
4619	MYH1	myosin, heavy chain 1, skeletal muscle, adult
4629	MYH11	myosin, heavy chain 11, smooth muscle
4647	MYO7A	myosin VIIA
51778	MYOZ2	myozenin 2
285622	NBPF22P	neuroblastoma breakpoint family, member 22, pseudogene
3340	NDST1	N-deacetylase/N-sulfotransferase (heparan glucosaminyl) 1
10276	NET1	neuroepithelial cell transforming 1
171176	NICN2P	nicolin 2, pseudogene
100132321	NIPA2P1	non imprinted in Prader-Willi/Angelman syndrome 2 pseudogene 1
91662	NLRP12	NLR family, pyrin domain containing 12
114548	NLRP3	NLR family, pyrin domain containing 3
4853	NOTCH2	notch 2
9970	NR1I3	nuclear receptor subfamily 1, group I, member 3
4923	NTSR1	neurotensin receptor 1 (high affinity)
266553	OFCC1	orofacial cleft 1 candidate 1
10215	OLIG2	oligodendrocyte lineage transcription factor 2
392390	OR1L6	olfactory receptor, family 1, subfamily L, member 6
26696	OR2T1	olfactory receptor, family 2, subfamily T, member 1
127077	OR2T11	olfactory receptor, family 2, subfamily T, member 11

119692	OR51S1	olfactory receptor, family 51, subfamily S, member 1
219447	OR5AS1	olfactory receptor, family 5, subfamily AS, member 1
150681	OR6B3	olfactory receptor, family 6, subfamily B, member 3
391114	OR6K3	olfactory receptor, family 6, subfamily K, member 3
114884	OSBPL10	oxysterol binding protein-like 10
5026	P2RX5	purinergic receptor P2X, ligand-gated ion channel, 5
5071	PARK2	parkinson protein 2, E3 ubiquitin protein ligase (parkin)
23783	PARP4P3	poly (ADP-ribose) polymerase family, member 4 pseudogene 3
5079	PAX5	paired box 5
55872	PBK	PDZ binding kinase
7703	PCGF2	polycomb group ring finger 2
253272	PCGF7P	polycomb group ring finger 7 pseudogene
5163	PDK1	pyruvate dehydrogenase kinase, isozyme 1
10630	PDPN	podoplanin
10158	PDZK1IP1	PDZK1 interacting protein 1
5314	PKHD1	polycystic kidney and hepatic disease 1 (autosomal recessive)
283748	PLA2G4D	phospholipase A2, group IVD (cytosolic)
5324	PLAG1	pleiomorphic adenoma gene 1
57480	PLEKHG1	pleckstrin homology domain containing, family G (with RhoGef domain) member 1
5347	PLK1	polo-like kinase 1
5408	PNLIPRP2	pancreatic lipase-related protein 2
56655	POLE4	polymerase (DNA-directed), epsilon 4 (p12 subunit)
64208	POPDC3	popeye domain containing 3
5470	PPEF2	protein phosphatase, EF-hand calcium binding domain 2
170540	PPIAP17	peptidylprolyl isomerase A (cyclophilin A) pseudogene 17
136242	PRSS37	protease, serine, 37
158471	PRUNE2	prune homolog 2 (Drosophila)
5724	PTAFR	platelet-activating factor receptor
5744	PTHLH	parathyroid hormone-like hormone
5796	PTPRK	protein tyrosine phosphatase, receptor type, K

55647	RAB20	RAB20, member RAS oncogene family
9609	RAB36	RAB36, member RAS oncogene family
115827	RAB3C	RAB3C, member RAS oncogene family
9545	RAB3D	RAB3D, member RAS oncogene family
55103	RALGPS2	Ral GEF with PH domain and SH3 binding motif 2
25780	RASGRP3	RAS guanyl releasing protein 3 (calcium and DAG-regulated)
143543	RBMXP3	RNA binding motif protein, X-linked pseudogene 3
116362	RBP7	retinol binding protein 7, cellular
348093	RBPMS2	RNA binding protein with multiple splicing 2
130120	REG3G	regenerating islet-derived 3 gamma
9185	REPS2	RALBP1 associated Eps domain containing 2
100131788	RPL26P29	ribosomal protein L26 pseudogene 29
646620	RPL3P8	ribosomal protein L3 pseudogene 8
344423	RPS12P3	ribosomal protein S12 pseudogene 3
390857	RPSAP57	ribosomal protein SA pseudogene 57
388015	RTL1	retrotransposon-like 1
84127	RUNDC2A	RUN domain containing 2A
6258	RXRG	retinoid X receptor, gamma
6447	SCG5	secretogranin V (7B2 protein)
64218	SEMA4A	sema domain, immunoglobulin domain (Ig), transmembrane domain (TM) and short cytoplasmic domain, (semaphorin) 4A
57190	SEPN1	selenoprotein N, 1
5055	SERPINB2	serpin peptidase inhibitor, clade B (ovalbumin), member 2
6317	SERPINB3	serpin peptidase inhibitor, clade B (ovalbumin), member 3
474174	SGCEP	sarcoglycan, epsilon, pseudogene
84251	SGIP1	SH3-domain GRB2-like (endophilin) interacting protein 1
117157	SH2D1B	SH2 domain containing 1B
284266	SIGLEC15	sialic acid binding Ig-like lectin 15
400709	SIGLEC16	sialic acid binding Ig-like lectin 16 (gene/pseudogene)
221150	SKA3	spindle and kinetochore associated complex subunit 3
89886	SLAMF9	SLAM family member 9

162515	SLC16A11	solute carrier family 16, member 11 (monocarboxylic acid transporter 11)
1317	SLC31A1	solute carrier family 31 (copper transporters), member 1
55089	SLC38A4	solute carrier family 38, member 4
81796	SLCO5A1	solute carrier organic anion transporter family, member 5A1
4184	SMCP	sperm mitochondria-associated cysteine-rich protein
767564	SNORD113-4	small nucleolar RNA, C/D box 113-4
79856	SNX22	sorting nexin 22
126506	SNX6P1	sorting nexin 6 pseudogene 1
10253	SPRY2	sprouty homolog 2 (Drosophila)
100131189	SSR1P2	signal sequence receptor, alpha pseudogene 2
6768	ST14	suppression of tumorigenicity 14 (colon carcinoma)
132204	SYNPR	synaptoporin
148281	SYT6	synaptotagmin VI
9287	TAAR2	trace amine associated receptor 2
338399	TAS2R62P	taste receptor, type 2, member 62, pseudogene
6915	TBXA2R	thromboxane A2 receptor
6918	TCEA1P1	transcription elongation factor A (SII), 1 pseudogene 1
8115	TCL1A	T-cell leukemia/lymphoma 1A
26136	TES	testis derived transcript (3 LIM domains)
81793	TLR10	toll-like receptor 10
57458	TMCC3	transmembrane and coiled-coil domain family 3
23423	TMED3	transmembrane emp24 protein transport domain containing 3
66000	TMEM108	transmembrane protein 108
115650	TNFRSF13C	tumor necrosis factor receptor superfamily, member 13C
7133	TNFRSF1B	tumor necrosis factor receptor superfamily, member 1B
93492	TPTE2	transmembrane phosphoinositide 3-phosphatase and tensin homolog 2
6965	TRG@	T cell receptor gamma locus
10221	TRIB1	tribbles homolog 1 (Drosophila)
100126474	TRNAF-GAA	transfer RNA phenylalanine (anticodon GAA)
7226	TRPM2	transient receptor potential cation channel, subfamily M, member 2

7106	TSPAN4	tetraspanin 4
284900	TTC28-AS1	TTC28 antisense RNA 1 (non-protein coding)
92104	TTC30A	tetratricopeptide repeat domain 30A
51807	TUBA8	tubulin, alpha 8
7368	UGT8	UDP glycosyltransferase 8
11326	VSIG4	V-set and immunoglobulin domain containing 4
7450	VWF	von Willebrand factor
144406	WDR66	WD repeat domain 66
25937	WWTR1	WW domain containing transcription regulator 1
129446	XIRP2	xin actin-binding repeat containing 2
646016	YWHAZP8	tyrosine 3-monooxygenase/tryptophan 5-monooxygenase activation protein, zeta pseudogene 8
389874	ZCCHC13	zinc finger, CCHC domain containing 13
219654	ZCCHC24	zinc finger, CCHC domain containing 24
9372	ZFYVE9	zinc finger, FYVE domain containing 9
7712	ZNF157	zinc finger protein 157
7761	ZNF214	zinc finger protein 214
51222	ZNF219	zinc finger protein 219
619279	ZNF704	zinc finger protein 704
100130832	[Not Available]	[Not Available]
100129978	[Not Available]	[Not Available]
100132092	[Not Available]	[Not Available]
643599	[Not Available]	[Not Available]
100130229	[Not Available]	[Not Available]
100129146	[Not Available]	[Not Available]
729715	[Not Available]	[Not Available]
729538	[Not Available]	[Not Available]
100130565	[Not Available]	[Not Available]
731797	[Not Available]	[Not Available]
100131269	[Not Available]	[Not Available]
728708	[Not Available]	[Not Available]

100130509	[Not Available]	[Not Available]
100131371	[Not Available]	[Not Available]
729653	[Not Available]	[Not Available]
100132575	[Not Available]	[Not Available]
728942	[Not Available]	[Not Available]
