# Supplementary material for: Gene-expression patterns in peripheral blood classify familial breast cancer susceptibility
Source: BMC Med Genomics. 2015 Nov 4;8:72. doi: 10.1186/s12920-015-0145-6 (PMC4634735; doi:10.1186/s12920-015-0145-6)
Supplement: Additional file 7: — Genes selected for the first half of the Ontario cohort. Genes selected via SVM-RFE from the Utah cohort for the Ontario (36 samples) biomarker predictions. (PDF 53 kb) [file 12920_2015_145_MOESM7_ESM.pdf]

Entrez Gene ID	Gene Symbol	Gene Name
87688	RPL7AP50	ribosomal protein L7a pseudogene 50
391106	VDAC1P9	voltage-dependent anion channel 1 pseudogene 9
100128709	[No Symbol]	[No Name]
646272	LOC646272	cytochrome b-c1 complex subunit 8-like
100128700	[No Symbol]	[No Name]
286495	TTC3P1	tetratricopeptide repeat domain 3 pseudogene 1
392282	RPS5P6	ribosomal protein S5 pseudogene 6
541468	C1orf190	chromosome 1 open reading frame 190
83604	TMEM47	transmembrane protein 47
400954	EML6	echinoderm microtubule associated protein like 6
647298	HSPD1P8	heat shock 60kDa protein 1 (chaperonin) pseudogene 8
100132626	LOC100132626	protein FAM103A1-like
440278	CATSPER2P1	cation channel, sperm associated 2 pseudogene 1
100128493	LOC100128493	ubiquitin-conjugating enzyme E2 variant 2 pseudogene
390155	OR5T1	olfactory receptor, family 5, subfamily T, member 1
128774	MRPS11P1	mitochondrial ribosomal protein S11 pseudogene 1
641518	LOC641518	hypothetical LOC641518
149157	[No Symbol]	[No Name]
7473	WNT3	wingless-type MMTV integration site family, member 3
54436	SH3TC1	SH3 domain and tetratricopeptide repeats 1
5999	RGS4	regulator of G-protein signaling 4
28869	IGKV6D-41	immunoglobulin kappa variable 6D-41 (non-functional)
55604	LRRC16A	leucine rich repeat containing 16A
449518	LOC449518	purinergic receptor P2Y, G-protein coupled, 10 pseudogene
79825	CCDC48	coiled-coil domain containing 48
3809	KIR2DS4	killer cell immunoglobulin-like receptor, two domains, short cytoplasmic tail, 4
442673	TUBG1P	tubulin, gamma 1 pseudogene
51266	CLEC1B	C-type lectin domain family 1, member B
100129822	[No Symbol]	[No Name]
100131609	HNRNPA1P2	heterogeneous nuclear ribonucleoprotein A1 pseudogene 2
389428	RPL5P18	ribosomal protein L5 pseudogene 18
100128386	LOC100128386	hypothetical LOC100128386

55270	NUDT15	nudix (nucleoside diphosphate linked moiety X)-type motif 15
649489	LOC649489	protein phosphatase 1, regulatory (inhibitor) subunit 2 pseudogene
643586	LOC643586	pyruvate kinase, muscle pseudogene
339778	C2orf70	chromosome 2 open reading frame 70
348825	TPRXL	tetra-peptide repeat homeobox-like
221016	CCDC7	coiled-coil domain containing 7
647034	RPS14P10	ribosomal protein S14 pseudogene 10
3805	KIR2DL4	killer cell immunoglobulin-like receptor, two domains, long cytoplasmic tail, 4
283314	MATL2963	hypothetical LOC283314
7380	UPK3A	uroplakin 3A
408029	C2orf27B	chromosome 2 open reading frame 27B
10461	MERTK	c-mer proto-oncogene tyrosine kinase
642677	LOC642677	family with sequence similarity 154, member B pseudogene
10877	CFHR4	complement factor H-related 4
283571	PROX2	prospero homeobox 2
340547	VSIG1	V-set and immunoglobulin domain containing 1
100129915	[No Symbol]	[No Name]
3426	CFI	complement factor I
51499	TRIAP1	TP53 regulated inhibitor of apoptosis 1
780813	PAICSP4	phosphoribosylaminoimidazole carboxylase, phosphoribosylaminoimidazole succinocarboxamide synthetase pseudogene 4
728707	[No Symbol]	[No Name]
130813	C2orf50	chromosome 2 open reading frame 50
100132310	LOC100132310	FCF1 small subunit (SSU) processome component homolog (S. cerevisiae) pseudogene
729486	IL9RP3	interleukin 9 receptor pseudogene 3
401433	LOC401433	hypothetical LOC401433
286122	C8orf31	chromosome 8 open reading frame 31
219902	TMEM136	transmembrane protein 136
199713	NLRP7	NLR family, pyrin domain containing 7
390282	LOC390282	eukaryotic translation initiation factor 3, subunit F pseudogene
1769	DNAH8	dynein, axonemal, heavy chain 8
100130249	PP2672	hypothetical LOC100130249
163778	SPRR4	small proline-rich protein 4
148641	SLC35F3	solute carrier family 35, member F3

400347	LOC400347	REX4, RNA exonuclease 4 homolog (S. cerevisiae) pseudogene
347333	KRT8P14	keratin 8 pseudogene 14
100128646	RPL10AP7	ribosomal protein L10a pseudogene 7
54035	PSMD4P1	proteasome (prosome, macropain) 26S subunit, non-ATPase, 4 pseudogene 1
8228	PNPLA4	patatin-like phospholipase domain containing 4
9363	RAB33A	RAB33A, member RAS oncogene family
344887	LOC344887	NmrA-like family domain containing 1 pseudogene
121270	OR11M1P	olfactory receptor, family 11, subfamily M, member 1 pseudogene
339736	AK2P2	adenylate kinase 2 pseudogene 2
6461	SHB	Src homology 2 domain containing adaptor protein B
4744	NEFH	neurofilament, heavy polypeptide
90499	LOC90499	hypothetical protein LOC90499
729041	LOC729041	fatty-acid amide hydrolase 1-like
2335	FN1	fibronectin 1
79625	C4orf31	chromosome 4 open reading frame 31
644915	METTL15P2	methyltransferase like 15 pseudogene 2
100132086	LOC100132086	adenylate kinase isoenzyme 6-like
2596	GAP43	growth associated protein 43
326617	PSMA3P	proteasome (prosome, macropain) subunit, alpha type, 3 pseudogene
646576	LOC646576	hypothetical LOC646576
3812	KIR3DL2	killer cell immunoglobulin-like receptor, three domains, long cytoplasmic tail, 2
100129958	KRT8P44	keratin 8 pseudogene 44
644662	LOC644662	hypothetical protein LOC644662
5054	SERPINE1	serpin peptidase inhibitor, clade E (nexin, plasminogen activator inhibitor type 1), member 1
339983	NAT8L	N-acetyltransferase 8-like (GCN5-related, putative)
100037267	LOC100037267	developmental pluripotency associated 4 pseudogene
130013	ACMSD	aminocarboxymuconate semialdehyde decarboxylase
219623	TMEM26	transmembrane protein 26
9241	NOG	noggin
100128050	LOC100128050	WD repeat domain 77 pseudogene
440603	BCL2L15	BCL2-like 15
152078	C3orf55	chromosome 3 open reading frame 55
201895	C4orf34	chromosome 4 open reading frame 34

100127889	C10orf131	chromosome 10 open reading frame 131
221711	SYCP2L	synaptonemal complex protein 2-like
8829	NRP1	neuropilin 1
1472	CST4	cystatin S
729451	LOC729451	hypothetical protein LOC729451
100128389	[No Symbol]	[No Name]
201798	TIGD4	tigger transposable element derived 4
118663	BTBD16	BTB (POZ) domain containing 16
6887	TAL2	T-cell acute lymphocytic leukemia 2
64410	KLHL25	kelch-like 25 (Drosophila)
442524	DPY19L2P3	dpy-19-like 2 pseudogene 3 (C. elegans)
259286	TAS2R40	taste receptor, type 2, member 40
731039	[No Symbol]	[No Name]
50835	TAS2R9	taste receptor, type 2, member 9
4157	MC1R	melanocortin 1 receptor (alpha melanocyte stimulating hormone receptor)
401703	LOC401703	splicing factor U2AF 35 kDa subunit-like
785	CACNB4	calcium channel, voltage-dependent, beta 4 subunit
100130268	LOC100130268	similar to hCG1648866
100130859	[No Symbol]	[No Name]
100128457	LOC100128457	similar to hCG2026341
100132214	[No Symbol]	[No Name]
26	ABP1	amiloride binding protein 1 (amine oxidase (copper-containing))
51676	ASB2	ankyrin repeat and SOCS box containing 2
27145	FILIP1	filamin A interacting protein 1
347051	SLC10A5	solute carrier family 10 (sodium/bile acid cotransporter family), member 5
100131819	[No Symbol]	[No Name]
728780	ANKDD1B	ankyrin repeat and death domain containing 1B
