# Supplementary material for: Gene-expression patterns in peripheral blood classify familial breast cancer susceptibility
Source: BMC Med Genomics. 2015 Nov 4;8:72. doi: 10.1186/s12920-015-0145-6 (PMC4634735; doi:10.1186/s12920-015-0145-6)
Supplement: Additional file 8: — Genes selected for the second half of the Ontario cohort. Genes selected via SVM-RFE from the Utah cohort for the Ontario (remaining samples) biomarker predictions. (PDF 67 kb) [file 12920_2015_145_MOESM8_ESM.pdf]

Entrez Gene ID	Gene Symbol	Gene Name
541468	C1orf190	chromosome 1 open reading frame 190
55057	AIM1L	absent in melanoma 1-like
221016	CCDC7	coiled-coil domain containing 7
7012	TERC	telomerase RNA component
148641	SLC35F3	solute carrier family 35, member F3
55270	NUDT15	nudix (nucleoside diphosphate linked moiety X)-type motif 15
647298	HSPD1P8	heat shock 60kDa protein 1 (chaperonin) pseudogene 8
392282	RPS5P6	ribosomal protein S5 pseudogene 6
728707	[No Symbol]	[No Name]
389428	RPL5P18	ribosomal protein L5 pseudogene 18
730255	RPL17P8	ribosomal protein L17 pseudogene 8
100130249	PP2672	hypothetical LOC100130249
4112	MAGEB1	melanoma antigen family B, 1
163778	SPRR4	small proline-rich protein 4
157927	C9orf62	chromosome 9 open reading frame 62
100129822	[No Symbol]	[No Name]
83604	TMEM47	transmembrane protein 47
729486	IL9RP3	interleukin 9 receptor pseudogene 3
390282	LOC390282	eukaryotic translation initiation factor 3, subunit F pseudogene
197370	NSMCE1	non-SMC element 1 homolog (S. cerevisiae)
728509	RPS19P7	ribosomal protein S19 pseudogene 7
286122	C8orf31	chromosome 8 open reading frame 31
442524	DPY19L2P3	dpy-19-like 2 pseudogene 3 (C. elegans)
647034	RPS14P10	ribosomal protein S14 pseudogene 10
400954	EML6	echinoderm microtubule associated protein like 6
339778	C2orf70	chromosome 2 open reading frame 70
100128700	[No Symbol]	[No Name]
730126	[No Symbol]	[No Name]
644662	LOC644662	hypothetical protein LOC644662
642677	LOC642677	family with sequence similarity 154, member B pseudogene
785	CACNB4	calcium channel, voltage-dependent, beta 4 subunit
149157	[No Symbol]	[No Name]

100128050	LOC100128050	WD repeat domain 77 pseudogene
729451	LOC729451	hypothetical protein LOC729451
51266	CLEC1B	C-type lectin domain family 1, member B
100128646	RPL10AP7	ribosomal protein L10a pseudogene 7
148645	C1orf211	chromosome 1 open reading frame 211
440278	CATSPER2P1	cation channel, sperm associated 2 pseudogene 1
653492	PSG10P	pregnancy specific beta-1-glycoprotein 10, pseudogene
347333	KRT8P14	keratin 8 pseudogene 14
391106	VDAC1P9	voltage-dependent anion channel 1 pseudogene 9
100128386	LOC100128386	hypothetical LOC100128386
441502	RPS26P11	ribosomal protein S26 pseudogene 11
100132086	LOC100132086	adenylate kinase isoenzyme 6-like
121270	OR11M1P	olfactory receptor, family 11, subfamily M, member 1 pseudogene
79022	TMEM106C	transmembrane protein 106C
408029	C2orf27B	chromosome 2 open reading frame 27B
6461	SHB	Src homology 2 domain containing adaptor protein B
27145	FILIP1	filamin A interacting protein 1
401433	LOC401433	hypothetical LOC401433
100131042	[No Symbol]	[No Name]
646576	LOC646576	hypothetical LOC646576
10877	CFHR4	complement factor H-related 4
326617	PSMA3P	proteasome (prosome, macropain) subunit, alpha type, 3 pseudogene
3394	IRF8	interferon regulatory factor 8
100128493	LOC100128493	ubiquitin-conjugating enzyme E2 variant 2 pseudogene
440603	BCL2L15	BCL2-like 15
344887	LOC344887	NmrA-like family domain containing 1 pseudogene
728050	[No Symbol]	[No Name]
645086	LOC645086	chromosome 11 open reading frame 58 pseudogene
649288	AK4P6	adenylate kinase 4 pseudogene 6
3872	KRT17	keratin 17
100128979	LOC100128979	hypothetical LOC100128979
100128457	LOC100128457	similar to hCG2026341
283553	LOC283553	hypothetical LOC283553

1823	DSC1	desmocollin 1
199713	NLRP7	NLR family, pyrin domain containing 7
91392	ZNF502	zinc finger protein 502
130813	C2orf50	chromosome 2 open reading frame 50
219623	TMEM26	transmembrane protein 26
2596	GAP43	growth associated protein 43
441505	LOC441505	stress-induced-phosphoprotein 1 pseudogene
643586	LOC643586	pyruvate kinase, muscle pseudogene
449518	LOC449518	purinergic receptor P2Y, G-protein coupled, 10 pseudogene
3426	CFI	complement factor I
128774	MRPS11P1	mitochondrial ribosomal protein S11 pseudogene 1
87688	RPL7AP50	ribosomal protein L7a pseudogene 50
7380	UPK3A	uroplakin 3A
84332	DYDC2	DPY30 domain containing 2
100129915	[No Symbol]	[No Name]
100132805	[No Symbol]	[No Name]
342666	FLJ43826	FLJ43826 protein
6862	T	T, brachyury homolog (mouse)
348825	TPRXL	tetra-peptide repeat homeobox-like
1472	CST4	cystatin S
388182	FLJ42289	hypothetical LOC388182
4342	MOS	v-mos Moloney murine sarcoma viral oncogene homolog
78998	C8orf51	chromosome 8 open reading frame 51
9241	NOG	noggin
780813	PAICSP4	phosphoribosylaminoimidazole carboxylase, phosphoribosylaminoimidazole succinocarboxamide synthetase pseudogene 4
26	ABP1	amiloride binding protein 1 (amine oxidase (copper-containing))
55540	IL17RB	interleukin 17 receptor B
100128709	[No Symbol]	[No Name]
100130268	LOC100130268	similar to hCG1648866
100128389	[No Symbol]	[No Name]
353194	LOC353194	keratin pseudogene
100131609	HNRNPA1P2	heterogeneous nuclear ribonucleoprotein A1 pseudogene 2
130500	CISD1P1	CDGSH iron sulfur domain 1 pseudogene 1

26256	CABYR	calcium binding tyrosine-(Y)-phosphorylation regulated
100127889	C10orf131	chromosome 10 open reading frame 131
100128417	[No Symbol]	[No Name]
79625	C4orf31	chromosome 4 open reading frame 31
83844	USP26	ubiquitin specific peptidase 26
128820	CST9LP1	cystatin 9-like pseudogene 1
100132214	[No Symbol]	[No Name]
26647	OR7E25P	olfactory receptor, family 7, subfamily E, member 25 pseudogene
8228	PNPLA4	patatin-like phospholipase domain containing 4
26590	OR8B7P	olfactory receptor, family 8, subfamily B, member 7 pseudogene
729041	LOC729041	fatty-acid amide hydrolase 1-like
100130859	[No Symbol]	[No Name]
221711	SYCP2L	synaptonemal complex protein 2-like
400165	C13orf35	chromosome 13 open reading frame 35
4157	MC1R	melanocortin 1 receptor (alpha melanocyte stimulating hormone receptor)
3809	KIR2DS4	killer cell immunoglobulin-like receptor, two domains, short cytoplasmic tail, 4
9288	TAAR3	trace amine associated receptor 3 (gene/pseudogene)
10461	MERTK	c-mer proto-oncogene tyrosine kinase
100129958	KRT8P44	keratin 8 pseudogene 44
100130321	LOC100130321	DNA fragmentation factor, 45kDa, alpha polypeptide pseudogene
100132626	LOC100132626	protein FAM103A1-like
729011	[No Symbol]	[No Name]
649489	LOC649489	protein phosphatase 1, regulatory (inhibitor) subunit 2 pseudogene
4744	NEFH	neurofilament, heavy polypeptide
7070	THY1	Thy-1 cell surface antigen
7644	ZNF91	zinc finger protein 91
144715	RAD9B	RAD9 homolog B (S. pombe)
653194	LOC653194	KH homology domain-containing protein 1-like
7473	WNT3	wingless-type MMTV integration site family, member 3
283314	MATL2963	hypothetical LOC283314
728545	[No Symbol]	[No Name]
151825	KRT18P43	keratin 18 pseudogene 43
647532	LOC647532	phenylalanine-tRNA synthetase-like, beta subunit pseudogene

644862	RPS28P3	ribosomal protein S28 pseudogene 3
100128403	[No Symbol]	[No Name]
3626	INHBC	inhibin, beta C
347051	SLC10A5	solute carrier family 10 (sodium/bile acid cotransporter family), member 5
283571	PROX2	prospero homeobox 2
100132510	GLRXP3	glutaredoxin (thioltransferase) pseudogene 3
25925	ZNF521	zinc finger protein 521
28869	IGKV6D-41	immunoglobulin kappa variable 6D-41 (non-functional)
387856	C12orf68	chromosome 12 open reading frame 68
606495	CYB5RL	cytochrome b5 reductase-like
644588	DNAJA1P3	DnaJ (Hsp40) homolog, subfamily A, member 1 pseudogene 3
56834	GPR137	G protein-coupled receptor 137
100130184	[No Symbol]	[No Name]
574447	MIR146B	microRNA 146b
8626	TP63	tumor protein p63
286495	TTC3P1	tetratricopeptide repeat domain 3 pseudogene 1
387924	OGFOD1P1	2-oxoglutarate and iron-dependent oxygenase domain containing 1 pseudogene 1
55702	CCDC94	coiled-coil domain containing 94
390844	ARIH2P1	ariadne homolog 2 pseudogene 1
5054	SERPINE1	serpin peptidase inhibitor, clade E (nexin, plasminogen activator inhibitor type 1), member 1
641518	LOC641518	hypothetical LOC641518
4085	MAD2L1	MAD2 mitotic arrest deficient-like 1 (yeast)
100131070	LOC100131070	mpv17-like protein 2-like
338094	FAM151A	family with sequence similarity 151, member A
388507	ZNF788	zinc finger family member 788
7164	TPD52L1	tumor protein D52-like 1
339983	NAT8L	N-acetyltransferase 8-like (GCN5-related, putative)
57570	TRMT5	TRM5 tRNA methyltransferase 5 homolog (S. cerevisiae)
100132053	RPL30P8	ribosomal protein L30 pseudogene 8
100131750	[No Symbol]	[No Name]
285231	FBXW12	F-box and WD repeat domain containing 12
400347	LOC400347	REX4, RNA exonuclease 4 homolog (S. cerevisiae) pseudogene
6887	TAL2	T-cell acute lymphocytic leukemia 2

100131602	[No Symbol]	[No Name]
3812	KIR3DL2	killer cell immunoglobulin-like receptor, three domains, long cytoplasmic tail, 2
3625	INHBB	inhibin, beta B
201895	C4orf34	chromosome 4 open reading frame 34
56891	LGALS14	lectin, galactoside-binding, soluble, 14
442673	TUBG1P	tubulin, gamma 1 pseudogene
2841	GPR18	G protein-coupled receptor 18
392301	SLC25A5P8	solute carrier family 25 (mitochondrial carrier; adenine nucleotide translocator), member 5 pseudogene 8
414318	C9orf106	chromosome 9 open reading frame 106
645233	LOC645233	thymine-DNA glycosylase pseudogene
23439	ATP1B4	ATPase, Na+/K+ transporting, beta 4 polypeptide
219902	TMEM136	transmembrane protein 136
166752	FREM3	FRAS1 related extracellular matrix 3
79825	CCDC48	coiled-coil domain containing 48
54035	PSMD4P1	proteasome (prosome, macropain) 26S subunit, non-ATPase, 4 pseudogene 1
646272	LOC646272	cytochrome b-c1 complex subunit 8-like
643669	LOC643669	hypothetical protein LOC643669
145259	RPSAP4	ribosomal protein SA pseudogene 4
2335	FN1	fibronectin 1
81849	ST6GALNAC5	ST6 (alpha-N-acetyl-neuraminyl-2,3-beta-galactosyl-1,3)-N-acetylgalactosaminide alpha-2,6-sialyltransferase 5
100132073	LOC100132073	cyclin B2 pseudogene
100133337	[No Symbol]	[No Name]
100130623	[No Symbol]	[No Name]
100132310	LOC100132310	FCF1 small subunit (SSU) processome component homolog (S. cerevisiae) pseudogene
5988	RFPL1	ret finger protein-like 1
84099	ID2B	inhibitor of DNA binding 2B, dominant negative helix-loop-helix protein (pseudogene)
644464	RPSAP61	ribosomal protein SA pseudogene 61
148823	C1orf150	chromosome 1 open reading frame 150
130162	C2orf63	chromosome 2 open reading frame 63
23754	RPL32P5	ribosomal protein L32 pseudogene 5
10316	NMUR1	neuromedin U receptor 1
401703	LOC401703	splicing factor U2AF 35 kDa subunit-like
1266	CNN3	calponin 3, acidic

728780	ANKDD1B	ankyrin repeat and death domain containing 1B
643182	LOC643182	upstream binding transcription factor, RNA polymerase I pseudogene
81889	FAHD1	fumarylacetoacetate hydrolase domain containing 1
344807	CD200R1L	CD200 receptor 1-like
140691	TRIM69	tripartite motif containing 69
644915	METTL15P2	methyltransferase like 15 pseudogene 2
139411	PTCHD1	patched domain containing 1
339736	AK2P2	adenylate kinase 2 pseudogene 2
118663	BTBD16	BTB (POZ) domain containing 16
64410	KLHL25	kelch-like 25 (Drosophila)
28466	IGHV1-45	immunoglobulin heavy variable 1-45
285987	DLX6-AS1	DLX6 antisense RNA 1 (non-protein coding)
130013	ACMSD	aminocarboxymuconate semialdehyde decarboxylase
259286	TAS2R40	taste receptor, type 2, member 40
9363	RAB33A	RAB33A, member RAS oncogene family
729950	LOC729950	hypothetical LOC729950
645474	S100A11P3	S100 calcium binding protein A11 pseudogene 3
653712	LOC653712	intraflagellar transport 122 homolog (Chlamydomonas) pseudogene
81050	OR5AC2	olfactory receptor, family 5, subfamily AC, member 2
3226	HOXC10	homeobox C10
392387	LOC392387	adenosylhomocysteinase pseudogene
126259	TMIGD2	transmembrane and immunoglobulin domain containing 2
81431	OR5AC1	olfactory receptor, family 5, subfamily AC, member 1 (gene/pseudogene)
79696	FAM164C	family with sequence similarity 164, member C
100131087	RPLP1P11	ribosomal protein, large, P1 pseudogene 11
644941	[No Symbol]	[No Name]
100127983	LOC100127983	hypothetical protein LOC100127983
51499	TRIAP1	TP53 regulated inhibitor of apoptosis 1
