# Supplementary material for: Gene-expression patterns in peripheral blood classify familial breast cancer susceptibility
Source: BMC Med Genomics. 2015 Nov 4;8:72. doi: 10.1186/s12920-015-0145-6 (PMC4634735; doi:10.1186/s12920-015-0145-6)
Supplement: Additional file 9: — Gene-level summary of expression data for Utah and Ontario cohorts. Fold change values represent the ratio of average gene expression for FBC individuals who developed cancer relative to expression levels for those who did not. (PDF 4554 kb) [file 12920_2015_145_MOESM9_ESM.pdf]

Entrez Gene ID	Gene Symbol	Gene Name	Utah Fold Change	Ontario1 Fold Change	Ontario2 Fold Change
1	A1BG	alpha-1-B glycoprotein	1.00294	1.017742	1.0005154
2	A2M	alpha-2-macroglobulin	1.00411	1.043409	0.9854786
3	A2MP1	alpha-2-macroglobulin pseudogene 1	1.00714	1.019343	0.979932
9	NAT1	N-acetyltransferase 1 (arylamine N-acetyltransferase)	1.00886	1.035319	1.0534696
10	NAT2	N-acetyltransferase 2 (arylamine N-acetyltransferase)	1.02116	0.996818	1.0158625
12	SERPINA3	serpin peptidase inhibitor, clade A (alpha-1 antiproteinase, antitrypsin), member 3	1	0.993337	0.9881893
13	AADAC	arylacetamide deacetylase (esterase)	1.01694	1.004655	0.9851908
14	AAMP	angio-associated, migratory cell protein	0.98094	1.060761	1.0473432
16	AARS	alanyl-tRNA synthetase	0.92167	1.015415	0.9637201
18	ABAT	4-aminobutyrate aminotransferase	0.97401	0.982356	0.9522662
19	ABCA1	ATP-binding cassette, sub-family A (ABC1), member 1	0.98259	0.969104	0.9231655
20	ABCA2	ATP-binding cassette, sub-family A (ABC1), member 2	0.98173	1.096359	0.9516605
21	ABCA3	ATP-binding cassette, sub-family A (ABC1), member 3	0.98923	0.976318	1.0349985
22	ABCB7	ATP-binding cassette, sub-family B (MDR/TAP), member 7	0.93684	1.047134	1.0995226
23	ABCF1	ATP-binding cassette, sub-family F (GCN20), member 1	0.9854	1.037686	0.9401831
24	ABCA4	ATP-binding cassette, sub-family A (ABC1), member 4	1.00281	0.980401	1.021358
25	ABL1	c-abl oncogene 1, non-receptor tyrosine kinase	0.97001	1.004426	1.0064301
26	ABP1	amiloride binding protein 1 (amine oxidase (copper-containing))	0.99668	0.975276	1.0399611
27	ABL2	v-abl Abelson murine leukemia viral oncogene homolog 2	0.98972	0.953148	0.9536299
28	ABO	ABO blood group (transferase A, alpha 1-3-N-acetylgalactosaminyltransferase; transferase B, alpha 1-3-galac	0.99212	0.990283	1.0371028
29	ABR	active BCR-related gene	0.98438	1.008736	1.0290757
30	ACAA1	acetyl-CoA acyltransferase 1	1.008	0.979358	1.01555
31	ACACA	acetyl-CoA carboxylase alpha	0.97848	0.950874	1.0133888
32	ACACB	acetyl-CoA carboxylase beta	0.92524	0.935984	0.9538425
33	ACADL	acyl-CoA dehydrogenase, long chain	0.99479	1.002312	1.0384004
34	ACADM	acyl-CoA dehydrogenase, C-4 to C-12 straight chain	0.98478	0.967459	1.0032962
35	ACADS	acyl-CoA dehydrogenase, C-2 to C-3 short chain	0.98771	0.956493	1.0516386
36	ACADSB	acyl-CoA dehydrogenase, short/branched chain	0.96009	0.951001	1.0856614
37	ACADVL	acyl-CoA dehydrogenase, very long chain	0.96486	1.073101	1.0444307
38	ACAT1	acetyl-CoA acetyltransferase 1	0.92191	1.022633	1.0036621

39	ACAT2	acetyl-CoA acetyltransferase 2	0.94602	0.98404	1.0251256
40	ACCN1	amiloride-sensitive cation channel 1, neuronal	0.99117	1.005837	1.0032437
41	ACCN2	amiloride-sensitive cation channel 2, neuronal	0.98901	0.993842	1.002503
43	ACHE	acetylcholinesterase	0.9994	1.003815	1.0462938
47	ACLY	ATP citrate lyase	0.9682	0.89278	1.0830621
48	ACO1	aconitase 1, soluble	0.93401	0.960291	1.06345
49	ACR	acrosin	1.0262	0.996619	0.9989781
50	ACO2	aconitase 2, mitochondrial	0.96606	0.95671	1.0354455
51	ACOX1	acyl-CoA oxidase 1, palmitoyl	0.91206	0.966155	0.9859762
52	ACP1	acid phosphatase 1, soluble	1.02886	0.882737	0.9975123
53	ACP2	acid phosphatase 2, lysosomal	0.94831	0.924376	1.0079128
54	ACP5	acid phosphatase 5, tartrate resistant	0.92148	0.918024	1.0501368
55	ACPP	acid phosphatase, prostate	1.02406	0.913578	1.0242479
56	ACRV1	acrosomal vesicle protein 1	1.01507	1.01298	0.9809299
58	ACTA1	actin, alpha 1, skeletal muscle	0.98151	1.02286	1.0819241
59	ACTA2	actin, alpha 2, smooth muscle, aorta	1.01112	1.010855	0.9939676
60	ACTB	actin, beta	1.01322	0.950804	0.9311895
62	ACTBP2	actin, beta pseudogene 2	1.01957	0.907973	1.0476614
68	ACTBP8	actin, beta pseudogene 8	0.98812	0.869139	1.0487058
70	ACTC1	actin, alpha, cardiac muscle 1	0.97493	0.986695	1.0187273
71	ACTG1	actin, gamma 1	0.9928	0.978344	0.9926928
72	ACTG2	actin, gamma 2, smooth muscle, enteric	1.00616	1.002068	0.9981948
73	ACTG1P1	actin, gamma 1 pseudogene 1	0.98162	0.971678	1.0466977
81	ACTN4	actinin, alpha 4	0.98979	0.98737	1.0338019
82	ACTG1P9	actin, gamma 1 pseudogene 9	0.99001	0.929508	1.0588137
83	ACTG1P10	actin, gamma 1 pseudogene 10	0.95313	0.952082	1.0106365
86	ACTL6A	actin-like 6A	1.0105	0.943093	1.0328847
87	ACTN1	actinin, alpha 1	0.94502	0.928571	0.9766444
88	ACTN2	actinin, alpha 2	0.98889	0.99074	1.0219255
89	ACTN3	actinin, alpha 3	1.00265	1.01242	1.0036123
90	ACVR1	activin A receptor, type I	1.0159	0.943686	0.9715162
91	ACVR1B	activin A receptor, type IB	0.99123	0.991428	0.9492053
92	ACVR2A	activin A receptor, type IIA	1.00959	0.994506	1.0055328

93	ACVR2B	activin A receptor, type IIB	1.01643	0.973498	1.032956
95	ACY1	aminoacylase 1	0.99357	0.954833	1.0293816
97	ACYP1	acylphosphatase 1, erythrocyte (common) type	0.99128	0.950805	1.024132
98	ACYP2	acylphosphatase 2, muscle type	0.99931	1.033252	0.9922113
100	ADA	adenosine deaminase	0.93593	1.005244	1.0567021
101	ADAM8	ADAM metallopeptidase domain 8	0.95313	1.031952	0.9282514
102	ADAM10	ADAM metallopeptidase domain 10	0.98924	1.039434	1.0597239
103	ADAR	adenosine deaminase, RNA-specific	1.00414	0.934185	1.0874323
104	ADARB1	adenosine deaminase, RNA-specific, B1	0.94639	1.018234	0.9819718
105	ADARB2	adenosine deaminase, RNA-specific, B2	0.99134	0.992399	1.0139659
107	ADCY1	adenylate cyclase 1 (brain)	1.01506	1.004913	1.0144846
108	ADCY2	adenylate cyclase 2 (brain)	1.01029	1.004023	1.0083896
109	ADCY3	adenylate cyclase 3	0.98621	0.990361	0.9670339
111	ADCY5	adenylate cyclase 5	1.00631	1.019202	0.9989865
112	ADCY6	adenylate cyclase 6	0.9902	1.00554	0.9939123
113	ADCY7	adenylate cyclase 7	0.97111	0.971375	1.0214992
114	ADCY8	adenylate cyclase 8 (brain)	0.99235	0.984021	1.0112727
115	ADCY9	adenylate cyclase 9	0.98862	0.98909	0.9673701
116	ADCYAP1	adenylate cyclase activating polypeptide 1 (pituitary)	0.98906	0.999112	1.0132901
117	ADCYAP1R1	adenylate cyclase activating polypeptide 1 (pituitary) receptor type I	1.02572	0.996166	1.0240802
118	ADD1	adducin 1 (alpha)	1.0053	1.074612	0.9329558
119	ADD2	adducin 2 (beta)	0.98939	0.98561	0.9963902
120	ADD3	adducin 3 (gamma)	0.96568	0.992113	0.9544398
123	PLIN2	perilipin 2	0.93291	0.999771	0.9292179
125	ADH1B	alcohol dehydrogenase 1B (class I), beta polypeptide	1.00961	0.989655	1.0542561
126	ADH1C	alcohol dehydrogenase 1C (class I), gamma polypeptide	0.99148	1.027334	1.1039217
127	ADH4	alcohol dehydrogenase 4 (class II), pi polypeptide	1.00198	0.972052	1.0032697
128	ADH5	alcohol dehydrogenase 5 (class III), chi polypeptide	1.0182	0.951643	1.026344
130	ADH6	alcohol dehydrogenase 6 (class V)	1.00896	0.986945	1.0283704
131	ADH7	alcohol dehydrogenase 7 (class IV), mu or sigma polypeptide	1.00753	0.961638	0.9981417
132	ADK	adenosine kinase	0.97788	0.939276	0.986698
133	ADM	adrenomedullin	0.99197	1.005912	1.0134805
134	ADORA1	adenosine A1 receptor	1.02534	1.016203	1.0057782

135	ADORA2A	adenosine A2a receptor	0.93952	1.09769	0.9525285
136	ADORA2B	adenosine A2b receptor	0.98655	0.977561	1.0376623
137	ADORA2BP	adenosine A2b receptor pseudogene 1	1.01695	1.015762	1.0216098
140	ADORA3	adenosine A3 receptor	1.01772	0.985269	1.0198865
141	ADPRH	ADP-ribosylarginine hydrolase	0.97807	0.995609	1.010096
142	PARP1	poly (ADP-ribose) polymerase 1	0.98098	0.988615	1.0481722
143	PARP4	poly (ADP-ribose) polymerase family, member 4	0.96883	0.966313	0.945091
144	PARP1P1	poly (ADP-ribose) polymerase family, member 1 pseudogene 1	0.99452	0.97243	1.0384918
145	PARP1P2	poly (ADP-ribose) polymerase family, member 1 pseudogene 2	0.98076	1.034271	1.1292064
146	ADRA1D	adrenergic, alpha-1D-, receptor	0.99462	1.020986	1.0524807
147	ADRA1B	adrenergic, alpha-1B-, receptor	1.00164	0.986005	1.0169868
148	ADRA1A	adrenergic, alpha-1A-, receptor	0.99473	0.997355	1.0145687
150	ADRA2A	adrenergic, alpha-2A-, receptor	0.9519	0.942197	0.9824387
151	ADRA2B	adrenergic, alpha-2B-, receptor	0.99549	1.03336	1.0672835
152	ADRA2C	adrenergic, alpha-2C-, receptor	0.99583	0.967812	1.0995667
153	ADRB1	adrenergic, beta-1-, receptor	0.98797	0.998223	0.9961574
154	ADRB2	adrenergic, beta-2-, receptor, surface	0.97488	1.087055	0.9203687
155	ADRB3	adrenergic, beta-3-, receptor	0.99701	1.011805	1.0068111
156	ADRBK1	adrenergic, beta, receptor kinase 1	0.98577	1.047438	1.0490009
157	ADRBK2	adrenergic, beta, receptor kinase 2	0.97771	1.030664	0.9801764
158	ADSL	adenylosuccinate lyase	0.92686	1.037906	1.0021877
159	ADSS	adenylosuccinate synthase	1.00624	1.053153	0.9504577
160	AP2A1	adaptor-related protein complex 2, alpha 1 subunit	1.00125	0.992019	1.0311932
161	AP2A2	adaptor-related protein complex 2, alpha 2 subunit	0.97054	0.988372	0.9890022
162	AP1B1	adaptor-related protein complex 1, beta 1 subunit	0.93921	0.930589	1.0015398
163	AP2B1	adaptor-related protein complex 2, beta 1 subunit	0.97955	0.990414	1.0096203
164	AP1G1	adaptor-related protein complex 1, gamma 1 subunit	0.95333	1.026339	1.0694194
165	AEBP1	AE binding protein 1	1.00398	0.99559	1.0104287
166	AES	amino-terminal enhancer of split	0.959	1.000596	1.0357083
167	CRISP1	cysteine-rich secretory protein 1	1.01711	1.028911	1.0472717
172	AFG3L1P	AFG3 ATPase family gene 3-like 1 (S. cerevisiae), pseudogene	0.98384	0.969756	1.0295646
173	AFM	afamin	1.01011	1.001792	0.9950221
174	AFP	alpha-fetoprotein	1.00936	0.99262	1.0024011

175	AGA	aspartylglucosaminidase	0.96535	0.947014	1.1085417
176	ACAN	aggrecan	1.00127	1.003548	1.0058131
177	AGER	advanced glycosylation end product-specific receptor	0.9992	1.025733	0.9859644
178	AGL	amylo-alpha-1, 6-glucosidase, 4-alpha-glucanotransferase	0.9718	0.965225	0.9737766
181	AGRP	agouti related protein homolog (mouse)	0.99853	0.996659	0.9952594
182	JAG1	jagged 1	0.98206	0.959666	1.0327107
183	AGT	angiotensinogen (serpin peptidase inhibitor, clade A, member 8)	1.00766	1.001988	1.0106878
185	AGTR1	angiotensin II receptor, type 1	1.00192	0.987188	0.9822829
186	AGTR2	angiotensin II receptor, type 2	1.00034	0.983289	1.0281857
187	APLNR	apelin receptor	0.96342	1.045398	1.0135084
189	AGXT	alanine-glyoxylate aminotransferase	1.00402	0.97425	1.0328755
190	NR0B1	nuclear receptor subfamily 0, group B, member 1	0.99557	0.983547	1.0296975
191	AHCY	adenosylhomocysteinase	0.95546	0.93759	1.0574586
196	AHR	aryl hydrocarbon receptor	1.00617	0.96484	1.0334323
197	AHSG	alpha-2-HS-glycoprotein	0.99669	1.019758	0.9925137
199	AIF1	allograft inflammatory factor 1	1.03291	0.835644	1.0198744
202	AIM1	absent in melanoma 1	1.00838	1.061088	0.972557
203	AK1	adenylate kinase 1	0.95473	1.042392	1.0334199
204	AK2	adenylate kinase 2	0.99243	1.038831	1.0176058
205	AK4	adenylate kinase 4	0.96919	1.00546	1.0169517
207	AKT1	v-akt murine thymoma viral oncogene homolog 1	0.9739	1.001765	1.0257233
208	AKT2	v-akt murine thymoma viral oncogene homolog 2	0.9714	0.995827	0.9761582
210	ALAD	aminolevulinate dehydratase	0.96816	0.953777	1.0781771
211	ALAS1	aminolevulinate, delta-, synthase 1	0.991	0.92825	0.9637486
212	ALAS2	aminolevulinate, delta-, synthase 2	1.02409	0.8237	0.9707767
213	ALB	albumin	1.01986	0.999625	1.0039726
214	ALCAM	activated leukocyte cell adhesion molecule	0.99408	0.975321	0.9945662
215	ABCD1	ATP-binding cassette, sub-family D (ALD), member 1	0.98427	0.983075	0.9371784
216	ALDH1A1	aldehyde dehydrogenase 1 family, member A1	0.97945	0.82521	0.9903526
218	ALDH3A1	aldehyde dehydrogenase 3 family, member A1	0.99422	0.993279	0.9959987
219	ALDH1B1	aldehyde dehydrogenase 1 family, member B1	0.90777	0.946537	0.9950167
220	ALDH1A3	aldehyde dehydrogenase 1 family, member A3	1.0025	0.999101	1.0189722
221	ALDH3B1	aldehyde dehydrogenase 3 family, member B1	0.99783	0.971193	1.0093736

222	ALDH3B2	aldehyde dehydrogenase 3 family, member B2	0.99578	0.969453	1.0202171
223	ALDH9A1	aldehyde dehydrogenase 9 family, member A1	0.98008	0.935772	1.079307
224	ALDH3A2	aldehyde dehydrogenase 3 family, member A2	0.98207	0.921665	1.0070773
225	ABCD2	ATP-binding cassette, sub-family D (ALD), member 2	0.87636	0.919149	0.8289293
226	ALDOA	aldolase A, fructose-bisphosphate	0.98855	0.983234	1.0283082
228	ALDOAP2	aldolase A, fructose-bisphosphate pseudogene 2	1.0105	1.024446	1.0262357
229	ALDOB	aldolase B, fructose-bisphosphate	1.02474	0.994214	1.0152553
230	ALDOC	aldolase C, fructose-bisphosphate	0.91007	0.962822	1.0085353
231	AKR1B1	aldo-keto reductase family 1, member B1 (aldose reductase)	0.97261	0.953626	0.9668104
236	AKR1B1P2	aldo-keto reductase family 1, member B1 pseudogene 2	0.95177	0.987212	1.0090121
238	ALK	anaplastic lymphoma receptor tyrosine kinase	1.01397	0.999908	1.0071049
239	ALOX12	arachidonate 12-lipoxygenase	0.92063	0.937251	0.9195435
240	ALOX5	arachidonate 5-lipoxygenase	0.97309	0.950391	0.9993698
241	ALOX5AP	arachidonate 5-lipoxygenase-activating protein	0.96651	1.019747	0.9267039
242	ALOX12B	arachidonate 12-lipoxygenase, 12R type	0.98423	0.983093	1.0070922
243	ALOX12P1	arachidonate 12-lipoxygenase pseudogene 1	0.99782	0.98681	0.9944067
245	ALOX12P2	arachidonate 12-lipoxygenase pseudogene 2	0.99455	0.991339	1.0231468
246	ALOX15	arachidonate 15-lipoxygenase	0.99691	1.002254	1.0381591
247	ALOX15B	arachidonate 15-lipoxygenase, type B	0.97864	1.005577	1.0341347
248	ALPI	alkaline phosphatase, intestinal	1.00192	1.000496	1.0151583
249	ALPL	alkaline phosphatase, liver/bone/kidney	0.99788	0.949074	0.9853671
251	ALPPL2	alkaline phosphatase, placental-like 2	1.02749	0.977418	1.0026538
257	ALX3	ALX homeobox 3	0.9934	0.988723	1.0682122
258	AMBN	ameloblastin (enamel matrix protein)	1.00537	0.99678	1.0004239
259	AMBP	alpha-1-microglobulin/bikunin precursor	0.99973	0.977125	1.0173469
262	AMD1	adenosylmethionine decarboxylase 1	1.01683	0.924616	0.9856913
265	AMELX	amelogenin, X-linked	0.98839	1.022672	0.9940353
267	AMFR	autocrine motility factor receptor	0.94711	0.959336	0.932336
268	AMH	anti-Mullerian hormone	0.97454	0.992265	1.0812488
269	AMHR2	anti-Mullerian hormone receptor, type II	1.02081	0.987782	1.0095177
270	AMPD1	adenosine monophosphate deaminase 1	1.0081	1.011163	0.9917326
271	AMPD2	adenosine monophosphate deaminase 2	1.02175	1.024793	0.910522
272	AMPD3	adenosine monophosphate deaminase 3	0.97359	0.983753	0.9953679

273	AMPH	amphiphysin	1.00299	0.997218	1.0205165
274	BIN1	bridging integrator 1	0.99276	1.03317	1.01636
275	AMT	aminomethyltransferase	1.00705	0.987612	1.0392614
280	AMY2B	amylase, alpha 2B (pancreatic)	0.99849	0.992141	0.9794887
284	ANGPT1	angiopoietin 1	0.97039	0.981429	0.9782209
285	ANGPT2	angiopoietin 2	0.99754	1.003802	0.9987189
286	ANK1	ankyrin 1, erythrocytic	0.96329	0.992568	1.0533395
287	ANK2	ankyrin 2, neuronal	1.00649	1.00334	1.0085328
288	ANK3	ankyrin 3, node of Ranvier (ankyrin G)	0.98615	0.853925	0.9050533
290	ANPEP	alanyl (membrane) aminopeptidase	0.9407	1.010947	0.9788408
291	SLC25A4	solute carrier family 25 (mitochondrial carrier; adenine nucleotide translocator), member 4	0.98977	0.997004	1.003861
292	SLC25A5	solute carrier family 25 (mitochondrial carrier; adenine nucleotide translocator), member 5	0.96263	0.953817	0.9735247
301	ANXA1	annexin A1	0.93571	0.823442	0.9975111
302	ANXA2	annexin A2	1.02367	1.004483	1.099651
303	ANXA2P1	annexin A2 pseudogene 1	1.02588	0.958417	1.0128495
305	ANXA2P3	annexin A2 pseudogene 3	1.02834	0.948301	1.0087084
306	ANXA3	annexin A3	1.00982	0.978141	0.9895791
307	ANXA4	annexin A4	1.02194	0.951465	1.0936673
308	ANXA5	annexin A5	1.04504	1.070608	1.0746489
309	ANXA6	annexin A6	1.00423	0.96781	1.0592234
310	ANXA7	annexin A7	0.95173	0.994504	1.0248077
311	ANXA11	annexin A11	0.96304	1.025144	1.0325815
312	ANXA13	annexin A13	1.01271	0.998301	0.9995863
313	AOAH	acyloxyacyl hydrolase (neutrophil)	1.06184	0.964333	0.9730703
314	AOC2	amine oxidase, copper containing 2 (retina-specific)	0.97576	1.031404	0.9786358
316	AOX1	aldehyde oxidase 1	1.01637	1.014665	0.9990813
317	APAF1	apoptotic peptidase activating factor 1	0.97905	0.923826	1.0285839
318	NUDT2	nudix (nucleoside diphosphate linked moiety X)-type motif 2	0.96568	0.955923	1.0706664
319	APOF	apolipoprotein F	1.0041	0.982282	0.9637718
320	APBA1	amyloid beta (A4) precursor protein-binding, family A, member 1	0.98934	0.992722	0.9892075
321	APBA2	amyloid beta (A4) precursor protein-binding, family A, member 2	0.94011	0.947513	0.9239147
322	APBB1	amyloid beta (A4) precursor protein-binding, family B, member 1 (Fe65)	0.93384	0.923528	0.9443916
323	APBB2	amyloid beta (A4) precursor protein-binding, family B, member 2	1.01548	0.972812	1.0241773

324	APC	adenomatous polyposis coli	1.00822	0.9505	1.0767672
325	APCS	amyloid P component, serum	0.99942	0.936306	1.024948
326	AIRE	autoimmune regulator	1.0027	1.011472	1.055236
327	APEH	N-acylaminoacyl-peptide hydrolase	0.98145	0.916855	1.0761039
328	APEX1	APEX nuclease (multifunctional DNA repair enzyme) 1	0.88732	0.899491	0.9836948
329	BIRC2	baculoviral IAP repeat containing 2	0.9652	1.02512	1.03503
330	BIRC3	baculoviral IAP repeat containing 3	0.98162	1.031772	1.0143727
331	XIAP	X-linked inhibitor of apoptosis	0.96887	0.986909	1.0284692
332	BIRC5	baculoviral IAP repeat containing 5	0.98786	0.974592	1.0096194
333	APLP1	amyloid beta (A4) precursor-like protein 1	0.99283	0.973203	1.0084225
334	APLP2	amyloid beta (A4) precursor-like protein 2	1.01676	0.968018	0.9812463
335	APOA1	apolipoprotein A-I	0.99177	1.036897	1.0274709
336	APOA2	apolipoprotein A-II	1.01835	1.009506	0.9750119
337	APOA4	apolipoprotein A-IV	0.99484	1.009669	1.0212674
338	APOB	apolipoprotein B (including Ag(x) antigen)	1.01339	0.99767	1.0195557
339	APOBEC1	apolipoprotein B mRNA editing enzyme, catalytic polypeptide 1	0.99919	0.998043	0.9523729
341	APOC1	apolipoprotein C-I	1.0081	0.975958	1.0566857
343	AQP8	aquaporin 8	1.0122	0.983456	1.019807
344	APOC2	apolipoprotein C-II	1.00124	1.02726	0.9279
345	APOC3	apolipoprotein C-III	1.00279	0.964308	0.9889753
346	APOC4	apolipoprotein C-IV	0.9806	1.037944	0.9871692
347	APOD	apolipoprotein D	1.01056	1.024404	0.9966874
348	APOE	apolipoprotein E	1.01564	0.989012	1.0269529
350	APOH	apolipoprotein H (beta-2-glycoprotein I)	0.99689	1.027997	1.0388005
351	APP	amyloid beta (A4) precursor protein	0.9844	0.982694	0.9768908
353	APRT	adenine phosphoribosyltransferase	0.98308	0.96359	1.0346713
354	KLK3	kallikrein-related peptidase 3	1.00467	1.014367	0.9513236
355	FAS	Fas (TNF receptor superfamily, member 6)	0.98657	1.030221	0.9453157
356	FASLG	Fas ligand (TNF superfamily, member 6)	1.00442	1.034334	0.9444636
357	SHROOM2	shroom family member 2	1.00638	1.000754	0.989312
358	AQP1	aquaporin 1 (Colton blood group)	0.98769	0.99443	1.010658
359	AQP2	aquaporin 2 (collecting duct)	0.99685	1.018883	0.9971894
360	AQP3	aquaporin 3 (Gill blood group)	0.91445	0.815054	0.9429032

361	AQP4	aquaporin 4	1.01075	0.986521	0.9736177
362	AQP5	aquaporin 5	0.99382	0.984829	1.0060027
363	AQP6	aquaporin 6, kidney specific	1.00027	1.000998	1.0306364
364	AQP7	aquaporin 7	0.96741	1.045552	0.9903516
366	AQP9	aquaporin 9	1.0047	1.003861	0.943245
367	AR	androgen receptor	0.99946	1.008232	0.9872626
368	ABCC6	ATP-binding cassette, sub-family C (CFTR/MRP), member 6	1.00017	1.017333	1.0116366
369	ARAF	v-raf murine sarcoma 3611 viral oncogene homolog	0.98691	0.957574	0.9852557
372	ARCN1	archain 1	0.96418	0.968625	1.0791248
373	TRIM23	tripartite motif containing 23	0.9801	0.915929	0.8845796
375	ARF1	ADP-ribosylation factor 1	1.0177	0.99647	0.9398092
377	ARF3	ADP-ribosylation factor 3	1.00611	0.984752	1.0361027
378	ARF4	ADP-ribosylation factor 4	1.02746	0.97466	0.9785982
379	ARL4D	ADP-ribosylation factor-like 4D	0.97521	1.004725	0.9728208
381	ARF5	ADP-ribosylation factor 5	0.97493	0.998153	0.9502776
382	ARF6	ADP-ribosylation factor 6	0.98422	0.902158	1.0632219
383	ARG1	arginase, liver	1.00554	0.920876	1.0037165
384	ARG2	arginase, type II	0.9624	0.978081	0.982377
387	RHOA	ras homolog gene family, member A	1.0146	0.987456	0.9074542
388	RHOB	ras homolog gene family, member B	1.01579	1.021024	0.9237079
389	RHOC	ras homolog gene family, member C	1.00027	0.989991	1.0064902
390	RND3	Rho family GTPase 3	1.00443	0.991701	1.0232305
391	RHOG	ras homolog gene family, member G (rho G)	0.98779	0.923515	0.9553472
392	ARHGAP1	Rho GTPase activating protein 1	0.96351	0.881938	1.0434333
393	ARHGAP4	Rho GTPase activating protein 4	0.9649	1.035279	0.9379761
394	ARHGAP5	Rho GTPase activating protein 5	0.96313	0.966745	0.9729942
395	ARHGAP6	Rho GTPase activating protein 6	0.98718	0.981624	1.0058977
396	ARHGDIA	Rho GDP dissociation inhibitor (GDI) alpha	0.98689	0.925653	0.9636617
397	ARHGDIB	Rho GDP dissociation inhibitor (GDI) beta	0.97686	0.972906	1.0210378
398	ARHGDIG	Rho GDP dissociation inhibitor (GDI) gamma	0.98203	0.967486	1.0320549
399	RHOH	ras homolog gene family, member H	0.95253	0.965625	0.9440946
400	ARL1	ADP-ribosylation factor-like 1	0.92155	1.036292	1.0201792
401	PHOX2A	paired-like homeobox 2a	0.98762	1.046235	1.0665011

402	ARL2	ADP-ribosylation factor-like 2	0.96376	0.923276	1.0542583
403	ARL3	ADP-ribosylation factor-like 3	0.99388	1.018875	1.0404318
405	ARNT	aryl hydrocarbon receptor nuclear translocator	1.00992	0.994466	1.0870056
406	ARNTL	aryl hydrocarbon receptor nuclear translocator-like	0.92776	1.063575	0.9874693
407	ARR3	arrestin 3, retinal (X-arrestin)	0.99963	0.976851	0.9904075
408	ARRB1	arrestin, beta 1	0.98388	0.941383	1.0266368
409	ARRB2	arrestin, beta 2	0.99391	1.024512	0.9713112
410	ARSA	arylsulfatase A	0.98176	0.947361	1.021882
411	ARSB	arylsulfatase B	1.02438	0.947632	0.9740411
412	STS	steroid sulfatase (microsomal), isozyme S	0.96058	0.940898	1.0293415
414	ARSD	arylsulfatase D	0.9711	0.912595	1.0678158
415	ARSE	arylsulfatase E (chondrodysplasia punctata 1)	0.99013	1.000361	1.0201378
416	ARSF	arylsulfatase F	0.99453	0.988616	1.0457456
417	ART1	ADP-ribosyltransferase 1	0.97733	1.017403	0.9804355
418	ART2P	ADP-ribosyltransferase 2 (RT6 antigen homolog, rat), pseudogene	0.96233	0.959363	1.0041431
419	ART3	ADP-ribosyltransferase 3	0.99702	0.980223	1.0260058
420	ART4	ADP-ribosyltransferase 4 (Dombrock blood group)	0.98283	1.00772	0.9963127
421	ARVCF	armadillo repeat gene deleted in velocardiofacial syndrome	0.98332	0.98443	1.0189418
427	ASAH1	N-acylsphingosine amidohydrolase (acid ceramidase) 1	0.99651	1.094752	0.894163
429	ASCL1	achaete-scute complex homolog 1 (Drosophila)	1.00121	0.99235	1.0067887
430	ASCL2	achaete-scute complex homolog 2 (Drosophila)	0.97533	0.939595	1.0411422
432	ASGR1	asialoglycoprotein receptor 1	0.98456	1.041403	1.0084004
433	ASGR2	asialoglycoprotein receptor 2	0.93567	0.928897	1.0059175
434	ASIP	agouti signaling protein	0.98037	1.018025	1.0042897
435	ASL	argininosuccinate lyase	0.99603	0.970288	1.0430993
439	ASNA1	arsA arsenite transporter, ATP-binding, homolog 1 (bacterial)	0.95677	0.837711	1.1503232
440	ASNS	asparagine synthetase (glutamine-hydrolyzing)	0.97613	0.980656	1.0078712
443	ASPA	aspartoacylase	0.99881	0.992556	1.0053422
444	ASPH	aspartate beta-hydroxylase	0.97155	0.997764	1.0277417
445	ASS1	argininosuccinate synthase 1	0.99391	0.983438	1.0140176
449	ASS1P4	argininosuccinate synthetase 1 pseudogene 4	0.99401	1.037316	1.0061377
450	ASS1P5	argininosuccinate synthetase 1 pseudogene 5	0.98671	1.044901	1.0271967
451	ASS1P6	argininosuccinate synthetase 1 pseudogene 6	0.9984	0.987468	1.0551739

453	ASS1P8	argininosuccinate synthetase 1 pseudogene 8	1.0213	1.001491	1.0112034
454	ASS1P9	argininosuccinate synthetase 1 pseudogene 9	1.00592	0.983927	1.0584615
455	ASS1P10	argininosuccinate synthetase 1 pseudogene 10	1.00531	0.987606	1.0384352
459	ASS1P14	argininosuccinate synthetase 1 pseudogene 14	1.0073	0.998444	1.0436439
460	ASTN1	astrotactin 1	1.0054	1.00651	1.0171837
462	SERPINC1	serpin peptidase inhibitor, clade C (antithrombin), member 1	1.00359	0.989378	1.0137393
463	ZFHX3	zinc finger homeobox 3	0.9866	0.949978	1.009811
466	ATF1	activating transcription factor 1	0.93607	0.964249	1.0005407
467	ATF3	activating transcription factor 3	0.99847	0.966231	1.0039687
468	ATF4	activating transcription factor 4 (tax-responsive enhancer element B67)	0.97835	0.98568	1.006741
471	ATIC	5-aminoimidazole-4-carboxamide ribonucleotide formyltransferase/IMP cyclohydrolase	0.98614	0.91867	1.0005391
472	ATM	ataxia telangiectasia mutated	0.93792	0.89673	0.8891912
473	RERE	arginine-glutamic acid dipeptide (RE) repeats	0.99848	1.084936	1.0003231
474	ATOH1	atonal homolog 1 (Drosophila)	0.99736	1.0296	0.9697767
475	ATOX1	ATX1 antioxidant protein 1 homolog (yeast)	1.01622	0.926306	1.0213355
476	ATP1A1	ATPase, Na+/K+ transporting, alpha 1 polypeptide	1.00591	0.974656	0.9918225
477	ATP1A2	ATPase, Na+/K+ transporting, alpha 2 polypeptide	1.0088	1.000576	1.0078602
478	ATP1A3	ATPase, Na+/K+ transporting, alpha 3 polypeptide	0.97111	0.966593	1.0105112
479	ATP12A	ATPase, H+/K+ transporting, nongastric, alpha polypeptide	0.9955	1.005773	1.0142102
480	ATP1A4	ATPase, Na+/K+ transporting, alpha 4 polypeptide	1.02062	0.959982	1.0222741
481	ATP1B1	ATPase, Na+/K+ transporting, beta 1 polypeptide	0.99806	0.964333	1.0211478
482	ATP1B2	ATPase, Na+/K+ transporting, beta 2 polypeptide	0.9849	0.986608	0.9526777
483	ATP1B3	ATPase, Na+/K+ transporting, beta 3 polypeptide	1.03838	0.904638	0.9936492
484	ATP1B3P1	ATPase, Na+/K+ transporting, beta 3 pseudogene	1.01394	1.080692	1.0875491
486	FXYD2	FXYD domain containing ion transport regulator 2	0.97872	1.017281	0.9921912
487	ATP2A1	ATPase, Ca++ transporting, cardiac muscle, fast twitch 1	1	0.992436	1.0083112
488	ATP2A2	ATPase, Ca++ transporting, cardiac muscle, slow twitch 2	0.99219	1.060066	1.0686715
489	ATP2A3	ATPase, Ca++ transporting, ubiquitous	0.95073	1.052409	0.967537
490	ATP2B1	ATPase, Ca++ transporting, plasma membrane 1	1.00514	1.174736	0.9161788
491	ATP2B2	ATPase, Ca++ transporting, plasma membrane 2	1.0089	1.014352	1.006358
492	ATP2B3	ATPase, Ca++ transporting, plasma membrane 3	0.98675	1.000937	1.0173027
493	ATP2B4	ATPase, Ca++ transporting, plasma membrane 4	1.0164	1.013695	0.9735749
495	ATP4A	ATPase, H+/K+ exchanging, alpha polypeptide	0.98478	0.989379	1.0119669

496	ATP4B	ATPase, H+/K+ exchanging, beta polypeptide	0.9893	1.02616	1.0251
498	ATP5A1	ATP synthase, H+ transporting, mitochondrial F1 complex, alpha subunit 1, cardiac muscle	0.9883	0.93368	1.1085056
501	ALDH7A1	aldehyde dehydrogenase 7 family, member A1	1.0186	0.990252	0.9862584
506	ATP5B	ATP synthase, H+ transporting, mitochondrial F1 complex, beta polypeptide	0.99446	0.964275	1.2048232
509	ATP5C1	ATP synthase, H+ transporting, mitochondrial F1 complex, gamma polypeptide 1	0.9452	0.966726	1.020538
513	ATP5D	ATP synthase, H+ transporting, mitochondrial F1 complex, delta subunit	0.94017	0.948047	1.089634
514	ATP5E	ATP synthase, H+ transporting, mitochondrial F1 complex, epsilon subunit	0.96929	1.036455	1.0585915
515	ATP5F1	ATP synthase, H+ transporting, mitochondrial Fo complex, subunit B1	1.03693	0.898543	1.0722068
516	ATP5G1	ATP synthase, H+ transporting, mitochondrial Fo complex, subunit C1 (subunit 9)	0.94669	0.988187	0.9820359
517	ATP5G2	ATP synthase, H+ transporting, mitochondrial Fo complex, subunit C2 (subunit 9)	0.96704	0.995722	1.0634837
518	ATP5G3	ATP synthase, H+ transporting, mitochondrial Fo complex, subunit C3 (subunit 9)	0.98389	1.009729	1.0328535
520	ATP5G2P1	ATP synthase, H+ transporting, mitochondrial Fo complex, subunit C2 (subunit 9) pseudogene 1	1.03604	1.118665	1.0130321
521	ATP5I	ATP synthase, H+ transporting, mitochondrial Fo complex, subunit E	0.97425	0.962048	1.0637431
522	ATP5J	ATP synthase, H+ transporting, mitochondrial Fo complex, subunit F6	0.92203	0.954853	1.0724249
523	ATP6V1A	ATPase, H+ transporting, lysosomal 70kDa, V1 subunit A	1.01761	0.9738	1.0007436
525	ATP6V1B1	ATPase, H+ transporting, lysosomal 56/58kDa, V1 subunit B1	1.02244	1.006587	1.0006361
526	ATP6V1B2	ATPase, H+ transporting, lysosomal 56/58kDa, V1 subunit B2	1.01492	1.100327	0.9498209
527	ATP6V0C	ATPase, H+ transporting, lysosomal 16kDa, V0 subunit c	1.00617	1.001982	0.8837288
528	ATP6V1C1	ATPase, H+ transporting, lysosomal 42kDa, V1 subunit C1	0.99367	0.967483	1.0557529
529	ATP6V1E1	ATPase, H+ transporting, lysosomal 31kDa, V1 subunit E1	0.93604	0.940137	1.0001741
533	ATP6V0B	ATPase, H+ transporting, lysosomal 21kDa, V0 subunit b	1.01403	1.044585	0.9732389
534	ATP6V1G2	ATPase, H+ transporting, lysosomal 13kDa, V1 subunit G2	0.99982	1.018737	0.9729062
535	ATP6V0A1	ATPase, H+ transporting, lysosomal V0 subunit a1	0.98432	0.967939	1.0560303
537	ATP6AP1	ATPase, H+ transporting, lysosomal accessory protein 1	0.99216	1.050263	1.0729891
538	ATP7A	ATPase, Cu++ transporting, alpha polypeptide	0.94699	0.986385	1.0425271
539	ATP5O	ATP synthase, H+ transporting, mitochondrial F1 complex, O subunit	0.97291	0.934329	0.9728154
540	ATP7B	ATPase, Cu++ transporting, beta polypeptide	0.99029	0.978808	0.9756571
545	ATR	ataxia telangiectasia and Rad3 related	0.98629	0.949619	1.0172567
546	ATRX	alpha thalassemia/mental retardation syndrome X-linked	0.93159	0.93042	1.0451566
547	KIF1A	kinesin family member 1A	0.99944	1.014911	1.0427558
549	AUH	AU RNA binding protein/enoyl-CoA hydratase	0.96512	0.994001	0.9519416
550	AUP1	ancient ubiquitous protein 1	0.97793	1.000472	1.0376984
551	AVP	arginine vasopressin	0.98891	1.016197	1.0242967

552	AVPR1A	arginine vasopressin receptor 1A	0.95799	0.837609	0.9224703
553	AVPR1B	arginine vasopressin receptor 1B	1.01562	1.01511	1.0097247
554	AVPR2	arginine vasopressin receptor 2	1.00821	1.001788	0.9785464
558	AXL	AXL receptor tyrosine kinase	1.0027	1.011191	0.9899299
563	AZGP1	alpha-2-glycoprotein 1, zinc-binding	1.03013	1.014685	0.9825965
566	AZU1	azurocidin 1	1.00946	1.016396	1.0441957
567	B2M	beta-2-microglobulin	1.01442	1.072296	0.9078486
570	BAAT	bile acid CoA: amino acid N-acyltransferase (glycine N-choloyltransferase)	0.99373	1.001225	0.998873
571	BACH1	BTB and CNC homology 1, basic leucine zipper transcription factor 1	0.99625	1.113401	1.0008781
572	BAD	BCL2-associated agonist of cell death	0.96443	0.999201	1.0206251
573	BAG1	BCL2-associated athanogene	0.91655	0.906811	1.0325061
575	BAI1	brain-specific angiogenesis inhibitor 1	0.99124	1.024782	1.0296094
576	BAI2	brain-specific angiogenesis inhibitor 2	1.00082	1.002337	0.9952494
577	BAI3	brain-specific angiogenesis inhibitor 3	1.00582	1.012904	1.0036936
578	BAK1	BCL2-antagonist/killer 1	1.00142	1.007237	1.0253108
579	NKX3-2	NK3 homeobox 2	0.98758	0.989526	1.0742514
580	BARD1	BRCA1 associated RING domain 1	0.98379	0.98927	1.064835
581	BAX	BCL2-associated X protein	0.94113	0.996119	1.0590053
582	BBS1	Bardet-Biedl syndrome 1	0.97236	0.992355	1.0568883
583	BBS2	Bardet-Biedl syndrome 2	0.95688	0.93557	0.8829023
585	BBS4	Bardet-Biedl syndrome 4	0.95581	0.952182	0.9945622
586	BCAT1	branched chain amino-acid transaminase 1, cytosolic	0.97424	1.000452	0.9985896
587	BCAT2	branched chain amino-acid transaminase 2, mitochondrial	0.96287	0.957398	1.007619
590	BCHE	butyrylcholinesterase	1.00518	1.002003	0.9973446
593	BCKDHA	branched chain keto acid dehydrogenase E1, alpha polypeptide	0.9684	0.886655	1.1002353
594	BCKDHB	branched chain keto acid dehydrogenase E1, beta polypeptide	0.97237	0.975603	0.9786969
595	CCND1	cyclin D1	0.98306	0.997769	1.0415663
596	BCL2	B-cell CLL/lymphoma 2	0.95992	1.032359	1.1163776
597	BCL2A1	BCL2-related protein A1	0.9433	1.046042	0.9355731
598	BCL2L1	BCL2-like 1	0.96147	0.951231	1.0282162
599	BCL2L2	BCL2-like 2	0.99084	1.000162	1.0080013
601	BAK1P2	BCL2-antagonist/killer 1 pseudogene 2	0.99096	1.008069	0.9797101
602	BCL3	B-cell CLL/lymphoma 3	1.00636	1.002717	0.99544

604	BCL6	B-cell CLL/lymphoma 6	1.04698	1.041346	0.9802497
605	BCL7A	B-cell CLL/lymphoma 7A	0.98844	0.984894	1.0384079
606	NBEAP1	neurobeachin pseudogene 1	0.97111	1.051807	1.0477091
607	BCL9	B-cell CLL/lymphoma 9	1.00307	0.965131	1.022054
608	TNFRSF17	tumor necrosis factor receptor superfamily, member 17	0.97529	1.017934	1.0464777
610	HCN2	hyperpolarization activated cyclic nucleotide-gated potassium channel 2	0.98765	1.069256	1.0439844
611	OPN1SW	opsin 1 (cone pigments), short-wave-sensitive	0.99512	0.98801	1.043329
613	BCR	breakpoint cluster region	0.97425	1.005302	0.9413476
617	BCS1L	BCS1-like (S. cerevisiae)	0.9798	0.950779	1.0215623
622	BDH1	3-hydroxybutyrate dehydrogenase, type 1	1.0008	0.991362	1.001726
623	BDKRB1	bradykinin receptor B1	1.01397	0.98516	1.0236719
624	BDKRB2	bradykinin receptor B2	0.99754	1.001837	1.0165574
627	BDNF	brain-derived neurotrophic factor	0.98636	1.006886	0.9929669
629	CFB	complement factor B	1.01364	1.030015	0.9625868
631	BFSP1	beaded filament structural protein 1, filensin	1.0075	0.988707	0.9974681
632	BGLAP	bone gamma-carboxyglutamate (gla) protein	1.00167	0.990973	1.0627271
633	BGN	biglycan	0.98956	0.993243	1.0017351
634	CEACAM1	carcinoembryonic antigen-related cell adhesion molecule 1 (biliary glycoprotein)	1.00837	0.967626	1.0015394
635	BHMT	betaine--homocysteine S-methyltransferase	0.98802	1.022255	1.016547
636	BICD1	bicaudal D homolog 1 (Drosophila)	0.98444	1.002142	1.0118496
637	BID	BH3 interacting domain death agonist	0.96075	0.978098	0.9495141
638	BIK	BCL2-interacting killer (apoptosis-inducing)	0.99866	1.013777	1.018858
639	PRDM1	PR domain containing 1, with ZNF domain	1.03471	1.004201	0.9134144
641	BLM	Bloom syndrome, RecQ helicase-like	0.98115	0.946943	1.0093708
642	BLMH	bleomycin hydrolase	0.97073	0.921012	1.0525041
643	CXCR5	chemokine (C-X-C motif) receptor 5	0.884	0.993628	0.9152131
644	BLVRA	biliverdin reductase A	1.03358	0.903971	0.9439801
645	BLVRB	biliverdin reductase B (flavin reductase (NADPH))	0.98412	1.03222	0.9550579
646	BNC1	basonuclin 1	0.99515	1.027453	1.0402133
648	BMI1	BMI1 polycomb ring finger oncogene	0.90776	0.92736	1.0032225
649	BMP1	bone morphogenetic protein 1	1.00117	1.000824	0.9927027
650	BMP2	bone morphogenetic protein 2	0.99533	1.018346	1.0159621
651	BMP3	bone morphogenetic protein 3	0.99075	0.989969	1.0567636

652	BMP4	bone morphogenetic protein 4	0.98142	1.017871	1.0458592
653	BMP5	bone morphogenetic protein 5	1.00199	0.994589	1.0209049
654	BMP6	bone morphogenetic protein 6	1.01875	0.99643	0.9961149
655	BMP7	bone morphogenetic protein 7	1.01091	1.013598	0.9941152
656	BMP8B	bone morphogenetic protein 8b	1.00183	1.01385	1.0549515
657	BMPR1A	bone morphogenetic protein receptor, type IA	0.98797	1.02345	1.052333
658	BMPR1B	bone morphogenetic protein receptor, type IB	0.99527	1.014156	0.9926471
659	BMPR2	bone morphogenetic protein receptor, type II (serine/threonine kinase)	1.00299	1.00017	1.0462049
660	BMX	BMX non-receptor tyrosine kinase	1.00877	0.998183	0.9918562
661	POLR3D	polymerase (RNA) III (DNA directed) polypeptide D, 44kDa	1.00778	0.94205	1.0552123
662	BNIP1	BCL2/adenovirus E1B 19kDa interacting protein 1	1.0022	0.957143	1.0084413
663	BNIP2	BCL2/adenovirus E1B 19kDa interacting protein 2	0.99055	0.98293	1.0421313
664	BNIP3	BCL2/adenovirus E1B 19kDa interacting protein 3	0.94695	0.99199	1.0002046
665	BNIP3L	BCL2/adenovirus E1B 19kDa interacting protein 3-like	1.01437	0.936951	0.9519299
666	BOK	BCL2-related ovarian killer	0.98905	1.016255	1.0597662
667	DST	dystonin	1.02215	0.992656	1.0086437
668	FOXL2	forkhead box L2	1.01439	0.97525	1.0339708
669	BPGM	2,3-bisphosphoglycerate mutase	1.01597	0.960485	1.1095392
670	BPHL	biphenyl hydrolase-like (serine hydrolase)	0.99103	0.988108	1.0125463
671	BPI	bactericidal/permeability-increasing protein	0.9971	0.981108	0.9880164
672	BRCA1	breast cancer 1, early onset	0.98049	0.966456	0.9969794
673	BRAF	v-raf murine sarcoma viral oncogene homolog B1	0.98105	0.979019	0.9638158
675	BRCA2	breast cancer 2, early onset	0.96039	0.967664	1.00321
676	BRDT	bromodomain, testis-specific	1.00336	1.015981	1.0039544
677	ZFP36L1	zinc finger protein 36, C3H type-like 1	0.97431	0.997392	0.9813747
678	ZFP36L2	zinc finger protein 36, C3H type-like 2	1.03258	0.977532	0.9688709
680	BRS3	bombesin-like receptor 3	1.00142	1.00472	1.0005742
682	BSG	basigin (Ok blood group)	0.98079	0.932701	1.1236899
683	BST1	bone marrow stromal cell antigen 1	1.03401	0.896095	1.0293158
684	BST2	bone marrow stromal cell antigen 2	0.92901	0.862384	1.0590156
685	BTC	betacellulin	1.01713	0.981498	1.0269555
686	BTD	biotinidase	0.98067	0.978377	1.0115194
687	KLF9	Kruppel-like factor 9	0.94948	0.854257	0.9831714

688	KLF5	Kruppel-like factor 5 (intestinal)	0.96994	1.01487	0.9604193
689	BTF3	basic transcription factor 3	1.00332	0.889721	1.0193968
690	BTF3P11	basic transcription factor 3 pseudogene 11	1.0154	0.958914	1.0970232
694	BTG1	B-cell translocation gene 1, anti-proliferative	0.97574	1.008559	1.010807
695	BTK	Bruton agammaglobulinemia tyrosine kinase	0.95945	0.893458	1.0820522
696	BTN1A1	butyrophilin, subfamily 1, member A1	1.01017	1.038313	1.0388301
699	BUB1	budding uninhibited by benzimidazoles 1 homolog (yeast)	0.99485	0.953847	1.0215841
701	BUB1B	budding uninhibited by benzimidazoles 1 homolog beta (yeast)	0.98612	0.975656	1.0189006
705	BYSL	bystin-like	0.9835	0.97079	1.0418402
706	TSPO	translocator protein (18kDa)	0.9872	0.962383	1.0513999
708	C1QBP	complement component 1, q subcomponent binding protein	0.95774	1.002067	1.0506507
710	SERPING1	serpin peptidase inhibitor, clade G (C1 inhibitor), member 1	1.00161	1.019948	0.9944307
712	C1QA	complement component 1, q subcomponent, A chain	0.98066	0.947141	1.0059972
713	C1QB	complement component 1, q subcomponent, B chain	1.03141	0.943136	0.9809294
714	C1QC	complement component 1, q subcomponent, C chain	1.02048	0.986177	1.0004401
715	C1R	complement component 1, r subcomponent	0.9785	0.989825	0.9902169
716	C1S	complement component 1, s subcomponent	1.00724	1.035427	0.9481613
717	C2	complement component 2	0.99218	0.993352	0.9343922
718	C3	complement component 3	1.00311	1.006901	1.0524889
719	C3AR1	complement component 3a receptor 1	1.02145	1.012439	1.0261391
722	C4BPA	complement component 4 binding protein, alpha	1.01577	1.000187	0.9871098
725	C4BPB	complement component 4 binding protein, beta	1.00533	0.981684	0.9881467
726	CAPN5	calpain 5	0.9901	1.006192	1.0103422
727	C5	complement component 5	0.97819	0.976466	0.9906072
728	C5AR1	complement component 5a receptor 1	1.00846	0.923467	0.9117722
729	C6	complement component 6	1.00913	1.008175	1.0067686
730	C7	complement component 7	1.01337	1.013852	0.9953164
731	C8A	complement component 8, alpha polypeptide	1.0077	0.988786	1.0208741
732	C8B	complement component 8, beta polypeptide	1.01876	1.001749	1.0140322
733	C8G	complement component 8, gamma polypeptide	1.00981	0.988039	1.0055963
734	OSGIN2	oxidative stress induced growth inhibitor family member 2	0.97616	0.940475	1.0236536
735	C9	complement component 9	0.99122	1.03428	1.0009971
738	C11orf2	chromosome 11 open reading frame 2	0.92013	0.96322	1.0805947

740	MRPL49	mitochondrial ribosomal protein L49	0.96353	0.976697	1.104592
741	ZNHIT2	zinc finger, HIT-type containing 2	0.99263	1.012466	1.0384372
744	MPPED2	metallophosphoesterase domain containing 2	0.99179	1.007653	1.0103836
745	C11orf9	chromosome 11 open reading frame 9	0.98799	1.000445	1.0132384
746	C11orf10	chromosome 11 open reading frame 10	0.95435	0.977913	1.0445516
747	DAGLA	diacylglycerol lipase, alpha	0.99005	0.966871	0.9807893
750	C16orf3	chromosome 16 open reading frame 3	1.01408	1.1104	0.9810153
752	FMNL1	formin-like 1	0.98036	1.013061	0.957134
753	C18orf1	chromosome 18 open reading frame 1	0.98395	1.03736	0.9350223
754	PTTG1IP	pituitary tumor-transforming 1 interacting protein	0.96765	1.043941	1.0274012
755	C21orf2	chromosome 21 open reading frame 2	0.98959	1.045133	0.9297263
757	TMEM50B	transmembrane protein 50B	0.93858	1.041881	0.8905338
758	MPPED1	metallophosphoesterase domain containing 1	0.99533	1.002021	1.0212532
759	CA1	carbonic anhydrase I	1.00701	0.960185	0.9959547
760	CA2	carbonic anhydrase II	0.94211	1.016129	0.9154713
761	CA3	carbonic anhydrase III, muscle specific	1.02138	1.005332	1.0537753
762	CA4	carbonic anhydrase IV	0.98965	0.982231	0.9861267
763	CA5A	carbonic anhydrase VA, mitochondrial	1.0015	0.996045	1.0190518
765	CA6	carbonic anhydrase VI	1.01021	0.952435	0.9712538
766	CA7	carbonic anhydrase VII	0.99561	1.034916	1.0415107
767	CA8	carbonic anhydrase VIII	1.00589	0.984658	1.003976
768	CA9	carbonic anhydrase IX	0.99243	1.015054	1.0233398
770	CA11	carbonic anhydrase XI	0.9651	1.007332	1.0385636
771	CA12	carbonic anhydrase XII	0.99509	0.975078	1.024761
773	CACNA1A	calcium channel, voltage-dependent, P/Q type, alpha 1A subunit	0.99935	0.987113	1.0000926
774	CACNA1B	calcium channel, voltage-dependent, N type, alpha 1B subunit	1.006	1.007772	1.0073253
775	CACNA1C	calcium channel, voltage-dependent, L type, alpha 1C subunit	1.00075	0.99805	1.0080399
777	CACNA1E	calcium channel, voltage-dependent, R type, alpha 1E subunit	1.00729	1.001509	1.0159451
778	CACNA1F	calcium channel, voltage-dependent, L type, alpha 1F subunit	0.99844	1.002602	1.0099634
779	CACNA1S	calcium channel, voltage-dependent, L type, alpha 1S subunit	1.00109	0.998523	1.0226197
780	DDR1	discoidin domain receptor tyrosine kinase 1	0.99991	0.984039	1.0190281
781	CACNA2D1	calcium channel, voltage-dependent, alpha 2/delta subunit 1	1.0059	0.998502	1.0182447
782	CACNB1	calcium channel, voltage-dependent, beta 1 subunit	0.99161	1.042111	1.0611747

783	CACNB2	calcium channel, voltage-dependent, beta 2 subunit	0.99187	1.002364	1.0071241
784	CACNB3	calcium channel, voltage-dependent, beta 3 subunit	0.98907	1.018271	1.0184788
785	CACNB4	calcium channel, voltage-dependent, beta 4 subunit	1.02091	0.989742	1.0180382
786	CACNG1	calcium channel, voltage-dependent, gamma subunit 1	0.99244	0.99564	0.9785655
788	SLC25A20	solute carrier family 25 (carnitine/acylcarnitine translocase), member 20	0.99793	1.000946	1.0680796
789	SLC25A20P	solute carrier family 25 (carnitine/acylcarnitine translocase), member 20 pseudogene 1	1.00452	1.013728	1.0168511
790	CAD	carbamoyl-phosphate synthetase 2, aspartate transcarbamylase, and dihydroorotase	0.9842	0.977687	0.9904335
793	CALB1	calbindin 1, 28kDa	0.99487	0.976103	1.0180726
794	CALB2	calbindin 2	1.00302	1.00009	0.9899488
795	S100G	S100 calcium binding protein G	0.96968	1.003559	1.0415288
796	CALCA	calcitonin-related polypeptide alpha	1.03198	0.994367	0.9997013
797	CALCB	calcitonin-related polypeptide beta	1.00896	0.99273	1.0285942
798	CALCP	calcitonin pseudogene	0.99715	1.059744	1.0536344
799	CALCR	calcitonin receptor	1.01401	0.998703	1.018339
800	CALD1	caldesmon 1	0.99518	1.005747	0.9891459
801	CALM1	calmodulin 1 (phosphorylase kinase, delta)	0.98737	0.950593	0.9761455
804	CALM1P2	calmodulin 1 (phosphorylase kinase, delta) pseudogene 2	1.01406	1.056792	1.0370551
805	CALM2	calmodulin 2 (phosphorylase kinase, delta)	1.02213	0.907102	0.9514706
806	CALM2P2	calmodulin 2 pseudogene 2	1.05595	1.014659	1.0044906
807	CALM2P3	calmodulin 2 pseudogene 3	1.00559	0.972407	1.01027
808	CALM3	calmodulin 3 (phosphorylase kinase, delta)	0.96112	0.998348	1.0243608
810	CALML3	calmodulin-like 3	0.98556	0.983235	0.9593053
811	CALR	calreticulin	0.97275	1.003337	1.081597
813	CALU	calumenin	1.03991	0.947328	0.9756256
814	CAMK4	calcium/calmodulin-dependent protein kinase IV	0.95721	0.966096	0.9770673
815	CAMK2A	calcium/calmodulin-dependent protein kinase II alpha	1.03439	1.136868	0.9163698
816	CAMK2B	calcium/calmodulin-dependent protein kinase II beta	1.00548	0.995303	1.0274495
817	CAMK2D	calcium/calmodulin-dependent protein kinase II delta	0.95707	0.963529	1.0554699
818	CAMK2G	calcium/calmodulin-dependent protein kinase II gamma	0.96622	0.969673	0.9858345
819	CAMLG	calcium modulating ligand	0.97116	0.944566	0.9473087
820	CAMP	cathelicidin antimicrobial peptide	1.0208	0.997812	1.008499
821	CANX	calnexin	0.99517	1.000361	0.9893803
822	CAPG	capping protein (actin filament), gelsolin-like	0.98826	0.868049	1.1201238

823	CAPN1	calpain 1, (mu/I) large subunit	0.93463	0.903366	1.1510858
824	CAPN2	calpain 2, (m/II) large subunit	1.01395	1.024784	0.9668887
825	CAPN3	calpain 3, (p94)	0.98359	1.03211	0.9196829
826	CAPNS1	calpain, small subunit 1	1.01767	0.958529	0.9839228
827	CAPN6	calpain 6	1.01257	1.007056	1.0274225
828	CAPS	calcyphosine	0.98947	0.946906	0.9860358
829	CAPZA1	capping protein (actin filament) muscle Z-line, alpha 1	1.06384	0.978695	0.9670866
830	CAPZA2	capping protein (actin filament) muscle Z-line, alpha 2	1.00783	0.95155	0.9952143
831	CAST	calpastatin	1.01954	0.96231	0.9635725
832	CAPZB	capping protein (actin filament) muscle Z-line, beta	0.99553	0.993548	0.985454
833	CARS	cysteinyl-tRNA synthetase	0.9472	1.006887	1.0616444
834	CASP1	caspase 1, apoptosis-related cysteine peptidase (interleukin 1, beta, convertase)	0.97778	0.949599	1.0708593
835	CASP2	caspase 2, apoptosis-related cysteine peptidase	0.99887	0.923994	1.1413499
836	CASP3	caspase 3, apoptosis-related cysteine peptidase	0.98316	0.96879	1.0585876
837	CASP4	caspase 4, apoptosis-related cysteine peptidase	0.97501	0.925607	1.0295297
838	CASP5	caspase 5, apoptosis-related cysteine peptidase	0.96267	0.996951	0.9134306
839	CASP6	caspase 6, apoptosis-related cysteine peptidase	0.96402	0.935616	1.0586667
840	CASP7	caspase 7, apoptosis-related cysteine peptidase	0.9534	1.008486	1.0047137
841	CASP8	caspase 8, apoptosis-related cysteine peptidase	1.01637	1.000529	0.985534
842	CASP9	caspase 9, apoptosis-related cysteine peptidase	0.96181	0.967932	0.9276335
843	CASP10	caspase 10, apoptosis-related cysteine peptidase	0.9903	0.953896	0.9694991
844	CASQ1	calsequestrin 1 (fast-twitch, skeletal muscle)	1.00664	1.012779	0.9789885
845	CASQ2	calsequestrin 2 (cardiac muscle)	1.01711	1.013669	1.0237367
846	CASR	calcium-sensing receptor	1.00739	1.00101	1.0071564
847	CAT	catalase	0.98052	0.927354	0.9563888
857	CAV1	caveolin 1, caveolae protein, 22kDa	0.9911	1.001567	1.0198755
858	CAV2	caveolin 2	1.02526	0.942841	0.9994352
859	CAV3	caveolin 3	0.97902	1.049098	1.001296
860	RUNX2	runt-related transcription factor 2	0.9943	0.99459	1.0115147
861	RUNX1	runt-related transcription factor 1	0.9683	1.084384	1.0162631
862	RUNX1T1	runt-related transcription factor 1; translocated to, 1 (cyclin D-related)	0.99549	0.992547	1.0050339
863	CBFA2T3	core-binding factor, runt domain, alpha subunit 2; translocated to, 3	0.99178	0.98316	1.0192291
864	RUNX3	runt-related transcription factor 3	1.02233	1.066597	0.9416931

865	CBFB	core-binding factor, beta subunit	0.97482	0.979922	1.0694257
866	SERPINA6	serpin peptidase inhibitor, clade A (alpha-1 antiproteinase, antitrypsin), member 6	1.00051	0.989629	0.9809284
867	CBL	Cas-Br-M (murine) ecotropic retroviral transforming sequence	0.98916	0.968127	1.0632434
868	CBLB	Cas-Br-M (murine) ecotropic retroviral transforming sequence b	0.98861	1.158107	0.9771289
869	CBLN1	cerebellin 1 precursor	0.99506	1.008085	1.0284507
871	SERPINH1	serpin peptidase inhibitor, clade H (heat shock protein 47), member 1, (collagen binding protein 1)	0.9696	0.970063	1.0120616
873	CBR1	carbonyl reductase 1	0.94128	0.951595	1.0359408
874	CBR3	carbonyl reductase 3	1.00167	0.978494	0.960458
875	CBS	cystathionine-beta-synthase	0.98622	0.993129	1.0354369
881	CCIN	calicin	0.98101	1.110715	0.9197425
883	CCBL1	cysteine conjugate-beta lyase, cytoplasmic	0.94788	0.98777	1.012763
885	CCK	cholecystokinin	1.00416	1.006852	1.0504435
886	CCKAR	cholecystokinin A receptor	1.01095	0.993878	0.9966077
887	CCKBR	cholecystokinin B receptor	0.98676	0.966502	1.0186607
889	KRIT1	KRIT1, ankyrin repeat containing	0.96964	0.934971	1.0069224
890	CCNA2	cyclin A2	0.98112	0.962363	1.0505896
891	CCNB1	cyclin B1	1.01138	0.948015	1.0196822
892	CCNC	cyclin C	0.99698	0.947734	1.0444862
894	CCND2	cyclin D2	0.95265	0.941785	0.9570273
895	CCND2P	cyclin D2 pseudogene	1.0344	0.974598	1.0299714
896	CCND3	cyclin D3	1.00025	0.955126	1.0389708
898	CCNE1	cyclin E1	0.98896	0.903908	1.0253372
899	CCNF	cyclin F	0.98249	0.99116	1.0105142
900	CCNG1	cyclin G1	0.94158	0.927782	0.9488327
901	CCNG2	cyclin G2	1.00525	0.905493	1.0132704
902	CCNH	cyclin H	0.98832	0.954937	0.9683773
904	CCNT1	cyclin T1	0.93396	0.92107	1.0232421
905	CCNT2	cyclin T2	1.00473	0.93131	0.9814599
908	CCT6A	chaperonin containing TCP1, subunit 6A (zeta 1)	0.99202	0.97652	1.0896249
909	CD1A	CD1a molecule	1.016	0.994549	0.9706687
910	CD1B	CD1b molecule	0.99486	1.003689	1.0393869
911	CD1C	CD1c molecule	1.01032	0.935322	0.936897
912	CD1D	CD1d molecule	1.03815	0.922525	0.9701062

913	CD1E	CD1e molecule	1.00483	0.935602	0.997526
914	CD2	CD2 molecule	0.97877	1.021476	0.964747
915	CD3D	CD3d molecule, delta (CD3-TCR complex)	0.90057	1.140991	0.9608539
916	CD3E	CD3e molecule, epsilon (CD3-TCR complex)	0.92946	0.998532	0.9709782
917	CD3G	CD3g molecule, gamma (CD3-TCR complex)	0.92437	0.852178	1.0097404
919	CD247	CD247 molecule	0.97969	0.905284	0.9586735
920	CD4	CD4 molecule	0.96525	0.902647	1.0096528
921	CD5	CD5 molecule	0.93845	1.042022	0.9888729
922	CD5L	CD5 molecule-like	1.01322	0.982784	1.005507
923	CD6	CD6 molecule	0.94861	1.049947	0.9961517
924	CD7	CD7 molecule	0.93751	1.023093	0.9937539
925	CD8A	CD8a molecule	0.96346	1.073542	0.9306329
928	CD9	CD9 molecule	0.94581	0.980148	0.9990592
929	CD14	CD14 molecule	1.01338	0.912268	1.0481161
932	MS4A3	membrane-spanning 4-domains, subfamily A, member 3 (hematopoietic cell-specific)	0.97812	0.947047	1.0217408
939	CD27	CD27 molecule	0.91651	0.815512	1.002675
940	CD28	CD28 molecule	0.94474	0.874395	0.9537329
941	CD80	CD80 molecule	1.03702	0.941334	0.9669815
942	CD86	CD86 molecule	1.00646	1.048183	0.971018
943	TNFRSF8	tumor necrosis factor receptor superfamily, member 8	1.00789	0.982156	1.0280526
944	TNFSF8	tumor necrosis factor (ligand) superfamily, member 8	0.97081	0.985959	0.9658888
945	CD33	CD33 molecule	1.01606	0.865518	1.0362712
946	SIGLEC6	sialic acid binding Ig-like lectin 6	0.99764	0.982131	0.9538641
947	CD34	CD34 molecule	1.00782	1.018024	0.9837814
948	CD36	CD36 molecule (thrombospondin receptor)	1.03518	0.856718	1.0107859
949	SCARB1	scavenger receptor class B, member 1	0.99202	0.990707	1.0200506
950	SCARB2	scavenger receptor class B, member 2	1.03802	0.974696	1.018729
951	CD37	CD37 molecule	0.97303	1.084348	1.0716539
952	CD38	CD38 molecule	0.98631	0.951636	1.0281915
953	ENTPD1	ectonucleoside triphosphate diphosphohydrolase 1	0.97135	0.956343	1.0719809
954	ENTPD2	ectonucleoside triphosphate diphosphohydrolase 2	0.98078	1.018587	1.0520241
955	ENTPD6	ectonucleoside triphosphate diphosphohydrolase 6 (putative)	0.94022	0.921373	1.023502
956	ENTPD3	ectonucleoside triphosphate diphosphohydrolase 3	1.01967	1.006978	1.0015218

957	ENTPD5	ectonucleoside triphosphate diphosphohydrolase 5	0.99032	0.987001	1.0400989
958	CD40	CD40 molecule, TNF receptor superfamily member 5	0.96398	1.020353	1.0591754
959	CD40LG	CD40 ligand	0.90234	0.89184	0.9463529
960	CD44	CD44 molecule (Indian blood group)	1.01478	1.034131	1.0012779
961	CD47	CD47 molecule	1.02291	0.951061	0.9526303
962	CD48	CD48 molecule	1.01665	0.922006	1.0201848
963	CD53	CD53 molecule	1.04828	0.992606	0.9705709
965	CD58	CD58 molecule	1.02856	1.028455	1.0775794
966	CD59	CD59 molecule, complement regulatory protein	0.93018	0.989651	1.0179291
967	CD63	CD63 molecule	0.99611	1.038101	0.9814944
968	CD68	CD68 molecule	0.98573	0.996025	0.9579735
969	CD69	CD69 molecule	0.94863	0.972889	0.9756256
970	CD70	CD70 molecule	1.00626	0.962271	1.021038
971	CD72	CD72 molecule	0.94563	1.072528	1.025812
974	CD79B	CD79b molecule, immunoglobulin-associated beta	0.90193	1.048674	1.0545038
975	CD81	CD81 molecule	0.97044	0.932129	1.0378688
976	CD97	CD97 molecule	0.97977	1.06678	0.9559296
977	CD151	CD151 molecule (Raph blood group)	0.99842	1.075624	0.9291512
978	CDA	cytidine deaminase	1.00259	0.980104	0.9560321
983	CDK1	cyclin-dependent kinase 1	0.98175	0.975236	1.0157726
984	CDK11B	cyclin-dependent kinase 11B	1.01542	0.990325	0.8610247
987	LRBA	LPS-responsive vesicle trafficking, beach and anchor containing	0.99911	0.876431	0.9895121
988	CDC5L	CDC5 cell division cycle 5-like (S. pombe)	0.9682	0.906752	1.0178805
989	7-Sep	septin 7	0.99643	0.961224	1.032962
990	CDC6	cell division cycle 6 homolog (S. cerevisiae)	0.98641	0.999492	1.0089333
991	CDC20	cell division cycle 20 homolog (S. cerevisiae)	1.02246	0.997969	1.014052
993	CDC25A	cell division cycle 25 homolog A (S. pombe)	1.02703	1.008303	1.0136498
994	CDC25B	cell division cycle 25 homolog B (S. pombe)	0.9293	0.988397	0.9344754
995	CDC25C	cell division cycle 25 homolog C (S. pombe)	0.99706	0.996339	1.0024632
996	CDC27	cell division cycle 27 homolog (S. cerevisiae)	0.95537	1.01174	1.0302254
997	CDC34	cell division cycle 34 homolog (S. cerevisiae)	0.95165	0.998646	1.0375066
998	CDC42	cell division cycle 42 (GTP binding protein, 25kDa)	1.04342	0.942109	0.9616169
999	CDH1	cadherin 1, type 1, E-cadherin (epithelial)	0.9892	1.013644	1.0084836

1000	CDH2	cadherin 2, type 1, N-cadherin (neuronal)	0.99148	0.988808	1.0128139
1001	CDH3	cadherin 3, type 1, P-cadherin (placental)	0.98802	1.033333	1.021999
1002	CDH4	cadherin 4, type 1, R-cadherin (retinal)	1.00553	0.985754	0.9860788
1003	CDH5	cadherin 5, type 2 (vascular endothelium)	0.99873	0.976558	0.979039
1004	CDH6	cadherin 6, type 2, K-cadherin (fetal kidney)	1.01539	1.007667	0.9970266
1005	CDH7	cadherin 7, type 2	1.00106	0.986865	0.9962906
1006	CDH8	cadherin 8, type 2	1.00035	0.983884	0.9956312
1007	CDH9	cadherin 9, type 2 (T1-cadherin)	1.00146	1.016505	1.034502
1008	CDH10	cadherin 10, type 2 (T2-cadherin)	0.99554	1.009871	1.0121634
1009	CDH11	cadherin 11, type 2, OB-cadherin (osteoblast)	0.99812	0.990867	0.9981884
1010	CDH12	cadherin 12, type 2 (N-cadherin 2)	1.00659	1.007111	1.0182571
1012	CDH13	cadherin 13, H-cadherin (heart)	0.99821	1.008608	1.0047498
1013	CDH15	cadherin 15, type 1, M-cadherin (myotubule)	0.98665	0.969158	1.083587
1014	CDH16	cadherin 16, KSP-cadherin	1.00107	1.012195	1.0264973
1015	CDH17	cadherin 17, LI cadherin (liver-intestine)	1.00381	0.990827	1.0037151
1016	CDH18	cadherin 18, type 2	1.00257	1.000286	1.0086267
1017	CDK2	cyclin-dependent kinase 2	0.92942	1.025056	1.0357079
1018	CDK3	cyclin-dependent kinase 3	0.96625	0.97065	1.0123878
1019	CDK4	cyclin-dependent kinase 4	0.96856	1.030343	1.0239926
1020	CDK5	cyclin-dependent kinase 5	0.93837	0.946551	1.1254783
1021	CDK6	cyclin-dependent kinase 6	1.00545	0.981123	1.067329
1022	CDK7	cyclin-dependent kinase 7	0.994	0.966154	1.0379366
1024	CDK8	cyclin-dependent kinase 8	0.93469	1.011423	0.9841138
1025	CDK9	cyclin-dependent kinase 9	0.94381	0.955186	1.0045579
1026	CDKN1A	cyclin-dependent kinase inhibitor 1A (p21, Cip1)	0.99289	1.053958	0.9657756
1027	CDKN1B	cyclin-dependent kinase inhibitor 1B (p27, Kip1)	0.94812	0.956112	1.004233
1028	CDKN1C	cyclin-dependent kinase inhibitor 1C (p57, Kip2)	0.99268	0.949459	1.0839563
1029	CDKN2A	cyclin-dependent kinase inhibitor 2A (melanoma, p16, inhibits CDK4)	0.97839	0.975119	1.0335067
1030	CDKN2B	cyclin-dependent kinase inhibitor 2B (p15, inhibits CDK4)	0.97966	0.949097	1.0786824
1031	CDKN2C	cyclin-dependent kinase inhibitor 2C (p18, inhibits CDK4)	1.00265	1.002342	0.973347
1032	CDKN2D	cyclin-dependent kinase inhibitor 2D (p19, inhibits CDK4)	0.94414	0.986742	0.9437675
1033	CDKN3	cyclin-dependent kinase inhibitor 3	1.0065	0.951644	1.0102877
1036	CDO1	cysteine dioxygenase, type I	0.98588	0.993133	1.0331246

1038	CDR1	cerebellar degeneration-related protein 1, 34kDa	1.00829	0.994287	1.0043919
1039	CDR2	cerebellar degeneration-related protein 2, 62kDa	0.93737	0.961354	0.9998315
1040	CDS1	CDP-diacylglycerol synthase (phosphatidate cytidylyltransferase) 1	1.00079	0.984401	0.9856957
1041	CDSN	corneodesmosin	1.01374	0.975116	1.0398527
1043	CD52	CD52 molecule	0.96917	0.919746	0.99856
1044	CDX1	caudal type homeobox 1	0.987	1.021357	1.0570542
1045	CDX2	caudal type homeobox 2	0.99042	0.97659	0.999556
1046	CDX4	caudal type homeobox 4	1.00008	1.020591	1.0070062
1047	CLGN	calmegin	0.99772	1.005076	1.0268922
1048	CEACAM5	carcinoembryonic antigen-related cell adhesion molecule 5	1.01325	1.006431	1.0112473
1050	CEBPA	CCAAT/enhancer binding protein (C/EBP), alpha	0.91863	0.921507	0.9930368
1051	CEBPB	CCAAT/enhancer binding protein (C/EBP), beta	1.02359	1.011331	0.8646819
1052	CEBPD	CCAAT/enhancer binding protein (C/EBP), delta	0.94191	1.115247	0.9681724
1053	CEBPE	CCAAT/enhancer binding protein (C/EBP), epsilon	0.98362	1.00919	1.0171613
1054	CEBPG	CCAAT/enhancer binding protein (C/EBP), gamma	0.9399	0.93142	0.9941739
1056	CEL	carboxyl ester lipase (bile salt-stimulated lipase)	0.97503	0.981416	1.0339492
1057	CELP	carboxyl ester lipase pseudogene	0.98951	0.99036	1.0986266
1058	CENPA	centromere protein A	0.98984	0.973745	0.9981754
1059	CENPB	centromere protein B, 80kDa	0.93676	0.937927	1.0690306
1060	CENPC1	centromere protein C 1	1.01344	0.823112	0.9143624
1061	CENPC1P1	centromere protein C 1 pseudogene 1	0.97793	0.969883	0.9812693
1062	CENPE	centromere protein E, 312kDa	1.0097	0.976548	1.0130691
1063	CENPF	centromere protein F, 350/400kDa (mitosin)	1.01015	0.974024	1.0319349
1066	CES1	carboxylesterase 1	1.03702	0.963113	0.9619495
1068	CETN1	centrin, EF-hand protein, 1	0.97713	1.025578	1.0147732
1069	CETN2	centrin, EF-hand protein, 2	0.92921	0.92807	1.0261009
1070	CETN3	centrin, EF-hand protein, 3	0.98706	0.968895	1.0196764
1071	CETP	cholesteryl ester transfer protein, plasma	0.96673	1.015034	0.9964323
1072	CFL1	cofilin 1 (non-muscle)	0.9878	0.994605	1.0083448
1073	CFL2	cofilin 2 (muscle)	0.96405	0.900924	0.9661768
1075	CTSC	cathepsin C	0.9759	1.138616	1.0133053
1080	CFTR	cystic fibrosis transmembrane conductance regulator (ATP-binding cassette sub-family C, member 7)	1.00259	1.03236	1.0114673
1081	CGA	glycoprotein hormones, alpha polypeptide	1.01515	0.996778	1.0110657

1084	CEACAM3	carcinoembryonic antigen-related cell adhesion molecule 3	1.00345	0.962759	0.9583559
1085	CEACAMP5	carcinoembryonic antigen-related cell adhesion molecule pseudogene 5	1.03271	0.981612	0.9766498
1087	CEACAM7	carcinoembryonic antigen-related cell adhesion molecule 7	0.97177	0.980771	0.9696182
1088	CEACAM8	carcinoembryonic antigen-related cell adhesion molecule 8	1.04738	0.97392	1.0040391
1089	CEACAM4	carcinoembryonic antigen-related cell adhesion molecule 4	1.03196	1.011499	1.0168356
1091	CEACAMP2	carcinoembryonic antigen-related cell adhesion molecule pseudogene 2	1.00136	1.004505	1.0010349
1092	CEACAMP3	carcinoembryonic antigen-related cell adhesion molecule pseudogene 3	1.01762	1.060453	0.9943522
1093	CEACAMP4	carcinoembryonic antigen-related cell adhesion molecule pseudogene 4	0.99124	1.024809	0.957809
1101	CHAD	chondroadherin	1.00982	0.997099	1.0278405
1102	RCBTB2	regulator of chromosome condensation (RCC1) and BTB (POZ) domain containing protein 2	0.99443	0.855157	1.0673595
1103	CHAT	choline O-acetyltransferase	1.00251	0.983379	1.0188592
1105	CHD1	chromodomain helicase DNA binding protein 1	1.01154	0.970327	0.9585107
1106	CHD2	chromodomain helicase DNA binding protein 2	0.94737	1.073409	0.9476333
1107	CHD3	chromodomain helicase DNA binding protein 3	0.95369	0.953022	0.9608957
1108	CHD4	chromodomain helicase DNA binding protein 4	0.95801	1.017605	0.9988178
1109	AKR1C4	aldo-keto reductase family 1, member C4 (chlordecone reductase; 3-alpha hydroxysteroid dehydrogenase, ty	0.99328	0.991972	1.0134081
1111	CHEK1	CHK1 checkpoint homolog (S. pombe)	0.97711	0.995175	0.9958195
1112	FOXN3	forkhead box N3	1.01606	0.986235	0.9112326
1113	CHGA	chromogranin A (parathyroid secretory protein 1)	0.99963	1.046634	1.0060451
1114	CHGB	chromogranin B (secretogranin 1)	1.01619	0.99635	0.9716882
1116	CHI3L1	chitinase 3-like 1 (cartilage glycoprotein-39)	1.00805	0.935721	1.0282664
1117	CHI3L2	chitinase 3-like 2	0.97536	1.018788	1.0497672
1118	CHIT1	chitinase 1 (chitotriosidase)	1.00071	1.000093	1.0298184
1119	CHKA	choline kinase alpha	0.99486	0.97693	0.9815924
1121	CHM	choroideremia (Rab escort protein 1)	0.95859	0.938558	1.0083397
1123	CHN1	chimerin (chimaerin) 1	0.98276	0.944651	0.9719983
1124	CHN2	chimerin (chimaerin) 2	1.02807	1.029112	0.9810302
1128	CHRM1	cholinergic receptor, muscarinic 1	0.99472	1.003088	0.9815315
1129	CHRM2	cholinergic receptor, muscarinic 2	1.00221	1.013227	1.0229833
1130	LYST	lysosomal trafficking regulator	1.05474	0.930657	1.0189181
1131	CHRM3	cholinergic receptor, muscarinic 3	0.99982	1.003455	0.9919914
1132	CHRM4	cholinergic receptor, muscarinic 4	1.04611	1.003779	0.9694719
1133	CHRM5	cholinergic receptor, muscarinic 5	1.00272	1.018312	0.9870417

1134	CHRNA1	cholinergic receptor, nicotinic, alpha 1 (muscle)	1.00284	1.015805	0.976864
1135	CHRNA2	cholinergic receptor, nicotinic, alpha 2 (neuronal)	0.98544	0.99861	0.9773948
1136	CHRNA3	cholinergic receptor, nicotinic, alpha 3	1.01227	1.041422	0.9846181
1137	CHRNA4	cholinergic receptor, nicotinic, alpha 4	0.98777	1.026936	1.0323956
1138	CHRNA5	cholinergic receptor, nicotinic, alpha 5	1.00008	1.032224	1.0076618
1139	CHRNA7	cholinergic receptor, nicotinic, alpha 7	0.99594	1.023464	1.0072946
1140	CHRNB1	cholinergic receptor, nicotinic, beta 1 (muscle)	0.97844	1.000817	1.0311019
1141	CHRNB2	cholinergic receptor, nicotinic, beta 2 (neuronal)	1.00478	1.023049	1.0166318
1142	CHRNB3	cholinergic receptor, nicotinic, beta 3	1.00252	1.002578	0.9772373
1143	CHRNB4	cholinergic receptor, nicotinic, beta 4	1.0148	1.018428	1.0228719
1144	CHRND	cholinergic receptor, nicotinic, delta	1.02751	1.002509	1.0038111
1145	CHRNE	cholinergic receptor, nicotinic, epsilon	0.98138	1.05519	0.9405314
1146	CHRNG	cholinergic receptor, nicotinic, gamma	0.99521	1.010799	1.0196607
1147	CHUK	conserved helix-loop-helix ubiquitous kinase	0.95712	0.982044	1.1002652
1149	CIDEA	cell death-inducing DFFA-like effector a	0.98955	0.996477	0.9990195
1152	CKB	creatine kinase, brain	0.98393	0.999187	1.0331432
1153	CIRBP	cold inducible RNA binding protein	0.97991	1.002586	1.0065905
1154	CISH	cytokine inducible SH2-containing protein	0.96208	0.931376	1.0230538
1155	TBCB	tubulin folding cofactor B	0.9597	0.968551	1.172398
1157	CKBP1	creatine kinase B pseudogene 1	0.9974	0.990735	1.0989543
1158	CKM	creatine kinase, muscle	0.97129	0.97231	1.0071082
1160	CKMT2	creatine kinase, mitochondrial 2 (sarcomeric)	0.99732	1.000937	1.0123087
1161	ERCC8	excision repair cross-complementing rodent repair deficiency, complementation group 8	0.98704	0.956987	1.0250268
1163	CKS1B	CDC28 protein kinase regulatory subunit 1B	0.99407	0.987727	1.0306029
1164	CKS2	CDC28 protein kinase regulatory subunit 2	0.98742	0.989342	0.9893168
1173	AP2M1	adaptor-related protein complex 2, mu 1 subunit	1.0037	0.949733	1.0095322
1174	AP1S1	adaptor-related protein complex 1, sigma 1 subunit	0.96193	0.915301	1.0979902
1175	AP2S1	adaptor-related protein complex 2, sigma 1 subunit	0.95483	1.0088	1.1180617
1176	AP3S1	adaptor-related protein complex 3, sigma 1 subunit	1.00339	0.954988	1.0032605
1178	CLC	Charcot-Leyden crystal protein	0.94367	0.943504	1.0528017
1179	CLCA1	chloride channel accessory 1	1.00825	1.02185	1.0055909
1180	CLCN1	chloride channel 1, skeletal muscle	1.00594	1.016309	1.0112015
1181	CLCN2	chloride channel 2	0.99797	1.019252	1.0056562

1182	CLCN3	chloride channel 3	0.98481	1.000341	1.0610249
1183	CLCN4	chloride channel 4	0.93355	0.948944	0.9507691
1184	CLCN5	chloride channel 5	1.01043	0.949685	1.0777773
1185	CLCN6	chloride channel 6	0.99223	1.050106	1.0798122
1186	CLCN7	chloride channel 7	0.96972	1.017632	0.9601892
1187	CLCNKA	chloride channel Ka	1.00658	1.003583	1.0258679
1188	CLCNKB	chloride channel Kb	0.99458	1.028943	1.0164838
1191	CLU	clusterin	0.94526	0.960297	0.9823754
1192	CLIC1	chloride intracellular channel 1	0.99656	1.021638	1.0109279
1193	CLIC2	chloride intracellular channel 2	0.99236	0.982259	1.0233923
1195	CLK1	CDC-like kinase 1	1.00476	0.979422	0.9608302
1196	CLK2	CDC-like kinase 2	1.00521	0.951466	1.0529354
1197	CLK2P	CDC-like kinase 2, pseudogene	0.95794	1.024216	1.0396377
1198	CLK3	CDC-like kinase 3	0.96861	0.934762	0.9836343
1200	TPP1	tripeptidyl peptidase I	0.98218	0.902744	0.930358
1201	CLN3	ceroid-lipofuscinosis, neuronal 3	0.96735	0.981908	1.0584692
1203	CLN5	ceroid-lipofuscinosis, neuronal 5	0.93652	0.936842	0.9874089
1204	CLNS1AP1	chloride channel, nucleotide-sensitive, 1A pseudogene 1	0.99733	0.940139	1.0320264
1207	CLNS1A	chloride channel, nucleotide-sensitive, 1A	0.93516	0.973041	1.0096505
1208	CLPS	colipase, pancreatic	0.98556	0.982203	1.0143491
1209	CLPTM1	cleft lip and palate associated transmembrane protein 1	0.99228	0.929848	1.1086948
1211	CLTA	clathrin, light chain A	0.95645	1.050931	1.1260323
1212	CLTB	clathrin, light chain B	0.96435	0.96437	1.0255203
1213	CLTC	clathrin, heavy chain (Hc)	0.99601	1.040768	1.1688426
1215	CMA1	chymase 1, mast cell	1	0.986614	1.0272464
1230	CCR1	chemokine (C-C motif) receptor 1	1.01905	0.953475	0.9470038
1232	CCR3	chemokine (C-C motif) receptor 3	0.97212	0.943295	0.9861325
1233	CCR4	chemokine (C-C motif) receptor 4	0.97681	0.989051	1.0226807
1234	CCR5	chemokine (C-C motif) receptor 5	1.00653	1.013225	0.8979668
1236	CCR7	chemokine (C-C motif) receptor 7	0.92655	0.907408	0.9386082
1237	CCR8	chemokine (C-C motif) receptor 8	0.98257	0.962201	1.0055216
1238	CCBP2	chemokine binding protein 2	0.99965	1.016689	0.9641853
1240	CMKLR1	chemokine-like receptor 1	1.00208	0.945707	0.9948587

1241	LTB4R	leukotriene B4 receptor	0.96893	1.124024	1.0262027
1244	ABCC2	ATP-binding cassette, sub-family C (CFTR/MRP), member 2	0.9954	0.98217	0.9600985
1258	CNGB1	cyclic nucleotide gated channel beta 1	0.99135	1.019619	1.0007502
1259	CNGA1	cyclic nucleotide gated channel alpha 1	1.00176	0.973464	0.9914252
1260	CNGA2	cyclic nucleotide gated channel alpha 2	1.00156	1.016309	0.9948334
1261	CNGA3	cyclic nucleotide gated channel alpha 3	1.0169	0.97457	0.9995001
1262	CNGA4	cyclic nucleotide gated channel alpha 4	0.98543	0.994665	1.0171319
1263	PLK3	polo-like kinase 3	0.99732	0.978619	0.891086
1264	CNN1	calponin 1, basic, smooth muscle	0.98662	0.981349	0.9981054
1265	CNN2	calponin 2	0.9082	0.935034	0.954023
1266	CNN3	calponin 3, acidic	0.9678	0.999358	0.9958475
1267	CNP	2',3'-cyclic nucleotide 3' phosphodiesterase	0.9564	0.964497	1.0803696
1268	CNR1	cannabinoid receptor 1 (brain)	0.98846	0.985712	1.017785
1269	CNR2	cannabinoid receptor 2 (macrophage)	1.0133	1.024904	1.0114079
1271	CNTFR	ciliary neurotrophic factor receptor	0.9988	0.991467	0.9999552
1272	CNTN1	contactin 1	0.99748	1.010343	1.0104458
1277	COL1A1	collagen, type I, alpha 1	0.99113	1.030562	1.0262515
1278	COL1A2	collagen, type I, alpha 2	1.01326	1.014154	1.0253417
1280	COL2A1	collagen, type II, alpha 1	0.98666	1.016366	1.0310961
1281	COL3A1	collagen, type III, alpha 1	1.00995	1.013502	1.0163151
1282	COL4A1	collagen, type IV, alpha 1	1.00233	1.004912	1.0133734
1284	COL4A2	collagen, type IV, alpha 2	0.99974	0.991931	1.0072464
1285	COL4A3	collagen, type IV, alpha 3 (Goodpasture antigen)	1.00046	0.992188	0.9834155
1286	COL4A4	collagen, type IV, alpha 4	0.99736	0.986342	0.9898506
1287	COL4A5	collagen, type IV, alpha 5	0.99391	0.996399	1.0002326
1288	COL4A6	collagen, type IV, alpha 6	1.01307	0.99497	1.0128114
1289	COL5A1	collagen, type V, alpha 1	0.99162	1.005964	1.0308103
1290	COL5A2	collagen, type V, alpha 2	0.99586	0.994793	0.99907
1291	COL6A1	collagen, type VI, alpha 1	0.98585	0.97089	1.0558073
1292	COL6A2	collagen, type VI, alpha 2	0.97823	1.031854	1.0004773
1293	COL6A3	collagen, type VI, alpha 3	1.00963	0.992171	0.9990679
1294	COL7A1	collagen, type VII, alpha 1	1.00887	1.016303	1.0095669
1295	COL8A1	collagen, type VIII, alpha 1	1.00213	0.999537	1.021019

1296	COL8A2	collagen, type VIII, alpha 2	1.01085	1.015914	1.0653928
1297	COL9A1	collagen, type IX, alpha 1	0.995	1.007467	1.0066478
1298	COL9A2	collagen, type IX, alpha 2	1.0011	1.015718	1.0481683
1299	COL9A3	collagen, type IX, alpha 3	1.00029	1.003211	1.0436849
1300	COL10A1	collagen, type X, alpha 1	0.99293	0.976235	1.0007716
1301	COL11A1	collagen, type XI, alpha 1	0.99844	1.016735	1.0176916
1302	COL11A2	collagen, type XI, alpha 2	0.99137	1.020403	1.0353458
1303	COL12A1	collagen, type XII, alpha 1	1.00281	1.010023	1.0138694
1305	COL13A1	collagen, type XIII, alpha 1	0.99548	1.011615	1.0197852
1306	COL15A1	collagen, type XV, alpha 1	0.99528	1.015246	0.9774787
1307	COL16A1	collagen, type XVI, alpha 1	1.0046	1.010745	1.0198725
1308	COL17A1	collagen, type XVII, alpha 1	1.00287	1.00075	0.9951646
1311	COMP	cartilage oligomeric matrix protein	1.00399	0.991725	1.0368723
1312	COMT	catechol-O-methyltransferase	0.99723	0.986993	1.0349321
1314	COPA	coatomer protein complex, subunit alpha	1.0135	1.145978	0.9900917
1315	COPB1	coatomer protein complex, subunit beta 1	0.93113	1.047159	1.1752526
1316	KLF6	Kruppel-like factor 6	0.94988	0.890082	0.8801321
1318	SLC31A2	solute carrier family 31 (copper transporters), member 2	0.99624	0.981373	0.9962147
1326	MAP3K8	mitogen-activated protein kinase kinase kinase 8	0.96894	1.06122	0.9153044
1327	COX4I1	cytochrome c oxidase subunit IV isoform 1	0.93597	0.99031	0.9414582
1329	COX5B	cytochrome c oxidase subunit Vb	0.99868	0.953179	1.0128267
1330	COX5BP1	cytochrome c oxidase subunit Vb pseudogene 1	1.01103	0.997273	0.9082808
1337	COX6A1	cytochrome c oxidase subunit VIa polypeptide 1	1.0047	0.9088	0.936738
1338	COX6A1P1	cytochrome c oxidase subunit VIa polypeptide 1 pseudogene 1	0.98461	1.026885	1.0552931
1339	COX6A2	cytochrome c oxidase subunit VIa polypeptide 2	0.98037	1.054335	1.0185336
1340	COX6B1	cytochrome c oxidase subunit VIb polypeptide 1 (ubiquitous)	0.95381	0.924544	1.086562
1343	COX6B1P3	cytochrome c oxidase subunit VIb polypeptide 1 (ubiquitous) pseudogene 3	0.99513	1.022707	0.9981893
1345	COX6C	cytochrome c oxidase subunit VIc	0.99903	0.939347	1.0384074
1346	COX7A1	cytochrome c oxidase subunit VIIa polypeptide 1 (muscle)	0.97063	1.004276	0.9952165
1347	COX7A2	cytochrome c oxidase subunit VIIa polypeptide 2 (liver)	0.98089	0.942525	1.0744789
1349	COX7B	cytochrome c oxidase subunit VIIb	0.95235	0.995204	1.0374247
1350	COX7C	cytochrome c oxidase subunit VIIc	1.01266	0.908342	0.9332728
1351	COX8A	cytochrome c oxidase subunit VIIIA (ubiquitous)	0.95747	0.940284	1.0489631

1352	COX10	COX10 homolog, cytochrome c oxidase assembly protein, heme A: farnesyltransferase (yeast)	0.98396	0.955186	1.0324989
1353	COX11	COX11 cytochrome c oxidase assembly homolog (yeast)	0.96712	0.974016	1.0346932
1355	COX15	COX15 homolog, cytochrome c oxidase assembly protein (yeast)	0.93449	0.987404	1.0346658
1357	CPA1	carboxypeptidase A1 (pancreatic)	1.00729	0.997874	1.0360686
1358	CPA2	carboxypeptidase A2 (pancreatic)	1.01985	0.988091	1.0068411
1359	CPA3	carboxypeptidase A3 (mast cell)	0.99291	0.971766	1.0214327
1360	CPB1	carboxypeptidase B1 (tissue)	1.00337	1.004898	1.0145409
1361	CPB2	carboxypeptidase B2 (plasma)	0.97927	0.98189	0.9908265
1362	CPD	carboxypeptidase D	0.95272	1.0587	0.9759596
1363	CPE	carboxypeptidase E	1.00547	1.001414	1.0224666
1364	CLDN4	claudin 4	1.01578	0.982616	1.0318624
1365	CLDN3	claudin 3	0.97727	0.997099	1.0186769
1366	CLDN7	claudin 7	0.9758	1.035636	1.0249785
1368	CPM	carboxypeptidase M	0.97366	0.91916	1.0224517
1370	CPN2	carboxypeptidase N, polypeptide 2	0.97338	1.062876	1.0506638
1371	CPOX	coproporphyrinogen oxidase	1.01141	1.019319	1.0514858
1373	CPS1	carbamoyl-phosphate synthase 1, mitochondrial	1.00849	0.995265	1.0098125
1374	CPT1A	carnitine palmitoyltransferase 1A (liver)	0.99168	0.99071	0.9514747
1376	CPT2	carnitine palmitoyltransferase 2	0.95876	0.953947	1.0687081
1378	CR1	complement component (3b/4b) receptor 1 (Knops blood group)	1.03046	0.98748	0.9790479
1379	CR1L	complement component (3b/4b) receptor 1-like	1.0232	0.976199	1.0140933
1380	CR2	complement component (3d/Epstein Barr virus) receptor 2	0.95668	0.983525	1.0013249
1381	CRABP1	cellular retinoic acid binding protein 1	0.97055	1.041846	1.0783225
1382	CRABP2	cellular retinoic acid binding protein 2	0.99033	1.013203	0.9949705
1384	CRAT	carnitine O-acetyltransferase	0.93908	0.917463	1.0541194
1385	CREB1	cAMP responsive element binding protein 1	1.00892	0.949601	1.0448829
1386	ATF2	activating transcription factor 2	0.99358	0.976222	1.1051299
1387	CREBBP	CREB binding protein	0.98147	0.977077	0.9873593
1388	ATF6B	activating transcription factor 6 beta	1.01767	0.961541	1.1075204
1389	CREBL2	cAMP responsive element binding protein-like 2	0.92969	0.911654	0.926196
1390	CREM	cAMP responsive element modulator	0.98226	1.02053	0.9198475
1392	CRH	corticotropin releasing hormone	1.00264	1.020541	0.9614356
1393	CRHBP	corticotropin releasing hormone binding protein	1.00128	1.017701	0.9674279

1394	CRHR1	corticotropin releasing hormone receptor 1	1.00479	1.025481	0.9979849
1395	CRHR2	corticotropin releasing hormone receptor 2	1.00427	1.009311	1.0134991
1396	CRIP1	cysteine-rich protein 1 (intestinal)	0.96586	0.993023	1.042399
1397	CRIP2	cysteine-rich protein 2	0.96083	0.913098	0.9741292
1398	CRK	v-crk sarcoma virus CT10 oncogene homolog (avian)	0.9774	0.942199	0.9580284
1399	CRKL	v-crk sarcoma virus CT10 oncogene homolog (avian)-like	0.93351	1.034089	1.0841537
1400	CRMP1	collapsin response mediator protein 1	1.00209	1.005565	1.0375289
1401	CRP	C-reactive protein, pentraxin-related	0.99661	0.988365	1.0216517
1404	HAPLN1	hyaluronan and proteoglycan link protein 1	0.99344	0.975714	1.0050079
1406	CRX	cone-rod homeobox	0.98426	1.017419	1.0151775
1407	CRY1	cryptochrome 1 (photolyase-like)	0.94388	1.014441	0.939212
1408	CRY2	cryptochrome 2 (photolyase-like)	0.94803	0.924676	0.9451521
1409	CRYAA	crystallin, alpha A	1.00337	1.002247	0.9865511
1410	CRYAB	crystallin, alpha B	0.99296	0.979553	1.0041219
1411	CRYBA1	crystallin, beta A1	1.00488	1.025212	0.9926113
1412	CRYBA2	crystallin, beta A2	0.9949	0.968485	1.0288305
1413	CRYBA4	crystallin, beta A4	1.00585	0.986555	0.9572312
1414	CRYBB1	crystallin, beta B1	0.97127	0.998084	1.0275884
1415	CRYBB2	crystallin, beta B2	0.998	0.975785	0.9616755
1416	CRYBB2P1	crystallin, beta B2 pseudogene 1	0.96805	0.975625	1.061197
1417	CRYBB3	crystallin, beta B3	0.98126	1.008004	1.0544651
1418	CRYGA	crystallin, gamma A	1.00196	1.061414	1.0325766
1419	CRYGB	crystallin, gamma B	1.03932	1.040542	0.9972394
1420	CRYGC	crystallin, gamma C	0.98637	1.004401	1.0605503
1421	CRYGD	crystallin, gamma D	1.02377	0.95856	1.0337925
1423	CRYGFP	crystallin, gamma F pseudogene	0.98315	0.986944	1.052699
1424	CRYGGP	crystallin, gamma G, pseudogene	0.96973	0.959337	1.0066326
1427	CRYGS	crystallin, gamma S	1.03904	0.988246	1.0083763
1428	CRYM	crystallin, mu	0.98931	0.997451	1.004757
1429	CRYZ	crystallin, zeta (quinone reductase)	0.96022	0.929193	0.9634016
1431	CS	citrate synthase	0.99207	1.052368	1.0210244
1432	MAPK14	mitogen-activated protein kinase 14	0.99718	1.007525	1.0162831
1434	CSE1L	CSE1 chromosome segregation 1-like (yeast)	0.96552	1.044969	1.0248694

1435	CSF1	colony stimulating factor 1 (macrophage)	0.99635	1.03344	0.938027
1436	CSF1R	colony stimulating factor 1 receptor	1.07805	0.851342	1.0426178
1437	CSF2	colony stimulating factor 2 (granulocyte-macrophage)	1.0082	1.023316	1.008527
1439	CSF2RB	colony stimulating factor 2 receptor, beta, low-affinity (granulocyte-macrophage)	0.97388	0.895617	0.961292
1440	CSF3	colony stimulating factor 3 (granulocyte)	0.98836	1.015649	1.0565358
1441	CSF3R	colony stimulating factor 3 receptor (granulocyte)	1.01809	0.939963	0.9623353
1444	CSHL1	chorionic somatomammotropin hormone-like 1	1.01323	1.001852	0.9936389
1445	CSK	c-src tyrosine kinase	0.94676	0.981175	0.9647595
1446	CSN1S1	casein alpha s1	1.01958	1.035495	1.0219997
1447	CSN2	casein beta	1.00774	1.014004	0.9968578
1448	CSN3	casein kappa	1.01334	1.006892	1.0153245
1452	CSNK1A1	casein kinase 1, alpha 1	1.01274	0.943183	0.9546596
1453	CSNK1D	casein kinase 1, delta	0.95622	1.12758	0.9614633
1454	CSNK1E	casein kinase 1, epsilon	0.96612	0.988251	0.9840991
1455	CSNK1G2	casein kinase 1, gamma 2	0.97927	1.007012	1.0170864
1456	CSNK1G3	casein kinase 1, gamma 3	0.99469	0.949672	1.0450585
1457	CSNK2A1	casein kinase 2, alpha 1 polypeptide	0.98905	0.968	1.0424893
1459	CSNK2A2	casein kinase 2, alpha prime polypeptide	0.95377	0.950282	1.0440082
1460	CSNK2B	casein kinase 2, beta polypeptide	1.00303	0.988458	1.0255541
1462	VCAN	versican	1.06213	0.928846	1.0910022
1463	NCAN	neurocan	0.99063	1.002406	1.0193447
1464	CSPG4	chondroitin sulfate proteoglycan 4	0.9937	0.973284	1.0026141
1465	CSRP1	cysteine and glycine-rich protein 1	0.98181	0.922847	1.0629119
1466	CSRP2	cysteine and glycine-rich protein 2	0.99447	1.026437	1.0154796
1468	SLC25A10	solute carrier family 25 (mitochondrial carrier; dicarboxylate transporter), member 10	0.98261	0.989018	1.0393905
1469	CST1	cystatin SN	0.97592	0.999128	0.9896072
1471	CST3	cystatin C	0.97313	0.981046	0.9822652
1472	CST4	cystatin S	1.04644	0.998744	1.0360379
1473	CST5	cystatin D	1.03836	1.047893	1.0292825
1474	CST6	cystatin E/M	0.9853	0.933179	1.0244324
1475	CSTA	cystatin A (stefin A)	0.9957	1.078867	1.0124692
1476	CSTB	cystatin B (stefin B)	0.97958	0.9863	1.0450102
1477	CSTF1	cleavage stimulation factor, 3' pre-RNA, subunit 1, 50kDa	0.90202	0.861128	1.0474162

1478	CSTF2	cleavage stimulation factor, 3' pre-RNA, subunit 2, 64kDa	0.94194	1.013151	1.0691449
1479	CSTF3	cleavage stimulation factor, 3' pre-RNA, subunit 3, 77kDa	0.94768	0.992529	1.0543735
1480	CSTP1	cystatin pseudogene 1	1.05976	1.017306	0.9822903
1481	CSTP2	cystatin pseudogene 2	0.99934	1.026119	1.0237297
1482	NKX2-5	NK2 homeobox 5	0.99565	0.986014	1.0712758
1486	CTBS	chitobiase, di-N-acetyl-	0.98437	0.917026	0.898942
1487	CTBP1	C-terminal binding protein 1	1.02752	0.976036	1.0114879
1488	CTBP2	C-terminal binding protein 2	0.97825	0.992346	0.9795676
1489	CTF1	cardiotrophin 1	0.99734	0.96485	1.1111111
1490	CTGF	connective tissue growth factor	1.00487	0.965601	0.9858858
1491	CTH	cystathionase (cystathionine gamma-lyase)	0.97571	0.998175	0.9942501
1493	CTLA4	cytotoxic T-lymphocyte-associated protein 4	0.99723	0.894639	0.9539514
1495	CTNNA1	catenin (cadherin-associated protein), alpha 1, 102kDa	1.01206	0.974751	1.0849695
1496	CTNNA2	catenin (cadherin-associated protein), alpha 2	1.01386	1.001509	1.0192935
1497	CTNS	cystinosin, lysosomal cystine transporter	0.92195	0.919028	1.0294962
1498	CTNNA1P1	catenin (cadherin-associated protein), alpha 1 pseudogene 1	1.00531	0.996971	1.0089343
1499	CTNNB1	catenin (cadherin-associated protein), beta 1, 88kDa	1.01321	0.974152	1.00145
1500	CTNND1	catenin (cadherin-associated protein), delta 1	0.95918	1.043556	1.0802443
1501	CTNND2	catenin (cadherin-associated protein), delta 2 (neural plakophilin-related arm-repeat protein)	1.00385	1.008902	1.0197274
1503	CTPS	CTP synthase	0.97027	1.004373	0.9744877
1504	CTRB1	chymotrypsinogen B1	0.98009	0.976129	1.05145
1506	CTRL	chymotrypsin-like	0.98432	0.985389	0.9795147
1508	CTSB	cathepsin B	1.00184	0.969054	1.0078247
1509	CTSD	cathepsin D	1.00005	1.041111	0.9759346
1510	CTSE	cathepsin E	1.02309	0.98059	0.9887788
1511	CTSG	cathepsin G	1.00323	0.983844	0.9877521
1512	CTSH	cathepsin H	0.93709	0.939129	1.1281254
1513	CTSK	cathepsin K	1.02205	1.038744	0.9672094
1514	CTSL1	cathepsin L1	1.00031	0.98758	1.0179198
1515	CTSL2	cathepsin L2	1.01104	1.021749	0.9899458
1518	CTSL1P8	cathepsin L1 pseudogene 8	0.98919	1.005578	1.008561
1519	CTSO	cathepsin O	1.00828	0.841508	0.9356742
1520	CTSS	cathepsin S	1.03223	0.852869	0.9520874

1521	CTSW	cathepsin W	0.96522	1.0874	0.9515371
1522	CTSZ	cathepsin Z	0.98078	0.885283	0.9606505
1523	CUX1	cut-like homeobox 1	1.00408	0.993244	0.9814793
1524	CX3CR1	chemokine (C-X3-C motif) receptor 1	1.02514	0.9038	0.9570182
1525	CXADR	coxsackie virus and adenovirus receptor	0.99351	0.974836	0.967799
1528	CYB5A	cytochrome b5 type A (microsomal)	0.98826	1.027044	1.0034079
1531	CYB5AP3	cytochrome b5 type A (microsomal) pseudogene 3	1.01159	1.037058	1.0165197
1532	CYB5AP4	cytochrome b5 type A (microsomal) pseudogene 4	1.02087	1.042383	1.0283973
1534	CYB561	cytochrome b-561	0.97958	0.964627	1.0528974
1535	CYBA	cytochrome b-245, alpha polypeptide	0.99688	1.074517	0.9701484
1536	CYBB	cytochrome b-245, beta polypeptide	1.03314	0.960873	0.99168
1537	CYC1	cytochrome c-1	1.00592	0.947732	1.1262657
1538	CYLC1	cylicin, basic protein of sperm head cytoskeleton 1	0.97875	1.01701	0.9942211
1539	CYLC2	cylicin, basic protein of sperm head cytoskeleton 2	1.01487	0.965221	0.9899578
1540	CYLD	cylindromatosis (turban tumor syndrome)	0.9392	1.06389	0.9717967
1543	CYP1A1	cytochrome P450, family 1, subfamily A, polypeptide 1	0.99334	1.029117	0.9895997
1544	CYP1A2	cytochrome P450, family 1, subfamily A, polypeptide 2	0.99527	0.990096	1.0315062
1545	CYP1B1	cytochrome P450, family 1, subfamily B, polypeptide 1	0.98616	0.992615	1.1310105
1549	CYP2A7	cytochrome P450, family 2, subfamily A, polypeptide 7	1.00378	0.939052	0.9737209
1550	CYP2A7P1	cytochrome P450, family 2, subfamily A, polypeptide 7 pseudogene 1	0.98324	1.011854	1.0259605
1553	CYP2A13	cytochrome P450, family 2, subfamily A, polypeptide 13	1.02162	0.986935	0.9675005
1555	CYP2B6	cytochrome P450, family 2, subfamily B, polypeptide 6	1.00435	1.024392	1.0462643
1556	CYP2B7P1	cytochrome P450, family 2, subfamily B, polypeptide 7 pseudogene 1	1.0042	0.995088	1.0165265
1557	CYP2C19	cytochrome P450, family 2, subfamily C, polypeptide 19	0.98287	1.025089	0.9884222
1558	CYP2C8	cytochrome P450, family 2, subfamily C, polypeptide 8	0.99433	1.00626	1.0053175
1559	CYP2C9	cytochrome P450, family 2, subfamily C, polypeptide 9	0.99964	0.985442	1.0277105
1562	CYP2C18	cytochrome P450, family 2, subfamily C, polypeptide 18	1.00009	1.021377	0.9734096
1564	CYP2D7P1	cytochrome P450, family 2, subfamily D, polypeptide 7 pseudogene 1	0.9798	0.973072	1.0005838
1568	CYP2D8P1	cytochrome P450, family 2, subfamily D, polypeptide 8 pseudogene 1	0.97552	0.952347	1.0292683
1571	CYP2E1	cytochrome P450, family 2, subfamily E, polypeptide 1	0.97789	0.999726	0.9956272
1572	CYP2F1	cytochrome P450, family 2, subfamily F, polypeptide 1	0.99595	0.963076	1.0089817
1573	CYP2J2	cytochrome P450, family 2, subfamily J, polypeptide 2	0.9784	0.992687	1.0389882
1579	CYP4A11	cytochrome P450, family 4, subfamily A, polypeptide 11	1.02081	0.983048	1.0101129

1580	CYP4B1	cytochrome P450, family 4, subfamily B, polypeptide 1	1.00517	1.013755	0.994884
1581	CYP7A1	cytochrome P450, family 7, subfamily A, polypeptide 1	0.99141	1.025444	1.0400767
1582	CYP8B1	cytochrome P450, family 8, subfamily B, polypeptide 1	1.01599	0.9913	0.9525145
1583	CYP11A1	cytochrome P450, family 11, subfamily A, polypeptide 1	0.99573	0.993799	1.0273351
1584	CYP11B1	cytochrome P450, family 11, subfamily B, polypeptide 1	1.00969	1.00354	1.0141034
1585	CYP11B2	cytochrome P450, family 11, subfamily B, polypeptide 2	1.00666	0.98706	1.0236593
1586	CYP17A1	cytochrome P450, family 17, subfamily A, polypeptide 1	0.99739	0.995706	0.9744223
1587	ADAM3A	ADAM metallopeptidase domain 3A	0.99108	0.975443	1.0292737
1588	CYP19A1	cytochrome P450, family 19, subfamily A, polypeptide 1	0.99274	0.990968	1.0016611
1591	CYP24A1	cytochrome P450, family 24, subfamily A, polypeptide 1	0.9825	1.001695	1.0202403
1592	CYP26A1	cytochrome P450, family 26, subfamily A, polypeptide 1	0.98279	0.938711	1.0126024
1593	CYP27A1	cytochrome P450, family 27, subfamily A, polypeptide 1	0.95464	1.004347	0.8939969
1594	CYP27B1	cytochrome P450, family 27, subfamily B, polypeptide 1	0.98444	1.001896	1.0269801
1595	CYP51A1	cytochrome P450, family 51, subfamily A, polypeptide 1	0.98559	0.988624	1.019113
1600	DAB1	disabled homolog 1 (Drosophila)	1.01173	0.99134	1.0204101
1601	DAB2	disabled homolog 2, mitogen-responsive phosphoprotein (Drosophila)	1.00015	0.901521	0.9969681
1602	DACH1	dachshund homolog 1 (Drosophila)	0.99242	1.013159	1.0000442
1603	DAD1	defender against cell death 1	1.01835	0.979268	0.9681444
1604	CD55	CD55 molecule, decay accelerating factor for complement (Cromer blood group)	1.00777	1.122339	0.9491436
1605	DAG1	dystroglycan 1 (dystrophin-associated glycoprotein 1)	1.0336	0.971674	1.0495377
1606	DGKA	diacylglycerol kinase, alpha 80kDa	0.93113	0.993992	0.9787255
1607	DGKB	diacylglycerol kinase, beta 90kDa	1.00816	1.010225	1.0096085
1608	DGKG	diacylglycerol kinase, gamma 90kDa	0.98179	0.978087	1.0005041
1609	DGKQ	diacylglycerol kinase, theta 110kDa	0.98207	0.972099	1.0000389
1610	DAO	D-amino-acid oxidase	1.00044	0.991446	1.0022415
1611	DAP	death-associated protein	0.98883	0.986007	0.9831402
1612	DAPK1	death-associated protein kinase 1	1.02208	0.988438	1.0805564
1613	DAPK3	death-associated protein kinase 3	0.95701	1.008464	1.016826
1615	DARS	aspartyl-tRNA synthetase	0.97106	0.971765	1.0197058
1616	DAXX	death-domain associated protein	0.95789	1.057689	1.0263114
1618	DAZL	deleted in azoospermia-like	1.00546	0.997825	1.0011357
1620	DBC1	deleted in bladder cancer 1	0.99768	0.989658	0.9999055
1621	DBH	dopamine beta-hydroxylase (dopamine beta-monooxygenase)	1.00664	0.973177	0.9863292

1622	DBI	diazepam binding inhibitor (GABA receptor modulator, acyl-CoA binding protein)	1.02594	0.950919	1.0226778
1623	DBIP2	diazepam binding inhibitor (GABA receptor modulator, acyl-CoA binding protein) pseudogene 2	0.98702	0.997821	1.0061773
1627	DBN1	drebrin 1	0.95972	1.022029	1.0136626
1628	DBP	D site of albumin promoter (albumin D-box) binding protein	0.95823	0.95082	1.0497829
1629	DBT	dihydrolipoamide branched chain transacylase E2	0.99447	0.972932	0.9688051
1630	DCC	deleted in colorectal carcinoma	1.00075	0.993125	0.9985889
1632	ECI1	enoyl-CoA delta isomerase 1	0.96625	0.94884	1.101156
1633	DCK	deoxycytidine kinase	0.95122	0.875558	1.0173431
1634	DCN	decorin	0.99936	1.005303	1.003197
1635	DCTD	dCMP deaminase	1.01147	0.982339	0.9903128
1636	ACE	angiotensin I converting enzyme (peptidyl-dipeptidase A) 1	0.98867	1.001287	0.9960232
1638	DCT	dopachrome tautomerase (dopachrome delta-isomerase, tyrosine-related protein 2)	0.99012	0.981974	0.9839484
1639	DCTN1	dynactin 1	0.99045	0.961856	1.0934469
1641	DCX	doublecortin	0.99128	0.987028	1.0060387
1642	DDB1	damage-specific DNA binding protein 1, 127kDa	0.97407	1.043932	1.1053577
1643	DDB2	damage-specific DNA binding protein 2, 48kDa	1.00248	0.981373	1.0101432
1644	DDC	dopa decarboxylase (aromatic L-amino acid decarboxylase)	1.00337	0.995456	0.9996388
1645	AKR1C1	aldo-keto reductase family 1, member C1 (dihydrodiol dehydrogenase 1; 20-alpha (3-alpha)-hydroxysteroid d	1.0148	1.020707	1.0227382
1646	AKR1C2	aldo-keto reductase family 1, member C2 (dihydrodiol dehydrogenase 2; bile acid binding protein; 3-alpha hy	0.99388	1.050009	0.9819928
1647	GADD45A	growth arrest and DNA-damage-inducible, alpha	1.0046	1.015446	1.0241241
1649	DDIT3	DNA-damage-inducible transcript 3	0.95647	0.990095	0.9650524
1650	DDOST	dolichyl-diphosphooligosaccharide--protein glycosyltransferase	1.01138	0.970694	1.0470885
1652	DDT	D-dopachrome tautomerase	0.9037	1.015806	1.0718764
1653	DDX1	DEAD (Asp-Glu-Ala-Asp) box polypeptide 1	0.96634	0.922179	1.0263692
1654	DDX3X	DEAD (Asp-Glu-Ala-Asp) box polypeptide 3, X-linked	0.97764	0.901007	1.0172744
1655	DDX5	DEAD (Asp-Glu-Ala-Asp) box polypeptide 5	1.04697	0.942906	0.9938457
1656	DDX6	DEAD (Asp-Glu-Ala-Asp) box polypeptide 6	0.97115	0.992486	0.9739054
1657	DMXL1	Dmx-like 1	0.99766	1.022214	0.9394327
1659	DHX8	DEAH (Asp-Glu-Ala-His) box polypeptide 8	0.92607	0.875325	0.9711771
1660	DHX9	DEAH (Asp-Glu-Ala-His) box polypeptide 9	0.99602	0.895532	0.9801155
1661	DHX9P1	DEAH (Asp-Glu-Ala-His) box polypeptide 9 pseudogene 1	1.00536	0.990502	1.0110336
1662	DDX10	DEAD (Asp-Glu-Ala-Asp) box polypeptide 10	0.97044	0.987863	0.9949202
1663	DDX11	DEAD/H (Asp-Glu-Ala-Asp/His) box polypeptide 11	0.95773	0.98504	1.037134

1665	DHX15	DEAH (Asp-Glu-Ala-His) box polypeptide 15	1.02356	0.96174	1.079345
1666	DECR1	2,4-dienoyl CoA reductase 1, mitochondrial	0.97431	0.956318	0.9717514
1669	DEFA4	defensin, alpha 4, corticostatin	0.97202	0.919223	0.9518832
1670	DEFA5	defensin, alpha 5, Paneth cell-specific	1.00118	1.030085	0.9958393
1671	DEFA6	defensin, alpha 6, Paneth cell-specific	0.99547	1.024417	0.9728424
1672	DEFB1	defensin, beta 1	0.99777	0.990285	0.9926325
1674	DES	desmin	1.00501	0.963332	1.0290845
1676	DFFA	DNA fragmentation factor, 45kDa, alpha polypeptide	0.95523	1.005199	1.0363267
1677	DFFB	DNA fragmentation factor, 40kDa, beta polypeptide (caspase-activated DNase)	0.98366	0.992708	1.0363925
1678	TIMM8A	translocase of inner mitochondrial membrane 8 homolog A (yeast)	0.99206	0.988627	1.055874
1687	DFNA5	deafness, autosomal dominant 5	1.01326	1.000916	1.0066225
1690	COCH	coagulation factor C homolog, cochlin (Limulus polyphemus)	0.97279	0.954176	0.9869833
1716	DGUOK	deoxyguanosine kinase	0.99014	0.949386	0.9722725
1717	DHCR7	7-dehydrocholesterol reductase	0.95832	0.986962	0.9981866
1718	DHCR24	24-dehydrocholesterol reductase	1.01783	0.964243	1.0563533
1719	DHFR	dihydrofolate reductase	1.01005	0.961715	1.0206203
1720	LOC1720	dihydrofolate reductase pseudogene	0.99279	0.976709	0.9742368
1723	DHODH	dihydroorotate dehydrogenase	0.98173	0.971284	0.9749703
1725	DHPS	deoxyhypusine synthase	0.94739	0.97709	1.0268279
1727	CYB5R3	cytochrome b5 reductase 3	0.92384	0.983745	1.0565327
1728	NQO1	NAD(P)H dehydrogenase, quinone 1	0.96875	0.981412	0.997765
1729	DIAPH1	diaphanous homolog 1 (Drosophila)	1.0206	1.001308	1.0265437
1730	DIAPH2	diaphanous homolog 2 (Drosophila)	0.98526	0.991046	1.0528377
1731	1-Sep	septin 1	0.92164	0.945814	1.0023577
1733	DIO1	deiodinase, iodothyronine, type I	1.01279	0.982437	0.9995874
1734	DIO2	deiodinase, iodothyronine, type II	0.98317	1.015059	0.9965609
1735	DIO3	deiodinase, iodothyronine, type III	0.97866	1.022311	1.0352134
1736	DKC1	dyskeratosis congenita 1, dyskerin	0.93107	0.995511	1.0396938
1737	DLAT	dihydrolipoamide S-acetyltransferase	0.92099	0.985452	1.024106
1738	DLD	dihydrolipoamide dehydrogenase	1.02968	0.94411	0.9111303
1739	DLG1	discs, large homolog 1 (Drosophila)	0.9836	1.040364	0.9972801
1740	DLG2	discs, large homolog 2 (Drosophila)	0.99637	1.006375	1.0052406
1741	DLG3	discs, large homolog 3 (Drosophila)	0.97109	1.045423	1.0216878

1742	DLG4	discs, large homolog 4 (Drosophila)	0.95566	0.937854	0.9775276
1743	DLST	dihydrolipoamide S-succinyltransferase (E2 component of 2-oxo-glutarate complex)	0.96737	0.932789	1.0511605
1744	DLSTP1	dihydrolipoamide S-succinyltransferase pseudogene 1	1.01104	0.972932	1.0305566
1745	DLX1	distal-less homeobox 1	0.98882	1.012037	1.0278051
1746	DLX2	distal-less homeobox 2	1.00301	0.9474	1.0443002
1747	DLX3	distal-less homeobox 3	0.97822	1.048949	1.0083196
1748	DLX4	distal-less homeobox 4	0.98497	0.995958	1.0021851
1749	DLX5	distal-less homeobox 5	0.99432	1.003995	1.0160629
1750	DLX6	distal-less homeobox 6	0.99881	0.993237	0.9966824
1755	DMBT1	deleted in malignant brain tumors 1	1.01655	0.979119	1.0161759
1756	DMD	dystrophin	0.99008	1.012122	0.9929734
1757	SARDH	sarcosine dehydrogenase	0.99194	0.999191	1.018221
1758	DMP1	dentin matrix acidic phosphoprotein 1	1.01221	1.038506	1.0093695
1759	DNM1	dynamin 1	0.97834	1.015823	0.9858347
1760	DMPK	dystrophia myotonica-protein kinase	0.98333	1.041698	1.0334357
1761	DMRT1	doublesex and mab-3 related transcription factor 1	1.00788	1.023683	0.9963373
1762	DMWD	dystrophia myotonica, WD repeat containing	1.00155	0.961429	1.0367061
1763	DNA2	DNA replication helicase 2 homolog (yeast)	0.95168	1.046295	1.0049419
1767	DNAH5	dynein, axonemal, heavy chain 5	1.00713	1.003711	1.0153014
1769	DNAH8	dynein, axonemal, heavy chain 8	1.01984	0.99737	1.0183398
1770	DNAH9	dynein, axonemal, heavy chain 9	0.99952	0.999059	1.004794
1773	DNASE1	deoxyribonuclease I	0.95147	0.945273	1.0326578
1774	DNASE1L1	deoxyribonuclease I-like 1	0.94991	0.948076	1.0359973
1775	DNASE1L2	deoxyribonuclease I-like 2	0.98209	0.993893	1.0811939
1776	DNASE1L3	deoxyribonuclease I-like 3	1.02164	1.014464	1.0349906
1777	DNASE2	deoxyribonuclease II, lysosomal	0.88717	0.803496	1.1723792
1778	DYNC1H1	dynein, cytoplasmic 1, heavy chain 1	0.96042	1.048993	0.9772172
1780	DYNC1I1	dynein, cytoplasmic 1, intermediate chain 1	1.00706	1.010579	1.0105183
1781	DYNC1I2	dynein, cytoplasmic 1, intermediate chain 2	1.02209	0.938815	1.1015952
1783	DYNC1LI2	dynein, cytoplasmic 1, light intermediate chain 2	0.97561	1.058881	1.0576864
1785	DNM2	dynamin 2	0.99007	1.007307	1.029807
1786	DNMT1	DNA (cytosine-5-)-methyltransferase 1	0.95209	0.917144	1.0126631
1787	TRDMT1	tRNA aspartic acid methyltransferase 1	0.93846	0.989111	0.9153311

1788	DNMT3A	DNA (cytosine-5-)-methyltransferase 3 alpha	0.98926	0.929486	1.0123457
1789	DNMT3B	DNA (cytosine-5-)-methyltransferase 3 beta	0.98493	0.992832	1.0521952
1791	DNTT	deoxynucleotidyltransferase, terminal	0.99004	0.973416	0.9895295
1793	DOCK1	dedicator of cytokinesis 1	0.99295	1.002266	0.9988579
1794	DOCK2	dedicator of cytokinesis 2	1.01075	0.959782	1.0499398
1795	DOCK3	dedicator of cytokinesis 3	1.01136	0.971048	1.0038433
1796	DOK1	docking protein 1, 62kDa (downstream of tyrosine kinase 1)	0.99931	0.975202	1.0497044
1797	DOM3Z	dom-3 homolog Z (C. elegans)	0.98553	0.968488	0.987704
1798	DPAGT1	dolichyl-phosphate (UDP-N-acetylglucosamine) N-acetylglucosaminephosphotransferase 1 (GlcNAc-1-P trans	0.979	0.974769	1.0632941
1800	DPEP1	dipeptidase 1 (renal)	0.9903	0.996459	1.0025601
1802	DPH2	DPH2 homolog (S. cerevisiae)	0.97821	0.949943	1.0321084
1803	DPP4	dipeptidyl-peptidase 4	0.93546	0.877976	1.052996
1804	DPP6	dipeptidyl-peptidase 6	1.01037	0.993104	1.0071102
1805	DPT	dermatopontin	1.01676	1.003702	1.0085323
1806	DPYD	dihydropyrimidine dehydrogenase	1.02831	0.962071	1.0077637
1807	DPYS	dihydropyrimidinase	0.99499	0.99123	1.0152351
1809	DPYSL3	dihydropyrimidinase-like 3	1.0155	1.012532	1.001113
1810	DR1	down-regulator of transcription 1, TBP-binding (negative cofactor 2)	1.01027	0.973146	1.0621813
1811	SLC26A3	solute carrier family 26, member 3	1.01578	0.996046	0.9988564
1812	DRD1	dopamine receptor D1	1.00357	0.964588	0.997502
1813	DRD2	dopamine receptor D2	0.99797	0.970234	0.9487497
1814	DRD3	dopamine receptor D3	1.00122	1.012549	1.009055
1815	DRD4	dopamine receptor D4	1.00903	0.996113	1.0679424
1816	DRD5	dopamine receptor D5	1.00285	1.064252	1.1031376
1819	DRG2	developmentally regulated GTP binding protein 2	0.95812	0.91964	1.0229261
1820	ARID3A	AT rich interactive domain 3A (BRIGHT-like)	0.97671	1.010532	1.0555313
1821	DRP2	dystrophin related protein 2	1.00335	0.980426	1.0020651
1822	ATN1	atrophin 1	0.97327	1.019021	0.9794669
1823	DSC1	desmocollin 1	0.90106	0.876634	0.8914922
1824	DSC2	desmocollin 2	0.92663	0.964241	1.0754402
1825	DSC3	desmocollin 3	0.99749	1.002562	1.0004697
1826	DSCAM	Down syndrome cell adhesion molecule	1	0.997255	1.0043602
1827	RCAN1	regulator of calcineurin 1	1.00504	0.983581	1.0279143

1828	DSG1	desmoglein 1	1.00036	1.013281	1.0031499
1829	DSG2	desmoglein 2	1.00273	1.009298	1.0088554
1830	DSG3	desmoglein 3	0.98368	0.9792	1.0164369
1831	TSC22D3	TSC22 domain family, member 3	0.95993	0.994393	0.9923807
1832	DSP	desmoplakin	0.98361	1.007901	1.0340193
1833	EPYC	epiphycan	0.99741	1.007947	0.9920668
1834	DSPP	dentin sialophosphoprotein	1.01542	0.986319	0.9965398
1836	SLC26A2	solute carrier family 26 (sulfate transporter), member 2	0.98917	0.898085	1.0197494
1837	DTNA	dystrobrevin, alpha	0.99714	0.994429	1.0121742
1838	DTNB	dystrobrevin, beta	0.98846	0.95578	0.9500022
1839	HBEGF	heparin-binding EGF-like growth factor	1.03974	0.959997	0.8832751
1840	DTX1	deltex homolog 1 (Drosophila)	0.96883	1.009826	0.9955387
1841	DTYMK	deoxythymidylate kinase (thymidylate kinase)	1.01608	1.033299	0.9870474
1843	DUSP1	dual specificity phosphatase 1	1.0297	0.999429	0.9774149
1844	DUSP2	dual specificity phosphatase 2	1.01451	1.024312	0.9801033
1845	DUSP3	dual specificity phosphatase 3	0.92202	0.974059	1.0194766
1846	DUSP4	dual specificity phosphatase 4	0.99111	1.048352	1.0272962
1847	DUSP5	dual specificity phosphatase 5	1.00919	1.052325	0.9610183
1848	DUSP6	dual specificity phosphatase 6	0.96101	0.858792	0.8779782
1849	DUSP7	dual specificity phosphatase 7	0.96702	0.939258	1.0881997
1850	DUSP8	dual specificity phosphatase 8	0.99333	1.008294	0.95352
1852	DUSP9	dual specificity phosphatase 9	0.99511	0.958682	1.06828
1854	DUT	deoxyuridine triphosphatase	0.96859	0.94908	1.0371986
1855	DVL1	dishevelled, dsh homolog 1 (Drosophila)	1.01732	1.025522	1.0775351
1856	DVL2	dishevelled, dsh homolog 2 (Drosophila)	0.94769	0.977526	1.0026667
1857	DVL3	dishevelled, dsh homolog 3 (Drosophila)	1.02553	1.005024	0.9920292
1859	DYRK1A	dual-specificity tyrosine-(Y)-phosphorylation regulated kinase 1A	0.96818	0.973485	0.9683483
1861	TOR1A	torsin family 1, member A (torsin A)	0.93176	0.973836	1.1049004
1869	E2F1	E2F transcription factor 1	0.98835	1.027694	1.0017696
1870	E2F2	E2F transcription factor 2	1.01793	1.008327	1.072835
1871	E2F3	E2F transcription factor 3	0.98817	0.98701	1.0203578
1874	E2F4	E2F transcription factor 4, p107/p130-binding	0.95021	0.947787	1.1325049
1875	E2F5	E2F transcription factor 5, p130-binding	0.98478	1.025376	1.0811434

1876	E2F6	E2F transcription factor 6	1.01087	0.941558	1.0233741
1877	E4F1	E4F transcription factor 1	0.96421	0.948358	1.048691
1880	GPR183	G protein-coupled receptor 183	0.93846	0.988366	0.9012818
1889	ECE1	endothelin converting enzyme 1	1.01174	1.01441	0.9571195
1890	TYMP	thymidine phosphorylase	0.99221	0.938139	0.9799931
1891	ECH1	enoyl CoA hydratase 1, peroxisomal	0.96458	0.894375	1.0455096
1892	ECHS1	enoyl CoA hydratase, short chain, 1, mitochondrial	0.94217	0.876618	1.1155896
1893	ECM1	extracellular matrix protein 1	1.01197	1.024339	0.9921744
1894	ECT2	epithelial cell transforming sequence 2 oncogene	0.99593	0.947915	1.0464491
1896	EDA	ectodysplasin A	0.98427	0.939229	0.976046
1901	S1PR1	sphingosine-1-phosphate receptor 1	0.92655	0.951907	1.0178361
1902	LPAR1	lysophosphatidic acid receptor 1	0.98882	1.012188	0.9818735
1903	S1PR3	sphingosine-1-phosphate receptor 3	0.94107	0.864831	1.0392247
1906	EDN1	endothelin 1	1.00931	1.01229	0.9862078
1907	EDN2	endothelin 2	1.00725	0.999913	0.9872687
1908	EDN3	endothelin 3	0.98087	1.004897	1.0053519
1909	EDNRA	endothelin receptor type A	1.00529	1.015071	1.0332184
1910	EDNRB	endothelin receptor type B	0.99897	1.016468	1.0115085
1911	PHC1	polyhomeotic homolog 1 (Drosophila)	0.9988	1.00323	1.0247079
1912	PHC2	polyhomeotic homolog 2 (Drosophila)	1.0296	0.985084	1.005309
1915	EEF1A1	eukaryotic translation elongation factor 1 alpha 1	1.01042	0.896834	0.8868859
1917	EEF1A2	eukaryotic translation elongation factor 1 alpha 2	0.99307	0.98561	1.0603693
1932	EEF1B2P1	eukaryotic translation elongation factor 1 beta 2 pseudogene 1	0.99184	0.965971	0.9796197
1933	EEF1B2	eukaryotic translation elongation factor 1 beta 2	0.94151	1.001288	0.9882106
1934	EEF1B2P2	eukaryotic translation elongation factor 1 beta 2 pseudogene 2	0.98462	0.968223	1.0492897
1936	EEF1D	eukaryotic translation elongation factor 1 delta (guanine nucleotide exchange protein)	0.99587	0.986855	0.9543814
1937	EEF1G	eukaryotic translation elongation factor 1 gamma	0.98102	1.008488	0.9920482
1938	EEF2	eukaryotic translation elongation factor 2	0.94945	0.866618	1.0631678
1939	EIF2D	eukaryotic translation initiation factor 2D	0.97219	0.920635	0.9916287
1942	EFNA1	ephrin-A1	0.99142	0.987725	1.0103601
1943	EFNA2	ephrin-A2	0.97638	1.024038	1.1490549
1944	EFNA3	ephrin-A3	0.98793	1.008226	1.0232725
1945	EFNA4	ephrin-A4	1.00917	0.979636	1.0450128

1946	EFNA5	ephrin-A5	1.00695	1.007417	1.0100735
1947	EFNB1	ephrin-B1	0.98195	1.044074	1.0481079
1948	EFNB2	ephrin-B2	0.97909	0.997969	1.0191331
1949	EFNB3	ephrin-B3	0.99638	1.01214	0.9889949
1950	EGF	epidermal growth factor	0.96991	0.939968	0.9764679
1951	CELSR3	cadherin, EGF LAG seven-pass G-type receptor 3 (flamingo homolog, Drosophila)	0.9943	1	1
1952	CELSR2	cadherin, EGF LAG seven-pass G-type receptor 2 (flamingo homolog, Drosophila)	1.00474	0.986409	0.9921202
1953	MEGF6	multiple EGF-like-domains 6	1.00065	1.006355	1.0118709
1954	MEGF8	multiple EGF-like-domains 8	0.9701	0.972114	1.043176
1955	MEGF9	multiple EGF-like-domains 9	0.98172	0.97434	1.0342679
1956	EGFR	epidermal growth factor receptor	1.00292	1.010606	1.0203511
1958	EGR1	early growth response 1	1.01074	1.001913	0.7462475
1959	EGR2	early growth response 2	0.99336	1.080237	0.9834446
1960	EGR3	early growth response 3	1.00951	0.9622	1.0203418
1961	EGR4	early growth response 4	1.00503	0.991729	1.0909885
1962	EHHADH	enoyl-CoA, hydratase/3-hydroxyacyl CoA dehydrogenase	0.99037	0.990928	1.0202162
1964	EIF1AX	eukaryotic translation initiation factor 1A, X-linked	0.96363	0.929053	0.9497185
1965	EIF2S1	eukaryotic translation initiation factor 2, subunit 1 alpha, 35kDa	0.94578	0.986137	1.0266184
1967	EIF2B1	eukaryotic translation initiation factor 2B, subunit 1 alpha, 26kDa	0.97185	0.900093	1.0608201
1968	EIF2S3	eukaryotic translation initiation factor 2, subunit 3 gamma, 52kDa	1.00058	1.045047	1.1575067
1969	EPHA2	EPH receptor A2	1.0148	0.974672	1.0048976
1973	EIF4A1	eukaryotic translation initiation factor 4A1	1.01266	0.941296	0.9263795
1974	EIF4A2	eukaryotic translation initiation factor 4A2	1.02249	0.981812	0.8866255
1975	EIF4B	eukaryotic translation initiation factor 4B	0.9714	0.965499	1.0039695
1977	EIF4E	eukaryotic translation initiation factor 4E	0.9993	0.962205	0.8989707
1978	EIF4EBP1	eukaryotic translation initiation factor 4E binding protein 1	0.97459	0.951401	1.0332617
1979	EIF4EBP2	eukaryotic translation initiation factor 4E binding protein 2	0.98637	0.967304	0.9958437
1981	EIF4G1	eukaryotic translation initiation factor 4 gamma, 1	0.99298	0.955334	1.0324229
1982	EIF4G2	eukaryotic translation initiation factor 4 gamma, 2	1.00516	0.865219	0.995509
1983	EIF5	eukaryotic translation initiation factor 5	0.95734	0.961888	0.9669589
1984	EIF5A	eukaryotic translation initiation factor 5A	1.0074	0.970967	0.9711043
1986	EIF5AP2	eukaryotic translation initiation factor 5A pseudogene 2	1.00228	0.97652	0.9930946
1990	CELA1	chymotrypsin-like elastase family, member 1	1.00676	1.044435	0.972333

1991	ELANE	elastase, neutrophil expressed	0.99004	0.968473	0.9914106
1992	SERPINB1	serpin peptidase inhibitor, clade B (ovalbumin), member 1	1.01885	1.017	0.9566799
1993	ELAVL2	ELAV (embryonic lethal, abnormal vision, Drosophila)-like 2 (Hu antigen B)	0.9826	0.984608	0.9952365
1994	ELAVL1	ELAV (embryonic lethal, abnormal vision, Drosophila)-like 1 (Hu antigen R)	0.98368	0.965328	1.0201398
1995	ELAVL3	ELAV (embryonic lethal, abnormal vision, Drosophila)-like 3 (Hu antigen C)	0.99021	1.011283	1.0127787
1996	ELAVL4	ELAV (embryonic lethal, abnormal vision, Drosophila)-like 4 (Hu antigen D)	1.00275	1.004015	1.0021851
1997	ELF1	E74-like factor 1 (ets domain transcription factor)	0.97192	0.968624	0.9969124
1998	ELF2	E74-like factor 2 (ets domain transcription factor)	1.0103	0.993312	0.9739445
1999	ELF3	E74-like factor 3 (ets domain transcription factor, epithelial-specific)	0.98905	1.005315	1.0174456
2000	ELF4	E74-like factor 4 (ets domain transcription factor)	0.94397	1.070448	1.0165636
2001	ELF5	E74-like factor 5 (ets domain transcription factor)	0.98599	1.010156	1.0315933
2002	ELK1	ELK1, member of ETS oncogene family	0.97622	1.021916	1.0164892
2004	ELK3	ELK3, ETS-domain protein (SRF accessory protein 2)	0.97472	0.992499	1.0109537
2005	ELK4	ELK4, ETS-domain protein (SRF accessory protein 1)	1.01325	0.894434	0.9190292
2006	ELN	elastin	1.00841	1.007221	0.9979796
2009	EML1	echinoderm microtubule associated protein like 1	0.99622	1.005158	1.0196301
2010	EMD	emerin	0.94173	1.00063	0.9446449
2011	MARK2	MAP/microtubule affinity-regulating kinase 2	0.96912	1.00048	1.0123957
2012	EMP1	epithelial membrane protein 1	0.97074	0.950304	0.9829197
2013	EMP2	epithelial membrane protein 2	1.00209	0.983193	1.0049344
2014	EMP3	epithelial membrane protein 3	0.98963	1.001117	0.9929385
2015	EMR1	egf-like module containing, mucin-like, hormone receptor-like 1	1.0166	0.858868	0.9689454
2016	EMX1	empty spiracles homeobox 1	1.00857	0.976842	1.0325413
2017	CTTN	cortactin	0.93904	0.987474	1.0093065
2018	EMX2	empty spiracles homeobox 2	0.99047	0.968268	1.0688199
2019	EN1	engrailed homeobox 1	0.993	1.00327	1.0141968
2020	EN2	engrailed homeobox 2	1.00166	1.010047	1.066607
2021	ENDOG	endonuclease G	0.98858	0.981793	1.0416005
2022	ENG	endoglin	0.96445	1.039791	0.9563132
2023	ENO1	enolase 1, (alpha)	1.04159	0.96921	1.0631799
2026	ENO2	enolase 2 (gamma, neuronal)	0.94752	1.006655	0.9926217
2027	ENO3	enolase 3 (beta, muscle)	0.98471	0.998772	1.0108099
2028	ENPEP	glutamyl aminopeptidase (aminopeptidase A)	1.00367	0.991007	1.0222119

2029	ENSA	endosulfine alpha	0.98869	0.90725	0.9162
2030	SLC29A1	solute carrier family 29 (nucleoside transporters), member 1	1.00784	0.978816	1.0104652
2033	EP300	E1A binding protein p300	0.98844	0.990685	0.9867141
2034	EPAS1	endothelial PAS domain protein 1	1.01579	0.984758	0.9958934
2035	EPB41	erythrocyte membrane protein band 4.1 (elliptocytosis 1, RH-linked)	1.00293	0.947625	0.9819208
2036	EPB41L1	erythrocyte membrane protein band 4.1-like 1	0.99719	0.990531	1.0145312
2037	EPB41L2	erythrocyte membrane protein band 4.1-like 2	0.99669	0.99975	1.0124294
2038	EPB42	erythrocyte membrane protein band 4.2	0.99822	0.975885	1.0105273
2039	EPB49	erythrocyte membrane protein band 4.9 (dematin)	0.99079	0.926614	1.03332
2040	STOM	stomatin	0.98802	0.938928	0.9947289
2041	EPHA1	EPH receptor A1	0.96138	1.004857	0.9665104
2042	EPHA3	EPH receptor A3	0.99946	1.016277	1.0254834
2043	EPHA4	EPH receptor A4	0.99708	1.001731	1.017047
2044	EPHA5	EPH receptor A5	1.00343	1.019873	1.0276529
2045	EPHA7	EPH receptor A7	1.00093	1.011021	0.997868
2046	EPHA8	EPH receptor A8	1.00032	1.033763	1.0089641
2047	EPHB1	EPH receptor B1	1.01693	0.981622	1.0080913
2048	EPHB2	EPH receptor B2	1.01446	1.005168	1.0027993
2049	EPHB3	EPH receptor B3	1.01179	0.997272	1.0153117
2050	EPHB4	EPH receptor B4	0.99119	1.005351	0.9879432
2051	EPHB6	EPH receptor B6	0.96451	0.946976	1.0085777
2052	EPHX1	epoxide hydrolase 1, microsomal (xenobiotic)	0.98575	0.997925	0.9876237
2053	EPHX2	epoxide hydrolase 2, cytoplasmic	0.94474	0.937756	0.9876775
2054	STX2	syntaxin 2	0.97523	0.903653	1.0205927
2055	CLN8	ceroid-lipofuscinosis, neuronal 8 (epilepsy, progressive with mental retardation)	1.01983	0.991606	0.9446126
2056	EPO	erythropoietin	1.00832	0.999754	0.9866978
2057	EPOR	erythropoietin receptor	0.97831	0.997075	0.9615522
2058	EPRS	glutamyl-prolyl-tRNA synthetase	0.97586	0.966715	0.9879388
2059	EPS8	epidermal growth factor receptor pathway substrate 8	0.9955	0.961759	0.993298
2060	EPS15	epidermal growth factor receptor pathway substrate 15	1.01221	0.968756	1.0210685
2063	NR2F6	nuclear receptor subfamily 2, group F, member 6	0.96487	0.961774	1.0299001
2064	ERBB2	v-erb-b2 erythroblastic leukemia viral oncogene homolog 2, neuro/glioblastoma derived oncogene homolog	0.97821	0.971472	1.0237962
2065	ERBB3	v-erb-b2 erythroblastic leukemia viral oncogene homolog 3 (avian)	0.99991	0.987305	0.9949019

2066	ERBB4	v-erb-a erythroblastic leukemia viral oncogene homolog 4 (avian)	1.01148	1.011297	1.0125718
2067	ERCC1	excision repair cross-complementing rodent repair deficiency, complementation group 1 (includes overlappi	0.97397	0.962329	1.0840594
2068	ERCC2	excision repair cross-complementing rodent repair deficiency, complementation group 2	0.95538	0.900634	1.0589063
2069	EREG	epiregulin	0.98015	1.142249	0.8590944
2070	EYA4	eyes absent homolog 4 (Drosophila)	1.00703	1.022786	1.014931
2071	ERCC3	excision repair cross-complementing rodent repair deficiency, complementation group 3 (xeroderma pigmen	0.99619	0.965366	1.0671276
2072	ERCC4	excision repair cross-complementing rodent repair deficiency, complementation group 4	0.92705	0.997792	1.0643841
2073	ERCC5	excision repair cross-complementing rodent repair deficiency, complementation group 5	0.92446	1.019549	0.9337193
2074	ERCC6	excision repair cross-complementing rodent repair deficiency, complementation group 6	0.9525	0.974195	1.0631409
2077	ERF	Ets2 repressor factor	1.00982	1.024805	0.922313
2078	ERG	v-ets erythroblastosis virus E26 oncogene homolog (avian)	1.0079	0.990527	0.9795446
2079	ERH	enhancer of rudimentary homolog (Drosophila)	1.03524	0.920202	1.0053507
2081	ERN1	endoplasmic reticulum to nucleus signaling 1	0.97074	1.012072	0.9566797
2091	FBL	fibrillarin	0.91208	1.000442	0.9901821
2098	ESD	esterase D	0.93677	0.96557	1.0617076
2099	ESR1	estrogen receptor 1	0.99676	1.000093	0.9849283
2100	ESR2	estrogen receptor 2 (ER beta)	1.00027	1.035588	1.0045253
2101	ESRRA	estrogen-related receptor alpha	1.00935	1.016623	1.016874
2103	ESRRB	estrogen-related receptor beta	1.00454	1.005833	0.9976243
2104	ESRRG	estrogen-related receptor gamma	1.00924	1.006721	1.0141062
2107	ETF1	eukaryotic translation termination factor 1	1.03009	1.019409	0.91096
2108	ETFA	electron-transfer-flavoprotein, alpha polypeptide	0.94705	1.042127	0.9883687
2109	ETFB	electron-transfer-flavoprotein, beta polypeptide	0.9906	1.026437	0.9931157
2110	ETFDH	electron-transferring-flavoprotein dehydrogenase	0.97713	0.955453	0.9957975
2113	ETS1	v-ets erythroblastosis virus E26 oncogene homolog 1 (avian)	0.97866	0.966193	0.8938352
2114	ETS2	v-ets erythroblastosis virus E26 oncogene homolog 2 (avian)	0.9912	1.096189	1.0057572
2115	ETV1	ets variant 1	0.99691	1.000093	1.0109551
2116	ETV2	ets variant 2	0.99883	1.006199	1.0464749
2117	ETV3	ets variant 3	0.99739	0.956573	0.9293026
2118	ETV4	ets variant 4	0.99273	0.970504	1.025968
2119	ETV5	ets variant 5	1.01574	0.979582	1.0106106
2120	ETV6	ets variant 6	1.04506	1.055223	0.9671195
2121	EVC	Ellis van Creveld syndrome	1.01235	0.984044	1.0166965

2122	MECOM	MDS1 and EVI1 complex locus	1.0045	1.017435	1.0216879
2123	EVI2A	ecotropic viral integration site 2A	0.91754	0.889081	0.9853471
2124	EVI2B	ecotropic viral integration site 2B	0.9769	1.015011	0.952556
2125	EVPL	envoplakin	1.0064	1.005158	1.0182582
2128	EVX1	even-skipped homeobox 1	1.00179	1.013484	1.0713097
2130	EWSR1	Ewing sarcoma breakpoint region 1	0.95971	0.939793	1.0838993
2131	EXT1	exostosin 1	1.01914	1.026839	0.9384598
2132	EXT2	exostosin 2	0.96193	0.959596	0.9990483
2134	EXTL1	exostoses (multiple)-like 1	0.99774	1.006175	1.0053386
2135	EXTL2	exostoses (multiple)-like 2	1.01275	1.022299	1.008539
2137	EXTL3	exostoses (multiple)-like 3	0.99269	0.955789	1.0320634
2138	EYA1	eyes absent homolog 1 (Drosophila)	0.99592	1.002771	0.9914349
2139	EYA2	eyes absent homolog 2 (Drosophila)	1.00241	0.978285	0.9985321
2140	EYA3	eyes absent homolog 3 (Drosophila)	0.98232	0.96274	1.0093761
2145	EZH1	enhancer of zeste homolog 1 (Drosophila)	0.9247	0.982809	0.9953324
2146	EZH2	enhancer of zeste homolog 2 (Drosophila)	0.99605	1.039306	1.0339065
2147	F2	coagulation factor II (thrombin)	0.99409	1.02449	1.0121895
2149	F2R	coagulation factor II (thrombin) receptor	0.99818	1.039603	0.9483982
2150	F2RL1	coagulation factor II (thrombin) receptor-like 1	0.97918	0.98311	1.0361078
2151	F2RL2	coagulation factor II (thrombin) receptor-like 2	1.01808	1.007018	1.0104975
2152	F3	coagulation factor III (thromboplastin, tissue factor)	0.98796	1.019238	0.9830147
2153	F5	coagulation factor V (proaccelerin, labile factor)	0.99966	0.885801	1.0993741
2155	F7	coagulation factor VII (serum prothrombin conversion accelerator)	0.99624	0.980832	1.021034
2157	F8	coagulation factor VIII, procoagulant component	0.98124	0.989026	1.0255598
2158	F9	coagulation factor IX	0.9898	0.992718	0.9851247
2159	F10	coagulation factor X	0.99129	1.016502	1.0067812
2160	F11	coagulation factor XI	1.01014	1.012539	1.0001884
2161	F12	coagulation factor XII (Hageman factor)	0.99849	1.028944	1.0635266
2162	F13A1	coagulation factor XIII, A1 polypeptide	0.97006	0.955166	1.0301999
2165	F13B	coagulation factor XIII, B polypeptide	0.98781	0.998243	1.0304134
2166	FAAH	fatty acid amide hydrolase	1.00626	1.020544	0.9996419
2167	FABP4	fatty acid binding protein 4, adipocyte	0.99246	0.991447	1.027874
2168	FABP1	fatty acid binding protein 1, liver	0.99849	0.994347	0.9725055

2169	FABP2	fatty acid binding protein 2, intestinal	0.99564	0.992207	1.0480283
2170	FABP3	fatty acid binding protein 3, muscle and heart (mammary-derived growth inhibitor)	1.01711	0.993436	0.9954414
2171	FABP5	fatty acid binding protein 5 (psoriasis-associated)	0.97704	1.004567	0.9872121
2172	FABP6	fatty acid binding protein 6, ileal	0.99927	0.998763	1.0105114
2173	FABP7	fatty acid binding protein 7, brain	1.01982	1.012917	1.0327273
2175	FANCA	Fanconi anemia, complementation group A	0.99477	0.988792	1.0073459
2176	FANCC	Fanconi anemia, complementation group C	0.98877	0.96515	1.0207042
2177	FANCD2	Fanconi anemia, complementation group D2	0.99465	0.970868	1.0201736
2178	FANCE	Fanconi anemia, complementation group E	1.00031	1.017162	0.9953804
2180	ACSL1	acyl-CoA synthetase long-chain family member 1	1.00955	1.128226	1.0283805
2181	ACSL3	acyl-CoA synthetase long-chain family member 3	1.0027	0.935996	0.9195787
2182	ACSL4	acyl-CoA synthetase long-chain family member 4	0.95558	1.076743	1.0199301
2185	PTK2B	PTK2B protein tyrosine kinase 2 beta	1.01517	1.054785	1.0007327
2186	BPTF	bromodomain PHD finger transcription factor	0.94454	0.983134	0.9840776
2187	FANCB	Fanconi anemia, complementation group B	0.95593	1.013328	1.003782
2188	FANCF	Fanconi anemia, complementation group F	0.8825	0.959687	1.1351567
2189	FANCG	Fanconi anemia, complementation group G	0.98498	1.000628	1.0208161
2191	FAP	fibroblast activation protein, alpha	0.99618	1.007393	1.0156099
2192	FBLN1	fibulin 1	0.99238	1.003482	0.9888969
2193	FARSA	phenylalanyl-tRNA synthetase, alpha subunit	0.9488	0.969142	0.9815628
2194	FASN	fatty acid synthase	0.97908	0.994345	1.012017
2195	FAT1	FAT tumor suppressor homolog 1 (Drosophila)	1.00391	0.99852	1.0161116
2196	FAT2	FAT tumor suppressor homolog 2 (Drosophila)	1.01131	1.028253	1.0195503
2197	FAU	Finkel-Biskis-Reilly murine sarcoma virus (FBR-MuSV) ubiquitously expressed	0.98534	1.037226	0.9581045
2199	FBLN2	fibulin 2	1.01386	1.008532	1.0099462
2200	FBN1	fibrillin 1	0.99287	0.986524	1.0038138
2201	FBN2	fibrillin 2	1.02012	0.964248	1.0318361
2202	EFEMP1	EGF containing fibulin-like extracellular matrix protein 1	1.00777	1.018445	0.9956356
2203	FBP1	fructose-1,6-bisphosphatase 1	0.97503	0.963148	0.9861517
2205	FCER1A	Fc fragment of IgE, high affinity I, receptor for; alpha polypeptide	0.94715	0.953624	0.988463
2206	MS4A2	membrane-spanning 4-domains, subfamily A, member 2 (Fc fragment of IgE, high affinity I, receptor for; beta	0.98329	0.981545	0.9998134
2207	FCER1G	Fc fragment of IgE, high affinity I, receptor for; gamma polypeptide	0.9956	1.000045	0.9595427
2212	FCGR2A	Fc fragment of IgG, low affinity IIa, receptor (CD32)	1.01155	0.935093	0.957876

2213	FCGR2B	Fc fragment of IgG, low affinity IIb, receptor (CD32)	1.03011	0.933134	0.9930616
2214	FCGR3A	Fc fragment of IgG, low affinity IIIa, receptor (CD16a)	1.03286	0.959928	0.8956215
2217	FCGRT	Fc fragment of IgG, receptor, transporter, alpha	0.99552	0.993032	1.0014327
2218	FKTN	fukutin	0.96768	0.967393	1.0330915
2219	FCN1	ficolin (collagen/fibrinogen domain containing) 1	1.01529	0.922073	1.060204
2220	FCN2	ficolin (collagen/fibrinogen domain containing lectin) 2 (hucolin)	0.99277	0.9814	1.0191802
2222	FDFT1	farnesyl-diphosphate farnesyltransferase 1	1.00748	1.040862	0.963579
2224	FDPS	farnesyl diphosphate synthase	0.98647	0.962836	1.004241
2228	FDPSP4	farnesyl diphosphate synthase pseudogene 4	0.97347	1.000176	1.0369102
2229	FDPSP5	farnesyl diphosphate synthase pseudogene 5	0.96903	0.964206	1.0389249
2230	FDX1	ferredoxin 1	0.99315	1.003725	0.9760703
2231	FDX1P1	ferredoxin 1 pseudogene 1	1.05302	1.032225	1.0311509
2232	FDXR	ferredoxin reductase	0.99564	1.027856	1.0550719
2235	FECH	ferrochelatase	0.94747	0.939569	1.052252
2236	FECHP1	ferrochelatase pseudogene 1	0.99298	1.029263	0.9613754
2237	FEN1	flap structure-specific endonuclease 1	0.98934	1.00106	1.045858
2239	GPC4	glypican 4	0.98755	1.007549	1.0045586
2241	FER	fer (fps/fes related) tyrosine kinase	0.99838	1.003628	1.0114028
2242	FES	feline sarcoma oncogene	0.95001	0.926118	1.025205
2243	FGA	fibrinogen alpha chain	1.00769	1.020529	1.0107536
2244	FGB	fibrinogen beta chain	1.0033	1.029104	0.9865577
2245	FGD1	FYVE, RhoGEF and PH domain containing 1	0.991	1.009988	0.9943718
2246	FGF1	fibroblast growth factor 1 (acidic)	1.01803	1.018849	1.0039841
2247	FGF2	fibroblast growth factor 2 (basic)	0.98091	0.980325	1.059272
2248	FGF3	fibroblast growth factor 3	0.98126	1.079137	1.0553506
2249	FGF4	fibroblast growth factor 4	0.98524	1.006335	1.0463283
2250	FGF5	fibroblast growth factor 5	0.99684	1.019949	1.023849
2251	FGF6	fibroblast growth factor 6	0.99955	1.016215	0.9869634
2252	FGF7	fibroblast growth factor 7	0.99359	1.020645	1.0075005
2253	FGF8	fibroblast growth factor 8 (androgen-induced)	1.01852	0.966471	1.0069842
2254	FGF9	fibroblast growth factor 9 (glia-activating factor)	0.98817	1.02505	0.9440313
2255	FGF10	fibroblast growth factor 10	1.00474	0.997857	1.0007958
2256	FGF11	fibroblast growth factor 11	0.99926	0.992045	1.019904

2257	FGF12	fibroblast growth factor 12	1.01286	1.005958	1.0178546
2259	FGF14	fibroblast growth factor 14	1.00123	1.01661	1.0040939
2260	FGFR1	fibroblast growth factor receptor 1	1.00009	1.054741	0.9847479
2261	FGFR3	fibroblast growth factor receptor 3	1.00079	1.008405	1.1060626
2262	GPC5	glypican 5	0.99249	1.007931	1.0036401
2263	FGFR2	fibroblast growth factor receptor 2	0.99981	0.991013	1.0122884
2264	FGFR4	fibroblast growth factor receptor 4	0.99668	1.029508	1.0570444
2266	FGG	fibrinogen gamma chain	0.9947	1.005693	1.0226105
2267	FGL1	fibrinogen-like 1	0.99787	1.007876	1.0097425
2268	FGR	Gardner-Rasheed feline sarcoma viral (v-fgr) oncogene homolog	1.04232	1.092823	0.9930831
2271	FH	fumarate hydratase	0.99593	0.984306	0.9884891
2272	FHIT	fragile histidine triad gene	0.99597	0.938317	0.9970883
2273	FHL1	four and a half LIM domains 1	0.97244	0.966125	0.9674324
2274	FHL2	four and a half LIM domains 2	1.01231	0.988263	0.9848864
2275	FHL3	four and a half LIM domains 3	0.98627	1.015198	0.9598014
2277	FIGF	c-fos induced growth factor (vascular endothelial growth factor D)	1.00789	0.993006	0.9931592
2280	FKBP1A	FK506 binding protein 1A, 12kDa	1.00654	0.984952	1.1186101
2281	FKBP1B	FK506 binding protein 1B, 12.6 kDa	1.00408	0.98689	0.9960269
2284	FKBP1AP3	FK506 binding protein 1A, 12kDa pseudogene 3	1.02855	0.973223	0.9899154
2286	FKBP2	FK506 binding protein 2, 13kDa	0.97283	1.030278	1.0565647
2287	FKBP3	FK506 binding protein 3, 25kDa	0.96151	0.928407	1.0355807
2288	FKBP4	FK506 binding protein 4, 59kDa	0.9801	0.985248	0.987636
2289	FKBP5	FK506 binding protein 5	0.99778	0.922742	0.8585999
2290	FOXG1	forkhead box G1	1.00494	1.01517	0.9897634
2294	FOXF1	forkhead box F1	0.9952	0.997809	0.9862895
2295	FOXF2	forkhead box F2	0.98602	1.022085	1.0273631
2296	FOXC1	forkhead box C1	0.99523	1.028227	1.0576291
2297	FOXD1	forkhead box D1	0.99613	1.038393	1.0541456
2299	FOXI1	forkhead box I1	0.99867	0.988973	0.9874597
2300	FOXL1	forkhead box L1	0.9763	1.026516	1.0530816
2301	FOXE3	forkhead box E3	0.99587	1.036246	1.0665024
2302	FOXJ1	forkhead box J1	1.00425	1.006192	1.0313928
2303	FOXC2	forkhead box C2 (MFH-1, mesenchyme forkhead 1)	0.99414	0.976359	1.1188788

2304	FOXE1	forkhead box E1 (thyroid transcription factor 2)	0.98239	0.998436	1.0669156
2305	FOXM1	forkhead box M1	1.00346	0.998967	1.0080429
2306	FOXD2	forkhead box D2	0.99036	1.035157	1.0684966
2307	FOXS1	forkhead box S1	1.02915	0.989039	1.0008507
2308	FOXO1	forkhead box O1	0.99222	0.967151	0.9669702
2309	FOXO3	forkhead box O3	1.02731	1.021998	0.9225415
2311	FOXO1B	forkhead box O1B pseudogene	1.00318	0.9722	1.000541
2312	FLG	filaggrin	0.99454	1.044094	1.0230427
2313	FLI1	Friend leukemia virus integration 1	0.96428	1.035074	0.9237934
2314	FLII	flightless I homolog (Drosophila)	0.95106	0.9843	1.0398615
2315	MLANA	melan-A	1.01786	1.017516	1.0023171
2316	FLNA	filamin A, alpha	1.02425	0.998403	0.9825003
2317	FLNB	filamin B, beta	1.01077	1.03628	0.9732991
2318	FLNC	filamin C, gamma	1.0102	0.992846	1.0152769
2319	FLOT2	flotillin 2	0.95268	0.920287	1.0273724
2321	FLT1	fms-related tyrosine kinase 1 (vascular endothelial growth factor/vascular permeability factor receptor)	0.99375	1.000663	1.0102346
2322	FLT3	fms-related tyrosine kinase 3	0.99895	0.946833	1.0011913
2323	FLT3LG	fms-related tyrosine kinase 3 ligand	0.94169	0.955439	1.0118727
2324	FLT4	fms-related tyrosine kinase 4	0.99899	1.03793	1.0189695
2326	FMO1	flavin containing monooxygenase 1	1.01592	1.007973	1.0258782
2327	FMO2	flavin containing monooxygenase 2 (non-functional)	1.00095	1.000284	1.016618
2329	FMO4	flavin containing monooxygenase 4	1.01049	0.946288	1.0231583
2330	FMO5	flavin containing monooxygenase 5	1.00594	0.978157	1.0055615
2331	FMOD	fibromodulin	1.01771	0.990067	0.9889598
2332	FMR1	fragile X mental retardation 1	0.95431	0.913232	0.9339658
2334	AFF2	AF4/FMR2 family, member 2	0.99499	1.006538	0.9888521
2335	FN1	fibronectin 1	1.01916	0.99477	1.0234641
2339	FNTA	farnesyltransferase, CAAX box, alpha	0.9793	1.000947	0.9542176
2341	FNTAP2	farnesyltransferase, CAAX box, alpha pseudogene 2	0.96614	0.994906	1.038309
2342	FNTB	farnesyltransferase, CAAX box, beta	0.982	1.000085	1.0475074
2346	FOLH1	folate hydrolase (prostate-specific membrane antigen) 1	0.985	1.015484	1.0144319
2348	FOLR1	folate receptor 1 (adult)	1.04524	0.997989	1.0103675
2350	FOLR2	folate receptor 2 (fetal)	0.92603	0.945778	1.0185072

2353	FOS	FBJ murine osteosarcoma viral oncogene homolog	0.9202	0.983492	0.9422603
2354	FOSB	FBJ murine osteosarcoma viral oncogene homolog B	0.98308	0.894679	0.8815041
2356	FPGS	folylpolyglutamate synthase	0.95202	1.011307	0.9908853
2357	FPR1	formyl peptide receptor 1	0.97611	0.91077	0.9928305
2358	FPR2	formyl peptide receptor 2	1.01244	1.162216	1.0060194
2359	FPR3	formyl peptide receptor 3	0.98999	1.063932	0.9667671
2395	FXN	frataxin	0.98529	0.987621	0.9764962
2444	FRK	fyn-related kinase	1.01437	1.008916	1.0078023
2475	MTOR	mechanistic target of rapamycin (serine/threonine kinase)	0.97576	0.903676	0.9751332
2483	FRG1	FSHD region gene 1	0.97311	0.923268	0.9636624
2487	FRZB	frizzled-related protein	1.00323	0.971939	1.017108
2488	FSHB	follicle stimulating hormone, beta polypeptide	1.02688	1.05138	0.9658684
2491	CENPI	centromere protein I	0.97552	0.96901	1.0208076
2492	FSHR	follicle stimulating hormone receptor	1.01916	1.004832	1.0068676
2494	NR5A2	nuclear receptor subfamily 5, group A, member 2	1.00055	1.009297	1.0128349
2495	FTH1	ferritin, heavy polypeptide 1	1.00614	1.145081	0.9395114
2497	FTH1P2	ferritin, heavy polypeptide 1 pseudogene 2	0.99608	0.982086	0.9497812
2499	FTH1P4	ferritin, heavy polypeptide 1 pseudogene 4	0.98639	0.994243	0.9530792
2500	FTH1P7	ferritin, heavy polypeptide 1 pseudogene 7	1.0132	1.067566	0.9610573
2502	FTH1P10	ferritin, heavy polypeptide 1 pseudogene 10	0.98732	0.985871	0.9036293
2503	FTH1P11	ferritin, heavy polypeptide 1 pseudogene 11	1.00511	1.021057	0.9414593
2504	FTH1P12	ferritin, heavy polypeptide 1 pseudogene 12	0.96718	0.959811	0.956833
2509	FTH1P5	ferritin, heavy polypeptide 1 pseudogene 5	0.97973	1.05337	0.9287097
2512	FTL	ferritin, light polypeptide	1	0.977489	1.0391329
2515	ADAM2	ADAM metallopeptidase domain 2	0.99459	1.012068	1.0103958
2516	NR5A1	nuclear receptor subfamily 5, group A, member 1	0.99295	0.986856	1.0332555
2517	FUCA1	fucosidase, alpha-L- 1, tissue	0.97692	1.019081	1.0606431
2518	FUCA1P1	fucosidase, alpha-L- 1, tissue pseudogene 1	1.00126	1.090213	0.9688495
2519	FUCA2	fucosidase, alpha-L- 2, plasma	1.04655	0.916548	1.1447539
2520	GAST	gastrin	1.02407	1.067677	0.9711088
2521	FUS	fused in sarcoma	0.99529	0.911057	1.0029489
2523	FUT1	fucosyltransferase 1 (galactoside 2-alpha-L-fucosyltransferase, H blood group)	0.97108	1.005329	1.0081146
2524	FUT2	fucosyltransferase 2 (secretor status included)	0.99879	1.02322	0.9782302

2525	FUT3	fucosyltransferase 3 (galactoside 3(4)-L-fucosyltransferase, Lewis blood group)	0.9824	1.072268	0.9994494
2526	FUT4	fucosyltransferase 4 (alpha (1,3) fucosyltransferase, myeloid-specific)	0.97303	1.070419	0.959133
2527	FUT5	fucosyltransferase 5 (alpha (1,3) fucosyltransferase)	0.98072	1.028631	0.9848456
2528	FUT6	fucosyltransferase 6 (alpha (1,3) fucosyltransferase)	1.01353	1.00588	0.9943876
2529	FUT7	fucosyltransferase 7 (alpha (1,3) fucosyltransferase)	1.01469	1.041678	0.9594984
2530	FUT8	fucosyltransferase 8 (alpha (1,6) fucosyltransferase)	0.96802	0.939618	0.9943294
2531	KDSR	3-ketodihydrosphingosine reductase	0.93539	0.992473	1.0966883
2532	DARC	Duffy blood group, chemokine receptor	1.01528	1.055993	1.0165988
2533	FYB	FYN binding protein	0.99427	0.937804	0.9479169
2534	FYN	FYN oncogene related to SRC, FGR, YES	1.01102	1.249271	0.947749
2535	FZD2	frizzled family receptor 2	0.96697	0.992371	1.1076201
2537	IFI6	interferon, alpha-inducible protein 6	0.89728	0.885284	1.1036157
2538	G6PC	glucose-6-phosphatase, catalytic subunit	1.01345	0.974657	1.0092117
2539	G6PD	glucose-6-phosphate dehydrogenase	0.98476	0.852152	1.0121414
2542	SLC37A4	solute carrier family 37 (glucose-6-phosphate transporter), member 4	0.96278	0.982256	1.028784
2543	GAGE1	G antigen 1	0.98022	1.04106	1.0180067
2547	XRCC6	X-ray repair complementing defective repair in Chinese hamster cells 6	0.98798	0.986951	0.984268
2548	GAA	glucosidase, alpha; acid	0.96438	0.945909	1.072413
2549	GAB1	GRB2-associated binding protein 1	0.99155	0.982127	1.0511692
2550	GABBR1	gamma-aminobutyric acid (GABA) B receptor, 1	1.01142	0.884677	0.9734266
2551	GABPA	GA binding protein transcription factor, alpha subunit 60kDa	0.99617	0.949348	1.0492989
2553	GABPB1	GA binding protein transcription factor, beta subunit 1	0.95126	1.066565	0.9086283
2554	GABRA1	gamma-aminobutyric acid (GABA) A receptor, alpha 1	1.00448	0.985537	0.9989072
2555	GABRA2	gamma-aminobutyric acid (GABA) A receptor, alpha 2	1.00285	1.007561	0.9975154
2556	GABRA3	gamma-aminobutyric acid (GABA) A receptor, alpha 3	0.99296	1.00154	0.9954506
2557	GABRA4	gamma-aminobutyric acid (GABA) A receptor, alpha 4	1.01055	1.009666	1.0041531
2558	GABRA5	gamma-aminobutyric acid (GABA) A receptor, alpha 5	0.99516	0.990654	1.0403973
2559	GABRA6	gamma-aminobutyric acid (GABA) A receptor, alpha 6	0.99526	0.978644	1.0123717
2560	GABRB1	gamma-aminobutyric acid (GABA) A receptor, beta 1	1.00851	1.024112	1.032414
2561	GABRB2	gamma-aminobutyric acid (GABA) A receptor, beta 2	1.00347	1.014485	1.0153559
2562	GABRB3	gamma-aminobutyric acid (GABA) A receptor, beta 3	1.00954	0.98965	0.985138
2563	GABRD	gamma-aminobutyric acid (GABA) A receptor, delta	1.001	0.970224	1.0225102
2564	GABRE	gamma-aminobutyric acid (GABA) A receptor, epsilon	0.99865	0.994916	0.9824669

2565	GABRG1	gamma-aminobutyric acid (GABA) A receptor, gamma 1	1.02894	1.005583	0.9908541
2566	GABRG2	gamma-aminobutyric acid (GABA) A receptor, gamma 2	1.00369	1.015834	1.0028313
2567	GABRG3	gamma-aminobutyric acid (GABA) A receptor, gamma 3	0.99115	1.000472	0.9948295
2569	GABRR1	gamma-aminobutyric acid (GABA) receptor, rho 1	1.01391	0.989862	1.0219419
2570	GABRR2	gamma-aminobutyric acid (GABA) receptor, rho 2	1.00604	1.007563	1.0185211
2571	GAD1	glutamate decarboxylase 1 (brain, 67kDa)	0.99972	1.017332	1.0113506
2572	GAD2	glutamate decarboxylase 2 (pancreatic islets and brain, 65kDa)	1.00081	1.00481	1.0200984
2580	GAK	cyclin G associated kinase	1.00915	0.970774	0.9494828
2581	GALC	galactosylceramidase	0.97487	1.016319	1.0087727
2582	GALE	UDP-galactose-4-epimerase	1.00081	0.990873	1.0017094
2583	B4GALNT1	beta-1,4-N-acetyl-galactosaminyl transferase 1	0.99615	0.982233	0.9893759
2584	GALK1	galactokinase 1	0.95925	0.97066	1.0438081
2585	GALK2	galactokinase 2	0.94128	0.974366	1.0184738
2587	GALR1	galanin receptor 1	0.99488	0.979956	1.0292003
2588	GALNS	galactosamine (N-acetyl)-6-sulfate sulfatase	0.97917	0.972783	0.9926019
2589	GALNT1	UDP-N-acetyl-alpha-D-galactosamine:polypeptide N-acetylgalactosaminyltransferase 1 (GalNAc-T1)	0.94822	0.938038	1.0681937
2590	GALNT2	UDP-N-acetyl-alpha-D-galactosamine:polypeptide N-acetylgalactosaminyltransferase 2 (GalNAc-T2)	1.02134	1.017772	1.0265398
2591	GALNT3	UDP-N-acetyl-alpha-D-galactosamine:polypeptide N-acetylgalactosaminyltransferase 3 (GalNAc-T3)	1.01789	1.021263	0.9507346
2592	GALT	galactose-1-phosphate uridylyltransferase	0.95373	0.907728	0.9655328
2593	GAMT	guanidinoacetate N-methyltransferase	0.95102	1.031886	1.0874712
2595	GANC	glucosidase, alpha; neutral C	0.9848	0.984098	1.040629
2596	GAP43	growth associated protein 43	1.0162	0.981122	1.0056617
2597	GAPDH	glyceraldehyde-3-phosphate dehydrogenase	0.96167	1.001415	1.0694156
2601	GAPDHP60	glyceraldehyde-3-phosphate dehydrogenase pseudogene 60	1.00919	0.941376	0.9918012
2609	GAPDHP67	glyceraldehyde 3 phosphate dehydrogenase pseudogene 67	1.00378	1.041633	1.0205572
2613	GAPDHP71	glyceraldehyde-3-phosphate dehydrogenase pseudogene 71	0.98892	1.001106	1.0136986
2615	LRRC32	leucine rich repeat containing 32	0.9668	0.987897	0.9136393
2616	GAPDHP1	glyceraldehyde-3-phosphate dehydrogenase pseudogene 1	0.98937	0.902198	1.0339655
2617	GARS	glycyl-tRNA synthetase	0.98107	0.909929	1.0667283
2618	GART	phosphoribosylglycinamide formyltransferase, phosphoribosylglycinamide synthetase, phosphoribosylamino	0.93692	0.983584	1.0160065
2619	GAS1	growth arrest-specific 1	0.99469	1.013554	1.0826429
2620	GAS2	growth arrest-specific 2	0.99499	1.022903	1.0016262
2621	GAS6	growth arrest-specific 6	1.00328	0.993527	1.0315482

2622	GAS8	growth arrest-specific 8	0.99079	0.976397	1.0221738
2623	GATA1	GATA binding protein 1 (globin transcription factor 1)	0.96322	0.996107	0.9800512
2624	GATA2	GATA binding protein 2	0.98861	0.967991	0.9718156
2625	GATA3	GATA binding protein 3	0.97653	1.006764	0.9779015
2626	GATA4	GATA binding protein 4	0.99326	1.012604	1.0335263
2627	GATA6	GATA binding protein 6	1.0099	1.030086	0.9719046
2628	GATM	glycine amidinotransferase (L-arginine:glycine amidinotransferase)	0.93732	1.003795	0.9783293
2629	GBA	glucosidase, beta, acid	0.99592	0.945113	1.1096384
2630	GBAP1	glucosidase, beta, acid pseudogene 1	1.01898	0.998335	1.0338168
2631	GBAS	glioblastoma amplified sequence	1.00936	0.898695	0.9872724
2632	GBE1	glucan (1,4-alpha-), branching enzyme 1	1.01577	1.003372	0.9886906
2633	GBP1	guanylate binding protein 1, interferon-inducible	0.98367	0.974504	0.98084
2634	GBP2	guanylate binding protein 2, interferon-inducible	1.01311	1.037849	0.9297524
2635	GBP3	guanylate binding protein 3	0.98335	0.988704	0.9502666
2636	GBX1	gastrulation brain homeobox 1	0.99596	1.02448	1.0170885
2637	GBX2	gastrulation brain homeobox 2	1.01854	1.018359	1.0465526
2638	GC	group-specific component (vitamin D binding protein)	0.99439	1.015443	0.996545
2639	GCDH	glutaryl-CoA dehydrogenase	0.96398	0.965212	1.0116345
2641	GCG	glucagon	1.0016	1.011928	1.0016814
2643	GCH1	GTP cyclohydrolase 1	0.99129	1.031602	1.0381419
2644	GCHFR	GTP cyclohydrolase I feedback regulator	0.99841	1.016467	1.0201063
2645	GCK	glucokinase (hexokinase 4)	1.01977	1.014195	1.0137807
2646	GCKR	glucokinase (hexokinase 4) regulator	1.01205	0.982491	1.0013963
2647	BLOC1S1	biogenesis of lysosomal organelles complex-1, subunit 1	0.92283	0.967642	1.1312917
2648	KAT2A	K(lysine) acetyltransferase 2A	0.94779	0.982295	1.0371428
2649	NR6A1	nuclear receptor subfamily 6, group A, member 1	1.0135	1.001838	1.0060785
2650	GCNT1	glucosaminyl (N-acetyl) transferase 1, core 2	0.98337	0.969544	1.0064566
2651	GCNT2	glucosaminyl (N-acetyl) transferase 2, I-branching enzyme (I blood group)	0.98944	0.940683	1.0345192
2653	GCSH	glycine cleavage system protein H (aminomethyl carrier)	1.01568	1.01418	1.0056605
2658	GDF2	growth differentiation factor 2	1.01328	0.976746	1.0151229
2660	MSTN	myostatin	1.01271	0.980239	1.0557515
2661	GDF9	growth differentiation factor 9	1.01224	1.001371	0.9901648
2662	GDF10	growth differentiation factor 10	1.00792	0.96202	1.0280318

2664	GDI1	GDP dissociation inhibitor 1	0.9657	1.004821	0.989111
2665	GDI2	GDP dissociation inhibitor 2	1.01191	1.038668	1.0203254
2667	GDI2P1	GDP dissociation inhibitor 2 pseudogene 1	0.99526	0.99104	1.0223478
2668	GDNF	glial cell derived neurotrophic factor	1.00082	0.974148	0.9985384
2669	GEM	GTP binding protein overexpressed in skeletal muscle	0.99096	1.013049	0.9910764
2670	GFAP	glial fibrillary acidic protein	0.99442	0.986929	1.022069
2671	GFER	growth factor, augmenter of liver regeneration	0.9645	1.03629	1.0892181
2672	GFI1	growth factor independent 1 transcription repressor	1.00239	1.075709	1.0095129
2673	GFPT1	glutamine--fructose-6-phosphate transaminase 1	1.02182	1.068984	1.0174324
2674	GFRA1	GDNF family receptor alpha 1	1.00028	0.989748	1.0023246
2675	GFRA2	GDNF family receptor alpha 2	1.0024	0.982475	1.0177007
2676	GFRA3	GDNF family receptor alpha 3	1.00459	0.987544	1.0314107
2677	GGCX	gamma-glutamyl carboxylase	1.02289	1.023076	1.0528256
2678	GGT1	gamma-glutamyltransferase 1	0.99648	1.019036	1.0262623
2683	B4GALT1	UDP-Gal:betaGlcNAc beta 1,4- galactosyltransferase, polypeptide 1	1.01682	1.078105	0.9798771
2686	GGT7	gamma-glutamyltransferase 7	0.95029	0.967543	0.9267996
2687	GGT5	gamma-glutamyltransferase 5	0.98431	1.008936	1.0259462
2689	GH2	growth hormone 2	0.98504	1.016411	0.9364312
2690	GHR	growth hormone receptor	1.00987	1	1.0126559
2691	GHRH	growth hormone releasing hormone	1.00314	0.972588	1.0127139
2692	GHRHR	growth hormone releasing hormone receptor	1.01036	0.983096	1.0065536
2693	GHSR	growth hormone secretagogue receptor	0.98692	1.049184	0.9836169
2694	GIF	gastric intrinsic factor (vitamin B synthesis)	1.00062	1.021778	1.0012414
2695	GIP	gastric inhibitory polypeptide	1.00769	1.007988	1.005285
2696	GIPR	gastric inhibitory polypeptide receptor	0.9847	1.02721	0.9967572
2697	GJA1	gap junction protein, alpha 1, 43kDa	1.01453	0.991449	1.0498713
2698	GJA1P1	gap junction protein, alpha 1, 43kDa pseudogene 1	0.97516	1.032142	0.9557087
2700	GJA3	gap junction protein, alpha 3, 46kDa	1.00916	0.991929	0.9959732
2701	GJA4	gap junction protein, alpha 4, 37kDa	0.99196	1.029115	0.9889184
2702	GJA5	gap junction protein, alpha 5, 40kDa	1.01501	1.007388	1.004522
2703	GJA8	gap junction protein, alpha 8, 50kDa	1.0096	0.995483	1.0225153
2705	GJB1	gap junction protein, beta 1, 32kDa	0.99114	0.966647	0.9896953
2706	GJB2	gap junction protein, beta 2, 26kDa	0.99896	1.020522	0.9992012

2707	GJB3	gap junction protein, beta 3, 31kDa	0.99237	0.998727	1.0156488
2709	GJB5	gap junction protein, beta 5, 31.1kDa	0.95243	0.963272	1.0366308
2710	GK	glycerol kinase	0.98396	1.076929	0.9202291
2712	GK2	glycerol kinase 2	1.02671	0.972817	1.0177589
2717	GLA	galactosidase, alpha	0.91601	0.911464	1.0623518
2719	GPC3	glypican 3	1.00386	1.009225	1.000844
2720	GLB1	galactosidase, beta 1	1.02679	1.014565	0.9894659
2729	GCLC	glutamate-cysteine ligase, catalytic subunit	1.02144	0.921894	1.035635
2730	GCLM	glutamate-cysteine ligase, modifier subunit	0.9902	0.973495	1.0507125
2731	GLDC	glycine dehydrogenase (decarboxylating)	1.01009	0.983756	1.0037564
2732	GLDCP1	glycine dehydrogenase (decarboxylase) pseudogene 1	1.00899	1.006978	1.0343066
2733	GLE1	GLE1 RNA export mediator homolog (yeast)	0.9107	0.949138	1.1110683
2734	GLG1	golgi glycoprotein 1	0.95209	0.928821	1.007137
2735	GLI1	GLI family zinc finger 1	1.00928	0.98871	1.0198375
2736	GLI2	GLI family zinc finger 2	0.99911	1.014024	1.0153146
2737	GLI3	GLI family zinc finger 3	0.99499	0.997454	1.0136083
2738	GLI4	GLI family zinc finger 4	1.00084	0.970141	0.9901605
2739	GLO1	glyoxalase I	0.99797	0.907176	1.0617716
2740	GLP1R	glucagon-like peptide 1 receptor	1.01393	0.991644	1.0244946
2741	GLRA1	glycine receptor, alpha 1	1.01511	1.003394	1.0174807
2742	GLRA2	glycine receptor, alpha 2	0.99748	0.998706	0.992754
2743	GLRB	glycine receptor, beta	1.00084	0.998652	1.028752
2744	GLS	glutaminase	0.98243	1.042621	0.9347186
2745	GLRX	glutaredoxin (thioltransferase)	1.0251	0.927545	1.0934733
2746	GLUD1	glutamate dehydrogenase 1	0.99011	0.94946	1.0117948
2747	GLUD2	glutamate dehydrogenase 2	1.00868	0.992591	1.0149278
2749	GLUD1P3	glutamate dehydrogenase 1 pseudogene 3	0.98106	0.9774	1.0353512
2750	GLUD1P4	glutamate dehydrogenase 1 pseudogene 4	0.9665	1.001638	0.9861992
2752	GLUL	glutamate-ammonia ligase	1.03188	0.990915	0.9411008
2753	GLULP1	glutamate-ammonia ligase (glutamine synthetase) pseudogene 1	1.02515	0.965051	0.9912967
2760	GM2A	GM2 ganglioside activator	0.96858	0.952517	1.0179641
2761	GM2AP1	GM2 ganglioside activator pseudogene 1	0.99815	1.055949	1.0469699
2762	GMDS	GDP-mannose 4,6-dehydratase	1.00199	0.987604	1.0220292

2764	GMFB	glia maturation factor, beta	0.98155	0.947318	0.9729384
2765	GML	glycosylphosphatidylinositol anchored molecule like protein	0.99806	1.014948	1.0483892
2766	GMPR	guanosine monophosphate reductase	0.97296	0.981706	1.0774935
2767	GNA11	guanine nucleotide binding protein (G protein), alpha 11 (Gq class)	0.99315	1.039462	1.0485859
2768	GNA12	guanine nucleotide binding protein (G protein) alpha 12	1.0054	0.99677	0.9686265
2769	GNA15	guanine nucleotide binding protein (G protein), alpha 15 (Gq class)	0.89365	1.030663	0.9944596
2770	GNAI1	guanine nucleotide binding protein (G protein), alpha inhibiting activity polypeptide 1	0.99513	0.980159	1.0492604
2771	GNAI2	guanine nucleotide binding protein (G protein), alpha inhibiting activity polypeptide 2	1.00176	1.021494	0.9554633
2773	GNAI3	guanine nucleotide binding protein (G protein), alpha inhibiting activity polypeptide 3	0.99686	0.955035	1.1186209
2775	GNAO1	guanine nucleotide binding protein (G protein), alpha activating activity polypeptide O	1.00778	1.018017	0.9733339
2776	GNAQ	guanine nucleotide binding protein (G protein), q polypeptide	1.00762	0.937134	0.9600739
2778	GNAS	GNAS complex locus	0.98086	0.967133	0.9665776
2779	GNAT1	guanine nucleotide binding protein (G protein), alpha transducing activity polypeptide 1	1.01148	1.009989	1.0107787
2780	GNAT2	guanine nucleotide binding protein (G protein), alpha transducing activity polypeptide 2	1.00126	1.028521	0.9861963
2781	GNAZ	guanine nucleotide binding protein (G protein), alpha z polypeptide	0.95211	0.971755	0.9648555
2782	GNB1	guanine nucleotide binding protein (G protein), beta polypeptide 1	1.02511	1.061935	0.9653388
2783	GNB2	guanine nucleotide binding protein (G protein), beta polypeptide 2	0.99209	0.977991	1.0455796
2784	GNB3	guanine nucleotide binding protein (G protein), beta polypeptide 3	0.98338	0.999455	1.0122809
2785	GNG3	guanine nucleotide binding protein (G protein), gamma 3	1.01003	0.989275	1.0242039
2786	GNG4	guanine nucleotide binding protein (G protein), gamma 4	1.00293	0.990473	1.0027645
2787	GNG5	guanine nucleotide binding protein (G protein), gamma 5	1.05034	0.932127	0.8912357
2789	GNG5P1	guanine nucleotide binding protein (G protein), gamma 5 pseudogene 1	1.0235	1.061594	0.9980016
2791	GNG11	guanine nucleotide binding protein (G protein), gamma 11	0.9614	0.821431	0.9262435
2792	GNGT1	guanine nucleotide binding protein (G protein), gamma transducing activity polypeptide 1	1.00996	0.992642	1.0038083
2793	GNGT2	guanine nucleotide binding protein (G protein), gamma transducing activity polypeptide 2	0.9536	0.98823	1.026384
2794	GNL1	guanine nucleotide binding protein-like 1	0.97318	0.988533	0.9095689
2796	GNRH1	gonadotropin-releasing hormone 1 (luteinizing-releasing hormone)	0.97562	1.029298	1.0416127
2797	GNRH2	gonadotropin-releasing hormone 2	0.99915	1.054675	1.0435408
2798	GNRHR	gonadotropin-releasing hormone receptor	1.01694	1.016562	1.0162535
2799	GNS	glucosamine (N-acetyl)-6-sulfatase	1.00367	0.995925	0.9961919
2800	GOLGA1	golgin A1	0.95851	0.985057	1.033714
2801	GOLGA2	golgin A2	0.9021	0.982853	1.0143897
2802	GOLGA3	golgin A3	0.95375	0.956774	0.9909533

2803	GOLGA4	golgin A4	0.98026	1.009174	0.9592586
2804	GOLGB1	golgin B1	0.99171	1.060803	1.0147267
2805	GOT1	glutamic-oxaloacetic transaminase 1, soluble (aspartate aminotransferase 1)	0.93774	1.015888	0.9972515
2806	GOT2	glutamic-oxaloacetic transaminase 2, mitochondrial (aspartate aminotransferase 2)	0.96274	1.052456	1.0195775
2810	SFN	stratifin	1.00509	0.979451	1.0610424
2811	GP1BA	glycoprotein Ib (platelet), alpha polypeptide	0.853	0.961543	0.959108
2813	GP2	glycoprotein 2 (zymogen granule membrane)	0.99283	0.993817	0.9827743
2814	GP5	glycoprotein V (platelet)	0.94428	0.942888	0.9714308
2815	GP9	glycoprotein IX (platelet)	0.94795	0.93441	0.9325416
2817	GPC1	glypican 1	0.98903	1.013316	1.0249871
2819	GPD1	glycerol-3-phosphate dehydrogenase 1 (soluble)	0.99956	0.988623	0.9942148
2820	GPD2	glycerol-3-phosphate dehydrogenase 2 (mitochondrial)	0.99036	0.937692	1.0032167
2821	GPI	glucose-6-phosphate isomerase	0.97665	0.985508	1.0454287
2822	GPLD1	glycosylphosphatidylinositol specific phospholipase D1	1.01122	0.982476	1.0320641
2823	GPM6A	glycoprotein M6A	0.99261	1.010953	1.0153196
2824	GPM6B	glycoprotein M6B	0.99983	1.005735	1.0027426
2825	GPR1	G protein-coupled receptor 1	0.99626	0.988918	1.0059737
2826	CCR10	chemokine (C-C motif) receptor 10	1.01663	0.984889	1.0664558
2827	GPR3	G protein-coupled receptor 3	1.00916	0.926865	0.9990887
2828	GPR4	G protein-coupled receptor 4	1.00255	1.014275	1.0298752
2829	XCR1	chemokine (C motif) receptor 1	0.99686	1.063596	1.0014101
2830	GPR6	G protein-coupled receptor 6	1.00286	1.014695	0.9815901
2832	NPBWR2	neuropeptides B/W receptor 2	0.97875	0.975752	1.0243696
2833	CXCR3	chemokine (C-X-C motif) receptor 3	0.9519	1.067695	1.0021634
2834	PRLHR	prolactin releasing hormone receptor	1.00312	0.955107	1.063203
2835	GPR12	G protein-coupled receptor 12	1.01621	0.982483	1.0064366
2837	UTS2R	urotensin 2 receptor	1.02969	0.993693	1.0903458
2838	GPR15	G protein-coupled receptor 15	0.93374	0.964051	1.1667299
2840	GPR17	G protein-coupled receptor 17	1.0025	0.992014	0.9968441
2841	GPR18	G protein-coupled receptor 18	0.85849	0.984129	0.9498232
2842	GPR19	G protein-coupled receptor 19	0.95757	1.027345	1.0555723
2843	GPR20	G protein-coupled receptor 20	0.99433	0.988714	1.0450675
2845	GPR22	G protein-coupled receptor 22	0.99617	0.991021	0.9905062

2846	LPAR4	lysophosphatidic acid receptor 4	0.93795	1.026336	0.9814688
2847	MCHR1	melanin-concentrating hormone receptor 1	1.02249	0.931707	1.0815417
2848	GPR25	G protein-coupled receptor 25	0.97699	1.04628	1.1017583
2849	GPR26	G protein-coupled receptor 26	1.01064	0.996142	0.9925678
2850	GPR27	G protein-coupled receptor 27	0.98457	0.900614	0.9978848
2852	GPER	G protein-coupled estrogen receptor 1	1.0026	1.020356	1.0085663
2853	GPR31	G protein-coupled receptor 31	0.98774	0.970648	0.982965
2854	GPR32	G protein-coupled receptor 32	1.01154	1.036703	1.0519634
2855	GPR32P1	G protein-coupled receptor 32, pseudogene 1	1.03232	0.906561	0.9804294
2856	GPR33	G protein-coupled receptor 33 (gene/pseudogene)	0.97256	0.973057	0.9667816
2857	GPR34	G protein-coupled receptor 34	1.0023	0.940193	0.9860245
2859	GPR35	G protein-coupled receptor 35	0.97786	1.007313	0.9333234
2861	GPR37	G protein-coupled receptor 37 (endothelin receptor type B-like)	1.00143	0.994421	1.0169891
2862	MLNR	motilin receptor	1.01263	0.992188	1.0465314
2863	GPR39	G protein-coupled receptor 39	1.00458	0.977396	1.0144646
2864	FFAR1	free fatty acid receptor 1	0.99412	1.087629	0.9554817
2867	FFAR2	free fatty acid receptor 2	0.98973	0.973534	0.9940295
2868	GRK4	G protein-coupled receptor kinase 4	1.00359	0.978074	0.989274
2869	GRK5	G protein-coupled receptor kinase 5	0.99537	1.07284	0.9728252
2870	GRK6	G protein-coupled receptor kinase 6	1.0289	0.981977	1.0197911
2872	MKNK2	MAP kinase interacting serine/threonine kinase 2	0.98756	1.067078	1.0514359
2873	GPS1	G protein pathway suppressor 1	0.98106	0.888184	1.08548
2874	GPS2	G protein pathway suppressor 2	0.92558	1.009222	1.0061225
2875	GPT	glutamic-pyruvate transaminase (alanine aminotransferase)	0.97979	0.996259	1.0629358
2876	GPX1	glutathione peroxidase 1	1.04443	0.923425	0.8932349
2877	GPX2	glutathione peroxidase 2 (gastrointestinal)	0.98779	0.997581	0.9920266
2878	GPX3	glutathione peroxidase 3 (plasma)	1	1.025143	0.9885407
2879	GPX4	glutathione peroxidase 4 (phospholipid hydroperoxidase)	0.89366	1.034955	1.0820578
2880	GPX5	glutathione peroxidase 5 (epididymal androgen-related protein)	1.0119	1.009227	1.0040888
2882	GPX7	glutathione peroxidase 7	0.9327	0.948218	0.9358385
2884	GPX1P2	glutathione peroxidase pseudogene 2	0.96692	1.086379	0.9935085
2885	GRB2	growth factor receptor-bound protein 2	0.97854	0.961453	1.0197532
2886	GRB7	growth factor receptor-bound protein 7	0.99751	0.985226	1.02181

2887	GRB10	growth factor receptor-bound protein 10	1.00839	0.991443	1.0060337
2888	GRB14	growth factor receptor-bound protein 14	0.99641	0.976824	1.0046166
2889	RAPGEF1	Rap guanine nucleotide exchange factor (GEF) 1	0.9679	1.077771	0.904922
2890	GRIA1	glutamate receptor, ionotropic, AMPA 1	1.00907	1.014491	1.023267
2891	GRIA2	glutamate receptor, ionotropic, AMPA 2	1.00999	0.994219	1.0108624
2892	GRIA3	glutamate receptor, ionotrophic, AMPA 3	0.99425	0.990772	1.0029507
2894	GRID1	glutamate receptor, ionotropic, delta 1	1.00119	0.998418	0.9845178
2895	GRID2	glutamate receptor, ionotropic, delta 2	1.01185	1.013341	1.0203414
2896	GRN	granulin	0.9945	0.946128	1.0349813
2897	GRIK1	glutamate receptor, ionotropic, kainate 1	1.00838	1.020187	1.019011
2898	GRIK2	glutamate receptor, ionotropic, kainate 2	1.00481	0.997938	1.003464
2899	GRIK3	glutamate receptor, ionotropic, kainate 3	1.00481	1.007479	1.0043009
2900	GRIK4	glutamate receptor, ionotropic, kainate 4	0.99919	1.023359	1.0049724
2901	GRIK5	glutamate receptor, ionotropic, kainate 5	0.98608	0.97217	1.0059238
2902	GRIN1	glutamate receptor, ionotropic, N-methyl D-aspartate 1	0.99542	0.986968	1.0340879
2903	GRIN2A	glutamate receptor, ionotropic, N-methyl D-aspartate 2A	1.00464	1.001544	1.0105927
2904	GRIN2B	glutamate receptor, ionotropic, N-methyl D-aspartate 2B	1.00803	1.000651	0.9986565
2905	GRIN2C	glutamate receptor, ionotropic, N-methyl D-aspartate 2C	1.00496	1.012548	0.9824746
2906	GRIN2D	glutamate receptor, ionotropic, N-methyl D-aspartate 2D	0.98689	1.007961	1.101189
2907	GRINA	glutamate receptor, ionotropic, N-methyl D-aspartate-associated protein 1 (glutamate binding)	1.00686	1.028212	0.9957541
2908	NR3C1	nuclear receptor subfamily 3, group C, member 1 (glucocorticoid receptor)	1.00157	0.990036	1.0444369
2909	ARHGAP35	Rho GTPase activating protein 35	0.9604	1.01581	0.962279
2911	GRM1	glutamate receptor, metabotropic 1	1.00641	1.000567	1.0026287
2912	GRM2	glutamate receptor, metabotropic 2	1.00964	0.988077	1.0231885
2913	GRM3	glutamate receptor, metabotropic 3	1.0047	0.99272	1.0042645
2914	GRM4	glutamate receptor, metabotropic 4	0.99429	1.010879	1.0282771
2915	GRM5	glutamate receptor, metabotropic 5	1.00176	1.003544	1.0133144
2916	GRM6	glutamate receptor, metabotropic 6	1.0156	1.041415	1.0267473
2917	GRM7	glutamate receptor, metabotropic 7	1.01389	0.98947	1.016414
2918	GRM8	glutamate receptor, metabotropic 8	1.01545	1.008552	1.0145052
2919	CXCL1	chemokine (C-X-C motif) ligand 1 (melanoma growth stimulating activity, alpha)	0.99488	1.010188	0.9448825
2920	CXCL2	chemokine (C-X-C motif) ligand 2	1.01529	1.334247	0.7550895
2921	CXCL3	chemokine (C-X-C motif) ligand 3	0.97149	1.044989	0.9773705

2922	GRP	gastrin-releasing peptide	1.0049	1.014373	1.0213526
2923	PDIA3	protein disulfide isomerase family A, member 3	0.9747	1.025256	0.9533168
2925	GRPR	gastrin-releasing peptide receptor	0.99966	1.004034	1.0068599
2926	GRSF1	G-rich RNA sequence binding factor 1	1.03794	0.948861	1.1305817
2928	GSC2	goosecoid homeobox 2	1.00097	0.901272	1.0742044
2931	GSK3A	glycogen synthase kinase 3 alpha	0.95784	1.034094	1.0489412
2932	GSK3B	glycogen synthase kinase 3 beta	1.01361	0.981404	1.0342458
2934	GSN	gelsolin	0.99336	0.966008	1.0202633
2935	GSPT1	G1 to S phase transition 1	0.9523	1.003863	0.966984
2936	GSR	glutathione reductase	0.95437	0.939367	1.1029365
2937	GSS	glutathione synthetase	0.94981	0.960212	1.0263801
2939	GSTA2	glutathione S-transferase alpha 2	1.0302	0.988164	0.9263529
2940	GSTA3	glutathione S-transferase alpha 3	1.02715	0.981156	0.9523205
2941	GSTA4	glutathione S-transferase alpha 4	1.01335	0.989087	1.0168655
2944	GSTM1	glutathione S-transferase mu 1	1.02973	1.027158	1.0910464
2945	GSTM5P1	glutathione S-transferase mu 5 pseudogene 1	0.95595	1.026332	1.0580311
2946	GSTM2	glutathione S-transferase mu 2 (muscle)	1.00224	0.988447	0.9907353
2947	GSTM3	glutathione S-transferase mu 3 (brain)	0.92497	0.957035	0.9715739
2948	GSTM4	glutathione S-transferase mu 4	1.00581	0.998969	1.0358087
2949	GSTM5	glutathione S-transferase mu 5	1.00666	0.980136	1.0181738
2950	GSTP1	glutathione S-transferase pi 1	0.98901	0.985154	1.0511523
2951	GSTP1P1	glutathione S-transferase pi 1 pseudogene 1	1.00261	0.98408	1.053272
2952	GSTT1	glutathione S-transferase theta 1	0.96991	1.045821	1.0461649
2954	GSTZ1	glutathione transferase zeta 1	0.96614	1.015287	1.0327289
2956	MSH6	mutS homolog 6 (E. coli)	1.01228	0.988678	1.0522146
2957	GTF2A1	general transcription factor IIA, 1, 19/37kDa	0.9228	1.013097	0.9820298
2958	GTF2A2	general transcription factor IIA, 2, 12kDa	0.96873	0.904458	1.0219667
2959	GTF2B	general transcription factor IIB	0.98193	0.919722	0.9472554
2960	GTF2E1	general transcription factor IIE, polypeptide 1, alpha 56kDa	0.98815	0.986134	1.0521799
2961	GTF2E2	general transcription factor IIE, polypeptide 2, beta 34kDa	0.95178	0.936187	1.0281597
2962	GTF2F1	general transcription factor IIF, polypeptide 1, 74kDa	0.91337	0.932868	1.1116075
2963	GTF2F2	general transcription factor IIF, polypeptide 2, 30kDa	0.97839	0.980338	1.011654
2965	GTF2H1	general transcription factor IIH, polypeptide 1, 62kDa	0.95528	1.013307	1.0509751

2967	GTF2H3	general transcription factor IIH, polypeptide 3, 34kDa	0.91863	0.887795	1.0002295
2968	GTF2H4	general transcription factor IIH, polypeptide 4, 52kDa	0.9762	0.966708	1.0525253
2969	GTF2I	general transcription factor IIi	0.96801	0.970006	1.0395433
2971	GTF3A	general transcription factor IIIA	0.91607	0.946213	0.9968833
2972	BRF1	BRF1 homolog, subunit of RNA polymerase III transcription initiation factor IIIB (S. cerevisiae)	0.97432	0.972863	0.958275
2974	GUCY1B2	guanylate cyclase 1, soluble, beta 2	0.99244	1.01026	1.0062291
2975	GTF3C1	general transcription factor IIIC, polypeptide 1, alpha 220kDa	0.94031	1.044961	1.0596104
2976	GTF3C2	general transcription factor IIIC, polypeptide 2, beta 110kDa	0.9904	0.999476	1.0537507
2977	GUCY1A2	guanylate cyclase 1, soluble, alpha 2	1.00584	0.98276	0.985775
2978	GUCA1A	guanylate cyclase activator 1A (retina)	0.99591	0.969826	0.997029
2979	GUCA1B	guanylate cyclase activator 1B (retina)	1.02114	0.964087	0.9961954
2980	GUCA2A	guanylate cyclase activator 2A (guanylin)	1.00396	0.976537	0.9970324
2981	GUCA2B	guanylate cyclase activator 2B (uroguanylin)	1.00743	0.979921	1.035491
2982	GUCY1A3	guanylate cyclase 1, soluble, alpha 3	0.98746	0.951841	1.019012
2983	GUCY1B3	guanylate cyclase 1, soluble, beta 3	0.96491	0.928918	1.0079258
2984	GUCY2C	guanylate cyclase 2C (heat stable enterotoxin receptor)	1.0032	1.00501	1.0095418
2986	GUCY2F	guanylate cyclase 2F, retinal	0.98971	1.009106	1.0013906
2987	GUK1	guanylate kinase 1	1.01861	1.032079	0.9334797
2989	GULOP	gulonolactone (L-) oxidase, pseudogene	0.98624	1.019275	1.0132453
2990	GUSB	glucuronidase, beta	1.01945	0.973172	0.9297933
2992	GYG1	glycogenin 1	1.04664	0.956548	1.1003224
2993	GYPA	glycophorin A (MNS blood group)	1.01043	0.965314	1.0007896
2994	GYPB	glycophorin B (MNS blood group)	0.95074	0.983135	0.9857478
2995	GYPC	glycophorin C (Gerbich blood group)	0.9486	1.039095	0.9771895
2996	GYPE	glycophorin E (MNS blood group)	0.98449	1.03962	1.0627332
2997	GYS1	glycogen synthase 1 (muscle)	0.96659	0.934275	1.0148886
2999	GZMH	granzyme H (cathepsin G-like 2, protein h-CCPX)	0.95735	1.012407	0.9690347
3000	GUCY2D	guanylate cyclase 2D, membrane (retina-specific)	1.00398	0.988456	1.0276617
3001	GZMA	granzyme A (granzyme 1, cytotoxic T-lymphocyte-associated serine esterase 3)	1.00242	0.99039	0.8875951
3003	GZMK	granzyme K (granzyme 3; tryptase II)	0.97652	0.971341	0.9412745
3004	GZMM	granzyme M (lymphocyte met-ase 1)	0.94255	1.04778	0.921518
3006	HIST1H1C	histone cluster 1, H1c	0.9717	0.932561	1.0230809
3007	HIST1H1D	histone cluster 1, H1d	0.92888	0.945644	0.985232

3008	HIST1H1E	histone cluster 1, H1e	0.97039	0.989262	0.974027
3009	HIST1H1B	histone cluster 1, H1b	0.94433	0.843894	0.9833584
3010	HIST1H1T	histone cluster 1, H1t	1.00603	0.959142	0.9889228
3012	HIST1H2AE	histone cluster 1, H2ae	0.96468	0.959382	0.9998348
3014	H2AFX	H2A histone family, member X	0.94925	1.070669	1.0818716
3015	H2AFZ	H2A histone family, member Z	1.00839	0.992376	0.9396724
3017	HIST1H2BD	histone cluster 1, H2bd	0.96659	0.939761	0.9739496
3018	HIST1H2BB	histone cluster 1, H2bb	0.98173	0.980535	1.040448
3020	H3F3A	H3 histone, family 3A	1.08382	0.956623	0.9033607
3021	H3F3B	H3 histone, family 3B (H3.3B)	0.94581	0.937721	0.9365348
3024	HIST1H1A	histone cluster 1, H1a	1.01632	0.980814	0.9654342
3026	HABP2	hyaluronan binding protein 2	1.00169	0.960399	0.9547977
3028	HSD17B10	hydroxysteroid (17-beta) dehydrogenase 10	0.97996	0.870448	1.1782192
3029	HAGH	hydroxyacylglutathione hydrolase	0.95393	0.963099	1.0872257
3030	HADHA	hydroxyacyl-CoA dehydrogenase/3-ketoacyl-CoA thiolase/enoyl-CoA hydratase (trifunctional protein), alpha	0.9818	1.059739	1.0463262
3031	HADHAP1	hydroxyacyl-CoA dehydrogenase/3-ketoacyl-CoA thiolase/enoyl-CoA hydratase (trifunctional protein), alpha	1.01028	0.965458	0.9768306
3032	HADHB	hydroxyacyl-CoA dehydrogenase/3-ketoacyl-CoA thiolase/enoyl-CoA hydratase (trifunctional protein), beta s	1.02454	1.004048	1.0333976
3033	HADH	hydroxyacyl-CoA dehydrogenase	0.9876	0.97819	1.0376425
3034	HAL	histidine ammonia-lyase	0.96809	1.050053	0.9855339
3035	HARS	histidyl-tRNA synthetase	0.98868	1.013065	1.0330965
3036	HAS1	hyaluronan synthase 1	0.99359	1.000628	1.015705
3037	HAS2	hyaluronan synthase 2	0.99939	0.99855	1.0001739
3038	HAS3	hyaluronan synthase 3	0.98528	1.007682	1.0194651
3041	HBAP1	hemoglobin, alpha pseudogene 1	0.99454	0.98289	0.968668
3042	HBM	hemoglobin, mu	0.93967	0.958669	1.0061814
3043	HBB	hemoglobin, beta	0.9907	0.855513	0.991443
3044	HBBP1	hemoglobin, beta pseudogene 1	0.98902	0.980192	1.0639265
3045	HBD	hemoglobin, delta	0.98232	0.949599	1.0136898
3046	HBE1	hemoglobin, epsilon 1	1.00066	0.983442	0.9868816
3049	HBQ1	hemoglobin, theta 1	0.94194	0.869367	0.9999597
3052	HCCS	holocytochrome c synthase	0.91179	0.941004	1.004213
3053	SERPIND1	serpin peptidase inhibitor, clade D (heparin cofactor), member 1	0.97575	0.981304	1.0147289
3054	HCFC1	host cell factor C1 (VP16-accessory protein)	0.96037	0.952087	1.0390202

3055	HCK	hemopoietic cell kinase	1.0121	1.01492	0.9302188
3059	HCLS1	hematopoietic cell-specific Lyn substrate 1	1.0514	0.974244	0.9491178
3060	HCRT	hypocretin (orexin) neuropeptide precursor	0.96161	1.031702	1.0240367
3061	HCRTR1	hypocretin (orexin) receptor 1	1.02389	1.004224	1.0073006
3062	HCRTR2	hypocretin (orexin) receptor 2	1.01953	0.990586	1.0017142
3064	HTT	huntingtin	0.98298	1.065817	1.0022262
3065	HDAC1	histone deacetylase 1	0.9939	0.951765	0.9910508
3066	HDAC2	histone deacetylase 2	0.9835	0.976523	1.0392277
3067	HDC	histidine decarboxylase	0.97499	0.920835	0.9849278
3068	HDGF	hepatoma-derived growth factor	0.9719	0.96066	1.0911666
3069	HDLBP	high density lipoprotein binding protein	0.99642	1.01445	1.0308533
3070	HELLS	helicase, lymphoid-specific	0.95591	0.955311	0.9854778
3071	NCKAP1L	NCK-associated protein 1-like	0.99272	0.926018	1.1022416
3073	HEXA	hexosaminidase A (alpha polypeptide)	0.96969	0.931764	1.0717084
3074	HEXB	hexosaminidase B (beta polypeptide)	0.98446	0.938893	1.0010943
3075	CFH	complement factor H	1.00286	0.982329	0.9479952
3077	HFE	hemochromatosis	0.99285	0.948577	1.0732833
3080	CFHR2	complement factor H-related 2	0.99898	0.980223	1.0020801
3081	HGD	homogentisate 1,2-dioxygenase	0.9684	0.954023	0.9697018
3082	HGF	hepatocyte growth factor (hepapoietin A; scatter factor)	1.01852	0.949709	1.0947742
3083	HGFAC	HGF activator	1.00559	0.956321	1.0508968
3084	NRG1	neuregulin 1	1.01016	0.994995	1.0167294
3087	HHEX	hematopoietically expressed homeobox	0.96925	0.899049	0.9813526
3090	HIC1	hypermethylated in cancer 1	0.98978	1.027744	0.9513712
3091	HIF1A	hypoxia inducible factor 1, alpha subunit (basic helix-loop-helix transcription factor)	0.96802	0.988692	0.9106732
3092	HIP1	huntingtin interacting protein 1	1.02791	0.975648	0.9978342
3093	UBE2K	ubiquitin-conjugating enzyme E2K	0.99759	1.012798	0.9604096
3094	HINT1	histidine triad nucleotide binding protein 1	1.04077	0.972067	0.9570824
3096	HIVEP1	human immunodeficiency virus type I enhancer binding protein 1	0.96419	0.983205	0.9701796
3097	HIVEP2	human immunodeficiency virus type I enhancer binding protein 2	0.96898	0.999396	0.9907172
3098	HK1	hexokinase 1	0.98968	1.015554	1.028564
3099	HK2	hexokinase 2	1.01778	0.997069	0.9691518
3101	HK3	hexokinase 3 (white cell)	1.03311	0.956233	1.0576361

3104	ZBTB48	zinc finger and BTB domain containing 48	0.98039	1.012698	0.9756594
3108	HLA-DMA	major histocompatibility complex, class II, DM alpha	1.02084	0.957559	1.0259138
3109	HLA-DMB	major histocompatibility complex, class II, DM beta	1.03748	0.997808	0.9674596
3110	MNX1	motor neuron and pancreas homeobox 1	1.01693	0.98038	1.0734179
3111	HLA-DOA	major histocompatibility complex, class II, DO alpha	0.96702	0.958297	0.9882705
3113	HLA-DPA1	major histocompatibility complex, class II, DP alpha 1	1.0343	0.929312	1.0181921
3115	HLA-DPB1	major histocompatibility complex, class II, DP beta 1	1.04369	0.990699	0.9091221
3116	HLA-DPB2	major histocompatibility complex, class II, DP beta 2 (pseudogene)	1.01186	0.973284	0.9920764
3118	HLA-DQA2	major histocompatibility complex, class II, DQ alpha 2	1.03619	0.94339	0.9875115
3120	HLA-DQB2	major histocompatibility complex, class II, DQ beta 2	1.01249	0.994076	1.0694295
3122	HLA-DRA	major histocompatibility complex, class II, DR alpha	0.99704	0.939339	1.0732283
3127	HLA-DRB5	major histocompatibility complex, class II, DR beta 5	0.99948	0.996123	1.0226804
3128	HLA-DRB6	major histocompatibility complex, class II, DR beta 6 (pseudogene)	1.04501	0.930836	1.0775702
3131	HLF	hepatic leukemia factor	0.99457	1.024619	1.0068662
3133	HLA-E	major histocompatibility complex, class I, E	1.01461	0.923369	0.9142472
3134	HLA-F	major histocompatibility complex, class I, F	1.02999	1.032424	0.9002063
3135	HLA-G	major histocompatibility complex, class I, G	0.9897	0.857403	1.0008154
3136	HLA-H	major histocompatibility complex, class I, H (pseudogene)	0.99224	0.983352	1.1034201
3137	HLA-J	major histocompatibility complex, class I, J (pseudogene)	0.97062	0.892654	1.0876466
3138	HLA-K	major histocompatibility complex, class I, K (pseudogene)	0.9589	0.891734	1.037897
3140	MR1	major histocompatibility complex, class I-related	1.01966	0.94756	1.0175278
3141	HLCS	holocarboxylase synthetase (biotin-(proprionyl-CoA-carboxylase (ATP-hydrolysing)) ligase)	0.97485	0.98341	0.996038
3142	HLX	H2.0-like homeobox	0.96859	0.986011	0.9737599
3145	HMBS	hydroxymethylbilane synthase	0.97756	0.986021	1.0282454
3146	HMGB1	high mobility group box 1	0.97712	0.930812	0.9553104
3148	HMGB2	high mobility group box 2	0.98227	0.960663	1.0008361
3149	HMGB3	high mobility group box 3	0.98408	0.99567	1.042975
3150	HMGN1	high mobility group nucleosome binding domain 1	0.9871	0.893818	0.9853508
3151	HMGN2	high mobility group nucleosomal binding domain 2	1.03928	0.818455	0.9498075
3152	HMGN2P11	high mobility group nucleosomal binding domain 2 pseudogene 11	1.00618	0.991054	1.0706902
3155	HMGCL	3-hydroxymethyl-3-methylglutaryl-CoA lyase	0.98725	0.999919	0.9917117
3156	HMGCR	3-hydroxy-3-methylglutaryl-CoA reductase	1.00263	1.000737	0.9564928
3157	HMGCS1	3-hydroxy-3-methylglutaryl-CoA synthase 1 (soluble)	0.99603	1.00795	0.9749735

3158	HMGCS2	3-hydroxy-3-methylglutaryl-CoA synthase 2 (mitochondrial)	1.01699	1	1.0202225
3159	HMGA1	high mobility group AT-hook 1	1.00416	0.993487	1.0046527
3161	HMMR	hyaluronan-mediated motility receptor (RHAMM)	1.01173	1.003508	1.011123
3162	HMOX1	heme oxygenase (decycling) 1	0.98565	0.951136	0.862654
3163	HMOX2	heme oxygenase (decycling) 2	0.93513	0.963541	0.9918976
3164	NR4A1	nuclear receptor subfamily 4, group A, member 1	1.02338	1.090288	0.9022803
3167	HMX2	H6 family homeobox 2	0.98188	0.994202	1.0171769
3169	FOXA1	forkhead box A1	0.98312	0.975626	0.9709812
3170	FOXA2	forkhead box A2	0.99258	1.00487	0.987144
3171	FOXA3	forkhead box A3	0.98438	1.01247	1.0417538
3172	HNF4A	hepatocyte nuclear factor 4, alpha	0.98987	1.013171	0.9984707
3174	HNF4G	hepatocyte nuclear factor 4, gamma	0.99006	1.015422	0.999763
3175	ONECUT1	one cut homeobox 1	1.00805	0.974343	1.0507021
3176	HNMT	histamine N-methyltransferase	1.00552	0.966186	0.9816778
3177	SLC29A2	solute carrier family 29 (nucleoside transporters), member 2	0.97403	0.994646	1.0044782
3178	HNRNPA1	heterogeneous nuclear ribonucleoprotein A1	1.04704	1.012281	1.1096256
3181	HNRNPA2B	heterogeneous nuclear ribonucleoprotein A2/B1	1.07991	1.01599	0.8775011
3182	HNRNPAB	heterogeneous nuclear ribonucleoprotein A/B	0.99748	0.933662	1.0848349
3183	HNRNPC	heterogeneous nuclear ribonucleoprotein C (C1/C2)	1.03959	1.051471	0.8851529
3184	HNRNPD	heterogeneous nuclear ribonucleoprotein D (AU-rich element RNA binding protein 1, 37kDa)	1.0367	0.833274	0.9827958
3185	HNRNPF	heterogeneous nuclear ribonucleoprotein F	0.96586	0.936769	1.0974599
3186	RBMXP1	RNA binding motif protein, X-linked pseudogene 1	1.02516	1.031093	0.9427128
3187	HNRNPH1	heterogeneous nuclear ribonucleoprotein H1 (H)	1.03385	0.99891	1.0010291
3188	HNRNPH2	heterogeneous nuclear ribonucleoprotein H2 (H')	0.96078	0.996734	1.0324476
3189	HNRNPH3	heterogeneous nuclear ribonucleoprotein H3 (2H9)	0.96516	0.898874	0.9704819
3190	HNRNPK	heterogeneous nuclear ribonucleoprotein K	1.03336	0.945292	0.8951173
3191	HNRNPL	heterogeneous nuclear ribonucleoprotein L	0.96293	1.043517	0.9171666
3192	HNRNPU	heterogeneous nuclear ribonucleoprotein U (scaffold attachment factor A)	1.02635	0.922703	0.9402318
3195	TLX1	T-cell leukemia homeobox 1	1.0072	0.989066	1.0119159
3196	TLX2	T-cell leukemia homeobox 2	0.98825	1.031909	1.0879158
3198	HOXA1	homeobox A1	1.01571	1.001515	1.00717
3199	HOXA2	homeobox A2	1.02203	0.993725	0.9646085
3200	HOXA3	homeobox A3	1.00025	1.034887	1.0157295

3201	HOXA4	homeobox A4	0.99273	1.026526	1.0062123
3202	HOXA5	homeobox A5	0.98454	0.99932	1.0158099
3203	HOXA6	homeobox A6	1.00284	0.9939	1.0014944
3204	HOXA7	homeobox A7	0.97225	0.995443	1.0364331
3205	HOXA9	homeobox A9	1.00466	1.010573	1.0322
3206	HOXA10	homeobox A10	0.97955	1.041057	1.0431553
3207	HOXA11	homeobox A11	1.02171	0.998555	1.0012247
3208	HPCA	hippocalcin	1.00524	1.027871	1.0373243
3209	HOXA13	homeobox A13	1.0131	0.954757	1.0818751
3211	HOXB1	homeobox B1	0.99862	1.02337	0.9999567
3212	HOXB2	homeobox B2	0.94539	0.931203	1.1198487
3213	HOXB3	homeobox B3	0.99613	0.972212	1.0206156
3214	HOXB4	homeobox B4	0.96587	1.016811	1.075847
3215	HOXB5	homeobox B5	1.00573	1.007029	1.0598068
3216	HOXB6	homeobox B6	0.99371	1.001749	1.0400144
3217	HOXB7	homeobox B7	0.97898	0.987887	1.0076508
3218	HOXB8	homeobox B8	0.9686	1.018587	1.0406178
3219	HOXB9	homeobox B9	0.98987	1.013182	1.0423037
3221	HOXC4	homeobox C4	0.98346	1.053465	1.032472
3224	HOXC8	homeobox C8	1.00892	1.014573	0.9925737
3225	HOXC9	homeobox C9	0.98853	1.014539	1.0160885
3226	HOXC10	homeobox C10	1.0079	0.986658	0.9990635
3227	HOXC11	homeobox C11	0.99303	1.007097	1.0273452
3228	HOXC12	homeobox C12	0.97151	1.025271	1.0539939
3229	HOXC13	homeobox C13	1.02249	0.99429	1.0213851
3231	HOXD1	homeobox D1	1.01174	0.966432	1.0119876
3232	HOXD3	homeobox D3	1.0004	1.029689	1.0119661
3233	HOXD4	homeobox D4	0.99859	0.992699	1.0226038
3234	HOXD8	homeobox D8	0.98791	1.040791	1.0219571
3235	HOXD9	homeobox D9	1.00391	1.020366	1.0329446
3236	HOXD10	homeobox D10	1.02612	1.014692	0.9944604
3237	HOXD11	homeobox D11	0.99098	1.02066	1.04508
3238	HOXD12	homeobox D12	0.97543	0.988634	1.0163145

3239	HOXD13	homeobox D13	0.97063	0.994819	1.0650699
3240	HP	haptoglobin	0.98304	0.976311	0.9889335
3241	HPCAL1	hippocalcin-like 1	0.99157	1.042248	0.9930728
3242	HPD	4-hydroxyphenylpyruvate dioxygenase	0.99151	1.017577	1.0057788
3248	HPGD	hydroxyprostaglandin dehydrogenase 15-(NAD)	0.98929	0.996609	1.004631
3249	HPN	hepsin	0.99863	1.006121	1.0341605
3250	HPR	haptoglobin-related protein	0.98538	1.024155	1.0475309
3251	HPRT1	hypoxanthine phosphoribosyltransferase 1	0.95111	0.863623	1.0251608
3257	HPS1	Hermansky-Pudlak syndrome 1	0.98152	0.989869	1.0311163
3263	HPX	hemopexin	1.00675	0.969571	0.9806724
3265	HRAS	v-Ha-ras Harvey rat sarcoma viral oncogene homolog	0.95616	0.991319	1.0441613
3266	ERAS	ES cell expressed Ras	0.99699	0.970318	1.047202
3267	AGFG1	ArfGAP with FG repeats 1	1.00967	1.002046	0.9841196
3268	AGFG2	ArfGAP with FG repeats 2	1.00297	1.026742	1.008035
3269	HRH1	histamine receptor H1	1.02358	1.005196	1.0101117
3270	HRC	histidine rich calcium binding protein	0.98871	0.972416	1.0023476
3274	HRH2	histamine receptor H2	1.03318	0.86805	1.0348837
3275	PRMT2	protein arginine methyltransferase 2	0.94338	1.026351	0.9168974
3276	PRMT1	protein arginine methyltransferase 1	0.96271	1.019563	1.0696854
3280	HES1	hairy and enhancer of split 1, (Drosophila)	1.00583	1.032255	1.0279429
3281	HSBP1	heat shock factor binding protein 1	0.93984	0.998349	0.9154125
3283	HSD3B1	hydroxy-delta-5-steroid dehydrogenase, 3 beta- and steroid delta-isomerase 1	1.01442	1.032453	0.9679305
3284	HSD3B2	hydroxy-delta-5-steroid dehydrogenase, 3 beta- and steroid delta-isomerase 2	1.00913	0.997751	0.9749496
3290	HSD11B1	hydroxysteroid (11-beta) dehydrogenase 1	1.01096	1.008499	0.9923707
3291	HSD11B2	hydroxysteroid (11-beta) dehydrogenase 2	0.99169	0.981972	1.0955939
3292	HSD17B1	hydroxysteroid (17-beta) dehydrogenase 1	1.00013	0.958237	1.0719531
3293	HSD17B3	hydroxysteroid (17-beta) dehydrogenase 3	0.99008	1.004233	0.9784383
3294	HSD17B2	hydroxysteroid (17-beta) dehydrogenase 2	0.99366	1.013645	1.0162075
3295	HSD17B4	hydroxysteroid (17-beta) dehydrogenase 4	1.02449	1.000921	1.0200378
3297	HSF1	heat shock transcription factor 1	1.00244	0.968231	1.0122856
3298	HSF2	heat shock transcription factor 2	0.99069	0.963215	0.9291596
3299	HSF4	heat shock transcription factor 4	0.99788	0.995435	1.0816992
3300	DNAJB2	DnaJ (Hsp40) homolog, subfamily B, member 2	0.94818	0.951921	0.9786398

3301	DNAJA1	DnaJ (Hsp40) homolog, subfamily A, member 1	0.92097	0.928994	0.9601626
3303	HSPA1A	heat shock 70kDa protein 1A	1.05021	1.085174	1.0410005
3304	HSPA1B	heat shock 70kDa protein 1B	0.97291	0.998191	1.0067751
3305	HSPA1L	heat shock 70kDa protein 1-like	0.9937	0.937859	0.9478769
3306	HSPA2	heat shock 70kDa protein 2	0.96616	1.078318	0.9940218
3308	HSPA4	heat shock 70kDa protein 4	1.01062	1.034902	1.1093726
3309	HSPA5	heat shock 70kDa protein 5 (glucose-regulated protein, 78kDa)	0.97637	0.92313	1.0171422
3312	HSPA8	heat shock 70kDa protein 8	0.96065	0.892025	0.9796003
3313	HSPA9	heat shock 70kDa protein 9 (mortalin)	1.0369	1.005617	0.9776356
3315	HSPB1	heat shock 27kDa protein 1	0.95578	0.974323	1.0073844
3316	HSPB2	heat shock 27kDa protein 2	0.96837	1.055924	0.9887473
3320	HSP90AA1	heat shock protein 90kDa alpha (cytosolic), class A member 1	0.96443	0.944378	0.9924626
3321	IGSF3	immunoglobulin superfamily, member 3	0.99403	1.022326	1.0066936
3322	HSP90AA3I	heat shock protein 90kDa alpha (cytosolic), class A member 3, pseudogene	1.00467	0.930893	0.9869984
3323	HSP90AA4I	heat shock protein 90kDa alpha (cytosolic), class A member 4, pseudogene	0.99371	1.013217	0.9799854
3324	HSP90AA2	heat shock protein 90kDa alpha (cytosolic), class A member 2	0.99769	0.879119	0.9863004
3326	HSP90AB1	heat shock protein 90kDa alpha (cytosolic), class B member 1	0.99757	0.960125	0.9466709
3327	HSP90AB3I	heat shock protein 90kDa alpha (cytosolic), class B member 3, pseudogene	0.98758	0.969006	1.0002437
3329	HSPD1	heat shock 60kDa protein 1 (chaperonin)	0.99652	1.067011	0.9983301
3336	HSPE1	heat shock 10kDa protein 1 (chaperonin 10)	1.00143	1.043211	1.0088829
3337	DNAJB1	DnaJ (Hsp40) homolog, subfamily B, member 1	0.90896	0.922509	0.9452959
3338	DNAJC4	DnaJ (Hsp40) homolog, subfamily C, member 4	0.97604	0.946339	1.02401
3339	HSPG2	heparan sulfate proteoglycan 2	1.00461	1.010876	1.0191071
3344	FOXN2	forkhead box N2	1.02246	0.914452	1.0297571
3346	HTN1	histatin 1	0.98368	0.98412	1.0457386
3347	HTN3	histatin 3	0.99303	1.009037	1.0242034
3350	HTR1A	5-hydroxytryptamine (serotonin) receptor 1A	0.98906	0.987614	1.0895
3351	HTR1B	5-hydroxytryptamine (serotonin) receptor 1B	1.00385	0.944128	1.0787392
3352	HTR1D	5-hydroxytryptamine (serotonin) receptor 1D	1.05535	1.048672	0.9374472
3354	HTR1E	5-hydroxytryptamine (serotonin) receptor 1E	1.02694	0.995443	1.0039562
3355	HTR1F	5-hydroxytryptamine (serotonin) receptor 1F	1.0215	0.983416	1.000685
3356	HTR2A	5-hydroxytryptamine (serotonin) receptor 2A	0.99707	0.984462	0.9959892
3357	HTR2B	5-hydroxytryptamine (serotonin) receptor 2B	1.00918	0.985753	1.0000449

3358	HTR2C	5-hydroxytryptamine (serotonin) receptor 2C	0.98297	0.992528	0.9812503
3359	HTR3A	5-hydroxytryptamine (serotonin) receptor 3A	0.99385	1.016879	0.9640895
3360	HTR4	5-hydroxytryptamine (serotonin) receptor 4	1.01556	0.997567	1.0219124
3361	HTR5A	5-hydroxytryptamine (serotonin) receptor 5A	0.99332	0.992398	0.9891641
3362	HTR6	5-hydroxytryptamine (serotonin) receptor 6	0.98589	0.993785	1.0381742
3363	HTR7	5-hydroxytryptamine (serotonin) receptor 7 (adenylate cyclase-coupled)	1.01281	0.996792	0.9710597
3364	HUS1	HUS1 checkpoint homolog (S. pombe)	1.00369	0.965824	0.9574589
3371	TNC	tenascin C	1.00408	0.989661	0.9821111
3373	HYAL1	hyaluronoglucosaminidase 1	0.99073	1.003783	0.977048
3375	IAPP	islet amyloid polypeptide	1.01162	0.991574	0.9974138
3376	IARS	isoleucyl-tRNA synthetase	0.95529	0.970741	1.0200824
3381	IBSP	integrin-binding sialoprotein	1.02297	1.005619	1.0056301
3382	ICA1	islet cell autoantigen 1, 69kDa	0.99501	0.989302	0.9875414
3383	ICAM1	intercellular adhesion molecule 1	0.95513	1.058215	0.9612849
3384	ICAM2	intercellular adhesion molecule 2	0.91895	0.903898	0.9566245
3385	ICAM3	intercellular adhesion molecule 3	0.97695	0.93011	1.1285583
3386	ICAM4	intercellular adhesion molecule 4 (Landsteiner-Wiener blood group)	0.93416	0.970624	1.0443914
3394	IRF8	interferon regulatory factor 8	1.00985	1.08827	1.0134722
3396	ICT1	immature colon carcinoma transcript 1	0.94673	0.98603	0.9799234
3397	ID1	inhibitor of DNA binding 1, dominant negative helix-loop-helix protein	0.99593	0.936636	0.9222911
3398	ID2	inhibitor of DNA binding 2, dominant negative helix-loop-helix protein	1.04189	0.995911	1.011016
3399	ID3	inhibitor of DNA binding 3, dominant negative helix-loop-helix protein	0.94062	0.90309	0.8892109
3400	ID4	inhibitor of DNA binding 4, dominant negative helix-loop-helix protein	0.98982	1.019646	1.0327565
3416	IDE	insulin-degrading enzyme	0.97541	0.982568	1.0562049
3417	IDH1	isocitrate dehydrogenase 1 (NADP+), soluble	0.99754	0.984994	1.03336
3418	IDH2	isocitrate dehydrogenase 2 (NADP+), mitochondrial	0.95955	0.989897	1.0486526
3419	IDH3A	isocitrate dehydrogenase 3 (NAD+) alpha	0.97771	0.972973	1.0855857
3420	IDH3B	isocitrate dehydrogenase 3 (NAD+) beta	0.94557	0.912659	1.0541354
3421	IDH3G	isocitrate dehydrogenase 3 (NAD+) gamma	0.95102	0.980569	1.0690965
3422	IDI1	isopentenyl-diphosphate delta isomerase 1	0.95581	0.920472	0.9872781
3423	IDS	iduronate 2-sulfatase	0.95179	0.938522	0.9846889
3425	IDUA	iduronidase, alpha-L-	0.99643	1.014216	1.0343841
3426	CFI	complement factor I	1.02149	1.013298	0.9944741

3428	IFI16	interferon, gamma-inducible protein 16	0.98407	0.963086	0.9696615
3429	IFI27	interferon, alpha-inducible protein 27	1.0081	0.991557	1.0181851
3430	IFI35	interferon-induced protein 35	0.92483	0.903806	1.1520923
3431	SP110	SP110 nuclear body protein	1.00348	0.922662	1.0279702
3433	IFIT2	interferon-induced protein with tetratricopeptide repeats 2	0.9453	0.902474	1.0249754
3434	IFIT1	interferon-induced protein with tetratricopeptide repeats 1	0.94011	0.917515	0.9743993
3437	IFIT3	interferon-induced protein with tetratricopeptide repeats 3	0.96434	0.891802	0.9953456
3440	IFNA2	interferon, alpha 2	1.00114	0.962919	0.9827364
3442	IFNA5	interferon, alpha 5	0.95913	1.034279	1.024119
3445	IFNA8	interferon, alpha 8	0.99632	0.989101	1.0378397
3448	IFNA14	interferon, alpha 14	1.02079	0.929709	0.9513671
3454	IFNAR1	interferon (alpha, beta and omega) receptor 1	0.92716	1.01248	1.0306948
3455	IFNAR2	interferon (alpha, beta and omega) receptor 2	0.96127	1.087825	0.9399496
3456	IFNB1	interferon, beta 1, fibroblast	0.98041	1.042722	1.0519947
3458	IFNG	interferon, gamma	0.91684	0.931482	0.8166773
3459	IFNGR1	interferon gamma receptor 1	1.04922	1.029143	0.9655512
3460	IFNGR2	interferon gamma receptor 2 (interferon gamma transducer 1)	0.99304	1.101849	0.9621991
3467	IFNW1	interferon, omega 1	0.98823	0.990536	0.9433455
3474	IFNWP19	interferon, omega 1 pseudogene 19	0.99609	1.002568	0.8787867
3475	IFRD1	interferon-related developmental regulator 1	0.98286	1.02273	0.9269818
3476	IGBP1	immunoglobulin (CD79A) binding protein 1	0.96157	0.897868	1.069333
3479	IGF1	insulin-like growth factor 1 (somatomedin C)	0.99991	1.023332	1.0098002
3480	IGF1R	insulin-like growth factor 1 receptor	0.96358	0.921747	0.8856112
3482	IGF2R	insulin-like growth factor 2 receptor	1.02833	1.111071	0.9765957
3483	IGFALS	insulin-like growth factor binding protein, acid labile subunit	0.98669	1.002853	1.028684
3484	IGFBP1	insulin-like growth factor binding protein 1	0.99949	1.031142	0.9983005
3485	IGFBP2	insulin-like growth factor binding protein 2, 36kDa	0.97678	1.053749	1.044219
3486	IGFBP3	insulin-like growth factor binding protein 3	0.99743	1.051305	1.0318903
3487	IGFBP4	insulin-like growth factor binding protein 4	0.99293	1.003848	0.9914097
3488	IGFBP5	insulin-like growth factor binding protein 5	1.01428	0.986143	1.0170545
3489	IGFBP6	insulin-like growth factor binding protein 6	0.99705	0.985214	1.0422374
3490	IGFBP7	insulin-like growth factor binding protein 7	1.02184	0.989398	0.9998187
3491	CYR61	cysteine-rich, angiogenic inducer, 61	0.99596	0.970784	1.0274714

3494	IGHA2	immunoglobulin heavy constant alpha 2 (A2m marker)	0.96154	0.955857	1.1223787
3497	IGHE	immunoglobulin heavy constant epsilon	0.97313	1.037578	1.0271896
3500	IGHG1	immunoglobulin heavy constant gamma 1 (G1m marker)	0.96795	0.904596	0.9965344
3501	IGHG2	immunoglobulin heavy constant gamma 2 (G2m marker)	1.01645	0.909326	1.024417
3502	IGHG3	immunoglobulin heavy constant gamma 3 (G3m marker)	1.03785	0.939782	0.9335562
3507	IGHM	immunoglobulin heavy constant mu	0.92043	1.02209	0.9722903
3508	IGHMBP2	immunoglobulin mu binding protein 2	0.96782	0.952333	1.0325034
3512	IGJ	immunoglobulin J polypeptide, linker protein for immunoglobulin alpha and mu polypeptides	1.07871	0.892793	1.1938604
3516	RBPJ	recombination signal binding protein for immunoglobulin kappa J region	0.98865	0.962312	0.9875689
3535	IGL@	immunoglobulin lambda locus	1.00605	1.007981	1.0082174
3543	IGLL1	immunoglobulin lambda-like polypeptide 1	0.97778	1.038639	1.0179636
3547	IGSF1	immunoglobulin superfamily, member 1	1.0097	0.983294	0.9913299
3549	IHH	Indian hedgehog	1.01866	0.987535	1.0404654
3550	IK	IK cytokine, down-regulator of HLA II	1.00562	0.930291	0.9540197
3551	IKBKB	inhibitor of kappa light polypeptide gene enhancer in B-cells, kinase beta	0.99109	0.952024	0.9677164
3552	IL1A	interleukin 1, alpha	1.01792	1.025223	0.9920696
3553	IL1B	interleukin 1, beta	0.97149	1.176243	0.7430692
3554	IL1R1	interleukin 1 receptor, type I	0.99258	1.041396	1.0311928
3556	IL1RAP	interleukin 1 receptor accessory protein	1.00689	1.02558	0.9934655
3557	IL1RN	interleukin 1 receptor antagonist	1.01806	1.14914	0.9267438
3558	IL2	interleukin 2	1.006	0.98603	1.0144901
3559	IL2RA	interleukin 2 receptor, alpha	0.98215	0.997202	1.0051335
3560	IL2RB	interleukin 2 receptor, beta	0.97989	0.969457	0.9955574
3561	IL2RG	interleukin 2 receptor, gamma	0.95529	1.022874	0.99732
3562	IL3	interleukin 3 (colony-stimulating factor, multiple)	1.00233	1.040717	1.0355964
3565	IL4	interleukin 4	1.00248	0.999641	0.9994252
3566	IL4R	interleukin 4 receptor	0.94658	0.98605	0.9938742
3567	IL5	interleukin 5 (colony-stimulating factor, eosinophil)	1.01066	1.029198	1.0030138
3568	IL5RA	interleukin 5 receptor, alpha	1.00356	1.007791	1.0083754
3569	IL6	interleukin 6 (interferon, beta 2)	0.9807	1.054929	0.9912018
3572	IL6ST	interleukin 6 signal transducer (gp130, oncostatin M receptor)	0.96369	0.863059	1.0186681
3573	IL6STP1	interleukin 6 signal transducer (gp130, oncostatin M receptor) pseudogene 1	0.9371	0.894355	0.9863054
3574	IL7	interleukin 7	0.9794	0.981252	1.0290552

3575	IL7R	interleukin 7 receptor	0.96422	0.971252	0.9460074
3576	IL8	interleukin 8	1.00229	1.076823	0.8716807
3577	CXCR1	chemokine (C-X-C motif) receptor 1	0.99062	0.914812	0.9381703
3578	IL9	interleukin 9	0.99604	1.018585	0.9900204
3579	CXCR2	chemokine (C-X-C motif) receptor 2	1.03579	0.883903	0.9116791
3580	CXCR2P1	chemokine (C-X-C motif) receptor 2 pseudogene 1	0.97733	1.027822	0.9806124
3585	IL9RP4	interleukin 9 receptor pseudogene 4	0.98888	1.033209	0.9835346
3586	IL10	interleukin 10	1.02293	0.99103	1.0112664
3587	IL10RA	interleukin 10 receptor, alpha	1.00857	1.085959	0.9746381
3588	IL10RB	interleukin 10 receptor, beta	1.0108	1.078753	0.9321351
3589	IL11	interleukin 11	0.98231	1.03304	0.9815599
3590	IL11RA	interleukin 11 receptor, alpha	0.97911	1.003193	0.9497063
3592	IL12A	interleukin 12A (natural killer cell stimulatory factor 1, cytotoxic lymphocyte maturation factor 1, p35)	0.99014	0.997163	1.0381957
3593	IL12B	interleukin 12B (natural killer cell stimulatory factor 2, cytotoxic lymphocyte maturation factor 2, p40)	1.00776	0.985658	1.0268924
3594	IL12RB1	interleukin 12 receptor, beta 1	0.96193	0.945944	1.0321869
3595	IL12RB2	interleukin 12 receptor, beta 2	0.99697	1.065669	0.9859492
3596	IL13	interleukin 13	1.0175	0.976434	1.0239146
3597	IL13RA1	interleukin 13 receptor, alpha 1	0.96413	1.006499	1.0118435
3600	IL15	interleukin 15	1.01785	0.974761	1.1034622
3601	IL15RA	interleukin 15 receptor, alpha	0.9614	1.005887	1.0359545
3603	IL16	interleukin 16 (lymphocyte chemoattractant factor)	0.92656	1.00859	0.9633017
3604	TNFRSF9	tumor necrosis factor receptor superfamily, member 9	1.01933	1.051435	0.9546996
3605	IL17A	interleukin 17A	1.00068	1.025415	0.9981997
3606	IL18	interleukin 18 (interferon-gamma-inducing factor)	0.97347	0.981321	0.9639672
3607	FOXK2	forkhead box K2	0.96871	1.01722	0.9873276
3608	ILF2	interleukin enhancer binding factor 2, 45kDa	0.97388	0.966659	0.9724494
3609	ILF3	interleukin enhancer binding factor 3, 90kDa	0.96969	1.035501	1.0113786
3611	ILK	integrin-linked kinase	0.93145	1.037634	0.9922141
3612	IMPA1	inositol(myo)-1(or 4)-monophosphatase 1	0.9389	0.98268	1.0320261
3613	IMPA2	inositol(myo)-1(or 4)-monophosphatase 2	0.98154	1.024541	1.0166446
3614	IMPDH1	IMP (inosine 5'-monophosphate) dehydrogenase 1	1.01752	1.024405	0.9788049
3615	IMPDH2	IMP (inosine 5'-monophosphate) dehydrogenase 2	0.98801	0.999923	0.9979322
3617	IMPG1	interphotoreceptor matrix proteoglycan 1	1.02415	1.00251	1.0080434

3619	INCENP	inner centromere protein antigens 135/155kDa	0.97404	0.950957	1.0047397
3620	IDO1	indoleamine 2,3-dioxygenase 1	1.00938	1.049658	1.0056605
3621	ING1	inhibitor of growth family, member 1	0.93928	0.986306	0.9992018
3622	ING2	inhibitor of growth family, member 2	0.97935	1.041931	1.0480172
3623	INHA	inhibin, alpha	1.02278	0.994587	1.032064
3624	INHBA	inhibin, beta A	1.01532	0.998982	1.0042218
3625	INHBB	inhibin, beta B	0.94633	1.008533	0.9684177
3626	INHBC	inhibin, beta C	1.02237	0.971122	0.9600246
3627	CXCL10	chemokine (C-X-C motif) ligand 10	0.98216	1.013794	0.9871406
3628	INPP1	inositol polyphosphate-1-phosphatase	0.99552	0.984603	1.0196095
3631	INPP4A	inositol polyphosphate-4-phosphatase, type I, 107kDa	0.9973	0.918661	0.9035526
3632	INPP5A	inositol polyphosphate-5-phosphatase, 40kDa	0.9917	0.989629	0.9610776
3633	INPP5B	inositol polyphosphate-5-phosphatase, 75kDa	0.98484	0.99555	1.045997
3635	INPP5D	inositol polyphosphate-5-phosphatase, 145kDa	1.03146	0.966167	0.9774848
3636	INPPL1	inositol polyphosphate phosphatase-like 1	0.96185	1.066732	1.0033392
3638	INSIG1	insulin induced gene 1	1.00457	0.87923	0.9063551
3640	INSL3	insulin-like 3 (Leydig cell)	1.00529	0.966908	0.9256121
3641	INSL4	insulin-like 4 (placenta)	1.0013	1.057463	0.9597979
3642	INSM1	insulinoma-associated 1	0.99993	1.006964	1.0742174
3643	INSR	insulin receptor	0.99426	1.022168	1.0272254
3645	INSRR	insulin receptor-related receptor	0.99921	1.012186	1.0016822
3646	EIF3E	eukaryotic translation initiation factor 3, subunit E	0.96442	0.930567	1.0749973
3647	EIF3EP1	eukaryotic translation initiation factor 3, subunit E pseudogene 1	0.99097	1.050397	1.0178362
3651	PDX1	pancreatic and duodenal homeobox 1	0.99472	0.988547	0.9955677
3652	IPP	intracisternal A particle-promoted polypeptide	0.99016	0.954562	0.9858651
3654	IRAK1	interleukin-1 receptor-associated kinase 1	0.95174	1.108174	0.9962423
3655	ITGA6	integrin, alpha 6	0.95085	0.962114	0.8403986
3656	IRAK2	interleukin-1 receptor-associated kinase 2	0.98029	1.042551	1.0204792
3658	IREB2	iron-responsive element binding protein 2	0.98105	1.001176	1.1178755
3659	IRF1	interferon regulatory factor 1	1.02689	1.088745	0.9730619
3660	IRF2	interferon regulatory factor 2	0.99605	1.01619	0.9317682
3661	IRF3	interferon regulatory factor 3	0.97787	0.988168	1.0436182
3662	IRF4	interferon regulatory factor 4	0.99243	1.039901	0.9821179

3663	IRF5	interferon regulatory factor 5	1.01085	1.001275	1.1075724
3664	IRF6	interferon regulatory factor 6	0.998	0.948895	0.9742799
3665	IRF7	interferon regulatory factor 7	0.97911	0.999917	1.0632086
3669	ISG20	interferon stimulated exonuclease gene 20kDa	0.94151	1.013862	0.9435945
3670	ISL1	ISL LIM homeobox 1	1.01643	0.989882	1.0511455
3671	ISLR	immunoglobulin superfamily containing leucine-rich repeat	1.01446	0.985621	1.0363714
3672	ITGA1	integrin, alpha 1	0.99776	0.972917	1.0377547
3673	ITGA2	integrin, alpha 2 (CD49B, alpha 2 subunit of VLA-2 receptor)	0.99975	0.971161	1.0174725
3674	ITGA2B	integrin, alpha 2b (platelet glycoprotein IIb of IIb/IIIa complex, antigen CD41)	0.90831	1.003724	0.9936357
3675	ITGA3	integrin, alpha 3 (antigen CD49C, alpha 3 subunit of VLA-3 receptor)	0.97414	1.008346	1.0425364
3676	ITGA4	integrin, alpha 4 (antigen CD49D, alpha 4 subunit of VLA-4 receptor)	1.03653	1.000528	0.9755287
3678	ITGA5	integrin, alpha 5 (fibronectin receptor, alpha polypeptide)	0.94735	1.034268	1.0101041
3679	ITGA7	integrin, alpha 7	1.00135	1.023039	1.0113956
3680	ITGA9	integrin, alpha 9	1.01186	1.011427	1.003841
3681	ITGAD	integrin, alpha D	0.99875	1.028201	0.9922507
3682	ITGAE	integrin, alpha E (antigen CD103, human mucosal lymphocyte antigen 1; alpha polypeptide)	0.99409	1.024818	1.0047376
3683	ITGAL	integrin, alpha L (antigen CD11A (p180), lymphocyte function-associated antigen 1; alpha polypeptide)	1.00905	1.118315	1.0506444
3684	ITGAM	integrin, alpha M (complement component 3 receptor 3 subunit)	0.9841	0.906565	1.0702236
3685	ITGAV	integrin, alpha V (vitronectin receptor, alpha polypeptide, antigen CD51)	0.99569	1.049209	1.0219314
3687	ITGAX	integrin, alpha X (complement component 3 receptor 4 subunit)	1.0367	1.041797	1.0013617
3688	ITGB1	integrin, beta 1 (fibronectin receptor, beta polypeptide, antigen CD29 includes MDF2, MSK12)	1.01125	0.8916	1.0139688
3689	ITGB2	integrin, beta 2 (complement component 3 receptor 3 and 4 subunit)	1.01414	0.948405	1.0809884
3690	ITGB3	integrin, beta 3 (platelet glycoprotein IIIa, antigen CD61)	0.94057	0.918624	0.9917928
3691	ITGB4	integrin, beta 4	0.99678	1.012931	1.0015494
3692	EIF6	eukaryotic translation initiation factor 6	0.90728	1.048937	1.1666053
3693	ITGB5	integrin, beta 5	0.99428	0.957873	1.0153578
3694	ITGB6	integrin, beta 6	1.01328	0.988493	1.0091787
3695	ITGB7	integrin, beta 7	0.90817	0.894186	1.0146213
3696	ITGB8	integrin, beta 8	1.00396	1.01991	1.0409966
3697	ITIH1	inter-alpha (globulin) inhibitor H1	0.99566	1.018052	1.0050247
3698	ITIH2	inter-alpha (globulin) inhibitor H2	0.99925	1.000737	0.9986843
3699	ITIH3	inter-alpha (globulin) inhibitor H3	1.00076	0.978499	1.021519
3700	ITIH4	inter-alpha (globulin) inhibitor H4 (plasma Kallikrein-sensitive glycoprotein)	1.025	0.956461	0.9721623

3702	ITK	IL2-inducible T-cell kinase	0.95241	1.056599	0.9742967
3703	STT3A	STT3, subunit of the oligosaccharyltransferase complex, homolog A (S. cerevisiae)	0.9744	1.035629	1.0691135
3704	ITPA	inosine triphosphatase (nucleoside triphosphate pyrophosphatase)	0.93979	0.993019	0.9991785
3705	ITPK1	inositol-tetrakisphosphate 1-kinase	0.99943	1.000174	0.9900807
3706	ITPKA	inositol-trisphosphate 3-kinase A	1.00324	1.001859	1.1152256
3707	ITPKB	inositol-trisphosphate 3-kinase B	0.9431	1.081838	0.9574628
3708	ITPR1	inositol 1,4,5-trisphosphate receptor, type 1	0.99725	1.035805	1.0120122
3709	ITPR2	inositol 1,4,5-trisphosphate receptor, type 2	0.96174	0.981732	1.0133763
3710	ITPR3	inositol 1,4,5-trisphosphate receptor, type 3	0.98645	0.970824	0.9622839
3712	IVD	isovaleryl-CoA dehydrogenase	0.95179	0.914866	1.0525167
3713	IVL	involucrin	0.99512	0.988578	0.9975757
3714	JAG2	jagged 2	0.98301	1.020508	1.0773101
3716	JAK1	Janus kinase 1	1.0197	0.93775	0.9633452
3717	JAK2	Janus kinase 2	0.94951	0.895309	1.0434171
3718	JAK3	Janus kinase 3	0.98383	0.883328	1.0256117
3720	JARID2	jumonji, AT rich interactive domain 2	1.01221	1.088872	1.0133739
3725	JUN	jun proto-oncogene	1.02869	0.946341	0.8930449
3726	JUNB	jun B proto-oncogene	0.99677	0.896239	0.9384727
3727	JUND	jun D proto-oncogene	0.9144	0.914517	0.9261931
3728	JUP	junction plakoglobin	0.9805	1.011744	1.0168537
3730	KAL1	Kallmann syndrome 1 sequence	0.99954	1.00206	1.0226181
3732	CD82	CD82 molecule	0.98221	1.014597	1.035321
3734	KALP	Kallmann syndrome sequence pseudogene	1.04251	0.99172	1.0392863
3735	KARS	lysyl-tRNA synthetase	0.97588	0.948869	1.0365473
3736	KCNA1	potassium voltage-gated channel, shaker-related subfamily, member 1 (episodic ataxia with myokymia)	0.97885	0.981324	1.021166
3737	KCNA2	potassium voltage-gated channel, shaker-related subfamily, member 2	1.00249	1.000681	1.0550545
3738	KCNA3	potassium voltage-gated channel, shaker-related subfamily, member 3	0.9694	0.871974	0.9547707
3739	KCNA4	potassium voltage-gated channel, shaker-related subfamily, member 4	0.98492	0.977129	0.9914718
3741	KCNA5	potassium voltage-gated channel, shaker-related subfamily, member 5	0.98229	0.962543	1.0074807
3742	KCNA6	potassium voltage-gated channel, shaker-related subfamily, member 6	0.98836	1.017962	1.0125916
3743	KCNA7	potassium voltage-gated channel, shaker-related subfamily, member 7	0.97036	0.966237	1.0452959
3744	KCNA10	potassium voltage-gated channel, shaker-related subfamily, member 10	1.02112	0.962249	0.9947533
3745	KCNB1	potassium voltage-gated channel, Shab-related subfamily, member 1	0.99491	0.984245	0.9944921

3746	KCNC1	potassium voltage-gated channel, Shaw-related subfamily, member 1	0.99949	1.007046	1.0005164
3747	KCNC2	potassium voltage-gated channel, Shaw-related subfamily, member 2	0.98792	0.996911	1.0059598
3748	KCNC3	potassium voltage-gated channel, Shaw-related subfamily, member 3	1.01771	1.039087	1.0333154
3749	KCNC4	potassium voltage-gated channel, Shaw-related subfamily, member 4	1.01381	0.98765	0.9830846
3750	KCND1	potassium voltage-gated channel, Shal-related subfamily, member 1	0.99218	1.020024	0.9997245
3751	KCND2	potassium voltage-gated channel, Shal-related subfamily, member 2	0.99963	0.993996	1.0086366
3752	KCND3	potassium voltage-gated channel, Shal-related subfamily, member 3	1.00995	1.008776	1.0134903
3753	KCNE1	potassium voltage-gated channel, Isk-related family, member 1	1.0182	0.987974	0.9913808
3754	KCNF1	potassium voltage-gated channel, subfamily F, member 1	1.00975	1.004177	1.0054426
3755	KCNG1	potassium voltage-gated channel, subfamily G, member 1	0.97108	1.009165	1.0105531
3756	KCNH1	potassium voltage-gated channel, subfamily H (eag-related), member 1	1.01186	1.00881	1.0084802
3757	KCNH2	potassium voltage-gated channel, subfamily H (eag-related), member 2	1.00283	1.006659	1.022135
3758	KCNJ1	potassium inwardly-rectifying channel, subfamily J, member 1	1.01107	1.008904	0.9953476
3759	KCNJ2	potassium inwardly-rectifying channel, subfamily J, member 2	0.93792	1.038235	1.0141807
3760	KCNJ3	potassium inwardly-rectifying channel, subfamily J, member 3	1.01075	0.994395	0.9948575
3761	KCNJ4	potassium inwardly-rectifying channel, subfamily J, member 4	0.99201	1.010406	1.0236559
3762	KCNJ5	potassium inwardly-rectifying channel, subfamily J, member 5	1.01993	0.989142	0.9701291
3763	KCNJ6	potassium inwardly-rectifying channel, subfamily J, member 6	1.00434	1.006264	1.0033382
3764	KCNJ8	potassium inwardly-rectifying channel, subfamily J, member 8	0.99345	0.996491	0.9926949
3765	KCNJ9	potassium inwardly-rectifying channel, subfamily J, member 9	0.98451	0.995197	1.022781
3766	KCNJ10	potassium inwardly-rectifying channel, subfamily J, member 10	0.99893	0.985272	1.0116138
3767	KCNJ11	potassium inwardly-rectifying channel, subfamily J, member 11	0.99717	0.938019	1.0288395
3768	KCNJ12	potassium inwardly-rectifying channel, subfamily J, member 12	0.98829	1.025018	1.056076
3769	KCNJ13	potassium inwardly-rectifying channel, subfamily J, member 13	1.00591	1.032261	0.9984742
3770	KCNJ14	potassium inwardly-rectifying channel, subfamily J, member 14	0.99061	1.000083	1.0490677
3772	KCNJ15	potassium inwardly-rectifying channel, subfamily J, member 15	1.01051	0.946564	0.926073
3773	KCNJ16	potassium inwardly-rectifying channel, subfamily J, member 16	1.00221	0.965058	0.977864
3775	KCNK1	potassium channel, subfamily K, member 1	1.00864	0.995391	1.0142103
3776	KCNK2	potassium channel, subfamily K, member 2	1.00687	1.014657	1.0130089
3777	KCNK3	potassium channel, subfamily K, member 3	1.00645	0.998412	0.9992569
3778	KCNMA1	potassium large conductance calcium-activated channel, subfamily M, alpha member 1	1.00547	0.988439	0.996129
3779	KCNMB1	potassium large conductance calcium-activated channel, subfamily M, beta member 1	1.02637	0.924934	1.0173628
3780	KCNN1	potassium intermediate/small conductance calcium-activated channel, subfamily N, member 1	1.00451	1.006644	1.01655

3781	KCNN2	potassium intermediate/small conductance calcium-activated channel, subfamily N, member 2	1.00984	1.014305	1.0204062
3783	KCNN4	potassium intermediate/small conductance calcium-activated channel, subfamily N, member 4	0.99407	0.994224	1.0684005
3784	KCNQ1	potassium voltage-gated channel, KQT-like subfamily, member 1	0.98352	0.968317	1.0056492
3785	KCNQ2	potassium voltage-gated channel, KQT-like subfamily, member 2	0.98737	0.993126	1.0250216
3786	KCNQ3	potassium voltage-gated channel, KQT-like subfamily, member 3	1.00254	0.99414	0.9877095
3787	KCNS1	potassium voltage-gated channel, delayed-rectifier, subfamily S, member 1	0.98954	1.007899	1.0200921
3788	KCNS2	potassium voltage-gated channel, delayed-rectifier, subfamily S, member 2	1.00078	1.008298	1.0703281
3790	KCNS3	potassium voltage-gated channel, delayed-rectifier, subfamily S, member 3	1.00356	0.991203	0.9999133
3791	KDR	kinase insert domain receptor (a type III receptor tyrosine kinase)	1.00708	1.008766	1.0236048
3795	KHK	ketohexokinase (fructokinase)	0.99879	1.002976	1.0325577
3796	KIF2A	kinesin heavy chain member 2A	0.99587	0.933015	0.9097354
3797	KIF3C	kinesin family member 3C	1.01716	0.95832	1.0524722
3798	KIF5A	kinesin family member 5A	0.99456	1.001209	1.0032127
3799	KIF5B	kinesin family member 5B	0.95401	0.955054	1.0514761
3800	KIF5C	kinesin family member 5C	0.99382	1.007712	0.9846046
3801	KIFC3	kinesin family member C3	0.9781	0.973983	1.0178698
3802	KIR2DL1	killer cell immunoglobulin-like receptor, two domains, long cytoplasmic tail, 1	0.99342	1.07543	0.9287625
3804	KIR2DL3	killer cell immunoglobulin-like receptor, two domains, long cytoplasmic tail, 3	0.98225	1.023758	0.9568459
3805	KIR2DL4	killer cell immunoglobulin-like receptor, two domains, long cytoplasmic tail, 4	0.98125	0.991708	1.003198
3809	KIR2DS4	killer cell immunoglobulin-like receptor, two domains, short cytoplasmic tail, 4	0.81927	0.797724	1.102106
3811	KIR3DL1	killer cell immunoglobulin-like receptor, three domains, long cytoplasmic tail, 1	0.94822	0.998266	1.021955
3812	KIR3DL2	killer cell immunoglobulin-like receptor, three domains, long cytoplasmic tail, 2	0.9841	1.020022	0.9152353
3814	KISS1	KiSS-1 metastasis-suppressor	0.9863	0.962507	0.9943712
3815	KIT	v-kit Hardy-Zuckerman 4 feline sarcoma viral oncogene homolog	1.00736	1.000914	0.9976366
3816	KLK1	kallikrein 1	0.99885	1.02101	0.9945859
3817	KLK2	kallikrein-related peptidase 2	1.01404	0.99847	1.0040317
3818	KLKB1	kallikrein B, plasma (Fletcher factor) 1	1.00311	0.988521	0.9884083
3820	KLRB1	killer cell lectin-like receptor subfamily B, member 1	0.89903	1.030716	0.9794131
3823	KLRC3	killer cell lectin-like receptor subfamily C, member 3	0.98399	1.093847	1.0364989
3824	KLRD1	killer cell lectin-like receptor subfamily D, member 1	1.01713	1.075161	0.9077746
3827	KNG1	kininogen 1	1.01215	1.011851	1.0176418
3831	KLC1	kinesin light chain 1	0.96213	1.057485	1.0213955
3832	KIF11	kinesin family member 11	0.9805	0.958492	1.0203317

3833	KIFC1	kinesin family member C1	1.00648	1.004063	1.0147708
3834	KIF25	kinesin family member 25	1.01872	1.015343	1.0136245
3835	KIF22	kinesin family member 22	0.96651	1.062627	1.0795634
3836	KPNA1	karyopherin alpha 1 (importin alpha 5)	1.00038	0.965788	0.9120202
3837	KPNB1	karyopherin (importin) beta 1	0.96821	1.01913	1.0577629
3838	KPNA2	karyopherin alpha 2 (RAG cohort 1, importin alpha 1)	0.9483	0.98769	1.0429279
3839	KPNA3	karyopherin alpha 3 (importin alpha 4)	0.95866	0.951428	1.0599616
3840	KPNA4	karyopherin alpha 4 (importin alpha 3)	1.00844	0.910345	0.9691662
3841	KPNA5	karyopherin alpha 5 (importin alpha 6)	0.94802	0.928615	0.8931817
3842	TNPO1	transportin 1	1.00797	0.979499	1.0758751
3843	IPO5	importin 5	0.9404	0.965276	1.0753907
3845	KRAS	v-Ki-ras2 Kirsten rat sarcoma viral oncogene homolog	0.96821	0.973639	1.0253887
3848	KRT1	keratin 1	1.02038	0.982856	0.9546907
3849	KRT2	keratin 2	0.99247	1.000977	1.0063735
3850	KRT3	keratin 3	0.99983	0.997977	0.9974298
3851	KRT4	keratin 4	1.00457	1.008616	0.971904
3852	KRT5	keratin 5	1.00893	1.008897	0.9896324
3853	KRT6A	keratin 6A	1	0.973052	0.9866895
3854	KRT6B	keratin 6B	1.00654	0.99553	1.0047521
3855	KRT7	keratin 7	1.00562	0.98833	1.0092822
3856	KRT8	keratin 8	0.98902	0.990232	0.9938607
3857	KRT9	keratin 9	1.0143	1.005571	1.0201867
3858	KRT10	keratin 10	0.99494	1.003974	1.0189824
3859	KRT12	keratin 12	0.98822	0.977816	0.9826168
3860	KRT13	keratin 13	1.01422	0.981427	1.0070961
3861	KRT14	keratin 14	1.00121	0.97612	0.983355
3866	KRT15	keratin 15	0.99883	0.981326	1.0066254
3868	KRT16	keratin 16	0.99717	1.015342	1.0039626
3872	KRT17	keratin 17	0.96301	0.985797	0.9798412
3875	KRT18	keratin 18	1.00948	1.035879	1.0156558
3879	KRT18P1	keratin 18 pseudogene 1	1.01169	0.97184	1.0306633
3880	KRT19	keratin 19	0.98924	0.971581	1.0383131
3881	KRT31	keratin 31	1.00866	0.953128	0.989201

3882	KRT32	keratin 32	0.98422	1.009736	0.9971966
3883	KRT33A	keratin 33A	0.99959	0.952455	0.9908425
3884	KRT33B	keratin 33B	1.00026	1.008244	0.9858183
3885	KRT34	keratin 34	0.9914	0.985921	1.032947
3886	KRT35	keratin 35	1.01194	1.014827	0.9631656
3887	KRT81	keratin 81	1.02081	0.954009	1.0281542
3888	KRT82	keratin 82	1.00246	0.978873	1.0283474
3889	KRT83	keratin 83	0.9974	1.026611	1.0031366
3890	KRT84	keratin 84	1.01424	0.978122	0.9825574
3891	KRT85	keratin 85	0.98533	0.997625	1.0012993
3892	KRT86	keratin 86	1.00916	0.919539	1.0346406
3895	KTN1	kinectin 1 (kinesin receptor)	0.93282	0.987111	1.047987
3897	L1CAM	L1 cell adhesion molecule	1.00496	1.012294	1.0198257
3898	LAD1	ladinin 1	1.01374	1.007085	1.0099405
3899	AFF3	AF4/FMR2 family, member 3	1.00995	1.005574	0.9977312
3902	LAG3	lymphocyte-activation gene 3	0.97652	1.010018	1.0487537
3903	LAIR1	leukocyte-associated immunoglobulin-like receptor 1	0.99676	1.021546	0.9512241
3904	LAIR2	leukocyte-associated immunoglobulin-like receptor 2	1.04992	1.020187	0.9819933
3906	LALBA	lactalbumin, alpha-	0.98276	1.012275	0.954418
3908	LAMA2	laminin, alpha 2	1.00827	0.995329	1.0111819
3909	LAMA3	laminin, alpha 3	1.00075	0.996309	1.001559
3910	LAMA4	laminin, alpha 4	1.00789	1.005233	1.0105393
3911	LAMA5	laminin, alpha 5	0.98816	0.996425	1.0483612
3913	LAMB2	laminin, beta 2 (laminin S)	1.00658	0.973435	0.9873842
3914	LAMB3	laminin, beta 3	1.00833	1.039308	0.9591028
3916	LAMP1	lysosomal-associated membrane protein 1	0.97003	0.951206	0.9956263
3918	LAMC2	laminin, gamma 2	1.00606	0.999819	0.9939285
3920	LAMP2	lysosomal-associated membrane protein 2	0.98225	0.962464	1.035826
3921	RPSA	ribosomal protein SA	1.01758	0.886032	0.9956041
3925	STMN1	stathmin 1	0.99123	0.954596	0.997984
3927	LASP1	LIM and SH3 protein 1	0.96395	0.978318	1.0062753
3929	LBP	lipopolysaccharide binding protein	1.01449	1.015347	0.9959912
3930	LBR	lamin B receptor	1.01703	1.023022	1.0366503

3931	LCAT	lecithin-cholesterol acyltransferase	1.00299	0.999552	1.0036853
3932	LCK	lymphocyte-specific protein tyrosine kinase	0.9274	1.103512	1.0512293
3933	LCN1	lipocalin 1 (tear prealbumin)	0.9827	1.007158	1.0534913
3934	LCN2	lipocalin 2	0.97818	1.01236	1.0191746
3936	LCP1	lymphocyte cytosolic protein 1 (L-plastin)	1.01037	0.9298	1.0328431
3937	LCP2	lymphocyte cytosolic protein 2 (SH2 domain containing leukocyte protein of 76kDa)	0.98185	0.97443	0.9613631
3938	LCT	lactase	1.01483	1.003054	1.0002808
3939	LDHA	lactate dehydrogenase A	0.97979	1.058599	1.0600967
3940	LDHAP1	lactate dehydrogenase A pseudogene 1	1.04443	0.978006	1.0204252
3945	LDHB	lactate dehydrogenase B	0.92698	0.941422	0.9844765
3947	LDHBP2	lactate dehydrogenase B pseudogene 2	0.95682	0.963306	1.0668495
3949	LDLR	low density lipoprotein receptor	0.96168	0.956423	1.0871951
3950	LECT2	leukocyte cell-derived chemotaxin 2	1.00144	0.992882	1.0420961
3952	LEP	leptin	1.01009	0.998124	1.0112726
3953	LEPR	leptin receptor	0.96062	0.95471	0.9826182
3954	LETM1	leucine zipper-EF-hand containing transmembrane protein 1	1.00084	0.983439	1.054079
3955	LFNG	LFNG O-fucosylpeptide 3-beta-N-acetylglucosaminyltransferase	0.99749	1.135301	0.939869
3956	LGALS1	lectin, galactoside-binding, soluble, 1	0.97742	1.023052	1.0462065
3957	LGALS2	lectin, galactoside-binding, soluble, 2	0.88635	0.825063	0.8867757
3958	LGALS3	lectin, galactoside-binding, soluble, 3	1.00081	0.968636	1.0455639
3959	LGALS3BP	lectin, galactoside-binding, soluble, 3 binding protein	0.94931	0.887979	1.0141268
3960	LGALS4	lectin, galactoside-binding, soluble, 4	1.00304	1.003791	0.9670185
3964	LGALS8	lectin, galactoside-binding, soluble, 8	1.01851	1.021993	1.0954497
3965	LGALS9	lectin, galactoside-binding, soluble, 9	1.0022	0.874062	1.0791999
3972	LHB	luteinizing hormone beta polypeptide	1.01703	1.000603	1.0310051
3973	LHCGR	luteinizing hormone/choriogonadotropin receptor	1.00691	1.011234	1.0331784
3975	LHX1	LIM homeobox 1	0.99778	0.998371	1.031031
3976	LIF	leukemia inhibitory factor (cholinergic differentiation factor)	1.00687	1.050781	0.9767729
3977	LIFR	leukemia inhibitory factor receptor alpha	1.00745	1.002514	1.0200184
3978	LIG1	ligase I, DNA, ATP-dependent	0.95628	0.975021	0.9996233
3980	LIG3	ligase III, DNA, ATP-dependent	0.97786	1.021091	1.0397048
3981	LIG4	ligase IV, DNA, ATP-dependent	0.94058	0.988921	0.9896085
3982	LIM2	lens intrinsic membrane protein 2, 19kDa	0.97337	0.989949	1.0188679

3983	ABLIM1	actin binding LIM protein 1	0.96206	1.014281	0.9696612
3984	LIMK1	LIM domain kinase 1	0.99698	1.000675	1.0689497
3985	LIMK2	LIM domain kinase 2	0.94506	1.079397	0.9728554
3987	LIMS1	LIM and senescent cell antigen-like domains 1	0.98083	1.059151	0.9698624
3988	LIPA	lipase A, lysosomal acid, cholesterol esterase	0.99263	0.862714	0.9494128
3990	LIPC	lipase, hepatic	0.98679	0.972151	1.0018063
3991	LIPE	lipase, hormone-sensitive	0.98217	0.998099	1.0272028
3992	FADS1	fatty acid desaturase 1	0.94684	1.03629	1.0836627
3993	LLGL2	lethal giant larvae homolog 2 (Drosophila)	0.98508	1.02557	1.0086772
3995	FADS3	fatty acid desaturase 3	0.98663	1.042549	1.0114804
3996	LLGL1	lethal giant larvae homolog 1 (Drosophila)	0.96094	0.930101	0.9692512
3998	LMAN1	lectin, mannose-binding, 1	0.92318	1.02127	1.0270065
4000	LMNA	lamin A/C	0.99407	0.980588	0.994672
4001	LMNB1	lamin B1	1.02046	0.942115	1.0390298
4004	LMO1	LIM domain only 1 (rhombotin 1)	1.00184	1.014975	1.0025879
4005	LMO2	LIM domain only 2 (rhombotin-like 1)	0.97661	0.9684	1.0514057
4007	PRICKLE3	prickle homolog 3 (Drosophila)	0.98653	1.01594	1.0156286
4008	LMO7	LIM domain 7	0.96726	0.941709	1.0158131
4009	LMX1A	LIM homeobox transcription factor 1, alpha	1.0084	0.994725	1.0056919
4010	LMX1B	LIM homeobox transcription factor 1, beta	0.98798	1.010223	0.9937186
4012	LNPEP	leucyl/cystinyl aminopeptidase	1.00773	0.942167	1.0832336
4013	VWA5A	von Willebrand factor A domain containing 5A	0.96668	0.903019	0.9693645
4014	LOR	loricrin	0.99576	1.055574	1.0363345
4015	LOX	lysyl oxidase	1.00009	1.020824	0.975985
4016	LOXL1	lysyl oxidase-like 1	0.97767	1.021249	1.0225828
4017	LOXL2	lysyl oxidase-like 2	0.99509	0.993995	0.9808036
4018	LPA	lipoprotein, Lp(a)	1.01765	1.017751	1.0247475
4023	LPL	lipoprotein lipase	0.9975	0.976352	1.0050626
4025	LPO	lactoperoxidase	0.99638	0.970428	0.9903888
4026	LPP	LIM domain containing preferred translocation partner in lipoma	1.00897	0.89365	0.9826136
4033	LRMP	lymphoid-restricted membrane protein	0.95962	1.115397	0.9694777
4034	LRCH4	leucine-rich repeats and calponin homology (CH) domain containing 4	0.98926	1.020846	0.9429699
4035	LRP1	low density lipoprotein receptor-related protein 1	1.00774	0.938501	0.9940382

4036	LRP2	low density lipoprotein receptor-related protein 2	1.01102	0.989688	1.0030174
4037	LRP3	low density lipoprotein receptor-related protein 3	0.98481	0.951441	1.0389415
4038	LRP4	low density lipoprotein receptor-related protein 4	1.00492	0.99695	1.0145354
4040	LRP6	low density lipoprotein receptor-related protein 6	0.95822	0.928372	0.9869745
4041	LRP5	low density lipoprotein receptor-related protein 5	0.98911	1.003904	1.0067564
4043	LRPAP1	low density lipoprotein receptor-related protein associated protein 1	0.98872	0.99231	1.0189213
4045	LSAMP	limbic system-associated membrane protein	1.00622	1.013642	1.0144034
4046	LSP1	lymphocyte-specific protein 1	0.98184	1.026314	0.9606894
4047	LSS	lanosterol synthase (2,3-oxidosqualene-lanosterol cyclase)	0.97467	0.985634	0.9967435
4048	LTA4H	leukotriene A4 hydrolase	0.99344	0.977835	1.0239912
4049	LTA	lymphotoxin alpha (TNF superfamily, member 1)	0.97217	1.025964	0.9865772
4050	LTB	lymphotoxin beta (TNF superfamily, member 3)	0.94139	0.958517	0.927786
4051	CYP4F3	cytochrome P450, family 4, subfamily F, polypeptide 3	0.99477	0.97105	0.9585156
4052	LTBP1	latent transforming growth factor beta binding protein 1	1.02408	0.972852	1.0064662
4053	LTBP2	latent transforming growth factor beta binding protein 2	0.99245	1.012113	0.9979402
4054	LTBP3	latent transforming growth factor beta binding protein 3	0.9231	0.955538	0.9309634
4055	LTBR	lymphotoxin beta receptor (TNFR superfamily, member 3)	0.96321	0.974376	1.0539339
4056	LTC4S	leukotriene C4 synthase	0.98265	1.033339	1.0962529
4057	LTF	lactotransferrin	1.00269	0.914625	1.0713216
4058	LTK	leukocyte receptor tyrosine kinase	0.98364	1.035073	1.0042513
4059	BCAM	basal cell adhesion molecule (Lutheran blood group)	0.9978	0.978239	1.0346272
4060	LUM	lumican	1.00087	1.01388	1.0082849
4061	LY6E	lymphocyte antigen 6 complex, locus E	0.96616	0.917756	1.1254899
4062	LY6H	lymphocyte antigen 6 complex, locus H	0.99297	1.024803	1.0552152
4063	LY9	lymphocyte antigen 9	0.96007	1.067566	0.9569611
4064	CD180	CD180 molecule	0.96198	0.9683	0.8721805
4065	LY75	lymphocyte antigen 75	0.98257	0.995713	1.0340775
4066	LYL1	lymphoblastic leukemia derived sequence 1	0.95643	0.982177	1.0687169
4067	LYN	v-yes-1 Yamaguchi sarcoma viral related oncogene homolog	1.03722	1.134983	1.1173791
4068	SH2D1A	SH2 domain containing 1A	0.92533	1.103749	0.9807339
4069	LYZ	lysozyme	0.99842	1.019577	1.0204482
4070	TACSTD2	tumor-associated calcium signal transducer 2	0.9876	0.950067	1.0256544
4071	TM4SF1	transmembrane 4 L six family member 1	1.03753	0.983257	1.046415

4072	EPCAM	epithelial cell adhesion molecule	1.00603	1.01935	1.0277778
4074	M6PR	mannose-6-phosphate receptor (cation dependent)	0.98427	0.930141	1.1477699
4076	CAPRIN1	cell cycle associated protein 1	0.96988	1.04562	1.1003019
4077	NBR1	neighbor of BRCA1 gene 1	0.91605	0.931266	0.9479695
4081	MAB21L1	mab-21-like 1 (C. elegans)	0.98809	0.993242	1.0263135
4082	MARCKS	myristoylated alanine-rich protein kinase C substrate	0.95017	1.162846	1.1399993
4084	MXD1	MAX dimerization protein 1	1.02984	1.00057	0.9888945
4085	MAD2L1	MAD2 mitotic arrest deficient-like 1 (yeast)	1.03068	0.933709	0.9907287
4086	SMAD1	SMAD family member 1	1.0164	0.976983	1.0275453
4087	SMAD2	SMAD family member 2	0.96775	1.105505	1.0483773
4088	SMAD3	SMAD family member 3	0.976	1.122614	0.9378185
4089	SMAD4	SMAD family member 4	0.91745	0.96871	0.9505536
4090	SMAD5	SMAD family member 5	0.99246	0.960466	1.07644
4091	SMAD6	SMAD family member 6	0.98078	0.966317	0.9993211
4092	SMAD7	SMAD family member 7	0.97197	1.064141	0.9800402
4093	SMAD9	SMAD family member 9	0.97711	1.009371	0.9991502
4094	MAF	v-maf musculoaponeurotic fibrosarcoma oncogene homolog (avian)	0.9599	1.032668	0.9897448
4097	MAFG	v-maf musculoaponeurotic fibrosarcoma oncogene homolog G (avian)	0.97868	0.95886	1.004851
4099	MAG	myelin associated glycoprotein	0.97603	1.005306	1.0449277
4100	MAGEA1	melanoma antigen family A, 1 (directs expression of antigen MZ2-E)	1.02535	0.974674	0.9961363
4103	MAGEA4	melanoma antigen family A, 4	0.98662	1.006869	1.0261074
4104	MAGEA5	melanoma antigen family A, 5	0.99959	1.034947	1.0571876
4106	MAGEA7P	melanoma antigen family A, 7, pseudogene	0.98791	1.029569	0.9835115
4107	MAGEA8	melanoma antigen family A, 8	1.0032	0.988292	1.0191329
4109	MAGEA10	melanoma antigen family A, 10	1.00853	1.01601	0.9965327
4111	MAGEA12	melanoma antigen family A, 12	0.98427	0.989149	0.9483556
4112	MAGEB1	melanoma antigen family B, 1	0.98202	0.997775	1.0104009
4113	MAGEB2	melanoma antigen family B, 2	0.9863	1.029152	1.0044689
4114	MAGEB3	melanoma antigen family B, 3	1.02362	1.025822	0.9893622
4115	MAGEB4	melanoma antigen family B, 4	1.01527	0.981378	1.0579607
4116	MAGOH	mago-nashi homolog, proliferation-associated (Drosophila)	0.99538	1.085298	0.9473297
4117	MAK	male germ cell-associated kinase	0.99603	1.027688	0.981306
4118	MAL	mal, T-cell differentiation protein	0.97115	0.902739	0.9478066

4121	MAN1A1	mannosidase, alpha, class 1A, member 1	1.00571	0.942705	1.0078404
4122	MAN2A2	mannosidase, alpha, class 2A, member 2	0.97886	1.024672	0.9780239
4123	MAN2C1	mannosidase, alpha, class 2C, member 1	0.96772	0.973915	1.0373825
4124	MAN2A1	mannosidase, alpha, class 2A, member 1	1.02281	0.928549	0.9530288
4125	MAN2B1	mannosidase, alpha, class 2B, member 1	0.97476	0.906254	1.0520859
4126	MANBA	mannosidase, beta A, lysosomal	1.00747	1.049225	0.9755579
4128	MAOA	monoamine oxidase A	1.00055	0.991995	1.0078107
4129	MAOB	monoamine oxidase B	0.98661	0.992072	0.9795251
4130	MAP1A	microtubule-associated protein 1A	0.95652	0.929469	0.977368
4131	MAP1B	microtubule-associated protein 1B	1.01517	0.994469	1.0149986
4133	MAP2	microtubule-associated protein 2	1.00699	0.995392	1.013161
4134	MAP4	microtubule-associated protein 4	0.9973	1.027088	1.0340431
4135	MAP6	microtubule-associated protein 6	1.0016	1.005811	1.006771
4137	MAPT	microtubule-associated protein tau	0.99504	0.971871	0.9923699
4139	MARK1	MAP/microtubule affinity-regulating kinase 1	0.99488	0.993089	1.0223025
4140	MARK3	MAP/microtubule affinity-regulating kinase 3	0.95733	1.107617	1.0393845
4141	MARS	methionyl-tRNA synthetase	0.98243	1.073314	1.0460647
4142	MAS1	MAS1 oncogene	0.99543	1.008288	0.974998
4143	MAT1A	methionine adenosyltransferase I, alpha	0.99764	0.984056	0.9991752
4144	MAT2A	methionine adenosyltransferase II, alpha	1.03714	0.873391	1.0268485
4145	MATK	megakaryocyte-associated tyrosine kinase	0.96718	1.032753	0.9679225
4146	MATN1	matrilin 1, cartilage matrix protein	1.00273	1.008655	0.9782039
4147	MATN2	matrilin 2	1.00076	1.012273	1.0202224
4148	MATN3	matrilin 3	0.99303	1.031292	1.0123736
4149	MAX	MYC associated factor X	0.98351	0.974608	0.9537299
4150	MAZ	MYC-associated zinc finger protein (purine-binding transcription factor)	0.94143	1.008551	1.1283262
4151	MB	myoglobin	1.0017	1.02843	1.01491
4152	MBD1	methyl-CpG binding domain protein 1	0.94019	1.011311	1.0657512
4153	MBL2	mannose-binding lectin (protein C) 2, soluble	1.03389	1.038554	0.9920806
4154	MBNL1	muscleblind-like (Drosophila)	1.00436	1.062455	1.0722468
4155	MBP	myelin basic protein	0.99737	0.994568	0.9066521
4157	MC1R	melanocortin 1 receptor (alpha melanocyte stimulating hormone receptor)	1.02063	0.967473	1.0005219
4158	MC2R	melanocortin 2 receptor (adrenocorticotropic hormone)	0.99379	1.034745	0.9928397

4159	MC3R	melanocortin 3 receptor	1.04663	1.000762	1.0012193
4160	MC4R	melanocortin 4 receptor	0.99775	1.096905	1.0214957
4161	MC5R	melanocortin 5 receptor	1.00312	1.033832	1.0388616
4162	MCAM	melanoma cell adhesion molecule	0.99525	1.02802	1.0060923
4163	MCC	mutated in colorectal cancers	1.01419	0.988019	1.0148966
4166	CHST6	carbohydrate (N-acetylglucosamine 6-O) sulfotransferase 6	0.98877	0.969655	0.9683731
4168	MCF2	MCF.2 cell line derived transforming sequence	0.98947	0.980694	0.9836022
4170	MCL1	myeloid cell leukemia sequence 1 (BCL2-related)	1.0136	0.912568	0.9956708
4171	MCM2	minichromosome maintenance complex component 2	0.98554	0.917826	1.0401154
4172	MCM3	minichromosome maintenance complex component 3	0.98387	0.986128	1.037904
4173	MCM4	minichromosome maintenance complex component 4	1.02369	0.877668	1.0451932
4174	MCM5	minichromosome maintenance complex component 5	0.94755	0.95421	1.1066572
4175	MCM6	minichromosome maintenance complex component 6	0.98213	1.017689	1.0294567
4176	MCM7	minichromosome maintenance complex component 7	0.99867	1.054028	1.0348861
4179	CD46	CD46 molecule, complement regulatory protein	1.01709	1.017536	1.0542636
4185	ADAM11	ADAM metallopeptidase domain 11	0.9943	1.000462	1.0202034
4188	MDFI	MyoD family inhibitor	1.00751	1.006963	1.0117616
4189	DNAJB9	DnaJ (Hsp40) homolog, subfamily B, member 9	0.98653	0.967193	1.0013912
4190	MDH1	malate dehydrogenase 1, NAD (soluble)	0.97357	0.916486	1.0377402
4191	MDH2	malate dehydrogenase 2, NAD (mitochondrial)	0.98431	0.867749	1.0695172
4192	MDK	midkine (neurite growth-promoting factor 2)	0.99175	0.978695	1.0251027
4193	MDM2	Mdm2 p53 binding protein homolog (mouse)	0.94252	0.9909	0.9878399
4194	MDM4	Mdm4 p53 binding protein homolog (mouse)	1.00296	1.080288	0.9163314
4197	[No Symbo	[No Name]	1.00028	1.00348	1.0116871
4199	ME1	malic enzyme 1, NADP(+)-dependent, cytosolic	0.99748	1.035329	1.0153677
4200	ME2	malic enzyme 2, NAD(+)-dependent, mitochondrial	0.98301	0.967767	0.9901946
4201	MEA1	male-enhanced antigen 1	0.93274	0.922383	1.0682556
4204	MECP2	methyl CpG binding protein 2 (Rett syndrome)	1.03036	1.14667	0.9534773
4205	MEF2A	myocyte enhancer factor 2A	1.00677	0.993788	0.9990724
4207	MEF2BNB-I	MEF2BNB-MEF2B readthrough	1.00512	0.989218	1.0426318
4208	MEF2C	myocyte enhancer factor 2C	1.01456	0.952954	1.014089
4209	MEF2D	myocyte enhancer factor 2D	1.00224	0.963347	0.9096112
4210	MEFV	Mediterranean fever	0.96277	0.932057	0.9568117

4211	MEIS1	Meis homeobox 1	1.00095	0.981959	0.999407
4212	MEIS2	Meis homeobox 2	1.00166	0.998866	1.0049233
4214	MAP3K1	mitogen-activated protein kinase kinase kinase 1	1.02893	0.957155	1.0381652
4215	MAP3K3	mitogen-activated protein kinase kinase kinase 3	0.9682	0.962483	0.9984055
4216	MAP3K4	mitogen-activated protein kinase kinase kinase 4	0.97154	1.034846	1.1057042
4217	MAP3K5	mitogen-activated protein kinase kinase kinase 5	0.97634	0.995402	0.990089
4218	RAB8A	RAB8A, member RAS oncogene family	0.96197	0.839553	1.1041433
4221	MEN1	multiple endocrine neoplasia I	0.93331	1.028687	0.9974079
4222	MEOX1	mesenchyme homeobox 1	0.96351	0.983385	1.0500971
4223	MEOX2	mesenchyme homeobox 2	1.00225	1.023682	1.0409325
4224	MEP1A	meprin A, alpha (PABA peptide hydrolase)	0.99758	0.98177	0.9949623
4225	MEP1B	meprin A, beta	0.98207	1.000184	0.9888293
4232	MEST	mesoderm specific transcript homolog (mouse)	0.99431	0.980806	1.0100575
4233	MET	met proto-oncogene (hepatocyte growth factor receptor)	0.99706	1.0167	0.9516774
4234	METTL1	methyltransferase like 1	0.97156	0.984247	1.0163046
4236	MFAP1	microfibrillar-associated protein 1	0.9239	0.935332	1.0824674
4237	MFAP2	microfibrillar-associated protein 2	1.02568	1.038556	1.0064105
4238	MFAP3	microfibrillar-associated protein 3	0.95191	1.000663	1.0143038
4240	MFGE8	milk fat globule-EGF factor 8 protein	0.98767	0.913429	0.949642
4241	MFI2	antigen p97 (melanoma associated) identified by monoclonal antibodies 133.2 and 96.5	1.00839	1.024775	1.0292377
4242	MFNG	MFNG O-fucosylpeptide 3-beta-N-acetylglucosaminyltransferase	0.96781	0.924178	1.1723645
4246	SCGB2A1	secretoglobin, family 2A, member 1	1.0085	0.98226	1.0185289
4247	MGAT2	mannosyl (alpha-1,6-)-glycoprotein beta-1,2-N-acetylglucosaminyltransferase	0.93169	1.088452	1.0474677
4248	MGAT3	mannosyl (beta-1,4-)-glycoprotein beta-1,4-N-acetylglucosaminyltransferase	0.98797	0.977157	1.0471698
4249	MGAT5	mannosyl (alpha-1,6-)-glycoprotein beta-1,6-N-acetyl-glucosaminyltransferase	0.9989	1.020744	0.9657253
4250	SCGB2A2	secretoglobin, family 2A, member 2	0.99642	1.020988	0.9888645
4253	CTAGE5	CTAGE family, member 5	0.95405	1.044968	1.0557076
4254	KITLG	KIT ligand	0.97738	1.016506	0.9480703
4255	MGMT	O-6-methylguanine-DNA methyltransferase	0.99538	0.965674	1.0302948
4256	MGP	matrix Gla protein	0.98834	1.010661	0.9741514
4257	MGST1	microsomal glutathione S-transferase 1	0.9579	0.925096	1.0669201
4258	MGST2	microsomal glutathione S-transferase 2	1.009	0.976349	1.0984122
4259	MGST3	microsomal glutathione S-transferase 3	1.02349	0.939766	1.0507024

4261	CIITA	class II, major histocompatibility complex, transactivator	0.95279	1.011755	1.0584813
4277	MICB	MHC class I polypeptide-related sequence B	0.98877	0.966953	1.128068
4279	MICD	MHC class I polypeptide-related sequence D (pseudogene)	1.01864	1.059422	0.9233424
4281	MID1	midline 1 (Opitz/BBB syndrome)	1.00122	0.980944	1.0110537
4282	MIF	macrophage migration inhibitory factor (glycosylation-inhibiting factor)	0.92329	0.933747	1.1397541
4283	CXCL9	chemokine (C-X-C motif) ligand 9	1.01379	0.955363	0.9915059
4284	MIP	major intrinsic protein of lens fiber	1.00511	0.960491	0.9981433
4285	MIPEP	mitochondrial intermediate peptidase	0.9878	0.965998	1.0396449
4286	MITF	microphthalmia-associated transcription factor	1.00485	0.974023	0.9953068
4287	ATXN3	ataxin 3	0.94917	0.996716	1.0575194
4288	MKI67	antigen identified by monoclonal antibody Ki-67	0.9838	0.922902	1.0461008
4289	MKLN1	muskelin 1, intracellular mediator containing kelch motifs	1.02329	0.972005	1.0073833
4291	MLF1	myeloid leukemia factor 1	0.99806	0.976292	1.0216971
4292	MLH1	mutL homolog 1, colon cancer, nonpolyposis type 2 (E. coli)	1.0181	0.933764	0.9654963
4293	MAP3K9	mitogen-activated protein kinase kinase kinase 9	0.99144	0.996495	0.9785268
4294	MAP3K10	mitogen-activated protein kinase kinase kinase 10	0.98667	1.042154	0.9707642
4295	MLN	motilin	1.00996	1.022901	1.0323437
4296	MAP3K11	mitogen-activated protein kinase kinase kinase 11	0.9781	0.955824	0.9345105
4297	MLL	myeloid/lymphoid or mixed-lineage leukemia (trithorax homolog, Drosophila)	0.96084	1.001441	1.0061432
4298	MLLT1	myeloid/lymphoid or mixed-lineage leukemia (trithorax homolog, Drosophila); translocated to, 1	0.97833	0.994843	1.0263648
4299	AFF1	AF4/FMR2 family, member 1	1.00452	0.950153	1.1063637
4300	MLLT3	myeloid/lymphoid or mixed-lineage leukemia (trithorax homolog, Drosophila); translocated to, 3	0.95678	0.96673	1.0131905
4301	MLLT4	myeloid/lymphoid or mixed-lineage leukemia (trithorax homolog, Drosophila); translocated to, 4	0.99506	0.987226	0.9674843
4302	MLLT6	myeloid/lymphoid or mixed-lineage leukemia (trithorax homolog, Drosophila); translocated to, 6	0.95573	1.015366	0.890562
4303	FOXO4	forkhead box O4	0.94036	0.916716	1.0111038
4306	NR3C2	nuclear receptor subfamily 3, group C, member 2	0.98406	0.954487	0.9538535
4308	TRPM1	transient receptor potential cation channel, subfamily M, member 1	0.99051	1.008382	1.0138902
4311	MME	membrane metallo-endopeptidase	1.01922	0.912029	0.9564798
4312	MMP1	matrix metallopeptidase 1 (interstitial collagenase)	0.93952	0.997471	1.0057099
4313	MMP2	matrix metallopeptidase 2 (gelatinase A, 72kDa gelatinase, 72kDa type IV collagenase)	1.00088	1.01672	1.0239483
4314	MMP3	matrix metallopeptidase 3 (stromelysin 1, progelatinase)	1.00036	1.0337	0.9865615
4316	MMP7	matrix metallopeptidase 7 (matrilysin, uterine)	0.97618	1.021703	0.9982355
4317	MMP8	matrix metallopeptidase 8 (neutrophil collagenase)	1.00647	0.932918	1.0556851

4318	MMP9	matrix metallopeptidase 9 (gelatinase B, 92kDa gelatinase, 92kDa type IV collagenase)	0.96971	0.943768	0.9815313
4319	MMP10	matrix metallopeptidase 10 (stromelysin 2)	1.00179	1.015316	1.0171134
4320	MMP11	matrix metallopeptidase 11 (stromelysin 3)	0.99926	0.951468	1.0370015
4321	MMP12	matrix metallopeptidase 12 (macrophage elastase)	0.9832	1.023336	1.0180787
4322	MMP13	matrix metallopeptidase 13 (collagenase 3)	0.99087	1.031353	1.0160886
4323	MMP14	matrix metallopeptidase 14 (membrane-inserted)	0.98629	0.959113	0.9958088
4324	MMP15	matrix metallopeptidase 15 (membrane-inserted)	0.98427	1.028062	1.0228932
4325	MMP16	matrix metallopeptidase 16 (membrane-inserted)	0.99881	1.002726	1.0021723
4326	MMP17	matrix metallopeptidase 17 (membrane-inserted)	0.99142	0.983018	1.0707926
4327	MMP19	matrix metallopeptidase 19	0.98998	0.992162	0.997347
4329	ALDH6A1	aldehyde dehydrogenase 6 family, member A1	0.91321	0.922146	0.9839384
4330	MN1	meningioma (disrupted in balanced translocation) 1	0.98744	1.028	0.9614144
4331	MNAT1	menage a trois homolog 1, cyclin H assembly factor (Xenopus laevis)	0.97733	1.008753	1.0122418
4332	MNDA	myeloid cell nuclear differentiation antigen	1.04641	0.85019	0.9519716
4335	MNT	MAX binding protein	1.00219	1.018323	0.9445644
4336	MOBP	myelin-associated oligodendrocyte basic protein	1.01277	0.969584	1.0355702
4337	MOCS1	molybdenum cofactor synthesis 1	0.98463	1.007456	1.022743
4338	MOCS2	molybdenum cofactor synthesis 2	0.98498	0.937597	0.963084
4340	MOG	myelin oligodendrocyte glycoprotein	1.00492	0.979844	1.0272087
4342	MOS	v-mos Moloney murine sarcoma viral oncogene homolog	1.02988	0.962136	1.1079741
4343	MOV10	Mov10, Moloney leukemia virus 10, homolog (mouse)	0.98206	0.938997	0.9774336
4350	MPG	N-methylpurine-DNA glycosylase	0.99437	0.995837	1.0460166
4351	MPI	mannose phosphate isomerase	0.93973	0.910879	1.0468478
4352	MPL	myeloproliferative leukemia virus oncogene	0.98962	0.934913	0.9818467
4353	MPO	myeloperoxidase	0.99755	0.956603	0.9854715
4354	MPP1	membrane protein, palmitoylated 1, 55kDa	0.95009	0.925847	1.0122719
4355	MPP2	membrane protein, palmitoylated 2 (MAGUK p55 subfamily member 2)	0.98592	1.026107	0.9818915
4356	MPP3	membrane protein, palmitoylated 3 (MAGUK p55 subfamily member 3)	0.99133	0.999065	1.0198675
4357	MPST	mercaptopyruvate sulfurtransferase	0.94928	0.970076	1.0024432
4358	MPV17	MpV17 mitochondrial inner membrane protein	0.99893	0.982331	1.0373294
4359	MPZ	myelin protein zero	0.99976	1.023388	0.9985851
4361	MRE11A	MRE11 meiotic recombination 11 homolog A (S. cerevisiae)	0.97452	1.044965	1.1158541
4362	MRE11B	MRE11 meiotic recombination 11 homolog B (S. cerevisiae)	0.98835	0.981132	1.0084814

4363	ABCC1	ATP-binding cassette, sub-family C (CFTR/MRP), member 1	0.98038	0.995931	1.0318976
4430	MYO1B	myosin IB	0.99741	1.018065	1.0514752
4435	CITED1	Cbp/p300-interacting transactivator, with Glu/Asp-rich carboxy-terminal domain, 1	1.00355	1.016738	0.9996401
4436	MSH2	mutS homolog 2, colon cancer, nonpolyposis type 1 (E. coli)	1.00796	1.003859	1.0092
4437	MSH3	mutS homolog 3 (E. coli)	0.99619	0.919108	1.0674133
4438	MSH4	mutS homolog 4 (E. coli)	0.99472	1.009807	1.0039385
4439	MSH5	mutS homolog 5 (E. coli)	1.0177	1.031033	1.0234515
4440	MSI1	musashi homolog 1 (Drosophila)	0.9788	1.018997	1.054907
4477	MSMB	microseminoprotein, beta-	0.99006	0.940721	0.9765666
4478	MSN	moesin	0.99961	1.039388	0.9666243
4479	MSNP1	moesin pseudogene 1	1.00094	0.857216	1.1582352
4481	MSR1	macrophage scavenger receptor 1	0.9633	1.08365	0.9304692
4482	MSRA	methionine sulfoxide reductase A	0.99514	0.950859	0.9770652
4485	MST1	macrophage stimulating 1 (hepatocyte growth factor-like)	0.96525	0.96769	1.0969668
4486	MST1R	macrophage stimulating 1 receptor (c-met-related tyrosine kinase)	1.00143	1.030946	1.0215039
4487	MSX1	msh homeobox 1	0.97296	1.032587	1.0420384
4488	MSX2	msh homeobox 2	0.99223	0.979973	1.0166729
4489	MT1A	metallothionein 1A	0.98695	1.020054	1.0027044
4490	MT1B	metallothionein 1B	1.00576	0.99827	1.0306247
4493	MT1E	metallothionein 1E	1.00302	1.038511	1.0346729
4494	MT1F	metallothionein 1F	0.94989	1.010813	1.0136148
4495	MT1G	metallothionein 1G	0.91999	1.011886	0.8092591
4496	MT1H	metallothionein 1H	1.0178	0.967773	0.9417949
4498	MT1JP	metallothionein 1J, pseudogene	0.95301	1.015352	0.9573653
4499	MT1M	metallothionein 1M	0.98176	1.032191	0.994995
4501	MT1X	metallothionein 1X	1.03868	1.179149	0.9010939
4502	MT2A	metallothionein 2A	0.98831	1.186378	0.9580327
4504	MT3	metallothionein 3	1.0171	1.011412	0.9742941
4507	MTAP	methylthioadenosine phosphorylase	0.94876	0.885402	1.0518
4515	MTCP1	mature T-cell proliferation 1	0.96912	0.946316	0.9878345
4520	MTF1	metal-regulatory transcription factor 1	1.02443	1.031461	0.9963728
4521	NUDT1	nudix (nucleoside diphosphate linked moiety X)-type motif 1	0.99665	1.027013	1.0391071
4522	MTHFD1	methylenetetrahydrofolate dehydrogenase (NADP+ dependent) 1, methenyltetrahydrofolate cyclohydrolase	0.96984	0.969699	1.0464568

4524	MTHFR	methylenetetrahydrofolate reductase (NAD(P)H)	0.99048	1.007872	0.9640046
4528	MTIF2	mitochondrial translational initiation factor 2	1.00665	0.850488	0.998609
4534	MTM1	myotubularin 1	0.95739	1.000551	0.9525925
4542	MYO1F	myosin IF	0.97907	1.000324	0.9699455
4543	MTNR1A	melatonin receptor 1A	1.0073	1.018359	0.9949324
4544	MTNR1B	melatonin receptor 1B	1.01095	1.009418	1.0301418
4547	MTTP	microsomal triglyceride transfer protein	1.01052	0.993192	1.0171835
4548	MTR	5-methyltetrahydrofolate-homocysteine methyltransferase	0.9627	0.906959	1.0377016
4552	MTRR	5-methyltetrahydrofolate-homocysteine methyltransferase reductase	0.98483	0.880152	1.0495921
4580	MTX1	metaxin 1	1.00194	1.033344	1.0127494
4582	MUC1	mucin 1, cell surface associated	1.02159	0.989144	0.9916098
4583	MUC2	mucin 2, oligomeric mucus/gel-forming	0.99992	0.975066	1.0362928
4584	MUC3A	mucin 3A, cell surface associated	1.02418	1.022974	1.0323698
4585	MUC4	mucin 4, cell surface associated	1.00823	1.002965	1.0290108
4588	MUC6	mucin 6, oligomeric mucus/gel-forming	0.98099	1.004196	1.0377474
4589	MUC7	mucin 7, secreted	0.99696	0.973874	0.9913641
4591	TRIM37	tripartite motif containing 37	0.94958	0.980205	1.0513662
4593	MUSK	muscle, skeletal, receptor tyrosine kinase	0.99297	1.021325	1.0139763
4594	MUT	methylmalonyl CoA mutase	0.99013	0.956535	1.0290858
4595	MUTYH	mutY homolog (E. coli)	0.9986	0.98939	0.9748594
4597	MVD	mevalonate (diphospho) decarboxylase	0.98035	0.996622	1.0261901
4598	MVK	mevalonate kinase	0.9846	0.964416	0.98807
4599	MX1	myxovirus (influenza virus) resistance 1, interferon-inducible protein p78 (mouse)	0.98035	0.858072	0.9999615
4600	MX2	myxovirus (influenza virus) resistance 2 (mouse)	0.98855	0.993517	1.0692289
4601	MXI1	MAX interactor 1	0.96789	0.958883	0.9532319
4602	MYB	v-myb myeloblastosis viral oncogene homolog (avian)	0.97526	1.002791	1.0216032
4603	MYBL1	v-myb myeloblastosis viral oncogene homolog (avian)-like 1	1.00291	1.047984	0.9551986
4604	MYBPC1	myosin binding protein C, slow type	0.99732	0.987058	0.9928739
4605	MYBL2	v-myb myeloblastosis viral oncogene homolog (avian)-like 2	1.00851	1.008589	1.0148624
4606	MYBPC2	myosin binding protein C, fast type	0.98347	1.002284	1.0045815
4607	MYBPC3	myosin binding protein C, cardiac	0.98272	0.983774	0.9805372
4608	MYBPH	myosin binding protein H	0.99438	1.005433	1.002581
4609	MYC	v-myc myelocytomatosis viral oncogene homolog (avian)	0.93749	0.906633	1.022585

4611	MYCL2	v-myc myelocytomatosis viral oncogene homolog 2 (avian)	1.00334	1.072433	0.9863867
4613	MYCN	v-myc myelocytomatosis viral related oncogene, neuroblastoma derived (avian)	0.98733	0.981907	1.0315656
4615	MYD88	myeloid differentiation primary response gene (88)	1.02663	0.894568	1.0529973
4616	GADD45B	growth arrest and DNA-damage-inducible, beta	0.94926	0.938249	1.0637546
4617	MYF5	myogenic factor 5	0.96664	1.019062	1.0155214
4618	MYF6	myogenic factor 6 (herculin)	0.98223	0.966514	1.0024144
4620	MYH2	myosin, heavy chain 2, skeletal muscle, adult	0.98886	0.993112	1.0059469
4621	MYH3	myosin, heavy chain 3, skeletal muscle, embryonic	0.98516	1.00112	1.0184824
4622	MYH4	myosin, heavy chain 4, skeletal muscle	0.99383	0.996827	1.0018827
4624	MYH6	myosin, heavy chain 6, cardiac muscle, alpha	0.99743	0.97612	1.0204044
4625	MYH7	myosin, heavy chain 7, cardiac muscle, beta	1.00081	0.980898	0.9990334
4626	MYH8	myosin, heavy chain 8, skeletal muscle, perinatal	0.99704	1.006378	1.0212084
4627	MYH9	myosin, heavy chain 9, non-muscle	0.98247	0.951156	0.9666525
4628	MYH10	myosin, heavy chain 10, non-muscle	0.99385	0.982158	1.0034619
4632	MYL1	myosin, light chain 1, alkali; skeletal, fast	1.01965	0.989481	1.0262883
4633	MYL2	myosin, light chain 2, regulatory, cardiac, slow	0.99523	1.003725	0.9871599
4634	MYL3	myosin, light chain 3, alkali; ventricular, skeletal, slow	1.03173	1.001052	0.9814681
4635	MYL4	myosin, light chain 4, alkali; atrial, embryonic	1.01047	0.969923	1.0037362
4636	MYL5	myosin, light chain 5, regulatory	1.00802	0.997778	1.0259251
4637	MYL6	myosin, light chain 6, alkali, smooth muscle and non-muscle	1.01246	0.951237	0.9264546
4638	MYLK	myosin light chain kinase	0.99771	0.972689	0.976773
4640	MYO1A	myosin IA	0.99608	0.989272	0.9932672
4641	MYO1C	myosin IC	0.95727	1.051788	1.0346652
4642	MYO1D	myosin ID	0.9942	1.012536	1.0091223
4643	MYO1E	myosin IE	0.97714	1.007578	1.0041962
4644	MYO5A	myosin VA (heavy chain 12, myoxin)	0.9459	0.923138	1.0628972
4645	MYO5B	myosin VB	0.98975	0.990176	1.0023242
4646	MYO6	myosin VI	0.99702	1.010347	0.9978028
4648	MYO7B	myosin VIIB	1.01028	0.976983	1.0384452
4649	MYO9A	myosin IXA	0.95953	0.963922	1.0080891
4650	MYO9B	myosin IXB	0.96841	1.006284	0.9630658
4651	MYO10	myosin X	1.00787	1.000093	1.0111453
4653	MYOC	myocilin, trabecular meshwork inducible glucocorticoid response	0.99756	0.99431	0.9965522

4654	MYOD1	myogenic differentiation 1	0.98626	0.994388	1.0624587
4656	MYOG	myogenin (myogenic factor 4)	1.02319	1.066089	1.017387
4659	PPP1R12A	protein phosphatase 1, regulatory (inhibitor) subunit 12A	0.95198	1.034422	1.0702259
4660	PPP1R12B	protein phosphatase 1, regulatory (inhibitor) subunit 12B	1.01656	1.011006	0.9677117
4661	MYT1	myelin transcription factor 1	1.00096	0.986434	0.9960453
4664	NAB1	NGFI-A binding protein 1 (EGR1 binding protein 1)	0.99506	1.005595	0.9888328
4665	NAB2	NGFI-A binding protein 2 (EGR1 binding protein 2)	0.98164	0.980833	1.0487991
4666	NACA	nascent polypeptide-associated complex alpha subunit	1.01461	0.991248	0.9786554
4668	NAGA	N-acetylgalactosaminidase, alpha-	1.00407	0.827515	1.083402
4669	NAGLU	N-acetylglucosaminidase, alpha	0.98171	0.978621	1.0598777
4670	HNRNPM	heterogeneous nuclear ribonucleoprotein M	1.04189	1.041623	0.9095889
4671	NAIP	NLR family, apoptosis inhibitory protein	0.95213	0.958534	1.0611378
4673	NAP1L1	nucleosome assembly protein 1-like 1	0.97657	0.898004	0.8426562
4674	NAP1L2	nucleosome assembly protein 1-like 2	0.9045	0.903815	0.9487293
4675	NAP1L3	nucleosome assembly protein 1-like 3	0.87459	0.859353	0.9162549
4676	NAP1L4	nucleosome assembly protein 1-like 4	0.93442	1.088737	0.9488645
4677	NARS	asparaginyl-tRNA synthetase	0.98989	0.939433	1.0209497
4678	NASP	nuclear autoantigenic sperm protein (histone-binding)	1.00299	0.996127	0.9958612
4680	CEACAM6	carcinoembryonic antigen-related cell adhesion molecule 6 (non-specific cross reacting antigen)	1.01074	1.031318	0.9955595
4681	NBL1	neuroblastoma, suppression of tumorigenicity 1	1.02564	0.982052	1.0052677
4682	NUBP1	nucleotide binding protein 1	0.95904	0.996235	1.010474
4683	NBN	nibrin	0.93451	0.994916	0.9621872
4684	NCAM1	neural cell adhesion molecule 1	1.00188	0.987404	0.9808963
4685	NCAM2	neural cell adhesion molecule 2	0.99517	1.010104	1.0048734
4686	NCBP1	nuclear cap binding protein subunit 1, 80kDa	0.95388	0.958605	1.0648347
4688	NCF2	neutrophil cytosolic factor 2	1.03066	0.954368	0.9333668
4689	NCF4	neutrophil cytosolic factor 4, 40kDa	0.94971	0.939632	1.0446629
4690	NCK1	NCK adaptor protein 1	0.99515	0.958527	0.9693337
4691	NCL	nucleolin	0.95361	0.943257	0.9931108
4692	NDN	necdin homolog (mouse)	1.0029	1.01022	1.0439655
4693	NDP	Norrie disease (pseudoglioma)	1.01546	1.022831	1.0132143
4694	NDUFA1	NADH dehydrogenase (ubiquinone) 1 alpha subcomplex, 1, 7.5kDa	0.9351	0.975962	0.9464599
4695	NDUFA2	NADH dehydrogenase (ubiquinone) 1 alpha subcomplex, 2, 8kDa	0.98065	0.931828	1.0439311

4696	NDUFA3	NADH dehydrogenase (ubiquinone) 1 alpha subcomplex, 3, 9kDa	1.00767	1.01971	0.9861618
4697	NDUFA4	NADH dehydrogenase (ubiquinone) 1 alpha subcomplex, 4, 9kDa	0.99666	0.933703	1.0152148
4698	NDUFA5	NADH dehydrogenase (ubiquinone) 1 alpha subcomplex, 5, 13kDa	0.98906	0.939409	0.9861943
4700	NDUFA6	NADH dehydrogenase (ubiquinone) 1 alpha subcomplex, 6, 14kDa	0.93922	0.921368	0.9766552
4701	NDUFA7	NADH dehydrogenase (ubiquinone) 1 alpha subcomplex, 7, 14.5kDa	0.94775	0.937548	1.0730484
4702	NDUFA8	NADH dehydrogenase (ubiquinone) 1 alpha subcomplex, 8, 19kDa	0.97933	0.937558	1.0470271
4703	NEB	nebulin	1.00562	0.995025	1.002166
4704	NDUFA9	NADH dehydrogenase (ubiquinone) 1 alpha subcomplex, 9, 39kDa	0.95839	0.984773	1.0950285
4705	NDUFA10	NADH dehydrogenase (ubiquinone) 1 alpha subcomplex, 10, 42kDa	0.98706	1.044374	0.9739649
4706	NDUFAB1	NADH dehydrogenase (ubiquinone) 1, alpha/beta subcomplex, 1, 8kDa	0.96419	1.024536	1.0316133
4707	NDUFB1	NADH dehydrogenase (ubiquinone) 1 beta subcomplex, 1, 7kDa	0.96482	0.953431	1.1016334
4708	NDUFB2	NADH dehydrogenase (ubiquinone) 1 beta subcomplex, 2, 8kDa	1.00423	0.972622	0.9798866
4709	NDUFB3	NADH dehydrogenase (ubiquinone) 1 beta subcomplex, 3, 12kDa	0.99459	0.975806	1.0437716
4710	NDUFB4	NADH dehydrogenase (ubiquinone) 1 beta subcomplex, 4, 15kDa	1.0343	0.944881	0.9600414
4711	NDUFB5	NADH dehydrogenase (ubiquinone) 1 beta subcomplex, 5, 16kDa	0.97909	0.814034	1.1231438
4712	NDUFB6	NADH dehydrogenase (ubiquinone) 1 beta subcomplex, 6, 17kDa	0.96564	0.989509	0.9992487
4713	NDUFB7	NADH dehydrogenase (ubiquinone) 1 beta subcomplex, 7, 18kDa	0.9733	0.97257	1.0590162
4714	NDUFB8	NADH dehydrogenase (ubiquinone) 1 beta subcomplex, 8, 19kDa	0.9612	0.950174	1.0911067
4715	NDUFB9	NADH dehydrogenase (ubiquinone) 1 beta subcomplex, 9, 22kDa	0.98939	0.889849	0.9607987
4716	NDUFB10	NADH dehydrogenase (ubiquinone) 1 beta subcomplex, 10, 22kDa	0.94541	1.018427	1.0612633
4717	NDUFC1	NADH dehydrogenase (ubiquinone) 1, subcomplex unknown, 1, 6kDa	0.95234	0.972234	1.0196449
4718	NDUFC2	NADH dehydrogenase (ubiquinone) 1, subcomplex unknown, 2, 14.5kDa	0.92607	0.937315	1.085758
4719	NDUFS1	NADH dehydrogenase (ubiquinone) Fe-S protein 1, 75kDa (NADH-coenzyme Q reductase)	0.99937	0.919117	1.0167612
4720	NDUFS2	NADH dehydrogenase (ubiquinone) Fe-S protein 2, 49kDa (NADH-coenzyme Q reductase)	0.98744	0.971825	1.0593546
4722	NDUFS3	NADH dehydrogenase (ubiquinone) Fe-S protein 3, 30kDa (NADH-coenzyme Q reductase)	0.9684	0.944138	1.0809841
4723	NDUFV1	NADH dehydrogenase (ubiquinone) flavoprotein 1, 51kDa	0.97603	0.964342	1.0926772
4724	NDUFS4	NADH dehydrogenase (ubiquinone) Fe-S protein 4, 18kDa (NADH-coenzyme Q reductase)	0.98835	0.920995	1.0593838
4725	NDUFS5	NADH dehydrogenase (ubiquinone) Fe-S protein 5, 15kDa (NADH-coenzyme Q reductase)	0.96608	0.888138	0.9990098
4726	NDUFS6	NADH dehydrogenase (ubiquinone) Fe-S protein 6, 13kDa (NADH-coenzyme Q reductase)	1.03938	0.930306	0.9761995
4728	NDUFS8	NADH dehydrogenase (ubiquinone) Fe-S protein 8, 23kDa (NADH-coenzyme Q reductase)	0.98516	1.018465	1.018936
4729	NDUFV2	NADH dehydrogenase (ubiquinone) flavoprotein 2, 24kDa	0.99527	1.04135	0.9473801
4731	NDUFV3	NADH dehydrogenase (ubiquinone) flavoprotein 3, 10kDa	0.96394	0.968779	1.0229018
4733	DRG1	developmentally regulated GTP binding protein 1	0.97721	0.954121	1.0526869

4734	NEDD4	neural precursor cell expressed, developmentally down-regulated 4	0.98061	0.96723	1.0145845
4735	2-Sep	septin 2	1.00633	1.072257	0.9959212
4736	RPL10A	ribosomal protein L10a	0.93405	1.011403	0.9571933
4738	NEDD8	neural precursor cell expressed, developmentally down-regulated 8	0.95381	0.978633	0.9910559
4739	NEDD9	neural precursor cell expressed, developmentally down-regulated 9	0.9933	1.064091	0.9783763
4741	NEFM	neurofilament, medium polypeptide	1.00748	0.982484	1.0195057
4744	NEFH	neurofilament, heavy polypeptide	1.00491	1.01129	1.0151838
4745	NELL1	NEL-like 1 (chicken)	1.00617	0.997915	0.9992462
4747	NEFL	neurofilament, light polypeptide	0.95345	0.926059	0.970282
4750	NEK1	NIMA (never in mitosis gene a)-related kinase 1	0.97826	1.015605	0.990991
4751	NEK2	NIMA (never in mitosis gene a)-related kinase 2	1.02055	0.964397	1.0260696
4752	NEK3	NIMA (never in mitosis gene a)-related kinase 3	0.96503	0.900106	1.1022767
4753	NELL2	NEL-like 2 (chicken)	0.92281	1.007534	0.9986812
4756	NEO1	neogenin 1	0.96161	0.937448	0.9471628
4758	NEU1	sialidase 1 (lysosomal sialidase)	0.96952	1.057859	1.0698162
4759	NEU2	sialidase 2 (cytosolic sialidase)	1.02542	0.986443	1.0593037
4760	NEUROD1	neurogenic differentiation 1	0.973	0.991505	1.0362106
4761	NEUROD2	neurogenic differentiation 2	0.98218	0.982368	1.0002423
4762	NEUROG1	neurogenin 1	0.98857	1.009235	1.0297685
4763	NF1	neurofibromin 1	0.95239	1.031259	0.9642475
4771	NF2	neurofibromin 2 (merlin)	0.97097	0.934936	1.0488369
4772	NFATC1	nuclear factor of activated T-cells, cytoplasmic, calcineurin-dependent 1	0.97334	0.984989	0.9712836
4773	NFATC2	nuclear factor of activated T-cells, cytoplasmic, calcineurin-dependent 2	0.99282	1.068009	0.9384251
4774	NFIA	nuclear factor I/A	1.0017	0.997141	0.9886241
4775	NFATC3	nuclear factor of activated T-cells, cytoplasmic, calcineurin-dependent 3	0.9472	1.016248	0.8916195
4776	NFATC4	nuclear factor of activated T-cells, cytoplasmic, calcineurin-dependent 4	0.99131	1.027749	1.0132623
4778	NFE2	nuclear factor (erythroid-derived 2), 45kDa	0.93165	0.866396	1.0537769
4779	NFE2L1	nuclear factor (erythroid-derived 2)-like 1	0.93746	0.918593	1.034973
4780	NFE2L2	nuclear factor (erythroid-derived 2)-like 2	1.02165	1.069951	0.9162742
4781	NFIB	nuclear factor I/B	0.98316	0.988923	1.0022246
4782	NFIC	nuclear factor I/C (CCAAT-binding transcription factor)	0.98042	0.988192	0.9973507
4783	NFIL3	nuclear factor, interleukin 3 regulated	0.97425	0.990318	0.9875414
4784	NFIX	nuclear factor I/X (CCAAT-binding transcription factor)	0.98362	0.982626	1.0114613

4790	NFKB1	nuclear factor of kappa light polypeptide gene enhancer in B-cells 1	0.97419	1.076278	0.9718782
4791	NFKB2	nuclear factor of kappa light polypeptide gene enhancer in B-cells 2 (p49/p100)	0.94523	1.119265	1.016124
4792	NFKBIA	nuclear factor of kappa light polypeptide gene enhancer in B-cells inhibitor, alpha	0.98308	1.09348	0.9470166
4793	NFKBIB	nuclear factor of kappa light polypeptide gene enhancer in B-cells inhibitor, beta	0.94086	1.020613	1.0195799
4794	NFKBIE	nuclear factor of kappa light polypeptide gene enhancer in B-cells inhibitor, epsilon	1.01134	1.032253	0.9424807
4795	NFKBIL1	nuclear factor of kappa light polypeptide gene enhancer in B-cells inhibitor-like 1	0.97797	0.999921	1.0508666
4796	TONSL	tonsoku-like, DNA repair protein	0.97091	1.018582	1.0495363
4798	NFRKB	nuclear factor related to kappaB binding protein	0.93681	0.889924	0.9847626
4799	NFX1	nuclear transcription factor, X-box binding 1	0.97499	0.980174	1.0058555
4800	NFYA	nuclear transcription factor Y, alpha	0.97569	0.926431	1.1100696
4801	NFYB	nuclear transcription factor Y, beta	0.9313	0.971499	1.1212074
4802	NFYC	nuclear transcription factor Y, gamma	0.99104	0.926071	0.9816624
4803	NGF	nerve growth factor (beta polypeptide)	1.01263	0.988025	1.0027168
4804	NGFR	nerve growth factor receptor	1.00077	0.993763	1.0217829
4807	NHLH1	nescient helix loop helix 1	1.00031	1.000877	1.0462717
4808	NHLH2	nescient helix loop helix 2	0.99613	1.012398	1.015211
4809	NHP2L1	NHP2 non-histone chromosome protein 2-like 1 (S. cerevisiae)	0.94139	0.994107	0.94319
4810	NHS	Nance-Horan syndrome (congenital cataracts and dental anomalies)	0.996	0.997605	1.0045584
4811	NID1	nidogen 1	0.9931	0.955376	1.0118464
4814	NINJ1	ninjurin 1	0.97787	1.040811	1.0334201
4815	NINJ2	ninjurin 2	0.98873	1.000727	1.0338766
4817	NIT1	nitrilase 1	0.95728	0.993011	0.9654328
4818	NKG7	natural killer cell group 7 sequence	0.98618	0.896529	0.9608861
4820	NKTR	natural killer-tumor recognition sequence	1.02098	1.033372	0.9187694
4821	NKX2-2	NK2 homeobox 2	0.98451	1.019318	1.0385225
4824	NKX3-1	NK3 homeobox 1	1.02452	0.957039	0.9530347
4825	NKX6-1	NK6 homeobox 1	0.97646	1.026005	1.071863
4826	NNAT	neuronatin	0.98676	0.982873	0.9893943
4828	NMB	neuromedin B	1.00236	1.028771	1.0119085
4829	NMBR	neuromedin B receptor	1.00709	0.962469	1.0481933
4832	NME3	non-metastatic cells 3, protein expressed in	0.95943	0.969314	1.0634976
4833	NME4	non-metastatic cells 4, protein expressed in	0.99437	0.976734	1.0289855
4835	NQO2	NAD(P)H dehydrogenase, quinone 2	1.01089	1.044565	0.9472803

4836	NMT1	N-myristoyltransferase 1	0.95026	0.980412	1.0324097
4837	NNMT	nicotinamide N-methyltransferase	1.00415	0.989944	0.9675909
4838	NODAL	nodal homolog (mouse)	1.01867	1.015604	1.0224612
4839	NOP2	NOP2 nucleolar protein homolog (yeast)	0.91895	0.99639	0.9974877
4841	NONO	non-POU domain containing, octamer-binding	1.02704	0.989953	0.9212629
4842	NOS1	nitric oxide synthase 1 (neuronal)	0.99691	1.000624	1.0020491
4843	NOS2	nitric oxide synthase 2, inducible	0.99112	1.008144	1.0042643
4846	NOS3	nitric oxide synthase 3 (endothelial cell)	0.99801	1.003772	0.9888983
4848	CNOT2	CCR4-NOT transcription complex, subunit 2	0.95283	0.949412	1.0026523
4849	CNOT3	CCR4-NOT transcription complex, subunit 3	0.94194	0.995248	0.9730888
4850	CNOT4	CCR4-NOT transcription complex, subunit 4	0.98646	0.973378	0.9986541
4851	NOTCH1	notch 1	0.9728	1.034227	0.9931348
4852	NPY	neuropeptide Y	1.00346	0.988099	1.0122753
4854	NOTCH3	notch 3	0.98108	1.014268	1.0413591
4855	NOTCH4	notch 4	1.02166	1.022709	1.0123354
4856	NOV	nephroblastoma overexpressed gene	0.98543	0.976217	0.9861062
4857	NOVA1	neuro-oncological ventral antigen 1	0.99917	1.015866	1
4858	NOVA2	neuro-oncological ventral antigen 2	0.97168	0.991937	1.0145005
4860	PNP	purine nucleoside phosphorylase	0.98279	0.923728	1.2813341
4861	NPAS1	neuronal PAS domain protein 1	0.99891	0.997802	1.0142512
4862	NPAS2	neuronal PAS domain protein 2	0.99645	0.985379	1.003575
4863	NPAT	nuclear protein, ataxia-telangiectasia locus	0.90304	1.022412	1.0139014
4864	NPC1	Niemann-Pick disease, type C1	0.97257	1.16143	1.0527013
4867	NPHP1	nephronophthisis 1 (juvenile)	1.01023	1.016095	1.0173115
4868	NPHS1	nephrosis 1, congenital, Finnish type (nephrin)	0.98245	1.001342	1.0103206
4869	NPM1	nucleophosmin (nucleolar phosphoprotein B23, numatrin)	1.00646	0.96921	0.9739782
4872	NPM1P3	nucleophosmin 1 (nucleolar phosphoprotein B23, numatrin) pseudogene 3	1.02095	0.988986	1.0015509
4874	NPM1P5	nucleophosmin 1 (nucleolar phosphoprotein B23, numatrin) pseudogene 5	0.97607	0.927718	1.0302664
4878	NPPA	natriuretic peptide A	1.00818	0.953093	0.9868511
4879	NPPB	natriuretic peptide B	1.00291	0.980682	1.0551038
4880	NPPC	natriuretic peptide C	0.97706	1.026822	1.036088
4881	NPR1	natriuretic peptide receptor A/guanylate cyclase A (atrionatriuretic peptide receptor A)	1.00961	0.979732	1.0220615
4882	NPR2	natriuretic peptide receptor B/guanylate cyclase B (atrionatriuretic peptide receptor B)	0.97197	1.014217	1.0206269

4883	NPR3	natriuretic peptide receptor C/guanylate cyclase C (atrionatriuretic peptide receptor C)	1.00064	1.015066	1.0011557
4884	NPTX1	neuronal pentraxin I	0.99336	1.024043	1.0640896
4885	NPTX2	neuronal pentraxin II	1.00575	1.039311	1.0369567
4886	NPY1R	neuropeptide Y receptor Y1	0.99415	1.010852	1.0113731
4887	NPY2R	neuropeptide Y receptor Y2	1.01197	0.966423	1.0078043
4888	NPY6R	neuropeptide Y receptor Y6 (pseudogene)	1.00997	1.037464	1.0181062
4889	NPY5R	neuropeptide Y receptor Y5	1.00933	0.975168	1.0180988
4891	SLC11A2	solute carrier family 11 (proton-coupled divalent metal ion transporters), member 2	0.98694	1.096807	0.954265
4892	NRAP	nebulin-related anchoring protein	1.00685	0.990878	0.9957561
4893	NRAS	neuroblastoma RAS viral (v-ras) oncogene homolog	1.01715	0.885597	1.067184
4897	NRCAM	neuronal cell adhesion molecule	0.98868	1.030711	0.9987039
4898	NRD1	nardilysin (N-arginine dibasic convertase)	1.01055	0.902002	1.0336411
4899	NRF1	nuclear respiratory factor 1	0.97073	0.959379	0.9943406
4900	NRGN	neurogranin (protein kinase C substrate, RC3)	0.9417	0.983804	0.9428103
4901	NRL	neural retina leucine zipper	0.98963	1.036202	0.9947662
4902	NRTN	neurturin	0.98787	1.013962	1.0814702
4904	YBX1	Y box binding protein 1	1.08039	0.945518	1.0104867
4905	NSF	N-ethylmaleimide-sensitive factor	0.96746	1.049764	0.9819724
4907	NT5E	5'-nucleotidase, ecto (CD73)	0.96437	0.968052	1.0118479
4908	NTF3	neurotrophin 3	1.00218	1.008019	1.0143578
4909	NTF4	neurotrophin 4	1.00529	1.046402	1.0355143
4911	NTF6B	neurotrophin 6 beta (pseudogene)	0.98067	1.038538	0.9449314
4913	NTHL1	nth endonuclease III-like 1 (E. coli)	0.97671	0.982101	1.0253703
4914	NTRK1	neurotrophic tyrosine kinase, receptor, type 1	1.00859	0.998417	0.9972286
4915	NTRK2	neurotrophic tyrosine kinase, receptor, type 2	0.99684	0.99858	1.0039583
4916	NTRK3	neurotrophic tyrosine kinase, receptor, type 3	0.99622	0.996359	0.9986821
4917	NTN3	netrin 3	0.99148	0.988948	1.0783088
4919	ROR1	receptor tyrosine kinase-like orphan receptor 1	1.0147	0.993752	1.0078396
4920	ROR2	receptor tyrosine kinase-like orphan receptor 2	0.98851	0.979267	1.0181136
4921	DDR2	discoidin domain receptor tyrosine kinase 2	1.0109	1.033854	0.9772435
4922	NTS	neurotensin	0.97907	0.987548	0.9901375
4924	NUCB1	nucleobindin 1	0.96556	0.890643	1.0589784
4925	NUCB2	nucleobindin 2	0.88845	1.001449	0.8267487

4926	NUMA1	nuclear mitotic apparatus protein 1	0.96409	0.964182	1.0207466
4927	NUP88	nucleoporin 88kDa	0.91942	1.043782	1.0746649
4928	NUP98	nucleoporin 98kDa	0.93895	1.146341	0.960287
4929	NR4A2	nuclear receptor subfamily 4, group A, member 2	1.00543	0.976937	0.9298665
4931	NVL	nuclear VCP-like	0.95265	1.000374	1.0675191
4935	GPR143	G protein-coupled receptor 143	0.98908	1.026062	0.9885873
4938	OAS1	2',5'-oligoadenylate synthetase 1, 40/46kDa	0.96946	0.918532	1.2061793
4939	OAS2	2'-5'-oligoadenylate synthetase 2, 69/71kDa	0.93493	0.928217	1.1081062
4940	OAS3	2'-5'-oligoadenylate synthetase 3, 100kDa	0.93863	0.882331	1.0893938
4942	OAT	ornithine aminotransferase	0.954	1.019429	0.9707639
4943	TBC1D25	TBC1 domain family, member 25	0.98537	0.991254	0.9768409
4946	OAZ1	ornithine decarboxylase antizyme 1	1.00319	0.95222	0.9375713
4947	OAZ2	ornithine decarboxylase antizyme 2	0.97496	1.04154	1.0129529
4948	OCA2	oculocutaneous albinism II	1.00828	0.993134	0.9870118
4950	[No Symbo	[No Name]	0.9966	1.000085	1.0176941
4952	OCRL	oculocerebrorenal syndrome of Lowe	0.98589	0.958059	1.0617076
4953	ODC1	ornithine decarboxylase 1	0.96162	0.930181	0.914559
4956	ODF1	outer dense fiber of sperm tails 1	1.01748	1.031289	1.0372539
4957	ODF2	outer dense fiber of sperm tails 2	0.96493	0.926216	1.0334403
4958	OMD	osteomodulin	0.99716	0.967169	1.0026267
4967	OGDH	oxoglutarate (alpha-ketoglutarate) dehydrogenase (lipoamide)	1.02095	0.950629	1.1318397
4968	OGG1	8-oxoguanine DNA glycosylase	1.01221	1.001421	1.0400282
4969	OGN	osteoglycin	0.99873	1.009355	0.991466
4973	OLR1	oxidized low density lipoprotein (lectin-like) receptor 1	0.97325	0.990482	0.8230425
4974	OMG	oligodendrocyte myelin glycoprotein	0.97704	1.039082	0.9566917
4976	OPA1	optic atrophy 1 (autosomal dominant)	0.99746	0.850769	1.0391698
4978	OPCML	opioid binding protein/cell adhesion molecule-like	0.99287	1.004342	1.0051152
4982	TNFRSF11B	tumor necrosis factor receptor superfamily, member 11b	0.98963	1.010905	1.019642
4983	OPHN1	oligophrenin 1	0.95963	1.009266	0.970313
4985	OPRD1	opioid receptor, delta 1	1.02578	1.001662	1.013406
4986	OPRK1	opioid receptor, kappa 1	1.00616	1.008487	1.0121637
4987	OPRL1	opiate receptor-like 1	0.99984	0.971401	0.9939677
4988	OPRM1	opioid receptor, mu 1	1.01466	0.994912	1.0099465

4990	SIX6	SIX homeobox 6	0.98334	0.974983	0.9844661
4992	OR1F1	olfactory receptor, family 1, subfamily F, member 1	1.02902	0.9659	0.9799392
4993	OR2C1	olfactory receptor, family 2, subfamily C, member 1	0.97929	0.996381	1.0567418
4994	OR3A1	olfactory receptor, family 3, subfamily A, member 1	0.97735	0.919217	1.070626
4995	OR3A2	olfactory receptor, family 3, subfamily A, member 2	0.98827	0.947051	0.9933746
4998	ORC1	origin recognition complex, subunit 1	1.01137	0.992163	1.0096254
4999	ORC2	origin recognition complex, subunit 2	0.96516	1.046162	1.0252768
5000	ORC4	origin recognition complex, subunit 4	0.98233	1.000612	1.0990913
5001	ORC5	origin recognition complex, subunit 5	0.99457	1.009742	1.0182291
5002	SLC22A18	solute carrier family 22, member 18	0.99678	0.98852	1.0110764
5003	SLC22A18A	solute carrier family 22 (organic cation transporter), member 18 antisense	1.00059	1.009895	0.9539118
5005	ORM2	orosomucoid 2	1.01021	1.03781	1.0148507
5007	OSBP	oxysterol binding protein	0.95378	0.988837	0.9695845
5008	OSM	oncostatin M	0.97757	1.011328	0.9051931
5009	OTC	ornithine carbamoyltransferase	1.0029	0.998071	1.016422
5010	CLDN11	claudin 11	1.00744	1.014397	1.0395576
5013	OTX1	orthodenticle homeobox 1	0.99725	1.025706	1.0124905
5015	OTX2	orthodenticle homeobox 2	0.99401	0.991919	0.9959976
5016	OVGP1	oviductal glycoprotein 1, 120kDa	0.98244	1.075503	0.9306013
5017	OVOL1	ovo-like 1(Drosophila)	0.98526	0.992684	1.0031725
5018	OXA1L	oxidase (cytochrome c) assembly 1-like	0.95556	0.911647	1.0278693
5019	OXCT1	3-oxoacid CoA transferase 1	0.96274	0.998547	1.0495356
5020	OXT	oxytocin, prepropeptide	1.00317	1.051097	1.0664457
5021	OXTR	oxytocin receptor	0.99338	0.99066	1.0112412
5023	P2RX1	purinergic receptor P2X, ligand-gated ion channel, 1	1.0045	1.019754	0.8689912
5024	P2RX3	purinergic receptor P2X, ligand-gated ion channel, 3	0.99886	0.996637	1.0102022
5025	P2RX4	purinergic receptor P2X, ligand-gated ion channel, 4	0.98558	1.055048	0.9334909
5027	P2RX7	purinergic receptor P2X, ligand-gated ion channel, 7	0.98534	1.030401	1.1149206
5028	P2RY1	purinergic receptor P2Y, G-protein coupled, 1	0.99444	0.88403	1.063949
5029	P2RY2	purinergic receptor P2Y, G-protein coupled, 2	1.01814	1.026572	0.9854875
5030	P2RY4	pyrimidinergic receptor P2Y, G-protein coupled, 4	0.99498	0.996585	1.0161284
5031	P2RY6	pyrimidinergic receptor P2Y, G-protein coupled, 6	1.01552	1.001659	0.9894182
5033	P4HA1	prolyl 4-hydroxylase, alpha polypeptide I	0.95772	1.053799	1.0968039

5034	P4HB	prolyl 4-hydroxylase, beta polypeptide	0.99213	0.999622	1.094653
5036	PA2G4	proliferation-associated 2G4, 38kDa	0.99085	1.017851	1.0419187
5037	PEBP1	phosphatidylethanolamine binding protein 1	1.0003	0.921842	0.9909129
5042	PABPC3	poly(A) binding protein, cytoplasmic 3	0.98648	1.023762	1.0213712
5045	FURIN	furin (paired basic amino acid cleaving enzyme)	0.94586	1.017018	1.0020215
5046	PCSK6	proprotein convertase subtilisin/kexin type 6	0.96278	0.986688	0.9847791
5047	PAEP	progestagen-associated endometrial protein	0.99492	0.98383	1.0155705
5048	PAFAH1B1	platelet-activating factor acetylhydrolase 1b, regulatory subunit 1 (45kDa)	0.98297	0.975466	0.9910248
5049	PAFAH1B2	platelet-activating factor acetylhydrolase 1b, catalytic subunit 2 (30kDa)	0.96375	0.988146	0.9913509
5050	PAFAH1B3	platelet-activating factor acetylhydrolase 1b, catalytic subunit 3 (29kDa)	0.97389	0.948836	1.0407479
5051	PAFAH2	platelet-activating factor acetylhydrolase 2, 40kDa	0.98173	1.023228	0.9447587
5052	PRDX1	peroxiredoxin 1	1.02066	0.929946	1.143456
5053	PAH	phenylalanine hydroxylase	1.00594	0.997831	0.9924308
5054	SERPINE1	serpin peptidase inhibitor, clade E (nexin, plasminogen activator inhibitor type 1), member 1	0.97598	0.953361	0.9877084
5058	PAK1	p21 protein (Cdc42/Rac)-activated kinase 1	0.9465	1.029466	1.0277159
5062	PAK2	p21 protein (Cdc42/Rac)-activated kinase 2	0.9878	0.989108	0.8998862
5063	PAK3	p21 protein (Cdc42/Rac)-activated kinase 3	0.9915	1.023169	0.9843313
5064	PALM	paralemmin	0.99448	0.997553	1.0026588
5066	PAM	peptidylglycine alpha-amidating monooxygenase	1.0082	0.950412	1.0096126
5067	CNTN3	contactin 3 (plasmacytoma associated)	1.01055	1.001877	1.0145117
5068	REG3A	regenerating islet-derived 3 alpha	1.01094	0.996067	1.0067927
5069	PAPPA	pregnancy-associated plasma protein A, pappalysin 1	1.0065	0.986636	1.0068998
5073	PARN	poly(A)-specific ribonuclease (deadenylation nuclease)	0.94256	0.967024	1.0064803
5074	PAWR	PRKC, apoptosis, WT1, regulator	0.97234	1.023935	1.0543294
5075	PAX1	paired box 1	0.98716	0.986406	1.0640159
5076	PAX2	paired box 2	1.00182	0.995853	1.0165807
5077	PAX3	paired box 3	1.00298	1.026248	1.0189105
5078	PAX4	paired box 4	1.00203	0.990396	1.0209828
5080	PAX6	paired box 6	0.99583	0.991449	1.0227761
5081	PAX7	paired box 7	0.9976	1.021854	1.0073907
5082	PDCL	phosducin-like	0.94786	1.014207	1.0924547
5083	PAX9	paired box 9	1.00309	0.994237	1.025648
5087	PBX1	pre-B-cell leukemia homeobox 1	1.00366	0.983656	0.9902904

5089	PBX2	pre-B-cell leukemia homeobox 2	1.02112	1.05305	0.907347
5090	PBX3	pre-B-cell leukemia homeobox 3	0.97816	1.004201	1.0364569
5091	PC	pyruvate carboxylase	0.98742	1.018875	1.014936
5092	PCBD1	pterin-4 alpha-carbinolamine dehydratase/dimerization cofactor of hepatocyte nuclear factor 1 alpha	0.92573	1.004121	1.066571
5093	PCBP1	poly(rC) binding protein 1	1.01331	0.990927	0.9943632
5094	PCBP2	poly(rC) binding protein 2	0.97214	0.975322	0.9009056
5095	PCCA	propionyl CoA carboxylase, alpha polypeptide	0.98081	0.979437	0.9827602
5096	PCCB	propionyl CoA carboxylase, beta polypeptide	0.97461	0.919304	1.0627875
5097	PCDH1	protocadherin 1	1.00155	0.99974	0.9853566
5099	PCDH7	protocadherin 7	0.9987	1.011501	1.0271021
5100	PCDH8	protocadherin 8	1.00406	0.999826	1.071713
5101	PCDH9	protocadherin 9	0.9808	0.995901	1.0020359
5104	SERPINA5	serpin peptidase inhibitor, clade A (alpha-1 antiproteinase, antitrypsin), member 5	1.00244	1.031297	1.0360974
5105	PCK1	phosphoenolpyruvate carboxykinase 1 (soluble)	1.00051	1.025691	1.0120784
5106	PCK2	phosphoenolpyruvate carboxykinase 2 (mitochondrial)	0.97651	0.974011	1.0116413
5108	PCM1	pericentriolar material 1	0.94234	0.902685	1.1962653
5110	PCMT1	protein-L-isoaspartate (D-aspartate) O-methyltransferase	1.00596	0.979136	1.0883997
5111	PCNA	proliferating cell nuclear antigen	0.948	0.917962	0.9668525
5116	PCNT	pericentrin	0.96321	0.965023	1.0014422
5118	PCOLCE	procollagen C-endopeptidase enhancer	1.0006	1.02325	1.043286
5119	CHMP1A	chromatin modifying protein 1A	0.96611	0.994448	1.0870413
5121	PCP4	Purkinje cell protein 4	1.00872	0.977277	0.9966084
5122	PCSK1	proprotein convertase subtilisin/kexin type 1	1.00176	1.010897	1.0204289
5125	PCSK5	proprotein convertase subtilisin/kexin type 5	0.94541	0.920882	0.9612499
5126	PCSK2	proprotein convertase subtilisin/kexin type 2	0.99811	0.984241	0.9912859
5127	CDK16	cyclin-dependent kinase 16	0.95927	0.991088	0.9859961
5128	CDK17	cyclin-dependent kinase 17	0.95353	1.057586	0.9428349
5129	CDK18	cyclin-dependent kinase 18	1.00336	0.988009	1.0149418
5130	PCYT1A	phosphate cytidylyltransferase 1, choline, alpha	1.00839	0.99175	0.9898324
5132	PDC	phosducin	1.0063	1.015969	1.0319217
5133	PDCD1	programmed cell death 1	1.00181	0.997937	0.9930964
5134	PDCD2	programmed cell death 2	1.01519	1.025474	1.0164446
5136	PDE1A	phosphodiesterase 1A, calmodulin-dependent	0.99685	1.008597	1.0116146

5137	PDE1C	phosphodiesterase 1C, calmodulin-dependent 70kDa	1.00302	0.998698	1.0210815
5138	PDE2A	phosphodiesterase 2A, cGMP-stimulated	1.00248	1.01344	0.9447297
5139	PDE3A	phosphodiesterase 3A, cGMP-inhibited	0.95697	0.964667	0.9670191
5140	PDE3B	phosphodiesterase 3B, cGMP-inhibited	0.91874	0.911545	0.9356364
5141	PDE4A	phosphodiesterase 4A, cAMP-specific	0.96027	1.143233	1.0602227
5142	PDE4B	phosphodiesterase 4B, cAMP-specific	1.00566	1.045139	0.9910504
5143	PDE4C	phosphodiesterase 4C, cAMP-specific	0.98944	1.006933	1.0273923
5144	PDE4D	phosphodiesterase 4D, cAMP-specific	1.00164	0.988347	0.9843237
5145	PDE6A	phosphodiesterase 6A, cGMP-specific, rod, alpha	1.02571	1.007294	1.0184605
5146	PDE6C	phosphodiesterase 6C, cGMP-specific, cone, alpha prime	0.99928	1.018545	0.9922439
5147	PDE6D	phosphodiesterase 6D, cGMP-specific, rod, delta	0.98475	0.989616	1.0431787
5148	PDE6G	phosphodiesterase 6G, cGMP-specific, rod, gamma	0.99414	1.008992	1.0070118
5149	PDE6H	phosphodiesterase 6H, cGMP-specific, cone, gamma	0.98448	1.013857	0.9788252
5150	PDE7A	phosphodiesterase 7A	0.9348	0.977197	0.8346098
5151	PDE8A	phosphodiesterase 8A	0.98127	1.009963	0.9397619
5152	PDE9A	phosphodiesterase 9A	0.97252	1.009595	0.9934298
5153	PDE1B	phosphodiesterase 1B, calmodulin-dependent	0.98372	1.057011	0.9795274
5154	PDGFA	platelet-derived growth factor alpha polypeptide	0.97196	0.896133	0.9698938
5155	PDGFB	platelet-derived growth factor beta polypeptide	0.97441	1.027176	1.0187044
5156	PDGFRA	platelet-derived growth factor receptor, alpha polypeptide	1.00468	0.981778	1.0181122
5157	PDGFRL	platelet-derived growth factor receptor-like	1.00426	1.007261	0.9997304
5158	PDE6B	phosphodiesterase 6B, cGMP-specific, rod, beta	0.9924	0.9785	1.0257261
5159	PDGFRB	platelet-derived growth factor receptor, beta polypeptide	1.01154	0.947773	1.0323277
5160	PDHA1	pyruvate dehydrogenase (lipoamide) alpha 1	0.94304	1.054349	0.9859402
5161	PDHA2	pyruvate dehydrogenase (lipoamide) alpha 2	1.01251	1.001188	1.0480544
5162	PDHB	pyruvate dehydrogenase (lipoamide) beta	0.99617	0.960199	1.0202395
5164	PDK2	pyruvate dehydrogenase kinase, isozyme 2	0.92855	0.892293	1.0258975
5165	PDK3	pyruvate dehydrogenase kinase, isozyme 3	0.97025	1.049491	0.9731172
5166	PDK4	pyruvate dehydrogenase kinase, isozyme 4	0.99553	0.902678	0.8406807
5167	ENPP1	ectonucleotide pyrophosphatase/phosphodiesterase 1	0.99955	0.99225	1.0191273
5168	ENPP2	ectonucleotide pyrophosphatase/phosphodiesterase 2	0.98404	0.977766	0.9696713
5169	ENPP3	ectonucleotide pyrophosphatase/phosphodiesterase 3	1.01095	0.959215	0.9938094
5170	PDPK1	3-phosphoinositide dependent protein kinase-1	0.9705	1.020943	0.9635815

5172	SLC26A4	solute carrier family 26, member 4	1.0044	1.003892	1.0275925
5173	PDYN	prodynorphin	0.98654	0.995337	1.0006956
5174	PDZK1	PDZ domain containing 1	0.98952	0.972621	1.0068398
5175	PECAM1	platelet/endothelial cell adhesion molecule	0.98946	1.066618	1.0170889
5176	SERPINF1	serpin peptidase inhibitor, clade F (alpha-2 antiplasmin, pigment epithelium derived factor), member 1	1.00566	1.029853	0.9968709
5179	PENK	proenkephalin	0.97395	1.032815	1.0579132
5184	PEPD	peptidase D	0.98925	0.971929	1.0225825
5187	PER1	period homolog 1 (Drosophila)	1.01535	1.024167	0.9305834
5188	PET112	PET112 homolog (yeast)	0.99924	0.974645	1.0157008
5189	PEX1	peroxisomal biogenesis factor 1	0.97926	0.9448	1.0171924
5190	PEX6	peroxisomal biogenesis factor 6	0.9839	1.060293	1.0947149
5191	PEX7	peroxisomal biogenesis factor 7	0.98271	0.960946	0.9913256
5192	PEX10	peroxisomal biogenesis factor 10	0.98023	0.965706	1.0497859
5193	PEX12	peroxisomal biogenesis factor 12	0.92037	0.967248	0.9906965
5194	PEX13	peroxisomal biogenesis factor 13	1.00797	1.022775	1.0613845
5195	PEX14	peroxisomal biogenesis factor 14	0.98492	0.950491	0.9581174
5196	PF4	platelet factor 4	0.84463	0.920458	0.9577511
5197	PF4V1	platelet factor 4 variant 1	0.82249	1.173583	0.9270734
5198	PFAS	phosphoribosylformylglycinamidine synthase	0.95711	1.003074	0.9863399
5199	CFP	complement factor properdin	0.98028	0.890118	0.9932922
5201	PFDN1	prefoldin subunit 1	0.98619	0.949153	0.953672
5202	PFDN2	prefoldin subunit 2	0.97806	0.937389	1.0228134
5203	PFDN4	prefoldin subunit 4	0.97096	0.900635	1.0090364
5204	PFDN5	prefoldin subunit 5	0.93693	1.00672	0.9650456
5205	ATP8B1	ATPase, aminophospholipid transporter, class I, type 8B, member 1	1.01469	1.005224	1.0065942
5207	PFKFB1	6-phosphofructo-2-kinase/fructose-2,6-biphosphatase 1	0.98793	0.99654	0.9949767
5208	PFKFB2	6-phosphofructo-2-kinase/fructose-2,6-biphosphatase 2	1.03555	0.9703	1.0039623
5209	PFKFB3	6-phosphofructo-2-kinase/fructose-2,6-biphosphatase 3	0.97399	1.002444	0.8788872
5210	PFKFB4	6-phosphofructo-2-kinase/fructose-2,6-biphosphatase 4	1.03276	0.980446	1.0513821
5211	PFKL	phosphofructokinase, liver	0.97742	0.978756	1.1033821
5212	VIT	vitrin	1.0089	1.000844	1.0196957
5213	PFKM	phosphofructokinase, muscle	0.95158	0.945053	1.0097679
5214	PFKP	phosphofructokinase, platelet	0.98703	0.974033	1.0298232

5216	PFN1	profilin 1	0.92421	1.063118	1.0437903
5217	PFN2	profilin 2	1.00174	0.891952	0.910507
5218	CDK14	cyclin-dependent kinase 14	1.01109	0.974915	1.043314
5222	PGA5	pepsinogen 5, group I (pepsinogen A)	1.03849	0.942471	0.9943129
5223	PGAM1	phosphoglycerate mutase 1 (brain)	1.01668	0.948536	0.9925752
5224	PGAM2	phosphoglycerate mutase 2 (muscle)	0.98362	0.99668	0.9815727
5225	PGC	progastricsin (pepsinogen C)	1.02197	1.00111	1.0070273
5226	PGD	phosphogluconate dehydrogenase	1.03941	1.020947	0.923554
5228	PGF	placental growth factor	0.99619	0.988277	1.0448248
5229	PGGT1B	protein geranylgeranyltransferase type I, beta subunit	1.03427	0.958933	1.0531874
5230	PGK1	phosphoglycerate kinase 1	0.98006	1.013052	1.0217408
5232	PGK2	phosphoglycerate kinase 2	1.0036	0.984705	0.9764588
5233	PGK1P2	phosphoglycerate kinase 1, pseudogene 2	0.99798	1.00341	1.0028247
5236	PGM1	phosphoglucomutase 1	0.996	0.960811	1.041407
5238	PGM3	phosphoglucomutase 3	1.01424	0.993628	1.0224018
5239	PGM5	phosphoglucomutase 5	0.99838	0.991557	1.0074961
5241	PGR	progesterone receptor	0.99733	1.037006	1.044624
5243	ABCB1	ATP-binding cassette, sub-family B (MDR/TAP), member 1	0.99487	1.086127	1.0015396
5245	PHB	prohibitin	0.94732	0.944894	1.0286097
5246	PHBP1	prohibitin pseudogene 1	1.01275	0.94566	1.0248026
5250	SLC25A3	solute carrier family 25 (mitochondrial carrier; phosphate carrier), member 3	1.03123	0.907721	0.9688718
5251	PHEX	phosphate regulating endopeptidase homolog, X-linked	1.01357	0.997965	0.9672478
5252	PHF1	PHD finger protein 1	0.95866	0.976723	0.8931561
5253	PHF2	PHD finger protein 2	0.94151	0.921826	0.991849
5255	PHKA1	phosphorylase kinase, alpha 1 (muscle)	0.99318	1.009909	1.0148376
5256	PHKA2	phosphorylase kinase, alpha 2 (liver)	0.96173	0.934702	0.9954073
5257	PHKB	phosphorylase kinase, beta	0.95201	0.968937	1.0535074
5258	PHKBP1	phosphorylase kinase, beta pseudogene 1	1.01404	0.963423	1.0227368
5259	PHKBP2	phosphorylase kinase, beta pseudogene 2	0.99463	0.976441	0.9511976
5260	PHKG1	phosphorylase kinase, gamma 1 (muscle)	1.00829	1.052089	0.9793652
5261	PHKG2	phosphorylase kinase, gamma 2 (testis)	0.95989	0.965517	1.0935175
5262	PHKG1P3	phosphorylase kinase, gamma 1 pseudogene 3	0.99879	0.954869	1.0567953
5264	PHYH	phytanoyl-CoA 2-hydroxylase	0.96868	0.962548	0.9813921

5265	SERPINA1	serpin peptidase inhibitor, clade A (alpha-1 antiproteinase, antitrypsin), member 1	1.0043	1.000696	1.0288472
5266	PI3	peptidase inhibitor 3, skin-derived	0.98745	1.024183	0.9542312
5267	SERPINA4	serpin peptidase inhibitor, clade A (alpha-1 antiproteinase, antitrypsin), member 4	0.99819	0.969589	1.0148072
5268	SERPINB5	serpin peptidase inhibitor, clade B (ovalbumin), member 5	1.01468	0.991035	0.9755549
5269	SERPINB6	serpin peptidase inhibitor, clade B (ovalbumin), member 6	1.00855	0.985401	0.9579843
5270	SERPINE2	serpin peptidase inhibitor, clade E (nexin, plasminogen activator inhibitor type 1), member 2	0.99141	0.979119	0.9627471
5271	SERPINB8	serpin peptidase inhibitor, clade B (ovalbumin), member 8	0.95536	0.938887	1.0378735
5272	SERPINB9	serpin peptidase inhibitor, clade B (ovalbumin), member 9	1.00345	1.099661	0.9446918
5273	SERPINB10	serpin peptidase inhibitor, clade B (ovalbumin), member 10	1.00441	1.032562	1.0847047
5274	SERPINI1	serpin peptidase inhibitor, clade I (neuroserpin), member 1	0.96244	0.963972	1.0447253
5275	SERPINB13	serpin peptidase inhibitor, clade B (ovalbumin), member 13	1.01371	0.997447	1.0118658
5276	SERPINI2	serpin peptidase inhibitor, clade I (pancpin), member 2	1.00366	0.98406	1.0141742
5277	PIGA	phosphatidylinositol glycan anchor biosynthesis, class A	0.95295	0.979147	0.9789099
5279	PIGC	phosphatidylinositol glycan anchor biosynthesis, class C	0.99182	0.897461	1.0170895
5281	PIGF	phosphatidylinositol glycan anchor biosynthesis, class F	1.03568	0.988693	1.0468884
5283	PIGH	phosphatidylinositol glycan anchor biosynthesis, class H	0.97755	0.943973	0.987927
5284	PIGR	polymeric immunoglobulin receptor	1.00916	0.985087	1.0291473
5286	PIK3C2A	phosphoinositide-3-kinase, class 2, alpha polypeptide	0.93455	1.083629	0.9674495
5287	PIK3C2B	phosphoinositide-3-kinase, class 2, beta polypeptide	1.0081	1.009598	1.0498275
5288	PIK3C2G	phosphoinositide-3-kinase, class 2, gamma polypeptide	1.00009	1.002491	0.9839867
5289	PIK3C3	phosphoinositide-3-kinase, class 3	0.95075	0.976618	1.0171816
5290	PIK3CA	phosphoinositide-3-kinase, catalytic, alpha polypeptide	0.98727	0.833495	1.0008279
5291	PIK3CB	phosphoinositide-3-kinase, catalytic, beta polypeptide	1.00322	0.98473	0.9024472
5292	PIM1	pim-1 oncogene	0.98564	0.994035	1.0478312
5293	PIK3CD	phosphoinositide-3-kinase, catalytic, delta polypeptide	0.98367	1.016967	0.9970736
5294	PIK3CG	phosphoinositide-3-kinase, catalytic, gamma polypeptide	1.02831	1.006834	1.0016473
5295	PIK3R1	phosphoinositide-3-kinase, regulatory subunit 1 (alpha)	1.02467	1.006625	0.9392554
5296	PIK3R2	phosphoinositide-3-kinase, regulatory subunit 2 (beta)	0.95824	0.930043	1.0711808
5297	PI4KA	phosphatidylinositol 4-kinase, catalytic, alpha	0.96954	0.99112	1.0165846
5298	PI4KB	phosphatidylinositol 4-kinase, catalytic, beta	1.00424	0.950897	1.0054027
5300	PIN1	peptidylprolyl cis/trans isomerase, NIMA-interacting 1	0.97351	0.967698	1.0256639
5303	PIN4	protein (peptidylprolyl cis/trans isomerase) NIMA-interacting, 4 (parvulin)	0.99383	0.991392	1.0034931
5304	PIP	prolactin-induced protein	1.01515	0.996774	1.0269766

5305	PIP4K2A	phosphatidylinositol-5-phosphate 4-kinase, type II, alpha	1.01015	1.073993	0.9732752
5306	PITPNA	phosphatidylinositol transfer protein, alpha	0.97015	0.99208	0.9898423
5307	PITX1	paired-like homeodomain 1	1.01498	1.009552	1.0002088
5308	PITX2	paired-like homeodomain 2	0.99048	1.032783	1.0497997
5309	PITX3	paired-like homeodomain 3	0.99181	0.983423	1.0046348
5310	PKD1	polycystic kidney disease 1 (autosomal dominant)	0.99566	0.995195	1.0617775
5311	PKD2	polycystic kidney disease 2 (autosomal dominant)	0.99822	0.989956	1.0460188
5313	PKLR	pyruvate kinase, liver and RBC	1.00188	0.998265	0.9990651
5315	PKM2	pyruvate kinase, muscle	1.01485	0.980255	1.0003329
5316	PKNOX1	PBX/knotted 1 homeobox 1	0.95783	0.985585	0.9455272
5317	PKP1	plakophilin 1 (ectodermal dysplasia/skin fragility syndrome)	1.01327	0.989562	0.9975721
5318	PKP2	plakophilin 2	0.97684	0.979315	0.9758489
5319	PLA2G1B	phospholipase A2, group IB (pancreas)	1.00163	1.029263	0.9642811
5320	PLA2G2A	phospholipase A2, group IIA (platelets, synovial fluid)	1.00532	1.025051	0.9785938
5321	PLA2G4A	phospholipase A2, group IVA (cytosolic, calcium-dependent)	0.9964	0.952213	1.0520816
5322	PLA2G5	phospholipase A2, group V	1.00564	1.00697	1.0259072
5325	PLAGL1	pleiomorphic adenoma gene-like 1	1.02854	0.963731	0.9750347
5326	PLAGL2	pleiomorphic adenoma gene-like 2	0.99299	0.988122	1.0200228
5327	PLAT	plasminogen activator, tissue	0.9873	1.006669	0.9843822
5328	PLAU	plasminogen activator, urokinase	1.01431	0.968797	0.983236
5329	PLAUR	plasminogen activator, urokinase receptor	0.9835	0.969565	0.9692098
5330	PLCB2	phospholipase C, beta 2	0.98222	0.926266	1.0261306
5331	PLCB3	phospholipase C, beta 3 (phosphatidylinositol-specific)	0.95304	1.042182	1.0067534
5332	PLCB4	phospholipase C, beta 4	0.99202	0.996943	0.995987
5333	PLCD1	phospholipase C, delta 1	0.9943	1.008899	1.0065542
5334	PLCL1	phospholipase C-like 1	0.98489	0.908225	0.9345924
5335	PLCG1	phospholipase C, gamma 1	0.94861	0.995713	0.9837273
5336	PLCG2	phospholipase C, gamma 2 (phosphatidylinositol-specific)	0.97731	1.078107	0.9901755
5337	PLD1	phospholipase D1, phosphatidylcholine-specific	0.99992	0.963319	1.0760799
5338	PLD2	phospholipase D2	0.96575	1.063697	0.9474038
5339	PLEC	plectin	0.96628	0.94408	1.0136052
5340	PLG	plasminogen	1.01191	0.995945	1.0141231
5341	PLEK	pleckstrin	1.02759	1.013761	0.9428776

5345	SERPINF2	serpin peptidase inhibitor, clade F (alpha-2 antiplasmin, pigment epithelium derived factor), member 2	0.99722	0.956078	1.0022035
5346	PLIN1	perilipin 1	1.00461	0.986897	1.0571184
5348	FXYD1	FXYD domain containing ion transport regulator 1	1.0023	1.017633	1.0705394
5349	FXYD3	FXYD domain containing ion transport regulator 3	1.01157	1.021454	1.0049577
5350	PLN	phospholamban	1.02018	1.016265	0.9732695
5351	PLOD1	procollagen-lysine 1, 2-oxoglutarate 5-dioxygenase 1	1.00241	0.925074	1.1035763
5352	PLOD2	procollagen-lysine, 2-oxoglutarate 5-dioxygenase 2	0.96797	0.921418	1.0349668
5354	PLP1	proteolipid protein 1	1.01158	1.025109	0.9927212
5355	PLP2	proteolipid protein 2 (colonic epithelium-enriched)	0.97155	1.023866	1.0691501
5356	PLRG1	pleiotropic regulator 1	0.98382	0.952555	1.050071
5357	PLS1	plastin 1	1.00244	0.977472	1.0077956
5358	PLS3	plastin 3	0.99506	1.003493	0.993622
5359	PLSCR1	phospholipid scramblase 1	1.05443	1.013801	1.0099358
5360	PLTP	phospholipid transfer protein	0.98597	0.970971	0.9941869
5361	PLXNA1	plexin A1	1.00882	0.972664	1.0051463
5362	PLXNA2	plexin A2	1.01156	0.970543	1.0002808
5364	PLXNB1	plexin B1	1.01416	1.010886	1.0136859
5365	PLXNB3	plexin B3	0.98203	0.99793	1.0182495
5366	PMAIP1	phorbol-12-myristate-13-acetate-induced protein 1	0.91791	1.007163	0.9624703
5367	PMCH	pro-melanin-concentrating hormone	0.97915	0.970005	1.0165473
5368	PNOC	prepronociceptin	0.97404	0.997818	1.0187339
5369	PMCHL1	pro-melanin-concentrating hormone-like 1, pseudogene	1.00174	0.959788	0.9816737
5370	PMCHL2	pro-melanin-concentrating hormone-like 2, pseudogene	1.00066	0.991071	1.049122
5371	PML	promyelocytic leukemia	0.9888	0.962204	1.0339747
5372	PMM1	phosphomannomutase 1	0.96064	1.05271	0.9932327
5373	PMM2	phosphomannomutase 2	0.95875	1.004131	1.0433418
5375	PMP2	peripheral myelin protein 2	0.96706	1.020779	1.0098437
5376	PMP22	peripheral myelin protein 22	0.99577	1.012676	0.9732289
5378	PMS1	PMS1 postmeiotic segregation increased 1 (S. cerevisiae)	0.99496	0.976976	0.9838046
5379	PMS2P1	postmeiotic segregation increased 2 pseudogene 1	0.98598	0.961705	0.995299
5382	PMS2P4	postmeiotic segregation increased 2 pseudogene 4	1.02391	0.970653	0.9837921
5393	EXOSC9	exosome component 9	0.98512	0.927011	1.0146945
5394	EXOSC10	exosome component 10	0.9976	1.011755	1.0121533

5395	PMS2	PMS2 postmeiotic segregation increased 2 (S. cerevisiae)	0.99083	0.957391	1.0946589
5396	PRRX1	paired related homeobox 1	1.00602	1.018486	1.019623
5406	PNLIP	pancreatic lipase	0.99011	1.04307	1.0031628
5407	PNLIPRP1	pancreatic lipase-related protein 1	0.98844	1.013152	0.9971274
5409	PNMT	phenylethanolamine N-methyltransferase	1.01478	0.998296	0.9547965
5411	PNN	pinin, desmosome associated protein	0.89777	1.085189	0.8965408
5412	UBL3	ubiquitin-like 3	0.98002	1.022751	0.97328
5413	5-Sep	septin 5	0.93031	0.932587	0.9461685
5414	4-Sep	septin 4	0.96701	0.982857	0.9795049
5420	PODXL	podocalyxin-like	0.99484	1.007954	1.0084594
5422	POLA1	polymerase (DNA directed), alpha 1, catalytic subunit	0.971	0.932618	1.0100923
5423	POLB	polymerase (DNA directed), beta	1.01794	1.012556	0.8784453
5424	POLD1	polymerase (DNA directed), delta 1, catalytic subunit 125kDa	0.97317	0.967499	1.0459653
5425	POLD2	polymerase (DNA directed), delta 2, regulatory subunit 50kDa	1.00209	0.967498	1.0094911
5426	POLE	polymerase (DNA directed), epsilon	0.97295	0.93191	1.0079993
5427	POLE2	polymerase (DNA directed), epsilon 2 (p59 subunit)	0.95412	0.94327	1.0118063
5428	POLG	polymerase (DNA directed), gamma	0.96092	0.963205	0.9660061
5429	POLH	polymerase (DNA directed), eta	1.00091	0.933509	1.0311712
5430	POLR2A	polymerase (RNA) II (DNA directed) polypeptide A, 220kDa	0.9762	0.986524	0.9280956
5431	POLR2B	polymerase (RNA) II (DNA directed) polypeptide B, 140kDa	1.00767	1.150784	1.0755189
5432	POLR2C	polymerase (RNA) II (DNA directed) polypeptide C, 33kDa	0.92925	0.946913	1.0288807
5433	POLR2D	polymerase (RNA) II (DNA directed) polypeptide D	0.9883	0.968167	1.0334148
5434	POLR2E	polymerase (RNA) II (DNA directed) polypeptide E, 25kDa	0.97354	0.992952	0.98204
5435	POLR2F	polymerase (RNA) II (DNA directed) polypeptide F	0.92476	0.989272	1.0221818
5436	POLR2G	polymerase (RNA) II (DNA directed) polypeptide G	0.89715	0.944077	1.128988
5437	POLR2H	polymerase (RNA) II (DNA directed) polypeptide H	0.96194	0.992336	1.0499024
5438	POLR2I	polymerase (RNA) II (DNA directed) polypeptide I, 14.5kDa	0.97473	0.994506	1.1030706
5439	POLR2J	polymerase (RNA) II (DNA directed) polypeptide J, 13.3kDa	0.97106	0.937213	1.0252205
5440	POLR2K	polymerase (RNA) II (DNA directed) polypeptide K, 7.0kDa	0.94522	0.96094	0.8875992
5441	POLR2L	polymerase (RNA) II (DNA directed) polypeptide L, 7.6kDa	1.01368	0.987035	1.1592332
5442	POLRMT	polymerase (RNA) mitochondrial (DNA directed)	0.9757	1.006106	1.0500525
5443	POMC	proopiomelanocortin	0.99156	0.989992	0.9785339
5444	PON1	paraoxonase 1	1.00971	0.997697	1.0314853

5445	PON2	paraoxonase 2	1.03246	0.976019	0.9935643
5446	PON3	paraoxonase 3	1.0078	1.013344	1.0121912
5447	POR	P450 (cytochrome) oxidoreductase	0.99731	1.002322	1.031694
5449	POU1F1	POU class 1 homeobox 1	0.99413	1.01374	1.0313209
5450	POU2AF1	POU class 2 associating factor 1	0.98935	1.033934	1.0112428
5451	POU2F1	POU class 2 homeobox 1	1.00454	0.939848	0.9516235
5452	POU2F2	POU class 2 homeobox 2	0.98878	1.010322	1.027936
5453	POU3F1	POU class 3 homeobox 1	1.00319	1.015583	1.1132349
5454	POU3F2	POU class 3 homeobox 2	0.98207	1.04007	1.0551542
5455	POU3F3	POU class 3 homeobox 3	0.99931	0.970912	1.0258115
5456	POU3F4	POU class 3 homeobox 4	0.98636	0.992191	1.0943059
5457	POU4F1	POU class 4 homeobox 1	0.9959	1.022092	1.067899
5458	POU4F2	POU class 4 homeobox 2	1.00816	1.024906	1.0287652
5459	POU4F3	POU class 4 homeobox 3	0.99616	1.000525	0.9636299
5460	POU5F1	POU class 5 homeobox 1	0.99838	0.963934	0.9992703
5463	POU6F1	POU class 6 homeobox 1	0.97405	0.945086	0.9518603
5464	PPA1	pyrophosphatase (inorganic) 1	0.96819	0.99361	1.0451723
5465	PPARA	peroxisome proliferator-activated receptor alpha	0.98291	1.009319	0.9889124
5467	PPARD	peroxisome proliferator-activated receptor delta	0.96821	0.980877	0.9802665
5468	PPARG	peroxisome proliferator-activated receptor gamma	1.00665	1.012664	0.9702431
5469	MED1	mediator complex subunit 1	0.91266	0.868627	0.9917948
5471	PPAT	phosphoribosyl pyrophosphate amidotransferase	0.99134	0.944916	1.0308465
5473	PPBP	pro-platelet basic protein (chemokine (C-X-C motif) ligand 7)	0.91724	1.05109	0.9845009
5475	PPEF1	protein phosphatase, EF-hand calcium binding domain 1	0.99469	1.005356	0.995652
5476	CTSA	cathepsin A	0.96292	0.942988	0.9976465
5478	PPIA	peptidylprolyl isomerase A (cyclophilin A)	1.05919	0.949752	1.027273
5479	PPIB	peptidylprolyl isomerase B (cyclophilin B)	0.98773	0.935271	1.1073414
5480	PPIC	peptidylprolyl isomerase C (cyclophilin C)	0.99246	1.016966	1.031176
5486	PPIAP14	peptidylprolyl isomerase A (cyclophilin A) pseudogene 14	0.96508	0.975115	1.0465623
5487	PPIAP15	peptidylprolyl isomerase A (cyclophilin A) pseudogene 15	1.02087	0.967842	1.017945
5488	PPIAP16	peptidylprolyl isomerase A (cyclophilin A) pseudogene 16	1.00318	1.016227	1.0561297
5489	PPIAP7	peptidylprolyl isomerase A (cyclophilin A) pseudogene 7	1.00855	1.054075	1.0311477
5491	PPIAP9	peptidylprolyl isomerase A (cyclophilin A) pseudogene 9	0.95842	0.977071	1.1038956

5493	PPL	periplakin	0.99598	1.004437	1.0003604
5494	PPM1A	protein phosphatase, Mg2+/Mn2+ dependent, 1A	0.96902	1.008715	1.0498765
5495	PPM1B	protein phosphatase, Mg2+/Mn2+ dependent, 1B	0.97956	0.913779	0.9914403
5496	PPM1G	protein phosphatase, Mg2+/Mn2+ dependent, 1G	0.98185	1.017453	1.0475937
5498	PPOX	protoporphyrinogen oxidase	0.97457	0.966716	1.0569639
5499	PPP1CA	protein phosphatase 1, catalytic subunit, alpha isozyme	0.96164	0.839833	1.1320996
5500	PPP1CB	protein phosphatase 1, catalytic subunit, beta isozyme	1.01213	0.99364	0.963323
5501	PPP1CC	protein phosphatase 1, catalytic subunit, gamma isozyme	0.98249	0.977261	1.0681784
5502	PPP1R1A	protein phosphatase 1, regulatory (inhibitor) subunit 1A	1.00229	1.006671	1.0273548
5504	PPP1R2	protein phosphatase 1, regulatory (inhibitor) subunit 2	0.97321	0.945253	0.9345126
5505	[No Symbo	[No Name]	1.01686	0.974983	1.0114598
5506	PPP1R3A	protein phosphatase 1, regulatory (inhibitor) subunit 3A	1.01091	1.005496	1.009022
5507	PPP1R3C	protein phosphatase 1, regulatory (inhibitor) subunit 3C	0.97645	1.020528	1.0486972
5509	PPP1R3D	protein phosphatase 1, regulatory (inhibitor) subunit 3D	0.95109	0.935327	1.0989518
5510	PPP1R7	protein phosphatase 1, regulatory (inhibitor) subunit 7	0.97104	0.972111	1.0754889
5511	PPP1R8	protein phosphatase 1, regulatory (inhibitor) subunit 8	0.95324	1.005812	1.0753696
5514	PPP1R10	protein phosphatase 1, regulatory (inhibitor) subunit 10	0.98776	1.081655	0.9767014
5515	PPP2CA	protein phosphatase 2, catalytic subunit, alpha isozyme	1.03212	0.927361	0.966349
5516	PPP2CB	protein phosphatase 2, catalytic subunit, beta isozyme	0.94316	0.997086	1.1276794
5517	PPP2CBP1	protein phosphatase 2, catalytic subunit, beta isozyme pseudogene 1	1.00648	0.976717	1.0168905
5518	PPP2R1A	protein phosphatase 2, regulatory subunit A, alpha	0.91894	0.953416	1.0521605
5519	PPP2R1B	protein phosphatase 2, regulatory subunit A, beta	0.95072	0.994296	0.9973198
5520	PPP2R2A	protein phosphatase 2, regulatory subunit B, alpha	0.97289	0.945544	0.9769404
5521	PPP2R2B	protein phosphatase 2, regulatory subunit B, beta	1.02567	1.016072	0.9783266
5522	PPP2R2C	protein phosphatase 2, regulatory subunit B, gamma	0.99442	1.002812	1.011355
5523	PPP2R3A	protein phosphatase 2, regulatory subunit B'', alpha	1.00938	0.993109	1.0005492
5524	PPP2R4	protein phosphatase 2A activator, regulatory subunit 4	0.93168	0.916096	1.1186679
5525	PPP2R5A	protein phosphatase 2, regulatory subunit B', alpha	0.97392	0.95511	0.9322498
5526	PPP2R5B	protein phosphatase 2, regulatory subunit B', beta	0.9626	1.063784	1.0074506
5527	PPP2R5C	protein phosphatase 2, regulatory subunit B', gamma	0.99475	1.092586	0.9889597
5528	PPP2R5D	protein phosphatase 2, regulatory subunit B', delta	0.98952	0.936194	1.0689854
5529	PPP2R5E	protein phosphatase 2, regulatory subunit B', epsilon isoform	0.97415	0.930486	1.0226595
5530	PPP3CA	protein phosphatase 3, catalytic subunit, alpha isozyme	1.01524	1.008211	1.0119753

5531	PPP4C	protein phosphatase 4, catalytic subunit	0.96529	0.948266	1.0158688
5532	PPP3CB	protein phosphatase 3, catalytic subunit, beta isozyme	0.9536	1.062901	1.043294
5533	PPP3CC	protein phosphatase 3, catalytic subunit, gamma isozyme	0.97376	1.116489	0.9679315
5534	PPP3R1	protein phosphatase 3, regulatory subunit B, alpha	1.01873	0.969191	1.0014968
5536	PPP5C	protein phosphatase 5, catalytic subunit	0.95422	0.959801	1.0270179
5537	PPP6C	protein phosphatase 6, catalytic subunit	0.9618	0.966444	0.9679959
5538	PPT1	palmitoyl-protein thioesterase 1	1.03014	0.873869	1.0617262
5539	PPY	pancreatic polypeptide	0.97987	1.051552	0.9695329
5540	PPYR1	pancreatic polypeptide receptor 1	1.00177	1.046535	1.0324137
5544	PRB3	proline-rich protein BstNI subfamily 3	1.02247	1.030007	1.0197587
5545	PRB4	proline-rich protein BstNI subfamily 4	1.00631	1.01088	1.0233204
5546	PRCC	papillary renal cell carcinoma (translocation-associated)	0.9861	1.023637	1.0985979
5547	PRCP	prolylcarboxypeptidase (angiotensinase C)	0.97291	0.97434	1.0453085
5549	PRELP	proline/arginine-rich end leucine-rich repeat protein	0.99854	0.981017	1.0016134
5550	PREP	prolyl endopeptidase	0.99179	0.912323	1.0415366
5551	PRF1	perforin 1 (pore forming protein)	1.02875	0.995387	0.8997884
5552	SRGN	serglycin	0.99687	0.998271	0.9537732
5553	PRG2	proteoglycan 2, bone marrow (natural killer cell activator, eosinophil granule major basic protein)	0.99284	1.054381	0.9988551
5555	PRH2	proline-rich protein HaeIII subfamily 2	1.00449	0.993185	0.9618852
5557	PRIM1	primase, DNA, polypeptide 1 (49kDa)	0.94607	0.984996	0.9667826
5558	PRIM2	primase, DNA, polypeptide 2 (58kDa)	0.98123	0.968174	0.9785173
5562	PRKAA1	protein kinase, AMP-activated, alpha 1 catalytic subunit	1.00344	0.928215	0.9630616
5563	PRKAA2	protein kinase, AMP-activated, alpha 2 catalytic subunit	1.02407	0.987604	1.0101488
5564	PRKAB1	protein kinase, AMP-activated, beta 1 non-catalytic subunit	0.91419	0.98207	1.1171476
5565	PRKAB2	protein kinase, AMP-activated, beta 2 non-catalytic subunit	0.97152	0.937033	1.1614366
5566	PRKACA	protein kinase, cAMP-dependent, catalytic, alpha	0.94431	0.985292	0.9997181
5567	PRKACB	protein kinase, cAMP-dependent, catalytic, beta	0.98448	0.972339	0.9517193
5568	PRKACG	protein kinase, cAMP-dependent, catalytic, gamma	0.99655	0.978061	0.9825445
5569	PKIA	protein kinase (cAMP-dependent, catalytic) inhibitor alpha	0.9845	0.968842	0.9976965
5570	PKIB	protein kinase (cAMP-dependent, catalytic) inhibitor beta	0.99759	0.992236	1.0094928
5571	PRKAG1	protein kinase, AMP-activated, gamma 1 non-catalytic subunit	0.93797	0.994474	1.0628977
5573	PRKAR1A	protein kinase, cAMP-dependent, regulatory, type I, alpha (tissue specific extinguisher 1)	1.01861	1.021268	1.0641885
5575	PRKAR1B	protein kinase, cAMP-dependent, regulatory, type I, beta	0.99051	1.010659	1.0239312

5576	PRKAR2A	protein kinase, cAMP-dependent, regulatory, type II, alpha	0.98525	0.887679	0.9531494
5577	PRKAR2B	protein kinase, cAMP-dependent, regulatory, type II, beta	0.96938	0.961761	0.9690091
5578	PRKCA	protein kinase C, alpha	0.98648	0.90566	0.896638
5579	PRKCB	protein kinase C, beta	1.01628	1.007156	0.9930903
5580	PRKCD	protein kinase C, delta	1.02305	1.020019	0.9455474
5581	PRKCE	protein kinase C, epsilon	0.98008	0.965647	0.9775069
5582	PRKCG	protein kinase C, gamma	0.98422	1.030624	0.9965761
5583	PRKCH	protein kinase C, eta	0.9835	1.074562	1.0190021
5584	PRKCI	protein kinase C, iota	1.00226	0.996636	0.9815867
5585	PKN1	protein kinase N1	0.99011	1.020286	1.0150679
5586	PKN2	protein kinase N2	0.98889	0.985869	0.9475044
5587	PRKD1	protein kinase D1	1.00173	1.006738	0.9918847
5588	PRKCQ	protein kinase C, theta	0.9325	1.097448	0.9347181
5589	PRKCSH	protein kinase C substrate 80K-H	0.94881	0.944631	1.0795498
5590	PRKCZ	protein kinase C, zeta	0.99668	0.99286	1.0087199
5591	PRKDC	protein kinase, DNA-activated, catalytic polypeptide	0.9546	0.878779	0.9986128
5592	PRKG1	protein kinase, cGMP-dependent, type I	0.99935	0.981597	1.0022814
5593	PRKG2	protein kinase, cGMP-dependent, type II	0.99633	0.95212	0.9791088
5594	MAPK1	mitogen-activated protein kinase 1	0.98905	1.038853	0.9706291
5595	MAPK3	mitogen-activated protein kinase 3	0.92104	0.938063	1.0716102
5596	MAPK4	mitogen-activated protein kinase 4	0.9981	1.000286	1.0076976
5597	MAPK6	mitogen-activated protein kinase 6	0.9792	1.01196	0.9735415
5598	MAPK7	mitogen-activated protein kinase 7	0.95549	1.025052	0.9921169
5599	MAPK8	mitogen-activated protein kinase 8	0.92281	0.94607	1.1177164
5600	MAPK11	mitogen-activated protein kinase 11	0.98122	0.980758	1.0161397
5601	MAPK9	mitogen-activated protein kinase 9	0.98892	0.970183	1.0193061
5602	MAPK10	mitogen-activated protein kinase 10	1.00364	1.000948	1.0203722
5603	MAPK13	mitogen-activated protein kinase 13	0.93838	0.943782	0.9700401
5604	MAP2K1	mitogen-activated protein kinase kinase 1	0.97602	1.016487	1.0030841
5605	MAP2K2	mitogen-activated protein kinase kinase 2	0.98576	1.014582	1.0340169
5606	MAP2K3	mitogen-activated protein kinase kinase 3	0.99319	0.984867	0.9932458
5607	MAP2K5	mitogen-activated protein kinase kinase 5	0.97922	0.982575	0.9969069
5608	MAP2K6	mitogen-activated protein kinase kinase 6	0.94437	0.964957	1.0573897

5609	MAP2K7	mitogen-activated protein kinase kinase 7	0.96207	0.963622	1.0286785
5610	EIF2AK2	eukaryotic translation initiation factor 2-alpha kinase 2	1.00049	0.897292	1.0575713
5611	DNAJC3	DnaJ (Hsp40) homolog, subfamily C, member 3	0.97418	0.983705	1.0283603
5612	PRKRIR	protein-kinase, interferon-inducible double stranded RNA dependent inhibitor, repressor of (P58 repressor)	0.97057	0.992912	1.0146865
5613	PRKX	protein kinase, X-linked	0.96032	1.012431	1.003122
5616	PRKY	protein kinase, Y-linked	0.99067	1.000532	1.0296952
5617	PRL	prolactin	0.99185	0.955466	1.0306525
5618	PRLR	prolactin receptor	1.00374	0.95877	1.0021435
5619	PRM1	protamine 1	0.97526	0.917833	0.9415247
5620	PRM2	protamine 2	0.99466	1.060249	1.015873
5621	PRNP	prion protein	0.99471	0.96068	0.9254837
5623	PSPN	persephin	0.98331	0.973237	0.9763547
5624	PROC	protein C (inactivator of coagulation factors Va and VIIIa)	1.0012	0.984409	0.9844056
5625	PRODH	proline dehydrogenase (oxidase) 1	0.99606	1.012393	1.0298848
5626	PROP1	PROP paired-like homeobox 1	0.99501	1.017987	1.0362076
5627	PROS1	protein S (alpha)	0.97126	1.008084	0.9707799
5628	PROSP	protein S pseudogene (beta)	0.987	0.973537	0.9760326
5629	PROX1	prospero homeobox 1	0.99944	1.018455	1.0006162
5630	PRPH	peripherin	0.99776	1.028556	1.0410157
5631	PRPS1	phosphoribosyl pyrophosphate synthetase 1	0.91041	1.024753	1.0541528
5634	PRPS2	phosphoribosyl pyrophosphate synthetase 2	0.95838	0.963595	1.0359725
5635	PRPSAP1	phosphoribosyl pyrophosphate synthetase-associated protein 1	0.91593	0.896828	1.0209022
5636	PRPSAP2	phosphoribosyl pyrophosphate synthetase-associated protein 2	0.91571	1.021727	0.9847831
5638	PRRG1	proline rich Gla (G-carboxyglutamic acid) 1	1.00027	0.997835	0.9470241
5639	PRRG2	proline rich Gla (G-carboxyglutamic acid) 2	0.97743	1.008841	1.037442
5641	LGMN	legumain	0.97127	1.02261	0.9922071
5646	PRSS3	protease, serine, 3	1.00074	1.010924	0.9955038
5648	MASP1	mannan-binding lectin serine peptidase 1 (C4/C2 activating component of Ra-reactive factor)	1.01159	1.035724	1.0090107
5649	RELN	reelin	1.00947	0.994495	1.0062
5650	KLK7	kallikrein-related peptidase 7	1.00738	1.034609	1.0252602
5651	TMPRSS15	transmembrane protease, serine 15	0.99179	1.001285	0.9961984
5652	PRSS8	protease, serine, 8	0.98986	0.971814	1.0254133
5653	KLK6	kallikrein-related peptidase 6	0.98791	1.020969	1.0048881

5654	HTRA1	HtrA serine peptidase 1	0.99991	0.993113	1.0100582
5655	KLK10	kallikrein-related peptidase 10	0.99737	0.992492	1.0211176
5657	PRTN3	proteinase 3	0.996	0.952349	1.0287789
5660	PSAP	prosaposin	1.00947	1.035792	0.9873889
5662	PSD	pleckstrin and Sec7 domain containing	0.98793	1.033912	1.0217655
5663	PSEN1	presenilin 1	0.9397	1.059393	1.0399656
5664	PSEN2	presenilin 2 (Alzheimer disease 4)	0.99125	0.938169	1.0287457
5669	PSG1	pregnancy specific beta-1-glycoprotein 1	1.01804	1.061685	1.0558713
5671	PSG3	pregnancy specific beta-1-glycoprotein 3	1.00112	1.009124	1.0248139
5672	PSG4	pregnancy specific beta-1-glycoprotein 4	0.998	1.083451	0.988314
5673	PSG5	pregnancy specific beta-1-glycoprotein 5	0.98944	1.040841	1.0006615
5675	PSG6	pregnancy specific beta-1-glycoprotein 6	1.01903	0.989922	0.9404977
5676	PSG7	pregnancy specific beta-1-glycoprotein 7 (gene/pseudogene)	1.01924	1.020575	1.0452932
5678	PSG9	pregnancy specific beta-1-glycoprotein 9	1.02379	0.963632	1.0071233
5681	PSKH1	protein serine kinase H1	0.98571	0.956444	1.020365
5682	PSMA1	proteasome (prosome, macropain) subunit, alpha type, 1	0.97328	0.989035	0.9101363
5683	PSMA2	proteasome (prosome, macropain) subunit, alpha type, 2	0.99063	0.946523	1.0389395
5684	PSMA3	proteasome (prosome, macropain) subunit, alpha type, 3	0.96011	0.963398	1.0612576
5685	PSMA4	proteasome (prosome, macropain) subunit, alpha type, 4	0.99172	1.035626	1.0542136
5686	PSMA5	proteasome (prosome, macropain) subunit, alpha type, 5	1.00603	0.966477	0.9895792
5687	PSMA6	proteasome (prosome, macropain) subunit, alpha type, 6	0.99898	1.043194	0.9831346
5688	PSMA7	proteasome (prosome, macropain) subunit, alpha type, 7	0.9191	0.995987	0.9671358
5689	PSMB1	proteasome (prosome, macropain) subunit, beta type, 1	1.00356	1.025796	1.0005313
5690	PSMB2	proteasome (prosome, macropain) subunit, beta type, 2	1.02936	0.98249	1.0233913
5691	PSMB3	proteasome (prosome, macropain) subunit, beta type, 3	0.95901	0.941077	1.0518768
5692	PSMB4	proteasome (prosome, macropain) subunit, beta type, 4	0.99612	0.958486	0.9544829
5693	PSMB5	proteasome (prosome, macropain) subunit, beta type, 5	0.95388	0.985455	0.999616
5694	PSMB6	proteasome (prosome, macropain) subunit, beta type, 6	0.96791	0.919018	1.1365127
5695	PSMB7	proteasome (prosome, macropain) subunit, beta type, 7	0.96657	0.897758	1.0237127
5696	PSMB8	proteasome (prosome, macropain) subunit, beta type, 8 (large multifunctional peptidase 7)	1.00989	0.820785	1.1590411
5697	PYY	peptide YY	1.00095	1.027775	1.014314
5698	PSMB9	proteasome (prosome, macropain) subunit, beta type, 9 (large multifunctional peptidase 2)	1.02808	0.903019	1.077205
5699	PSMB10	proteasome (prosome, macropain) subunit, beta type, 10	0.93895	0.938005	1.0091203

5700	PSMC1	proteasome (prosome, macropain) 26S subunit, ATPase, 1	1.00435	0.936476	1.0340165
5701	PSMC2	proteasome (prosome, macropain) 26S subunit, ATPase, 2	0.99883	0.897714	0.9819745
5702	PSMC3	proteasome (prosome, macropain) 26S subunit, ATPase, 3	0.91079	0.947368	1.1014879
5703	PSMC3P	proteasome (prosome, macropain) 26S subunit, ATPase, 3 pseudogene	0.98407	0.994608	1.0213663
5704	PSMC4	proteasome (prosome, macropain) 26S subunit, ATPase, 4	0.91288	0.96522	0.9795045
5705	PSMC5	proteasome (prosome, macropain) 26S subunit, ATPase, 5	0.93612	0.98253	1.0316502
5706	PSMC6	proteasome (prosome, macropain) 26S subunit, ATPase, 6	0.98899	0.964645	0.9843733
5707	PSMD1	proteasome (prosome, macropain) 26S subunit, non-ATPase, 1	1.00206	0.977554	1.0473761
5708	PSMD2	proteasome (prosome, macropain) 26S subunit, non-ATPase, 2	0.97295	1.043967	0.9646402
5709	PSMD3	proteasome (prosome, macropain) 26S subunit, non-ATPase, 3	0.97118	0.984572	1.0117358
5710	PSMD4	proteasome (prosome, macropain) 26S subunit, non-ATPase, 4	1.02078	1.000178	1.0096899
5711	PSMD5	proteasome (prosome, macropain) 26S subunit, non-ATPase, 5	0.91537	1.068349	0.9555223
5713	PSMD7	proteasome (prosome, macropain) 26S subunit, non-ATPase, 7	0.95339	0.925309	1.0697857
5714	PSMD8	proteasome (prosome, macropain) 26S subunit, non-ATPase, 8	0.92165	0.958698	1.0422269
5715	PSMD9	proteasome (prosome, macropain) 26S subunit, non-ATPase, 9	0.96737	0.98503	1.0670384
5716	PSMD10	proteasome (prosome, macropain) 26S subunit, non-ATPase, 10	0.94195	0.970537	1.0611931
5717	PSMD11	proteasome (prosome, macropain) 26S subunit, non-ATPase, 11	0.94574	1.009408	1.0251967
5718	PSMD12	proteasome (prosome, macropain) 26S subunit, non-ATPase, 12	0.9814	1.023161	1.0236802
5719	PSMD13	proteasome (prosome, macropain) 26S subunit, non-ATPase, 13	0.94727	1.060443	0.9850436
5720	PSME1	proteasome (prosome, macropain) activator subunit 1 (PA28 alpha)	0.96225	1.039061	1.0589779
5721	PSME2	proteasome (prosome, macropain) activator subunit 2 (PA28 beta)	0.94225	1.047835	1.1202667
5723	PSPH	phosphoserine phosphatase	1.00095	0.994119	1.0001874
5725	PTBP1	polypyrimidine tract binding protein 1	0.96323	0.913601	1.0696049
5726	TAS2R38	taste receptor, type 2, member 38	1.03092	1.02914	1.0176626
5727	PTCH1	patched 1	0.98043	1.091826	1.0017821
5728	PTEN	phosphatase and tensin homolog	1.03057	1.074706	1.0831981
5729	PTGDR	prostaglandin D2 receptor (DP)	1.00075	0.970785	0.9047324
5730	PTGDS	prostaglandin D2 synthase 21kDa (brain)	1.00461	1.001903	1.043495
5731	PTGER1	prostaglandin E receptor 1 (subtype EP1), 42kDa	0.99244	1.052513	1.0095444
5732	PTGER2	prostaglandin E receptor 2 (subtype EP2), 53kDa	1.00082	0.938752	1.0384125
5733	PTGER3	prostaglandin E receptor 3 (subtype EP3)	1.00691	0.984219	1.0099201
5734	PTGER4	prostaglandin E receptor 4 (subtype EP4)	0.98059	0.959565	0.9365097
5737	PTGFR	prostaglandin F receptor (FP)	1.0047	0.984001	0.9962924

5738	PTGFRN	prostaglandin F2 receptor negative regulator	0.98808	0.969293	1.0005291
5739	PTGIR	prostaglandin I2 (prostacyclin) receptor (IP)	0.97073	0.973348	0.9900849
5740	PTGIS	prostaglandin I2 (prostacyclin) synthase	1.00838	1.020529	1.0134051
5741	PTH	parathyroid hormone	0.99282	0.993901	1.0302406
5742	PTGS1	prostaglandin-endoperoxide synthase 1 (prostaglandin G/H synthase and cyclooxygenase)	0.95393	0.939727	0.9652556
5743	PTGS2	prostaglandin-endoperoxide synthase 2 (prostaglandin G/H synthase and cyclooxygenase)	1.01703	1.066003	0.87568
5745	PTH1R	parathyroid hormone 1 receptor	0.98874	1.008973	1.0430239
5746	PTH2R	parathyroid hormone 2 receptor	0.99344	0.992898	0.9908969
5747	PTK2	PTK2 protein tyrosine kinase 2	0.97373	0.963577	0.9760095
5753	PTK6	PTK6 protein tyrosine kinase 6	0.99516	0.986358	1.0390369
5754	PTK7	PTK7 protein tyrosine kinase 7	1.01252	0.975533	1.0159644
5756	TWF1	twinfilin, actin-binding protein, homolog 1 (Drosophila)	0.97337	1.034317	0.9663288
5757	PTMA	prothymosin, alpha	1.01556	1.057217	0.9406848
5760	PTMAP3	prothymosin, alpha pseudogene 3 (gene sequence 34)	0.93379	0.996762	1.1275102
5763	PTMS	parathymosin	0.96629	1.006345	1.0554468
5764	PTN	pleiotrophin	1.01124	0.990485	1.0033137
5768	QSOX1	quiescin Q6 sulfhydryl oxidase 1	1.02161	0.931621	0.979273
5769	PTP4A2P2	protein tyrosine phosphatase type IVA, member 2 pseudogene 2	0.89028	0.857648	0.8679798
5770	PTPN1	protein tyrosine phosphatase, non-receptor type 1	0.98235	0.982706	0.9846574
5771	PTPN2	protein tyrosine phosphatase, non-receptor type 2	0.97517	0.980362	0.9848211
5774	PTPN3	protein tyrosine phosphatase, non-receptor type 3	0.98501	1.00423	1.0050107
5775	PTPN4	protein tyrosine phosphatase, non-receptor type 4 (megakaryocyte)	1.00035	0.971667	0.864804
5777	PTPN6	protein tyrosine phosphatase, non-receptor type 6	0.98442	0.984595	1.1047821
5778	PTPN7	protein tyrosine phosphatase, non-receptor type 7	0.99588	0.906459	0.9539672
5780	PTPN9	protein tyrosine phosphatase, non-receptor type 9	0.95097	0.944226	1.0652737
5781	PTPN11	protein tyrosine phosphatase, non-receptor type 11	0.99316	0.992652	1.0220619
5782	PTPN12	protein tyrosine phosphatase, non-receptor type 12	1.02239	1.03019	1.0362588
5783	PTPN13	protein tyrosine phosphatase, non-receptor type 13 (APO-1/CD95 (Fas)-associated phosphatase)	1.01812	1.000705	1.0335452
5784	PTPN14	protein tyrosine phosphatase, non-receptor type 14	1.0016	0.999808	1.0072255
5786	PTPRA	protein tyrosine phosphatase, receptor type, A	0.94825	0.999348	0.9924753
5787	PTPRB	protein tyrosine phosphatase, receptor type, B	0.9945	0.995937	1.0165614
5788	PTPRC	protein tyrosine phosphatase, receptor type, C	1.03643	1.085123	1.0227019
5789	PTPRD	protein tyrosine phosphatase, receptor type, D	0.99452	0.999811	1.0091157

5790	PTPRCAP	protein tyrosine phosphatase, receptor type, C-associated protein	0.93891	1.005024	1.0755643
5791	PTPRE	protein tyrosine phosphatase, receptor type, E	0.98628	1.027856	0.9753013
5792	PTPRF	protein tyrosine phosphatase, receptor type, F	1.00857	1.011404	1.0116959
5793	PTPRG	protein tyrosine phosphatase, receptor type, G	1.01148	0.99858	1.0040317
5794	PTPRH	protein tyrosine phosphatase, receptor type, H	1.00327	1.007301	1.0029101
5795	PTPRJ	protein tyrosine phosphatase, receptor type, J	1.0046	1.144046	1.0133761
5797	PTPRM	protein tyrosine phosphatase, receptor type, M	0.99973	1.003288	1.014724
5798	PTPRN	protein tyrosine phosphatase, receptor type, N	1.01186	1.007325	1.0233405
5799	PTPRN2	protein tyrosine phosphatase, receptor type, N polypeptide 2	1.00321	0.998799	1.0108963
5800	PTPRO	protein tyrosine phosphatase, receptor type, O	0.99335	1.019353	1.0152678
5801	PTPRR	protein tyrosine phosphatase, receptor type, R	0.99581	0.993297	1.0193253
5802	PTPRS	protein tyrosine phosphatase, receptor type, S	0.98894	0.999908	0.9820671
5803	PTPRZ1	protein tyrosine phosphatase, receptor-type, Z polypeptide 1	1.00981	1.007955	1.0072639
5805	PTS	6-pyruvoyltetrahydropterin synthase	1.02529	0.943951	0.9000464
5806	PTX3	pentraxin 3, long	1.01779	1.044343	0.868064
5810	RAD1	RAD1 homolog (S. pombe)	0.98708	0.937052	1.0361942
5813	PURA	purine-rich element binding protein A	0.9791	0.922004	0.9770487
5814	PURB	purine-rich element binding protein B	0.98233	1.003479	1.0067702
5816	PVALB	parvalbumin	0.88987	0.955255	0.9345701
5817	PVR	poliovirus receptor	0.97077	0.933436	1.0124674
5818	PVRL1	poliovirus receptor-related 1 (herpesvirus entry mediator C)	0.9959	0.992672	1.0157096
5819	PVRL2	poliovirus receptor-related 2 (herpesvirus entry mediator B)	0.98817	1.022198	0.9640854
5820	PVT1	Pvt1 oncogene (non-protein coding)	0.98434	0.974385	1.0082842
5822	PWP2	PWP2 periodic tryptophan protein homolog (yeast)	0.92746	1.036602	1.0133815
5824	PEX19	peroxisomal biogenesis factor 19	0.9709	0.953069	1.0141554
5825	ABCD3	ATP-binding cassette, sub-family D (ALD), member 3	0.97824	1.010557	1.0117549
5826	ABCD4	ATP-binding cassette, sub-family D (ALD), member 4	0.98687	1.007566	1.0182142
5827	PXMP2	peroxisomal membrane protein 2, 22kDa	0.97105	1.001784	1.0405713
5828	PEX2	peroxisomal biogenesis factor 2	0.9562	0.955431	0.983566
5829	PXN	paxillin	0.9775	0.996185	1.0075197
5830	PEX5	peroxisomal biogenesis factor 5	0.95319	0.989269	1.0157911
5831	PYCR1	pyrroline-5-carboxylate reductase 1	0.98508	0.999559	1.0143229
5832	ALDH18A1	aldehyde dehydrogenase 18 family, member A1	0.90442	0.924832	1.0134797

5833	PCYT2	phosphate cytidylyltransferase 2, ethanolamine	0.95952	0.914556	1.0529189
5834	PYGB	phosphorylase, glycogen; brain	0.9476	0.956877	0.9657623
5836	PYGL	phosphorylase, glycogen, liver	0.95306	0.921265	0.9896762
5837	PYGM	phosphorylase, glycogen, muscle	0.98313	0.998054	0.9563299
5858	PZP	pregnancy-zone protein	0.99908	0.996705	1.0018132
5859	QARS	glutaminyl-tRNA synthetase	1.00265	1.045478	1.1637341
5860	QDPR	quinoid dihydropteridine reductase	0.98505	0.921647	1.060277
5861	RAB1A	RAB1A, member RAS oncogene family	1.02094	1.043838	0.9689141
5862	RAB2A	RAB2A, member RAS oncogene family	0.99933	0.939452	1.0078548
5863	RGL2	ral guanine nucleotide dissociation stimulator-like 2	0.99574	1.034288	0.9837743
5864	RAB3A	RAB3A, member RAS oncogene family	0.97742	1.015633	0.9617509
5865	RAB3B	RAB3B, member RAS oncogene family	0.99929	1.024361	1.0255163
5866	RAB3IL1	RAB3A interacting protein (rabin3)-like 1	0.97594	1.008159	1.039767
5867	RAB4A	RAB4A, member RAS oncogene family	0.99497	0.96777	1.065649
5868	RAB5A	RAB5A, member RAS oncogene family	0.99546	0.978174	0.9502477
5869	RAB5B	RAB5B, member RAS oncogene family	0.93208	0.887315	1.0746728
5870	RAB6A	RAB6A, member RAS oncogene family	1.00495	1.01346	0.9925565
5871	MAP4K2	mitogen-activated protein kinase kinase kinase kinase 2	0.95101	0.958895	1.0223493
5872	RAB13	RAB13, member RAS oncogene family	0.98078	1.04734	1.0257696
5873	RAB27A	RAB27A, member RAS oncogene family	0.98141	0.973155	1.0794729
5874	RAB27B	RAB27B, member RAS oncogene family	0.91784	0.915756	1.0179992
5875	RABGGTA	Rab geranylgeranyltransferase, alpha subunit	0.96835	0.968869	0.9735092
5876	RABGGTB	Rab geranylgeranyltransferase, beta subunit	1.07548	0.966635	1.0349077
5877	RABIF	RAB interacting factor	1.01521	0.928245	1.0260924
5878	RAB5C	RAB5C, member RAS oncogene family	0.98949	1.016297	0.9894815
5879	RAC1	ras-related C3 botulinum toxin substrate 1 (rho family, small GTP binding protein Rac1)	1.00844	1.078972	0.9608342
5880	RAC2	ras-related C3 botulinum toxin substrate 2 (rho family, small GTP binding protein Rac2)	0.98217	1.057691	1.0382959
5881	RAC3	ras-related C3 botulinum toxin substrate 3 (rho family, small GTP binding protein Rac3)	1.003	1.015035	1.049499
5883	RAD9A	RAD9 homolog A (S. pombe)	0.99295	1.023888	1.0787785
5884	RAD17	RAD17 homolog (S. pombe)	0.98061	1.008082	0.9850293
5885	RAD21	RAD21 homolog (S. pombe)	0.95377	0.978623	0.9713781
5886	RAD23A	RAD23 homolog A (S. cerevisiae)	0.93881	1.023297	1.0828532
5887	RAD23B	RAD23 homolog B (S. cerevisiae)	0.94904	0.974417	1.0857704

5888	RAD51	RAD51 homolog (S. cerevisiae)	1.01283	1.036179	1.0140521
5889	RAD51C	RAD51 homolog C (S. cerevisiae)	0.97093	0.962812	1.0283784
5890	RAD51L1	RAD51-like 1 (S. cerevisiae)	0.99121	0.969056	1.039497
5891	RAGE	renal tumor antigen	0.98018	1.000946	0.9961464
5892	RAD51L3	RAD51-like 3 (S. cerevisiae)	0.95785	0.961836	0.9710084
5893	RAD52	RAD52 homolog (S. cerevisiae)	0.95207	0.999568	1.0077223
5894	RAF1	v-raf-1 murine leukemia viral oncogene homolog 1	1.01265	1.047497	0.920097
5896	RAG1	recombination activating gene 1	1.00128	0.941932	1.0101707
5897	RAG2	recombination activating gene 2	0.99075	0.980631	1.014227
5898	RALA	v-ral simian leukemia viral oncogene homolog A (ras related)	0.98997	0.963678	0.9841999
5899	RALB	v-ral simian leukemia viral oncogene homolog B (ras related; GTP binding protein)	0.9909	0.955462	1.1158957
5900	RALGDS	ral guanine nucleotide dissociation stimulator	0.97347	1.029315	0.984404
5901	RAN	RAN, member RAS oncogene family	0.93773	0.987338	1.0212462
5902	RANBP1	RAN binding protein 1	0.98556	1.029054	1.0394154
5903	RANBP2	RAN binding protein 2	1.02139	1.08032	0.9403178
5905	RANGAP1	Ran GTPase activating protein 1	0.97543	1.043545	0.9924346
5906	RAP1A	RAP1A, member of RAS oncogene family	1.0229	1.020037	0.9301353
5907	RAP1AP	RAP1A, member of RAS oncogene family pseudogene	1.02036	0.988241	0.9800487
5908	RAP1B	RAP1B, member of RAS oncogene family	0.954	1.054912	0.9508485
5909	RAP1GAP	RAP1 GTPase activating protein	0.99429	1.013263	1.0159024
5910	RAP1GDS1	RAP1, GTP-GDP dissociation stimulator 1	0.99928	0.993996	1.0190332
5911	RAP2A	RAP2A, member of RAS oncogene family	0.9771	1.042753	1.0790052
5912	RAP2B	RAP2B, member of RAS oncogene family	0.99995	0.912696	0.9700691
5913	RAPSN	receptor-associated protein of the synapse	0.99086	0.972213	1.0285664
5914	RARA	retinoic acid receptor, alpha	0.99506	1.010618	0.9791135
5915	RARB	retinoic acid receptor, beta	1.00256	0.994663	0.9995717
5916	RARG	retinoic acid receptor, gamma	0.94484	1.000415	0.9805673
5917	RARS	arginyl-tRNA synthetase	1.02018	0.990421	1.0068252
5918	RARRES1	retinoic acid receptor responder (tazarotene induced) 1	0.98402	1.01268	0.9853988
5919	RARRES2	retinoic acid receptor responder (tazarotene induced) 2	1.0102	1.023722	1.0031432
5920	RARRES3	retinoic acid receptor responder (tazarotene induced) 3	0.91978	0.910772	0.9221598
5921	RASA1	RAS p21 protein activator (GTPase activating protein) 1	0.99562	1.012926	0.9866206
5922	RASA2	RAS p21 protein activator 2	0.98077	1.109906	0.9923748

5923	RASGRF1	Ras protein-specific guanine nucleotide-releasing factor 1	0.99527	1.005834	1.0211704
5924	RASGRF2	Ras protein-specific guanine nucleotide-releasing factor 2	0.9777	0.997546	0.8969058
5925	RB1	retinoblastoma 1	0.96867	1.026137	0.9889014
5926	ARID4A	AT rich interactive domain 4A (RBP1-like)	0.93956	0.983929	1.0082464
5927	KDM5A	lysine (K)-specific demethylase 5A	0.94941	1.031686	1.0823959
5928	RBBP4	retinoblastoma binding protein 4	1.01296	0.993333	0.9748094
5929	RBBP5	retinoblastoma binding protein 5	0.96391	0.966629	1.1056188
5930	RBBP6	retinoblastoma binding protein 6	0.94504	0.911836	0.9940895
5931	RBBP7	retinoblastoma binding protein 7	0.91782	0.953704	0.9997425
5932	RBBP8	retinoblastoma binding protein 8	0.95913	1.045289	1.0673144
5933	RBL1	retinoblastoma-like 1 (p107)	0.91149	1.049784	0.9757428
5934	RBL2	retinoblastoma-like 2 (p130)	0.95103	1.004429	0.9433666
5935	RBM3	RNA binding motif (RNP1, RRM) protein 3	0.97789	1.01105	1.0595755
5936	RBM4	RNA binding motif protein 4	0.93926	0.974629	0.9771381
5937	RBMS1	RNA binding motif, single stranded interacting protein 1	1.00088	1.002372	0.989376
5938	RBMS1P1	RNA binding motif, single stranded interacting protein 1 pseudogene 1	0.88296	0.916512	0.9351475
5939	RBMS2	RNA binding motif, single stranded interacting protein 2	1.00764	0.963533	1.007522
5947	RBP1	retinol binding protein 1, cellular	1.01406	0.985484	1.0149903
5948	RBP2	retinol binding protein 2, cellular	1.01037	0.99435	1.0125238
5949	RBP3	retinol binding protein 3, interstitial	1.00335	1.03004	1.0052197
5950	RBP4	retinol binding protein 4, plasma	0.99991	0.981651	0.9968323
5954	RCN1	reticulocalbin 1, EF-hand calcium binding domain	0.98464	0.986635	1.0282051
5955	RCN2	reticulocalbin 2, EF-hand calcium binding domain	0.92394	1.032243	1.0251228
5957	RCVRN	recoverin	0.98936	1.027582	0.9621335
5959	RDH5	retinol dehydrogenase 5 (11-cis/9-cis)	1.01878	0.910451	0.9748923
5961	PRPH2	peripherin 2 (retinal degeneration, slow)	0.99576	0.990139	1.0035784
5962	RDX	radixin	0.9675	0.984295	1.0419646
5964	RDXP2	radixin pseudogene 2	1.01812	0.935751	1.0570941
5965	RECQL	RecQ protein-like (DNA helicase Q1-like)	0.94884	0.905376	1.0582948
5966	REL	v-rel reticuloendotheliosis viral oncogene homolog (avian)	1.01361	1.057974	0.9718381
5967	REG1A	regenerating islet-derived 1 alpha	0.99584	1.023858	0.9991053
5968	REG1B	regenerating islet-derived 1 beta	0.97149	1.015959	0.9906367
5969	REG1P	regenerating islet-derived 1 pseudogene	1.0101	0.992064	0.9736102

5970	RELA	v-rel reticuloendotheliosis viral oncogene homolog A (avian)	0.94499	1.024454	1.0007532
5971	RELB	v-rel reticuloendotheliosis viral oncogene homolog B	0.94968	1.030411	0.9811122
5972	REN	renin	1.01413	0.985302	1.0160229
5973	RENBP	renin binding protein	0.9657	1.016128	1.0297882
5976	UPF1	UPF1 regulator of nonsense transcripts homolog (yeast)	0.9537	0.958336	1.0060948
5977	DPF2	D4, zinc and double PHD fingers family 2	0.94393	0.96225	1.062201
5978	REST	RE1-silencing transcription factor	0.99639	0.849529	1.0057836
5979	RET	ret proto-oncogene	0.99164	1.018929	1.0087711
5980	REV3L	REV3-like, catalytic subunit of DNA polymerase zeta (yeast)	0.97708	1.005703	0.9739376
5981	RFC1	replication factor C (activator 1) 1, 145kDa	0.98523	0.929229	1.0123093
5982	RFC2	replication factor C (activator 1) 2, 40kDa	0.98161	0.972735	1.0131461
5983	RFC3	replication factor C (activator 1) 3, 38kDa	0.98283	0.958154	1.0612632
5984	RFC4	replication factor C (activator 1) 4, 37kDa	0.97943	0.932681	1.0336233
5985	RFC5	replication factor C (activator 1) 5, 36.5kDa	0.95109	0.997533	1.013203
5986	RFNG	RFNG O-fucosylpeptide 3-beta-N-acetylglucosaminyltransferase	0.97807	1.054933	1.0846499
5987	TRIM27	tripartite motif containing 27	0.98165	0.892444	1.0518911
5988	RFPL1	ret finger protein-like 1	0.98602	0.990903	0.9606376
5989	RFX1	regulatory factor X, 1 (influences HLA class II expression)	0.98672	1.009097	1.0275151
5990	RFX2	regulatory factor X, 2 (influences HLA class II expression)	0.97239	1.001727	1.0049141
5991	RFX3	regulatory factor X, 3 (influences HLA class II expression)	0.96034	0.960069	1.1436911
5992	RFX4	regulatory factor X, 4 (influences HLA class II expression)	1.00294	1.007888	1.0089405
5993	RFX5	regulatory factor X, 5 (influences HLA class II expression)	0.98432	0.985221	0.9689616
5994	RFXAP	regulatory factor X-associated protein	0.98127	1.01789	1.0467507
5995	RGR	retinal G protein coupled receptor	1.00704	0.989348	1.006913
5996	RGS1	regulator of G-protein signaling 1	1.04538	0.984284	0.8639831
5997	RGS2	regulator of G-protein signaling 2, 24kDa	1.02775	0.969204	0.972236
5998	RGS3	regulator of G-protein signaling 3	0.97955	1.017099	0.9995362
5999	RGS4	regulator of G-protein signaling 4	1.02233	1.015343	0.9911473
6000	RGS7	regulator of G-protein signaling 7	1.00465	0.996229	1.013245
6001	RGS10	regulator of G-protein signaling 10	0.96966	1.004935	0.9892676
6002	RGS12	regulator of G-protein signaling 12	0.98795	0.99875	0.9811653
6003	RGS13	regulator of G-protein signaling 13	1.00009	0.992659	1.0024625
6004	RGS16	regulator of G-protein signaling 16	1.0313	1.021802	1.0127317

6005	RHAG	Rh-associated glycoprotein	1.00428	0.980932	1.0045705
6006	RHCE	Rh blood group, CcEe antigens	0.94504	0.979228	0.9591403
6007	RHD	Rh blood group, D antigen	0.97992	0.995585	0.9673197
6009	RHEB	Ras homolog enriched in brain	1.03343	0.989922	0.9621861
6010	RHO	rhodopsin	0.9939	0.983945	1.0344078
6011	GRK1	G protein-coupled receptor kinase 1	0.99388	1.025453	1.0138853
6013	RLN1	relaxin 1	0.95965	1.080006	1.0196516
6014	RIT2	Ras-like without CAAX 2	1.00505	1.014605	1.0226316
6015	RING1	ring finger protein 1	0.95712	0.954055	0.9989011
6016	RIT1	Ras-like without CAAX 1	1.01451	0.938301	1.023714
6017	RLBP1	retinaldehyde binding protein 1	1.00206	0.994939	0.9863609
6018	RLF	rearranged L-myc fusion	0.98946	0.946659	0.9276015
6019	RLN2	relaxin 2	0.99853	1.022218	1.0425578
6035	RNASE1	ribonuclease, RNase A family, 1 (pancreatic)	0.99104	0.982169	1.0362649
6037	RNASE3	ribonuclease, RNase A family, 3	1.01601	0.922808	1.0072795
6038	RNASE4	ribonuclease, RNase A family, 4	1.00794	0.935164	0.9925953
6039	RNASE6	ribonuclease, RNase A family, k6	0.95091	0.781038	0.974709
6041	RNASEL	ribonuclease L (2',5'-oligoisoadenylate synthetase-dependent)	0.97958	0.871397	1.0006789
6045	RNF2	ring finger protein 2	0.97889	0.977712	1.0171311
6046	BRD2	bromodomain containing 2	1.01534	0.921076	1.0300724
6047	RNF4	ring finger protein 4	1.01012	0.986108	1.0153032
6048	RNF5	ring finger protein 5	0.98211	0.998714	1.0020407
6049	RNF6	ring finger protein (C3H2C3 type) 6	0.91781	0.946251	1.0863261
6050	RNH1	ribonuclease/angiogenin inhibitor 1	0.98723	0.900801	1.0366501
6051	RNPEP	arginyl aminopeptidase (aminopeptidase B)	1.03073	0.968567	1.1304795
6059	ABCE1	ATP-binding cassette, sub-family E (OABP), member 1	0.99702	0.953507	1.0546386
6070	RNU4-8P	RNA, U4 small nuclear 8, pseudogene	0.97261	1.017495	1.1340407
6075	RNU7-55P	RNA, U7 small nuclear 55 pseudogene	1.01613	0.973092	1.0177817
6090	RNY5	RNA, Ro-associated Y5	0.99846	1.046535	0.9944808
6091	ROBO1	roundabout, axon guidance receptor, homolog 1 (Drosophila)	0.99829	1.009614	1.0073959
6092	ROBO2	roundabout, axon guidance receptor, homolog 2 (Drosophila)	1.00506	1.000674	1.0148243
6093	ROCK1	Rho-associated, coiled-coil containing protein kinase 1	0.97574	0.989604	1.0661492
6094	ROM1	retinal outer segment membrane protein 1	0.99803	0.993586	1.0414849

6095	RORA	RAR-related orphan receptor A	1.00733	1.013964	0.9956525
6096	RORB	RAR-related orphan receptor B	0.99896	1.017726	0.9927935
6097	RORC	RAR-related orphan receptor C	0.99899	1.038483	1.0135032
6098	ROS1	c-ros oncogene 1 , receptor tyrosine kinase	1.01096	1.009855	0.9987144
6100	RP9	retinitis pigmentosa 9 (autosomal dominant)	1.00291	1.014734	1.022317
6101	RP1	retinitis pigmentosa 1 (autosomal dominant)	0.99435	0.979044	1.0190226
6102	RP2	retinitis pigmentosa 2 (X-linked recessive)	0.9906	1.01833	0.9535222
6103	RPGR	retinitis pigmentosa GTPase regulator	0.97011	1.035199	0.9871631
6117	RPA1	replication protein A1, 70kDa	0.91842	0.979891	0.9609492
6118	RPA2	replication protein A2, 32kDa	0.91383	1.093217	0.971213
6119	RPA3	replication protein A3, 14kDa	0.99339	0.987294	1.0251891
6120	RPE	ribulose-5-phosphate-3-epimerase	1.00099	0.929645	1.0466148
6121	RPE65	retinal pigment epithelium-specific protein 65kDa	1.02244	1.000448	0.9967219
6122	RPL3	ribosomal protein L3	0.98272	0.916575	0.9902981
6123	RPL3L	ribosomal protein L3-like	0.99217	0.996047	1.0302542
6124	RPL4	ribosomal protein L4	0.92867	0.943465	0.9861744
6125	RPL5	ribosomal protein L5	1.04878	0.990112	0.942019
6128	RPL6	ribosomal protein L6	0.95626	1.034797	0.9985363
6129	RPL7	ribosomal protein L7	1.00614	0.955566	1.0336466
6130	RPL7A	ribosomal protein L7a	0.99068	0.954414	1.0130334
6132	RPL8	ribosomal protein L8	1.00313	0.986626	1.0221988
6133	RPL9	ribosomal protein L9	1.04963	0.941872	0.9356027
6134	RPL10	ribosomal protein L10	1.02048	1.003114	0.984726
6135	RPL11	ribosomal protein L11	1.03411	0.932671	0.947524
6136	RPL12	ribosomal protein L12	0.9898	1.001496	0.9876847
6137	RPL13	ribosomal protein L13	0.95609	1.028822	1.0080238
6138	RPL15	ribosomal protein L15	0.99461	0.959616	1.0488846
6141	RPL18	ribosomal protein L18	0.96554	0.996701	0.9774386
6142	RPL18A	ribosomal protein L18a	0.95008	0.952249	0.9619453
6143	RPL19	ribosomal protein L19	1.01122	0.9461	0.9893736
6144	RPL21	ribosomal protein L21	0.98741	1.016944	1.0158514
6146	RPL22	ribosomal protein L22	0.99259	0.901245	1.0223182
6147	RPL23A	ribosomal protein L23a	0.984	0.936215	1.0505625

6148	RPL23AP1	ribosomal protein L23a pseudogene 1	1.07246	1.04855	0.7949712
6150	MRPL23	mitochondrial ribosomal protein L23	0.95314	0.953949	1.1011866
6152	RPL24	ribosomal protein L24	1.03579	0.911644	1.0001842
6154	RPL26	ribosomal protein L26	0.94501	0.979524	1.0814961
6155	RPL27	ribosomal protein L27	0.9815	0.932268	0.9618777
6156	RPL30	ribosomal protein L30	0.94177	1.064317	0.8733383
6157	RPL27A	ribosomal protein L27a	0.9267	0.970667	0.97643
6158	RPL28	ribosomal protein L28	0.97189	0.978273	1.0088887
6159	RPL29	ribosomal protein L29	0.99671	0.992588	0.9960259
6160	RPL31	ribosomal protein L31	0.99362	1.024707	0.9977572
6161	RPL32	ribosomal protein L32	1.00732	0.974608	0.9814795
6164	RPL34	ribosomal protein L34	1.02685	0.961001	1.0041754
6165	RPL35A	ribosomal protein L35a	1.07743	0.950859	1.0343359
6166	RPL36AL	ribosomal protein L36a-like	0.94519	0.900327	1.0664039
6167	RPL37	ribosomal protein L37	1.02366	0.936462	1.0489329
6168	RPL37A	ribosomal protein L37a	0.99923	1.026469	0.9977328
6169	RPL38	ribosomal protein L38	0.99131	0.97312	1.000969
6170	RPL39	ribosomal protein L39	0.98995	0.948745	0.9619713
6171	RPL41	ribosomal protein L41	0.95444	0.951705	1.0664235
6173	RPL36A	ribosomal protein L36a	1.01327	0.996285	0.9660877
6175	RPLP0	ribosomal protein, large, P0	0.98546	0.949384	1.0244137
6176	RPLP1	ribosomal protein, large, P1	0.97047	0.954312	1.0237497
6181	RPLP2	ribosomal protein, large, P2	0.98645	0.992786	1.0006808
6182	MRPL12	mitochondrial ribosomal protein L12	0.98735	1.012127	1.0398429
6183	MRPS12	mitochondrial ribosomal protein S12	0.95315	0.919585	1.0214982
6184	RPN1	ribophorin I	1.0046	0.973516	1.1355378
6185	RPN2	ribophorin II	0.95069	1.053909	1.0555556
6187	RPS2	ribosomal protein S2	0.93494	0.957353	1.0650675
6188	RPS3	ribosomal protein S3	0.97531	0.952108	0.9802166
6189	RPS3A	ribosomal protein S3A	1.03991	0.89108	0.9214298
6191	RPS4X	ribosomal protein S4, X-linked	0.93131	0.970548	0.9938889
6192	RPS4Y1	ribosomal protein S4, Y-linked 1	0.9825	0.935927	0.9835203
6193	RPS5	ribosomal protein S5	0.98283	1.004617	1.0387976

6194	RPS6	ribosomal protein S6	0.94257	0.963827	1.0044477
6195	RPS6KA1	ribosomal protein S6 kinase, 90kDa, polypeptide 1	0.97457	0.986395	0.994633
6196	RPS6KA2	ribosomal protein S6 kinase, 90kDa, polypeptide 2	0.99074	0.984523	1.0077118
6197	RPS6KA3	ribosomal protein S6 kinase, 90kDa, polypeptide 3	0.94709	0.978054	1.0291884
6198	RPS6KB1	ribosomal protein S6 kinase, 70kDa, polypeptide 1	0.97462	0.957384	1.0016181
6199	RPS6KB2	ribosomal protein S6 kinase, 70kDa, polypeptide 2	0.94372	0.997648	1.0294234
6201	RPS7	ribosomal protein S7	0.97661	0.960259	1.0123654
6202	RPS8	ribosomal protein S8	0.98074	1.068063	1.0678866
6203	RPS9	ribosomal protein S9	0.98651	0.950356	1.0267852
6204	RPS10	ribosomal protein S10	1.00463	1.035154	1.0127997
6205	RPS11	ribosomal protein S11	1.00668	1.034947	0.9889709
6206	RPS12	ribosomal protein S12	1.00176	0.895462	0.9522528
6207	RPS13	ribosomal protein S13	1.01654	1.020094	0.9950493
6208	RPS14	ribosomal protein S14	0.97665	0.942887	0.9650957
6209	RPS15	ribosomal protein S15	0.99166	0.990978	0.9915309
6210	RPS15A	ribosomal protein S15a	0.98678	0.954679	0.9335422
6217	RPS16	ribosomal protein S16	0.96503	1.021739	0.96543
6222	RPS18	ribosomal protein S18	1.02587	1.0735	0.9721264
6223	RPS19	ribosomal protein S19	0.98547	1.013303	1.0167377
6224	RPS20	ribosomal protein S20	0.96194	0.919688	0.9326912
6225	RPS20P3	ribosomal protein S20 pseudogene 3	1.0505	0.979604	0.9330562
6227	RPS21	ribosomal protein S21	0.95572	1.029408	1.0730702
6228	RPS23	ribosomal protein S23	0.9881	0.92965	1.0637152
6229	RPS24	ribosomal protein S24	0.94366	0.936256	0.9417054
6230	RPS25	ribosomal protein S25	0.99351	0.940314	0.9473238
6231	RPS26	ribosomal protein S26	0.9561	1.081129	1.011586
6232	RPS27	ribosomal protein S27	0.97052	0.950977	0.8944885
6233	RPS27A	ribosomal protein S27a	1.00244	0.947343	0.991149
6234	RPS28	ribosomal protein S28	0.92908	0.954432	0.9839742
6235	RPS29	ribosomal protein S29	1.0045	0.954111	0.9328467
6236	RRAD	Ras-related associated with diabetes	1.00199	1.003577	1.0309037
6237	RRAS	related RAS viral (r-ras) oncogene homolog	0.97309	0.916861	1.0198842
6238	RRBP1	ribosome binding protein 1 homolog 180kDa (dog)	1.00276	1.062085	1.0048673

6239	RREB1	ras responsive element binding protein 1	1.00757	1.00573	0.9524103
6240	RRM1	ribonucleotide reductase M1	0.90519	0.887921	1.0424127
6241	RRM2	ribonucleotide reductase M2	0.99816	0.934479	1.0200102
6242	RTKN	rhotekin	1.00665	1.002724	1.000235
6246	RRM2P4	ribonucleotide reductase M2 polypeptide pseudogene 4	0.9935	0.968503	1.020881
6247	RS1	retinoschisin 1	0.99429	1.010485	0.9904856
6248	RSC1A1	regulatory solute carrier protein, family 1, member 1	0.97077	0.999516	1.0406107
6249	CLIP1	CAP-GLY domain containing linker protein 1	0.97376	0.986184	0.9703158
6251	RSU1	Ras suppressor protein 1	0.97145	0.984162	0.9898999
6252	RTN1	reticulon 1	0.98362	0.962853	0.9986858
6253	RTN2	reticulon 2	0.96824	1.020067	1.0611172
6256	RXRA	retinoid X receptor, alpha	0.98228	1.011629	1.0120295
6257	RXRB	retinoid X receptor, beta	0.98409	0.962632	1.0185401
6259	RYK	RYK receptor-like tyrosine kinase	0.96378	0.967433	0.9741316
6260	RYKP1	RYK receptor-like tyrosine kinase pseudogene 1	1.00353	0.991706	1.040489
6261	RYR1	ryanodine receptor 1 (skeletal)	0.99159	0.987823	1.0217518
6262	RYR2	ryanodine receptor 2 (cardiac)	1.0035	1.008051	1.0162609
6263	RYR3	ryanodine receptor 3	0.99769	1.003273	1.0051962
6271	S100A1	S100 calcium binding protein A1	0.99852	0.981232	1.0026252
6272	SORT1	sortilin 1	1.00804	0.892876	1.1361198
6273	S100A2	S100 calcium binding protein A2	1.01788	0.999314	1.0038108
6274	S100A3	S100 calcium binding protein A3	0.99414	1.003944	1.008617
6275	S100A4	S100 calcium binding protein A4	1.02178	0.996379	0.9641302
6276	S100A5	S100 calcium binding protein A5	1.0255	1.009635	0.9805929
6277	S100A6	S100 calcium binding protein A6	0.94364	0.997145	0.993349
6278	S100A7	S100 calcium binding protein A7	0.97887	1.021003	1.0251616
6279	S100A8	S100 calcium binding protein A8	1.05778	0.936886	0.9776751
6280	S100A9	S100 calcium binding protein A9	0.973	0.889002	0.9480509
6281	S100A10	S100 calcium binding protein A10	1.03029	0.961954	0.9303929
6282	S100A11	S100 calcium binding protein A11	0.97216	0.950754	0.8859581
6283	S100A12	S100 calcium binding protein A12	1.01323	1.028742	1.0012121
6284	S100A13	S100 calcium binding protein A13	1.00962	0.976673	1.0209857
6285	S100B	S100 calcium binding protein B	0.93745	1.034072	0.9656169

6286	S100P	S100 calcium binding protein P	1.01947	0.939557	0.9603842
6288	SAA1	serum amyloid A1	1.01878	1.028692	0.9625991
6289	SAA2	serum amyloid A2	0.99072	0.912152	0.9538041
6290	SAA3P	serum amyloid A3 pseudogene	0.99042	0.991925	0.9816514
6291	SAA4	serum amyloid A4, constitutive	0.98471	0.987555	1.0117404
6293	VPS52	vacuolar protein sorting 52 homolog (S. cerevisiae)	0.98842	0.856143	0.9967491
6294	SAFB	scaffold attachment factor B	0.92522	1.014252	0.9682478
6295	SAG	S-antigen; retina and pineal gland (arrestin)	0.99954	0.998342	1.0134316
6296	ACSM3	acyl-CoA synthetase medium-chain family member 3	0.96277	0.968328	1.0186722
6297	SALL2	sal-like 2 (Drosophila)	0.96859	0.966977	1.0033568
6299	SALL1	sal-like 1 (Drosophila)	0.98588	1.019988	1.007184
6300	MAPK12	mitogen-activated protein kinase 12	0.98582	0.997178	1.0471663
6301	SARS	seryl-tRNA synthetase	0.98123	1.058389	0.9780493
6302	TSPAN31	tetraspanin 31	0.92743	1.039303	1.0060035
6303	SAT1	spermidine/spermine N1-acetyltransferase 1	0.94911	1.084923	0.9593265
6304	SATB1	SATB homeobox 1	0.95574	0.914915	0.913136
6305	SBF1	SET binding factor 1	0.98934	1.033033	1.03419
6307	SC4MOL	sterol-C4-methyl oxidase-like	0.97164	0.994089	1.0109647
6309	SC5DL	sterol-C5-desaturase (ERG3 delta-5-desaturase homolog, S. cerevisiae)-like	0.93716	1.026384	1.0066063
6310	ATXN1	ataxin 1	1.01208	0.979822	0.98175
6311	ATXN2	ataxin 2	0.94819	1.017104	0.9794451
6314	ATXN7	ataxin 7	1.00852	0.937607	0.9672373
6315	ATXN8OS	ATXN8 opposite strand (non-protein coding)	0.99044	1.025517	1.0041248
6318	SERPINB4	serpin peptidase inhibitor, clade B (ovalbumin), member 4	0.98253	1.00581	0.9940364
6319	SCD	stearoyl-CoA desaturase (delta-9-desaturase)	0.98381	1.05902	1.0243525
6320	CLEC11A	C-type lectin domain family 11, member A	0.98084	1.074995	1.0225473
6322	SCML1	sex comb on midleg-like 1 (Drosophila)	0.96399	1.02509	0.8610254
6323	SCN1A	sodium channel, voltage-gated, type I, alpha subunit	1.00153	1.011568	1.0301966
6324	SCN1B	sodium channel, voltage-gated, type I, beta	0.98986	1.015486	0.9420215
6326	SCN2A	sodium channel, voltage-gated, type II, alpha subunit	1.00474	1.007608	1.017618
6327	SCN2B	sodium channel, voltage-gated, type II, beta	1.02197	0.98891	1.0203085
6328	SCN3A	sodium channel, voltage-gated, type III, alpha subunit	0.98046	1.017477	0.9763575
6329	SCN4A	sodium channel, voltage-gated, type IV, alpha subunit	0.99421	1.000639	1.0229496

6330	SCN4B	sodium channel, voltage-gated, type IV, beta	0.99891	1.023066	0.9750603
6331	SCN5A	sodium channel, voltage-gated, type V, alpha subunit	0.99911	0.995485	0.9780068
6332	SCN7A	sodium channel, voltage-gated, type VII, alpha	1.00567	1.007338	1.0296008
6334	SCN8A	sodium channel, voltage gated, type VIII, alpha subunit	1.00009	1.012046	1.0006618
6335	SCN9A	sodium channel, voltage-gated, type IX, alpha subunit	1.01885	0.96783	0.9898778
6336	SCN10A	sodium channel, voltage-gated, type X, alpha subunit	1.00538	1.000185	1.0214323
6337	SCNN1A	sodium channel, nonvoltage-gated 1 alpha	0.98352	0.999004	0.9646313
6338	SCNN1B	sodium channel, nonvoltage-gated 1, beta	0.99566	1.008604	1.012145
6339	SCNN1D	sodium channel, nonvoltage-gated 1, delta	0.99852	1.001555	1.0231793
6340	SCNN1G	sodium channel, nonvoltage-gated 1, gamma	0.99158	0.998177	0.9975466
6341	SCO1	SCO cytochrome oxidase deficient homolog 1 (yeast)	0.95366	0.992379	1.0229938
6342	SCP2	sterol carrier protein 2	1.00792	0.930015	1.0867017
6343	SCT	secretin	1.0048	1.038624	0.9968479
6344	SCTR	secretin receptor	1.00513	1.000544	1.0264298
6345	SRL	sarcalumenin	0.98588	1.030016	1.021366
6346	CCL1	chemokine (C-C motif) ligand 1	0.98553	0.986051	1.0060136
6347	CCL2	chemokine (C-C motif) ligand 2	0.99221	0.983731	0.9792622
6348	CCL3	chemokine (C-C motif) ligand 3	1.06194	1.149137	0.9753366
6352	CCL5	chemokine (C-C motif) ligand 5	0.96808	1.035042	0.9763198
6354	CCL7	chemokine (C-C motif) ligand 7	0.99235	1.033359	1.0060835
6355	CCL8	chemokine (C-C motif) ligand 8	1.02633	0.963371	1.0102445
6356	CCL11	chemokine (C-C motif) ligand 11	1.0304	0.966838	1.005477
6357	CCL13	chemokine (C-C motif) ligand 13	1.01079	1.017872	1.0142881
6360	CCL16	chemokine (C-C motif) ligand 16	1.01346	0.998062	0.9968179
6362	CCL18	chemokine (C-C motif) ligand 18 (pulmonary and activation-regulated)	1.03383	0.985847	1.0428546
6363	CCL19	chemokine (C-C motif) ligand 19	1.00132	0.997753	0.9691612
6364	CCL20	chemokine (C-C motif) ligand 20	1.01911	1.060101	0.9608405
6366	CCL21	chemokine (C-C motif) ligand 21	0.99012	0.954346	1.0010118
6367	CCL22	chemokine (C-C motif) ligand 22	0.97218	1.003112	1.0224554
6368	CCL23	chemokine (C-C motif) ligand 23	0.99207	0.976058	0.9856681
6369	CCL24	chemokine (C-C motif) ligand 24	1.01493	1.031498	1.0075446
6370	CCL25	chemokine (C-C motif) ligand 25	0.9867	1.03429	1.0507553
6372	CXCL6	chemokine (C-X-C motif) ligand 6 (granulocyte chemotactic protein 2)	1.00038	1.06271	0.9825828

6373	CXCL11	chemokine (C-X-C motif) ligand 11	0.98512	0.948238	1.0471855
6374	CXCL5	chemokine (C-X-C motif) ligand 5	1.06114	1.150947	0.9431586
6375	XCL1	chemokine (C motif) ligand 1	1.02335	1.003739	0.9968366
6376	CX3CL1	chemokine (C-X3-C motif) ligand 1	0.99524	0.99681	0.9873518
6382	SDC1	syndecan 1	0.98358	1.023217	1.040654
6383	SDC2	syndecan 2	1.00142	1.002585	0.9384743
6385	SDC4	syndecan 4	0.91383	1.048751	0.9736284
6386	SDCBP	syndecan binding protein (syntenin)	1.04264	0.984465	1.0121518
6387	CXCL12	chemokine (C-X-C motif) ligand 12	0.98451	0.985358	1.0265392
6388	SDF2	stromal cell-derived factor 2	0.93126	1.025959	1.0787903
6389	SDHA	succinate dehydrogenase complex, subunit A, flavoprotein (Fp)	0.96587	0.999191	0.9626433
6390	SDHB	succinate dehydrogenase complex, subunit B, iron sulfur (Ip)	1.01547	0.895514	1.0217661
6391	SDHC	succinate dehydrogenase complex, subunit C, integral membrane protein, 15kDa	1.01198	1.008795	1.0043844
6392	SDHD	succinate dehydrogenase complex, subunit D, integral membrane protein	1.01536	0.986795	1.0449787
6396	SEC13	SEC13 homolog (S. cerevisiae)	0.99412	0.962628	0.9795179
6397	SEC14L1	SEC14-like 1 (S. cerevisiae)	0.98867	0.996573	0.9421472
6398	SECTM1	secreted and transmembrane 1	0.97119	1.011254	1.0027971
6399	TRAPPC2	trafficking protein particle complex 2	0.98558	1.002723	0.9752199
6400	SEL1L	sel-1 suppressor of lin-12-like (C. elegans)	0.97554	1.005226	1.1889233
6401	SELE	selectin E	1.0127	1.006679	1.0204658
6402	SELL	selectin L	0.99399	0.89333	1.0258106
6403	SELP	selectin P (granule membrane protein 140kDa, antigen CD62)	0.95256	0.887609	1.0409856
6404	SELPLG	selectin P ligand	0.94306	0.987633	1.035694
6405	SEMA3F	sema domain, immunoglobulin domain (Ig), short basic domain, secreted, (semaphorin) 3F	1.01349	1.023858	0.9811068
6406	SEMG1	semenogelin I	1.02295	1.021417	1.010331
6407	SEMG2	semenogelin II	1.00089	1.021338	0.985134
6414	SEPP1	selenoprotein P, plasma, 1	1.00119	1.004621	1.0236007
6415	SEPW1	selenoprotein W, 1	0.95952	0.974949	1.0269133
6416	MAP2K4	mitogen-activated protein kinase kinase 4	0.94487	1.001542	0.9500918
6418	SET	SET nuclear oncogene	0.96316	0.99235	1.035836
6419	SETMAR	SET domain and mariner transposase fusion gene	0.97197	0.907375	1.0456374
6421	SFPQ	splicing factor proline/glutamine-rich	1.02694	0.978948	0.9872124
6422	SFRP1	secreted frizzled-related protein 1	0.98829	0.992848	0.9953139

6423	SFRP2	secreted frizzled-related protein 2	1.01361	1.004049	1.0532539
6424	SFRP4	secreted frizzled-related protein 4	1.01144	1.024413	1.0298146
6425	SFRP5	secreted frizzled-related protein 5	0.97892	0.971522	1.0093055
6426	SRSF1	serine/arginine-rich splicing factor 1	1.01335	1.034223	1.0250513
6427	SRSF2	serine/arginine-rich splicing factor 2	0.9862	0.90781	1.1532422
6428	SRSF3	serine/arginine-rich splicing factor 3	0.99391	0.883121	0.9573359
6429	SRSF4	serine/arginine-rich splicing factor 4	1.00686	0.978587	0.9773635
6430	SRSF5	serine/arginine-rich splicing factor 5	0.95826	0.95046	0.9966662
6431	SRSF6	serine/arginine-rich splicing factor 6	0.94016	0.96617	1.03287
6432	SRSF7	serine/arginine-rich splicing factor 7	0.98403	0.970254	1.0470918
6433	SFSWAP	splicing factor, suppressor of white-apricot homolog (Drosophila)	0.95167	0.946291	0.966355
6434	TRA2B	transformer 2 beta homolog (Drosophila)	1.01861	0.872724	0.9003811
6439	SFTPB	surfactant protein B	0.99875	1.006841	1.0152183
6440	SFTPC	surfactant protein C	1.00864	1.019766	1.0151522
6441	SFTPD	surfactant protein D	1.01133	1.000876	0.9995286
6442	SGCA	sarcoglycan, alpha (50kDa dystrophin-associated glycoprotein)	1.00441	1.005509	1.0027922
6443	SGCB	sarcoglycan, beta (43kDa dystrophin-associated glycoprotein)	0.98409	0.934786	1.0186625
6444	SGCD	sarcoglycan, delta (35kDa dystrophin-associated glycoprotein)	0.99508	1.000749	0.9825256
6445	SGCG	sarcoglycan, gamma (35kDa dystrophin-associated glycoprotein)	1.0066	1.005505	1.022345
6446	SGK1	serum/glucocorticoid regulated kinase 1	0.99557	1.134638	0.9120302
6448	SGSH	N-sulfoglucosamine sulfohydrolase	0.9759	1.038259	0.9806517
6449	SGTA	small glutamine-rich tetratricopeptide repeat (TPR)-containing, alpha	0.9559	1.005368	1.0093356
6450	SH3BGR	SH3 domain binding glutamic acid-rich protein	1.0048	1.005007	0.993977
6451	SH3BGRL	SH3 domain binding glutamic acid-rich protein like	0.96813	0.969998	1.0148204
6452	SH3BP2	SH3-domain binding protein 2	1.02911	1.026012	0.9547457
6453	ITSN1	intersectin 1 (SH3 domain protein)	0.98108	0.967513	0.9859844
6455	SH3GL1	SH3-domain GRB2-like 1	0.96926	0.970874	1.0070353
6456	SH3GL2	SH3-domain GRB2-like 2	1.00805	1.009656	0.9930971
6457	SH3GL3	SH3-domain GRB2-like 3	1.01367	1.001129	0.9911931
6461	SHB	Src homology 2 domain containing adaptor protein B	1.01957	1.008131	0.9838146
6462	SHBG	sex hormone-binding globulin	0.98622	1.0265	1.0193375
6464	SHC1	SHC (Src homology 2 domain containing) transforming protein 1	0.99373	1.01331	0.9471629
6466	SHC1P2	SHC (Src homology 2 domain containing) transforming protein 1 pseudogene 2	0.99105	0.999303	1.0220799

6468	FBXW4	F-box and WD repeat domain containing 4	0.98816	1.047849	0.987583
6469	SHH	sonic hedgehog	0.97732	0.994888	1.0656184
6470	SHMT1	serine hydroxymethyltransferase 1 (soluble)	0.98586	0.964594	1.0176511
6472	SHMT2	serine hydroxymethyltransferase 2 (mitochondrial)	0.94796	0.931939	1.0251932
6474	SHOX2	short stature homeobox 2	1.00995	1.035705	1.0731815
6476	SI	sucrase-isomaltase (alpha-glucosidase)	0.99686	0.9943	1.0266967
6477	SIAH1	seven in absentia homolog 1 (Drosophila)	0.99195	0.940977	0.9556678
6478	SIAH2	seven in absentia homolog 2 (Drosophila)	1.00717	0.979492	0.993377
6480	ST6GAL1	ST6 beta-galactosamide alpha-2,6-sialyltranferase 1	0.98094	1.063	0.9429604
6482	ST3GAL1	ST3 beta-galactoside alpha-2,3-sialyltransferase 1	0.97185	1.075762	0.9564789
6483	ST3GAL2	ST3 beta-galactoside alpha-2,3-sialyltransferase 2	0.96908	0.999921	1.0779992
6484	ST3GAL4	ST3 beta-galactoside alpha-2,3-sialyltransferase 4	0.98993	1.028888	1.0199938
6487	ST3GAL3	ST3 beta-galactoside alpha-2,3-sialyltransferase 3	0.98663	0.974847	1.0220548
6489	ST8SIA1	ST8 alpha-N-acetyl-neuraminide alpha-2,8-sialyltransferase 1	0.98234	0.987379	1.000087
6490	PMEL	premelanosome protein	0.98879	0.998736	0.9783366
6491	STIL	SCL/TAL1 interrupting locus	0.98687	0.952776	1.0248797
6492	SIM1	single-minded homolog 1 (Drosophila)	0.99468	1.002317	1.027362
6493	SIM2	single-minded homolog 2 (Drosophila)	0.99547	0.980297	1.0072087
6494	SIPA1	signal-induced proliferation-associated 1	0.9804	1.106294	0.9709316
6495	SIX1	SIX homeobox 1	1.00888	0.985615	0.9887122
6496	SIX3	SIX homeobox 3	1.00108	0.9842	1.052901
6497	SKI	v-ski sarcoma viral oncogene homolog (avian)	0.98207	1.016349	0.943304
6498	SKIL	SKI-like oncogene	1.00425	1.063425	0.9717838
6499	SKIV2L	superkiller viralicidic activity 2-like (S. cerevisiae)	0.97285	1.023823	0.9791254
6500	SKP1	S-phase kinase-associated protein 1	1.02048	0.978295	0.8406655
6502	SKP2	S-phase kinase-associated protein 2 (p45)	0.9899	0.996362	1.0419518
6503	SLA	Src-like-adaptor	0.95987	1.164529	0.9667983
6504	SLAMF1	signaling lymphocytic activation molecule family member 1	0.91981	0.964601	1.0350189
6505	SLC1A1	solute carrier family 1 (neuronal/epithelial high affinity glutamate transporter, system Xag), member 1	0.99074	0.989706	1.0100444
6506	SLC1A2	solute carrier family 1 (glial high affinity glutamate transporter), member 2	1.01373	1.009618	1.0090464
6507	SLC1A3	solute carrier family 1 (glial high affinity glutamate transporter), member 3	1.01305	1.008633	1.0012945
6508	SLC4A3	solute carrier family 4, anion exchanger, member 3	1.00715	1.007834	0.9972388
6509	SLC1A4	solute carrier family 1 (glutamate/neutral amino acid transporter), member 4	0.99983	1.002254	1.0422938

6510	SLC1A5	solute carrier family 1 (neutral amino acid transporter), member 5	0.97505	0.971253	1.1586675
6511	SLC1A6	solute carrier family 1 (high affinity aspartate/glutamate transporter), member 6	1.0093	1.000089	1.006632
6512	SLC1A7	solute carrier family 1 (glutamate transporter), member 7	1.00493	1.019487	1.0193638
6513	SLC2A1	solute carrier family 2 (facilitated glucose transporter), member 1	1.00886	1.017651	1.127075
6514	SLC2A2	solute carrier family 2 (facilitated glucose transporter), member 2	0.99523	1.014646	1.012775
6515	SLC2A3	solute carrier family 2 (facilitated glucose transporter), member 3	0.96522	0.964266	0.9361448
6517	SLC2A4	solute carrier family 2 (facilitated glucose transporter), member 4	0.97874	0.996298	0.9995148
6518	SLC2A5	solute carrier family 2 (facilitated glucose/fructose transporter), member 5	1.00799	1.015797	1.0285169
6519	SLC3A1	solute carrier family 3 (cystine, dibasic and neutral amino acid transporters, activator of cystine, dibasic and r	0.99098	0.995867	0.9755299
6520	SLC3A2	solute carrier family 3 (activators of dibasic and neutral amino acid transport), member 2	0.97243	0.950486	1.0211404
6521	SLC4A1	solute carrier family 4, anion exchanger, member 1 (erythrocyte membrane protein band 3, Diego blood grou	1.00129	0.812909	0.9895202
6522	SLC4A2	solute carrier family 4, anion exchanger, member 2 (erythrocyte membrane protein band 3-like 1)	1.01846	0.997821	1.109229
6523	SLC5A1	solute carrier family 5 (sodium/glucose cotransporter), member 1	1.01642	0.981659	0.9941488
6524	SLC5A2	solute carrier family 5 (sodium/glucose cotransporter), member 2	0.99437	1.027443	1.0343744
6525	SMTN	smoothelin	0.99027	1.000555	1.0301045
6527	SLC5A4	solute carrier family 5 (low affinity glucose cotransporter), member 4	0.99594	0.977795	0.9978727
6528	SLC5A5	solute carrier family 5 (sodium iodide symporter), member 5	0.9852	1.008787	1.0095579
6529	SLC6A1	solute carrier family 6 (neurotransmitter transporter, GABA), member 1	1.00291	1.021412	1.0363021
6530	SLC6A2	solute carrier family 6 (neurotransmitter transporter, noradrenalin), member 2	0.99274	1.00433	1.0112984
6531	SLC6A3	solute carrier family 6 (neurotransmitter transporter, dopamine), member 3	0.99527	0.999256	1.0450281
6532	SLC6A4	solute carrier family 6 (neurotransmitter transporter, serotonin), member 4	0.95345	0.947306	0.9933109
6533	SLC6A6	solute carrier family 6 (neurotransmitter transporter, taurine), member 6	1.0244	1.002055	1.0030737
6534	SLC6A7	solute carrier family 6 (neurotransmitter transporter, L-proline), member 7	0.99562	1.031139	1.0447237
6535	SLC6A8	solute carrier family 6 (neurotransmitter transporter, creatine), member 8	1.00043	1.015803	1.0662009
6536	SLC6A9	solute carrier family 6 (neurotransmitter transporter, glycine), member 9	0.99784	1.016364	1.0232382
6538	SLC6A11	solute carrier family 6 (neurotransmitter transporter, GABA), member 11	1.00364	1.005336	1.0121671
6539	SLC6A12	solute carrier family 6 (neurotransmitter transporter, betaine/GABA), member 12	0.99001	0.989786	0.9687458
6540	SLC6A13	solute carrier family 6 (neurotransmitter transporter, GABA), member 13	1	1.010864	1.0221085
6541	SLC7A1	solute carrier family 7 (cationic amino acid transporter, y+ system), member 1	0.97922	1.030931	0.9896516
6542	SLC7A2	solute carrier family 7 (cationic amino acid transporter, y+ system), member 2	1.01601	0.981858	1.0148165
6543	SLC8A2	solute carrier family 8 (sodium/calcium exchanger), member 2	0.98639	1.002985	1.0088378
6545	SLC7A4	solute carrier family 7 (cationic amino acid transporter, y+ system), member 4	1.01464	1.011168	0.9626866
6546	SLC8A1	solute carrier family 8 (sodium/calcium exchanger), member 1	1.04172	0.893612	0.9939118

6547	SLC8A3	solute carrier family 8 (sodium/calcium exchanger), member 3	0.98609	1.002332	0.9905333
6548	SLC9A1	solute carrier family 9 (sodium/hydrogen exchanger), member 1	1.01257	0.975778	0.9399067
6549	SLC9A2	solute carrier family 9 (sodium/hydrogen exchanger), member 2	1.01366	0.998236	1.0280792
6550	SLC9A3	solute carrier family 9 (sodium/hydrogen exchanger), member 3	1.0005	0.967017	1.0526953
6553	SLC9A5	solute carrier family 9 (sodium/hydrogen exchanger), member 5	0.99069	1.014079	0.9882167
6554	SLC10A1	solute carrier family 10 (sodium/bile acid cotransporter family), member 1	0.99198	1.006656	0.9612207
6555	SLC10A2	solute carrier family 10 (sodium/bile acid cotransporter family), member 2	0.98467	0.97231	1.0060896
6556	SLC11A1	solute carrier family 11 (proton-coupled divalent metal ion transporters), member 1	1.06201	1.018972	0.9806711
6557	SLC12A1	solute carrier family 12 (sodium/potassium/chloride transporters), member 1	1.00402	1.00746	1.0006581
6558	SLC12A2	solute carrier family 12 (sodium/potassium/chloride transporters), member 2	0.98404	0.943386	1.0200331
6559	SLC12A3	solute carrier family 12 (sodium/chloride transporters), member 3	1.00009	1.006255	1.0024427
6560	SLC12A4	solute carrier family 12 (potassium/chloride transporters), member 4	0.98277	1.034172	0.9957292
6561	SLC13A1	solute carrier family 13 (sodium/sulfate symporters), member 1	1.00655	0.988063	1.0027093
6563	SLC14A1	solute carrier family 14 (urea transporter), member 1 (Kidd blood group)	0.99588	0.924774	0.9988631
6564	SLC15A1	solute carrier family 15 (oligopeptide transporter), member 1	0.98565	1.000659	1.0210364
6565	SLC15A2	solute carrier family 15 (H+/peptide transporter), member 2	1.00097	0.935826	1.0537423
6566	SLC16A1	solute carrier family 16, member 1 (monocarboxylic acid transporter 1)	0.99525	0.980223	0.9996009
6567	SLC16A2	solute carrier family 16, member 2 (monocarboxylic acid transporter 8)	0.99973	0.993794	1.0010266
6568	SLC17A1	solute carrier family 17 (sodium phosphate), member 1	1.00342	0.991492	1.004169
6569	SLC34A1	solute carrier family 34 (sodium phosphate), member 1	1.00576	0.979743	0.9934746
6570	SLC18A1	solute carrier family 18 (vesicular monoamine), member 1	0.99582	1.011984	1.0084808
6571	SLC18A2	solute carrier family 18 (vesicular monoamine), member 2	0.97251	0.917181	1.0551273
6573	SLC19A1	solute carrier family 19 (folate transporter), member 1	0.98626	1.034822	1.0276311
6574	SLC20A1	solute carrier family 20 (phosphate transporter), member 1	1.06391	1.058827	0.9715899
6575	SLC20A2	solute carrier family 20 (phosphate transporter), member 2	0.95917	1.003483	0.9582093
6576	SLC25A1	solute carrier family 25 (mitochondrial carrier; citrate transporter), member 1	0.94441	0.930546	1.1016858
6578	SLCO2A1	solute carrier organic anion transporter family, member 2A1	1.01123	1.006148	1.014172
6579	SLCO1A2	solute carrier organic anion transporter family, member 1A2	1.00732	1.008316	0.9772907
6580	SLC22A1	solute carrier family 22 (organic cation transporter), member 1	1.00413	0.980311	0.997261
6581	SLC22A3	solute carrier family 22 (extraneuronal monoamine transporter), member 3	1.00334	1.020731	1.0032047
6582	SLC22A2	solute carrier family 22 (organic cation transporter), member 2	1.00852	1.011025	0.9878763
6583	SLC22A4	solute carrier family 22 (organic cation/ergothioneine transporter), member 4	1.02372	0.939113	0.945962
6584	SLC22A5	solute carrier family 22 (organic cation/carnitine transporter), member 5	1.00707	0.975031	0.995753

6585	SLIT1	slit homolog 1 (Drosophila)	0.99345	0.98145	1.0202844
6586	SLIT3	slit homolog 3 (Drosophila)	1.00859	1.001868	1.0088973
6588	SLN	sarcolipin	1.0173	1.039224	1.0675202
6590	SLPI	secretory leukocyte peptidase inhibitor	1.04763	1.021805	0.9829188
6591	SNAI2	snail homolog 2 (Drosophila)	0.99199	0.995595	1.0272006
6594	SMARCA1	SWI/SNF related, matrix associated, actin dependent regulator of chromatin, subfamily a, member 1	0.98771	0.991873	0.9803393
6595	SMARCA2	SWI/SNF related, matrix associated, actin dependent regulator of chromatin, subfamily a, member 2	0.94866	1.034813	1.0284299
6596	HLTF	helicase-like transcription factor	0.97389	0.953191	0.9722896
6597	SMARCA4	SWI/SNF related, matrix associated, actin dependent regulator of chromatin, subfamily a, member 4	0.96057	1.015056	1.0215936
6598	SMARCB1	SWI/SNF related, matrix associated, actin dependent regulator of chromatin, subfamily b, member 1	0.94792	0.9206	1.0181302
6599	SMARCC1	SWI/SNF related, matrix associated, actin dependent regulator of chromatin, subfamily c, member 1	0.99146	0.91233	1.0223809
6601	SMARCC2	SWI/SNF related, matrix associated, actin dependent regulator of chromatin, subfamily c, member 2	0.96024	0.964945	1.0116961
6602	SMARCD1	SWI/SNF related, matrix associated, actin dependent regulator of chromatin, subfamily d, member 1	0.91806	1.02079	1.0729858
6603	SMARCD2	SWI/SNF related, matrix associated, actin dependent regulator of chromatin, subfamily d, member 2	0.92953	0.946117	1.1122987
6604	SMARCD3	SWI/SNF related, matrix associated, actin dependent regulator of chromatin, subfamily d, member 3	0.99457	0.993172	1.0081689
6605	SMARCE1	SWI/SNF related, matrix associated, actin dependent regulator of chromatin, subfamily e, member 1	0.98443	0.920965	0.9803773
6608	SMO	smoothened, frizzled family receptor	0.99851	1.031345	1.0352645
6609	SMPD1	sphingomyelin phosphodiesterase 1, acid lysosomal	0.92027	0.991056	0.9652758
6610	SMPD2	sphingomyelin phosphodiesterase 2, neutral membrane (neutral sphingomyelinase)	0.97935	1.006688	0.9967901
6611	SMS	spermine synthase	1.00386	1.062603	1.0204294
6612	SUMO3	SMT3 suppressor of mif two 3 homolog 3 (S. cerevisiae)	0.97753	0.955869	1.0549619
6613	SUMO2	SMT3 suppressor of mif two 3 homolog 2 (S. cerevisiae)	1.03407	0.903005	1.0305123
6614	SIGLEC1	sialic acid binding Ig-like lectin 1, sialoadhesin	1.00033	0.955768	0.9885371
6615	SNAI1	snail homolog 1 (Drosophila)	0.97058	1.072404	0.9634764
6616	SNAP25	synaptosomal-associated protein, 25kDa	1.00504	1.013643	1.024517
6617	SNAPC1	small nuclear RNA activating complex, polypeptide 1, 43kDa	0.93846	0.967665	0.992
6618	SNAPC2	small nuclear RNA activating complex, polypeptide 2, 45kDa	0.95445	0.945236	1.1063767
6619	SNAPC3	small nuclear RNA activating complex, polypeptide 3, 50kDa	0.9712	1.023447	1.077937
6620	SNCB	synuclein, beta	1.012	1.051193	1.0201489
6621	SNAPC4	small nuclear RNA activating complex, polypeptide 4, 190kDa	0.9907	0.980059	1.0343607
6622	SNCA	synuclein, alpha (non A4 component of amyloid precursor)	0.97589	0.970473	0.9963871
6623	SNCG	synuclein, gamma (breast cancer-specific protein 1)	0.98812	1.030728	1.0442678
6624	FSCN1	fascin homolog 1, actin-bundling protein (Strongylocentrotus purpuratus)	1	1.033848	1.0606469

6625	SNRNP70	small nuclear ribonucleoprotein 70kDa (U1)	0.94529	0.969372	0.9716513
6626	SNRPA	small nuclear ribonucleoprotein polypeptide A	0.97099	1.020764	1.0126572
6627	SNRPA1	small nuclear ribonucleoprotein polypeptide A'	0.99415	0.955723	0.9633044
6628	SNRPB	small nuclear ribonucleoprotein polypeptides B and B1	0.92489	0.985929	0.9874828
6629	SNRPB2	small nuclear ribonucleoprotein polypeptide B	0.96676	0.955533	1.0314497
6631	SNRPC	small nuclear ribonucleoprotein polypeptide C	0.98961	0.947918	0.957124
6632	SNRPD1	small nuclear ribonucleoprotein D1 polypeptide 16kDa	0.94322	1.009859	1.0435245
6633	SNRPD2	small nuclear ribonucleoprotein D2 polypeptide 16.5kDa	0.943	1.06641	1.0378268
6634	SNRPD3	small nuclear ribonucleoprotein D3 polypeptide 18kDa	0.96964	0.885211	1.1414872
6635	SNRPE	small nuclear ribonucleoprotein polypeptide E	1.02673	0.936108	1.0335781
6636	SNRPF	small nuclear ribonucleoprotein polypeptide F	0.95957	0.926819	1.0749286
6637	SNRPG	small nuclear ribonucleoprotein polypeptide G	1.02952	0.889193	0.8829988
6638	SNRPN	small nuclear ribonucleoprotein polypeptide N	0.97154	0.991586	1.0101698
6640	SNTA1	syntrophin, alpha 1 (dystrophin-associated protein A1, 59kDa, acidic component)	1.00112	0.986485	1.0421147
6641	SNTB1	syntrophin, beta 1 (dystrophin-associated protein A1, 59kDa, basic component 1)	0.97457	0.985988	1.0230164
6642	SNX1	sorting nexin 1	0.96609	0.975618	1.0293698
6643	SNX2	sorting nexin 2	1.0094	0.853614	1.0633356
6645	SNTB2	syntrophin, beta 2 (dystrophin-associated protein A1, 59kDa, basic component 2)	0.9875	0.9903	1.0229673
6646	SOAT1	sterol O-acyltransferase 1	1.02363	1.010457	0.9361951
6647	SOD1	superoxide dismutase 1, soluble	0.95599	0.90678	0.9894919
6648	SOD2	superoxide dismutase 2, mitochondrial	1.02433	1.171125	0.9100442
6649	SOD3	superoxide dismutase 3, extracellular	0.97978	0.98457	1.0770751
6650	SOLH	small optic lobes homolog (Drosophila)	0.99759	1.012903	1.0556676
6651	SON	SON DNA binding protein	0.96428	1.015857	1.0276711
6652	SORD	sorbitol dehydrogenase	0.95827	1.025013	1.0087522
6653	SORL1	sortilin-related receptor, L(DLR class) A repeats containing	0.96985	0.886077	0.9895889
6654	SOS1	son of sevenless homolog 1 (Drosophila)	1.00023	1.008533	0.9820714
6655	SOS2	son of sevenless homolog 2 (Drosophila)	0.95193	0.932264	1.0232627
6656	SOX1	SRY (sex determining region Y)-box 1	0.99589	1.090843	1.1216954
6657	SOX2	SRY (sex determining region Y)-box 2	0.97988	0.965349	1.033172
6658	SOX3	SRY (sex determining region Y)-box 3	0.97597	1.026465	1.0802454
6659	SOX4	SRY (sex determining region Y)-box 4	0.98447	1.01827	1.1191746
6660	SOX5	SRY (sex determining region Y)-box 5	0.99151	0.990376	1.0162456

6661	SOX5P	SRY (sex determining region Y)-box 5 pseudogene	1.00638	1.010487	1.0273325
6662	SOX9	SRY (sex determining region Y)-box 9	1.01951	1.019068	1.0010596
6663	SOX10	SRY (sex determining region Y)-box 10	0.99071	1.021771	0.9717357
6664	SOX11	SRY (sex determining region Y)-box 11	0.99162	1.001513	1.0398471
6665	SOX15	SRY (sex determining region Y)-box 15	0.99052	0.968667	1.0094687
6666	SOX12	SRY (sex determining region Y)-box 12	0.97763	1.016886	1.0780906
6667	SP1	Sp1 transcription factor	0.99182	1.005901	1.0920835
6668	SP2	Sp2 transcription factor	0.97874	0.942929	0.9473045
6670	SP3	Sp3 transcription factor	1.03962	0.931112	1.0150274
6671	SP4	Sp4 transcription factor	0.96346	0.961536	0.9738349
6672	SP100	SP100 nuclear antigen	0.96844	0.988542	1.0118509
6674	SPAG1	sperm associated antigen 1	1.00434	1.003696	1.0022304
6675	UAP1	UDP-N-acteylglucosamine pyrophosphorylase 1	1.00167	0.982351	0.9174259
6676	SPAG4	sperm associated antigen 4	0.99582	1.101314	1.0090782
6677	SPAM1	sperm adhesion molecule 1 (PH-20 hyaluronidase, zona pellucida binding)	1.00161	0.972485	1.0385437
6678	SPARC	secreted protein, acidic, cysteine-rich (osteonectin)	0.98353	0.969875	1.0212667
6683	SPAST	spastin	0.98214	0.979406	1.0288958
6687	SPG7	spastic paraplegia 7 (pure and complicated autosomal recessive)	0.98272	1.007095	1.0093436
6688	SPI1	spleen focus forming virus (SFFV) proviral integration oncogene spi1	0.98309	1.077764	1.0067766
6689	SPIB	Spi-B transcription factor (Spi-1/PU.1 related)	0.974	1.055283	1.018644
6690	SPINK1	serine peptidase inhibitor, Kazal type 1	1.0083	1.049066	1.024388
6691	SPINK2	serine peptidase inhibitor, Kazal type 2 (acrosin-trypsin inhibitor)	0.99458	0.973029	1.0547162
6692	SPINT1	serine peptidase inhibitor, Kunitz type 1	0.9524	0.904507	1.0900355
6693	SPN	sialophorin	0.99497	0.911728	1.0533007
6694	SPP2	secreted phosphoprotein 2, 24kDa	0.99947	1.043878	1.0192238
6695	SPOCK1	sparc/osteonectin, cwcv and kazal-like domains proteoglycan (testican) 1	1.01183	1.012676	0.9920613
6696	SPP1	secreted phosphoprotein 1	1.01361	1.020651	0.9795863
6697	SPR	sepiapterin reductase (7,8-dihydrobiopterin:NADP+ oxidoreductase)	1.01893	1.027782	1.0670291
6698	SPRR1A	small proline-rich protein 1A	1.03181	1.028452	0.9559815
6699	SPRR1B	small proline-rich protein 1B	0.99441	0.982582	0.9685102
6702	SPRR2C	small proline-rich protein 2C (pseudogene)	1.0354	0.953606	1.0395374
6707	SPRR3	small proline-rich protein 3	1.00116	1.026505	1.0047285
6708	SPTA1	spectrin, alpha, erythrocytic 1 (elliptocytosis 2)	1.0182	1.00345	0.9952889

6709	SPTAN1	spectrin, alpha, non-erythrocytic 1 (alpha-fodrin)	0.96761	0.995313	1.0936757
6710	SPTB	spectrin, beta, erythrocytic	0.96604	0.996541	0.9875572
6711	SPTBN1	spectrin, beta, non-erythrocytic 1	0.97185	1.00843	1.0449331
6712	SPTBN2	spectrin, beta, non-erythrocytic 2	0.99795	0.98729	1.0212494
6713	SQLE	squalene epoxidase	0.98023	1.028732	0.9999626
6714	SRC	v-src sarcoma (Schmidt-Ruppin A-2) viral oncogene homolog (avian)	0.94923	1.017985	1.0006731
6715	SRD5A1	steroid-5-alpha-reductase, alpha polypeptide 1 (3-oxo-5 alpha-steroid delta 4-dehydrogenase alpha 1)	0.9877	1.007266	1.0968539
6716	SRD5A2	steroid-5-alpha-reductase, alpha polypeptide 2 (3-oxo-5 alpha-steroid delta 4-dehydrogenase alpha 2)	1.0116	0.988842	1.0130719
6717	SRI	sorcin	0.9746	1.00546	1.0392883
6718	AKR1D1	aldo-keto reductase family 1, member D1 (delta 4-3-ketosteroid-5-beta-reductase)	1.01825	0.980357	1.0239007
6719	SRD5A1P1	steroid-5-alpha-reductase, alpha polypeptide 1 pseudogene 1 (3-oxo-5 alpha-steroid delta 4-dehydrogenase	1.00073	0.995916	1.0521291
6720	SREBF1	sterol regulatory element binding transcription factor 1	0.98859	0.999845	0.9601721
6721	SREBF2	sterol regulatory element binding transcription factor 2	0.9865	1.084711	1.0282166
6722	SRF	serum response factor (c-fos serum response element-binding transcription factor)	0.96871	0.991627	1.0198968
6723	SRM	spermidine synthase	1.00226	0.913475	1.0729381
6725	SRMS	src-related kinase lacking C-terminal regulatory tyrosine and N-terminal myristylation sites	0.96567	1.00572	1.0750507
6726	SRP9	signal recognition particle 9kDa	1.01829	0.937761	0.9714002
6727	SRP14	signal recognition particle 14kDa (homologous Alu RNA binding protein)	0.99096	0.992549	0.9611232
6728	SRP19	signal recognition particle 19kDa	0.99654	0.959679	1.000979
6729	SRP54	signal recognition particle 54kDa	0.9395	1.080527	0.9957309
6730	SRP68	signal recognition particle 68kDa	0.94159	0.92342	1.0627173
6731	SRP72	signal recognition particle 72kDa	0.99213	0.919656	1.0488275
6732	SRPK1	SRSF protein kinase 1	0.98892	1.067134	0.9773505
6733	SRPK2	SRSF protein kinase 2	0.99505	0.92887	0.9370875
6734	SRPR	signal recognition particle receptor (docking protein)	0.986	1.054264	0.9804075
6736	SRY	sex determining region Y	1.02036	0.964452	1.0416268
6737	TRIM21	tripartite motif containing 21	0.89503	0.861084	1.0551016
6738	TROVE2	TROVE domain family, member 2	0.99927	0.951541	0.9597185
6741	SSB	Sjogren syndrome antigen B (autoantigen La)	1.004	0.959529	0.928679
6742	SSBP1	single-stranded DNA binding protein 1	0.99572	0.904624	1.06611
6744	SSFA2	sperm specific antigen 2	0.98707	0.896189	1.1048747
6745	SSR1	signal sequence receptor, alpha	1.01532	0.951412	1.1120585
6746	SSR2	signal sequence receptor, beta (translocon-associated protein beta)	1.02119	0.994048	0.9807454

6747	SSR3	signal sequence receptor, gamma (translocon-associated protein gamma)	0.99193	1.008192	1.1085893
6748	SSR4	signal sequence receptor, delta (translocon-associated protein delta)	0.92792	1.000219	1.0623075
6749	SSRP1	structure specific recognition protein 1	0.93945	0.957193	1.0109294
6750	SST	somatostatin	1.00942	1.027054	1.0505872
6751	SSTR1	somatostatin receptor 1	1.00977	1.041096	1.0198827
6752	SSTR2	somatostatin receptor 2	0.97996	1.011593	1.0447775
6753	SSTR3	somatostatin receptor 3	0.952	1.004853	0.9670565
6754	SSTR4	somatostatin receptor 4	0.98799	1.020306	1.058636
6755	SSTR5	somatostatin receptor 5	0.98521	1.037306	1.0379284
6756	SSX1	synovial sarcoma, X breakpoint 1	0.98909	1.039893	0.9726176
6758	SSX5	synovial sarcoma, X breakpoint 5	1.00925	1.012627	0.9635238
6760	SS18	synovial sarcoma translocation, chromosome 18	0.94283	1.039255	1.1554006
6764	ST5	suppression of tumorigenicity 5	0.9959	0.992906	1.0104578
6767	ST13	suppression of tumorigenicity 13 (colon carcinoma) (Hsp70 interacting protein)	0.97764	0.999567	0.9777622
6769	STAC	SH3 and cysteine rich domain	1.00589	0.9957	1.005576
6770	STAR	steroidogenic acute regulatory protein	1.00062	1.026875	1.0033621
6771	STARP1	steroidogenic acute regulatory protein pseudogene 1	0.98072	0.956955	0.9671136
6772	STAT1	signal transducer and activator of transcription 1, 91kDa	1.02425	1.006321	1.1965274
6773	STAT2	signal transducer and activator of transcription 2, 113kDa	0.99059	1.056317	1.123122
6774	STAT3	signal transducer and activator of transcription 3 (acute-phase response factor)	0.98249	1.031763	0.9702452
6775	STAT4	signal transducer and activator of transcription 4	0.98413	1.099541	0.9781856
6776	STAT5A	signal transducer and activator of transcription 5A	0.96656	1.023359	1.0045774
6777	STAT5B	signal transducer and activator of transcription 5B	0.94569	1.047484	0.8966959
6778	STAT6	signal transducer and activator of transcription 6, interleukin-4 induced	0.98738	0.901879	1.2100634
6779	STATH	statherin	1.01275	0.992636	1.0354636
6780	STAU1	staufen, RNA binding protein, homolog 1 (Drosophila)	0.93502	1.080513	0.9442928
6781	STC1	stanniocalcin 1	0.98403	1.000283	0.9867614
6782	HSPA13	heat shock protein 70kDa family, member 13	0.90354	0.937235	1.1098866
6783	SULT1E1	sulfotransferase family 1E, estrogen-preferring, member 1	0.99397	1.001833	1.0022871
6785	ELOVL4	ELOVL fatty acid elongase 4	1.00141	0.999137	1.010529
6786	STIM1	stromal interaction molecule 1	0.96894	0.980421	0.9612072
6787	NEK4	NIMA (never in mitosis gene a)-related kinase 4	0.97339	0.947442	1.0256866
6788	STK3	serine/threonine kinase 3	0.979	0.955556	0.9871869

6789	STK4	serine/threonine kinase 4	0.96482	1.053756	1.0844026
6790	AURKA	aurora kinase A	0.97918	0.954913	1.0039219
6792	CDKL5	cyclin-dependent kinase-like 5	0.97741	0.985637	0.9954686
6793	STK10	serine/threonine kinase 10	1.00894	1.060881	0.9353306
6794	STK11	serine/threonine kinase 11	0.98473	1.008153	1.0598317
6795	AURKC	aurora kinase C	0.99377	1.051504	0.9907429
6799	SULT1A2	sulfotransferase family, cytosolic, 1A, phenol-preferring, member 2	0.97904	1.012554	0.9920299
6801	STRN	striatin, calmodulin binding protein	0.97091	0.923461	1.0398552
6804	STX1A	syntaxin 1A (brain)	0.99677	1.005296	0.9624328
6809	STX3	syntaxin 3	1.00069	1.010395	1.020103
6810	STX4	syntaxin 4	0.94504	1.089043	0.9440356
6811	STX5	syntaxin 5	0.97669	0.998457	0.9685553
6812	STXBP1	syntaxin binding protein 1	0.97958	0.945847	0.9894438
6813	STXBP2	syntaxin binding protein 2	0.91558	0.975535	0.873694
6814	STXBP3	syntaxin binding protein 3	1.01471	1.003182	0.9896204
6815	STYX	serine/threonine/tyrosine interacting protein	0.9204	0.877515	0.8674477
6817	SULT1A1	sulfotransferase family, cytosolic, 1A, phenol-preferring, member 1	1.00895	0.992517	0.9548688
6819	SULT1C2	sulfotransferase family, cytosolic, 1C, member 2	1.00996	0.965605	1.0242302
6820	SULT2B1	sulfotransferase family, cytosolic, 2B, member 1	0.99784	0.990357	0.9712211
6821	SUOX	sulfite oxidase	1.00395	0.96871	1.0612297
6822	SULT2A1	sulfotransferase family, cytosolic, 2A, dehydroepiandrosterone (DHEA)-preferring, member 1	0.99877	0.990849	1.0050058
6824	ETF1P1	eukaryotic translation termination factor 1 pseudogene 1	1.00194	0.961279	1.0470578
6825	ETF1P2	eukaryotic translation termination factor 1 pseudogene 2	1.01123	1.043809	0.9624459
6827	SUPT4H1	suppressor of Ty 4 homolog 1 (S. cerevisiae)	0.92806	1.018315	0.9994766
6829	SUPT5H	suppressor of Ty 5 homolog (S. cerevisiae)	0.95951	0.990353	0.9781027
6830	SUPT6H	suppressor of Ty 6 homolog (S. cerevisiae)	0.97463	1.173429	0.9560851
6832	SUPV3L1	suppressor of var1, 3-like 1 (S. cerevisiae)	0.95571	1.023629	1.0128196
6833	ABCC8	ATP-binding cassette, sub-family C (CFTR/MRP), member 8	1.00599	1.003781	0.9833986
6834	SURF1	surfeit 1	0.9345	0.964146	1.0288388
6835	SURF2	surfeit 2	0.95784	0.997704	1.0682617
6836	SURF4	surfeit 4	0.96828	1.092335	1.0200106
6837	MED22	mediator complex subunit 22	0.96244	0.973171	1.0873353
6838	SURF6	surfeit 6	0.95842	1.016064	0.9635356

6839	SUV39H1	suppressor of variegation 3-9 homolog 1 (Drosophila)	0.94686	0.950893	1.0888531
6840	SVIL	supervillin	0.97837	0.981079	0.9445685
6843	VAMP1	vesicle-associated membrane protein 1 (synaptobrevin 1)	0.95	1.04065	0.9333377
6844	VAMP2	vesicle-associated membrane protein 2 (synaptobrevin 2)	0.97546	0.987362	0.9270038
6847	SYCP1	synaptonemal complex protein 1	0.99379	1.036931	1.0193001
6850	SYK	spleen tyrosine kinase	1.02216	0.970059	1.0838557
6853	SYN1	synapsin I	0.9953	1.02242	0.9698635
6854	SYN2	synapsin II	1.00793	1.020929	1.030355
6855	SYP	synaptophysin	0.98168	0.99114	1.0168772
6856	SYPL1	synaptophysin-like 1	0.98546	0.926449	0.9956951
6857	SYT1	synaptotagmin I	0.99954	0.988527	1.0085414
6860	SYT4	synaptotagmin IV	0.99045	0.960486	0.9675295
6861	SYT5	synaptotagmin V	0.98787	1.009327	1.0245096
6862	T	T, brachyury homolog (mouse)	1.0149	1.025316	1.0040853
6863	TAC1	tachykinin, precursor 1	0.99748	1.021677	1.0016511
6865	TACR2	tachykinin receptor 2	0.99323	0.989264	0.9950782
6866	TAC3	tachykinin 3	1.00417	1.033931	0.9874717
6867	TACC1	transforming, acidic coiled-coil containing protein 1	1.00726	0.925345	0.9370279
6868	ADAM17	ADAM metallopeptidase domain 17	1.02528	1.152291	1.0376329
6869	TACR1	tachykinin receptor 1	1.00942	1.007148	1.0197673
6870	TACR3	tachykinin receptor 3	0.99029	0.989533	1.0117251
6871	TADA2A	transcriptional adaptor 2A	0.94311	0.975566	0.9919458
6872	TAF1	TAF1 RNA polymerase II, TATA box binding protein (TBP)-associated factor, 250kDa	0.95875	1.015851	0.9881303
6873	TAF2	TAF2 RNA polymerase II, TATA box binding protein (TBP)-associated factor, 150kDa	0.9416	1.033088	1.0763683
6874	TAF4	TAF4 RNA polymerase II, TATA box binding protein (TBP)-associated factor, 135kDa	0.98446	0.957072	1.0158015
6875	TAF4B	TAF4b RNA polymerase II, TATA box binding protein (TBP)-associated factor, 105kDa	0.94387	0.980678	1.0320541
6876	TAGLN	transgelin	1.02079	1.113565	0.9646457
6877	TAF5	TAF5 RNA polymerase II, TATA box binding protein (TBP)-associated factor, 100kDa	0.91815	1.022126	1.0460627
6878	TAF6	TAF6 RNA polymerase II, TATA box binding protein (TBP)-associated factor, 80kDa	0.97378	1.001824	0.9892139
6879	TAF7	TAF7 RNA polymerase II, TATA box binding protein (TBP)-associated factor, 55kDa	0.94288	0.935495	1.0199948
6880	TAF9	TAF9 RNA polymerase II, TATA box binding protein (TBP)-associated factor, 32kDa	0.98363	0.919495	0.994485
6881	TAF10	TAF10 RNA polymerase II, TATA box binding protein (TBP)-associated factor, 30kDa	0.98073	0.898473	0.9905173
6882	TAF11	TAF11 RNA polymerase II, TATA box binding protein (TBP)-associated factor, 28kDa	1.01282	0.815822	0.8815431

6883	TAF12	TAF12 RNA polymerase II, TATA box binding protein (TBP)-associated factor, 20kDa	0.9498	0.995208	1.0121107
6884	TAF13	TAF13 RNA polymerase II, TATA box binding protein (TBP)-associated factor, 18kDa	0.96591	0.959432	0.9495227
6885	MAP3K7	mitogen-activated protein kinase kinase kinase 7	0.95991	1.011844	1.0071029
6886	TAL1	T-cell acute lymphocytic leukemia 1	0.98153	0.88182	0.9645563
6887	TAL2	T-cell acute lymphocytic leukemia 2	1.03238	0.99685	0.973218
6888	TALDO1	transaldolase 1	0.9637	0.907686	1.0339625
6890	TAP1	transporter 1, ATP-binding cassette, sub-family B (MDR/TAP)	0.99429	0.970384	1.0596518
6891	TAP2	transporter 2, ATP-binding cassette, sub-family B (MDR/TAP)	0.99005	0.973904	1.061546
6892	TAPBP	TAP binding protein (tapasin)	1.02806	1.002047	0.9073782
6894	TARBP1	TAR (HIV-1) RNA binding protein 1	1.02132	0.963558	0.9624433
6895	TARBP2	TAR (HIV-1) RNA binding protein 2	0.96737	1.014996	1.0679333
6897	TARS	threonyl-tRNA synthetase	0.96926	0.964791	1.0129688
6898	TAT	tyrosine aminotransferase	0.98266	0.988277	0.9880326
6899	TBX1	T-box 1	0.987	1.000089	1.0566581
6900	CNTN2	contactin 2 (axonal)	1.00271	1.014493	1.0162809
6901	TAZ	tafazzin	0.9466	1.0481	1.0501186
6902	TBCA	tubulin folding cofactor A	1.01356	1.004427	0.9849618
6903	TBCC	tubulin folding cofactor C	0.90591	0.90348	0.9261673
6904	TBCD	tubulin folding cofactor D	0.9989	0.962219	1.0153413
6905	TBCE	tubulin folding cofactor E	1.01369	0.972585	1.0084299
6906	SERPINA7	serpin peptidase inhibitor, clade A (alpha-1 antiproteinase, antitrypsin), member 7	1.00587	1.006031	0.9991755
6907	TBL1X	transducin (beta)-like 1X-linked	0.97059	1.039855	0.982455
6908	TBP	TATA box binding protein	0.97357	0.877126	1.0242001
6909	TBX2	T-box 2	0.98635	0.969123	1.0399928
6910	TBX5	T-box 5	0.98639	0.998326	1.0060866
6911	TBX6	T-box 6	0.99706	0.98741	1.0019271
6913	TBX15	T-box 15	1.00527	1.008917	1.014256
6916	TBXAS1	thromboxane A synthase 1 (platelet)	1.07626	0.952494	0.9477755
6917	TCEA1	transcription elongation factor A (SII), 1	0.99876	0.943644	0.9908943
6919	TCEA2	transcription elongation factor A (SII), 2	0.96503	1.058071	1.0721262
6920	TCEA3	transcription elongation factor A (SII), 3	0.93333	0.954225	0.9381676
6921	TCEB1	transcription elongation factor B (SIII), polypeptide 1 (15kDa, elongin C)	1.00662	0.957839	1.0148974
6922	SKP1P1	S-phase kinase-associated protein 1 pseudogene 1	1.03822	1.021719	1.0348851

6923	TCEB2	transcription elongation factor B (SIII), polypeptide 2 (18kDa, elongin B)	0.9871	0.99553	1.0120875
6924	TCEB3	transcription elongation factor B (SIII), polypeptide 3 (110kDa, elongin A)	0.99957	0.968249	1.0928717
6925	TCF4	transcription factor 4	0.99442	1.05712	1.0873631
6926	TBX3	T-box 3	0.99198	1.042969	1.0315469
6927	HNF1A	HNF1 homeobox A	1.01008	0.991627	1.0065726
6928	HNF1B	HNF1 homeobox B	0.99717	0.994981	0.999323
6929	TCF3	transcription factor 3 (E2A immunoglobulin enhancer binding factors E12/E47)	0.98866	1.033996	1.0278781
6932	TCF7	transcription factor 7 (T-cell specific, HMG-box)	0.94436	0.941511	0.9325651
6934	TCF7L2	transcription factor 7-like 2 (T-cell specific, HMG-box)	1.04616	1.058605	1.0129985
6935	ZEB1	zinc finger E-box binding homeobox 1	0.95021	1.057966	1.0187963
6936	C2orf3	chromosome 2 open reading frame 3	0.9855	0.921196	0.987074
6938	TCF12	transcription factor 12	0.98204	0.975228	1.0051908
6939	TCF15	transcription factor 15 (basic helix-loop-helix)	0.98387	1.01067	1.0401999
6940	ZNF354A	zinc finger protein 354A	0.97084	0.932066	0.9988474
6941	TCF19	transcription factor 19	0.97396	0.95427	1.0284909
6942	TCF20	transcription factor 20 (AR1)	0.95658	0.96256	1.037605
6943	TCF21	transcription factor 21	0.98487	0.98292	1.0247272
6944	VPS72	vacuolar protein sorting 72 homolog (S. cerevisiae)	0.96841	0.998221	1.0516722
6945	MLX	MAX-like protein X	0.96844	0.985045	1.0972711
6947	TCN1	transcobalamin I (vitamin B12 binding protein, R binder family)	0.9987	0.983599	1.0386634
6948	TCN2	transcobalamin II	0.9673	0.933338	0.9381476
6949	TCOF1	Treacher Collins-Franceschetti syndrome 1	0.99556	0.986866	0.9884136
6950	TCP1	t-complex 1	1.00113	0.95779	0.9716485
6952	TCP1P2	t-complex 1 pseudogene 2	0.99926	1.035874	1.0419932
6954	TCP11	t-complex 11 homolog (mouse)	1.00562	1.00174	1.0010238
6955	TRA@	T cell receptor alpha locus	0.97887	0.986877	0.9979486
6957	TRB@	T cell receptor beta locus	1.0047	0.997476	0.9686848
6958	TRBV29OR9	T cell receptor beta variable 29/OR9-2 (non-functional)	0.99033	0.948737	1.0179157
6960	TRBV25OR9	T cell receptor beta variable 25/OR9-2 (pseudogene)	0.98349	1.04977	1.0294223
6975	TECTB	tectorin beta	0.9986	1.0082	1.0004126
6988	TCTA	T-cell leukemia translocation altered gene	0.98135	0.958567	1.0221596
6990	DYNLT3	dynein, light chain, Tctex-type 3	0.94657	0.979338	0.9807698
6991	TCTE3	t-complex-associated-testis-expressed 3	1.00108	0.990899	0.9856637

6992	PPP1R11	protein phosphatase 1, regulatory (inhibitor) subunit 11	0.97957	0.987732	0.9584222
6993	DYNLT1	dynein, light chain, Tctex-type 1	1.03116	0.97034	0.923724
6996	TDG	thymine-DNA glycosylase	0.98234	0.973267	0.9844741
6997	TDGF1	teratocarcinoma-derived growth factor 1	1.02846	0.967778	1.0849038
6998	TDGF3	teratocarcinoma-derived growth factor 3, pseudogene	1.00238	1.073935	1.0450657
6999	TDO2	tryptophan 2,3-dioxygenase	0.99562	1.017414	1.038734
7001	PRDX2	peroxiredoxin 2	0.94459	0.989148	1.0418705
7002	TDPX2	thioredoxin-dependent peroxide reductase 2	0.96586	1.005898	0.9912023
7003	TEAD1	TEA domain family member 1 (SV40 transcriptional enhancer factor)	1.00233	0.985499	1.0164979
7004	TEAD4	TEA domain family member 4	0.99281	0.986653	1.0231694
7005	TEAD3	TEA domain family member 3	1.00979	1.007715	1.0144417
7006	TEC	tec protein tyrosine kinase	1.00468	0.956897	1.0364488
7007	TECTA	tectorin alpha	0.99064	1.002898	1.0026073
7008	TEF	thyrotrophic embryonic factor	0.96754	0.98756	1.0568935
7009	TMBIM6	transmembrane BAX inhibitor motif containing 6	0.99332	0.90459	0.9671082
7010	TEK	TEK tyrosine kinase, endothelial	0.99656	1.010115	0.9965789
7011	TEP1	telomerase-associated protein 1	0.99237	0.913903	1.0144152
7012	TERC	telomerase RNA component	0.91657	0.972084	1.0472992
7013	TERF1	telomeric repeat binding factor (NIMA-interacting) 1	1.01546	0.955458	1.0064084
7014	TERF2	telomeric repeat binding factor 2	0.9267	0.986688	0.9891753
7015	TERT	telomerase reverse transcriptase	0.99782	1.005358	1.0326049
7016	TESK1	testis-specific kinase 1	0.94698	0.979621	1.0585657
7018	TF	transferrin	1.01271	1.018926	1.0129199
7019	TFAM	transcription factor A, mitochondrial	0.93432	0.930144	1.1066225
7020	TFAP2A	transcription factor AP-2 alpha (activating enhancer binding protein 2 alpha)	0.9823	0.99699	1.0310313
7021	TFAP2B	transcription factor AP-2 beta (activating enhancer binding protein 2 beta)	1.00835	1.000541	1.0066601
7022	TFAP2C	transcription factor AP-2 gamma (activating enhancer binding protein 2 gamma)	0.98841	1.022452	0.9807419
7023	TFAP4	transcription factor AP-4 (activating enhancer binding protein 4)	0.9536	0.981857	0.9682121
7024	TFCP2	transcription factor CP2	0.93807	1.007197	1.0007066
7025	NR2F1	nuclear receptor subfamily 2, group F, member 1	1.00254	1.01944	1.0144115
7026	NR2F2	nuclear receptor subfamily 2, group F, member 2	0.98496	1.011557	1.0479446
7027	TFDP1	transcription factor Dp-1	0.9992	1.040084	0.9702757
7029	TFDP2	transcription factor Dp-2 (E2F dimerization partner 2)	1.00879	0.945099	1.0276904

7030	TFE3	transcription factor binding to IGHM enhancer 3	0.95014	0.962121	0.9903827
7031	TFF1	trefoil factor 1	1.03292	0.976631	0.981124
7032	TFF2	trefoil factor 2	0.97324	0.986656	1.04636
7033	TFF3	trefoil factor 3 (intestinal)	0.99285	1.000086	1.0231411
7035	TFPI	tissue factor pathway inhibitor (lipoprotein-associated coagulation inhibitor)	0.96996	0.942845	0.9796473
7036	TFR2	transferrin receptor 2	0.99459	0.973691	1.0126605
7037	TFRC	transferrin receptor (p90, CD71)	1.06192	0.984814	0.9583983
7038	TG	thyroglobulin	1.00549	1.009466	0.9772444
7039	TGFA	transforming growth factor, alpha	1.01366	0.991457	1.0073787
7040	TGFB1	transforming growth factor, beta 1	0.93127	1.046529	1.0137235
7041	TGFB1I1	transforming growth factor beta 1 induced transcript 1	0.95347	0.973083	1.0396769
7042	TGFB2	transforming growth factor, beta 2	1.01141	0.974045	0.9735276
7043	TGFB3	transforming growth factor, beta 3	1.00993	0.975393	0.9688612
7044	LEFTY2	left-right determination factor 2	1.01244	0.993911	1.0440956
7045	TGFBI	transforming growth factor, beta-induced, 68kDa	1.01796	0.980849	1.0194874
7046	TGFBR1	transforming growth factor, beta receptor 1	0.94604	1.052894	0.9819919
7047	TGM4	transglutaminase 4 (prostate)	1.00399	0.998708	1.0310263
7048	TGFBR2	transforming growth factor, beta receptor II (70/80kDa)	0.95225	0.880529	0.9672764
7049	TGFBR3	transforming growth factor, beta receptor III	1.03334	1.116741	0.9020177
7050	TGIF1	TGFB-induced factor homeobox 1	0.9692	1.005013	0.9766556
7051	TGM1	transglutaminase 1 (K polypeptide epidermal type I, protein-glutamine-gamma-glutamyltransferase)	0.98973	1.022852	1.0243389
7052	TGM2	transglutaminase 2 (C polypeptide, protein-glutamine-gamma-glutamyltransferase)	0.99525	1.040699	0.9756776
7053	TGM3	transglutaminase 3 (E polypeptide, protein-glutamine-gamma-glutamyltransferase)	1.0064	0.988576	0.9766704
7054	TH	tyrosine hydroxylase	0.99525	1.020462	1.0045704
7056	THBD	thrombomodulin	0.97518	0.968058	0.8086786
7057	THBS1	thrombospondin 1	0.97266	1.027206	0.8640648
7058	THBS2	thrombospondin 2	1.01572	1.014888	0.9961158
7059	THBS3	thrombospondin 3	1.02275	1.034374	1.0069388
7060	THBS4	thrombospondin 4	1.00295	1.002698	1.0050823
7062	TCHH	trichohyalin	0.99896	0.99484	1.0125131
7064	THOP1	thimet oligopeptidase 1	0.96785	0.990249	1.0142305
7066	THPO	thrombopoietin	1.02754	0.99489	0.979825
7067	THRA	thyroid hormone receptor, alpha	0.91767	0.976449	0.9518009

7068	THRB	thyroid hormone receptor, beta (erythroblastic leukemia viral (v-erb-a) oncogene homolog 2, avian)	1.00115	0.992584	0.9890499
7069	THRSP	thyroid hormone responsive	1.01159	0.990169	0.9904493
7070	THY1	Thy-1 cell surface antigen	1.01693	0.996799	1.0228574
7071	KLF10	Kruppel-like factor 10	1.00222	0.911735	0.9632369
7072	TIA1	TIA1 cytotoxic granule-associated RNA binding protein	1.02589	0.950379	1.0445106
7073	TIAL1	TIA1 cytotoxic granule-associated RNA binding protein-like 1	0.95975	0.938545	1.0545566
7074	TIAM1	T-cell lymphoma invasion and metastasis 1	0.96839	0.907172	1.0044328
7075	TIE1	tyrosine kinase with immunoglobulin-like and EGF-like domains 1	1.0059	1.001861	1.0170079
7076	TIMP1	TIMP metallopeptidase inhibitor 1	1.00463	1.002657	0.9629996
7077	TIMP2	TIMP metallopeptidase inhibitor 2	0.98228	1.018833	0.9838304
7078	TIMP3	TIMP metallopeptidase inhibitor 3	1.00255	0.974892	0.997888
7079	TIMP4	TIMP metallopeptidase inhibitor 4	1.0274	1.010453	1.0082874
7080	NKX2-1	NK2 homeobox 1	1.00339	1.018204	1.0133584
7082	TJP1	tight junction protein 1 (zona occludens 1)	0.99773	0.998677	1.005705
7083	TK1	thymidine kinase 1, soluble	0.98629	1.030637	1.017835
7084	TK2	thymidine kinase 2, mitochondrial	0.96097	1.037111	0.9948169
7086	TKT	transketolase	1.02711	0.982615	0.9958776
7087	ICAM5	intercellular adhesion molecule 5, telencephalin	0.97895	0.993439	1.0312228
7088	TLE1	transducin-like enhancer of split 1 (E(sp1) homolog, Drosophila)	0.9853	1.043794	0.9705616
7089	TLE2	transducin-like enhancer of split 2 (E(sp1) homolog, Drosophila)	0.97561	0.990303	0.9959177
7090	TLE3	transducin-like enhancer of split 3 (E(sp1) homolog, Drosophila)	0.95374	0.953864	0.9660005
7091	TLE4	transducin-like enhancer of split 4 (E(sp1) homolog, Drosophila)	1.00943	0.994039	1.0143667
7092	TLL1	tolloid-like 1	1.00479	1.012318	1.0048776
7093	TLL2	tolloid-like 2	1.0013	0.999447	1.0028292
7094	TLN1	talin 1	0.95286	1.057161	0.9841775
7095	SEC62	SEC62 homolog (S. cerevisiae)	0.99682	0.955794	1.0352072
7096	TLR1	toll-like receptor 1	1.01924	0.961409	1.2108062
7097	TLR2	toll-like receptor 2	1.05406	0.983322	0.9930487
7098	TLR3	toll-like receptor 3	0.99768	0.960936	0.9545023
7099	TLR4	toll-like receptor 4	0.97765	0.972896	0.9937952
7100	TLR5	toll-like receptor 5	1.02315	1.003524	1.0002462
7101	NR2E1	nuclear receptor subfamily 2, group E, member 1	0.98974	1.008357	1.0152058
7102	TSPAN7	tetraspanin 7	0.99263	0.989472	0.9976344

7103	TSPAN8	tetraspanin 8	0.99036	1.037047	1.0140897
7104	TM4SF4	transmembrane 4 L six family member 4	1.02049	0.992907	0.9977622
7105	TSPAN6	tetraspanin 6	0.99089	1.016174	0.9578779
7107	GPR137B	G protein-coupled receptor 137B	0.99971	0.920642	1.0119703
7108	TM7SF2	transmembrane 7 superfamily member 2	0.96298	1.020686	1.0130135
7109	TRAPPC10	trafficking protein particle complex 10	0.93412	0.925662	1.0141337
7110	TMF1	TATA element modulatory factor 1	1.01074	0.960673	0.9625551
7111	TMOD1	tropomodulin 1	0.98113	0.99392	1.0265124
7112	TMPO	thymopoietin	0.9704	0.958134	1.1613122
7113	TMPRSS2	transmembrane protease, serine 2	0.99026	1.006057	1.005401
7114	TMSB4X	thymosin beta 4, X-linked	0.98129	1.042385	1.0156701
7115	TMSB4XP1	thymosin beta 4, X-linked pseudogene 1	0.98099	0.955277	0.9628478
7122	CLDN5	claudin 5	0.95446	0.951595	1.0078563
7123	CLEC3B	C-type lectin domain family 3, member B	0.99855	1.000782	0.9971103
7124	TNF	tumor necrosis factor	0.96924	1.061181	0.9680742
7125	TNNC2	troponin C type 2 (fast)	0.96075	0.995583	0.9986966
7126	TNFAIP1	tumor necrosis factor, alpha-induced protein 1 (endothelial)	0.94032	0.993936	1.0454434
7127	TNFAIP2	tumor necrosis factor, alpha-induced protein 2	0.99888	1.222069	0.9620844
7128	TNFAIP3	tumor necrosis factor, alpha-induced protein 3	1.03	0.959178	0.8737878
7130	TNFAIP6	tumor necrosis factor, alpha-induced protein 6	1.02591	1.042016	0.946309
7132	TNFRSF1A	tumor necrosis factor receptor superfamily, member 1A	0.94226	0.986633	0.9659114
7134	TNNC1	troponin C type 1 (slow)	1.0197	0.972672	0.9909016
7135	TNNI1	troponin I type 1 (skeletal, slow)	1.01342	1.030319	0.9989926
7136	TNNI2	troponin I type 2 (skeletal, fast)	0.9942	1.024568	0.9649256
7137	TNNI3	troponin I type 3 (cardiac)	0.99631	1.053206	1.035064
7138	TNNT1	troponin T type 1 (skeletal, slow)	0.99879	1.007786	0.9927369
7139	TNNT2	troponin T type 2 (cardiac)	1.0085	0.976266	1.0116477
7140	TNNT3	troponin T type 3 (skeletal, fast)	0.99114	1.03298	1.0088346
7141	TNP1	transition protein 1 (during histone to protamine replacement)	1.01039	1.041667	0.98057
7142	TNP2	transition protein 2 (during histone to protamine replacement)	1.01902	1.008267	1.00511
7143	TNR	tenascin R (restrictin, janusin)	1.01235	0.99684	0.9983667
7145	TNS1	tensin 1	1.00419	0.979457	1.0007627
7148	TNXB	tenascin XB	0.98062	1.019835	1.0124598

7150	TOP1	topoisomerase (DNA) I	0.97422	0.989622	0.9761452
7153	TOP2A	topoisomerase (DNA) II alpha 170kDa	0.95952	0.910706	1.0359007
7155	TOP2B	topoisomerase (DNA) II beta 180kDa	1.00419	1.086148	0.9903522
7156	TOP3A	topoisomerase (DNA) III alpha	0.95073	0.958047	1.1144775
7157	TP53	tumor protein p53	0.96624	0.989019	0.9434856
7158	TP53BP1	tumor protein p53 binding protein 1	0.95617	1.003591	1.0235045
7159	TP53BP2	tumor protein p53 binding protein, 2	0.98367	0.991667	0.9479145
7161	TP73	tumor protein p73	1.0027	1.014948	1.0378932
7162	TPBG	trophoblast glycoprotein	0.98029	0.972864	1.0913093
7163	TPD52	tumor protein D52	0.99015	1.085769	1.0743674
7164	TPD52L1	tumor protein D52-like 1	0.99253	1.020681	1.0186581
7165	TPD52L2	tumor protein D52-like 2	0.99336	0.938484	0.9575111
7166	TPH1	tryptophan hydroxylase 1	0.97273	0.904513	0.9465182
7167	TPI1	triosephosphate isomerase 1	0.96376	1.063654	1.0556691
7168	TPM1	tropomyosin 1 (alpha)	0.96872	0.927364	0.9774527
7169	TPM2	tropomyosin 2 (beta)	0.99188	1.003658	1.0214011
7170	TPM3	tropomyosin 3	1.01462	0.994882	0.9922462
7171	TPM4	tropomyosin 4	1.01591	0.960952	1.029025
7172	TPMT	thiopurine S-methyltransferase	1.02149	1.001303	1.0137104
7173	TPO	thyroid peroxidase	1.00255	1.014129	1.0117202
7174	TPP2	tripeptidyl peptidase II	0.93609	0.936231	0.9743608
7175	TPR	translocated promoter region (to activated MET oncogene)	0.96287	1.006632	1.1329089
7178	TPT1	tumor protein, translationally-controlled 1	1.00789	0.894271	0.9505853
7179	TPTE	transmembrane phosphatase with tensin homology	0.98293	1.028692	1.0179109
7180	CRISP2	cysteine-rich secretory protein 2	0.99167	1.012595	0.9863412
7181	NR2C1	nuclear receptor subfamily 2, group C, member 1	0.92082	0.963575	1.014301
7182	NR2C2	nuclear receptor subfamily 2, group C, member 2	0.99067	1.007983	0.9522111
7184	HSP90B1	heat shock protein 90kDa beta (Grp94), member 1	0.98244	0.954121	1.0160339
7185	TRAF1	TNF receptor-associated factor 1	0.92811	1.086105	0.9871995
7186	TRAF2	TNF receptor-associated factor 2	0.98601	1.015252	1.0378613
7187	TRAF3	TNF receptor-associated factor 3	0.98494	1.028864	1.0057075
7188	TRAF5	TNF receptor-associated factor 5	0.97995	1.006301	0.97505
7189	TRAF6	TNF receptor-associated factor 6	0.99213	0.990467	1.0253823

7200	TRH	thyrotropin-releasing hormone	1.00374	0.978769	1.0333093
7201	TRHR	thyrotropin-releasing hormone receptor	0.98692	1.025137	1.0684967
7203	CCT3	chaperonin containing TCP1, subunit 3 (gamma)	0.97331	0.991364	1.0672998
7204	TRIO	triple functional domain (PTPRF interacting)	1.0114	1.022999	1.0420538
7205	TRIP6	thyroid hormone receptor interactor 6	0.99966	0.999825	1.0138228
7216	TRO	trophinin	0.98418	0.992022	1.0008866
7220	TRPC1	transient receptor potential cation channel, subfamily C, member 1	0.99945	0.951789	0.9638902
7221	TRPC2	transient receptor potential cation channel, subfamily C, member 2, pseudogene	0.96537	1.016179	1.017769
7222	TRPC3	transient receptor potential cation channel, subfamily C, member 3	0.98976	1.012203	1.0360512
7223	TRPC4	transient receptor potential cation channel, subfamily C, member 4	0.99699	0.974469	0.9909952
7224	TRPC5	transient receptor potential cation channel, subfamily C, member 5	0.98717	0.997825	0.9962171
7225	TRPC6	transient receptor potential cation channel, subfamily C, member 6	0.99605	0.979499	0.9981692
7227	TRPS1	trichorhinophalangeal syndrome I	0.98776	1.036164	1.0141217
7236	TRNAT1	transfer RNA threonine 1 (anticodon UGU)	0.97815	1.034558	1.1098795
7247	TSN	translin	0.9866	0.939739	1.0760846
7248	TSC1	tuberous sclerosis 1	0.93874	0.942538	1.0933903
7249	TSC2	tuberous sclerosis 2	0.99375	1.00045	1.0282986
7251	TSG101	tumor susceptibility gene 101	0.93607	1.015638	0.9564584
7252	TSHB	thyroid stimulating hormone, beta	1.02572	1.009916	1.0532236
7253	TSHR	thyroid stimulating hormone receptor	0.9988	1.005722	1.0169684
7257	TSNAX	translin-associated factor X	1.01144	0.935057	0.9688156
7259	TSPYL1	TSPY-like 1	0.9797	0.892563	0.9829614
7260	TSSC1	tumor suppressing subtransferable candidate 1	1.00035	0.967056	1.0188948
7262	PHLDA2	pleckstrin homology-like domain, family A, member 2	0.97703	1.125851	0.9616799
7263	TST	thiosulfate sulfurtransferase (rhodanese)	0.93649	0.988873	1.0428622
7264	TSTA3	tissue specific transplantation antigen P35B	0.938	0.891764	1.1097654
7265	TTC1	tetratricopeptide repeat domain 1	0.98153	0.938204	1.0526183
7266	DNAJC7	DnaJ (Hsp40) homolog, subfamily C, member 7	0.9803	0.916428	0.9096923
7267	TTC3	tetratricopeptide repeat domain 3	0.95715	1.02533	0.9927056
7268	TTC4	tetratricopeptide repeat domain 4	0.97779	0.95133	1.0393941
7270	TTF1	transcription termination factor, RNA polymerase I	0.94374	0.943158	1.1000875
7272	TTK	TTK protein kinase	0.98915	0.970631	1.0217705
7273	TTN	titin	0.99155	0.923021	1.0220294

7274	TTPA	tocopherol (alpha) transfer protein	0.99529	1.01601	1.0128043
7275	TUB	tubby homolog (mouse)	0.99658	1.006049	1.0231591
7276	TTR	transthyretin	1.00282	1.040811	0.9791607
7277	TUBA4A	tubulin, alpha 4a	0.96665	0.991208	0.9630373
7278	TUBA3C	tubulin, alpha 3c	0.94645	0.983577	1.0367323
7280	TUBB2A	tubulin, beta 2A	0.98228	1.014017	0.9486169
7283	TUBG1	tubulin, gamma 1	0.98571	1.04556	1.026877
7284	TUFM	Tu translation elongation factor, mitochondrial	0.95727	0.905902	1.073861
7286	TUFT1	tuftelin 1	1.00411	0.968859	0.9781448
7287	TULP1	tubby like protein 1	0.99816	1.034163	0.9988003
7288	TULP2	tubby like protein 2	1.00304	1.002482	1.0140858
7289	TULP3	tubby like protein 3	0.96831	0.988115	1.0427884
7290	HIRA	HIR histone cell cycle regulation defective homolog A (S. cerevisiae)	0.95343	1.026626	1.0498752
7291	TWIST1	twist homolog 1 (Drosophila)	0.9932	0.993525	1.0167475
7292	TNFSF4	tumor necrosis factor (ligand) superfamily, member 4	0.97712	0.926524	1.028312
7293	TNFRSF4	tumor necrosis factor receptor superfamily, member 4	0.99809	0.964352	1.0293222
7294	TXK	TXK tyrosine kinase	0.95116	0.857334	0.7907812
7295	TXN	thioredoxin	0.92965	0.945768	1.0629263
7296	TXNRD1	thioredoxin reductase 1	0.94084	1.012178	1.0827853
7297	TYK2	tyrosine kinase 2	0.97527	1.036656	1.0075462
7298	TYMS	thymidylate synthetase	1.00425	0.990011	1.0239709
7299	TYR	tyrosinase (oculocutaneous albinism IA)	1.01388	1.008632	1.0230165
7301	TYRO3	TYRO3 protein tyrosine kinase	0.98938	1.043873	1.0380638
7305	TYROBP	TYRO protein tyrosine kinase binding protein	0.9711	1.012825	0.9774011
7306	TYRP1	tyrosinase-related protein 1	0.99912	1.0296	1.0285249
7307	U2AF1	U2 small nuclear RNA auxiliary factor 1	0.98608	0.992196	0.9729351
7310	ZRSR1	zinc finger (CCCH type), RNA-binding motif and serine/arginine rich 1	1.0003	1.01414	1.0716279
7311	UBA52	ubiquitin A-52 residue ribosomal protein fusion product 1	0.95043	0.920933	1.0288158
7312	UBA52P1	ubiquitin A-52 residue ribosomal protein fusion product 1 pseudogene 1	1.00066	1.031614	0.9745343
7314	UBB	ubiquitin B	0.92424	0.979004	0.9420737
7316	UBC	ubiquitin C	0.94303	0.933524	0.9580784
7317	UBA1	ubiquitin-like modifier activating enzyme 1	0.95188	1.044038	1.0450286
7318	UBA7	ubiquitin-like modifier activating enzyme 7	0.9952	0.861856	0.9665206

7319	UBE2A	ubiquitin-conjugating enzyme E2A	0.96759	1.056799	1.0681425
7320	UBE2B	ubiquitin-conjugating enzyme E2B	1.01435	0.906049	0.9074801
7321	UBE2D1	ubiquitin-conjugating enzyme E2D 1	1.00646	1.066698	1.0348483
7322	UBE2D2	ubiquitin-conjugating enzyme E2D 2	1.00577	0.952191	0.917568
7323	UBE2D3	ubiquitin-conjugating enzyme E2D 3	1.01758	0.974181	0.9448411
7324	UBE2E1	ubiquitin-conjugating enzyme E2E 1	1.02235	0.951138	0.9448841
7325	UBE2E2	ubiquitin-conjugating enzyme E2E 2	1.00893	1.00063	1.009123
7326	UBE2G1	ubiquitin-conjugating enzyme E2G 1	0.96571	0.986303	0.9749541
7327	UBE2G2	ubiquitin-conjugating enzyme E2G 2	0.95271	0.95535	1.1210485
7328	UBE2H	ubiquitin-conjugating enzyme E2H	1.00079	1.00879	0.8960268
7329	UBE2I	ubiquitin-conjugating enzyme E2I	0.96761	0.972578	1.0035082
7332	UBE2L3	ubiquitin-conjugating enzyme E2L 3	0.95021	1.025692	1.0256505
7334	UBE2N	ubiquitin-conjugating enzyme E2N	0.92783	0.903176	0.9767162
7336	UBE2V2	ubiquitin-conjugating enzyme E2 variant 2	0.98143	0.959044	0.9459437
7337	UBE3A	ubiquitin protein ligase E3A	0.92186	0.931972	1.0439834
7339	UBE3AP2	ubiquitin protein ligase E3A pseudogene 2	0.98852	0.974954	1.0153173
7341	SUMO1	SMT3 suppressor of mif two 3 homolog 1 (S. cerevisiae)	0.99816	0.975095	0.9182332
7342	UBP1	upstream binding protein 1 (LBP-1a)	0.9982	1.009285	0.8812708
7343	UBTF	upstream binding transcription factor, RNA polymerase I	0.89762	0.92157	0.9588786
7345	UCHL1	ubiquitin carboxyl-terminal esterase L1 (ubiquitin thiolesterase)	1.01264	1.01229	1.0213393
7347	UCHL3	ubiquitin carboxyl-terminal esterase L3 (ubiquitin thiolesterase)	0.97738	0.979316	1.0721313
7348	UPK1B	uroplakin 1B	1.00393	1.051998	1.0025063
7349	UCN	urocortin	0.97334	1.021721	1.1000626
7350	UCP1	uncoupling protein 1 (mitochondrial, proton carrier)	0.99933	1.013468	1.0196516
7351	UCP2	uncoupling protein 2 (mitochondrial, proton carrier)	0.99336	0.884871	0.9694788
7352	UCP3	uncoupling protein 3 (mitochondrial, proton carrier)	1.00033	0.978624	1.0014717
7353	UFD1L	ubiquitin fusion degradation 1 like (yeast)	0.94695	0.978517	0.977838
7355	SLC35A2	solute carrier family 35 (UDP-galactose transporter), member A2	0.96385	1.023496	1.0158224
7356	SCGB1A1	secretoglobin, family 1A, member 1 (uteroglobin)	1.00538	0.964267	1.0136773
7357	UGCG	UDP-glucose ceramide glucosyltransferase	0.99815	1.007861	1.0244089
7358	UGDH	UDP-glucose 6-dehydrogenase	0.9734	0.968208	1.0370876
7360	UGP2	UDP-glucose pyrophosphorylase 2	1.01504	0.991717	1.0648118
7363	UGT2B4	UDP glucuronosyltransferase 2 family, polypeptide B4	1.02397	1.007752	1.0338584

7364	UGT2B7	UDP glucuronosyltransferase 2 family, polypeptide B7	1.00238	1.053185	1.0653076
7365	UGT2B10	UDP glucuronosyltransferase 2 family, polypeptide B10	1.00033	0.97649	1.0024191
7367	UGT2B17	UDP glucuronosyltransferase 2 family, polypeptide B17	1.0174	0.980972	1.0508221
7369	UMOD	uromodulin	1	1.004975	1.0264172
7371	UCK2	uridine-cytidine kinase 2	1.02445	1.014774	1.000974
7372	UMPS	uridine monophosphate synthetase	0.95468	0.898848	1.0504692
7373	COL14A1	collagen, type XIV, alpha 1	1.0014	1.013516	1.0148
7374	UNG	uracil-DNA glycosylase	0.95265	0.963891	1.0534274
7375	USP4	ubiquitin specific peptidase 4 (proto-oncogene)	1.03923	0.936221	0.953481
7376	NR1H2	nuclear receptor subfamily 1, group H, member 2	0.90663	0.927871	0.9631961
7378	UPP1	uridine phosphorylase 1	1.01833	0.999413	0.9681051
7379	UPK2	uroplakin 2	1.02309	1.011988	0.9527488
7380	UPK3A	uroplakin 3A	0.96997	1.017568	0.9694319
7381	UQCRB	ubiquinol-cytochrome c reductase binding protein	0.9549	0.927692	0.9326279
7384	UQCRC1	ubiquinol-cytochrome c reductase core protein I	0.99765	0.909085	1.0942315
7385	UQCRC2	ubiquinol-cytochrome c reductase core protein II	0.97773	0.890044	1.1161072
7386	UQCRFS1	ubiquinol-cytochrome c reductase, Rieske iron-sulfur polypeptide 1	1.04769	0.978307	0.9886193
7388	UQCRH	ubiquinol-cytochrome c reductase hinge protein	1.05485	1.022593	1.0637093
7389	UROD	uroporphyrinogen decarboxylase	0.96739	1.037631	0.9882816
7390	UROS	uroporphyrinogen III synthase	0.96576	0.956112	1.0338358
7391	USF1	upstream transcription factor 1	0.97267	1.050283	1.0188148
7392	USF2	upstream transcription factor 2, c-fos interacting	0.96292	1.030267	0.9860096
7398	USP1	ubiquitin specific peptidase 1	0.98537	0.975426	1.1069406
7399	USH2A	Usher syndrome 2A (autosomal recessive, mild)	1.01042	1.011555	1.0115063
7401	CLRN1	clarin 1	1.00173	0.989513	0.9998618
7402	UTRN	utrophin	1.01366	0.895346	0.949177
7403	KDM6A	lysine (K)-specific demethylase 6A	0.91782	1.12285	0.9480196
7404	UTY	ubiquitously transcribed tetratricopeptide repeat gene, Y-linked	0.99759	0.987993	1.0164487
7405	UVRAG	UV radiation resistance associated gene	0.98116	1.04883	0.9764489
7407	VARS	valyl-tRNA synthetase	0.99246	0.988924	1.0512709
7408	VASP	vasodilator-stimulated phosphoprotein	0.94783	1.023271	1.0036687
7409	VAV1	vav 1 guanine nucleotide exchange factor	0.9406	1.17367	0.9721967
7410	VAV2	vav 2 guanine nucleotide exchange factor	0.98749	0.979315	0.9839528

7411	VBP1	von Hippel-Lindau binding protein 1	0.93085	1.007173	1.0243974
7412	VCAM1	vascular cell adhesion molecule 1	0.99894	0.99342	1.0094887
7414	VCL	vinculin	0.99751	0.958709	0.9769412
7415	VCP	valosin containing protein	0.96183	0.973222	0.9547029
7416	VDAC1	voltage-dependent anion channel 1	1.01738	1.030578	1.0110113
7417	VDAC2	voltage-dependent anion channel 2	0.98676	0.980293	0.9706305
7419	VDAC3	voltage-dependent anion channel 3	0.98321	0.898763	0.9942355
7421	VDR	vitamin D (1,25- dihydroxyvitamin D3) receptor	0.98425	1.009978	1.0259654
7422	VEGFA	vascular endothelial growth factor A	0.99193	1.017913	0.9703951
7423	VEGFB	vascular endothelial growth factor B	0.97409	1.034241	0.9842692
7424	VEGFC	vascular endothelial growth factor C	0.99018	0.951597	1.0111381
7425	VGF	VGF nerve growth factor inducible	0.99413	1.010009	1.0524109
7428	VHL	von Hippel-Lindau tumor suppressor	0.9639	0.958076	0.9383934
7429	VIL1	villin 1	0.99828	0.958552	0.94697
7430	EZR	ezrin	1.01712	1.027816	0.9758371
7431	VIM	vimentin	0.96584	1.002442	0.9598251
7432	VIP	vasoactive intestinal peptide	1.01072	0.985127	1.0039695
7433	VIPR1	vasoactive intestinal peptide receptor 1	0.99253	0.937452	1.0080837
7434	VIPR2	vasoactive intestinal peptide receptor 2	0.99673	0.985371	1.0061734
7436	VLDLR	very low density lipoprotein receptor	0.99657	0.991216	1.0017373
7439	BEST1	bestrophin 1	0.96091	1.018671	0.9673792
7442	TRPV1	transient receptor potential cation channel, subfamily V, member 1	0.98332	1.015822	1.0222917
7443	VRK1	vaccinia related kinase 1	0.94879	0.972582	1.0250686
7444	VRK2	vaccinia related kinase 2	1.00802	1.000067	1.0139299
7447	VSNL1	visinin-like 1	1.00232	0.999907	1.008355
7448	VTN	vitronectin	1.00639	1.022957	1.0300615
7451	VWFP1	von Willebrand factor pseudogene 1	1.00277	0.969868	0.9762237
7453	WARS	tryptophanyl-tRNA synthetase	1.01317	0.980284	0.9753825
7454	WAS	Wiskott-Aldrich syndrome (eczema-thrombocytopenia)	0.94073	0.994961	0.9518267
7455	ZAN	zonadhesin	1.00273	1.022098	1.0146485
7456	WIPF1	WAS/WASL interacting protein family, member 1	1.01033	0.935365	0.9432687
7458	EIF4H	eukaryotic translation initiation factor 4H	1.04231	0.986972	0.9282575
7461	CLIP2	CAP-GLY domain containing linker protein 2	1.00458	0.958705	1.0425024

7462	LAT2	linker for activation of T cells family, member 2	0.98573	0.984733	1.0963366
7464	CORO2A	coronin, actin binding protein, 2A	0.95745	0.979289	0.994917
7465	WEE1	WEE1 homolog (S. pombe)	0.96151	0.971566	1.0216783
7466	WFS1	Wolfram syndrome 1 (wolframin)	1.01309	0.964324	1.0690694
7468	WHSC1	Wolf-Hirschhorn syndrome candidate 1	1.01447	0.984105	0.9945
7469	WHSC2	Wolf-Hirschhorn syndrome candidate 2	1.00043	0.955058	1.0024376
7471	WNT1	wingless-type MMTV integration site family, member 1	0.97147	1.051069	0.9780295
7472	WNT2	wingless-type MMTV integration site family member 2	1.00565	1.016159	1.0508554
7473	WNT3	wingless-type MMTV integration site family, member 3	1.015	1.039465	1.0224983
7474	WNT5A	wingless-type MMTV integration site family, member 5A	0.99132	1.010426	1.0172057
7475	WNT6	wingless-type MMTV integration site family, member 6	0.99188	1.00389	1.0615415
7476	WNT7A	wingless-type MMTV integration site family, member 7A	0.98832	0.974082	1.0010562
7477	WNT7B	wingless-type MMTV integration site family, member 7B	1.0005	1.012556	1.0313395
7478	WNT8A	wingless-type MMTV integration site family, member 8A	0.9846	0.981555	1.0281103
7479	WNT8B	wingless-type MMTV integration site family, member 8B	1.01305	0.961431	1.0113385
7480	WNT10B	wingless-type MMTV integration site family, member 10B	0.97009	1.001358	0.9994485
7481	WNT11	wingless-type MMTV integration site family, member 11	0.99471	0.945247	1.0148442
7482	WNT2B	wingless-type MMTV integration site family, member 2B	1.00463	1.015394	1.0106869
7483	WNT9A	wingless-type MMTV integration site family, member 9A	0.9837	0.98378	1.0092531
7484	WNT9B	wingless-type MMTV integration site family, member 9B	0.97985	0.953529	1.0058485
7485	WRB	tryptophan rich basic protein	0.98583	0.935371	0.9495124
7486	WRN	Werner syndrome, RecQ helicase-like	0.97643	0.932565	0.9699509
7490	WT1	Wilms tumor 1	0.99955	1.009429	1.0279303
7494	XBP1	X-box binding protein 1	0.99434	1.035923	1.0308763
7495	XBP1P1	X-box binding protein 1 pseudogene 1	0.99901	1.004825	1.0447321
7498	XDH	xanthine dehydrogenase	1.01324	0.993819	0.9994848
7499	XG	Xg blood group	0.99506	0.987949	1.0123072
7500	XGPY	Xg pseudogene, Y-linked	1.01448	1.004382	1.023444
7503	XIST	X (inactive)-specific transcript (non-protein coding)	1.00823	0.998188	0.9668397
7504	XK	X-linked Kx blood group (McLeod syndrome)	0.99236	0.965485	0.9716217
7507	XPA	xeroderma pigmentosum, complementation group A	0.93384	0.994014	0.9252666
7508	XPC	xeroderma pigmentosum, complementation group C	0.95418	1.026965	1.0346353
7511	XPNPEP1	X-prolyl aminopeptidase (aminopeptidase P) 1, soluble	0.99605	1.021479	1.0101854

7512	XPNPEP2	X-prolyl aminopeptidase (aminopeptidase P) 2, membrane-bound	1.01312	1.008779	0.9900424
7514	XPO1	exportin 1 (CRM1 homolog, yeast)	1.01332	0.817619	0.9839247
7515	XRCC1	X-ray repair complementing defective repair in Chinese hamster cells 1	0.94576	0.975382	1.0423378
7516	XRCC2	X-ray repair complementing defective repair in Chinese hamster cells 2	0.99891	1.016594	1.0584177
7517	XRCC3	X-ray repair complementing defective repair in Chinese hamster cells 3	0.98641	0.968543	1.0140484
7518	XRCC4	X-ray repair complementing defective repair in Chinese hamster cells 4	0.99457	0.949376	1.031368
7520	XRCC5	X-ray repair complementing defective repair in Chinese hamster cells 5 (double-strand-break rejoining)	1.00502	0.953606	1.0885431
7525	YES1	v-yes-1 Yamaguchi sarcoma viral oncogene homolog 1	1.00942	0.998408	0.9247838
7528	YY1	YY1 transcription factor	0.99451	0.987107	1.0354334
7529	YWHAB	tyrosine 3-monooxygenase/tryptophan 5-monooxygenase activation protein, beta polypeptide	0.97028	0.937775	1.0587976
7531	YWHAE	tyrosine 3-monooxygenase/tryptophan 5-monooxygenase activation protein, epsilon polypeptide	0.95928	0.999191	0.9349876
7532	YWHAG	tyrosine 3-monooxygenase/tryptophan 5-monooxygenase activation protein, gamma polypeptide	0.98659	0.972357	1.0644043
7533	YWHAH	tyrosine 3-monooxygenase/tryptophan 5-monooxygenase activation protein, eta polypeptide	0.94307	0.91051	0.9089639
7534	YWHAZ	tyrosine 3-monooxygenase/tryptophan 5-monooxygenase activation protein, zeta polypeptide	0.97565	0.932157	0.9450061
7535	ZAP70	zeta-chain (TCR) associated protein kinase 70kDa	0.97501	0.980714	0.9617295
7536	SF1	splicing factor 1	0.97625	0.926201	0.9352059
7538	ZFP36	zinc finger protein 36, C3H type, homolog (mouse)	0.99872	1.051893	0.8963948
7539	ZFP37	zinc finger protein 37 homolog (mouse)	0.98333	1.014712	1.0345788
7541	ZFP161	zinc finger protein 161 homolog (mouse)	0.9024	0.97768	0.9793763
7542	ZFPL1	zinc finger protein-like 1	0.95338	0.979196	1.0315898
7543	ZFX	zinc finger protein, X-linked	0.94292	0.996299	1.0220841
7544	ZFY	zinc finger protein, Y-linked	0.98994	0.922946	1.0080838
7545	ZIC1	Zic family member 1	1.00342	1.03976	1.008257
7546	ZIC2	Zic family member 2	0.98628	1.024147	1.0618942
7547	ZIC3	Zic family member 3	0.99292	1.025944	1.0125652
7549	ZNF2	zinc finger protein 2	0.97201	1.002721	1.0184195
7551	ZNF3	zinc finger protein 3	0.97204	0.934837	1.0035943
7552	ZNF711	zinc finger protein 711	0.98487	0.992442	0.9990625
7553	ZNF7	zinc finger protein 7	0.98905	1.001332	0.9640049
7554	ZNF8	zinc finger protein 8	0.95559	0.982423	0.9978079
7555	CNBP	CCHC-type zinc finger, nucleic acid binding protein	1.01883	1.029735	0.9349809
7556	ZNF10	zinc finger protein 10	0.93203	0.963795	0.9240049
7559	ZNF12	zinc finger protein 12	0.98209	1.004256	0.9915112

7561	ZNF14	zinc finger protein 14	0.89418	0.945603	0.9129955
7562	ZNF708	zinc finger protein 708	0.95167	1.000069	0.901889
7564	ZNF16	zinc finger protein 16	0.98088	0.989253	0.9989244
7565	ZNF17	zinc finger protein 17	0.90156	1.014404	1.0338302
7566	ZNF18	zinc finger protein 18	0.96253	1.000652	1.0163046
7567	ZNF19	zinc finger protein 19	0.97476	0.97262	1.0162222
7568	ZNF20	zinc finger protein 20	0.96991	1.033018	1.0612049
7569	ZNF182	zinc finger protein 182	0.93754	1.012611	1.0023684
7570	ZNF22	zinc finger protein 22 (KOX 15)	0.93574	0.938296	1.0591797
7571	ZNF23	zinc finger protein 23 (KOX 16)	0.95005	0.928928	1.0153784
7572	ZNF24	zinc finger protein 24	0.95696	0.925153	0.9595376
7574	ZNF26	zinc finger protein 26	0.94737	0.973382	1.0515919
7576	ZNF28	zinc finger protein 28	0.91504	0.89226	1.0509208
7579	ZSCAN20	zinc finger and SCAN domain containing 20	1.00324	1.008523	1.0179742
7580	ZNF32	zinc finger protein 32	0.94025	0.950357	0.9980601
7581	ZNF33A	zinc finger protein 33A	0.96205	0.986121	0.905601
7582	ZNF33B	zinc finger protein 33B	0.96924	1.081134	1.0211478
7584	ZNF35	zinc finger protein 35	1.01197	0.937845	1.0439013
7586	ZKSCAN1	zinc finger with KRAB and SCAN domains 1	0.99941	0.89302	1.059977
7587	ZNF37A	zinc finger protein 37A	0.90541	1.028068	1.0228869
7589	ZSCAN21	zinc finger and SCAN domain containing 21	0.9818	0.933417	1.0479826
7592	ZNF41	zinc finger protein 41	0.95819	0.959686	0.9947168
7593	MZF1	myeloid zinc finger 1	0.95835	0.987254	1.0167093
7594	ZNF43	zinc finger protein 43	0.93524	0.919847	0.9989548
7596	ZNF45	zinc finger protein 45	0.90707	0.99522	1.1391812
7597	ZBTB25	zinc finger and BTB domain containing 25	0.92826	0.948408	0.9856847
7617	ZNF66P	zinc finger protein 66, pseudogene	0.99623	0.985124	1.0022617
7620	ZNF69	zinc finger protein 69	0.96511	0.97563	0.985605
7621	ZNF70	zinc finger protein 70	1.01166	0.971134	1.0240737
7625	ZNF74	zinc finger protein 74	0.97082	1.008494	1.0674356
7626	ZNF75D	zinc finger protein 75D	0.9606	1.012398	0.9791878
7627	ZNF75A	zinc finger protein 75a	0.93797	0.962511	0.9832114
7628	ZNF75BP	zinc finger protein 75B, pseudogene	0.98589	1.022381	1.0995813

7629	ZNF76	zinc finger protein 76	1.00124	1.013585	0.9691633
7633	ZNF79	zinc finger protein 79	0.9559	1.02375	1.0292866
7634	ZNF80	zinc finger protein 80	0.96274	0.972516	0.9793192
7637	ZNF84	zinc finger protein 84	0.92264	0.966494	1.0626121
7638	ZNF221	zinc finger protein 221	0.97803	0.992737	1.007654
7639	ZNF85	zinc finger protein 85	0.9697	1.001352	1.0413034
7643	ZNF90	zinc finger protein 90	0.97747	0.979193	1.0004123
7644	ZNF91	zinc finger protein 91	0.87677	0.9366	0.9574249
7652	ZNF99	zinc finger protein 99	0.98677	0.988354	0.9804061
7673	ZNF222	zinc finger protein 222	1.00057	0.93407	0.9863385
7675	ZNF121	zinc finger protein 121	0.94827	1.025928	0.9142287
7678	ZNF124	zinc finger protein 124	0.98046	0.915281	0.9041407
7681	MKRN3	makorin ring finger protein 3	0.99855	0.971914	0.9812718
7682	MKRN4P	makorin ring finger protein 4, pseudogene	0.98433	0.971945	0.9938889
7683	MKRN5P	makorin ring finger protein 5, pseudogene	0.98493	1.035717	0.9904231
7686	MKRN7P	makorin ring finger protein 7, pseudogene	0.98995	1.008337	0.9862943
7690	ZNF131	zinc finger protein 131	0.97261	0.950103	0.9473495
7691	ZNF132	zinc finger protein 132	0.95208	0.957682	1.0078128
7692	ZNF133	zinc finger protein 133	0.99074	1.004005	1.0138786
7693	ZNF134	zinc finger protein 134	0.90058	0.877022	0.9828416
7694	ZNF135	zinc finger protein 135	0.98422	1.012567	0.9870982
7695	ZNF136	zinc finger protein 136	0.90976	0.847668	0.9132804
7696	ZNF137P	zinc finger protein 137, pseudogene	0.96488	1.023371	1.0181442
7697	ZNF138	zinc finger protein 138	0.98139	0.969498	0.9784821
7699	ZNF140	zinc finger protein 140	0.93686	0.994683	1.0357185
7700	ZNF141	zinc finger protein 141	0.95643	0.963655	0.9492893
7701	ZNF142	zinc finger protein 142	0.98762	0.941426	1.0769669
7702	ZNF143	zinc finger protein 143	0.90738	1.016249	1.008747
7704	ZBTB16	zinc finger and BTB domain containing 16	0.98989	1.029176	0.9754246
7705	ZNF146	zinc finger protein 146	0.94911	1.010105	0.9957202
7706	TRIM25	tripartite motif containing 25	0.97256	0.957196	1.2050434
7707	ZNF148	zinc finger protein 148	0.96897	0.947467	1.0334693
7709	ZBTB17	zinc finger and BTB domain containing 17	0.99211	1.006247	0.9603808

7710	ZNF154	zinc finger protein 154	0.94255	1.007713	0.9682675
7711	ZNF155	zinc finger protein 155	0.96564	1.027493	1.0337291
7716	VEZF1	vascular endothelial zinc finger 1	0.95493	0.952056	1.0728547
7718	ZNF165	zinc finger protein 165	0.98791	1.00843	0.9721376
7726	TRIM26	tripartite motif containing 26	0.99933	0.961536	1.034207
7727	ZNF174	zinc finger protein 174	0.92293	0.996001	1.0131534
7728	ZNF175	zinc finger protein 175	0.90232	0.913625	1.0400198
7730	ZNF177	zinc finger protein 177	0.97123	1.032112	1.0029445
7732	RNF112	ring finger protein 112	0.99088	0.984505	1.0381237
7733	ZNF180	zinc finger protein 180	0.94108	1.012234	1.0293633
7737	RNF113A	ring finger protein 113A	0.95256	0.985244	1.0901359
7738	ZNF184	zinc finger protein 184	0.93844	1.043758	0.8743769
7739	ZNF185	zinc finger protein 185 (LIM domain)	1.00709	0.88048	0.9555469
7741	ZNF187	zinc finger protein 187	0.96698	0.986393	1.0222538
7743	ZNF189	zinc finger protein 189	0.93961	0.998017	0.9375481
7745	ZNF192	zinc finger protein 192	0.98315	0.8906	0.9075921
7746	ZNF193	zinc finger protein 193	0.99612	0.963052	1.0508931
7748	ZNF195	zinc finger protein 195	0.93475	1.038454	1.0828674
7750	ZMYM2	zinc finger, MYM-type 2	0.94446	1.091493	0.9930414
7752	ZNF200	zinc finger protein 200	0.95748	0.976671	0.9955079
7753	ZNF202	zinc finger protein 202	0.95506	0.989213	1.0376294
7754	ZNF204P	zinc finger protein 204, pseudogene	0.96244	0.930606	0.9174472
7755	ZNF205	zinc finger protein 205	0.99714	0.992154	1.0349055
7756	ZNF207	zinc finger protein 207	0.93834	0.986588	1.1058612
7757	ZNF208	zinc finger protein 208	0.97286	0.97539	0.9629312
7760	ZNF213	zinc finger protein 213	0.99144	0.975393	1.0083883
7762	ZNF215	zinc finger protein 215	1.00563	1.051957	1.0340862
7763	ZFAND5	zinc finger, AN1-type domain 5	0.97637	0.968044	1.0055981
7764	ZNF217	zinc finger protein 217	0.94334	0.974075	1.1029368
7766	ZNF223	zinc finger protein 223	0.98848	1.006502	1.0861821
7767	ZNF224	zinc finger protein 224	0.94128	0.967388	1.1335002
7768	ZNF225	zinc finger protein 225	0.97108	1.034924	1.0350682
7769	ZNF226	zinc finger protein 226	0.97576	0.988489	1.0262012

7770	ZNF227	zinc finger protein 227	0.9568	1.065037	1.0205689
7771	ZFP112	zinc finger protein 112 homolog (mouse)	1.00952	0.987891	1.0109789
7772	ZNF229	zinc finger protein 229	0.97335	0.962365	1.0265439
7773	ZNF230	zinc finger protein 230	0.92271	0.973148	1.0229291
7775	ZNF232	zinc finger protein 232	0.94679	0.961403	1.014051
7776	ZNF236	zinc finger protein 236	0.95382	0.897036	1.0363818
7779	SLC30A1	solute carrier family 30 (zinc transporter), member 1	1.01553	0.951144	0.9195338
7780	SLC30A2	solute carrier family 30 (zinc transporter), member 2	1.01992	0.960985	0.9923398
7781	SLC30A3	solute carrier family 30 (zinc transporter), member 3	1.01429	1.017719	1.0114913
7782	SLC30A4	solute carrier family 30 (zinc transporter), member 4	0.95468	1.036808	0.9718147
7783	ZP2	zona pellucida glycoprotein 2 (sperm receptor)	1.004	0.994516	1.0001341
7784	ZP3	zona pellucida glycoprotein 3 (sperm receptor)	1.00426	1.007409	1.0153205
7786	MAP3K12	mitogen-activated protein kinase kinase kinase 12	0.95714	0.935106	0.9764339
7789	ZXDA	zinc finger, X-linked, duplicated A	0.96447	0.926659	0.9415133
7791	ZYX	zyxin	0.99665	0.936081	0.950507
7798	LUZP1	leucine zipper protein 1	0.97945	1.014133	0.9406151
7799	PRDM2	PR domain containing 2, with ZNF domain	1.00287	0.985989	0.9256281
7802	DNALI1	dynein, axonemal, light intermediate chain 1	1.00692	0.997168	1.0216283
7803	PTP4A1	protein tyrosine phosphatase type IVA, member 1	1.00847	0.944278	0.8876829
7804	LRP8	low density lipoprotein receptor-related protein 8, apolipoprotein e receptor	1.00172	0.954699	1.029631
7805	LAPTM5	lysosomal protein transmembrane 5	1.02164	0.986066	0.9439261
7809	BSND	Bartter syndrome, infantile, with sensorineural deafness (Barttin)	0.99918	1.029552	1.0073142
7812	CSDE1	cold shock domain containing E1, RNA-binding	1.01175	0.931916	0.9675762
7813	EVI5	ecotropic viral integration site 5	1.01319	0.915913	0.9692139
7818	DAP3	death associated protein 3	0.99314	0.979971	1.0171072
7827	NPHS2	nephrosis 2, idiopathic, steroid-resistant (podocin)	1.02414	1.042108	0.9958148
7832	BTG2	BTG family, member 2	1.02023	0.937424	0.9431137
7837	PXDN	peroxidasin homolog (Drosophila)	1.01018	1.011158	1.0200458
7840	ALMS1	Alstrom syndrome 1	0.98044	0.952548	0.998309
7841	MOGS	mannosyl-oligosaccharide glucosidase	0.98542	1.035523	1.0465586
7844	RNF103	ring finger protein 103	1.01414	0.967658	0.9381294
7846	TUBA1A	tubulin, alpha 1a	0.97409	0.930296	0.9909496
7849	PAX8	paired box 8	0.99981	0.992616	1.0197058

7850	IL1R2	interleukin 1 receptor, type II	1.02139	0.983986	0.9406205
7851	MALL	mal, T-cell differentiation protein-like	0.98611	0.992951	0.9961601
7852	CXCR4	chemokine (C-X-C motif) receptor 4	0.96604	1.038407	0.9116143
7855	FZD5	frizzled family receptor 5	1.01806	0.997279	1.039793
7857	SCG2	secretogranin II	0.98978	1.016189	1.0052799
7862	BRPF1	bromodomain and PHD finger containing, 1	0.98892	0.912066	1.042946
7866	IFRD2	interferon-related developmental regulator 2	0.99232	0.952381	1.0459653
7869	SEMA3B	sema domain, immunoglobulin domain (Ig), short basic domain, secreted, (semaphorin) 3B	0.99831	1.009019	1.0114372
7871	SLMAP	sarcolemma associated protein	0.99862	0.948786	0.9635442
7873	MANF	mesencephalic astrocyte-derived neurotrophic factor	0.97467	0.965575	0.9925785
7874	USP7	ubiquitin specific peptidase 7 (herpes virus-associated)	0.9698	0.901491	0.9462336
7879	RAB7A	RAB7A, member RAS oncogene family	0.99484	0.951847	0.9488881
7881	KCNAB1	potassium voltage-gated channel, shaker-related subfamily, beta member 1	1.01196	1.007995	1.0047933
7884	SLBP	stem-loop binding protein	0.99076	0.974159	1.0863491
7903	ST8SIA4	ST8 alpha-N-acetyl-neuraminide alpha-2,8-sialyltransferase 4	1.00769	1.039437	1.0134357
7905	REEP5	receptor accessory protein 5	1.02549	0.952981	1.0907441
7913	DEK	DEK oncogene	0.99393	0.932138	1.1085973
7915	ALDH5A1	aldehyde dehydrogenase 5 family, member A1	0.9613	0.951522	1.0469915
7916	PRRC2A	proline-rich coiled-coil 2A	0.99584	0.994663	1.0381458
7917	BAG6	BCL2-associated athanogene 6	0.99372	0.968794	1.0833122
7918	GPANK1	G patch domain and ankyrin repeats 1	0.99258	0.977408	0.9587126
7919	DDX39B	DEAD (Asp-Glu-Ala-Asp) box polypeptide 39B	1.03115	0.902298	1.0429382
7920	ABHD16A	abhydrolase domain containing 16A	0.9664	0.985051	0.940297
7922	SLC39A7	solute carrier family 39 (zinc transporter), member 7	0.94474	0.901089	1.0951541
7923	HSD17B8	hydroxysteroid (17-beta) dehydrogenase 8	0.96912	0.983621	1.0251164
7936	RDBP	RD RNA binding protein	0.9372	0.998634	1.0047228
7940	LST1	leukocyte specific transcript 1	0.99487	0.952834	1.0539288
7941	PLA2G7	phospholipase A2, group VII (platelet-activating factor acetylhydrolase, plasma)	1.05312	1.011006	1.0264285
7942	TFEB	transcription factor EB	0.99137	0.995394	1.0147388
7957	EPM2A	epilepsy, progressive myoclonus type 2A, Lafora disease (laforin)	0.99828	1.010806	0.9617221
7965	AIMP2	aminoacyl tRNA synthetase complex-interacting multifunctional protein 2	0.96967	0.92791	1.0639133
7975	MAFK	v-maf musculoaponeurotic fibrosarcoma oncogene homolog K (avian)	0.98064	0.961053	0.9584455
7976	FZD3	frizzled family receptor 3	0.98146	0.960931	0.9692583

7978	MTERF	mitochondrial transcription termination factor	0.98158	0.943399	1.0497045
7979	SHFM1	split hand/foot malformation (ectrodactyly) type 1	1.04397	1.007718	0.9496879
7980	TFPI2	tissue factor pathway inhibitor 2	0.99736	1.003314	1.0310976
7982	ST7	suppression of tumorigenicity 7	0.99788	0.967144	0.9951242
7984	ARHGEF5	Rho guanine nucleotide exchange factor (GEF) 5	0.95811	1.077313	1.0108559
7988	ZNF212	zinc finger protein 212	0.98644	0.980842	1.0166183
7991	TUSC3	tumor suppressor candidate 3	0.99385	0.996154	1.0186271
7993	UBXN8	UBX domain protein 8	0.98674	0.99795	1.0574352
7994	MYST3	MYST histone acetyltransferase (monocytic leukemia) 3	0.93617	1.052384	0.9606272
8000	PSCA	prostate stem cell antigen	1.00637	1.002451	1.0119255
8001	GLRA3	glycine receptor, alpha 3	0.99344	1.00548	1.0185636
8013	NR4A3	nuclear receptor subfamily 4, group A, member 3	0.99183	1.00509	0.9254193
8019	BRD3	bromodomain containing 3	0.98119	0.995837	1.0361551
8021	NUP214	nucleoporin 214kDa	0.96341	0.921678	0.9516597
8022	LHX3	LIM homeobox 3	1.00671	0.989766	1.0810161
8027	STAM	signal transducing adaptor molecule (SH3 domain and ITAM motif) 1	0.95234	0.994933	1.0021045
8028	MLLT10	myeloid/lymphoid or mixed-lineage leukemia (trithorax homolog, Drosophila); translocated to, 10	0.94626	0.959695	1.0413541
8029	CUBN	cubilin (intrinsic factor-cobalamin receptor)	0.96289	0.979674	0.924574
8030	CCDC6	coiled-coil domain containing 6	0.98946	0.990198	1.0639806
8031	NCOA4	nuclear receptor coactivator 4	0.95014	1.009584	1.0279587
8034	SLC25A16	solute carrier family 25 (mitochondrial carrier; Graves disease autoantigen), member 16	0.9433	0.954032	0.9979694
8036	SHOC2	soc-2 suppressor of clear homolog (C. elegans)	0.98517	1.116502	1.1321155
8038	ADAM12	ADAM metallopeptidase domain 12	1.00202	0.991992	0.9828401
8045	RASSF7	Ras association (RalGDS/AF-6) domain family (N-terminal) member 7	0.98154	0.953684	1.0757245
8048	CSRP3	cysteine and glycine-rich protein 3 (cardiac LIM protein)	0.99795	0.966015	1.0112169
8050	PDHX	pyruvate dehydrogenase complex, component X	0.92781	0.999269	1.0672789
8061	FOSL1	FOS-like antigen 1	0.97867	0.941338	0.9640901
8065	CUL5	cullin 5	0.94247	1.053245	1.1315004
8073	PTP4A2	protein tyrosine phosphatase type IVA, member 2	1.03257	1.015934	0.8983872
8074	FGF23	fibroblast growth factor 23	0.99125	1.000416	1.0028134
8076	MFAP5	microfibrillar associated protein 5	0.99056	0.978335	1.0252064
8078	USP5	ubiquitin specific peptidase 5 (isopeptidase T)	0.91368	0.96855	1.0854606
8079	MLF2	myeloid leukemia factor 2	0.96043	1.043559	1.0763799

8082	SSPN	sarcospan (Kras oncogene-associated gene)	0.99294	0.999164	1.0200651
8085	MLL2	myeloid/lymphoid or mixed-lineage leukemia 2	0.9875	1.025649	0.9406795
8086	AAAS	achalasia, adrenocortical insufficiency, alacrimia	0.97006	0.875126	1.0328709
8087	FXR1	fragile X mental retardation, autosomal homolog 1	0.96765	1.004615	0.9643963
8089	YEATS4	YEATS domain containing 4	0.89534	0.994073	1.0717543
8091	HMGA2	high mobility group AT-hook 2	0.99418	1.023953	0.9836359
8092	ALX1	ALX homeobox 1	1.00558	1.026493	1.0099991
8099	CDK2AP1	cyclin-dependent kinase 2 associated protein 1	0.96215	0.977135	1.04204
8100	IFT88	intraflagellar transport 88 homolog (Chlamydomonas)	0.95711	0.995245	1.0430541
8106	PABPN1	poly(A) binding protein, nuclear 1	0.98655	0.968156	0.9582405
8110	DPF3	D4, zinc and double PHD fingers, family 3	1.00149	0.990282	1.0105513
8111	GPR68	G protein-coupled receptor 68	0.98066	1.046296	1.0343689
8120	AP3B2	adaptor-related protein complex 3, beta 2 subunit	0.99699	0.998805	0.9859022
8125	ANP32A	acidic (leucine-rich) nuclear phosphoprotein 32 family, member A	0.97644	1.044834	0.9462267
8128	ST8SIA2	ST8 alpha-N-acetyl-neuraminide alpha-2,8-sialyltransferase 2	0.99819	1.002439	1.0023347
8131	NPRL3	nitrogen permease regulator-like 3 (S. cerevisiae)	0.99079	0.983436	1.0732505
8139	GAN	gigaxonin	0.97495	0.991379	1.0731234
8140	SLC7A5	solute carrier family 7 (cationic amino acid transporter, y+ system), member 5	1.01043	0.993672	1.0061068
8148	TAF15	TAF15 RNA polymerase II, TATA box binding protein (TBP)-associated factor, 68kDa	0.95499	0.951246	1.0082149
8153	RND2	Rho family GTPase 2	1.0111	1.007233	0.9856214
8161	COIL	coilin	0.94977	0.930661	1.0314066
8165	AKAP1	A kinase (PRKA) anchor protein 1	0.97981	0.900071	1.0003245
8170	SLC14A2	solute carrier family 14 (urea transporter), member 2	1.0111	1.029523	0.9542519
8174	MADCAM1	mucosal vascular addressin cell adhesion molecule 1	1.00932	0.990175	1.0758477
8175	SF3A2	splicing factor 3a, subunit 2, 66kDa	0.94713	0.959809	1.0009449
8178	ELL	elongation factor RNA polymerase II	0.98839	1.020749	0.9724066
8187	ZNF239	zinc finger protein 239	0.97621	0.980664	1.0180894
8189	SYMPK	symplekin	0.96431	0.983563	1.0238539
8190	MIA	melanoma inhibitory activity	0.99975	0.984855	1.0303503
8192	CLPP	ClpP caseinolytic peptidase, ATP-dependent, proteolytic subunit homolog (E. coli)	0.9593	0.935032	1.065681
8193	DPF1	D4, zinc and double PHD fingers family 1	1.00607	1.030462	1.019663
8195	MKKS	McKusick-Kaufman syndrome	0.94946	0.989085	1.031228
8200	GDF5	growth differentiation factor 5	0.98845	1.016992	0.9774617

8202	NCOA3	nuclear receptor coactivator 3	0.98514	1.000699	1.1159714
8204	NRIP1	nuclear receptor interacting protein 1	0.96708	0.914767	1.0476937
8208	CHAF1B	chromatin assembly factor 1, subunit B (p60)	0.99019	0.965086	0.9920914
8209	C21orf33	chromosome 21 open reading frame 33	0.96391	0.991587	0.9736186
8214	DGCR6	DiGeorge syndrome critical region gene 6	0.98428	0.97913	0.9746124
8216	LZTR1	leucine-zipper-like transcription regulator 1	0.98078	0.925594	1.0094949
8218	CLTCL1	clathrin, heavy chain-like 1	0.99434	0.99484	1.0136527
8220	DGCR14	DiGeorge syndrome critical region gene 14	0.91946	0.904733	1.0267134
8224	SYN3	synapsin III	1.00046	0.977396	1.0002848
8226	HDHD1	haloacid dehalogenase-like hydrolase domain containing 1	0.98099	0.986492	0.9916143
8228	PNPLA4	patatin-like phospholipase domain containing 4	0.91031	0.944779	1.0158759
8233	ZRSR2	zinc finger (CCCH type), RNA-binding motif and serine/arginine rich 2	1.00331	0.991868	0.999417
8237	USP11	ubiquitin specific peptidase 11	0.91767	1.030421	1.0497549
8239	USP9X	ubiquitin specific peptidase 9, X-linked	0.95572	1.025451	0.9675009
8241	RBM10	RNA binding motif protein 10	0.95495	1.005677	1.0001229
8242	KDM5C	lysine (K)-specific demethylase 5C	0.97408	1.022794	0.9763822
8243	SMC1A	structural maintenance of chromosomes 1A	0.95531	0.983012	1.0008201
8251	HNRNPDP	heterogeneous nuclear ribonucleoprotein D (AU-rich element RNA binding protein 1, 37kDa) pseudogene	0.98841	0.98903	1.050477
8260	NAA10	N(alpha)-acetyltransferase 10, NatA catalytic subunit	0.94861	1.013975	1.0753805
8266	UBL4A	ubiquitin-like 4A	0.89095	0.982989	1.0435688
8269	TMEM187	transmembrane protein 187	0.95544	0.976906	1.0465214
8270	LAGE3	L antigen family, member 3	0.91686	0.877062	1.0549514
8273	SLC10A3	solute carrier family 10 (sodium/bile acid cotransporter family), member 3	0.92874	1.025704	1.0320069
8277	TKTL1	transketolase-like 1	0.97641	1.062342	0.8813042
8284	KDM5D	lysine (K)-specific demethylase 5D	0.98561	1.000367	1.0031507
8287	USP9Y	ubiquitin specific peptidase 9, Y-linked	0.9941	0.967784	1.030553
8288	EPX	eosinophil peroxidase	0.9914	1.03002	1.0076971
8289	ARID1A	AT rich interactive domain 1A (SWI-like)	0.97702	0.924645	0.9557108
8290	HIST3H3	histone cluster 3, H3	0.98749	1.000867	1.0996164
8291	DYSF	dysferlin, limb girdle muscular dystrophy 2B (autosomal recessive)	1.00839	0.947281	0.9944494
8292	COLQ	collagen-like tail subunit (single strand of homotrimer) of asymmetric acetylcholinesterase	0.98655	0.996198	1.009812
8294	HIST1H4I	histone cluster 1, H4i	0.91844	0.897545	1.0323074
8295	TRRAP	transformation/transcription domain-associated protein	0.97372	0.87326	0.995188

8301	PICALM	phosphatidylinositol binding clathrin assembly protein	1.01726	1.083873	1.067045
8302	KLRC4	killer cell lectin-like receptor subfamily C, member 4	1.04003	1.091782	1.0306487
8303	SNN	stannin	0.96898	0.916379	0.9487342
8309	ACOX2	acyl-CoA oxidase 2, branched chain	1.01716	0.988159	0.9964565
8310	ACOX3	acyl-CoA oxidase 3, pristanoyl	1.00096	0.985621	1.0265026
8312	AXIN1	axin 1	0.98074	1.031667	0.9778609
8313	AXIN2	axin 2	0.97876	0.992274	0.9658867
8314	BAP1	BRCA1 associated protein-1 (ubiquitin carboxy-terminal hydrolase)	0.96147	0.955352	0.9668407
8315	BRAP	BRCA1 associated protein	0.91834	0.934994	1.029218
8317	CDC7	cell division cycle 7 homolog (S. cerevisiae)	0.98134	1.009482	1.0518829
8318	CDC45	cell division cycle 45 homolog (S. cerevisiae)	0.99464	1.001321	1.0107271
8320	EOMES	eomesodermin	0.97926	1.006132	0.9471999
8321	FZD1	frizzled family receptor 1	0.99143	0.984259	1.0998906
8322	FZD4	frizzled family receptor 4	1.00894	1.026759	0.9798049
8323	FZD6	frizzled family receptor 6	1.01248	0.905135	1.019449
8324	FZD7	frizzled family receptor 7	1.02368	1.031069	1.0149187
8325	FZD8	frizzled family receptor 8	0.98088	0.978579	1.0741807
8326	FZD9	frizzled family receptor 9	1.02213	1.017445	1.0212428
8328	GFI1B	growth factor independent 1B transcription repressor	0.95404	0.952037	0.9887183
8330	HIST1H2AK	histone cluster 1, H2ak	0.96084	0.906147	0.9757214
8331	HIST1H2AJ	histone cluster 1, H2aj	1.02184	0.970972	1.0093934
8332	HIST1H2AL	histone cluster 1, H2al	0.94009	0.928423	1.045952
8333	HIST1H2AP	histone cluster 1, H2a, pseudogene 4	1.00092	0.907799	1.0619625
8334	HIST1H2AC	histone cluster 1, H2ac	0.97792	0.83047	1.1385575
8335	HIST1H2AB	histone cluster 1, H2ab	0.95234	0.974016	0.9290973
8336	HIST1H2AM	histone cluster 1, H2am	0.98694	0.893049	0.9835344
8338	HIST2H2AC	histone cluster 2, H2ac	0.99587	0.814522	0.9638632
8339	HIST1H2BG	histone cluster 1, H2bg	0.95879	0.923475	0.9787353
8341	HIST1H2BN	histone cluster 1, H2bn	0.99481	0.922558	1.0576853
8342	HIST1H2BM	histone cluster 1, H2bm	0.99235	0.961482	0.9992827
8345	HIST1H2BH	histone cluster 1, H2bh	0.93866	0.8385	0.9753289
8346	HIST1H2BI	histone cluster 1, H2bi	0.92424	0.940887	1.0104376
8347	HIST1H2BC	histone cluster 1, H2bc	0.93531	0.896538	0.9775972

8348	HIST1H2BO	histone cluster 1, H2bo	0.97959	0.951623	1.0377838
8349	HIST2H2BE	histone cluster 2, H2be	0.97379	0.956932	0.9075767
8352	HIST1H3C	histone cluster 1, H3c	0.9765	0.917476	1.1129285
8353	HIST1H3E	histone cluster 1, H3e	0.96284	0.942927	1.0255294
8354	HIST1H3I	histone cluster 1, H3i	0.9844	0.941594	1.1006367
8355	HIST1H3G	histone cluster 1, H3g	0.99603	0.923433	1.0883003
8356	HIST1H3J	histone cluster 1, H3j	1.01277	0.884604	1.0330411
8360	HIST1H4D	histone cluster 1, H4d	0.9181	0.923749	1.0103508
8361	HIST1H4F	histone cluster 1, H4f	0.94333	0.958117	1.0837352
8364	HIST1H4C	histone cluster 1, H4c	0.89366	0.817021	1.069246
8365	HIST1H4H	histone cluster 1, H4h	0.92758	0.900176	0.9696684
8366	HIST1H4B	histone cluster 1, H4b	0.95583	0.849445	0.9807809
8367	HIST1H4E	histone cluster 1, H4e	0.88515	0.891135	1.0956712
8368	HIST1H4L	histone cluster 1, H4l	0.88463	0.936061	0.9964336
8369	HIST1H4G	histone cluster 1, H4g	0.98446	1.00905	1.04045
8372	HYAL3	hyaluronoglucosaminidase 3	0.98767	0.96084	1.0441996
8379	MAD1L1	MAD1 mitotic arrest deficient-like 1 (yeast)	0.99983	1.001623	1.0052779
8382	NME5	non-metastatic cells 5, protein expressed in (nucleoside-diphosphate kinase)	0.98497	0.988256	0.9776321
8383	OR1A1	olfactory receptor, family 1, subfamily A, member 1	0.98002	1.020139	1.077686
8384	OR1D3P	olfactory receptor, family 1, subfamily D, member 3 pseudogene	1.01879	0.92899	1.0273523
8387	OR1E1	olfactory receptor, family 1, subfamily E, member 1	1.01482	1.016797	1.0318455
8389	OR1E3	olfactory receptor, family 1, subfamily E, member 3 (gene/pseudogene)	0.95911	1.000343	0.9416717
8390	OR1G1	olfactory receptor, family 1, subfamily G, member 1	1.0151	0.99498	0.9822959
8391	OR1P1	olfactory receptor, family 1, subfamily P, member 1 (gene/pseudogene)	1.01734	0.989783	1.0151191
8394	PIP5K1A	phosphatidylinositol-4-phosphate 5-kinase, type I, alpha	1.00159	1.058071	0.9695986
8395	PIP5K1B	phosphatidylinositol-4-phosphate 5-kinase, type I, beta	0.98059	0.993982	1.0040337
8396	PIP4K2B	phosphatidylinositol-5-phosphate 4-kinase, type II, beta	0.98219	0.937656	1.0473784
8398	PLA2G6	phospholipase A2, group VI (cytosolic, calcium-independent)	0.97068	0.939251	1.0173492
8399	PLA2G10	phospholipase A2, group X	0.99816	1.062821	0.9939559
8402	SLC25A11	solute carrier family 25 (mitochondrial carrier; oxoglutarate carrier), member 11	0.96323	0.924379	1.0646755
8403	SOX14	SRY (sex determining region Y)-box 14	1.0213	0.978239	0.9897544
8404	SPARCL1	SPARC-like 1 (hevin)	1.01132	0.999449	1.0075925
8405	SPOP	speckle-type POZ protein	0.92892	0.939508	0.9879224

8406	SRPX	sushi-repeat containing protein, X-linked	0.99298	0.996834	1.0204968
8407	TAGLN2	transgelin 2	1.0136	0.951633	0.93579
8408	ULK1	unc-51-like kinase 1 (C. elegans)	0.99243	1.033622	1.0020249
8409	UXT	ubiquitously-expressed transcript	0.96987	0.977204	0.9876335
8411	EEA1	early endosome antigen 1	0.9471	0.957878	0.9947461
8412	BCAR3	breast cancer anti-estrogen resistance 3	1.01374	1.010105	0.9855152
8416	ANXA9	annexin A9	0.99991	1.015935	0.9947568
8417	STX7	syntaxin 7	1.02416	0.964589	1.1132016
8418	CMAHP	cytidine monophospho-N-acetylneuraminic acid hydroxylase, pseudogene	0.98354	0.999475	0.939257
8419	BFSP2	beaded filament structural protein 2, phakinin	1.00522	0.995814	1.030334
8424	BBOX1	butyrobetaine (gamma), 2-oxoglutarate dioxygenase (gamma-butyrobetaine hydroxylase) 1	0.99282	1.003912	1.0172445
8425	LTBP4	latent transforming growth factor beta binding protein 4	0.9772	1.017314	1.0583957
8427	ZNF282	zinc finger protein 282	0.9947	0.969115	1.0536186
8428	STK24	serine/threonine kinase 24	0.99943	1.008372	0.9783727
8431	NR0B2	nuclear receptor subfamily 0, group B, member 2	1.01922	1.003568	0.9615511
8433	UTF1	undifferentiated embryonic cell transcription factor 1	0.96538	1.050553	1.0658243
8434	RECK	reversion-inducing-cysteine-rich protein with kazal motifs	0.95886	0.957181	0.9444026
8435	SOAT2	sterol O-acyltransferase 2	0.9776	0.983939	0.9917979
8436	SDPR	serum deprivation response	0.97138	0.941996	0.9742499
8437	RASAL1	RAS protein activator like 1 (GAP1 like)	0.97578	1.013626	0.9925775
8438	RAD54L	RAD54-like (S. cerevisiae)	1.01577	0.987216	1.0076827
8439	NSMAF	neutral sphingomyelinase (N-SMase) activation associated factor	0.9656	1.034453	1.0599452
8440	NCK2	NCK adaptor protein 2	0.99075	0.992399	0.9469673
8443	GNPAT	glyceronephosphate O-acyltransferase	1.01342	1.067312	1.035866
8444	DYRK3	dual-specificity tyrosine-(Y)-phosphorylation regulated kinase 3	0.98515	1.010629	1.0384545
8445	DYRK2	dual-specificity tyrosine-(Y)-phosphorylation regulated kinase 2	0.95098	0.94776	0.9248136
8446	DUSP11	dual specificity phosphatase 11 (RNA/RNP complex 1-interacting)	0.97292	0.935744	1.0204985
8448	DOC2A	double C2-like domains, alpha	0.98831	1.006773	1.0178763
8449	DHX16	DEAH (Asp-Glu-Ala-His) box polypeptide 16	0.96618	0.929061	1.0056547
8450	CUL4B	cullin 4B	0.94876	1.026467	1.0695505
8451	CUL4A	cullin 4A	0.9348	0.957872	1.0263185
8452	CUL3	cullin 3	0.98434	0.877758	0.9440941
8453	CUL2	cullin 2	0.92091	0.968863	1.0579406

8454	CUL1	cullin 1	0.97493	1.046076	1.0017699
8455	ATRN	attractin	0.96149	0.965093	1.0318327
8456	FOXN1	forkhead box N1	1.00092	0.999395	1.0163082
8458	TTF2	transcription termination factor, RNA polymerase II	0.99002	1.005141	1.0138263
8459	TPST2	tyrosylprotein sulfotransferase 2	0.97572	1.000695	1.035131
8460	TPST1	tyrosylprotein sulfotransferase 1	0.99649	0.990834	0.9997324
8462	KLF11	Kruppel-like factor 11	1.0042	0.956488	0.9540209
8463	TEAD2	TEA domain family member 2	1.01141	1.016876	1.0190046
8464	SUPT3H	suppressor of Ty 3 homolog (S. cerevisiae)	0.98936	0.994435	0.967016
8467	SMARCA5	SWI/SNF related, matrix associated, actin dependent regulator of chromatin, subfamily a, member 5	0.95862	0.937973	0.978546
8468	FKBP6	FK506 binding protein 6, 36kDa	0.98508	0.979693	1.0227783
8470	SORBS2	sorbin and SH3 domain containing 2	1.01006	1.004209	1.0126667
8471	IRS4	insulin receptor substrate 4	1.00015	0.961082	1.0045455
8473	OGT	O-linked N-acetylglucosamine (GlcNAc) transferase (UDP-N-acetylglucosamine:polypeptide-N-acetylglucosan	0.97812	0.962459	1.1226317
8475	[No Symbo	[No Name]	0.97939	0.998912	1.0759408
8476	CDC42BPA	CDC42 binding protein kinase alpha (DMPK-like)	0.99894	0.958183	1.0097825
8477	GPR65	G protein-coupled receptor 65	0.97864	1.075558	1.039844
8479	HIRIP3	HIRA interacting protein 3	0.97237	0.985458	1.0139956
8480	RAE1	RAE1 RNA export 1 homolog (S. pombe)	0.9452	0.930052	1.0476152
8481	OFD1	oral-facial-digital syndrome 1	0.92748	1.031157	1.0161829
8482	SEMA7A	semaphorin 7A, GPI membrane anchor (John Milton Hagen blood group)	0.9691	1.014007	1.0159161
8483	CILP	cartilage intermediate layer protein, nucleotide pyrophosphohydrolase	0.99692	1.017754	1.0005705
8484	GALR3	galanin receptor 3	0.99284	1.016641	1.0214185
8487	SIP1	survival of motor neuron protein interacting protein 1	0.98573	0.992823	1.0122502
8489	RPL23AP3	ribosomal protein L23a pseudogene 3	0.95612	0.965289	1.1123931
8490	RGS5	regulator of G-protein signaling 5	1.01168	1.013482	1.0217003
8491	MAP4K3	mitogen-activated protein kinase kinase kinase kinase 3	1.00837	0.937447	0.9848515
8492	PRSS12	protease, serine, 12 (neurotrypsin, motopsin)	1.00892	0.982522	1.0306627
8493	PPM1D	protein phosphatase, Mg2+/Mn2+ dependent, 1D	0.91854	0.998025	1.0895576
8495	PPFIBP2	PTPRF interacting protein, binding protein 2 (liprin beta 2)	0.97637	0.990783	1.0295029
8496	PPFIBP1	PTPRF interacting protein, binding protein 1 (liprin beta 1)	0.98267	0.986156	1.0092472
8497	PPFIA4	protein tyrosine phosphatase, receptor type, f polypeptide (PTPRF), interacting protein (liprin), alpha 4	1.0176	0.980965	1.0160614
8498	RANBP3	RAN binding protein 3	0.95769	0.98304	1.0206454

8499	PPFIA2	protein tyrosine phosphatase, receptor type, f polypeptide (PTPRF), interacting protein (liprin), alpha 2	0.98874	0.990635	1.0043988
8500	PPFIA1	protein tyrosine phosphatase, receptor type, f polypeptide (PTPRF), interacting protein (liprin), alpha 1	0.97955	0.980566	0.955679
8501	SLC43A1	solute carrier family 43, member 1	0.9656	0.976933	1.0179227
8502	PKP4	plakophilin 4	1.0006	0.983517	1.009004
8503	PIK3R3	phosphoinositide-3-kinase, regulatory subunit 3 (gamma)	1.01058	1.014813	1.0368434
8504	PEX3	peroxisomal biogenesis factor 3	0.98925	0.990569	1.0102356
8506	CNTNAP1	contactin associated protein 1	0.99543	0.997086	1.0031414
8507	ENC1	ectodermal-neural cortex 1 (with BTB-like domain)	1.04652	0.980874	0.9506674
8508	NIPSNAP1	nipsnap homolog 1 (C. elegans)	0.96243	1.022676	0.9912962
8509	NDST2	N-deacetylase/N-sulfotransferase (heparan glucosaminyl) 2	0.98304	1.031199	0.9918266
8512	MBL1P	mannose-binding lectin (protein A) 1, pseudogene	1.00638	1.043621	1.0200825
8513	LIPF	lipase, gastric	0.9954	0.999727	0.9832262
8514	KCNAB2	potassium voltage-gated channel, shaker-related subfamily, beta member 2	1.00928	0.95067	0.971671
8515	ITGA10	integrin, alpha 10	1.00767	1.009973	1.0032303
8516	ITGA8	integrin, alpha 8	1.00502	0.995148	0.9944494
8517	IKBKG	inhibitor of kappa light polypeptide gene enhancer in B-cells, kinase gamma	0.9135	1.047836	1.0216581
8518	IKBKAP	inhibitor of kappa light polypeptide gene enhancer in B-cells, kinase complex-associated protein	0.95023	0.945488	1.0733735
8519	IFITM1	interferon induced transmembrane protein 1 (9-27)	0.91686	0.837607	1.0871164
8520	HAT1	histone acetyltransferase 1	0.98134	0.944821	1.0156239
8521	GCM1	glial cells missing homolog 1 (Drosophila)	1.01299	0.997224	0.9810076
8522	GAS7	growth arrest-specific 7	0.98655	0.964822	0.9753153
8525	DGKZ	diacylglycerol kinase, zeta	0.97654	0.987523	0.988645
8526	DGKE	diacylglycerol kinase, epsilon 64kDa	0.9034	0.919622	0.9912398
8527	DGKD	diacylglycerol kinase, delta 130kDa	1.00044	1.029348	0.9699112
8528	DDO	D-aspartate oxidase	1.01905	0.943592	0.9640949
8529	CYP4F2	cytochrome P450, family 4, subfamily F, polypeptide 2	0.98569	1.018374	0.9379562
8530	CST7	cystatin F (leukocystatin)	0.99766	1.119278	0.9231124
8531	CSDA	cold shock domain protein A	0.98193	0.933595	0.9980074
8532	CPZ	carboxypeptidase Z	1.01208	0.999302	1.0300731
8533	COPS3	COP9 constitutive photomorphogenic homolog subunit 3 (Arabidopsis)	0.92831	0.976089	1.0859234
8534	CHST1	carbohydrate (keratan sulfate Gal-6) sulfotransferase 1	1.01104	1.018983	1.0457145
8535	CBX4	chromobox homolog 4	0.95024	0.923318	0.9112154
8536	CAMK1	calcium/calmodulin-dependent protein kinase I	1.03227	0.924419	1.0330176

8537	BCAS1	breast carcinoma amplified sequence 1	0.98625	0.982229	1.0201884
8538	BARX2	BARX homeobox 2	1.00235	1.004923	0.9829601
8539	API5	apoptosis inhibitor 5	0.94167	0.930538	1.1049267
8540	AGPS	alkylglycerone phosphate synthase	0.98144	0.868182	1.0584411
8541	PPFIA3	protein tyrosine phosphatase, receptor type, f polypeptide (PTPRF), interacting protein (liprin), alpha 3	0.99262	0.981711	1.0424318
8542	APOL1	apolipoprotein L, 1	0.91604	0.940802	1.0349869
8543	LMO4	LIM domain only 4	1.00508	1.014597	1.0354027
8544	PIR	pirin (iron-binding nuclear protein)	0.99231	0.985776	1.0043777
8545	CGGBP1	CGG triplet repeat binding protein 1	0.97687	0.939759	1.0356248
8546	AP3B1	adaptor-related protein complex 3, beta 1 subunit	0.99244	0.94883	1.0147328
8547	FCN3	ficolin (collagen/fibrinogen domain containing) 3 (Hakata antigen)	1.00912	1.03098	0.9774623
8548	BLZF1	basic leucine zipper nuclear factor 1	0.98047	0.967381	1.0402695
8549	LGR5	leucine-rich repeat containing G protein-coupled receptor 5	1.00074	1.021898	1.0277097
8550	MAPKAPK5	mitogen-activated protein kinase-activated protein kinase 5	0.94737	0.964047	1.0108492
8553	BHLHE40	basic helix-loop-helix family, member e40	1.01942	1.113653	0.9564425
8554	PIAS1	protein inhibitor of activated STAT, 1	1.00471	0.972419	1.0430813
8555	CDC14B	CDC14 cell division cycle 14 homolog B (S. cerevisiae)	0.95777	1.002534	0.987344
8556	CDC14A	CDC14 cell division cycle 14 homolog A (S. cerevisiae)	0.99711	1.056252	0.9988712
8557	TCAP	titin-cap (telethonin)	1.01198	1.044274	0.8717461
8558	CDK10	cyclin-dependent kinase 10	1.01883	0.938922	1.0022051
8559	PRPF18	PRP18 pre-mRNA processing factor 18 homolog (S. cerevisiae)	0.95822	0.979225	1.1017494
8560	DEGS1	degenerative spermatocyte homolog 1, lipid desaturase (Drosophila)	0.98185	0.981604	1.0411788
8562	DENR	density-regulated protein	0.94177	0.951238	1.0136283
8563	THOC5	THO complex 5	0.93225	0.943522	1.0778714
8564	KMO	kynurenine 3-monooxygenase (kynurenine 3-hydroxylase)	1.01749	1.072734	0.9592031
8565	YARS	tyrosyl-tRNA synthetase	0.98313	1.03197	1.0342348
8566	PDXK	pyridoxal (pyridoxine, vitamin B6) kinase	0.97586	0.994732	1.0525599
8567	MADD	MAP-kinase activating death domain	0.96366	0.998084	0.9873093
8568	RRP1	ribosomal RNA processing 1 homolog (S. cerevisiae)	0.94195	1.000229	1.0029244
8569	MKNK1	MAP kinase interacting serine/threonine kinase 1	1.02659	0.974393	0.9882299
8570	KHSRP	KH-type splicing regulatory protein	0.9326	0.976298	1.0244376
8572	PDLIM4	PDZ and LIM domain 4	1.01323	1.020826	1.0083455
8573	CASK	calcium/calmodulin-dependent serine protein kinase (MAGUK family)	0.94029	0.914594	1.0070623

8574	AKR7A2	aldo-keto reductase family 7, member A2 (aflatoxin aldehyde reductase)	0.97059	0.960546	1.050364
8575	PRKRA	protein kinase, interferon-inducible double stranded RNA dependent activator	0.98545	0.945492	0.9256527
8576	STK16	serine/threonine kinase 16	0.95085	0.973985	1.0181833
8577	TMEFF1	transmembrane protein with EGF-like and two follistatin-like domains 1	0.9707	0.964137	0.9437763
8578	SCARF1	scavenger receptor class F, member 1	0.97876	0.976545	0.9761499
8580	OR8C1P	olfactory receptor, family 8, subfamily C, member 1 pseudogene	0.9856	0.97847	0.9359569
8581	LY6D	lymphocyte antigen 6 complex, locus D	0.9935	1.019221	1.0225151
8582	OR8B1P	olfactory receptor, family 8, subfamily B, member 1 pseudogene	0.99638	0.926511	0.960576
8583	OR7E41P	olfactory receptor, family 7, subfamily E, member 41 pseudogene	0.99976	0.987988	1.0138928
8587	OR7E2P	olfactory receptor, family 7, subfamily E, member 2 pseudogene	0.98796	0.985474	0.9814239
8588	OR7E15P	olfactory receptor, family 7, subfamily E, member 15 pseudogene	1.02016	1.026513	0.9457865
8590	OR6A2	olfactory receptor, family 6, subfamily A, member 2	1.01258	0.989485	1.0189718
8591	OR5G1P	olfactory receptor, family 5, subfamily G, member 1 pseudogene	0.97668	1.071321	0.9427822
8594	OR5D3P	olfactory receptor, family 5, subfamily D, member 3 pseudogene	0.9919	1.031064	0.9897867
8595	OR5D2P	olfactory receptor, family 5, subfamily D, member 2 pseudogene	1.02137	1.004771	1.0097486
8596	OR4A1P	olfactory receptor, family 4, subfamily A, member 1 pseudogene	1.01232	1.05195	1.018857
8600	TNFSF11	tumor necrosis factor (ligand) superfamily, member 11	0.99822	0.965171	1.0203072
8601	RGS20	regulator of G-protein signaling 20	1.00368	1.009187	1.0308607
8602	NOP14	NOP14 nucleolar protein homolog (yeast)	0.9945	1.003026	0.9505354
8603	FAM193A	family with sequence similarity 193, member A	0.97769	1.012961	0.9617589
8604	SLC25A12	solute carrier family 25 (mitochondrial carrier, Aralar), member 12	0.98985	0.897536	1.0164659
8605	PLA2G4C	phospholipase A2, group IVC (cytosolic, calcium-independent)	0.98377	1.008314	0.9880053
8607	RUVBL1	RuvB-like 1 (E. coli)	0.96959	0.94606	1.0051489
8608	RDH16	retinol dehydrogenase 16 (all-trans)	1.0232	0.975464	0.9961045
8609	KLF7	Kruppel-like factor 7 (ubiquitous)	0.99787	1.03964	0.974905
8611	PPAP2A	phosphatidic acid phosphatase type 2A	1.00416	0.965028	0.9889373
8612	PPAP2C	phosphatidic acid phosphatase type 2C	1.00786	0.987179	1.0599181
8613	PPAP2B	phosphatidic acid phosphatase type 2B	0.99939	0.995225	0.9660078
8614	STC2	stanniocalcin 2	1.00043	1.003621	1.0371527
8615	USO1	USO1 vesicle docking protein homolog (yeast)	1.00214	0.987044	0.9818713
8618	CADPS	Ca++-dependent secretion activator	1.00802	1.008553	1.0170895
8620	NPFF	neuropeptide FF-amide peptide precursor	0.9674	0.999077	1.0204477
8621	CDK13	cyclin-dependent kinase 13	0.99019	0.932848	0.9718305

8622	PDE8B	phosphodiesterase 8B	1.01469	0.979609	1.0150369
8624	PSMG1	proteasome (prosome, macropain) assembly chaperone 1	0.97979	0.96457	1.0488786
8625	RFXANK	regulatory factor X-associated ankyrin-containing protein	0.94714	0.98617	1.0484735
8626	TP63	tumor protein p63	1.01499	0.998431	1.007526
8629	JRK	jerky homolog (mouse)	0.95153	0.951851	1.019164
8630	HSD17B6	hydroxysteroid (17-beta) dehydrogenase 6 homolog (mouse)	0.98979	0.992805	0.9930184
8631	SKAP1	src kinase associated phosphoprotein 1	0.93898	0.949486	0.9733978
8632	DNAH17	dynein, axonemal, heavy chain 17	0.98823	0.994861	1.006114
8633	UNC5C	unc-5 homolog C (C. elegans)	1.00505	1.008607	0.9996658
8634	RTCD1	RNA terminal phosphate cyclase domain 1	1.00671	0.928456	1.0145358
8635	RNASET2	ribonuclease T2	0.99185	1.003955	1.0818524
8636	SSNA1	Sjogren syndrome nuclear autoantigen 1	0.97173	1.042578	1.1433465
8638	OASL	2'-5'-oligoadenylate synthetase-like	0.94263	1.045994	0.996944
8639	AOC3	amine oxidase, copper containing 3 (vascular adhesion protein 1)	0.98743	0.948566	0.9870561
8642	DCHS1	dachsous 1 (Drosophila)	0.98356	0.969891	0.9940431
8643	PTCH2	patched 2	1.00467	1.028126	0.9873953
8645	KCNK5	potassium channel, subfamily K, member 5	0.99601	1.028412	1.0133596
8646	CHRD	chordin	1.00544	0.98977	1.023968
8647	ABCB11	ATP-binding cassette, sub-family B (MDR/TAP), member 11	1.00247	1.003641	1.0061243
8648	NCOA1	nuclear receptor coactivator 1	0.99816	0.891048	0.9288185
8649	LAMTOR3	late endosomal/lysosomal adaptor, MAPK and MTOR activator 3	0.97358	0.883668	0.9658712
8650	NUMB	numb homolog (Drosophila)	0.97474	1.013644	0.9283019
8651	SOCS1	suppressor of cytokine signaling 1	0.99195	0.984844	0.9839394
8653	DDX3Y	DEAD (Asp-Glu-Ala-Asp) box polypeptide 3, Y-linked	0.99268	0.966285	0.9969644
8654	PDE5A	phosphodiesterase 5A, cGMP-specific	1.0038	0.900618	0.9774373
8655	DYNLL1	dynein, light chain, LC8-type 1	0.99866	0.993791	1.0269587
8658	TNKS	tankyrase, TRF1-interacting ankyrin-related ADP-ribose polymerase	0.97524	1.011427	0.9997668
8659	ALDH4A1	aldehyde dehydrogenase 4 family, member A1	0.99327	0.993857	1.0427124
8660	IRS2	insulin receptor substrate 2	1.00701	0.970904	0.9479591
8661	EIF3A	eukaryotic translation initiation factor 3, subunit A	0.95325	1.034434	1.1233195
8662	EIF3B	eukaryotic translation initiation factor 3, subunit B	0.99925	0.99064	1.0830499
8664	EIF3D	eukaryotic translation initiation factor 3, subunit D	0.98141	0.996258	0.9591112
8665	EIF3F	eukaryotic translation initiation factor 3, subunit F	1.00309	0.951935	0.9760065

8666	EIF3G	eukaryotic translation initiation factor 3, subunit G	0.95436	0.961519	1.1333762
8667	EIF3H	eukaryotic translation initiation factor 3, subunit H	0.97035	0.893426	1.0007492
8668	EIF3I	eukaryotic translation initiation factor 3, subunit I	0.98794	0.891816	1.1305572
8669	EIF3J	eukaryotic translation initiation factor 3, subunit J	0.94554	0.982607	0.9114506
8671	SLC4A4	solute carrier family 4, sodium bicarbonate cotransporter, member 4	1.00542	1.01228	0.989572
8672	EIF4G3	eukaryotic translation initiation factor 4 gamma, 3	1.02182	1.012307	0.999227
8673	VAMP8	vesicle-associated membrane protein 8 (endobrevin)	1.00958	0.928259	1.0348837
8674	VAMP4	vesicle-associated membrane protein 4	1.00556	1.00798	0.9574
8675	STX16	syntaxin 16	0.95923	0.987133	0.9940209
8676	STX11	syntaxin 11	0.9823	0.975296	0.9238407
8677	STX10	syntaxin 10	0.95939	0.87974	1.1049522
8678	BECN1	beclin 1, autophagy related	0.94767	0.932587	0.9618627
8681	JMJD7-PLA	JMJD7-PLA2G4B readthrough	0.97524	0.936478	0.9439552
8682	PEA15	phosphoprotein enriched in astrocytes 15	0.96582	0.923042	1.0781494
8683	SRSF9	serine/arginine-rich splicing factor 9	1.0219	0.926256	0.9637114
8685	MARCO	macrophage receptor with collagenous structure	1.01001	0.955789	1.0045804
8686	KRT41P	keratin 41 pseudogene	0.98403	0.972241	0.9927388
8687	KRT38	keratin 38	0.99688	1.035177	0.9717019
8688	KRT37	keratin 37	1.01413	1.003766	0.9945401
8689	KRT36	keratin 36	0.98853	0.963612	1.0429029
8690	JRKL	jerky homolog-like (mouse)	0.92187	1.050793	0.9997676
8692	HYAL2	hyaluronoglucosaminidase 2	1.00183	1.02314	1.0323377
8694	DGAT1	diacylglycerol O-acyltransferase 1	1.00042	1.01354	1.0759694
8697	CDC23	cell division cycle 23 homolog (S. cerevisiae)	0.93788	0.932427	1.0701414
8698	S1PR4	sphingosine-1-phosphate receptor 4	0.93843	0.94096	0.9062065
8701	DNAH11	dynein, axonemal, heavy chain 11	1.00607	0.995359	1.0140859
8702	B4GALT4	UDP-Gal:betaGlcNAc beta 1,4- galactosyltransferase, polypeptide 4	1.00099	0.964467	0.9549067
8703	B4GALT3	UDP-Gal:betaGlcNAc beta 1,4- galactosyltransferase, polypeptide 3	0.97562	0.928468	1.0621717
8704	B4GALT2	UDP-Gal:betaGlcNAc beta 1,4- galactosyltransferase, polypeptide 2	0.99481	1.042791	1.0454179
8705	B3GALT4	UDP-Gal:betaGlcNAc beta 1,3-galactosyltransferase, polypeptide 4	0.99409	0.977305	0.9470104
8706	B3GALNT1	beta-1,3-N-acetylgalactosaminyltransferase 1 (globoside blood group)	1.00952	0.997346	0.9910963
8707	B3GALT2	UDP-Gal:betaGlcNAc beta 1,3-galactosyltransferase, polypeptide 2	1.00871	1.006611	1.0032678
8708	B3GALT1	UDP-Gal:betaGlcNAc beta 1,3-galactosyltransferase, polypeptide 1	0.99848	1.025113	1.0094035

8710	SERPINB7	serpin peptidase inhibitor, clade B (ovalbumin), member 7	0.9971	1.00868	1.0071458
8711	TNK1	tyrosine kinase, non-receptor, 1	0.97855	1.017268	1.0115596
8712	PAGE1	P antigen family, member 1 (prostate associated)	1.00684	1.013122	1.0044902
8714	ABCC3	ATP-binding cassette, sub-family C (CFTR/MRP), member 3	1.01883	0.939645	0.9640447
8715	NOL4	nucleolar protein 4	0.99891	1.00149	1.0146501
8717	TRADD	TNFRSF1A-associated via death domain	0.99903	0.971743	1.1052118
8718	TNFRSF25	tumor necrosis factor receptor superfamily, member 25	0.97008	0.982049	0.9773118
8720	MBTPS1	membrane-bound transcription factor peptidase, site 1	0.94447	0.9113	0.9935889
8721	EDF1	endothelial differentiation-related factor 1	0.97612	0.992765	1.0076803
8722	CTSF	cathepsin F	0.95002	0.925311	0.9862903
8723	SNX4	sorting nexin 4	0.99035	0.924068	1.0260998
8724	SNX3	sorting nexin 3	1.00565	0.968137	1.0021405
8725	C19orf2	chromosome 19 open reading frame 2	0.90448	1.048125	1.0761613
8726	EED	embryonic ectoderm development	0.97441	0.901572	1.0437736
8727	CTNNAL1	catenin (cadherin-associated protein), alpha-like 1	0.96121	1.012141	0.9815306
8729	GBF1	golgi brefeldin A resistant guanine nucleotide exchange factor 1	0.98493	1.030718	1.0582469
8731	RNMT	RNA (guanine-7-) methyltransferase	0.94728	1.026357	1.0537845
8732	RNGTT	RNA guanylyltransferase and 5'-phosphatase	0.97055	0.899757	0.9724299
8733	GPAA1	glycosylphosphatidylinositol anchor attachment protein 1 homolog (yeast)	1.01019	0.917373	1.0997165
8735	MYH13	myosin, heavy chain 13, skeletal muscle	0.99237	0.9923	1.0097818
8736	MYOM1	myomesin 1, 185kDa	0.9779	0.977752	0.9745455
8737	RIPK1	receptor (TNFRSF)-interacting serine-threonine kinase 1	0.98266	0.946762	0.9638289
8738	CRADD	CASP2 and RIPK1 domain containing adaptor with death domain	0.99672	1.015872	0.9602996
8739	HRK	harakiri, BCL2 interacting protein (contains only BH3 domain)	1.00411	1.013441	1.0352331
8740	TNFSF14	tumor necrosis factor (ligand) superfamily, member 14	0.97885	1.061845	1.0100343
8743	TNFSF10	tumor necrosis factor (ligand) superfamily, member 10	1.00787	0.938242	1.0973483
8744	TNFSF9	tumor necrosis factor (ligand) superfamily, member 9	0.97569	0.982601	0.9935182
8745	ADAM23	ADAM metallopeptidase domain 23	0.98363	0.97495	0.9975071
8747	ADAM21	ADAM metallopeptidase domain 21	1.00443	1.023223	0.9639907
8748	ADAM20	ADAM metallopeptidase domain 20	1.01513	1.008326	0.9648863
8749	ADAM18	ADAM metallopeptidase domain 18	0.99886	0.996911	0.992205
8751	ADAM15	ADAM metallopeptidase domain 15	0.98616	1.000075	1.0076973
8754	ADAM9	ADAM metallopeptidase domain 9	1.01214	0.934325	1.0034463

8755	ADAM6	ADAM metallopeptidase domain 6 (pseudogene)	0.98204	1.004997	1.0095921
8756	ADAM7	ADAM metallopeptidase domain 7	0.99861	0.991625	0.9943567
8759	ADAM1	ADAM metallopeptidase domain 1, pseudogene	0.94527	0.978012	1.0280949
8760	CDS2	CDP-diacylglycerol synthase (phosphatidate cytidylyltransferase) 2	0.96474	1.025217	0.9460826
8761	PABPC4	poly(A) binding protein, cytoplasmic 4 (inducible form)	0.99767	0.906866	0.9810066
8763	CD164	CD164 molecule, sialomucin	1.02268	0.891343	1.0815352
8764	TNFRSF14	tumor necrosis factor receptor superfamily, member 14 (herpesvirus entry mediator)	1.00128	1.000484	0.991117
8766	RAB11A	RAB11A, member RAS oncogene family	0.93373	1.023237	1.0234569
8767	RIPK2	receptor-interacting serine-threonine kinase 2	1.00596	0.984028	0.8986622
8771	TNFRSF6B	tumor necrosis factor receptor superfamily, member 6b, decoy	0.97551	0.995927	1.0212006
8772	FADD	Fas (TNFRSF6)-associated via death domain	0.9183	0.978478	0.9912732
8773	SNAP23	synaptosomal-associated protein, 23kDa	0.96914	0.993778	1.0212426
8774	NAPG	N-ethylmaleimide-sensitive factor attachment protein, gamma	0.95617	0.938417	1.0389491
8775	NAPA	N-ethylmaleimide-sensitive factor attachment protein, alpha	0.96254	1.072554	0.9949227
8776	MTMR1	myotubularin related protein 1	0.96157	0.978177	1.0049872
8777	MPDZ	multiple PDZ domain protein	0.99628	1.001453	0.9950996
8778	SIGLEC5	sialic acid binding Ig-like lectin 5	0.9775	0.962833	0.9401924
8780	RIOK3	RIO kinase 3 (yeast)	0.99303	0.933283	0.979648
8784	TNFRSF18	tumor necrosis factor receptor superfamily, member 18	0.99394	0.952253	1.0544905
8785	MATN4	matrilin 4	0.98633	0.996976	1.0391658
8786	RGS11	regulator of G-protein signaling 11	0.97904	1.009276	1.0394592
8787	RGS9	regulator of G-protein signaling 9	1.00257	1.016469	1.0234421
8788	DLK1	delta-like 1 homolog (Drosophila)	0.98951	1.011877	1.0301144
8789	FBP2	fructose-1,6-bisphosphatase 2	0.99272	0.995879	0.994691
8790	FPGT	fucose-1-phosphate guanylyltransferase	0.99575	0.970249	1.0537849
8792	TNFRSF11A	tumor necrosis factor receptor superfamily, member 11a, NFKB activator	0.99825	0.983247	1.0032429
8793	TNFRSF10D	tumor necrosis factor receptor superfamily, member 10d, decoy with truncated death domain	0.97094	0.951248	1.0700075
8794	TNFRSF10C	tumor necrosis factor receptor superfamily, member 10c, decoy without an intracellular domain	0.95362	0.960764	0.9384885
8795	TNFRSF10B	tumor necrosis factor receptor superfamily, member 10b	0.94688	0.964314	1.0207545
8796	SCEL	sciellin	0.99524	0.999446	0.9915247
8797	TNFRSF10A	tumor necrosis factor receptor superfamily, member 10a	0.95629	0.912329	0.9483174
8798	DYRK4	dual-specificity tyrosine-(Y)-phosphorylation regulated kinase 4	0.95362	1.020154	1.0408029
8799	PEX11B	peroxisomal biogenesis factor 11 beta	0.98634	1.000344	1.1039738

8800	PEX11A	peroxisomal biogenesis factor 11 alpha	0.97277	0.978601	1.0034511
8801	SUCLG2	succinate-CoA ligase, GDP-forming, beta subunit	0.99885	0.918444	0.9679392
8802	SUCLG1	succinate-CoA ligase, alpha subunit	1.00793	0.938287	0.9511808
8803	SUCLA2	succinate-CoA ligase, ADP-forming, beta subunit	0.95464	1.024382	1.0522669
8805	TRIM24	tripartite motif containing 24	1.00224	0.985833	0.9845273
8807	IL18RAP	interleukin 18 receptor accessory protein	1.05548	0.885215	0.8415722
8808	IL1RL2	interleukin 1 receptor-like 2	0.99163	0.98228	1.0091497
8809	IL18R1	interleukin 18 receptor 1	0.99966	1.049747	1.0687694
8811	GALR2	galanin receptor 2	0.97512	0.950012	1.0043173
8812	CCNK	cyclin K	0.95755	0.977362	0.998337
8813	DPM1	dolichyl-phosphate mannosyltransferase polypeptide 1, catalytic subunit	0.94027	0.951843	0.9639593
8814	CDKL1	cyclin-dependent kinase-like 1 (CDC2-related kinase)	0.96691	0.950911	1.0469913
8815	BANF1	barrier to autointegration factor 1	0.97689	1.041563	1.0375332
8816	DCAF5	DDB1 and CUL4 associated factor 5	0.97351	0.977405	1.0147009
8817	FGF18	fibroblast growth factor 18	0.99323	1.007036	1.0107904
8818	DPM2	dolichyl-phosphate mannosyltransferase polypeptide 2, regulatory subunit	0.96605	0.954331	1.1041181
8819	SAP30	Sin3A-associated protein, 30kDa	0.99306	1.0097	1.0663193
8820	HESX1	HESX homeobox 1	0.97616	0.993262	0.9888448
8821	INPP4B	inositol polyphosphate-4-phosphatase, type II, 105kDa	0.97678	0.896399	0.9594757
8822	FGF17	fibroblast growth factor 17	1.00123	0.949579	1.025641
8823	FGF16	fibroblast growth factor 16	0.99658	1.032642	1.0224863
8824	CES2	carboxylesterase 2	0.92254	0.971952	1.0417081
8825	LIN7A	lin-7 homolog A (C. elegans)	0.96187	0.944969	1.008268
8826	IQGAP1	IQ motif containing GTPase activating protein 1	0.96491	1.02154	0.9430979
8828	NRP2	neuropilin 2	1.00438	0.99389	1.0006244
8829	NRP1	neuropilin 1	1.00699	1.002099	1.0023319
8831	SYNGAP1	synaptic Ras GTPase activating protein 1	0.98142	1.024023	1.0232616
8832	CD84	CD84 molecule	1.02768	0.988599	0.9362744
8833	GMPS	guanine monphosphate synthetase	0.99176	0.913506	1.0108273
8834	TMEM11	transmembrane protein 11	0.98271	0.994478	1.006519
8835	SOCS2	suppressor of cytokine signaling 2	0.95767	0.986744	1.0813001
8836	GGH	gamma-glutamyl hydrolase (conjugase, folylpolygammaglutamyl hydrolase)	0.99812	0.949399	1.0111262
8837	CFLAR	CASP8 and FADD-like apoptosis regulator	0.99261	1.014361	0.92985

8838	WISP3	WNT1 inducible signaling pathway protein 3	1.01414	1.006985	0.9835337
8839	WISP2	WNT1 inducible signaling pathway protein 2	0.99454	1.017519	0.9826473
8840	WISP1	WNT1 inducible signaling pathway protein 1	1.01753	1.016978	1.0190716
8841	HDAC3	histone deacetylase 3	1.01727	1.028166	1.0775245
8842	PROM1	prominin 1	1.00194	1.012694	0.9837656
8843	HCAR3	hydroxycarboxylic acid receptor 3	0.94753	1.013975	0.8757358
8844	KSR1	kinase suppressor of ras 1	1.0216	0.988888	1.0409649
8846	ALKBH1	alkB, alkylation repair homolog 1 (E. coli)	0.96234	1.014423	1.103145
8847	DLEU2	deleted in lymphocytic leukemia 2 (non-protein coding)	0.96222	1.025282	0.9167618
8848	TSC22D1	TSC22 domain family, member 1	0.95545	0.981795	0.9503303
8850	KAT2B	K(lysine) acetyltransferase 2B	1.01307	0.96423	1.0079586
8851	CDK5R1	cyclin-dependent kinase 5, regulatory subunit 1 (p35)	0.96653	0.954665	1.0155347
8852	AKAP4	A kinase (PRKA) anchor protein 4	0.99579	1.031017	0.9845081
8853	ASAP2	ArfGAP with SH3 domain, ankyrin repeat and PH domain 2	0.99252	0.957208	0.9799514
8854	ALDH1A2	aldehyde dehydrogenase 1 family, member A2	1.00428	1.015489	0.9907897
8856	NR1I2	nuclear receptor subfamily 1, group I, member 2	1.00761	1.030107	1.0406318
8857	FCGBP	Fc fragment of IgG binding protein	0.96382	0.998153	0.9876336
8858	PROZ	protein Z, vitamin K-dependent plasma glycoprotein	0.99813	1.009076	1.0342076
8859	STK19	serine/threonine kinase 19	0.99905	0.978612	1.0003806
8861	LDB1	LIM domain binding 1	0.95773	1.006671	0.9544636
8862	APLN	apelin	0.99615	0.999634	1.0067722
8863	PER3	period homolog 3 (Drosophila)	0.99967	0.960421	0.8978333
8864	PER2	period homolog 2 (Drosophila)	0.99746	0.971683	0.979705
8867	SYNJ1	synaptojanin 1	0.95239	1.023924	1.0285872
8869	ST3GAL5	ST3 beta-galactoside alpha-2,3-sialyltransferase 5	0.99387	0.962963	0.9785366
8871	SYNJ2	synaptojanin 2	0.9707	0.991298	0.9743678
8872	CDC123	cell division cycle 123 homolog (S. cerevisiae)	0.93593	0.987845	1.0980673
8874	ARHGEF7	Rho guanine nucleotide exchange factor (GEF) 7	0.97272	1.017452	1.0531456
8875	VNN2	vanin 2	0.97136	0.846522	1.0129295
8876	VNN1	vanin 1	1.04771	0.971855	0.9551667
8877	SPHK1	sphingosine kinase 1	0.95535	1.027993	0.9152844
8878	SQSTM1	sequestosome 1	1.00361	0.953713	0.9160929
8879	SGPL1	sphingosine-1-phosphate lyase 1	0.95122	0.966816	1.0512253

8880	FUBP1	far upstream element (FUSE) binding protein 1	0.99188	0.910859	1.0401033
8881	CDC16	cell division cycle 16 homolog (S. cerevisiae)	0.95939	0.989267	1.0503084
8882	ZNF259	zinc finger protein 259	0.98002	1.015179	0.9751081
8883	NAE1	NEDD8 activating enzyme E1 subunit 1	0.92008	0.941451	1.0881279
8884	SLC5A6	solute carrier family 5 (sodium-dependent vitamin transporter), member 6	0.98694	0.981456	1.0558099
8886	DDX18	DEAD (Asp-Glu-Ala-Asp) box polypeptide 18	1.02755	0.945183	0.972316
8887	TAX1BP1	Tax1 (human T-cell leukemia virus type I) binding protein 1	1.00304	0.885696	0.974193
8888	MCM3AP	minichromosome maintenance complex component 3 associated protein	0.95308	1.040438	1.0320555
8890	EIF2B4	eukaryotic translation initiation factor 2B, subunit 4 delta, 67kDa	0.98239	0.973103	1.0688589
8891	EIF2B3	eukaryotic translation initiation factor 2B, subunit 3 gamma, 58kDa	0.99899	0.976234	1.0067618
8892	EIF2B2	eukaryotic translation initiation factor 2B, subunit 2 beta, 39kDa	0.90839	0.920642	0.996483
8893	EIF2B5	eukaryotic translation initiation factor 2B, subunit 5 epsilon, 82kDa	0.97185	0.925306	1.0878914
8894	EIF2S2	eukaryotic translation initiation factor 2, subunit 2 beta, 38kDa	0.99205	0.964135	0.9644036
8895	CPNE3	copine III	0.98075	0.945404	1.0815578
8896	BUD31	BUD31 homolog (S. cerevisiae)	0.94812	0.983209	0.9724221
8897	MTMR3	myotubularin related protein 3	0.9838	1.008863	1.0024792
8898	MTMR2	myotubularin related protein 2	0.9481	0.990138	1.0153362
8899	PRPF4B	PRP4 pre-mRNA processing factor 4 homolog B (yeast)	1.04942	0.850277	0.9773979
8900	CCNA1	cyclin A1	1.00761	0.967561	0.9909269
8904	CPNE1	copine I	0.97326	0.946546	1.1220472
8905	AP1S2	adaptor-related protein complex 1, sigma 2 subunit	0.99261	1.0026	1.0041896
8906	AP1G2	adaptor-related protein complex 1, gamma 2 subunit	0.95682	0.946936	1.0121455
8907	AP1M1	adaptor-related protein complex 1, mu 1 subunit	0.96198	0.921929	1.0878933
8908	GYG2	glycogenin 2	1.01657	1.030934	1.0098004
8909	ENDOU	endonuclease, polyU-specific	1.00227	1.02758	0.972684
8910	SGCE	sarcoglycan, epsilon	1.00042	1.015046	1.0010017
8911	CACNA1I	calcium channel, voltage-dependent, T type, alpha 1I subunit	0.97292	0.940963	1.0013884
8912	CACNA1H	calcium channel, voltage-dependent, T type, alpha 1H subunit	0.98536	0.994251	1.0198866
8913	CACNA1G	calcium channel, voltage-dependent, T type, alpha 1G subunit	0.99536	0.974842	1.0278187
8914	TIMELESS	timeless homolog (Drosophila)	0.9796	0.937056	1.0248267
8915	BCL10	B-cell CLL/lymphoma 10	0.99486	0.910943	1.0231135
8916	HERC3	hect domain and RLD 3	1.00319	0.94197	0.9446357
8924	HERC2	hect domain and RLD 2	0.9889	0.941952	1.0172441

8925	HERC1	hect (homologous to the E6-AP (UBE3A) carboxyl terminus) domain and RCC1 (CHC1)-like domain (RLD) 1	0.95003	1.076857	1.0159576
8927	BSN	bassoon (presynaptic cytomatrix protein)	0.99458	1.006031	0.9975575
8928	FOXH1	forkhead box H1	1.00257	0.993412	0.9944578
8929	PHOX2B	paired-like homeobox 2b	1.00302	0.972484	1.0402183
8930	MBD4	methyl-CpG binding domain protein 4	0.99085	1.111664	0.9920616
8932	MBD2	methyl-CpG binding domain protein 2	0.96645	0.982249	1.0627266
8933	FAM127A	family with sequence similarity 127, member A	1.00269	0.988889	1.0652461
8934	RAB7L1	RAB7, member RAS oncogene family-like 1	1.00518	0.948022	0.9157822
8935	SKAP2	src kinase associated phosphoprotein 2	1.00789	0.940547	1.0463267
8936	WASF1	WAS protein family, member 1	1.00275	0.979777	1.0266008
8938	BAIAP3	BAI1-associated protein 3	1.00311	0.965352	0.9658173
8939	FUBP3	far upstream element (FUSE) binding protein 3	0.9662	1.013857	1.0575373
8940	TOP3B	topoisomerase (DNA) III beta	0.98149	0.916622	1.0976647
8941	CDK5R2	cyclin-dependent kinase 5, regulatory subunit 2 (p39)	0.99663	1.021662	0.9967638
8942	KYNU	kynureninase	1.00945	1.190241	0.9943761
8943	AP3D1	adaptor-related protein complex 3, delta 1 subunit	0.94887	0.926795	1.0419201
8945	BTRC	beta-transducin repeat containing	0.9683	0.967229	1.0337344
8968	HIST1H3F	histone cluster 1, H3f	0.94951	0.960459	1.0530061
8969	HIST1H2AG	histone cluster 1, H2ag	0.93382	0.843497	0.9977057
8970	HIST1H2BJ	histone cluster 1, H2bj	0.937	1.000068	1.0093
8971	H1FX	H1 histone family, member X	0.98954	0.999088	0.916148
8973	CHRNA6	cholinergic receptor, nicotinic, alpha 6	1.00376	1.007777	1.0094634
8974	P4HA2	prolyl 4-hydroxylase, alpha polypeptide II	1.00971	0.997599	1.0182982
8975	USP13	ubiquitin specific peptidase 13 (isopeptidase T-3)	0.98889	0.976361	0.9996165
8976	WASL	Wiskott-Aldrich syndrome-like	0.96761	0.925802	1.0610951
8985	PLOD3	procollagen-lysine, 2-oxoglutarate 5-dioxygenase 3	1.0065	0.912008	1.1049907
8986	RPS6KA4	ribosomal protein S6 kinase, 90kDa, polypeptide 4	0.965	0.904539	1.0882925
8987	STBD1	starch binding domain 1	0.99413	1.023578	1.0144218
8988	HSPB3	heat shock 27kDa protein 3	0.99121	0.921216	0.93887
8989	TRPA1	transient receptor potential cation channel, subfamily A, member 1	0.99484	1.017512	1.0214774
8991	SELENBP1	selenium binding protein 1	1.02385	0.957368	1.0347418
8992	ATP6V0E1	ATPase, H+ transporting, lysosomal 9kDa, V0 subunit e1	1.01943	0.99328	0.9853249
8993	PGLYRP1	peptidoglycan recognition protein 1	0.97878	0.993735	0.9798821

8994	LIMD1	LIM domains containing 1	1.03714	1.009404	1.0404239
8995	TNFSF18	tumor necrosis factor (ligand) superfamily, member 18	0.99455	0.97624	0.988464
8996	NOL3	nucleolar protein 3 (apoptosis repressor with CARD domain)	0.97805	1.052279	1.0626631
8997	KALRN	kalirin, RhoGEF kinase	1.0053	0.986349	1.0022665
8999	CDKL2	cyclin-dependent kinase-like 2 (CDC2-related kinase)	0.99838	1.007542	1.0213554
9001	HAP1	huntingtin-associated protein 1	1.00102	1.007128	0.9977427
9002	F2RL3	coagulation factor II (thrombin) receptor-like 3	0.9666	0.98624	1.0724402
9013	TAF1C	TATA box binding protein (TBP)-associated factor, RNA polymerase I, C, 110kDa	0.95761	0.97964	0.9730975
9014	TAF1B	TATA box binding protein (TBP)-associated factor, RNA polymerase I, B, 63kDa	0.99179	0.961909	1.0420182
9015	TAF1A	TATA box binding protein (TBP)-associated factor, RNA polymerase I, A, 48kDa	0.99554	1.031262	1.0682147
9016	SLC25A14	solute carrier family 25 (mitochondrial carrier, brain), member 14	0.96758	0.990569	1.1275483
9019	MPZL1	myelin protein zero-like 1	1.00346	0.983614	1.0292781
9020	MAP3K14	mitogen-activated protein kinase kinase kinase 14	0.96699	0.951754	0.9646932
9021	SOCS3	suppressor of cytokine signaling 3	0.98422	0.950543	0.9259135
9022	CLIC3	chloride intracellular channel 3	0.96774	0.992097	0.9733874
9023	CH25H	cholesterol 25-hydroxylase	1.00647	0.948858	1.0196438
9024	BRSK2	BR serine/threonine kinase 2	0.98702	1.000265	1.030383
9025	RNF8	ring finger protein 8	0.99841	0.986592	1.0253881
9026	HIP1R	huntingtin interacting protein 1 related	0.94735	1.068467	1.0010772
9027	NAT8	N-acetyltransferase 8 (GCN5-related, putative)	1.01558	0.974555	1.0085784
9028	RHBDL1	rhomboid, veinlet-like 1 (Drosophila)	1.01021	0.983505	0.9717871
9031	BAZ1B	bromodomain adjacent to zinc finger domain, 1B	0.97642	1.010283	1.0832148
9032	TM4SF5	transmembrane 4 L six family member 5	1.00305	0.9934	1.0094805
9033	PKD2L1	polycystic kidney disease 2-like 1	1.01195	0.98306	0.998874
9034	CCRL2	chemokine (C-C motif) receptor-like 2	1.01517	1.110119	0.9217599
9037	SEMA5A	sema domain, seven thrombospondin repeats (type 1 and type 1-like), transmembrane domain (TM) and sho	1.01315	0.997923	1.0133398
9038	TAAR5	trace amine associated receptor 5	1.03075	0.97443	0.9998342
9039	UBA3	ubiquitin-like modifier activating enzyme 3	1.03608	0.921489	1.1177577
9040	UBE2M	ubiquitin-conjugating enzyme E2M	0.97949	0.979853	1.0336411
9043	SPAG9	sperm associated antigen 9	0.93027	1.049366	0.9847757
9044	BTAF1	BTAF1 RNA polymerase II, B-TFIID transcription factor-associated, 170kDa (Mot1 homolog, S. cerevisiae)	0.95671	1.07007	0.9792852
9045	RPL14	ribosomal protein L14	0.99243	0.969382	0.9354885
9046	DOK2	docking protein 2, 56kDa	0.97353	0.911642	0.9534122

9047	SH2D2A	SH2 domain containing 2A	1.01542	1.097916	0.9578279
9048	ARTN	artemin	1.01238	0.982067	1.0374428
9049	AIP	aryl hydrocarbon receptor interacting protein	0.93685	0.989833	1.1611547
9050	PSTPIP2	proline-serine-threonine phosphatase interacting protein 2	0.94271	1.024018	1.0034162
9051	PSTPIP1	proline-serine-threonine phosphatase interacting protein 1	0.95666	1.037873	1.0003651
9052	GPRC5A	G protein-coupled receptor, family C, group 5, member A	0.99168	1.015252	1.0056158
9053	MAP7	microtubule-associated protein 7	1.00147	0.994317	0.9968239
9054	NFS1	NFS1 nitrogen fixation 1 homolog (S. cerevisiae)	0.95698	0.951607	1.0548783
9055	PRC1	protein regulator of cytokinesis 1	0.96428	0.922872	1.0165891
9056	SLC7A7	solute carrier family 7 (cationic amino acid transporter, y+ system), member 7	1.00744	0.877644	1.0321967
9057	SLC7A6	solute carrier family 7 (cationic amino acid transporter, y+ system), member 6	0.95588	0.950199	1.0037429
9058	SLC13A2	solute carrier family 13 (sodium-dependent dicarboxylate transporter), member 2	1.01287	0.992637	1.0257839
9060	PAPSS2	3'-phosphoadenosine 5'-phosphosulfate synthase 2	0.98567	1.019036	0.9830165
9061	PAPSS1	3'-phosphoadenosine 5'-phosphosulfate synthase 1	1.01365	0.988725	1.1535393
9063	PIAS2	protein inhibitor of activated STAT, 2	0.92221	0.966014	0.98801
9064	MAP3K6	mitogen-activated protein kinase kinase kinase 6	1.0052	1.011203	1.0599294
9066	SYT7	synaptotagmin VII	0.99294	1.019703	1.0384213
9068	ANGPTL1	angiopoietin-like 1	1.00561	1.015239	1.003516
9069	CLDN12	claudin 12	1.00076	0.99815	0.9774934
9070	ASH2L	ash2 (absent, small, or homeotic)-like (Drosophila)	0.9992	0.941239	1.0125593
9071	CLDN10	claudin 10	0.99098	1.022924	1.0121549
9073	CLDN8	claudin 8	1.00645	0.979271	1.0242535
9075	CLDN2	claudin 2	1.00526	0.991699	0.9833538
9076	CLDN1	claudin 1	1.00273	1.005401	1.0018909
9077	DIRAS3	DIRAS family, GTP-binding RAS-like 3	0.9798	0.990797	1.0170206
9079	LDB2	LIM domain binding 2	1.00823	0.995612	0.9998578
9080	CLDN9	claudin 9	0.99152	0.954466	1.0238336
9086	EIF1AY	eukaryotic translation initiation factor 1A, Y-linked	0.98341	0.967041	1.0429807
9087	TMSB4Y	thymosin beta 4, Y-linked	0.96832	0.964389	1.0621396
9088	PKMYT1	protein kinase, membrane associated tyrosine/threonine 1	0.98962	1.035215	1.0461104
9091	PIGQ	phosphatidylinositol glycan anchor biosynthesis, class Q	0.9744	0.991352	1.0783815
9092	SART1	squamous cell carcinoma antigen recognized by T cells	0.97205	0.983648	1.002397
9093	DNAJA3	DnaJ (Hsp40) homolog, subfamily A, member 3	0.94319	0.938382	0.9605592

9094	UNC119	unc-119 homolog (C. elegans)	0.96133	1.05264	1.0081657
9095	TBX19	T-box 19	0.99974	1.012116	0.9943934
9096	TBX18	T-box 18	0.99039	0.979677	1.0069616
9097	USP14	ubiquitin specific peptidase 14 (tRNA-guanine transglycosylase)	0.95337	0.939012	0.9981642
9098	USP6	ubiquitin specific peptidase 6 (Tre-2 oncogene)	1.00449	1.019015	1.0201546
9099	USP2	ubiquitin specific peptidase 2	0.99085	1.001193	0.9805766
9100	USP10	ubiquitin specific peptidase 10	0.99411	0.942376	0.9784819
9101	USP8	ubiquitin specific peptidase 8	0.93518	1.065954	0.9918009
9104	RGN	regucalcin (senescence marker protein-30)	0.99974	1.00693	1.0133002
9107	MTMR6	myotubularin related protein 6	0.93043	0.957289	0.9973502
9108	MTMR7	myotubularin related protein 7	1.00633	1.020135	1.0151382
9110	MTMR4	myotubularin related protein 4	0.95004	0.944118	1.012386
9111	NMI	N-myc (and STAT) interactor	1.03483	0.950577	1.0012653
9112	MTA1	metastasis associated 1	0.96163	1.003796	1.0426685
9113	LATS1	LATS, large tumor suppressor, homolog 1 (Drosophila)	0.99327	0.949766	1.0291318
9114	ATP6V0D1	ATPase, H+ transporting, lysosomal 38kDa, V0 subunit d1	0.97481	1.020663	0.9959516
9117	SEC22C	SEC22 vesicle trafficking protein homolog C (S. cerevisiae)	0.97338	0.947014	1.0496518
9118	INA	internexin neuronal intermediate filament protein, alpha	0.98854	1.015498	1.0265052
9119	KRT75	keratin 75	0.9967	1.013027	1.0186723
9120	SLC16A6	solute carrier family 16, member 6 (monocarboxylic acid transporter 7)	0.98968	1.032227	0.8468508
9121	SLC16A5	solute carrier family 16, member 5 (monocarboxylic acid transporter 6)	0.97369	0.954317	0.9991472
9122	SLC16A4	solute carrier family 16, member 4 (monocarboxylic acid transporter 5)	1.00093	1.029438	1.0375843
9123	SLC16A3	solute carrier family 16, member 3 (monocarboxylic acid transporter 4)	1.01385	1.083798	0.9659548
9124	PDLIM1	PDZ and LIM domain 1	0.98681	0.998933	1.0159128
9125	RQCD1	RCD1 required for cell differentiation1 homolog (S. pombe)	1.01459	1.037646	1.0036974
9126	SMC3	structural maintenance of chromosomes 3	0.93298	0.901395	0.9803378
9127	P2RX6	purinergic receptor P2X, ligand-gated ion channel, 6	0.98589	0.987009	1.0213518
9128	PRPF4	PRP4 pre-mRNA processing factor 4 homolog (yeast)	0.91555	0.987758	0.9844784
9129	PRPF3	PRP3 pre-mRNA processing factor 3 homolog (S. cerevisiae)	0.99754	1.014082	0.936728
9130	FAM50A	family with sequence similarity 50, member A	0.97396	1.071118	1.0337323
9131	AIFM1	apoptosis-inducing factor, mitochondrion-associated, 1	0.94785	1.005938	1.0638482
9132	KCNQ4	potassium voltage-gated channel, KQT-like subfamily, member 4	0.98623	1.02639	1.0545761
9133	CCNB2	cyclin B2	1.00938	1.001169	1.0078327

9134	CCNE2	cyclin E2	0.99317	0.981658	1.0273751
9135	RABEP1	rabaptin, RAB GTPase binding effector protein 1	0.9531	0.996369	1.0225506
9136	RRP9	ribosomal RNA processing 9, small subunit (SSU) processome component, homolog (yeast)	0.9754	0.982157	1.0386005
9138	ARHGEF1	Rho guanine nucleotide exchange factor (GEF) 1	0.9855	1.011006	0.9752597
9139	CBFA2T2	core-binding factor, runt domain, alpha subunit 2; translocated to, 2	0.95593	1.022322	0.9776749
9140	ATG12	ATG12 autophagy related 12 homolog (S. cerevisiae)	1.02408	0.997022	0.97505
9141	PDCD5	programmed cell death 5	0.97405	1.002657	1.0421684
9142	CXorf1	chromosome X open reading frame 1	0.99181	0.971961	1.0647852
9143	SYNGR3	synaptogyrin 3	0.93714	1.03901	1.1148432
9144	SYNGR2	synaptogyrin 2	0.97299	1.052338	1.007488
9145	SYNGR1	synaptogyrin 1	1.00436	0.990986	1.0075307
9146	HGS	hepatocyte growth factor-regulated tyrosine kinase substrate	0.96358	1.050326	1.0568707
9147	NEMF	nuclear export mediator factor	0.93488	0.989256	0.9079241
9148	NEURL	neuralized homolog (Drosophila)	1.00247	0.998717	1.0274241
9149	DYRK1B	dual-specificity tyrosine-(Y)-phosphorylation regulated kinase 1B	0.96934	0.959614	1.088949
9150	CTDP1	CTD (carboxy-terminal domain, RNA polymerase II, polypeptide A) phosphatase, subunit 1	0.97534	1.067889	0.9806862
9152	SLC6A5	solute carrier family 6 (neurotransmitter transporter, glycine), member 5	0.99222	1.026785	1.003516
9153	SLC28A2	solute carrier family 28 (sodium-coupled nucleoside transporter), member 2	1.01553	1.01983	0.9759993
9154	SLC28A1	solute carrier family 28 (sodium-coupled nucleoside transporter), member 1	1.008	1.007226	0.9875954
9156	EXO1	exonuclease 1	1.01244	0.944995	1.0092979
9158	FIBP	fibroblast growth factor (acidic) intracellular binding protein	0.95901	0.903346	1.068926
9159	PCSK7	proprotein convertase subtilisin/kexin type 7	0.949	0.998765	0.9708288
9162	DGKI	diacylglycerol kinase, iota	1.01026	1.007839	1.0117682
9166	EBAG9	estrogen receptor binding site associated, antigen, 9	0.96724	0.919745	1.0693971
9167	COX7A2L	cytochrome c oxidase subunit VIIa polypeptide 2 like	0.97812	0.940333	0.9910091
9168	TMSB10	thymosin beta 10	0.96596	0.961857	1.1041627
9169	SCAF11	SR-related CTD-associated factor 11	0.94497	0.998225	1.0213113
9170	LPAR2	lysophosphatidic acid receptor 2	1.0051	1.021471	1.0026772
9172	MYOM2	myomesin (M-protein) 2, 165kDa	1.01653	1.026093	0.9423588
9173	IL1RL1	interleukin 1 receptor-like 1	1.01487	0.990833	0.9926052
9175	MAP3K13	mitogen-activated protein kinase kinase kinase 13	1.01162	1.003208	1.0089234
9177	HTR3B	5-hydroxytryptamine (serotonin) receptor 3B	0.99984	0.99316	0.9702414
9179	AP4M1	adaptor-related protein complex 4, mu 1 subunit	0.98444	1.011583	1.0564671

9180	OSMR	oncostatin M receptor	1.00795	1.029915	1.0007254
9181	ARHGEF2	Rho/Rac guanine nucleotide exchange factor (GEF) 2	1.00034	1.030003	0.9539119
9182	RASSF9	Ras association (RalGDS/AF-6) domain family (N-terminal) member 9	1.01857	0.982066	0.9820285
9183	ZW10	ZW10, kinetochore associated, homolog (Drosophila)	0.91766	0.954444	1.029391
9184	BUB3	budding uninhibited by benzimidazoles 3 homolog (yeast)	0.95024	1.006524	1.0839121
9187	SLC24A1	solute carrier family 24 (sodium/potassium/calcium exchanger), member 1	1.00313	1.02942	0.9987822
9188	DDX21	DEAD (Asp-Glu-Ala-Asp) box polypeptide 21	0.93069	1.015231	1.0709228
9191	DEDD	death effector domain containing	0.96185	0.965961	1.1057584
9194	SLC16A7	solute carrier family 16, member 7 (monocarboxylic acid transporter 2)	0.94694	0.937284	0.9863616
9196	KCNAB3	potassium voltage-gated channel, shaker-related subfamily, beta member 3	0.97633	1.047075	0.9692922
9197	SLC33A1	solute carrier family 33 (acetyl-CoA transporter), member 1	1.01053	0.931006	1.0963303
9200	PTPLA	protein tyrosine phosphatase-like (proline instead of catalytic arginine), member A	0.97755	0.971282	1.0094312
9201	DCLK1	doublecortin-like kinase 1	0.99897	0.995094	1.0160499
9202	ZMYM4	zinc finger, MYM-type 4	0.97823	0.989546	0.9919708
9203	ZMYM3	zinc finger, MYM-type 3	0.94116	0.950048	1.0279993
9204	ZMYM6	zinc finger, MYM-type 6	1.01605	0.994251	1.0895726
9205	ZMYM5	zinc finger, MYM-type 5	0.95872	1.000731	0.9716018
9206	RAD17P2	RAD17 homolog (S. pombe) pseudogene 2	1.00318	1.024362	1.0159152
9208	LRRFIP1	leucine rich repeat (in FLII) interacting protein 1	1.00203	1.017834	0.9428297
9209	LRRFIP2	leucine rich repeat (in FLII) interacting protein 2	0.98842	1.004536	0.9993047
9210	BMP15	bone morphogenetic protein 15	1.0041	1.033163	0.9717795
9211	LGI1	leucine-rich, glioma inactivated 1	1.00881	1.023223	0.9946991
9212	AURKB	aurora kinase B	0.98661	0.992681	1.0141635
9213	XPR1	xenotropic and polytropic retrovirus receptor 1	1.00538	1.000856	0.9412475
9214	FAIM3	Fas apoptotic inhibitory molecule 3	0.93487	0.886515	0.9141645
9215	LARGE	like-glycosyltransferase	0.99287	0.976648	1.0118064
9217	VAPB	VAMP (vesicle-associated membrane protein)-associated protein B and C	0.91336	1.020347	1.0336516
9218	VAPA	VAMP (vesicle-associated membrane protein)-associated protein A, 33kDa	0.98425	0.929245	0.9418656
9219	MTA2	metastasis associated 1 family, member 2	0.95261	0.925519	1.1095355
9221	NOLC1	nucleolar and coiled-body phosphoprotein 1	0.89799	0.976333	0.9773889
9223	MAGI1	membrane associated guanylate kinase, WW and PDZ domain containing 1	1.0104	1.013958	1.0116857
9227	LRAT	lecithin retinol acyltransferase (phosphatidylcholine--retinol O-acyltransferase)	0.99753	1.018775	1.0019471
9228	DLGAP2	discs, large (Drosophila) homolog-associated protein 2	0.99097	0.995008	1.0180552

9229	DLGAP1	discs, large (Drosophila) homolog-associated protein 1	0.99735	0.994216	1.007097
9230	RAB11B	RAB11B, member RAS oncogene family	0.96444	0.976717	1.0753216
9231	DLG5	discs, large homolog 5 (Drosophila)	1.00518	1.016043	0.989251
9232	PTTG1	pituitary tumor-transforming 1	0.96779	0.973058	0.9923087
9235	IL32	interleukin 32	0.91157	1.005652	1.0365079
9236	CCPG1	cell cycle progression 1	0.9305	0.958871	0.9972089
9238	TBRG4	transforming growth factor beta regulator 4	0.98414	0.974726	0.9821499
9240	PNMA1	paraneoplastic antigen MA1	0.97499	1.032164	0.9243937
9241	NOG	noggin	0.79621	0.896253	0.8384886
9242	MSC	musculin	0.9783	1.118213	1.0029939
9244	CRLF1	cytokine receptor-like factor 1	0.9836	0.964	1.0418549
9245	GCNT3	glucosaminyl (N-acetyl) transferase 3, mucin type	1.01632	1.012469	1.0166215
9246	UBE2L6	ubiquitin-conjugating enzyme E2L 6	0.95437	0.989168	1.0569544
9247	GCM2	glial cells missing homolog 2 (Drosophila)	1.02116	0.989215	1.0278252
9248	GPR50	G protein-coupled receptor 50	1.01134	1.014868	0.9958487
9249	DHRS3	dehydrogenase/reductase (SDR family) member 3	0.9834	0.976613	0.9678534
9252	RPS6KA5	ribosomal protein S6 kinase, 90kDa, polypeptide 5	0.91695	0.945995	0.960444
9253	NUMBL	numb homolog (Drosophila)-like	0.99735	1.031463	1.0353826
9254	CACNA2D2	calcium channel, voltage-dependent, alpha 2/delta subunit 2	1.01022	1.041818	0.9604835
9255	AIMP1	aminoacyl tRNA synthetase complex-interacting multifunctional protein 1	1.01764	0.878957	1.0434654
9256	BZRAP1	benzodiazapine receptor (peripheral) associated protein 1	1.00797	1.045038	0.9948386
9258	MFHAS1	malignant fibrous histiocytoma amplified sequence 1	0.98297	0.977018	1.0544118
9260	PDLIM7	PDZ and LIM domain 7 (enigma)	0.99968	0.969851	0.9822107
9261	MAPKAPK2	mitogen-activated protein kinase-activated protein kinase 2	1.02797	1.037092	0.9598742
9262	STK17B	serine/threonine kinase 17b	1.02929	0.895972	0.9928058
9263	STK17A	serine/threonine kinase 17a	0.95791	0.999562	0.9140856
9265	CYTH3	cytohesin 3	1.00341	1.005146	1.0169184
9266	CYTH2	cytohesin 2	0.92456	1.04596	1.0924922
9267	CYTH1	cytohesin 1	0.95552	1.038233	0.9665727
9270	ITGB1BP1	integrin beta 1 binding protein 1	0.99229	0.941309	1.0460767
9271	PIWIL1	piwi-like 1 (Drosophila)	1.00528	1.005742	1.0084738
9274	BCL7C	B-cell CLL/lymphoma 7C	0.96449	0.975234	1.0294412
9275	BCL7B	B-cell CLL/lymphoma 7B	0.97679	0.911727	1.0095827

9276	COPB2	coatomer protein complex, subunit beta 2 (beta prime)	1.00402	0.974039	1.0187339
9277	WDR46	WD repeat domain 46	0.99598	1.005534	1.0656632
9278	ZBTB22	zinc finger and BTB domain containing 22	0.98709	0.967382	1.0175979
9282	MED14	mediator complex subunit 14	0.9688	1.067713	1.0834567
9283	GPR37L1	G protein-coupled receptor 37 like 1	1.00422	0.983338	1.0092703
9288	TAAR3	trace amine associated receptor 3 (gene/pseudogene)	0.98737	1.04916	0.9724165
9289	GPR56	G protein-coupled receptor 56	0.99573	0.96885	0.9796802
9290	GPR55	G protein-coupled receptor 55	1.0102	0.958175	1.0233913
9292	GPR53P	G protein-coupled receptor 53, pseudogene	1.03322	0.970676	0.9654028
9294	S1PR2	sphingosine-1-phosphate receptor 2	0.96729	1.008128	1.0484381
9295	SRSF11	serine/arginine-rich splicing factor 11	1.0365	0.913971	0.9150556
9296	ATP6V1F	ATPase, H+ transporting, lysosomal 14kDa, V1 subunit F	1.00891	0.998692	0.9922486
9306	SOCS6	suppressor of cytokine signaling 6	0.95427	0.99153	1.0140774
9308	CD83	CD83 molecule	1.02785	1.045135	0.9372698
9310	ZNF235	zinc finger protein 235	0.92813	0.996622	1.0111558
9311	ACCN3	amiloride-sensitive cation channel 3	1.0146	1.001061	0.9812966
9312	KCNB2	potassium voltage-gated channel, Shab-related subfamily, member 2	1.01082	1.002667	1.0115445
9313	MMP20	matrix metallopeptidase 20	1.00046	0.997966	1.0089224
9314	KLF4	Kruppel-like factor 4 (gut)	0.98274	0.894584	1.0118906
9315	C5orf13	chromosome 5 open reading frame 13	1.00569	1.009789	1.0270175
9317	PTER	phosphotriesterase related	0.96837	0.968232	1.0059413
9318	COPS2	COP9 constitutive photomorphogenic homolog subunit 2 (Arabidopsis)	0.93213	0.880659	1.1299055
9319	TRIP13	thyroid hormone receptor interactor 13	0.99037	0.984649	1.0260127
9320	TRIP12	thyroid hormone receptor interactor 12	1.02779	1.00761	1.0519382
9321	TRIP11	thyroid hormone receptor interactor 11	0.91649	1.032414	1.0068227
9322	TRIP10	thyroid hormone receptor interactor 10	0.96303	1.027668	1.0967512
9324	HMGN3	high mobility group nucleosomal binding domain 3	0.99354	0.988916	1.0498927
9325	TRIP4	thyroid hormone receptor interactor 4	0.97977	1.006168	1.0514402
9326	ZNHIT3	zinc finger, HIT-type containing 3	0.91631	0.923759	1.0441023
9328	GTF3C5	general transcription factor IIIC, polypeptide 5, 63kDa	0.94291	0.961053	1.046647
9329	GTF3C4	general transcription factor IIIC, polypeptide 4, 90kDa	0.91817	0.932442	1.0932231
9330	GTF3C3	general transcription factor IIIC, polypeptide 3, 102kDa	0.97198	0.911195	1.0607669
9331	B4GALT6	UDP-Gal:betaGlcNAc beta 1,4- galactosyltransferase, polypeptide 6	0.98148	1.000623	1.0274156

9332	CD163	CD163 molecule	0.94594	0.88021	1.0211071
9333	TGM5	transglutaminase 5	1.0056	1.008702	0.9994854
9334	B4GALT5	UDP-Gal:betaGlcNAc beta 1,4- galactosyltransferase, polypeptide 5	1.00203	1.119873	1.0502729
9337	CNOT8	CCR4-NOT transcription complex, subunit 8	1.01219	0.953122	0.9660716
9338	TCEAL1	transcription elongation factor A (SII)-like 1	0.93947	1.021214	0.9657067
9340	GLP2R	glucagon-like peptide 2 receptor	1.0217	1.016647	0.9941824
9341	VAMP3	vesicle-associated membrane protein 3 (cellubrevin)	1.0024	0.948256	0.9569201
9342	SNAP29	synaptosomal-associated protein, 29kDa	0.95246	0.939587	0.9776251
9343	EFTUD2	elongation factor Tu GTP binding domain containing 2	0.93917	0.930267	1.1334721
9344	TAOK2	TAO kinase 2	0.96001	0.969686	1.0566327
9348	NDST3	N-deacetylase/N-sulfotransferase (heparan glucosaminyl) 3	1.00652	0.993938	1.0053211
9349	RPL23	ribosomal protein L23	0.95585	0.94885	1.013002
9350	CER1	cerberus 1, cysteine knot superfamily, homolog (Xenopus laevis)	0.98449	0.995041	0.9841871
9351	SLC9A3R2	solute carrier family 9 (sodium/hydrogen exchanger), member 3 regulator 2	1.00097	1.00989	1.0162801
9352	TXNL1	thioredoxin-like 1	0.97628	0.955366	1.0582765
9353	SLIT2	slit homolog 2 (Drosophila)	0.99963	1.003671	1.008906
9354	UBE4A	ubiquitination factor E4A	0.96958	0.915395	1.0427674
9355	LHX2	LIM homeobox 2	0.98533	0.975002	1.0291455
9356	SLC22A6	solute carrier family 22 (organic anion transporter), member 6	1.0028	1.014937	1.02005
9358	ITGBL1	integrin, beta-like 1 (with EGF-like repeat domains)	0.99856	0.999097	1.0014115
9360	PPIG	peptidylprolyl isomerase G (cyclophilin G)	0.97513	0.930496	0.8988748
9361	LONP1	lon peptidase 1, mitochondrial	0.95768	1.041628	0.9587302
9362	CPNE6	copine VI (neuronal)	0.99863	0.989213	1.0166378
9363	RAB33A	RAB33A, member RAS oncogene family	0.97853	1.002837	1.0045047
9364	RAB28	RAB28, member RAS oncogene family	0.98587	0.989506	1.0815224
9365	KL	klotho	1.00249	1.034829	1.0261269
9366	RAB9BP1	RAB9B, member RAS oncogene family pseudogene 1	0.97854	0.95837	1.0182693
9367	RAB9A	RAB9A, member RAS oncogene family	0.99021	0.983183	0.9892519
9368	SLC9A3R1	solute carrier family 9 (sodium/hydrogen exchanger), member 3 regulator 1	0.9494	1.025485	0.9887754
9369	NRXN3	neurexin 3	1.00296	1.004164	1.0027161
9370	ADIPOQ	adiponectin, C1Q and collagen domain containing	1.02261	0.993242	1.0041949
9371	KIF3B	kinesin family member 3B	0.95366	1.018566	1.0643191
9373	PLAA	phospholipase A2-activating protein	0.94233	0.894132	1.0785436

9374	PPT2	palmitoyl-protein thioesterase 2	0.96931	0.986592	1.0162718
9375	TM9SF2	transmembrane 9 superfamily member 2	0.97503	0.973028	0.9991524
9376	SLC22A8	solute carrier family 22 (organic anion transporter), member 8	1.00511	0.984242	1.0212766
9377	COX5A	cytochrome c oxidase subunit Va	0.95994	0.974245	1.1048282
9378	NRXN1	neurexin 1	1.00438	1.008549	1.0203297
9379	NRXN2	neurexin 2	0.98947	1.008257	1.0455059
9380	GRHPR	glyoxylate reductase/hydroxypyruvate reductase	0.92219	0.983766	1.0230206
9381	OTOF	otoferlin	1.00104	0.997819	0.9957631
9382	COG1	component of oligomeric golgi complex 1	0.93968	1.018347	0.98088
9388	LIPG	lipase, endothelial	1.01175	1.027108	1.0298585
9389	SLC22A14	solute carrier family 22, member 14	1.00192	1.00458	0.9892615
9390	SLC22A13	solute carrier family 22 (organic anion transporter), member 13	1.02102	1.010119	1.0207268
9391	CIAO1	cytosolic iron-sulfur protein assembly 1	0.97537	1.038438	1.0568161
9392	TGFBRAP1	transforming growth factor, beta receptor associated protein 1	0.98722	0.95615	1.0523194
9394	HS6ST1	heparan sulfate 6-O-sulfotransferase 1	1.01209	0.997816	1.0005955
9397	NMT2	N-myristoyltransferase 2	0.9152	0.980988	1.027086
9398	CD101	CD101 molecule	1.04211	0.893144	1.1350474
9399	STOML1	stomatin (EPB72)-like 1	0.97451	0.946189	1.0109555
9400	RECQL5	RecQ protein-like 5	0.98711	0.973805	0.9793713
9401	RECQL4	RecQ protein-like 4	1.00996	0.980662	1.0367761
9402	GRAP2	GRB2-related adaptor protein 2	0.96244	1.111151	0.977351
9403	15-Sep	15 kDa selenoprotein	1.00879	0.972613	1.0645874
9404	LPXN	leupaxin	0.95776	1.030006	1.0451697
9406	ZRANB2	zinc finger, RAN-binding domain containing 2	1.01406	0.768593	1.0343186
9407	TMPRSS11I	transmembrane protease, serine 11D	0.98655	1.011423	0.9864713
9409	PEX16	peroxisomal biogenesis factor 16	0.98395	0.964984	1.0348669
9410	SNRNP40	small nuclear ribonucleoprotein 40kDa (U5)	1.00868	0.934221	0.9973067
9411	ARHGAP29	Rho GTPase activating protein 29	1.00314	1.011068	1.0445142
9412	MED21	mediator complex subunit 21	0.92587	0.921358	1.0280723
9413	FAM189A2	family with sequence similarity 189, member A2	1.00551	1.003645	1.0095745
9414	TJP2	tight junction protein 2 (zona occludens 2)	0.99556	0.884765	1.0100212
9415	FADS2	fatty acid desaturase 2	0.93205	1.064363	1.1035294
9416	DDX23	DEAD (Asp-Glu-Ala-Asp) box polypeptide 23	0.91106	1.02881	1.0310064

9419	CRIPT	cysteine-rich PDZ-binding protein	0.96717	0.961446	0.9501751
9420	CYP7B1	cytochrome P450, family 7, subfamily B, polypeptide 1	1.00607	1.009064	1.0161251
9421	HAND1	heart and neural crest derivatives expressed 1	1.00631	1.010583	1.0783543
9422	ZNF264	zinc finger protein 264	0.92514	1.089616	1.0978385
9423	NTN1	netrin 1	1.00354	1.015473	1.0181205
9425	CDYL	chromodomain protein, Y-like	0.99443	0.99771	1.0300587
9427	ECEL1	endothelin converting enzyme-like 1	1.00327	1.006491	1.0385054
9429	ABCG2	ATP-binding cassette, sub-family G (WHITE), member 2	1.01184	0.990114	1.002301
9431	OR7E22P	olfactory receptor, family 7, subfamily E, member 22 pseudogene	0.99346	1.017294	0.990137
9435	CHST2	carbohydrate (N-acetylglucosamine-6-O) sulfotransferase 2	0.95363	0.911464	1.0114483
9436	NCR2	natural cytotoxicity triggering receptor 2	1.02507	1.01079	1.0001739
9437	NCR1	natural cytotoxicity triggering receptor 1	0.9367	0.956851	0.9352574
9439	MED23	mediator complex subunit 23	0.98622	0.883818	1.0097825
9440	MED17	mediator complex subunit 17	0.93967	0.975186	1.1053985
9441	MED26	mediator complex subunit 26	0.99935	0.993509	0.99649
9442	MED27	mediator complex subunit 27	0.99392	0.983569	1.0031575
9443	MED7	mediator complex subunit 7	1.00383	0.969932	0.9850452
9444	QKI	quaking homolog, KH domain RNA binding (mouse)	1.06108	0.951822	0.9889177
9445	ITM2B	integral membrane protein 2B	0.98589	0.959549	0.9918551
9446	GSTO1	glutathione S-transferase omega 1	0.95017	1.000819	0.9820706
9447	AIM2	absent in melanoma 2	0.99442	0.975966	1.0414481
9448	MAP4K4	mitogen-activated protein kinase kinase kinase kinase 4	0.98515	0.991602	0.9668824
9450	LY86	lymphocyte antigen 86	0.97937	0.900383	1.0622937
9451	EIF2AK3	eukaryotic translation initiation factor 2-alpha kinase 3	1.00624	0.953525	0.9527625
9452	ITM2A	integral membrane protein 2A	0.87309	1.000351	0.9148278
9453	GGPS1	geranylgeranyl diphosphate synthase 1	0.96294	0.999197	0.9922566
9454	HOMER3	homer homolog 3 (Drosophila)	0.98825	1.006784	0.9966273
9456	HOMER1	homer homolog 1 (Drosophila)	0.99429	1.042128	1.0125075
9457	FHL5	four and a half LIM domains 5	0.98906	1.019504	0.9871513
9459	ARHGEF6	Rac/Cdc42 guanine nucleotide exchange factor (GEF) 6	0.96038	0.956257	1.0345787
9462	RASAL2	RAS protein activator like 2	0.99871	0.996107	1.0212397
9463	PICK1	protein interacting with PRKCA 1	0.97443	0.991633	1.0365011
9464	HAND2	heart and neural crest derivatives expressed 2	0.99156	0.988101	1.0534163

9465	AKAP7	A kinase (PRKA) anchor protein 7	0.9938	0.965081	0.9409616
9466	IL27RA	interleukin 27 receptor, alpha	0.91542	0.987759	1.0464321
9467	SH3BP5	SH3-domain binding protein 5 (BTK-associated)	0.98937	1.047812	0.9314838
9468	PCYT1B	phosphate cytidylyltransferase 1, choline, beta	0.97339	0.939693	0.981617
9469	CHST3	carbohydrate (chondroitin 6) sulfotransferase 3	1.00353	0.992957	1.0076975
9470	EIF4E2	eukaryotic translation initiation factor 4E family member 2	0.99114	0.932244	0.9976684
9472	AKAP6	A kinase (PRKA) anchor protein 6	0.99018	0.977732	0.9547204
9473	C1orf38	chromosome 1 open reading frame 38	1.02141	1.111653	0.9071291
9474	ATG5	ATG5 autophagy related 5 homolog (S. cerevisiae)	0.99904	0.881513	0.9610241
9475	ROCK2	Rho-associated, coiled-coil containing protein kinase 2	1.00582	0.934271	1.029376
9476	NAPSA	napsin A aspartic peptidase	1.00109	1.023634	0.9730664
9477	MED20	mediator complex subunit 20	1.00157	0.950151	0.9869245
9478	CABP1	calcium binding protein 1	0.96888	0.967981	1.0085587
9479	MAPK8IP1	mitogen-activated protein kinase 8 interacting protein 1	1.00474	1.023157	1.0080542
9480	ONECUT2	one cut homeobox 2	0.99143	1.015749	1.0300791
9481	SLC25A27	solute carrier family 25, member 27	1.00326	1.004476	0.9952738
9482	STX8	syntaxin 8	0.9844	0.97555	1.0063286
9486	CHST10	carbohydrate sulfotransferase 10	0.99658	0.961013	1.0206264
9487	PIGL	phosphatidylinositol glycan anchor biosynthesis, class L	0.96936	0.986117	0.9935506
9488	PIGB	phosphatidylinositol glycan anchor biosynthesis, class B	0.94478	0.946235	1.0951007
9489	PGS1	phosphatidylglycerophosphate synthase 1	0.95768	1.005002	0.9673859
9491	PSMF1	proteasome (prosome, macropain) inhibitor subunit 1 (PI31)	0.95491	0.99506	1.1209911
9493	KIF23	kinesin family member 23	0.97457	0.968255	1.0225028
9495	AKAP5	A kinase (PRKA) anchor protein 5	0.96505	1.074246	1.002059
9496	TBX4	T-box 4	0.98809	1.025104	0.9992644
9497	SLC4A7	solute carrier family 4, sodium bicarbonate cotransporter, member 7	0.9786	1.03314	0.9962813
9498	SLC4A8	solute carrier family 4, sodium bicarbonate cotransporter, member 8	0.99711	0.984754	0.9999559
9499	MYOT	myotilin	1.01043	1.003236	1.0075274
9500	MAGED1	melanoma antigen family D, 1	0.95569	0.955568	1.0218093
9501	RPH3AL	rabphilin 3A-like (without C2 domains)	0.9926	1.016676	1.0078854
9506	PAGE4	P antigen family, member 4 (prostate associated)	0.99323	0.998894	1.0267106
9507	ADAMTS4	ADAM metallopeptidase with thrombospondin type 1 motif, 4	0.99729	1.011224	1.0025948
9508	ADAMTS3	ADAM metallopeptidase with thrombospondin type 1 motif, 3	1.00474	0.989907	1.0066721

9509	ADAMTS2	ADAM metallopeptidase with thrombospondin type 1 motif, 2	1.00939	0.999376	1.0145205
9512	PMPCB	peptidase (mitochondrial processing) beta	0.99832	0.951522	1.0180661
9513	FXR2	fragile X mental retardation, autosomal homolog 2	0.90167	0.982793	1.0393907
9514	GAL3ST1	galactose-3-O-sulfotransferase 1	1.01027	0.988364	1.0052719
9515	STXBP5L	syntaxin binding protein 5-like	1.00868	1.008383	1.0127358
9516	LITAF	lipopolysaccharide-induced TNF factor	0.97286	0.965542	0.9068375
9517	SPTLC2	serine palmitoyltransferase, long chain base subunit 2	0.98517	1.022435	0.9863003
9518	GDF15	growth differentiation factor 15	0.99753	1.021441	1.008026
9519	TBPL1	TBP-like 1	0.99583	0.920083	1.0139603
9520	NPEPPS	aminopeptidase puromycin sensitive	0.98517	0.98789	1.0320168
9521	EEF1E1	eukaryotic translation elongation factor 1 epsilon 1	0.9934	0.990408	1.0411762
9522	SCAMP1	secretory carrier membrane protein 1	0.96297	0.890794	1.0522604
9524	TECR	trans-2,3-enoyl-CoA reductase	0.95679	1.012111	1.0331434
9525	VPS4B	vacuolar protein sorting 4 homolog B (S. cerevisiae)	0.93545	1.009737	1.0251445
9526	MPDU1	mannose-P-dolichol utilization defect 1	0.93084	0.886641	1.1348063
9527	GOSR1	golgi SNAP receptor complex member 1	0.96723	0.960285	1.0568792
9528	TMEM59	transmembrane protein 59	0.99715	0.871876	0.9852305
9529	BAG5	BCL2-associated athanogene 5	0.91554	0.965912	1.0703967
9530	BAG4	BCL2-associated athanogene 4	0.97054	0.898167	1.0004118
9531	BAG3	BCL2-associated athanogene 3	0.96237	0.952197	1.015279
9532	BAG2	BCL2-associated athanogene 2	0.98785	0.971091	1.0105194
9533	POLR1C	polymerase (RNA) I polypeptide C, 30kDa	0.9666	0.921925	1.0708537
9534	ZNF254	zinc finger protein 254	0.95515	1.023802	0.9564587
9535	GMFG	glia maturation factor, gamma	0.98802	0.975003	1.0297165
9536	PTGES	prostaglandin E synthase	0.99872	1.103512	0.9400152
9537	TP53I11	tumor protein p53 inducible protein 11	1.00705	1.043356	1.0079914
9538	EI24	etoposide induced 2.4 mRNA	0.92407	0.999923	1.0389409
9540	TP53I3	tumor protein p53 inducible protein 3	0.99915	0.935649	1.0914624
9541	CIR1	corepressor interacting with RBPJ, 1	1.00375	0.926771	0.927046
9542	NRG2	neuregulin 2	1.01318	0.999621	1.0075605
9543	IGDCC3	immunoglobulin superfamily, DCC subclass, member 3	1.00395	0.997801	1.0025063
9546	APBA3	amyloid beta (A4) precursor protein-binding, family A, member 3	0.98698	0.951776	1.0499203
9547	CXCL14	chemokine (C-X-C motif) ligand 14	1.01722	1.016449	1.0059008

9550	ATP6V1G1	ATPase, H+ transporting, lysosomal 13kDa, V1 subunit G1	0.98937	0.921948	0.9700629
9551	ATP5J2	ATP synthase, H+ transporting, mitochondrial Fo complex, subunit F2	1.03183	1.043451	1.0091194
9552	SPAG7	sperm associated antigen 7	0.97837	1.035897	1.0245521
9553	MRPL33	mitochondrial ribosomal protein L33	1	0.951594	0.9543734
9554	SEC22B	SEC22 vesicle trafficking protein homolog B (S. cerevisiae) (gene/pseudogene)	1.00923	1.022898	0.9355544
9555	H2AFY	H2A histone family, member Y	1.02822	1.006156	0.956135
9556	C14orf2	chromosome 14 open reading frame 2	0.93046	0.994142	1.0899404
9557	CHD1L	chromodomain helicase DNA binding protein 1-like	0.9872	0.912056	1.0749312
9559	VPS26A	vacuolar protein sorting 26 homolog A (S. pombe)	0.97456	1.040208	0.9695252
9562	MINPP1	multiple inositol-polyphosphate phosphatase 1	0.8964	0.937374	1.023189
9563	H6PD	hexose-6-phosphate dehydrogenase (glucose 1-dehydrogenase)	0.9949	0.939132	0.9728058
9564	BCAR1	breast cancer anti-estrogen resistance 1	0.99225	1.036197	1.0233789
9567	GTPBP1	GTP binding protein 1	0.97324	1.011874	0.920354
9568	GABBR2	gamma-aminobutyric acid (GABA) B receptor, 2	0.99722	1.001389	1.0089684
9569	GTF2IRD1	GTF2I repeat domain containing 1	1.00959	0.988962	1.0199409
9570	GOSR2	golgi SNAP receptor complex member 2	0.97572	0.954565	1.0079368
9572	NR1D1	nuclear receptor subfamily 1, group D, member 1	0.96332	0.986431	0.9499482
9573	GDF3	growth differentiation factor 3	0.97072	1.046186	1.0278674
9575	CLOCK	clock homolog (mouse)	0.97212	1.001133	0.945549
9576	SPAG6	sperm associated antigen 6	0.99425	0.988205	1.0072204
9577	BRE	brain and reproductive organ-expressed (TNFRSF1A modulator)	0.9936	1.060565	0.9673544
9578	CDC42BPB	CDC42 binding protein kinase beta (DMPK-like)	0.97144	0.954748	1.0566888
9580	SOX13	SRY (sex determining region Y)-box 13	1.00152	0.997484	1.0453642
9581	PREPL	prolyl endopeptidase-like	0.98319	0.948556	1.0346715
9582	APOBEC3B	apolipoprotein B mRNA editing enzyme, catalytic polypeptide-like 3B	0.99674	1.085932	1.0184124
9583	ENTPD4	ectonucleoside triphosphate diphosphohydrolase 4	0.95497	0.993635	0.9466151
9584	RBM39	RNA binding motif protein 39	0.99861	0.883525	0.9542677
9585	KIF20B	kinesin family member 20B	0.93672	0.926029	1.094288
9586	CREB5	cAMP responsive element binding protein 5	1.01478	0.982357	1.0222159
9587	MAD2L1BP	MAD2L1 binding protein	0.9163	0.917724	0.9987661
9588	PRDX6	peroxiredoxin 6	1.00577	0.96694	0.9706686
9589	WTAP	Wilms tumor 1 associated protein	1.02827	0.999187	0.9343062
9590	AKAP12	A kinase (PRKA) anchor protein 12	0.99659	0.985393	0.9866351

9592	IER2	immediate early response 2	0.94512	0.933992	0.8521087
9595	CYTIP	cytohesin 1 interacting protein	1.0157	1.067458	0.9991303
9596	OR1R1P	olfactory receptor, family 1, subfamily R, member 1 pseudogene	0.98135	0.99186	1.0514173
9597	SMAD5-AS1	SMAD5 antisense RNA 1 (non-protein coding)	0.98858	0.96972	0.9675918
9600	PITPNM1	phosphatidylinositol transfer protein, membrane-associated 1	0.99773	0.992651	0.9655172
9601	PDIA4	protein disulfide isomerase family A, member 4	1.00067	0.927244	1.0592033
9603	NFE2L3	nuclear factor (erythroid-derived 2)-like 3	1.00199	1.022264	0.9953286
9604	RNF14	ring finger protein 14	0.9911	0.880133	1.1637675
9605	C16orf7	chromosome 16 open reading frame 7	0.9828	1.035428	1.0462325
9607	CARTPT	CART prepropeptide	1	1.017168	1.0207971
9610	RIN1	Ras and Rab interactor 1	0.97628	0.954807	0.9608576
9611	NCOR1	nuclear receptor corepressor 1	0.96535	1.043491	1.0446735
9612	NCOR2	nuclear receptor corepressor 2	0.98536	1.089581	0.9751284
9615	GDA	guanine deaminase	1.00747	1.005218	1.0005762
9616	RNF7	ring finger protein 7	1.01426	0.984945	0.9819649
9617	MTRF1	mitochondrial translational release factor 1	0.93382	1.009935	1.0816934
9618	TRAF4	TNF receptor-associated factor 4	1.01904	1.041801	0.9904026
9619	ABCG1	ATP-binding cassette, sub-family G (WHITE), member 1	0.96832	1.011981	0.9754012
9620	CELSR1	cadherin, EGF LAG seven-pass G-type receptor 1 (flamingo homolog, Drosophila)	0.98898	0.989322	0.9661032
9622	KLK4	kallikrein-related peptidase 4	0.98258	1.003909	1.0087299
9623	TCL1B	T-cell leukemia/lymphoma 1B	0.95275	0.993541	1.0347173
9625	AATK	apoptosis-associated tyrosine kinase	0.99219	1.01775	0.9835735
9626	GUCA1C	guanylate cyclase activator 1C	1.01721	1.008225	1.015062
9627	SNCAIP	synuclein, alpha interacting protein	1.00891	0.995938	1.0312013
9628	RGS6	regulator of G-protein signaling 6	0.99268	0.995414	0.9865634
9629	CLCA3P	chloride channel accessory 3, pseudogene	1.0019	1.024194	1.0169859
9630	GNA14	guanine nucleotide binding protein (G protein), alpha 14	1.00666	1.009598	0.9969005
9631	NUP155	nucleoporin 155kDa	0.98666	1.000404	1.0892176
9632	SEC24C	SEC24 family, member C (S. cerevisiae)	0.95397	1.099725	1.1209228
9633	MTL5	metallothionein-like 5, testis-specific (tesmin)	0.98774	0.99548	1.0055363
9635	CLCA2	chloride channel accessory 2	1.01217	1.006393	1.0145556
9636	ISG15	ISG15 ubiquitin-like modifier	1.02197	0.95878	1.0306719
9637	FEZ2	fasciculation and elongation protein zeta 2 (zygin II)	0.99048	0.943355	1.0923885

9638	FEZ1	fasciculation and elongation protein zeta 1 (zygin I)	0.98848	1.00914	0.9747049
9639	ARHGEF10	Rho guanine nucleotide exchange factor (GEF) 10	0.99966	1.004446	0.9884718
9640	ZNF592	zinc finger protein 592	0.98149	0.97189	1.0366251
9641	IKBKE	inhibitor of kappa light polypeptide gene enhancer in B-cells, kinase epsilon	0.98368	0.958962	1.0631681
9643	MORF4L2	mortality factor 4 like 2	0.95758	1.014749	1.0348715
9644	SH3PXD2A	SH3 and PX domains 2A	0.98737	0.988108	1.0300393
9645	MICAL2	microtubule associated monoxygenase, calponin and LIM domain containing 2	0.96758	0.895703	1.0114074
9646	CTR9	Ctr9, Paf1/RNA polymerase II complex component, homolog (S. cerevisiae)	0.92039	0.91442	1.0155847
9647	PPM1F	protein phosphatase, Mg2+/Mn2+ dependent, 1F	0.98436	0.997001	1.0193608
9648	GCC2	GRIP and coiled-coil domain containing 2	0.95116	1.02869	0.830466
9649	RALGPS1	Ral GEF with PH domain and SH3 binding motif 1	0.9938	0.987651	0.9872071
9650	MTFR1	mitochondrial fission regulator 1	0.95119	0.919259	1.0677323
9651	PLCH2	phospholipase C, eta 2	0.98103	0.961535	0.9744499
9652	TTC37	tetratricopeptide repeat domain 37	0.9874	0.953113	1.0903053
9653	HS2ST1	heparan sulfate 2-O-sulfotransferase 1	0.99515	0.961087	1.0484846
9654	TTLL4	tubulin tyrosine ligase-like family, member 4	1.03543	1.072459	1.0083685
9655	SOCS5	suppressor of cytokine signaling 5	0.99781	0.983991	0.9921217
9656	MDC1	mediator of DNA-damage checkpoint 1	0.98565	1.018909	0.943987
9657	IQCB1	IQ motif containing B1	0.98329	0.908965	1.0869633
9658	ZNF516	zinc finger protein 516	0.97896	0.992208	0.9589281
9659	PDE4DIP	phosphodiesterase 4D interacting protein	0.99944	1.01024	0.9729993
9662	CEP135	centrosomal protein 135kDa	0.97694	0.963059	1.0560671
9663	LPIN2	lipin 2	0.93452	1.044778	0.9536744
9665	KIAA0430	KIAA0430	0.96097	1.052668	1.0868067
9666	DZIP3	DAZ interacting protein 3, zinc finger	0.98905	0.991851	1.0808081
9667	SAFB2	scaffold attachment factor B2	0.94338	0.995106	0.9387023
9668	ZNF432	zinc finger protein 432	0.95924	0.987377	1.0005531
9669	EIF5B	eukaryotic translation initiation factor 5B	0.952	0.980895	1.0033319
9670	IPO13	importin 13	0.96385	1.014533	1.0249404
9671	WSCD2	WSC domain containing 2	0.98246	1.020885	1.0129147
9672	SDC3	syndecan 3	1.01803	0.964845	1.0048557
9673	SLC25A44	solute carrier family 25, member 44	0.97424	0.973109	1.0284538
9674	KIAA0040	KIAA0040	1.01651	1.010063	1.0721096

9675	TTI1	Tel2 interacting protein 1 homolog (S. pombe)	0.94134	0.979813	1.0452385
9677	PPIP5K1	diphosphoinositol pentakisphosphate kinase 1	0.97079	0.950209	0.9886497
9678	PHF14	PHD finger protein 14	0.99757	0.936778	0.9967143
9679	FAM53B	family with sequence similarity 53, member B	1.02274	1.051673	0.9269007
9681	DEPDC5	DEP domain containing 5	0.96698	0.9643	1.0094251
9682	KDM4A	lysine (K)-specific demethylase 4A	0.98714	0.985994	1.0433142
9683	N4BP1	NEDD4 binding protein 1	0.96824	0.993709	0.9360553
9684	LRRC14	leucine rich repeat containing 14	0.99861	0.950094	1.0289574
9685	CLINT1	clathrin interactor 1	1.00611	0.940515	1.0011616
9686	VGLL4	vestigial like 4 (Drosophila)	0.99487	0.980778	1.0282329
9687	GREB1	growth regulation by estrogen in breast cancer 1	1.00386	1.006556	1.0131025
9688	NUP93	nucleoporin 93kDa	0.95603	0.889759	1.0872049
9689	BZW1	basic leucine zipper and W2 domains 1	1.02705	1.041657	1.0260619
9690	UBE3C	ubiquitin protein ligase E3C	0.97321	0.963884	0.9913947
9692	KIAA0391	KIAA0391	0.91476	1.057472	1.0659083
9693	RAPGEF2	Rap guanine nucleotide exchange factor (GEF) 2	1.00553	1.057173	0.9933877
9694	TTC35	tetratricopeptide repeat domain 35	0.98726	1.003247	0.9822596
9695	EDEM1	ER degradation enhancer, mannosidase alpha-like 1	1.03999	0.975515	0.9620371
9696	CROCC	ciliary rootlet coiled-coil, rootletin	0.99123	0.997235	1.0252308
9697	TRAM2	translocation associated membrane protein 2	1.00119	0.937791	1.0361077
9698	PUM1	pumilio homolog 1 (Drosophila)	0.98165	0.914571	0.9466253
9699	RIMS2	regulating synaptic membrane exocytosis 2	1.00693	1.007172	1.0321989
9700	ESPL1	extra spindle pole bodies homolog 1 (S. cerevisiae)	0.99767	0.997276	0.9998547
9701	PPP6R2	protein phosphatase 6, regulatory subunit 2	0.95302	1.004192	0.952627
9702	CEP57	centrosomal protein 57kDa	0.94987	0.993022	1.0182038
9703	KIAA0100	KIAA0100	0.96619	0.949841	1.0971421
9704	DHX34	DEAH (Asp-Glu-Ala-His) box polypeptide 34	0.98836	0.989667	0.9656379
9705	ST18	suppression of tumorigenicity 18 (breast carcinoma) (zinc finger protein)	1.00642	1.01665	1.0079372
9706	ULK2	unc-51-like kinase 2 (C. elegans)	0.97212	0.879455	0.97317
9709	HERPUD1	homocysteine-inducible, endoplasmic reticulum stress-inducible, ubiquitin-like domain member 1	0.99946	1.012685	0.9911231
9710	KIAA0355	KIAA0355	0.94554	1.005601	0.9811222
9711	KIAA0226	KIAA0226	0.99755	1.130586	0.9338758
9712	USP6NL	USP6 N-terminal like	0.94013	1.021058	1.0510421

9715	FAM131B	family with sequence similarity 131, member B	0.9968	0.99802	1.0052601
9716	AQR	aquarius homolog (mouse)	0.93504	1.060266	1.1643077
9717	SEC14L5	SEC14-like 5 (S. cerevisiae)	0.96551	0.964618	0.9884119
9718	ECE2	endothelin converting enzyme 2	1.00524	0.983852	1.0219972
9719	ADAMTSL2	ADAMTS-like 2	0.98407	0.985419	1.0435642
9721	GPRIN2	G protein regulated inducer of neurite outgrowth 2	0.99885	0.956555	0.9783972
9722	NOS1AP	nitric oxide synthase 1 (neuronal) adaptor protein	1.01304	1.01274	0.9991879
9723	SEMA3E	sema domain, immunoglobulin domain (Ig), short basic domain, secreted, (semaphorin) 3E	1.00036	0.993927	1.0194439
9724	UTP14C	UTP14, U3 small nucleolar ribonucleoprotein, homolog C (yeast)	0.94028	0.975221	0.9907254
9725	TMEM63A	transmembrane protein 63A	0.97642	0.960356	0.8302581
9726	ZNF646	zinc finger protein 646	0.96383	1.018031	0.9776773
9727	RAB11FIP3	RAB11 family interacting protein 3 (class II)	0.97302	1.011833	1.0041134
9728	SECISBP2L	SECIS binding protein 2-like	0.93683	0.999087	1.0273832
9729	KIAA0408	KIAA0408	0.96966	0.921626	1.001624
9730	VPRBP	Vpr (HIV-1) binding protein	0.97628	0.983776	1.0655969
9731	CEP104	centrosomal protein 104kDa	0.9793	1.007138	0.9988167
9732	DOCK4	dedicator of cytokinesis 4	1.01612	1.021812	0.9860641
9733	SART3	squamous cell carcinoma antigen recognized by T cells 3	0.98339	0.979512	0.9670999
9734	HDAC9	histone deacetylase 9	1.01022	0.960965	1.0157073
9735	KNTC1	kinetochore associated 1	0.95694	0.985672	1.0483409
9736	USP34	ubiquitin specific peptidase 34	0.99791	0.990057	0.9535629
9737	GPRASP1	G protein-coupled receptor associated sorting protein 1	0.92224	0.965252	0.9515637
9738	CCP110	centriolar coiled coil protein 110kDa	0.93957	1.010522	1.1236916
9739	SETD1A	SET domain containing 1A	0.95884	0.997279	1.0248075
9741	LAPTM4A	lysosomal protein transmembrane 4 alpha	1.01452	0.918057	1.054617
9742	IFT140	intraflagellar transport 140 homolog (Chlamydomonas)	0.98466	1.002101	1.0140138
9743	ARHGAP32	Rho GTPase activating protein 32	0.9511	0.93826	0.9607305
9744	ACAP1	ArfGAP with coiled-coil, ankyrin repeat and PH domains 1	0.94799	1.0676	1.0134516
9745	ZNF536	zinc finger protein 536	0.99759	0.993423	1.0003238
9746	CLSTN3	calsyntenin 3	1.01593	1.060606	0.9683762
9747	FAM115A	family with sequence similarity 115, member A	0.98869	1.014502	1.0472973
9748	SLK	STE20-like kinase	0.96147	1.078518	1.0446351
9749	PHACTR2	phosphatase and actin regulator 2	0.96933	0.985354	0.9592192

9750	FAM65B	family with sequence similarity 65, member B	0.99881	0.901432	0.8048287
9751	SNPH	syntaphilin	0.97187	0.993657	0.9735584
9753	ZSCAN12	zinc finger and SCAN domain containing 12	0.93659	0.958144	1.0169331
9754	STARD8	StAR-related lipid transfer (START) domain containing 8	0.96676	1.082928	0.982034
9755	TBKBP1	TBK1 binding protein 1	0.9965	1.051769	0.9751685
9757	MLL4	myeloid/lymphoid or mixed-lineage leukemia 4	0.97427	1.013234	0.9399422
9758	FRMPD4	FERM and PDZ domain containing 4	0.99394	0.993149	1.001226
9759	HDAC4	histone deacetylase 4	1.00057	0.993057	0.9835054
9760	TOX	thymocyte selection-associated high mobility group box	1.00041	1.018313	1.0134112
9761	MLEC	malectin	0.95493	0.958016	1.0258285
9762	ProSAPiP1	ProSAPiP1 protein	0.97593	1.048222	0.9590846
9764	KIAA0513	KIAA0513	0.97743	1.0105	0.9618696
9765	ZFYVE16	zinc finger, FYVE domain containing 16	0.9993	0.998133	1.008241
9766	KIAA0247	KIAA0247	0.97306	1.055815	0.9754802
9767	PHF16	PHD finger protein 16	0.98744	0.993979	0.9933618
9768	KIAA0101	KIAA0101	0.9807	0.95829	1.0276268
9770	RASSF2	Ras association (RalGDS/AF-6) domain family member 2	0.96582	0.96048	1.0143601
9771	RAPGEF5	Rap guanine nucleotide exchange factor (GEF) 5	0.99748	0.990155	1.0292006
9772	KIAA0195	KIAA0195	0.96895	0.922723	1.003791
9774	BCLAF1	BCL2-associated transcription factor 1	1.00606	0.882933	0.9828377
9775	EIF4A3	eukaryotic translation initiation factor 4A3	0.92357	1.070362	1.002929
9776	ATG13	ATG13 autophagy related 13 homolog (S. cerevisiae)	0.9378	0.943727	1.0252159
9777	TM9SF4	transmembrane 9 superfamily protein member 4	0.96174	0.928032	1.0722758
9778	KIAA0232	KIAA0232	1.00348	1.058017	1.0542738
9779	TBC1D5	TBC1 domain family, member 5	1.00623	0.896727	1.0092405
9780	FAM38A	family with sequence similarity 38, member A	1.01362	0.940818	1.1274287
9781	RNF144A	ring finger protein 144A	0.96681	0.967431	1.0101418
9782	MATR3	matrin 3	1.02006	0.934123	1.0243165
9783	RIMS3	regulating synaptic membrane exocytosis 3	1.01062	1.026118	1.0136974
9784	SNX17	sorting nexin 17	0.99305	0.935161	1.0440522
9785	DHX38	DEAH (Asp-Glu-Ala-His) box polypeptide 38	0.94529	1.041351	1.0577995
9786	KIAA0586	KIAA0586	0.91299	0.964654	1.0272755
9787	DLGAP5	discs, large (Drosophila) homolog-associated protein 5	0.97292	0.961937	1.0189644

9788	MTSS1	metastasis suppressor 1	1.04519	1.081166	1.0086128
9789	SPCS2	signal peptidase complex subunit 2 homolog (S. cerevisiae)	1.02085	1.032506	1.0358094
9790	BMS1	BMS1 homolog, ribosome assembly protein (yeast)	0.96709	0.948644	0.9960144
9791	PTDSS1	phosphatidylserine synthase 1	0.99536	0.93282	1.0435248
9792	SERTAD2	SERTA domain containing 2	0.99584	0.886876	0.9425871
9793	CKAP5	cytoskeleton associated protein 5	0.94112	0.946171	1.0291895
9794	MAML1	mastermind-like 1 (Drosophila)	1.00971	1.044787	1.0049592
9796	PHYHIP	phytanoyl-CoA 2-hydroxylase interacting protein	0.99885	1.016258	1.0044839
9797	TATDN2	TatD DNase domain containing 2	0.95718	1.026515	0.9451823
9798	KIAA0174	KIAA0174	0.99269	1.015074	0.9653079
9801	MRPL19	mitochondrial ribosomal protein L19	0.98117	0.982754	1.0288398
9802	DAZAP2	DAZ associated protein 2	0.96648	0.925334	1.0243278
9804	TOMM20	translocase of outer mitochondrial membrane 20 homolog (yeast)	0.95524	0.948727	0.9864897
9805	SCRN1	secernin 1	1.0179	0.908747	1.0187004
9806	SPOCK2	sparc/osteonectin, cwcv and kazal-like domains proteoglycan (testican) 2	0.94216	1.020876	1.0169092
9807	IP6K1	inositol hexakisphosphate kinase 1	1.00021	1.032042	0.9745501
9810	RNF40	ring finger protein 40	0.94732	1.081247	1.0508161
9811	CTIF	CBP80/20-dependent translation initiation factor	0.99483	0.982704	1.0182041
9812	KIAA0141	KIAA0141	0.98817	0.88173	1.0357338
9813	KIAA0494	KIAA0494	0.99267	0.870243	0.9816679
9814	SFI1	Sfi1 homolog, spindle assembly associated (yeast)	0.94151	0.990571	0.9269142
9815	GIT2	G protein-coupled receptor kinase interacting ArfGAP 2	0.95675	1.013647	1.0020015
9816	URB2	URB2 ribosome biogenesis 2 homolog (S. cerevisiae)	0.97444	0.912251	1.0821618
9817	KEAP1	kelch-like ECH-associated protein 1	0.95725	1.002583	1.0852668
9818	NUPL1	nucleoporin like 1	0.96196	1.074953	0.9624711
9819	TSC22D2	TSC22 domain family, member 2	0.98137	0.993503	0.9770132
9820	CUL7	cullin 7	1.0053	0.988587	0.9856276
9821	RB1CC1	RB1-inducible coiled-coil 1	0.95916	1.013233	0.9383018
9823	ARMCX2	armadillo repeat containing, X-linked 2	0.95959	1.025407	1.0264199
9824	ARHGAP11	Rho GTPase activating protein 11A	0.97642	1.061323	1.0257399
9825	SPATA2	spermatogenesis associated 2	0.95983	0.97513	0.9500382
9826	ARHGEF11	Rho guanine nucleotide exchange factor (GEF) 11	1.00453	0.893717	1.0585334
9827	RGP1	RGP1 retrograde golgi transport homolog (S. cerevisiae)	0.95285	0.986576	1.0226436

9828	ARHGEF17	Rho guanine nucleotide exchange factor (GEF) 17	1.00113	0.995388	1.0193769
9829	DNAJC6	DnaJ (Hsp40) homolog, subfamily C, member 6	0.99796	0.9664	1.0031883
9830	TRIM14	tripartite motif containing 14	0.95399	0.938942	0.9812334
9831	ZNF623	zinc finger protein 623	0.96419	0.885524	0.9628023
9832	JAKMIP2	janus kinase and microtubule interacting protein 2	1.00325	0.992982	0.946537
9833	MELK	maternal embryonic leucine zipper kinase	0.97872	0.981922	1.0156877
9836	LCMT2	leucine carboxyl methyltransferase 2	0.8577	0.937463	0.9400677
9837	GINS1	GINS complex subunit 1 (Psf1 homolog)	0.98619	0.999489	1.0213797
9839	ZEB2	zinc finger E-box binding homeobox 2	1.08647	1.083586	0.9769194
9840	KIAA0748	KIAA0748	0.91883	0.860582	0.8836698
9841	ZBTB24	zinc finger and BTB domain containing 24	0.98212	0.965434	1.0459254
9842	PLEKHM1	pleckstrin homology domain containing, family M (with RUN domain) member 1	0.93866	0.990763	1.0249536
9843	HEPH	hephaestin	0.99703	0.981291	1.0096692
9844	ELMO1	engulfment and cell motility 1	1.01059	0.917259	0.9523134
9846	GAB2	GRB2-associated binding protein 2	1.00599	1.008853	1.0402817
9847	KIAA0528	KIAA0528	0.92198	0.997865	1.0231308
9848	MFAP3L	microfibrillar-associated protein 3-like	0.94653	0.8972	0.9930083
9849	ZNF518A	zinc finger protein 518A	0.95355	0.931929	0.9843646
9851	KIAA0753	KIAA0753	0.96173	1.035192	1.0897596
9852	EPM2AIP1	EPM2A (laforin) interacting protein 1	1.03652	0.944883	0.98624
9853	RUSC2	RUN and SH3 domain containing 2	0.98869	0.981093	0.9773306
9854	C2CD2L	C2CD2-like	0.94691	0.943211	1.0038863
9855	FARP2	FERM, RhoGEF and pleckstrin domain protein 2	1.00202	0.953806	0.9644513
9856	KIAA0319	KIAA0319	1.00099	1.016827	0.9953541
9857	CEP350	centrosomal protein 350kDa	0.96945	1.015956	1.069359
9858	KIAA0649	KIAA0649	0.99463	0.977635	1.045977
9859	CEP170	centrosomal protein 170kDa	0.97719	0.960745	1.0096083
9860	LRIG2	leucine-rich repeats and immunoglobulin-like domains 2	0.93589	0.976472	0.9666726
9861	PSMD6	proteasome (prosome, macropain) 26S subunit, non-ATPase, 6	1.02482	0.923068	1.0082222
9862	MED24	mediator complex subunit 24	0.94884	0.933987	0.9967213
9863	MAGI2	membrane associated guanylate kinase, WW and PDZ domain containing 2	1.00924	1.011741	1.0168177
9865	TRIL	TLR4 interactor with leucine-rich repeats	1.01466	1.008968	1.017392
9866	TRIM66	tripartite motif containing 66	0.96093	0.903213	1.0880725

9867	PJA2	praja ring finger 2	1.02764	1.005967	1.0137305
9868	TOMM70A	translocase of outer mitochondrial membrane 70 homolog A (S. cerevisiae)	0.95245	0.898876	1.0651981
9869	SETDB1	SET domain, bifurcated 1	0.98438	0.979304	1.0876351
9870	KIAA0317	KIAA0317	0.95681	1.010417	0.9883591
9871	SEC24D	SEC24 family, member D (S. cerevisiae)	1.02602	0.954493	1.0327957
9873	FCHSD2	FCH and double SH3 domains 2	0.97291	1.048377	0.9929681
9874	TLK1	tousled-like kinase 1	0.99913	0.946014	1.0124251
9877	ZC3H11A	zinc finger CCCH-type containing 11A	1.00444	0.970435	0.9156358
9878	TOX4	TOX high mobility group box family member 4	0.97852	0.943987	0.9427033
9879	DDX46	DEAD (Asp-Glu-Ala-Asp) box polypeptide 46	0.98015	0.969679	1.0039723
9880	ZBTB39	zinc finger and BTB domain containing 39	0.8949	0.982109	1.0751009
9881	TRANK1	tetratricopeptide repeat and ankyrin repeat containing 1	0.98817	1.028678	0.9804748
9882	TBC1D4	TBC1 domain family, member 4	0.98159	0.956693	0.9803725
9883	POM121	POM121 membrane glycoprotein	1.02216	1.007156	0.972187
9885	OSBPL2	oxysterol binding protein-like 2	0.96356	1.036858	0.9797384
9886	RHOBTB1	Rho-related BTB domain containing 1	0.98654	0.981914	0.9963859
9887	SMG7	Smg-7 homolog, nonsense mediated mRNA decay factor (C. elegans)	0.99047	1.006804	1.0713211
9889	ZBED4	zinc finger, BED-type containing 4	0.96809	1.02747	1.0396675
9890	LPPR4	lipid phosphate phosphatase-related protein type 4	1.01559	1.041975	1.006448
9891	NUAK1	NUAK family, SNF1-like kinase, 1	1.01065	1.04818	0.9827618
9892	SNAP91	synaptosomal-associated protein, 91kDa homolog (mouse)	1.00203	0.996803	1.0057271
9894	TELO2	TEL2, telomere maintenance 2, homolog (S. cerevisiae)	0.99778	0.996989	1.0573915
9895	TECPR2	tectonin beta-propeller repeat containing 2	0.96917	1.019874	0.9415766
9896	FIG4	FIG4 homolog, SAC1 lipid phosphatase domain containing (S. cerevisiae)	0.99813	0.93668	1.1542862
9897	KIAA0196	KIAA0196	0.93981	0.957381	1.0616014
9898	UBAP2L	ubiquitin associated protein 2-like	1.00429	1.067884	0.9822172
9899	SV2B	synaptic vesicle glycoprotein 2B	1.00953	0.979517	0.9953139
9900	SV2A	synaptic vesicle glycoprotein 2A	0.98495	0.998199	1.0301798
9901	SRGAP3	SLIT-ROBO Rho GTPase activating protein 3	1.02246	0.981221	0.9904492
9902	MRC2	mannose receptor, C type 2	0.99461	0.973113	1.0301037
9903	KLHL21	kelch-like 21 (Drosophila)	1.00843	0.95033	0.9032962
9904	RBM19	RNA binding motif protein 19	0.93279	0.93673	1.0017206
9905	SGSM2	small G protein signaling modulator 2	0.96506	0.983749	1.0030005

9906	SLC35E2	solute carrier family 35, member E2	1.0351	0.953512	1.0534817
9907	KIAA0415	KIAA0415	1.00278	0.997819	1.0097156
9908	G3BP2	GTPase activating protein (SH3 domain) binding protein 2	1.00133	0.948956	0.902305
9909	DENND4B	DENN/MADD domain containing 4B	1.00419	0.932124	0.994147
9910	RABGAP1L	RAB GTPase activating protein 1-like	1.00954	0.996434	0.9926298
9911	TMCC2	transmembrane and coiled-coil domain family 2	1.00401	0.976273	1.0119849
9912	ARHGAP44	Rho GTPase activating protein 44	0.99412	0.982001	1.0095465
9913	SUPT7L	suppressor of Ty 7 (S. cerevisiae)-like	0.97708	0.88743	1.0635407
9914	ATP2C2	ATPase, Ca++ transporting, type 2C, member 2	0.99493	1.013665	0.9947851
9915	ARNT2	aryl-hydrocarbon receptor nuclear translocator 2	1.00092	0.994943	0.9866777
9917	FAM20B	family with sequence similarity 20, member B	1.00076	0.882378	1.0257193
9918	NCAPD2	non-SMC condensin I complex, subunit D2	0.93729	0.875216	1.1161563
9919	SEC16A	SEC16 homolog A (S. cerevisiae)	0.98051	1.008128	1.0024366
9920	KBTBD11	kelch repeat and BTB (POZ) domain containing 11	0.98311	0.969697	1.0244052
9921	RNF10	ring finger protein 10	0.96246	0.999792	0.9872454
9922	IQSEC1	IQ motif and Sec7 domain 1	0.98873	0.98665	0.9401889
9923	ZBTB40	zinc finger and BTB domain containing 40	0.98084	0.875704	0.950562
9924	PAN2	PAN2 poly(A) specific ribonuclease subunit homolog (S. cerevisiae)	0.9623	0.916189	0.9927302
9925	ZBTB5	zinc finger and BTB domain containing 5	0.97857	0.938698	0.9365731
9926	LPGAT1	lysophosphatidylglycerol acyltransferase 1	1.00725	0.949122	0.9876036
9927	MFN2	mitofusin 2	0.95133	1.03079	0.9573203
9928	KIF14	kinesin family member 14	0.99086	0.925604	1.0134687
9929	JOSD1	Josephin domain containing 1	0.94211	1.03933	0.9222706
9931	HELZ	helicase with zinc finger	0.97351	0.966916	0.9971365
9933	KIAA0020	KIAA0020	0.95241	0.991445	1.0420508
9934	P2RY14	purinergic receptor P2Y, G-protein coupled, 14	0.98575	0.969081	1.0081597
9935	MAFB	v-maf musculoaponeurotic fibrosarcoma oncogene homolog B (avian)	0.98792	0.863868	0.962984
9936	CD302	CD302 molecule	1.00483	0.916112	1.0365357
9937	DCLRE1A	DNA cross-link repair 1A	0.89393	0.98729	1.0662142
9938	ARHGAP25	Rho GTPase activating protein 25	0.97092	1.022301	0.8943506
9939	RBM8A	RNA binding motif protein 8A	1.03991	0.967989	0.9350514
9940	DLEC1	deleted in lung and esophageal cancer 1	1.00726	0.985299	1.0108326
9941	EXOG	endo/exonuclease (5'-3'), endonuclease G-like	0.99283	0.906211	0.9969607

9942	XYLB	xylulokinase homolog (H. influenzae)	0.99958	0.973038	1.0125064
9943	OXSR1	oxidative-stress responsive 1	1.00046	0.976585	0.9926321
9945	GFPT2	glutamine-fructose-6-phosphate transaminase 2	1.00428	0.988622	1.0095194
9946	CRYZL1	crystallin, zeta (quinone reductase)-like 1	0.97952	0.959216	1.0523689
9947	MAGEC1	melanoma antigen family C, 1	0.97938	1.024338	0.9830724
9948	WDR1	WD repeat domain 1	1.01	1.002274	0.9459576
9949	AMMECR1	Alport syndrome, mental retardation, midface hypoplasia and elliptocytosis chromosomal region gene 1	0.96155	0.954188	1.0923754
9950	GOLGA5	golgin A5	0.94595	1.006054	1.0254036
9951	HS3ST4	heparan sulfate (glucosamine) 3-O-sulfotransferase 4	0.99429	1.003883	0.9926518
9953	HS3ST3B1	heparan sulfate (glucosamine) 3-O-sulfotransferase 3B1	0.96608	1.026231	0.9602047
9955	HS3ST3A1	heparan sulfate (glucosamine) 3-O-sulfotransferase 3A1	0.98501	1.003433	0.994979
9956	HS3ST2	heparan sulfate (glucosamine) 3-O-sulfotransferase 2	1.00364	0.990104	0.9995331
9957	HS3ST1	heparan sulfate (glucosamine) 3-O-sulfotransferase 1	1.00251	1.019265	1.0379157
9958	USP15	ubiquitin specific peptidase 15	0.97248	1.018906	1.0847363
9960	USP3	ubiquitin specific peptidase 3	0.98687	0.933027	1.01385
9961	MVP	major vault protein	0.94092	0.969062	1.0757296
9962	SLC23A2	solute carrier family 23 (nucleobase transporters), member 2	0.96838	1.041138	0.9785775
9963	SLC23A1	solute carrier family 23 (nucleobase transporters), member 1	1.01736	1.000179	1.0340851
9965	FGF19	fibroblast growth factor 19	0.99512	0.980368	1.0083925
9966	TNFSF15	tumor necrosis factor (ligand) superfamily, member 15	0.96969	1.004281	1.0007624
9967	THRAP3	thyroid hormone receptor associated protein 3	0.98577	0.984877	0.9319296
9968	MED12	mediator complex subunit 12	0.98169	1.068291	0.9840167
9969	MED13	mediator complex subunit 13	0.95952	1.083517	1.016354
9971	NR1H4	nuclear receptor subfamily 1, group H, member 4	1.00154	1.004104	1.0033957
9972	NUP153	nucleoporin 153kDa	1.01575	0.957644	0.9891692
9973	CCS	copper chaperone for superoxide dismutase	0.97772	1.015068	1.0697875
9975	NR1D2	nuclear receptor subfamily 1, group D, member 2	0.98199	0.936801	0.9673013
9976	CLEC2B	C-type lectin domain family 2, member B	0.91667	0.732091	0.9490417
9978	RBX1	ring-box 1, E3 ubiquitin protein ligase	0.9704	1.024627	0.9484149
9980	DOPEY2	dopey family member 2	0.98673	0.899083	0.9946634
9982	FGFBP1	fibroblast growth factor binding protein 1	1.02977	0.98498	0.9259
9984	THOC1	THO complex 1	0.93527	0.941077	1.0322472
9985	REC8	REC8 homolog (yeast)	0.97427	1.07877	0.9623724

9986	RCE1	RCE1 homolog, prenyl protein peptidase (S. cerevisiae)	0.98859	0.981347	1.027208
9987	HNRPDL	heterogeneous nuclear ribonucleoprotein D-like	1.01463	0.960663	1.0903666
9988	DMTF1	cyclin D binding myb-like transcription factor 1	1.03377	0.970959	1.0127984
9989	PPP4R1	protein phosphatase 4, regulatory subunit 1	0.96168	1.025432	1.0671809
9990	SLC12A6	solute carrier family 12 (potassium/chloride transporters), member 6	0.94472	0.970348	1.010904
9991	ROD1	ROD1 regulator of differentiation 1 (S. pombe)	1.00394	1.003699	1.0848374
9992	KCNE2	potassium voltage-gated channel, Isk-related family, member 2	1.02815	0.988226	1.0104609
9993	DGCR2	DiGeorge syndrome critical region gene 2	0.95858	0.966006	0.9434492
9994	CASP8AP2	caspase 8 associated protein 2	0.97742	0.909376	0.9803238
9997	SCO2	SCO cytochrome oxidase deficient homolog 2 (yeast)	1.01346	0.954774	0.9851945
10000	AKT3	v-akt murine thymoma viral oncogene homolog 3 (protein kinase B, gamma)	1.00657	0.989914	0.9070723
10001	MED6	mediator complex subunit 6	0.92583	0.955987	1.0618973
10002	NR2E3	nuclear receptor subfamily 2, group E, member 3	0.98725	1.021995	0.9784166
10003	NAALAD2	N-acetylated alpha-linked acidic dipeptidase 2	1.00121	0.999173	1.0029104
10004	NAALADL1	N-acetylated alpha-linked acidic dipeptidase-like 1	0.97397	0.959674	1.0044321
10005	ACOT8	acyl-CoA thioesterase 8	0.96627	0.984928	1.0513129
10006	ABI1	abl-interactor 1	0.9444	1.035735	1.0507593
10007	GNPDA1	glucosamine-6-phosphate deaminase 1	1.00697	0.972989	1.0510848
10008	KCNE3	potassium voltage-gated channel, Isk-related family, member 3	0.98088	0.937846	0.9874162
10009	ZBTB33	zinc finger and BTB domain containing 33	0.9501	0.937438	1.0684961
10010	TANK	TRAF family member-associated NFKB activator	0.99846	1.054486	1.0162053
10011	SRA1	steroid receptor RNA activator 1	0.99136	0.951599	0.9949989
10013	HDAC6	histone deacetylase 6	0.97158	0.943012	1.067969
10014	HDAC5	histone deacetylase 5	0.9574	1.018546	0.9575835
10015	PDCD6IP	programmed cell death 6 interacting protein	1.00529	0.986932	1.0834546
10016	PDCD6	programmed cell death 6	1.02265	0.996536	1.0652703
10017	BCL2L10	BCL2-like 10 (apoptosis facilitator)	0.97969	0.982001	0.9955114
10018	BCL2L11	BCL2-like 11 (apoptosis facilitator)	0.9943	1.034047	0.9759961
10019	SH2B3	SH2B adaptor protein 3	1.00421	0.9738	0.9456795
10020	GNE	glucosamine (UDP-N-acetyl)-2-epimerase/N-acetylmannosamine kinase	0.90893	0.970602	1.084245
10021	HCN4	hyperpolarization activated cyclic nucleotide-gated potassium channel 4	0.97935	0.995896	1.0445871
10022	INSL5	insulin-like 5	1.01029	1.002995	1.0218018
10023	FRAT1	frequently rearranged in advanced T-cell lymphomas	0.93068	0.954726	0.9483939

10024	TROAP	trophinin associated protein (tastin)	1.00017	0.971177	1.0136295
10025	MED16	mediator complex subunit 16	0.96082	0.966256	1.0411939
10026	PIGK	phosphatidylinositol glycan anchor biosynthesis, class K	0.92756	0.91243	1.0285476
10029	CASKP1	calcium/calmodulin-dependent serine protein kinase (MAGUK family) pseudogene 1	0.98371	0.973451	1.0525197
10031	SHROOM2I	shroom family member 2 pseudogene 1	1.00169	0.997042	0.9804185
10034	ARSDP1	arylsulfatase D pseudogene 1	0.98981	1.038122	0.987802
10036	CHAF1A	chromatin assembly factor 1, subunit A (p150)	0.97495	0.982069	1.0277543
10038	PARP2	poly (ADP-ribose) polymerase 2	0.95867	0.941367	0.9965657
10039	PARP3	poly (ADP-ribose) polymerase family, member 3	0.98273	0.949977	1.0559792
10040	TOM1L1	target of myb1 (chicken)-like 1	0.97645	0.972597	0.9904542
10042	HMGXB4	HMG box domain containing 4	0.93519	0.973764	1.0829044
10043	TOM1	target of myb1 (chicken)	0.95683	1.006108	1.0122395
10044	SH2D3C	SH2 domain containing 3C	0.98628	1.024554	0.9583177
10045	SH2D3A	SH2 domain containing 3A	0.95593	1.07295	0.9892951
10046	MAMLD1	mastermind-like domain containing 1	0.98195	1.013628	0.9966568
10047	CST8	cystatin 8 (cystatin-related epididymal specific)	0.99311	1.038462	1.0145188
10048	RANBP9	RAN binding protein 9	0.98708	0.999366	0.9358952
10049	DNAJB6	DnaJ (Hsp40) homolog, subfamily B, member 6	1.0469	0.979358	0.9607716
10050	SLC17A4	solute carrier family 17 (sodium phosphate), member 4	0.99342	1.024987	1.0032374
10051	SMC4	structural maintenance of chromosomes 4	0.98762	1.002217	1.0408633
10052	GJC1	gap junction protein, gamma 1, 45kDa	0.98957	1.023569	1.0004598
10053	AP1M2	adaptor-related protein complex 1, mu 2 subunit	0.97761	1.010126	1.0014468
10054	UBA2	ubiquitin-like modifier activating enzyme 2	0.94219	0.935522	0.9549035
10055	SAE1	SUMO1 activating enzyme subunit 1	0.96744	0.998656	1.0165108
10056	FARSB	phenylalanyl-tRNA synthetase, beta subunit	1.00077	0.894271	1.0120635
10057	ABCC5	ATP-binding cassette, sub-family C (CFTR/MRP), member 5	1.01148	0.944679	1.0034417
10058	ABCB6	ATP-binding cassette, sub-family B (MDR/TAP), member 6	1.00134	1.011729	1.0077067
10059	DNM1L	dynamin 1-like	0.94084	0.95357	1.0460361
10060	ABCC9	ATP-binding cassette, sub-family C (CFTR/MRP), member 9	1.01201	1.00733	1.0069626
10061	ABCF2	ATP-binding cassette, sub-family F (GCN20), member 2	0.96602	1.002231	1.0447244
10062	NR1H3	nuclear receptor subfamily 1, group H, member 3	0.96337	0.973045	1.062733
10063	COX17	COX17 cytochrome c oxidase assembly homolog (S. cerevisiae)	0.99694	0.981172	1.0216022
10064	VDAC1P2	voltage-dependent anion channel 1 pseudogene 2	0.97987	0.916322	1.0686523

10066	SCAMP2	secretory carrier membrane protein 2	0.96394	0.899052	1.0683056
10067	SCAMP3	secretory carrier membrane protein 3	0.96689	0.992547	1.0355157
10069	RWDD2B	RWD domain containing 2B	0.94686	1.010613	1.0442105
10071	MUC12	mucin 12, cell surface associated	1.00525	1.012662	1.0061066
10072	DPP3	dipeptidyl-peptidase 3	0.95183	0.957447	1.1258049
10073	SNUPN	snurportin 1	0.93888	0.973257	1.0067326
10075	HUWE1	HECT, UBA and WWE domain containing 1	0.96615	1.121448	1.1049153
10076	PTPRU	protein tyrosine phosphatase, receptor type, U	1	0.997508	1.0259219
10077	TSPAN32	tetraspanin 32	0.96068	0.998518	0.9899946
10078	TSSC4	tumor suppressing subtransferable candidate 4	0.93905	1.035678	1.0330871
10081	PDCD7	programmed cell death 7	0.94018	0.974702	1.0862834
10082	GPC6	glypican 6	1.00092	1.003843	0.9984914
10083	USH1C	Usher syndrome 1C (autosomal recessive, severe)	1.00205	0.999355	1.0148882
10084	PQBP1	polyglutamine binding protein 1	0.9395	0.9687	1.0972471
10085	EDIL3	EGF-like repeats and discoidin I-like domains 3	1.01271	1.014662	1.0171808
10086	HHLA1	HERV-H LTR-associating 1	1.00748	1.009678	0.9979003
10087	COL4A3BP	collagen, type IV, alpha 3 (Goodpasture antigen) binding protein	1.00922	0.951532	1.0352189
10089	KCNK7	potassium channel, subfamily K, member 7	0.97959	0.976368	0.9920954
10090	UST	uronyl-2-sulfotransferase	1.00965	1.023429	0.9954777
10092	ARPC5	actin related protein 2/3 complex, subunit 5, 16kDa	1.00997	0.968718	1.0790364
10093	ARPC4	actin related protein 2/3 complex, subunit 4, 20kDa	1.02505	0.960866	1.0399922
10094	ARPC3	actin related protein 2/3 complex, subunit 3, 21kDa	0.97041	0.99452	1.0100494
10095	ARPC1B	actin related protein 2/3 complex, subunit 1B, 41kDa	1.01393	0.97706	1.0145243
10096	ACTR3	ARP3 actin-related protein 3 homolog (yeast)	1.03568	1.049178	0.9749193
10097	ACTR2	ARP2 actin-related protein 2 homolog (yeast)	1.07276	0.984467	0.9616763
10098	TSPAN5	tetraspanin 5	1.01737	0.954977	0.9955374
10099	TSPAN3	tetraspanin 3	0.95532	1.073242	1.0375189
10100	TSPAN2	tetraspanin 2	0.9844	0.940319	1.0412801
10101	NUBP2	nucleotide binding protein 2	0.94665	1.010218	1.0687185
10102	TSFM	Ts translation elongation factor, mitochondrial	0.92985	0.87295	1.1160338
10103	TSPAN1	tetraspanin 1	1.00081	1.033119	1.02326
10105	PPIF	peptidylprolyl isomerase F	0.97958	1.0308	0.9134559
10106	CTDSP2	CTD (carboxy-terminal domain, RNA polymerase II, polypeptide A) small phosphatase 2	0.97174	0.916555	1.0206114

10107	TRIM10	tripartite motif containing 10	1.01553	0.998437	1.0143501
10109	ARPC2	actin related protein 2/3 complex, subunit 2, 34kDa	1.0354	1.015583	0.9299505
10110	SGK2	serum/glucocorticoid regulated kinase 2	1.00807	0.999728	0.9929571
10111	RAD50	RAD50 homolog (S. cerevisiae)	1.00194	0.914345	0.9797831
10112	KIF20A	kinesin family member 20A	1.02175	1.003764	1.0178675
10113	PREB	prolactin regulatory element binding	0.99701	0.944853	0.9975849
10114	HIPK3	homeodomain interacting protein kinase 3	0.9727	0.961173	1.0992951
10116	FEM1B	fem-1 homolog b (C. elegans)	0.99026	1.006651	1.0863416
10117	ENAM	enamelin	1.01068	1.001324	0.9712073
10120	ACTR1B	ARP1 actin-related protein 1 homolog B, centractin beta (yeast)	0.97129	0.977644	0.9363991
10121	ACTR1A	ARP1 actin-related protein 1 homolog A, centractin alpha (yeast)	0.91069	1.046529	1.0452748
10123	ARL4C	ADP-ribosylation factor-like 4C	1.00522	0.985807	0.9932654
10124	ARL4A	ADP-ribosylation factor-like 4A	0.98722	0.966307	0.9543715
10125	RASGRP1	RAS guanyl releasing protein 1 (calcium and DAG-regulated)	0.9452	1.089387	1.001796
10126	DNAL4	dynein, axonemal, light chain 4	0.98309	1.007395	1.0233449
10127	ZNF263	zinc finger protein 263	0.9133	0.946395	1.0509985
10128	LRPPRC	leucine-rich PPR-motif containing	0.98475	0.86331	0.9917416
10129	FRY	furry homolog (Drosophila)	0.96946	0.997985	1.0407304
10130	PDIA6	protein disulfide isomerase family A, member 6	1.02989	0.999821	1.170789
10131	TRAP1	TNF receptor-associated protein 1	0.93162	0.978772	0.9978138
10133	OPTN	optineurin	0.96128	1.034475	1.0079785
10134	BCAP31	B-cell receptor-associated protein 31	0.96925	0.992507	1.1153384
10135	NAMPT	nicotinamide phosphoribosyltransferase	1.0992	0.954477	0.8856984
10138	YAF2	YY1 associated factor 2	0.95384	1.02429	0.9994604
10139	ARFRP1	ADP-ribosylation factor related protein 1	0.98707	1.014009	1.0026265
10140	TOB1	transducer of ERBB2, 1	0.96098	0.795325	1.0206292
10141	C4orf6	chromosome 4 open reading frame 6	1.05282	1.045826	0.9813928
10142	AKAP9	A kinase (PRKA) anchor protein (yotiao) 9	0.99604	1.005511	0.9392429
10143	CLEC3A	C-type lectin domain family 3, member A	0.99267	0.985192	1.0119032
10144	FAM13A	family with sequence similarity 13, member A	1.00341	0.939802	1.0927461
10146	G3BP1	GTPase activating protein (SH3 domain) binding protein 1	1.00073	0.934794	0.9749866
10147	SUGP2	SURP and G patch domain containing 2	0.96065	1.038578	1.0389273
10148	EBI3	Epstein-Barr virus induced 3	0.9932	0.997548	1.0135292

10149	GPR64	G protein-coupled receptor 64	1	1.010773	0.9974677
10150	MBNL2	muscleblind-like 2 (Drosophila)	0.97405	1.026769	1.0477563
10151	HNRNPA3P	heterogeneous nuclear ribonucleoprotein A3 pseudogene 1	1.00198	0.992566	1.0351341
10152	ABI2	abl-interactor 2	0.98666	0.998973	1.0122624
10153	CEBPZ	CCAAT/enhancer binding protein (C/EBP), zeta	1.00772	0.911637	0.9227569
10154	PLXNC1	plexin C1	0.99105	1.035975	0.9638315
10155	TRIM28	tripartite motif containing 28	0.98494	1.001888	1.0412701
10157	AASS	aminoadipate-semialdehyde synthase	1.01155	0.932155	0.9535151
10159	ATP6AP2	ATPase, H+ transporting, lysosomal accessory protein 2	0.97326	0.999555	0.9658313
10160	FARP1	FERM, RhoGEF (ARHGEF) and pleckstrin domain protein 1 (chondrocyte-derived)	1.01147	0.988661	1.0048847
10161	LPAR6	lysophosphatidic acid receptor 6	0.97724	0.856264	0.9855354
10162	LPCAT3	lysophosphatidylcholine acyltransferase 3	0.96411	0.933786	0.9383318
10163	WASF2	WAS protein family, member 2	1.02706	1.012362	0.9729178
10164	CHST4	carbohydrate (N-acetylglucosamine 6-O) sulfotransferase 4	1.00241	0.930307	1.007471
10165	SLC25A13	solute carrier family 25, member 13 (citrin)	1.00934	0.990677	0.9911216
10166	SLC25A15	solute carrier family 25 (mitochondrial carrier; ornithine transporter) member 15	0.96891	0.95576	1.0593615
10168	ZNF197	zinc finger protein 197	0.96893	0.937438	1.0485513
10169	SERF2	small EDRK-rich factor 2	0.95189	0.930201	0.9699637
10170	DHRS9	dehydrogenase/reductase (SDR family) member 9	1.00731	0.974108	0.9968393
10171	RCL1	RNA terminal phosphate cyclase-like 1	0.98341	0.917331	1.0114133
10172	ZNF256	zinc finger protein 256	0.95881	0.963164	0.9623391
10174	SORBS3	sorbin and SH3 domain containing 3	0.96501	0.988528	0.9839714
10175	CNIH	cornichon homolog (Drosophila)	0.91221	0.97707	1.1430857
10178	ODZ1	odz, odd Oz/ten-m homolog 1 (Drosophila)	0.99301	0.995011	0.9904853
10179	RBM7	RNA binding motif protein 7	0.93173	0.92782	0.9114702
10180	RBM6	RNA binding motif protein 6	0.98773	0.997212	1.016177
10181	RBM5	RNA binding motif protein 5	0.98275	0.968261	0.9957465
10184	LHFPL2	lipoma HMGIC fusion partner-like 2	0.99668	0.983029	1.0176692
10186	LHFP	lipoma HMGIC fusion partner	1.00764	0.989428	0.9864343
10188	TNK2	tyrosine kinase, non-receptor, 2	1.00418	0.993378	0.999346
10189	THOC4	THO complex 4	0.9346	1.029669	1.0334344
10190	TXNDC9	thioredoxin domain containing 9	0.96284	0.997793	1.0876854
10193	RNF41	ring finger protein 41	0.93118	0.999729	1.0800643

10194	TSHZ1	teashirt zinc finger homeobox 1	0.95237	1.010976	0.9731218
10195	ALG3	asparagine-linked glycosylation 3, alpha-1,3- mannosyltransferase homolog (S. cerevisiae)	0.9915	0.941407	1.0726427
10196	PRMT3	protein arginine methyltransferase 3	0.94825	0.943477	1.0271235
10197	PSME3	proteasome (prosome, macropain) activator subunit 3 (PA28 gamma; Ki)	0.93486	0.958985	1.1331956
10198	MPHOSPH9	M-phase phosphoprotein 9	0.94909	0.953116	1.0306348
10199	MPHOSPH1	M-phase phosphoprotein 10 (U3 small nucleolar ribonucleoprotein)	0.97634	0.906533	1.0624073
10200	MPHOSPH6	M-phase phosphoprotein 6	0.98602	0.986479	0.9571797
10201	NME6	non-metastatic cells 6, protein expressed in (nucleoside-diphosphate kinase)	0.98511	0.998164	0.9991526
10202	DHRS2	dehydrogenase/reductase (SDR family) member 2	0.99854	0.982237	1.018174
10203	CALCRL	calcitonin receptor-like	0.98162	1.044092	1.03867
10204	NUTF2	nuclear transport factor 2	0.96328	0.943996	1.0891487
10205	MPZL2	myelin protein zero-like 2	0.99696	0.990218	0.9183151
10206	TRIM13	tripartite motif containing 13	0.97148	0.970333	0.9575979
10207	INADL	InaD-like (Drosophila)	0.97402	0.93846	0.9672285
10208	USPL1	ubiquitin specific peptidase like 1	0.93535	0.953387	1.0039494
10209	EIF1	eukaryotic translation initiation factor 1	0.97348	0.913843	0.9205074
10210	TOPORS	topoisomerase I binding, arginine/serine-rich, E3 ubiquitin protein ligase	0.91673	0.933772	0.9878489
10211	FLOT1	flotillin 1	0.9756	1.032906	1.0279212
10212	DDX39A	DEAD (Asp-Glu-Ala-Asp) box polypeptide 39A	0.9398	1.082343	1.0197305
10213	PSMD14	proteasome (prosome, macropain) 26S subunit, non-ATPase, 14	0.98917	0.972824	1.0383269
10214	SSX3	synovial sarcoma, X breakpoint 3	1.01145	1.02111	1.0372724
10216	PRG4	proteoglycan 4	1.00777	1.015561	1.0078917
10217	CTDSPL	CTD (carboxy-terminal domain, RNA polymerase II, polypeptide A) small phosphatase-like	1.00976	0.989065	0.9722905
10219	KLRG1	killer cell lectin-like receptor subfamily G, member 1	0.92836	1.129049	0.9391281
10220	GDF11	growth differentiation factor 11	0.97203	0.988348	1.0690153
10223	GPA33	glycoprotein A33 (transmembrane)	0.99526	0.947883	0.9573747
10224	ZNF443	zinc finger protein 443	0.91478	0.906324	1.0042018
10225	CD96	CD96 molecule	0.97442	1.009905	0.9653411
10226	PLIN3	perilipin 3	0.98877	0.953149	1.0343569
10227	MFSD10	major facilitator superfamily domain containing 10	1.00605	1.088504	1.0595285
10228	STX6	syntaxin 6	0.9988	1.041524	1.0452883
10229	COQ7	coenzyme Q7 homolog, ubiquinone (yeast)	0.94025	0.993708	1.0000833
10230	NBR2	neighbor of BRCA1 gene 2 (non-protein coding)	0.97202	0.993709	0.9765178

10231	RCAN2	regulator of calcineurin 2	1.01157	1.012268	1.0003164
10232	MSLN	mesothelin	1.00737	1.004228	1.0677014
10233	LRRC23	leucine rich repeat containing 23	0.97449	0.959945	1.0138229
10234	LRRC17	leucine rich repeat containing 17	1.00659	1.026879	1.0127967
10235	RASGRP2	RAS guanyl releasing protein 2 (calcium and DAG-regulated)	0.93201	0.936607	0.9628235
10236	HNRNPR	heterogeneous nuclear ribonucleoprotein R	1.00802	1.006487	1.0673103
10237	SLC35B1	solute carrier family 35, member B1	0.94207	0.976173	0.9779708
10238	DCAF7	DDB1 and CUL4 associated factor 7	0.9734	0.959472	1.0099439
10239	AP3S2	adaptor-related protein complex 3, sigma 2 subunit	0.97081	0.986155	1.0072318
10240	MRPS31	mitochondrial ribosomal protein S31	0.9605	0.922762	1.0592452
10241	CALCOCO2	calcium binding and coiled-coil domain 2	0.97331	1.021881	0.9938555
10242	KCNMB2	potassium large conductance calcium-activated channel, subfamily M, beta member 2	1.0164	1.015467	1.0099285
10243	GPHN	gephyrin	0.97128	0.952658	1.0238259
10244	RABEPK	Rab9 effector protein with kelch motifs	0.94914	0.998639	0.982653
10245	TIMM17B	translocase of inner mitochondrial membrane 17 homolog B (yeast)	0.96578	0.962436	0.9698446
10246	SLC17A2	solute carrier family 17 (sodium phosphate), member 2	0.99023	1.014014	1.0117658
10247	HRSP12	heat-responsive protein 12	0.95676	0.927955	1.0017415
10248	POP7	processing of precursor 7, ribonuclease P/MRP subunit (S. cerevisiae)	0.96781	0.871708	1.025401
10249	GLYAT	glycine-N-acyltransferase	1.02001	1.002866	1.0230159
10250	SRRM1	serine/arginine repetitive matrix 1	1.04378	0.964201	0.9384715
10252	SPRY1	sprouty homolog 1, antagonist of FGF signaling (Drosophila)	0.96148	0.971689	1.0193831
10254	STAM2	signal transducing adaptor molecule (SH3 domain and ITAM motif) 2	0.98625	0.920301	1.005807
10255	HCG9	HLA complex group 9 (non-protein coding)	0.96916	1.054532	1.0107438
10256	CNKSR1	connector enhancer of kinase suppressor of Ras 1	1.00722	0.968682	0.9862234
10257	ABCC4	ATP-binding cassette, sub-family C (CFTR/MRP), member 4	0.96113	0.972516	1.0305688
10260	DENND4A	DENN/MADD domain containing 4A	0.91799	1.167196	0.9657175
10261	IGSF6	immunoglobulin superfamily, member 6	0.99482	0.952473	0.9304294
10262	SF3B4	splicing factor 3b, subunit 4, 49kDa	1.00381	0.961093	1.0525811
10263	CDK2AP2	cyclin-dependent kinase 2 associated protein 2	0.9746	0.980921	1.0605445
10265	IRX5	iroquois homeobox 5	0.97713	0.971744	0.9974405
10266	RAMP2	receptor (G protein-coupled) activity modifying protein 2	1.00835	1.006662	0.9891087
10267	RAMP1	receptor (G protein-coupled) activity modifying protein 1	1.00467	1.021883	1.0150549
10268	RAMP3	receptor (G protein-coupled) activity modifying protein 3	0.98897	1.010975	0.9969403

10269	ZMPSTE24	zinc metallopeptidase (STE24 homolog, S. cerevisiae)	1.00972	0.898545	1.04459
10270	AKAP8	A kinase (PRKA) anchor protein 8	0.964	0.96505	1.0062575
10272	FSTL3	follistatin-like 3 (secreted glycoprotein)	0.99387	1.044269	1.1014866
10273	STUB1	STIP1 homology and U-box containing protein 1, E3 ubiquitin protein ligase	0.95218	1.01554	1.064796
10274	STAG1	stromal antigen 1	0.98247	1.032873	0.9202431
10277	UBE4B	ubiquitination factor E4B	0.99138	1.03008	1.0193209
10278	EFS	embryonal Fyn-associated substrate	0.99633	0.991431	0.9965106
10279	PRSS16	protease, serine, 16 (thymus)	1.00077	1.016243	1.0289089
10280	SIGMAR1	sigma non-opioid intracellular receptor 1	0.97867	0.905408	1.1199193
10281	DSCR4	Down syndrome critical region gene 4	0.99144	1.044727	1.0167553
10282	BET1	blocked early in transport 1 homolog (S. cerevisiae)	0.99246	0.944568	0.9970999
10283	CWC27	CWC27 spliceosome-associated protein homolog (S. cerevisiae)	0.99881	0.925034	1.0217308
10284	SAP18	Sin3A-associated protein, 18kDa	0.92552	0.958041	0.9941204
10285	SMNDC1	survival motor neuron domain containing 1	0.98528	0.955165	0.971347
10286	BCAS2	breast carcinoma amplified sequence 2	1.00603	0.905153	0.9349188
10287	RGS19	regulator of G-protein signaling 19	0.97778	0.907566	1.0799499
10288	LILRB2	leukocyte immunoglobulin-like receptor, subfamily B (with TM and ITIM domains), member 2	1.00883	0.886945	0.8742027
10289	EIF1B	eukaryotic translation initiation factor 1B	0.97769	1.049388	0.9320166
10290	SPEG	SPEG complex locus	0.99536	1.01777	1.024701
10291	SF3A1	splicing factor 3a, subunit 1, 120kDa	0.92611	1.011968	0.9615276
10293	TRAIP	TRAF interacting protein	1.01317	0.996349	1.0084192
10294	DNAJA2	DnaJ (Hsp40) homolog, subfamily A, member 2	0.93673	0.952466	0.991634
10295	BCKDK	branched chain ketoacid dehydrogenase kinase	0.96117	0.967028	1.0281846
10296	MAEA	macrophage erythroblast attacher	1.0322	0.990438	0.9358341
10297	APC2	adenomatosis polyposis coli 2	0.99433	1.005544	1.0670761
10298	PAK4	p21 protein (Cdc42/Rac)-activated kinase 4	0.98501	1.010421	1.0724353
10299	6-Mar	membrane-associated ring finger (C3HC4) 6	1.03123	0.912511	0.9980019
10300	KATNB1	katanin p80 (WD repeat containing) subunit B 1	0.96558	0.970277	1.0126421
10301	DLEU1	deleted in lymphocytic leukemia 1 (non-protein coding)	0.96556	1.032137	1.0581801
10302	SNAPC5	small nuclear RNA activating complex, polypeptide 5, 19kDa	0.93152	0.945671	1.0279879
10307	APBB3	amyloid beta (A4) precursor protein-binding, family B, member 3	1.00531	0.98	0.9912012
10308	ZNF267	zinc finger protein 267	0.97361	0.982292	0.9524086
10309	CCNO	cyclin O	0.98484	1.030443	1.0562767

10311	DSCR3	Down syndrome critical region gene 3	0.98434	0.978433	1.0026481
10312	TCIRG1	T-cell, immune regulator 1, ATPase, H+ transporting, lysosomal V0 subunit A3	1.01091	1.063767	0.9732662
10313	RTN3	reticulon 3	0.98308	0.977141	1.0464985
10314	LANCL1	LanC lantibiotic synthetase component C-like 1 (bacterial)	0.97979	0.922733	0.9846541
10316	NMUR1	neuromedin U receptor 1	1.01871	0.983645	0.9650242
10317	B3GALT5	UDP-Gal:betaGlcNAc beta 1,3-galactosyltransferase, polypeptide 5	1.01469	0.966333	0.9854829
10318	TNIP1	TNFAIP3 interacting protein 1	1.01255	1.016099	1.0307445
10319	LAMC3	laminin, gamma 3	0.9893	0.989748	1.0171781
10320	IKZF1	IKAROS family zinc finger 1 (Ikaros)	0.98862	0.981305	0.9146535
10321	CRISP3	cysteine-rich secretory protein 3	1.00844	0.983413	1.0408682
10322	SMYD5	SMYD family member 5	0.94705	0.91726	0.9620407
10324	KBTBD10	kelch repeat and BTB (POZ) domain containing 10	0.99584	0.949912	0.9912183
10325	RRAGB	Ras-related GTP binding B	0.95401	0.994402	1.0469891
10326	SIRPB1	signal-regulatory protein beta 1	1.22792	0.883734	1.068786
10327	AKR1A1	aldo-keto reductase family 1, member A1 (aldehyde reductase)	1.01086	0.9028	1.1170209
10328	COX4NB	COX4 neighbor	0.93119	1.020465	1.0032141
10329	TMEM5	transmembrane protein 5	0.96012	0.938718	0.9970086
10330	CNPY2	canopy 2 homolog (zebrafish)	0.96477	1.017197	1.1235759
10331	B3GNT3	UDP-GlcNAc:betaGal beta-1,3-N-acetylglucosaminyltransferase 3	0.99391	0.984446	0.9897902
10332	CLEC4M	C-type lectin domain family 4, member M	0.98899	0.986643	0.9849504
10333	TLR6	toll-like receptor 6	0.97193	1.002485	1.1054637
10336	PCGF3	polycomb group ring finger 3	1.00453	1.017364	0.9862823
10341	HIST1H2AP	histone cluster 1, H2a, pseudogene 5	0.95732	0.942934	1.0982266
10342	TFG	TRK-fused gene	0.9848	0.9754	1.0222402
10343	PKDREJ	polycystic kidney disease (polycystin) and REJ homolog (sperm receptor for egg jelly homolog, sea urchin)	0.99636	1.003228	1.0007422
10344	CCL26	chemokine (C-C motif) ligand 26	0.98687	0.959127	1.0099386
10345	TRDN	triadin	1.00748	1.009152	1.0110881
10346	TRIM22	tripartite motif containing 22	0.98825	0.967967	1.0859104
10347	ABCA7	ATP-binding cassette, sub-family A (ABC1), member 7	0.9647	0.911439	0.9882482
10349	ABCA10	ATP-binding cassette, sub-family A (ABC1), member 10	0.98599	0.968217	1.0022437
10350	ABCA9	ATP-binding cassette, sub-family A (ABC1), member 9	0.99336	1.002881	1.0224357
10351	ABCA8	ATP-binding cassette, sub-family A (ABC1), member 8	0.98445	1.000747	1.0019173
10352	WARS2	tryptophanyl tRNA synthetase 2, mitochondrial	0.98167	0.965142	1.0310248

10355	HMGB1P4	high mobility group box 1 pseudogene 4	0.99251	1.060883	1.0143606
10360	NPM3	nucleophosmin/nucleoplasmin 3	0.96831	0.971601	1.0082873
10361	NPM2	nucleophosmin/nucleoplasmin 2	1.00773	1.009256	1.0284771
10362	HMG20B	high mobility group 20B	0.95942	1.028209	1.0359374
10363	HMG20A	high mobility group 20A	0.9414	1.016397	1.0559609
10365	KLF2	Kruppel-like factor 2 (lung)	0.95317	0.989743	0.9072391
10367	CBARA1	calcium binding atopy-related autoantigen 1	0.96007	1.04504	1.0322745
10368	CACNG3	calcium channel, voltage-dependent, gamma subunit 3	1.0059	1.00814	0.9903499
10369	CACNG2	calcium channel, voltage-dependent, gamma subunit 2	0.99672	1.010224	1.0090901
10370	CITED2	Cbp/p300-interacting transactivator, with Glu/Asp-rich carboxy-terminal domain, 2	0.99788	0.909651	0.9793256
10371	SEMA3A	sema domain, immunoglobulin domain (Ig), short basic domain, secreted, (semaphorin) 3A	0.99663	1.011224	0.9998142
10376	TUBA1B	tubulin, alpha 1b	1.00126	1.021362	1.0048685
10379	IRF9	interferon regulatory factor 9	0.95816	0.972347	0.9914711
10380	BPNT1	3'(2'), 5'-bisphosphate nucleotidase 1	0.96815	0.965149	0.9873029
10381	TUBB3	tubulin, beta 3	0.99324	1.021368	0.985964
10382	TUBB4	tubulin, beta 4	0.97566	1.016934	1.0419289
10383	TUBB2C	tubulin, beta 2C	0.91944	0.954928	1.1258042
10384	BTN3A3	butyrophilin, subfamily 3, member A3	1.01679	0.929319	1.0619767
10385	BTN2A2	butyrophilin, subfamily 2, member A2	1.04104	0.968981	0.9592133
10388	SYCP2	synaptonemal complex protein 2	0.98178	0.992342	0.968271
10389	SCML2	sex comb on midleg-like 2 (Drosophila)	0.99065	0.980526	0.990128
10390	CEPT1	choline/ethanolamine phosphotransferase 1	1.01062	0.983078	1.0741439
10391	CORO2B	coronin, actin binding protein, 2B	1.0039	1.01252	1.0047494
10392	NOD1	nucleotide-binding oligomerization domain containing 1	0.98589	0.960802	1.0063583
10393	ANAPC10	anaphase promoting complex subunit 10	1.0117	0.925161	0.9973499
10394	PRG3	proteoglycan 3	0.98505	1.015898	1.0201193
10395	DLC1	deleted in liver cancer 1	0.99926	0.99972	0.9997132
10396	ATP8A1	ATPase, aminophospholipid transporter (APLT), class I, type 8A, member 1	0.99326	1.055556	1.0733095
10397	NDRG1	N-myc downstream regulated 1	0.9649	1.002383	0.9802485
10398	MYL9	myosin, light chain 9, regulatory	0.99334	1.036652	0.9568507
10399	GNB2L1	guanine nucleotide binding protein (G protein), beta polypeptide 2-like 1	1.0013	0.928317	1.0191344
10400	PEMT	phosphatidylethanolamine N-methyltransferase	0.98792	0.972765	1.034128
10401	PIAS3	protein inhibitor of activated STAT, 3	1.01381	0.945198	0.9592944

10402	ST3GAL6	ST3 beta-galactoside alpha-2,3-sialyltransferase 6	1.00179	1.038818	0.9629836
10403	NDC80	NDC80 homolog, kinetochore complex component (S. cerevisiae)	0.96517	0.977836	1.0171197
10404	PGCP	plasma glutamate carboxypeptidase	1.003	0.943861	0.9898809
10406	WFDC2	WAP four-disulfide core domain 2	1.0061	1.006096	1.026174
10409	BASP1	brain abundant, membrane attached signal protein 1	1.01068	1.044897	0.9618119
10410	IFITM3	interferon induced transmembrane protein 3	0.95491	1.037246	1.0331334
10411	RAPGEF3	Rap guanine nucleotide exchange factor (GEF) 3	0.99585	0.992858	0.9932723
10412	NSA2	NSA2 ribosome biogenesis homolog (S. cerevisiae)	1.0029	0.975547	0.9821333
10413	YAP1	Yes-associated protein 1	1.0094	1.01322	0.9865873
10415	SNAI1P1	snail homolog 1 (Drosophila) pseudogene 1	1.00369	1.022112	1.0392966
10417	SPON2	spondin 2, extracellular matrix protein	1.05398	0.967685	0.9221351
10418	SPON1	spondin 1, extracellular matrix protein	0.98283	0.990184	0.9971666
10419	PRMT5	protein arginine methyltransferase 5	0.93071	0.868278	1.042627
10420	TESK2	testis-specific kinase 2	1.00393	1.033212	0.9903122
10421	CD2BP2	CD2 (cytoplasmic tail) binding protein 2	0.92153	1.030477	1.1133966
10422	UBAC1	UBA domain containing 1	0.97137	0.957155	1.0983105
10423	CDIPT	CDP-diacylglycerol--inositol 3-phosphatidyltransferase	0.95425	0.884251	1.0599084
10424	PGRMC2	progesterone receptor membrane component 2	0.99506	1.024273	0.9644837
10425	ARIH2	ariadne homolog 2 (Drosophila)	0.98787	0.976934	1.0146029
10426	TUBGCP3	tubulin, gamma complex associated protein 3	0.97136	0.924598	1.0288043
10427	SEC24B	SEC24 family, member B (S. cerevisiae)	1.01655	1.014066	0.9667643
10428	CFDP1	craniofacial development protein 1	0.99273	1.012896	0.9676496
10430	TMEM147	transmembrane protein 147	0.95728	0.967101	1.0226518
10431	[No Symbo	[No Name]	0.96522	0.894296	1.045528
10432	RBM14	RNA binding motif protein 14	0.94401	0.926412	1.0040549
10434	LYPLA1	lysophospholipase I	0.98543	0.950462	1.0568527
10435	CDC42EP2	CDC42 effector protein (Rho GTPase binding) 2	0.9812	1.008097	1.0073264
10436	EMG1	EMG1 nucleolar protein homolog (S. cerevisiae)	0.91111	0.98898	1.017649
10437	IFI30	interferon, gamma-inducible protein 30	1.02316	0.975639	1.0884852
10438	C1D	C1D nuclear receptor corepressor	0.97881	0.935086	0.9622356
10439	OLFM1	olfactomedin 1	0.9782	1.016286	0.9628567
10440	TIMM17A	translocase of inner mitochondrial membrane 17 homolog A (yeast)	0.99138	0.933153	1.0523884
10443	N4BP2L2	NEDD4 binding protein 2-like 2	0.95462	1.009414	0.9113011

10444	ZER1	zer-1 homolog (C. elegans)	0.92653	0.994715	0.9878855
10445	MCRS1	microspherule protein 1	0.94202	0.92889	1.0107635
10446	LRRN2	leucine rich repeat neuronal 2	1.00899	0.98209	0.9979843
10447	FAM3C	family with sequence similarity 3, member C	1.01511	1.018493	1.0363631
10449	ACAA2	acetyl-CoA acyltransferase 2	0.93362	0.962787	1.097926
10450	PPIE	peptidylprolyl isomerase E (cyclophilin E)	1.00837	0.939168	1.0658394
10451	VAV3	vav 3 guanine nucleotide exchange factor	1.004	1.007232	0.9990025
10452	TOMM40	translocase of outer mitochondrial membrane 40 homolog (yeast)	0.99553	1	1.0026049
10454	TAB1	TGF-beta activated kinase 1/MAP3K7 binding protein 1	0.96846	1.007002	0.9965885
10455	ECI2	enoyl-CoA delta isomerase 2	0.97451	0.940427	0.9362962
10456	HAX1	HCLS1 associated protein X-1	0.96716	0.999638	0.9724624
10457	GPNMB	glycoprotein (transmembrane) nmb	1.00912	0.997085	1.0242272
10458	BAIAP2	BAI1-associated protein 2	0.9989	0.985732	0.9953468
10459	MAD2L2	MAD2 mitotic arrest deficient-like 2 (yeast)	0.97027	0.956883	1.0569391
10460	TACC3	transforming, acidic coiled-coil containing protein 3	0.97604	0.933385	1.0042518
10461	MERTK	c-mer proto-oncogene tyrosine kinase	1.05085	0.945071	1.0625615
10462	CLEC10A	C-type lectin domain family 10, member A	0.98466	0.953924	0.9553647
10463	SLC30A9	solute carrier family 30 (zinc transporter), member 9	0.97836	0.987702	1.0308668
10464	PIBF1	progesterone immunomodulatory binding factor 1	0.9626	0.966626	1.0545695
10465	PPIH	peptidylprolyl isomerase H (cyclophilin H)	1.02107	0.905453	1.0406929
10466	COG5	component of oligomeric golgi complex 5	0.9826	0.975829	0.9283937
10467	ZNHIT1	zinc finger, HIT-type containing 1	0.99649	0.990354	1.0802326
10468	FST	follistatin	0.99931	0.971994	1.0008636
10469	TIMM44	translocase of inner mitochondrial membrane 44 homolog (yeast)	0.98495	1.013555	1.0057745
10471	PFDN6	prefoldin subunit 6	0.95944	0.986912	0.9882919
10472	ZNF238	zinc finger protein 238	0.98549	0.852154	0.9375633
10473	HMGN4	high mobility group nucleosomal binding domain 4	0.9843	0.87722	0.9812062
10474	TADA3	transcriptional adaptor 3	0.96035	0.977784	1.0533544
10475	TRIM38	tripartite motif containing 38	1.04349	1.042218	0.9110299
10476	ATP5H	ATP synthase, H+ transporting, mitochondrial Fo complex, subunit d	0.97545	0.983838	0.9781635
10477	UBE2E3	ubiquitin-conjugating enzyme E2E 3	1.01923	0.946857	0.9305521
10478	SLC25A17	solute carrier family 25 (mitochondrial carrier; peroxisomal membrane protein, 34kDa), member 17	0.95618	1.006625	0.9746378
10479	SLC9A6	solute carrier family 9 (sodium/hydrogen exchanger), member 6	0.94032	0.95988	1.0333288

10480	EIF3M	eukaryotic translation initiation factor 3, subunit M	0.95326	0.97424	1.0557214
10481	HOXB13	homeobox B13	1.00375	1.000351	1.011
10482	NXF1	nuclear RNA export factor 1	0.96773	1.057579	1.0306376
10483	SEC23B	Sec23 homolog B (S. cerevisiae)	0.95011	1.054188	1.0301085
10484	SEC23A	Sec23 homolog A (S. cerevisiae)	0.9415	1.02467	0.9493974
10485	C1orf61	chromosome 1 open reading frame 61	1.00935	1.005823	1.022136
10486	CAP2	CAP, adenylate cyclase-associated protein, 2 (yeast)	1.0055	0.990033	1.0056315
10487	CAP1	CAP, adenylate cyclase-associated protein 1 (yeast)	1.01775	1.014732	1.0066916
10488	CREB3	cAMP responsive element binding protein 3	0.89713	0.983258	1.001406
10489	LRRC41	leucine rich repeat containing 41	0.9844	0.937426	1.0368825
10490	VTI1B	vesicle transport through interaction with t-SNAREs homolog 1B (yeast)	0.98864	0.992753	0.9900415
10492	SYNCRIP	synaptotagmin binding, cytoplasmic RNA interacting protein	0.99452	0.939833	0.9936619
10493	VAT1	vesicle amine transport protein 1 homolog (T. californica)	0.93329	1.059673	1.0751458
10494	STK25	serine/threonine kinase 25	1.00026	0.96219	1.0287524
10495	ENOX2	ecto-NOX disulfide-thiol exchanger 2	0.95607	0.954354	1.0291121
10497	UNC13B	unc-13 homolog B (C. elegans)	0.98915	0.98431	1.0055286
10498	CARM1	coactivator-associated arginine methyltransferase 1	0.94923	0.91617	1.1054849
10499	NCOA2	nuclear receptor coactivator 2	0.9891	1.019276	0.9862321
10500	SEMA6C	sema domain, transmembrane domain (TM), and cytoplasmic domain, (semaphorin) 6C	1.0122	0.99507	1.0437868
10501	SEMA6B	sema domain, transmembrane domain (TM), and cytoplasmic domain, (semaphorin) 6B	0.9649	1.255063	0.9613708
10505	SEMA4F	sema domain, immunoglobulin domain (Ig), transmembrane domain (TM) and short cytoplasmic domain, (se	0.98499	0.9778	0.9957078
10507	SEMA4D	sema domain, immunoglobulin domain (Ig), transmembrane domain (TM) and short cytoplasmic domain, (se	0.94934	1.024818	0.9430195
10509	SEMA4B	sema domain, immunoglobulin domain (Ig), transmembrane domain (TM) and short cytoplasmic domain, (se	0.95103	0.986915	0.998645
10512	SEMA3C	sema domain, immunoglobulin domain (Ig), short basic domain, secreted, (semaphorin) 3C	1.00871	0.985618	1.0067616
10513	APPBP2	amyloid beta precursor protein (cytoplasmic tail) binding protein 2	0.95702	0.995907	1.0879576
10514	MYBBP1A	MYB binding protein (P160) 1a	0.95193	0.901769	1.0046098
10516	FBLN5	fibulin 5	0.98774	0.986032	0.9913108
10517	FBXW10	F-box and WD repeat domain containing 10	0.96147	1.080459	0.9961968
10518	CIB2	calcium and integrin binding family member 2	0.99603	1.013285	1.019254
10519	CIB1	calcium and integrin binding 1 (calmyrin)	0.96708	1.048117	0.9348112
10520	ZNF211	zinc finger protein 211	0.89303	0.944912	1.0871717
10521	DDX17	DEAD (Asp-Glu-Ala-Asp) box polypeptide 17	1.00031	0.963507	1.0643231
10522	DEAF1	deformed epidermal autoregulatory factor 1 (Drosophila)	0.98077	0.96446	1.0417543

10523	CHERP	calcium homeostasis endoplasmic reticulum protein	0.94384	1.008845	0.9994586
10524	KAT5	K(lysine) acetyltransferase 5	0.92419	0.962939	1.003881
10525	HYOU1	hypoxia up-regulated 1	0.94515	0.924242	1.0640471
10526	IPO8	importin 8	0.95488	0.939014	1.0514919
10527	IPO7	importin 7	0.9349	1.079698	1.0375838
10528	NOP56	NOP56 ribonucleoprotein homolog (yeast)	0.93524	1.155832	1.009434
10529	NEBL	nebulette	0.98903	0.995142	0.9964303
10531	PITRM1	pitrilysin metallopeptidase 1	0.94117	0.949496	1.0085569
10533	ATG7	ATG7 autophagy related 7 homolog (S. cerevisiae)	0.99706	0.987294	0.900958
10534	SSSCA1	Sjogren syndrome/scleroderma autoantigen 1	0.92235	0.962736	0.958446
10535	RNASEH2A	ribonuclease H2, subunit A	0.94338	0.956908	1.0247215
10536	LEPREL2	leprecan-like 2	0.99339	0.967254	1.0130823
10537	UBD	ubiquitin D	0.9986	1.046665	0.974683
10538	BATF	basic leucine zipper transcription factor, ATF-like	0.94827	1.004283	1.0405904
10539	GLRX3	glutaredoxin 3	0.98112	0.986941	1.0341494
10540	DCTN2	dynactin 2 (p50)	0.95028	1.007152	0.982731
10541	ANP32B	acidic (leucine-rich) nuclear phosphoprotein 32 family, member B	0.95939	1.015281	1.0011905
10542	HBXIP	hepatitis B virus x interacting protein	0.99807	0.866424	0.9993714
10544	PROCR	protein C receptor, endothelial	0.98557	0.941152	0.9932421
10548	TM9SF1	transmembrane 9 superfamily member 1	0.94929	1.012293	1.1117208
10549	PRDX4	peroxiredoxin 4	0.96751	1.006127	1.0240813
10550	ARL6IP5	ADP-ribosylation-like factor 6 interacting protein 5	1.00867	0.888702	0.9787149
10551	AGR2	anterior gradient homolog 2 (Xenopus laevis)	1.01122	0.991098	1.0080909
10552	ARPC1A	actin related protein 2/3 complex, subunit 1A, 41kDa	1.03413	1.000655	0.9824266
10553	HTATIP2	HIV-1 Tat interactive protein 2, 30kDa	0.94828	0.898759	1.0672507
10554	AGPAT1	1-acylglycerol-3-phosphate O-acyltransferase 1 (lysophosphatidic acid acyltransferase, alpha)	1.02859	0.962404	0.9822759
10555	AGPAT2	1-acylglycerol-3-phosphate O-acyltransferase 2 (lysophosphatidic acid acyltransferase, beta)	0.9829	0.987836	1.062637
10556	RPP30	ribonuclease P/MRP 30kDa subunit	0.93703	0.870063	1.0910029
10557	RPP38	ribonuclease P/MRP 38kDa subunit	0.95471	0.946873	1.0750636
10558	SPTLC1	serine palmitoyltransferase, long chain base subunit 1	0.9734	0.980111	1.0825962
10559	SLC35A1	solute carrier family 35 (CMP-sialic acid transporter), member A1	0.97462	0.911474	1.0640652
10560	SLC19A2	solute carrier family 19 (thiamine transporter), member 2	0.98521	0.952632	0.9507774
10561	IFI44	interferon-induced protein 44	1.00777	0.873957	1.1041582

10562	OLFM4	olfactomedin 4	1.00673	1.001884	0.9905765
10563	CXCL13	chemokine (C-X-C motif) ligand 13	1.00867	0.977528	1.0177327
10564	ARFGEF2	ADP-ribosylation factor guanine nucleotide-exchange factor 2 (brefeldin A-inhibited)	0.95705	1.059652	0.9078139
10565	ARFGEF1	ADP-ribosylation factor guanine nucleotide-exchange factor 1(brefeldin A-inhibited)	0.95344	1.083804	0.9970122
10566	AKAP3	A kinase (PRKA) anchor protein 3	0.99065	0.98616	1.0573283
10567	RABAC1	Rab acceptor 1 (prenylated)	0.96473	1.011941	1.0469858
10568	SLC34A2	solute carrier family 34 (sodium phosphate), member 2	1.00359	0.999615	1.0224855
10569	SLU7	SLU7 splicing factor homolog (S. cerevisiae)	0.97365	0.886858	0.9650141
10570	DPYSL4	dihydropyrimidinase-like 4	0.97902	0.995758	1.0104304
10572	SIVA1	SIVA1, apoptosis-inducing factor	0.98256	0.954426	0.9829753
10573	MRPL28	mitochondrial ribosomal protein L28	0.95386	1.057339	1.0976296
10574	CCT7	chaperonin containing TCP1, subunit 7 (eta)	0.97777	0.974789	1.0033913
10575	CCT4	chaperonin containing TCP1, subunit 4 (delta)	0.96542	0.986723	1.0463098
10576	CCT2	chaperonin containing TCP1, subunit 2 (beta)	0.9351	0.988263	1.0991286
10577	NPC2	Niemann-Pick disease, type C2	0.98906	0.966609	1.0055842
10578	GNLY	granulysin	1.04767	0.976476	0.8970268
10579	TACC2	transforming, acidic coiled-coil containing protein 2	1.00028	0.997293	1.0177487
10580	SORBS1	sorbin and SH3 domain containing 1	1.0018	0.99886	0.9817461
10584	COLEC10	collectin sub-family member 10 (C-type lectin)	1.00312	0.995909	1.0195352
10585	POMT1	protein-O-mannosyltransferase 1	0.96173	1.027906	1.0386074
10586	MAB21L2	mab-21-like 2 (C. elegans)	0.97371	0.989805	0.9980284
10587	TXNRD2	thioredoxin reductase 2	0.98127	0.981719	1.0050316
10588	MTHFS	5,10-methenyltetrahydrofolate synthetase (5-formyltetrahydrofolate cyclo-ligase)	0.95622	0.988296	1.0106417
10589	DRAP1	DR1-associated protein 1 (negative cofactor 2 alpha)	0.96486	0.943652	1.1565217
10590	SCGN	secretagogin, EF-hand calcium binding protein	1.00212	1.024601	1.0027616
10591	C6orf108	chromosome 6 open reading frame 108	0.99572	0.942215	1.0665843
10592	SMC2	structural maintenance of chromosomes 2	0.94848	0.918722	1.0011557
10594	PRPF8	PRP8 pre-mRNA processing factor 8 homolog (S. cerevisiae)	0.99128	0.961824	0.9788083
10595	ERN2	endoplasmic reticulum to nucleus signaling 2	0.99334	0.996943	1.0359624
10598	AHSA1	AHA1, activator of heat shock 90kDa protein ATPase homolog 1 (yeast)	0.9393	1.016114	1.0222075
10599	SLCO1B1	solute carrier organic anion transporter family, member 1B1	0.99635	1.014589	1.0247637
10600	USP16	ubiquitin specific peptidase 16	0.94335	0.930545	0.987367
10602	CDC42EP3	CDC42 effector protein (Rho GTPase binding) 3	1.03615	0.942682	0.9703916

10603	SH2B2	SH2B adaptor protein 2	1.01837	0.961887	0.9702679
10605	PAIP1	poly(A) binding protein interacting protein 1	1.00824	0.970312	1.0390687
10606	PAICS	phosphoribosylaminoimidazole carboxylase, phosphoribosylaminoimidazole succinocarboxamide synthetase	0.97317	0.886047	0.9701079
10607	TBL3	transducin (beta)-like 3	0.97654	0.968017	1.0284643
10608	MXD4	MAX dimerization protein 4	0.99163	1.061121	1.0111814
10609	LEPREL4	leprecan-like 4	0.98604	0.986561	0.976841
10610	ST6GALNAC	ST6 (alpha-N-acetyl-neuraminyl-2,3-beta-galactosyl-1,3)-N-acetylgalactosaminide alpha-2,6-sialyltransferase	0.95593	1.009368	0.9358847
10611	PDLIM5	PDZ and LIM domain 5	0.98667	1.002616	0.9583385
10612	TRIM3	tripartite motif containing 3	0.98509	1.003175	1.0086795
10613	ERLIN1	ER lipid raft associated 1	0.96528	1.016459	0.9809439
10614	HEXIM1	hexamethylene bis-acetamide inducible 1	0.91237	0.949702	1.0116641
10615	SPAG5	sperm associated antigen 5	0.9919	1.027823	0.9975853
10616	RBCK1	RanBP-type and C3HC4-type zinc finger containing 1	0.94862	0.92724	1.0654723
10617	STAMBP	STAM binding protein	0.9918	0.951851	1.0283046
10618	TGOLN2	trans-golgi network protein 2	0.99942	0.993904	0.9915458
10620	ARID3B	AT rich interactive domain 3B (BRIGHT-like)	0.9972	0.978095	0.9692457
10621	POLR3F	polymerase (RNA) III (DNA directed) polypeptide F, 39 kDa	0.9558	0.992199	1.05889
10622	POLR3G	polymerase (RNA) III (DNA directed) polypeptide G (32kD)	0.98539	0.98093	1.0095543
10623	POLR3C	polymerase (RNA) III (DNA directed) polypeptide C (62kD)	0.97142	1	1.1131323
10625	IVNS1ABP	influenza virus NS1A binding protein	1.00654	1.079924	1.0000612
10626	TRIM16	tripartite motif containing 16	0.96932	0.959724	1.0055915
10627	MYL12A	myosin, light chain 12A, regulatory, non-sarcomeric	0.95513	0.980626	0.9175674
10628	TXNIP	thioredoxin interacting protein	1.0359	0.93325	1.0212686
10629	TAF6L	TAF6-like RNA polymerase II, p300/CBP-associated factor (PCAF)-associated factor, 65kDa	0.92951	0.973074	1.0501879
10631	POSTN	periostin, osteoblast specific factor	1.00361	0.965971	1.0089069
10632	ATP5L	ATP synthase, H+ transporting, mitochondrial Fo complex, subunit G	0.97127	1.015028	1.0073408
10633	RASL10A	RAS-like, family 10, member A	1.00887	1.006627	1.0238255
10634	GAS2L1	growth arrest-specific 2 like 1	0.93779	0.935832	1.0273184
10635	RAD51AP1	RAD51 associated protein 1	1.00507	0.955829	1.0123445
10636	RGS14	regulator of G-protein signaling 14	0.98096	0.985094	1.1211081
10637	LEFTY1	left-right determination factor 1	1.00264	0.993329	0.9940257
10640	EXOC5	exocyst complex component 5	0.94948	1.009313	1.0216414
10641	NPRL2	nitrogen permease regulator-like 2 (S. cerevisiae)	0.95661	1.02796	1.0254202

10642	IGF2BP1	insulin-like growth factor 2 mRNA binding protein 1	0.9866	0.989287	0.9975344
10643	IGF2BP3	insulin-like growth factor 2 mRNA binding protein 3	0.9937	0.987794	1.0145633
10644	IGF2BP2	insulin-like growth factor 2 mRNA binding protein 2	0.9972	0.974902	0.9696895
10645	CAMKK2	calcium/calmodulin-dependent protein kinase kinase 2, beta	1.00369	1.04027	0.9536061
10647	SCGB1D2	secretoglobin, family 1D, member 2	0.9826	0.994483	0.9881799
10648	SCGB1D1	secretoglobin, family 1D, member 1	1.01811	1.002097	0.9965956
10650	SLMO1	slowmo homolog 1 (Drosophila)	0.99287	1.04666	1.0378354
10651	MTX2	metaxin 2	0.96263	0.977979	0.9948116
10652	YKT6	YKT6 v-SNARE homolog (S. cerevisiae)	1.00336	0.925477	1.0433135
10653	SPINT2	serine peptidase inhibitor, Kunitz type, 2	0.9468	1.061714	1.0476975
10654	PMVK	phosphomevalonate kinase	0.9782	1.057405	1.0547634
10655	DMRT2	doublesex and mab-3 related transcription factor 2	0.98226	0.953707	1.053782
10656	KHDRBS3	KH domain containing, RNA binding, signal transduction associated 3	0.99752	1.023249	1.0207341
10657	KHDRBS1	KH domain containing, RNA binding, signal transduction associated 1	0.98165	1.048849	0.957545
10658	CELF1	CUGBP, Elav-like family member 1	0.97978	1.039737	1.0509511
10659	CELF2	CUGBP, Elav-like family member 2	1.0198	0.937908	0.9875556
10660	LBX1	ladybird homeobox 1	0.99732	1.021717	1.1043092
10661	KLF1	Kruppel-like factor 1 (erythroid)	0.96756	0.995266	1.0276143
10663	CXCR6	chemokine (C-X-C motif) receptor 6	0.98451	1.065008	0.9685549
10664	CTCF	CCCTC-binding factor (zinc finger protein)	0.93954	0.942794	1.0788694
10665	C6orf10	chromosome 6 open reading frame 10	0.99818	0.980469	1.0212118
10666	CD226	CD226 molecule	0.95821	0.929303	1.0168521
10667	FARS2	phenylalanyl-tRNA synthetase 2, mitochondrial	0.97946	0.980278	1.013717
10668	CGRRF1	cell growth regulator with ring finger domain 1	0.95155	0.98722	0.9647358
10669	CGREF1	cell growth regulator with EF-hand domain 1	1.00869	1.016978	1.0066987
10670	RRAGA	Ras-related GTP binding A	0.97216	0.982605	0.994058
10671	DCTN6	dynactin 6	0.99611	0.977738	0.9323939
10672	GNA13	guanine nucleotide binding protein (G protein), alpha 13	0.99179	1.042306	0.9125151
10673	TNFSF13B	tumor necrosis factor (ligand) superfamily, member 13b	0.96732	0.979755	1.0601403
10675	CSPG5	chondroitin sulfate proteoglycan 5 (neuroglycan C)	1.00096	0.993392	1.0268602
10677	AVIL	advillin	1.00224	1.008916	0.9689122
10678	B3GNT2	UDP-GlcNAc:betaGal beta-1,3-N-acetylglucosaminyltransferase 2	0.95684	0.989647	0.9754812
10681	GNB5	guanine nucleotide binding protein (G protein), beta 5	0.97481	0.990083	0.9631429

10682	EBP	emopamil binding protein (sterol isomerase)	0.92959	1.03189	1.0316024
10683	DLL3	delta-like 3 (Drosophila)	1.0057	0.981864	1.032883
10686	CLDN16	claudin 16	1.01683	1.048859	1.0112965
10687	PNMA2	paraneoplastic antigen MA2	0.99394	1.02354	1.0212979
10690	FUT9	fucosyltransferase 9 (alpha (1,3) fucosyltransferase)	1.00322	1.010856	1.0147802
10691	GMEB1	glucocorticoid modulatory element binding protein 1	0.98567	1.013284	1.119779
10692	RRH	retinal pigment epithelium-derived rhodopsin homolog	0.99833	1.005803	1.0269056
10693	CCT6B	chaperonin containing TCP1, subunit 6B (zeta 2)	0.97512	0.963904	0.9467687
10694	CCT8	chaperonin containing TCP1, subunit 8 (theta)	0.97944	0.934457	1.0716748
10695	CNPY3	canopy 3 homolog (zebrafish)	1.00262	0.946421	1.0231272
10712	FAM189B	family with sequence similarity 189, member B	0.98123	0.958726	1.0432023
10713	USP39	ubiquitin specific peptidase 39	0.97032	0.955307	1.0068291
10714	POLD3	polymerase (DNA-directed), delta 3, accessory subunit	0.96831	0.917328	1.1249562
10716	TBR1	T-box, brain, 1	1.00279	1.002359	1.0627943
10717	AP4B1	adaptor-related protein complex 4, beta 1 subunit	1.02163	1.016557	1.1179611
10718	NRG3	neuregulin 3	1.00822	1.012827	1.0011567
10720	UGT2B11	UDP glucuronosyltransferase 2 family, polypeptide B11	1.01591	1.034796	0.9538647
10721	POLQ	polymerase (DNA directed), theta	1.00554	0.98828	1.0137331
10723	SLC12A7	solute carrier family 12 (potassium/chloride transporters), member 7	1.02764	0.910773	0.9857462
10724	MGEA5	meningioma expressed antigen 5 (hyaluronidase)	0.97146	1.131316	0.9306618
10725	NFAT5	nuclear factor of activated T-cells 5, tonicity-responsive	0.97006	1.120569	0.9839568
10726	NUDC	nuclear distribution gene C homolog (A. nidulans)	0.98766	0.982237	1.0169388
10728	PTGES3	prostaglandin E synthase 3 (cytosolic)	0.99407	1.021174	0.9620513
10730	YME1L1	YME1-like 1 (S. cerevisiae)	0.98934	0.864425	1.0609447
10732	TCFL5	transcription factor-like 5 (basic helix-loop-helix)	0.99527	0.984279	1.0078727
10733	PLK4	polo-like kinase 4	0.98808	0.909979	1.0185287
10734	STAG3	stromal antigen 3	0.99737	1.006229	0.9966247
10735	STAG2	stromal antigen 2	0.93528	1.094553	0.968811
10736	SIX2	SIX homeobox 2	0.99809	0.955665	1.0753988
10737	RFPL3-AS1	RFPL3 antisense RNA 1 (non-protein coding)	0.97342	1.025863	1.0225046
10738	RFPL3	ret finger protein-like 3	0.98569	0.952196	1.0181619
10740	RFPL1-AS1	RFPL1 antisense RNA 1 (non-protein coding)	0.98279	1.05386	1.0943729
10741	RBBP9	retinoblastoma binding protein 9	0.91162	0.951425	1.0718747

10742	RAI2	retinoic acid induced 2	0.99908	0.93307	1.0271646
10743	RAI1	retinoic acid induced 1	1.00156	1.031528	1.0064053
10745	PHTF1	putative homeodomain transcription factor 1	1.00193	0.996764	1.0397473
10746	MAP3K2	mitogen-activated protein kinase kinase kinase 2	1.00171	1.123654	0.9521152
10747	MASP2	mannan-binding lectin serine peptidase 2	1.00059	0.998435	0.9960725
10748	KLRAP1	killer cell lectin-like receptor subfamily A pseudogene 1	1.03053	1.051928	1.0371327
10749	KIF1C	kinesin family member 1C	0.96423	0.984185	1.0289849
10750	GRAP	GRB2-related adaptor protein	1.0017	1.014076	0.9844895
10752	CHL1	cell adhesion molecule with homology to L1CAM (close homolog of L1)	0.99513	0.999629	1.0050334
10753	CAPN9	calpain 9	1.00986	1.001355	1.0285638
10755	GIPC1	GIPC PDZ domain containing family, member 1	0.96966	0.961881	1.0035824
10758	TRAF3IP2	TRAF3 interacting protein 2	0.97912	1.011269	0.9901008
10761	PLAC1	placenta-specific 1	1.01863	1.007826	0.9875136
10762	NUP50	nucleoporin 50kDa	0.97043	0.978525	0.9848633
10763	NES	nestin	0.99272	0.991234	1.01424
10765	KDM5B	lysine (K)-specific demethylase 5B	0.99049	1.071175	1.0278373
10766	TOB2	transducer of ERBB2, 2	0.94133	1.017474	1.0104066
10767	HBS1L	HBS1-like (S. cerevisiae)	0.97843	0.905806	1.0225115
10768	AHCYL1	adenosylhomocysteinase-like 1	0.98363	0.989704	1.0197933
10769	PLK2	polo-like kinase 2	0.99631	1.031505	0.9602187
10771	ZMYND11	zinc finger, MYND-type containing 11	0.95764	1.039024	1.012801
10772	SRSF10	serine/arginine-rich splicing factor 10	0.99919	0.864352	0.9324315
10773	ZBTB6	zinc finger and BTB domain containing 6	0.92425	0.998478	1.048861
10775	POP4	processing of precursor 4, ribonuclease P/MRP subunit (S. cerevisiae)	0.92154	0.908798	1.0497225
10776	ARPP19	cAMP-regulated phosphoprotein, 19kDa	1.00564	0.929809	0.9305091
10777	ARPP21	cAMP-regulated phosphoprotein, 21kDa	1.0036	1.000279	1.0150566
10778	ZNF271	zinc finger protein 271	0.87422	0.910353	0.9266405
10780	ZNF234	zinc finger protein 234	0.93729	1.044293	0.9688406
10781	ZNF266	zinc finger protein 266	0.9341	0.977353	1.0460649
10782	ZNF274	zinc finger protein 274	0.95014	0.968491	0.9445491
10783	NEK6	NIMA (never in mitosis gene a)-related kinase 6	0.99923	0.953798	1.0066169
10785	WDR4	WD repeat domain 4	0.96425	0.965426	1.0228061
10786	SLC17A3	solute carrier family 17 (sodium phosphate), member 3	0.99958	1.015636	0.9885466

10787	NCKAP1	NCK-associated protein 1	0.98925	0.933353	1.0184641
10788	IQGAP2	IQ motif containing GTPase activating protein 2	1.03474	0.915757	0.9655346
10791	VAMP5	vesicle-associated membrane protein 5 (myobrevin)	0.99976	1.037763	1.1225248
10793	ZNF273	zinc finger protein 273	0.95226	0.959049	1.0382861
10794	ZNF460	zinc finger protein 460	0.94264	0.926432	0.9856281
10795	ZNF268	zinc finger protein 268	0.95231	0.951779	1.0638017
10797	MTHFD2	methylenetetrahydrofolate dehydrogenase (NADP+ dependent) 2, methenyltetrahydrofolate cyclohydrolase	1.03351	1.003451	1.0615588
10798	OR5I1	olfactory receptor, family 5, subfamily I, member 1	1.00434	0.999086	0.9360676
10799	RPP40	ribonuclease P/MRP 40kDa subunit	0.96746	0.959679	1.0241246
10800	CYSLTR1	cysteinyl leukotriene receptor 1	0.95802	0.914866	1.0328343
10801	9-Sep	septin 9	0.98206	1.037287	1.0223761
10802	SEC24A	SEC24 family, member A (S. cerevisiae)	1.00035	0.977899	1.0509465
10803	CCR9	chemokine (C-C motif) receptor 9	1.00224	1.014935	0.9536681
10804	GJB6	gap junction protein, beta 6, 30kDa	0.97141	0.990243	0.9576213
10806	SDCCAG8	serologically defined colon cancer antigen 8	0.98124	1.010506	1.0347735
10807	SDCCAG3	serologically defined colon cancer antigen 3	0.97005	1.017735	1.0181312
10808	HSPH1	heat shock 105kDa/110kDa protein 1	0.9374	1.034872	1.0452473
10809	STARD10	StAR-related lipid transfer (START) domain containing 10	0.98272	1.021243	0.9945451
10810	WASF3	WAS protein family, member 3	0.96419	1.011245	1.0003337
10811	NOXA1	NADPH oxidase activator 1	1.00667	0.987292	1.0378768
10813	UTP14A	UTP14, U3 small nucleolar ribonucleoprotein, homolog A (yeast)	0.96012	0.938328	0.9914101
10814	CPLX2	complexin 2	1.004	1.008332	1.0191468
10815	CPLX1	complexin 1	1.004	1.007023	1.0320566
10817	FRS3	fibroblast growth factor receptor substrate 3	1.00466	0.967464	1.044807
10818	FRS2	fibroblast growth factor receptor substrate 2	0.96024	0.958062	1.0855668
10820	OR7E13P	olfactory receptor, family 7, subfamily E, member 13 pseudogene	1.02667	1.054852	1.0291226
10825	NEU3	sialidase 3 (membrane sialidase)	0.98218	0.99247	1.0835392
10826	C5orf4	chromosome 5 open reading frame 4	0.98648	0.908648	1.000924
10827	FAM114A2	family with sequence similarity 114, member A2	0.95103	0.939213	1.0173693
10828	NPM1P19	nucleophosmin 1 (nucleolar phosphoprotein B23, numatrin) pseudogene 19	0.99466	1.01906	1.0447295
10829	NPM1P18	nucleophosmin 1 (nucleolar phosphoprotein B23, numatrin) pseudogene 18	0.97623	0.988789	0.9898432
10833	NPM1P14	nucleophosmin 1 (nucleolar phosphoprotein B23, numatrin) pseudogene 14	1.02803	0.963615	1.0481433
10834	NPM1P13	nucleophosmin 1 (nucleolar phosphoprotein B23, numatrin) pseudogene 13	0.97796	0.99866	1.0415843

10835	NPM1P12	nucleophosmin 1 (nucleolar phosphoprotein B23, numatrin) pseudogene 12	0.97498	1.019156	1.0167529
10838	ZNF275	zinc finger protein 275	0.95473	0.916104	1.0352886
10840	ALDH1L1	aldehyde dehydrogenase 1 family, member L1	1.003	1.006226	1.0315669
10841	FTCD	formiminotransferase cyclodeaminase	1.00651	0.996208	1.0435091
10842	C7orf16	chromosome 7 open reading frame 16	1.04738	1.015634	0.9373718
10844	TUBGCP2	tubulin, gamma complex associated protein 2	0.98031	1.025433	0.9486637
10845	CLPX	ClpX caseinolytic peptidase X homolog (E. coli)	0.94429	1.024643	1.0966322
10846	PDE10A	phosphodiesterase 10A	1.01076	1.003922	1.020516
10847	SRCAP	Snf2-related CREBBP activator protein	0.96253	0.969596	0.9169262
10848	PPP1R13L	protein phosphatase 1, regulatory (inhibitor) subunit 13 like	0.99001	0.95166	1.06572
10849	CD3EAP	CD3e molecule, epsilon associated protein	0.9804	0.969697	1.0240095
10850	CCL27	chemokine (C-C motif) ligand 27	1	0.985541	1.0230972
10855	HPSE	heparanase	0.99366	0.899266	1.0220706
10856	RUVBL2	RuvB-like 2 (E. coli)	0.9417	0.921241	1.0479982
10857	PGRMC1	progesterone receptor membrane component 1	0.94761	0.87097	0.9757544
10858	CYP46A1	cytochrome P450, family 46, subfamily A, polypeptide 1	1.00532	1.017291	1.0123994
10859	LILRB1	leukocyte immunoglobulin-like receptor, subfamily B (with TM and ITIM domains), member 1	0.99565	1.004339	1.0900017
10861	SLC26A1	solute carrier family 26 (sulfate transporter), member 1	0.9979	1.026868	1.0150383
10864	SLC22A7	solute carrier family 22 (organic anion transporter), member 7	1.00461	0.985488	0.9832983
10865	ARID5A	AT rich interactive domain 5A (MRF1-like)	1.00565	1.010366	1.00699
10866	HCP5	HLA complex P5	0.96089	0.947391	1.0162347
10867	TSPAN9	tetraspanin 9	0.9817	1.002609	0.996719
10868	USP20	ubiquitin specific peptidase 20	0.9274	0.975855	0.9775034
10869	USP19	ubiquitin specific peptidase 19	0.98271	0.933976	1.0540892
10870	HCST	hematopoietic cell signal transducer	0.98135	0.947287	0.9854726
10871	CD300C	CD300c molecule	1.00064	0.961927	1.0428133
10873	ME3	malic enzyme 3, NADP(+)-dependent, mitochondrial	0.98737	1.000763	0.9863688
10874	NMU	neuromedin U	0.9843	0.985249	1.0097104
10875	FGL2	fibrinogen-like 2	1.02159	0.736081	0.8988564
10876	EDDM3A	epididymal protein 3A	0.98807	1.0617	1.0076115
10877	CFHR4	complement factor H-related 4	1.02402	1.022317	0.9811742
10878	CFHR3	complement factor H-related 3	0.99941	0.969101	1.0100733
10879	SMR3B	submaxillary gland androgen regulated protein 3B	0.9917	0.992759	1.0234488

10880	ACTL7B	actin-like 7B	0.98316	1.034576	0.9255323
10881	ACTL7A	actin-like 7A	0.97372	1.069398	0.9985903
10882	C1QL1	complement component 1, q subcomponent-like 1	0.99349	1.029482	1.0679374
10884	MRPS30	mitochondrial ribosomal protein S30	0.9698	0.927595	1.0549467
10885	WDR3	WD repeat domain 3	0.96397	0.939721	1.069201
10886	NPFFR2	neuropeptide FF receptor 2	1.00508	0.994867	1.0160004
10887	PROKR1	prokineticin receptor 1	0.99397	1.04486	0.9969057
10888	GPR83	G protein-coupled receptor 83	0.99429	0.991579	1.0019168
10890	RAB10	RAB10, member RAS oncogene family	1.01645	1.036892	0.9903422
10891	PPARGC1A	peroxisome proliferator-activated receptor gamma, coactivator 1 alpha	1.00594	0.996302	0.9980285
10892	MALT1	mucosa associated lymphoid tissue lymphoma translocation gene 1	0.94639	1.023795	0.9727741
10893	MMP24	matrix metallopeptidase 24 (membrane-inserted)	0.98415	0.990685	0.9720076
10894	LYVE1	lymphatic vessel endothelial hyaluronan receptor 1	0.99128	1.009964	1.0214176
10895	PPBPL2	pro-platelet basic protein-like 2	1.0044	0.987654	0.9638412
10897	YIF1A	Yip1 interacting factor homolog A (S. cerevisiae)	0.9473	0.954667	1.0636866
10898	CPSF4	cleavage and polyadenylation specific factor 4, 30kDa	0.9962	0.987757	1.0287906
10899	JTB	jumping translocation breakpoint	0.98308	0.941129	1.0864581
10900	RUNDC3A	RUN domain containing 3A	0.9997	0.991312	1.0060073
10902	BRD8	bromodomain containing 8	0.97398	0.975591	1.0155676
10903	MTMR11	myotubularin related protein 11	1.02884	0.996065	0.9821325
10904	BLCAP	bladder cancer associated protein	0.94772	0.969765	0.9557623
10905	MAN1A2	mannosidase, alpha, class 1A, member 2	0.98593	0.969189	0.9795639
10906	TRAFD1	TRAF-type zinc finger domain containing 1	0.93698	0.950501	1.0608561
10907	TXNL4A	thioredoxin-like 4A	0.94676	0.983214	1.0221143
10908	PNPLA6	patatin-like phospholipase domain containing 6	0.96512	1.014206	1.0272505
10910	SUGT1	SGT1, suppressor of G2 allele of SKP1 (S. cerevisiae)	0.93786	0.994202	1.0805441
10911	UTS2	urotensin 2	0.97868	0.902433	0.9600067
10912	GADD45G	growth arrest and DNA-damage-inducible, gamma	0.97776	1.002067	1.0262395
10913	EDAR	ectodysplasin A receptor	0.98332	0.987212	0.9684353
10914	PAPOLA	poly(A) polymerase alpha	0.95127	1.045113	1.1099744
10915	TCERG1	transcription elongation regulator 1	0.99469	0.873388	1.0956553
10916	MAGED2	melanoma antigen family D, 2	0.92361	0.937309	1.0054913
10917	BTNL3	butyrophilin-like 3	0.99157	1.010133	1.0344107

10919	EHMT2	euchromatic histone-lysine N-methyltransferase 2	0.95663	1.013946	1.0217723
10920	COPS8	COP9 constitutive photomorphogenic homolog subunit 8 (Arabidopsis)	0.98949	0.912083	0.9270844
10921	RNPS1	RNA binding protein S1, serine-rich domain	1.00681	1.000724	0.970904
10922	FASTK	Fas-activated serine/threonine kinase	0.97841	0.98038	1.0580572
10923	SUB1	SUB1 homolog (S. cerevisiae)	1.04752	0.903488	0.9924914
10924	SMPDL3A	sphingomyelin phosphodiesterase, acid-like 3A	1.01095	0.948741	0.9707223
10926	DBF4	DBF4 homolog (S. cerevisiae)	0.98863	0.971281	0.9938751
10927	SPIN1	spindlin 1	0.9337	0.936128	0.9651365
10928	RALBP1	ralA binding protein 1	0.93167	0.95144	1.0547666
10929	SRSF8	serine/arginine-rich splicing factor 8	0.88422	0.898764	1.0095934
10930	APOBEC2	apolipoprotein B mRNA editing enzyme, catalytic polypeptide-like 2	1.01837	0.975425	0.9625756
10933	MORF4L1	mortality factor 4 like 1	0.97113	0.965996	0.938122
10934	MORF4	mortality factor 4	0.98888	1.005884	0.9553301
10935	PRDX3	peroxiredoxin 3	0.9547	0.986297	1.0478702
10936	GPR75	G protein-coupled receptor 75	0.99034	0.977184	1.0412946
10938	EHD1	EH-domain containing 1	0.9439	1.077088	0.930004
10939	AFG3L2	AFG3 ATPase family gene 3-like 2 (S. cerevisiae)	0.975	0.996139	1.0503316
10940	POP1	processing of precursor 1, ribonuclease P/MRP subunit (S. cerevisiae)	0.98057	1.011168	1.014683
10941	UGT2A1	UDP glucuronosyltransferase 2 family, polypeptide A1, complex locus	1.01047	0.992921	1.0177689
10942	PRSS21	protease, serine, 21 (testisin)	0.98758	1.059748	1.089632
10943	MSL3	male-specific lethal 3 homolog (Drosophila)	0.94954	0.978991	1.1305592
10944	C11orf58	chromosome 11 open reading frame 58	0.96983	0.891663	0.9531898
10945	KDELR1	KDEL (Lys-Asp-Glu-Leu) endoplasmic reticulum protein retention receptor 1	0.93794	1.038326	1.0931759
10946	SF3A3	splicing factor 3a, subunit 3, 60kDa	1.01132	0.959028	1.0699928
10947	AP3M2	adaptor-related protein complex 3, mu 2 subunit	0.99288	0.949073	1.0882911
10948	STARD3	StAR-related lipid transfer (START) domain containing 3	0.96953	1.004666	0.9880191
10949	HNRNPA0	heterogeneous nuclear ribonucleoprotein A0	0.98641	0.940088	0.9441067
10950	BTG3	BTG family, member 3	0.98479	0.976465	0.9833527
10951	CBX1	chromobox homolog 1	1.01247	1.040658	1.0526222
10952	SEC61B	Sec61 beta subunit	1.00181	1.054088	1.1083843
10953	TOMM34	translocase of outer mitochondrial membrane 34	0.9527	0.917936	1.0373316
10954	PDIA5	protein disulfide isomerase family A, member 5	0.99939	0.98884	1.0239565
10955	SERINC3	serine incorporator 3	0.97812	1.065302	1.0610615

10956	OS9	osteosarcoma amplified 9, endoplasmic reticulum lectin	0.9523	0.933985	1.0572295
10957	PNRC1	proline-rich nuclear receptor coactivator 1	1.00996	0.967818	0.9055021
10959	TMED2	transmembrane emp24 domain trafficking protein 2	0.99394	1.021385	1.0345814
10960	LMAN2	lectin, mannose-binding 2	0.98259	0.984657	1.1611092
10961	ERP29	endoplasmic reticulum protein 29	0.9553	1.063771	1.1447295
10962	MLLT11	myeloid/lymphoid or mixed-lineage leukemia (trithorax homolog, Drosophila); translocated to, 11	0.96252	1.053008	1.0281439
10963	STIP1	stress-induced-phosphoprotein 1	0.90963	1.107046	1.0012044
10964	IFI44L	interferon-induced protein 44-like	0.94545	0.910715	1.1585208
10965	ACOT2	acyl-CoA thioesterase 2	0.95502	0.887119	1.0838359
10966	RAB40B	RAB40B, member RAS oncogene family	1.00152	0.996985	1.0155637
10969	EBNA1BP2	EBNA1 binding protein 2	0.96421	0.91525	1.0121022
10971	YWHAQ	tyrosine 3-monooxygenase/tryptophan 5-monooxygenase activation protein, theta polypeptide	1.00442	1.042304	0.977593
10972	TMED10	transmembrane emp24-like trafficking protein 10 (yeast)	0.99084	0.936311	0.9991379
10973	ASCC3	activating signal cointegrator 1 complex subunit 3	0.97439	0.895679	1.0131027
10974	C10orf116	chromosome 10 open reading frame 116	0.99984	0.997399	1.0205807
10975	UQCR11	ubiquinol-cytochrome c reductase, complex III subunit XI	0.9226	0.981393	1.0277273
10978	CLP1	CLP1, cleavage and polyadenylation factor I subunit, homolog (S. cerevisiae)	0.93274	0.891974	1.0491159
10979	FERMT2	fermitin family member 2	0.98593	1.020914	1.0300417
10980	COPS6	COP9 constitutive photomorphogenic homolog subunit 6 (Arabidopsis)	0.97552	0.90952	1.1383133
10981	RAB32	RAB32, member RAS oncogene family	1.00061	0.979071	0.9475015
10982	MAPRE2	microtubule-associated protein, RP/EB family, member 2	0.97501	0.988073	0.9176669
10983	CCNI	cyclin I	1.02124	0.942528	0.9593029
10984	KCNQ1OT1	KCNQ1 overlapping transcript 1 (non-protein coding)	1.01903	0.934179	1.0052812
10985	GCN1L1	GCN1 general control of amino-acid synthesis 1-like 1 (yeast)	0.94609	1.005713	1.1005102
10987	COPS5	COP9 constitutive photomorphogenic homolog subunit 5 (Arabidopsis)	0.94225	0.893359	1.054003
10988	METAP2	methionyl aminopeptidase 2	0.9569	0.984127	1.0504518
10989	IMMT	inner membrane protein, mitochondrial	1.01849	1.010822	0.9980653
10990	LILRB5	leukocyte immunoglobulin-like receptor, subfamily B (with TM and ITIM domains), member 5	0.99183	1.020698	1.0127579
10991	SLC38A3	solute carrier family 38, member 3	1.00476	1.001992	0.9974241
10992	SF3B2	splicing factor 3b, subunit 2, 145kDa	0.92585	0.89608	0.9992062
10993	SDS	serine dehydratase	1.00698	1.01014	0.9623626
10994	ILVBL	ilvB (bacterial acetolactate synthase)-like	0.97078	1.013415	1.0555266
10998	SLC27A5	solute carrier family 27 (fatty acid transporter), member 5	0.98351	0.990149	1.0105362

10999	SLC27A4	solute carrier family 27 (fatty acid transporter), member 4	0.93652	0.921753	1.0797853
11000	SLC27A3	solute carrier family 27 (fatty acid transporter), member 3	0.98774	0.926287	1.0523512
11001	SLC27A2	solute carrier family 27 (fatty acid transporter), member 2	1.00826	0.990686	1.0405594
11004	KIF2C	kinesin family member 2C	1.00528	0.980043	1.013812
11005	SPINK5	serine peptidase inhibitor, Kazal type 5	0.99848	1.008266	1.0209257
11006	LILRB4	leukocyte immunoglobulin-like receptor, subfamily B (with TM and ITIM domains), member 4	0.9883	0.968849	1.063399
11007	CCDC85B	coiled-coil domain containing 85B	0.96439	1.002866	1.062965
11009	IL24	interleukin 24	0.96767	0.961423	0.9612348
11011	TLK2	tousled-like kinase 2	0.9779	1.036401	0.951602
11012	KLK11	kallikrein-related peptidase 11	1.00592	1.042309	1.00226
11013	TMSB15A	thymosin beta 15a	0.98005	0.973932	1.0180369
11014	KDELR2	KDEL (Lys-Asp-Glu-Leu) endoplasmic reticulum protein retention receptor 2	1.04444	0.912704	1.0766817
11015	KDELR3	KDEL (Lys-Asp-Glu-Leu) endoplasmic reticulum protein retention receptor 3	0.9911	1.022244	1.0102641
11016	ATF7	activating transcription factor 7	0.99276	1.024909	1.0898697
11017	SNRNP27	small nuclear ribonucleoprotein 27kDa (U4/U6.U5)	0.98566	0.911374	0.9860591
11018	TMED1	transmembrane emp24 protein transport domain containing 1	0.96648	1.010099	1.0616978
11019	LIAS	lipoic acid synthetase	0.99134	0.933974	0.999166
11020	IFT27	intraflagellar transport 27 homolog (Chlamydomonas)	0.97099	0.938831	1.010762
11021	RAB35	RAB35, member RAS oncogene family	0.96455	1.004202	1.0507618
11022	TDRKH	tudor and KH domain containing	0.98221	0.971776	0.9745845
11023	VAX1	ventral anterior homeobox 1	0.98675	1.004811	1.031616
11024	LILRA1	leukocyte immunoglobulin-like receptor, subfamily A (with TM domain), member 1	1.02924	0.87398	0.9672806
11025	LILRB3	leukocyte immunoglobulin-like receptor, subfamily B (with TM and ITIM domains), member 3	0.95186	0.97492	0.8977019
11026	LILRA3	leukocyte immunoglobulin-like receptor, subfamily A (without TM domain), member 3	0.95835	0.981718	0.9027219
11027	LILRA2	leukocyte immunoglobulin-like receptor, subfamily A (with TM domain), member 2	0.9547	0.858269	1.0208952
11030	RBPMS	RNA binding protein with multiple splicing	1.00747	1.019712	1.0009304
11031	RAB31	RAB31, member RAS oncogene family	1.00718	1.018361	1.0074913
11033	ADAP1	ArfGAP with dual PH domains 1	0.98739	0.99918	1.0360668
11034	DSTN	destrin (actin depolymerizing factor)	0.98959	0.993999	0.9643453
11035	RIPK3	receptor-interacting serine-threonine kinase 3	0.97031	0.922771	0.9563064
11036	GTF2A1L	general transcription factor IIA, 1-like	0.99227	0.958573	1.0148928
11040	PIM2	pim-2 oncogene	0.93824	0.99613	0.9935473
11041	B3GNT1	UDP-GlcNAc:betaGal beta-1,3-N-acetylglucosaminyltransferase 1	0.9516	0.940046	1.0832359

11043	MID2	midline 2	0.93134	0.896459	0.9765991
11044	PAPD7	PAP associated domain containing 7	1.01281	0.953417	0.9918947
11045	UPK1A	uroplakin 1A	1.00615	0.979749	0.9833579
11046	SLC35D2	solute carrier family 35, member D2	0.95156	0.92268	1.0035198
11047	ADRM1	adhesion regulating molecule 1	0.97923	0.970111	1.0647405
11051	NUDT21	nudix (nucleoside diphosphate linked moiety X)-type motif 21	0.94406	0.922675	1.0989027
11052	CPSF6	cleavage and polyadenylation specific factor 6, 68kDa	0.94055	0.914697	0.9921971
11054	OGFR	opioid growth factor receptor	0.9561	1.033437	0.9636496
11055	ZPBP	zona pellucida binding protein	1.00127	0.991611	1.0108245
11056	DDX52	DEAD (Asp-Glu-Ala-Asp) box polypeptide 52	0.94823	0.927833	1.0180424
11057	ABHD2	abhydrolase domain containing 2	0.99524	1.031356	1.0089712
11059	WWP1	WW domain containing E3 ubiquitin protein ligase 1	0.97173	0.926414	0.9013589
11060	WWP2	WW domain containing E3 ubiquitin protein ligase 2	0.96926	0.940612	0.9650664
11061	LECT1	leukocyte cell derived chemotaxin 1	1.00548	0.996074	1.0240615
11062	DUS4L	dihydrouridine synthase 4-like (S. cerevisiae)	1.00967	0.962943	1.0132532
11063	SOX30	SRY (sex determining region Y)-box 30	0.98417	1.023193	1.00732
11064	CNTRL	centriolin	0.92849	1.039374	1.0338578
11065	UBE2C	ubiquitin-conjugating enzyme E2C	0.98366	1.024547	1.0085863
11066	SNRNP35	small nuclear ribonucleoprotein 35kDa (U11/U12)	0.96079	0.909126	1.054924
11067	C10orf10	chromosome 10 open reading frame 10	1.01179	0.98033	1.019262
11068	CYB561D2	cytochrome b-561 domain containing 2	0.99327	0.970792	1.0430085
11069	RAPGEF4	Rap guanine nucleotide exchange factor (GEF) 4	1.01014	0.998955	1.0160423
11070	TMEM115	transmembrane protein 115	0.96435	1.026779	0.9348442
11072	DUSP14	dual specificity phosphatase 14	0.96229	0.965019	1.0225095
11073	TOPBP1	topoisomerase (DNA) II binding protein 1	0.98348	0.899912	1.0712852
11074	TRIM31	tripartite motif containing 31	0.98063	0.985832	1.0104061
11075	STMN2	stathmin-like 2	0.99485	1.020551	0.9788945
11076	TPPP	tubulin polymerization promoting protein	0.99884	0.983395	0.9941655
11077	HSF2BP	heat shock transcription factor 2 binding protein	0.97695	0.976397	1.0131128
11078	TRIOBP	TRIO and F-actin binding protein	1.00326	0.96168	1.0427778
11079	RER1	RER1 retention in endoplasmic reticulum 1 homolog (S. cerevisiae)	1.01985	1.005809	0.9838435
11080	DNAJB4	DnaJ (Hsp40) homolog, subfamily B, member 4	0.96824	0.901769	0.9652062
11081	KERA	keratocan	0.972	1.000272	1.0283929

11082	ESM1	endothelial cell-specific molecule 1	0.99618	0.995736	0.9812264
11083	DIDO1	death inducer-obliterator 1	0.96083	0.996858	0.9929218
11085	ADAM30	ADAM metallopeptidase domain 30	1.00807	1.033	1.0240473
11086	ADAM29	ADAM metallopeptidase domain 29	1.0178	1.015585	1.0224345
11092	C9orf9	chromosome 9 open reading frame 9	0.98603	0.979899	1.0209529
11093	ADAMTS13	ADAM metallopeptidase with thrombospondin type 1 motif, 13	1.00413	1.000902	1.0147525
11094	C9orf7	chromosome 9 open reading frame 7	1.00201	1.062534	0.9772104
11095	ADAMTS8	ADAM metallopeptidase with thrombospondin type 1 motif, 8	0.98939	1.005106	0.99465
11096	ADAMTS5	ADAM metallopeptidase with thrombospondin type 1 motif, 5	0.9814	1.001853	1.005039
11097	NUPL2	nucleoporin like 2	1.02858	0.989471	1.0326451
11098	PRSS23	protease, serine, 23	0.9795	0.953653	0.8965022
11099	PTPN21	protein tyrosine phosphatase, non-receptor type 21	0.99032	1.016249	1.0112732
11100	HNRNPUL1	heterogeneous nuclear ribonucleoprotein U-like 1	0.96301	1.077206	1.0078147
11101	ATE1	arginyltransferase 1	0.95209	0.910893	1.0559611
11102	RPP14	ribonuclease P/MRP 14kDa subunit	0.97955	0.907125	0.9591161
11103	KRR1	KRR1, small subunit (SSU) processome component, homolog (yeast)	0.91174	0.863289	1.0450207
11104	KATNA1	katanin p60 (ATPase containing) subunit A 1	1.00625	0.976726	1.0634666
11105	PRDM7	PR domain containing 7	0.97194	1.032726	1.0280565
11107	PRDM5	PR domain containing 5	0.97667	0.975866	1.0002777
11108	PRDM4	PR domain containing 4	0.94382	0.965218	0.9527002
11112	HIBADH	3-hydroxyisobutyrate dehydrogenase	0.99273	1.005741	0.9909373
11113	CIT	citron (rho-interacting, serine/threonine kinase 21)	0.99618	0.996869	1.0151267
11116	FGFR1OP	FGFR1 oncogene partner	0.98509	1.026824	1.049322
11117	EMILIN1	elastin microfibril interfacer 1	1.00483	0.999765	1.043566
11118	BTN3A2	butyrophilin, subfamily 3, member A2	0.98361	0.964462	1.0060097
11119	BTN3A1	butyrophilin, subfamily 3, member A1	1.02705	0.897162	0.9492788
11120	BTN2A1	butyrophilin, subfamily 2, member A1	1.04143	1.083435	1.0253025
11122	PTPRT	protein tyrosine phosphatase, receptor type, T	1.00382	0.984815	0.9979079
11123	RCAN3	RCAN family member 3	0.95248	0.919618	0.9316439
11124	FAF1	Fas (TNFRSF6) associated factor 1	0.97881	0.927106	0.9927396
11126	CD160	CD160 molecule	0.92396	1.071236	0.9152964
11127	KIF3A	kinesin family member 3A	0.97876	1.011608	1.0576529
11128	POLR3A	polymerase (RNA) III (DNA directed) polypeptide A, 155kDa	0.93671	0.928987	0.9704662

11129	CLASRP	CLK4-associating serine/arginine rich protein	0.97243	0.983794	0.9680372
11130	ZWINT	ZW10 interactor	0.98782	1.015547	1.0085198
11131	CAPN11	calpain 11	1.01004	1.008158	0.9836386
11132	CAPN10	calpain 10	1.00178	0.983501	0.9958897
11133	KPTN	kaptin (actin binding protein)	0.9745	0.987741	1.0448469
11135	CDC42EP1	CDC42 effector protein (Rho GTPase binding) 1	0.98896	0.951762	0.9747679
11136	SLC7A9	solute carrier family 7 (cationic amino acid transporter, y+ system), member 9	0.98833	0.99991	1.0496336
11137	PWP1	PWP1 homolog (S. cerevisiae)	0.91617	0.949543	1.0972786
11138	TBC1D8	TBC1 domain family, member 8 (with GRAM domain)	1.01604	1.060857	1.00187
11140	CDC37	cell division cycle 37 homolog (S. cerevisiae)	0.93436	1.024368	1.0298141
11141	IL1RAPL1	interleukin 1 receptor accessory protein-like 1	1.00293	1.016353	0.9981245
11142	PKIG	protein kinase (cAMP-dependent, catalytic) inhibitor gamma	0.99267	1.013009	0.9954432
11143	MYST2	MYST histone acetyltransferase 2	1.00815	0.958376	0.9945232
11144	DMC1	DMC1 dosage suppressor of mck1 homolog, meiosis-specific homologous recombination (yeast)	0.9769	0.929822	1.0127277
11145	PLA2G16	phospholipase A2, group XVI	0.99624	1.035297	1.0342918
11146	GLMN	glomulin, FKBP associated protein	0.98879	0.9753	1.0124002
11147	HHLA3	HERV-H LTR-associating 3	0.98943	0.925679	0.9680674
11148	HHLA2	HERV-H LTR-associating 2	1.02175	1.000372	1.0054868
11149	BVES	blood vessel epicardial substance	1.02374	1.019578	1.0161996
11151	CORO1A	coronin, actin binding protein, 1A	0.97946	0.922302	1.0570848
11152	WDR45	WD repeat domain 45	0.99701	1.019859	1.0361855
11153	FICD	FIC domain containing	0.96358	0.960702	1.0408331
11154	AP4S1	adaptor-related protein complex 4, sigma 1 subunit	0.97344	1.016956	1.0001205
11155	LDB3	LIM domain binding 3	1.00217	1.009272	1.0027736
11156	PTP4A3	protein tyrosine phosphatase type IVA, member 3	1.00517	1.002424	1.0350204
11157	LSM6	LSM6 homolog, U6 small nuclear RNA associated (S. cerevisiae)	1.02533	0.970616	0.9303271
11158	RABL2B	RAB, member of RAS oncogene family-like 2B	0.98252	0.944879	1.0016109
11160	ERLIN2	ER lipid raft associated 2	0.99615	1.002088	0.9836554
11161	C14orf1	chromosome 14 open reading frame 1	0.9368	0.914704	1.027027
11162	NUDT6	nudix (nucleoside diphosphate linked moiety X)-type motif 6	0.97569	0.970498	1.0063683
11163	NUDT4	nudix (nucleoside diphosphate linked moiety X)-type motif 4	0.99558	0.919068	1.0399869
11164	NUDT5	nudix (nucleoside diphosphate linked moiety X)-type motif 5	0.94262	0.999195	1.0225428
11165	NUDT3	nudix (nucleoside diphosphate linked moiety X)-type motif 3	0.99156	0.925544	1.0242368

11166	SOX21	SRY (sex determining region Y)-box 21	0.96644	1.008764	1.0491789
11167	FSTL1	follistatin-like 1	0.97264	0.978278	0.9898371
11168	PSIP1	PC4 and SFRS1 interacting protein 1	0.88071	0.955639	0.9798445
11169	WDHD1	WD repeat and HMG-box DNA binding protein 1	0.95703	1.004555	1.0131998
11170	FAM107A	family with sequence similarity 107, member A	1.02505	0.951385	1.0125707
11171	STRAP	serine/threonine kinase receptor associated protein	0.93411	0.969476	0.973136
11172	INSL6	insulin-like 6	0.99841	1.009137	1.019356
11173	ADAMTS7	ADAM metallopeptidase with thrombospondin type 1 motif, 7	0.99568	1.006772	1.0443016
11174	ADAMTS6	ADAM metallopeptidase with thrombospondin type 1 motif, 6	0.99811	1.022514	0.9907321
11176	BAZ2A	bromodomain adjacent to zinc finger domain, 2A	0.9934	1.1055	1.0016627
11177	BAZ1A	bromodomain adjacent to zinc finger domain, 1A	0.93803	1.013136	1.0386468
11178	LZTS1	leucine zipper, putative tumor suppressor 1	0.9975	1.007193	0.9994739
11179	ZNF277	zinc finger protein 277	1.00252	0.978679	1.0256454
11180	WDR6	WD repeat domain 6	1.01812	0.917978	0.9622341
11181	TREH	trehalase (brush-border membrane glycoprotein)	1.0095	0.986571	1.0048701
11182	SLC2A6	solute carrier family 2 (facilitated glucose transporter), member 6	0.99745	1.084426	0.8945337
11183	MAP4K5	mitogen-activated protein kinase kinase kinase kinase 5	0.97823	1.01568	0.9825224
11184	MAP4K1	mitogen-activated protein kinase kinase kinase kinase 1	0.97065	0.964006	0.9907324
11185	INMT	indolethylamine N-methyltransferase	1.01044	1.008464	1.0089682
11186	RASSF1	Ras association (RalGDS/AF-6) domain family member 1	1.01599	1.030446	0.9505487
11187	PKP3	plakophilin 3	0.99219	1.0006	1.0502028
11188	NISCH	nischarin	1.00154	0.929196	0.968638
11189	CELF3	CUGBP, Elav-like family member 3	0.9925	1.016778	1.0197869
11190	CEP250	centrosomal protein 250kDa	0.94455	0.968117	1.004195
11193	WBP4	WW domain binding protein 4 (formin binding protein 21)	0.92866	0.977093	1.077536
11194	ABCB8	ATP-binding cassette, sub-family B (MDR/TAP), member 8	0.9823	1.017568	1.0191619
11196	SEC23IP	SEC23 interacting protein	0.93952	1.003031	1.063833
11197	WIF1	WNT inhibitory factor 1	1.01504	1.025731	1.0085212
11198	SUPT16H	suppressor of Ty 16 homolog (S. cerevisiae)	0.94512	0.982957	1.0662692
11199	ANXA10	annexin A10	1.00081	1.011424	0.9997203
11200	CHEK2	CHK2 checkpoint homolog (S. pombe)	0.96037	0.978915	0.9998226
11201	POLI	polymerase (DNA directed) iota	0.95176	0.95329	1.0269531
11211	FZD10	frizzled family receptor 10	0.98325	0.985486	0.9872748

11212	PROSC	proline synthetase co-transcribed homolog (bacterial)	0.98787	0.955159	1.0429125
11213	IRAK3	interleukin-1 receptor-associated kinase 3	0.98809	1.106491	1.1109827
11214	AKAP13	A kinase (PRKA) anchor protein 13	0.96364	1.05022	1.0252286
11215	AKAP11	A kinase (PRKA) anchor protein 11	0.96103	1.05129	0.9930694
11216	AKAP10	A kinase (PRKA) anchor protein 10	0.94324	0.999368	0.9910524
11218	DDX20	DEAD (Asp-Glu-Ala-Asp) box polypeptide 20	0.949	0.958316	1.0642195
11219	TREX2	three prime repair exonuclease 2	0.99907	0.973127	1.0688928
11221	DUSP10	dual specificity phosphatase 10	1.01719	0.907074	0.9743947
11222	MRPL3	mitochondrial ribosomal protein L3	0.9961	0.961508	0.9974817
11224	RPL35	ribosomal protein L35	0.98663	0.95898	0.9909856
11226	GALNT6	UDP-N-acetyl-alpha-D-galactosamine:polypeptide N-acetylgalactosaminyltransferase 6 (GalNAc-T6)	0.97641	1.020361	0.9878954
11227	GALNT5	UDP-N-acetyl-alpha-D-galactosamine:polypeptide N-acetylgalactosaminyltransferase 5 (GalNAc-T5)	1.00825	1.002196	1.0269744
11228	RASSF8	Ras association (RalGDS/AF-6) domain family (N-terminal) member 8	1.00424	0.989806	1.0029431
11230	PRAF2	PRA1 domain family, member 2	0.96931	1.02226	1.0169566
11231	SEC63	SEC63 homolog (S. cerevisiae)	1.01227	0.936862	1.0350871
11232	POLG2	polymerase (DNA directed), gamma 2, accessory subunit	0.94985	0.978517	1.0434588
11234	HPS5	Hermansky-Pudlak syndrome 5	0.97328	0.971139	1.0167593
11235	PDCD10	programmed cell death 10	0.99781	1.034196	1.1289074
11236	RNF139	ring finger protein 139	0.9967	0.90276	0.9018658
11237	RNF24	ring finger protein 24	0.98361	0.989709	1.0062112
11238	CA5B	carbonic anhydrase VB, mitochondrial	0.95179	1.03585	0.9425318
11240	PADI2	peptidyl arginine deiminase, type II	1.01428	1.001912	0.9496876
11243	PMF1	polyamine-modulated factor 1	1.01397	0.945969	1.0500786
11244	ZHX1	zinc fingers and homeoboxes 1	0.95734	0.997784	1.0630515
11245	GPR176	G protein-coupled receptor 176	0.99898	0.997108	1.0024698
11247	NXPH4	neurexophilin 4	0.9932	1.055625	1.0464295
11248	NXPH3	neurexophilin 3	0.99394	1.000558	0.9415735
11249	NXPH2	neurexophilin 2	0.9868	1.016927	1.0131334
11250	GPR45	G protein-coupled receptor 45	1.0291	0.955982	1.0177464
11251	GPR44	G protein-coupled receptor 44	0.99836	0.95023	0.9618267
11252	PACSIN2	protein kinase C and casein kinase substrate in neurons 2	0.9555	1.025565	0.9924252
11253	MAN1B1	mannosidase, alpha, class 1B, member 1	0.96701	0.973266	1.000474
11254	SLC6A14	solute carrier family 6 (amino acid transporter), member 14	0.98322	0.973845	1.0180509

11255	HRH3	histamine receptor H3	0.99069	0.990392	1.0390357
11258	DCTN3	dynactin 3 (p22)	0.97845	0.986332	1.0356668
11259	FILIP1L	filamin A interacting protein 1-like	1.00428	0.999251	1.0084852
11260	XPOT	exportin, tRNA (nuclear export receptor for tRNAs)	0.94567	0.998155	1.0345357
11261	CHP	calcium binding protein P22	0.98891	0.983771	1.0182507
11262	SP140	SP140 nuclear body protein	1.01814	1.05805	0.9667406
11264	PXMP4	peroxisomal membrane protein 4, 24kDa	0.95284	0.953829	0.9987992
11266	DUSP12	dual specificity phosphatase 12	0.97696	0.960194	1.0887236
11267	SNF8	SNF8, ESCRT-II complex subunit, homolog (S. cerevisiae)	0.97385	0.955202	1.003718
11269	DDX19B	DEAD (Asp-Glu-Ala-As) box polypeptide 19B	0.94037	0.982515	1.0056915
11270	NRM	nurim (nuclear envelope membrane protein)	0.99659	0.838249	1.0817242
11272	PRR4	proline rich 4 (lacrimal)	0.97782	0.98347	1.0368961
11273	ATXN2L	ataxin 2-like	0.98142	1.029815	0.9281543
11274	USP18	ubiquitin specific peptidase 18	0.966	0.930797	1.0451006
11275	KLHL2	kelch-like 2, Mayven (Drosophila)	1.02119	0.995424	1.0700511
11276	SYNRG	synergin, gamma	0.9651	1.011294	1.0361182
11277	TREX1	three prime repair exonuclease 1	0.94807	0.921226	1.1110372
11278	KLF12	Kruppel-like factor 12	0.98993	0.98238	0.9990041
11279	KLF8	Kruppel-like factor 8	0.97478	0.971046	1.0338291
11280	SCN11A	sodium channel, voltage-gated, type XI, alpha subunit	1.00536	1.019166	1.0001393
11281	POU6F2	POU class 6 homeobox 2	1.01266	0.995724	1.0140273
11282	MGAT4B	mannosyl (alpha-1,3-)-glycoprotein beta-1,4-N-acetylglucosaminyltransferase, isozyme B	0.98425	0.95189	0.9402454
11283	CYP4F8	cytochrome P450, family 4, subfamily F, polypeptide 8	1.00455	0.969889	1.0194778
11284	PNKP	polynucleotide kinase 3'-phosphatase	0.97986	1.016719	1.0356003
11285	B4GALT7	xylosylprotein beta 1,4-galactosyltransferase, polypeptide 7 (galactosyltransferase I)	0.9831	0.993147	0.9843927
11309	SLCO2B1	solute carrier organic anion transporter family, member 2B1	0.99577	0.991515	0.9981486
11311	VPS45	vacuolar protein sorting 45 homolog (S. cerevisiae)	1.00717	0.958275	1.058235
11313	LYPLA2	lysophospholipase II	0.99352	0.964865	1.0649599
11314	CD300A	CD300a molecule	0.94681	1.047423	0.9530071
11315	PARK7	Parkinson disease (autosomal recessive, early onset) 7	1.00587	0.965434	0.9987686
11316	COPE	coatomer protein complex, subunit epsilon	0.96579	0.958757	1.0026127
11317	RBPJL	recombination signal binding protein for immunoglobulin kappa J region-like	0.98155	0.980053	1.048183
11318	GPR182	G protein-coupled receptor 182	1.00671	0.992905	0.994298

11319	ECD	ecdysoneless homolog (Drosophila)	0.93445	0.957333	0.9556176
11320	MGAT4A	mannosyl (alpha-1,3-)-glycoprotein beta-1,4-N-acetylglucosaminyltransferase, isozyme A	0.96292	0.860671	0.8932722
11321	GPN1	GPN-loop GTPase 1	0.93044	0.91817	1.1070795
11322	TMC6	transmembrane channel-like 6	0.9695	1.057943	0.9197358
11325	DDX42	DEAD (Asp-Glu-Ala-Asp) box polypeptide 42	0.9696	1.026396	1.1382571
11328	FKBP9	FK506 binding protein 9, 63 kDa	1.00762	0.930511	1.0329735
11329	STK38	serine/threonine kinase 38	1.01274	0.957958	0.9266329
11330	CTRC	chymotrypsin C (caldecrin)	1.00249	1.015896	0.9895676
11331	PHB2	prohibitin 2	0.95927	1.039222	1.0737784
11332	ACOT7	acyl-CoA thioesterase 7	0.99013	1.009963	1.036765
11333	PDAP1	PDGFA associated protein 1	0.98854	1.03735	0.924117
11334	TUSC2	tumor suppressor candidate 2	0.97777	0.996436	1.1175832
11335	CBX3	chromobox homolog 3	1.00921	0.953824	0.9772779
11336	EXOC3	exocyst complex component 3	0.9886	1.030442	1.0350706
11337	GABARAP	GABA(A) receptor-associated protein	0.97159	1.018687	0.9021513
11338	U2AF2	U2 small nuclear RNA auxiliary factor 2	0.92187	0.94409	1.033448
11339	OIP5	Opa interacting protein 5	0.9782	1.004374	1.0081143
11340	EXOSC8	exosome component 8	0.91609	0.988971	1.0089664
11341	SCRG1	stimulator of chondrogenesis 1	1.02939	1.036923	1.0245943
11342	RNF13	ring finger protein 13	1.03011	0.986207	0.9221179
11343	MGLL	monoglyceride lipase	1.00928	1.018462	0.9709499
11344	TWF2	twinfilin, actin-binding protein, homolog 2 (Drosophila)	0.98681	0.994076	1.0147339
11345	GABARAPL	GABA(A) receptor-associated protein-like 2	0.95615	0.920152	0.9811179
11346	SYNPO	synaptopodin	1.00032	0.985816	1.0320131
22794	CASC3	cancer susceptibility candidate 3	0.95036	1.046158	1.0107054
22795	NID2	nidogen 2 (osteonidogen)	0.99287	1.012709	1.0107076
22796	COG2	component of oligomeric golgi complex 2	0.97401	0.919256	1.0521786
22797	TFEC	transcription factor EC	1.05755	0.925563	0.968342
22798	LAMB4	laminin, beta 4	1.02147	0.993135	1.0166357
22800	RRAS2	related RAS viral (r-ras) oncogene homolog 2	0.98581	1.0339	1.0292501
22801	ITGA11	integrin, alpha 11	1.00958	0.991631	1.0172197
22802	CLCA4	chloride channel accessory 4	0.99818	1.009843	1.0400926
22803	XRN2	5'-3' exoribonuclease 2	0.96751	0.984114	1.1562043

22806	IKZF3	IKAROS family zinc finger 3 (Aiolos)	0.93709	1.02581	1.0225416
22807	IKZF2	IKAROS family zinc finger 2 (Helios)	0.97994	1.046589	0.9868374
22808	MRAS	muscle RAS oncogene homolog	1.00852	1.006098	0.9603668
22809	ATF5	activating transcription factor 5	0.99421	1.046495	1.0283169
22815	TDGF4	teratocarcinoma-derived growth factor 4 (pseudogene)	0.98463	0.937398	1.0188217
22818	COPZ1	coatomer protein complex, subunit zeta 1	0.95609	0.948687	1.0357638
22820	COPG	coatomer protein complex, subunit gamma	0.97819	0.917299	1.0824655
22821	RASA3	RAS p21 protein activator 3	0.99082	0.996566	0.9733481
22822	PHLDA1	pleckstrin homology-like domain, family A, member 1	0.96997	1.039552	0.8439424
22823	MTF2	metal response element binding transcription factor 2	0.99087	0.944685	1.0355533
22824	HSPA4L	heat shock 70kDa protein 4-like	0.99892	0.967709	0.9918217
22826	DNAJC8	DnaJ (Hsp40) homolog, subfamily C, member 8	1.00621	0.979424	0.9991842
22827	PUF60	poly-U binding splicing factor 60KDa	0.9534	0.964096	1.0888339
22828	SCAF8	SR-related CTD-associated factor 8	0.96765	0.886223	0.8742097
22829	NLGN4Y	neuroligin 4, Y-linked	1.00008	1.00317	0.9920588
22832	KIAA1009	KIAA1009	0.97586	0.955072	1.004576
22834	ZNF652	zinc finger protein 652	0.96676	0.984447	0.9601344
22835	ZFP30	zinc finger protein 30 homolog (mouse)	0.96866	1.046689	1.0271484
22836	RHOBTB3	Rho-related BTB domain containing 3	1.0026	0.974093	1.0327329
22837	COBLL1	COBL-like 1	1.01892	1.030211	0.933132
22838	RNF44	ring finger protein 44	1.03109	1.038531	0.9576839
22839	DLGAP4	discs, large (Drosophila) homolog-associated protein 4	0.99313	1.044316	0.9816006
22841	RAB11FIP2	RAB11 family interacting protein 2 (class I)	0.96574	0.870324	1.1037743
22843	PPM1E	protein phosphatase, Mg2+/Mn2+ dependent, 1E	0.99581	0.998881	1.0116604
22844	FRMPD1	FERM and PDZ domain containing 1	0.99964	1.011947	1.0012625
22845	DOLK	dolichol kinase	0.94968	0.857739	1.0877503
22846	VASH1	vasohibin 1	1.00397	1.079784	0.961852
22847	ZNF507	zinc finger protein 507	0.91847	0.932388	1.0573331
22848	AAK1	AP2 associated kinase 1	0.99591	0.985441	0.9328891
22849	CPEB3	cytoplasmic polyadenylation element binding protein 3	0.99585	1.02339	0.9809015
22850	ADNP2	ADNP homeobox 2	0.89609	1.058454	0.9278973
22852	ANKRD26	ankyrin repeat domain 26	0.96592	0.961621	1.0267326
22853	LMTK2	lemur tyrosine kinase 2	1.01028	0.939852	0.9210555

22854	NTNG1	netrin G1	1.00518	1.00306	1.0264505
22856	CHSY1	chondroitin sulfate synthase 1	0.98646	1.037448	0.9784028
22858	ICK	intestinal cell (MAK-like) kinase	0.99233	0.928258	1.0463324
22859	LPHN1	latrophilin 1	0.97133	0.979881	0.9732793
22861	NLRP1	NLR family, pyrin domain containing 1	0.98083	1.039423	0.9527671
22862	FNDC3A	fibronectin type III domain containing 3A	0.94641	0.999644	1.0069527
22863	ATG14	ATG14 autophagy related 14 homolog (S. cerevisiae)	0.93936	0.888415	1.0168966
22864	R3HDM2	R3H domain containing 2	0.97602	0.917304	0.9464273
22865	SLITRK3	SLIT and NTRK-like family, member 3	1.01582	1.002896	1.0286612
22866	CNKSR2	connector enhancer of kinase suppressor of Ras 2	0.94891	0.955056	0.9425429
22868	FASTKD2	FAST kinase domains 2	0.99753	0.965056	1.0694933
22869	ZNF510	zinc finger protein 510	0.93274	0.989004	0.9349005
22870	PPP6R1	protein phosphatase 6, regulatory subunit 1	0.95575	0.961159	1.0044432
22871	NLGN1	neuroligin 1	1.0054	1.017398	1.0258324
22872	SEC31A	SEC31 homolog A (S. cerevisiae)	1.00178	1.054201	1.0464138
22873	DZIP1	DAZ interacting protein 1	0.9934	1.007603	0.994773
22874	PLEKHA6	pleckstrin homology domain containing, family A member 6	1.00841	1.007033	1.0072426
22875	ENPP4	ectonucleotide pyrophosphatase/phosphodiesterase 4 (putative)	1.01324	0.958432	1.0146202
22876	INPP5F	inositol polyphosphate-5-phosphatase F	0.97552	0.977568	0.954648
22877	MLXIP	MLX interacting protein	0.96879	0.975795	0.9331748
22878	TRAPPC8	trafficking protein particle complex 8	0.94318	0.978211	1.0386488
22879	MON1B	MON1 homolog B (yeast)	0.9145	1.034842	0.9274494
22880	MORC2	MORC family CW-type zinc finger 2	0.92537	0.993404	1.0646275
22881	ANKRD6	ankyrin repeat domain 6	1.00208	0.976255	1.0017565
22882	ZHX2	zinc fingers and homeoboxes 2	1.01635	1.032223	0.9070783
22883	CLSTN1	calsyntenin 1	1.00727	0.944047	0.9863857
22884	WDR37	WD repeat domain 37	0.95284	1.019162	1.013576
22885	ABLIM3	actin binding LIM protein family, member 3	0.99585	0.968131	0.9978453
22887	FOXJ3	forkhead box J3	0.99111	0.952113	0.9995877
22888	UBOX5	U-box domain containing 5	0.94991	1.026931	0.9620314
22889	KIAA0907	KIAA0907	0.98857	1.015265	0.9911219
22890	ZBTB1	zinc finger and BTB domain containing 1	0.93158	1.040388	0.9845748
22891	ZNF365	zinc finger protein 365	1.00093	0.99274	1.0018962

22893	BAHD1	bromo adjacent homology domain containing 1	0.94127	0.978346	1.0254298
22894	DIS3	DIS3 mitotic control homolog (S. cerevisiae)	0.94457	0.80961	1.1477027
22895	RPH3A	rabphilin 3A homolog (mouse)	1.00465	0.954342	1.0310074
22897	CEP164	centrosomal protein 164kDa	0.95909	0.976647	1.0034087
22898	DENND3	DENN/MADD domain containing 3	1.03087	1.093887	1.0055357
22899	ARHGEF15	Rho guanine nucleotide exchange factor (GEF) 15	1.0006	1.005882	1.0222345
22900	CARD8	caspase recruitment domain family, member 8	0.96064	0.922879	1.0530084
22901	ARSG	arylsulfatase G	0.97639	1.025643	0.9607418
22902	RUFY3	RUN and FYVE domain containing 3	1.00817	1.0114	1.0023634
22903	BTBD3	BTB (POZ) domain containing 3	0.98801	0.928774	1.0857423
22904	SBNO2	strawberry notch homolog 2 (Drosophila)	0.98042	1.039346	0.9893247
22905	EPN2	epsin 2	0.983	0.995437	1.0078038
22906	TRAK1	trafficking protein, kinesin binding 1	1.01844	1.010352	1.0324594
22907	DHX30	DEAH (Asp-Glu-Ala-His) box polypeptide 30	0.9898	0.946632	1.0491736
22908	SACM1L	SAC1 suppressor of actin mutations 1-like (yeast)	0.99132	1.022623	0.9958178
22909	FAN1	FANCD2/FANCI-associated nuclease 1	0.95808	0.961438	1.0906592
22911	WDR47	WD repeat domain 47	0.95932	0.994546	1.0429051
22913	RALY	RNA binding protein, autoantigenic (hnRNP-associated with lethal yellow homolog (mouse))	0.96693	1.031348	1.0085152
22915	MMRN1	multimerin 1	0.94259	0.870205	0.9868778
22916	NCBP2	nuclear cap binding protein subunit 2, 20kDa	0.9811	0.907972	1.0162324
22917	ZP1	zona pellucida glycoprotein 1 (sperm receptor)	1.01183	1.024566	0.9959668
22919	MAPRE1	microtubule-associated protein, RP/EB family, member 1	0.97111	0.980844	0.9684607
22920	KIFAP3	kinesin-associated protein 3	0.96505	0.973362	0.9933519
22921	MSRB2	methionine sulfoxide reductase B2	0.96332	1.00528	1.0692291
22924	MAPRE3	microtubule-associated protein, RP/EB family, member 3	1.00664	0.989397	0.985307
22925	PLA2R1	phospholipase A2 receptor 1, 180kDa	1.00947	1.003512	1.006768
22926	ATF6	activating transcription factor 6	0.97887	0.952212	0.9740645
22927	HABP4	hyaluronan binding protein 4	0.94716	1.073495	0.9181862
22928	SEPHS2	selenophosphate synthetase 2	0.9449	1.03221	1.0973493
22929	SEPHS1	selenophosphate synthetase 1	0.96062	0.969649	1.0188649
22930	RAB3GAP1	RAB3 GTPase activating protein subunit 1 (catalytic)	0.97106	0.952737	1.0441613
22931	RAB18	RAB18, member RAS oncogene family	0.97057	0.9918	1.0034707
22933	SIRT2	sirtuin 2	0.93448	0.944905	1.0177113

22934	RPIA	ribose 5-phosphate isomerase A	0.9663	0.884173	1.1310859
22936	ELL2	elongation factor, RNA polymerase II, 2	1.01803	0.980712	0.9519049
22937	SCAP	SREBF chaperone	0.99722	0.973916	1.0134279
22938	SNW1	SNW domain containing 1	0.94143	0.970058	0.9892541
22941	SHANK2	SH3 and multiple ankyrin repeat domains 2	0.9943	0.998983	1.0192197
22943	DKK1	dickkopf homolog 1 (Xenopus laevis)	0.98742	0.981104	1.0232826
22944	KIN	KIN, antigenic determinant of recA protein homolog (mouse)	0.95239	0.973726	1.0898104
22948	CCT5	chaperonin containing TCP1, subunit 5 (epsilon)	0.98848	0.911386	1.0183741
22949	PTGR1	prostaglandin reductase 1	0.9948	1.012044	1.0008134
22950	SLC4A1AP	solute carrier family 4 (anion exchanger), member 1, adaptor protein	0.98855	0.878809	1.0625423
22952	CYP2G1P	cytochrome P450, family 2, subfamily G, polypeptide 1 pseudogene	0.97389	1.081424	1.0196671
22953	P2RX2	purinergic receptor P2X, ligand-gated ion channel, 2	0.99583	0.976682	1.0066602
22954	TRIM32	tripartite motif containing 32	0.92665	0.985994	1.0251652
22955	SCMH1	sex comb on midleg homolog 1 (Drosophila)	0.99188	0.974745	0.9972046
22969	RPL41P3	ribosomal protein L41 pseudogene 3	0.96381	0.959649	0.9958327
22974	TPX2	TPX2, microtubule-associated, homolog (Xenopus laevis)	0.98739	0.979464	1.0227207
22976	PAXIP1	PAX interacting (with transcription-activation domain) protein 1	0.99863	0.968894	1.0196142
22977	AKR7A3	aldo-keto reductase family 7, member A3 (aflatoxin aldehyde reductase)	1.02861	0.99273	1.0047158
22978	NT5C2	5'-nucleotidase, cytosolic II	0.97491	1.01125	1.0376861
22979	EFR3B	EFR3 homolog B (S. cerevisiae)	1.00913	0.997884	1.0301803
22980	TCF25	transcription factor 25 (basic helix-loop-helix)	0.97185	0.977625	1.005149
22981	NINL	ninein-like	1.00692	0.988732	0.985017
22982	DIP2C	DIP2 disco-interacting protein 2 homolog C (Drosophila)	0.98722	1.00124	0.9762829
22983	MAST1	microtubule associated serine/threonine kinase 1	0.98649	1.033565	1.0423253
22984	PDCD11	programmed cell death 11	0.94497	0.930736	0.9783612
22985	ACIN1	apoptotic chromatin condensation inducer 1	0.94056	0.969835	1.0621655
22986	SORCS3	sortilin-related VPS10 domain containing receptor 3	0.98707	0.971918	0.9686269
22987	SV2C	synaptic vesicle glycoprotein 2C	1.00821	1.02969	1.0217312
22989	MYH15	myosin, heavy chain 15	1.00739	1.003778	1.0245229
22990	PCNX	pecanex homolog (Drosophila)	0.93502	1.01944	0.9801379
22992	KDM2A	lysine (K)-specific demethylase 2A	0.97878	0.988448	0.980585
22993	HMGXB3	HMG box domain containing 3	0.97154	0.929898	1.0306832
22994	AZI1	5-azacytidine induced 1	0.99977	0.988921	1.0551825

22995	CEP152	centrosomal protein 152kDa	0.98276	0.993988	1.0361993
22996	TTC39A	tetratricopeptide repeat domain 39A	1.00725	0.986316	1.0145385
22997	IGSF9B	immunoglobulin superfamily, member 9B	1.00427	0.984369	0.9795738
22998	LIMCH1	LIM and calponin homology domains 1	1.00228	1.013328	0.9989079
22999	RIMS1	regulating synaptic membrane exocytosis 1	1.01384	1.01247	1.0094344
23001	WDFY3	WD repeat and FYVE domain containing 3	1.01222	0.92818	0.9541898
23002	DAAM1	dishevelled associated activator of morphogenesis 1	0.96392	0.946398	0.9885363
23005	MAPKBP1	mitogen-activated protein kinase binding protein 1	1.00229	0.982667	1.0308378
23007	PLCH1	phospholipase C, eta 1	0.96597	0.932289	0.9719379
23008	KLHDC10	kelch domain containing 10	1.0071	0.960035	1.0642224
23011	RAB21	RAB21, member RAS oncogene family	0.96939	0.998959	1.0109265
23012	STK38L	serine/threonine kinase 38 like	0.98195	1.075252	1.0248582
23013	SPEN	spen homolog, transcriptional regulator (Drosophila)	0.9948	1.045375	0.9055424
23014	FBXO21	F-box protein 21	0.95915	0.873278	0.9878412
23016	EXOSC7	exosome component 7	0.9746	0.961164	1.0172328
23017	FAIM2	Fas apoptotic inhibitory molecule 2	1.00772	0.994941	1.0066887
23019	CNOT1	CCR4-NOT transcription complex, subunit 1	0.95838	1.003395	1.0339817
23020	SNRNP200	small nuclear ribonucleoprotein 200kDa (U5)	0.99297	1.052648	1.0566011
23022	PALLD	palladin, cytoskeletal associated protein	1.01152	1.013335	1.008085
23023	TMCC1	transmembrane and coiled-coil domain family 1	1.00192	0.966411	0.9402516
23024	PDZRN3	PDZ domain containing ring finger 3	1.00267	0.988022	0.9968188
23025	UNC13A	unc-13 homolog A (C. elegans)	0.9988	0.99943	1.0117258
23026	MYO16	myosin XVI	0.98764	1.002866	0.9999521
23028	KDM1A	lysine (K)-specific demethylase 1A	0.98238	1.00898	1.0703738
23029	RBM34	RNA binding motif protein 34	0.97597	0.925534	1.0199603
23030	KDM4B	lysine (K)-specific demethylase 4B	0.98319	1.056998	0.9773095
23031	MAST3	microtubule associated serine/threonine kinase 3	0.9837	1.057803	1.0344271
23032	USP33	ubiquitin specific peptidase 33	1.00512	0.938144	0.9515579
23033	DOPEY1	dopey family member 1	0.94616	0.944864	1.0246294
23034	SAMD4A	sterile alpha motif domain containing 4A	0.99354	0.99451	1.0061183
23035	PHLPP2	PH domain and leucine rich repeat protein phosphatase 2	0.92961	0.951261	1.0448679
23036	ZNF292	zinc finger protein 292	1.01438	0.926709	1.0409714
23037	PDZD2	PDZ domain containing 2	1.00389	0.99732	1.0067281

23038	WDTC1	WD and tetratricopeptide repeats 1	1.01755	0.940242	0.9769097
23039	XPO7	exportin 7	1.01075	0.969735	1.058555
23040	MYT1L	myelin transcription factor 1-like	0.99788	0.996078	1.0054838
23041	MON2	MON2 homolog (S. cerevisiae)	0.93118	0.957917	1.1436137
23042	PDXDC1	pyridoxal-dependent decarboxylase domain containing 1	0.97809	1.101582	1.0350945
23043	TNIK	TRAF2 and NCK interacting kinase	0.97148	0.976308	0.9657922
23046	KIF21B	kinesin family member 21B	0.99516	0.991993	1.0120525
23047	PDS5B	PDS5, regulator of cohesion maintenance, homolog B (S. cerevisiae)	0.95632	0.986422	1.0874461
23048	FNBP1	formin binding protein 1	0.97655	1.152428	1.0185826
23049	SMG1	SMG1 homolog, phosphatidylinositol 3-kinase-related kinase (C. elegans)	0.95312	1.062732	1.1405526
23051	ZHX3	zinc fingers and homeoboxes 3	0.98056	1.003295	0.997052
23052	ENDOD1	endonuclease domain containing 1	0.93172	0.981743	1.0810117
23053	KIAA0913	KIAA0913	0.98375	1.026627	0.9649386
23054	NCOA6	nuclear receptor coactivator 6	0.9694	0.951305	0.9767631
23057	NMNAT2	nicotinamide nucleotide adenylyltransferase 2	1.0097	1.00182	1.0118839
23059	CLUAP1	clusterin associated protein 1	0.94272	0.906581	1.0270419
23060	ZNF609	zinc finger protein 609	0.97816	1.00179	1.0141525
23061	TBC1D9B	TBC1 domain family, member 9B (with GRAM domain)	0.99761	0.909547	1.0521269
23062	GGA2	golgi-associated, gamma adaptin ear containing, ARF binding protein 2	0.94725	1.127193	0.9729481
23063	WAPAL	wings apart-like homolog (Drosophila)	0.94141	0.964155	0.9995331
23064	SETX	senataxin	0.9674	0.993557	1.0362583
23065	KIAA0090	KIAA0090	0.9595	0.909715	1.0481857
23066	CAND2	cullin-associated and neddylation-dissociated 2 (putative)	0.99608	1.002668	1.0054715
23067	SETD1B	SET domain containing 1B	0.9711	1.006072	0.9756843
23070	FTSJD2	FtsJ methyltransferase domain containing 2	0.97902	0.877476	1.0293223
23071	ERP44	endoplasmic reticulum protein 44	0.97066	1.021528	1.1063997
23072	HECW1	HECT, C2 and WW domain containing E3 ubiquitin protein ligase 1	1.00158	0.995267	1.0079309
23074	UHRF1BP1l	UHRF1 binding protein 1-like	0.94625	1.012568	0.9693871
23075	SWAP70	SWAP switching B-cell complex 70kDa subunit	0.95923	1.036442	1.0259598
23076	RRP1B	ribosomal RNA processing 1 homolog B (S. cerevisiae)	0.9264	0.896276	1.0145963
23077	MYCBP2	MYC binding protein 2	0.95446	0.911637	1.0554102
23078	KIAA0564	KIAA0564	0.95474	0.957739	0.9868245
23080	AVL9	AVL9 homolog (S. cerevisiase)	0.98728	1.028141	0.9799181

23081	KDM4C	lysine (K)-specific demethylase 4C	0.99157	0.999724	1.0429443
23082	PPRC1	peroxisome proliferator-activated receptor gamma, coactivator-related 1	0.96487	1.038696	1.0025926
23085	ERC1	ELKS/RAB6-interacting/CAST family member 1	0.95822	0.976561	0.9608243
23086	EXPH5	exophilin 5	0.97737	0.962544	0.9667833
23087	TRIM35	tripartite motif containing 35	0.95276	0.890066	1.0445354
23089	PEG10	paternally expressed 10	0.99574	0.993949	1.0148401
23090	ZNF423	zinc finger protein 423	0.99569	1.014637	1.0048364
23091	ZC3H13	zinc finger CCCH-type containing 13	0.92992	0.985905	1.0665674
23092	ARHGAP26	Rho GTPase activating protein 26	1.04003	0.969277	0.9816814
23093	TTLL5	tubulin tyrosine ligase-like family, member 5	0.97776	0.958375	1.042747
23094	SIPA1L3	signal-induced proliferation-associated 1 like 3	0.97697	1.003097	0.969855
23095	KIF1B	kinesin family member 1B	1.00284	0.982321	0.9520558
23096	IQSEC2	IQ motif and Sec7 domain 2	0.98649	0.979773	1.0091517
23097	CDK19	cyclin-dependent kinase 19	0.99917	1.008032	1.0416167
23098	SARM1	sterile alpha and TIR motif containing 1	0.97244	0.962212	1.0475509
23099	ZBTB43	zinc finger and BTB domain containing 43	1.03482	1.021256	1.0088652
23101	MCF2L2	MCF.2 cell line derived transforming sequence-like 2	1.01105	0.986476	0.9975452
23102	TBC1D2B	TBC1 domain family, member 2B	0.93323	1.031021	1.077015
23105	FSTL4	follistatin-like 4	1.00321	1.001315	1.0032722
23107	MRPS27	mitochondrial ribosomal protein S27	0.97824	0.929651	1.0023969
23108	RAP1GAP2	RAP1 GTPase activating protein 2	0.97339	0.980797	1.0140798
23109	DDN	dendrin	0.98846	1.006642	1.0187205
23111	SPG20	spastic paraplegia 20 (Troyer syndrome)	0.95536	0.973203	0.9511395
23112	TNRC6B	trinucleotide repeat containing 6B	0.97521	0.978973	0.9418617
23113	CUL9	cullin 9	0.96516	0.972335	0.9663365
23114	NFASC	neurofascin	1.00264	1.005175	1.0034169
23116	FAM179B	family with sequence similarity 179, member B	0.95029	1.006678	1.1043394
23118	TAB2	TGF-beta activated kinase 1/MAP3K7 binding protein 2	1.00289	0.910599	0.9714251
23119	HIC2	hypermethylated in cancer 2	0.99106	1.017869	0.9161952
23120	ATP10B	ATPase, class V, type 10B	1.01024	0.996478	1.0142715
23122	CLASP2	cytoplasmic linker associated protein 2	0.9869	0.991591	0.9547504
23125	CAMTA2	calmodulin binding transcription activator 2	0.99475	0.978869	1.0022244
23126	POGZ	pogo transposable element with ZNF domain	0.97236	0.977919	1.027201

23127	GLT25D2	glycosyltransferase 25 domain containing 2	1.00044	1.013913	1.0093441
23129	PLXND1	plexin D1	0.9885	0.969869	1.0540768
23130	ATG2A	ATG2 autophagy related 2 homolog A (S. cerevisiae)	0.96985	1.042737	0.9183611
23131	GPATCH8	G patch domain containing 8	0.94768	0.885538	0.9544085
23132	RAD54L2	RAD54-like 2 (S. cerevisiae)	0.98954	0.977139	1.0302252
23133	PHF8	PHD finger protein 8	0.93456	1.105424	0.9588153
23135	KDM6B	lysine (K)-specific demethylase 6B	0.9671	1.09786	0.9489256
23136	EPB41L3	erythrocyte membrane protein band 4.1-like 3	0.95544	1.053259	1.0006149
23137	SMC5	structural maintenance of chromosomes 5	0.93524	0.973453	1.0929858
23138	N4BP3	NEDD4 binding protein 3	0.98808	1.03235	1.0356257
23139	MAST2	microtubule associated serine/threonine kinase 2	1.00643	0.944623	1.0226519
23140	ZZEF1	zinc finger, ZZ-type with EF-hand domain 1	0.97549	1.003445	0.9608905
23141	ANKLE2	ankyrin repeat and LEM domain containing 2	0.93822	1.071066	1.0435141
23142	DCUN1D4	DCN1, defective in cullin neddylation 1, domain containing 4 (S. cerevisiae)	0.96674	1.034215	1.0391361
23143	LRCH1	leucine-rich repeats and calponin homology (CH) domain containing 1	0.9757	1.013607	1.0911939
23144	ZC3H3	zinc finger CCCH-type containing 3	1.00008	0.955444	0.9947809
23145	SSPO	SCO-spondin homolog (Bos taurus)	0.99932	1.018543	0.9971865
23148	NACAD	NAC alpha domain containing	1.01237	1.015487	1.0186491
23149	FCHO1	FCH domain only 1	0.93324	0.998926	1.0779044
23150	FRMD4B	FERM domain containing 4B	0.97751	0.946249	1.0086434
23151	GRAMD4	GRAM domain containing 4	0.96857	0.891148	1.0940304
23152	CIC	capicua homolog (Drosophila)	0.98717	1.04814	0.9515729
23154	NCDN	neurochondrin	1.00226	0.9826	0.9803235
23155	CLCC1	chloride channel CLIC-like 1	0.97468	1.024863	0.9465874
23157	6-Sep	septin 6	0.97116	0.941603	0.9526481
23158	TBC1D9	TBC1 domain family, member 9 (with GRAM domain)	1.01922	0.973169	0.9399666
23160	WDR43	WD repeat domain 43	0.97912	0.883612	1.0305775
23161	SNX13	sorting nexin 13	0.98899	1.06301	0.9823701
23162	MAPK8IP3	mitogen-activated protein kinase 8 interacting protein 3	0.99447	1.001835	0.9798133
23163	GGA3	golgi-associated, gamma adaptin ear containing, ARF binding protein 3	0.97895	0.988697	0.9904898
23164	MPRIP	myosin phosphatase Rho interacting protein	0.9836	1.001719	1.0183223
23165	NUP205	nucleoporin 205kDa	0.96915	0.971559	1.0562577
23166	STAB1	stabilin 1	1.03701	0.931983	0.9972827

23167	EFR3A	EFR3 homolog A (S. cerevisiae)	0.99617	0.980272	1.042507
23168	RTF1	Rtf1, Paf1/RNA polymerase II complex component, homolog (S. cerevisiae)	0.92306	0.931281	0.973161
23169	SLC35D1	solute carrier family 35 (UDP-glucuronic acid/UDP-N-acetylgalactosamine dual transporter), member D1	1.01063	0.948303	0.9952487
23170	TTLL12	tubulin tyrosine ligase-like family, member 12	0.97639	0.872591	1.0675775
23171	GPD1L	glycerol-3-phosphate dehydrogenase 1-like	0.9784	0.947221	0.9725824
23172	FAM175B	family with sequence similarity 175, member B	0.9049	0.976573	1.0823634
23173	METAP1	methionyl aminopeptidase 1	0.99326	0.903343	1.0553449
23174	ZCCHC14	zinc finger, CCHC domain containing 14	0.95302	0.975792	0.9232371
23175	LPIN1	lipin 1	0.95755	1.084597	0.9545968
23176	8-Sep	septin 8	0.982	0.976717	1.0405086
23177	CEP68	centrosomal protein 68kDa	0.99376	0.938694	0.9488858
23178	PASK	PAS domain containing serine/threonine kinase	0.97366	0.938935	1.0118091
23179	RGL1	ral guanine nucleotide dissociation stimulator-like 1	1.01449	0.991588	0.9904485
23180	RFTN1	raftlin, lipid raft linker 1	1.00364	1.021929	1.0467297
23181	DIP2A	DIP2 disco-interacting protein 2 homolog A (Drosophila)	0.98657	1.056143	0.9293293
23184	MESDC2	mesoderm development candidate 2	0.94539	1.038891	1.0866448
23185	LARP4B	La ribonucleoprotein domain family, member 4B	0.95219	0.947383	1.1171047
23186	RCOR1	REST corepressor 1	0.9703	0.969153	0.9744009
23187	PHLDB1	pleckstrin homology-like domain, family B, member 1	0.99113	1.020731	1.0047334
23189	KANK1	KN motif and ankyrin repeat domains 1	0.96883	0.971607	0.9370173
23190	UBXN4	UBX domain protein 4	1.01309	0.945502	0.9506349
23191	CYFIP1	cytoplasmic FMR1 interacting protein 1	0.94678	0.931495	1.0957898
23192	ATG4B	ATG4 autophagy related 4 homolog B (S. cerevisiae)	0.99727	0.940374	0.9474875
23193	GANAB	glucosidase, alpha; neutral AB	0.97103	0.941041	1.0713004
23194	FBXL7	F-box and leucine-rich repeat protein 7	1.00874	1.013372	1.0058917
23195	MDN1	MDN1, midasin homolog (yeast)	0.9642	0.998543	1.0229296
23196	FAM120A	family with sequence similarity 120A	0.98262	1.023975	1.0026455
23197	FAF2	Fas associated factor family member 2	0.98487	0.923055	0.9783731
23198	PSME4	proteasome (prosome, macropain) activator subunit 4	1.00046	1.042052	1.0266057
23199	KIAA0182	KIAA0182	0.96678	1.04741	0.9753307
23200	ATP11B	ATPase, class VI, type 11B	0.99574	1.033618	1.012818
23201	FAM168A	family with sequence similarity 168, member A	0.98144	1.032193	1.056037
23203	PMPCA	peptidase (mitochondrial processing) alpha	0.97836	0.977847	1.0254227

23204	ARL6IP1	ADP-ribosylation factor-like 6 interacting protein 1	0.98557	0.985636	1.0160541
23205	ACSBG1	acyl-CoA synthetase bubblegum family member 1	0.9786	0.987636	0.9884645
23207	PLEKHM2	pleckstrin homology domain containing, family M (with RUN domain) member 2	1.01367	1.043217	0.9262367
23208	SYT11	synaptotagmin XI	1.02091	1.028009	1.0872457
23209	MLC1	megalencephalic leukoencephalopathy with subcortical cysts 1	0.98185	0.972426	1.0174191
23210	JMJD6	jumonji domain containing 6	0.94584	1.018349	1.0035628
23211	ZC3H4	zinc finger CCCH-type containing 4	0.96092	1.013284	0.9812136
23212	RRS1	RRS1 ribosome biogenesis regulator homolog (S. cerevisiae)	0.95255	1.035173	1.0249868
23213	SULF1	sulfatase 1	1.0036	0.98964	1.0118645
23214	XPO6	exportin 6	0.95106	1.000286	0.9439307
23215	PRRC2C	proline-rich coiled-coil 2C	1.00091	1.010214	0.9482163
23216	TBC1D1	TBC1 (tre-2/USP6, BUB2, cdc16) domain family, member 1	1.01205	0.947153	0.9979963
23217	ZFR2	zinc finger RNA binding protein 2	0.99212	1.019019	1.0298427
23218	NBEAL2	neurobeachin-like 2	1.0419	1.081465	1.0253623
23219	FBXO28	F-box protein 28	0.98057	1.00029	1.0451695
23220	DTX4	deltex homolog 4 (Drosophila)	0.98609	1.022811	1.0073032
23221	RHOBTB2	Rho-related BTB domain containing 2	0.97474	0.915556	0.95599
23223	RRP12	ribosomal RNA processing 12 homolog (S. cerevisiae)	0.97775	1.000218	1.0270751
23224	SYNE2	spectrin repeat containing, nuclear envelope 2	0.96037	0.956427	1.0507604
23225	NUP210	nucleoporin 210kDa	1.00588	0.957591	1.0083656
23228	PLCL2	phospholipase C-like 2	1.016	0.978473	0.9356524
23229	ARHGEF9	Cdc42 guanine nucleotide exchange factor (GEF) 9	0.95729	0.97166	1.0018283
23230	VPS13A	vacuolar protein sorting 13 homolog A (S. cerevisiae)	0.9528	1.002841	1.0893667
23231	SEL1L3	sel-1 suppressor of lin-12-like 3 (C. elegans)	0.9964	1.015581	0.8705874
23232	TBC1D12	TBC1 domain family, member 12	0.97181	0.947056	0.953123
23233	EXOC6B	exocyst complex component 6B	0.98634	0.9185	0.950065
23234	DNAJC9	DnaJ (Hsp40) homolog, subfamily C, member 9	0.9927	0.939757	1.1868442
23235	SIK2	salt-inducible kinase 2	0.97612	1.037053	1.0664557
23236	PLCB1	phospholipase C, beta 1 (phosphoinositide-specific)	0.98924	0.933577	1.017019
23237	ARC	activity-regulated cytoskeleton-associated protein	0.98948	1.00173	0.9803421
23239	PHLPP1	PH domain and leucine rich repeat protein phosphatase 1	0.96492	1.032706	0.9866608
23240	KIAA0922	KIAA0922	0.96174	0.988561	1.0525228
23241	PACS2	phosphofurin acidic cluster sorting protein 2	0.97864	0.977271	1.0154868

23242	COBL	cordon-bleu homolog (mouse)	1.00484	0.989595	1.003656
23243	ANKRD28	ankyrin repeat domain 28	0.97944	0.983533	1.1207234
23244	PDS5A	PDS5, regulator of cohesion maintenance, homolog A (S. cerevisiae)	0.98823	1.003159	0.9912686
23245	ASTN2	astrotactin 2	0.99716	0.988384	0.9925076
23246	BOP1	block of proliferation 1	1.00412	0.979658	1.0615685
23247	KIAA0556	KIAA0556	0.97776	0.996075	0.9988309
23248	RPRD2	regulation of nuclear pre-mRNA domain containing 2	0.97067	0.935634	0.990935
23250	ATP11A	ATPase, class VI, type 11A	0.962	0.915554	1.0171569
23251	KIAA1024	KIAA1024	1.00261	0.994506	1.0232452
23252	OTUD3	OTU domain containing 3	0.98246	0.921309	1.073384
23253	ANKRD12	ankyrin repeat domain 12	0.94827	1.056876	0.9165103
23255	CCDC165	coiled-coil domain containing 165	0.99047	0.999908	0.9808224
23256	SCFD1	sec1 family domain containing 1	0.95377	0.931417	1.0697145
23258	DENND5A	DENN/MADD domain containing 5A	0.98925	1.112949	0.9769019
23259	DDHD2	DDHD domain containing 2	0.97931	0.924571	1.0353432
23261	CAMTA1	calmodulin binding transcription activator 1	1.00547	0.98371	0.9935189
23262	PPIP5K2	diphosphoinositol pentakisphosphate kinase 2	0.99877	0.819595	0.9734129
23263	MCF2L	MCF.2 cell line derived transforming sequence-like	0.97746	0.997652	1.0118524
23264	ZC3H7B	zinc finger CCCH-type containing 7B	0.95401	0.947504	0.995205
23265	EXOC7	exocyst complex component 7	0.94175	0.888913	1.0467458
23266	LPHN2	latrophilin 2	1.0094	1.006954	1.0222601
23268	DNMBP	dynamin binding protein	0.99178	1.006509	0.9895165
23269	MGA	MAX gene associated	0.94897	0.989405	0.9791826
23270	TSPYL4	TSPY-like 4	0.97135	0.919903	0.9976311
23271	CAMSAP1L	calmodulin regulated spectrin-associated protein 1-like 1	0.97951	0.95472	0.9892963
23272	C3orf63	chromosome 3 open reading frame 63	1.00039	0.949921	1.0072752
23274	CLEC16A	C-type lectin domain family 16, member A	0.98073	0.989054	1.0407314
23275	POFUT2	protein O-fucosyltransferase 2	0.9699	1.046308	0.9712614
23276	KLHL18	kelch-like 18 (Drosophila)	1.00158	0.978483	1.0206792
23277	KIAA0664	KIAA0664	0.96368	0.947998	1.0622077
23279	NUP160	nucleoporin 160kDa	0.93502	1.015183	0.9859471
23281	MTUS2	microtubule associated tumor suppressor candidate 2	0.99655	0.999356	1.0033704
23283	CSTF2T	cleavage stimulation factor, 3' pre-RNA, subunit 2, 64kDa, tau variant	0.91999	0.912979	1.0689249

23284	LPHN3	latrophilin 3	1.00859	1.007847	1.0252141
23286	WWC1	WW and C2 domain containing 1	1.00756	0.999905	1.0176473
23287	AGTPBP1	ATP/GTP binding protein 1	0.98333	0.968066	1.0348754
23288	IQCE	IQ motif containing E	1.01517	0.996411	1.000214
23291	FBXW11	F-box and WD repeat domain containing 11	0.99986	1.02043	0.9920533
23293	SMG6	Smg-6 homolog, nonsense mediated mRNA decay factor (C. elegans)	0.98913	0.988974	0.9521124
23294	ANKS1A	ankyrin repeat and sterile alpha motif domain containing 1A	0.99197	0.972553	1.0283524
23295	MGRN1	mahogunin, ring finger 1	0.96327	1.004287	1.005803
23299	BICD2	bicaudal D homolog 2 (Drosophila)	0.96253	0.967264	1.0047963
23300	ATMIN	ATM interactor	0.90423	0.876761	1.0479133
23301	EHBP1	EH domain binding protein 1	0.98576	0.951475	1.0322027
23302	WSCD1	WSC domain containing 1	0.9873	0.996388	1.0221221
23303	KIF13B	kinesin family member 13B	0.9747	1.012193	0.9812793
23304	UBR2	ubiquitin protein ligase E3 component n-recognin 2	1.00076	0.93906	0.8680601
23305	ACSL6	acyl-CoA synthetase long-chain family member 6	0.9862	0.979448	1.0022936
23306	TMEM194A	transmembrane protein 194A	0.95633	0.981878	1.0716193
23307	FKBP15	FK506 binding protein 15, 133kDa	0.99504	0.951363	1.0398472
23308	ICOSLG	inducible T-cell co-stimulator ligand	0.98952	1.068045	0.958248
23309	SIN3B	SIN3 homolog B, transcription regulator (yeast)	0.98306	0.992073	1.039997
23310	NCAPD3	non-SMC condensin II complex, subunit D3	0.92075	0.954324	1.0153834
23312	DMXL2	Dmx-like 2	1.02027	1.064636	0.9662892
23313	KIAA0930	KIAA0930	0.99111	0.961249	1.0291359
23314	SATB2	SATB homeobox 2	1.0062	0.998838	1.0317777
23315	SLC9A8	solute carrier family 9 (sodium/hydrogen exchanger), member 8	0.98	1.096289	0.9848858
23316	CUX2	cut-like homeobox 2	0.993	0.995666	1.0076357
23317	DNAJC13	DnaJ (Hsp40) homolog, subfamily C, member 13	0.99814	0.922115	1.1115646
23318	ZCCHC11	zinc finger, CCHC domain containing 11	0.97387	0.979688	0.9161295
23321	TRIM2	tripartite motif containing 2	1.01069	0.989139	1.0255412
23322	RPGRIP1L	RPGRIP1-like	0.98366	0.983044	1.0021878
23324	MAN2B2	mannosidase, alpha, class 2B, member 2	0.95251	0.864431	0.9803867
23325	KIAA1033	KIAA1033	0.97802	1.06177	1.1127167
23326	USP22	ubiquitin specific peptidase 22	0.98862	0.910675	0.9534121
23327	NEDD4L	neural precursor cell expressed, developmentally down-regulated 4-like	0.99313	0.995705	1.015839

23328	SASH1	SAM and SH3 domain containing 1	1.00009	0.978772	1.0369915
23329	TBC1D30	TBC1 domain family, member 30	0.99008	1.005024	0.9984521
23331	TTC28	tetratricopeptide repeat domain 28	0.98606	0.985378	0.9871758
23332	CLASP1	cytoplasmic linker associated protein 1	0.9808	1.004493	1.0251491
23333	DPY19L1	dpy-19-like 1 (C. elegans)	0.99168	0.976643	0.9725099
23334	KIAA0467	KIAA0467	0.97858	0.945008	0.8713838
23335	WDR7	WD repeat domain 7	0.95487	0.943442	0.9688889
23336	SYNM	synemin, intermediate filament protein	0.97832	0.981297	0.9763927
23338	PHF15	PHD finger protein 15	0.99407	0.911147	0.9624538
23339	VPS39	vacuolar protein sorting 39 homolog (S. cerevisiae)	0.9855	0.957755	1.0653205
23341	DNAJC16	DnaJ (Hsp40) homolog, subfamily C, member 16	0.99816	0.965665	1.063293
23344	ESYT1	extended synaptotagmin-like protein 1	0.93686	0.924997	1.0403376
23345	SYNE1	spectrin repeat containing, nuclear envelope 1	1.0203	0.973925	0.9256626
23347	SMCHD1	structural maintenance of chromosomes flexible hinge domain containing 1	0.94566	1.14974	1.0146439
23348	DOCK9	dedicator of cytokinesis 9	0.92718	0.930031	0.9282771
23349	KIAA1045	KIAA1045	0.99934	0.981877	0.9952458
23350	U2SURP	U2 snRNP-associated SURP domain containing	1.01277	0.856177	1.0289773
23351	KHNYN	KH and NYN domain containing	0.9511	1.064531	0.9416066
23352	UBR4	ubiquitin protein ligase E3 component n-recognin 4	0.99156	1.187268	0.9918207
23353	SUN1	Sad1 and UNC84 domain containing 1	1.01533	0.999271	1.0068332
23354	HAUS5	HAUS augmin-like complex, subunit 5	0.97043	1.022759	1.0072144
23355	VPS8	vacuolar protein sorting 8 homolog (S. cerevisiae)	0.98277	1.037606	0.9110508
23357	ANGEL1	angel homolog 1 (Drosophila)	0.98597	0.920535	1.0026281
23358	USP24	ubiquitin specific peptidase 24	0.98847	1.09744	0.9700406
23360	FNBP4	formin binding protein 4	0.91256	1.082035	0.9197796
23361	ZNF629	zinc finger protein 629	0.95789	0.994696	1.0771118
23362	PSD3	pleckstrin and Sec7 domain containing 3	0.9835	1.001338	1.007843
23363	OBSL1	obscurin-like 1	0.99312	1.012806	1.0260975
23365	ARHGEF12	Rho guanine nucleotide exchange factor (GEF) 12	0.97849	0.92524	1.0919659
23366	KIAA0895	KIAA0895	1.01055	0.973077	1.0156679
23367	LARP1	La ribonucleoprotein domain family, member 1	1.00732	1.018494	1.0543055
23368	PPP1R13B	protein phosphatase 1, regulatory (inhibitor) subunit 13B	0.97591	1.011022	0.9568445
23369	PUM2	pumilio homolog 2 (Drosophila)	0.98788	0.94092	1.0593198

23370	ARHGEF18	Rho/Rac guanine nucleotide exchange factor (GEF) 18	0.96245	0.942183	0.9325228
23371	TENC1	tensin like C1 domain containing phosphatase (tensin 2)	1.00628	1.009727	1.0027948
23373	CRTC1	CREB regulated transcription coactivator 1	0.98742	1.002678	1.0048595
23376	KIAA0776	KIAA0776	1.01039	0.958174	1.0026487
23378	RRP8	ribosomal RNA processing 8, methyltransferase, homolog (yeast)	0.96966	1.05663	1.0252909
23379	KIAA0947	KIAA0947	0.96892	0.849796	1.0839501
23380	SRGAP2	SLIT-ROBO Rho GTPase activating protein 2	1.03689	0.917058	0.9456801
23381	SMG5	Smg-5 homolog, nonsense mediated mRNA decay factor (C. elegans)	1.00604	0.93862	1.0455957
23382	AHCYL2	adenosylhomocysteinase-like 2	0.99635	0.973259	1.0419272
23383	MAU2	MAU2 chromatid cohesion factor homolog (C. elegans)	0.98171	0.950485	0.9693541
23384	SPECC1L	sperm antigen with calponin homology and coiled-coil domains 1-like	0.94201	1.009294	1.0284898
23385	NCSTN	nicastrin	1.0181	0.874277	1.0746163
23386	NUDCD3	NudC domain containing 3	0.97337	0.948384	1.0131089
23387	SIK3	SIK family kinase 3	0.98139	1.042252	0.940236
23389	MED13L	mediator complex subunit 13-like	0.99084	1.072705	1.0294882
23390	ZDHHC17	zinc finger, DHHC-type containing 17	0.96659	0.981756	1.0693247
23392	KIAA0368	KIAA0368	0.98084	1.055519	1.1041407
23394	ADNP	activity-dependent neuroprotector homeobox	0.98546	0.999844	1.0177582
23395	LARS2	leucyl-tRNA synthetase 2, mitochondrial	0.96662	0.876716	1.0015859
23396	PIP5K1C	phosphatidylinositol-4-phosphate 5-kinase, type I, gamma	0.96556	1.059178	0.98488
23397	NCAPH	non-SMC condensin I complex, subunit H	1.00192	1.007218	1.0158118
23398	PPWD1	peptidylprolyl isomerase domain and WD repeat containing 1	0.97158	0.960487	0.8949135
23399	CTDNEP1	CTD nuclear envelope phosphatase 1	1.02878	1.029864	0.9945204
23400	ATP13A2	ATPase type 13A2	1.00208	0.997207	1.0026614
23401	FRAT2	frequently rearranged in advanced T-cell lymphomas 2	0.95759	0.978026	0.9690923
23403	FBXO46	F-box protein 46	0.94861	1.005836	1.0471939
23404	EXOSC2	exosome component 2	0.92783	1.021929	1.0527852
23405	DICER1	dicer 1, ribonuclease type III	0.96647	1.034963	1.1908943
23406	COTL1	coactosin-like 1 (Dictyostelium)	1.03357	0.975865	1.0175018
23408	SIRT5	sirtuin 5	1.02224	1.000795	1.0064313
23409	SIRT4	sirtuin 4	0.99842	0.996039	1.0103332
23410	SIRT3	sirtuin 3	0.99112	0.986557	1.0085421
23411	SIRT1	sirtuin 1	0.93119	0.889282	1.0077024

23412	COMMD3	COMM domain containing 3	0.94107	0.965077	1.0660482
23413	NCS1	neuronal calcium sensor 1	0.98917	1.000186	1.0172383
23414	ZFPM2	zinc finger protein, multitype 2	1.01077	1.011545	1.0127628
23415	KCNH4	potassium voltage-gated channel, subfamily H (eag-related), member 4	0.99772	1.009502	1.0030482
23416	KCNH3	potassium voltage-gated channel, subfamily H (eag-related), member 3	0.98934	0.99108	1.0054386
23417	MLYCD	malonyl-CoA decarboxylase	0.95564	1.001552	1.037056
23418	CRB1	crumbs homolog 1 (Drosophila)	1.00737	0.989926	1.0041841
23421	ITGB3BP	integrin beta 3 binding protein (beta3-endonexin)	0.99747	1.025239	0.9691812
23424	TDRD7	tudor domain containing 7	0.96232	1.053723	1.0608367
23426	GRIP1	glutamate receptor interacting protein 1	0.99758	0.988019	0.9896917
23428	SLC7A8	solute carrier family 7 (amino acid transporter, L-type), member 8	1.00328	0.97831	0.9698578
23429	RYBP	RING1 and YY1 binding protein	1.03643	0.952135	0.9641157
23430	TPSD1	tryptase delta 1	0.96288	1.010367	1.0524129
23431	AP4E1	adaptor-related protein complex 4, epsilon 1 subunit	0.95675	1.00007	1.0840238
23432	GPR161	G protein-coupled receptor 161	1.00832	0.977746	0.9885493
23433	RHOQ	ras homolog gene family, member Q	1.05065	1.074248	0.8910114
23434	C3orf27	chromosome 3 open reading frame 27	0.98532	0.970368	0.9669545
23435	TARDBP	TAR DNA binding protein	1.06186	0.989432	0.9136399
23436	CELA3B	chymotrypsin-like elastase family, member 3B	1.02712	1.026451	1.008277
23438	HARS2	histidyl-tRNA synthetase 2, mitochondrial (putative)	0.9763	1.080139	1.0421281
23439	ATP1B4	ATPase, Na+/K+ transporting, beta 4 polypeptide	1.01498	0.993951	1.0058458
23440	OTP	orthopedia homeobox	0.9972	0.980892	1.0546539
23443	SLC35A3	solute carrier family 35 (UDP-N-acetylglucosamine (UDP-GlcNAc) transporter), member A3	1.00257	0.982424	1.0507816
23446	SLC44A1	solute carrier family 44, member 1	0.97308	1.038858	1.0311913
23450	SF3B3	splicing factor 3b, subunit 3, 130kDa	0.94371	0.987204	1.0558934
23451	SF3B1	splicing factor 3b, subunit 1, 155kDa	1.01976	0.903638	1.0400328
23452	ANGPTL2	angiopoietin-like 2	1.01845	0.973998	1.0117319
23456	ABCB10	ATP-binding cassette, sub-family B (MDR/TAP), member 10	0.99489	0.999165	1.0396575
23457	ABCB9	ATP-binding cassette, sub-family B (MDR/TAP), member 9	0.98147	0.985166	1.0173827
23460	ABCA6	ATP-binding cassette, sub-family A (ABC1), member 6	0.98929	0.986626	1.008699
23461	ABCA5	ATP-binding cassette, sub-family A (ABC1), member 5	0.93366	0.898877	1.051532
23462	HEY1	hairy/enhancer-of-split related with YRPW motif 1	1.00154	1.001275	0.9985148
23463	ICMT	isoprenylcysteine carboxyl methyltransferase	0.94642	0.94049	1.1209452

23464	GCAT	glycine C-acetyltransferase	1.00325	0.995656	0.9834218
23466	CBX6	chromobox homolog 6	0.95798	1.074534	0.9509334
23467	NPTXR	neuronal pentraxin receptor	1.00023	0.974627	1.0154011
23468	CBX5	chromobox homolog 5	0.95774	0.972762	0.9352825
23469	PHF3	PHD finger protein 3	0.98826	0.931976	1.0013102
23471	TRAM1	translocation associated membrane protein 1	0.97289	1.034718	1.0645038
23473	CAPN7	calpain 7	0.97967	0.92905	0.9358481
23474	ETHE1	ethylmalonic encephalopathy 1	0.95617	1.011849	1.0607635
23475	QPRT	quinolinate phosphoribosyltransferase	1.0135	0.991088	1.0139394
23476	BRD4	bromodomain containing 4	0.97454	1.117985	0.9471146
23478	SEC11A	SEC11 homolog A (S. cerevisiae)	0.98551	0.972648	0.9988342
23479	ISCU	iron-sulfur cluster scaffold homolog (E. coli)	0.9423	0.94547	1.0295407
23480	SEC61G	Sec61 gamma subunit	0.96755	0.919226	0.9959845
23481	PES1	pescadillo homolog 1, containing BRCT domain (zebrafish)	0.92458	0.917289	1.026791
23483	TGDS	TDP-glucose 4,6-dehydratase	0.93015	1.016556	1.071326
23484	LEPROTL1	leptin receptor overlapping transcript-like 1	0.97325	0.905564	0.9802932
23491	CES3	carboxylesterase 3	0.99909	1.043996	0.9896696
23492	CBX7	chromobox homolog 7	0.93282	1.000838	0.897511
23493	HEY2	hairy/enhancer-of-split related with YRPW motif 2	0.99705	0.961564	1.0040055
23495	TNFRSF13B	tumor necrosis factor receptor superfamily, member 13B	0.9923	0.968889	1.0155477
23498	HAAO	3-hydroxyanthranilate 3,4-dioxygenase	1.03725	1.005687	1.0275537
23499	MACF1	microtubule-actin crosslinking factor 1	0.99844	0.954488	1.0104151
23500	DAAM2	dishevelled associated activator of morphogenesis 2	1.00193	0.998354	1.016872
23503	ZFYVE26	zinc finger, FYVE domain containing 26	0.95827	0.943876	0.984157
23504	RIMBP2	RIMS binding protein 2	1.00333	1.001644	1.0203068
23505	TMEM131	transmembrane protein 131	1.0079	1.070822	0.9812763
23506	KIAA0240	KIAA0240	1.0011	0.940414	1.0223444
23507	LRRC8B	leucine rich repeat containing 8 family, member B	0.94163	0.950224	0.9410934
23508	TTC9	tetratricopeptide repeat domain 9	0.93884	1.002227	1.0509402
23509	POFUT1	protein O-fucosyltransferase 1	0.96063	0.961868	1.0057084
23510	KCTD2	potassium channel tetramerisation domain containing 2	0.9411	0.93408	1.0474168
23511	NUP188	nucleoporin 188kDa	0.93695	0.980445	1.0105614
23512	SUZ12	suppressor of zeste 12 homolog (Drosophila)	0.95133	0.988507	1.0221377

23513	SCRIB	scribbled homolog (Drosophila)	0.9885	0.955724	1.1008785
23514	KIAA0146	KIAA0146	0.99868	0.995841	0.9820816
23515	MORC3	MORC family CW-type zinc finger 3	0.92434	0.941596	1.0258348
23516	SLC39A14	solute carrier family 39 (zinc transporter), member 14	0.99164	0.985949	1.0231783
23517	SKIV2L2	superkiller viralicidic activity 2-like 2 (S. cerevisiae)	0.99574	0.943662	1.0744046
23518	R3HDM1	R3H domain containing 1	0.98683	0.97534	0.9571446
23519	ANP32D	acidic (leucine-rich) nuclear phosphoprotein 32 family, member D	0.99645	0.997097	0.9812662
23520	ANP32C	acidic (leucine-rich) nuclear phosphoprotein 32 family, member C	0.98521	1.084227	1.0741172
23521	RPL13A	ribosomal protein L13a	1.01278	1.01444	1.0509308
23522	MYST4	MYST histone acetyltransferase (monocytic leukemia) 4	0.95604	0.977942	1.0847789
23523	CABIN1	calcineurin binding protein 1	0.97631	0.957078	1.0206955
23524	SRRM2	serine/arginine repetitive matrix 2	1.03471	1.05175	0.9607549
23526	HMHA1	histocompatibility (minor) HA-1	0.96651	0.976892	0.9290942
23527	ACAP2	ArfGAP with coiled-coil, ankyrin repeat and PH domains 2	1.01681	1.00677	1.0183301
23528	ZNF281	zinc finger protein 281	0.98496	0.986377	1.033182
23529	CLCF1	cardiotrophin-like cytokine factor 1	0.99415	1.038758	0.9632386
23530	NNT	nicotinamide nucleotide transhydrogenase	1.00243	0.88379	1.0399861
23531	MMD	monocyte to macrophage differentiation-associated	0.94804	1.015861	0.9307428
23532	PRAME	preferentially expressed antigen in melanoma	0.99384	1.031983	1.0016655
23533	PIK3R5	phosphoinositide-3-kinase, regulatory subunit 5	0.96155	1.123306	0.9452226
23534	TNPO3	transportin 3	0.9819	0.919566	1.0854825
23536	ADAT1	adenosine deaminase, tRNA-specific 1	0.94895	0.96629	0.9779688
23538	OR52A1	olfactory receptor, family 52, subfamily A, member 1	1.02919	0.98592	1.0016189
23539	SLC16A8	solute carrier family 16, member 8 (monocarboxylic acid transporter 3)	1.00217	0.968957	1.083186
23541	SEC14L2	SEC14-like 2 (S. cerevisiae)	0.95287	1.028545	0.8640666
23542	MAPK8IP2	mitogen-activated protein kinase 8 interacting protein 2	0.99272	1.004596	1.0388546
23543	RBFOX2	RNA binding protein, fox-1 homolog (C. elegans) 2	0.99328	0.983699	0.998009
23544	SEZ6L	seizure related 6 homolog (mouse)-like	1.00927	0.9835	0.9830397
23545	ATP6V0A2	ATPase, H+ transporting, lysosomal V0 subunit a2	0.94758	0.942514	1.0470399
23546	SYNGR4	synaptogyrin 4	1.01436	1.000172	0.9938645
23547	LILRA4	leukocyte immunoglobulin-like receptor, subfamily A (with TM domain), member 4	1.05012	0.949642	1.033135
23548	TTC33	tetratricopeptide repeat domain 33	0.95232	0.924411	0.8974989
23549	DNPEP	aspartyl aminopeptidase	0.98581	0.977415	1.0428028

23550	PSD4	pleckstrin and Sec7 domain containing 4	0.97129	0.943817	0.9713096
23551	RASD2	RASD family, member 2	0.99011	1.024687	0.9814247
23552	CDK20	cyclin-dependent kinase 20	0.9731	0.984777	0.9818784
23553	HYAL4	hyaluronoglucosaminidase 4	1.0137	1.013005	0.992924
23554	TSPAN12	tetraspanin 12	1.01573	1.014161	1.0057652
23555	TSPAN15	tetraspanin 15	0.99059	1.012639	0.9862423
23556	PIGN	phosphatidylinositol glycan anchor biosynthesis, class N	0.94593	0.960685	0.9863977
23557	SNAPIN	SNAP-associated protein	0.99591	0.947556	0.9757079
23558	WBP2	WW domain binding protein 2	0.96283	0.98173	0.9674131
23559	WBP1	WW domain binding protein 1	1.00184	0.98244	0.9751424
23560	GTPBP4	GTP binding protein 4	0.92478	1.056036	1.0282086
23562	CLDN14	claudin 14	0.99055	1.038142	1.0105612
23563	CHST5	carbohydrate (N-acetylglucosamine 6-O) sulfotransferase 5	0.99218	1.009145	1.0144192
23564	DDAH2	dimethylarginine dimethylaminohydrolase 2	0.98844	1.039494	0.9853472
23566	LPAR3	lysophosphatidic acid receptor 3	1.01221	1.015751	1.0458665
23567	ZNF346	zinc finger protein 346	0.97968	0.981267	0.9825471
23568	ARL2BP	ADP-ribosylation factor-like 2 binding protein	0.96729	0.945449	1.0590432
23569	PADI4	peptidyl arginine deiminase, type IV	1.06551	0.946134	0.9745039
23576	DDAH1	dimethylarginine dimethylaminohydrolase 1	1.00355	1.020642	1.0261352
23580	CDC42EP4	CDC42 effector protein (Rho GTPase binding) 4	0.97237	1.021318	1.0582252
23581	CASP14	caspase 14, apoptosis-related cysteine peptidase	1.00733	1.005034	1.0240915
23582	CCNDBP1	cyclin D-type binding-protein 1	0.9524	0.966917	1.0480189
23583	SMUG1	single-strand-selective monofunctional uracil-DNA glycosylase 1	0.98916	1.012102	1.010129
23584	VSIG2	V-set and immunoglobulin domain containing 2	0.94989	0.972306	1.0261527
23585	TMEM50A	transmembrane protein 50A	1.01306	0.98854	0.9757231
23586	DDX58	DEAD (Asp-Glu-Ala-Asp) box polypeptide 58	0.94035	0.965449	1.0256176
23587	C17orf81	chromosome 17 open reading frame 81	0.91017	0.957283	1.0825184
23588	KLHDC2	kelch domain containing 2	0.93683	0.91099	0.9339809
23589	CARHSP1	calcium regulated heat stable protein 1, 24kDa	0.97276	1.030345	1.0333696
23590	PDSS1	prenyl (decaprenyl) diphosphate synthase, subunit 1	0.98548	1.018989	1.0240472
23592	LEMD3	LEM domain containing 3	0.94899	1.006666	1.0646056
23593	HEBP2	heme binding protein 2	1.00214	0.886107	0.983274
23594	ORC6	origin recognition complex, subunit 6	1.01357	0.935818	1.0146694

23595	ORC3	origin recognition complex, subunit 3	0.97226	0.961833	1.0032146
23596	OPN3	opsin 3	0.99809	1.006725	1.0096751
23597	ACOT9	acyl-CoA thioesterase 9	0.97837	1.114188	0.938423
23598	PATZ1	POZ (BTB) and AT hook containing zinc finger 1	0.94169	0.992915	1.029925
23600	AMACR	alpha-methylacyl-CoA racemase	0.98878	0.984423	0.9992908
23601	CLEC5A	C-type lectin domain family 5, member A	1.02737	1.030763	1.0606177
23603	CORO1C	coronin, actin binding protein, 1C	0.97366	0.966366	0.9613049
23604	DAPK2	death-associated protein kinase 2	1.0043	1.034268	1.0131313
23605	HMGN2P9	high mobility group nucleosomal binding domain 2 pseudogene 9	0.99208	1.053954	0.9875704
23607	CD2AP	CD2-associated protein	0.99848	0.945488	1.0938406
23608	MKRN1	makorin ring finger protein 1	1.02986	0.94692	0.9686894
23609	MKRN2	makorin ring finger protein 2	0.95486	0.960512	0.9990644
23610	MKRN6P	makorin ring finger protein 6, pseudogene	1.01529	1.040686	0.9727906
23612	PHLDA3	pleckstrin homology-like domain, family A, member 3	1.00037	1.017813	1.0835743
23613	ZMYND8	zinc finger, MYND-type containing 8	0.95507	0.990712	0.9669714
23614	PPY2	pancreatic polypeptide 2	0.99984	1.008145	1.0589105
23615	PYY2	peptide YY, 2 (seminalplasmin)	0.99402	1.047058	1.0188807
23616	SH3BP1	SH3-domain binding protein 1	0.9549	0.974969	0.9851263
23617	TSSK2	testis-specific serine kinase 2	1.00275	1.025824	1.0076026
23619	ZIM2	zinc finger, imprinted 2	0.99322	0.957318	0.9636804
23620	NTSR2	neurotensin receptor 2	1.00487	1.012539	1.0310849
23621	BACE1	beta-site APP-cleaving enzyme 1	0.99122	0.942912	1.0026136
23623	RUSC1	RUN and SH3 domain containing 1	0.98671	0.98837	1.0743607
23624	CBLC	Cas-Br-M (murine) ecotropic retroviral transforming sequence c	0.98973	1.015246	1.0246903
23625	FAM89B	family with sequence similarity 89, member B	0.93611	0.894031	1.0361038
23626	SPO11	SPO11 meiotic protein covalently bound to DSB homolog (S. cerevisiae)	0.99061	1.001936	1.0428917
23627	PRND	prion protein 2 (dublet)	1.01501	1.009704	1.0227089
23629	BRD7P3	bromodomain containing 7 pseudogene 3	1.01858	0.996692	0.9930045
23630	KCNE1L	KCNE1-like	0.99818	0.967351	1.0269231
23632	CA14	carbonic anhydrase XIV	1.00644	1.021831	0.9696619
23633	KPNA6	karyopherin alpha 6 (importin alpha 7)	0.99184	0.957673	0.9879478
23635	SSBP2	single-stranded DNA binding protein 2	0.96922	1.002058	1.0124358
23637	RABGAP1	RAB GTPase activating protein 1	0.93083	0.959197	1.0724045

23639	LRRC6	leucine rich repeat containing 6	1.00397	1.044468	1.018633
23640	HSPBP1	HSPA (heat shock 70kDa) binding protein, cytoplasmic cochaperone 1	0.96989	1.017278	1.0806194
23641	LDOC1	leucine zipper, down-regulated in cancer 1	0.91703	0.851869	0.9725649
23642	SNHG1	small nucleolar RNA host gene 1 (non-protein coding)	0.89135	0.893337	0.9849643
23643	LY96	lymphocyte antigen 96	0.99824	0.964671	1.0232147
23644	EDC4	enhancer of mRNA decapping 4	0.95469	1.012204	1.0276876
23645	PPP1R15A	protein phosphatase 1, regulatory (inhibitor) subunit 15A	0.91563	1.068754	0.9071043
23646	PLD3	phospholipase D family, member 3	0.95188	0.959223	1.0493894
23647	ARFIP2	ADP-ribosylation factor interacting protein 2	0.95598	0.989321	0.997646
23648	SSBP3	single stranded DNA binding protein 3	1.02376	0.986531	0.9221655
23649	POLA2	polymerase (DNA directed), alpha 2 (70kD subunit)	0.95372	0.948549	1.0318024
23650	TRIM29	tripartite motif containing 29	0.99516	1.012097	1.0153544
23654	PLXNB2	plexin B2	1.01887	1.02027	1.0495508
23657	SLC7A11	solute carrier family 7, (cationic amino acid transporter, y+ system) member 11	1.01771	1.022493	0.9946524
23658	LSM5	LSM5 homolog, U6 small nuclear RNA associated (S. cerevisiae)	0.96746	0.919593	0.9722846
23659	PLA2G15	phospholipase A2, group XV	0.97603	0.95204	1.0505147
23660	ZKSCAN5	zinc finger with KRAB and SCAN domains 5	0.96379	0.979128	1.0439062
23666	UBBP4	ubiquitin B pseudogene 4	0.95428	1.022972	1.0856702
23667	UBBP3	ubiquitin B pseudogene 3	0.99957	0.953242	1.0281273
23668	UBBP2	ubiquitin B pseudogene 2	0.94965	0.962791	1.1535968
23670	TMEM2	transmembrane protein 2	0.95519	1.057622	0.9882581
23671	TMEFF2	transmembrane protein with EGF-like and two follistatin-like domains 2	0.99624	0.994773	1.0166399
23673	STX12	syntaxin 12	1.0026	1.005144	0.9542763
23676	SMPX	small muscle protein, X-linked	0.99332	1.001149	0.99054
23677	SH3BP4	SH3-domain binding protein 4	1.01545	0.992167	0.988115
23678	SGK3	serum/glucocorticoid regulated kinase family, member 3	0.99492	0.985285	0.9718015
23682	RAB38	RAB38, member RAS oncogene family	0.9986	1.031607	1.0213969
23683	PRKD3	protein kinase D3	0.94837	1.06125	0.9531287
23704	KCNE4	potassium voltage-gated channel, Isk-related family, member 4	0.9817	0.943103	1.0061144
23705	CADM1	cell adhesion molecule 1	1.00385	1.010708	1.0050198
23708	GSPT2	G1 to S phase transition 2	0.8965	0.885593	1.0274535
23710	GABARAPL	GABA(A) receptor-associated protein like 1	0.97245	0.989066	0.9590853
23729	SHPK	sedoheptulokinase	0.96712	0.902257	1.0754198

23731	C9orf5	chromosome 9 open reading frame 5	0.95737	1.009028	1.0406975
23732	C9orf4	chromosome 9 open reading frame 4	0.98816	0.965872	0.9870214
23738	OFD1P17	OFD1 pseudogene 17	1.00072	0.981923	1.0317151
23741	EID1	EP300 interacting inhibitor of differentiation 1	0.93648	0.867013	1.1498342
23742	C15orf2	chromosome 15 open reading frame 2	0.99945	0.989983	1.0057371
23743	BHMT2	betaine--homocysteine S-methyltransferase 2	0.99973	0.980893	1.0163113
23746	AIPL1	aryl hydrocarbon receptor interacting protein-like 1	0.98842	0.98822	0.9707737
23752	TSSK1A	testis-specific serine kinase 1A pseudogene	1.02548	0.928012	1.0205758
23753	SDF2L1	stromal cell-derived factor 2-like 1	0.93521	0.984914	1.025337
23754	RPL32P5	ribosomal protein L32 pseudogene 5	0.91616	1.016356	1.0271622
23759	PPIL2	peptidylprolyl isomerase (cyclophilin)-like 2	0.94407	0.98648	1.0203858
23760	PITPNB	phosphatidylinositol transfer protein, beta	0.94371	0.994016	1.1082368
23761	PISD	phosphatidylserine decarboxylase	0.97512	0.98152	1.0518048
23762	OSBP2	oxysterol binding protein 2	0.97193	1.003008	0.97983
23764	MAFF	v-maf musculoaponeurotic fibrosarcoma oncogene homolog F (avian)	1.01995	1.034638	0.9817157
23765	IL17RA	interleukin 17 receptor A	0.99465	0.922803	0.9989009
23766	GABARAPL	GABA(A) receptors associated protein like 3, pseudogene	0.9778	0.981389	0.9591395
23767	FLRT3	fibronectin leucine rich transmembrane protein 3	0.99644	1.028904	0.9968368
23768	FLRT2	fibronectin leucine rich transmembrane protein 2	1.00845	0.998039	1.0099665
23769	FLRT1	fibronectin leucine rich transmembrane protein 1	1.01098	0.956082	0.995487
23770	FKBP8	FK506 binding protein 8, 38kDa	0.96566	0.918124	1.0258379
23772	CSF2RBP1	colony stimulating factor 2 receptor, beta, low-affinity (granulocyte-macrophage) pseudogene 1	0.97725	1.003028	0.9708135
23774	BRD1	bromodomain containing 1	0.9833	1.069422	0.9442944
23780	APOL2	apolipoprotein L, 2	0.97311	0.997388	1.0087931
23786	BCL2L13	BCL2-like 13 (apoptosis facilitator)	0.9643	0.997361	1.0500272
23787	MTCH1	mitochondrial carrier 1	1.00926	1.019725	0.9735369
23788	MTCH2	mitochondrial carrier 2	0.97927	1.012128	1.0429995
24137	KIF4A	kinesin family member 4A	1.00326	1.011575	0.9999044
24138	IFIT5	interferon-induced protein with tetratricopeptide repeats 5	0.94542	0.95615	1.0842889
24139	EML2	echinoderm microtubule associated protein like 2	0.97489	1.013859	1.020818
24140	FTSJ1	FtsJ homolog 1 (E. coli)	0.93974	0.993411	1.0369534
24141	C20orf103	chromosome 20 open reading frame 103	0.98324	0.957737	1.0281568
24144	TFIP11	tuftelin interacting protein 11	0.92624	0.955857	1.0647721

24145	PANX1	pannexin 1	0.98344	0.978162	1.0469751
24146	CLDN15	claudin 15	0.99574	1.020601	1.0244339
24147	FJX1	four jointed box 1 (Drosophila)	1.00068	1.022238	1.0240996
24148	PRPF6	PRP6 pre-mRNA processing factor 6 homolog (S. cerevisiae)	0.92173	0.965702	0.956617
24149	ZNF318	zinc finger protein 318	0.9865	1.018783	1.0370602
25758	C11orf41	chromosome 11 open reading frame 41	0.99628	1.000463	0.9961011
25759	SHC2	SHC (Src homology 2 domain containing) transforming protein 2	0.99941	1.01347	1.0354616
25764	C15orf63	chromosome 15 open reading frame 63	0.93238	1.03883	0.9983976
25766	PRPF40B	PRP40 pre-mRNA processing factor 40 homolog B (S. cerevisiae)	0.98886	1.009287	1.0393551
25769	SLC24A2	solute carrier family 24 (sodium/potassium/calcium exchanger), member 2	1.00425	0.971513	0.9986365
25770	C22orf31	chromosome 22 open reading frame 31	0.99623	0.972491	1.0104258
25771	TBC1D22A	TBC1 domain family, member 22A	1.00458	1.024822	0.9851724
25775	C22orf24	chromosome 22 open reading frame 24	0.97529	0.97889	0.9677874
25776	CBY1	chibby homolog 1 (Drosophila)	0.97618	1.028218	0.9990242
25777	SUN2	Sad1 and UNC84 domain containing 2	0.95135	0.986472	0.9535424
25778	DSTYK	dual serine/threonine and tyrosine protein kinase	1.00042	0.937514	1.0067395
25782	RAB3GAP2	RAB3 GTPase activating protein subunit 2 (non-catalytic)	0.97604	0.947738	1.0708698
25788	RAD54B	RAD54 homolog B (S. cerevisiae)	0.95876	0.978053	1.0126293
25789	TMEM59L	transmembrane protein 59-like	1.01251	0.974261	0.9981293
25790	CCDC19	coiled-coil domain containing 19	0.99384	1.028791	0.9999189
25791	NGEF	neuronal guanine nucleotide exchange factor	1.0021	0.997318	1.008558
25792	CIZ1	CDKN1A interacting zinc finger protein 1	0.95846	0.964007	1.0138136
25793	FBXO7	F-box protein 7	0.94961	0.91427	0.9771135
25794	FSCN2	fascin homolog 2, actin-bundling protein, retinal (Strongylocentrotus purpuratus)	0.99175	0.995422	1.0404676
25796	PGLS	6-phosphogluconolactonase	0.98423	0.99983	1.0857935
25797	QPCT	glutaminyl-peptide cyclotransferase	1.0411	0.934938	0.940605
25798	BRI3	brain protein I3	1.02147	0.961802	0.9475964
25799	ZNF324	zinc finger protein 324	0.92472	1.020053	1.000215
25800	SLC39A6	solute carrier family 39 (zinc transporter), member 6	0.92799	0.962043	1.0017861
25801	GCA	grancalcin, EF-hand calcium binding protein	1.03914	1.077791	1.1413447
25802	LMOD1	leiomodin 1 (smooth muscle)	1.02569	0.989066	1.0126782
25803	SPDEF	SAM pointed domain containing ets transcription factor	0.99711	1.012166	0.9580352
25804	LSM4	LSM4 homolog, U6 small nuclear RNA associated (S. cerevisiae)	0.9808	0.994891	1.0624513

25805	BAMBI	BMP and activin membrane-bound inhibitor homolog (Xenopus laevis)	1.02828	0.98381	0.9807319
25806	VAX2	ventral anterior homeobox 2	1.00565	1.016803	1.0097401
25807	RHBDD3	rhomboid domain containing 3	0.98594	0.992044	1.044676
25809	TTLL1	tubulin tyrosine ligase-like family, member 1	0.93688	0.938716	1.0319384
25813	SAMM50	sorting and assembly machinery component 50 homolog (S. cerevisiae)	0.95646	0.984165	1.0330349
25814	ATXN10	ataxin 10	0.9576	0.945765	1.0471213
25816	TNFAIP8	tumor necrosis factor, alpha-induced protein 8	0.96959	1.059275	1.0342445
25817	FAM19A5	family with sequence similarity 19 (chemokine (C-C motif)-like), member A5	0.98484	1.007229	1.0127992
25818	KLK5	kallikrein-related peptidase 5	1.00467	0.971158	1.0104327
25819	CCRN4L	CCR4 carbon catabolite repression 4-like (S. cerevisiae)	0.99764	1.078678	0.8753998
25820	ARIH1	ariadne homolog, ubiquitin-conjugating enzyme E2 binding protein, 1 (Drosophila)	0.96427	1.000128	1.002299
25821	MTO1	mitochondrial translation optimization 1 homolog (S. cerevisiae)	0.99454	0.912252	1.0455568
25822	DNAJB5	DnaJ (Hsp40) homolog, subfamily B, member 5	0.99514	0.98157	1.0079091
25823	TPSG1	tryptase gamma 1	0.99841	0.994784	0.9481801
25824	PRDX5	peroxiredoxin 5	0.98769	0.981476	1.0462809
25825	BACE2	beta-site APP-cleaving enzyme 2	0.97365	1.014392	1.0046342
25827	FBXL2	F-box and leucine-rich repeat protein 2	1.00808	0.986448	0.9984096
25828	TXN2	thioredoxin 2	0.97397	0.936285	1.0247884
25829	TMEM184E	transmembrane protein 184B	0.96917	1.064141	0.9642712
25830	SULT4A1	sulfotransferase family 4A, member 1	0.9967	1.018922	1.0227657
25831	HECTD1	HECT domain containing 1	0.96143	0.985551	1.0374105
25833	POU2F3	POU class 2 homeobox 3	1.00082	1.017574	1.0175257
25834	MGAT4C	mannosyl (alpha-1,3-)-glycoprotein beta-1,4-N-acetylglucosaminyltransferase, isozyme C (putative)	0.99491	1.007539	0.9848735
25836	NIPBL	Nipped-B homolog (Drosophila)	0.9757	0.985094	0.9596484
25837	RAB26	RAB26, member RAS oncogene family	1.00415	1.016613	1.0336511
25839	COG4	component of oligomeric golgi complex 4	0.94732	0.902821	1.0105226
25840	METTL7A	methyltransferase like 7A	0.99353	0.93922	1.0241306
25841	ABTB2	ankyrin repeat and BTB (POZ) domain containing 2	0.99079	1.025073	1.0072359
25842	ASF1A	ASF1 anti-silencing function 1 homolog A (S. cerevisiae)	0.93713	0.95802	1.0595795
25843	MOBKL3	MOB1, Mps One Binder kinase activator-like 3 (yeast)	1.00093	0.969929	0.9868609
25844	YIPF3	Yip1 domain family, member 3	0.98464	0.968857	1.0176985
25845	PP7080	hypothetical LOC25845	0.91939	0.950179	0.9609602
25847	ANAPC13	anaphase promoting complex subunit 13	0.99688	0.97539	1.0779764

25849	PARM1	prostate androgen-regulated mucin-like protein 1	1.0183	0.987416	1.0175372
25850	ZNF345	zinc finger protein 345	0.951	0.933843	1.0415781
25851	TECPR1	tectonin beta-propeller repeat containing 1	0.97329	0.975064	0.9577166
25852	ARMC8	armadillo repeat containing 8	0.98595	0.969787	1.0448184
25853	DCAF12	DDB1 and CUL4 associated factor 12	0.95979	0.868291	1.1963539
25854	FAM149A	family with sequence similarity 149, member A	1.00198	1.005543	1.018936
25855	BRMS1	breast cancer metastasis suppressor 1	0.92226	0.946326	1.0987528
25858	C11orf20	chromosome 11 open reading frame 20	0.97293	1.008297	1.0468729
25859	PART1	prostate androgen-regulated transcript 1 (non-protein coding)	1.00018	1.003961	1.0207699
25861	DFNB31	deafness, autosomal recessive 31	0.98036	1.035875	0.9801265
25862	USP49	ubiquitin specific peptidase 49	0.99487	0.953108	0.9871736
25864	ABHD14A	abhydrolase domain containing 14A	0.97797	0.978515	1.0676296
25865	PRKD2	protein kinase D2	0.96065	0.983021	0.9343766
25870	SUMF2	sulfatase modifying factor 2	0.98778	0.951253	1.0471204
25871	C3orf17	chromosome 3 open reading frame 17	1.00704	0.904864	0.9665044
25873	RPL36	ribosomal protein L36	0.93985	1.112202	1.0574565
25874	BRP44	brain protein 44	0.95663	0.92034	1.0259102
25875	LETMD1	LETM1 domain containing 1	0.98153	0.992191	0.9640058
25876	SPEF1	sperm flagellar 1	0.9788	0.999409	1.0582591
25878	MXRA5	matrix-remodelling associated 5	0.99047	1.008141	0.9953391
25879	DCAF13	DDB1 and CUL4 associated factor 13	0.98259	1.020504	1.0709925
25880	TMEM186	transmembrane protein 186	0.94937	0.917238	1.0261197
25884	CHRDL2	chordin-like 2	1.00282	1.010487	0.9681167
25885	POLR1A	polymerase (RNA) I polypeptide A, 194kDa	0.99444	0.95295	0.9764253
25886	POC1A	POC1 centriolar protein homolog A (Chlamydomonas)	1.01427	0.985575	1.0146236
25888	ZNF473	zinc finger protein 473	0.9252	1.004283	1.0506816
25890	ABI3BP	ABI family, member 3 (NESH) binding protein	1.01716	1.01252	0.9905243
25891	PAMR1	peptidase domain containing associated with muscle regeneration 1	0.9931	0.997769	0.9920344
25893	TRIM58	tripartite motif containing 58	1.00041	0.951839	0.9997392
25894	PLEKHG4	pleckstrin homology domain containing, family G (with RhoGef domain) member 4	0.98575	0.981156	1.0202401
25895	METTL21B	methyltransferase like 21B	0.99197	0.993851	0.9968084
25896	INTS7	integrator complex subunit 7	0.97375	0.943087	1.0214289
25897	RNF19A	ring finger protein 19A	0.96248	0.96174	1.0275372

25898	RCHY1	ring finger and CHY zinc finger domain containing 1	0.96253	0.941038	0.9933466
25900	IFFO1	intermediate filament family orphan 1	0.9684	0.964583	1.0195647
25901	CCDC28A	coiled-coil domain containing 28A	0.9525	0.934184	0.9179629
25902	MTHFD1L	methylenetetrahydrofolate dehydrogenase (NADP+ dependent) 1-like	0.98886	0.975458	1.020573
25903	OLFML2B	olfactomedin-like 2B	0.99927	1.009861	0.9986155
25904	CNOT10	CCR4-NOT transcription complex, subunit 10	1.00088	0.987922	1.0632863
25906	C11orf51	chromosome 11 open reading frame 51	0.958	0.916234	1.0591885
25907	TMEM158	transmembrane protein 158 (gene/pseudogene)	1.03812	0.998626	0.9293329
25909	AHCTF1	AT hook containing transcription factor 1	0.99228	0.929745	0.9650919
25911	DPCD	deleted in primary ciliary dyskinesia homolog (mouse)	0.99223	1.025318	1.0344043
25912	C1orf43	chromosome 1 open reading frame 43	1.00129	0.942915	1.0698855
25913	POT1	protection of telomeres 1 homolog (S. pombe)	0.96666	0.927524	1.0042471
25914	RTTN	rotatin	0.92263	0.979277	1.016879
25915	NDUFAF3	NADH dehydrogenase (ubiquinone) 1 alpha subcomplex, assembly factor 3	0.96022	0.935941	0.9467383
25917	THUMPD3	THUMP domain containing 3	0.96961	0.835906	1.0120982
25920	COBRA1	cofactor of BRCA1	0.95776	0.921018	1.0234627
25921	ZDHHC5	zinc finger, DHHC-type containing 5	0.94075	0.976419	1.0780198
25923	ATL3	atlastin GTPase 3	0.94366	0.887654	1.1207058
25924	MYRIP	myosin VIIA and Rab interacting protein	1.00729	1.009515	1.0138557
25925	ZNF521	zinc finger protein 521	1.01174	0.982537	1.0037272
25926	NOL11	nucleolar protein 11	0.97244	0.936591	1.0441477
25927	CNRIP1	cannabinoid receptor interacting protein 1	0.99245	1.002872	1.0379286
25928	SOSTDC1	sclerostin domain containing 1	0.98372	0.998045	1.0065271
25929	GEMIN5	gem (nuclear organelle) associated protein 5	0.94879	0.981012	0.9869994
25930	PTPN23	protein tyrosine phosphatase, non-receptor type 23	0.982	1.01848	1.031691
25932	CLIC4	chloride intracellular channel 4	0.98543	1.124527	1.0085862
25934	NIPSNAP3A	nipsnap homolog 3A (C. elegans)	0.93042	0.877266	1.063058
25936	NSL1	NSL1, MIND kinetochore complex component, homolog (S. cerevisiae)	0.98789	1.005677	1.0518436
25938	HEATR5A	HEAT repeat containing 5A	0.95515	0.887123	1.0315969
25939	SAMHD1	SAM domain and HD domain 1	1.00761	0.978341	0.9771717
25940	FAM98A	family with sequence similarity 98, member A	0.98612	0.893213	1.0054814
25941	C18orf10	chromosome 18 open reading frame 10	0.97496	0.934839	1.0338971
25942	SIN3A	SIN3 homolog A, transcription regulator (yeast)	0.93526	1.01768	0.964963

25943	C20orf194	chromosome 20 open reading frame 194	0.97692	0.97548	0.9968932
25945	PVRL3	poliovirus receptor-related 3	0.96613	0.993509	1.0143579
25946	ZNF385A	zinc finger protein 385A	0.96161	1.044981	0.975811
25948	KBTBD2	kelch repeat and BTB (POZ) domain containing 2	0.98294	0.965374	0.9607118
25949	SYF2	SYF2 homolog, RNA splicing factor (S. cerevisiae)	0.95254	0.875247	0.9865226
25950	RWDD3	RWD domain containing 3	1.00895	0.912463	1.0775912
25953	PNKD	paroxysmal nonkinesigenic dyskinesia	1.01111	0.994681	0.9556075
25956	SEC31B	SEC31 homolog B (S. cerevisiae)	0.98643	1.03199	0.940683
25957	PNISR	PNN-interacting serine/arginine-rich protein	0.99371	0.875694	1.0220793
25959	KANK2	KN motif and ankyrin repeat domains 2	0.99059	0.999017	1.0272363
25960	GPR124	G protein-coupled receptor 124	1.00794	1.010601	1.0301091
25961	NUDT13	nudix (nucleoside diphosphate linked moiety X)-type motif 13	0.99808	1.003002	1.0108071
25962	KIAA1429	KIAA1429	0.9144	1.013312	1.128112
25963	TMEM87A	transmembrane protein 87A	0.95824	0.926669	1.102355
25966	C2CD2	C2 calcium-dependent domain containing 2	0.97362	0.969186	1.0086038
25970	SH2B1	SH2B adaptor protein 1	0.95288	0.985492	0.9692208
25972	UNC50	unc-50 homolog (C. elegans)	1.02303	0.988429	1.0117832
25973	PARS2	prolyl-tRNA synthetase 2, mitochondrial (putative)	1.0012	1.014716	1.0204662
25974	MMACHC	methylmalonic aciduria (cobalamin deficiency) cblC type, with homocystinuria	0.99513	0.995906	1.0066658
25975	EGFL6	EGF-like-domain, multiple 6	0.99843	1.021888	0.9826346
25976	TIPARP	TCDD-inducible poly(ADP-ribose) polymerase	1.00533	1.004585	1.0760534
25977	NECAP1	NECAP endocytosis associated 1	0.9621	0.996718	1.0109203
25978	CHMP2B	chromatin modifying protein 2B	1.02351	0.906164	0.9858996
25979	DHRS7B	dehydrogenase/reductase (SDR family) member 7B	0.98039	0.976163	0.9962125
25980	C20orf4	chromosome 20 open reading frame 4	0.9688	0.945012	1.0509971
25981	DNAH1	dynein, axonemal, heavy chain 1	0.96986	0.95255	0.9284978
25983	NGDN	neuroguidin, EIF4E binding protein	0.89212	0.93174	1.0788282
25984	KRT23	keratin 23 (histone deacetylase inducible)	0.98919	0.957626	0.9355718
25987	TSKU	tsukushi small leucine rich proteoglycan homolog (Xenopus laevis)	1.00744	0.971508	0.9966283
25988	HINFP	histone H4 transcription factor	0.95868	0.99509	1.0929953
25989	ULK3	unc-51-like kinase 3 (C. elegans)	0.96169	0.956259	1.0098055
25992	SNED1	sushi, nidogen and EGF-like domains 1	0.99124	0.971461	0.9569507
25994	HIGD1A	HIG1 hypoxia inducible domain family, member 1A	1.00033	0.97354	1.0084285

25996	REXO2	REX2, RNA exonuclease 2 homolog (S. cerevisiae)	0.93779	1.095835	0.9948734
25998	IBTK	inhibitor of Bruton agammaglobulinemia tyrosine kinase	0.98079	0.952339	1.0218075
25999	CLIP3	CAP-GLY domain containing linker protein 3	0.98736	1.018372	1.0452164
26000	TBC1D10B	TBC1 domain family, member 10B	0.97274	1.022534	1.0511004
26001	RNF167	ring finger protein 167	0.9441	0.950912	1.0062011
26002	MOXD1	monooxygenase, DBH-like 1	1.01409	1.018215	1.0067185
26003	GORASP2	golgi reassembly stacking protein 2, 55kDa	0.98526	0.987256	1.0530427
26005	C2CD3	C2 calcium-dependent domain containing 3	0.96678	0.99206	0.9719503
26007	DAK	dihydroxyacetone kinase 2 homolog (S. cerevisiae)	0.9706	0.918421	1.031716
26009	ZZZ3	zinc finger, ZZ-type containing 3	0.97016	1.031177	1.0385721
26010	SPATS2L	spermatogenesis associated, serine-rich 2-like	0.99775	0.984615	1.016941
26011	ODZ4	odz, odd Oz/ten-m homolog 4 (Drosophila)	0.99093	0.9968	0.9941363
26012	NELF	nasal embryonic LHRH factor	0.96834	1.041045	0.9537489
26013	L3MBTL1	l(3)mbt-like 1 (Drosophila)	0.98456	0.997267	1.0207038
26015	RPAP1	RNA polymerase II associated protein 1	0.96302	0.968356	0.9997043
26017	FAM32A	family with sequence similarity 32, member A	0.91885	0.92872	1.113259
26018	LRIG1	leucine-rich repeats and immunoglobulin-like domains 1	0.99985	1.032956	0.9668366
26019	UPF2	UPF2 regulator of nonsense transcripts homolog (yeast)	0.95153	0.963979	1.1082415
26020	LRP10	low density lipoprotein receptor-related protein 10	0.92656	0.940349	1.0264507
26022	TMEM98	transmembrane protein 98	0.99108	0.997272	1.0101826
26024	PTCD1	pentatricopeptide repeat domain 1	0.97707	0.944814	1.0384289
26027	ACOT11	acyl-CoA thioesterase 11	1.0072	1.00992	1.0219273
26030	PLEKHG3	pleckstrin homology domain containing, family G (with RhoGef domain) member 3	0.97828	1.012851	1.0193098
26031	OSBPL3	oxysterol binding protein-like 3	0.99	1.035606	1.0345191
26032	SUSD5	sushi domain containing 5	0.99432	0.998128	1.010906
26033	ATRNL1	attractin-like 1	1	1.003369	1.0159302
26034	IPCEF1	interaction protein for cytohesin exchange factors 1	0.9666	0.930667	1.02068
26035	GLCE	glucuronic acid epimerase	0.94602	0.945757	1.0574743
26036	ZNF451	zinc finger protein 451	0.99557	0.9812	0.9298164
26037	SIPA1L1	signal-induced proliferation-associated 1 like 1	0.97561	0.968998	1.0190165
26038	CHD5	chromodomain helicase DNA binding protein 5	0.99091	1.008957	1.0061717
26039	SS18L1	synovial sarcoma translocation gene on chromosome 18-like 1	0.98533	0.971063	0.9796186
26040	SETBP1	SET binding protein 1	1.02113	1.009584	0.9749293

26043	UBXN7	UBX domain protein 7	1.00494	0.973123	1.0409011
26045	LRRTM2	leucine rich repeat transmembrane neuronal 2	0.99983	0.958315	0.9609885
26046	LTN1	listerin E3 ubiquitin protein ligase 1	0.94891	0.992607	1.1386922
26047	CNTNAP2	contactin associated protein-like 2	1.00407	0.990021	1.0137464
26048	ZNF500	zinc finger protein 500	0.97843	0.931461	1.0354813
26049	FAM169A	family with sequence similarity 169, member A	0.95703	0.893851	0.9668835
26050	SLITRK5	SLIT and NTRK-like family, member 5	0.99778	0.979681	1.0145225
26051	PPP1R16B	protein phosphatase 1, regulatory (inhibitor) subunit 16B	0.99227	1.126245	0.9114342
26052	DNM3	dynamin 3	0.98304	0.965821	1.0033077
26053	AUTS2	autism susceptibility candidate 2	1.02292	0.976511	1.0035929
26054	SENP6	SUMO1/sentrin specific peptidase 6	0.99853	0.945634	0.9903283
26056	RAB11FIP5	RAB11 family interacting protein 5 (class I)	0.99757	1.010516	0.9851442
26057	ANKRD17	ankyrin repeat domain 17	0.98528	0.995368	0.9625803
26058	GIGYF2	GRB10 interacting GYF protein 2	0.97957	0.993525	1.031436
26059	ERC2	ELKS/RAB6-interacting/CAST family member 2	1.01492	0.997908	1.0051675
26060	APPL1	adaptor protein, phosphotyrosine interaction, PH domain and leucine zipper containing 1	0.97761	1.052493	1.1613713
26061	HACL1	2-hydroxyacyl-CoA lyase 1	0.96968	0.893543	1.1275729
26062	HYALP1	hyaluronoglucosaminidase pseudogene 1	1.00065	1.040329	1.0341235
26063	DECR2	2,4-dienoyl CoA reductase 2, peroxisomal	0.99039	0.977665	1.0217528
26064	RAI14	retinoic acid induced 14	1.00828	0.998848	0.9840213
26065	LSM14A	LSM14A, SCD6 homolog A (S. cerevisiae)	0.96292	0.988351	0.9420128
26073	POLDIP2	polymerase (DNA-directed), delta interacting protein 2	0.94907	0.955194	1.1143055
26074	C20orf26	chromosome 20 open reading frame 26	0.99389	1.005221	1.0046334
26083	TBC1D29	TBC1 domain family, member 29	0.99975	1.054266	0.9878793
26084	ARHGEF26	Rho guanine nucleotide exchange factor (GEF) 26	1.0067	0.998904	1.0131481
26085	KLK13	kallikrein-related peptidase 13	1.00115	0.990366	1.0227352
26086	GPSM1	G-protein signaling modulator 1	0.99353	0.997701	1.0078021
26088	GGA1	golgi-associated, gamma adaptin ear containing, ARF binding protein 1	0.97811	0.960561	0.998273
26090	ABHD12	abhydrolase domain containing 12	0.98764	0.965792	1.0389726
26091	HERC4	hect domain and RLD 4	0.95529	1.001124	1.0499903
26092	TOR1AIP1	torsin A interacting protein 1	0.97905	0.921382	0.9506767
26093	CCDC9	coiled-coil domain containing 9	0.97802	0.969451	0.9873102
26094	DCAF4	DDB1 and CUL4 associated factor 4	0.94187	0.988374	1.0448585

26097	CHTOP	chromatin target of PRMT1	0.96458	0.863507	1.071756
26098	C10orf137	chromosome 10 open reading frame 137	0.93249	0.901042	1.1420485
26099	C1orf144	chromosome 1 open reading frame 144	0.99467	0.935115	0.9578659
26100	WIPI2	WD repeat domain, phosphoinositide interacting 2	0.98051	0.899278	0.9630789
26103	LRIT1	leucine-rich repeat, immunoglobulin-like and transmembrane domains 1	1.00138	1.009954	1.0425052
26108	PYGO1	pygopus homolog 1 (Drosophila)	1.03138	1.019355	1.0278298
26112	CCDC69	coiled-coil domain containing 69	1.01482	1.027118	0.9897996
26115	TANC2	tetratricopeptide repeat, ankyrin repeat and coiled-coil containing 2	0.97049	0.997387	0.9723996
26118	WSB1	WD repeat and SOCS box containing 1	1.01717	1.007197	0.9772473
26119	LDLRAP1	low density lipoprotein receptor adaptor protein 1	0.93172	0.939239	0.8379456
26121	PRPF31	PRP31 pre-mRNA processing factor 31 homolog (S. cerevisiae)	0.93384	0.965615	1.0527909
26122	EPC2	enhancer of polycomb homolog 2 (Drosophila)	0.98844	0.945352	0.9946182
26123	TCTN3	tectonic family member 3	0.90968	0.97327	1.0318506
26127	FGFR1OP2	FGFR1 oncogene partner 2	0.93427	1.042208	0.9952204
26128	KIAA1279	KIAA1279	0.93097	1.00474	1.0323726
26130	GAPVD1	GTPase activating protein and VPS9 domains 1	0.95576	1.08357	0.981512
26133	TRPC4AP	transient receptor potential cation channel, subfamily C, member 4 associated protein	0.96955	1.106616	1.0254966
26135	SERBP1	SERPINE1 mRNA binding protein 1	1.00918	0.97939	0.9904822
26137	ZBTB20	zinc finger and BTB domain containing 20	0.98689	0.995963	0.897267
26138	C8orf71	chromosome 8 open reading frame 71	1.00232	1.002983	0.9999127
26140	TTLL3	tubulin tyrosine ligase-like family, member 3	1.01755	1.024826	1.000186
26145	IRF2BP1	interferon regulatory factor 2 binding protein 1	0.95525	1.038942	1.0025246
26146	TRAF3IP1	TNF receptor-associated factor 3 interacting protein 1	0.99601	0.976761	1.0159963
26147	PHF19	PHD finger protein 19	0.96294	1.00894	1.0586158
26148	C10orf12	chromosome 10 open reading frame 12	0.93254	0.958483	0.9889776
26150	RIBC2	RIB43A domain with coiled-coils 2	0.99155	0.972024	0.9862034
26151	NAT9	N-acetyltransferase 9 (GCN5-related, putative)	0.92475	1.011167	1.0308747
26152	ZNF337	zinc finger protein 337	0.94091	0.973576	1.0835339
26153	KIF26A	kinesin family member 26A	0.97959	0.992705	1.0533872
26154	ABCA12	ATP-binding cassette, sub-family A (ABC1), member 12	1.01183	0.997854	1.0163307
26155	NOC2L	nucleolar complex associated 2 homolog (S. cerevisiae)	0.96738	0.95605	1.0559572
26156	RSL1D1	ribosomal L1 domain containing 1	0.9393	0.835665	1.0934591
26157	GIMAP2	GTPase, IMAP family member 2	0.96193	0.858815	1.0656359

26160	IFT172	intraflagellar transport 172 homolog (Chlamydomonas)	1.00889	0.998976	0.9920467
26164	GTPBP5	GTP binding protein 5 (putative)	0.95417	0.986053	0.9904589
26166	RGS22	regulator of G-protein signaling 22	0.99375	0.995048	1.0143054
26167	PCDHB5	protocadherin beta 5	0.99961	1.035519	1.0046941
26168	SENP3	SUMO1/sentrin/SMT3 specific peptidase 3	0.95958	0.925834	0.9974054
26173	INTS1	integrator complex subunit 1	0.99968	0.992093	1.0326933
26175	C14orf109	chromosome 14 open reading frame 109	0.97367	1.017246	1.0044607
26184	OR1F2P	olfactory receptor, family 1, subfamily F, member 2	1.01698	0.981655	0.9433474
26188	OR1C1	olfactory receptor, family 1, subfamily C, member 1	1.0067	0.98776	1.054517
26189	OR1A2	olfactory receptor, family 1, subfamily A, member 2	1.03762	0.987643	1.0040271
26190	FBXW2	F-box and WD repeat domain containing 2	0.97629	0.930679	1.0343841
26191	PTPN22	protein tyrosine phosphatase, non-receptor type 22 (lymphoid)	1.01498	1.090343	1.0002367
26205	GMEB2	glucocorticoid modulatory element binding protein 2	0.96685	0.969294	0.9617933
26206	SPAG8	sperm associated antigen 8	0.98659	0.993509	1.0295842
26207	PITPNC1	phosphatidylinositol transfer protein, cytoplasmic 1	1.00366	1.01854	0.9276868
26211	OR2F1	olfactory receptor, family 2, subfamily F, member 1	1.00588	1.021923	1.0037246
26212	OR2B6	olfactory receptor, family 2, subfamily B, member 6	0.98112	0.947446	1.0072564
26219	OR1J4	olfactory receptor, family 1, subfamily J, member 4	1.02325	0.957473	1.0139999
26220	DGCR5	DiGeorge syndrome critical region gene 5 (non-protein coding)	0.99294	0.999174	NA
26223	FBXL21	F-box and leucine-rich repeat protein 21 (gene/pseudogene)	0.97606	0.94374	1.0064712
26224	FBXL3	F-box and leucine-rich repeat protein 3	0.94674	0.940033	1.0077318
26225	ARL5A	ADP-ribosylation factor-like 5A	1.00789	1.020645	1.0452935
26227	PHGDH	phosphoglycerate dehydrogenase	0.99848	0.984223	1.0167748
26228	STAP1	signal transducing adaptor family member 1	0.93776	0.946469	1.0873395
26229	B3GAT3	beta-1,3-glucuronyltransferase 3 (glucuronosyltransferase I)	0.96478	1.035164	1.0571299
26230	TIAM2	T-cell lymphoma invasion and metastasis 2	1.00575	1.010029	0.9943605
26231	LRRC29	leucine rich repeat containing 29	0.99286	1.004232	1.0240448
26232	FBXO2	F-box protein 2	1.00196	1.00185	1.0863144
26233	FBXL6	F-box and leucine-rich repeat protein 6	0.97387	1.042111	1.0766078
26234	FBXL5	F-box and leucine-rich repeat protein 5	1.03165	0.974696	1.0199327
26235	FBXL4	F-box and leucine-rich repeat protein 4	0.96116	0.98209	1.0125724
26238	C6orf123	chromosome 6 open reading frame 123	1.01222	1.020598	0.9688406
26240	FAM50B	family with sequence similarity 50, member B	0.9507	0.921673	1.0467655

26242	OR4C1P	olfactory receptor, family 4, subfamily C, member 1 pseudogene	1.02395	0.994891	0.9184443
26245	OR2M4	olfactory receptor, family 2, subfamily M, member 4	0.98628	0.989157	0.9841736
26248	OR2K2	olfactory receptor, family 2, subfamily K, member 2	0.98622	1.036843	0.903721
26249	KLHL3	kelch-like 3 (Drosophila)	1	0.964548	0.9962108
26251	KCNG2	potassium voltage-gated channel, subfamily G, member 2	1.00204	1.038131	1.0555675
26253	CLEC4E	C-type lectin domain family 4, member E	0.93468	1.15536	0.9738089
26254	OPTC	opticin	1.00338	0.9941	1.00335
26255	PTTG3P	pituitary tumor-transforming 3, pseudogene	0.95793	0.925189	0.9600631
26256	CABYR	calcium binding tyrosine-(Y)-phosphorylation regulated	0.9666	0.995685	1.0108779
26257	NKX2-8	NK2 homeobox 8	0.99046	1.003134	1.0189336
26258	PLDN	pallidin homolog (mouse)	0.96802	0.918203	0.9683144
26259	FBXW8	F-box and WD repeat domain containing 8	0.96843	0.994998	1.0297778
26260	FBXO25	F-box protein 25	0.97283	0.91039	1.0534697
26261	FBXO24	F-box protein 24	0.98899	1.011472	1.0093273
26262	TSPAN17	tetraspanin 17	0.95239	0.971643	1.0638823
26263	FBXO22	F-box protein 22	0.97213	0.962121	1.1062516
26266	SLC13A4	solute carrier family 13 (sodium/sulfate symporters), member 4	1.03177	0.984741	0.9893617
26267	FBXO10	F-box protein 10	0.9901	0.98714	0.9914905
26268	FBXO9	F-box protein 9	1.01648	0.929701	1.0523984
26269	FBXO8	F-box protein 8	0.96057	0.911455	1.0455864
26270	FBXO6	F-box protein 6	0.9588	0.946279	1.0874506
26271	FBXO5	F-box protein 5	0.94889	1.00371	1.0871987
26272	FBXO4	F-box protein 4	0.98072	0.949357	1.0216137
26273	FBXO3	F-box protein 3	0.92953	0.892233	0.9904861
26275	HIBCH	3-hydroxyisobutyryl-CoA hydrolase	1.00225	0.985518	0.992615
26276	VPS33B	vacuolar protein sorting 33 homolog B (yeast)	0.93242	0.991821	1.1431578
26277	TINF2	TERF1 (TRF1)-interacting nuclear factor 2	0.90776	1.008492	0.9118597
26278	SACS	spastic ataxia of Charlevoix-Saguenay (sacsin)	0.96158	0.954684	1.0102318
26279	PLA2G2D	phospholipase A2, group IID	1.00779	1.028103	1.0691682
26280	IL1RAPL2	interleukin 1 receptor accessory protein-like 2	1.00193	1.005537	1.0006663
26281	FGF20	fibroblast growth factor 20	1.00054	1.064773	1.0007329
26284	ERAL1	Era G-protein-like 1 (E. coli)	0.9235	0.884365	1.0069959
26285	CLDN17	claudin 17	0.99772	1.012045	0.997898

26286	ARFGAP3	ADP-ribosylation factor GTPase activating protein 3	0.95229	1.040091	0.9648929
26289	AK5	adenylate kinase 5	0.94952	0.98272	0.9577614
26290	GALNT8	UDP-N-acetyl-alpha-D-galactosamine:polypeptide N-acetylgalactosaminyltransferase 8 (GalNAc-T8)	0.98929	1.008701	1.0101364
26291	FGF21	fibroblast growth factor 21	1.00289	1.034645	1.0302901
26292	MYCBP	c-myc binding protein	1.01662	1.053773	1.0605728
26297	SERGEF	secretion regulating guanine nucleotide exchange factor	0.93334	0.954276	1.0238276
26298	EHF	ets homologous factor	0.99733	1.011564	1.0208603
26301	GBGT1	globoside alpha-1,3-N-acetylgalactosaminyltransferase 1	0.97275	0.92488	1.0521211
26330	GAPDHS	glyceraldehyde-3-phosphate dehydrogenase, spermatogenic	0.99184	1.02455	1.0232175
26333	OR7A17	olfactory receptor, family 7, subfamily A, member 17	0.98925	1.009113	1.0209918
26335	OR7A15P	olfactory receptor, family 7, subfamily A, member 15 pseudogene	1.02341	0.994501	0.967872
26339	OR5K1	olfactory receptor, family 5, subfamily K, member 1	1.00412	0.990172	1.0023927
26341	OR5H1	olfactory receptor, family 5, subfamily H, member 1	0.99432	1.004345	1.0104809
26343	OR5E1P	olfactory receptor, family 5, subfamily E, member 1 pseudogene	0.99773	1.01587	0.9983012
26353	HSPB8	heat shock 22kDa protein 8	0.97398	1.025599	0.9935336
26354	GNL3	guanine nucleotide binding protein-like 3 (nucleolar)	1.02326	0.965755	1.0218556
26355	FAM162A	family with sequence similarity 162, member A	0.99337	0.983239	0.9852261
26468	LHX6	LIM homeobox 6	0.9934	0.980389	0.9871387
26469	PTPN18	protein tyrosine phosphatase, non-receptor type 18 (brain-derived)	1.03034	0.854607	0.9696365
26470	SEZ6L2	seizure related 6 homolog (mouse)-like 2	0.98123	1.035003	0.9948012
26471	NUPR1	nuclear protein, transcriptional regulator, 1	1.01511	0.977127	0.9819066
26472	PPP1R14B	protein phosphatase 1, regulatory (inhibitor) subunit 14B	0.9899	0.976674	1.0077485
26476	OR10J1	olfactory receptor, family 10, subfamily J, member 1	1.03237	0.97291	1.0427095
26479	OR7E62P	olfactory receptor, family 7, subfamily E, member 62 pseudogene	1.01052	0.969389	1.0360029
26492	OR8G2	olfactory receptor, family 8, subfamily G, member 2	0.98414	0.996688	1.0351453
26493	OR8B8	olfactory receptor, family 8, subfamily B, member 8	1.00015	1.017143	1.0220025
26496	OR10A3	olfactory receptor, family 10, subfamily A, member 3	1.02382	1.002173	0.9533271
26497	OR10D3	olfactory receptor, family 10, subfamily D, member 3 (non-functional)	1.03573	1.039434	0.9573597
26499	PLEK2	pleckstrin 2	0.98497	0.976357	1.0085158
26502	NARF	nuclear prelamin A recognition factor	0.95822	1.040344	1.0579607
26503	SLC17A5	solute carrier family 17 (anion/sugar transporter), member 5	0.99546	1.005032	0.9478078
26504	CNNM4	cyclin M4	1.00323	0.968663	1.0034602
26505	CNNM3	cyclin M3	0.98265	0.957815	0.9442374

26507	CNNM1	cyclin M1	1.00232	1.006537	1.009315
26508	HEYL	hairy/enhancer-of-split related with YRPW motif-like	1.0008	1.013979	1.0247971
26509	MYOF	myoferlin	1.05051	0.942414	0.9759201
26511	CHIC2	cysteine-rich hydrophobic domain 2	0.98914	0.894077	0.9593553
26512	INTS6	integrator complex subunit 6	0.95647	1.005602	1.0152243
26514	RPL34P1	ribosomal protein L34 pseudogene 1	0.97661	0.980856	0.9991666
26515	FXC1	fracture callus 1 homolog (rat)	0.91567	0.953293	0.9553583
26516	RPS5P1	ribosomal protein S5 pseudogene 1	1.01156	1.041959	1.0098379
26517	TIMM13	translocase of inner mitochondrial membrane 13 homolog (yeast)	0.88586	0.760748	1.1755935
26519	TIMM10	translocase of inner mitochondrial membrane 10 homolog (yeast)	0.95842	1.002765	1.0341812
26520	TIMM9	translocase of inner mitochondrial membrane 9 homolog (yeast)	0.92505	0.868437	1.095292
26521	TIMM8B	translocase of inner mitochondrial membrane 8 homolog B (yeast)	0.90119	0.992594	1.1455353
26523	EIF2C1	eukaryotic translation initiation factor 2C, 1	1.01405	1.049202	1.0046924
26524	LATS2	LATS, large tumor suppressor, homolog 2 (Drosophila)	0.97442	0.950229	0.9823678
26525	IL36RN	interleukin 36 receptor antagonist	1.01211	0.987583	0.9978713
26526	TSPAN16	tetraspanin 16	0.9739	1.001285	0.9979939
26528	DAZAP1	DAZ associated protein 1	0.97225	0.959669	1.0287018
26529	OR12D2	olfactory receptor, family 12, subfamily D, member 2	1.03014	0.992482	1.0139844
26530	OR12D1P	olfactory receptor, family 12, subfamily D, member 1 pseudogene	1.02053	1.045282	1.0572642
26531	OR11A1	olfactory receptor, family 11, subfamily A, member 1	1.00134	1.005082	0.967958
26533	OR10G3	olfactory receptor, family 10, subfamily G, member 3	1.00474	1.007657	1.0119841
26537	OR51A1P	olfactory receptor, family 51, subfamily A, member 1 pseudogene	0.99914	1.019235	0.977529
26539	OR10H1	olfactory receptor, family 10, subfamily H, member 1	1.0134	0.97919	0.9165243
26541	OR10D1P	olfactory receptor, family 10, subfamily D, member 1 pseudogene	0.99689	1.009241	0.9548744
26548	ITGB1BP2	integrin beta 1 binding protein (melusin) 2	0.99823	0.993148	0.9712182
26574	AATF	apoptosis antagonizing transcription factor	0.95711	0.944522	1.0547929
26575	RGS17	regulator of G-protein signaling 17	1.00535	1.024192	0.9930445
26576	SRPK3	SRSF protein kinase 3	0.98788	0.998182	1.029696
26577	PCOLCE2	procollagen C-endopeptidase enhancer 2	1.02107	1.002866	1.0136949
26578	OSTF1	osteoclast stimulating factor 1	0.95744	1.032518	0.9850417
26579	MYEOV	myeloma overexpressed (in a subset of t(11;14) positive multiple myelomas)	1.00473	0.979501	0.9904496
26580	BSCL2	Berardinelli-Seip congenital lipodystrophy 2 (seipin)	0.95813	0.942982	1.1071296
26585	GREM1	gremlin 1	0.99775	1.001604	0.9756573

26586	CKAP2	cytoskeleton associated protein 2	0.9496	0.910755	1.1593457
26589	MRPL46	mitochondrial ribosomal protein L46	0.94401	1.008974	1.0895086
26590	OR8B7P	olfactory receptor, family 8, subfamily B, member 7 pseudogene	0.99575	1.034307	1.0319658
26591	OR8B6P	olfactory receptor, family 8, subfamily B, member 6 pseudogene	1.01395	1.014838	0.9649189
26592	OR8B5P	olfactory receptor, family 8, subfamily B, member 5 pseudogene	0.99454	0.997476	1.0073275
26608	TBL2	transducin (beta)-like 2	0.98398	1.010186	0.9788471
26610	ELP4	elongation protein 4 homolog (S. cerevisiae)	0.95451	0.959398	1.0043878
26628	OR7E47P	olfactory receptor, family 7, subfamily E, member 47 pseudogene	1.0087	1.162859	0.937248
26640	OR7E33P	olfactory receptor, family 7, subfamily E, member 33 pseudogene	0.99304	1.03498	0.9935424
26645	OR7E28P	olfactory receptor, family 7, subfamily E, member 28 pseudogene	1.02082	0.982225	1.0039298
26647	OR7E25P	olfactory receptor, family 7, subfamily E, member 25 pseudogene	1.02806	0.979238	1.0346075
26648	OR7E24	olfactory receptor, family 7, subfamily E, member 24	1.02512	1.023013	0.9874358
26651	OR7E19P	olfactory receptor, family 7, subfamily E, member 19 pseudogene	1.0016	0.974419	0.9799624
26656	OR7D1P	olfactory receptor, family 7, subfamily D, member 1 pseudogene	0.9683	1.022982	1.0746141
26658	OR7C2	olfactory receptor, family 7, subfamily C, member 2	0.99757	1.037948	0.9799877
26659	OR7A5	olfactory receptor, family 7, subfamily A, member 5	1.00644	1.003311	0.9616292
26661	OR7A8P	olfactory receptor, family 7, subfamily A, member 8 pseudogene	1.00513	1.001145	0.9878309
26664	OR7C1	olfactory receptor, family 7, subfamily C, member 1	0.99419	0.953611	1.0250905
26668	OR7A3P	olfactory receptor, family 7, subfamily A, member 3 pseudogene	1.0111	0.969027	0.9749423
26669	OR7A11P	olfactory receptor, family 7, subfamily A, member 11 pseudogene	0.98892	0.989169	1.0161938
26687	OR4E1	olfactory receptor, family 4, subfamily E, member 1 (gene/pseudogene)	0.97796	0.979698	0.9648659
26689	OR4D1	olfactory receptor, family 4, subfamily D, member 1	0.98663	0.963537	0.98345
26690	OR4B2P	olfactory receptor, family 4, subfamily B, member 2 pseudogene	1.00318	1.011943	0.9696161
26692	OR2W1	olfactory receptor, family 2, subfamily W, member 1	0.99609	0.9641	1.0010545
26693	OR2V1	olfactory receptor, family 2, subfamily V, member 1	1.03563	1.033809	0.9950853
26694	OR2U2P	olfactory receptor, family 2, subfamily U, member 2 pseudogene	1.02286	1.012521	0.9909785
26701	OR2N1P	olfactory receptor, family 2, subfamily N, member 1 pseudogene	1.01039	1.046222	1.0063272
26707	OR2J2	olfactory receptor, family 2, subfamily J, member 2	1.0566	1.072944	0.9643889
26713	OR2H5P	olfactory receptor, family 2, subfamily H, member 5 pseudogene	0.98787	1.019089	0.9706074
26716	OR2H1	olfactory receptor, family 2, subfamily H, member 1	1.00162	0.985196	0.9963228
26717	OR2G1P	olfactory receptor, family 2, subfamily G, member 1 pseudogene	1.0071	1.022152	0.9924092
26735	OR1L3	olfactory receptor, family 1, subfamily L, member 3	0.97295	0.973951	1.0544731
26737	OR1L1	olfactory receptor, family 1, subfamily L, member 1	0.9769	1.051595	1.0270162

26740	OR1J2	olfactory receptor, family 1, subfamily J, member 2	1.00386	1.016432	1.0387556
26742	OR1H1P	olfactory receptor, family 1, subfamily H, member 1 pseudogene	1.01183	1.02024	0.9953845
26747	NUFIP1	nuclear fragile X mental retardation protein interacting protein 1	0.96761	0.970273	1.0230446
26750	RPS6KC1	ribosomal protein S6 kinase, 52kDa, polypeptide 1	0.99377	0.917777	1.0447725
26751	SH3YL1	SH3 domain containing, Ysc84-like 1 (S. cerevisiae)	0.97867	1.026781	1.0328395
26762	HAVCR1	hepatitis A virus cellular receptor 1	1.01187	0.981099	0.9643977
26777	SNORA71A	small nucleolar RNA, H/ACA box 71A	0.98536	0.90553	1.0947608
26788	SNORD60	small nucleolar RNA, C/D box 60	0.88516	1.049878	1.0151851
26823	RNU12-2P	RNA, U12 small nuclear 2, pseudogene	1.03403	1.014747	1.0407533
26829	RNU5E	RNA, U5E small nuclear	0.99384	1.028513	0.9844654
26831	RNU5A	RNA, U5A small nuclear	1.01819	1.046169	1.0267452
26852	RNU2-5P	RNA, U2 small nuclear 5, pseudogene	1.00101	1.003907	1.0177043
26855	RNU2-2	RNA, U2 small nuclear 2	0.94792	1.016134	0.9928743
26872	STEAP1	six transmembrane epithelial antigen of the prostate 1	1.00456	1.042734	1.0201935
26873	OPLAH	5-oxoprolinase (ATP-hydrolysing)	0.9939	1.009888	1.0396969
26952	SMR3A	submaxillary gland androgen regulated protein 3A	1.02933	1.057759	0.9854787
26953	RANBP6	RAN binding protein 6	0.91887	0.986539	1.1055429
26958	COPG2	coatomer protein complex, subunit gamma 2	0.99513	0.943223	1.042211
26959	HBP1	HMG-box transcription factor 1	1	0.916869	0.9911941
26960	NBEA	neurobeachin	0.97467	0.96508	1.0147428
26973	CHORDC1	cysteine and histidine-rich domain (CHORD) containing 1	0.94315	0.992058	0.9987451
26974	ZNF285	zinc finger protein 285	0.95638	1.02252	1.0365405
26980	PABPC1P1	poly(A) binding protein, cytoplasmic 1 pseudogene 1	0.98629	0.967828	0.9423973
26982	ABCD1P3	ATP-binding cassette, sub-family D (ALD), member 1, pseudogene 3	0.98708	1.02269	1.0039752
26984	SEC22A	SEC22 vesicle trafficking protein homolog A (S. cerevisiae)	0.97641	0.94692	0.9562009
26985	AP3M1	adaptor-related protein complex 3, mu 1 subunit	0.96776	0.957327	1.102846
26986	PABPC1	poly(A) binding protein, cytoplasmic 1	1.0371	1.045204	1.0065003
26993	AKAP8L	A kinase (PRKA) anchor protein 8-like	0.95942	0.982442	1.0321466
26994	RNF11	ring finger protein 11	0.99176	1.010635	0.9238155
26995	TRUB2	TruB pseudouridine (psi) synthase homolog 2 (E. coli)	0.92242	0.979575	0.9735374
26996	GPR160	G protein-coupled receptor 160	0.99244	1.00378	0.9881085
26998	FETUB	fetuin B	1.01338	0.967496	0.9979763
26999	CYFIP2	cytoplasmic FMR1 interacting protein 2	0.9734	1.026552	0.9258208

27000	DNAJC2	DnaJ (Hsp40) homolog, subfamily C, member 2	1.00297	0.902595	1.0042583
27004	TCL6	T-cell leukemia/lymphoma 6 (non-protein coding)	0.98892	0.985438	1.0633601
27005	USP21	ubiquitin specific peptidase 21	0.97347	0.952015	1.0070592
27006	FGF22	fibroblast growth factor 22	0.99935	1.00863	1.03536
27010	TPK1	thiamin pyrophosphokinase 1	0.99538	0.993328	0.9514499
27012	KCNV1	potassium channel, subfamily V, member 1	1.00938	0.990005	0.9991658
27013	CNPPD1	cyclin Pas1/PHO80 domain containing 1	0.97783	1.00846	1.0202723
27018	NGFRAP1	nerve growth factor receptor (TNFRSF16) associated protein 1	0.91745	0.945198	0.9843133
27019	DNAI1	dynein, axonemal, intermediate chain 1	1.00853	0.988648	0.9968484
27020	NPTN	neuroplastin	0.97494	1.00044	1.0953659
27022	FOXD3	forkhead box D3	0.99835	1.004248	1.0846004
27023	FOXB1	forkhead box B1	0.97765	1.022825	1.0807545
27030	MLH3	mutL homolog 3 (E. coli)	0.94475	0.969487	1.0294279
27031	NPHP3	nephronophthisis 3 (adolescent)	0.98213	0.936071	0.9402008
27032	ATP2C1	ATPase, Ca++ transporting, type 2C, member 1	1.00468	0.99197	1.0752869
27033	ZBTB32	zinc finger and BTB domain containing 32	1.00309	0.974224	0.9968883
27034	ACAD8	acyl-CoA dehydrogenase family, member 8	0.9673	1.032963	1.009781
27035	NOX1	NADPH oxidase 1	0.99982	1.0076	1.0085106
27036	SIGLEC7	sialic acid binding Ig-like lectin 7	0.96457	0.881524	0.9091933
27037	TRMT2A	TRM2 tRNA methyltransferase 2 homolog A (S. cerevisiae)	0.96288	0.980076	1.0095253
27039	PKD2L2	polycystic kidney disease 2-like 2	0.99763	1	1.0055264
27040	LAT	linker for activation of T cells	0.94178	0.926638	0.9590637
27042	DIEXF	digestive organ expansion factor homolog (zebrafish)	0.96216	0.951677	1.0327827
27043	PELP1	proline, glutamate and leucine rich protein 1	0.97715	1.015385	0.9928095
27044	SND1	staphylococcal nuclease and tudor domain containing 1	1.0159	1.013779	1.0360797
27063	ANKRD1	ankyrin repeat domain 1 (cardiac muscle)	1.00837	1.014838	1.0215073
27065	D4S234E	DNA segment on chromosome 4 (unique) 234 expressed sequence	0.98741	0.954586	1.0387077
27067	STAU2	staufen, RNA binding protein, homolog 2 (Drosophila)	0.98343	0.97116	1.0631939
27068	PPA2	pyrophosphatase (inorganic) 2	0.97772	0.918179	1.0402509
27069	GHITM	growth hormone inducible transmembrane protein	0.98473	0.941016	1.0657843
27071	DAPP1	dual adaptor of phosphotyrosine and 3-phosphoinositides	0.95723	0.948036	1.0353819
27072	VPS41	vacuolar protein sorting 41 homolog (S. cerevisiae)	1.01156	0.988695	1.0592361
27074	LAMP3	lysosomal-associated membrane protein 3	1.00255	0.966541	1.0241049

27075	TSPAN13	tetraspanin 13	0.97293	0.98493	0.9804371
27076	LYPD3	LY6/PLAUR domain containing 3	0.97976	0.992096	1.0250317
27077	B9D1	B9 protein domain 1	0.99439	1.024474	1.0185521
27079	RPUSD2	RNA pseudouridylate synthase domain containing 2	0.94274	0.996268	1.0429993
27085	MTBP	Mdm2, transformed 3T3 cell double minute 2, p53 binding protein (mouse) binding protein, 104kDa	0.97201	0.950307	1.0072383
27086	FOXP1	forkhead box P1	0.99903	0.900064	0.9699853
27087	B3GAT1	beta-1,3-glucuronyltransferase 1 (glucuronosyltransferase P)	0.99816	1.021038	0.9917086
27089	UQCRQ	ubiquinol-cytochrome c reductase, complex III subunit VII, 9.5kDa	0.97591	0.971341	0.9926604
27090	ST6GALNA(	ST6 (alpha-N-acetyl-neuraminyl-2,3-beta-galactosyl-1,3)-N-acetylgalactosaminide alpha-2,6-sialyltransferase	0.95389	1.01077	0.9927132
27091	CACNG5	calcium channel, voltage-dependent, gamma subunit 5	0.98168	0.980106	0.9548785
27092	CACNG4	calcium channel, voltage-dependent, gamma subunit 4	0.99228	1.005333	1.0058065
27093	KCNMB3P1	potassium large conductance calcium-activated channel, subfamily M, beta member 3 pseudogene 1	1.01723	1.065117	0.9953502
27094	KCNMB3	potassium large conductance calcium-activated channel, subfamily M beta member 3	0.98298	1.002018	0.9905858
27095	TRAPPC3	trafficking protein particle complex 3	0.98751	0.902212	1.0266237
27097	TAF5L	TAF5-like RNA polymerase II, p300/CBP-associated factor (PCAF)-associated factor, 65kDa	0.9945	0.921488	1.0503629
27098	CLUL1	clusterin-like 1 (retinal)	0.99028	1.000547	0.9994057
27101	CACYBP	calcyclin binding protein	1.01005	0.970908	1.0106536
27102	EIF2AK1	eukaryotic translation initiation factor 2-alpha kinase 1	1.01384	0.894951	1.0566377
27106	ARRDC2	arrestin domain containing 2	0.98849	1.028745	0.9801562
27107	ZBTB11	zinc finger and BTB domain containing 11	0.99185	0.954937	0.8988355
27109	ATP5S	ATP synthase, H+ transporting, mitochondrial Fo complex, subunit s (factor B)	0.95391	0.978073	1.0409161
27111	SDCBP2	syndecan binding protein (syntenin) 2	0.99192	1.001291	1.001291
27112	FAM155B	family with sequence similarity 155, member B	1.00604	1.008514	1.0203763
27113	BBC3	BCL2 binding component 3	0.95085	1.010373	1.0190527
27115	PDE7B	phosphodiesterase 7B	1.00582	1.007765	1.0028075
27120	DKKL1	dickkopf-like 1	0.98171	0.976836	1.0464337
27121	DKK4	dickkopf homolog 4 (Xenopus laevis)	0.98223	1.023235	0.9973938
27122	DKK3	dickkopf homolog 3 (Xenopus laevis)	1.00071	1.01802	0.9898754
27123	DKK2	dickkopf homolog 2 (Xenopus laevis)	1.0123	1.034441	1.0037044
27124	INPP5J	inositol polyphosphate-5-phosphatase J	0.9876	0.995144	0.9817883
27125	AFF4	AF4/FMR2 family, member 4	0.98858	0.938879	0.9410595
27127	SMC1B	structural maintenance of chromosomes 1B	0.99	1.002745	1.0145489
27128	CYTH4	cytohesin 4	0.96874	0.999807	0.9989607

27129	HSPB7	heat shock 27kDa protein family, member 7 (cardiovascular)	0.99552	0.990073	1.0173019
27130	INVS	inversin	0.95314	1.013236	1.0257569
27131	SNX5	sorting nexin 5	0.96868	1.131019	1.0531244
27132	CPNE7	copine VII	0.99626	1.009991	1.0172016
27133	KCNH5	potassium voltage-gated channel, subfamily H (eag-related), member 5	1.00561	1.000824	0.9990499
27134	TJP3	tight junction protein 3 (zona occludens 3)	0.96497	0.945419	0.9556706
27136	MORC1	MORC family CW-type zinc finger 1	0.99165	0.97789	0.9745422
27141	CIDEB	cell death-inducing DFFA-like effector b	0.99719	0.893172	1.0123136
27143	KIAA1274	KIAA1274	0.98593	0.991697	1.0005508
27145	FILIP1	filamin A interacting protein 1	1.02492	1.010778	1.024507
27146	FAM184B	family with sequence similarity 184, member B	1.0072	1.006892	0.9940973
27147	DENND2A	DENN/MADD domain containing 2A	0.99801	1.006827	1.0162586
27148	STK36	serine/threonine kinase 36	1.0085	0.991191	1.016392
27151	CPAMD8	C3 and PZP-like, alpha-2-macroglobulin domain containing 8	0.98735	1.010522	1.0069882
27152	INTU	inturned planar cell polarity effector homolog (Drosophila)	1.00959	0.995151	1.006123
27153	ZNF777	zinc finger protein 777	0.97948	0.945682	1.0394476
27154	BRPF3	bromodomain and PHD finger containing, 3	0.98766	0.965277	0.9813813
27156	RTDR1	rhabdoid tumor deletion region gene 1	0.98785	0.980791	0.9832888
27158	NDOR1	NADPH dependent diflavin oxidoreductase 1	0.9747	1.036134	1.0333063
27159	CHIA	chitinase, acidic	1.00948	1.023675	1.0076277
27160	INGX	inhibitor of growth family, X-linked, pseudogene	1.01478	1.063809	0.9318563
27161	EIF2C2	eukaryotic translation initiation factor 2C, 2	0.97608	1.042985	0.932983
27163	NAAA	N-acylethanolamine acid amidase	0.99945	0.903059	1.0053366
27164	SALL3	sal-like 3 (Drosophila)	1.00109	0.981845	1.0727644
27165	GLS2	glutaminase 2 (liver, mitochondrial)	0.98139	0.963214	1.0141077
27166	PRELID1	PRELI domain containing 1	1.03979	0.968041	1.0534377
27170	COTL1P1	coactosin-like 1 (Dictyostelium) pseudogene 1	0.96779	1.065802	0.9849424
27173	SLC39A1	solute carrier family 39 (zinc transporter), member 1	0.98849	0.994401	1.1044945
27175	TUBG2	tubulin, gamma 2	0.96432	0.944282	1.0143417
27177	IL36B	interleukin 36, beta	1.02962	1.02461	1.0016885
27178	IL37	interleukin 37	1.01034	1.032334	1.0216331
27179	IL36A	interleukin 36, alpha	1.03259	0.99824	1.0416515
27180	SIGLEC9	sialic acid binding Ig-like lectin 9	0.97249	0.952368	0.9617316

27181	SIGLEC8	sialic acid binding Ig-like lectin 8	1.00716	0.973988	0.9995554
27183	VPS4A	vacuolar protein sorting 4 homolog A (S. cerevisiae)	0.94274	1.026442	1.077107
27184	DISC2	disrupted in schizophrenia 2 (non-protein coding)	1.01171	0.959621	1.0292792
27185	DISC1	disrupted in schizophrenia 1	1.00569	1.008773	1.0163578
27189	IL17C	interleukin 17C	0.99747	1.037971	0.981408
27190	IL17B	interleukin 17B	1.02267	1.016395	1.0188205
27197	GPR82	G protein-coupled receptor 82	1.00598	1.011211	0.9977301
27198	HCAR1	hydroxycarboxylic acid receptor 1	1.02564	0.984515	0.9868671
27199	OXGR1	oxoglutarate (alpha-ketoglutarate) receptor 1	1.00303	1.031895	0.9710973
27200	GPR79	G protein-coupled receptor 79, pseudogene	1.03069	0.979355	1.0087448
27201	GPR78	G protein-coupled receptor 78	1.02035	0.962578	0.9882423
27202	GPR77	G protein-coupled receptor 77	1.0119	0.892986	0.8966957
27229	TUBGCP4	tubulin, gamma complex associated protein 4	0.93607	1.001086	1.0152504
27230	SERP1	stress-associated endoplasmic reticulum protein 1	1.02012	0.980029	0.8544011
27231	ITGB1BP3	integrin beta 1 binding protein 3	0.97715	1.025719	1.0252667
27232	GNMT	glycine N-methyltransferase	1.00365	0.993574	0.9800957
27233	SULT1C4	sulfotransferase family, cytosolic, 1C, member 4	1.0079	0.997504	1.0305226
27235	COQ2	coenzyme Q2 homolog, prenyltransferase (yeast)	1.00572	0.882119	0.9932902
27236	ARFIP1	ADP-ribosylation factor interacting protein 1	0.98827	0.987263	1.0103969
27237	ARHGEF16	Rho guanine nucleotide exchange factor (GEF) 16	0.99628	1.002997	1.0204496
27238	GPKOW	G patch domain and KOW motifs	0.9153	1.00287	1.0956274
27239	GPR162	G protein-coupled receptor 162	0.99592	0.906077	1.0761489
27240	SIT1	signaling threshold regulating transmembrane adaptor 1	0.89545	1.017124	1.0794475
27241	BBS9	Bardet-Biedl syndrome 9	0.98581	0.947333	0.9666951
27242	TNFRSF21	tumor necrosis factor receptor superfamily, member 21	1.00797	0.969885	0.9525109
27243	CHMP2A	chromatin modifying protein 2A	0.97844	0.938383	1.1177218
27244	SESN1	sestrin 1	0.97655	0.973376	0.9424433
27245	AHDC1	AT hook, DNA binding motif, containing 1	0.99873	0.972938	1.0128629
27246	RNF115	ring finger protein 115	1.0002	1.044501	0.9904416
27247	NFU1	NFU1 iron-sulfur cluster scaffold homolog (S. cerevisiae)	0.9786	0.96892	1.0507998
27248	ERLEC1	endoplasmic reticulum lectin 1	0.99471	1.018494	1.1067405
27249	MMADHC	methylmalonic aciduria (cobalamin deficiency) cblD type, with homocystinuria	1.01535	0.922847	0.9466031
27250	PDCD4	programmed cell death 4 (neoplastic transformation inhibitor)	0.98276	1.005194	0.9836128

27252	KLHL20	kelch-like 20 (Drosophila)	0.98703	0.978649	0.986686
27253	PCDH17	protocadherin 17	0.98431	0.971441	0.9789056
27254	CSDC2	cold shock domain containing C2, RNA binding	0.9906	0.978582	1.0362708
27255	CNTN6	contactin 6	1.01545	1.015905	1.0059668
27257	LSM1	LSM1 homolog, U6 small nuclear RNA associated (S. cerevisiae)	0.95924	0.990194	1.0284966
27258	LSM3	LSM3 homolog, U6 small nuclear RNA associated (S. cerevisiae)	0.97824	0.884873	1.0173916
27283	TINAG	tubulointerstitial nephritis antigen	0.99408	1.00652	1.0098294
27284	SULT1B1	sulfotransferase family, cytosolic, 1B, member 1	0.92365	0.869734	0.8747789
27285	TEKT2	tektin 2 (testicular)	1.00388	0.986531	0.9982168
27286	SRPX2	sushi-repeat containing protein, X-linked 2	0.99562	1.008837	1.0114901
27287	VENTX	VENT homeobox	0.99901	1.022476	1.021664
27288	RBMXL2	RNA binding motif protein, X-linked-like 2	0.98906	0.944232	1.1055008
27289	RND1	Rho family GTPase 1	1.01513	0.970507	0.9688424
27290	SPINK4	serine peptidase inhibitor, Kazal type 4	0.99227	0.986111	1.0190865
27291	C10orf28	chromosome 10 open reading frame 28	0.93538	0.990391	1.0233153
27292	DIMT1L	DIM1 dimethyladenosine transferase 1-like (S. cerevisiae)	0.96922	1.020228	1.040204
27293	SMPDL3B	sphingomyelin phosphodiesterase, acid-like 3B	1.01424	1.00833	0.9917197
27294	DHDH	dihydrodiol dehydrogenase (dimeric)	0.99175	0.968423	0.9844437
27295	PDLIM3	PDZ and LIM domain 3	1.00892	0.988663	1.0064577
27296	TP53TG5	TP53 target 5	0.99612	1.00449	0.984073
27297	CRCP	CGRP receptor component	0.99323	1.023614	0.9733435
27299	ADAMDEC1	ADAM-like, decysin 1	1.01129	0.99282	1.0040899
27300	ZNF544	zinc finger protein 544	0.95691	0.998719	1.0154071
27301	APEX2	APEX nuclease (apurinic/apyrimidinic endonuclease) 2	0.94479	0.988403	1.0218226
27302	BMP10	bone morphogenetic protein 10	1.00532	1.006563	0.9855048
27303	RBMS3	RNA binding motif, single stranded interacting protein 3	1.00471	1.006593	1.0080063
27304	MOCS3	molybdenum cofactor synthesis 3	0.95526	0.928259	1.0164244
27306	HPGDS	hematopoietic prostaglandin D synthase	1.00043	0.976641	1.0080677
27309	ZNF330	zinc finger protein 330	1.01722	0.935601	1.0194072
27314	RAB30	RAB30, member RAS oncogene family	0.96811	0.968992	0.9893167
27315	PGAP2	post-GPI attachment to proteins 2	0.98582	1.018899	1.0259705
27316	RBMX	RNA binding motif protein, X-linked	0.93506	0.978702	0.935446
27319	BHLHE22	basic helix-loop-helix family, member e22	1.00423	0.986463	1.0487047

27324	TOX3	TOX high mobility group box family member 3	1.00916	1.000999	0.9850211
27327	TNRC6A	trinucleotide repeat containing 6A	0.94734	0.939445	1.0050723
27328	PCDH11X	protocadherin 11 X-linked	1.00899	0.992945	1.0166055
27329	ANGPTL3	angiopoietin-like 3	1.00299	1.024215	1.0476892
27330	RPS6KA6	ribosomal protein S6 kinase, 90kDa, polypeptide 6	1.00291	0.998593	0.9882486
27332	ZNF638	zinc finger protein 638	0.96611	0.986249	1.0441852
27333	GOLIM4	golgi integral membrane protein 4	0.9912	0.950445	1.0404634
27334	P2RY10	purinergic receptor P2Y, G-protein coupled, 10	0.93517	0.906546	1.020069
27335	EIF3K	eukaryotic translation initiation factor 3, subunit K	0.95712	1.016127	1.0597767
27336	HTATSF1	HIV-1 Tat specific factor 1	0.93162	1.023247	1.0116961
27338	UBE2S	ubiquitin-conjugating enzyme E2S	1.02147	0.953561	0.9860373
27339	PRPF19	PRP19/PSO4 pre-mRNA processing factor 19 homolog (S. cerevisiae)	0.94946	0.970935	1.0463802
27340	UTP20	UTP20, small subunit (SSU) processome component, homolog (yeast)	0.94488	0.909538	0.9991162
27341	RRP7A	ribosomal RNA processing 7 homolog A (S. cerevisiae)	0.97971	1.09084	0.9655829
27342	RABGEF1	RAB guanine nucleotide exchange factor (GEF) 1	0.99465	1.040719	0.9371491
27343	POLL	polymerase (DNA directed), lambda	0.98532	0.98555	1.029095
27344	PCSK1N	proprotein convertase subtilisin/kexin type 1 inhibitor	0.97711	0.992223	1.0870248
27345	KCNMB4	potassium large conductance calcium-activated channel, subfamily M, beta member 4	0.96741	0.956764	1.0209568
27346	TMEM97	transmembrane protein 97	0.97983	0.960397	1.0213435
27347	STK39	serine threonine kinase 39	0.98847	0.934791	0.9347391
27348	TOR1B	torsin family 1, member B (torsin B)	0.92283	0.986266	0.9893977
27349	MCAT	malonyl CoA:ACP acyltransferase (mitochondrial)	0.94257	0.937791	1.1466459
27350	APOBEC3C	apolipoprotein B mRNA editing enzyme, catalytic polypeptide-like 3C	0.92649	0.885048	1.1745887
27351	PPPDE2	PPPDE peptidase domain containing 2	0.97178	1.033717	0.9900538
27352	SGSM3	small G protein signaling modulator 3	0.96332	0.964758	1.0532568
27429	HTRA2	HtrA serine peptidase 2	0.96534	1.027536	1.0576939
27430	MAT2B	methionine adenosyltransferase II, beta	0.97605	0.871459	0.9997089
27433	TOR2A	torsin family 2, member A	0.95997	0.980903	1.0710165
27434	POLM	polymerase (DNA directed), mu	0.97483	1.000518	0.9583106
27436	EML4	echinoderm microtubule associated protein like 4	1.0191	1.022404	0.9323688
27437	HSFY1P1	heat shock transcription factor, Y-linked 1 pseudogene 1	1.00158	1.016916	0.9881243
27439	CECR6	cat eye syndrome chromosome region, candidate 6	0.98938	0.988082	1.0495303
27440	CECR5	cat eye syndrome chromosome region, candidate 5	0.97121	1.005212	1.0271344

27443	CECR2	cat eye syndrome chromosome region, candidate 2	1.00027	1.01586	1.0365245
27445	PCLO	piccolo (presynaptic cytomatrix protein)	1.00404	1.000663	1.0159447
28231	SLCO4A1	solute carrier organic anion transporter family, member 4A1	0.9945	1.030145	0.9858641
28232	SLCO3A1	solute carrier organic anion transporter family, member 3A1	0.99308	0.978389	0.988773
28234	SLCO1B3	solute carrier organic anion transporter family, member 1B3	0.99172	0.987114	1.0209639
28316	CDH20	cadherin 20, type 2	0.99077	0.995793	0.9889094
28337	IGHVIV-44-	immunoglobulin heavy variable (IV)-44-1 (pseudogene)	1.0109	1.031497	1.0057631
28338	IGHVIII-82	immunoglobulin heavy variable (III)-82 (pseudogene)	1.00669	0.998865	1.0104783
28339	IGHVIII-76-	immunoglobulin heavy variable (III)-76-1 (pseudogene)	1.02664	0.939681	1.011353
28341	IGHVIII-67-	immunoglobulin heavy variable (III)-67-3 (pseudogene)	0.98135	1.126322	1.0445427
28346	IGHVIII-38-	immunoglobulin heavy variable (III)-38-1 (pseudogene)	1.03348	1.00008	1.0104472
28347	IGHVIII-26-	immunoglobulin heavy variable (III)-26-1 (pseudogene)	1.0049	0.974337	0.9981055
28353	IGHVIII-5-2	immunoglobulin heavy variable (III)-5-2 (pseudogene)	0.97141	1.048858	0.9741988
28357	IGHVII-74-1	immunoglobulin heavy variable (II)-74-1 (pseudogene)	0.99742	1.069267	0.9071181
28363	IGHVII-51-2	immunoglobulin heavy variable (II)-51-2 (pseudogene)	0.99891	1.04343	0.9988041
28365	IGHVII-46-1	immunoglobulin heavy variable (II)-46-1 (pseudogene)	1.00421	0.986139	0.9549172
28376	IGHVII-15-1	immunoglobulin heavy variable (II)-15-1 (pseudogene)	1.00549	1.022759	0.9594944
28378	IGHV7-81	immunoglobulin heavy variable 7-81 (non-functional)	1.0271	1.009348	0.9884855
28380	IGHV7-56	immunoglobulin heavy variable 7-56 (pseudogene)	0.97154	1.039704	0.9980774
28382	IGHV7-34-1	immunoglobulin heavy variable 7-34-1 (pseudogene)	0.99721	1.000963	0.9791648
28383	IGHV7-27	immunoglobulin heavy variable 7-27 (pseudogene)	0.98387	0.950978	0.9664281
28385	IGHV6-1	immunoglobulin heavy variable 6-1	0.97998	0.958194	1.1413197
28387	IGHV5-78	immunoglobulin heavy variable 5-78 (pseudogene)	0.9996	0.898809	0.9769562
28388	IGHV5-51	immunoglobulin heavy variable 5-51	0.97376	1.019302	1.0397934
28390	IGHV4-80	immunoglobulin heavy variable 4-80 (pseudogene)	0.9589	1.006578	1.0221044
28393	IGHV4-55	immunoglobulin heavy variable 4-55 (pseudogene)	0.99231	1.03291	1.0444333
28394	IGHV4-39	immunoglobulin heavy variable 4-39	0.94556	1.007173	1.0385237
28395	IGHV4-34	immunoglobulin heavy variable 4-34	0.99906	0.964065	1.0526478
28396	IGHV4-31	immunoglobulin heavy variable 4-31	0.99473	1.043321	1.0863218
28400	IGHV4-28	immunoglobulin heavy variable 4-28	0.96149	1.023845	1.0825632
28406	IGHV3-76	immunoglobulin heavy variable 3-76 (pseudogene)	1.00523	0.986781	1.0026883
28407	IGHV3-75	immunoglobulin heavy variable 3-75 (pseudogene)	1.01509	0.979214	0.9878471
28409	IGHV3-73	immunoglobulin heavy variable 3-73	0.97363	0.942395	1.0705799

28413	IGHV3-65	immunoglobulin heavy variable 3-65 (pseudogene)	1.03744	0.941892	1.0756315
28414	IGHV3-64	immunoglobulin heavy variable 3-64	0.97449	1.074312	1.0210586
28421	IGHV3-52	immunoglobulin heavy variable 3-52 (pseudogene)	0.99827	0.9344	0.9875614
28422	IGHV3-50	immunoglobulin heavy variable 3-50 (pseudogene)	1.04366	0.991772	1.0061166
28423	IGHV3-49	immunoglobulin heavy variable 3-49	0.96056	0.994929	1.0068524
28426	IGHV3-43	immunoglobulin heavy variable 3-43	0.98254	0.997479	1.0177647
28428	IGHV3-41	immunoglobulin heavy variable 3-41 (pseudogene)	0.99742	1.054544	1.0553107
28429	IGHV3-38	immunoglobulin heavy variable 3-38 (non-functional)	1.02075	1.065401	1.030164
28430	IGHV3-37	immunoglobulin heavy variable 3-37 (pseudogene)	1.03555	1.005374	0.9676538
28443	IGHV3-22	immunoglobulin heavy variable 3-22 (pseudogene)	0.9338	0.955347	1.1115837
28445	IGHV3-20	immunoglobulin heavy variable 3-20	1.00764	0.977208	0.9878412
28448	IGHV3-15	immunoglobulin heavy variable 3-15	0.95277	1.022719	1.1522918
28449	IGHV3-13	immunoglobulin heavy variable 3-13	0.98419	0.972113	1.0815013
28451	IGHV3-9	immunoglobulin heavy variable 3-9	0.95707	1.008648	1.1805246
28452	IGHV3-7	immunoglobulin heavy variable 3-7	0.9749	1.111814	1.0839853
28453	IGHV3-6	immunoglobulin heavy variable 3-6 (pseudogene)	1.00949	0.999759	1.0453651
28455	IGHV2-26	immunoglobulin heavy variable 2-26	1.0046	1.037152	0.9549058
28456	IGHV2-10	immunoglobulin heavy variable 2-10 (pseudogene)	0.98716	1.058537	1.0049041
28457	IGHV2-5	immunoglobulin heavy variable 2-5	0.99008	1.0422	0.9810519
28461	IGHV1-69	immunoglobulin heavy variable 1-69	1.01964	0.976164	1.0536419
28462	IGHV1-68	immunoglobulin heavy variable 1-68 (pseudogene)	0.99615	0.912714	0.9817954
28463	IGHV1-67	immunoglobulin heavy variable 1-67 (pseudogene)	0.99629	1.075272	0.8900914
28464	IGHV1-58	immunoglobulin heavy variable 1-58	1.00817	1.015566	0.9636782
28465	IGHV1-46	immunoglobulin heavy variable 1-46	1.02638	1.004488	1.0796939
28466	IGHV1-45	immunoglobulin heavy variable 1-45	0.95472	1.051932	0.9843675
28467	IGHV1-24	immunoglobulin heavy variable 1-24	0.97759	1.018681	1.114569
28468	IGHV1-18	immunoglobulin heavy variable 1-18	0.89016	1.006976	1.0293315
28469	IGHV1-17	immunoglobulin heavy variable 1-17 (pseudogene)	0.98513	1.002725	1.0725944
28511	NKIRAS2	NFKB inhibitor interacting Ras-like 2	0.96648	0.989253	1.0555269
28512	NKIRAS1	NFKB inhibitor interacting Ras-like 1	0.96596	1.007532	0.9787913
28513	CDH19	cadherin 19, type 2	0.99179	0.996329	0.983913
28514	DLL1	delta-like 1 (Drosophila)	0.98867	0.985402	0.9765017
28516	TRDV3	T cell receptor delta variable 3	0.96736	1.01498	1.0847499

28557	TRBV30	T cell receptor beta variable 30 (gene/pseudogene)	1.00305	0.939068	0.9776411
28869	IGKV6D-41	immunoglobulin kappa variable 6D-41 (non-functional)	0.9784	1.005189	1.0140587
28907	IGKV5-2	immunoglobulin kappa variable 5-2	1.00872	1.067971	1.0413641
28951	TRIB2	tribbles homolog 2 (Drosophila)	0.97405	0.864121	0.9885229
28952	CCDC22	coiled-coil domain containing 22	0.95971	0.96185	1.120294
28954	REM1	RAS (RAD and GEM)-like GTP-binding 1	0.99678	1.050057	0.9849439
28955	DEXI	Dexi homolog (mouse)	0.98917	0.994029	1.0253528
28956	LAMTOR2	late endosomal/lysosomal adaptor, MAPK and MTOR activator 2	0.99921	1.05519	0.9809758
28957	MRPS28	mitochondrial ribosomal protein S28	0.96767	0.993247	1.0065046
28958	CCDC56	coiled-coil domain containing 56	0.90179	1.029715	1.0118836
28959	TMEM176E	transmembrane protein 176B	1.0657	1.039611	0.9946176
28960	DCPS	decapping enzyme, scavenger	0.95371	0.963221	0.9942272
28962	OSTM1	osteopetrosis associated transmembrane protein 1	0.9851	0.921407	0.9543821
28964	GIT1	G protein-coupled receptor kinase interacting ArfGAP 1	0.94795	0.971279	1.0585307
28965	SLC27A6	solute carrier family 27 (fatty acid transporter), member 6	0.98712	1.032105	1.0125212
28966	SNX24	sorting nexin 24	0.99846	1.020763	1.0113848
28968	SLC6A16	solute carrier family 6, member 16	1.01062	1.032566	0.9643857
28969	BZW2	basic leucine zipper and W2 domains 2	0.98763	0.930601	1.0235892
28970	C11orf54	chromosome 11 open reading frame 54	0.91958	0.952773	1.0561004
28971	C11orf67	chromosome 11 open reading frame 67	0.9802	1.02024	1.0512134
28972	SPCS1	signal peptidase complex subunit 1 homolog (S. cerevisiae)	0.98353	0.951197	1.0172358
28973	MRPS18B	mitochondrial ribosomal protein S18B	0.96165	0.964718	1.0333191
28974	C19orf53	chromosome 19 open reading frame 53	0.94455	0.991408	1.0061741
28976	ACAD9	acyl-CoA dehydrogenase family, member 9	0.97307	0.94886	0.9477023
28977	MRPL42	mitochondrial ribosomal protein L42	0.91216	0.943805	1.0210806
28978	TMEM14A	transmembrane protein 14A	0.9974	0.980953	1.0608402
28981	IFT81	intraflagellar transport 81 homolog (Chlamydomonas)	0.97778	0.970687	1.0211071
28982	FLVCR1	feline leukemia virus subgroup C cellular receptor 1	0.99482	0.959026	1.0216762
28984	C13orf15	chromosome 13 open reading frame 15	0.93197	0.978308	0.934173
28985	MCTS1	malignant T cell amplified sequence 1	0.9668	1.055857	1.0086753
28986	MAGEH1	melanoma antigen family H, 1	0.90607	0.956959	1.00134
28987	NOB1	NIN1/RPN12 binding protein 1 homolog (S. cerevisiae)	0.94632	0.984783	1.0495435
28988	DBNL	drebrin-like	1.01177	0.972262	0.9429905

28989	METTL11A	methyltransferase like 11A	0.96304	0.972591	0.9635293
28990	ASTE1	asteroid homolog 1 (Drosophila)	0.94578	0.944057	1.0228213
28991	COMMD5	COMM domain containing 5	0.96543	1.034528	1.0574569
28992	MACROD1	MACRO domain containing 1	0.97882	0.994233	1.065981
28998	MRPL13	mitochondrial ribosomal protein L13	0.96542	0.974856	1.0335683
28999	KLF15	Kruppel-like factor 15	1.01966	1.02156	1.0379213
29015	SLC43A3	solute carrier family 43, member 3	0.94881	1.117794	1.0392157
29018	PRO1768	PRO1768	0.97077	1.089042	0.9672151
29023	[No Symbo	[No Name]	1.0093	1.013258	0.9815835
29028	ATAD2	ATPase family, AAA domain containing 2	0.96259	0.922517	1.0164983
29035	C16orf72	chromosome 16 open reading frame 72	0.99204	0.959082	0.9942197
29058	C20orf30	chromosome 20 open reading frame 30	0.92579	0.920856	0.9429789
29062	WDR91	WD repeat domain 91	0.99337	0.973763	0.9840434
29063	ZCCHC4	zinc finger, CCHC domain containing 4	0.97722	0.935197	0.9824071
29066	ZC3H7A	zinc finger CCCH-type containing 7A	0.94441	1.041725	0.9618873
29068	ZBTB44	zinc finger and BTB domain containing 44	0.92872	0.945487	0.9781557
29070	CCDC113	coiled-coil domain containing 113	1.0031	1.003358	1.000907
29071	C1GALT1C1	C1GALT1-specific chaperone 1	0.94778	0.956726	1.0491727
29072	SETD2	SET domain containing 2	1.00133	0.999084	0.951877
29074	MRPL18	mitochondrial ribosomal protein L18	0.96801	0.939902	1.0305791
29078	NDUFAF4	NADH dehydrogenase (ubiquinone) 1 alpha subcomplex, assembly factor 4	0.93709	0.99461	0.9764323
29079	MED4	mediator complex subunit 4	0.92622	0.911107	0.9830424
29080	CCDC59	coiled-coil domain containing 59	0.95804	0.908107	0.9077866
29081	METTL5	methyltransferase like 5	1.00034	0.927716	1.0526232
29082	CHMP4A	chromatin modifying protein 4A	0.90917	1.022183	0.9425657
29083	GTPBP8	GTP-binding protein 8 (putative)	0.943	0.927381	0.9129971
29085	PHPT1	phosphohistidine phosphatase 1	1.00122	1.023192	1.0591583
29086	BABAM1	BRISC and BRCA1 A complex member 1	0.94135	0.943275	1.135567
29087	THYN1	thymocyte nuclear protein 1	0.90631	0.927691	1.1178145
29088	MRPL15	mitochondrial ribosomal protein L15	0.96326	0.998189	1.0343607
29089	UBE2T	ubiquitin-conjugating enzyme E2T (putative)	0.99847	1.021941	1.0074644
29090	C18orf55	chromosome 18 open reading frame 55	0.95885	0.982103	1.0269108
29091	STXBP6	syntaxin binding protein 6 (amisyn)	0.99487	0.996615	1.0086686

29093	MRPL22	mitochondrial ribosomal protein L22	0.99165	0.98381	1.0801725
29094	HSPC159	galectin-related protein	0.94052	0.965927	0.9417193
29095	ORMDL2	ORM1-like 2 (S. cerevisiae)	0.92754	1.036461	1.1222248
29097	CNIH4	cornichon homolog 4 (Drosophila)	1.02427	1.037784	0.9904781
29098	RANGRF	RAN guanine nucleotide release factor	0.90483	1.000632	1.0102592
29099	COMMD9	COMM domain containing 9	0.92224	0.926726	1.026325
29100	TMEM208	transmembrane protein 208	0.93185	1.035178	1.0242541
29101	SSU72	SSU72 RNA polymerase II CTD phosphatase homolog (S. cerevisiae)	0.99041	0.987101	0.9523038
29102	DROSHA	drosha, ribonuclease type III	1.00288	1.001502	0.9947086
29103	DNAJC15	DnaJ (Hsp40) homolog, subfamily C, member 15	0.95051	0.905708	0.9903665
29104	N6AMT1	N-6 adenine-specific DNA methyltransferase 1 (putative)	0.94558	0.995864	0.9930775
29105	C16orf80	chromosome 16 open reading frame 80	0.90325	0.820959	1.0085849
29106	SCG3	secretogranin III	0.98786	0.983672	1.0424433
29107	NXT1	NTF2-like export factor 1	0.94786	0.938306	0.9865888
29108	PYCARD	PYD and CARD domain containing	0.94156	0.84975	1.0379829
29109	FHOD1	formin homology 2 domain containing 1	0.96574	1.054818	1.0190553
29110	TBK1	TANK-binding kinase 1	0.94306	1.012972	1.1585588
29113	C6orf15	chromosome 6 open reading frame 15	1.01989	0.983444	1.0163824
29114	TAGLN3	transgelin 3	1.01497	1.03539	1.0085206
29115	SAP30BP	SAP30 binding protein	0.97891	1.003907	0.9480787
29116	MYLIP	myosin regulatory light chain interacting protein	0.97072	0.87797	1.008058
29117	BRD7	bromodomain containing 7	0.97462	0.980208	0.9662636
29118	DDX25	DEAD (Asp-Glu-Ala-Asp) box polypeptide 25	0.99733	1.000365	1.0184787
29119	CTNNA3	catenin (cadherin-associated protein), alpha 3	0.99858	0.994729	0.9992507
29121	CLEC2D	C-type lectin domain family 2, member D	0.92309	1.05239	0.9399824
29122	PRSS50	protease, serine, 50	0.9887	0.993668	1.0192179
29123	ANKRD11	ankyrin repeat domain 11	0.99432	1.012591	0.9702916
29124	LGALS13	lectin, galactoside-binding, soluble, 13	1.02403	1.004573	1.0408956
29125	C11orf21	chromosome 11 open reading frame 21	0.98732	1.003198	0.9810103
29126	CD274	CD274 molecule	0.995	0.973805	1.0394446
29127	RACGAP1	Rac GTPase activating protein 1	0.94188	0.99725	1.0409384
29128	UHRF1	ubiquitin-like with PHD and ring finger domains 1	0.9901	1.003099	1.0341979
29760	BLNK	B-cell linker	0.99176	1.034649	0.9669609

29761	USP25	ubiquitin specific peptidase 25	0.93817	1.008111	0.9680734
29763	PACSIN3	protein kinase C and casein kinase substrate in neurons 3	0.99291	1.012258	1.0642709
29765	TMOD4	tropomodulin 4 (muscle)	0.97928	0.966777	0.995225
29766	TMOD3	tropomodulin 3 (ubiquitous)	0.95435	0.950409	0.9811983
29767	TMOD2	tropomodulin 2 (neuronal)	0.96085	1.002133	1.0136542
29775	CARD10	caspase recruitment domain family, member 10	1.00083	0.989601	1.0454545
29777	ABT1	activator of basal transcription 1	0.96188	0.873776	1.0980196
29780	PARVB	parvin, beta	0.96993	1.022939	0.9741964
29781	NCAPH2	non-SMC condensin II complex, subunit H2	0.95767	0.986572	1.0001617
29785	CYP2S1	cytochrome P450, family 2, subfamily S, polypeptide 1	0.98638	1.015131	0.9654249
29789	OLA1	Obg-like ATPase 1	0.99535	0.979161	1.0355361
29796	UQCR10	ubiquinol-cytochrome c reductase, complex III subunit X	0.93222	0.906549	1.0403826
29798	C2orf27A	chromosome 2 open reading frame 27A	1.02663	1.027296	1.044204
29799	YPEL1	yippee-like 1 (Drosophila)	0.9837	1.00781	0.9679801
29800	ZDHHC1	zinc finger, DHHC-type containing 1	0.98292	1.016488	1.0189086
29801	ZDHHC8	zinc finger, DHHC-type containing 8	0.99063	0.966856	1.0659377
29802	VPREB3	pre-B lymphocyte 3	0.91909	0.961355	1.0742853
29803	REPIN1	replication initiator 1	0.99738	0.934996	1.0446222
29841	GRHL1	grainyhead-like 1 (Drosophila)	1.01104	0.985901	0.9775594
29842	TFCP2L1	transcription factor CP2-like 1	0.99263	1.006584	0.9581305
29843	SENP1	SUMO1/sentrin specific peptidase 1	0.93324	1.0114	1.0717753
29844	TFPT	TCF3 (E2A) fusion partner (in childhood Leukemia)	0.93738	0.95134	1.0649245
29850	TRPM5	transient receptor potential cation channel, subfamily M, member 5	0.99328	1.064818	1.035981
29851	ICOS	inducible T-cell co-stimulator	0.91145	0.920008	0.9595532
29855	UBN1	ubinuclein 1	0.95549	0.999237	1.0180707
29880	ALG5	asparagine-linked glycosylation 5, dolichyl-phosphate beta-glucosyltransferase homolog (S. cerevisiae)	0.96136	0.96365	1.0649242
29881	NPC1L1	NPC1 (Niemann-Pick disease, type C1, gene)-like 1	1.0045	0.998497	1.0192845
29882	ANAPC2	anaphase promoting complex subunit 2	0.9606	0.9636	1.0692608
29883	CNOT7	CCR4-NOT transcription complex, subunit 7	0.95956	0.94815	1.0945561
29886	SNX8	sorting nexin 8	0.98899	1.012139	1.0422025
29887	SNX10	sorting nexin 10	1.01955	1.057751	1.0805408
29888	STRN4	striatin, calmodulin binding protein 4	0.94702	1.038959	1.0023916
29889	GNL2	guanine nucleotide binding protein-like 2 (nucleolar)	1.01249	0.893816	1.0185748

29890	RBM15B	RNA binding motif protein 15B	0.98899	0.921566	1.0132228
29893	PSMC3IP	PSMC3 interacting protein	0.9926	1.042459	1.0028746
29894	CPSF1	cleavage and polyadenylation specific factor 1, 160kDa	0.97014	0.935703	1.078097
29895	MYLPF	myosin light chain, phosphorylatable, fast skeletal muscle	0.99538	1.022743	1.0386663
29896	TRA2A	transformer 2 alpha homolog (Drosophila)	1.00284	0.867823	0.9261213
29899	GPSM2	G-protein signaling modulator 2	0.99677	0.949774	1.0173791
29901	SAC3D1	SAC3 domain containing 1	0.99312	0.980202	1.0511537
29902	C12orf24	chromosome 12 open reading frame 24	0.94335	0.927505	1.0034208
29903	CCDC106	coiled-coil domain containing 106	0.99899	1.049058	1.0223329
29904	EEF2K	eukaryotic elongation factor-2 kinase	0.95571	1.006895	1.0554678
29906	ST8SIA5	ST8 alpha-N-acetyl-neuraminide alpha-2,8-sialyltransferase 5	0.9861	0.9819	1.004674
29907	SNX15	sorting nexin 15	0.9296	0.878802	1.0575274
29909	GPR171	G protein-coupled receptor 171	0.99525	0.935488	1.0541799
29911	HOOK2	hook homolog 2 (Drosophila)	0.98642	0.997018	1.0566174
29914	UBIAD1	UbiA prenyltransferase domain containing 1	0.9693	0.905193	1.0284773
29915	HCFC2	host cell factor C2	0.93962	0.99065	1.1607581
29916	SNX11	sorting nexin 11	0.94596	1.03659	1.098137
29919	C18orf8	chromosome 18 open reading frame 8	0.94597	1.066994	0.9698995
29920	PYCR2	pyrroline-5-carboxylate reductase family, member 2	0.98709	0.916788	1.0634672
29922	NME7	non-metastatic cells 7, protein expressed in (nucleoside-diphosphate kinase)	0.98691	0.994477	1.0160282
29923	C7orf68	chromosome 7 open reading frame 68	1.02849	1.01466	0.9338417
29924	EPN1	epsin 1	0.94943	0.963473	1.0532549
29925	GMPPB	GDP-mannose pyrophosphorylase B	1.02739	1.005275	1.0451428
29926	GMPPA	GDP-mannose pyrophosphorylase A	0.97708	0.947005	1.0975381
29927	SEC61A1	Sec61 alpha 1 subunit (S. cerevisiae)	1.02543	1.017029	1.1106207
29928	TIMM22	translocase of inner mitochondrial membrane 22 homolog (yeast)	0.944	1.029457	1.0682734
29929	ALG6	asparagine-linked glycosylation 6, alpha-1,3-glucosyltransferase homolog (S. cerevisiae)	0.97303	0.986056	1.0323158
29930	PCDHB1	protocadherin beta 1	1.01885	1.047556	1.0533823
29933	GPR132	G protein-coupled receptor 132	0.94171	1.063602	0.9164187
29934	SNX12	sorting nexin 12	0.88533	0.970692	1.0812565
29937	NENF	neuron derived neurotrophic factor	0.98182	0.982692	1.0390869
29940	DSE	dermatan sulfate epimerase	1.01846	1.042462	1.0448334
29941	PKN3	protein kinase N3	0.98232	1.021064	1.0376597

29942	PURG	purine-rich element binding protein G	0.98854	0.981618	0.9885642
29943	PADI1	peptidyl arginine deiminase, type I	1.00859	1.016976	1.0110956
29944	PNMA3	paraneoplastic antigen MA3	0.95707	0.939763	1.0383089
29945	ANAPC4	anaphase promoting complex subunit 4	0.97463	0.986623	1.0282822
29946	SERTAD3	SERTA domain containing 3	0.97182	1.017904	0.9930047
29947	DNMT3L	DNA (cytosine-5-)-methyltransferase 3-like	0.99603	1.007481	1.0270342
29948	OSGIN1	oxidative stress induced growth inhibitor 1	1.00569	0.981836	1.0216713
29949	IL19	interleukin 19	1.01222	1.024298	1.0455447
29950	SERTAD1	SERTA domain containing 1	0.96975	0.97741	0.9791626
29951	PDZRN4	PDZ domain containing ring finger 4	1.01319	1.004231	1.0196499
29952	DPP7	dipeptidyl-peptidase 7	0.94356	0.943778	1.1223251
29953	TRHDE	thyrotropin-releasing hormone degrading enzyme	0.97932	0.988839	1.0091265
29954	POMT2	protein-O-mannosyltransferase 2	0.98425	1.00723	1.0231409
29956	LASS2	LAG1 homolog, ceramide synthase 2	0.97626	0.878457	1.0175618
29957	SLC25A24	solute carrier family 25 (mitochondrial carrier; phosphate carrier), member 24	1.00836	0.882353	1.0512044
29958	DMGDH	dimethylglycine dehydrogenase	1.00912	0.993715	0.9958089
29959	NRBP1	nuclear receptor binding protein 1	0.9628	1.056028	0.9491052
29960	FTSJ2	FtsJ homolog 2 (E. coli)	0.935	0.904879	1.0860522
29964	PRICKLE4	prickle homolog 4 (Drosophila)	0.99088	0.973068	0.9840471
29965	C16orf5	chromosome 16 open reading frame 5	0.96766	0.96249	0.9606933
29966	STRN3	striatin, calmodulin binding protein 3	0.95088	1.026369	1.0149678
29967	LRP12	low density lipoprotein receptor-related protein 12	0.9505	0.949577	1.100017
29968	PSAT1	phosphoserine aminotransferase 1	1.00077	0.978703	0.9911977
29969	MDFIC	MyoD family inhibitor domain containing	0.97578	0.999228	1.1425526
29970	SCHIP1	schwannomin interacting protein 1	1.0056	0.989233	1.005171
29974	A1CF	APOBEC1 complementation factor	0.99898	1.000935	1.0031239
29978	UBQLN2	ubiquilin 2	0.94838	0.955538	1.0266787
29979	UBQLN1	ubiquilin 1	0.93941	0.861108	1.0253853
29980	DONSON	downstream neighbor of SON	0.97395	0.971376	0.9923394
29982	NRBF2	nuclear receptor binding factor 2	0.99749	0.905643	1.050346
29984	RHOD	ras homolog gene family, member D	1.00027	0.992701	1.0058548
29985	SLC39A3	solute carrier family 39 (zinc transporter), member 3	0.90693	0.925678	1.1032047
29986	SLC39A2	solute carrier family 39 (zinc transporter), member 2	1.02326	1.028358	0.9792092

29988	SLC2A8	solute carrier family 2 (facilitated glucose transporter), member 8	0.97734	1.013989	1.051615
29989	OBP2B	odorant binding protein 2B	0.98273	1.015785	0.95656
29990	PILRB	paired immunoglobin-like type 2 receptor beta	1.00025	1.058807	0.8981508
29991	OBP2A	odorant binding protein 2A	0.93675	1.039416	1.0859381
29992	PILRA	paired immunoglobin-like type 2 receptor alpha	1.00609	1.045835	0.9600797
29993	PACSIN1	protein kinase C and casein kinase substrate in neurons 1	1.0021	1.007276	0.9971287
29994	BAZ2B	bromodomain adjacent to zinc finger domain, 2B	1.00997	0.951325	1.0461404
29995	LMCD1	LIM and cysteine-rich domains 1	1.01937	0.984629	0.9982298
29997	GLTSCR2	glioma tumor suppressor candidate region gene 2	0.95336	1.002543	0.9569677
29998	GLTSCR1	glioma tumor suppressor candidate region gene 1	0.97986	0.983038	0.9994358
29999	FSCN3	fascin homolog 3, actin-bundling protein, testicular (Strongylocentrotus purpuratus)	0.99955	1.007551	0.9483903
30000	TNPO2	transportin 2	0.93596	1.008522	1.0333061
30001	ERO1L	ERO1-like (S. cerevisiae)	0.9499	0.936696	1.0227219
30008	EFEMP2	EGF containing fibulin-like extracellular matrix protein 2	1.00713	1.038876	1.0256812
30009	TBX21	T-box 21	0.94586	1.078118	1.0332512
30010	NXPH1	neurexophilin 1	1.00106	1.000185	0.9990388
30011	SH3KBP1	SH3-domain kinase binding protein 1	0.99694	1.021938	1.0408667
30012	TLX3	T-cell leukemia homeobox 3	1.01068	0.917955	1.0824407
30061	SLC40A1	solute carrier family 40 (iron-regulated transporter), member 1	0.95816	0.89696	0.9046429
30062	RAX	retina and anterior neural fold homeobox	0.98377	1.013745	1.0565297
30811	HUNK	hormonally up-regulated Neu-associated kinase	0.99203	0.998701	1.0065431
30812	SOX8	SRY (sex determining region Y)-box 8	0.97413	1.005114	1.0813215
30813	VSX1	visual system homeobox 1	0.98355	0.99991	1.0338372
30814	PLA2G2E	phospholipase A2, group IIE	0.99267	0.972924	1.0431784
30815	ST6GALNA(	ST6 (alpha-N-acetyl-neuraminyl-2,3-beta-galactosyl-1,3)-N-acetylgalactosaminide alpha-2,6-sialyltransferase	0.95402	1.09827	1.0257742
30817	EMR2	egf-like module containing, mucin-like, hormone receptor-like 2	1.01066	0.9404	1.044233
30818	KCNIP3	Kv channel interacting protein 3, calsenilin	1.00017	1.005236	1.0090392
30820	KCNIP1	Kv channel interacting protein 1	1.01617	0.999721	1.0120666
30827	CXXC1	CXXC finger protein 1	0.94977	0.98289	1.0043459
30832	ZNF354C	zinc finger protein 354C	1.01927	0.916549	0.9617709
30833	NT5C	5', 3'-nucleotidase, cytosolic	0.97116	1.064917	1.1212555
30834	ZNRD1	zinc ribbon domain containing 1	0.97772	0.955845	0.9811292
30835	CD209	CD209 molecule	0.97665	0.971196	1.0006223

30836	DNTTIP2	deoxynucleotidyltransferase, terminal, interacting protein 2	0.98363	0.829578	0.9600807
30837	SOCS7	suppressor of cytokine signaling 7	0.96616	0.996263	1.038444
30844	EHD4	EH-domain containing 4	0.98976	0.922087	0.9865082
30845	EHD3	EH-domain containing 3	1.0088	0.957685	0.9899179
30846	EHD2	EH-domain containing 2	0.96489	1.021152	1.0204595
30848	CTAG2	cancer/testis antigen 2	0.97473	1.010724	1.0563953
30849	PIK3R4	phosphoinositide-3-kinase, regulatory subunit 4	0.98462	1.024817	1.0313351
30850	CDR2L	cerebellar degeneration-related protein 2-like	0.98207	0.987378	1.0045593
30851	TAX1BP3	Tax1 (human T-cell leukemia virus type I) binding protein 3	0.94503	0.946474	1.0523862
30968	STOML2	stomatin (EPB72)-like 2	0.92337	0.863705	1.0618831
43847	KLK14	kallikrein-related peptidase 14	0.99608	1.018908	1.0368677
43849	KLK12	kallikrein-related peptidase 12	0.99648	0.999914	1.0093754
49854	ZNF295	zinc finger protein 295	0.96172	0.938588	0.9357208
49855	SCAPER	S-phase cyclin A-associated protein in the ER	0.96683	1.01812	0.9991674
49856	WRAP73	WD repeat containing, antisense to TP73	1.01002	0.988697	1.0431274
49860	CRNN	cornulin	0.98971	0.981964	0.9817175
49861	CLDN20	claudin 20	1.01742	1.034401	1.0019351
50484	RRM2B	ribonucleotide reductase M2 B (TP53 inducible)	0.93751	1.018349	1.0992999
50485	SMARCAL1	SWI/SNF related, matrix associated, actin dependent regulator of chromatin, subfamily a-like 1	0.98045	0.952493	1.0233509
50486	G0S2	G0/G1switch 2	0.97548	0.974211	0.8929106
50487	PLA2G3	phospholipase A2, group III	1.02246	0.986341	1.0135567
50488	MINK1	misshapen-like kinase 1	0.96292	1.044907	0.968484
50489	CD207	CD207 molecule, langerin	1.00783	1.016392	0.9801068
50506	DUOX2	dual oxidase 2	0.99275	0.996293	0.9943453
50507	NOX4	NADPH oxidase 4	0.99893	1.018246	0.9994423
50508	NOX3	NADPH oxidase 3	1.01206	1.004044	0.9963455
50509	COL5A3	collagen, type V, alpha 3	0.98764	1.014715	1.0510099
50511	SYCP3	synaptonemal complex protein 3	0.99814	0.993112	1.0244295
50512	PODXL2	podocalyxin-like 2	1.00768	0.992948	0.9870038
50514	1-Dec	deleted in esophageal cancer 1	0.99573	0.963687	1.0163229
50515	CHST11	carbohydrate (chondroitin 4) sulfotransferase 11	1.01345	0.980644	1.031876
50604	IL20	interleukin 20	1.00373	1.010293	1.0125519
50613	UBQLN3	ubiquilin 3	1	0.982509	1.0211958

50614	GALNT9	UDP-N-acetyl-alpha-D-galactosamine:polypeptide N-acetylgalactosaminyltransferase 9 (GalNAc-T9)	0.98167	1.010567	1.0273967
50615	IL21R	interleukin 21 receptor	0.95404	1.153733	1.065481
50616	IL22	interleukin 22	1.00507	1.028096	1.0504225
50617	ATP6V0A4	ATPase, H+ transporting, lysosomal V0 subunit a4	1.01025	1.002123	0.9950834
50618	ITSN2	intersectin 2	0.96469	0.960412	0.9308625
50619	DEF6	differentially expressed in FDCP 6 homolog (mouse)	0.99841	1.014123	1.0069594
50624	CUZD1	CUB and zona pellucida-like domains 1	0.96759	0.980724	1.0405931
50626	CYHR1	cysteine/histidine-rich 1	0.96048	1.027715	1.0696418
50628	GEMIN4	gem (nuclear organelle) associated protein 4	0.93449	1.004384	1.0490945
50631	YBX1P1	Y box binding protein 1 pseudogene 1	0.94374	0.975157	1.1478167
50632	CALY	calcyon neuron-specific vesicular protein	1.0095	0.978373	1.0863081
50636	ANO7	anoctamin 7	1	1.002141	1.0392036
50640	PNPLA8	patatin-like phospholipase domain containing 8	1.00696	0.97892	0.9491655
50649	ARHGEF4	Rho guanine nucleotide exchange factor (GEF) 4	1.00051	1.004349	1.0184664
50650	ARHGEF3	Rho guanine nucleotide exchange factor (GEF) 3	0.97312	0.973893	0.9654823
50651	SLC45A1	solute carrier family 45, member 1	0.9929	0.992252	1.0013562
50674	NEUROG3	neurogenin 3	1.00766	1.014384	1.0603746
50700	RDH8	retinol dehydrogenase 8 (all-trans)	0.99841	0.987806	0.9817955
50717	DCAF8	DDB1 and CUL4 associated factor 8	1.00105	1.082464	0.9218895
50801	KCNK4	potassium channel, subfamily K, member 4	0.99053	0.991224	1.0545455
50802	IGK@	immunoglobulin kappa locus	1.0312	1.041442	1.0066498
50804	MYEF2	myelin expression factor 2	0.98877	0.972231	1.1240291
50805	IRX4	iroquois homeobox 4	0.97749	1.030458	1.1046875
50807	ASAP1	ArfGAP with SH3 domain, ankyrin repeat and PH domain 1	0.98367	1.054042	1.0154475
50808	AK3	adenylate kinase 3	0.95461	0.928593	1.0721873
50809	HP1BP3	heterochromatin protein 1, binding protein 3	1.0008	0.912645	1.0037658
50810	HDGFRP3	hepatoma-derived growth factor, related protein 3	0.9819	0.990755	1.0167923
50813	COPS7A	COP9 constitutive photomorphogenic homolog subunit 7A (Arabidopsis)	0.93394	0.923968	1.0112025
50814	NSDHL	NAD(P) dependent steroid dehydrogenase-like	0.93714	0.91023	1.028432
50831	TAS2R3	taste receptor, type 2, member 3	1.01826	1.034304	0.9042714
50832	TAS2R4	taste receptor, type 2, member 4	1.02293	1.016248	0.9583126
50833	TAS2R16	taste receptor, type 2, member 16	1.01135	0.982069	1.0549388
50834	TAS2R1	taste receptor, type 2, member 1	1.04283	0.888524	1.0841167

50835	TAS2R9	taste receptor, type 2, member 9	1.0222	0.957746	0.974947
50836	TAS2R8	taste receptor, type 2, member 8	1.02291	0.99148	0.9614722
50837	TAS2R7	taste receptor, type 2, member 7	1.00483	1.053147	0.9851118
50839	TAS2R10	taste receptor, type 2, member 10	0.96829	0.979503	1.072573
50846	DHH	desert hedgehog	0.99567	1.0219	0.9621737
50848	F11R	F11 receptor	0.99756	1.013277	1.0550054
50852	TRAT1	T cell receptor associated transmembrane adaptor 1	0.95437	0.91023	0.9476489
50853	VILL	villin-like	0.98103	0.977007	0.9968427
50854	C6orf48	chromosome 6 open reading frame 48	1.00481	0.958456	0.9900378
50855	PARD6A	par-6 partitioning defective 6 homolog alpha (C. elegans)	0.97617	1.000086	1.034812
50856	CLEC4A	C-type lectin domain family 4, member A	0.9948	1.024654	0.9558462
50859	SPOCK3	sparc/osteonectin, cwcv and kazal-like domains proteoglycan (testican) 3	0.99929	0.973369	1.0012162
50861	STMN3	stathmin-like 3	0.96893	0.962612	1.0010642
50862	RNF141	ring finger protein 141	0.95704	0.933621	1.0101133
50863	NTM	neurotrimin	1.00655	1.000184	1.0057765
50865	HEBP1	heme binding protein 1	0.94393	0.94356	1.0036104
50937	CDON	Cdon homolog (mouse)	0.98882	0.99621	0.9814106
50939	IMPG2	interphotoreceptor matrix proteoglycan 2	1.00622	1.000456	1.0114117
50940	PDE11A	phosphodiesterase 11A	1.00986	1.00168	1.0131126
50943	FOXP3	forkhead box P3	0.97714	0.968051	0.9842385
50944	SHANK1	SH3 and multiple ankyrin repeat domains 1	0.98997	0.960995	1.009763
50945	TBX22	T-box 22	0.99869	0.981156	1.0427111
50964	SOST	sclerostin	0.9717	0.961004	0.9769634
50999	TMED5	transmembrane emp24 protein transport domain containing 5	1.01243	0.995959	0.9735127
51000	SLC35B3	solute carrier family 35, member B3	1.00032	0.97626	0.9848546
51001	MTERFD1	MTERF domain containing 1	0.95159	0.916594	1.0191108
51002	TPRKB	TP53RK binding protein	0.99665	0.939713	0.9981392
51003	MED31	mediator complex subunit 31	0.93137	1.025237	1.0182049
51004	COQ6	coenzyme Q6 homolog, monooxygenase (S. cerevisiae)	0.99312	0.973537	1.0688466
51005	AMDHD2	amidohydrolase domain containing 2	1.00727	1.071281	0.9480391
51006	SLC35C2	solute carrier family 35, member C2	0.95892	1.000432	0.9935891
51008	ASCC1	activating signal cointegrator 1 complex subunit 1	0.95057	1.015411	1.0425403
51009	DERL2	Der1-like domain family, member 2	0.93288	0.982935	1.0786116

51010	EXOSC3	exosome component 3	0.9065	0.994328	1.0932587
51011	FAHD2A	fumarylacetoacetate hydrolase domain containing 2A	0.99865	0.928472	0.9786236
51012	SLMO2	slowmo homolog 2 (Drosophila)	0.9316	0.929267	1.1403509
51013	EXOSC1	exosome component 1	0.95374	1.019027	1.1591077
51015	ISOC1	isochorismatase domain containing 1	0.98612	0.937177	1.0597888
51016	FAM158A	family with sequence similarity 158, member A	0.98015	1.022675	0.9558128
51018	RRP15	ribosomal RNA processing 15 homolog (S. cerevisiae)	0.99489	0.930684	1.0343894
51019	CCDC53	coiled-coil domain containing 53	0.94559	1.001919	0.9856195
51020	HDDC2	HD domain containing 2	0.95547	0.987944	1.0292827
51021	MRPS16	mitochondrial ribosomal protein S16	0.95847	0.944483	1.0198975
51022	GLRX2	glutaredoxin 2	0.97749	0.941442	1.0554723
51023	MRPS18C	mitochondrial ribosomal protein S18C	0.98503	0.926936	0.9990886
51024	FIS1	fission 1 (mitochondrial outer membrane) homolog (S. cerevisiae)	0.94878	1.014095	1.0205317
51025	PAM16	presequence translocase-associated motor 16 homolog (S. cerevisiae)	1.00236	1.020524	0.9939186
51026	GOLT1B	golgi transport 1B	0.94293	0.884601	1.0894421
51027	BOLA1	bolA homolog 1 (E. coli)	0.96312	0.960524	0.9837234
51028	VPS36	vacuolar protein sorting 36 homolog (S. cerevisiae)	0.95736	0.955009	1.086428
51029	PPPDE1	PPPDE peptidase domain containing 1	1.00189	0.983477	1.0008647
51030	FAM18B1	family with sequence similarity 18, member B1	0.97714	0.917596	1.0303517
51031	GLOD4	glyoxalase domain containing 4	0.94155	0.903829	1.0222834
51032	CELA2B	chymotrypsin-like elastase family, member 2B	0.97445	1.00641	0.9888921
51035	UBXN1	UBX domain protein 1	0.93227	1.085712	0.9645703
51042	ZNF593	zinc finger protein 593	0.97201	0.994428	1.0357985
51043	ZBTB7B	zinc finger and BTB domain containing 7B	1.00117	1.022297	0.9631968
51046	ST8SIA3	ST8 alpha-N-acetyl-neuraminide alpha-2,8-sialyltransferase 3	0.99247	1.006548	0.9870634
51050	PI15	peptidase inhibitor 15	1.01511	0.998691	1.0115916
51052	PRLH	prolactin releasing hormone	0.99389	1.029623	1.0174087
51053	GMNN	geminin, DNA replication inhibitor	0.99037	0.980137	1.0281426
51054	PLEKHA8P1	pleckstrin homology domain containing, family A member 8 pseudogene 1	0.96117	0.971057	0.9720628
51056	LAP3	leucine aminopeptidase 3	1.05615	1.057794	1.144562
51057	WDPCP	WD repeat containing planar cell polarity effector	0.97609	0.934436	0.9683358
51058	ZNF691	zinc finger protein 691	0.98757	1.015055	1.0211919
51059	FAM135B	family with sequence similarity 135, member B	1.00873	0.989243	1.0156505

51060	TXNDC12	thioredoxin domain containing 12 (endoplasmic reticulum)	0.99811	1.013135	1.0677651
51061	TXNDC11	thioredoxin domain containing 11	0.96251	0.982473	1.0594953
51062	ATL1	atlastin GTPase 1	0.94521	0.942994	1.0024834
51063	CALHM2	calcium homeostasis modulator 2	0.99901	1.030395	1.1035789
51065	RPS27L	ribosomal protein S27-like	0.90934	0.916375	0.9920292
51066	C3orf32	chromosome 3 open reading frame 32	0.99991	1.032972	0.9995566
51067	YARS2	tyrosyl-tRNA synthetase 2, mitochondrial	0.93487	1.029052	1.0243081
51068	NMD3	NMD3 homolog (S. cerevisiae)	0.97004	0.967606	1.0463215
51069	MRPL2	mitochondrial ribosomal protein L2	0.95991	0.947633	1.0566323
51070	NOSIP	nitric oxide synthase interacting protein	0.93835	0.985896	0.9602714
51071	DERA	deoxyribose-phosphate aldolase (putative)	0.97238	0.943377	1.0413514
51072	MEMO1	mediator of cell motility 1	1.01559	0.99276	0.9374906
51073	MRPL4	mitochondrial ribosomal protein L4	0.96817	0.941222	1.0341415
51074	APIP	APAF1 interacting protein	1.0218	0.9711	0.9917748
51075	TMX2	thioredoxin-related transmembrane protein 2	0.95449	0.937846	1.0362478
51076	CUTC	cutC copper transporter homolog (E. coli)	0.94124	0.989308	1.0494409
51077	FCF1	FCF1 small subunit (SSU) processome component homolog (S. cerevisiae)	0.97088	0.987121	1.0062082
51078	THAP4	THAP domain containing 4	0.99734	0.980737	0.9807811
51079	NDUFA13	NADH dehydrogenase (ubiquinone) 1 alpha subcomplex, 13	0.96449	1.040298	1.0410099
51081	MRPS7	mitochondrial ribosomal protein S7	0.94617	0.946094	1.075825
51082	POLR1D	polymerase (RNA) I polypeptide D, 16kDa	0.93552	0.971182	1.1028151
51083	GAL	galanin prepropeptide	0.97631	0.99573	0.9912485
51084	CRYL1	crystallin, lambda 1	0.98973	0.987168	0.9776169
51085	MLXIPL	MLX interacting protein-like	0.98919	1.014854	0.9547811
51086	TNNI3K	TNNI3 interacting kinase	1.00844	0.996908	1.0054614
51087	YBX2	Y box binding protein 2	1.00395	1.032919	1.0130588
51088	KLHL5	kelch-like 5 (Drosophila)	0.97489	0.997726	0.9490909
51090	PLLP	plasmolipin	0.99955	1.010039	1.007405
51091	SEPSECS	Sep (O-phosphoserine) tRNA:Sec (selenocysteine) tRNA synthase	0.97461	0.892116	0.9299854
51092	SIDT2	SID1 transmembrane family, member 2	1.01863	0.986243	1.0525775
51093	RRNAD1	ribosomal RNA adenine dimethylase domain containing 1	0.99839	0.983075	1.0176021
51094	ADIPOR1	adiponectin receptor 1	1.02206	0.867222	0.9730525
51095	TRNT1	tRNA nucleotidyl transferase, CCA-adding, 1	0.95938	0.984945	1.0478395

51096	UTP18	UTP18, small subunit (SSU) processome component, homolog (yeast)	0.94786	0.988437	1.0835787
51097	SCCPDH	saccharopine dehydrogenase (putative)	0.99941	0.987235	1.0640809
51098	IFT52	intraflagellar transport 52 homolog (Chlamydomonas)	0.9242	0.951265	1.0422051
51099	ABHD5	abhydrolase domain containing 5	0.99632	0.953846	1.0341061
51100	SH3GLB1	SH3-domain GRB2-like endophilin B1	1.00025	0.977793	1.0597414
51101	FAM164A	family with sequence similarity 164, member A	1.00354	0.923052	1.0255069
51102	MECR	mitochondrial trans-2-enoyl-CoA reductase	0.99116	0.96161	1.0066577
51103	NDUFAF1	NADH dehydrogenase (ubiquinone) 1 alpha subcomplex, assembly factor 1	0.91682	0.976332	0.9685714
51104	FAM108B1	family with sequence similarity 108, member B1	0.95794	1.099107	1.1337996
51105	PHF20L1	PHD finger protein 20-like 1	1.00736	1.020835	0.9879843
51106	TFB1M	transcription factor B1, mitochondrial	0.96009	0.993809	0.9740453
51107	APH1A	anterior pharynx defective 1 homolog A (C. elegans)	0.97877	0.979971	1.0444629
51108	METTL9	methyltransferase like 9	0.9856	0.993384	0.9880473
51109	RDH11	retinol dehydrogenase 11 (all-trans/9-cis/11-cis)	0.94501	0.909056	1.0229247
51110	LACTB2	lactamase, beta 2	0.95825	0.971707	0.9777497
51111	SUV420H1	suppressor of variegation 4-20 homolog 1 (Drosophila)	0.96238	0.93037	1.0019589
51112	TTC15	tetratricopeptide repeat domain 15	0.98467	0.964239	0.9736141
51114	ZDHHC9	zinc finger, DHHC-type containing 9	0.99463	1.020868	1.0127889
51115	FAM82B	family with sequence similarity 82, member B	0.96151	0.985666	1.0165398
51116	MRPS2	mitochondrial ribosomal protein S2	0.93984	0.975563	1.0615867
51117	COQ4	coenzyme Q4 homolog (S. cerevisiae)	0.94438	0.909352	1.0415394
51118	UTP11L	UTP11-like, U3 small nucleolar ribonucleoprotein, (yeast)	0.96979	0.964032	1.1288145
51119	SBDS	Shwachman-Bodian-Diamond syndrome	1.0017	0.844128	0.9947592
51121	RPL26L1	ribosomal protein L26-like 1	0.98488	0.996385	0.999848
51122	COMMD2	COMM domain containing 2	0.97189	0.872423	1.0534284
51123	ZNF706	zinc finger protein 706	0.95009	1.007682	0.9560712
51124	IER3IP1	immediate early response 3 interacting protein 1	0.90455	1.032181	1.0920502
51125	GOLGA7	golgin A7	1.01358	1.014495	0.9715398
51126	NAA20	N(alpha)-acetyltransferase 20, NatB catalytic subunit	0.94264	0.932292	1.0842548
51127	TRIM17	tripartite motif containing 17	1.01295	0.968352	1.0305317
51128	SAR1B	SAR1 homolog B (S. cerevisiae)	0.99526	1.028896	1.049064
51129	ANGPTL4	angiopoietin-like 4	0.98171	1.048045	1.0135405
51130	ASB3	ankyrin repeat and SOCS box containing 3	0.98551	0.98007	0.9940701

51131	PHF11	PHD finger protein 11	0.98537	1.052753	0.9226604
51132	RLIM	ring finger protein, LIM domain interacting	0.93384	0.947861	0.947758
51133	KCTD3	potassium channel tetramerisation domain containing 3	0.9786	0.927346	1.0216287
51134	CCDC41	coiled-coil domain containing 41	0.97108	1.067844	1.0200522
51135	IRAK4	interleukin-1 receptor-associated kinase 4	0.95145	0.99923	0.944773
51136	RNFT1	ring finger protein, transmembrane 1	0.96578	0.902932	1.0055753
51138	COPS4	COP9 constitutive photomorphogenic homolog subunit 4 (Arabidopsis)	0.96752	0.97326	1.0645355
51141	INSIG2	insulin induced gene 2	0.99137	1.072549	1.0235438
51142	CHCHD2	coiled-coil-helix-coiled-coil-helix domain containing 2	0.97488	0.946314	0.9326561
51143	DYNC1LI1	dynein, cytoplasmic 1, light intermediate chain 1	1.02447	0.922328	1.0505439
51144	HSD17B12	hydroxysteroid (17-beta) dehydrogenase 12	0.9862	0.998688	0.9771277
51145	LOC51145	erythrocyte transmembrane protein	0.99384	0.96895	1.0247026
51147	ING4	inhibitor of growth family, member 4	0.91687	1.006093	0.9751648
51148	CERCAM	cerebral endothelial cell adhesion molecule	0.99136	0.987287	1.0461998
51149	C5orf45	chromosome 5 open reading frame 45	0.97695	1.012232	0.9910922
51150	SDF4	stromal cell derived factor 4	0.97708	1.006201	1.0299668
51151	SLC45A2	solute carrier family 45, member 2	1.02094	0.997205	1.0264077
51154	MRTO4	mRNA turnover 4 homolog (S. cerevisiae)	0.98567	0.990845	1.0347553
51155	HN1	hematological and neurological expressed 1	0.94981	0.966903	1.0281194
51156	SERPINA10	serpin peptidase inhibitor, clade A (alpha-1 antiproteinase, antitrypsin), member 10	1.01716	0.978361	0.9758737
51157	ZNF580	zinc finger protein 580	0.97611	1.063284	1.0649043
51160	VPS28	vacuolar protein sorting 28 homolog (S. cerevisiae)	0.92886	1.011412	1.0492154
51161	C3orf18	chromosome 3 open reading frame 18	0.98974	0.957875	1.0123372
51162	EGFL7	EGF-like-domain, multiple 7	0.99141	0.985244	1.0269207
51163	DBR1	debranching enzyme homolog 1 (S. cerevisiae)	0.97004	0.968439	1.1161167
51164	DCTN4	dynactin 4 (p62)	0.99228	0.979863	0.922063
51166	AADAT	aminoadipate aminotransferase	1.00576	1.016173	1.0303399
51167	CYB5R4	cytochrome b5 reductase 4	1.01099	0.916302	0.9981612
51168	MYO15A	myosin XVA	0.99024	1.008426	1.0138479
51170	HSD17B11	hydroxysteroid (17-beta) dehydrogenase 11	1.00079	0.909328	1.0528422
51171	HSD17B14	hydroxysteroid (17-beta) dehydrogenase 14	0.96303	0.982867	1.0154668
51172	NAGPA	N-acetylglucosamine-1-phosphodiester alpha-N-acetylglucosaminidase	0.97734	0.891436	1.0908343
51174	TUBD1	tubulin, delta 1	0.89182	1.034699	1.0647431

51175	TUBE1	tubulin, epsilon 1	0.98699	0.875017	1.0068975
51176	LEF1	lymphoid enhancer-binding factor 1	0.94798	0.925454	0.8695979
51177	PLEKHO1	pleckstrin homology domain containing, family O member 1	1.01243	0.972894	0.9236764
51179	HAO2	hydroxyacid oxidase 2 (long chain)	1.01214	1.001707	1.0081569
51181	DCXR	dicarbonyl/L-xylulose reductase	0.93569	0.978159	1.0477268
51182	HSPA14	heat shock 70kDa protein 14	0.92787	0.875289	1.0718266
51184	GPN3	GPN-loop GTPase 3	0.95446	1.046675	1.1003294
51185	CRBN	cereblon	1.00596	0.808818	1.0255858
51186	WBP5	WW domain binding protein 5	0.96007	1.002312	1.0001843
51187	RSL24D1	ribosomal L24 domain containing 1	1.01527	0.943155	1.0150611
51188	SS18L2	synovial sarcoma translocation gene on chromosome 18-like 2	0.97579	0.89373	1.0807478
51191	HERC5	hect domain and RLD 5	0.9534	0.934841	1.0289925
51192	CKLF	chemokine-like factor	0.979	0.941437	0.9752702
51193	ZNF639	zinc finger protein 639	1.00177	0.947621	0.9597911
51194	IPO11	importin 11	0.96817	0.980535	1.0388931
51195	RAPGEFL1	Rap guanine nucleotide exchange factor (GEF)-like 1	0.99573	1.061032	0.9503163
51196	PLCE1	phospholipase C, epsilon 1	0.99379	0.982868	0.9972031
51198	C9orf53	chromosome 9 open reading frame 53	0.97206	0.972267	1.0335365
51199	NIN	ninein (GSK3B interacting protein)	0.94482	0.985045	1.0183798
51200	CPA4	carboxypeptidase A4	1.0121	1.002998	0.9952411
51201	ZDHHC2	zinc finger, DHHC-type containing 2	0.98484	0.984682	0.9898872
51202	DDX47	DEAD (Asp-Glu-Ala-Asp) box polypeptide 47	0.92149	0.903617	1.0544468
51203	NUSAP1	nucleolar and spindle associated protein 1	0.93427	0.935625	1.0570378
51204	TACO1	translational activator of mitochondrially encoded cytochrome c oxidase I	0.91173	0.958261	1.052355
51205	ACP6	acid phosphatase 6, lysophosphatidic	0.97663	0.993658	0.9653738
51206	GP6	glycoprotein VI (platelet)	0.9117	0.91034	0.9867191
51207	DUSP13	dual specificity phosphatase 13	0.99409	1.020027	1.0024846
51208	CLDN18	claudin 18	0.99464	1.022692	1.0101574
51209	RAB9B	RAB9B, member RAS oncogene family	0.9764	0.947248	0.9970412
51213	LUZP4	leucine zipper protein 4	0.98231	1.035129	1.0238889
51214	IGF2AS	insulin-like growth factor 2 antisense	0.98272	0.978354	1.0328675
51218	GLRX5	glutaredoxin 5	0.99645	0.99447	1.0601346
51224	TCEB3B	transcription elongation factor B polypeptide 3B (elongin A2)	1.00576	0.942337	1.0368658

51225	ABI3	ABI family, member 3	1.00491	0.879036	1.0937364
51226	COPZ2	coatomer protein complex, subunit zeta 2	0.97879	1.023508	0.9868223
51227	PIGP	phosphatidylinositol glycan anchor biosynthesis, class P	0.95401	0.979883	0.9812909
51228	GLTP	glycolipid transfer protein	0.99587	0.966608	0.9588933
51230	PHF20	PHD finger protein 20	0.93632	1.110274	0.9262927
51231	VRK3	vaccinia related kinase 3	0.94484	1.02925	1.0116459
51232	CRIM1	cysteine rich transmembrane BMP regulator 1 (chordin-like)	1.01359	0.99964	1.0168661
51233	C22orf43	chromosome 22 open reading frame 43	1.007	1.025409	0.9201289
51234	TMEM85	transmembrane protein 85	0.93561	0.910265	1.0778365
51237	MZB1	marginal zone B and B1 cell-specific protein	1.03402	1.040231	1.0360958
51239	ANKRD39	ankyrin repeat domain 39	1.00494	1.006372	1.1033478
51241	COX16	COX16 cytochrome c oxidase assembly homolog (S. cerevisiae)	0.94426	0.964681	1.0510471
51244	C3orf19	chromosome 3 open reading frame 19	0.96793	0.90259	1.0166212
51246	SHISA5	shisa homolog 5 (Xenopus laevis)	1.0153	0.899483	1.1322789
51247	PAIP2	poly(A) binding protein interacting protein 2	1.00462	0.919415	0.9323839
51248	PDZD11	PDZ domain containing 11	0.97976	0.957952	1.0450093
51249	TMEM69	transmembrane protein 69	0.98645	0.94205	1.0623342
51250	C6orf203	chromosome 6 open reading frame 203	1.02982	0.914105	0.9818392
51251	NT5C3	5'-nucleotidase, cytosolic III	1.00782	1.005928	1.0421738
51252	FAM178B	family with sequence similarity 178, member B	1.0142	0.9703	1.0004412
51253	MRPL37	mitochondrial ribosomal protein L37	0.95694	0.918827	1.0002748
51255	RNF181	ring finger protein 181	0.98334	0.985802	1.0843762
51256	TBC1D7	TBC1 domain family, member 7	0.98403	0.940527	0.9196636
51257	2-Mar	membrane-associated ring finger (C3HC4) 2	0.97138	1.007003	1.012452
51258	MRPL51	mitochondrial ribosomal protein L51	0.91461	0.946434	0.9877618
51259	TMEM216	transmembrane protein 216	0.94503	0.981912	1.0056465
51260	CXorf26	chromosome X open reading frame 26	0.92942	1.029859	1.022307
51263	MRPL30	mitochondrial ribosomal protein L30	0.95963	0.930723	1.0438812
51264	MRPL27	mitochondrial ribosomal protein L27	0.94633	0.95722	1.0579444
51265	CDKL3	cyclin-dependent kinase-like 3	1.00178	0.994104	0.9723917
51266	CLEC1B	C-type lectin domain family 1, member B	0.81557	0.90953	0.9672091
51267	CLEC1A	C-type lectin domain family 1, member A	0.95886	1.014967	0.9802734
51268	PIPOX	pipecolic acid oxidase	0.96935	1.010103	0.986337

51270	TFDP3	transcription factor Dp family, member 3	0.98627	1.000971	1.0188385
51271	UBAP1	ubiquitin associated protein 1	0.96095	1.040014	1.008391
51272	BET1L	blocked early in transport 1 homolog (S. cerevisiae)-like	0.95245	0.946684	0.9553712
51274	KLF3	Kruppel-like factor 3 (basic)	1.01126	1.001478	0.957682
51276	ZNF571	zinc finger protein 571	0.94877	1.031836	0.9622292
51277	DNAJC27	DnaJ (Hsp40) homolog, subfamily C, member 27	0.99534	0.969445	0.9835612
51278	IER5	immediate early response 5	0.97766	0.934428	0.9727362
51279	C1RL	complement component 1, r subcomponent-like	0.96019	0.938256	1.0040849
51281	ANKMY1	ankyrin repeat and MYND domain containing 1	0.99268	0.997274	1.0092013
51282	SCAND1	SCAN domain containing 1	0.94323	0.980026	1.1584348
51283	BFAR	bifunctional apoptosis regulator	0.93056	1.052262	1.0957765
51284	TLR7	toll-like receptor 7	0.96717	1.072267	1.0344122
51285	RASL12	RAS-like, family 12	0.99601	1.009331	0.9997741
51286	CEND1	cell cycle exit and neuronal differentiation 1	0.99083	1.016004	1.006647
51287	CHCHD8	coiled-coil-helix-coiled-coil-helix domain containing 8	0.97985	0.986974	1.0185475
51289	RXFP3	relaxin/insulin-like family peptide receptor 3	0.98539	0.969453	1.0214881
51290	ERGIC2	ERGIC and golgi 2	0.91616	0.94835	0.9933004
51291	GMIP	GEM interacting protein	0.98741	1.01467	0.9555094
51292	GMPR2	guanosine monophosphate reductase 2	0.95524	1.009014	1.0701204
51293	CD320	CD320 molecule	0.94651	1.064014	1.0717578
51294	PCDH12	protocadherin 12	1.01376	1.006137	0.974384
51295	ECSIT	ECSIT homolog (Drosophila)	0.97241	0.982688	1.0270084
51296	SLC15A3	solute carrier family 15, member 3	0.97943	1.044821	0.9367233
51297	PLUNC	palate, lung and nasal epithelium associated	1.01489	0.980965	0.9715857
51298	THEG	Theg homolog (mouse)	0.99901	1.048116	1.0205808
51299	NRN1	neuritin 1	0.99452	1.017602	1.0482414
51300	C3orf1	chromosome 3 open reading frame 1	0.98367	0.994939	0.9864266
51301	GCNT4	glucosaminyl (N-acetyl) transferase 4, core 2	0.92287	0.903226	0.9308683
51302	CYP39A1	cytochrome P450, family 39, subfamily A, polypeptide 1	1.0035	0.982666	0.9994936
51303	FKBP11	FK506 binding protein 11, 19 kDa	0.98796	1.111127	0.9236046
51304	ZDHHC3	zinc finger, DHHC-type containing 3	1.01457	0.99967	0.9956907
51305	KCNK9	potassium channel, subfamily K, member 9	1.00793	1.010441	1.0210867
51306	FAM13B	family with sequence similarity 13, member B	1.00456	0.984074	1.0044262

51307	FAM53C	family with sequence similarity 53, member C	1.01281	0.989004	0.9654229
51308	REEP2	receptor accessory protein 2	0.99788	1.003288	1.0259238
51309	ARMCX1	armadillo repeat containing, X-linked 1	0.99975	0.967938	0.9686301
51310	SLC22A17	solute carrier family 22, member 17	0.95972	0.989017	0.9336711
51311	TLR8	toll-like receptor 8	0.96289	0.927327	1.0400206
51312	SLC25A37	solute carrier family 25, member 37	0.99497	0.872789	1.0019456
51313	FAM198B	family with sequence similarity 198, member B	1.05255	0.842701	1.0269593
51314	TXNDC3	thioredoxin domain containing 3 (spermatozoa)	1.00298	0.972675	0.9692881
51315	KRCC1	lysine-rich coiled-coil 1	0.96703	0.958814	0.9580422
51316	PLAC8	placenta-specific 8	0.97718	0.922346	0.9155942
51317	PHF21A	PHD finger protein 21A	0.99927	1.009562	1.0750595
51318	MRPL35	mitochondrial ribosomal protein L35	0.97976	0.997507	1.0356838
51319	RSRC1	arginine/serine-rich coiled-coil 1	1.00558	0.887533	1.0033739
51320	MEX3C	mex-3 homolog C (C. elegans)	0.97726	0.975251	1.0086752
51321	AMZ2	archaelysin family metallopeptidase 2	0.93936	1.08089	0.967246
51322	WAC	WW domain containing adaptor with coiled-coil	0.94483	1.077338	0.9842992
51324	SPG21	spastic paraplegia 21 (autosomal recessive, Mast syndrome)	0.96214	1.028696	1.1260773
51327	AHSP	alpha hemoglobin stabilizing protein	1.01007	1.008439	1.0046615
51329	ARL6IP4	ADP-ribosylation-like factor 6 interacting protein 4	0.95172	0.97476	1.0590034
51330	TNFRSF12A	tumor necrosis factor receptor superfamily, member 12A	1.00045	0.991141	0.981435
51332	SPTBN5	spectrin, beta, non-erythrocytic 5	0.99247	1.001987	1.0381147
51333	ZNF771	zinc finger protein 771	1.00162	1.021894	1.0438282
51334	PRR16	proline rich 16	0.98274	1.021288	0.9951682
51335	NGRN	neugrin, neurite outgrowth associated	0.92328	0.974504	1.0699959
51337	C8orf55	chromosome 8 open reading frame 55	0.97749	1.022992	1.050343
51338	MS4A4A	membrane-spanning 4-domains, subfamily A, member 4	1.0011	0.903499	0.9769942
51339	DACT1	dapper, antagonist of beta-catenin, homolog 1 (Xenopus laevis)	0.89147	0.960486	1.0330046
51340	CRNKL1	crooked neck pre-mRNA splicing factor-like 1 (Drosophila)	0.91507	0.954121	1.1209081
51341	ZBTB7A	zinc finger and BTB domain containing 7A	0.95315	0.965989	0.9673063
51343	FZR1	fizzy/cell division cycle 20 related 1 (Drosophila)	0.93137	1.024067	1.0107837
51347	TAOK3	TAO kinase 3	0.95752	1.024501	1.0613325
51348	KLRF1	killer cell lectin-like receptor subfamily F, member 1	0.94912	1.032147	0.9050255
51350	KRT76	keratin 76	1.01773	1.014944	0.9835175

51351	ZNF117	zinc finger protein 117	1.00495	1.007451	0.9744705
51352	WT1-AS	WT1 antisense RNA (non-protein coding)	0.99806	0.96574	0.9981218
51360	MBTPS2	membrane-bound transcription factor peptidase, site 2	0.9494	0.961527	1.0476304
51361	HOOK1	hook homolog 1 (Drosophila)	0.97302	0.933388	0.9975135
51362	CDC40	cell division cycle 40 homolog (S. cerevisiae)	0.99692	0.769528	1.0880256
51363	CHST15	carbohydrate (N-acetylgalactosamine 4-sulfate 6-O) sulfotransferase 15	0.99693	1.068899	0.9593661
51364	ZMYND10	zinc finger, MYND-type containing 10	1.00838	1	1.0254666
51365	PLA1A	phospholipase A1 member A	1.0032	0.999815	0.9972428
51366	UBR5	ubiquitin protein ligase E3 component n-recognin 5	0.94047	1.129343	1.0639658
51367	POP5	processing of precursor 5, ribonuclease P/MRP subunit (S. cerevisiae)	0.91516	0.906916	1.0447423
51368	TEX264	testis expressed 264	0.9888	0.983359	1.0220226
51371	POMP	proteasome maturation protein	0.96391	0.976524	1.0230792
51372	CCDC72	coiled-coil domain containing 72	1.04093	0.997901	0.9496834
51373	MRPS17	mitochondrial ribosomal protein S17	1.01567	0.918249	0.9817725
51374	C2orf28	chromosome 2 open reading frame 28	0.98325	0.92701	1.0611282
51375	SNX7	sorting nexin 7	0.99543	1.006105	1.0356798
51377	UCHL5	ubiquitin carboxyl-terminal hydrolase L5	0.99405	0.950032	1.0172191
51378	ANGPT4	angiopoietin 4	1.00028	0.983832	1.0129786
51379	CRLF3	cytokine receptor-like factor 3	0.95321	1.019534	1.0010424
51380	CSAD	cysteine sulfinic acid decarboxylase	1.01887	0.992382	0.9666416
51382	ATP6V1D	ATPase, H+ transporting, lysosomal 34kDa, V1 subunit D	0.95552	0.984827	1.0553091
51384	WNT16	wingless-type MMTV integration site family, member 16	1.01224	0.960675	1.0064424
51385	ZNF589	zinc finger protein 589	1.00383	0.935389	1.0244224
51386	EIF3L	eukaryotic translation initiation factor 3, subunit L	0.96502	0.908209	1.1409051
51388	NIP7	nuclear import 7 homolog (S. cerevisiae)	0.93948	0.980787	1.0994517
51389	RWDD1	RWD domain containing 1	1.0118	0.92634	0.9798746
51390	AIG1	androgen-induced 1	0.99732	0.975996	1.0284615
51393	TRPV2	transient receptor potential cation channel, subfamily V, member 2	0.94471	1.014159	1.0582825
51397	COMMD10	COMM domain containing 10	0.99368	0.899748	1.006128
51398	C19orf56	chromosome 19 open reading frame 56	0.98789	0.886136	1.0865851
51399	TRAPPC4	trafficking protein particle complex 4	0.93815	0.938096	1.0109346
51400	PPME1	protein phosphatase methylesterase 1	0.97	1.034247	1.0258776
51406	NOL7	nucleolar protein 7, 27kDa	0.98387	0.891207	1.0434028

51409	HEMK1	HemK methyltransferase family member 1	0.97742	0.937966	1.0235354
51411	BIN2	bridging integrator 2	0.94851	0.887415	0.9515881
51412	ACTL6B	actin-like 6B	1.00009	1.020999	1.0124767
51421	AMOTL2	angiomotin like 2	1.00465	1.011504	1.0043242
51422	PRKAG2	protein kinase, AMP-activated, gamma 2 non-catalytic subunit	0.9987	1.010788	0.9876099
51426	POLK	polymerase (DNA directed) kappa	0.98443	0.956042	1.0873053
51427	ZNF107	zinc finger protein 107	0.95326	0.944144	0.9609508
51428	DDX41	DEAD (Asp-Glu-Ala-Asp) box polypeptide 41	0.984	0.945948	1.1214068
51429	SNX9	sorting nexin 9	0.98544	0.926675	0.9849969
51430	C1orf9	chromosome 1 open reading frame 9	0.98161	0.979671	0.9290282
51433	ANAPC5	anaphase promoting complex subunit 5	0.98344	0.964674	1.0063651
51434	ANAPC7	anaphase promoting complex subunit 7	0.98524	0.958161	1.0197239
51435	SCARA3	scavenger receptor class A, member 3	0.99298	0.992383	1.0257972
51438	MAGEC2	melanoma antigen family C, 2	1.01351	0.985575	1.0590416
51439	FAM8A1	family with sequence similarity 8, member A1	0.97761	0.954098	0.9944904
51440	HPCAL4	hippocalcin like 4	0.95185	0.947381	0.9587994
51441	YTHDF2	YTH domain family, member 2	1.0066	0.929782	1.0204208
51442	VGLL1	vestigial like 1 (Drosophila)	0.986	1.007358	1.0145369
51444	RNF138	ring finger protein 138	0.96849	1.032238	0.9893912
51447	IP6K2	inositol hexakisphosphate kinase 2	0.98051	0.957502	0.9516878
51449	PCYOX1	prenylcysteine oxidase 1	0.95011	0.984803	1.0444922
51450	PRRX2	paired related homeobox 2	0.99401	0.982535	1.0334218
51451	LCMT1	leucine carboxyl methyltransferase 1	0.96685	0.963971	1.0525112
51454	GULP1	GULP, engulfment adaptor PTB domain containing 1	0.99937	0.997129	1.0001383
51455	REV1	REV1 homolog (S. cerevisiae)	0.99405	0.976448	0.9959303
51458	RHCG	Rh family, C glycoprotein	0.99886	0.998971	1.0299807
51460	SFMBT1	Scm-like with four mbt domains 1	0.96299	0.903102	0.9504334
51465	UBE2J1	ubiquitin-conjugating enzyme E2, J1, U	1.01328	1.034963	1.0609383
51466	EVL	Enah/Vasp-like	0.94605	0.999448	0.9293482
51471	NAT8B	N-acetyltransferase 8B (GCN5-related, putative, gene/pseudogene)	0.95889	0.979901	0.9225708
51473	DCDC2	doublecortin domain containing 2	1.00928	1.03135	0.9974057
51474	LIMA1	LIM domain and actin binding 1	0.96109	1.041386	0.9944251
51475	CABP2	calcium binding protein 2	0.99311	0.981656	1.0705509

51477	ISYNA1	inositol-3-phosphate synthase 1	0.96569	1.103383	1.0215239
51478	HSD17B7	hydroxysteroid (17-beta) dehydrogenase 7	1.02382	0.884236	1.1243644
51479	ANKFY1	ankyrin repeat and FYVE domain containing 1	0.95444	1.002394	1.015804
51490	C9orf114	chromosome 9 open reading frame 114	0.92652	0.934376	1.0302056
51491	NOP16	NOP16 nucleolar protein homolog (yeast)	0.96126	1.024405	1.0217401
51493	C22orf28	chromosome 22 open reading frame 28	0.98716	0.989199	0.975689
51495	PTPLAD1	protein tyrosine phosphatase-like A domain containing 1	0.96309	1.004639	1.0198037
51496	CTDSPL2	CTD (carboxy-terminal domain, RNA polymerase II, polypeptide A) small phosphatase like 2	0.94087	0.964701	1.0225712
51497	TH1L	TH1-like (Drosophila)	0.90405	0.991361	1.0533303
51499	TRIAP1	TP53 regulated inhibitor of apoptosis 1	0.93089	1.009866	1.0612308
51501	C11orf73	chromosome 11 open reading frame 73	0.94414	1.037566	1.0152916
51503	CWC15	CWC15 spliceosome-associated protein homolog (S. cerevisiae)	0.92034	0.941242	1.1012127
51504	TRMT112	tRNA methyltransferase 11-2 homolog (S. cerevisiae)	0.93802	0.93841	1.0640548
51506	UFC1	ubiquitin-fold modifier conjugating enzyme 1	0.96736	0.918892	1.0561002
51507	C20orf43	chromosome 20 open reading frame 43	0.97108	1.010796	0.9671365
51510	CHMP5	chromatin modifying protein 5	0.97786	0.976141	1.0209828
51512	GTSE1	G-2 and S-phase expressed 1	1.00074	0.989521	1.0106007
51513	ETV7	ets variant 7	0.99463	0.999013	1.0181957
51514	DTL	denticleless homolog (Drosophila)	0.99323	0.958037	1.0190819
51517	NCKIPSD	NCK interacting protein with SH3 domain	1.0004	0.989242	1.0031586
51520	LARS	leucyl-tRNA synthetase	0.9858	0.871806	1.031669
51522	TMEM14C	transmembrane protein 14C	0.96961	0.948356	1.0578155
51523	CXXC5	CXXC finger protein 5	0.98374	0.938264	1.0331405
51524	TMEM138	transmembrane protein 138	0.95318	0.957261	0.9863195
51526	C20orf111	chromosome 20 open reading frame 111	0.93231	0.904319	0.9841291
51527	C14orf129	chromosome 14 open reading frame 129	0.95166	0.947691	1.1025632
51528	JKAMP	JNK1/MAPK8-associated membrane protein	0.94959	0.96332	1.1720767
51529	ANAPC11	anaphase promoting complex subunit 11	0.9923	1.008836	1.034468
51530	ZC3HC1	zinc finger, C3HC-type containing 1	0.99009	0.937158	1.0053306
51531	C9orf156	chromosome 9 open reading frame 156	0.91334	0.979539	0.9488041
51533	PHF7	PHD finger protein 7	1.0276	0.971999	0.9678624
51534	VTA1	Vps20-associated 1 homolog (S. cerevisiae)	0.99564	0.965799	1.1253199
51535	PPHLN1	periphilin 1	0.94896	0.88485	0.9703946

51537	MTFP1	mitochondrial fission process 1	1.00814	1.057628	0.9835165
51538	ZCCHC17	zinc finger, CCHC domain containing 17	0.99105	1.02838	0.9478142
51540	SCLY	selenocysteine lyase	0.98547	0.989966	1.0283713
51542	VPS54	vacuolar protein sorting 54 homolog (S. cerevisiae)	0.98883	0.975276	1.0246999
51545	ZNF581	zinc finger protein 581	0.92361	0.963427	1.0206202
51547	SIRT7	sirtuin 7	0.95917	0.977377	1.0330444
51548	SIRT6	sirtuin 6	0.96906	0.994542	1.0297014
51550	CINP	cyclin-dependent kinase 2 interacting protein	0.9787	1.002589	1.0134312
51552	RAB14	RAB14, member RAS oncogene family	0.95734	0.881	1.0000314
51554	CCRL1	chemokine (C-C motif) receptor-like 1	1.0251	1.042572	1.032278
51555	PEX5L	peroxisomal biogenesis factor 5-like	1.00367	1.008061	1.010517
51557	LGSN	lengsin, lens protein with glutamine synthetase domain	1.02148	1.008231	0.998591
51559	NT5DC3	5'-nucleotidase domain containing 3	1.0064	0.974361	1.053339
51560	RAB6B	RAB6B, member RAS oncogene family	0.99769	0.995528	0.9902494
51561	IL23A	interleukin 23, alpha subunit p19	0.95152	1.006917	0.9761435
51562	MBIP	MAP3K12 binding inhibitory protein 1	0.94669	0.879303	1.0427506
51564	HDAC7	histone deacetylase 7	0.97315	0.992577	0.9458747
51566	ARMCX3	armadillo repeat containing, X-linked 3	0.95679	0.957696	1.0534847
51567	TDP2	tyrosyl-DNA phosphodiesterase 2	1.00774	0.966593	0.9268987
51569	UFM1	ubiquitin-fold modifier 1	0.95242	0.926946	1.1124227
51571	FAM49B	family with sequence similarity 49, member B	0.96363	1.074948	1.0438349
51573	GDE1	glycerophosphodiester phosphodiesterase 1	0.94464	0.969438	1.0891621
51574	LARP7	La ribonucleoprotein domain family, member 7	0.97689	0.909987	1.0262026
51575	ESF1	ESF1, nucleolar pre-rRNA processing protein, homolog (S. cerevisiae)	0.92967	0.971208	1.0458416
51582	AZIN1	antizyme inhibitor 1	0.96682	1.028195	1.1156316
51585	PCF11	PCF11, cleavage and polyadenylation factor subunit, homolog (S. cerevisiae)	0.94992	1	1.0153676
51586	MED15	mediator complex subunit 15	0.95451	1.013993	0.9855313
51588	PIAS4	protein inhibitor of activated STAT, 4	0.93624	0.984111	1.0503412
51592	TRIM33	tripartite motif containing 33	1.012	0.932999	1.0027597
51593	SRRT	serrate RNA effector molecule homolog (Arabidopsis)	0.98547	1.022024	1.0018973
51594	NBAS	neuroblastoma amplified sequence	0.9762	0.930649	1.035325
51596	CUTA	cutA divalent cation tolerance homolog (E. coli)	0.97969	0.985955	1.0094055
51599	LSR	lipolysis stimulated lipoprotein receptor	0.95611	0.982281	1.010286

51601	LIPT1	lipoyltransferase 1	0.95816	0.940435	1.0302829
51602	NOP58	NOP58 ribonucleoprotein homolog (yeast)	0.99082	1.008264	0.97578
51603	METTL13	methyltransferase like 13	0.94655	0.965269	1.0148594
51604	PIGT	phosphatidylinositol glycan anchor biosynthesis, class T	0.95289	1.018981	0.9712176
51605	TRMT6	tRNA methyltransferase 6 homolog (S. cerevisiae)	0.95352	0.925775	1.0770321
51606	ATP6V1H	ATPase, H+ transporting, lysosomal 50/57kDa, V1 subunit H	0.94963	0.926382	1.0431853
51608	GET4	golgi to ER traffic protein 4 homolog (S. cerevisiae)	0.98683	1.057497	1.0055266
51611	DPH5	DPH5 homolog (S. cerevisiae)	1.00452	0.982953	1.0057322
51614	ERGIC3	ERGIC and golgi 3	0.979	0.937933	1.1286779
51616	TAF9B	TAF9B RNA polymerase II, TATA box binding protein (TBP)-associated factor, 31kDa	0.86464	0.843423	1.0623898
51617	HMP19	HMP19 protein	1.00302	1.010102	1.030475
51619	UBE2D4	ubiquitin-conjugating enzyme E2D 4 (putative)	1.01764	0.959635	1.0193819
51621	KLF13	Kruppel-like factor 13	0.96889	0.955073	0.9448707
51626	DYNC2LI1	dynein, cytoplasmic 2, light intermediate chain 1	0.99761	0.975226	1.0061845
51629	SLC25A39	solute carrier family 25, member 39	0.98116	0.956686	1.0473489
51631	LUC7L2	LUC7-like 2 (S. cerevisiae)	0.99504	0.839219	0.9100701
51633	OTUD6B	OTU domain containing 6B	1.008	0.870455	1.0113618
51634	RBMX2	RNA binding motif protein, X-linked 2	0.9718	0.982582	1.0240833
51635	DHRS7	dehydrogenase/reductase (SDR family) member 7	0.95199	0.963074	1.0338511
51637	C14orf166	chromosome 14 open reading frame 166	0.91616	0.943538	0.9925007
51639	SF3B14	splicing factor 3B, 14 kDa subunit	0.97453	0.979754	1.0014483
51642	MRPL48	mitochondrial ribosomal protein L48	0.99028	0.978942	0.9908052
51643	TMBIM4	transmembrane BAX inhibitor motif containing 4	0.98281	1.0185	1.0537618
51645	PPIL1	peptidylprolyl isomerase (cyclophilin)-like 1	0.92091	0.896574	1.015481
51646	YPEL5	yippee-like 5 (Drosophila)	1.02289	0.87175	0.9647869
51647	FAM96B	family with sequence similarity 96, member B	0.95905	1.072042	1.1228717
51649	MRPS23	mitochondrial ribosomal protein S23	0.92245	0.989504	1.096656
51650	MRPS33	mitochondrial ribosomal protein S33	0.95152	0.952205	0.9418539
51651	PTRH2	peptidyl-tRNA hydrolase 2	0.95208	0.980741	1.0310667
51652	VPS24	vacuolar protein sorting 24 homolog (S. cerevisiae)	1.0056	0.958351	0.9841391
51654	CDK5RAP1	CDK5 regulatory subunit associated protein 1	0.95328	0.985025	1.0021586
51655	RASD1	RAS, dexamethasone-induced 1	0.99483	1.055032	1.0667109
51657	STYXL1	serine/threonine/tyrosine interacting-like 1	1.01281	0.956783	1.0371668

51659	GINS2	GINS complex subunit 2 (Psf2 homolog)	1.01703	1.021831	1.0113218
51660	BRP44L	brain protein 44-like	1.03756	0.969131	0.9580243
51661	FKBP7	FK506 binding protein 7	1.00736	1.011365	1.0069454
51663	ZFR	zinc finger RNA binding protein	0.99098	0.929296	0.9897987
51665	ASB1	ankyrin repeat and SOCS box containing 1	0.96856	0.968353	1.0837782
51666	ASB4	ankyrin repeat and SOCS box containing 4	1.00697	1.018129	1.0163856
51667	NUB1	negative regulator of ubiquitin-like proteins 1	1.00468	0.982093	0.9898176
51668	HSPB11	heat shock protein family B (small), member 11	0.99103	1.025136	1.010405
51669	TMEM66	transmembrane protein 66	0.97161	0.965502	0.9462543
51673	TPPP3	tubulin polymerization-promoting protein family member 3	0.97674	0.972644	0.9964367
51676	ASB2	ankyrin repeat and SOCS box containing 2	1.01071	1.015567	0.9749159
51678	MPP6	membrane protein, palmitoylated 6 (MAGUK p55 subfamily member 6)	0.97152	0.9901	0.9617981
51684	SUFU	suppressor of fused homolog (Drosophila)	0.98805	0.965685	0.9881205
51686	OAZ3	ornithine decarboxylase antizyme 3	1.00599	1.002588	0.9942831
51690	LSM7	LSM7 homolog, U6 small nuclear RNA associated (S. cerevisiae)	0.95088	1.03736	1.0492563
51691	NAA38	N(alpha)-acetyltransferase 38, NatC auxiliary subunit	1.00687	0.852732	1.0479438
51692	CPSF3	cleavage and polyadenylation specific factor 3, 73kDa	0.99908	0.928929	1.0131547
51693	TRAPPC2L	trafficking protein particle complex 2-like	0.93676	0.958238	1.0326911
51696	HECA	headcase homolog (Drosophila)	0.99557	0.927516	0.9757687
51699	VPS29	vacuolar protein sorting 29 homolog (S. cerevisiae)	0.96863	0.938659	1.0557444
51700	CYB5R2	cytochrome b5 reductase 2	1.00473	0.99721	1.0053064
51701	NLK	nemo-like kinase	0.96842	1.014874	1.1032146
51702	PADI3	peptidyl arginine deiminase, type III	1.01191	1.055698	1.0046656
51703	ACSL5	acyl-CoA synthetase long-chain family member 5	0.9655	0.975076	0.970142
51704	GPRC5B	G protein-coupled receptor, family C, group 5, member B	0.97829	0.985508	1.0122393
51705	EMCN	endomucin	1.00185	0.990386	1.0190175
51706	CYB5R1	cytochrome b5 reductase 1	0.98336	0.988664	0.9333821
51710	ZNF44	zinc finger protein 44	0.93911	0.967872	0.9350463
51714	SELT	selenoprotein T	1.00286	0.97321	0.9612668
51715	RAB23	RAB23, member RAS oncogene family	1.01498	0.988077	1.0410109
51719	CAB39	calcium binding protein 39	0.99857	0.928125	1.0281458
51720	UIMC1	ubiquitin interaction motif containing 1	0.99533	0.982968	1.0731928
51725	FBXO40	F-box protein 40	0.99358	0.983852	0.963459

51726	DNAJB11	DnaJ (Hsp40) homolog, subfamily B, member 11	1.02939	0.991658	0.9714447
51727	CMPK1	cytidine monophosphate (UMP-CMP) kinase 1, cytosolic	1.00665	0.96153	0.9586061
51728	POLR3K	polymerase (RNA) III (DNA directed) polypeptide K, 12.3 kDa	0.97076	0.949838	1.0394083
51729	WBP11	WW domain binding protein 11	0.94013	0.913517	0.9392562
51733	UPB1	ureidopropionase, beta	0.96495	1.050659	1.0004927
51734	SEPX1	selenoprotein X, 1	1.01258	1.041324	1.0571895
51735	RAPGEF6	Rap guanine nucleotide exchange factor (GEF) 6	0.948	0.978178	0.9089086
51738	GHRL	ghrelin/obestatin prepropeptide	0.99246	0.975071	0.9622293
51741	WWOX	WW domain containing oxidoreductase	0.99211	0.981682	0.9703165
51742	ARID4B	AT rich interactive domain 4B (RBP1-like)	0.98279	0.987753	0.9737886
51744	CD244	CD244 molecule, natural killer cell receptor 2B4	1.0128	1.059934	0.9752673
51747	LUC7L3	LUC7-like 3 (S. cerevisiae)	0.97635	0.956302	1.0190684
51750	RTEL1	regulator of telomere elongation helicase 1	0.97791	0.981124	1.0453304
51751	HIGD1B	HIG1 hypoxia inducible domain family, member 1B	0.98322	1.035635	1.0369822
51752	ERAP1	endoplasmic reticulum aminopeptidase 1	0.98363	1.010911	1.065747
51754	TMEM8B	transmembrane protein 8B	1.00907	0.978123	0.9927824
51755	CDK12	cyclin-dependent kinase 12	0.932	1.022276	0.9741459
51759	C9orf78	chromosome 9 open reading frame 78	0.93404	0.900301	0.9808001
51760	SYT17	synaptotagmin XVII	0.99391	0.988892	1.0152545
51761	ATP8A2	ATPase, aminophospholipid transporter, class I, type 8A, member 2	0.99767	1.011765	0.9910823
51762	RAB8B	RAB8B, member RAS oncogene family	0.96373	1.049065	1.0102161
51763	INPP5K	inositol polyphosphate-5-phosphatase K	0.95338	0.958012	0.945834
51764	GNG13	guanine nucleotide binding protein (G protein), gamma 13	0.98472	1.037872	0.9876634
51765	MST4	serine/threonine protein kinase MST4	0.9593	1.041777	1.1205348
51768	TM7SF3	transmembrane 7 superfamily member 3	0.93696	0.917102	1.0135663
51773	RSF1	remodeling and spacing factor 1	0.9425	0.99227	1.0221794
51776	ZAK	sterile alpha motif and leucine zipper containing kinase AZK	1.02819	0.985073	1.0566849
51780	KDM3B	lysine (K)-specific demethylase 3B	1.01337	1.038002	0.974426
51802	ACCN5	amiloride-sensitive cation channel 5, intestinal	1.00976	1.003953	1.0134859
51804	SIX4	SIX homeobox 4	1.01265	0.990822	1.0981267
51805	COQ3	coenzyme Q3 homolog, methyltransferase (S. cerevisiae)	0.99037	1.0015	1.0162162
51806	CALML5	calmodulin-like 5	0.99874	1.033099	1.0001943
51808	PHAX	phosphorylated adaptor for RNA export	0.96179	0.889757	1.1262355

51809	GALNT7	UDP-N-acetyl-alpha-D-galactosamine:polypeptide N-acetylgalactosaminyltransferase 7 (GalNAc-T7)	0.98523	0.969233	0.9889105
51816	CECR1	cat eye syndrome chromosome region, candidate 1	0.978	0.892744	0.9927349
53335	BCL11A	B-cell CLL/lymphoma 11A (zinc finger protein)	1.00869	1.041154	0.9275468
53338	CAPZA1P	capping protein (actin filament) muscle Z-line, alpha 1 pseudogene	0.97486	0.957204	1.0130222
53339	BTBD1	BTB (POZ) domain containing 1	0.95568	1.040683	1.1492043
53340	SPA17	sperm autoantigenic protein 17	0.99357	0.979313	1.0465002
53342	IL17D	interleukin 17D	0.99174	1.011783	1.0706342
53343	NUDT9	nudix (nucleoside diphosphate linked moiety X)-type motif 9	0.97816	0.950995	1.0048727
53344	CHIC1	cysteine-rich hydrophobic domain 1	0.93918	0.943147	1.0356998
53345	TM6SF2	transmembrane 6 superfamily member 2	1.0168	0.99204	1.0292933
53346	TM6SF1	transmembrane 6 superfamily member 1	0.94832	0.915323	1.0859921
53347	UBASH3A	ubiquitin associated and SH3 domain containing A	0.95209	1.090579	0.9367835
53349	ZFYVE1	zinc finger, FYVE domain containing 1	0.96362	1.023954	1.0680915
53353	LRP1B	low density lipoprotein receptor-related protein 1B	1.01091	1.004893	1.0198522
53354	PANK1	pantothenate kinase 1	0.98837	0.993944	1.0229726
53358	SHC3	SHC (Src homology 2 domain containing) transforming protein 3	0.99292	0.986678	1.0017502
53371	NUP54	nucleoporin 54kDa	0.99393	0.882435	0.9326543
53373	TPCN1	two pore segment channel 1	0.95817	1.006743	1.0306875
53405	CLIC5	chloride intracellular channel 5	1.0134	0.998818	0.9931262
53407	STX18	syntaxin 18	1.00686	0.996946	0.9701091
53409	ATP5LP3	ATP synthase, H+ transporting, mitochondrial Fo complex, subunit g, pseudogene 3	0.98886	0.917048	1.0740551
53411	ATP5LP1	ATP synthase, H+ transporting, mitochondrial Fo complex, subunit g, pseudogene 1	0.99503	0.915121	0.9879858
53615	MBD3	methyl-CpG binding domain protein 3	0.96789	1.003755	1.0883345
53616	ADAM22	ADAM metallopeptidase domain 22	1.00964	0.973668	0.9977773
53630	BCMO1	beta-carotene 15,15'-monooxygenase 1	1.00722	0.997415	1.0007637
53632	PRKAG3	protein kinase, AMP-activated, gamma 3 non-catalytic subunit	1.0264	1.029761	1.0020468
53635	PTOV1	prostate tumor overexpressed 1	0.99263	1.010674	1.035671
53637	S1PR5	sphingosine-1-phosphate receptor 5	1.00177	0.92531	0.8955302
53820	DSCR6	Down syndrome critical region gene 6	0.97179	0.992811	1.0198239
53822	FXYD7	FXYD domain containing ion transport regulator 7	0.97946	1.004726	1.0212325
53827	FXYD5	FXYD domain containing ion transport regulator 5	0.9633	1.029058	0.9901341
53828	FXYD4	FXYD domain containing ion transport regulator 4	1.01469	0.989754	1.0231889
53829	P2RY13	purinergic receptor P2Y, G-protein coupled, 13	1.00246	0.916443	1.0016689

53831	GPR84	G protein-coupled receptor 84	1.02492	1.049504	0.9059308
53832	IL20RA	interleukin 20 receptor, alpha	1.009	1.012775	1.0019807
53833	IL20RB	interleukin 20 receptor beta	1.01083	1.013289	1.0083556
53834	FGFRL1	fibroblast growth factor receptor-like 1	0.99331	1.04059	1.0138378
53836	GPR87	G protein-coupled receptor 87	1.00856	0.977741	1.0052223
53838	C11orf24	chromosome 11 open reading frame 24	0.94049	0.988977	0.9917327
53841	CDHR5	cadherin-related family member 5	0.99946	0.991197	1.0425512
53842	CLDN22	claudin 22	0.9971	0.985812	0.9877309
53904	MYO3A	myosin IIIA	0.98965	1.005886	1.0077147
53905	DUOX1	dual oxidase 1	0.98671	1.000649	1.0150488
53916	RAB4B	RAB4B, member RAS oncogene family	0.988	0.925361	1.0478624
53917	RAB24	RAB24, member RAS oncogene family	1.03062	0.992749	1.0817096
53919	SLCO1C1	solute carrier organic anion transporter family, member 1C1	1.00136	1.010346	0.9841727
53938	PPIL3	peptidylprolyl isomerase (cyclophilin)-like 3	1.01575	1.031905	0.9434247
53940	FTHL17	ferritin, heavy polypeptide-like 17	0.96267	1.009838	0.9861966
53944	CSNK1G1	casein kinase 1, gamma 1	0.94271	0.952749	1.0049326
53947	A4GALT	alpha 1,4-galactosyltransferase	1.00503	1.060397	0.9677974
53981	CPSF2	cleavage and polyadenylation specific factor 2, 100kDa	0.93706	1.001857	1.0137601
54014	BRWD1	bromodomain and WD repeat domain containing 1	0.93112	0.936353	0.9784747
54015	VDAC2P1	voltage-dependent anion channel 2 pseudogene 1	0.95684	1.018721	0.9614426
54016	TUBAP	tubulin, alpha pseudogene	0.99929	0.968454	0.9977673
54019	SLC6A6P1	solute carrier family 6, member 6 pseudogene 1	0.98583	0.950933	0.9834358
54020	SLC37A1	solute carrier family 37 (glycerol-3-phosphate transporter), member 1	0.95608	1.045889	0.9692666
54026	RPL34P3	ribosomal protein L34 pseudogene 3	0.97729	0.986445	1.0157938
54028	RPL23P2	ribosomal protein L23 pseudogene 2	0.94466	1.000688	1.0633645
54031	RIMKLBP1	ribosomal modification protein rimK-like family member B pseudogene 1	1.02833	0.993225	0.9988176
54033	RBM11	RNA binding motif protein 11	0.95028	0.944462	0.9184513
54035	PSMD4P1	proteasome (prosome, macropain) 26S subunit, non-ATPase, 4 pseudogene 1	0.95913	1.092765	1.0132235
54036	PPP1R2P2	protein phosphatase 1, regulatory (inhibitor) subunit 2 pseudogene 2	0.99834	0.941567	1.033686
54038	POLR2CP	polymerase (RNA) II (DNA directed) polypeptide C, pseudogene	0.9781	0.983013	1.0193027
54039	PCBP3	poly(rC) binding protein 3	0.99713	0.995278	1.0013785
54040	PCBP2P1	poly(rC) binding protein 2 pseudogene 1	0.97251	0.967598	0.9772337
54047	HSPD1P7	heat shock 60kDa protein 1 (chaperonin) pseudogene 7	0.9921	1.00062	0.9940969

54049	H2AFZP1	H2A histone family, member Z pseudogene 1	1.00641	1.018815	0.9430142
54052	EIF4A1P1	eukaryotic translation initiation factor 4A1 pseudogene 1	1.00963	1.021416	1.0107641
54053	EIF3FP1	eukaryotic translation initiation factor 3, subunit F pseudogene 1	0.99747	0.986319	1.0364006
54054	EEF1A1P1	eukaryotic translation elongation factor 1 alpha 1 pseudogene	1.01056	0.992878	1.1135123
54058	C21orf58	chromosome 21 open reading frame 58	0.98725	1.013837	1.0373509
54059	YBEY	ybeY metallopeptidase (putative)	0.99791	1.000608	1.0334728
54065	FAM165B	family with sequence similarity 165, member B	0.98691	1.002424	0.978556
54067	C21orf49	chromosome 21 open reading frame 49	0.99228	0.97964	1.0138717
54069	MIS18A	MIS18 kinetochore protein homolog A (S. pombe)	0.95338	1.000656	1.0219217
54073	[No Symbo	[No Name]	0.99838	0.994028	0.9922193
54084	TSPEAR	thrombospondin-type laminin G domain and EAR repeats	1.0046	1.000472	0.996509
54093	SETD4	SET domain containing 4	0.95558	0.950985	1.008678
54097	FAM3B	family with sequence similarity 3, member B	1.00415	1.009826	0.9878645
54098	C1QBPP	complement component 1, q subcomponent binding protein, pseudogene	1.01335	1.028048	1.0206326
54099	FBXW11P1	F-box and WD repeat domain containing 11 pseudogene 1	1.00662	0.908208	1.091722
54101	RIPK4	receptor-interacting serine-threonine kinase 4	0.99223	0.982285	1.0080136
54102	CLIC6	chloride intracellular channel 6	1.00838	1.00946	0.9947366
54103	PION	pigeon homolog (Drosophila)	1.02655	0.984018	0.9659091
54106	TLR9	toll-like receptor 9	1.01878	1.037781	1.0123436
54107	POLE3	polymerase (DNA directed), epsilon 3 (p17 subunit)	0.93962	1.019165	1.0414666
54108	CHRAC1	chromatin accessibility complex 1	0.96946	1.031541	1.158665
54112	GPR88	G protein-coupled receptor 88	0.99732	0.966431	1.0659806
54148	MRPL39	mitochondrial ribosomal protein L39	0.94239	0.965068	1.0446429
54149	C21orf91	chromosome 21 open reading frame 91	0.93643	0.949683	1.0575281
54165	DCUN1D1	DCN1, defective in cullin neddylation 1, domain containing 1 (S. cerevisiae)	1.01602	0.973761	0.9877278
54187	NANS	N-acetylneuraminic acid synthase	0.94516	1.001329	1.0769479
54205	CYCS	cytochrome c, somatic	0.99441	0.936422	0.931706
54206	ERRFI1	ERBB receptor feedback inhibitor 1	0.99197	1.021251	0.9256435
54207	KCNK10	potassium channel, subfamily K, member 10	1.01185	0.991628	1.0173279
54209	TREM2	triggering receptor expressed on myeloid cells 2	1.01781	1.000505	0.9890554
54210	TREM1	triggering receptor expressed on myeloid cells 1	1.00587	0.974233	0.9650889
54212	SNTG1	syntrophin, gamma 1	1.00895	1.002069	1.0169719
54221	SNTG2	syntrophin, gamma 2	0.99963	1.011853	1.0230324

54328	GPR173	G protein-coupled receptor 173	0.99014	1.000091	0.9904616
54329	GPR85	G protein-coupled receptor 85	1.00346	1.020189	1.0085934
54331	GNG2	guanine nucleotide binding protein (G protein), gamma 2	1.0082	1.037585	0.9577759
54332	GDAP1	ganglioside-induced differentiation-associated protein 1	1.00092	0.978311	0.9927896
54344	DPM3	dolichyl-phosphate mannosyltransferase polypeptide 3	1.00385	1.046925	1.100957
54345	SOX18	SRY (sex determining region Y)-box 18	0.98812	1.09714	1.025454
54346	UNC93A	unc-93 homolog A (C. elegans)	0.99906	1.003718	1.0269268
54360	CYTL1	cytokine-like 1	1.01952	0.965474	0.9698427
54361	WNT4	wingless-type MMTV integration site family, member 4	1.0092	0.977331	1.0229853
54363	HAO1	hydroxyacid oxidase (glycolate oxidase) 1	0.99939	1.002996	1.0366856
54386	TERF2IP	telomeric repeat binding factor 2, interacting protein	0.96686	0.978599	0.9628453
54407	SLC38A2	solute carrier family 38, member 2	0.96351	0.979294	1.1123492
54413	NLGN3	neuroligin 3	0.98804	1.026589	0.9576758
54414	SIAE	sialic acid acetylesterase	0.92389	0.89239	0.9820244
54429	TAS2R5	taste receptor, type 2, member 5	1.00058	0.966049	1.0546928
54431	DNAJC10	DnaJ (Hsp40) homolog, subfamily C, member 10	1.00367	1.012244	1.1094299
54432	YIPF1	Yip1 domain family, member 1	0.97201	0.961058	1.0448158
54433	GAR1	GAR1 ribonucleoprotein homolog (yeast)	0.99745	0.996585	1.0041487
54434	SSH1	slingshot homolog 1 (Drosophila)	0.96624	1.005831	0.9940125
54436	SH3TC1	SH3 domain and tetratricopeptide repeats 1	1.03817	0.945204	0.9244368
54437	SEMA5B	sema domain, seven thrombospondin repeats (type 1 and type 1-like), transmembrane domain (TM) and sho	1.01153	1.004607	1.0238967
54438	GFOD1	glucose-fructose oxidoreductase domain containing 1	0.99933	0.960883	1.0185931
54439	RBM27	RNA binding motif protein 27	0.96975	0.970403	1.0147974
54440	SASH3	SAM and SH3 domain containing 3	0.962	0.986822	1.0197014
54442	KCTD5	potassium channel tetramerisation domain containing 5	0.98507	0.966861	1.0882341
54443	ANLN	anillin, actin binding protein	1.00447	0.994364	1.0194007
54453	RIN2	Ras and Rab interactor 2	1.00559	1.05124	0.9675339
54454	ATAD2B	ATPase family, AAA domain containing 2B	0.98563	0.997917	0.94913
54455	FBXO42	F-box protein 42	0.99678	1.059006	1.0439268
54456	MOV10L1	Mov10l1, Moloney leukemia virus 10-like 1, homolog (mouse)	0.98921	1.009376	1.0135466
54457	TAF7L	TAF7-like RNA polymerase II, TATA box binding protein (TBP)-associated factor, 50kDa	0.99326	0.990062	1.0022753
54458	PRR13	proline rich 13	0.96218	0.984559	0.9284787
54460	MRPS21	mitochondrial ribosomal protein S21	0.97563	0.976044	1.0343594

54461	FBXW5	F-box and WD repeat domain containing 5	0.98008	0.95447	1.064837
54462	FAM190B	family with sequence similarity 190, member B	0.94753	0.995401	0.9755038
54463	FAM134B	family with sequence similarity 134, member B	0.97615	1.006835	0.9215897
54464	XRN1	5'-3' exoribonuclease 1	0.99605	1.001961	1.0142159
54465	ETAA1	Ewing tumor-associated antigen 1	0.9639	0.904179	1.0090505
54466	SPIN2A	spindlin family, member 2A	1.01512	0.979974	1.0232228
54467	ANKIB1	ankyrin repeat and IBR domain containing 1	0.98991	0.978255	0.9798469
54468	MIOS	missing oocyte, meiosis regulator, homolog (Drosophila)	1.00099	0.946251	0.9823352
54469	ZFAND6	zinc finger, AN1-type domain 6	0.96089	0.943405	0.9978901
54470	ARMCX6	armadillo repeat containing, X-linked 6	0.92036	0.956501	1.0347311
54471	SMCR7L	Smith-Magenis syndrome chromosome region, candidate 7-like	0.93048	0.961024	1.0922576
54472	TOLLIP	toll interacting protein	0.95107	0.939645	0.9840192
54474	KRT20	keratin 20	0.99422	0.977139	1.009171
54475	NLE1	notchless homolog 1 (Drosophila)	0.94575	0.971303	1.0157382
54476	RNF216	ring finger protein 216	0.96555	0.967937	0.9669691
54477	PLEKHA5	pleckstrin homology domain containing, family A member 5	0.98054	0.999058	0.9792209
54478	FAM64A	family with sequence similarity 64, member A	0.99804	0.972876	1.0213038
54480	CHPF2	chondroitin polymerizing factor 2	0.98591	1.019091	1.050437
54482	CCDC76	coiled-coil domain containing 76	0.96675	0.981913	1.0205602
54487	DGCR8	DiGeorge syndrome critical region gene 8	0.95339	0.937272	0.980191
54490	UGT2B28	UDP glucuronosyltransferase 2 family, polypeptide B28	1.00865	1.04217	1.0139411
54491	FAM105A	family with sequence similarity 105, member A	1.0253	0.870476	0.9674259
54492	NEURL1B	neuralized homolog 1B (Drosophila)	1.00173	1.003675	1.0573204
54494	C11orf71	chromosome 11 open reading frame 71	0.92512	0.915676	0.913904
54495	TMX3	thioredoxin-related transmembrane protein 3	0.92762	0.988228	1.1930727
54496	PRMT7	protein arginine methyltransferase 7	0.946	0.94057	0.9686854
54497	HEATR5B	HEAT repeat containing 5B	1.00065	0.960441	0.9910996
54498	SMOX	spermine oxidase	0.94355	1.063833	0.9321064
54499	TMCO1	transmembrane and coiled-coil domains 1	0.98414	0.93281	1.0565292
54502	RBM47	RNA binding motif protein 47	1.01237	1.012975	0.9622634
54503	ZDHHC13	zinc finger, DHHC-type containing 13	0.95931	0.98811	0.9965082
54504	CPVL	carboxypeptidase, vitellogenic-like	1.02031	0.967799	1.0346406
54505	DHX29	DEAH (Asp-Glu-Ala-His) box polypeptide 29	0.97846	0.949951	1.0474938

54507	ADAMTSL4	ADAMTS-like 4	1.02351	1.005606	1.0553479
54508	FLJ11235	hypothetical FLJ11235	0.99974	0.997783	1.0789164
54509	RHOF	ras homolog gene family, member F (in filopodia)	0.92997	1.029823	0.9732327
54510	PCDH18	protocadherin 18	1.02121	0.973576	1.0221153
54511	HMGCLL1	3-hydroxymethyl-3-methylglutaryl-CoA lyase-like 1	1.01178	0.995374	0.9806649
54512	EXOSC4	exosome component 4	0.98492	1.048746	1.0468033
54514	DDX4	DEAD (Asp-Glu-Ala-Asp) box polypeptide 4	0.98921	0.965045	1.0146315
54516	MTRF1L	mitochondrial translational release factor 1-like	0.99743	0.959043	0.9404665
54517	PUS7	pseudouridylate synthase 7 homolog (S. cerevisiae)	0.97878	0.967487	0.998904
54518	APBB1IP	amyloid beta (A4) precursor protein-binding, family B, member 1 interacting protein	0.94675	1.053353	1.0163101
54520	CCDC93	coiled-coil domain containing 93	0.98141	1.031931	0.892318
54521	WDR44	WD repeat domain 44	0.92873	1.017698	0.8928415
54522	ANKRD16	ankyrin repeat domain 16	0.97935	0.975174	1.0064712
54529	ASNSD1	asparagine synthetase domain containing 1	0.95851	0.894141	0.9841313
54530	[No Symbo	[No Name]	0.98784	0.98145	0.9772948
54531	MIER2	mesoderm induction early response 1, family member 2	0.97502	0.955496	1.0788722
54532	USP53	ubiquitin specific peptidase 53	0.96384	0.977916	0.9815808
54534	MRPL50	mitochondrial ribosomal protein L50	0.86775	0.923936	1.1124699
54535	CCHCR1	coiled-coil alpha-helical rod protein 1	1.00453	0.979276	1.0267389
54536	EXOC6	exocyst complex component 6	0.96402	0.96923	0.9972211
54537	FAM35A	family with sequence similarity 35, member A	0.98103	0.980109	0.9908357
54538	ROBO4	roundabout homolog 4, magic roundabout (Drosophila)	0.99779	1.00829	1.026285
54539	NDUFB11	NADH dehydrogenase (ubiquinone) 1 beta subcomplex, 11, 17.3kDa	0.91043	0.956535	1.1014449
54540	FAM193B	family with sequence similarity 193, member B	0.97781	0.983909	0.9671618
54541	DDIT4	DNA-damage-inducible transcript 4	0.94366	0.998912	0.9489892
54542	RC3H2	ring finger and CCCH-type domains 2	0.94466	1.053763	0.9844863
54543	TOMM7	translocase of outer mitochondrial membrane 7 homolog (yeast)	0.93802	0.874276	0.8934363
54544	CRCT1	cysteine-rich C-terminal 1	0.98854	0.963535	0.9771259
54545	MTMR12	myotubularin related protein 12	1.00177	0.998828	0.9751303
54546	RNF186	ring finger protein 186	1.02565	0.90466	0.9854307
54549	SDK2	sidekick homolog 2 (chicken)	0.97495	0.984826	0.9626257
54550	NECAB2	N-terminal EF-hand calcium binding protein 2	0.98893	1.00732	1.0250527
54551	MAGEL2	MAGE-like 2	0.9971	1.02038	1.0366564

54552	GNL3L	guanine nucleotide binding protein-like 3 (nucleolar)-like	0.91585	0.983027	1.0281885
54553	DKFZP434I(	hypothetical protein DKFZP434I0714	1.00682	0.977087	1.0665078
54554	WDR5B	WD repeat domain 5B	0.95624	0.977386	0.9687952
54555	DDX49	DEAD (Asp-Glu-Ala-Asp) box polypeptide 49	0.92631	0.926432	1.0429028
54556	ING3	inhibitor of growth family, member 3	0.96678	0.89002	1.0096697
54557	SGTB	small glutamine-rich tetratricopeptide repeat (TPR)-containing, beta	0.99643	0.939311	0.8982796
54558	SPATA6	spermatogenesis associated 6	1.0153	0.960856	0.977605
54566	EPB41L4B	erythrocyte membrane protein band 4.1 like 4B	1.00968	0.993982	1.0175021
54567	DLL4	delta-like 4 (Drosophila)	0.97538	0.977421	1.0385858
54569	UGT2B27P	UDP glucuronosyltransferase 2 family, polypeptide B27 pseudogene	1.01592	1.049651	1.0115914
54570	UGT2B26P	UDP glucuronosyltransferase 2 family, polypeptide B26 pseudogene	1.00022	1.102953	1.0804124
54574	UGT1A11P	UDP glucuronosyltransferase 1 family, polypeptide A11 pseudogene	0.98744	1.014233	1.0448331
54583	EGLN1	egl nine homolog 1 (C. elegans)	0.9783	0.991424	1.0465497
54584	GNB1L	guanine nucleotide binding protein (G protein), beta polypeptide 1-like	0.98086	1.007344	1.0384547
54585	LZTFL1	leucine zipper transcription factor-like 1	1.00776	0.976357	1.003698
54586	C9orf11	chromosome 9 open reading frame 11	0.98123	0.986622	1.0399018
54587	MXRA8	matrix-remodelling associated 8	0.97027	1.000727	0.9915415
54596	L1TD1	LINE-1 type transposase domain containing 1	0.99192	0.958491	1.0205324
54602	NDFIP2	Nedd4 family interacting protein 2	0.98085	1.001327	1.0145587
54606	DDX56	DEAD (Asp-Glu-Ala-Asp) box polypeptide 56	0.981	0.991398	0.9834625
54617	INO80	INO80 homolog (S. cerevisiae)	0.96939	0.960071	1.0727845
54619	CCNJ	cyclin J	0.97176	0.931093	1.0016317
54620	FBXL19	F-box and leucine-rich repeat protein 19	0.98366	0.976324	1.0793558
54621	VSIG10	V-set and immunoglobulin domain containing 10	1.0059	0.988278	1.0041608
54622	ARL15	ADP-ribosylation factor-like 15	1.00847	0.987863	0.9481491
54623	PAF1	Paf1, RNA polymerase II associated factor, homolog (S. cerevisiae)	0.91991	1.029401	1.000448
54625	PARP14	poly (ADP-ribose) polymerase family, member 14	1.02344	0.915974	1.1652826
54626	HES2	hairy and enhancer of split 2 (Drosophila)	0.98627	1.027278	1.1130742
54627	KIAA1383	KIAA1383	1.00329	0.904827	1.0624358
54629	FAM63B	family with sequence similarity 63, member B	0.96555	0.999922	1.0645223
54660	PCDHB18	protocadherin beta 18 pseudogene	0.99047	0.990351	1.114126
54661	PCDHB17	protocadherin beta 17 pseudogene	0.98496	0.978711	0.9942742
54662	TBC1D13	TBC1 domain family, member 13	0.89845	0.965347	1.0164507

54663	WDR74	WD repeat domain 74	0.92724	0.960496	1.0662705
54664	TMEM106E	transmembrane protein 106B	0.94475	0.884358	0.9393502
54665	RSBN1	round spermatid basic protein 1	0.99044	1.021761	0.9574766
54674	LRRN3	leucine rich repeat neuronal 3	0.90555	1.045011	0.9559473
54675	CRLS1	cardiolipin synthase 1	0.95259	1.087704	1.0559144
54676	GTPBP2	GTP binding protein 2	0.97633	1.008462	0.9920401
54677	CROT	carnitine O-octanoyltransferase	0.99638	0.935932	1.009826
54680	ZNHIT6	zinc finger, HIT-type containing 6	0.99831	0.987052	1.0478677
54681	P4HTM	prolyl 4-hydroxylase, transmembrane (endoplasmic reticulum)	0.98422	1.015249	1.0360502
54682	MANSC1	MANSC domain containing 1	0.96452	0.979317	0.9815508
54700	RRN3	RRN3 RNA polymerase I transcription factor homolog (S. cerevisiae)	0.91583	0.998433	0.9990425
54704	PDP1	pyruvate dehyrogenase phosphatase catalytic subunit 1	1.02777	1.046204	1.0255214
54707	GPN2	GPN-loop GTPase 2	0.99307	0.969788	1.0362732
54708	5-Mar	membrane-associated ring finger (C3HC4) 5	0.96315	0.959316	0.9942443
54714	CNGB3	cyclic nucleotide gated channel beta 3	0.9959	1.008422	1.0025203
54715	RBFOX1	RNA binding protein, fox-1 homolog (C. elegans) 1	1.00367	1.001331	1.0140224
54716	SLC6A20	solute carrier family 6 (proline IMINO transporter), member 20	0.99548	0.990102	1.0106314
54718	BTN2A3	butyrophilin, subfamily 2, member A3	1.04046	1.006446	0.9615688
54726	OTUD4	OTU domain containing 4	0.96765	0.941817	0.9076692
54729	NKX1-1	NK1 homeobox 1	0.98352	1.011284	1.0443035
54732	TMED9	transmembrane emp24 protein transport domain containing 9	0.97935	0.969503	1.056198
54733	SLC35F2	solute carrier family 35, member F2	0.95611	1.008529	1.0422548
54734	RAB39	RAB39, member RAS oncogene family	0.98205	0.923943	1.0292664
54737	MPHOSPH8	M-phase phosphoprotein 8	0.92407	0.945937	0.9059829
54738	FEV	FEV (ETS oncogene family)	0.9997	1.036525	1.0286336
54739	XAF1	XIAP associated factor 1	0.96681	0.856396	1.1436505
54742	LY6K	lymphocyte antigen 6 complex, locus K	1.02165	1.018134	1.0201071
54749	EPDR1	ependymin related protein 1 (zebrafish)	0.98911	1.014024	1.0211625
54751	FBLIM1	filamin binding LIM protein 1	1.01236	1.026174	1.0259865
54752	FNDC8	fibronectin type III domain containing 8	0.98306	0.994079	1.037625
54753	ZNF853	zinc finger protein 853	0.98489	1.01912	1.0124645
54756	IL17RD	interleukin 17 receptor D	1.00568	0.995262	1.0216509
54757	FAM20A	family with sequence similarity 20, member A	1.01194	0.997995	0.9913036

54758	KLHDC4	kelch domain containing 4	0.96951	1.029082	1.0207884
54760	PCSK4	proprotein convertase subtilisin/kexin type 4	0.99963	0.994346	1.0472922
54762	GRAMD1C	GRAM domain containing 1C	1.00214	0.998791	0.9889639
54763	ROPN1	rhophilin associated tail protein 1	1.00626	0.965359	0.9797483
54764	ZRANB1	zinc finger, RAN-binding domain containing 1	0.96011	0.974656	0.9361562
54765	TRIM44	tripartite motif containing 44	0.96212	0.954271	1.0231639
54766	BTG4	B-cell translocation gene 4	0.99218	1.029355	1.0186253
54768	HYDIN	hydrocephalus inducing homolog (mouse)	0.99758	1.010821	0.9948591
54769	DIRAS2	DIRAS family, GTP-binding RAS-like 2	0.98448	0.978608	0.9761637
54776	PPP1R12C	protein phosphatase 1, regulatory (inhibitor) subunit 12C	0.96676	1.072581	1.0284283
54777	C10orf92	chromosome 10 open reading frame 92	0.98621	0.969591	1.0475607
54778	RNF111	ring finger protein 111	0.95061	1.022826	1.0150625
54780	NSMCE4A	non-SMC element 4 homolog A (S. cerevisiae)	0.92743	0.992711	1.0107228
54784	ALKBH4	alkB, alkylation repair homolog 4 (E. coli)	0.94958	0.989817	1.0533998
54785	C17orf59	chromosome 17 open reading frame 59	0.95671	0.909163	1.0621242
54788	DNAJB12	DnaJ (Hsp40) homolog, subfamily B, member 12	0.99548	0.980389	0.9888655
54790	TET2	tet oncogene family member 2	1.0348	0.999653	1.0155274
54793	KCTD9	potassium channel tetramerisation domain containing 9	0.99681	1.017221	1.0773039
54795	TRPM4	transient receptor potential cation channel, subfamily M, member 4	0.99625	0.97871	0.9904159
54796	BNC2	basonuclin 2	0.99433	1.004395	0.9820492
54797	MED18	mediator complex subunit 18	0.97098	0.990718	1.0159733
54798	DCHS2	dachsous 2 (Drosophila)	1.00278	1.015309	1.013042
54799	MBTD1	mbt domain containing 1	0.96844	0.99406	1.0145081
54800	KLHL24	kelch-like 24 (Drosophila)	0.98715	0.94897	0.9350763
54801	HAUS6	HAUS augmin-like complex, subunit 6	0.92613	0.980879	1.0059034
54802	TRIT1	tRNA isopentenyltransferase 1	0.98615	0.881795	1.0367727
54805	CNNM2	cyclin M2	0.98819	0.977437	1.006554
54806	AHI1	Abelson helper integration site 1	0.99395	0.982458	0.9844252
54807	ZNF586	zinc finger protein 586	0.94263	0.996779	0.9650732
54808	DYM	dymeclin	0.97604	1.007811	0.9397125
54809	SAMD9	sterile alpha motif domain containing 9	0.97782	0.821315	0.9716464
54810	GIPC2	GIPC PDZ domain containing family, member 2	1.00258	1.040237	1.0094044
54811	ZNF562	zinc finger protein 562	0.96837	1.044594	1.0280677

54812	AFTPH	aftiphilin	0.98961	0.954402	1.0244215
54813	KLHL28	kelch-like 28 (Drosophila)	0.94976	0.959083	1.0122572
54814	QPCTL	glutaminyl-peptide cyclotransferase-like	0.96052	1.084623	1.0412009
54815	GATAD2A	GATA zinc finger domain containing 2A	0.97585	0.990044	0.9581442
54816	ZNF280D	zinc finger protein 280D	0.9439	0.950493	0.9340558
54819	ZCCHC10	zinc finger, CCHC domain containing 10	1.00572	0.961881	1.007538
54820	NDE1	nudE nuclear distribution gene E homolog 1 (A. nidulans)	0.95783	0.966258	0.9879445
54821	ERCC6L	excision repair cross-complementing rodent repair deficiency, complementation group 6-like	0.97463	1.053689	1.0196723
54822	TRPM7	transient receptor potential cation channel, subfamily M, member 7	0.94273	1.008322	1.1151717
54823	SWT1	SWT1 RNA endoribonuclease homolog (S. cerevisiae)	0.96962	0.988276	1.004659
54825	CDHR2	cadherin-related family member 2	0.99746	1.027138	0.9987447
54826	GIN1	gypsy retrotransposon integrase 1	0.94398	1.019251	1.0481101
54827	FAM55D	family with sequence similarity 55, member D	0.99938	1.011624	1.016898
54828	BCAS3	breast carcinoma amplified sequence 3	0.97533	1.010724	0.9802567
54829	ASPN	asporin	0.99659	0.997173	0.9953064
54830	NUP62CL	nucleoporin 62kDa C-terminal like	0.98461	1.004905	1.0120867
54831	BEST2	bestrophin 2	1.00746	0.983566	1.0033772
54832	VPS13C	vacuolar protein sorting 13 homolog C (S. cerevisiae)	0.96483	0.89812	1.0302189
54834	GDAP2	ganglioside induced differentiation associated protein 2	1.03398	1.011844	0.9947927
54836	BSPRY	B-box and SPRY domain containing	0.99174	0.995083	1.0440867
54838	C10orf26	chromosome 10 open reading frame 26	0.98182	1.012181	0.974928
54839	LRRC49	leucine rich repeat containing 49	0.9926	0.992222	1.002567
54840	APTX	aprataxin	0.9863	1.015064	1.0460184
54841	BIVM	basic, immunoglobulin-like variable motif containing	0.95109	0.942568	1.0219171
54842	MFSD6	major facilitator superfamily domain containing 6	0.99316	0.929176	0.9697895
54843	SYTL2	synaptotagmin-like 2	0.98035	1.06402	0.9187602
54845	ESRP1	epithelial splicing regulatory protein 1	1.00901	1.006318	1.0205441
54847	SIDT1	SID1 transmembrane family, member 1	0.98459	0.889123	1.003372
54848	ARHGEF38	Rho guanine nucleotide exchange factor (GEF) 38	1.00728	0.993998	0.9927465
54849	DEF8	differentially expressed in FDCP 8 homolog (mouse)	0.97104	0.972072	1.0557605
54850	FBXL12	F-box and leucine-rich repeat protein 12	0.95651	0.956085	1.1233489
54851	ANKRD49	ankyrin repeat domain 49	0.90414	0.847232	0.9241146
54852	PAQR5	progestin and adipoQ receptor family member V	1.0176	0.979678	1.0187482

54853	WDR55	WD repeat domain 55	0.97903	1.004921	1.0697233
54854	FAM83E	family with sequence similarity 83, member E	0.99922	0.980467	1.0183727
54855	FAM46C	family with sequence similarity 46, member C	1.05542	1.025772	0.9468228
54856	GON4L	gon-4-like (C. elegans)	0.98986	0.987014	1.0322444
54857	GDPD2	glycerophosphodiester phosphodiesterase domain containing 2	0.9945	0.961263	0.9968771
54858	PGPEP1	pyroglutamyl-peptidase I	0.95604	1.016193	0.9662332
54859	C3orf75	chromosome 3 open reading frame 75	0.97824	0.963058	1.0204973
54860	MS4A12	membrane-spanning 4-domains, subfamily A, member 12	0.99593	1.037422	1.0110643
54861	SNRK	SNF related kinase	0.99856	0.930789	0.9769808
54862	CC2D1A	coiled-coil and C2 domain containing 1A	0.98298	0.902236	1.0547968
54863	C9orf167	chromosome 9 open reading frame 167	0.97626	0.895901	1.0549852
54865	GPATCH4	G patch domain containing 4	0.99601	0.926598	1.0158692
54866	PPP1R14D	protein phosphatase 1, regulatory (inhibitor) subunit 14D	0.99769	1.004218	0.9617072
54867	TMEM214	transmembrane protein 214	0.99624	0.97374	1.0797888
54868	TMEM104	transmembrane protein 104	0.98113	0.987927	0.9482086
54869	EPS8L1	EPS8-like 1	0.99722	1.019722	1.0303113
54870	QRICH1	glutamine-rich 1	0.9858	0.950615	0.9939117
54872	PIGG	phosphatidylinositol glycan anchor biosynthesis, class G	0.99097	0.951847	1.0057567
54873	PALMD	palmdelphin	1.0111	1.005381	1.0205492
54874	FNBP1L	formin binding protein 1-like	0.98296	0.967304	1.008114
54875	CNTLN	centlein, centrosomal protein	0.97936	0.936503	1.0191929
54876	DCAF16	DDB1 and CUL4 associated factor 16	1.02558	0.921987	0.9057541
54877	ZCCHC2	zinc finger, CCHC domain containing 2	0.95315	0.98769	0.9713313
54878	DPP8	dipeptidyl-peptidase 8	0.96812	1.011585	1.0512821
54879	ST7L	suppression of tumorigenicity 7 like	0.98631	1.004829	0.9334907
54880	BCOR	BCL6 corepressor	0.97699	1.013445	0.9980345
54881	TEX10	testis expressed 10	0.95195	1.051415	1.090746
54883	CWC25	CWC25 spliceosome-associated protein homolog (S. cerevisiae)	0.92174	1.048788	1.0454357
54884	RETSAT	retinol saturase (all-trans-retinol 13,14-reductase)	0.9622	1.021425	1.1149207
54885	TBC1D8B	TBC1 domain family, member 8B (with GRAM domain)	0.98271	0.989606	0.9943465
54886	LPPR1	lipid phosphate phosphatase-related protein type 1	0.99568	0.987414	1.0162362
54887	UHRF1BP1	UHRF1 binding protein 1	1.00939	0.859381	1.0529758
54888	NSUN2	NOP2/Sun domain family, member 2	1.0136	0.963494	1.0647835

54890	ALKBH5	alkB, alkylation repair homolog 5 (E. coli)	0.94145	1.005343	0.9572261
54891	INO80D	INO80 complex subunit D	0.98938	0.938746	0.9218952
54892	NCAPG2	non-SMC condensin II complex, subunit G2	0.95906	0.982582	1.0383137
54893	MTMR10	myotubularin related protein 10	0.9682	0.872268	1.0416244
54894	RNF43	ring finger protein 43	0.99711	1.011717	1.0044954
54896	PQLC2	PQ loop repeat containing 2	0.9801	1.024161	1.0175289
54897	CASZ1	castor zinc finger 1	1.0044	0.993347	1.0031749
54898	ELOVL2	ELOVL fatty acid elongase 2	0.99712	1.005864	1.0298311
54899	PXK	PX domain containing serine/threonine kinase	1.03516	0.975836	1.0023852
54900	LAX1	lymphocyte transmembrane adaptor 1	0.93313	0.940793	0.9135454
54901	CDKAL1	CDK5 regulatory subunit associated protein 1-like 1	0.99119	0.889082	0.9704675
54902	TTC19	tetratricopeptide repeat domain 19	0.95821	0.960114	0.946855
54903	MKS1	Meckel syndrome, type 1	0.9387	0.973769	1.0153504
54904	WHSC1L1	Wolf-Hirschhorn syndrome candidate 1-like 1	0.94484	0.982305	0.9891995
54905	CYP2W1	cytochrome P450, family 2, subfamily W, polypeptide 1	1.00046	1.023388	1.0223701
54906	C10orf18	chromosome 10 open reading frame 18	0.92423	0.977343	1.1174568
54908	CCDC99	coiled-coil domain containing 99	0.98672	0.996136	1.0366635
54910	SEMA4C	sema domain, immunoglobulin domain (Ig), transmembrane domain (TM) and short cytoplasmic domain, (se	0.97886	1.014026	1.0131882
54913	RPP25	ribonuclease P/MRP 25kDa subunit	0.96158	1.019642	1.0894709
54914	KIAA1797	KIAA1797	0.90582	0.938011	1.0200873
54915	YTHDF1	YTH domain family, member 1	0.92071	0.981345	1.0028721
54916	C14orf101	chromosome 14 open reading frame 101	0.9412	0.90788	1.0253249
54918	CMTM6	CKLF-like MARVEL transmembrane domain containing 6	1.03032	0.888418	0.9558366
54919	HEATR2	HEAT repeat containing 2	1.00108	0.96158	1.0293694
54920	DUS2L	dihydrouridine synthase 2-like, SMM1 homolog (S. cerevisiae)	0.94397	1.055405	1.040098
54921	CHTF8	CTF8, chromosome transmission fidelity factor 8 homolog (S. cerevisiae)	0.96452	0.957272	0.9980349
54922	RASIP1	Ras interacting protein 1	1.00295	0.988621	1.0085072
54923	LIME1	Lck interacting transmembrane adaptor 1	0.95765	1.024992	1.1318959
54925	ZNF434	zinc finger protein 434	0.93635	0.982325	1.0090943
54926	UBE2R2	ubiquitin-conjugating enzyme E2R 2	0.98149	1.073025	0.8960201
54927	CHCHD3	coiled-coil-helix-coiled-coil-helix domain containing 3	0.98749	1.022307	0.9929008
54928	IMPAD1	inositol monophosphatase domain containing 1	0.94848	1.036346	1.1382468
54929	TMEM161A	transmembrane protein 161A	0.9814	1.02862	1.0777947

54930	HAUS4	HAUS augmin-like complex, subunit 4	0.95915	0.981874	1.0546676
54931	RG9MTD1	RNA (guanine-9-) methyltransferase domain containing 1	0.9578	0.967996	0.9971536
54932	EXD3	exonuclease 3'-5' domain containing 3	1.00071	1.003384	1.0232497
54933	RHBDL2	rhomboid, veinlet-like 2 (Drosophila)	1.00027	0.977191	1.0044365
54934	C12orf41	chromosome 12 open reading frame 41	0.93331	0.884681	1.0908949
54935	DUSP23	dual specificity phosphatase 23	1.00875	1.00847	0.9733603
54936	ADPRHL2	ADP-ribosylhydrolase like 2	0.99535	0.951179	1.0253091
54937	SOHLH2	spermatogenesis and oogenesis specific basic helix-loop-helix 2	0.99727	0.993117	0.976826
54938	SARS2	seryl-tRNA synthetase 2, mitochondrial	0.96867	0.934428	1.0397226
54939	COMMD4	COMM domain containing 4	0.96353	0.985545	1.0734914
54940	OCIAD1	OCIA domain containing 1	1.00799	0.907174	0.9956426
54941	RNF125	ring finger protein 125	0.929	1.064057	1.0460111
54942	C9orf6	chromosome 9 open reading frame 6	0.97998	0.944998	0.9796421
54943	DNAJC28	DnaJ (Hsp40) homolog, subfamily C, member 28	0.98248	1.0224	1.0400813
54944	FLJ20464	hypothetical protein FLJ20464	0.96018	0.941643	1.0429355
54946	SLC41A3	solute carrier family 41, member 3	0.97242	0.968096	1.0037634
54947	LPCAT2	lysophosphatidylcholine acyltransferase 2	1.01659	1.034422	1.0413629
54948	MRPL16	mitochondrial ribosomal protein L16	0.93063	1.006363	1.0957343
54949	SDHAF2	succinate dehydrogenase complex assembly factor 2	0.93292	0.942442	1.0548717
54951	COMMD8	COMM domain containing 8	1.00468	0.927927	1.0300535
54952	TRNAU1AP	tRNA selenocysteine 1 associated protein 1	1.00014	1.002883	1.0500208
54953	C1orf27	chromosome 1 open reading frame 27	0.97147	0.876348	1.0088734
54954	FAM120C	family with sequence similarity 120C	1.0003	1.008166	1.0211324
54955	C1orf109	chromosome 1 open reading frame 109	0.97673	0.96515	1.0485567
54956	PARP16	poly (ADP-ribose) polymerase family, member 16	0.92606	0.957291	1.0401977
54957	TXNL4B	thioredoxin-like 4B	0.904	0.925114	1.0078873
54958	TMEM160	transmembrane protein 160	0.97753	1.058795	1.1237658
54959	ODAM	odontogenic, ameloblast asssociated	1.00094	1.002437	1.009214
54960	GEMIN8	gem (nuclear organelle) associated protein 8	0.97976	1.025123	1.0255063
54961	SSH3	slingshot homolog 3 (Drosophila)	0.98581	1.017145	1.0307361
54962	TIPIN	TIMELESS interacting protein	0.97737	0.968991	1.004393
54963	UCKL1	uridine-cytidine kinase 1-like 1	0.98008	1.041358	1.03667
54964	C1orf56	chromosome 1 open reading frame 56	1.00679	1.039303	0.9622626

54965	PIGX	phosphatidylinositol glycan anchor biosynthesis, class X	0.97542	1.010483	1.0177523
54967	CXorf48	chromosome X open reading frame 48	0.98567	0.996905	0.9580044
54968	TMEM70	transmembrane protein 70	0.95112	1.056225	1.1224442
54969	C4orf27	chromosome 4 open reading frame 27	0.98623	1.008299	1.0121935
54970	TTC12	tetratricopeptide repeat domain 12	0.99023	0.951985	0.9349543
54971	BANP	BTG3 associated nuclear protein	0.97917	1.042412	0.9844314
54972	TMEM132A	transmembrane protein 132A	0.98921	1.013548	1.0284059
54973	CPSF3L	cleavage and polyadenylation specific factor 3-like	0.98327	0.991558	1.0347382
54974	THG1L	tRNA-histidine guanylyltransferase 1-like (S. cerevisiae)	0.93934	0.979475	1.0298949
54976	C20orf27	chromosome 20 open reading frame 27	1.01086	0.977723	1.0635294
54977	SLC25A38	solute carrier family 25, member 38	0.99256	0.978833	1.0285911
54978	C2orf18	chromosome 2 open reading frame 18	0.99693	0.910383	1.0160467
54979	HRASLS2	HRAS-like suppressor 2	1.01466	0.984483	1.0384114
54980	C2orf42	chromosome 2 open reading frame 42	0.97009	0.952094	1.0172669
54981	C9orf95	chromosome 9 open reading frame 95	0.94551	0.907947	0.9691151
54982	CLN6	ceroid-lipofuscinosis, neuronal 6, late infantile, variant	0.95742	0.924872	1.0727067
54984	PINX1	PIN2/TERF1 interacting, telomerase inhibitor 1	0.95061	0.97332	1.0311884
54985	HCFC1R1	host cell factor C1 regulator 1 (XPO1 dependent)	0.97501	0.966601	1.0566926
54986	ULK4	unc-51-like kinase 4 (C. elegans)	0.98922	0.924261	0.9510698
54987	C1orf123	chromosome 1 open reading frame 123	0.95613	0.936276	1.0682252
54988	ACSM5	acyl-CoA synthetase medium-chain family member 5	1.00856	1.009728	0.9701851
54989	ZNF770	zinc finger protein 770	0.88988	1.030418	1.0559257
54991	C1orf159	chromosome 1 open reading frame 159	1.00531	1.007309	1.0094272
54993	ZSCAN2	zinc finger and SCAN domain containing 2	0.96263	1.003635	1.0343049
54994	C20orf11	chromosome 20 open reading frame 11	0.92043	0.971112	1.0653663
54995	OXSM	3-oxoacyl-ACP synthase, mitochondrial	0.98387	0.962603	1.0210381
54996	MOSC2	MOCO sulphurase C-terminal domain containing 2	0.99345	0.996669	1.0025139
54997	TESC	tescalcin	0.98418	0.971224	1.0372757
54998	AURKAIP1	aurora kinase A interacting protein 1	0.98578	0.973296	1.0927835
55000	TUG1	taurine upregulated 1 (non-protein coding)	0.99259	0.893317	0.969143
55001	TTC22	tetratricopeptide repeat domain 22	1.03078	0.972993	1.0000821
55002	TMCO3	transmembrane and coiled-coil domains 3	0.95924	1.0007	1.0620854
55003	PAK1IP1	PAK1 interacting protein 1	0.95076	0.848148	1.0823219

55004	LAMTOR1	late endosomal/lysosomal adaptor, MAPK and MTOR activator 1	0.90571	0.930424	1.0563746
55005	RMND1	required for meiotic nuclear division 1 homolog (S. cerevisiae)	0.97115	0.998359	1.0496038
55006	TRMT61B	tRNA methyltransferase 61 homolog B (S. cerevisiae)	1.01591	0.930203	1.0135305
55007	FAM118A	family with sequence similarity 118, member A	0.95206	0.976889	0.9024103
55008	HERC6	hect domain and RLD 6	0.96022	0.892857	1.0215389
55009	C19orf24	chromosome 19 open reading frame 24	0.96714	1.100361	1.0631076
55010	C12orf48	chromosome 12 open reading frame 48	0.97053	0.965044	1.0179063
55011	PIH1D1	PIH1 domain containing 1	0.93406	0.995723	1.0849724
55012	PPP2R3C	protein phosphatase 2, regulatory subunit B'', gamma	0.95132	1.001172	1.0244114
55013	CCDC109B	coiled-coil domain containing 109B	0.97885	0.971186	0.977775
55014	STX17	syntaxin 17	0.95788	0.928643	1.0233277
55015	PRPF39	PRP39 pre-mRNA processing factor 39 homolog (S. cerevisiae)	0.92677	0.932378	1.0719785
55016	1-Mar	membrane-associated ring finger (C3HC4) 1	0.98304	1.039594	0.9493283
55017	C14orf119	chromosome 14 open reading frame 119	0.93207	0.965493	0.9853556
55018	C17orf73	chromosome 17 open reading frame 73	0.99242	1.015556	0.9983727
55020	TTC38	tetratricopeptide repeat domain 38	0.95904	1.05822	0.9989173
55022	PID1	phosphotyrosine interaction domain containing 1	1.03161	1.106216	0.9526136
55023	PHIP	pleckstrin homology domain interacting protein	1.00542	0.921877	1.1111111
55025	FLJ20712	hypothetical FLJ20712	1.00584	0.965144	0.9655292
55026	FAM70A	family with sequence similarity 70, member A	1.00298	1.019029	1.016968
55027	HEATR3	HEAT repeat containing 3	0.99496	0.972762	1.01977
55028	C17orf80	chromosome 17 open reading frame 80	0.95036	0.995342	0.9891199
55030	FBXO34	F-box protein 34	0.96316	1.116331	0.9655219
55031	USP47	ubiquitin specific peptidase 47	0.94334	1.042536	0.9457146
55032	SLC35A5	solute carrier family 35, member A5	1.02042	0.924102	1.0482483
55033	FKBP14	FK506 binding protein 14, 22 kDa	1.01832	0.972973	1.0465638
55034	MOCOS	molybdenum cofactor sulfurase	1.00017	1.020627	1.0120921
55035	NOL8	nucleolar protein 8	0.92117	1.035496	1.0992278
55036	CCDC40	coiled-coil domain containing 40	0.99976	1.009222	0.9997717
55037	PTCD3	Pentatricopeptide repeat domain 3	1.01071	0.978154	1.0894776
55038	CDCA4	cell division cycle associated 4	0.95543	0.942093	0.9848792
55039	TRMT12	tRNA methyltransferase 12 homolog (S. cerevisiae)	0.91975	0.960101	1.1173032
55040	EPN3	epsin 3	0.99782	0.982524	1.0370043

55041	PLEKHB2	pleckstrin homology domain containing, family B (evectins) member 2	0.99444	0.973588	1.0173726
55048	VPS37C	vacuolar protein sorting 37 homolog C (S. cerevisiae)	0.99189	0.974092	0.9997819
55049	C19orf60	chromosome 19 open reading frame 60	0.95943	1.016999	1.1589169
55051	C14orf102	chromosome 14 open reading frame 102	0.96226	0.955519	1.0836061
55052	MRPL20	mitochondrial ribosomal protein L20	1.01344	0.937791	1.0576754
55054	ATG16L1	ATG16 autophagy related 16-like 1 (S. cerevisiae)	0.985	1.035509	0.9339853
55055	ZWILCH	Zwilch, kinetochore associated, homolog (Drosophila)	0.9515	0.971699	1.039656
55057	AIM1L	absent in melanoma 1-like	1.01156	0.983813	1.0287986
55061	SUSD4	sushi domain containing 4	0.99921	0.976572	1.0071745
55062	WIPI1	WD repeat domain, phosphoinositide interacting 1	0.96954	1.050229	0.9699939
55063	ZCWPW1	zinc finger, CW type with PWWP domain 1	0.984	1.001037	0.9813215
55064	C9orf68	chromosome 9 open reading frame 68	1.00277	0.959678	0.9799237
55065	GPR172B	G protein-coupled receptor 172B	1.02342	1.019476	0.9535455
55066	PDPR	pyruvate dehydrogenase phosphatase regulatory subunit	0.97054	0.975388	1.1475136
55068	ENOX1	ecto-NOX disulfide-thiol exchanger 1	1.00204	1.005809	1.0221285
55069	C7orf42	chromosome 7 open reading frame 42	1.00458	0.970017	1.0315156
55070	DET1	de-etiolated homolog 1 (Arabidopsis)	0.92117	0.892412	0.9750231
55071	C9orf40	chromosome 9 open reading frame 40	0.94804	1.015565	1.0775292
55072	RNF31	ring finger protein 31	0.96316	0.957624	1.0336664
55073	LRRC37A4	leucine rich repeat containing 37, member A4 (pseudogene)	0.90608	0.87777	0.8002187
55074	OXR1	oxidation resistance 1	0.97084	1.033486	1.0595395
55075	UACA	uveal autoantigen with coiled-coil domains and ankyrin repeats	0.96841	0.987498	0.9940924
55076	TMEM45A	transmembrane protein 45A	1.0075	0.995947	0.99165
55079	FEZF2	FEZ family zinc finger 2	0.99598	1.042865	1.0580679
55080	TAPBPL	TAP binding protein-like	0.96863	0.991673	1.0167492
55081	IFT57	intraflagellar transport 57 homolog (Chlamydomonas)	0.98155	0.931161	0.967081
55082	ARGLU1	arginine and glutamate rich 1	0.9666	0.976651	1.1629315
55083	KIF26B	kinesin family member 26B	1.00898	1.004644	1.0108155
55084	SOBP	sine oculis binding protein homolog (Drosophila)	1.00484	1.010907	1.0054443
55086	CXorf57	chromosome X open reading frame 57	0.95951	1.047447	1.0108134
55088	C10orf118	chromosome 10 open reading frame 118	0.94623	0.977451	0.972393
55090	MED9	mediator complex subunit 9	0.98086	0.917042	1.0172061
55092	TMEM51	transmembrane protein 51	0.9787	0.996309	1.0218313

55093	WDYHV1	WDYHV motif containing 1	0.96512	0.941347	1.038131
55094	GPATCH1	G patch domain containing 1	0.93385	1.002486	1.0161324
55095	SAMD4B	sterile alpha motif domain containing 4B	0.98185	1.059469	0.9270925
55100	WDR70	WD repeat domain 70	0.99235	0.926094	1.0318707
55101	ATP5SL	ATP5S-like	0.94324	0.903145	1.0415006
55102	ATG2B	ATG2 autophagy related 2 homolog B (S. cerevisiae)	0.93435	1.021941	1.1329741
55105	GPATCH2	G patch domain containing 2	0.9936	1.012003	1.026009
55106	SLFN12	schlafen family member 12	0.97525	0.968582	1.0102144
55107	ANO1	anoctamin 1, calcium activated chloride channel	0.98897	1.014636	1.0200923
55108	BSDC1	BSD domain containing 1	0.98883	0.934536	0.9103634
55109	AGGF1	angiogenic factor with G patch and FHA domains 1	0.97272	0.952781	1.0433273
55110	MAGOHB	mago-nashi homolog B (Drosophila)	0.97679	0.956656	0.9953553
55111	PLEKHJ1	pleckstrin homology domain containing, family J member 1	0.95898	0.960414	1.1327678
55112	WDR60	WD repeat domain 60	0.99914	0.983327	0.9803972
55113	XKR8	XK, Kell blood group complex subunit-related family, member 8	1.01852	0.960217	1.0425924
55114	ARHGAP17	Rho GTPase activating protein 17	0.98859	0.999929	1.0926727
55116	TMEM39B	transmembrane protein 39B	0.97606	0.976047	1.0457452
55117	SLC6A15	solute carrier family 6 (neutral amino acid transporter), member 15	1.00009	1.018008	1.0001803
55118	CRTAC1	cartilage acidic protein 1	0.98679	1.008563	1.0197961
55119	PRPF38B	PRP38 pre-mRNA processing factor 38 (yeast) domain containing B	1.04231	0.942926	0.9750715
55120	FANCL	Fanconi anemia, complementation group L	0.97953	1.014323	0.9993801
55122	AKIRIN2	akirin 2	1.02687	0.914292	0.878299
55124	PIWIL2	piwi-like 2 (Drosophila)	1.00993	1.000191	0.9883052
55125	CEP192	centrosomal protein 192kDa	0.93287	0.983794	1.0830389
55127	HEATR1	HEAT repeat containing 1	0.96379	0.977776	1.0708078
55128	TRIM68	tripartite motif containing 68	0.92426	0.929226	0.9909137
55129	ANO10	anoctamin 10	1.01148	0.958406	1.030594
55130	ARMC4	armadillo repeat containing 4	0.98676	1.001135	1.0021251
55131	RBM28	RNA binding motif protein 28	1.01947	0.989871	0.9817396
55132	LARP1B	La ribonucleoprotein domain family, member 1B	0.99293	0.994219	1.0263822
55133	SRBD1	S1 RNA binding domain 1	0.98658	0.953892	1.0097675
55135	WRAP53	WD repeat containing, antisense to TP53	0.95761	0.962771	1.0326282
55137	FIGN	fidgetin	1.01148	1.00739	0.9926728

55138	FAM90A1	family with sequence similarity 90, member A1	0.99447	0.996756	1.0144375
55139	ANKZF1	ankyrin repeat and zinc finger domain containing 1	0.96926	1.007549	1.064088
55140	ELP3	elongation protein 3 homolog (S. cerevisiae)	0.99899	0.92489	0.9421158
55142	HAUS2	HAUS augmin-like complex, subunit 2	0.93256	0.920008	1.0241609
55143	CDCA8	cell division cycle associated 8	0.99762	0.988556	1.0146258
55144	LRRC8D	leucine rich repeat containing 8 family, member D	0.98653	1.047655	1.0135427
55145	THAP1	THAP domain containing, apoptosis associated protein 1	0.91528	0.870583	1.0706326
55146	ZDHHC4	zinc finger, DHHC-type containing 4	1.00875	1.010856	1.0238564
55147	RBM23	RNA binding motif protein 23	0.95701	1.111275	0.999504
55148	UBR7	ubiquitin protein ligase E3 component n-recognin 7 (putative)	0.90199	0.936876	1.0652264
55149	MTPAP	mitochondrial poly(A) polymerase	0.94004	0.87903	1.0800979
55150	C19orf73	chromosome 19 open reading frame 73	0.96399	0.974605	1.0325897
55151	TMEM38B	transmembrane protein 38B	0.89928	0.957714	0.9263347
55152	DALRD3	DALR anticodon binding domain containing 3	0.98596	0.962267	1.0019526
55153	SDAD1	SDA1 domain containing 1	0.9851	0.912161	1.0078579
55154	MSTO1	misato homolog 1 (Drosophila)	1.02786	1.021312	0.9829838
55156	ARMC1	armadillo repeat containing 1	0.92892	0.98466	1.1257836
55157	DARS2	aspartyl-tRNA synthetase 2, mitochondrial	0.98894	0.976249	1.0031723
55159	RFWD3	ring finger and WD repeat domain 3	0.94881	1.018588	1.0681238
55160	ARHGEF10l	Rho guanine nucleotide exchange factor (GEF) 10-like	1.01707	0.988858	1.0002255
55161	TMEM33	transmembrane protein 33	1.00923	1.072341	1.0656232
55163	PNPO	pyridoxamine 5'-phosphate oxidase	0.88766	0.958238	1.0782803
55164	SHQ1	SHQ1 homolog (S. cerevisiae)	0.97412	0.990182	1.0159553
55165	CEP55	centrosomal protein 55kDa	0.99183	0.984551	1.0241565
55166	CENPQ	centromere protein Q	0.98729	1.008659	1.005585
55167	MSL2	male-specific lethal 2 homolog (Drosophila)	0.9969	1.013103	0.9605793
55168	MRPS18A	mitochondrial ribosomal protein S18A	0.99085	0.926058	1.0438478
55170	PRMT6	protein arginine methyltransferase 6	0.95918	0.901591	0.9771252
55171	TBCCD1	TBCC domain containing 1	0.95983	0.950047	1.0578922
55172	C14orf104	chromosome 14 open reading frame 104	0.9726	1.03423	0.9982963
55173	MRPS10	mitochondrial ribosomal protein S10	0.99884	0.909494	1.0057417
55174	INTS10	integrator complex subunit 10	0.98261	1.016461	1.015353
55175	KLHL11	kelch-like 11 (Drosophila)	0.98988	0.846181	1.1484485

55176	SEC61A2	Sec61 alpha 2 subunit (S. cerevisiae)	0.99392	0.968121	1.0143364
55177	FAM82A2	family with sequence similarity 82, member A2	0.97117	1.020538	0.9712774
55178	RNMTL1	RNA methyltransferase like 1	0.91925	0.974368	1.0560552
55179	FAIM	Fas apoptotic inhibitory molecule	0.96821	0.9437	1.0370698
55180	LINS	lines homolog (Drosophila)	0.93495	0.895535	1.0077191
55181	C17orf71	chromosome 17 open reading frame 71	0.92833	0.89458	1.0554402
55182	RNF220	ring finger protein 220	1.01401	0.986577	1.0284279
55183	RIF1	RAP1 interacting factor homolog (yeast)	0.98274	0.996853	1.0752874
55184	C20orf12	chromosome 20 open reading frame 12	0.98758	0.997969	0.9949887
55186	SLC25A36	solute carrier family 25, member 36	1.02139	0.934276	1.0208461
55187	VPS13D	vacuolar protein sorting 13 homolog D (S. cerevisiae)	0.99031	1.022567	0.9604372
55188	RIC8B	resistance to inhibitors of cholinesterase 8 homolog B (C. elegans)	0.96325	0.964636	1.0021745
55190	NUDT11	nudix (nucleoside diphosphate linked moiety X)-type motif 11	0.98515	1.024041	0.9860964
55191	NADSYN1	NAD synthetase 1	0.97391	0.942497	1.074511
55192	DNAJC17	DnaJ (Hsp40) homolog, subfamily C, member 17	0.99133	0.965341	1.0249267
55193	PBRM1	polybromo 1	0.98789	0.984038	1.0128412
55194	FAM176B	family with sequence similarity 176, member B	0.99442	1.050406	1.0811043
55195	C14orf105	chromosome 14 open reading frame 105	0.99901	0.965257	0.995257
55196	C12orf35	chromosome 12 open reading frame 35	0.99026	0.935136	0.9017139
55197	RPRD1A	regulation of nuclear pre-mRNA domain containing 1A	0.95932	1.001667	1.0411425
55198	APPL2	adaptor protein, phosphotyrosine interaction, PH domain and leucine zipper containing 2	0.93077	0.98609	0.9850715
55200	PLEKHG6	pleckstrin homology domain containing, family G (with RhoGef domain) member 6	0.98564	1.043533	1.0160414
55201	MAP1S	microtubule-associated protein 1S	1.00014	1.021713	1.0541174
55203	LGI2	leucine-rich repeat LGI family, member 2	1.00313	0.980248	0.9726724
55204	GOLPH3L	golgi phosphoprotein 3-like	0.98446	1.014501	1.0590084
55205	ZNF532	zinc finger protein 532	1.0052	1.019753	0.9870201
55206	SBNO1	strawberry notch homolog 1 (Drosophila)	0.94529	1.035441	1.1187021
55207	ARL8B	ADP-ribosylation factor-like 8B	1.03015	0.974906	0.9453998
55208	DCUN1D2	DCN1, defective in cullin neddylation 1, domain containing 2 (S. cerevisiae)	0.9727	1.001869	0.94168
55209	SETD5	SET domain containing 5	0.97067	1.032543	1.0278906
55210	ATAD3A	ATPase family, AAA domain containing 3A	0.99969	0.999923	1.0576966
55211	DPPA4	developmental pluripotency associated 4	1.01321	0.996788	1.0222525
55212	BBS7	Bardet-Biedl syndrome 7	1	1.004984	1.0569534

55213	RCBTB1	regulator of chromosome condensation (RCC1) and BTB (POZ) domain containing protein 1	0.97853	0.916969	1.1314501
55214	LEPREL1	leprecan-like 1	1.00368	1.010876	1.0295006
55215	FANCI	Fanconi anemia, complementation group I	0.96536	0.992966	0.9838967
55216	C11orf57	chromosome 11 open reading frame 57	0.91911	1.015732	1.1417125
55217	TMLHE	trimethyllysine hydroxylase, epsilon	0.96382	0.999622	0.994314
55218	EXD2	exonuclease 3'-5' domain containing 2	0.95729	0.987632	1.0105379
55219	TMEM57	transmembrane protein 57	1.00771	1.002017	0.9365055
55220	KLHDC8A	kelch domain containing 8A	1.00758	1.019891	1.0382253
55222	LRRC20	leucine rich repeat containing 20	1.02629	1.006877	1.0143213
55223	TRIM62	tripartite motif containing 62	0.98712	0.9083	0.9858112
55224	ETNK2	ethanolamine kinase 2	1.01399	0.972082	0.9632746
55225	RAVER2	ribonucleoprotein, PTB-binding 2	0.99661	0.902891	0.9752665
55226	NAT10	N-acetyltransferase 10 (GCN5-related)	0.95058	0.91106	1.0514764
55227	LRRC1	leucine rich repeat containing 1	1.01233	0.992144	1.0064898
55228	PNMAL1	PNMA-like 1	1.00927	0.980769	0.9837507
55229	PANK4	pantothenate kinase 4	1.00215	1.01964	0.9739088
55230	USP40	ubiquitin specific peptidase 40	0.99107	1.001204	1.0283483
55231	CCDC87	coiled-coil domain containing 87	0.98763	0.9909	0.9796208
55233	MOBKL1B	MOB1, Mps One Binder kinase activator-like 1B (yeast)	1.02041	0.952163	0.9303806
55234	SMU1	smu-1 suppressor of mec-8 and unc-52 homolog (C. elegans)	0.95701	0.99598	0.9528505
55236	UBA6	ubiquitin-like modifier activating enzyme 6	0.98488	0.940702	1.014752
55237	VRTN	vertebrae development homolog (pig)	0.97517	0.930142	0.9931334
55238	SLC38A7	solute carrier family 38, member 7	0.99077	1.008443	1.0124715
55239	OGFOD1	2-oxoglutarate and iron-dependent oxygenase domain containing 1	0.90651	0.972612	1.054774
55240	STEAP3	STEAP family member 3	0.99693	0.961454	1.0445906
55243	KIRREL	kin of IRRE like (Drosophila)	1.01227	0.994031	1.0132394
55244	SLC47A1	solute carrier family 47, member 1	1.00581	0.99832	1.0308824
55245	UQCC	ubiquinol-cytochrome c reductase complex chaperone	0.96098	1.00091	1.0286629
55246	CCDC25	coiled-coil domain containing 25	0.97382	0.989802	0.9944805
55247	NEIL3	nei endonuclease VIII-like 3 (E. coli)	0.98815	0.950086	1.0119145
55248	TMEM206	transmembrane protein 206	1.00015	0.959767	1.0611828
55249	YY1AP1	YY1 associated protein 1	1.00989	0.998048	0.9660355
55250	ELP2	elongation protein 2 homolog (S. cerevisiae)	0.92936	0.926985	1.0010205

55251	PCMTD2	protein-L-isoaspartate (D-aspartate) O-methyltransferase domain containing 2	0.91744	0.878643	0.9426536
55252	ASXL2	additional sex combs like 2 (Drosophila)	0.96155	0.981954	0.9798197
55253	TYW1	tRNA-yW synthesizing protein 1 homolog (S. cerevisiae)	0.97392	1.003712	1.0248702
55254	TMEM39A	transmembrane protein 39A	1.01413	0.9932	1.001026
55255	WDR41	WD repeat domain 41	1.01807	0.928037	1.0508749
55256	ADI1	acireductone dioxygenase 1	0.99367	0.93415	1.0623613
55257	C20orf20	chromosome 20 open reading frame 20	0.96231	1.024881	0.9990604
55258	THNSL2	threonine synthase-like 2 (S. cerevisiae)	0.9977	0.993948	0.9837857
55259	CASC1	cancer susceptibility candidate 1	0.98501	0.990199	0.9972124
55260	TMEM143	transmembrane protein 143	0.98672	1.001793	1.0269838
55262	C7orf43	chromosome 7 open reading frame 43	0.97606	1.038159	1.0122417
55264	[No Symbo	[No Name]	0.99568	0.986663	1.0011143
55266	TMEM19	transmembrane protein 19	0.94186	0.984011	1.0463504
55267	C22orf26	chromosome 22 open reading frame 26	0.99121	0.971486	1.0214354
55268	ECHDC2	enoyl CoA hydratase domain containing 2	0.98999	0.931244	0.9714679
55269	PSPC1	paraspeckle component 1	0.95027	1.007924	0.9966872
55270	NUDT15	nudix (nucleoside diphosphate linked moiety X)-type motif 15	0.95696	0.962747	0.9919023
55272	IMP3	IMP3, U3 small nucleolar ribonucleoprotein, homolog (yeast)	0.89235	0.98475	1.0531207
55273	TMEM100	transmembrane protein 100	1.00621	0.993004	1.0371997
55274	PHF10	PHD finger protein 10	0.9917	0.937313	0.905344
55275	VPS53	vacuolar protein sorting 53 homolog (S. cerevisiae)	0.97033	1.013752	0.9366516
55276	PGM2	phosphoglucomutase 2	0.9945	0.868712	1.1475301
55277	FGGY	FGGY carbohydrate kinase domain containing	1.00378	1.007497	1.0018413
55278	QRSL1	glutaminyl-tRNA synthase (glutamine-hydrolyzing)-like 1	1.00709	0.93718	1.0535838
55279	ZNF654	zinc finger protein 654	1.00776	0.956296	1.0870297
55280	CWF19L1	CWF19-like 1, cell cycle control (S. pombe)	0.96524	0.966192	0.974551
55281	TMEM140	transmembrane protein 140	1.01357	0.790074	0.923684
55282	LRRC36	leucine rich repeat containing 36	1.00335	1.00471	1.007796
55283	MCOLN3	mucolipin 3	0.9926	1.011511	1.020202
55284	UBE2W	ubiquitin-conjugating enzyme E2W (putative)	0.96878	0.980304	1.0018571
55285	RBM41	RNA binding motif protein 41	0.94304	0.97109	1.0278386
55286	C4orf19	chromosome 4 open reading frame 19	1.01382	1.025979	1.0174377
55287	TMEM40	transmembrane protein 40	0.95254	0.97433	0.9726459

55288	RHOT1	ras homolog gene family, member T1	0.99041	0.96996	1.0780525
55289	ACOXL	acyl-CoA oxidase-like	1.00969	1.002282	1.0168301
55290	BRF2	BRF2, subunit of RNA polymerase III transcription initiation factor, BRF1-like	0.93099	0.926881	1.0713724
55291	PPP6R3	protein phosphatase 6, regulatory subunit 3	0.95944	1.040057	1.0085924
55293	UEVLD	UEV and lactate/malate dehyrogenase domains	0.91607	0.988605	1.1059692
55294	FBXW7	F-box and WD repeat domain containing 7	0.99378	0.96513	1.0161528
55295	KLHL26	kelch-like 26 (Drosophila)	0.96898	0.99044	1.0340933
55296	TBC1D19	TBC1 domain family, member 19	0.9769	0.962823	1.0237185
55297	CCDC91	coiled-coil domain containing 91	0.92023	0.959482	0.9207887
55299	BRIX1	BRX1, biogenesis of ribosomes, homolog (S. cerevisiae)	0.94246	0.92325	1.1004952
55300	PI4K2B	phosphatidylinositol 4-kinase type 2 beta	0.96971	0.981764	0.9948102
55301	OLAH	oleoyl-ACP hydrolase	0.98853	0.999815	1.0033776
55303	GIMAP4	GTPase, IMAP family member 4	0.97648	0.916567	0.9227964
55304	SPTLC3	serine palmitoyltransferase, long chain base subunit 3	0.99054	1.000759	0.9953389
55308	DDX19A	DEAD (Asp-Glu-Ala-As) box polypeptide 19A	0.97811	1.000156	0.96829
55311	ZNF444	zinc finger protein 444	1.00835	0.991154	1.0607715
55312	RFK	riboflavin kinase	0.96372	1.008688	1.0630459
55313	CPPED1	calcineurin-like phosphoesterase domain containing 1	1.00972	0.956085	0.9753758
55314	TMEM144	transmembrane protein 144	1.00067	0.966863	1.0268477
55315	SLC29A3	solute carrier family 29 (nucleoside transporters), member 3	0.99095	0.973528	0.9729984
55316	RSAD1	radical S-adenosyl methionine domain containing 1	0.93682	0.940898	1.0318777
55317	C20orf29	chromosome 20 open reading frame 29	0.95656	0.95212	1.1036452
55319	C4orf43	chromosome 4 open reading frame 43	1.01451	1.036115	1.0493735
55320	MIS18BP1	MIS18 binding protein 1	0.97212	1.005046	1.129285
55321	C20orf46	chromosome 20 open reading frame 46	0.99199	0.972822	0.9996022
55322	C5orf22	chromosome 5 open reading frame 22	0.95582	0.987269	1.0612309
55324	ABCF3	ATP-binding cassette, sub-family F (GCN20), member 3	0.9767	1.020467	0.9786652
55325	UFSP2	UFM1-specific peptidase 2	0.96643	0.884007	1.0034535
55326	AGPAT5	1-acylglycerol-3-phosphate O-acyltransferase 5 (lysophosphatidic acid acyltransferase, epsilon)	0.97069	1.055328	1.0546461
55327	LIN7C	lin-7 homolog C (C. elegans)	0.92924	0.917072	1.0365515
55328	RNLS	renalase, FAD-dependent amine oxidase	0.98269	0.978643	1.006015
55329	MNS1	meiosis-specific nuclear structural 1	0.9877	0.997173	1.0346707
55330	CNO	cappuccino homolog (mouse)	0.99758	0.978972	1.0407584

55331	ACER3	alkaline ceramidase 3	0.9649	0.96692	1.018525
55332	DRAM1	DNA-damage regulated autophagy modulator 1	0.96972	1.028741	1.0269175
55333	SYNJ2BP	synaptojanin 2 binding protein	0.95628	0.909588	1.0079702
55334	SLC39A9	solute carrier family 39 (zinc transporter), member 9	0.96994	0.988824	1.043946
55335	NIPSNAP3B	nipsnap homolog 3B (C. elegans)	0.97398	0.943988	1.0262416
55336	FBXL8	F-box and leucine-rich repeat protein 8	0.96912	0.943792	1.112766
55337	C19orf66	chromosome 19 open reading frame 66	0.96816	1.003024	1.0791992
55339	WDR33	WD repeat domain 33	0.97744	0.968457	0.9313119
55340	GIMAP5	GTPase, IMAP family member 5	0.97272	0.846752	0.8840352
55341	LSG1	large subunit GTPase 1 homolog (S. cerevisiae)	0.96346	0.883925	1.0884956
55342	STRBP	spermatid perinuclear RNA binding protein	0.95124	0.996127	1.0223294
55343	SLC35C1	solute carrier family 35, member C1	0.96828	0.955991	1.0015157
55345	C4orf21	chromosome 4 open reading frame 21	0.99992	0.968167	1.0160321
55346	TCP11L1	t-complex 11 (mouse)-like 1	0.95383	0.969536	0.9962056
55347	ABHD10	abhydrolase domain containing 10	0.99396	0.978908	1.0535157
55349	CHDH	choline dehydrogenase	1.01257	1.011364	1.0020936
55350	VNN3	vanin 3	1.02576	1.010335	0.939554
55351	STK32B	serine/threonine kinase 32B	1.01931	0.989764	1.0041611
55352	C17orf79	chromosome 17 open reading frame 79	0.97513	0.953715	1.0569615
55353	LAPTM4B	lysosomal protein transmembrane 4 beta	0.95868	0.964809	1.0155556
55355	HJURP	Holliday junction recognition protein	1.00748	1.008297	1.0033289
55356	SLC22A15	solute carrier family 22, member 15	1.01132	0.990241	1.0068616
55357	TBC1D2	TBC1 domain family, member 2	0.94065	1.057465	0.9831312
55359	STYK1	serine/threonine/tyrosine kinase 1	0.99823	1.049923	1.0240506
55361	PI4K2A	phosphatidylinositol 4-kinase type 2 alpha	0.87677	1.033364	0.9873292
55362	TMEM63B	transmembrane protein 63B	1.0101	1.026076	1.0558576
55363	HEMGN	hemogen	0.9374	0.911942	0.9842605
55364	IMPACT	Impact homolog (mouse)	0.9414	0.950056	0.9987559
55365	TMEM176A	transmembrane protein 176A	1.06162	1.074964	1.0299559
55366	LGR4	leucine-rich repeat containing G protein-coupled receptor 4	0.98106	0.991769	0.982239
55367	PIDD	p53-induced death domain protein	0.99863	1.045011	1.0427921
55370	PPP4R1L	protein phosphatase 4, regulatory subunit 1-like	0.97774	0.998204	0.9225233
55374	TMCO6	transmembrane and coiled-coil domains 6	1.02287	0.967598	0.9909872

55379	LRRC59	leucine rich repeat containing 59	0.96222	0.938889	1.1461988
55384	MEG3	maternally expressed 3 (non-protein coding)	1.01412	1.020933	0.9981645
55388	MCM10	minichromosome maintenance complex component 10	1.00055	0.978	1.0077287
55410	NCRNA001	non-protein coding RNA 185	0.98631	0.965167	0.975447
55421	C17orf85	chromosome 17 open reading frame 85	0.96657	1.004197	1.065207
55422	ZNF331	zinc finger protein 331	0.95696	1.055371	0.9279368
55423	SIRPG	signal-regulatory protein gamma	0.93704	0.895245	1.0109813
55425	KIAA1704	KIAA1704	0.9376	0.938	1.0511996
55432	YOD1	YOD1 OTU deubiquinating enzyme 1 homolog (S. cerevisiae)	1.00801	0.93969	0.9868996
55435	AP1AR	adaptor-related protein complex 1 associated regulatory protein	0.98943	1.002293	0.9189855
55437	STRADB	STE20-related kinase adaptor beta	1.00279	0.983881	0.9751391
55450	CAMK2N1	calcium/calmodulin-dependent protein kinase II inhibitor 1	0.95854	1.010433	1.0333506
55454	CSGALNAC	chondroitin sulfate N-acetylgalactosaminyltransferase 2	0.98255	0.961496	0.9681745
55466	DNAJA4	DnaJ (Hsp40) homolog, subfamily A, member 4	0.98426	0.949551	0.9372748
55471	C2orf56	chromosome 2 open reading frame 56	1.02564	1.01543	0.9609914
55486	PARL	presenilin associated, rhomboid-like	0.98944	0.956462	1.0137529
55500	ETNK1	ethanolamine kinase 1	0.9784	0.994604	1.1168481
55501	CHST12	carbohydrate (chondroitin 4) sulfotransferase 12	0.98502	1.016414	0.9895998
55502	HES6	hairy and enhancer of split 6 (Drosophila)	0.98345	1.021695	1.1066949
55503	TRPV6	transient receptor potential cation channel, subfamily V, member 6	1.02	1.018766	0.9966538
55504	TNFRSF19	tumor necrosis factor receptor superfamily, member 19	0.98629	0.998975	0.9983575
55505	NOP10	NOP10 ribonucleoprotein homolog (yeast)	0.90422	0.96183	1.0807187
55506	H2AFY2	H2A histone family, member Y2	0.9899	0.989687	0.9797018
55507	GPRC5D	G protein-coupled receptor, family C, group 5, member D	1.04924	1.008454	0.9919317
55508	SLC35E3	solute carrier family 35, member E3	0.96688	0.952123	1.0406327
55509	BATF3	basic leucine zipper transcription factor, ATF-like 3	1.01479	1.025316	1.0505441
55510	DDX43	DEAD (Asp-Glu-Ala-Asp) box polypeptide 43	0.98758	1.060581	0.9688813
55511	SAGE1	sarcoma antigen 1	1.00073	0.984007	0.9872086
55512	SMPD3	sphingomyelin phosphodiesterase 3, neutral membrane (neutral sphingomyelinase II)	0.9958	0.970853	1.0138045
55515	ACCN4	amiloride-sensitive cation channel 4, pituitary	0.9953	1	1.0229968
55520	ELAC1	elaC homolog 1 (E. coli)	0.96771	0.963404	0.9915993
55521	TRIM36	tripartite motif containing 36	0.99428	1.035651	1.0388173
55526	DHTKD1	dehydrogenase E1 and transketolase domain containing 1	0.99731	0.958576	1.02945

55527	FEM1A	fem-1 homolog a (C. elegans)	0.94731	0.944397	1.1387754
55529	TMEM55A	transmembrane protein 55A	0.9069	0.932369	1.0259816
55530	SVOP	SV2 related protein homolog (rat)	1.01842	0.992981	0.9855328
55531	ELMOD1	ELMO/CED-12 domain containing 1	1.0082	1.003097	1.0031033
55532	SLC30A10	solute carrier family 30, member 10	1.01154	0.984553	1.0330706
55534	MAML3	mastermind-like 3 (Drosophila)	1.00843	0.977422	0.9955529
55536	CDCA7L	cell division cycle associated 7-like	1.0056	0.970597	1.0104618
55539	KCNQ1DN	KCNQ1 downstream neighbor	1.01256	1.042532	0.9939321
55540	IL17RB	interleukin 17 receptor B	1.01138	0.986366	1.0020766
55544	RBM38	RNA binding motif protein 38	0.97133	1.04843	0.948843
55545	MSX2P1	msh homeobox 2 pseudogene 1	0.97119	0.980769	1.109935
55552	ZNF823	zinc finger protein 823	0.97741	1.003245	1.0287482
55553	SOX6	SRY (sex determining region Y)-box 6	0.98591	1.004859	1.0080919
55554	KLK15	kallikrein-related peptidase 15	0.98863	1.01195	0.9794183
55556	ENOSF1	enolase superfamily member 1	0.98505	1.065843	0.9432723
55558	PLXNA3	plexin A3	0.96953	0.997647	1.0456253
55559	HAUS7	HAUS augmin-like complex, subunit 7	0.97166	0.972931	1.0316699
55561	CDC42BPG	CDC42 binding protein kinase gamma (DMPK-like)	0.9811	1.016448	0.9773264
55565	ZNF821	zinc finger protein 821	0.99435	0.977603	0.9772576
55567	DNAH3	dynein, axonemal, heavy chain 3	0.99739	0.987975	1.0171212
55568	GALNT10	UDP-N-acetyl-alpha-D-galactosamine:polypeptide N-acetylgalactosaminyltransferase 10 (GalNAc-T10)	1.00741	0.95265	1.0496409
55571	C2orf29	chromosome 2 open reading frame 29	0.99095	0.958947	1.025339
55572	FOXRED1	FAD-dependent oxidoreductase domain containing 1	0.97514	1.017846	1.0190661
55573	CDV3	CDV3 homolog (mouse)	1.00489	1.003928	1.0593109
55576	STAB2	stabilin 2	1.00198	0.997264	0.9863401
55577	NAGK	N-acetylglucosamine kinase	1.07415	0.965702	0.9957559
55578	FAM48A	family with sequence similarity 48, member A	0.93092	0.971105	1.0850148
55582	KIF27	kinesin family member 27	0.98895	1.005328	1.0357512
55584	CHRNA9	cholinergic receptor, nicotinic, alpha 9	1.00463	1.017325	1.0100878
55585	UBE2Q1	ubiquitin-conjugating enzyme E2Q family member 1	0.98098	0.945313	1.0093288
55586	MIOX	myo-inositol oxygenase	0.98989	1.03346	1.0387894
55588	MED29	mediator complex subunit 29	0.89766	0.976653	0.9437316
55589	BMP2K	BMP2 inducible kinase	1.02408	0.982716	1.0731019

55591	VEZT	vezatin, adherens junctions transmembrane protein	0.96249	0.932394	1.0245104
55592	GOLGA2B	golgin A2 family, member B	0.9717	0.966581	1.003591
55593	OTUD5	OTU domain containing 5	0.96859	1.018084	0.9524531
55596	ZCCHC8	zinc finger, CCHC domain containing 8	0.95439	0.98375	1.0079758
55599	RNPC3	RNA-binding region (RNP1, RRM) containing 3	1.05846	0.960717	0.9851916
55600	ITLN1	intelectin 1 (galactofuranose binding)	1.00222	0.967518	1.0263425
55601	DDX60	DEAD (Asp-Glu-Ala-Asp) box polypeptide 60	0.96855	0.971346	1.0295743
55602	CDKN2AIP	CDKN2A interacting protein	0.93083	0.929547	0.9795361
55603	FAM46A	family with sequence similarity 46, member A	0.96905	0.959207	0.9995099
55604	LRRC16A	leucine rich repeat containing 16A	0.9773	0.990803	1.0433512
55605	KIF21A	kinesin family member 21A	0.96954	1.06786	0.9424541
55607	PPP1R9A	protein phosphatase 1, regulatory (inhibitor) subunit 9A	1.00704	0.986826	0.9897617
55608	ANKRD10	ankyrin repeat domain 10	0.98319	0.994744	1.1204875
55609	ZNF280C	zinc finger protein 280C	0.9359	0.974735	1.0496584
55610	CCDC132	coiled-coil domain containing 132	0.95516	0.908954	1.0857346
55611	OTUB1	OTU domain, ubiquitin aldehyde binding 1	0.94497	0.961632	1.0927052
55612	FERMT1	fermitin family member 1	1.00324	0.999537	0.9941381
55613	MTMR8	myotubularin related protein 8	0.96608	0.972851	0.9541869
55614	KIF16B	kinesin family member 16B	0.96768	0.931704	0.9716768
55615	PRR5	proline rich 5 (renal)	1.01272	1.036943	0.9939131
55616	ASAP3	ArfGAP with SH3 domain, ankyrin repeat and PH domain 3	1.00758	0.999367	1.001232
55617	TASP1	taspase, threonine aspartase, 1	0.99414	0.949762	1.0414665
55619	DOCK10	dedicator of cytokinesis 10	0.97028	0.920243	0.856876
55620	STAP2	signal transducing adaptor family member 2	0.98467	1.012933	1.0228506
55621	TRMT1	TRM1 tRNA methyltransferase 1 homolog (S. cerevisiae)	0.95053	1.001321	1.0342734
55622	TTC27	tetratricopeptide repeat domain 27	0.98415	0.892674	0.9628751
55623	THUMPD1	THUMP domain containing 1	0.93832	0.909676	0.9455965
55624	POMGNT1	protein O-linked mannose beta1,2-N-acetylglucosaminyltransferase	0.97843	0.944983	1.0251664
55625	ZDHHC7	zinc finger, DHHC-type containing 7	0.96311	0.997094	1.0318627
55626	AMBRA1	autophagy/beclin-1 regulator 1	0.96473	0.954254	0.9842725
55627	SMPD4	sphingomyelin phosphodiesterase 4, neutral membrane (neutral sphingomyelinase-3)	0.98488	0.936614	1.0099343
55628	ZNF407	zinc finger protein 407	0.98606	0.872635	0.963783
55629	PNRC2	proline-rich nuclear receptor coactivator 2	1.00399	0.808671	0.9306853

55630	SLC39A4	solute carrier family 39 (zinc transporter), member 4	0.97376	0.942132	1.0645119
55631	LRRC40	leucine rich repeat containing 40	0.99782	0.928911	1.0080192
55632	G2E3	G2/M-phase specific E3 ubiquitin protein ligase	0.9762	0.940184	1.0362874
55633	TBC1D22B	TBC1 domain family, member 22B	0.98741	1.000974	1.0455549
55634	ZNF673	zinc finger family member 673	0.93762	0.982306	0.9968299
55635	DEPDC1	DEP domain containing 1	0.99966	1.005631	1.0187837
55636	CHD7	chromodomain helicase DNA binding protein 7	0.96396	0.988355	1.0372354
55638	SYBU	syntabulin (syntaxin-interacting)	0.98304	1.013046	1.0030186
55640	FLVCR2	feline leukemia virus subgroup C cellular receptor family, member 2	0.99413	1.058378	1.0684496
55643	BTBD2	BTB (POZ) domain containing 2	0.93334	0.960538	1.0972153
55644	OSGEP	O-sialoglycoprotein endopeptidase	0.96852	0.99961	0.9720721
55646	LYAR	Ly1 antibody reactive homolog (mouse)	0.97646	1.012464	0.9537174
55650	PIGV	phosphatidylinositol glycan anchor biosynthesis, class V	0.96266	1.058752	1.0546578
55651	NHP2	NHP2 ribonucleoprotein homolog (yeast)	1.00151	0.926486	1.0879264
55652	SLC48A1	solute carrier family 48 (heme transporter), member 1	0.98107	0.948849	1.0209119
55653	BCAS4	breast carcinoma amplified sequence 4	0.97991	1.010445	1.0437784
55654	TMEM127	transmembrane protein 127	1.01775	1.008351	0.9492352
55655	NLRP2	NLR family, pyrin domain containing 2	1.00418	1.109431	0.9800864
55656	INTS8	integrator complex subunit 8	1.02248	1.001801	1.0673508
55657	ZNF692	zinc finger protein 692	0.99661	1.000171	0.9903922
55658	RNF126	ring finger protein 126	0.98195	1.004507	1.0656229
55659	ZNF416	zinc finger protein 416	0.90286	0.977526	1.0772953
55660	PRPF40A	PRP40 pre-mRNA processing factor 40 homolog A (S. cerevisiae)	1.00947	0.866047	1.0618816
55661	DDX27	DEAD (Asp-Glu-Ala-Asp) box polypeptide 27	0.89135	0.953102	1.0442639
55662	HIF1AN	hypoxia inducible factor 1, alpha subunit inhibitor	0.93549	0.927486	1.047433
55663	ZNF446	zinc finger protein 446	0.9899	0.982189	1.0019101
55664	CDC37L1	cell division cycle 37 homolog (S. cerevisiae)-like 1	0.94203	0.941715	0.8958979
55665	URGCP	upregulator of cell proliferation	1.001	0.960504	0.9862967
55666	NPLOC4	nuclear protein localization 4 homolog (S. cerevisiae)	0.98292	0.956381	1.0510515
55667	DENND4C	DENN/MADD domain containing 4C	0.90385	1.000495	1.0476833
55668	C14orf118	chromosome 14 open reading frame 118	0.948	0.967494	1.024471
55669	MFN1	mitofusin 1	1.00084	1.011821	1.0075212
55670	PEX26	peroxisomal biogenesis factor 26	0.95826	0.966004	1.0345228

55671	SMEK1	SMEK homolog 1, suppressor of mek1 (Dictyostelium)	0.96264	0.952286	0.9867153
55672	NBPF1	neuroblastoma breakpoint family, member 1	0.98296	0.975681	1.0025872
55676	SLC30A6	solute carrier family 30 (zinc transporter), member 6	0.94961	0.954275	1.0655953
55677	IWS1	IWS1 homolog (S. cerevisiae)	0.97594	0.999039	1.1133855
55679	LIMS2	LIM and senescent cell antigen-like domains 2	0.99686	1.014119	0.9877774
55680	RUFY2	RUN and FYVE domain containing 2	0.94969	1.024324	1.0785336
55681	SCYL2	SCY1-like 2 (S. cerevisiae)	0.95963	1.083896	0.9086734
55683	KIAA1310	KIAA1310	0.98633	1.074095	0.9165755
55684	C9orf86	chromosome 9 open reading frame 86	1.00016	0.995356	1.0503886
55686	MREG	melanoregulin	0.99627	0.982688	1.0325647
55687	TRMU	tRNA 5-methylaminomethyl-2-thiouridylate methyltransferase	0.9631	1.019193	1.066569
55689	YEATS2	YEATS domain containing 2	0.98272	1.010972	1.0256171
55690	PACS1	phosphofurin acidic cluster sorting protein 1	0.92613	0.970345	0.8489018
55691	FRMD4A	FERM domain containing 4A	0.99432	0.995655	0.9955127
55692	LUC7L	LUC7-like (S. cerevisiae)	0.95455	1.003574	0.977608
55693	KDM4D	lysine (K)-specific demethylase 4D	0.99071	0.986987	1.0132112
55695	NSUN5	NOP2/Sun domain family, member 5	1.02428	0.883683	1.0791263
55696	RBM22	RNA binding motif protein 22	0.96758	0.993659	0.995852
55697	VAC14	Vac14 homolog (S. cerevisiae)	0.96391	0.970351	1.01491
55698	RADIL	Ras association and DIL domains	1.00219	1.009373	1.039288
55699	IARS2	isoleucyl-tRNA synthetase 2, mitochondrial	0.97633	0.965138	1.0407457
55700	MAP7D1	MAP7 domain containing 1	0.97838	0.967308	0.9757595
55701	ARHGEF40	Rho guanine nucleotide exchange factor (GEF) 40	0.96934	0.940875	0.9846002
55702	CCDC94	coiled-coil domain containing 94	0.93251	0.955947	1.0682632
55703	POLR3B	polymerase (RNA) III (DNA directed) polypeptide B	0.95798	0.920341	1.0106508
55704	CCDC88A	coiled-coil domain containing 88A	1.04039	0.900154	1.1494695
55705	IPO9	importin 9	0.96565	0.939722	1.023366
55706	TMEM48	transmembrane protein 48	0.98512	1.034198	1.0189764
55707	NECAP2	NECAP endocytosis associated 2	1.02397	0.933537	0.9995236
55709	KBTBD4	kelch repeat and BTB (POZ) domain containing 4	0.90592	0.990549	1.1406069
55711	FAR2	fatty acyl CoA reductase 2	0.97875	0.946615	1.0757308
55713	ZNF334	zinc finger protein 334	0.99689	0.988911	1.0118329
55714	ODZ3	odz, odd Oz/ten-m homolog 3 (Drosophila)	1.00765	1.011837	1.0122093

55715	DOK4	docking protein 4	0.9728	0.99121	1.0159588
55716	LMBR1L	limb region 1 homolog (mouse)-like	0.97116	0.995859	0.9765035
55717	WDR11	WD repeat domain 11	0.98551	1.018564	1.1290729
55718	POLR3E	polymerase (RNA) III (DNA directed) polypeptide E (80kD)	0.92905	1.007933	0.9077097
55719	FAM178A	family with sequence similarity 178, member A	0.9459	0.95985	1.0005168
55720	TSR1	TSR1, 20S rRNA accumulation, homolog (S. cerevisiae)	0.93598	0.99834	1.068938
55721	IQCC	IQ motif containing C	1.01648	0.942762	1.0221669
55722	CEP72	centrosomal protein 72kDa	0.98872	0.985239	1.0003963
55723	ASF1B	ASF1 anti-silencing function 1 homolog B (S. cerevisiae)	0.96965	0.977142	1.0483213
55726	C12orf11	chromosome 12 open reading frame 11	0.90905	1.018153	1.0776823
55727	BTBD7	BTB (POZ) domain containing 7	0.9774	0.969893	1.0637553
55728	N4BP2	NEDD4 binding protein 2	0.96467	1.007129	0.9706792
55729	ATF7IP	activating transcription factor 7 interacting protein	0.95419	0.996974	0.9632254
55731	C17orf63	chromosome 17 open reading frame 63	0.97684	1.010418	0.9780409
55732	C1orf112	chromosome 1 open reading frame 112	0.99765	0.979943	1.0128071
55733	HHAT	hedgehog acyltransferase	1.00999	0.979148	1.0118429
55734	ZFP64	zinc finger protein 64 homolog (mouse)	0.96461	0.989386	0.9856029
55735	DNAJC11	DnaJ (Hsp40) homolog, subfamily C, member 11	0.99624	0.904016	0.9649983
55737	VPS35	vacuolar protein sorting 35 homolog (S. cerevisiae)	0.97416	0.990836	1.0071326
55738	ARFGAP1	ADP-ribosylation factor GTPase activating protein 1	0.98434	1.020958	0.9708768
55739	CARKD	carbohydrate kinase domain containing	0.94181	0.924713	1.0804378
55740	ENAH	enabled homolog (Drosophila)	1.00091	0.998196	0.995466
55741	EDEM2	ER degradation enhancer, mannosidase alpha-like 2	0.97451	0.969482	1.0368936
55742	PARVA	parvin, alpha	0.98684	0.984552	0.9791984
55743	CHFR	checkpoint with forkhead and ring finger domains	0.96269	0.94228	0.9881254
55744	C7orf44	chromosome 7 open reading frame 44	0.98434	0.942392	1.0535396
55745	MUDENG	MU-2/AP1M2 domain containing, death-inducing	0.94329	1.090777	1.0482177
55746	NUP133	nucleoporin 133kDa	0.99332	0.95356	1.0806877
55748	CNDP2	CNDP dipeptidase 2 (metallopeptidase M20 family)	0.96446	0.967697	1.0060027
55749	CCAR1	cell division cycle and apoptosis regulator 1	0.93408	1.007591	1.0217718
55750	AGK	acylglycerol kinase	1.01422	0.959455	0.971354
55751	TMEM184C	transmembrane protein 184C	0.98312	1.021103	0.9331463
55752	11-Sep	septin 11	0.99703	0.945448	1.0475101

55753	OGDHL	oxoglutarate dehydrogenase-like	1	1.01973	1.0207676
55754	TMEM30A	transmembrane protein 30A	0.99653	0.955726	1.1214044
55755	CDK5RAP2	CDK5 regulatory subunit associated protein 2	0.93775	0.943035	1.006209
55756	INTS9	integrator complex subunit 9	0.95007	0.999419	1.0028381
55757	UGGT2	UDP-glucose glycoprotein glucosyltransferase 2	0.97421	0.968741	1.0409247
55758	RCOR3	REST corepressor 3	1.00047	0.990944	0.9896639
55759	WDR12	WD repeat domain 12	0.97284	0.948874	1.0047579
55760	DHX32	DEAH (Asp-Glu-Ala-His) box polypeptide 32	0.95877	0.970621	1.0623617
55761	TTC17	tetratricopeptide repeat domain 17	0.94862	0.972057	1.0659215
55762	ZNF701	zinc finger protein 701	0.8988	0.943825	1.0072021
55763	EXOC1	exocyst complex component 1	1.00305	1.019308	1.0469074
55764	IFT122	intraflagellar transport 122 homolog (Chlamydomonas)	1.00337	0.976271	0.9970475
55765	C1orf106	chromosome 1 open reading frame 106	1.00962	1.021849	0.9994442
55766	H2AFJ	H2A histone family, member J	0.95449	0.989727	0.9591114
55768	NGLY1	N-glycanase 1	0.99994	0.989705	1.011185
55769	ZNF83	zinc finger protein 83	0.95815	0.990688	0.9772506
55770	EXOC2	exocyst complex component 2	0.97701	0.987984	0.9760773
55771	PRR11	proline rich 11	0.95745	0.907485	1.0562938
55773	TBC1D23	TBC1 domain family, member 23	0.97194	0.951815	0.9846241
55775	TDP1	tyrosyl-DNA phosphodiesterase 1	0.95179	0.976641	0.9689758
55776	C6orf64	chromosome 6 open reading frame 64	0.99319	0.985316	0.9028167
55777	MBD5	methyl-CpG binding domain protein 5	0.9851	0.924404	1.0323772
55778	ZNF839	zinc finger protein 839	0.94892	1.00431	1.0044958
55779	WDR52	WD repeat domain 52	0.96855	0.943713	0.9946511
55780	C6orf70	chromosome 6 open reading frame 70	1.01142	0.988444	1.0693474
55781	RIOK2	RIO kinase 2 (yeast)	0.98153	0.922553	1.0337607
55783	FTSJD1	FtsJ methyltransferase domain containing 1	0.94911	0.953042	1.1106559
55784	MCTP2	multiple C2 domains, transmembrane 2	0.98711	1.10676	1.0387905
55785	FGD6	FYVE, RhoGEF and PH domain containing 6	0.9802	0.964328	0.989536
55786	ZNF415	zinc finger protein 415	0.98683	0.987711	0.9700772
55787	TXLNG	taxilin gamma	0.92737	0.986124	1.0229085
55788	LMBRD1	LMBR1 domain containing 1	1.00971	0.847887	0.9928216
55789	DEPDC1B	DEP domain containing 1B	1.0031	1.01732	1.0151434

55790	CSGALNACT	chondroitin sulfate N-acetylgalactosaminyltransferase 1	0.98713	0.955913	0.968139
55791	LRIF1	ligand dependent nuclear receptor interacting factor 1	0.97594	0.960399	0.937436
55793	FAM63A	family with sequence similarity 63, member A	0.96689	0.916244	0.9723254
55794	DDX28	DEAD (Asp-Glu-Ala-Asp) box polypeptide 28	0.92718	0.957962	1.0113015
55795	PCID2	PCI domain containing 2	0.89758	1.066143	0.9911052
55796	MBNL3	muscleblind-like 3 (Drosophila)	0.97033	0.893616	1.1104326
55798	METTL2B	methyltransferase like 2B	0.98107	1.064289	1.0093745
55799	CACNA2D3	calcium channel, voltage-dependent, alpha 2/delta subunit 3	1.0105	0.993645	1.0022513
55800	SCN3B	sodium channel, voltage-gated, type III, beta	0.99818	0.987926	0.9836782
55801	IL26	interleukin 26	0.99135	1.037255	1.0040787
55802	DCP1A	DCP1 decapping enzyme homolog A (S. cerevisiae)	0.99959	0.920384	0.9023167
55803	ADAP2	ArfGAP with dual PH domains 2	0.98579	0.97028	1.0116073
55805	LRP2BP	LRP2 binding protein	1.02281	1.016486	1.004767
55806	HR	hairless homolog (mouse)	1.00016	0.978644	1.0493559
55808	ST6GALNAC	ST6 (alpha-N-acetyl-neuraminyl-2,3-beta-galactosyl-1,3)-N-acetylgalactosaminide alpha-2,6-sialyltransferase	1.02174	0.98447	0.9835255
55809	TRERF1	transcriptional regulating factor 1	0.96495	0.975994	0.9944828
55811	ADCY10	adenylate cyclase 10 (soluble)	0.99525	0.989156	1.0186983
55812	SPATA7	spermatogenesis associated 7	0.96863	0.988196	1.0227108
55813	UTP6	UTP6, small subunit (SSU) processome component, homolog (yeast)	0.96283	1.000236	1.0273594
55814	BDP1	B double prime 1, subunit of RNA polymerase III transcription initiation factor IIIB	0.96066	0.949259	1.0418863
55815	TSNAXIP1	translin-associated factor X interacting protein 1	0.99843	1.001202	1.0014094
55816	DOK5	docking protein 5	0.99738	0.987934	1.0105183
55818	KDM3A	lysine (K)-specific demethylase 3A	0.97726	0.983429	1.0205178
55819	RNF130	ring finger protein 130	1.03202	0.949816	1.0139441
55821	ALLC	allantoicase	1.01633	1.008599	1.0265187
55823	VPS11	vacuolar protein sorting 11 homolog (S. cerevisiae)	0.91354	0.890899	1.0709343
55824	PAG1	phosphoprotein associated with glycosphingolipid microdomains 1	0.95779	0.984087	0.9800246
55825	PECR	peroxisomal trans-2-enoyl-CoA reductase	0.98902	0.94444	1.0230437
55827	DCAF6	DDB1 and CUL4 associated factor 6	0.99173	0.980102	1.001382
55829	SELS	selenoprotein S	0.95445	1.07165	1.0246395
55830	GLT8D1	glycosyltransferase 8 domain containing 1	0.97052	0.953685	1.0833886
55831	TMEM111	transmembrane protein 111	0.92983	0.821324	0.9498436
55832	CAND1	cullin-associated and neddylation-dissociated 1	0.93456	0.962149	1.0444031

55833	UBAP2	ubiquitin associated protein 2	0.94623	0.993037	1.0013534
55835	CENPJ	centromere protein J	0.97567	0.969123	1.0181933
55837	EAPP	E2F-associated phosphoprotein	0.91079	0.942419	0.9428882
55839	CENPN	centromere protein N	0.99296	1.004818	1.0155605
55840	EAF2	ELL associated factor 2	0.99992	0.984882	1.0263456
55841	WWC3	WWC family member 3	0.96891	0.989466	0.9770849
55843	ARHGAP15	Rho GTPase activating protein 15	0.99366	0.854526	0.8740547
55844	PPP2R2D	protein phosphatase 2, regulatory subunit B, delta	0.94395	1.069475	1.0081528
55845	BRK1	BRICK1, SCAR/WAVE actin-nucleating complex subunit	0.97436	0.988795	1.0183691
55846	ITFG2	integrin alpha FG-GAP repeat containing 2	0.95828	0.960529	0.9906407
55847	CISD1	CDGSH iron sulfur domain 1	0.97901	0.982192	1.0352233
55848	C9orf46	chromosome 9 open reading frame 46	0.96299	0.972046	1.031063
55850	USE1	unconventional SNARE in the ER 1 homolog (S. cerevisiae)	0.94043	0.952265	1.0273113
55851	PSENEN	presenilin enhancer 2 homolog (C. elegans)	0.94931	0.917035	1.1508212
55852	TEX2	testis expressed 2	0.99487	0.959753	1.0173117
55853	IDI2-AS1	IDI2 antisense RNA 1 (non-protein coding)	1.00498	0.999285	0.9717405
55854	ZC3H15	zinc finger CCCH-type containing 15	1.00721	0.871618	0.9815077
55856	ACOT13	acyl-CoA thioesterase 13	0.97097	0.976331	1.0360021
55857	PLK1S1	polo-like kinase 1 substrate 1	0.98321	1.02233	0.9250428
55858	TMEM165	transmembrane protein 165	1.02653	1.088351	0.9765135
55859	BEX1	brain expressed, X-linked 1	1.01293	0.933696	0.9990229
55860	ACTR10	actin-related protein 10 homolog (S. cerevisiae)	0.94555	0.992618	0.9272151
55862	ECHDC1	enoyl CoA hydratase domain containing 1	1.00007	0.961823	1.0315101
55863	TMEM126B	transmembrane protein 126B	0.94475	1.023943	1.1075071
55867	SLC22A11	solute carrier family 22 (organic anion/urate transporter), member 11	0.98205	1.005427	0.998134
55869	HDAC8	histone deacetylase 8	0.99353	0.975329	0.9967472
55870	ASH1L	ash1 (absent, small, or homeotic)-like (Drosophila)	0.9781	1.004497	0.9546356
55876	GSDMB	gasdermin B	0.96673	1.035592	0.9760111
55879	GABRQ	gamma-aminobutyric acid (GABA) receptor, theta	0.9939	1.002067	1.0021239
55884	WSB2	WD repeat and SOCS box containing 2	0.97455	0.949132	1.136553
55885	LMO3	LIM domain only 3 (rhombotin-like 2)	0.98964	1.018219	1.0043739
55888	ZNF167	zinc finger protein 167	0.98075	1.006489	1.0842848
55890	GPRC5C	G protein-coupled receptor, family C, group 5, member C	0.98493	0.997807	0.9909432

55892	MYNN	myoneurin	1.00757	0.927953	0.9388658
55893	ZNF395	zinc finger protein 395	0.93435	0.974408	1.0170747
55897	MESP1	mesoderm posterior 1 homolog (mouse)	0.98996	0.976979	1.0019352
55898	UNC45A	unc-45 homolog A (C. elegans)	0.95276	0.919123	1.0628464
55900	ZNF302	zinc finger protein 302	0.94812	0.933252	1.0594665
55901	THSD1	thrombospondin, type I, domain containing 1	1.00062	0.997933	0.9789559
55902	ACSS2	acyl-CoA synthetase short-chain family member 2	0.98055	0.926426	1.0093744
55904	MLL5	myeloid/lymphoid or mixed-lineage leukemia 5 (trithorax homolog, Drosophila)	1.01055	1.085304	0.9447635
55905	RNF114	ring finger protein 114	0.92729	0.967551	1.0190186
55906	ZC4H2	zinc finger, C4H2 domain containing	0.94118	0.951749	0.940481
55907	CMAS	cytidine monophosphate N-acetylneuraminic acid synthetase	0.96302	0.938754	1.0343528
55908	LOC55908	hepatocellular carcinoma-associated gene TD26	1.00037	1.017535	1.0674177
55909	BIN3	bridging integrator 3	0.96322	1.01743	0.9549343
55911	APOBR	apolipoprotein B receptor	0.94168	0.994879	0.9665756
55914	ERBB2IP	erbb2 interacting protein	1.00234	0.920833	1.0258947
55915	LANCL2	LanC lantibiotic synthetase component C-like 2 (bacterial)	0.96434	0.938582	1.0231815
55916	NXT2	nuclear transport factor 2-like export factor 2	0.95805	0.922477	1.0622436
55917	CTTNBP2NI	CTTNBP2 N-terminal like	0.98722	0.976129	0.9993547
55920	RCC2	regulator of chromosome condensation 2	1.00013	0.951358	1.1013918
55922	NKRF	NFKB repressing factor	0.94014	1.007726	0.9939565
55924	C1orf183	chromosome 1 open reading frame 183	1.01255	0.992315	1.0128416
55929	DMAP1	DNA methyltransferase 1 associated protein 1	0.98614	0.985251	1.0381535
55930	MYO5C	myosin VC	0.98886	0.995196	0.9953016
55937	APOM	apolipoprotein M	1.03097	0.994622	0.9615255
55954	ZMAT5	zinc finger, matrin-type 5	0.93779	0.947865	1.0074357
55957	LIN37	lin-37 homolog (C. elegans)	0.95447	1.018613	1.0206356
55958	KLHL9	kelch-like 9 (Drosophila)	0.91618	0.923738	1.1287566
55959	SULF2	sulfatase 2	0.97573	1.06106	0.9947291
55964	3-Sep	septin 3	0.99391	0.97346	1.0023264
55966	AJAP1	adherens junctions associated protein 1	1.00528	1.006274	1.0131899
55967	NDUFA12	NADH dehydrogenase (ubiquinone) 1 alpha subcomplex, 12	0.95966	0.962083	1.1516634
55968	NSFL1C	NSFL1 (p97) cofactor (p47)	0.9571	0.907185	1.0472418
55969	C20orf24	chromosome 20 open reading frame 24	0.99423	1.066804	1.0410629

55970	GNG12	guanine nucleotide binding protein (G protein), gamma 12	1.0242	1.023592	0.985076
55971	BAIAP2L1	BAI1-associated protein 2-like 1	1.00811	0.987624	1.0071667
55972	SLC25A40	solute carrier family 25, member 40	0.99702	0.984855	1.0641663
55973	BCAP29	B-cell receptor-associated protein 29	1.01254	0.984783	1.0116035
55974	SLC50A1	solute carrier family 50 (sugar transporter), member 1	0.96702	0.928577	1.0898674
55975	KLHL7	kelch-like 7 (Drosophila)	1.01336	0.986178	1.0243924
55998	NXF5	nuclear RNA export factor 5	1.02065	1.023441	1.0243689
55999	NXF4	nuclear RNA export factor 4 pseudogene	0.99764	1.013675	0.9736405
56000	NXF3	nuclear RNA export factor 3	0.99794	0.98469	0.9937684
56005	C19orf10	chromosome 19 open reading frame 10	0.98016	1.002465	1.0699304
56006	C19orf61	chromosome 19 open reading frame 61	0.95182	0.990103	0.9753309
56033	BARX1	BARX homeobox 1	0.98437	1.036601	1.0816198
56034	PDGFC	platelet derived growth factor C	1.00397	0.984581	1.0252141
56052	ALG1	asparagine-linked glycosylation 1, beta-1,4-mannosyltransferase homolog (S. cerevisiae)	0.95148	0.906399	1.0793341
56061	UBFD1	ubiquitin family domain containing 1	0.98169	0.970854	1.0723336
56062	KLHL4	kelch-like 4 (Drosophila)	1.00226	1.011433	1.0267988
56063	TMEM234	transmembrane protein 234	0.99891	0.989048	0.980314
56114	PCDHGA1	protocadherin gamma subfamily A, 1	1.00782	1.018367	0.9921705
56121	PCDHB15	protocadherin beta 15	0.99157	1.052241	1.0574096
56122	PCDHB14	protocadherin beta 14	0.98799	1.00932	1.0064987
56123	PCDHB13	protocadherin beta 13	0.97098	0.978393	1.0068905
56124	PCDHB12	protocadherin beta 12	0.99907	1.026473	1.0061839
56125	PCDHB11	protocadherin beta 11	0.98633	1.053209	0.9942176
56126	PCDHB10	protocadherin beta 10	1.00727	0.992484	1.0230651
56127	PCDHB9	protocadherin beta 9	0.99209	1.029619	1.0169419
56128	PCDHB8	protocadherin beta 8	0.97899	0.980156	1.0200904
56129	PCDHB7	protocadherin beta 7	1.02892	1.02099	0.9553088
56130	PCDHB6	protocadherin beta 6	1.00612	0.943395	1.0092763
56131	PCDHB4	protocadherin beta 4	0.99123	0.967381	1.0343747
56132	PCDHB3	protocadherin beta 3	0.98117	0.970113	1.044544
56133	PCDHB2	protocadherin beta 2	0.98702	1.000854	0.9788814
56154	TEX15	testis expressed 15	1.0069	0.999196	1.0271774
56155	TEX14	testis expressed 14	0.98286	1.033802	0.9631343

56156	TEX13B	testis expressed 13B	1.00842	1.001128	1.0061978
56157	TEX13A	testis expressed 13A	0.98759	0.9888	1.007132
56158	TEX12	testis expressed 12	0.98207	0.966229	0.9929106
56159	TEX11	testis expressed 11	1.00193	1.015484	1.0074254
56160	NDNL2	necdin-like 2	0.91429	0.932343	0.991904
56163	RNF17	ring finger protein 17	0.97965	1.00192	0.99731
56164	STK31	serine/threonine kinase 31	1.0025	0.992789	1.0030395
56165	TDRD1	tudor domain containing 1	0.99529	1.013235	1.0113047
56169	GSDMC	gasdermin C	0.9937	0.980989	0.9891279
56171	DNAH7	dynein, axonemal, heavy chain 7	1.00627	1.005858	1.0121504
56172	ANKH	ankylosis, progressive homolog (mouse)	0.97764	0.967296	0.9275362
56180	MOSPD1	motile sperm domain containing 1	0.94457	0.952864	1.0262398
56181	FAM54B	family with sequence similarity 54, member B	0.99423	0.971094	1.0462287
56203	LMOD3	leiomodin 3 (fetal)	1.01508	1.012429	1.0255017
56204	KIAA1370	KIAA1370	0.93134	0.952703	0.9916846
56241	SUSD2	sushi domain containing 2	1.00513	1.006928	1.0118216
56242	ZNF253	zinc finger protein 253	0.90875	1.000233	0.991738
56243	KIAA1217	KIAA1217	1.00372	1.001989	1.0042227
56244	BTNL2	butyrophilin-like 2 (MHC class II associated)	1.03636	1.085465	0.9950351
56245	C21orf62	chromosome 21 open reading frame 62	1.00936	0.993611	0.9941626
56246	MRAP	melanocortin 2 receptor accessory protein	1.00323	1.045323	1.0086505
56252	YLPM1	YLP motif containing 1	0.9671	0.978808	0.9182769
56253	CRTAM	cytotoxic and regulatory T cell molecule	0.95438	1.134212	1.0013542
56254	RNF20	ring finger protein 20	0.93858	0.941096	1.0351388
56255	TMX4	thioredoxin-related transmembrane protein 4	0.93993	0.880164	1.029712
56256	SERTAD4	SERTA domain containing 4	1.02745	1.047946	0.9937715
56257	MEPCE	methylphosphate capping enzyme	0.93602	0.939341	0.9726689
56259	CTNNBL1	catenin, beta like 1	0.9877	0.963759	0.9777651
56260	C8orf44	chromosome 8 open reading frame 44	1.01427	0.956041	0.9347486
56261	GPCPD1	glycerophosphocholine phosphodiesterase GDE1 homolog (S. cerevisiae)	1.01994	1.04581	0.9075865
56262	LRRC8A	leucine rich repeat containing 8 family, member A	0.94963	0.956408	1.0384175
56265	CPXM1	carboxypeptidase X (M14 family), member 1	0.98376	0.981887	1.0068348
56267	CCBL2	cysteine conjugate-beta lyase 2	1.00184	1.009398	1.0845439

56269	IRGC	immunity-related GTPase family, cinema	0.99195	0.991809	0.9689135
56270	WDR45L	WDR45-like	0.98993	1.005567	0.9780887
56271	BEX4	brain expressed, X-linked 4	0.94063	0.924585	0.9166586
56287	GKN1	gastrokine 1	1.0025	1.001547	1.0318379
56288	PARD3	par-3 partitioning defective 3 homolog (C. elegans)	0.98983	0.969297	0.9985434
56300	IL36G	interleukin 36, gamma	1.00896	1.017934	1.0333686
56301	SLC7A10	solute carrier family 7, (neutral amino acid transporter, y+ system) member 10	0.99257	0.982948	1.0113462
56302	TRPV5	transient receptor potential cation channel, subfamily V, member 5	1.02321	0.989786	1.0406614
56311	ANKRD7	ankyrin repeat domain 7	1.00995	0.962276	1.0371815
56339	METTL3	methyltransferase like 3	0.96263	1.049524	1.0741559
56341	PRMT8	protein arginine methyltransferase 8	0.99758	0.989669	1.0217696
56413	LTB4R2	leukotriene B4 receptor 2	0.99151	1.002542	0.9710442
56474	CTPS2	CTP synthase II	0.96717	0.966549	0.9858602
56475	RPRM	reprimo, TP53 dependent G2 arrest mediator candidate	0.97437	1.002546	1.029033
56477	CCL28	chemokine (C-C motif) ligand 28	0.97156	0.967897	1.0094828
56478	EIF4ENIF1	eukaryotic translation initiation factor 4E nuclear import factor 1	0.96487	0.952307	1.026679
56479	KCNQ5	potassium voltage-gated channel, KQT-like subfamily, member 5	0.98731	0.983864	1.0450592
56521	DNAJC12	DnaJ (Hsp40) homolog, subfamily C, member 12	1.01214	1.015207	1.0287612
56547	MMP26	matrix metallopeptidase 26	0.98618	0.964591	0.9761747
56548	CHST7	carbohydrate (N-acetylglucosamine 6-O) sulfotransferase 7	0.97106	1.090995	0.9899984
56603	CYP26B1	cytochrome P450, family 26, subfamily B, polypeptide 1	0.98926	1.011159	1.0310179
56604	TUBB4Q	tubulin, beta polypeptide 4, member Q, pseudogene	1.01651	1.03125	0.9377965
56605	ERO1LB	ERO1-like beta (S. cerevisiae)	0.97773	0.987226	0.9767659
56606	SLC2A9	solute carrier family 2 (facilitated glucose transporter), member 9	1.00236	0.984554	1.005988
56616	DIABLO	diablo, IAP-binding mitochondrial protein	0.94414	0.996341	0.943734
56623	INPP5E	inositol polyphosphate-5-phosphatase, 72 kDa	0.94711	0.99329	1.0186446
56624	ASAH2	N-acylsphingosine amidohydrolase (non-lysosomal ceramidase) 2	1.03336	1.042495	0.9658485
56647	BCCIP	BRCA2 and CDKN1A interacting protein	0.95356	0.90474	1.0227369
56648	EIF5A2	eukaryotic translation initiation factor 5A2	0.98906	0.945261	0.9352448
56649	TMPRSS4	transmembrane protease, serine 4	0.99739	1.021125	0.9874704
56650	CLDND1	claudin domain containing 1	1.00319	0.964492	1.0760515
56652	C10orf2	chromosome 10 open reading frame 2	0.92057	0.9663	1.0159131
56654	NPDC1	neural proliferation, differentiation and control, 1	0.98958	1.02553	0.974728

56656	OR2S2	olfactory receptor, family 2, subfamily S, member 2	0.98849	0.985198	1.0594762
56658	TRIM39	tripartite motif containing 39	0.98236	0.983408	0.9135348
56659	KCNK13	potassium channel, subfamily K, member 13	0.99982	1.012145	1.0287516
56660	KCNK12	potassium channel, subfamily K, member 12	1.00532	0.982016	0.9905761
56666	PANX2	pannexin 2	1.01765	0.991678	0.9555867
56667	MUC13	mucin 13, cell surface associated	1.00143	0.998194	1.0033917
56670	SUCNR1	succinate receptor 1	0.95229	0.947825	1.055473
56672	AKIP1	A kinase (PRKA) interacting protein 1	0.94328	1.013362	1.0932006
56674	TMEM9B	TMEM9 domain family, member B	0.95118	0.916867	1.0409102
56675	NRIP3	nuclear receptor interacting protein 3	0.98591	0.970755	0.932623
56676	ASCL3	achaete-scute complex homolog 3 (Drosophila)	0.99165	1.005067	1.0210576
56677	FABP3P2	fatty acid binding protein 3, pseudogene 2	1.0259	1.052394	0.9275297
56681	SAR1A	SAR1 homolog A (S. cerevisiae)	0.95971	0.969007	0.9853425
56683	C21orf59	chromosome 21 open reading frame 59	0.94222	0.956751	0.9908423
56704	JPH1	junctophilin 1	0.99583	1.001117	0.9963415
56729	RETN	resistin	1.00627	0.969035	0.9687636
56731	SLC2A4RG	SLC2A4 regulator	0.97288	1.003248	1.0070994
56751	BARHL1	BarH-like homeobox 1	0.98632	0.996024	1.0816552
56776	FMN2	formin 2	1.00365	1.002179	1.0206073
56829	ZC3HAV1	zinc finger CCCH-type, antiviral 1	1.00179	0.959465	0.9522587
56832	IFNK	interferon, kappa	1.00472	1.014444	1.0087847
56833	SLAMF8	SLAM family member 8	1.0406	0.976442	1.0314003
56834	GPR137	G protein-coupled receptor 137	0.9831	1.008789	1.055838
56848	SPHK2	sphingosine kinase 2	0.97764	0.983896	1.033612
56849	TCEAL7	transcription elongation factor A (SII)-like 7	1.03996	1.053314	0.9913427
56850	GRIPAP1	GRIP1 associated protein 1	0.95232	1.094064	0.9564865
56851	C15orf24	chromosome 15 open reading frame 24	0.91861	0.935408	1.0559211
56852	RAD18	RAD18 homolog (S. cerevisiae)	0.99663	0.995401	1.0451968
56853	CELF4	CUGBP, Elav-like family member 4	1.00527	0.985808	1.0018239
56882	CDC42SE1	CDC42 small effector 1	0.98839	0.997219	0.9339565
56884	FSTL5	follistatin-like 5	1.01125	1.017223	1.0251488
56886	UGGT1	UDP-glucose glycoprotein glucosyltransferase 1	1.00501	0.980695	1.0269412
56888	KCMF1	potassium channel modulatory factor 1	1.00367	0.948079	0.9338342

56889	TM9SF3	transmembrane 9 superfamily member 3	0.9772	0.910595	1.0017747
56890	MDM1	Mdm1 nuclear protein homolog (mouse)	0.93494	0.879709	1.0277278
56891	LGALS14	lectin, galactoside-binding, soluble, 14	0.98549	1.03216	1.0523623
56892	C8orf4	chromosome 8 open reading frame 4	0.99606	1.026112	0.9796112
56893	UBQLN4	ubiquilin 4	0.9933	0.98336	1.0090448
56894	AGPAT3	1-acylglycerol-3-phosphate O-acyltransferase 3	0.96585	0.950545	1.0644149
56895	AGPAT4	1-acylglycerol-3-phosphate O-acyltransferase 4 (lysophosphatidic acid acyltransferase, delta)	1.03017	1.042131	0.9675217
56896	DPYSL5	dihydropyrimidinase-like 5	1.0047	0.984504	1.0148253
56897	WRNIP1	Werner helicase interacting protein 1	0.98812	1.029854	1.0534871
56898	BDH2	3-hydroxybutyrate dehydrogenase, type 2	0.97374	0.963062	1.0394432
56899	ANKS1B	ankyrin repeat and sterile alpha motif domain containing 1B	0.98919	1.00393	1.012097
56900	TMEM167E	transmembrane protein 167B	1.00863	0.933243	1.0059701
56901	NDUFA4L2	NADH dehydrogenase (ubiquinone) 1 alpha subcomplex, 4-like 2	0.98584	1.016058	1.0366916
56902	PNO1	partner of NOB1 homolog (S. cerevisiae)	0.94578	0.916744	1.0628722
56904	SH3GLB2	SH3-domain GRB2-like endophilin B2	0.96537	0.951612	1.0225993
56905	C15orf39	chromosome 15 open reading frame 39	0.9761	1.046295	1.0180191
56906	THAP10	THAP domain containing 10	0.98222	1.077053	1.0488002
56907	SPIRE1	spire homolog 1 (Drosophila)	0.99138	0.99342	0.972852
56910	STARD7	StAR-related lipid transfer (START) domain containing 7	0.98912	1.044299	1.0431741
56911	C21orf7	chromosome 21 open reading frame 7	0.96825	0.993669	0.9657713
56912	IFT46	intraflagellar transport 46 homolog (Chlamydomonas)	0.90425	0.962522	1.0188007
56913	C1GALT1	core 1 synthase, glycoprotein-N-acetylgalactosamine 3-beta-galactosyltransferase, 1	0.97832	0.921898	1.0421188
56914	OTOR	otoraplin	1.03295	1.003731	1.0017909
56915	EXOSC5	exosome component 5	0.93986	0.930459	1.0134212
56916	SMARCAD1	SWI/SNF-related, matrix-associated actin-dependent regulator of chromatin, subfamily a, containing DEAD/H	0.97723	0.916137	1.0545182
56917	MEIS3	Meis homeobox 3	1.00059	0.974998	1.0053554
56918	C2orf83	chromosome 2 open reading frame 83	0.985	1.013234	1.0136836
56919	DHX33	DEAH (Asp-Glu-Ala-His) box polypeptide 33	0.92931	0.951449	0.974152
56920	SEMA3G	sema domain, immunoglobulin domain (Ig), short basic domain, secreted, (semaphorin) 3G	1.01339	1.032191	0.9922249
56922	MCCC1	methylcrotonoyl-CoA carboxylase 1 (alpha)	0.99951	1.050874	1.0392581
56923	NMUR2	neuromedin U receptor 2	1.02897	0.979728	1.0035146
56924	PAK6	p21 protein (Cdc42/Rac)-activated kinase 6	1.00157	1.007574	1.011738
56925	LXN	latexin	0.96828	0.954439	1.0408599

56926	NCLN	nicalin	0.95171	0.929477	1.0439227
56927	GPR108	G protein-coupled receptor 108	0.94816	1.02737	1.0634525
56928	SPPL2B	signal peptide peptidase-like 2B	0.96858	0.991756	1.0027637
56929	FEM1C	fem-1 homolog c (C. elegans)	0.98906	1.007762	0.9901375
56931	DUS3L	dihydrouridine synthase 3-like (S. cerevisiae)	0.96022	0.973987	1.0455206
56934	CA10	carbonic anhydrase X	0.99362	0.983644	0.987055
56935	C11orf75	chromosome 11 open reading frame 75	0.97247	1.09135	0.9962975
56937	PMEPA1	prostate transmembrane protein, androgen induced 1	0.98111	1.056781	0.9877529
56938	ARNTL2	aryl hydrocarbon receptor nuclear translocator-like 2	0.98581	1.008707	1.0193476
56940	DUSP22	dual specificity phosphatase 22	0.98967	0.985697	1.0447201
56941	C3orf37	chromosome 3 open reading frame 37	0.97688	0.951165	1.011849
56942	C16orf61	chromosome 16 open reading frame 61	0.98633	0.962624	0.9753998
56943	ENY2	enhancer of yellow 2 homolog (Drosophila)	0.99505	0.935447	1.0131921
56944	OLFML3	olfactomedin-like 3	0.99948	0.99724	0.9905934
56945	MRPS22	mitochondrial ribosomal protein S22	0.95546	0.988632	1.1104212
56946	C11orf30	chromosome 11 open reading frame 30	0.91044	0.959468	0.9957487
56947	MFF	mitochondrial fission factor	0.99562	0.959605	1.0586262
56948	SDR39U1	short chain dehydrogenase/reductase family 39U, member 1	0.92187	0.909642	1.0012926
56949	XAB2	XPA binding protein 2	0.94636	1.028749	0.9890909
56950	SMYD2	SET and MYND domain containing 2	0.96423	0.872644	1.0145294
56951	C5orf15	chromosome 5 open reading frame 15	0.99057	0.960733	1.0508738
56952	PRTFDC1	phosphoribosyl transferase domain containing 1	0.95425	0.959682	1.0072167
56953	NT5M	5',3'-nucleotidase, mitochondrial	0.98253	0.997655	1.0458479
56954	NIT2	nitrilase family, member 2	0.98796	1.028993	1.0445202
56955	MEPE	matrix extracellular phosphoglycoprotein	1	1.028566	1.0187621
56956	LHX9	LIM homeobox 9	0.99839	0.984439	1.0398124
56957	OTUD7B	OTU domain containing 7B	0.98982	1.017674	1.0452546
56959	[No Symbo	[No Name]	0.95586	0.947937	1.0437327
56961	SHD	Src homology 2 domain containing transforming protein D	0.99158	0.992411	0.9720182
56963	RGMA	RGM domain family, member A	1.00423	0.970578	1.0145973
56964	WDR93	WD repeat domain 93	1.00376	1.003735	0.9764098
56965	PARP6	poly (ADP-ribose) polymerase family, member 6	0.97693	1.034137	0.9383906
56967	C14orf132	chromosome 14 open reading frame 132	1.00075	1.008016	1.0067774

56970	ATXN7L3	ataxin 7-like 3	0.90131	0.977747	0.9428662
56971	CEACAM19	carcinoembryonic antigen-related cell adhesion molecule 19	1.01226	0.989724	0.9821712
56975	FAM20C	family with sequence similarity 20, member C	0.99841	1.035936	0.9541851
56977	STOX2	storkhead box 2	0.99829	0.985057	1.0236096
56978	PRDM8	PR domain containing 8	1.00008	1.022882	0.9972097
56979	PRDM9	PR domain containing 9	1.02814	0.964102	1.0105625
56980	PRDM10	PR domain containing 10	0.95802	1.017679	0.9927619
56981	PRDM11	PR domain containing 11	0.99939	0.992001	0.9956328
56983	POGLUT1	protein O-glucosyltransferase 1	0.9673	1.015598	1.0711272
56984	PSMG2	proteasome (prosome, macropain) assembly chaperone 2	0.95454	1.023803	1.0665856
56985	C17orf48	chromosome 17 open reading frame 48	0.91821	0.815157	0.8589974
56986	DTWD1	DTW domain containing 1	0.98186	0.973552	1.0641367
56987	BBX	bobby sox homolog (Drosophila)	0.98401	0.899406	0.9299731
56990	CDC42SE2	CDC42 small effector 2	1.03149	1.076108	0.9457423
56992	KIF15	kinesin family member 15	0.99117	0.971042	1.0253939
56993	TOMM22	translocase of outer mitochondrial membrane 22 homolog (yeast)	0.94659	0.962627	1.113825
56994	CHPT1	choline phosphotransferase 1	0.98614	0.971785	0.9687975
56995	TULP4	tubby like protein 4	0.99709	0.926922	0.9860189
56996	SLC12A9	solute carrier family 12 (potassium/chloride transporters), member 9	1.0088	0.925203	1.086269
56997	ADCK3	aarF domain containing kinase 3	0.98645	0.993277	1.0133605
56998	CTNNBIP1	catenin, beta interacting protein 1	1.0208	0.988269	0.9910856
56999	ADAMTS9	ADAM metallopeptidase with thrombospondin type 1 motif, 9	1.00748	1.006223	1.0135548
57001	ACN9	ACN9 homolog (S. cerevisiae)	0.99087	1.012439	1.0012495
57002	C7orf36	chromosome 7 open reading frame 36	0.93683	0.948341	0.9932793
57003	CCDC47	coiled-coil domain containing 47	0.95856	1.053978	1.1120576
57007	CXCR7	chemokine (C-X-C motif) receptor 7	0.99253	0.991953	0.9958312
57010	CABP4	calcium binding protein 4	0.99103	1.031014	0.9860643
57016	AKR1B10	aldo-keto reductase family 1, member B10 (aldose reductase)	1.01381	0.995995	1.0280544
57017	COQ9	coenzyme Q9 homolog (S. cerevisiae)	0.96652	0.991805	0.9827557
57018	CCNL1	cyclin L1	1.0201	0.963442	1.0099504
57019	CIAPIN1	cytokine induced apoptosis inhibitor 1	1.01804	1.04217	1.0318662
57020	C16orf62	chromosome 16 open reading frame 62	0.9536	0.948158	1.0089003
57026	PDXP	pyridoxal (pyridoxine, vitamin B6) phosphatase	0.98805	0.947258	1.2345481

57030	SLC17A7	solute carrier family 17 (sodium-dependent inorganic phosphate cotransporter), member 7	0.99329	1.001482	1.0589283
57035	C1orf63	chromosome 1 open reading frame 63	1.00923	0.931227	0.8878751
57037	ANKMY2	ankyrin repeat and MYND domain containing 2	0.98994	1.02306	0.974123
57038	RARS2	arginyl-tRNA synthetase 2, mitochondrial	0.97295	0.91673	1.0263073
57045	TWSG1	twisted gastrulation homolog 1 (Drosophila)	0.96363	1.05552	0.9915009
57047	PLSCR2	phospholipid scramblase 2	1.01655	0.994193	0.9948063
57048	PLSCR3	phospholipid scramblase 3	0.95632	0.933284	0.9985548
57050	UTP3	UTP3, small subunit (SSU) processome component, homolog (S. cerevisiae)	0.96626	0.906538	1.1054534
57052	[No Symbo	[No Name]	1.00854	1.009641	1.0472571
57053	CHRNA10	cholinergic receptor, nicotinic, alpha 10	0.99957	0.981622	0.9969845
57057	TBX20	T-box 20	1.00978	1.016588	1.0074858
57060	PCBP4	poly(rC) binding protein 4	1.00172	0.983193	1.0276794
57062	DDX24	DEAD (Asp-Glu-Ala-Asp) box polypeptide 24	0.94193	0.987243	0.9119237
57082	CASC5	cancer susceptibility candidate 5	0.95771	0.942752	1.0361025
57084	SLC17A6	solute carrier family 17 (sodium-dependent inorganic phosphate cotransporter), member 6	1.00813	1.004451	0.985432
57085	AGTRAP	angiotensin II receptor-associated protein	1.00924	0.975461	0.9385876
57088	PLSCR4	phospholipid scramblase 4	0.99127	0.993116	0.9952135
57089	ENTPD7	ectonucleoside triphosphate diphosphohydrolase 7	0.97606	0.985458	1.050761
57091	CASS4	Cas scaffolding protein family member 4	0.94649	0.939106	0.9761617
57092	PCNP	PEST proteolytic signal containing nuclear protein	1.00709	0.940524	0.9618879
57094	CPA6	carboxypeptidase A6	1.00503	0.997849	0.991947
57095	PITHD1	PITH (C-terminal proteasome-interacting domain of thioredoxin-like) domain containing 1	0.96646	1.006193	0.9877824
57096	RPGRIP1	retinitis pigmentosa GTPase regulator interacting protein 1	0.98754	0.980517	1.0151708
57097	PARP11	poly (ADP-ribose) polymerase family, member 11	0.92406	0.961227	0.907999
57099	AVEN	apoptosis, caspase activation inhibitor	0.98686	1.002065	1.0139899
57101	ANO2	anoctamin 2	0.9791	0.985909	0.9851821
57102	C12orf4	chromosome 12 open reading frame 4	0.97542	0.983325	1.0958264
57103	C12orf5	chromosome 12 open reading frame 5	0.97904	0.93966	1.1501177
57104	PNPLA2	patatin-like phospholipase domain containing 2	0.96588	0.960657	0.9715088
57105	CYSLTR2	cysteinyl leukotriene receptor 2	0.9835	0.978915	0.9003401
57106	NAT14	N-acetyltransferase 14 (GCN5-related, putative)	1.0017	1.040362	0.9963656
57107	PDSS2	prenyl (decaprenyl) diphosphate synthase, subunit 2	0.99763	0.981456	1.0058759
57109	REXO4	REX4, RNA exonuclease 4 homolog (S. cerevisiae)	0.91869	1.019048	1.0475058

57110	HRASLS	HRAS-like suppressor	0.94684	1.036078	1.0221534
57111	RAB25	RAB25, member RAS oncogene family	1.02585	1.034386	1.0233071
57113	TRPC7	transient receptor potential cation channel, subfamily C, member 7	1.02146	0.991872	1.0094667
57115	PGLYRP4	peptidoglycan recognition protein 4	1.02563	0.970213	1.0565659
57116	ZNF695	zinc finger protein 695	0.99822	0.98922	1.0298706
57117	INTS12	integrator complex subunit 12	1.00328	0.923564	1.0539118
57118	CAMK1D	calcium/calmodulin-dependent protein kinase ID	1.00071	0.958363	0.9874179
57119	SPINLW1	serine peptidase inhibitor-like, with Kunitz and WAP domains 1 (eppin)	1.00099	0.984468	0.9638638
57120	GOPC	golgi-associated PDZ and coiled-coil motif containing	0.98065	0.908924	0.950175
57121	LPAR5	lysophosphatidic acid receptor 5	0.94268	0.983052	0.9574686
57122	NUP107	nucleoporin 107kDa	0.94688	1.030314	1.0739836
57124	CD248	CD248 molecule, endosialin	0.9595	1.059027	1.0615885
57125	PLXDC1	plexin domain containing 1	0.98087	0.966699	0.9751159
57126	CD177	CD177 molecule	0.9744	1.002377	1.0245562
57127	RHBG	Rh family, B glycoprotein (gene/pseudogene)	0.99836	1.005555	1.0133286
57128	LYRM4	LYR motif containing 4	0.98539	0.953521	0.9890557
57129	MRPL47	mitochondrial ribosomal protein L47	0.93827	0.950387	1.0842334
57130	ATP13A1	ATPase type 13A1	0.97491	1.003019	0.9672883
57134	MAN1C1	mannosidase, alpha, class 1C, member 1	0.97449	0.936033	0.8963696
57136	C20orf3	chromosome 20 open reading frame 3	0.93683	1.074608	0.9503815
57139	RGL3	ral guanine nucleotide dissociation stimulator-like 3	0.99861	1.026235	0.9927704
57140	RNPEPL1	arginyl aminopeptidase (aminopeptidase B)-like 1	0.97391	0.902308	1.0322668
57142	RTN4	reticulon 4	1.02082	0.970427	0.9863336
57143	ADCK1	aarF domain containing kinase 1	0.99228	0.970323	1.0267827
57144	PAK7	p21 protein (Cdc42/Rac)-activated kinase 7	0.99866	0.985717	0.9909222
57146	TMEM159	transmembrane protein 159	0.94853	0.961286	1.0245752
57147	SCYL3	SCY1-like 3 (S. cerevisiae)	0.97113	1.008017	0.9718907
57148	RALGAPB	Ral GTPase activating protein, beta subunit (non-catalytic)	0.95381	1.064395	1.0150939
57149	LYRM1	LYR motif containing 1	0.91936	1.017733	1.006275
57150	C6orf162	chromosome 6 open reading frame 162	0.96093	0.912647	1.1089364
57151	LYZL6	lysozyme-like 6	0.98863	1.00339	0.9755844
57152	SLURP1	secreted LY6/PLAUR domain containing 1	0.9981	1.057462	1.0804598
57153	SLC44A2	solute carrier family 44, member 2	0.98569	0.975922	1.1346241

57154	SMURF1	SMAD specific E3 ubiquitin protein ligase 1	0.99271	0.956801	0.9723703
57156	TMEM63C	transmembrane protein 63C	0.99475	1.017059	1.0053328
57157	PHTF2	putative homeodomain transcription factor 2	0.99461	0.976448	0.9854525
57158	JPH2	junctophilin 2	0.9921	1.002301	1.0259408
57159	TRIM54	tripartite motif containing 54	1.01331	1.007132	1.0131756
57161	PELI2	pellino homolog 2 (Drosophila)	0.98184	0.988218	1.0210437
57162	PELI1	pellino homolog 1 (Drosophila)	1.0493	0.953882	0.9327013
57165	GJC2	gap junction protein, gamma 2, 47kDa	1.00311	1.012284	1.0703047
57167	SALL4	sal-like 4 (Drosophila)	1.00271	0.991662	0.9867269
57168	ASPHD2	aspartate beta-hydroxylase domain containing 2	0.97843	0.881083	1.0896918
57169	ZNFX1	zinc finger, NFX1-type containing 1	0.93829	1.039524	0.9368156
57171	DOLPP1	dolichyl pyrophosphate phosphatase 1	0.9473	0.971397	1.0376137
57172	CAMK1G	calcium/calmodulin-dependent protein kinase IG	1.01007	1.003306	0.9852468
57175	CORO1B	coronin, actin binding protein, 1B	0.97586	0.897833	1.0805795
57176	VARS2	valyl-tRNA synthetase 2, mitochondrial (putative)	0.97986	0.983155	1.0208676
57178	ZMIZ1	zinc finger, MIZ-type containing 1	0.98303	1.003509	0.9655619
57179	KIAA1191	KIAA1191	1.01284	0.932438	1.0432099
57180	ACTR3B	ARP3 actin-related protein 3 homolog B (yeast)	0.98507	0.990706	1.0164436
57181	SLC39A10	solute carrier family 39 (zinc transporter), member 10	0.96817	0.980529	0.9563263
57182	ANKRD50	ankyrin repeat domain 50	1.05079	0.971799	1.096992
57184	C15orf17	chromosome 15 open reading frame 17	0.94681	0.942319	1.0838976
57185	NIPAL3	NIPA-like domain containing 3	0.96216	0.984369	0.8958995
57186	RALGAPA2	Ral GTPase activating protein, alpha subunit 2 (catalytic)	0.9589	0.945518	1.043311
57187	THOC2	THO complex 2	0.94651	1.009711	1.0677823
57188	ADAMTSL3	ADAMTS-like 3	0.99104	0.989435	0.9977148
57189	KIAA1147	KIAA1147	0.98547	0.919117	0.9710178
57191	VN1R1	vomeronasal 1 receptor 1	0.93696	0.942122	0.9108539
57192	MCOLN1	mucolipin 1	0.99708	1.05458	0.9813191
57194	ATP10A	ATPase, class V, type 10A	0.94087	0.995341	0.9336182
57198	ATP8B2	ATPase, class I, type 8B, member 2	0.95974	0.934022	1.0276247
57205	ATP10D	ATPase, class V, type 10D	1.00413	1.056504	1.1242524
57209	ZNF248	zinc finger protein 248	0.93556	0.981326	1.0486957
57210	SLC45A4	solute carrier family 45, member 4	0.98021	1.024421	0.9727897

57211	GPR126	G protein-coupled receptor 126	1.01028	1.006491	0.9945691
57212	TP73-AS1	TP73 antisense RNA 1 (non-protein coding)	0.98881	0.980902	0.9760221
57213	SPRYD7	SPRY domain containing 7	0.96165	1.006899	1.0061532
57214	KIAA1199	KIAA1199	0.99769	0.997423	0.9989766
57215	THAP11	THAP domain containing 11	0.92052	0.857791	1.0400653
57216	VANGL2	vang-like 2 (van gogh, Drosophila)	1.0169	1.008315	1.0356863
57217	TTC7A	tetratricopeptide repeat domain 7A	0.99642	0.957871	0.9824853
57221	KIAA1244	KIAA1244	1.0096	0.999421	1.0024153
57222	ERGIC1	endoplasmic reticulum-golgi intermediate compartment (ERGIC) 1	1.00722	1.039665	0.9498178
57223	SMEK2	SMEK homolog 2, suppressor of mek1 (Dictyostelium)	1.02147	0.978501	0.9780035
57224	NHSL1	NHS-like 1	1.00286	1.00397	1.0298141
57226	LYRM2	LYR motif containing 2	0.97988	0.995577	1.0128528
57228	SMAGP	small cell adhesion glycoprotein	0.95262	1.010366	0.9700145
57231	SNX14	sorting nexin 14	0.96607	0.961787	1.0561476
57232	ZNF630	zinc finger protein 630	0.99516	0.948419	0.9681922
57238	[No Symbo	[No Name]	0.97354	0.966942	0.9783898
57282	SLC4A10	solute carrier family 4, sodium bicarbonate transporter, member 10	0.96581	0.993317	0.9583401
57325	CSRP2BP	CSRP2 binding protein	0.94448	0.964538	1.044539
57326	PBXIP1	pre-B-cell leukemia homeobox interacting protein 1	0.96334	1.037351	0.9871348
57332	CBX8	chromobox homolog 8	0.97958	1.039478	1.0453767
57333	RCN3	reticulocalbin 3, EF-hand calcium binding domain	0.99804	1.008535	0.9435355
57335	ZNF286A	zinc finger protein 286A	0.95163	0.896154	1.088636
57336	ZNF287	zinc finger protein 287	0.94827	0.999589	1.0267047
57337	SENP7	SUMO1/sentrin specific peptidase 7	0.97152	0.969196	0.9037172
57338	JPH3	junctophilin 3	0.99748	0.989984	1.0194846
57343	ZNF304	zinc finger protein 304	0.87382	1.032323	1.0206076
57348	TTYH1	tweety homolog 1 (Drosophila)	0.98861	1.026481	1.0101619
57369	GJD2	gap junction protein, delta 2, 36kDa	0.94803	0.994429	0.9969004
57379	AICDA	activation-induced cytidine deaminase	0.97109	1.042146	1.0089089
57380	MRS2	MRS2 magnesium homeostasis factor homolog (S. cerevisiae)	0.9914	0.997926	1.0263147
57381	RHOJ	ras homolog gene family, member J	0.99472	1.006057	1.0037362
57393	TMEM27	transmembrane protein 27	0.99897	1.038045	1.0214769
57396	CLK4	CDC-like kinase 4	0.97122	0.960377	0.8990593

57402	S100A14	S100 calcium binding protein A14	1.01414	1.023976	0.9701804
57403	RAB22A	RAB22A, member RAS oncogene family	0.96294	0.987469	0.9707653
57404	CYP20A1	cytochrome P450, family 20, subfamily A, polypeptide 1	0.97433	0.935395	0.9918758
57405	SPC25	SPC25, NDC80 kinetochore complex component, homolog (S. cerevisiae)	0.98751	0.963005	1.0210168
57406	ABHD6	abhydrolase domain containing 6	1.0038	1.006672	0.9959001
57407	NMRAL1	NmrA-like family domain containing 1	0.9457	0.97056	1.0182153
57408	LRTM1	leucine-rich repeats and transmembrane domains 1	1.00683	1.004659	0.9673846
57409	MIF4GD	MIF4G domain containing	0.94284	0.914415	1.0332248
57410	SCYL1	SCY1-like 1 (S. cerevisiae)	0.93987	1.019679	1.0522313
57412	AS3MT	arsenic (+3 oxidation state) methyltransferase	0.97261	0.96664	1.0355737
57414	RHBDD2	rhomboid domain containing 2	0.98573	1.037827	0.9818012
57415	C3orf14	chromosome 3 open reading frame 14	0.98501	1.016062	1.0531779
57418	WDR18	WD repeat domain 18	0.93675	0.935052	1.105395
57419	SLC24A3	solute carrier family 24 (sodium/potassium/calcium exchanger), member 3	0.97855	0.972752	1.0044328
57446	NDRG3	NDRG family member 3	0.93373	0.949777	1.042265
57447	NDRG2	NDRG family member 2	0.94769	0.955608	0.9782286
57448	BIRC6	baculoviral IAP repeat containing 6	0.99155	1.010616	0.991032
57449	PLEKHG5	pleckstrin homology domain containing, family G (with RhoGef domain) member 5	1.0021	1.010871	1.0342108
57451	ODZ2	odz, odd Oz/ten-m homolog 2 (Drosophila)	1.00767	1.020423	1.0145709
57452	GALNTL1	UDP-N-acetyl-alpha-D-galactosamine:polypeptide N-acetylgalactosaminyltransferase-like 1	1.00386	0.982562	1.0017904
57453	DSCAML1	Down syndrome cell adhesion molecule like 1	1.00242	1.004964	1.0122057
57455	REXO1	REX1, RNA exonuclease 1 homolog (S. cerevisiae)	0.98993	0.975644	1.0029615
57456	KIAA1143	KIAA1143	1.03011	0.93025	0.9606334
57459	GATAD2B	GATA zinc finger domain containing 2B	0.98437	1.04889	0.9360529
57460	PPM1H	protein phosphatase, Mg2+/Mn2+ dependent, 1H	0.98512	0.982387	1.0026207
57461	ISY1	ISY1 splicing factor homolog (S. cerevisiae)	0.95167	0.964192	1.0594965
57462	KIAA1161	KIAA1161	1.00278	1.007166	1.0270951
57463	AMIGO1	adhesion molecule with Ig-like domain 1	0.98341	0.972322	0.9195761
57464	FAM40B	family with sequence similarity 40, member B	1.0108	0.963851	1.0015143
57465	TBC1D24	TBC1 domain family, member 24	1.00506	0.98405	1.0192993
57466	SCAF4	SR-related CTD-associated factor 4	0.9653	0.884576	1.041221
57467	HHATL	hedgehog acyltransferase-like	0.99778	1.010689	0.9847461
57468	SLC12A5	solute carrier family 12 (potassium/chloride transporter), member 5	1.00278	1.008776	0.9969901

57469	PNMAL2	PNMA-like 2	0.99635	0.973381	1.0495092
57470	LRRC47	leucine rich repeat containing 47	0.98411	0.933314	1.0773002
57471	ERMN	ermin, ERM-like protein	0.99403	1.03896	0.8235158
57472	CNOT6	CCR4-NOT transcription complex, subunit 6	0.97954	0.915147	0.9875051
57473	ZNF512B	zinc finger protein 512B	0.97249	0.981809	1.0577048
57474	ZNF490	zinc finger protein 490	0.90924	0.994147	1.1235412
57475	PLEKHH1	pleckstrin homology domain containing, family H (with MyTH4 domain) member 1	1.01018	0.976369	1.003028
57476	GRAMD1B	GRAM domain containing 1B	0.98021	0.970829	1.0180111
57477	SHROOM4	shroom family member 4	0.98942	0.99841	0.9923238
57478	USP31	ubiquitin specific peptidase 31	0.94457	0.963591	1.0266029
57479	PRR12	proline rich 12	0.95363	1.018951	0.9294984
57481	KIAA1210	KIAA1210	1.01166	0.993706	1.0082606
57482	KIAA1211	KIAA1211	1.00238	1.010141	1.0151132
57484	RNF150	ring finger protein 150	1.01123	0.995886	1.0086272
57486	NLN	neurolysin (metallopeptidase M3 family)	1.00423	0.941959	1.0246087
57488	ESYT2	extended synaptotagmin-like protein 2	0.95373	1.141686	0.9375075
57489	ODF2L	outer dense fiber of sperm tails 2-like	0.9709	0.95953	0.8923361
57491	AHRR	aryl-hydrocarbon receptor repressor	1.00518	1.014814	1.0117591
57492	ARID1B	AT rich interactive domain 1B (SWI1-like)	0.97053	0.84047	0.9871365
57493	HEG1	HEG homolog 1 (zebrafish)	0.99293	1.089449	1.0049739
57494	RIMKLB	ribosomal modification protein rimK-like family member B	0.97956	1.009381	1.0285381
57495	KIAA1239	KIAA1239	0.99835	0.993238	1.0072082
57496	MKL2	MKL/myocardin-like 2	0.97953	0.959792	1.0043912
57497	LRFN2	leucine rich repeat and fibronectin type III domain containing 2	1.01395	0.985439	0.9985678
57498	KIDINS220	kinase D-interacting substrate, 220kDa	1.0325	0.957952	1.0042848
57501	KIAA1257	KIAA1257	0.99494	1.016482	1.0117933
57502	NLGN4X	neuroligin 4, X-linked	1.00155	0.996	1.0111195
57504	MTA3	metastasis associated 1 family, member 3	1.00321	0.980264	1.013056
57505	AARS2	alanyl-tRNA synthetase 2, mitochondrial (putative)	0.98656	0.943199	0.9924412
57506	MAVS	mitochondrial antiviral signaling protein	0.94267	0.922866	1.0060973
57507	ZNF608	zinc finger protein 608	1.00339	0.988635	1.0142411
57508	INTS2	integrator complex subunit 2	0.92413	1.01153	1.043403
57509	MTUS1	microtubule associated tumor suppressor 1	0.98696	0.992086	0.9684015

57510	XPO5	exportin 5	0.98087	1.062279	0.9675907
57511	COG6	component of oligomeric golgi complex 6	0.94052	0.968276	1.0762842
57512	GPR158	G protein-coupled receptor 158	0.99107	1.015576	1.0119233
57513	CASKIN2	CASK interacting protein 2	0.9864	0.977147	1.0468957
57514	ARHGAP31	Rho GTPase activating protein 31	1.01761	1.017165	1.0040758
57515	SERINC1	serine incorporator 1	0.9875	0.978065	1.0140388
57519	STARD9	StAR-related lipid transfer (START) domain containing 9	0.98243	0.978468	1.025564
57520	HECW2	HECT, C2 and WW domain containing E3 ubiquitin protein ligase 2	1.01085	1.0062	1.0160991
57521	RPTOR	regulatory associated protein of MTOR, complex 1	0.97802	0.976065	0.9895718
57522	SRGAP1	SLIT-ROBO Rho GTPase activating protein 1	0.9922	1.015175	0.9949877
57523	NYNRIN	NYN domain and retroviral integrase containing	0.98804	1.009196	1.033749
57524	CASKIN1	CASK interacting protein 1	0.99411	1.027482	1.0301246
57526	PCDH19	protocadherin 19	0.99429	1.023315	0.9991841
57528	KCTD16	potassium channel tetramerisation domain containing 16	1.00957	0.994499	1.0200157
57529	RGAG1	retrotransposon gag domain containing 1	0.99121	0.980981	1.0379388
57530	CGN	cingulin	1.00587	0.975303	1.021671
57531	HACE1	HECT domain and ankyrin repeat containing, E3 ubiquitin protein ligase 1	0.99569	0.992331	1.0569657
57532	NUFIP2	nuclear fragile X mental retardation protein interacting protein 2	0.98113	0.896431	1.0088376
57533	TBC1D14	TBC1 domain family, member 14	1.00162	0.935543	1.0234942
57534	MIB1	mindbomb homolog 1 (Drosophila)	0.96373	0.981599	1.0342202
57535	KIAA1324	KIAA1324	0.97534	0.983712	0.9746262
57536	KIAA1328	KIAA1328	0.97333	0.97236	1.0154104
57537	SORCS2	sortilin-related VPS10 domain containing receptor 2	1.00519	1.014124	1.0139208
57538	ALPK3	alpha-kinase 3	0.99926	1.002751	1.0047495
57539	WDR35	WD repeat domain 35	0.98046	0.903933	1.1057549
57540	PTCHD2	patched domain containing 2	0.98659	0.99338	1.0204929
57541	ZNF398	zinc finger protein 398	0.99139	0.946775	1.0057695
57542	KLHDC5	kelch domain containing 5	0.98176	0.927757	0.9963505
57544	TXNDC16	thioredoxin domain containing 16	0.93539	1.039861	0.9563536
57545	CC2D2A	coiled-coil and C2 domain containing 2A	1.0022	1.016776	1.0135941
57546	PDP2	pyruvate dehyrogenase phosphatase catalytic subunit 2	0.94086	0.935494	1.0386172
57547	ZNF624	zinc finger protein 624	0.94062	1.000979	1.1046107
57549	IGSF9	immunoglobulin superfamily, member 9	0.9921	1.030945	1.0279746

57551	TAOK1	TAO kinase 1	0.9622	0.952705	0.9408389
57552	NCEH1	neutral cholesterol ester hydrolase 1	1.0116	1.00034	1.0061205
57553	MICAL3	microtubule associated monoxygenase, calponin and LIM domain containing 3	0.96733	1.018659	1.0112617
57554	LRRC7	leucine rich repeat containing 7	1.00388	1.012091	1.0008125
57555	NLGN2	neuroligin 2	0.99346	1.027957	1.0051157
57556	SEMA6A	sema domain, transmembrane domain (TM), and cytoplasmic domain, (semaphorin) 6A	1.0035	0.995703	1.0083116
57558	USP35	ubiquitin specific peptidase 35	1.01625	0.997552	1.0189296
57559	STAMBPL1	STAM binding protein-like 1	0.93173	0.958534	1.0208034
57560	IFT80	intraflagellar transport 80 homolog (Chlamydomonas)	0.96592	0.969262	0.9967119
57561	ARRDC3	arrestin domain containing 3	0.96819	0.802734	0.839022
57562	KIAA1377	KIAA1377	0.97117	0.963993	0.9507736
57563	KLHL8	kelch-like 8 (Drosophila)	1.00743	0.965547	1.1381215
57567	ZNF319	zinc finger protein 319	0.9464	0.993922	1.0021973
57568	SIPA1L2	signal-induced proliferation-associated 1 like 2	0.9964	0.963178	0.9959796
57569	ARHGAP20	Rho GTPase activating protein 20	0.99156	0.99321	1.0125875
57570	TRMT5	TRM5 tRNA methyltransferase 5 homolog (S. cerevisiae)	0.90885	0.970479	1.030546
57571	CARNS1	carnosine synthase 1	0.98244	1.035012	1.0025406
57572	DOCK6	dedicator of cytokinesis 6	0.99396	1.007764	1.0007827
57573	ZNF471	zinc finger protein 471	0.97965	1.024615	1.018916
57574	4-Mar	membrane-associated ring finger (C3HC4) 4	1.00101	1.01682	1.0191704
57575	PCDH10	protocadherin 10	1.0017	1.009421	1.0405077
57576	KIF17	kinesin family member 17	1.01026	0.998589	1.0107409
57577	KIAA1407	KIAA1407	1.00604	0.97351	1.0472295
57578	KIAA1409	KIAA1409	0.99235	0.994263	0.9930005
57579	FAM135A	family with sequence similarity 135, member A	1.0026	1.006201	1.0040731
57580	PREX1	phosphatidylinositol-3,4,5-trisphosphate-dependent Rac exchange factor 1	0.96345	1.001216	1.023172
57582	KCNT1	potassium channel, subfamily T, member 1	0.99356	0.991373	1.0464996
57583	TMEM181	transmembrane protein 181	0.98615	0.989372	0.9584864
57584	ARHGAP21	Rho GTPase activating protein 21	0.99052	0.987903	1.0294118
57585	CRAMP1L	Crm, cramped-like (Drosophila)	0.98237	1.006905	1.0049308
57586	SYT13	synaptotagmin XIII	0.99511	1.025783	1.0151109
57587	KIAA1430	KIAA1430	0.98387	0.909873	0.9828822
57589	KIAA1432	KIAA1432	0.98122	1.030448	1.1392072

57590	WDFY1	WD repeat and FYVE domain containing 1	0.99338	1.010561	1.0188992
57591	MKL1	megakaryoblastic leukemia (translocation) 1	0.97279	1.027728	1.025996
57592	ZNF687	zinc finger protein 687	1.01946	1.016331	0.995379
57593	EBF4	early B-cell factor 4	0.99353	0.99585	1.0145057
57594	HOMEZ	homeobox and leucine zipper encoding	0.94778	0.965497	0.9497423
57595	PDZD4	PDZ domain containing 4	0.99993	0.971222	0.9820525
57596	BEGAIN	brain-enriched guanylate kinase-associated homolog (rat)	0.9959	1.002393	0.9982632
57597	BAHCC1	BAH domain and coiled-coil containing 1	1.00337	1.017693	1.0658756
57599	WDR48	WD repeat domain 48	0.97561	0.867634	0.9635515
57600	FNIP2	folliculin interacting protein 2	1.01799	1.027969	0.9716977
57602	USP36	ubiquitin specific peptidase 36	0.95061	1.04805	0.978669
57604	KIAA1456	KIAA1456	1.00907	1.018889	1.0137617
57605	PITPNM2	phosphatidylinositol transfer protein, membrane-associated 2	0.94639	0.962094	0.9415015
57606	SLAIN2	SLAIN motif family, member 2	1.00815	0.952712	1.0531196
57608	KIAA1462	KIAA1462	0.99026	0.987325	0.9954697
57609	DIP2B	DIP2 disco-interacting protein 2 homolog B (Drosophila)	0.95815	0.95974	1.0260985
57610	RANBP10	RAN binding protein 10	0.97206	0.991127	1.1110698
57611	ISLR2	immunoglobulin superfamily containing leucine-rich repeat 2	0.99903	0.98549	1.0219076
57613	KIAA1467	KIAA1467	0.96546	0.96904	1.0656859
57614	KIAA1468	KIAA1468	0.94979	0.980985	1.0855602
57615	ZNF492	zinc finger protein 492	0.96035	1.021522	0.9841782
57616	TSHZ3	teashirt zinc finger homeobox 3	0.98898	0.980678	1.0206708
57617	VPS18	vacuolar protein sorting 18 homolog (S. cerevisiae)	0.93799	0.994966	0.9840949
57619	SHROOM3	shroom family member 3	1.00497	1.009088	1.0165148
57620	STIM2	stromal interaction molecule 2	0.98979	0.962008	1.0431934
57621	ZBTB2	zinc finger and BTB domain containing 2	0.979	1.00809	1.000573
57622	LRFN1	leucine rich repeat and fibronectin type III domain containing 1	0.9833	0.979066	0.9461344
57623	ZFAT	zinc finger and AT hook domain containing	1.00725	0.961174	0.9904658
57624	KIAA1486	KIAA1486	1.00727	1.002759	1.0174604
57626	KLHL1	kelch-like 1 (Drosophila)	1.00694	1.010134	1.0163136
57628	DPP10	dipeptidyl-peptidase 10 (non-functional)	1.01148	1.000959	1.0044925
57630	SH3RF1	SH3 domain containing ring finger 1	1.01559	1.003697	0.9970458
57631	LRCH2	leucine-rich repeats and calponin homology (CH) domain containing 2	0.98225	0.970586	1.0158847

57633	LRRN1	leucine rich repeat neuronal 1	1.00552	0.96124	0.986818
57634	EP400	E1A binding protein p400	0.98631	0.993324	0.9393285
57636	ARHGAP23	Rho GTPase activating protein 23	0.98163	0.992454	1.0060749
57639	CCDC146	coiled-coil domain containing 146	0.96621	1.099197	0.9739911
57642	COL20A1	collagen, type XX, alpha 1	0.98727	0.999544	1.0301118
57643	ZSWIM5	zinc finger, SWIM-type containing 5	0.99589	1.010119	0.9848246
57644	MYH7B	myosin, heavy chain 7B, cardiac muscle, beta	0.99155	1.025817	1.0599403
57645	POGK	pogo transposable element with KRAB domain	0.97366	0.818493	1.1004703
57646	USP28	ubiquitin specific peptidase 28	0.93512	1.041283	0.9508699
57647	DHX37	DEAH (Asp-Glu-Ala-His) box polypeptide 37	0.95901	0.915437	1.0262268
57648	KIAA1522	KIAA1522	0.99919	1.021012	1.026651
57649	PHF12	PHD finger protein 12	0.9644	1.063568	0.955631
57650	KIAA1524	KIAA1524	1.00463	0.937298	1.0328558
57653	BDAG1	LOC100499484-C9orf174 readthrough	0.97687	0.945527	0.9759726
57654	KIAA1530	KIAA1530	1.00368	1.001759	0.9795714
57655	GRAMD1A	GRAM domain containing 1A	0.98055	1.049768	0.9756504
57657	HCN3	hyperpolarization activated cyclic nucleotide-gated potassium channel 3	1.01058	1.016539	1.0442981
57658	CALCOCO1	calcium binding and coiled-coil domain 1	0.96866	1.008872	1.0434751
57659	ZBTB4	zinc finger and BTB domain containing 4	0.90586	1.033918	0.9301358
57661	PHRF1	PHD and ring finger domains 1	0.95221	0.96157	0.9666135
57662	KIAA1543	KIAA1543	1	0.959677	1.0445155
57663	USP29	ubiquitin specific peptidase 29	1.0058	0.97095	0.9942177
57664	PLEKHA4	pleckstrin homology domain containing, family A (phosphoinositide binding specific) member 4	0.98586	0.970394	1.0180379
57665	RDH14	retinol dehydrogenase 14 (all-trans/9-cis/11-cis)	0.97871	0.942685	1.039881
57666	FBRSL1	fibrosin-like 1	1.00668	1.028576	1.0067697
57669	EPB41L5	erythrocyte membrane protein band 4.1 like 5	0.97273	1.006151	0.9739971
57670	KIAA1549	KIAA1549	1.00727	0.995786	0.9961781
57673	BEND3	BEN domain containing 3	1.00309	0.978875	1.0387331
57674	RNF213	ring finger protein 213	0.98148	0.901097	0.981139
57677	ZFP14	zinc finger protein 14 homolog (mouse)	0.95502	0.978025	0.9833582
57678	GPAM	glycerol-3-phosphate acyltransferase, mitochondrial	0.93442	0.926055	1.0657628
57679	ALS2	amyotrophic lateral sclerosis 2 (juvenile)	0.97922	1.042425	1.0290446
57680	CHD8	chromodomain helicase DNA binding protein 8	0.9552	1.078669	1.1029685

57683	ZDBF2	zinc finger, DBF-type containing 2	0.97804	0.981355	1.0644056
57684	ZBTB26	zinc finger and BTB domain containing 26	0.97499	0.999129	1.0931844
57685	CACHD1	cache domain containing 1	0.99144	1.010992	0.9768559
57687	VAT1L	vesicle amine transport protein 1 homolog (T. californica)-like	1.00288	0.993206	0.9880401
57688	ZSWIM6	zinc finger, SWIM-type containing 6	1.01208	1.043571	0.8864729
57689	LRRC4C	leucine rich repeat containing 4C	0.99729	0.994221	1.0080887
57690	TNRC6C	trinucleotide repeat containing 6C	0.9609	0.962602	0.8746535
57691	KIAA1586	KIAA1586	0.9793	0.926231	1.0029835
57692	MAGEE1	melanoma antigen family E, 1	0.94817	0.957042	0.9650014
57693	ZNF317	zinc finger protein 317	0.95406	0.937882	1.0459538
57695	USP37	ubiquitin specific peptidase 37	0.97713	1.00032	1.0168207
57696	DDX55	DEAD (Asp-Glu-Ala-Asp) box polypeptide 55	0.9591	1.009081	1.0114141
57697	FANCM	Fanconi anemia, complementation group M	0.94843	0.973203	1.027217
57698	KIAA1598	KIAA1598	0.97913	1.02467	1.0277512
57699	CPNE5	copine V	1.0157	0.974786	1.0194483
57700	FAM160B1	family with sequence similarity 160, member B1	0.96696	0.91608	1.017223
57701	NCKAP5L	NCK-associated protein 5-like	0.99754	1.031123	0.9962023
57703	CWC22	CWC22 spliceosome-associated protein homolog (S. cerevisiae)	0.97584	0.991388	1.0848533
57704	GBA2	glucosidase, beta (bile acid) 2	0.9669	1.041379	1.0026599
57705	WDFY4	WDFY family member 4	1.01093	1.041416	0.9793706
57706	DENND1A	DENN/MADD domain containing 1A	1.00936	0.988001	1.0348993
57707	KIAA1609	KIAA1609	1.00156	1.00753	1.0367908
57708	MIER1	mesoderm induction early response 1 homolog (Xenopus laevis)	0.9711	0.897499	0.9356082
57709	SLC7A14	solute carrier family 7 (cationic amino acid transporter, y+ system), member 14	0.99862	1.008455	1.0033873
57710	KIAA1614	KIAA1614	1.00087	0.994262	0.9952749
57711	ZNF529	zinc finger protein 529	0.93969	1.024019	0.9483365
57713	SFMBT2	Scm-like with four mbt domains 2	0.96418	1.004826	1.0072295
57714	[No Symbo	[No Name]	0.98808	0.883887	1.0454854
57715	SEMA4G	sema domain, immunoglobulin domain (Ig), transmembrane domain (TM) and short cytoplasmic domain, (se	1.00779	0.998575	1.0379163
57716	PRX	periaxin	0.99968	0.987248	1.0383406
57717	PCDHB16	protocadherin beta 16	1.04499	1.023209	1.0416817
57718	PPP4R4	protein phosphatase 4, regulatory subunit 4	1.00357	0.977218	0.9555388
57719	ANO8	anoctamin 8	0.98309	0.946534	1.0146795

57720	GPR107	G protein-coupled receptor 107	0.96301	0.962231	1.0750481
57721	METTL14	methyltransferase like 14	0.99206	0.937504	1.0689971
57722	IGDCC4	immunoglobulin superfamily, DCC subclass, member 4	1.00307	0.981446	0.989454
57724	EPG5	ectopic P-granules autophagy protein 5 homolog (C. elegans)	0.92853	1.202553	0.9992718
57727	NCOA5	nuclear receptor coactivator 5	0.96017	0.996048	1.0998696
57728	WDR19	WD repeat domain 19	0.97076	1.002987	1.0262576
57730	ANKRD36B	ankyrin repeat domain 36B	1.03048	0.899387	1.0419036
57731	SPTBN4	spectrin, beta, non-erythrocytic 4	0.98267	1.026583	1.0604373
57732	ZFYVE28	zinc finger, FYVE domain containing 28	1.0036	0.985439	1.0052737
57733	GBA3	glucosidase, beta, acid 3 (cytosolic)	1.0075	0.994991	1.0159388
57758	SCUBE2	signal peptide, CUB domain, EGF-like 2	0.99701	0.982873	0.9930191
57761	TRIB3	tribbles homolog 3 (Drosophila)	0.95834	0.984993	1.0715479
57763	ANKRA2	ankyrin repeat, family A (RFXANK-like), 2	1.00644	0.932732	0.9441489
57786	RBAK	RB-associated KRAB zinc finger	0.97293	1.010727	1.0044566
57787	MARK4	MAP/microtubule affinity-regulating kinase 4	0.97205	1.04628	1.0011658
57794	SUGP1	SURP and G patch domain containing 1	0.96665	1.006119	1.0672638
57795	FAM5B	family with sequence similarity 5, member B	1.01696	1.009272	1.0135967
57798	GATAD1	GATA zinc finger domain containing 1	0.99305	0.95524	1.061587
57799	RAB40C	RAB40C, member RAS oncogene family	0.98618	1.005912	1.0115949
57801	HES4	hairy and enhancer of split 4 (Drosophila)	0.97977	0.990092	1.0861894
57804	POLD4	polymerase (DNA-directed), delta 4	0.94404	0.969237	1.060835
57805	KIAA1967	KIAA1967	0.98511	1.027822	1.0099444
57817	HAMP	hepcidin antimicrobial peptide	0.99593	1.004892	0.9819032
57818	G6PC2	glucose-6-phosphatase, catalytic, 2	0.98464	0.999176	0.9989365
57819	LSM2	LSM2 homolog, U6 small nuclear RNA associated (S. cerevisiae)	0.97913	0.966517	1.0206867
57820	CCNB1IP1	cyclin B1 interacting protein 1, E3 ubiquitin protein ligase	0.96432	0.941732	0.9963393
57821	C1orf114	chromosome 1 open reading frame 114	1.02156	1.009053	1.0299287
57822	GRHL3	grainyhead-like 3 (Drosophila)	0.99926	1.013135	1.0284242
57823	SLAMF7	SLAM family member 7	1.04098	0.983419	0.8819492
57824	HMHB1	histocompatibility (minor) HB-1	0.98596	1.061962	1.0233246
57826	RAP2C	RAP2C, member of RAS oncogene family	1.00931	1.060572	1.0454448
57827	C6orf47	chromosome 6 open reading frame 47	0.96982	1.013635	1.0008656
57828	CATSPERG	cation channel, sperm-associated, gamma	0.98543	1.038345	0.9924342

57829	ZP4	zona pellucida glycoprotein 4	1.00496	1.01371	1.0099964
57834	CYP4F11	cytochrome P450, family 4, subfamily F, polypeptide 11	1.00751	1.00622	0.9900211
57835	SLC4A5	solute carrier family 4, sodium bicarbonate cotransporter, member 5	0.99973	0.96538	0.9539085
57862	ZNF410	zinc finger protein 410	0.92728	0.981373	0.9808183
57863	CADM3	cell adhesion molecule 3	1.00434	0.986382	1.0046053
57864	SLC46A2	solute carrier family 46, member 2	0.94297	1.009292	0.9871057
58155	PTBP2	polypyrimidine tract binding protein 2	0.9987	0.961419	0.9643384
58157	NGB	neuroglobin	1.00551	1.000541	1.0445076
58158	NEUROD4	neurogenic differentiation 4	1.0242	1.024349	1.0242318
58160	NFE4	transcription factor NF-E4	1.01522	1.023094	0.9504354
58189	WFDC1	WAP four-disulfide core domain 1	1.00345	1.008029	1.0129006
58190	CTDSP1	CTD (carboxy-terminal domain, RNA polymerase II, polypeptide A) small phosphatase 1	1.00263	0.85675	1.0300017
58191	CXCL16	chemokine (C-X-C motif) ligand 16	0.97722	1.087284	0.8943938
58472	SQRDL	sulfide quinone reductase-like (yeast)	0.95849	0.958022	0.9817856
58473	PLEKHB1	pleckstrin homology domain containing, family B (evectins) member 1	0.93468	0.937122	0.9650223
58475	MS4A7	membrane-spanning 4-domains, subfamily A, member 7	1.01159	1.0208	0.8837132
58476	TP53INP2	tumor protein p53 inducible nuclear protein 2	0.99097	0.974684	0.9331399
58477	SRPRB	signal recognition particle receptor, B subunit	0.96685	0.956828	1.0955722
58478	ENOPH1	enolase-phosphatase 1	0.98167	0.910173	1.032895
58480	RHOU	ras homolog gene family, member U	1.04577	0.916497	0.8863622
58484	NLRC4	NLR family, CARD domain containing 4	1.06243	1.050652	0.9703005
58485	TRAPPC1	trafficking protein particle complex 1	0.97858	0.928752	1.0782099
58486	ZBED5	zinc finger, BED-type containing 5	0.95672	0.95071	0.9461228
58487	CREBZF	CREB/ATF bZIP transcription factor	0.97015	0.91721	1.085522
58488	PCTP	phosphatidylcholine transfer protein	0.96199	1.034491	1.0220659
58489	FAM108C1	family with sequence similarity 108, member C1	0.98169	1.010399	1.0135568
58490	RPRD1B	regulation of nuclear pre-mRNA domain containing 1B	0.97578	0.967742	0.9440024
58491	ZNF71	zinc finger protein 71	0.95498	0.96539	1.0658817
58492	ZNF77	zinc finger protein 77	0.94363	0.945919	1.0107501
58493	C9orf80	chromosome 9 open reading frame 80	0.95384	0.978407	1.0006808
58494	JAM2	junctional adhesion molecule 2	0.98922	0.992398	1.0228245
58495	OVOL2	ovo-like 2 (Drosophila)	0.98095	0.991487	1.0003499
58496	LY6G5B	lymphocyte antigen 6 complex, locus G5B	1.08412	0.987437	0.9522139

58497	PRUNE	prune homolog (Drosophila)	0.99739	0.920236	0.9236971
58498	MYL7	myosin, light chain 7, regulatory	0.99234	1.002987	1.0302127
58499	ZNF462	zinc finger protein 462	0.98514	0.99462	1.0146294
58500	ZNF250	zinc finger protein 250	0.96847	1.018817	0.9402362
58503	PROL1	proline rich, lacrimal 1	1.00442	1.006093	1.0252043
58504	ARHGAP22	Rho GTPase activating protein 22	0.98593	0.991145	1.0073596
58505	OSTC	oligosaccharyltransferase complex subunit	1.02159	0.99993	1.0591687
58506	SCAF1	SR-related CTD-associated factor 1	0.97609	1.055526	1.0156053
58508	MLL3	myeloid/lymphoid or mixed-lineage leukemia 3	1.00389	1.064354	0.8294561
58509	C19orf29	chromosome 19 open reading frame 29	0.96651	1.022485	1.0682613
58510	PRODH2	proline dehydrogenase (oxidase) 2	0.98358	1.000361	1.0192127
58512	DLGAP3	discs, large (Drosophila) homolog-associated protein 3	0.99163	1.008684	1.0332576
58513	EPS15L1	epidermal growth factor receptor pathway substrate 15-like 1	0.98669	1.030363	1.0379084
58515	SELK	selenoprotein K	0.96374	0.941422	0.9856307
58516	FAM60A	family with sequence similarity 60, member A	0.97653	0.947686	1.0266893
58517	RBM25	RNA binding motif protein 25	0.95332	0.931256	1.023697
58524	DMRT3	doublesex and mab-3 related transcription factor 3	0.992	1.020963	1.0285191
58525	WIZ	widely interspaced zinc finger motifs	0.99318	1.032314	1.0686783
58526	MID1IP1	MID1 interacting protein 1 (gastrulation specific G12 homolog (zebrafish))	0.97697	0.943038	0.9609035
58527	C6orf115	chromosome 6 open reading frame 115	1.00536	0.901806	1.0107518
58528	RRAGD	Ras-related GTP binding D	0.99688	0.931359	0.9848803
58529	MYOZ1	myozenin 1	0.98249	1.002351	1.0399555
58530	LY6G6D	lymphocyte antigen 6 complex, locus G6D	1.00156	0.993941	1.0297831
58531	PRM3	protamine 3	0.96479	0.993125	1.0431117
58533	SNX6	sorting nexin 6	0.96243	0.961857	0.9853696
58538	MPP4	membrane protein, palmitoylated 4 (MAGUK p55 subfamily member 4)	1.013	1.001919	1.0073176
58985	IL22RA1	interleukin 22 receptor, alpha 1	0.99855	1.007311	1.0197702
58986	TMEM8A	transmembrane protein 8A	0.92855	0.952032	1.095056
59067	IL21	interleukin 21	1.00607	0.978839	0.9501742
59082	CARD18	caspase recruitment domain family, member 18	1.0158	1.032946	0.9577574
59084	ENPP5	ectonucleotide pyrophosphatase/phosphodiesterase 5 (putative)	1.00785	0.969325	0.994788
59269	HIVEP3	human immunodeficiency virus type I enhancer binding protein 3	0.98782	0.944088	0.9861886
59271	C21orf63	chromosome 21 open reading frame 63	0.99573	1.000645	0.9667123

59272	ACE2	angiotensin I converting enzyme (peptidyl-dipeptidase A) 2	0.99272	1.020929	1.015439
59274	MESDC1	mesoderm development candidate 1	0.94885	1.065032	1.0819384
59277	NTN4	netrin 4	1.00385	0.998891	1.0011769
59283	CACNG8	calcium channel, voltage-dependent, gamma subunit 8	0.97262	1.03148	1.0997972
59284	CACNG7	calcium channel, voltage-dependent, gamma subunit 7	1.00655	1.040516	0.9978551
59285	CACNG6	calcium channel, voltage-dependent, gamma subunit 6	0.98249	0.992672	1.0366772
59286	UBL5	ubiquitin-like 5	0.97309	0.933977	0.9542255
59307	SIGIRR	single immunoglobulin and toll-interleukin 1 receptor (TIR) domain	0.95816	0.946353	1.0033125
59335	PRDM12	PR domain containing 12	0.96725	1.019057	1.0228181
59336	PRDM13	PR domain containing 13	0.98656	1.009827	1.071728
59338	PLEKHA1	pleckstrin homology domain containing, family A (phosphoinositide binding specific) member 1	0.91391	1.050645	1.0150662
59339	PLEKHA2	pleckstrin homology domain containing, family A (phosphoinositide binding specific) member 2	1.00936	1.084733	0.9613482
59340	HRH4	histamine receptor H4	0.98587	0.920244	0.9885092
59341	TRPV4	transient receptor potential cation channel, subfamily V, member 4	0.9927	1.007736	1.0140008
59342	SCPEP1	serine carboxypeptidase 1	0.98472	0.923287	1.078125
59343	SENP2	SUMO1/sentrin/SMT3 specific peptidase 2	0.99459	0.953677	0.9668751
59344	ALOXE3	arachidonate lipoxygenase 3	0.97753	0.978351	1.025667
59345	GNB4	guanine nucleotide binding protein (G protein), beta polypeptide 4	1.04821	0.947926	1.0084192
59348	ZNF350	zinc finger protein 350	0.95265	0.953262	0.9436889
59349	KLHL12	kelch-like 12 (Drosophila)	0.99441	0.938299	1.0164522
59350	RXFP1	relaxin/insulin-like family peptide receptor 1	0.99805	1.008155	1.0038526
59351	PBOV1	prostate and breast cancer overexpressed 1	1.01781	1.022705	1.0110131
59352	LGR6	leucine-rich repeat containing G protein-coupled receptor 6	0.98518	1.020744	0.9615667
59353	TMEM35	transmembrane protein 35	0.99921	1.01449	1.023701
60312	AFAP1	actin filament associated protein 1	1.00182	1.028329	0.9543649
60313	GPBP1L1	GC-rich promoter binding protein 1-like 1	1.00298	1.016912	1.0604827
60314	C12orf10	chromosome 12 open reading frame 10	0.95057	0.981536	1.0500787
60343	FAM3A	family with sequence similarity 3, member A	0.97407	0.985224	1.0295955
60370	AVPI1	arginine vasopressin-induced 1	1.00718	0.984337	0.8878563
60385	TSKS	testis-specific serine kinase substrate	0.99035	0.970697	1.0190076
60386	SLC25A19	solute carrier family 25 (mitochondrial thiamine pyrophosphate carrier), member 19	0.95975	0.947876	1.0160294
60401	EDA2R	ectodysplasin A2 receptor	1.00008	0.978074	1.0093602
60412	EXOC4	exocyst complex component 4	1.01038	0.909857	0.9770442

60436	TGIF2	TGFB-induced factor homeobox 2	0.96829	1.109613	0.9473806
60437	CDH26	cadherin 26	0.97961	1.002563	0.9981643
60468	BACH2	BTB and CNC homology 1, basic leucine zipper transcription factor 2	0.98326	0.973915	0.998416
60481	ELOVL5	ELOVL fatty acid elongase 5	0.9959	1.027473	1.01878
60482	SLC5A7	solute carrier family 5 (choline transporter), member 7	1.00256	1.004566	1.0172468
60484	HAPLN2	hyaluronan and proteoglycan link protein 2	1.01777	0.99083	0.9966783
60485	SAV1	salvador homolog 1 (Drosophila)	0.93862	0.928258	1.0593075
60487	TRMT11	tRNA methyltransferase 11 homolog (S. cerevisiae)	0.94418	0.977842	1.027925
60488	MRPS35	mitochondrial ribosomal protein S35	0.97965	0.9434	1.0418202
60489	APOBEC3G	apolipoprotein B mRNA editing enzyme, catalytic polypeptide-like 3G	0.89819	1.047934	1.0843993
60490	PPCDC	phosphopantothenoylcysteine decarboxylase	0.96312	1.026458	0.988668
60491	NIF3L1	NIF3 NGG1 interacting factor 3-like 1 (S. pombe)	1.01523	0.948025	1.0313643
60492	CCDC90B	coiled-coil domain containing 90B	0.96189	0.953425	1.0464255
60494	CCDC81	coiled-coil domain containing 81	0.99374	1.000285	0.9985274
60495	HPSE2	heparanase 2	0.99714	0.988909	1.006909
60496	AASDHPPT	aminoadipate-semialdehyde dehydrogenase-phosphopantetheinyl transferase	0.91722	0.950443	1.1487495
60506	NYX	nyctalopin	0.98909	1.024159	1.0033615
60509	AGBL5	ATP/GTP binding protein-like 5	0.99613	0.956032	0.9712202
60526	C2orf43	chromosome 2 open reading frame 43	0.98315	0.967577	1.0290283
60528	ELAC2	elaC homolog 2 (E. coli)	0.93977	0.921561	1.0293985
60529	ALX4	ALX homeobox 4	0.99101	0.966841	1.0081975
60558	GUF1	GUF1 GTPase homolog (S. cerevisiae)	0.99634	0.859129	0.9551749
60559	SPCS3	signal peptidase complex subunit 3 homolog (S. cerevisiae)	1.04428	0.952926	1.1120255
60560	NAA35	N(alpha)-acetyltransferase 35, NatC auxiliary subunit	0.96329	0.919084	1.0805085
60561	RINT1	RAD50 interactor 1	0.98955	1.013461	1.0244931
60592	SCOC	short coiled-coil protein	1.01305	1.000274	1.0209526
60598	KCNK15	potassium channel, subfamily K, member 15	0.98571	0.987129	1.0375806
60625	DHX35	DEAH (Asp-Glu-Ala-His) box polypeptide 35	0.98418	0.853333	1.092626
60626	RIC8A	resistance to inhibitors of cholinesterase 8 homolog A (C. elegans)	0.95088	0.976426	1.0168451
60672	MIIP	migration and invasion inhibitory protein	0.98318	1.003521	1.0780567
60673	C12orf44	chromosome 12 open reading frame 44	1.00913	1.002949	1.0139328
60675	PROK2	prokineticin 2	1.06088	1.019755	1.0592454
60676	PAPPA2	pappalysin 2	1.00844	1.00554	0.9951308

60677	CELF6	CUGBP, Elav-like family member 6	0.97284	1.037318	0.9785639
60678	EEFSEC	eukaryotic elongation factor, selenocysteine-tRNA-specific	1.01486	0.94027	0.9830523
60680	CELF5	CUGBP, Elav-like family member 5	0.99376	0.983816	1.0096904
60681	FKBP10	FK506 binding protein 10, 65 kDa	0.98442	1.001531	1.0247836
60682	SMAP1	small ArfGAP 1	0.9709	0.956333	0.9970191
60684	C4orf41	chromosome 4 open reading frame 41	1.00679	0.963882	1.0780931
60685	ZFAND3	zinc finger, AN1-type domain 3	1.00404	1.030372	1.0253419
60686	C14orf93	chromosome 14 open reading frame 93	0.92905	0.963069	1.0075729
63027	SLC22A23	solute carrier family 22, member 23	0.99862	0.968231	0.9668094
63035	BCORL1	BCL6 corepressor-like 1	0.97309	1.00372	0.9886329
63036	CELA2A	chymotrypsin-like elastase family, member 2A	0.99105	0.984647	1.0004975
63826	SRR	serine racemase	0.94981	0.956832	1.0501452
63827	BCAN	brevican	1.00537	1.01431	1.0395558
63874	ABHD4	abhydrolase domain containing 4	0.91028	0.99748	0.9053125
63875	MRPL17	mitochondrial ribosomal protein L17	0.92991	0.906051	1.072446
63876	PKNOX2	PBX/knotted 1 homeobox 2	1.00656	1.01303	1.0004165
63877	FAM204A	family with sequence similarity 204, member A	0.98411	0.949173	1.1290111
63891	RNF123	ring finger protein 123	1.03092	0.979381	1.0394437
63892	THADA	thyroid adenoma associated	0.9903	0.935878	1.0298699
63893	UBE2O	ubiquitin-conjugating enzyme E2O	0.95789	0.994074	1.0402527
63894	VIPAR	VPS33B interacting protein, apical-basolateral polarity regulator	0.96144	0.935134	1.0536648
63897	HEATR6	HEAT repeat containing 6	0.92436	1.099752	1.0762238
63898	SH2D4A	SH2 domain containing 4A	1.01588	1.00157	1.0189373
63899	NSUN3	NOP2/Sun domain family, member 3	0.99215	0.934425	1.0560517
63901	FAM111A	family with sequence similarity 111, member A	1.00983	0.944922	1.1317782
63904	DUSP21	dual specificity phosphatase 21	1.01012	0.993496	0.9641995
63905	MANBAL	mannosidase, beta A, lysosomal-like	0.98737	1.00573	1.0326059
63906	GPATCH3	G patch domain containing 3	0.9932	0.947595	1.0300921
63908	NAPB	N-ethylmaleimide-sensitive factor attachment protein, beta	0.95669	1.015795	0.9896907
63910	SLC17A9	solute carrier family 17, member 9	0.99103	1.034056	1.0269573
63916	ELMO2	engulfment and cell motility 2	0.97162	1.050748	0.9835717
63917	GALNT11	UDP-N-acetyl-alpha-D-galactosamine:polypeptide N-acetylgalactosaminyltransferase 11 (GalNAc-T11)	1.00788	1.017723	0.9393646
63920	C5orf54	chromosome 5 open reading frame 54	0.98498	1.006111	1.0456183

63922	CHTF18	CTF18, chromosome transmission fidelity factor 18 homolog (S. cerevisiae)	0.97441	1.049154	1.0662812
63923	TNN	tenascin N	1.00018	1.01917	1.0124684
63924	CIDEC	cell death-inducing DFFA-like effector c	1.0086	0.986432	0.9976668
63925	ZNF335	zinc finger protein 335	0.9814	1.006186	0.9978513
63926	ANKRD5	ankyrin repeat domain 5	0.95538	0.977258	1.0402329
63928	CHP2	calcineurin B homologous protein 2	0.9784	1.010571	1.0095541
63929	XPNPEP3	X-prolyl aminopeptidase (aminopeptidase P) 3, putative	0.95214	0.98312	1.0039206
63930	LOC63930	hypothetical LOC63930	0.99799	1.010515	1.0337775
63931	MRPS14	mitochondrial ribosomal protein S14	0.95236	0.933665	1.0498569
63932	CXorf56	chromosome X open reading frame 56	0.91741	1.015763	0.9955266
63933	CCDC90A	coiled-coil domain containing 90A	0.97884	0.984045	1.0211235
63934	ZNF667	zinc finger protein 667	0.97867	1.015979	0.9856829
63935	PCIF1	PDX1 C-terminal inhibiting factor 1	0.94311	1.042788	0.9768081
63939	C20orf177	chromosome 20 open reading frame 177	0.89876	0.918217	1.1951642
63940	GPSM3	G-protein signaling modulator 3	0.99025	1.065986	0.9912041
63941	NECAB3	N-terminal EF-hand calcium binding protein 3	0.98672	0.994619	1.0069212
63943	FKBPL	FK506 binding protein like	0.99334	1.033908	0.9957923
63946	DMRTC2	DMRT-like family C2	0.98678	1.002023	1.000231
63948	DMRTB1	DMRT-like family B with proline-rich C-terminal, 1	1.00177	1.020687	1.0334171
63950	DMRTA2	DMRT-like family A2	0.99602	1.003895	1.0700808
63951	DMRTA1	DMRT-like family A1	1.00185	0.96563	1.0916443
63967	CLSPN	claspin	1.01659	0.988696	1.0178932
63969	[No Symbo	[No Name]	0.986	1.011333	0.99081
63970	TP53AIP1	tumor protein p53 regulated apoptosis inducing protein 1	0.98679	0.983245	0.9864083
63971	KIF13A	kinesin family member 13A	1.01092	0.924913	1.0066468
63973	NEUROG2	neurogenin 2	0.98725	1.039801	1.0384398
63974	NEUROD6	neurogenic differentiation 6	1.01615	0.964202	1.0503544
63976	PRDM16	PR domain containing 16	1.00068	1.025134	1.0179208
63977	PRDM15	PR domain containing 15	0.96735	0.949285	1.000863
63978	PRDM14	PR domain containing 14	0.99457	0.989823	0.9824041
63979	FIGNL1	fidgetin-like 1	0.9943	0.995368	1.0154031
63982	ANO3	anoctamin 3	1.00268	1.033863	0.9994396
64002	PCGEM1	prostate-specific transcript 1 (non-protein coding)	1.01424	1.034048	0.9674626

64005	MYO1G	myosin IG	1.03755	1.030135	1.0016733
64061	TSPYL2	TSPY-like 2	0.92596	1.079219	0.8873516
64062	RBM26	RNA binding motif protein 26	0.9288	0.95319	1.0944017
64063	PRSS22	protease, serine, 22	1.00557	0.952834	1.0448446
64065	PERP	PERP, TP53 apoptosis effector	1.00673	0.990627	1.0134043
64066	MMP27	matrix metallopeptidase 27	1.00109	0.991032	1.0048046
64067	NPAS3	neuronal PAS domain protein 3	0.99178	1.002908	0.9988829
64072	CDH23	cadherin-related 23	1.00824	0.950957	1.0186502
64073	C19orf33	chromosome 19 open reading frame 33	0.95365	1.049457	0.9973731
64077	LHPP	phospholysine phosphohistidine inorganic pyrophosphate phosphatase	0.98272	0.979582	1.0000915
64078	SLC28A3	solute carrier family 28 (sodium-coupled nucleoside transporter), member 3	1.00233	0.995803	1.0158687
64080	RBKS	ribokinase	1.00461	0.98847	0.9707026
64081	PBLD	phenazine biosynthesis-like protein domain containing	0.97638	0.989007	0.9759
64083	GOLPH3	golgi phosphoprotein 3 (coat-protein)	0.99518	0.878317	1.0075159
64084	CLSTN2	calsyntenin 2	1.01215	1.016623	1.0074367
64087	MCCC2	methylcrotonoyl-CoA carboxylase 2 (beta)	1.00277	0.934568	0.9885009
64089	SNX16	sorting nexin 16	0.93101	0.977014	1.0621093
64090	GAL3ST2	galactose-3-O-sulfotransferase 2	0.97897	1.061218	1.0305623
64091	POPDC2	popeye domain containing 2	0.98948	0.987301	1.0301123
64092	SAMSN1	SAM domain, SH3 domain and nuclear localization signals 1	0.98657	1.028033	0.988961
64093	SMOC1	SPARC related modular calcium binding 1	1.00668	1.003404	0.996085
64094	SMOC2	SPARC related modular calcium binding 2	1.00485	1.011439	1.0249352
64096	GFRA4	GDNF family receptor alpha 4	0.99726	0.990473	1.0181505
64097	EPB41L4A	erythrocyte membrane protein band 4.1 like 4A	1.02697	0.987461	0.972724
64098	PARVG	parvin, gamma	0.94932	1.02404	0.9913067
64100	ELSPBP1	epididymal sperm binding protein 1	0.97787	1.002939	1.0000436
64101	LRRC4	leucine rich repeat containing 4	0.97592	0.945504	0.950434
64102	TNMD	tenomodulin	1.01178	0.998082	1.0086253
64105	CENPK	centromere protein K	0.98337	1.087755	1.0136728
64106	NPFFR1	neuropeptide FF receptor 1	0.99667	0.990548	1.0265271
64108	RTP4	receptor (chemosensory) transporter protein 4	0.95987	1.008201	1.0208751
64110	MAGEF1	melanoma antigen family F, 1	0.98871	0.872703	1.0574398
64111	NPVF	neuropeptide VF precursor	0.99642	1.020005	1.030748

64112	MOAP1	modulator of apoptosis 1	0.96368	0.90515	1.0045164
64114	TMBIM1	transmembrane BAX inhibitor motif containing 1	0.99306	1.009235	0.9701392
64115	C10orf54	chromosome 10 open reading frame 54	1.00341	0.935072	0.9690937
64116	SLC39A8	solute carrier family 39 (zinc transporter), member 8	0.98693	1.029745	1.0028609
64118	DUS1L	dihydrouridine synthase 1-like (S. cerevisiae)	0.96783	0.998132	1.0661711
64121	RRAGC	Ras-related GTP binding C	1.0015	0.956917	1.0700166
64122	FN3K	fructosamine 3 kinase	0.97308	1.022716	0.9783754
64123	ELTD1	EGF, latrophilin and seven transmembrane domain containing 1	1.01512	0.988323	1.0048247
64127	NOD2	nucleotide-binding oligomerization domain containing 2	0.96744	1.055524	0.9635454
64129	TINAGL1	tubulointerstitial nephritis antigen-like 1	1.00075	1.000186	0.9987078
64130	LIN7B	lin-7 homolog B (C. elegans)	0.98645	0.997725	1.0656492
64131	XYLT1	xylosyltransferase I	1.00439	0.988958	0.955828
64132	XYLT2	xylosyltransferase II	0.95152	0.962562	1.0242838
64135	IFIH1	interferon induced with helicase C domain 1	0.99643	1.037991	0.961368
64137	ABCG4	ATP-binding cassette, sub-family G (WHITE), member 4	1.00198	1.018155	0.9852062
64145	ZFYVE20	zinc finger, FYVE domain containing 20	1.00624	0.893897	0.9162107
64147	KIF9	kinesin family member 9	1.00115	0.987384	1.0056156
64149	C17orf75	chromosome 17 open reading frame 75	0.90206	1.02734	1.040498
64150	DIO3OS	DIO3 opposite strand (non-protein coding)	1.01234	0.973839	0.9996212
64151	NCAPG	non-SMC condensin I complex, subunit G	1.02092	0.98105	1.029084
64167	ERAP2	endoplasmic reticulum aminopeptidase 2	0.97435	0.896725	0.9722332
64168	NECAB1	N-terminal EF-hand calcium binding protein 1	1.00211	1.007461	1.0220297
64170	CARD9	caspase recruitment domain family, member 9	0.98105	0.989401	1.075463
64172	OSGEPL1	O-sialoglycoprotein endopeptidase-like 1	0.96371	0.984676	1.013142
64173	[No Symbo	[No Name]	0.98818	1.006106	1.0028928
64174	DPEP2	dipeptidase 2	0.97274	0.910809	0.9936682
64175	LEPRE1	leucine proline-enriched proteoglycan (leprecan) 1	0.99202	1.0233	1.0327618
64180	DPEP3	dipeptidase 3	1.00624	0.990568	1.0169968
64184	EDDM3B	epididymal protein 3B	0.99565	0.999315	0.9881604
64207	IRF2BPL	interferon regulatory factor 2 binding protein-like	0.96937	0.982483	1.0329485
64210	MMS19	MMS19 nucleotide excision repair homolog (S. cerevisiae)	0.95875	0.997613	1.0710089
64211	LHX5	LIM homeobox 5	0.99718	1.005111	1.0662201
64215	DNAJC1	DnaJ (Hsp40) homolog, subfamily C, member 1	0.96819	1.016447	0.9910503

64216	TFB2M	transcription factor B2, mitochondrial	0.97605	0.931987	1.037073
64219	PJA1	praja ring finger 1	0.93854	1.02179	0.9843578
64220	STRA6	stimulated by retinoic acid gene 6 homolog (mouse)	1.00857	0.996419	1.0070757
64221	ROBO3	roundabout, axon guidance receptor, homolog 3 (Drosophila)	0.98928	0.982944	1.0347044
64222	TOR3A	torsin family 3, member A	0.98244	0.963642	1.0283773
64223	MLST8	MTOR associated protein, LST8 homolog (S. cerevisiae)	0.98134	0.995753	1.0492846
64224	HERPUD2	HERPUD family member 2	1.00409	1.047358	0.9634884
64225	ATL2	atlastin GTPase 2	0.99347	0.928403	0.9262812
64227	ERP29P1	endoplasmic reticulum lumenal protein 29 pseudogene 1	0.99494	1.026517	0.9383156
64231	MS4A6A	membrane-spanning 4-domains, subfamily A, member 6A	0.98937	0.943574	1.0401332
64232	MS4A5	membrane-spanning 4-domains, subfamily A, member 5	1.00498	1.00832	0.9617309
64236	PDLIM2	PDZ and LIM domain 2 (mystique)	0.99263	0.97732	1.0536668
64240	ABCG5	ATP-binding cassette, sub-family G (WHITE), member 5	1.00083	1.021573	1.0018852
64241	ABCG8	ATP-binding cassette, sub-family G (WHITE), member 8	0.99143	1.000998	1.0359927
64282	PAPD5	PAP associated domain containing 5	0.95326	0.98275	0.8906949
64283	RGNEF	190 kDa guanine nucleotide exchange factor	1.01125	1.029599	1.0081497
64284	RAB17	RAB17, member RAS oncogene family	1.00979	1.005486	1.0326398
64285	RHBDF1	rhomboid 5 homolog 1 (Drosophila)	0.97572	1.024061	1.0290167
64288	ZNF323	zinc finger protein 323	1.02058	0.991421	1.0042875
64318	NOC3L	nucleolar complex associated 3 homolog (S. cerevisiae)	0.93833	1.03253	1.0599925
64319	FBRS	fibrosin	0.94951	1.009635	0.9683245
64320	RNF25	ring finger protein 25	0.92186	1.026565	1.0690954
64321	SOX17	SRY (sex determining region Y)-box 17	1.00062	1.035077	1.0943168
64324	NSD1	nuclear receptor binding SET domain protein 1	0.99395	0.947242	0.9278967
64326	RFWD2	ring finger and WD repeat domain 2	0.99704	0.975854	0.9595242
64327	LMBR1	limb region 1 homolog (mouse)	0.99103	0.927037	0.8882162
64328	XPO4	exportin 4	0.93778	0.913866	1.1088713
64332	NFKBIZ	nuclear factor of kappa light polypeptide gene enhancer in B-cells inhibitor, zeta	1.04695	1.055686	0.8570968
64333	ARHGAP9	Rho GTPase activating protein 9	0.97933	1.032669	1.0419782
64342	HS1BP3	HCLS1 binding protein 3	0.99462	0.981094	1.0081982
64343	AZI2	5-azacytidine induced 2	0.99696	0.974378	1.0791091
64344	HIF3A	hypoxia inducible factor 3, alpha subunit	0.99354	1.000833	1.0294692
64359	NXN	nucleoredoxin	0.98967	1.012244	1.0237831

64374	SIL1	SIL1 homolog, endoplasmic reticulum chaperone (S. cerevisiae)	1.00504	0.964298	1.0246547
64375	IKZF4	IKAROS family zinc finger 4 (Eos)	0.98665	0.972083	1.0079911
64376	IKZF5	IKAROS family zinc finger 5 (Pegasus)	0.9246	0.883538	1.0266547
64377	CHST8	carbohydrate (N-acetylgalactosamine 4-0) sulfotransferase 8	0.99289	1.016344	1.0158402
64386	MMP25	matrix metallopeptidase 25	1.02178	1.001789	0.9638155
64388	GREM2	gremlin 2	0.99495	0.985105	1.021637
64393	ZMAT3	zinc finger, matrin-type 3	0.9451	0.894693	0.9812369
64395	GMCL1	germ cell-less homolog 1 (Drosophila)	0.98553	0.991423	1.0282854
64396	GMCL1L	germ cell-less homolog 1 (Drosophila)-like	0.98911	1.019387	1.0010973
64397	ZFP106	zinc finger protein 106 homolog (mouse)	0.98014	0.971098	1.0948738
64398	MPP5	membrane protein, palmitoylated 5 (MAGUK p55 subfamily member 5)	0.96162	0.930444	1.0310257
64399	HHIP	hedgehog interacting protein	0.99761	0.99712	1.0177879
64400	AKTIP	AKT interacting protein	0.9641	1.065275	0.9990335
64403	CDH24	cadherin 24, type 2	1.01456	1.008367	1.045639
64405	CDH22	cadherin 22, type 2	0.99984	1.019618	1.0458757
64407	RGS18	regulator of G-protein signaling 18	0.94238	0.841824	0.9840394
64409	WBSCR17	Williams-Beuren syndrome chromosome region 17	1.00855	1.008897	1.0045215
64410	KLHL25	kelch-like 25 (Drosophila)	0.96817	0.971187	0.9774156
64411	ARAP3	ArfGAP with RhoGAP domain, ankyrin repeat and PH domain 3	1.016	0.9644	1.0091766
64412	GZF1	GDNF-inducible zinc finger protein 1	0.92493	1.041357	0.8966247
64417	C5orf28	chromosome 5 open reading frame 28	1.01803	0.889424	1.0613389
64418	TMEM168	transmembrane protein 168	1.0129	0.972656	1.0374514
64419	MTMR14	myotubularin related protein 14	0.99909	0.980472	1.0064721
64420	SUSD1	sushi domain containing 1	0.98108	0.964128	0.9854339
64421	DCLRE1C	DNA cross-link repair 1C	0.91558	0.939006	1.0501439
64422	ATG3	ATG3 autophagy related 3 homolog (S. cerevisiae)	1.02078	0.95126	0.9909082
64423	INF2	inverted formin, FH2 and WH2 domain containing	0.98419	1.001113	1.0164465
64425	POLR1E	polymerase (RNA) I polypeptide E, 53kDa	0.89335	0.900891	1.023071
64426	SUDS3	suppressor of defective silencing 3 homolog (S. cerevisiae)	0.98102	0.976652	1.0363562
64427	TTC31	tetratricopeptide repeat domain 31	0.97758	0.916377	1.0521128
64428	NARFL	nuclear prelamin A recognition factor-like	0.95638	0.970783	1.0396706
64429	ZDHHC6	zinc finger, DHHC-type containing 6	0.95651	0.944132	1.1203204
64430	C14orf135	chromosome 14 open reading frame 135	0.94579	0.985734	1.069098

64431	ACTR6	ARP6 actin-related protein 6 homolog (yeast)	0.93053	0.87203	1.0526107
64432	MRPS25	mitochondrial ribosomal protein S25	0.99261	0.944238	0.9762921
64434	NOM1	nucleolar protein with MIF4G domain 1	1.00187	0.922205	1.01457
64446	DNAI2	dynein, axonemal, intermediate chain 2	0.99968	1.028461	1.0238201
64478	CSMD1	CUB and Sushi multiple domains 1	1.01467	1.010993	1.0150361
64506	CPEB1	cytoplasmic polyadenylation element binding protein 1	1.00536	0.986498	1.0016462
64518	TEKT3	tektin 3	0.98951	0.977335	1.018396
64577	ALDH8A1	aldehyde dehydrogenase 8 family, member A1	1.00664	0.972619	1.0047146
64579	NDST4	N-deacetylase/N-sulfotransferase (heparan glucosaminyl) 4	0.99955	1.013923	1.0272258
64581	CLEC7A	C-type lectin domain family 7, member A	1.05976	1.035401	0.9816428
64582	GPR135	G protein-coupled receptor 135	0.99259	1.02732	1.063747
64593	RBMY3AP	RNA binding motif protein, Y-linked, family 3, member A pseudogene	1.02669	0.964051	0.9936306
64595	TTTY15	testis-specific transcript, Y-linked 15 (non-protein coding)	0.99243	0.984527	1.0245064
64598	MOSPD3	motile sperm domain containing 3	0.99112	0.977364	1.0501864
64599	GIGYF1	GRB10 interacting GYF protein 1	0.95978	0.971132	0.9950909
64600	PLA2G2F	phospholipase A2, group IIF	0.99974	0.986771	1.0190346
64601	VPS16	vacuolar protein sorting 16 homolog (S. cerevisiae)	0.95052	0.926526	1.0866166
64641	EBF2	early B-cell factor 2	0.99635	1.006508	0.9918867
64645	HIAT1	hippocampus abundant transcript 1	1.0137	0.941927	0.9841382
64651	CSRNP1	cysteine-serine-rich nuclear protein 1	1.02154	1.005801	0.893915
64682	ANAPC1	anaphase promoting complex subunit 1	0.96864	1.028261	1.0129907
64689	GORASP1	golgi reassembly stacking protein 1, 65kDa	0.99632	0.954712	0.9461069
64693	CTAGE1	cutaneous T-cell lymphoma-associated antigen 1	1.00186	0.9788	1.0614179
64699	TMPRSS3	transmembrane protease, serine 3	0.9985	0.996694	1.0191251
64708	COPS7B	COP9 constitutive photomorphogenic homolog subunit 7B (Arabidopsis)	1.00082	0.929227	1.0733977
64710	NUCKS1	nuclear casein kinase and cyclin-dependent kinase substrate 1	0.97158	0.963954	0.9903996
64711	HS3ST6	heparan sulfate (glucosamine) 3-O-sulfotransferase 6	1.00037	1.0243	1.0848748
64714	PDIA2	protein disulfide isomerase family A, member 2	1.00915	0.956126	1.0128228
64718	UNKL	unkempt homolog (Drosophila)-like	0.9768	0.976733	1.008609
64743	WDR13	WD repeat domain 13	0.95859	0.943215	1.0668151
64744	SMAP2	small ArfGAP2	1.03024	1.114167	0.9354468
64745	METTL17	methyltransferase like 17	0.99334	1.008392	1.1052773
64746	ACBD3	acyl-CoA binding domain containing 3	1.02996	0.984728	1.0886423

64747	MFSD1	major facilitator superfamily domain containing 1	1.02922	0.897556	0.9519945
64748	LPPR2	lipid phosphate phosphatase-related protein type 2	0.96561	1.037846	0.9948139
64750	SMURF2	SMAD specific E3 ubiquitin protein ligase 2	0.93756	1.045701	0.9952491
64753	CCDC136	coiled-coil domain containing 136	1.0058	0.992351	0.979766
64754	SMYD3	SET and MYND domain containing 3	1.00206	0.940018	0.9942718
64755	C16orf58	chromosome 16 open reading frame 58	0.96738	1.060304	1.0233172
64756	ATPAF1	ATP synthase mitochondrial F1 complex assembly factor 1	1.00088	0.977555	1.0238382
64757	MOSC1	MOCO sulphurase C-terminal domain containing 1	1.00124	0.940612	1.0224062
64759	TNS3	tensin 3	1.00447	0.973788	1.0351509
64760	FAM160B2	family with sequence similarity 160, member B2	0.99161	0.980692	0.9991666
64761	PARP12	poly (ADP-ribose) polymerase family, member 12	0.9734	0.941892	1.0160464
64762	FAM59A	family with sequence similarity 59, member A	1.00878	1.001513	0.9903564
64763	ZNF574	zinc finger protein 574	0.91865	0.982305	1.034311
64764	CREB3L2	cAMP responsive element binding protein 3-like 2	1.0107	1.013866	1.0562486
64766	S100PBP	S100P binding protein	0.99606	0.965255	0.9956819
64768	IPPK	inositol 1,3,4,5,6-pentakisphosphate 2-kinase	0.94981	1.001554	1.0536023
64769	MEAF6	MYST/Esa1-associated factor 6	1.00229	0.92326	1.0908271
64770	CCDC14	coiled-coil domain containing 14	0.99111	0.927259	1.0373472
64771	C6orf106	chromosome 6 open reading frame 106	0.98348	1.021924	1.0113761
64772	ENGASE	endo-beta-N-acetylglucosaminidase	0.97705	0.935724	0.9497511
64773	FAM113A	family with sequence similarity 113, member A	0.9494	0.952106	0.9830861
64776	C11orf1	chromosome 11 open reading frame 1	0.93612	0.957163	1.0152453
64777	RMND5B	required for meiotic nuclear division 5 homolog B (S. cerevisiae)	0.98718	0.934465	0.9394934
64778	FNDC3B	fibronectin type III domain containing 3B	1.03041	1.036337	0.989517
64779	MTHFSD	methenyltetrahydrofolate synthetase domain containing	0.93926	0.919589	1.0855039
64780	MICAL1	microtubule associated monoxygenase, calponin and LIM domain containing 1	1.01157	1.015646	0.9964084
64781	CERK	ceramide kinase	0.94689	1.105322	0.9377678
64782	AEN	apoptosis enhancing nuclease	0.95644	0.996638	1.0107015
64783	RBM15	RNA binding motif protein 15	0.99344	1.016136	1.0451653
64784	CRTC3	CREB regulated transcription coactivator 3	0.94108	0.939885	0.9444927
64785	GINS3	GINS complex subunit 3 (Psf3 homolog)	0.97972	0.993679	1.0072015
64786	TBC1D15	TBC1 domain family, member 15	0.93874	1.030981	0.9821413
64787	EPS8L2	EPS8-like 2	0.98639	0.988316	1.0762094

64788	LMF1	lipase maturation factor 1	0.96874	0.971172	0.9720086
64789	DEM1	defects in morphology 1 homolog (S. cerevisiae)	0.96477	0.962393	1.0437844
64792	RABL5	RAB, member RAS oncogene family-like 5	0.98168	0.971284	1.0212962
64793	CEP85	centrosomal protein 85kDa	1.00712	0.903585	1.0085949
64794	DDX31	DEAD (Asp-Glu-Ala-Asp) box polypeptide 31	0.95518	0.961438	1.0385329
64795	RMND5A	required for meiotic nuclear division 5 homolog A (S. cerevisiae)	1.03889	0.950205	1.0639457
64798	DEPTOR	DEP domain containing MTOR-interacting protein	1.00585	0.993054	1.0071956
64799	IQCH	IQ motif containing H	0.99449	0.976829	1.0056738
64800	EFCAB6	EF-hand calcium binding domain 6	0.99053	1.004065	0.9934297
64801	ARV1	ARV1 homolog (S. cerevisiae)	0.9858	0.955733	1.0046235
64802	NMNAT1	nicotinamide nucleotide adenylyltransferase 1	0.99273	0.966982	1.0244831
64805	P2RY12	purinergic receptor P2Y, G-protein coupled, 12	0.95368	0.905155	0.9858431
64806	IL25	interleukin 25	0.9909	0.983964	1.0081308
64816	CYP3A43	cytochrome P450, family 3, subfamily A, polypeptide 43	1.01706	0.996149	0.9923255
64834	ELOVL1	ELOVL fatty acid elongase 1	1.00846	1.068603	1.1022818
64837	KLC2	kinesin light chain 2	0.97954	1.050787	1.0327236
64838	FNDC4	fibronectin type III domain containing 4	0.99901	1.021595	1.0394054
64839	FBXL17	F-box and leucine-rich repeat protein 17	1.00327	0.976984	0.9399099
64840	PORCN	porcupine homolog (Drosophila)	0.97161	0.990854	0.9833123
64841	GNPNAT1	glucosamine-phosphate N-acetyltransferase 1	0.94625	0.950857	1.0124939
64843	ISL2	ISL LIM homeobox 2	0.98402	1.035075	1.0649153
64844	7-Mar	membrane-associated ring finger (C3HC4) 7	0.99906	0.883264	0.9328633
64847	SPATA20	spermatogenesis associated 20	0.99272	0.924065	1.0390662
64848	YTHDC2	YTH domain containing 2	1.01419	0.922736	0.9350599
64849	SLC13A3	solute carrier family 13 (sodium-dependent dicarboxylate transporter), member 3	0.99193	1.003103	0.9967923
64850	AGXT2L1	alanine-glyoxylate aminotransferase 2-like 1	1.00152	0.998384	0.9899267
64852	TUT1	terminal uridylyl transferase 1, U6 snRNA-specific	0.95421	1.010007	1.0377098
64853	AIDA	axin interactor, dorsalization associated	1.03063	0.904509	0.9464134
64854	USP46	ubiquitin specific peptidase 46	1.00179	0.911401	1.0924843
64855	FAM129B	family with sequence similarity 129, member B	0.9726	1.096261	0.9762954
64856	VWA1	von Willebrand factor A domain containing 1	0.99862	1.066667	1.099144
64857	PLEKHG2	pleckstrin homology domain containing, family G (with RhoGef domain) member 2	0.99252	1.020761	0.8981471
64858	DCLRE1B	DNA cross-link repair 1B	0.9504	0.969489	1.0673117

64859	OBFC2A	oligonucleotide/oligosaccharide-binding fold containing 2A	1.0713	0.98466	0.8656263
64860	ARMCX5	armadillo repeat containing, X-linked 5	0.94851	0.984223	1.0761317
64863	METTL4	methyltransferase like 4	0.90656	0.989477	1.0684963
64864	RFX7	regulatory factor X, 7	0.96517	1.074667	1.0931014
64866	CDCP1	CUB domain containing protein 1	1.03031	1.017128	1.0241112
64881	PCDH20	protocadherin 20	0.99033	1.024725	0.989445
64895	PAPOLG	poly(A) polymerase gamma	0.98531	0.985557	1.0527711
64897	C12orf43	chromosome 12 open reading frame 43	0.89682	0.910341	1.0358065
64900	LPIN3	lipin 3	1.00168	0.991843	0.993246
64901	RANBP17	RAN binding protein 17	1.00396	1.007119	0.999953
64902	AGXT2	alanine--glyoxylate aminotransferase 2	1.00738	1.007205	0.9935068
64919	BCL11B	B-cell CLL/lymphoma 11B (zinc finger protein)	0.96621	1.069201	0.9843758
64921	CASD1	CAS1 domain containing 1	0.9734	0.882756	0.9497243
64922	LRRC19	leucine rich repeat containing 19	1.01105	1.025657	1.0153737
64924	SLC30A5	solute carrier family 30 (zinc transporter), member 5	0.99314	0.92544	0.9723544
64925	CCDC71	coiled-coil domain containing 71	0.96013	0.957469	0.9833028
64926	RASAL3	RAS protein activator like 3	0.94954	1.058778	1.0599587
64927	TTC23	tetratricopeptide repeat domain 23	1.00275	0.996467	1.0067856
64928	MRPL14	mitochondrial ribosomal protein L14	0.96556	0.954995	1.0415913
64939	SMNP	survival motor neuron pseudogene	1.01907	0.985472	0.9539876
64940	STAG3L4	stromal antigen 3-like 4	1.01373	0.966315	1.0384073
64943	NT5DC2	5'-nucleotidase domain containing 2	1.00372	1.022104	1.0433473
64946	CENPH	centromere protein H	0.99736	0.967151	1.0031606
64949	MRPS26	mitochondrial ribosomal protein S26	0.96832	1.027886	1.0523222
64951	MRPS24	mitochondrial ribosomal protein S24	0.99808	1.012511	1.0494714
64960	MRPS15	mitochondrial ribosomal protein S15	0.95658	1.002503	1.063027
64963	MRPS11	mitochondrial ribosomal protein S11	0.96511	1.011029	1.0478537
64965	MRPS9	mitochondrial ribosomal protein S9	0.9781	0.915696	1.0095875
64968	MRPS6	mitochondrial ribosomal protein S6	1.00032	1.081456	1.0057201
64969	MRPS5	mitochondrial ribosomal protein S5	1.03562	0.98436	1.0351191
64975	MRPL41	mitochondrial ribosomal protein L41	0.96789	0.95397	1.0221611
64976	MRPL40	mitochondrial ribosomal protein L40	0.94845	0.982933	0.9802737
64978	MRPL38	mitochondrial ribosomal protein L38	0.95852	0.970115	1.03273

64981	MRPL34	mitochondrial ribosomal protein L34	0.93545	0.934727	1.0502262
64983	MRPL32	mitochondrial ribosomal protein L32	0.9935	0.891262	0.9970164
65003	MRPL11	mitochondrial ribosomal protein L11	0.99012	0.981591	1.0154299
65005	MRPL9	mitochondrial ribosomal protein L9	0.96687	0.908461	0.994263
65008	MRPL1	mitochondrial ribosomal protein L1	0.97663	0.981981	1.010367
65009	NDRG4	NDRG family member 4	0.99042	1.010549	0.9964368
65010	SLC26A6	solute carrier family 26, member 6	1.02769	1.037957	1.0643959
65012	SLC26A10	solute carrier family 26, member 10	1.00361	1.004055	1.0024762
65018	PINK1	PTEN induced putative kinase 1	0.98426	0.98272	1.0160581
65055	REEP1	receptor accessory protein 1	1.00652	1.000827	1.0135003
65056	GPBP1	GC-rich promoter binding protein 1	0.98084	1.048739	0.8346065
65057	ACD	adrenocortical dysplasia homolog (mouse)	0.95811	1.060141	1.0166126
65059	RAPH1	Ras association (RalGDS/AF-6) and pleckstrin homology domains 1	1.00092	0.977356	0.9656807
65061	CDK15	cyclin-dependent kinase 15	1.02091	1.007465	1.0136781
65062	TMEM237	transmembrane protein 237	0.99811	0.983147	1.0205965
65065	NBEAL1	neurobeachin-like 1	0.98706	0.98775	1.026087
65078	RTN4R	reticulon 4 receptor	1.00047	0.969506	0.9941759
65080	MRPL44	mitochondrial ribosomal protein L44	0.96292	0.849398	1.0378272
65082	VPS33A	vacuolar protein sorting 33 homolog A (S. cerevisiae)	0.91843	0.974035	1.0480299
65083	NOL6	nucleolar protein family 6 (RNA-associated)	0.95752	0.950637	1.0063027
65084	TMEM135	transmembrane protein 135	0.97558	0.973254	0.9764452
65094	JMJD4	jumonji domain containing 4	0.95547	0.996385	1.0205601
65095	KRI1	KRI1 homolog (S. cerevisiae)	0.92901	1.025343	0.9713856
65108	MARCKSL1	MARCKS-like 1	0.97476	1.014877	1.0484041
65109	UPF3B	UPF3 regulator of nonsense transcripts homolog B (yeast)	0.9565	1.029304	0.9869594
65110	UPF3A	UPF3 regulator of nonsense transcripts homolog A (yeast)	0.99067	0.961755	1.0205758
65117	RSRC2	arginine/serine-rich coiled-coil 2	0.93061	0.912188	0.9642335
65123	INTS3	integrator complex subunit 3	0.99958	0.955108	1.0448674
65124	ANKRD57	ankyrin repeat domain 57	0.97318	0.967909	1.0512952
65125	WNK1	WNK lysine deficient protein kinase 1	0.9847	1.053682	1.0049519
65217	PCDH15	protocadherin-related 15	0.99409	0.998232	1.0147949
65220	NADK	NAD kinase	0.98461	1.024062	1.0410083
65243	ZNF643	zinc finger protein 643	0.97258	0.976571	0.9827216

65244	SPATS2	spermatogenesis associated, serine-rich 2	0.98002	0.984566	1.0096119
65249	ZSWIM4	zinc finger, SWIM-type containing 4	0.98821	1.02708	1.0281304
65250	C5orf42	chromosome 5 open reading frame 42	0.97496	0.927125	1.0008662
65251	ZNF649	zinc finger protein 649	0.9467	0.942881	1.039594
65258	MPPE1	metallophosphoesterase 1	1.00464	0.984492	0.9740017
65260	SELRC1	Sel1 repeat containing 1	0.9774	0.991637	1.1097053
65263	PYCRL	pyrroline-5-carboxylate reductase-like	0.9989	0.970149	0.9864382
65264	UBE2Z	ubiquitin-conjugating enzyme E2Z	0.92864	1.057125	1.1317859
65265	C8orf33	chromosome 8 open reading frame 33	0.98899	0.885489	1.0500877
65266	WNK4	WNK lysine deficient protein kinase 4	0.99187	1.010563	1.0375598
65267	WNK3	WNK lysine deficient protein kinase 3	1.00323	1.005201	1.0053659
65268	WNK2	WNK lysine deficient protein kinase 2	1.00156	1.001233	1.0250593
65944	OR2B8P	olfactory receptor, family 2, subfamily B, member 8 pseudogene	1.01534	1.034403	1.0091595
65975	STK33	serine/threonine kinase 33	0.98588	1.011111	1.0066743
65977	PLEKHA3	pleckstrin homology domain containing, family A (phosphoinositide binding specific) member 3	1.01524	0.97155	1.0054636
65979	PHACTR4	phosphatase and actin regulator 4	1.00111	1.00153	1.0114153
65980	BRD9	bromodomain containing 9	0.98299	0.967554	1.0255098
65981	CAPRIN2	caprin family member 2	0.93732	0.898252	0.9873699
65982	ZSCAN18	zinc finger and SCAN domain containing 18	0.96067	1.048242	0.97277
65983	GRAMD3	GRAM domain containing 3	0.98999	1.022995	0.9904278
65985	AACS	acetoacetyl-CoA synthetase	0.98785	0.967091	1.0054178
65986	ZBTB10	zinc finger and BTB domain containing 10	1.00352	1.004037	0.9462156
65987	KCTD14	potassium channel tetramerisation domain containing 14	0.99293	0.987895	1.0236721
65988	ZNF747	zinc finger protein 747	0.9432	0.977163	1.0923005
65989	DLK2	delta-like 2 homolog (Drosophila)	1.03021	1.005859	1.0274345
65990	FAM173A	family with sequence similarity 173, member A	0.97085	1.018675	1.1485844
65991	FUNDC2	FUN14 domain containing 2	0.9921	0.975311	1.0407019
65992	DDRGK1	DDRGK domain containing 1	0.98769	0.977357	1.0369566
65993	MRPS34	mitochondrial ribosomal protein S34	0.88555	1.005823	1.0469094
65997	RASL11B	RAS-like, family 11, member B	1.01052	0.989662	1.021256
65998	C11orf95	chromosome 11 open reading frame 95	0.97879	1.031308	1.0750517
65999	LRRC61	leucine rich repeat containing 61	0.99645	0.927641	0.9921449
66002	CYP4F12	cytochrome P450, family 4, subfamily F, polypeptide 12	1.02495	1.046972	0.9473552

66004	LYNX1	Ly6/neurotoxin 1	1.01031	1.005748	1.0053137
66005	CHID1	chitinase domain containing 1	0.99159	0.953548	1.0528642
66008	TRAK2	trafficking protein, kinesin binding 2	0.99801	0.895287	0.9932749
66035	SLC2A11	solute carrier family 2 (facilitated glucose transporter), member 11	0.98328	0.989628	1.0335877
66036	MTMR9	myotubularin related protein 9	0.99111	0.970627	1.0446196
66037	BOLL	bol, boule-like (Drosophila)	0.98606	1.019959	1.0061914
78986	DUSP26	dual specificity phosphatase 26 (putative)	0.98544	0.995094	0.9933402
78987	CRELD1	cysteine-rich with EGF-like domains 1	0.99108	1.004075	1.0320588
78988	MRP63	mitochondrial ribosomal protein 63	0.93268	0.921915	0.9809721
78989	COLEC11	collectin sub-family member 11	1.00402	1.011611	1.0232515
78990	OTUB2	OTU domain, ubiquitin aldehyde binding 2	0.99196	0.966833	0.9948485
78991	PCYOX1L	prenylcysteine oxidase 1 like	0.99544	0.912651	0.90105
78992	YIPF2	Yip1 domain family, member 2	0.96442	0.9833	1.0344034
78994	PRR14	proline rich 14	0.94884	1.035594	0.9753921
78995	C17orf53	chromosome 17 open reading frame 53	1.00036	1.010517	0.9980987
78996	C7orf49	chromosome 7 open reading frame 49	0.97896	0.981096	0.965713
78997	GDAP1L1	ganglioside-induced differentiation-associated protein 1-like 1	0.99382	1.0058	0.9946621
78998	C8orf51	chromosome 8 open reading frame 51	1.03852	1.004338	0.9612238
78999	LRFN4	leucine rich repeat and fibronectin type III domain containing 4	1.01137	0.969127	1.0493871
79000	C1orf135	chromosome 1 open reading frame 135	1.00276	0.971497	1.0016941
79001	VKORC1	vitamin K epoxide reductase complex, subunit 1	0.98867	0.981318	1.0759488
79002	C19orf43	chromosome 19 open reading frame 43	0.97041	1.026287	1.0886804
79003	MIS12	MIS12, MIND kinetochore complex component, homolog (S. pombe)	0.92651	0.964573	1.0104522
79004	CUEDC2	CUE domain containing 2	0.96801	0.973021	1.0225733
79005	SCNM1	sodium channel modifier 1	0.96233	1.040532	1.0418458
79006	METRN	meteorin, glial cell differentiation regulator	0.96833	0.959633	1.1071732
79007	DBNDD1	dysbindin (dystrobrevin binding protein 1) domain containing 1	0.98208	0.96924	1.0315789
79009	DDX50	DEAD (Asp-Glu-Ala-Asp) box polypeptide 50	0.95914	0.989788	0.9747059
79012	CAMKV	CaM kinase-like vesicle-associated	1.00564	0.994745	1.0113124
79016	DDA1	DET1 and DDB1 associated 1	0.98389	0.945205	0.9982189
79017	GGCT	gamma-glutamylcyclotransferase	1.00091	1.009193	1.0419167
79018	C17orf39	chromosome 17 open reading frame 39	0.94959	0.955592	1.0416985
79019	CENPM	centromere protein M	1.00526	0.987074	0.9920489

79020	C7orf25	chromosome 7 open reading frame 25	0.95374	0.973802	1.0345586
79022	TMEM106C	transmembrane protein 106C	0.86511	0.858282	1.0670534
79023	NUP37	nucleoporin 37kDa	0.95361	0.920078	1.0370033
79025	C20orf195	chromosome 20 open reading frame 195	0.99874	0.95601	1.0206808
79026	AHNAK	AHNAK nucleoprotein	1.00504	1.010318	1.0744891
79027	ZNF655	zinc finger protein 655	0.99504	0.969429	1.0031695
79029	SPATA5L1	spermatogenesis associated 5-like 1	0.95936	1.059859	1.0209975
79031	PDCL3	phosducin-like 3	1.02665	0.941538	1.0566734
79033	ERI3	ERI1 exoribonuclease family member 3	0.99063	0.999094	0.9869657
79034	C7orf26	chromosome 7 open reading frame 26	0.99969	0.925963	0.9980473
79035	OBFC2B	oligonucleotide/oligosaccharide-binding fold containing 2B	0.98063	0.961336	1.0358044
79036	C19orf50	chromosome 19 open reading frame 50	0.97921	0.979118	0.9660523
79037	PVRIG	poliovirus receptor related immunoglobulin domain containing	0.9641	0.96837	0.966577
79038	ZFYVE21	zinc finger, FYVE domain containing 21	0.97383	0.966967	1.090393
79039	DDX54	DEAD (Asp-Glu-Ala-Asp) box polypeptide 54	0.95898	0.916568	1.108607
79041	TMEM38A	transmembrane protein 38A	0.98759	0.961254	1.0505098
79042	TSEN34	tRNA splicing endonuclease 34 homolog (S. cerevisiae)	0.9765	1.044484	0.9968803
79047	KCTD15	potassium channel tetramerisation domain containing 15	0.98235	0.940904	1.1099258
79048	SECISBP2	SECIS binding protein 2	0.92542	0.788227	0.9182648
79050	NOC4L	nucleolar complex associated 4 homolog (S. cerevisiae)	0.96022	0.928958	1.0292905
79053	ALG8	asparagine-linked glycosylation 8, alpha-1,3-glucosyltransferase homolog (S. cerevisiae)	0.96038	0.941259	1.0238026
79054	TRPM8	transient receptor potential cation channel, subfamily M, member 8	1.00489	0.996237	1.0129029
79056	PRRG4	proline rich Gla (G-carboxyglutamic acid) 4 (transmembrane)	0.96733	0.897063	1.0849778
79057	PRRG3	proline rich Gla (G-carboxyglutamic acid) 3 (transmembrane)	0.98795	1.00454	1.0276656
79058	ASPSCR1	alveolar soft part sarcoma chromosome region, candidate 1	0.96909	1.013782	1.0900158
79064	TMEM223	transmembrane protein 223	0.92952	0.932763	0.9291472
79065	ATG9A	ATG9 autophagy related 9 homolog A (S. cerevisiae)	0.97871	0.990821	0.9895763
79066	METTL16	methyltransferase like 16	0.91653	0.912809	0.9985452
79068	FTO	fat mass and obesity associated	0.99314	0.997863	1.0524252
79070	KDELC1	KDEL (Lys-Asp-Glu-Leu) containing 1	0.99078	0.982022	1.0133878
79071	ELOVL6	ELOVL fatty acid elongase 6	1.01303	1.012193	1.0175985
79072	FASTKD3	FAST kinase domains 3	0.96371	0.988341	1.0742303
79073	TMEM109	transmembrane protein 109	0.96546	0.894302	1.0173026

79074	C2orf49	chromosome 2 open reading frame 49	0.96205	0.902494	1.0407703
79075	DSCC1	defective in sister chromatid cohesion 1 homolog (S. cerevisiae)	0.98566	0.957456	0.9940462
79077	DCTPP1	dCTP pyrophosphatase 1	0.98699	0.921408	1.0542553
79078	C1orf50	chromosome 1 open reading frame 50	0.97188	0.898916	1.001324
79080	CCDC86	coiled-coil domain containing 86	0.96297	0.97049	1.0666377
79081	C11orf48	chromosome 11 open reading frame 48	0.962	1.008294	1.0603272
79083	MLPH	melanophilin	1.00073	1.010515	1.0257384
79084	WDR77	WD repeat domain 77	1.01464	0.912973	1.0162137
79085	SLC25A23	solute carrier family 25 (mitochondrial carrier; phosphate carrier), member 23	0.97158	0.966607	1.0125292
79086	C19orf42	chromosome 19 open reading frame 42	0.93808	0.934393	1.0379104
79087	ALG12	asparagine-linked glycosylation 12, alpha-1,6-mannosyltransferase homolog (S. cerevisiae)	0.93838	0.972568	0.9924124
79088	ZNF426	zinc finger protein 426	0.91092	0.959341	0.970727
79089	TMUB2	transmembrane and ubiquitin-like domain containing 2	0.94126	0.95523	0.9829563
79090	TRAPPC6A	trafficking protein particle complex 6A	0.9662	0.997309	1.0495561
79091	METTL22	methyltransferase like 22	0.98233	0.924956	1.0055343
79092	CARD14	caspase recruitment domain family, member 14	0.99817	1.02522	1.0310219
79094	CHAC1	ChaC, cation transport regulator homolog 1 (E. coli)	1.00452	1.023885	1.0128276
79095	C9orf16	chromosome 9 open reading frame 16	0.95245	0.992304	1.0342468
79096	C11orf49	chromosome 11 open reading frame 49	0.98356	1.018508	1.0268316
79098	C1orf116	chromosome 1 open reading frame 116	0.97513	0.957296	0.9806694
79101	TAF1D	TATA box binding protein (TBP)-associated factor, RNA polymerase I, D, 41kDa	0.92102	0.98946	1.0130693
79102	RNF26	ring finger protein 26	0.94888	0.915635	1.1464498
79109	MAPKAP1	mitogen-activated protein kinase associated protein 1	0.97323	0.976082	1.0067469
79132	DHX58	DEXH (Asp-Glu-X-His) box polypeptide 58	0.93606	0.907074	1.0448105
79133	C20orf7	chromosome 20 open reading frame 7	0.96893	1.014123	1.0342687
79134	TMEM185B	transmembrane protein 185B	0.95259	0.946401	0.9567791
79135	APOO	apolipoprotein O	0.99356	0.976269	1.0109227
79136	LY6G6E	lymphocyte antigen 6 complex, locus G6E	1.01072	0.986461	0.9532109
79137	FAM134A	family with sequence similarity 134, member A	0.9964	0.933001	1.0345801
79139	DERL1	Der1-like domain family, member 1	0.94942	1.007115	1.1100307
79140	CCDC28B	coiled-coil domain containing 28B	1.01034	1.035363	1.0133638
79142	PHF23	PHD finger protein 23	0.87044	0.984238	1.0475613
79143	MBOAT7	membrane bound O-acyltransferase domain containing 7	0.99002	1.042562	0.9960327

79144	PPDPF	pancreatic progenitor cell differentiation and proliferation factor homolog (zebrafish)	0.97039	0.945297	0.894267
79145	CHCHD7	coiled-coil-helix-coiled-coil-helix domain containing 7	0.99567	0.897421	0.9802118
79147	FKRP	fukutin related protein	0.96576	1.016746	0.9951695
79148	MMP28	matrix metallopeptidase 28	0.98444	1.011003	1.0030589
79149	ZSCAN5A	zinc finger and SCAN domain containing 5A	0.98993	1.073361	1.0900361
79152	FA2H	fatty acid 2-hydroxylase	1.00091	0.991196	1.0094771
79153	GDPD3	glycerophosphodiester phosphodiesterase domain containing 3	0.96122	0.95597	1.0268355
79154	DHRS11	dehydrogenase/reductase (SDR family) member 11	0.99439	0.97236	1.0355757
79155	TNIP2	TNFAIP3 interacting protein 2	1.004	1.023661	1.0038726
79156	PLEKHF1	pleckstrin homology domain containing, family F (with FYVE domain) member 1	0.9536	1.031245	1.0558272
79157	MFSD11	major facilitator superfamily domain containing 11	0.94978	0.993249	1.0081589
79158	GNPTAB	N-acetylglucosamine-1-phosphate transferase, alpha and beta subunits	0.93402	1.031257	0.9346213
79159	NOL12	nucleolar protein 12	0.93711	0.937843	1.074998
79160	MGC4294	hypothetical MGC4294	1.00848	0.985365	0.9749822
79161	C7orf23	chromosome 7 open reading frame 23	0.96823	0.916978	0.9929545
79165	LENG1	leukocyte receptor cluster (LRC) member 1	0.95737	1.043329	0.9737391
79166	LILRP2	leukocyte immunoglobulin-like receptor pseudogene 2	0.97395	0.974329	1.0297711
79167	LILRP1	leukocyte immunoglobulin-like receptor pseudogene 1	0.99428	1.072765	1.0455112
79168	LILRA6	leukocyte immunoglobulin-like receptor, subfamily A (with TM domain), member 6	1.0095	1.105932	1.0595993
79169	C1orf35	chromosome 1 open reading frame 35	0.99816	1.011044	1.0344665
79170	PRR15L	proline rich 15-like	1.00148	0.965913	1.0173155
79171	RBM42	RNA binding motif protein 42	0.93296	0.966573	1.0372265
79172	CENPO	centromere protein O	0.98182	0.981085	1.0216585
79173	C19orf57	chromosome 19 open reading frame 57	0.99518	1.007536	1.0031328
79174	CRELD2	cysteine-rich with EGF-like domains 2	0.96066	0.98371	1.0896738
79175	ZNF343	zinc finger protein 343	0.93709	1.007686	1.032127
79176	FBXL15	F-box and leucine-rich repeat protein 15	0.97124	0.970111	1.0534203
79177	ZNF576	zinc finger protein 576	0.94172	1.019807	1.0413849
79178	THTPA	thiamine triphosphatase	0.96852	0.977447	1.0030214
79180	EFHD2	EF-hand domain family, member D2	1.03572	0.96916	0.990002
79183	TTPAL	tocopherol (alpha) transfer protein-like	0.9122	0.976523	1.0480178
79184	BRCC3	BRCA1/BRCA2-containing complex, subunit 3	0.97303	0.98654	1.026068
79187	FSD1	fibronectin type III and SPRY domain containing 1	0.96819	1.001918	1.0283889

79188	TMEM43	transmembrane protein 43	0.97908	0.930226	1.1262891
79190	IRX6	iroquois homeobox 6	1.00646	0.989716	1.0139895
79191	IRX3	iroquois homeobox 3	1.00744	0.99528	1.0715401
79192	IRX1	iroquois homeobox 1	0.97519	1.004335	0.9902049
79228	THOC6	THO complex 6 homolog (Drosophila)	0.97707	0.912528	1.100423
79230	ZNF557	zinc finger protein 557	0.93118	0.954208	1.0626713
79231	ZNF33AP1	zinc finger protein 33A pseudogene 1	0.98878	1.019242	1.0362004
79232	ZNF37C	zinc finger protein 37C (pseudogene)	1.03754	1.044244	0.9682944
79258	MMEL1	membrane metallo-endopeptidase-like 1	1.00445	1.017376	1.0289953
79269	DCAF10	DDB1 and CUL4 associated factor 10	0.94529	0.947626	0.9799465
79272	OR52X1P	olfactory receptor, family 52, subfamily X, member 1 pseudogene	1.00999	1.022082	0.9868722
79274	OR52L2P	olfactory receptor, family 52, subfamily L, member 2 pseudogene	1.00197	0.997217	0.9976826
79283	OR5P1P	olfactory receptor, family 5, subfamily P, member 1 pseudogene	0.97853	1.010894	0.9728373
79290	OR13A1	olfactory receptor, family 13, subfamily A, member 1	1.00107	1.017401	1.0161269
79291	OR5H7P	olfactory receptor, family 5, subfamily H, member 7 pseudogene	1.04171	0.984281	1.0571452
79295	OR5H6	olfactory receptor, family 5, subfamily H, member 6	0.95848	0.996465	1.0130458
79296	OR52E1	olfactory receptor, family 52, subfamily E, member 1 (gene/pseudogene)	1.00705	1.029798	1.0611921
79300	OR51P1P	olfactory receptor, family 51, subfamily P, member 1 pseudogene	1.01308	1.019435	1.0085832
79302	OR52J1P	olfactory receptor, family 52, subfamily J, member 1 pseudogene	1.02301	1.057387	0.9369795
79308	OR4P1P	olfactory receptor, family 4, subfamily P, member 1 pseudogene	1.00083	1.023049	1.0019733
79310	OR5H2	olfactory receptor, family 5, subfamily H, member 2	1.00527	1.001342	1.0619889
79312	OR1AA1P	olfactory receptor, family 1, subfamily AA, member 1 pseudogene	1.00455	1.004115	0.9632176
79313	OR2AD1P	olfactory receptor, family 2, subfamily AD, member 1 pseudogene	1.00936	0.982794	0.9989761
79314	OR4K6P	olfactory receptor, family 4, subfamily K, member 6 pseudogene	1.02559	1.061438	1.0241326
79315	OR7E91P	olfactory receptor, family 7, subfamily E, member 91 pseudogene	1.01432	1.028151	1.0006576
79316	OR8B9P	olfactory receptor, family 8, subfamily B, member 9 pseudogene	0.98327	0.958923	1.0162755
79317	OR4K5	olfactory receptor, family 4, subfamily K, member 5	1.01667	1.005156	0.9601946
79318	OR4K4P	olfactory receptor, family 4, subfamily K, member 4 pseudogene	1.00334	0.999733	1.0300829
79321	OR6M3P	olfactory receptor, family 6, subfamily M, member 3 pseudogene	0.99693	1.012845	0.9890066
79323	OR6E1P	olfactory receptor, family 6, subfamily E, member 1 pseudogene	0.98939	0.990976	1.0143836
79324	OR51G1	olfactory receptor, family 51, subfamily G, member 1	1.03381	1.098832	1.0117474
79331	OR2AF1P	olfactory receptor, family 2, subfamily AF, member 1 pseudogene	0.98161	0.961174	1.0126531
79336	OR5AH1P	olfactory receptor, family 5, subfamily AH, member 1 pseudogene	1.01083	0.996659	0.9969631

79339	OR51B4	olfactory receptor, family 51, subfamily B, member 4	0.98919	0.97238	0.9555641
79342	OR51B3P	olfactory receptor, family 51, subfamily B, member 3 pseudogene	0.98696	1.017873	1.0112956
79345	OR51B2	olfactory receptor, family 51, subfamily B, member 2	0.98752	0.974831	0.990442
79346	OR4C5	olfactory receptor, family 4, subfamily C, member 5	1.05538	1.199917	1.0007184
79363	RSG1	REM2 and RAB-like small GTPase 1	0.98249	0.987076	0.9388534
79364	ZXDC	ZXD family zinc finger C	0.99899	0.938331	1.0030836
79365	BHLHE41	basic helix-loop-helix family, member e41	0.9864	0.980801	1.0093187
79366	HMGN5	high mobility group nucleosome binding domain 5	0.99365	1.010506	1.0041091
79369	B3GNT4	UDP-GlcNAc:betaGal beta-1,3-N-acetylglucosaminyltransferase 4	1.00873	0.991485	0.9646276
79370	BCL2L14	BCL2-like 14 (apoptosis facilitator)	0.98873	0.979827	0.9957379
79400	NOX5	NADPH oxidase, EF-hand calcium binding domain 5	0.99837	0.971301	1.0289135
79411	GLB1L	galactosidase, beta 1-like	0.98476	0.945757	1.0228061
79412	KREMEN2	kringle containing transmembrane protein 2	0.98664	1.004556	1.0981962
79413	ZBED2	zinc finger, BED-type containing 2	1.03314	1.069648	0.9074244
79414	LRFN3	leucine rich repeat and fibronectin type III domain containing 3	0.9516	1.00159	1.0303533
79415	C17orf62	chromosome 17 open reading frame 62	0.97484	0.983827	1.0818083
79441	HAUS3	HAUS augmin-like complex, subunit 3	0.99191	0.921662	0.9621251
79442	LRRC2	leucine rich repeat containing 2	1.00335	0.987465	1.0173487
79443	FYCO1	FYVE and coiled-coil domain containing 1	0.9865	0.987763	1.004081
79444	BIRC7	baculoviral IAP repeat containing 7	0.95987	0.971666	1.0303314
79446	WDR25	WD repeat domain 25	0.98778	1.021435	0.991513
79465	ULBP3	UL16 binding protein 3	0.98913	1.000588	1.0226996
79470	OR51J1	olfactory receptor, family 51, subfamily J, member 1 (gene/pseudogene)	0.98311	0.979235	1.0650047
79473	OR52N1	olfactory receptor, family 52, subfamily N, member 1	0.9765	0.981485	1.0128001
79476	OR5AL2P	olfactory receptor, family 5, subfamily AL, member 2 pseudogene	0.98434	1.005202	1.0368324
79482	OR5AL1	olfactory receptor, family 5, subfamily AL, member 1 (gene/pseudogene)	1.00881	0.967349	0.9429583
79483	OR5BD1P	olfactory receptor, family 5, subfamily BD, member 1 pseudogene	0.97077	0.995124	1.0365362
79488	OR10D5P	olfactory receptor, family 10, subfamily D, member 5 pseudogene	1.03702	0.948764	0.9627806
79490	OR10G6	olfactory receptor, family 10, subfamily G, member 6	1.03202	1.013316	1.005554
79498	OR8I1P	olfactory receptor, family 8, subfamily I, member 1 pseudogene	1.00104	1.017119	0.984267
79499	OR7L1P	olfactory receptor, family 7, subfamily L, member 1 pseudogene	1.03927	0.946661	1.0182218
79508	OR5M7P	olfactory receptor, family 5, subfamily M, member 7 pseudogene	0.99365	0.944712	0.9932643
79512	OR5M6P	olfactory receptor, family 5, subfamily M, member 6 pseudogene	1.00305	1.027894	0.9906614

79514	OR10T1P	olfactory receptor, family 10, subfamily T, member 1 pseudogene	1.01843	1.027524	0.9808659
79515	OR10G5P	olfactory receptor, family 10, subfamily G, member 5 pseudogene	0.97585	1.028073	0.9924633
79521	OR52S1P	olfactory receptor, family 52, subfamily S, member 1 pseudogene	0.98642	1.027995	0.9370249
79523	OR5M2P	olfactory receptor, family 5, subfamily M, member 2 pseudogene	0.98823	1.012763	0.981763
79525	OR51A5P	olfactory receptor, family 51, subfamily A, member 5 pseudogene	1.00541	0.976651	0.9815008
79526	OR4C10P	olfactory receptor, family 4, subfamily C, member 10 pseudogene	0.99125	1.005822	0.9776098
79530	OR4C9P	olfactory receptor, family 4, subfamily C, member 9 pseudogene	0.97692	0.929811	1.0340461
79532	OR51A3P	olfactory receptor, family 51, subfamily A, member 3 pseudogene	0.95036	1.013864	1.0367049
79533	OR52E3P	olfactory receptor, family 52, subfamily E, member 3 pseudogene	0.98365	0.953345	0.9715039
79538	OR13I1P	olfactory receptor, family 13, subfamily I, member 1 pseudogene	0.96688	0.966373	1.0294031
79544	OR4K1	olfactory receptor, family 4, subfamily K, member 1	0.99463	1.041028	0.9668944
79546	OR6M2P	olfactory receptor, family 6, subfamily M, member 2 pseudogene	1.00084	1.014407	0.9718559
79547	OR2AL1P	olfactory receptor, family 2, subfamily AL, member 1 pseudogene	1.00222	1.054741	0.8884397
79549	OR6J1	olfactory receptor, family 6, subfamily J, member 1	1.0088	0.969005	0.9638894
79550	OR4C4P	olfactory receptor, family 4, subfamily C, member 4 pseudogene	1.01095	1.022294	1.0466123
79567	FAM65A	family with sequence similarity 65, member A	0.98192	1.067405	0.9696461
79568	C2orf47	chromosome 2 open reading frame 47	0.99674	0.953254	1.0223686
79570	NKAIN1	Na+/K+ transporting ATPase interacting 1	1.01226	0.998576	1.0284739
79571	GCC1	GRIP and coiled-coil domain containing 1	0.93945	0.965774	1.0521848
79572	ATP13A3	ATPase type 13A3	0.97613	1.036392	0.9981753
79573	TTC13	tetratricopeptide repeat domain 13	1.00926	0.955724	0.9985212
79574	EPS8L3	EPS8-like 3	1.00799	1.041109	1.0295652
79575	ABHD8	abhydrolase domain containing 8	0.96589	0.95613	1.0192853
79576	NKAP	NFKB activating protein	0.91908	0.976011	0.9964728
79577	CDC73	cell division cycle 73, Paf1/RNA polymerase II complex component, homolog (S. cerevisiae)	0.96382	0.962138	0.9942238
79581	GPR172A	G protein-coupled receptor 172A	0.98571	0.93925	1.0603582
79582	SPAG16	sperm associated antigen 16	1.00751	1.002384	1.0185408
79583	TMEM231	transmembrane protein 231	0.97608	1.054368	1.0218204
79585	CORO7	coronin 7	0.98529	1.015383	0.9894564
79586	CHPF	chondroitin polymerizing factor	1.00143	0.988165	1.0056119
79587	CARS2	cysteinyl-tRNA synthetase 2, mitochondrial (putative)	0.96434	0.961364	1.0100937
79589	RNF128	ring finger protein 128	0.98814	1.019519	1.0186089
79590	MRPL24	mitochondrial ribosomal protein L24	0.97071	0.938344	1.0254714

79591	C10orf76	chromosome 10 open reading frame 76	0.95538	0.973949	1.0188986
79594	MUL1	mitochondrial E3 ubiquitin protein ligase 1	0.96047	0.851573	1.0881257
79595	SAP130	Sin3A-associated protein, 130kDa	0.98778	0.959769	1.0076796
79596	RNF219	ring finger protein 219	0.92966	1.017707	1.0777278
79598	CEP97	centrosomal protein 97kDa	0.9977	0.96973	1.0776584
79600	TCTN1	tectonic family member 1	0.95561	1.007289	1.0015783
79602	ADIPOR2	adiponectin receptor 2	0.95465	1.034612	1.0305646
79603	LASS4	LAG1 homolog, ceramide synthase 4	0.99627	0.983988	1.0065413
79605	PGBD5	piggyBac transposable element derived 5	1.00291	1.009241	1.0197383
79607	FAM118B	family with sequence similarity 118, member B	0.98556	0.942466	1.0132756
79608	RIC3	resistance to inhibitors of cholinesterase 3 homolog (C. elegans)	0.95456	0.956495	1.0016739
79609	METTL21D	methyltransferase like 21D	0.98964	0.998431	0.9245624
79611	ACSS3	acyl-CoA synthetase short-chain family member 3	0.99759	1.009913	0.9933073
79612	NAA16	N(alpha)-acetyltransferase 16, NatA auxiliary subunit	0.91233	0.932016	1.02727
79613	TMCO7	transmembrane and coiled-coil domains 7	0.94114	0.97353	0.9943973
79614	[No Symbo	[No Name]	0.99293	0.948599	0.9454642
79616	CCNJL	cyclin J-like	1.01161	0.986023	0.9989683
79618	HMBOX1	homeobox containing 1	1.00955	1.044585	0.9956997
79621	RNASEH2B	ribonuclease H2, subunit B	0.91801	1.056662	0.9397652
79622	SNRNP25	small nuclear ribonucleoprotein 25kDa (U11/U12)	0.98078	0.978472	1.0204377
79624	C6orf211	chromosome 6 open reading frame 211	0.98898	0.93947	1.055004
79625	C4orf31	chromosome 4 open reading frame 31	0.9871	1.001355	0.9764163
79626	TNFAIP8L2	tumor necrosis factor, alpha-induced protein 8-like 2	0.93874	1.015931	1.0185071
79627	OGFRL1	opioid growth factor receptor-like 1	1.01738	0.93521	0.9635001
79628	SH3TC2	SH3 domain and tetratricopeptide repeats 2	1.00674	0.964995	0.9925341
79629	OCEL1	occludin/ELL domain containing 1	0.95612	0.887225	1.0687156
79630	C1orf54	chromosome 1 open reading frame 54	1.03213	0.958231	0.9931394
79631	EFTUD1	elongation factor Tu GTP binding domain containing 1	0.94047	1.046355	1.0005351
79632	FAM184A	family with sequence similarity 184, member A	0.98341	1.000454	0.9908108
79633	FAT4	FAT tumor suppressor homolog 4 (Drosophila)	0.98786	0.993855	1.0057127
79634	SCRN3	secernin 3	0.97789	0.930148	1.0042745
79635	CCDC121	coiled-coil domain containing 121	1.00173	1.032491	1.0213203
79637	ARMC7	armadillo repeat containing 7	0.95463	0.985442	1.0290212

79639	TMEM53	transmembrane protein 53	1.00817	1.031899	1.0035026
79640	C22orf46	chromosome 22 open reading frame 46	0.96368	0.948623	0.9606749
79641	ROGDI	rogdi homolog (Drosophila)	0.96047	0.983258	1.0881299
79642	ARSJ	arylsulfatase family, member J	0.99425	0.986972	1.0031096
79643	CHMP6	chromatin modifying protein 6	0.94393	0.942248	1.0134994
79644	SRD5A3	steroid 5 alpha-reductase 3	1.01494	1.017937	1.0095269
79645	EFCAB1	EF-hand calcium binding domain 1	0.98746	1.022086	0.9880963
79646	PANK3	pantothenate kinase 3	0.98559	1.0243	1.0734833
79647	AKIRIN1	akirin 1	0.98941	1.01075	1.0639202
79648	MCPH1	microcephalin 1	0.98818	0.985958	1.0077576
79649	MAP7D3	MAP7 domain containing 3	0.95334	0.927453	1.0266
79650	C16orf57	chromosome 16 open reading frame 57	0.9987	0.993445	1.0633995
79651	RHBDF2	rhomboid 5 homolog 2 (Drosophila)	1	1.101671	0.9772419
79652	TMEM204	transmembrane protein 204	0.948	0.96805	0.9827005
79654	HECTD3	HECT domain containing 3	0.96785	0.927001	0.9740716
79657	RPAP3	RNA polymerase II associated protein 3	0.94971	1.014321	1.04235
79658	ARHGAP10	Rho GTPase activating protein 10	0.99956	0.983248	1.0174898
79659	DYNC2H1	dynein, cytoplasmic 2, heavy chain 1	0.97432	1.001617	0.9763978
79660	PPP1R3B	protein phosphatase 1, regulatory (inhibitor) subunit 3B	0.96008	0.971997	1.034189
79661	NEIL1	nei endonuclease VIII-like 1 (E. coli)	0.99398	1.02659	1.028722
79663	HSPBAP1	HSPB (heat shock 27kDa) associated protein 1	0.98201	0.942631	0.9185089
79664	NARG2	NMDA receptor regulated 2	0.92404	0.911402	1.0598059
79665	DHX40	DEAH (Asp-Glu-Ala-His) box polypeptide 40	0.93885	1.000412	0.954204
79666	PLEKHF2	pleckstrin homology domain containing, family F (with FYVE domain) member 2	0.97646	0.964918	1.0837881
79667	FLJ13197	hypothetical FLJ13197	0.9721	1.010344	0.9611042
79668	PARP8	poly (ADP-ribose) polymerase family, member 8	1.00387	1.013178	0.9639239
79669	C3orf52	chromosome 3 open reading frame 52	0.99105	1.021331	1.0035944
79670	ZCCHC6	zinc finger, CCHC domain containing 6	0.97623	1.022106	0.9708167
79671	NLRX1	NLR family member X1	0.96341	0.93884	1.059799
79672	FN3KRP	fructosamine 3 kinase related protein	0.94258	0.948399	0.9924624
79673	ZNF329	zinc finger protein 329	0.95791	0.968324	0.9993246
79674	VEPH1	ventricular zone expressed PH domain homolog 1 (zebrafish)	1.01121	0.954833	0.9862805
79675	FASTKD1	FAST kinase domains 1	0.98622	0.971937	0.9954063

79676	OGFOD2	2-oxoglutarate and iron-dependent oxygenase domain containing 2	0.98987	0.947849	0.9858589
79677	SMC6	structural maintenance of chromosomes 6	1.02395	1.003244	1.003367
79679	VTCN1	V-set domain containing T cell activation inhibitor 1	1.00791	1.018043	1.0010018
79682	MLF1IP	MLF1 interacting protein	0.98098	0.970497	1.0503538
79683	ZDHHC14	zinc finger, DHHC-type containing 14	1.00162	0.998119	0.9987569
79684	C11orf61	chromosome 11 open reading frame 61	0.96617	1.052336	1.0878491
79685	SAP30L	SAP30-like	0.99343	0.932599	1.0234369
79686	C14orf139	chromosome 14 open reading frame 139	0.97637	0.927198	1.0772788
79689	STEAP4	STEAP family member 4	1.09072	1.068966	0.9340294
79690	GAL3ST4	galactose-3-O-sulfotransferase 4	0.97895	1.001654	0.9595548
79691	QTRTD1	queuine tRNA-ribosyltransferase domain containing 1	0.9873	0.888768	1.1111276
79692	ZNF322A	zinc finger protein 322A	1.01199	1.041539	1.0115759
79693	YRDC	yrdC domain containing (E. coli)	1.00656	0.990535	1.0405926
79694	MANEA	mannosidase, endo-alpha	0.99651	0.934626	1.036234
79695	GALNT12	UDP-N-acetyl-alpha-D-galactosamine:polypeptide N-acetylgalactosaminyltransferase 12 (GalNAc-T12)	0.96854	0.977768	0.9634326
79696	FAM164C	family with sequence similarity 164, member C	1.02225	1.025581	0.9749376
79697	C14orf169	chromosome 14 open reading frame 169	0.92705	0.933955	1.0436996
79698	ZMAT4	zinc finger, matrin-type 4	0.99181	0.982118	0.9764466
79699	ZYG11B	zyg-11 homolog B (C. elegans)	0.94208	0.958797	0.9996466
79701	C17orf101	chromosome 17 open reading frame 101	0.98194	0.985594	1.0251225
79703	C11orf80	chromosome 11 open reading frame 80	0.9705	0.996051	1.0456382
79705	LRRK1	leucine-rich repeat kinase 1	0.99742	0.923679	1.0403195
79706	PRKRIP1	PRKR interacting protein 1 (IL11 inducible)	0.97877	0.980734	0.9613512
79707	NOL9	nucleolar protein 9	0.96845	0.891519	1.0161826
79709	GLT25D1	glycosyltransferase 25 domain containing 1	0.95358	0.90534	1.0464283
79710	MORC4	MORC family CW-type zinc finger 4	0.95942	0.973873	0.9583965
79711	IPO4	importin 4	0.93038	0.954539	1.0697108
79712	GTDC1	glycosyltransferase-like domain containing 1	1.00316	1.00762	1.0014674
79713	IGFLR1	IGF-like family receptor 1	0.95924	0.928757	1.0538091
79714	CCDC51	coiled-coil domain containing 51	0.99443	0.959652	1.0272942
79716	NPEPL1	aminopeptidase-like 1	0.95922	1.0254	1.0522045
79717	PPCS	phosphopantothenoylcysteine synthetase	0.98273	0.891471	1.0484352
79718	TBL1XR1	transducin (beta)-like 1 X-linked receptor 1	1.00417	1.023379	0.9903516

79719	AAGAB	alpha- and gamma-adaptin binding protein	0.96419	0.964933	0.9823837
79720	VPS37B	vacuolar protein sorting 37 homolog B (S. cerevisiae)	0.98484	1.035895	0.9608079
79722	ANKRD55	ankyrin repeat domain 55	0.98561	0.971246	0.9724645
79723	SUV39H2	suppressor of variegation 3-9 homolog 2 (Drosophila)	0.98137	0.962245	0.9922326
79724	ZNF768	zinc finger protein 768	0.94696	0.932175	1.0263474
79725	THAP9	THAP domain containing 9	0.97386	1	0.9388187
79726	WDR59	WD repeat domain 59	0.95675	1.023991	1.0011403
79727	LIN28A	lin-28 homolog A (C. elegans)	0.97358	0.99354	1.0261881
79728	PALB2	partner and localizer of BRCA2	0.95412	0.985543	1.0634173
79729	SH3D21	SH3 domain containing 21	1.00663	0.991657	0.995632
79730	NSUN7	NOP2/Sun domain family, member 7	0.9906	0.990092	1.0105009
79731	NARS2	asparaginyl-tRNA synthetase 2, mitochondrial (putative)	0.93853	0.980657	0.9888089
79733	E2F8	E2F transcription factor 8	1.01087	1.004946	1.0153013
79734	KCTD17	potassium channel tetramerisation domain containing 17	0.98952	0.971062	1.0869711
79735	TBC1D17	TBC1 domain family, member 17	0.95172	1.034318	1.0267745
79736	C17orf42	chromosome 17 open reading frame 42	0.94593	0.930201	1.0879821
79738	BBS10	Bardet-Biedl syndrome 10	0.93057	1.014011	1.0526684
79739	TTLL7	tubulin tyrosine ligase-like family, member 7	0.99772	1.010489	1.0210611
79740	ZBBX	zinc finger, B-box domain containing	1.00844	1.010587	1.0205594
79741	C10orf68	chromosome 10 open reading frame 68	0.97916	0.95999	0.9580523
79742	CXorf36	chromosome X open reading frame 36	0.99807	0.951512	0.9747151
79744	ZNF419	zinc finger protein 419	0.93078	0.987474	1.0435288
79745	CLIP4	CAP-GLY domain containing linker protein family, member 4	1.0057	1.086941	0.8741391
79746	ECHDC3	enoyl CoA hydratase domain containing 3	1.02006	0.972275	1.0303109
79747	C6orf103	chromosome 6 open reading frame 103	1.00262	0.993388	1.0083741
79748	LMAN1L	lectin, mannose-binding, 1 like	0.9939	1.007242	1.0033877
79750	ZNF385D	zinc finger protein 385D	0.9984	1.014847	0.9851925
79751	SLC25A22	solute carrier family 25 (mitochondrial carrier: glutamate), member 22	0.95841	0.90923	1.0864714
79752	ZFAND1	zinc finger, AN1-type domain 1	0.9419	0.970402	0.9401952
79753	SNIP1	Smad nuclear interacting protein 1	0.98608	0.973732	0.9727904
79754	ASB13	ankyrin repeat and SOCS box containing 13	0.97543	0.958036	1.0066466
79755	ZNF750	zinc finger protein 750	0.98086	1.019798	1.012307
79758	DHRS12	dehydrogenase/reductase (SDR family) member 12	0.9711	0.975121	0.944795

79759	ZNF668	zinc finger protein 668	1.00517	0.996522	0.9745406
79760	GEMIN7	gem (nuclear organelle) associated protein 7	0.97378	0.968032	1.0560752
79762	C1orf115	chromosome 1 open reading frame 115	1.01227	0.948181	1.031897
79763	ISOC2	isochorismatase domain containing 2	0.99058	0.984926	1.0536184
79767	ELMO3	engulfment and cell motility 3	0.99444	1.001622	1.0554526
79768	C15orf29	chromosome 15 open reading frame 29	0.97418	1.031658	0.9635793
79770	TXNDC15	thioredoxin domain containing 15	0.99045	0.951375	1.0610136
79772	MCTP1	multiple C2 domains, transmembrane 1	0.99977	0.997756	1.0363036
79774	GRTP1	growth hormone regulated TBC protein 1	0.9857	1.013759	1.0148804
79776	ZFHX4	zinc finger homeobox 4	1.00577	1.001926	1.0037635
79777	ACBD4	acyl-CoA binding domain containing 4	0.92665	0.962049	0.9995114
79778	MICALL2	MICAL-like 2	0.9988	1.003637	1.0026981
79780	CCDC82	coiled-coil domain containing 82	0.92906	1.021648	1.1085538
79781	IQCA1	IQ motif containing with AAA domain 1	0.9967	0.992653	1.0179188
79782	LRRC31	leucine rich repeat containing 31	0.99553	0.995418	1.0130609
79783	C7orf10	chromosome 7 open reading frame 10	1.00335	0.97792	1.0006633
79784	MYH14	myosin, heavy chain 14, non-muscle	0.99143	1.014454	1.0706161
79785	RERGL	RERG/RAS-like	0.99794	0.996536	0.9883892
79786	KLHL36	kelch-like 36 (Drosophila)	0.93407	0.947602	0.9863433
79788	ZNF665	zinc finger protein 665	0.98	1.012362	1.0235191
79789	CLMN	calmin (calponin-like, transmembrane)	1.00051	1.038042	0.936167
79791	FBXO31	F-box protein 31	0.94458	0.982667	1.0076062
79792	GSDMD	gasdermin D	0.98346	0.970337	1.0365256
79794	C12orf49	chromosome 12 open reading frame 49	0.9396	0.993697	1.1026313
79796	ALG9	asparagine-linked glycosylation 9, alpha-1,2-mannosyltransferase homolog (S. cerevisiae)	0.96876	0.940589	1.0578217
79797	ZNF408	zinc finger protein 408	0.96709	0.991256	1.0524226
79798	ARMC5	armadillo repeat containing 5	0.9949	0.981671	1.0441249
79799	UGT2A3	UDP glucuronosyltransferase 2 family, polypeptide A3	1.00026	0.992593	1.0129312
79800	ALS2CR8	amyotrophic lateral sclerosis 2 (juvenile) chromosome region, candidate 8	0.9623	0.961187	0.9947727
79801	SHCBP1	SHC SH2-domain binding protein 1	1.00748	0.901527	1.0316462
79802	HHIPL2	HHIP-like 2	1.01733	1.021739	1.0039694
79803	HPS6	Hermansky-Pudlak syndrome 6	0.9109	0.939944	1.1273501
79804	NBLA00301	Nbla00301	1.00972	1.013261	1.0053235

79805	VASH2	vasohibin 2	1.00629	1.005361	1.0125096
79807	GSTCD	glutathione S-transferase, C-terminal domain containing	0.99904	0.966216	1.0144399
79809	TTC21B	tetratricopeptide repeat domain 21B	0.9568	0.871901	0.9853147
79810	PTCD2	pentatricopeptide repeat domain 2	0.98869	0.938262	0.9726038
79811	SLTM	SAFB-like, transcription modulator	0.9313	0.962192	0.9233364
79812	MMRN2	multimerin 2	1.01202	1.01023	0.9666145
79813	EHMT1	euchromatic histone-lysine N-methyltransferase 1	0.96312	1.028765	1.0352227
79814	AGMAT	agmatine ureohydrolase (agmatinase)	0.93803	0.947168	1.0114295
79815	NIPAL2	NIPA-like domain containing 2	0.99785	0.950723	1.0572197
79816	TLE6	transducin-like enhancer of split 6 (E(sp1) homolog, Drosophila)	0.99123	0.984017	1.0334448
79817	MOBKL2B	MOB1, Mps One Binder kinase activator-like 2B (yeast)	0.97833	1.026212	0.9802765
79818	ZNF552	zinc finger protein 552	0.95129	0.979657	0.959994
79819	WDR78	WD repeat domain 78	1.00305	0.981201	0.9812066
79820	CATSPERB	cation channel, sperm-associated, beta	0.97815	0.988243	1.0024173
79822	ARHGAP28	Rho GTPase activating protein 28	0.99527	0.98165	1.0010688
79823	CAMKMT	calmodulin-lysine N-methyltransferase	0.97969	0.957291	1.0270132
79825	CCDC48	coiled-coil domain containing 48	1.03129	0.958605	1.0246301
79827	CLMP	CXADR-like membrane protein	0.99874	0.981838	0.9901546
79828	METTL8	methyltransferase like 8	1.01325	0.986428	0.9893823
79829	NAA40	N(alpha)-acetyltransferase 40, NatD catalytic subunit, homolog (S. cerevisiae)	0.97304	0.953314	1.0345899
79830	ZMYM1	zinc finger, MYM-type 1	0.99063	0.908542	0.8885298
79831	JMJD5	jumonji domain containing 5	0.95582	0.988395	0.9650222
79832	QSER1	glutamine and serine rich 1	0.93191	1.001462	1.0758327
79833	GEMIN6	gem (nuclear organelle) associated protein 6	1.02126	0.960646	0.9996407
79834	PEAK1	NKF3 kinase family member	0.9697	1.009593	1.0091863
79836	LONRF3	LON peptidase N-terminal domain and ring finger 3	0.98573	0.950492	0.9532124
79837	PIP4K2C	phosphatidylinositol-5-phosphate 4-kinase, type II, gamma	0.92937	0.944934	1.0036552
79838	TMC5	transmembrane channel-like 5	0.99269	0.992755	0.9829226
79839	CCDC102B	coiled-coil domain containing 102B	0.9909	0.998864	1.0058
79840	NHEJ1	nonhomologous end-joining factor 1	0.98935	0.985942	1.0387482
79841	AGBL2	ATP/GTP binding protein-like 2	0.9591	1.005987	1.0175
79842	ZBTB3	zinc finger and BTB domain containing 3	0.91454	0.923109	0.9960665
79843	FAM124B	family with sequence similarity 124B	1.00528	0.981486	1.0008146

79844	ZDHHC11	zinc finger, DHHC-type containing 11	1.00634	1.002869	0.9933559
79845	RNF122	ring finger protein 122	0.96708	0.992712	0.9546856
79846	C7orf63	chromosome 7 open reading frame 63	0.99785	0.996034	1.0141482
79847	TMEM180	transmembrane protein 180	0.96496	0.960794	1.0364903
79848	CSPP1	centrosome and spindle pole associated protein 1	0.98819	0.993395	1.0935588
79849	PDZD3	PDZ domain containing 3	1.00416	1.004465	0.9992235
79850	FAM57A	family with sequence similarity 57, member A	0.99186	1.036045	1.0086652
79853	TM4SF20	transmembrane 4 L six family member 20	1.02334	0.950239	0.9987945
79857	FLJ13224	hypothetical LOC79857	0.99657	0.941298	0.9387323
79858	NEK11	NIMA (never in mitosis gene a)- related kinase 11	0.9961	0.978873	1.0170347
79861	TUBAL3	tubulin, alpha-like 3	0.98356	0.991745	1.0072076
79862	ZNF669	zinc finger protein 669	0.934	0.983325	0.9780913
79863	RBFA	ribosome binding factor A (putative)	0.93539	0.94678	1.0575241
79864	C11orf63	chromosome 11 open reading frame 63	0.97776	0.990526	0.9825485
79865	TREML2	triggering receptor expressed on myeloid cells-like 2	1.00833	1.011207	1.0665781
79866	C13orf34	chromosome 13 open reading frame 34	0.95451	0.996561	1.0983614
79867	TCTN2	tectonic family member 2	0.9626	0.956352	1.0010012
79868	ALG13	asparagine-linked glycosylation 13 homolog (S. cerevisiae)	0.94028	1.152447	1.0748504
79869	CPSF7	cleavage and polyadenylation specific factor 7, 59kDa	0.97924	0.944821	0.9897641
79870	BAALC	brain and acute leukemia, cytoplasmic	1.01832	1.015232	1.0010122
79871	RPAP2	RNA polymerase II associated protein 2	0.97377	1.033986	0.9763234
79872	CBLL1	Cas-Br-M (murine) ecotropic retroviral transforming sequence-like 1	0.97194	0.986356	0.996222
79873	NUDT18	nudix (nucleoside diphosphate linked moiety X)-type motif 18	0.97633	1.073903	1.0642143
79874	RABEP2	rabaptin, RAB GTPase binding effector protein 2	0.98464	1.010492	1.0800784
79875	THSD4	thrombospondin, type I, domain containing 4	1.00283	0.992064	0.9888565
79876	UBA5	ubiquitin-like modifier activating enzyme 5	1.02239	0.90859	1.1149515
79877	DCAKD	dephospho-CoA kinase domain containing	0.97144	0.975581	1.0196613
79879	CCDC134	coiled-coil domain containing 134	1.00725	0.969891	1.0476156
79882	ZC3H14	zinc finger CCCH-type containing 14	0.94555	1.014293	1.0806092
79883	PODNL1	podocan-like 1	0.99003	1.021455	1.036002
79884	MAP9	microtubule-associated protein 9	0.98401	0.970516	1.0129894
79885	HDAC11	histone deacetylase 11	0.9968	1.007438	1.0185609
79886	C9orf82	chromosome 9 open reading frame 82	0.93938	1.031198	1.0791778

79887	PLBD1	phospholipase B domain containing 1	0.97832	0.885588	1.0676441
79888	LPCAT1	lysophosphatidylcholine acyltransferase 1	1.03141	1.038754	1.0473444
79890	RIN3	Ras and Rab interactor 3	0.97646	1.019143	0.9676031
79891	ZNF671	zinc finger protein 671	0.95752	0.955521	1.055711
79892	MCMBP	minichromosome maintenance complex binding protein	0.93871	1.008207	1.1878044
79893	GGNBP2	gametogenetin binding protein 2	0.97253	0.912627	0.99075
79894	ZNF672	zinc finger protein 672	0.9615	0.993818	0.9599511
79895	ATP8B4	ATPase, class I, type 8B, member 4	0.98703	0.99232	1.0312108
79896	THNSL1	threonine synthase-like 1 (S. cerevisiae)	0.95039	0.967375	1.0162066
79897	RPP21	ribonuclease P/MRP 21kDa subunit	0.95717	1.007908	1.0748415
79898	ZNF613	zinc finger protein 613	0.96912	0.991763	1.0069052
79899	PRR5L	proline rich 5 like	1.00033	0.989816	0.9224646
79902	NUP85	nucleoporin 85kDa	0.9716	1.031175	1.0892412
79903	NAT15	N-acetyltransferase 15 (GCN5-related, putative)	0.97532	0.938056	1.003784
79905	TMC7	transmembrane channel-like 7	1.0078	0.983377	0.9864303
79906	MORN1	MORN repeat containing 1	1.00587	1.004072	1.0466475
79908	BTNL8	butyrophilin-like 8	0.99415	0.988138	0.9857037
79912	PYROXD1	pyridine nucleotide-disulphide oxidoreductase domain 1	0.9972	0.966176	0.9825459
79913	ACTR5	ARP5 actin-related protein 5 homolog (yeast)	0.927	0.986278	1.0369133
79915	ATAD5	ATPase family, AAA domain containing 5	0.97723	0.953763	1.0264164
79917	MAGIX	MAGI family member, X-linked	1.00241	0.999741	1.0472468
79918	SETD6	SET domain containing 6	0.95684	0.954266	1.0647964
79919	C2orf54	chromosome 2 open reading frame 54	1.0156	1.037419	0.9839038
79921	TCEAL4	transcription elongation factor A (SII)-like 4	0.9572	0.946475	1.0176807
79922	MRM1	mitochondrial rRNA methyltransferase 1 homolog (S. cerevisiae)	0.92589	1.0127	1.0389124
79924	ADM2	adrenomedullin 2	0.97539	1.015628	1.0798175
79925	SPEF2	sperm flagellar 2	0.9859	0.987243	0.9723851
79927	GRRP1	glycine/arginine rich protein 1	1.00085	0.989746	1.0540903
79929	MAP6D1	MAP6 domain containing 1	0.97921	1.012346	1.0052883
79930	DOK3	docking protein 3	1.01466	1.004903	1.0388644
79931	TNIP3	TNFAIP3 interacting protein 3	1.00322	0.98458	1.0036969
79932	KIAA0319L	KIAA0319-like	0.98977	0.935165	1.0007757
79933	SYNPO2L	synaptopodin 2-like	1.00054	0.976325	1.0073461

79934	ADCK4	aarF domain containing kinase 4	0.95961	1.009674	1.0137219
79935	CNTD2	cyclin N-terminal domain containing 2	0.99993	0.978048	1.0598872
79939	SLC35E1	solute carrier family 35, member E1	0.97865	0.991701	1.095195
79940	C6orf155	chromosome 6 open reading frame 155	1.01572	1.009455	1.0529017
79943	ZNF696	zinc finger protein 696	0.99153	0.987797	1.0693917
79944	L2HGDH	L-2-hydroxyglutarate dehydrogenase	0.96986	0.986304	1.0207217
79946	C10orf95	chromosome 10 open reading frame 95	0.99135	0.979259	0.9936745
79947	DHDDS	dehydrodolichyl diphosphate synthase	1.00007	0.954007	1.0153704
79948	LPPR3	lipid phosphate phosphatase-related protein type 3	0.99304	1.02049	1.0905852
79949	C10orf81	chromosome 10 open reading frame 81	1.00416	1.019071	0.9783338
79953	TMEM90B	transmembrane protein 90B	0.99648	0.990949	1.0166453
79954	NOL10	nucleolar protein 10	0.98693	0.865263	1.014908
79955	PDZD7	PDZ domain containing 7	0.98803	0.986365	0.9743634
79956	ERMP1	endoplasmic reticulum metallopeptidase 1	0.92211	0.893852	0.9633218
79957	PAQR6	progestin and adipoQ receptor family member VI	1.01278	0.984581	1.051227
79958	DENND1C	DENN/MADD domain containing 1C	0.95121	0.995685	0.9762409
79959	CEP76	centrosomal protein 76kDa	0.93349	0.995385	1.0678139
79960	PHF17	PHD finger protein 17	0.97672	0.91126	1.0153943
79961	DENND2D	DENN/MADD domain containing 2D	0.97673	0.958741	0.9975258
79962	DNAJC22	DnaJ (Hsp40) homolog, subfamily C, member 22	0.99281	0.999472	1.0251274
79966	SCD5	stearoyl-CoA desaturase 5	1.00969	0.998777	0.9953826
79968	WDR76	WD repeat domain 76	0.9397	1.000788	1.0095653
79969	ATAT1	alpha tubulin acetyltransferase 1	0.99487	0.972328	1.0061174
79970	ZNF767	zinc finger family member 767	0.97308	0.96933	0.9471682
79971	WLS	wntless homolog (Drosophila)	1.01518	1.003124	1.0259221
79973	ZNF442	zinc finger protein 442	0.98145	0.968915	0.9999106
79974	C7orf58	chromosome 7 open reading frame 58	0.99716	0.962774	0.9684963
79977	GRHL2	grainyhead-like 2 (Drosophila)	1.00223	0.995843	1.0264787
79979	TRMT2B	TRM2 tRNA methyltransferase 2 homolog B (S. cerevisiae)	0.92572	0.951911	0.972925
79980	DSN1	DSN1, MIND kinetochore complex component, homolog (S. cerevisiae)	0.9264	0.953818	1.0142669
79981	FRMD1	FERM domain containing 1	0.99076	1.019044	1.0379439
79982	DNAJB14	DnaJ (Hsp40) homolog, subfamily B, member 14	1.01792	0.968509	0.992149
79983	POF1B	premature ovarian failure, 1B	0.9993	1.006211	0.9950958

79986	ZNF702P	zinc finger protein 702, pseudogene	1.02251	0.98271	1.0083608
79987	SVEP1	sushi, von Willebrand factor type A, EGF and pentraxin domain containing 1	0.99953	1.007244	1.001835
79989	TTC26	tetratricopeptide repeat domain 26	0.99753	0.983286	1.0119305
79990	PLEKHH3	pleckstrin homology domain containing, family H (with MyTH4 domain) member 3	0.99047	0.943592	1.0576635
79991	OBFC1	oligonucleotide/oligosaccharide-binding fold containing 1	0.93741	0.959597	0.9462908
79993	ELOVL7	ELOVL fatty acid elongase 7	0.9861	0.969681	0.9972922
79998	ANKRD53	ankyrin repeat domain 53	0.98322	1.015076	1.0373905
80000	GREB1L	growth regulation by estrogen in breast cancer-like	0.99396	1.001045	0.9899406
80003	PCNXL2	pecanex-like 2 (Drosophila)	0.98498	0.968123	0.9420278
80004	ESRP2	epithelial splicing regulatory protein 2	0.99047	1.01504	1.0363497
80005	DOCK5	dedicator of cytokinesis 5	1.03313	1.049582	0.957204
80006	C5orf44	chromosome 5 open reading frame 44	0.99333	0.887025	1.025965
80007	C10orf88	chromosome 10 open reading frame 88	0.95656	0.993938	1.0054835
80008	TMEM156	transmembrane protein 156	0.99116	1.003243	1.0934147
80010	RMI1	RMI1, RecQ mediated genome instability 1, homolog (S. cerevisiae)	0.93113	0.95664	1.1000085
80011	FAM192A	family with sequence similarity 192, member A	1.02085	1.022496	1.0685652
80012	PHC3	polyhomeotic homolog 3 (Drosophila)	0.9967	0.996404	0.9631486
80013	FAM188A	family with sequence similarity 188, member A	0.9093	1.032123	1.158326
80014	WWC2	WW and C2 domain containing 2	0.99439	0.967059	0.9611676
80017	C14orf159	chromosome 14 open reading frame 159	0.97012	0.98291	1.0476006
80018	NAA25	N(alpha)-acetyltransferase 25, NatB auxiliary subunit	0.94355	0.994164	1.0056007
80019	UBTD1	ubiquitin domain containing 1	0.9851	0.978317	0.9896748
80021	TMEM62	transmembrane protein 62	0.95901	1.030643	0.9871537
80022	MYO15B	myosin XVB pseudogene	0.96546	0.958195	0.9735401
80023	NRSN2	neurensin 2	0.97873	0.980699	1.013253
80024	SLC24A6	solute carrier family 24 (sodium/potassium/calcium exchanger), member 6	0.96645	0.940528	0.9787001
80025	PANK2	pantothenate kinase 2	1.0049	0.963644	1.1123937
80028	FBXL18	F-box and leucine-rich repeat protein 18	1.00431	1.017399	1.0163129
80031	SEMA6D	sema domain, transmembrane domain (TM), and cytoplasmic domain, (semaphorin) 6D	0.99697	0.988024	1.0015144
80032	ZNF556	zinc finger protein 556	0.97125	1.037957	1.0200326
80034	CSRNP3	cysteine-serine-rich nuclear protein 3	1.01072	0.977292	1.0180012
80036	TRPM3	transient receptor potential cation channel, subfamily M, member 3	0.99896	0.993147	1.003188
80045	GPR157	G protein-coupled receptor 157	0.99524	0.964403	1.0142023

80047	[No Symbo	[No Name]	0.95646	0.987737	0.9012761
80054	LOC80054	hypothetical LOC80054	0.99879	1.026173	0.951171
80055	PGAP1	post-GPI attachment to proteins 1	0.98324	0.976158	1.0011776
80059	LRRTM4	leucine rich repeat transmembrane neuronal 4	1.00009	0.957764	1.0427777
80063	ATF7IP2	activating transcription factor 7 interacting protein 2	0.89374	0.987601	1.0199435
80067	DCAF17	DDB1 and CUL4 associated factor 17	0.9823	0.951977	0.9920479
80070	ADAMTS20	ADAM metallopeptidase with thrombospondin type 1 motif, 20	0.98311	1.017457	1.0052331
80071	CCDC15	coiled-coil domain containing 15	0.98233	0.957297	1.0185305
80072	C15orf34	chromosome 15 open reading frame 34	0.98295	1.030446	0.943742
80086	TUBA4B	tubulin, alpha 4b (pseudogene)	0.97948	0.999359	0.9869461
80092	[No Symbo	[No Name]	1.02227	1.061522	1.0069269
80093	[No Symbo	[No Name]	1.00029	0.990802	1.0428778
80095	ZNF606	zinc finger protein 606	0.95277	0.95098	0.992435
80097	MZT2B	mitotic spindle organizing protein 2B	1.04259	1.02702	1.0078613
80099	C7orf69	chromosome 7 open reading frame 69	0.97372	1.03311	1.0355178
80100	[No Symbo	[No Name]	0.96388	0.970583	0.9063798
80108	ZFP2	zinc finger protein 2 homolog (mouse)	0.98414	0.996905	0.9905438
80110	ZNF614	zinc finger protein 614	0.94305	0.978105	1.0035073
80111	C3orf36	chromosome 3 open reading frame 36	0.97724	0.963697	1.0130619
80114	BICC1	bicaudal C homolog 1 (Drosophila)	1.00341	0.982341	0.9923798
80115	BAIAP2L2	BAI1-associated protein 2-like 2	0.98121	0.997999	1.0381382
80117	ARL14	ADP-ribosylation factor-like 14	1.00168	1.013865	0.9837099
80119	PIF1	PIF1 5'-to-3' DNA helicase homolog (S. cerevisiae)	1.00225	1.046657	1.0206987
80122	YSK4	YSK4 Sps1/Ste20-related kinase homolog (S. cerevisiae)	0.99855	1.003274	1.0073642
80124	VCPIP1	valosin containing protein (p97)/p47 complex interacting protein 1	0.94239	1.026338	1.086905
80125	CCDC33	coiled-coil domain containing 33	1.00297	1.003647	1.0338983
80127	C14orf45	chromosome 14 open reading frame 45	0.96895	0.978476	1.0161374
80128	TRIM46	tripartite motif containing 46	0.99941	1.020574	1.0287904
80129	C6orf97	chromosome 6 open reading frame 97	1.01409	0.991694	0.9981812
80131	LRRC8E	leucine rich repeat containing 8 family, member E	0.98489	1.006606	1.0456619
80133	C1orf129	chromosome 1 open reading frame 129	0.99982	1.013882	1.0015608
80135	RPF1	ribosome production factor 1 homolog (S. cerevisiae)	0.97835	0.880434	1.0041124
80139	ZNF703	zinc finger protein 703	0.99292	0.94526	0.9809405

80142	PTGES2	prostaglandin E synthase 2	0.98116	0.934274	1.012732
80143	SIKE1	suppressor of IKBKE 1	0.97505	0.957838	1.0110973
80144	FRAS1	Fraser syndrome 1	1.00978	0.996128	1.004037
80145	THOC7	THO complex 7 homolog (Drosophila)	0.93968	0.935767	1.0517434
80146	UXS1	UDP-glucuronate decarboxylase 1	0.98997	0.984334	0.9850045
80148	PQLC1	PQ loop repeat containing 1	0.96537	1.003774	0.9763834
80149	ZC3H12A	zinc finger CCCH-type containing 12A	0.99767	1.01264	0.9116458
80150	ASRGL1	asparaginase like 1	1.00064	0.997467	1.0431464
80152	CENPT	centromere protein T	0.97832	0.990716	0.9763071
80153	EDC3	enhancer of mRNA decapping 3 homolog (S. cerevisiae)	0.982	1.021873	1.0054406
80155	NAA15	N(alpha)-acetyltransferase 15, NatA auxiliary subunit	0.9765	0.972316	1.0674242
80157	CWH43	cell wall biogenesis 43 C-terminal homolog (S. cerevisiae)	1.00028	1.010599	1.0086875
80162	ATHL1	ATH1, acid trehalase-like 1 (yeast)	0.95795	1.022684	0.9267609
80164	FLJ22184	hypothetical protein FLJ22184	0.98626	1.016549	1.0822782
80167	C4orf29	chromosome 4 open reading frame 29	0.96567	1.008013	0.9790291
80168	MOGAT2	monoacylglycerol O-acyltransferase 2	0.99357	1.022791	0.9976116
80169	CTC1	CTS telomere maintenance complex component 1	0.97238	0.974971	1.0148906
80173	IFT74	intraflagellar transport 74 homolog (Chlamydomonas)	0.9607	0.953745	1.0107044
80174	DBF4B	DBF4 homolog B (S. cerevisiae)	0.98936	0.979197	1.0013128
80176	SPSB1	splA/ryanodine receptor domain and SOCS box containing 1	1.00652	0.995555	1.015183
80177	MYCT1	myc target 1	0.90917	0.925015	0.9758912
80178	C16orf59	chromosome 16 open reading frame 59	0.98083	1.041168	1.0287708
80179	MYO19	myosin XIX	0.97913	0.982211	1.0184562
80183	C13orf18	chromosome 13 open reading frame 18	1.00961	1.165498	0.906272
80184	CEP290	centrosomal protein 290kDa	0.9498	0.993869	1.0175618
80185	C8orf41	chromosome 8 open reading frame 41	0.90413	1.02479	1.1311934
80194	TMEM134	transmembrane protein 134	0.9639	1.020801	1.0636833
80195	C10orf57	chromosome 10 open reading frame 57	0.95316	0.954845	1.0271014
80196	RNF34	ring finger protein 34	0.95316	0.978616	0.9993244
80198	MUS81	MUS81 endonuclease homolog (S. cerevisiae)	0.95662	0.960135	1.0185874
80199	FUZ	fuzzy homolog (Drosophila)	0.95324	0.962327	1.0561915
80201	HKDC1	hexokinase domain containing 1	0.96994	0.979051	0.9712386
80204	FBXO11	F-box protein 11	1.01156	0.950728	0.9254855

80205	CHD9	chromodomain helicase DNA binding protein 9	0.979	0.983609	1.0591117
80206	FHOD3	formin homology 2 domain containing 3	1.0045	1.008498	1.0036213
80207	OPA3	optic atrophy 3 (autosomal recessive, with chorea and spastic paraplegia)	0.9618	0.98235	1.0147326
80208	SPG11	spastic paraplegia 11 (autosomal recessive)	0.92961	0.998559	1.057155
80209	C13orf23	chromosome 13 open reading frame 23	0.95772	0.958668	1.1462887
80210	ARMC9	armadillo repeat containing 9	1.00573	1.000756	1.0194541
80212	CCDC92	coiled-coil domain containing 92	0.97793	0.98506	0.9022646
80213	TM2D3	TM2 domain containing 3	0.93611	0.979517	0.9060591
80216	ALPK1	alpha-kinase 1	1.00511	0.963693	1.0763851
80217	WDR96	WD repeat domain 96	0.99382	0.999538	1.0047851
80218	NAA50	N(alpha)-acetyltransferase 50, NatE catalytic subunit	1.02856	1.073847	0.9183015
80219	COQ10B	coenzyme Q10 homolog B (S. cerevisiae)	0.98358	0.957639	1.0038132
80221	ACSF2	acyl-CoA synthetase family member 2	0.9831	0.965135	1.0130953
80222	TARS2	threonyl-tRNA synthetase 2, mitochondrial (putative)	0.9751	0.957063	0.9921064
80223	RAB11FIP1	RAB11 family interacting protein 1 (class I)	0.99023	0.957952	0.9201681
80224	NUBPL	nucleotide binding protein-like	0.98664	0.948561	1.0048708
80227	PAAF1	proteasomal ATPase-associated factor 1	0.97047	0.979335	1.0448476
80228	ORAI2	ORAI calcium release-activated calcium modulator 2	1.0152	0.957924	0.9688908
80230	RUFY1	RUN and FYVE domain containing 1	0.97778	0.953349	0.981826
80231	CXorf21	chromosome X open reading frame 21	0.91182	1.039199	0.9787927
80232	WDR26	WD repeat domain 26	1.05211	0.961595	1.0071151
80233	C17orf70	chromosome 17 open reading frame 70	0.98536	0.971704	1.0461489
80235	PIGZ	phosphatidylinositol glycan anchor biosynthesis, class Z	1.00883	0.986034	1.0036623
80237	ELL3	elongation factor RNA polymerase II-like 3	0.98188	1.027686	1.0213222
80243	PREX2	phosphatidylinositol-3,4,5-trisphosphate-dependent Rac exchange factor 2	0.99757	0.99754	1.0201793
80254	CEP63	centrosomal protein 63kDa	0.99749	0.975146	1.0139243
80255	SLC35F5	solute carrier family 35, member F5	1.00238	1.057774	0.9549516
80256	KIAA1539	KIAA1539	0.97253	0.993366	0.9894898
80262	C16orf70	chromosome 16 open reading frame 70	0.96279	0.915721	1.0300347
80263	TRIM45	tripartite motif containing 45	1.01383	1.012692	1.0218209
80264	ZNF430	zinc finger protein 430	0.97836	0.906356	0.9278151
80267	EDEM3	ER degradation enhancer, mannosidase alpha-like 3	0.98812	0.921078	0.9890399
80270	HSD3B7	hydroxy-delta-5-steroid dehydrogenase, 3 beta- and steroid delta-isomerase 7	0.9968	1.032695	1.0087708

80271	ITPKC	inositol-trisphosphate 3-kinase C	0.97764	1.036117	0.9914882
80273	GRPEL1	GrpE-like 1, mitochondrial (E. coli)	0.96354	0.953226	0.9996803
80274	SCUBE1	signal peptide, CUB domain, EGF-like 1	0.99568	1.019642	0.9957526
80279	CDK5RAP3	CDK5 regulatory subunit associated protein 3	0.95149	1.101302	1.0460188
80298	MTERFD3	MTERF domain containing 3	0.95396	1.03416	1.0452211
80301	PLEKHO2	pleckstrin homology domain containing, family O member 2	0.98036	1.056448	0.9401538
80303	EFHD1	EF-hand domain family, member D1	0.99251	0.994855	1.0011346
80304	C2orf44	chromosome 2 open reading frame 44	0.96174	0.992728	1.0472906
80305	TRABD	TraB domain containing	0.97359	1.129869	0.9776299
80306	MED28	mediator complex subunit 28	0.99542	0.930394	1.0828776
80307	FER1L4	fer-1-like 4 (C. elegans) pseudogene	0.9931	1.009093	1.0121563
80308	FLAD1	FAD1 flavin adenine dinucleotide synthetase homolog (S. cerevisiae)	1.0003	0.978944	1.0796723
80309	SPHKAP	SPHK1 interactor, AKAP domain containing	1.00676	0.993503	1.0141003
80310	PDGFD	platelet derived growth factor D	0.99685	1.050398	0.9797713
80311	KLHL15	kelch-like 15 (Drosophila)	0.98688	0.950489	1.0325219
80312	TET1	tet oncogene 1	0.97495	0.974203	1.0064677
80313	LRRC27	leucine rich repeat containing 27	0.99309	0.993653	1.0229051
80314	EPC1	enhancer of polycomb homolog 1 (Drosophila)	0.92192	0.924888	1.0094328
80315	CPEB4	cytoplasmic polyadenylation element binding protein 4	1.01782	0.935304	0.992955
80316	PPP1R2P9	protein phosphatase 1, regulatory (inhibitor) subunit 2 pseudogene 9	0.98963	0.984982	0.9983153
80317	ZKSCAN3	zinc finger with KRAB and SCAN domains 3	0.98729	0.982414	1.0084374
80318	GKAP1	G kinase anchoring protein 1	0.95901	0.966452	0.9934253
80319	CXXC4	CXXC finger protein 4	1.01892	1.010414	1.0133151
80320	SP6	Sp6 transcription factor	0.994	0.998358	1.0214425
80321	CEP70	centrosomal protein 70kDa	0.96186	0.942475	0.9410666
80323	CCDC68	coiled-coil domain containing 68	0.99721	1.000741	1.0180104
80324	PUS1	pseudouridylate synthase 1	0.97258	0.991476	1.0008773
80325	ABTB1	ankyrin repeat and BTB (POZ) domain containing 1	0.99868	0.959747	0.9922519
80326	WNT10A	wingless-type MMTV integration site family, member 10A	0.99703	0.985014	1.0827061
80328	ULBP2	UL16 binding protein 2	0.99103	1.069446	1.0385545
80329	ULBP1	UL16 binding protein 1	0.98995	1.014081	1.0289567
80331	DNAJC5	DnaJ (Hsp40) homolog, subfamily C, member 5	0.9811	1.132983	1.009734
80332	ADAM33	ADAM metallopeptidase domain 33	0.99617	1.013407	1.020931

80333	KCNIP4	Kv channel interacting protein 4	1.01314	0.98414	1.0097624
80335	WDR82	WD repeat domain 82	0.9837	1.085652	0.9245549
80336	PABPC1L	poly(A) binding protein, cytoplasmic 1-like	0.97134	0.991545	0.9741759
80339	PNPLA3	patatin-like phospholipase domain containing 3	0.98431	1.025417	1.0348395
80341	BPIL1	bactericidal/permeability-increasing protein-like 1	1.00241	0.974022	0.9791704
80342	TRAF3IP3	TRAF3 interacting protein 3	0.97918	0.9788	0.873851
80343	SEL1L2	sel-1 suppressor of lin-12-like 2 (C. elegans)	0.99272	1.008548	0.9895259
80344	DCAF11	DDB1 and CUL4 associated factor 11	0.90316	1.053465	0.9887663
80345	ZSCAN16	zinc finger and SCAN domain containing 16	0.95455	0.996695	0.90321
80346	REEP4	receptor accessory protein 4	0.93826	0.934642	1.0564111
80347	COASY	CoA synthase	0.94395	1.006019	1.0430039
80349	WDR61	WD repeat domain 61	0.98321	1.039336	1.041336
80350	LPAL2	lipoprotein, Lp(a)-like 2, pseudogene	1.0382	0.989272	0.985444
80351	TNKS2	tankyrase, TRF1-interacting ankyrin-related ADP-ribose polymerase 2	0.9482	0.929421	1.0068986
80352	RNF39	ring finger protein 39	1.00091	1.046446	1.0455802
80380	PDCD1LG2	programmed cell death 1 ligand 2	0.99554	1.004593	1.0151932
80381	CD276	CD276 molecule	1.00444	0.98068	0.9992141
80700	UBXN6	UBX domain protein 6	0.96107	0.961098	0.9812614
80701	AKR1D1P1	aldo-keto reductase family 1, member D1 pseudogene 1	0.95706	0.963628	0.8757577
80704	SLC19A3	solute carrier family 19, member 3	1.00602	0.990949	1.0278641
80705	TSGA10	testis specific, 10	0.98728	0.989637	0.9827265
80709	AKNA	AT-hook transcription factor	0.96548	1.056516	0.9322023
80712	ESX1	ESX homeobox 1	0.9922	1.055763	0.968497
80713	MTIF2P1	mitochondrial translational initiation factor 2 pseudogene 1	1.03308	0.964893	1.0381867
80714	PBX4	pre-B-cell leukemia homeobox 4	0.97563	1.07652	0.919462
80723	TMEM22	transmembrane protein 22	1.00698	1.015013	1.0089566
80724	ACAD10	acyl-CoA dehydrogenase family, member 10	0.95554	0.951989	1.0020398
80725	SRCIN1	SRC kinase signaling inhibitor 1	1.0144	0.999821	1.0586303
80726	KIAA1683	KIAA1683	1.00128	0.973678	0.9519139
80727	TTYH3	tweety homolog 3 (Drosophila)	1.01966	0.983483	0.9222882
80728	ARHGAP39	Rho GTPase activating protein 39	0.99355	0.976878	1.0186972
80731	THSD7B	thrombospondin, type I, domain containing 7B	1.00709	0.992317	1.0208125
80736	SLC44A4	solute carrier family 44, member 4	1.01047	1.006433	1.0060583

80737	C6orf27	chromosome 6 open reading frame 27	1.01086	1.027712	1.0231026
80739	C6orf25	chromosome 6 open reading frame 25	1.0383	0.973915	1.0083932
80740	LY6G6C	lymphocyte antigen 6 complex, locus G6C	1.00427	1.032571	0.9689269
80741	LY6G5C	lymphocyte antigen 6 complex, locus G5C	0.9475	0.974315	0.9370646
80742	PRR3	proline rich 3	0.98657	0.928528	1.0510113
80745	THUMPD2	THUMP domain containing 2	0.98413	0.909468	1.044945
80746	TSEN2	tRNA splicing endonuclease 2 homolog (S. cerevisiae)	0.99975	0.988584	0.9903112
80755	AARSD1	alanyl-tRNA synthetase domain containing 1	0.96486	1.024408	0.9970367
80757	TMEM121	transmembrane protein 121	1.0062	1.060528	1.0931635
80758	PRR7	proline rich 7 (synaptic)	1.00486	1.069721	0.9511869
80759	KHDC1	KH homology domain containing 1	1.00479	0.973755	0.9967353
80760	ITIH5	inter-alpha (globulin) inhibitor H5	1.00307	0.986613	1.0078539
80761	UPK3B	uroplakin 3B	0.9853	1.011067	1.0291479
80762	NDFIP1	Nedd4 family interacting protein 1	0.96917	0.937421	0.9069274
80763	C12orf39	chromosome 12 open reading frame 39	0.93825	0.944575	1.0484846
80764	THAP7	THAP domain containing 7	0.95769	0.942727	0.9219713
80765	STARD5	StAR-related lipid transfer (START) domain containing 5	0.99541	1.004246	0.9184423
80772	GLTPD1	glycolipid transfer protein domain containing 1	0.98166	0.97957	1.0391932
80774	LIMD2	LIM domain containing 2	0.95607	0.975043	1.0140759
80775	TMEM177	transmembrane protein 177	0.96164	0.947943	1.0686988
80776	B9D2	B9 protein domain 2	0.97	0.941485	0.9826868
80777	CYB5B	cytochrome b5 type B (outer mitochondrial membrane)	0.95536	1.013372	0.9950365
80778	ZNF34	zinc finger protein 34	0.95547	0.97897	0.999244
80781	COL18A1	collagen, type XVIII, alpha 1	0.9821	0.980301	0.9746565
80789	INTS5	integrator complex subunit 5	0.95221	1.003324	1.1298964
80790	CMIP	c-Maf-inducing protein	0.99723	1.11265	0.9338633
80816	ASXL3	additional sex combs like 3 (Drosophila)	1.00064	0.987222	1.0093179
80817	CEP44	centrosomal protein 44kDa	0.97746	0.895126	1.0096417
80818	ZNF436	zinc finger protein 436	0.97404	0.957051	1.0536995
80820	EEPD1	endonuclease/exonuclease/phosphatase family domain containing 1	1.00176	1.066041	0.9734229
80821	DDHD1	DDHD domain containing 1	0.9239	0.955268	1.1602905
80823	BHLHB9	basic helix-loop-helix domain containing, class B, 9	0.99386	1.012162	1.0290003
80824	DUSP16	dual specificity phosphatase 16	0.90614	1.013462	1.0175501

80830	APOL6	apolipoprotein L, 6	0.91969	1.024878	0.9490312
80831	APOL5	apolipoprotein L, 5	1.00494	1.03192	0.9985055
80832	APOL4	apolipoprotein L, 4	1.01344	1.01418	0.9913677
80833	APOL3	apolipoprotein L, 3	0.93903	0.98633	0.9845605
80834	TAS1R2	taste receptor, type 1, member 2	1.02165	0.99708	1.0394538
80835	TAS1R1	taste receptor, type 1, member 1	1.00623	1.032276	1.0167466
80851	SH3BP5L	SH3-binding domain protein 5-like	0.98489	0.981409	0.9938219
80852	GRIP2	glutamate receptor interacting protein 2	0.98842	1.021635	1.0107665
80853	JHDM1D	jumonji C domain containing histone demethylase 1 homolog D (S. cerevisiae)	0.99128	0.971186	0.9945567
80854	SETD7	SET domain containing (lysine methyltransferase) 7	1.00688	0.924305	1.0233028
80856	KIAA1715	KIAA1715	0.98118	0.953882	1.0230888
80863	PRRT1	proline-rich transmembrane protein 1	0.98319	0.996452	1.0367523
80864	EGFL8	EGF-like-domain, multiple 8	1.01831	1.01121	0.9962027
80868	HCG4B	HLA complex group 4B (non-protein coding)	0.96538	1.033404	0.9692862
80895	ILKAP	integrin-linked kinase-associated serine/threonine phosphatase	0.98889	0.969011	0.9319858
80896	NPL	N-acetylneuraminate pyruvate lyase (dihydrodipicolinate synthase)	1.00709	0.903056	0.9472551
80975	TMPRSS5	transmembrane protease, serine 5	1.01463	1.002825	0.972968
81025	GJA9	gap junction protein, alpha 9, 59kDa	0.99021	0.987938	0.9998634
81027	TUBB1	tubulin, beta 1	0.92664	0.946167	0.9972285
81029	WNT5B	wingless-type MMTV integration site family, member 5B	0.99241	0.976045	1.00423
81030	ZBP1	Z-DNA binding protein 1	0.93922	1.079392	0.9900021
81031	SLC2A10	solute carrier family 2 (facilitated glucose transporter), member 10	0.99695	1.021635	0.9866172
81033	KCNH6	potassium voltage-gated channel, subfamily H (eag-related), member 6	0.98861	1.028419	0.9752593
81034	SLC25A32	solute carrier family 25, member 32	0.93568	1.04991	1.0599193
81035	COLEC12	collectin sub-family member 12	1.00265	0.998753	1.0083454
81037	CLPTM1L	CLPTM1-like	0.98934	0.995256	1.0843662
81047	OR5BJ1P	olfactory receptor, family 5, subfamily BJ, member 1 pseudogene	1.01817	0.979079	0.9619631
81050	OR5AC2	olfactory receptor, family 5, subfamily AC, member 2	0.98135	1.006208	1.0269836
81075	OR5BH1P	olfactory receptor, family 5, subfamily BH, member 1 pseudogene	1.00655	0.982179	0.9874628
81076	OR5AW1P	olfactory receptor, family 5, subfamily AW, member 1 pseudogene	1.03647	0.98538	1.0859121
81110	OR11J2P	olfactory receptor, family 11, subfamily J, member 2 pseudogene	1.01702	1.026754	1.0399872
81113	OR11H3P	olfactory receptor, family 11, subfamily H, member 3 pseudogene	1.05898	1.050803	0.9199917
81115	OR7E106P	olfactory receptor, family 7, subfamily E, member 106 pseudogene	1.0166	1.015999	1.0205591

81126	OR4K16P	olfactory receptor, family 4, subfamily K, member 16 pseudogene	0.99711	0.995742	0.9988131
81136	OR7E111P	olfactory receptor, family 7, subfamily E, member 111 pseudogene	0.98819	1.062625	0.9576007
81138	OR7E101P	olfactory receptor, family 7, subfamily E, member 101 pseudogene	1.03804	0.949038	0.9301559
81140	OR9R1P	olfactory receptor, family 9, subfamily R, member 1 pseudogene	1.00395	1.065527	1.0127444
81153	OR9M1P	olfactory receptor, family 9, subfamily M, member 1, pseudogene	1.01639	1.022061	0.9894948
81155	OR9I3P	olfactory receptor, family 9, subfamily I, member 3 pseudogene	0.99992	1.009774	1.0476067
81159	OR9G3P	olfactory receptor, family 9, subfamily G, member 3 pseudogene	0.9969	0.989674	0.9711039
81160	OR9G2P	olfactory receptor, family 9, subfamily G, member 2 pseudogene	1.01762	0.984826	1.0117949
81164	OR8L1P	olfactory receptor, family 8, subfamily L, member 1 pseudogene	1.00016	1.062135	1.0399458
81168	OR8J3	olfactory receptor, family 8, subfamily J, member 3	0.99744	1.042961	1.0399397
81169	OR8J2	olfactory receptor, family 8, subfamily J, member 2 (gene/pseudogene)	1.03151	1.023433	0.983271
81182	OR5W1P	olfactory receptor, family 5, subfamily W, member 1 pseudogene	0.98867	0.990118	1.0075892
81185	OR5P4P	olfactory receptor, family 5, subfamily P, member 4 pseudogene	1.02762	0.954522	1.0424767
81187	OR5M13P	olfactory receptor, family 5, subfamily M, member 13 pseudogene	0.97471	0.995168	1.0124138
81188	OR5M12P	olfactory receptor, family 5, subfamily M, member 12 pseudogene	1.03082	0.972802	0.9921902
81191	OR5G5P	olfactory receptor, family 5, subfamily G, member 5 pseudogene	0.98024	1.011785	1.0263169
81192	OR5G4P	olfactory receptor, family 5, subfamily G, member 4 pseudogene	0.96544	1.071078	1.0322049
81193	OR5G3	olfactory receptor, family 5, subfamily G, member 3 (gene/pseudogene)	0.9821	0.992057	1.0189111
81194	OR5F2P	olfactory receptor, family 5, subfamily F, member 2 pseudogene	0.97718	0.987775	0.9612312
81196	OR5D17P	olfactory receptor, family 5, subfamily D, member 17 pseudogene	1.02007	0.982665	1.0647623
81198	OR5D15P	olfactory receptor, family 5, subfamily D, member 15 pseudogene	1.00339	0.978281	1.0311391
81202	OR5BQ1P	olfactory receptor, family 5, subfamily BQ, member 1 pseudogene	0.97891	0.979843	1.015295
81204	OR5BN1P	olfactory receptor, family 5, subfamily BN, member 1 pseudogene	0.98336	1.082426	1.0287146
81207	OR5BE1P	olfactory receptor, family 5, subfamily BE, member 1 pseudogene	1.01578	0.97598	0.9489667
81208	OR5BC1P	olfactory receptor, family 5, subfamily BC, member 1 pseudogene	0.97202	1.013283	1.0316872
81209	OR5BB1P	olfactory receptor, family 5, subfamily BB, member 1 pseudogene	1.03254	1.01406	1.0319616
81210	OR5BA1P	olfactory receptor, family 5, subfamily BA, member 1 pseudogene	1.03581	0.993362	1.0029364
81211	OR5B19P	olfactory receptor, family 5, subfamily B, member 19 pseudogene	0.96993	1.057911	1.004124
81215	OR5B15P	olfactory receptor, family 5, subfamily B, member 15 pseudogene	0.99307	1.034721	1.0000866
81221	OR5AQ1P	olfactory receptor, family 5, subfamily AQ, member 1 pseudogene	0.97503	1.045763	0.9786881
81223	OR5AP1P	olfactory receptor, family 5, subfamily AP, member 1 pseudogene	0.9963	1.016758	1.0243975
81224	OR5AN2P	olfactory receptor, family 5, subfamily AN, member 2 pseudogene	1.00478	1.02586	1.0225664
81226	OR5AM1P	olfactory receptor, family 5, subfamily AM, member 1 pseudogene	0.95603	1.00875	0.9800827

81228	OR5AK3P	olfactory receptor, family 5, subfamily AK, member 3 pseudogene	1.01751	0.976194	1.0290425
81230	OR5AK1P	olfactory receptor, family 5, subfamily AK, member 1 pseudogene	1.0194	1.064412	1.0175284
81236	OR56A7P	olfactory receptor, family 56, subfamily A, member 7 pseudogene	0.9972	1.063673	0.9608841
81242	OR52V1P	olfactory receptor, family 52, subfamily V, member 1 pseudogene	1.00755	1.005078	1.0018643
81243	OR52U1P	olfactory receptor, family 52, subfamily U, member 1 pseudogene	0.98183	1.006801	0.9795442
81244	OR52T1P	olfactory receptor, family 52, subfamily T, member 1 pseudogene	0.99617	1.015425	0.9954542
81248	OR52P1P	olfactory receptor, family 52, subfamily P, member 1 pseudogene	1.00582	0.927147	0.9343139
81251	OR52N3P	olfactory receptor, family 52, subfamily N, member 3 pseudogene	1.03447	1.004229	0.983073
81261	OR52H2P	olfactory receptor, family 52, subfamily H, member 2 pseudogene	1.00024	1.025918	1.0197472
81264	OR52E7P	olfactory receptor, family 52, subfamily E, member 7 pseudogene	1.00868	1.02576	1.009399
81270	OR52B5P	olfactory receptor, family 52, subfamily B, member 5 pseudogene	0.97903	0.987843	0.9932851
81274	OR52B1P	olfactory receptor, family 52, subfamily B, member 1 pseudogene	1.00606	0.943074	1.0216101
81278	OR51K1P	olfactory receptor, family 51, subfamily K, member 1 pseudogene	0.99257	1.009664	0.9518192
81282	OR51G2	olfactory receptor, family 51, subfamily G, member 2	1.00334	1.052716	1.0550062
81285	OR51E2	olfactory receptor, family 51, subfamily E, member 2	1.03143	0.967405	1.0152126
81295	OR51A10P	olfactory receptor, family 51, subfamily A, member 10 pseudogene	1.00108	1.037678	1.0147053
81300	OR4P4	olfactory receptor, family 4, subfamily P, member 4	0.97882	1.089209	1.0082264
81304	OR4D7P	olfactory receptor, family 4, subfamily D, member 7 pseudogene	0.98882	1.039132	1.042085
81309	OR4C15	olfactory receptor, family 4, subfamily C, member 15	0.94834	1.01024	0.9926517
81310	OR4C14P	olfactory receptor, family 4, subfamily C, member 14 pseudogene	1.01865	1.032152	0.973005
81315	OR4A8P	olfactory receptor, family 4, subfamily A, member 8 pseudogene	0.98125	1.025021	1.0102487
81322	OR4A21P	olfactory receptor, family 4, subfamily A, member 21 pseudogene	0.98972	1.007006	1.0065768
81324	OR4A19P	olfactory receptor, family 4, subfamily A, member 19 pseudogene	1.00699	1.020268	1.0287019
81325	OR4A18P	olfactory receptor, family 4, subfamily A, member 18 pseudogene	1.01079	1.093887	1.0445678
81327	OR4A16	olfactory receptor, family 4, subfamily A, member 16	1.01877	1.012213	0.9730957
81328	OR4A15	olfactory receptor, family 4, subfamily A, member 15	1.02935	1.016503	0.9622194
81330	OR4A13P	olfactory receptor, family 4, subfamily A, member 13 pseudogene	0.99508	1.045376	1.052816
81331	OR4A12P	olfactory receptor, family 4, subfamily A, member 12 pseudogene	1.00345	1.033392	0.9393927
81336	OR2AH1P	olfactory receptor, family 2, subfamily AH, member 1 pseudogene	0.99624	1.014294	1.0063746
81340	OR10Y1P	olfactory receptor, family 10, subfamily Y, member 1 pseudogene	1.00555	1.012287	1.0500154
81341	OR10W1	olfactory receptor, family 10, subfamily W, member 1	1.02929	1.044585	0.9708311
81343	OR10V2P	olfactory receptor, family 10, subfamily V, member 2 pseudogene	1.00931	1.047798	1.031405
81345	OR10Q2P	olfactory receptor, family 10, subfamily Q, member 2 pseudogene	0.99786	0.918052	1.0352034

81352	OR7M1P	olfactory receptor, family 7, subfamily M, member 1 pseudogene	0.96954	0.956394	0.9679089
81353	OR7E115P	olfactory receptor, family 7, subfamily E, member 115 pseudogene	0.97678	1.086874	1.0217814
81366	OR2AM1P	olfactory receptor, family 2, subfamily AM, member 1 pseudogene	1.03375	0.972085	1.0479908
81406	OR2W6P	olfactory receptor, family 2, subfamily W, member 6 pseudogene	0.99013	1.023219	1.0076427
81407	OR2W4P	olfactory receptor, family 2, subfamily W, member 4 pseudogene	1.02219	1.040428	1.0489848
81431	OR5AC1	olfactory receptor, family 5, subfamily AC, member 1 (gene/pseudogene)	1.00393	0.979161	1.0460442
81439	OR9H1P	olfactory receptor, family 9, subfamily H, member 1 pseudogene	1.01491	0.953568	0.9975129
81440	OR6R1P	olfactory receptor, family 6, subfamily R, member 1 pseudogene	0.98127	0.923378	0.9513656
81449	OR6K1P	olfactory receptor, family 6, subfamily K, member 1 pseudogene	1.04514	1.037425	0.9701854
81469	OR2G3	olfactory receptor, family 2, subfamily G, member 3	1.0075	1.020953	1.0543757
81470	OR2G2	olfactory receptor, family 2, subfamily G, member 2	0.98846	0.943362	0.9646499
81472	OR2C3	olfactory receptor, family 2, subfamily C, member 3	0.9806	0.970205	1.005898
81487	OR10AA1P	olfactory receptor, family 10, subfamily AA, member 1 pseudogene	1.04201	1.015415	1.0114971
81490	PTDSS2	phosphatidylserine synthase 2	0.99124	1.007887	1.0415052
81491	GPR63	G protein-coupled receptor 63	0.99133	0.992791	1.0158104
81492	RSPH6A	radial spoke head 6 homolog A (Chlamydomonas)	1.00483	0.982554	0.9984587
81493	SYNC	syncoilin, intermediate filament protein	1.02952	1.019139	0.9861284
81494	CFHR5	complement factor H-related 5	0.99393	1.011383	1.0321356
81501	TM7SF4	transmembrane 7 superfamily member 4	1.01276	1.027081	0.9843426
81502	HM13	histocompatibility (minor) 13	0.98458	1.00238	1.0337652
81532	MOB2	Mps one binder kinase activator-like 2	0.95248	0.980841	0.9837197
81533	ITFG1	integrin alpha FG-GAP repeat containing 1	0.9484	0.982635	1.0188013
81537	SGPP1	sphingosine-1-phosphate phosphatase 1	0.95784	0.97748	1.0320734
81539	SLC38A1	solute carrier family 38, member 1	0.96891	1.150958	0.9424999
81542	TMX1	thioredoxin-related transmembrane protein 1	0.95384	0.948521	1.1675883
81543	LRRC3	leucine rich repeat containing 3	0.99524	0.961671	1.0647423
81544	GDPD5	glycerophosphodiester phosphodiesterase domain containing 5	0.99067	0.918249	0.9851031
81545	FBXO38	F-box protein 38	0.98316	0.968044	1.0043676
81550	TDRD3	tudor domain containing 3	0.94111	0.945401	1.0503175
81551	STMN4	stathmin-like 4	1.01464	0.982969	0.9988864
81552	VOPP1	vesicular, overexpressed in cancer, prosurvival protein 1	1.0378	1.036604	0.9950578
81553	FAM49A	family with sequence similarity 49, member A	1.02168	1.050227	0.9436508
81554	WBSCR16	Williams-Beuren syndrome chromosome region 16	1.00195	0.954005	1.0668727

81555	YIPF5	Yip1 domain family, member 5	0.96043	0.994827	1.0404568
81556	C15orf44	chromosome 15 open reading frame 44	0.96435	0.909051	1.0294315
81558	FAM117A	family with sequence similarity 117, member A	0.96051	0.974171	0.9819325
81559	TRIM11	tripartite motif containing 11	1.00998	1.003437	0.9566258
81562	LMAN2L	lectin, mannose-binding 2-like	1.01647	0.987983	1.0327359
81563	C1orf21	chromosome 1 open reading frame 21	1.06276	1.030984	0.9632511
81565	NDEL1	nudE nuclear distribution gene E homolog (A. nidulans)-like 1	0.96869	0.92319	0.9823235
81566	CSRNP2	cysteine-serine-rich nuclear protein 2	0.95742	0.970369	0.9713078
81567	TXNDC5	thioredoxin domain containing 5 (endoplasmic reticulum)	1.01845	1.020294	0.9943739
81569	ACTL8	actin-like 8	1.00469	1.047961	1.0201611
81570	CLPB	ClpB caseinolytic peptidase B homolog (E. coli)	0.9529	0.983201	1.0222032
81571	NCRNA002	non-protein coding RNA 287	0.95328	1.095672	0.9746442
81572	PDRG1	p53 and DNA-damage regulated 1	0.93575	0.974803	1.0316306
81573	ANKRD13C	ankyrin repeat domain 13C	0.9855	1.008211	0.9634949
81575	APOLD1	apolipoprotein L domain containing 1	0.97354	1.028304	1.0828653
81576	CCDC130	coiled-coil domain containing 130	0.93292	1.042162	1.0055428
81577	GFOD2	glucose-fructose oxidoreductase domain containing 2	0.98624	1.00236	1.0609132
81578	COL21A1	collagen, type XXI, alpha 1	1.00888	1.016117	1.0016
81579	PLA2G12A	phospholipase A2, group XIIA	0.98235	0.998114	0.9816621
81602	CDADC1	cytidine and dCMP deaminase domain containing 1	0.94613	1.030428	1.0180424
81603	TRIM8	tripartite motif containing 8	0.9795	0.935564	1.00583
81605	URM1	ubiquitin related modifier 1	0.96342	0.904138	1.0549498
81606	LBH	limb bud and heart development homolog (mouse)	0.89549	0.977912	0.9310842
81607	PVRL4	poliovirus receptor-related 4	1.00801	1.008961	0.9783296
81608	FIP1L1	FIP1 like 1 (S. cerevisiae)	0.98289	0.861785	1.0345172
81609	SNX27	sorting nexin family member 27	1.02416	0.971475	0.9686028
81610	FAM83D	family with sequence similarity 83, member D	0.97924	1.024268	1.0343894
81611	ANP32E	acidic (leucine-rich) nuclear phosphoprotein 32 family, member E	1.03519	0.903388	1.0368024
81614	NIPA2	non imprinted in Prader-Willi/Angelman syndrome 2	0.97313	1.042108	1.029771
81615	TMEM163	transmembrane protein 163	0.99225	0.997072	1.001608
81616	ACSBG2	acyl-CoA synthetase bubblegum family member 2	0.99012	0.973431	0.9836171
81617	CAB39L	calcium binding protein 39-like	0.97892	0.945825	0.9992747
81618	ITM2C	integral membrane protein 2C	1.01857	0.907142	1.0413634

81619	TSPAN14	tetraspanin 14	0.96809	0.938591	0.9952889
81620	CDT1	chromatin licensing and DNA replication factor 1	0.98869	0.992217	1.0436882
81621	KAZALD1	Kazal-type serine peptidase inhibitor domain 1	0.9807	0.97411	1.0438813
81622	UNC93B1	unc-93 homolog B1 (C. elegans)	0.98025	0.906899	1.0946697
81623	DEFB126	defensin, beta 126	1.00099	0.998269	1.0314548
81624	DIAPH3	diaphanous homolog 3 (Drosophila)	0.99593	0.993617	1.003258
81626	SHCBP1L	SHC SH2-domain binding protein 1-like	1.00168	0.991237	1.0013435
81627	TRMT1L	TRM1 tRNA methyltransferase 1-like	0.9717	0.96609	1.0116801
81628	TSC22D4	TSC22 domain family, member 4	1.00123	1.005346	0.9401729
81629	TSSK3	testis-specific serine kinase 3	1.01387	1.002557	0.9461799
81631	MAP1LC3B	microtubule-associated protein 1 light chain 3 beta	1.02413	0.990342	0.8683436
81669	CCNL2	cyclin L2	0.9914	0.934146	1.0358012
81671	VMP1	vacuole membrane protein 1	0.96035	1.008036	1.021533
81688	C6orf62	chromosome 6 open reading frame 62	1.04115	0.984246	0.9326586
81689	ISCA1	iron-sulfur cluster assembly 1 homolog (S. cerevisiae)	0.99159	0.953715	0.9359969
81691	LOC81691	exonuclease NEF-sp	0.99308	0.977045	1.0138046
81693	AMN	amnionless homolog (mouse)	0.98048	1.012581	1.1301174
81694	OR2W2P	olfactory receptor, family 2, subfamily W, member 2 pseudogene	1.03136	0.984453	1.0391031
81695	OR2B7P	olfactory receptor, family 2, subfamily B, member 7 pseudogene	1.00585	1.060364	1.0090014
81696	OR5V1	olfactory receptor, family 5, subfamily V, member 1	0.98556	0.998956	1.0378654
81697	OR2B2	olfactory receptor, family 2, subfamily B, member 2	1.02597	1.036362	1.0147341
81704	DOCK8	dedicator of cytokinesis 8	0.97786	0.963619	1.0087197
81706	PPP1R14C	protein phosphatase 1, regulatory (inhibitor) subunit 14C	1.00109	1.019807	1.0159552
81786	TRIM7	tripartite motif containing 7	0.98925	1.031779	1.0121183
81787	DKFZP547L	hypothetical protein DKFZp547L112	0.9865	0.989397	1.048865
81788	NUAK2	NUAK family, SNF1-like kinase, 2	0.95825	0.960952	0.9672238
81789	TIGD6	tigger transposable element derived 6	1.01247	1.002537	1.0306352
81790	RNF170	ring finger protein 170	0.96599	0.988992	0.9805556
81792	ADAMTS12	ADAM metallopeptidase with thrombospondin type 1 motif, 12	1.00995	1.010854	0.9972839
81794	ADAMTS10	ADAM metallopeptidase with thrombospondin type 1 motif, 10	0.95513	0.989987	0.9422565
81797	OR12D3	olfactory receptor, family 12, subfamily D, member 3	0.97715	1.008552	1.0228118
81831	NETO2	neuropilin (NRP) and tolloid (TLL)-like 2	0.97554	1.033404	1.0310465
81832	NETO1	neuropilin (NRP) and tolloid (TLL)-like 1	0.98797	0.985105	0.9739801

81833	SPACA1	sperm acrosome associated 1	0.99883	0.990803	0.9977482
81839	VANGL1	vang-like 1 (van gogh, Drosophila)	1.01479	1.006333	1.0309714
81844	TRIM56	tripartite motif containing 56	1.00801	0.976537	0.9847071
81846	SBF2	SET binding factor 2	0.97089	1.003497	0.9519256
81847	RNF146	ring finger protein 146	1.05968	0.967477	0.9950987
81848	SPRY4	sprouty homolog 4 (Drosophila)	1.01907	1.023161	0.9999056
81849	ST6GALNAC	ST6 (alpha-N-acetyl-neuraminyl-2,3-beta-galactosyl-1,3)-N-acetylgalactosaminide alpha-2,6-sialyltransferase	1.01195	1.00666	1.015153
81851	KRTAP1-1	keratin associated protein 1-1	1.01633	0.95575	0.9930723
81853	TMEM14B	transmembrane protein 14B	0.96789	0.90675	1.0841926
81855	SFXN3	sideroflexin 3	0.95176	0.949927	1.0078082
81856	ZNF611	zinc finger protein 611	0.89846	0.994037	0.981913
81857	MED25	mediator complex subunit 25	0.96975	0.955432	1.011838
81858	SHARPIN	SHANK-associated RH domain interactor	0.96575	0.903798	1.0669751
81870	KRTAP9-9	keratin associated protein 9-9	1.01442	0.974988	0.9793458
81871	KRTAP4-6	keratin associated protein 4-6	1.00711	0.991756	1.027817
81872	KRTAP2-1	keratin associated protein 2-1	0.98916	0.965011	1.0706853
81873	ARPC5L	actin related protein 2/3 complex, subunit 5-like	0.98227	1.037278	1.063926
81875	ISG20L2	interferon stimulated exonuclease gene 20kDa-like 2	1.00797	0.930218	1.0172693
81876	RAB1B	RAB1B, member RAS oncogene family	0.98157	0.993728	1.0732654
81887	LAS1L	LAS1-like (S. cerevisiae)	0.96591	0.910307	0.9817741
81888	HYI	hydroxypyruvate isomerase (putative)	1.00912	0.992607	0.9920167
81889	FAHD1	fumarylacetoacetate hydrolase domain containing 1	0.91653	0.975092	1.0794409
81890	QTRT1	queuine tRNA-ribosyltransferase 1	0.93893	0.998165	1.0163127
81892	C14orf156	chromosome 14 open reading frame 156	0.96777	0.954131	1.0137374
81894	SLC25A28	solute carrier family 25, member 28	0.96085	0.922473	0.95126
81926	FAM108A1	family with sequence similarity 108, member A1	1.01039	0.987312	1.0107322
81928	CABLES2	Cdk5 and Abl enzyme substrate 2	0.99361	1.00355	1.0072177
81929	SEH1L	SEH1-like (S. cerevisiae)	0.92351	1.003535	1.0414768
81930	KIF18A	kinesin family member 18A	0.97727	0.95894	0.9886159
81931	ZNF93	zinc finger protein 93	0.98392	0.956296	1.0154168
81932	HDHD3	haloacid dehalogenase-like hydrolase domain containing 3	0.9702	0.94823	1.0700375
82500	PKP2P1	plakophilin 2 pseudogene 1	0.97133	0.983011	0.9603263
83259	PCDH11Y	protocadherin 11 Y-linked	0.98208	0.962537	1.0434077

83394	PITPNM3	PITPNM family member 3	0.98971	0.985002	1.0092724
83401	ELOVL3	ELOVL fatty acid elongase 3	0.99414	1.010041	0.9752263
83416	FCRL5	Fc receptor-like 5	1.03986	1.028306	1.0882144
83417	FCRL4	Fc receptor-like 4	1.00319	1.013125	1.0035997
83439	TCF7L1	transcription factor 7-like 1 (T-cell specific, HMG-box)	1.01128	1.003184	1.0060606
83440	ADPGK	ADP-dependent glucokinase	0.99267	0.961385	1.0030167
83442	SH3BGRL3	SH3 domain binding glutamic acid-rich protein like 3	1.00293	0.961794	0.9679254
83443	SF3B5	splicing factor 3b, subunit 5, 10kDa	0.91493	1.000167	1.1214822
83444	INO80B	INO80 complex subunit B	0.96311	0.979816	0.9611131
83445	GSG1	germ cell associated 1	0.99853	0.981223	0.993494
83446	CCDC70	coiled-coil domain containing 70	0.99943	1.051208	1.001037
83447	SLC25A31	solute carrier family 25 (mitochondrial carrier; adenine nucleotide translocator), member 31	1.00301	1.008359	1.0117259
83448	PUS7L	pseudouridylate synthase 7 homolog (S. cerevisiae)-like	0.92337	0.931834	1.063868
83449	PMFBP1	polyamine modulated factor 1 binding protein 1	0.99596	1.02178	1.010852
83450	LRRC48	leucine rich repeat containing 48	0.99566	0.992132	1.0014379
83451	ABHD11	abhydrolase domain containing 11	1.008	0.991798	1.0276063
83452	RAB33B	RAB33B, member RAS oncogene family	0.93582	0.970751	1.06271
83460	TMEM93	transmembrane protein 93	0.93564	0.994728	1.0488848
83461	CDCA3	cell division cycle associated 3	0.99371	1.013949	1.0065965
83463	MXD3	MAX dimerization protein 3	0.99908	1.012948	1.0434565
83464	APH1B	anterior pharynx defective 1 homolog B (C. elegans)	0.94931	0.953182	0.992286
83468	GLT8D2	glycosyltransferase 8 domain containing 2	1.00506	1.012546	1.024277
83473	KATNAL2	katanin p60 subunit A-like 2	0.9918	0.993363	1.0086239
83475	DOHH	deoxyhypusine hydroxylase/monooxygenase	0.96901	1.055009	1.1107467
83478	ARHGAP24	Rho GTPase activating protein 24	0.99727	1.039453	1.05664
83479	DDX59	DEAD (Asp-Glu-Ala-Asp) box polypeptide 59	0.99399	0.931551	0.9971786
83480	PUS3	pseudouridylate synthase 3	0.901	0.958353	0.9987116
83481	EPPK1	epiplakin 1	0.9451	0.977154	0.9426917
83482	SCRT1	scratch homolog 1, zinc finger protein (Drosophila)	0.99615	1.008184	1.0215088
83483	PLVAP	plasmalemma vesicle associated protein	0.99886	0.956859	1.027247
83528	CYP51P1	cytochrome P450, family 51, pseudogene 1	1.02082	0.964304	1.0217438
83538	TTC25	tetratricopeptide repeat domain 25	1.00052	0.974759	0.9853029
83539	CHST9	carbohydrate (N-acetylgalactosamine 4-0) sulfotransferase 9	0.99972	0.997569	0.9930485

83540	NUF2	NUF2, NDC80 kinetochore complex component, homolog (S. cerevisiae)	1.00377	0.954455	1.0235305
83541	FAM110A	family with sequence similarity 110, member A	0.96509	0.950798	1.0224307
83543	AIF1L	allograft inflammatory factor 1-like	0.99371	0.983869	0.9917348
83544	DNAL1	dynein, axonemal, light chain 1	0.95934	0.970222	1.0666189
83546	RTBDN	retbindin	0.96956	0.986749	1.0469469
83547	RILP	Rab interacting lysosomal protein	0.97328	0.980356	1.0620067
83548	COG3	component of oligomeric golgi complex 3	0.93818	0.959586	1.1597796
83549	UCK1	uridine-cytidine kinase 1	0.94213	1.00179	1.03457
83550	GPR101	G protein-coupled receptor 101	1.01242	1.028657	0.992264
83551	TAAR8	trace amine associated receptor 8	1.03953	1.017099	1.05798
83552	MFRP	membrane frizzled-related protein	1.00987	0.994747	0.9896031
83590	TMUB1	transmembrane and ubiquitin-like domain containing 1	1.00828	1.047469	0.9918827
83591	THAP2	THAP domain containing, apoptosis associated protein 2	0.96748	0.977099	1.0664925
83592	AKR1E2	aldo-keto reductase family 1, member E2	0.97524	0.977235	1.0279159
83593	RASSF5	Ras association (RalGDS/AF-6) domain family member 5	0.98592	1.005531	0.9535037
83594	NUDT12	nudix (nucleoside diphosphate linked moiety X)-type motif 12	1.00627	1.00386	1.0187716
83595	SOX7	SRY (sex determining region Y)-box 7	0.98036	1.023987	1.0310019
83596	BCL2L12	BCL2-like 12 (proline rich)	0.98555	0.986594	1.0305834
83597	RTP3	receptor (chemosensory) transporter protein 3	1.00481	1.014545	1.0283652
83598	LUZP3P	leucine zipper protein 3, pseudogene	1.01847	1.015523	1.0507964
83604	TMEM47	transmembrane protein 47	1.02285	0.968004	1.0109694
83605	CCM2	cerebral cavernous malformation 2	0.98383	0.997567	0.9200705
83606	C22orf13	chromosome 22 open reading frame 13	0.97607	0.996029	0.95426
83607	AMMECR1L	AMME chromosomal region gene 1-like	1.00905	0.97216	0.9719766
83608	C18orf21	chromosome 18 open reading frame 21	0.88415	0.920209	1.0442922
83636	C19orf12	chromosome 19 open reading frame 12	0.97626	0.979519	1.0190098
83637	ZMIZ2	zinc finger, MIZ-type containing 2	1.00753	1.049632	0.9819769
83638	C11orf68	chromosome 11 open reading frame 68	0.91015	0.944628	1.0478226
83639	TEX101	testis expressed 101	1.00495	1.007051	0.9955867
83640	FAM103A1	family with sequence similarity 103, member A1	0.95165	0.946013	0.9909185
83641	FAM107B	family with sequence similarity 107, member B	0.978	1.046007	0.8812477
83642	SELO	selenoprotein O	0.99114	1.009509	0.9948598
83643	CCDC3	coiled-coil domain containing 3	1.00387	0.972901	0.9938176

83648	FAM167A	family with sequence similarity 167, member A	1.0031	0.974931	1.0301127
83656	C8orf12	chromosome 8 open reading frame 12	0.99329	1.009713	1.0229737
83657	DYNLRB2	dynein, light chain, roadblock-type 2	1.00335	0.989518	1.0552175
83658	DYNLRB1	dynein, light chain, roadblock-type 1	0.95255	1.007695	1.0429802
83659	TEKT1	tektin 1	0.9951	1.033424	1.004106
83660	TLN2	talin 2	0.99551	0.996705	1.0168408
83661	MS4A8B	membrane-spanning 4-domains, subfamily A, member 8B	1.01661	1.005516	1.0140221
83666	PARP9	poly (ADP-ribose) polymerase family, member 9	1.00016	0.960516	1.0921751
83667	SESN2	sestrin 2	0.95614	0.95561	1.0227334
83690	CRISPLD1	cysteine-rich secretory protein LCCL domain containing 1	1.00625	1.007636	1.0150072
83692	CD99L2	CD99 molecule-like 2	0.9669	0.968477	0.9849987
83693	HSDL1	hydroxysteroid dehydrogenase like 1	0.92755	1.065647	1.0495176
83694	RPS6KL1	ribosomal protein S6 kinase-like 1	0.99283	0.977349	0.9687415
83695	C12orf32	chromosome 12 open reading frame 32	0.94716	0.900911	0.9791289
83696	TRAPPC9	trafficking protein particle complex 9	0.98997	0.987839	0.9557082
83697	SLC4A9	solute carrier family 4, sodium bicarbonate cotransporter, member 9	1.0094	1.012083	1.0163857
83698	CALN1	calneuron 1	1.00474	1.005863	1.0088496
83699	SH3BGRL2	SH3 domain binding glutamic acid-rich protein like 2	0.97563	0.942249	0.9378461
83700	JAM3	junctional adhesion molecule 3	0.96506	0.965982	0.971285
83706	FERMT3	fermitin family member 3	0.95145	1.010798	1.029375
83707	TRPT1	tRNA phosphotransferase 1	0.99186	1.023006	1.0274977
83714	NRIP2	nuclear receptor interacting protein 2	1.00664	1.019972	1.0306737
83715	ESPN	espin	0.98934	0.984411	1.0555578
83716	CRISPLD2	cysteine-rich secretory protein LCCL domain containing 2	0.99832	0.922983	1.0632705
83719	YPEL3	yippee-like 3 (Drosophila)	0.98472	0.955759	0.9038859
83723	FAM57B	family with sequence similarity 57, member B	0.9904	0.974228	0.9795346
83729	INHBE	inhibin, beta E	1.00213	1.011242	1.037085
83732	RIOK1	RIO kinase 1 (yeast)	0.95909	0.965206	1.0471176
83733	SLC25A18	solute carrier family 25 (mitochondrial carrier), member 18	1.00008	1.005566	1.0176789
83734	ATG10	ATG10 autophagy related 10 homolog (S. cerevisiae)	0.99767	0.98878	0.9842613
83737	ITCH	itchy E3 ubiquitin protein ligase homolog (mouse)	0.96104	1.100508	0.9859021
83741	TFAP2D	transcription factor AP-2 delta (activating enhancer binding protein 2 delta)	0.98991	0.987368	1.0024144
83743	GRWD1	glutamate-rich WD repeat containing 1	0.94639	0.97024	1.0464381

83744	ZNF484	zinc finger protein 484	0.90139	0.898067	0.9826657
83746	L3MBTL2	l(3)mbt-like 2 (Drosophila)	0.95517	0.915586	0.9849873
83752	LONP2	lon peptidase 2, peroxisomal	0.94863	0.939772	1.0739724
83756	TAS1R3	taste receptor, type 1, member 3	0.99976	0.998088	1.0312845
83757	CYP2G2P	cytochrome P450, family 2, subfamily G, polypeptide 2 pseudogene	1.01534	0.994976	0.9623995
83758	RBP5	retinol binding protein 5, cellular	1.04073	0.989897	1.0075615
83759	RBM4B	RNA binding motif protein 4B	0.97386	1.021424	1.0379427
83786	FRMD8	FERM domain containing 8	0.95635	1.000797	1.0062865
83787	ARMC10	armadillo repeat containing 10	0.97279	0.976018	0.9474993
83795	KCNK16	potassium channel, subfamily K, member 16	1.01533	1.017276	0.9980919
83844	USP26	ubiquitin specific peptidase 26	0.95404	1.056239	0.9561277
83849	SYT15	synaptotagmin XV	0.989	0.998887	1.0221501
83850	ESYT3	extended synaptotagmin-like protein 3	1.00141	1.005269	1.0130132
83851	SYT16	synaptotagmin XVI	0.98862	0.989557	0.9938812
83852	SETDB2	SET domain, bifurcated 2	0.9661	1.007618	1.000926
83853	ROPN1L	rhophilin associated tail protein 1-like	1.01252	0.999717	0.9998664
83854	ANGPTL6	angiopoietin-like 6	0.99851	0.973348	1.0558412
83855	KLF16	Kruppel-like factor 16	0.96561	0.996951	1.0908945
83856	FSD1L	fibronectin type III and SPRY domain containing 1-like	0.95275	0.974049	1.0118473
83857	TMTC1	transmembrane and tetratricopeptide repeat containing 1	0.99756	0.998075	1.0474073
83858	ATAD3B	ATPase family, AAA domain containing 3B	1.01682	1.016506	0.9535847
83860	TAF3	TAF3 RNA polymerase II, TATA box binding protein (TBP)-associated factor, 140kDa	0.9616	0.967491	1.0123908
83861	RSPH3	radial spoke 3 homolog (Chlamydomonas)	0.9972	0.975495	1.016562
83862	TMEM120A	transmembrane protein 120A	0.95099	0.998142	1.0687555
83863	TTTY5	testis-specific transcript, Y-linked 5 (non-protein coding)	0.98704	0.967083	1.0020785
83866	TTTY11	testis-specific transcript, Y-linked 11 (non-protein coding)	0.98373	0.98031	0.979841
83867	TTTY12	testis-specific transcript, Y-linked 12 (non-protein coding)	0.98748	1.020846	0.9840294
83868	TTTY13	testis-specific transcript, Y-linked 13 (non-protein coding)	0.99992	0.983524	0.9995696
83869	TTTY14	testis-specific transcript, Y-linked 14 (non-protein coding)	0.98539	0.972301	0.995368
83871	RAB34	RAB34, member RAS oncogene family	0.97824	0.996518	0.974443
83873	GPR61	G protein-coupled receptor 61	0.99875	0.973915	0.9664124
83874	TBC1D10A	TBC1 domain family, member 10A	0.94059	1.103936	0.9730921
83875	BCO2	beta-carotene oxygenase 2	0.99439	1.034892	0.9889594

83876	MRO	maestro	1.00576	1.005662	1.0021954
83877	TM2D2	TM2 domain containing 2	0.90234	0.955733	1.0032046
83878	USHBP1	Usher syndrome 1C binding protein 1	0.99905	0.961787	0.9846482
83879	CDCA7	cell division cycle associated 7	1.01714	0.934653	1.0345581
83881	MIXL1	Mix paired-like homeobox	0.97132	1.009425	1.0571633
83882	TSPAN10	tetraspanin 10	1.00947	0.999648	1.0155967
83884	SLC25A2	solute carrier family 25 (mitochondrial carrier; ornithine transporter) member 2	1.01528	0.934362	0.9823226
83886	PRSS27	protease, serine 27	0.98836	0.973964	1.0075439
83887	TTLL2	tubulin tyrosine ligase-like family, member 2	1.01892	0.973574	1.0216098
83888	FGFBP2	fibroblast growth factor binding protein 2	0.9936	0.90709	0.8916039
83889	WDR87	WD repeat domain 87	1.00842	1.003256	0.9775105
83890	SPATA9	spermatogenesis associated 9	1.00704	0.98663	1.0237336
83891	SNX25	sorting nexin 25	0.9787	0.982627	0.9888572
83892	KCTD10	potassium channel tetramerisation domain containing 10	0.94166	0.939824	1.0051657
83893	SPATA16	spermatogenesis associated 16	1.01088	1.017071	1.0052326
83894	TTC29	tetratricopeptide repeat domain 29	0.99648	0.990477	1.0262883
83895	KRTAP1-5	keratin associated protein 1-5	1.02082	1.034586	1.0164835
83896	KRTAP3-1	keratin associated protein 3-1	0.99735	0.951043	0.9773577
83897	KRTAP3-2	keratin associated protein 3-2	1.00208	0.969027	0.9904445
83902	KRTAP17-1	keratin associated protein 17-1	1.01472	0.927437	1.0780388
83903	GSG2	germ cell associated 2 (haspin)	1.01527	1.009848	1.0112852
83930	STARD3NL	STARD3 N-terminal like	0.9903	1.01555	0.984914
83931	STK40	serine/threonine kinase 40	1.01882	1.032108	0.9444999
83932	C1orf124	chromosome 1 open reading frame 124	0.97159	0.916188	0.9887913
83933	HDAC10	histone deacetylase 10	0.98461	1.00604	1.0394785
83935	TMEM133	transmembrane protein 133	1.0019	0.956559	0.9974516
83937	RASSF4	Ras association (RalGDS/AF-6) domain family member 4	0.99082	0.930259	1.0210044
83938	C10orf11	chromosome 10 open reading frame 11	0.99674	0.963848	0.995158
83939	EIF2A	eukaryotic translation initiation factor 2A, 65kDa	0.98983	0.977195	0.9968692
83940	TATDN1	TatD DNase domain containing 1	0.97692	0.977262	1.0264662
83941	TM2D1	TM2 domain containing 1	1.00006	0.946948	1.0347806
83943	IMMP2L	IMP2 inner mitochondrial membrane peptidase-like (S. cerevisiae)	0.9888	0.960072	0.9996154
83953	FCAMR	Fc receptor, IgA, IgM, high affinity	1.00974	1.016689	1.012935

83955	NACAP1	nascent-polypeptide-associated complex alpha polypeptide pseudogene 1	1.01526	0.986429	0.986282
83956	RACGAP1P	Rac GTPase activating protein 1 pseudogene	0.99861	1.030695	1.0367369
83957	FKSG43	FERM domain-containing protein 8 pseudogene	1.01557	1.043904	1.0044053
83959	SLC4A11	solute carrier family 4, sodium borate transporter, member 11	0.99058	1.017721	1.061365
83982	IFI27L2	interferon, alpha-inducible protein 27-like 2	0.94904	1.081796	1.0087597
83983	TSSK6	testis-specific serine kinase 6	0.9324	0.93683	1.0141
83985	SPNS1	spinster homolog 1 (Drosophila)	0.98449	0.973805	1.0268311
83986	ITFG3	integrin alpha FG-GAP repeat containing 3	0.95901	0.97082	1.0064372
83987	CCDC8	coiled-coil domain containing 8	0.96741	1.03051	0.9958272
83988	NCALD	neurocalcin delta	0.98638	1.055468	0.9880952
83989	FAM172A	family with sequence similarity 172, member A	0.99554	0.883057	0.9769571
83990	BRIP1	BRCA1 interacting protein C-terminal helicase 1	0.97896	0.992991	1.0026886
83992	CTTNBP2	cortactin binding protein 2	1.00463	0.999437	1.0149695
83998	REG4	regenerating islet-derived family, member 4	0.99555	1.028605	1.0181835
83999	KREMEN1	kringle containing transmembrane protein 1	1.0029	1.053639	0.9985549
84000	TMPRSS13	transmembrane protease, serine 13	1.00009	0.994389	0.9979237
84002	B3GNT5	UDP-GlcNAc:betaGal beta-1,3-N-acetylglucosaminyltransferase 5	1.02563	1.005672	0.9420825
84033	OBSCN	obscurin, cytoskeletal calmodulin and titin-interacting RhoGEF	0.98859	0.978683	0.9508197
84034	EMILIN2	elastin microfibril interfacer 2	0.98013	1.015751	1.0143479
84054	PCDHB19P	protocadherin beta 19 pseudogene	0.9892	0.988847	1.0206153
84056	KATNAL1	katanin p60 subunit A-like 1	0.9654	0.928227	1.0247844
84057	MND1	meiotic nuclear divisions 1 homolog (S. cerevisiae)	1.00677	0.993686	1.0199053
84058	WDR54	WD repeat domain 54	0.96657	1.032402	1.0552069
84059	GPR98	G protein-coupled receptor 98	0.99574	0.99552	1.0073875
84060	C7orf64	chromosome 7 open reading frame 64	0.95856	0.879923	1.0162599
84061	MAGT1	magnesium transporter 1	0.97634	1.0298	1.0612579
84062	DTNBP1	dystrobrevin binding protein 1	0.99492	0.989971	0.9917617
84063	KIRREL2	kin of IRRE like 2 (Drosophila)	0.99275	1.015288	1.0026055
84064	HDHD2	haloacid dehalogenase-like hydrolase domain containing 2	0.95194	0.957471	0.996536
84065	TMEM222	transmembrane protein 222	0.98332	0.950805	1.0425441
84066	C1orf49	chromosome 1 open reading frame 49	0.99585	1.012496	1.0360761
84067	FAM160A2	family with sequence similarity 160, member A2	0.97317	1.019965	0.9985065
84068	SLC10A7	solute carrier family 10 (sodium/bile acid cotransporter family), member 7	0.98597	0.986908	1.0178935

84069	PLEKHN1	pleckstrin homology domain containing, family N member 1	1.00626	0.972681	1.0615735
84070	FAM186B	family with sequence similarity 186, member B	0.99436	1.008707	0.9951921
84071	ARMC2	armadillo repeat containing 2	0.99782	0.976329	1.0067571
84072	HORMAD1	HORMA domain containing 1	1.03259	0.990099	0.9605253
84073	MYCBPAP	MYCBP associated protein	0.98604	1.007973	1.0226964
84074	QRICH2	glutamine rich 2	0.99523	1	0.9947125
84075	FSCB	fibrous sheath CABYR binding protein	0.97505	1.02481	0.9449397
84076	TKTL2	transketolase-like 2	1.00375	0.974803	0.997528
84077	C3orf20	chromosome 3 open reading frame 20	1.00784	0.964084	0.9964671
84078	KBTBD7	kelch repeat and BTB (POZ) domain containing 7	0.91477	0.987744	1.0007697
84079	ANKRD27	ankyrin repeat domain 27 (VPS9 domain)	0.94091	0.970547	1.0294572
84080	C16orf48	chromosome 16 open reading frame 48	0.99714	0.962727	1.047666
84081	NSRP1	nuclear speckle splicing regulatory protein 1	0.96669	0.987568	1.0235815
84083	ZRANB3	zinc finger, RAN-binding domain containing 3	0.99798	0.97397	0.9976773
84085	FBXO30	F-box protein 30	0.98608	0.976349	1.083741
84099	ID2B	inhibitor of DNA binding 2B, dominant negative helix-loop-helix protein (pseudogene)	1.01996	0.963234	0.9904887
84100	ARL6	ADP-ribosylation factor-like 6	0.95982	0.967439	1.0269801
84101	USP44	ubiquitin specific peptidase 44	0.95122	0.984222	0.9226633
84102	SLC41A2	solute carrier family 41, member 2	0.99805	1.018283	0.9833024
84103	C4orf17	chromosome 4 open reading frame 17	1.0075	1.007045	1.0370648
84105	PCBD2	pterin-4 alpha-carbinolamine dehydratase/dimerization cofactor of hepatocyte nuclear factor 1 alpha (TCF1)	1.01848	1.019374	0.9883287
84106	PRAM1	PML-RARA regulated adaptor molecule 1	0.99939	0.950751	1.0276007
84107	ZIC4	Zic family member 4	0.99751	1.025	1.0193524
84108	PCGF6	polycomb group ring finger 6	1.00731	0.973904	1.021358
84109	QRFPR	pyroglutamylated RFamide peptide receptor	1.0102	1.018307	1.0152939
84124	ZNF394	zinc finger protein 394	0.99456	0.903609	0.9494848
84125	LRRIQ1	leucine-rich repeats and IQ motif containing 1	0.99076	0.99454	1.0186399
84126	ATRIP	ATR interacting protein	0.97779	0.95621	1.0268421
84128	WDR75	WD repeat domain 75	0.97044	0.916295	1.0876721
84129	ACAD11	acyl-CoA dehydrogenase family, member 11	0.98668	0.996288	1.0212379
84131	CEP78	centrosomal protein 78kDa	0.93057	1.101943	0.9652978
84132	USP42	ubiquitin specific peptidase 42	0.99145	0.884759	0.9227479
84133	ZNRF3	zinc and ring finger 3	1.00795	0.969653	0.9611114

84134	TOMM40L	translocase of outer mitochondrial membrane 40 homolog (yeast)-like	1.01154	1.051692	0.9087988
84135	UTP15	UTP15, U3 small nucleolar ribonucleoprotein, homolog (S. cerevisiae)	0.95823	0.852707	1.0332972
84138	SLC7A6OS	solute carrier family 7, member 6 opposite strand	0.95225	0.977357	1.0785122
84140	FAM161A	family with sequence similarity 161, member A	0.99956	0.961288	1.020878
84141	FAM176A	family with sequence similarity 176, member A	1.00684	0.990711	1.0077849
84142	FAM175A	family with sequence similarity 175, member A	0.97389	0.951201	1.0186909
84144	SYDE2	synapse defective 1, Rho GTPase, homolog 2 (C. elegans)	0.98282	0.940861	0.9453032
84146	ZNF644	zinc finger protein 644	0.9805	0.87129	0.9012671
84148	MYST1	MYST histone acetyltransferase 1	0.94288	0.957111	1.0341728
84152	PPP1R1B	protein phosphatase 1, regulatory (inhibitor) subunit 1B	1.00756	0.996318	1.0210777
84153	RNASEH2C	ribonuclease H2, subunit C	0.94325	0.95999	1.0799775
84154	RPF2	ribosome production factor 2 homolog (S. cerevisiae)	0.9799	0.962004	0.9948016
84159	ARID5B	AT rich interactive domain 5B (MRF1-like)	0.97803	1.054521	0.9452223
84162	KIAA1109	KIAA1109	0.9942	1.007202	0.9982879
84164	ASCC2	activating signal cointegrator 1 complex subunit 2	0.95614	0.951056	1.0966398
84166	NLRC5	NLR family, CARD domain containing 5	0.9847	1.055885	1.0555968
84167	C19orf44	chromosome 19 open reading frame 44	0.96752	0.959195	1.0153189
84168	ANTXR1	anthrax toxin receptor 1	1.00929	1.023282	1.0058084
84171	LOXL4	lysyl oxidase-like 4	0.98511	1.001916	1.0132874
84172	POLR1B	polymerase (RNA) I polypeptide B, 128kDa	0.98304	0.94331	1.0254002
84173	ELMOD3	ELMO/CED-12 domain containing 3	0.99134	0.999066	0.9694967
84174	SLA2	Src-like-adaptor 2	0.90125	1.024865	0.9384198
84176	MYH16	myosin, heavy chain 16 pseudogene	1.00549	1.023816	1.0320504
84179	MFSD7	major facilitator superfamily domain containing 7	1.02231	0.985984	1.0099892
84181	CHD6	chromodomain helicase DNA binding protein 6	0.93783	0.979102	1.0018637
84186	ZCCHC7	zinc finger, CCHC domain containing 7	0.97688	1.058884	1.0296274
84187	TMEM164	transmembrane protein 164	0.98913	0.985191	1.0079197
84188	FAR1	fatty acyl CoA reductase 1	0.99718	0.951548	1.0399854
84189	SLITRK6	SLIT and NTRK-like family, member 6	0.99886	0.986854	0.997868
84190	C12orf26	chromosome 12 open reading frame 26	0.9471	0.96162	1.0081211
84191	FAM96A	family with sequence similarity 96, member A	0.97888	0.967485	1.0382413
84193	SETD3	SET domain containing 3	0.96211	0.950738	1.075122
84196	USP48	ubiquitin specific peptidase 48	0.99195	0.961107	1.1013001

84197	SGK196	protein kinase-like protein SgK196	0.99987	0.943125	1.0480247
84203	TXNDC2	thioredoxin domain containing 2 (spermatozoa)	0.98258	1.02007	1.0025133
84206	MEX3B	mex-3 homolog B (C. elegans)	0.99687	0.943283	1.0249708
84215	ZNF541	zinc finger protein 541	0.99862	1.005006	1.001199
84216	TMEM117	transmembrane protein 117	0.98876	0.945191	0.9910353
84217	ZMYND12	zinc finger, MYND-type containing 12	0.97379	0.985927	0.9857241
84219	WDR24	WD repeat domain 24	0.96201	0.962755	1.0562231
84221	C21orf56	chromosome 21 open reading frame 56	0.96953	1.016386	1.1240197
84222	TMEM191A	transmembrane protein 191A	0.98876	1.011831	1.0823965
84223	IQCG	IQ motif containing G	0.99337	0.968939	1.0086059
84224	NBPF3	neuroblastoma breakpoint family, member 3	0.96777	0.897236	0.9616316
84225	ZMYND15	zinc finger, MYND-type containing 15	1.0125	1.006389	0.9833276
84226	C2orf16	chromosome 2 open reading frame 16	1.02482	1.044481	0.9992146
84229	CCDC135	coiled-coil domain containing 135	0.98543	1.01601	0.9848625
84230	LRRC8C	leucine rich repeat containing 8 family, member C	0.98869	0.976814	0.9895204
84231	TRAF7	TNF receptor-associated factor 7	0.93838	0.968531	1.0618625
84232	MAF1	MAF1 homolog (S. cerevisiae)	0.97364	0.964957	1.0198498
84233	TMEM126A	transmembrane protein 126A	0.96189	1.001646	0.9958642
84236	RHBDD1	rhomboid domain containing 1	0.98726	0.978354	1.0357779
84239	ATP13A4	ATPase type 13A4	1.00149	0.983748	1.0134399
84240	ZCCHC9	zinc finger, CCHC domain containing 9	0.99961	0.925013	0.9839586
84243	ZDHHC18	zinc finger, DHHC-type containing 18	1.0093	0.987293	1.0529966
84245	MRI1	methylthioribose-1-phosphate isomerase homolog (S. cerevisiae)	0.95227	0.912531	1.0218591
84246	MED10	mediator complex subunit 10	1.00021	0.918083	1.0720501
84247	LDOC1L	leucine zipper, down-regulated in cancer 1-like	0.92353	1.07641	0.9782118
84248	FYTTD1	forty-two-three domain containing 1	0.97161	1.004105	1.0190974
84249	PSD2	pleckstrin and Sec7 domain containing 2	0.9925	1.030529	1.0041152
84250	ANKRD32	ankyrin repeat domain 32	0.98489	1.058386	1.1058768
84253	GARNL3	GTPase activating Rap/RanGAP domain-like 3	0.984	1.003033	1.012704
84254	CAMKK1	calcium/calmodulin-dependent protein kinase kinase 1, alpha	1.00329	1.040711	0.9612338
84255	SLC37A3	solute carrier family 37 (glycerol-3-phosphate transporter), member 3	1.01145	0.954169	1.0023817
84256	FLYWCH1	FLYWCH-type zinc finger 1	1.01483	1.035677	0.9839745
84258	SYT3	synaptotagmin III	0.98464	0.980037	0.9750722

84259	DCUN1D5	DCN1, defective in cullin neddylation 1, domain containing 5 (S. cerevisiae)	0.93258	0.902841	1.0383531
84260	TCHP	trichoplein, keratin filament binding	0.95304	0.962894	0.9898467
84261	FBXW9	F-box and WD repeat domain containing 9	0.98124	1.007001	1.018236
84262	PSMG3	proteasome (prosome, macropain) assembly chaperone 3	0.9671	0.953158	1.0089223
84263	HSDL2	hydroxysteroid dehydrogenase like 2	0.97925	0.99689	1.0122125
84264	HAGHL	hydroxyacylglutathione hydrolase-like	0.98797	0.977218	1.0937487
84265	POLR3GL	polymerase (RNA) III (DNA directed) polypeptide G (32kD)-like	0.98303	0.951809	0.992597
84266	ALKBH7	alkB, alkylation repair homolog 7 (E. coli)	0.96173	0.99347	1.1077158
84267	C9orf64	chromosome 9 open reading frame 64	0.96512	0.971188	0.992208
84268	RPAIN	RPA interacting protein	0.978	1.048845	1.0539091
84269	CHCHD5	coiled-coil-helix-coiled-coil-helix domain containing 5	0.98237	0.971032	1.0374978
84270	C9orf89	chromosome 9 open reading frame 89	0.96319	1.043062	0.967419
84271	POLDIP3	polymerase (DNA-directed), delta interacting protein 3	0.95026	1.035625	1.03894
84272	YIPF4	Yip1 domain family, member 4	0.98464	0.997844	0.962432
84273	C4orf14	chromosome 4 open reading frame 14	0.94017	0.921211	1.0653762
84274	COQ5	coenzyme Q5 homolog, methyltransferase (S. cerevisiae)	0.94305	1.00357	1.0056456
84275	SLC25A33	solute carrier family 25, member 33	1.03112	0.963903	0.9984697
84276	NICN1	nicolin 1	0.99036	0.995803	1.0249241
84277	DNAJC30	DnaJ (Hsp40) homolog, subfamily C, member 30	0.94005	0.862213	1.0370344
84279	PRADC1	protease-associated domain containing 1	0.97462	0.982629	0.9918785
84280	BTBD10	BTB (POZ) domain containing 10	0.9746	0.951866	1.0133695
84281	C2orf88	chromosome 2 open reading frame 88	0.9757	0.91063	0.9630551
84282	RNF135	ring finger protein 135	0.96291	0.943849	1.0604563
84283	TMEM79	transmembrane protein 79	0.99188	0.980258	1.0295337
84284	NTPCR	nucleoside-triphosphatase, cancer-related	0.99327	0.985033	1.0318317
84285	EIF1AD	eukaryotic translation initiation factor 1A domain containing	0.87702	0.900793	1.0782627
84286	TMEM175	transmembrane protein 175	1.01433	1.026667	0.9479035
84287	ZDHHC16	zinc finger, DHHC-type containing 16	0.93429	0.987662	0.9834387
84288	EFCAB2	EF-hand calcium binding domain 2	1.00294	0.987598	1.0157116
84289	ING5	inhibitor of growth family, member 5	0.97826	0.95583	1.0019443
84292	WDR83	WD repeat domain 83	0.98166	1.021126	1.0258802
84293	C10orf58	chromosome 10 open reading frame 58	0.99706	1.017629	1.0160634
84294	UTP23	UTP23, small subunit (SSU) processome component, homolog (yeast)	0.93281	0.913842	1.0711383

84295	PHF6	PHD finger protein 6	0.96621	0.95057	1.0361621
84296	GINS4	GINS complex subunit 4 (Sld5 homolog)	0.99612	1.026533	1.0240596
84298	LLPH	LLP homolog, long-term synaptic facilitation (Aplysia)	0.97092	0.915241	1.0799983
84299	C17orf37	chromosome 17 open reading frame 37	0.9176	0.92968	0.959459
84300	C6orf125	chromosome 6 open reading frame 125	0.99407	1.014856	1.0227322
84301	DDI2	DNA-damage inducible 1 homolog 2 (S. cerevisiae)	0.99423	1.084601	0.9953286
84302	C9orf125	chromosome 9 open reading frame 125	1.01334	0.974313	1.0098839
84303	CHCHD6	coiled-coil-helix-coiled-coil-helix domain containing 6	1.01427	1.001398	1.0095793
84304	NUDT22	nudix (nucleoside diphosphate linked moiety X)-type motif 22	0.96891	1.030093	1.0148064
84305	WIBG	within bgcn homolog (Drosophila)	0.95156	0.966367	1.0155054
84306	PDCD2L	programmed cell death 2-like	0.95519	0.995448	1.0321945
84307	ZNF397	zinc finger protein 397	0.97153	0.982464	0.9775891
84309	NUDT16L1	nudix (nucleoside diphosphate linked moiety X)-type motif 16-like 1	0.97169	0.919578	1.0028239
84310	C7orf50	chromosome 7 open reading frame 50	1.0056	1.010431	1.0142566
84311	MRPL45	mitochondrial ribosomal protein L45	0.92874	0.959734	1.0506873
84312	BRMS1L	breast cancer metastasis-suppressor 1-like	0.94591	1.029407	1.0395186
84313	VPS25	vacuolar protein sorting 25 homolog (S. cerevisiae)	0.94936	0.94967	0.9985466
84314	TMEM107	transmembrane protein 107	0.97187	0.970766	1.0471517
84315	MON1A	MON1 homolog A (yeast)	0.9884	1.028037	1.0108393
84316	LSMD1	LSM domain containing 1	0.9235	0.97768	1.0464901
84317	CCDC115	coiled-coil domain containing 115	0.95419	0.86995	1.0905502
84318	CCDC77	coiled-coil domain containing 77	0.95136	0.895352	0.9928271
84319	C3orf26	chromosome 3 open reading frame 26	0.9945	0.999557	0.9947833
84320	ACBD6	acyl-CoA binding domain containing 6	0.98514	0.929359	1.0015078
84324	SARNP	SAP domain containing ribonucleoprotein	0.97997	0.986362	0.9484317
84326	C16orf13	chromosome 16 open reading frame 13	0.93957	0.936294	0.9902652
84327	ZBED3	zinc finger, BED-type containing 3	1.01146	0.991781	1.0211881
84328	LZIC	leucine zipper and CTNNBIP1 domain containing	0.98767	0.871371	1.0990566
84330	ZNF414	zinc finger protein 414	0.9532	0.995117	1.0459253
84331	FAM195A	family with sequence similarity 195, member A	0.99523	1.013966	1.0621488
84332	DYDC2	DPY30 domain containing 2	0.98687	1.003749	1.002999
84333	PCGF5	polycomb group ring finger 5	0.93396	1.034877	1.0378988
84334	C14orf153	chromosome 14 open reading frame 153	0.97976	1.037698	1.0248863

84335	AKT1S1	AKT1 substrate 1 (proline-rich)	0.98232	0.985051	1.0752587
84336	TMEM101	transmembrane protein 101	0.94877	0.992656	1.0284687
84337	ELOF1	elongation factor 1 homolog (S. cerevisiae)	0.95546	1.007396	1.0411731
84340	GFM2	G elongation factor, mitochondrial 2	0.98455	0.994837	1.0673073
84342	COG8	component of oligomeric golgi complex 8	0.93296	0.957638	1.0492698
84343	HPS3	Hermansky-Pudlak syndrome 3	0.99326	0.987998	0.9253062
84364	ARFGAP2	ADP-ribosylation factor GTPase activating protein 2	0.97319	0.911793	1.0767973
84365	MKI67IP	MKI67 (FHA domain) interacting nucleolar phosphoprotein	0.93227	0.909593	1.0412378
84366	PRAC	prostate cancer susceptibility candidate	0.97732	1.017111	1.0446672
84376	HOOK3	hook homolog 3 (Drosophila)	1.02482	0.963333	0.9790298
84417	C2orf40	chromosome 2 open reading frame 40	0.99976	1.028479	1.040428
84418	C5orf32	chromosome 5 open reading frame 32	1.00911	1.041395	1.0155609
84419	C15orf48	chromosome 15 open reading frame 48	0.96454	1.090551	0.8500561
84432	PROK1	prokineticin 1	1.00183	1.046599	0.9770894
84433	CARD11	caspase recruitment domain family, member 11	0.9892	1.014429	0.9369691
84435	GPR123	G protein-coupled receptor 123	0.97873	0.991318	1.0292799
84436	ZNF528	zinc finger protein 528	0.94892	0.953904	1.004399
84437	KIAA1826	KIAA1826	0.89257	0.99251	1.1497108
84439	HHIPL1	HHIP-like 1	1.01002	0.998825	1.0339477
84440	RAB11FIP4	RAB11 family interacting protein 4 (class II)	0.97966	0.994031	0.9757862
84441	MAML2	mastermind-like 2 (Drosophila)	0.95078	0.950036	1.0045432
84443	FRMPD3	FERM and PDZ domain containing 3	0.99015	1.005919	0.973206
84444	DOT1L	DOT1-like, histone H3 methyltransferase (S. cerevisiae)	0.97916	1.068621	1.0157259
84445	LZTS2	leucine zipper, putative tumor suppressor 2	0.96875	1.007725	1.0210344
84446	BRSK1	BR serine/threonine kinase 1	0.99086	1	1.021723
84447	SYVN1	synovial apoptosis inhibitor 1, synoviolin	0.97731	0.983404	0.994213
84448	ABLIM2	actin binding LIM protein family, member 2	1.00685	1.00467	1.0118547
84449	ZNF333	zinc finger protein 333	0.98479	1.07088	0.9911753
84450	ZNF512	zinc finger protein 512	0.95622	0.936959	1.0594032
84451	KIAA1804	mixed lineage kinase 4	1.00026	0.982114	1.0312894
84455	EFCAB7	EF-hand calcium binding domain 7	1.00606	0.956769	1.0236119
84456	L3MBTL3	l(3)mbt-like 3 (Drosophila)	0.97547	0.88014	0.9817755
84457	PHYHIPL	phytanoyl-CoA 2-hydroxylase interacting protein-like	1.00484	1.021279	0.9910054

84458	LCOR	ligand dependent nuclear receptor corepressor	0.96643	0.979071	0.9843355
84460	ZMAT1	zinc finger, matrin-type 1	0.94559	0.956051	0.9260407
84461	NEURL4	neuralized homolog 4 (Drosophila)	0.96435	1.029184	1.0026202
84464	SLX4	SLX4 structure-specific endonuclease subunit homolog (S. cerevisiae)	0.98578	0.964784	0.972954
84465	MEGF11	multiple EGF-like-domains 11	1.00329	1.002154	1.0071806
84466	MEGF10	multiple EGF-like-domains 10	1.00038	1.018315	1.0118116
84467	FBN3	fibrillin 3	0.98935	0.989265	1.0056477
84498	FAM120B	family with sequence similarity 120B	0.97839	0.928963	0.9672542
84501	SPIRE2	spire homolog 2 (Drosophila)	0.99506	0.980745	1.0486322
84502	JPH4	junctophilin 4	0.99604	0.984231	1.0414166
84503	ZNF527	zinc finger protein 527	0.94338	1.026282	1.0749237
84504	NKX6-2	NK6 homeobox 2	0.97473	1.060417	1.0915008
84513	PPAPDC1B	phosphatidic acid phosphatase type 2 domain containing 1B	0.94636	0.996645	1.0799627
84514	GHDC	GH3 domain containing	0.95939	0.961829	1.0898121
84515	MCM8	minichromosome maintenance complex component 8	0.93679	0.98176	0.9784985
84516	DCTN5	dynactin 5 (p25)	0.95188	0.956866	1.0878624
84517	ARPM1	actin related protein M1	1.01672	1.043549	1.0191868
84518	CNFN	cornifelin	0.98726	0.980739	1.0504925
84519	ACRBP	acrosin binding protein	0.95832	0.930184	1.0108861
84520	C14orf142	chromosome 14 open reading frame 142	0.95871	0.974024	1.0667395
84522	JAGN1	jagunal homolog 1 (Drosophila)	0.96027	0.928863	1.0776937
84524	ZC3H8	zinc finger CCCH-type containing 8	0.97984	0.998903	0.9687962
84525	HOPX	HOP homeobox	1.01052	0.995688	1.0092965
84527	ZNF559	zinc finger protein 559	0.93399	0.988393	0.9184532
84529	C15orf41	chromosome 15 open reading frame 41	0.97864	0.957997	1.0053776
84530	SRRM4	serine/arginine repetitive matrix 4	0.99804	1.013244	0.9909784
84532	ACSS1	acyl-CoA synthetase short-chain family member 1	0.97979	0.975185	0.96691
84536	C21orf67	chromosome 21 open reading frame 67	1.00384	1.017224	1.0092843
84539	MCHR2	melanin-concentrating hormone receptor 2	1.00621	1.006701	1.0248475
84541	KBTBD8	kelch repeat and BTB (POZ) domain containing 8	0.96466	0.98015	0.9346016
84542	KIAA1841	KIAA1841	0.99752	0.97804	1.0379724
84545	MRPL43	mitochondrial ribosomal protein L43	0.94556	0.970255	1.0781785
84547	PGBD1	piggyBac transposable element derived 1	1.01358	1.007461	1.007533

84549	MAK16	MAK16 homolog (S. cerevisiae)	0.92893	0.941224	1.0729996
84552	PARD6G	par-6 partitioning defective 6 homolog gamma (C. elegans)	0.99803	0.999005	1.0095256
84553	C6orf168	chromosome 6 open reading frame 168	1.01329	1.021579	1.0185151
84557	MAP1LC3A	microtubule-associated protein 1 light chain 3 alpha	0.98262	1.008032	1.0051491
84560	MT4	metallothionein 4	1.01785	1.01074	1.0026913
84561	SLC12A8	solute carrier family 12 (potassium/chloride transporters), member 8	1.00471	1.000094	1.0062899
84569	LYZL1	lysozyme-like 1	0.98811	1.042286	1.0392031
84570	COL25A1	collagen, type XXV, alpha 1	1.00416	1.01002	1.0217163
84572	GNPTG	N-acetylglucosamine-1-phosphate transferase, gamma subunit	0.9467	0.971969	1.0832011
84612	PARD6B	par-6 partitioning defective 6 homolog beta (C. elegans)	0.97406	0.914673	1.0203079
84614	ZBTB37	zinc finger and BTB domain containing 37	1.01666	0.937984	0.9414799
84616	KRTAP4-4	keratin associated protein 4-4	1.00758	0.981347	1.0120672
84617	TUBB6	tubulin, beta 6	0.98307	0.938162	1.0950688
84618	NT5C1A	5'-nucleotidase, cytosolic IA	1.00307	1.005408	0.9908837
84619	ZGPAT	zinc finger, CCCH-type with G patch domain	0.97291	1.003265	1.0550687
84620	ST6GAL2	ST6 beta-galactosamide alpha-2,6-sialyltranferase 2	0.99116	0.997481	1.0000918
84622	ZNF594	zinc finger protein 594	0.9629	1.004159	0.9874705
84623	KIRREL3	kin of IRRE like 3 (Drosophila)	1.00569	0.997753	0.9934769
84624	FNDC1	fibronectin type III domain containing 1	1.00701	1.003373	1.014186
84626	KRBA1	KRAB-A domain containing 1	0.98695	0.995589	1.0156628
84627	ZNF469	zinc finger protein 469	0.99569	1.005451	1.0060046
84628	NTNG2	netrin G2	0.99119	0.987704	1.0053286
84629	TNRC18	trinucleotide repeat containing 18	0.99717	0.953419	1.0271102
84630	TTBK1	tau tubulin kinase 1	1.00642	1.034166	1.0316788
84631	SLITRK2	SLIT and NTRK-like family, member 2	1.00844	0.969053	1.0551504
84632	AFAP1L2	actin filament associated protein 1-like 2	0.99072	0.971683	0.9913547
84634	KISS1R	KISS1 receptor	0.98714	1.006512	1.0734374
84636	GPR174	G protein-coupled receptor 174	0.92296	1.026366	0.9708669
84639	IL1F10	interleukin 1 family, member 10 (theta)	0.99681	1.004048	1.0060611
84640	USP38	ubiquitin specific peptidase 38	0.99131	0.961407	1.0207411
84641	HIATL1	hippocampus abundant transcript-like 1	0.9552	0.974967	1.0841108
84643	KIF2B	kinesin family member 2B	1.00233	1.033247	1.0077869
84645	C22orf23	chromosome 22 open reading frame 23	1.00098	0.978138	0.9994544

84647	PLA2G12B	phospholipase A2, group XIIB	1.01374	1.032816	1.0309595
84649	DGAT2	diacylglycerol O-acyltransferase 2	0.99486	1.062377	0.9087817
84650	EBPL	emopamil binding protein-like	0.96868	0.999456	1.0402954
84651	SPINK7	serine peptidase inhibitor, Kazal type 7 (putative)	0.99268	0.997978	1.0174953
84654	SPZ1	spermatogenic leucine zipper 1	1.00839	0.967118	1.0288353
84656	GLYR1	glyoxylate reductase 1 homolog (Arabidopsis)	0.9733	1.010394	0.9619311
84657	GHRLOS2	ghrelin opposite strand RNA 2 (non-protein coding)	1.00078	1.049111	0.8796878
84658	EMR3	egf-like module containing, mucin-like, hormone receptor-like 3	1.00437	0.981657	0.9465312
84659	RNASE7	ribonuclease, RNase A family, 7	1.00907	1.018921	1.0080603
84660	CCDC62	coiled-coil domain containing 62	0.97276	0.991512	0.978819
84661	DPY30	dpy-30 homolog (C. elegans)	0.95592	0.96458	1.0040347
84662	GLIS2	GLIS family zinc finger 2	0.98142	0.973789	1.0413526
84663	CYorf15B	chromosome Y open reading frame 15B	0.99781	0.994234	1.0157417
84665	MYPN	myopalladin	1.00186	0.99907	0.9953671
84666	RETNLB	resistin like beta	0.99276	1.000935	1.0301664
84667	HES7	hairy and enhancer of split 7 (Drosophila)	0.99036	0.996232	1.106582
84668	FAM126A	family with sequence similarity 126, member A	0.9945	1.023324	1.0757378
84669	USP32	ubiquitin specific peptidase 32	0.9683	1.014015	1.0417236
84671	ZNF347	zinc finger protein 347	0.96943	0.981664	0.9889512
84674	CARD6	caspase recruitment domain family, member 6	1.01354	0.986943	0.9183992
84675	TRIM55	tripartite motif containing 55	1.02202	0.993283	1.0222576
84676	TRIM63	tripartite motif containing 63	1.00537	0.999132	1.0054069
84677	DSCR8	Down syndrome critical region gene 8	1.01114	1.007878	0.9940114
84678	KDM2B	lysine (K)-specific demethylase 2B	0.98505	1.081685	0.9859355
84679	SLC9A7	solute carrier family 9 (sodium/hydrogen exchanger), member 7	0.97327	0.999183	1.0020374
84680	ACCS	1-aminocyclopropane-1-carboxylate synthase homolog (Arabidopsis)(non-functional)	0.98208	0.99376	1.0551194
84681	HINT2	histidine triad nucleotide binding protein 2	0.97071	1.011314	1.059943
84684	INSM2	insulinoma-associated 2	0.98744	1.009201	1.0260676
84687	PPP1R9B	protein phosphatase 1, regulatory (inhibitor) subunit 9B	0.94309	0.932722	1.0380798
84688	C9orf24	chromosome 9 open reading frame 24	0.98946	0.982164	1.0425532
84689	MS4A14	membrane-spanning 4-domains, subfamily A, member 14	0.98464	0.868617	1.0378862
84690	SPATA22	spermatogenesis associated 22	1.00276	1.031848	1.0108359
84691	FAM71F1	family with sequence similarity 71, member F1	1.01439	0.971042	1.0147554

84692	CCDC54	coiled-coil domain containing 54	1.01264	1.033552	1.0090917
84693	MCEE	methylmalonyl CoA epimerase	0.98675	0.962793	1.0332662
84694	GJA10	gap junction protein, alpha 10, 62kDa	1.02994	1.025711	0.9881801
84695	LOXL3	lysyl oxidase-like 3	1.01658	0.941818	1.0424763
84696	ABHD1	abhydrolase domain containing 1	1.01447	1.030375	0.9787888
84698	CAPS2	calcyphosine 2	0.98626	0.992138	1.0066815
84699	CREB3L3	cAMP responsive element binding protein 3-like 3	0.97774	0.968837	0.9876095
84700	MYO18B	myosin XVIIIB	1.00424	0.990693	0.982855
84701	COX4I2	cytochrome c oxidase subunit IV isoform 2 (lung)	0.9821	0.990016	0.9921075
84705	GTPBP3	GTP binding protein 3 (mitochondrial)	0.97477	0.967463	1.0372053
84706	GPT2	glutamic pyruvate transaminase (alanine aminotransferase) 2	1.00164	1.003753	0.9947776
84707	BEX2	brain expressed X-linked 2	0.9555	0.953992	0.9603714
84708	LNX1	ligand of numb-protein X 1	1.00874	1.008188	1.0132945
84709	C4orf49	chromosome 4 open reading frame 49	0.99656	0.978409	1.0061658
84717	HDGFRP2	hepatoma-derived growth factor-related protein 2	0.96872	1.011231	1.0688981
84720	PIGO	phosphatidylinositol glycan anchor biosynthesis, class O	0.96803	0.963871	1.055425
84722	PSRC1	proline/serine-rich coiled-coil 1	1.01345	1.005155	1.0231132
84725	PLEKHA8	pleckstrin homology domain containing, family A (phosphoinositide binding specific) member 8	0.99958	1.031657	0.993007
84726	PRRC2B	proline-rich coiled-coil 2B	0.94722	1.007202	1.0103217
84727	SPSB2	splA/ryanodine receptor domain and SOCS box containing 2	0.96627	0.997833	0.9876779
84733	CBX2	chromobox homolog 2	0.99019	0.984537	1.0191355
84734	FAM167B	family with sequence similarity 167, member B	0.95255	0.988137	0.977801
84735	CNDP1	carnosine dipeptidase 1 (metallopeptidase M20 family)	1.0028	1.023096	0.9637495
84740	AFAP1-AS1	AFAP1 antisense RNA 1 (non-protein coding)	0.98413	0.907687	1.0147806
84747	UNC119B	unc-119 homolog B (C. elegans)	0.91954	0.968941	1.1379499
84749	USP30	ubiquitin specific peptidase 30	0.94294	0.90652	0.9640002
84750	FUT10	fucosyltransferase 10 (alpha (1,3) fucosyltransferase)	1.0009	1.025912	0.9861228
84752	B3GNT9	UDP-GlcNAc:betaGal beta-1,3-N-acetylglucosaminyltransferase 9	1.00206	1.031517	1.0805227
84759	PCGF1	polycomb group ring finger 1	0.99125	0.980246	1.0384123
84765	ZNF577	zinc finger protein 577	0.96195	0.989117	0.9770906
84766	EFCAB4B	EF-hand calcium binding domain 4B	0.97769	1.025464	1.0017179
84769	MPV17L2	MPV17 mitochondrial membrane protein-like 2	0.96813	0.998799	0.9882099
84775	ZNF607	zinc finger protein 607	0.96024	1.019434	0.9719515

84779	NAA11	N(alpha)-acetyltransferase 11, NatA catalytic subunit	1.03893	1.044559	1.0323005
84787	SUV420H2	suppressor of variegation 4-20 homolog 2 (Drosophila)	0.99136	0.996899	0.9839339
84790	TUBA1C	tubulin, alpha 1c	0.97377	1.000904	1.0457835
84791	C1orf97	chromosome 1 open reading frame 97	1.00137	1.002648	1.0246261
84792	C7orf70	chromosome 7 open reading frame 70	0.98321	0.982168	1.0405642
84795	PYROXD2	pyridine nucleotide-disulphide oxidoreductase domain 2	0.9721	1.003737	0.9746215
84798	C19orf48	chromosome 19 open reading frame 48	0.9579	1.003848	0.9919809
84803	AGPAT9	1-acylglycerol-3-phosphate O-acyltransferase 9	1.01079	1.025535	0.9502301
84804	MFSD9	major facilitator superfamily domain containing 9	0.99689	0.983508	1.0325182
84807	NFKBID	nuclear factor of kappa light polypeptide gene enhancer in B-cells inhibitor, delta	0.96934	1.082805	0.9553841
84808	C1orf170	chromosome 1 open reading frame 170	0.97376	1.028194	1.0218935
84809	CROCCP2	ciliary rootlet coiled-coil, rootletin pseudogene 2	0.98462	1.024004	1.0790592
84811	BUD13	BUD13 homolog (S. cerevisiae)	0.92666	1.050151	1.1449775
84812	PLCD4	phospholipase C, delta 4	0.99863	0.982812	0.985988
84814	PPAPDC3	phosphatidic acid phosphatase type 2 domain containing 3	0.98515	1.004323	0.9810997
84816	RTN4IP1	reticulon 4 interacting protein 1	0.99685	0.967652	1.0038873
84817	TXNDC17	thioredoxin domain containing 17	0.93891	0.97554	1.0399649
84818	IL17RC	interleukin 17 receptor C	1.00831	0.982668	1.0431921
84823	LMNB2	lamin B2	0.9568	1.039284	1.0237707
84826	SFT2D3	SFT2 domain containing 3	0.96643	0.893116	0.9777334
84830	C6orf105	chromosome 6 open reading frame 105	0.97477	0.972618	0.9831441
84832	ANKRD36B	ankyrin repeat domain 36B pseudogene 1	1.04712	0.929605	0.9795162
84833	USMG5	up-regulated during skeletal muscle growth 5 homolog (mouse)	0.92975	0.956157	1.060704
84836	ABHD14B	abhydrolase domain containing 14B	0.97556	0.959444	1.0348739
84837	C14orf128	chromosome 14 open reading frame 128	0.98298	0.950306	0.9665661
84838	ZNF496	zinc finger protein 496	0.99353	0.977565	1.0136295
84839	RAX2	retina and anterior neural fold homeobox 2	0.99978	0.970621	1.0131876
84842	HPDL	4-hydroxyphenylpyruvate dioxygenase-like	0.98452	1.046081	0.9978611
84843	MGC15705	hypothetical protein MGC15705	0.97694	0.996134	0.8652741
84844	PHF5A	PHD finger protein 5A	0.96675	0.989116	1.000037
84848	MGC16121	hypothetical protein MGC16121	1.00254	1.026076	1.0171911
84850	C9orf70	chromosome 9 open reading frame 70	1.01703	1.070834	1.0935137
84851	TRIM52	tripartite motif containing 52	0.97253	1.0504	0.8570952

84856	LOC84856	hypothetical LOC84856	0.98841	0.992943	1.0183357
84858	ZNF503	zinc finger protein 503	0.96841	0.913516	0.9981802
84859	LRCH3	leucine-rich repeats and calponin homology (CH) domain containing 3	0.98607	1.017076	1.0272084
84861	KLHL22	kelch-like 22 (Drosophila)	0.9787	0.957213	0.9482942
84864	MINA	MYC induced nuclear antigen	0.98109	0.940916	1.028954
84865	CCDC142	coiled-coil domain containing 142	0.98218	1.014407	1.0013395
84866	TMEM25	transmembrane protein 25	0.99114	0.979368	0.9971996
84867	PTPN5	protein tyrosine phosphatase, non-receptor type 5 (striatum-enriched)	0.98506	0.976834	1.0099359
84868	HAVCR2	hepatitis A virus cellular receptor 2	1.00666	1.048226	0.9426951
84869	CBR4	carbonyl reductase 4	0.98952	1.029529	1.0143231
84870	RSPO3	R-spondin 3 homolog (Xenopus laevis)	0.9958	1.030811	1.048913
84871	AGBL4	ATP/GTP binding protein-like 4	1.01428	0.998783	1.0145118
84872	ZC3H10	zinc finger CCCH-type containing 10	0.96388	0.90857	0.9605673
84873	GPR128	G protein-coupled receptor 128	1.01262	0.992136	1.0453847
84874	ZNF514	zinc finger protein 514	0.97854	1.006943	1.0058178
84875	PARP10	poly (ADP-ribose) polymerase family, member 10	0.96191	0.933811	1.0903302
84876	ORAI1	ORAI calcium release-activated calcium modulator 1	0.9652	0.968841	1.01997
84878	ZBTB45	zinc finger and BTB domain containing 45	0.97277	1.024449	1.104142
84879	MFSD2A	major facilitator superfamily domain containing 2A	1.01323	1.000247	0.9728183
84881	RPUSD4	RNA pseudouridylate synthase domain containing 4	0.93522	0.978499	1.0196875
84885	ZDHHC12	zinc finger, DHHC-type containing 12	0.96366	0.958673	1.0324953
84886	C1orf198	chromosome 1 open reading frame 198	0.98733	0.91126	0.9475908
84888	SPPL2A	signal peptide peptidase-like 2A	1.00967	1.058132	0.9701047
84889	SLC7A3	solute carrier family 7 (cationic amino acid transporter, y+ system), member 3	0.99157	1.031509	1.0224465
84890	ADO	2-aminoethanethiol (cysteamine) dioxygenase	0.9405	0.925405	1.0407405
84891	ZSCAN10	zinc finger and SCAN domain containing 10	0.97369	0.979843	1.0046274
84892	C3orf39	chromosome 3 open reading frame 39	0.97572	0.959414	0.964918
84893	FBXO18	F-box protein, helicase, 18	0.96136	0.965483	1.019386
84894	LINGO1	leucine rich repeat and Ig domain containing 1	0.98172	0.975826	0.9929891
84895	FAM73B	family with sequence similarity 73, member B	0.94799	0.98913	1.019405
84896	ATAD1	ATPase family, AAA domain containing 1	0.96665	0.954466	1.0060909
84897	TBRG1	transforming growth factor beta regulator 1	0.97186	1.009374	1.1272927
84898	PLXDC2	plexin domain containing 2	1.00508	0.922119	1.0034772

84899	TMTC4	transmembrane and tetratricopeptide repeat containing 4	0.9995	0.976752	1.0426406
84900	RNFT2	ring finger protein, transmembrane 2	0.97093	0.979734	1.0033608
84901	NFATC2IP	nuclear factor of activated T-cells, cytoplasmic, calcineurin-dependent 2 interacting protein	0.97207	0.992297	1.1120376
84902	CEP89	centrosomal protein 89kDa	0.98739	1.0017	1.0132867
84904	C9orf100	chromosome 9 open reading frame 100	0.98843	1.019481	1.0323014
84905	ZNF341	zinc finger protein 341	0.97718	0.964001	1.0212968
84908	FAM136A	family with sequence similarity 136, member A	0.99871	1	1.0262274
84909	C9orf3	chromosome 9 open reading frame 3	0.99417	0.959941	1.0063848
84910	TMEM87B	transmembrane protein 87B	0.98897	0.967471	1.1223688
84911	ZNF382	zinc finger protein 382	0.89648	0.969131	1.0471748
84912	SLC35B4	solute carrier family 35, member B4	0.99873	0.934122	1.0162039
84913	ATOH8	atonal homolog 8 (Drosophila)	1.00076	1.026466	1.0423993
84914	ZNF587	zinc finger protein 587	0.98009	1.006087	1.0061873
84915	C12orf34	chromosome 12 open reading frame 34	0.98426	0.972733	1.0165894
84916	CIRH1A	cirrhosis, autosomal recessive 1A (cirhin)	0.92376	1.011795	1.0497887
84918	LRP11	low density lipoprotein receptor-related protein 11	1.005	0.99617	1.0220458
84919	PPP1R15B	protein phosphatase 1, regulatory (inhibitor) subunit 15B	0.95107	0.99297	0.9139374
84922	FIZ1	FLT3-interacting zinc finger 1	0.97173	1.038507	1.1121731
84923	FAM104A	family with sequence similarity 104, member A	0.97319	0.935407	1.1106146
84924	ZNF566	zinc finger protein 566	0.90712	1.050987	1.0240018
84925	DIRC2	disrupted in renal carcinoma 2	1.00178	1.068898	0.9749925
84926	SPRYD3	SPRY domain containing 3	0.94729	0.941308	0.9895288
84928	TMEM209	transmembrane protein 209	0.98646	0.817438	1.0432425
84929	FIBCD1	fibrinogen C domain containing 1	0.98066	1.026082	1.0178939
84930	MASTL	microtubule associated serine/threonine kinase-like	0.93345	0.910532	1.0690053
84931	LOC84931	hypothetical LOC84931	0.99942	1.004435	1.0396848
84932	RAB2B	RAB2B, member RAS oncogene family	0.94793	0.977069	1.0896646
84933	C8orf76	chromosome 8 open reading frame 76	1.01004	1.05403	1.0364839
84934	C12orf52	chromosome 12 open reading frame 52	0.9479	0.974645	1.0377451
84935	C13orf33	chromosome 13 open reading frame 33	0.98887	0.98629	1.011789
84936	ZFYVE19	zinc finger, FYVE domain containing 19	0.92175	0.935005	1.0197413
84937	ZNRF1	zinc and ring finger 1	0.99302	1.006295	1.021559
84938	ATG4C	ATG4 autophagy related 4 homolog C (S. cerevisiae)	0.98539	0.977468	0.9681302

84939	MUM1	melanoma associated antigen (mutated) 1	0.97114	0.942951	1.0302022
84940	CORO6	coronin 6	0.98535	0.995766	1.0857457
84941	HSH2D	hematopoietic SH2 domain containing	0.97611	0.974389	1.0393069
84942	WDR73	WD repeat domain 73	0.94401	0.975493	1.0775097
84944	MAEL	maelstrom homolog (Drosophila)	0.98537	0.986668	1.0126465
84945	ABHD13	abhydrolase domain containing 13	0.94545	0.967557	1.1081921
84946	LTV1	LTV1 homolog (S. cerevisiae)	0.99132	0.957848	1.0257052
84947	SERAC1	serine active site containing 1	0.97954	0.950492	0.9595666
84948	TIGD5	tigger transposable element derived 5	0.98809	1.032848	1.0902914
84950	PRPF38A	PRP38 pre-mRNA processing factor 38 (yeast) domain containing A	0.98142	0.940129	1.0072595
84951	TNS4	tensin 4	1.00097	0.989425	0.9846692
84952	CGNL1	cingulin-like 1	0.98891	1.001454	0.9971017
84953	MICALCL	MICAL C-terminal like	0.96686	0.908558	1.0058291
84954	MPND	MPN domain containing	0.97493	0.985731	1.0598739
84955	NUDCD1	NudC domain containing 1	0.93874	1.021346	1.0260481
84957	RELT	RELT tumor necrosis factor receptor	1.00183	1.021918	1.0025633
84958	SYTL1	synaptotagmin-like 1	1.01801	0.953031	0.9819472
84959	UBASH3B	ubiquitin associated and SH3 domain containing B	0.96982	0.990602	0.9817647
84960	KIAA1984	KIAA1984	0.99346	1.016543	1.0375046
84961	FBXL20	F-box and leucine-rich repeat protein 20	0.97719	0.956962	0.9393875
84962	JUB	jub, ajuba homolog (Xenopus laevis)	0.98799	0.9713	1.015728
84964	ALKBH6	alkB, alkylation repair homolog 6 (E. coli)	0.94809	1.058497	1.042274
84966	IGSF21	immunoglobin superfamily, member 21	0.99897	1.0165	1.0149296
84967	LSM10	LSM10, U7 small nuclear RNA associated	0.97782	0.913656	1.011744
84969	TOX2	TOX high mobility group box family member 2	0.98073	1.00914	1.0120436
84970	C1orf94	chromosome 1 open reading frame 94	1.00657	0.994184	1.0028213
84971	ATG4D	ATG4 autophagy related 4 homolog D (S. cerevisiae)	0.96181	1.030471	1.0095466
84975	MFSD5	major facilitator superfamily domain containing 5	0.97123	0.921911	1.2094528
84976	DISP1	dispatched homolog 1 (Drosophila)	0.99596	0.986412	0.9759574
84978	FRMD5	FERM domain containing 5	1.00102	0.993254	0.9801421
84981	C17orf91	chromosome 17 open reading frame 91	1.00447	1.04733	0.9916728
84984	CEP19	centrosomal protein 19kDa	0.98347	1.012402	1.0621354
84985	FAM83A	family with sequence similarity 83, member A	1.00533	0.987629	1.0289154

84986	ARHGAP19	Rho GTPase activating protein 19	0.94276	0.94954	1.1000114
84987	C12orf62	chromosome 12 open reading frame 62	0.92769	0.887338	0.9536206
84988	PPP1R16A	protein phosphatase 1, regulatory (inhibitor) subunit 16A	0.99261	1.002147	1.1169624
84991	RBM17	RNA binding motif protein 17	0.92484	0.996809	1.0131037
84992	PIGY	phosphatidylinositol glycan anchor biosynthesis, class Y	0.98694	0.946521	0.9908693
84993	UBL7	ubiquitin-like 7 (bone marrow stromal cell-derived)	0.93562	0.891011	1.0929199
84996	C21orf119	chromosome 21 open reading frame 119	0.95096	0.96992	1.0127418
85004	RERG	RAS-like, estrogen-regulated, growth inhibitor	0.9978	0.963575	1.0201558
85007	AGXT2L2	alanine-glyoxylate aminotransferase 2-like 2	0.99351	0.89505	1.0590261
85012	TCEAL3	transcription elongation factor A (SII)-like 3	0.98878	1.005564	1.1057685
85013	TMEM128	transmembrane protein 128	0.95771	0.944404	0.9451841
85014	TMEM141	transmembrane protein 141	0.94822	0.891427	1.0451036
85015	USP45	ubiquitin specific peptidase 45	0.97617	1.022135	1.0052783
85016	C11orf70	chromosome 11 open reading frame 70	0.98806	1.026891	1.0190643
85019	C18orf45	chromosome 18 open reading frame 45	0.98351	0.967104	1.0163515
85021	REPS1	RALBP1 associated Eps domain containing 1	0.98704	0.934552	1.0040959
85025	TMEM60	transmembrane protein 60	0.96778	0.916162	1.0182031
85026	C9orf37	chromosome 9 open reading frame 37	0.971	0.990427	0.9926934
85027	C5orf62	chromosome 5 open reading frame 62	0.99993	0.933217	0.9796859
85235	HIST1H2AH	histone cluster 1, H2ah	0.98279	0.865999	1.0099977
85236	HIST1H2BK	histone cluster 1, H2bk	0.99693	0.944298	0.9922253
85285	KRTAP4-1	keratin associated protein 4-1	1.02037	0.981484	0.9908487
85290	KRTAP4-3	keratin associated protein 4-3	0.99201	1.009714	0.9723372
85291	KRTAP4-2	keratin associated protein 4-2	0.99638	0.94793	1.0632182
85293	KRTAP3-3	keratin associated protein 3-3	1.01989	0.987541	1.0547923
85300	ATCAY	ataxia, cerebellar, Cayman type	1.00129	1.020911	1.0331629
85301	COL27A1	collagen, type XXVII, alpha 1	0.99823	1.012134	1.0380623
85302	FBF1	Fas (TNFRSF6) binding factor 1	0.9577	0.995781	0.9740847
85303	HIST1H2AP	histone cluster 1, H2a, pseudogene 2	0.98941	0.896146	1.0823321
85313	PPIL4	peptidylprolyl isomerase (cyclophilin)-like 4	0.98996	0.884017	0.9322991
85315	PAQR8	progestin and adipoQ receptor family member VIII	0.96675	0.998948	1.0044625
85320	ABCC11	ATP-binding cassette, sub-family C (CFTR/MRP), member 11	0.99636	0.99022	0.9954522
85329	LGALS12	lectin, galactoside-binding, soluble, 12	0.91128	0.943182	0.974756

85340	KRT124P	keratin 124 pseudogene	0.99606	1.002936	0.9289206
85344	KRT123P	keratin 123 pseudogene	1.00466	0.997347	1.029048
85348	KRT122P	keratin 122 pseudogene	0.98695	1.009046	0.9995517
85349	KRT121P	keratin 121 pseudogene	1.00729	1.023192	1.0193969
85352	KIAA1644	KIAA1644	0.97799	1.01862	1.005114
85358	SHANK3	SH3 and multiple ankyrin repeat domains 3	0.98976	1.026688	1.0511281
85359	DGCR6L	DiGeorge syndrome critical region gene 6-like	0.9258	0.989219	1.0269318
85360	SYDE1	synapse defective 1, Rho GTPase, homolog 1 (C. elegans)	0.99805	0.985819	1.024334
85363	TRIM5	tripartite motif containing 5	0.95492	1.013764	1.0340457
85364	ZCCHC3	zinc finger, CCHC domain containing 3	0.94868	0.935766	1.0896514
85365	ALG2	asparagine-linked glycosylation 2, alpha-1,3-mannosyltransferase homolog (S. cerevisiae)	0.91767	1.076501	1.0357551
85366	MYLK2	myosin light chain kinase 2	0.98239	0.984147	0.9992158
85369	FAM40A	family with sequence similarity 40, member A	0.9701	0.899355	1.0016698
85371	KIAA1656	KIAA1656 protein	0.99065	0.94918	1.022686
85377	MICALL1	MICAL-like 1	0.95442	1.005397	1.0018373
85378	TUBGCP6	tubulin, gamma complex associated protein 6	0.9713	0.985516	0.9252314
85379	KIAA1671	KIAA1671	1.00529	1.005726	1.0090875
85395	C21orf70	chromosome 21 open reading frame 70	0.99832	1.003068	1.0599824
85397	RGS8	regulator of G-protein signaling 8	1.02832	1.002359	1.042053
85403	EAF1	ELL associated factor 1	0.99261	0.973438	0.9850071
85406	DNAJC14	DnaJ (Hsp40) homolog, subfamily C, member 14	0.91638	0.902108	1.025641
85407	NKD1	naked cuticle homolog 1 (Drosophila)	0.98615	0.999716	0.9975396
85409	NKD2	naked cuticle homolog 2 (Drosophila)	1.00208	1.009564	0.9833311
85413	SLC22A16	solute carrier family 22 (organic cation/carnitine transporter), member 16	0.98824	1.018055	1.007116
85414	SLC45A3	solute carrier family 45, member 3	0.98749	0.981818	1.0350434
85415	RHPN2	rhophilin, Rho GTPase binding protein 2	0.9962	1.001823	1.0158036
85416	ZIC5	Zic family member 5	0.99119	1.019795	1.0552335
85417	CCNB3	cyclin B3	0.99964	1.02412	0.9961207
85437	ZCRB1	zinc finger CCHC-type and RNA binding motif 1	0.93369	0.926453	1.0081051
85438	CABS1	calcium-binding protein, spermatid-specific 1	1.0226	0.98669	1.0457091
85439	STON2	stonin 2	1.00378	0.989844	0.9930398
85440	DOCK7	dedicator of cytokinesis 7	0.97864	0.936954	0.9995993
85441	PRIC285	peroxisomal proliferator-activated receptor A interacting complex 285	0.93563	0.898541	0.9889604

85442	KNDC1	kinase non-catalytic C-lobe domain (KIND) containing 1	0.99556	1.013939	1.0134106
85443	DCLK3	doublecortin-like kinase 3	1.02506	1.032306	1.0221738
85444	LRRCC1	leucine rich repeat and coiled-coil domain containing 1	0.97516	0.934983	0.9872502
85445	CNTNAP4	contactin associated protein-like 4	0.99859	0.984525	1.0063512
85446	ZFHX2	zinc finger homeobox 2	1.00382	0.997074	1.0156437
85449	KIAA1755	KIAA1755	0.99335	0.99721	0.9941163
85450	ITPRIP	inositol 1,4,5-trisphosphate receptor interacting protein	0.98927	1.040464	0.9345699
85451	UNK	unkempt homolog (Drosophila)	0.97423	0.947872	0.9984092
85452	KIAA1751	KIAA1751	0.99763	0.994395	1.0117049
85453	TSPYL5	TSPY-like 5	0.95332	0.956065	0.9984864
85455	DISP2	dispatched homolog 2 (Drosophila)	0.99126	0.984693	0.9991627
85456	TNKS1BP1	tankyrase 1 binding protein 1, 182kDa	0.99645	0.986867	0.9849242
85457	KIAA1737	KIAA1737	0.94294	0.955847	1.117686
85458	DIXDC1	DIX domain containing 1	0.97934	1.007805	1.015866
85460	ZNF518B	zinc finger protein 518B	0.99588	0.956001	0.9362755
85461	TANC1	tetratricopeptide repeat, ankyrin repeat and coiled-coil containing 1	1.00469	1.006161	1.009847
85462	FHDC1	FH2 domain containing 1	1.00178	0.98415	0.999954
85463	ZC3H12C	zinc finger CCCH-type containing 12C	0.98068	1.033486	0.996498
85464	SSH2	slingshot homolog 2 (Drosophila)	1.00853	0.9939	0.9226058
85465	EPT1	ethanolaminephosphotransferase 1 (CDP-ethanolamine-specific)	0.99846	1.00222	1.0317841
85474	LBX2	ladybird homeobox 2	0.98054	1.000162	1.0876432
85476	GFM1	G elongation factor, mitochondrial 1	0.99882	0.985698	1.0582973
85477	SCIN	scinderin	1.00144	0.981847	1.0237863
85478	CCDC65	coiled-coil domain containing 65	0.90376	1.074773	0.9675203
85479	DNAJC5B	DnaJ (Hsp40) homolog, subfamily C, member 5 beta	1.00733	1.004733	1.0132435
85480	TSLP	thymic stromal lymphopoietin	0.98487	1.012877	0.9993226
85481	PSKH2	protein serine kinase H2	1.03215	0.974483	1.0010613
85486	RPS19P1	ribosomal protein S19 pseudogene 1	0.97419	0.929395	0.9951391
85508	SCRT2	scratch homolog 2, zinc finger protein (Drosophila)	0.98587	1.007501	1.0197217
85509	MBD3L1	methyl-CpG binding domain protein 3-like 1	0.99052	0.959235	1.0663218
85569	GALP	galanin-like peptide	0.99096	0.966454	1.0544803
85824	RPL12P12	ribosomal protein L12 pseudogene 12	0.90779	0.97487	1.1090332
85865	GTPBP10	GTP-binding protein 10 (putative)	0.96105	0.942153	0.9005681

86123	YWHAZP5	tyrosine 3-monooxygenase/tryptophan 5-monooxygenase activation protein, zeta pseudogene 5	1.01263	0.959657	1.0054991
87171	ARPC3P1	actin related protein 2/3 complex, subunit 3 pseudogene 1	0.99329	0.936512	1.0708146
87178	PNPT1	polyribonucleotide nucleotidyltransferase 1	0.96741	0.864638	1.0893764
87688	RPL7AP50	ribosomal protein L7a pseudogene 50	0.87202	1.035804	1.0061761
87769	A2LD1	AIG2-like domain 1	0.9814	0.975586	1.019745
88455	ANKRD13A	ankyrin repeat domain 13A	0.96253	0.978578	0.9840697
88745	RRP36	ribosomal RNA processing 36 homolog (S. cerevisiae)	0.95046	1.034254	1.1103465
89122	TRIM4	tripartite motif containing 4	0.98729	0.937126	1.0053615
89765	RSPH1	radial spoke head 1 homolog (Chlamydomonas)	0.9885	0.995241	0.9886435
89766	UMODL1	uromodulin-like 1	1.00134	0.989747	1.0132576
89777	SERPINB12	serpin peptidase inhibitor, clade B (ovalbumin), member 12	1.0006	1.014483	0.9668778
89778	SERPINB11	serpin peptidase inhibitor, clade B (ovalbumin), member 11 (gene/pseudogene)	1.00164	0.965256	1.0145414
89780	WNT3A	wingless-type MMTV integration site family, member 3A	0.99224	1.015174	1.017681
89781	HPS4	Hermansky-Pudlak syndrome 4	0.9726	0.952799	0.9753262
89782	LMLN	leishmanolysin-like (metallopeptidase M8 family)	0.98442	0.97274	0.9869693
89790	SIGLEC10	sialic acid binding Ig-like lectin 10	1.01342	0.988404	0.9645773
89792	GAL3ST3	galactose-3-O-sulfotransferase 3	0.99246	0.990269	1.0208906
89795	NAV3	neuron navigator 3	1.00657	0.993862	1.0001425
89796	NAV1	neuron navigator 1	1.0164	0.990302	1.0220197
89797	NAV2	neuron navigator 2	0.99834	1.00037	1.0104973
89801	PPP1R3F	protein phosphatase 1, regulatory (inhibitor) subunit 3F	0.94981	1.015781	0.9952344
89822	KCNK17	potassium channel, subfamily K, member 17	1.01944	0.970424	1.0427807
89839	ARHGAP11	Rho GTPase activating protein 11B	0.99359	1.08291	1.0354743
89844	LOC89844	mitochondrial RNA polymerase pseudogene	1.02758	1.125762	0.9569638
89845	ABCC10	ATP-binding cassette, sub-family C (CFTR/MRP), member 10	0.99047	0.963777	1.0351886
89846	FGD3	FYVE, RhoGEF and PH domain containing 3	0.94694	1.032256	0.9683052
89848	FCHSD1	FCH and double SH3 domains 1	0.95837	1.012433	0.9562776
89849	ATG16L2	ATG16 autophagy related 16-like 2 (S. cerevisiae)	0.98975	1.070311	0.9199875
89853	FAM125B	family with sequence similarity 125, member B	0.98283	1.040585	0.968108
89857	KLHL6	kelch-like 6 (Drosophila)	1.00618	0.974119	0.9621489
89858	SIGLEC12	sialic acid binding Ig-like lectin 12	0.99185	1.010807	1.0008788
89866	SEC16B	SEC16 homolog B (S. cerevisiae)	1.01586	1.022325	1.0113385
89869	PLCZ1	phospholipase C, zeta 1	1.00252	1.024275	1.0098188

89870	TRIM15	tripartite motif containing 15	1.01271	1.015105	1.0252263
89872	AQP10	aquaporin 10	0.98781	0.999417	0.9812887
89874	SLC25A21	solute carrier family 25 (mitochondrial oxodicarboxylate carrier), member 21	0.99689	1.00782	0.9978398
89876	C3orf15	chromosome 3 open reading frame 15	1.00415	1.003326	1.0360206
89882	TPD52L3	tumor protein D52-like 3	0.99133	0.993276	1.013641
89883	OR6W1P	olfactory receptor, family 6, subfamily W, member 1 pseudogene	1.00928	0.977591	1.0316974
89884	LHX4	LIM homeobox 4	1.01047	1.002332	1.0077002
89885	FATE1	fetal and adult testis expressed 1	1.01921	1.003883	0.988076
89887	ZNF628	zinc finger protein 628	0.98936	1.011661	1.004137
89890	KBTBD6	kelch repeat and BTB (POZ) domain containing 6	0.94289	0.992164	1.0322313
89891	WDR34	WD repeat domain 34	0.99358	0.993203	1.0527987
89894	TMEM116	transmembrane protein 116	0.92753	0.961788	1.0037334
89910	UBE3B	ubiquitin protein ligase E3B	0.97201	0.911052	1.0455277
89919	C14orf56	chromosome 14 open reading frame 56	1.00205	0.998883	0.9965654
89927	C16orf45	chromosome 16 open reading frame 45	1.00608	0.990507	1.0034526
89932	PAPLN	papilin, proteoglycan-like sulfated glycoprotein	1.00154	1.013499	1.0172642
89941	RHOT2	ras homolog gene family, member T2	0.98679	1.032318	1.0763552
89944	GLB1L2	galactosidase, beta 1-like 2	0.99038	1.007594	1.0304276
89953	KLC4	kinesin light chain 4	0.98311	0.981711	0.9826353
89958	C9orf140	chromosome 9 open reading frame 140	1.00465	1.027079	1.0528092
89970	RSPRY1	ring finger and SPRY domain containing 1	0.93063	1.060643	1.0352359
89978	ATPBD4	ATP binding domain 4	0.96556	0.946122	1.0133491
90007	MIDN	midnolin	0.96776	0.901618	0.9517118
90011	KIR3DX1	killer cell immunoglobulin-like receptor, three domains, X1	0.98734	1.024459	1.0286678
90019	SYT8	synaptotagmin VIII	1.00016	1.030455	1.0327914
90024	FLJ20021	hypothetical LOC90024	0.99868	0.947955	1.0348672
90025	UBE2CBP	ubiquitin-conjugating enzyme E2C binding protein	0.97995	0.981622	1.0246713
90050	FAM181A	family with sequence similarity 181, member A	0.98489	1.021722	1.0017515
90060	CCDC120	coiled-coil domain containing 120	0.98534	0.972198	1.0222729
90070	LACRT	lacritin	0.99275	0.995199	0.9830045
90075	ZNF30	zinc finger protein 30	0.93066	0.980831	1.0429473
90102	PHLDB2	pleckstrin homology-like domain, family B, member 2	0.98938	1.015713	0.9853329
90113	VWA5B2	von Willebrand factor A domain containing 5B2	1.01099	1.016923	1.0438797

90120	C9orf69	chromosome 9 open reading frame 69	0.94067	0.957017	1.0589568
90121	TSR2	TSR2, 20S rRNA accumulation, homolog (S. cerevisiae)	0.90917	0.889168	0.9632298
90133	KRT8P12	keratin 8 pseudogene 12	0.98675	0.990302	1.024022
90134	KCNH7	potassium voltage-gated channel, subfamily H (eag-related), member 7	1.01124	0.990687	1.0011878
90135	BTBD6	BTB (POZ) domain containing 6	1.00185	0.971143	0.9790683
90139	TSPAN18	tetraspanin 18	0.97636	0.969352	0.9804835
90141	EFCAB11	EF-hand calcium binding domain 11	0.98924	0.979707	1.023291
90161	HS6ST2	heparan sulfate 6-O-sulfotransferase 2	1.00416	0.996594	1.0209679
90167	FRMD7	FERM domain containing 7	0.99215	1.003689	0.9956633
90187	EMILIN3	elastin microfibril interfacer 3	0.97997	1.020464	1.0345954
90193	RPL7P6	ribosomal protein L7 pseudogene 6	1.04786	0.99344	1.0279587
90199	WFDC8	WAP four-disulfide core domain 8	0.99549	1.043523	0.9670778
90203	SNX21	sorting nexin family member 21	0.99172	0.984874	0.9956922
90204	ZSWIM1	zinc finger, SWIM-type containing 1	0.89968	0.919617	1.0308624
90226	UCN2	urocortin 2	0.99329	0.974448	1.0328577
90231	KIAA2013	KIAA2013	0.98695	0.955017	1.0424532
90233	ZNF551	zinc finger protein 551	0.91834	0.973471	1.0729526
90249	UNC5A	unc-5 homolog A (C. elegans)	0.99902	1.001658	1.014091
90268	FAM105B	family with sequence similarity 105, member B	0.97322	1.01384	1.064282
90271	NCRNA002	non-protein coding RNA 263	0.97418	0.995585	1.0109711
90273	CEACAM21	carcinoembryonic antigen-related cell adhesion molecule 21	0.93536	0.998289	0.9458274
90288	C3orf25	chromosome 3 open reading frame 25	0.98857	1.026012	0.9837116
90293	KLHL13	kelch-like 13 (Drosophila)	0.99482	0.968014	0.974151
90313	TP53I13	tumor protein p53 inducible protein 13	0.98842	1.000081	1.0094369
90317	ZNF616	zinc finger protein 616	0.93539	1.00653	0.9799179
90321	ZNF766	zinc finger protein 766	0.94284	0.932222	0.9454586
90324	CCDC97	coiled-coil domain containing 97	0.94809	1.033877	0.9642107
90326	THAP3	THAP domain containing, apoptosis associated protein 3	1.01514	1.033333	0.9934833
90332	EXOC3L2	exocyst complex component 3-like 2	0.96288	0.955501	1.0434144
90333	ZNF468	zinc finger protein 468	0.94212	1.00438	1.0404664
90338	ZNF160	zinc finger protein 160	0.94696	0.917973	1.03216
90342	FER1L5	fer-1-like 5 (C. elegans)	1.01243	0.978405	0.9817574
90352	MAGOH3P	mago-nashi homolog 2, proliferation-associated (Drosophila), pseudogene	1.01445	1.0236	1.0740108

90353	CTU1	cytosolic thiouridylase subunit 1 homolog (S. pombe)	0.97483	1.014204	1.1318585
90355	C5orf30	chromosome 5 open reading frame 30	0.97392	0.938253	1.0522509
90362	FAM110B	family with sequence similarity 110, member B	1.01057	0.98351	1.0076623
90378	SAMD1	sterile alpha motif domain containing 1	0.992	0.980182	1.0923745
90379	DCAF15	DDB1 and CUL4 associated factor 15	0.93909	0.960822	1.0754344
90381	C15orf42	chromosome 15 open reading frame 42	1.00259	0.970013	1.0163486
90390	MED30	mediator complex subunit 30	1.00307	0.887676	0.9616792
90407	TMEM41A	transmembrane protein 41A	0.97528	0.967797	1.0045785
90410	IFT20	intraflagellar transport 20 homolog (Chlamydomonas)	0.95598	0.932727	1.0306798
90411	MCFD2	multiple coagulation factor deficiency 2	0.99841	1.044406	1.1014283
90416	C15orf57	chromosome 15 open reading frame 57	0.9682	1.001538	1.0811423
90417	C15orf23	chromosome 15 open reading frame 23	0.93943	0.989821	1.0439168
90423	ATP6V1E2	ATPase, H+ transporting, lysosomal 31kDa, V1 subunit E2	0.98184	0.997039	1.0296938
90427	BMF	Bcl2 modifying factor	0.94774	1.01658	0.9652553
90441	ZNF622	zinc finger protein 622	0.96144	0.931574	1.0483053
90459	ERI1	exoribonuclease 1	0.95579	0.933353	1.0871926
90462	ZNF605	zinc finger protein 605	0.98376	0.991519	1.041776
90480	GADD45GI	growth arrest and DNA-damage-inducible, gamma interacting protein 1	0.95537	0.926186	1.1191508
90485	ZNF835	zinc finger protein 835	0.96912	0.975845	1.012121
90488	C12orf23	chromosome 12 open reading frame 23	0.955	1.063528	1.0125359
90499	LOC90499	hypothetical protein LOC90499	0.94675	0.905841	1.0313722
90506	LRRC46	leucine rich repeat containing 46	1.00806	0.964142	1.0127439
90507	SCRN2	secernin 2	0.94685	0.931088	1.023921
90522	YIF1B	Yip1 interacting factor homolog B (S. cerevisiae)	0.96281	0.993208	1.1075517
90523	MLIP	muscular LMNA-interacting protein	1.01331	0.987404	1.0203334
90525	SHF	Src homology 2 domain containing F	0.98959	0.990689	1.0009752
90527	DUOXA1	dual oxidase maturation factor 1	1.01386	1.002593	0.9934315
90529	C1orf201	chromosome 1 open reading frame 201	0.97592	1.010238	1.0109231
90550	CCDC109A	coiled-coil domain containing 109A	0.97157	1.044953	1.0367508
90557	CCDC74A	coiled-coil domain containing 74A	0.99495	1.032758	1.017833
90576	ZNF799	zinc finger protein 799	0.919	0.854328	1.0571539
90580	C19orf52	chromosome 19 open reading frame 52	0.93543	0.953404	1.1346642
90586	AOC4	AOC3 pseudogene	1.01862	1.030184	0.9604047

90589	ZNF625	zinc finger protein 625	0.99292	0.947389	1.016831
90592	ZNF700	zinc finger protein 700	0.95898	0.915581	1.0098311
90594	ZNF439	zinc finger protein 439	0.98144	0.94184	0.9625524
90624	LYRM7	Lyrm7 homolog (mouse)	0.96767	1.007737	0.99234
90627	STARD13	StAR-related lipid transfer (START) domain containing 13	1.00246	0.999437	1.0003814
90632	C6orf176	chromosome 6 open reading frame 176	0.99843	1.017055	0.9953702
90634	N4BP2L1	NEDD4 binding protein 2-like 1	0.95145	0.951763	0.9699009
90637	ZFAND2A	zinc finger, AN1-type domain 2A	0.97265	0.89273	0.9977281
90639	COX19	COX19 cytochrome c oxidase assembly homolog (S. cerevisiae)	1.02215	0.938006	0.9844803
90649	ZNF486	zinc finger protein 486	0.96756	0.924513	1.1287586
90665	TBL1Y	transducin (beta)-like 1, Y-linked	0.99515	0.981569	0.9722666
90668	LRRC16B	leucine rich repeat containing 16B	1.00609	0.978017	1.0128637
90673	PPP1R3E	protein phosphatase 1, regulatory (inhibitor) subunit 3E	0.94472	0.97728	0.9529734
90678	LRSAM1	leucine rich repeat and sterile alpha motif containing 1	0.97041	1.021266	1.0079324
90693	CCDC126	coiled-coil domain containing 126	1.00319	1.016007	1.0967692
90701	SEC11C	SEC11 homolog C (S. cerevisiae)	0.99148	1.014559	1.0360308
90736	FAM104B	family with sequence similarity 104, member B	0.96473	0.961476	1.0303638
90737	PAGE5	P antigen family, member 5 (prostate associated)	0.99866	1.075251	1.0030081
90768	MGC45800	hypothetical LOC90768	0.99554	1.020124	0.9827184
90780	PYGO2	pygopus homolog 2 (Drosophila)	0.99127	1.078001	1.0039569
90799	CEP95	centrosomal protein 95kDa	0.95387	1.016007	0.9682075
90806	ANGEL2	angel homolog 2 (Drosophila)	0.98178	0.962052	1.0296624
90809	TMEM55B	transmembrane protein 55B	0.9694	1.013611	0.9335924
90826	PRMT10	protein arginine methyltransferase 10 (putative)	0.99302	0.895691	0.9305358
90827	ZNF479	zinc finger protein 479	1.01626	0.961974	1.042884
90835	C16orf93	chromosome 16 open reading frame 93	1.00899	0.98732	1.0233573
90843	TCEAL8	transcription elongation factor A (SII)-like 8	0.92547	0.91411	1.0214011
90850	ZNF598	zinc finger protein 598	0.98072	1.047309	1.0676267
90853	SPOCD1	SPOC domain containing 1	1.00455	0.958033	0.9991513
90859	NT5C3P1	5'-nucleotidase, cytosolic III pseudogene 1	1.01806	1.055391	0.9892702
90861	HN1L	hematological and neurological expressed 1-like	0.95527	1.02404	1.0168631
90864	SPSB3	splA/ryanodine receptor domain and SOCS box containing 3	0.96684	0.98357	1.1368514
90865	IL33	interleukin 33	0.99168	0.996086	0.9949023

90871	C9orf123	chromosome 9 open reading frame 123	0.86004	0.867499	1.0744596
90874	ZNF697	zinc finger protein 697	1.00522	0.998267	0.9759123
90933	TRIM41	tripartite motif containing 41	0.99272	1.009148	0.9811306
90952	ESAM	endothelial cell adhesion molecule	0.94979	0.976858	1.060121
90956	ADCK2	aarF domain containing kinase 2	0.98679	0.95228	0.9691073
90957	DHX57	DEAH (Asp-Glu-Ala-Asp/His) box polypeptide 57	0.97099	0.958381	1.0653395
90987	ZNF251	zinc finger protein 251	0.96206	0.983593	1.0113985
90990	KIFC2	kinesin family member C2	1.02346	1.032549	1.0178423
90993	CREB3L1	cAMP responsive element binding protein 3-like 1	0.987	0.998599	1.0236716
91010	FMNL3	formin-like 3	0.94935	1.005552	0.9936638
91012	LASS5	LAG1 homolog, ceramide synthase 5	0.96526	0.979206	1.0837608
91039	DPP9	dipeptidyl-peptidase 9	0.94667	0.957676	0.9647001
91050	CCDC149	coiled-coil domain containing 149	1.00547	0.994782	0.9955507
91056	DKFZp761E	DKFZp761E198 protein	0.9677	0.942541	0.9207625
91057	CCDC34	coiled-coil domain containing 34	0.99134	1.015842	1.0181387
91074	ANKRD30A	ankyrin repeat domain 30A	0.9819	0.985478	1.019649
91107	TRIM47	tripartite motif containing 47	0.98894	1.060617	1.0369878
91120	ZNF682	zinc finger protein 682	0.96875	0.954859	0.9520259
91133	L3MBTL4	l(3)mbt-like 4 (Drosophila)	0.98229	0.987265	1.013222
91137	SLC25A46	solute carrier family 25, member 46	0.99127	0.905028	1.0424415
91147	TMEM67	transmembrane protein 67	0.97817	1.001759	1.0649585
91151	TIGD7	tigger transposable element derived 7	0.95811	0.992404	0.9664297
91156	IGFN1	immunoglobulin-like and fibronectin type III domain containing 1	1.02115	1.005683	0.9911765
91179	SCARF2	scavenger receptor class F, member 2	0.99692	0.991267	1.0685061
91181	NUP210L	nucleoporin 210kDa-like	1.00647	0.988921	0.9935894
91252	SLC39A13	solute carrier family 39 (zinc transporter), member 13	0.96057	0.950692	0.9980691
91272	BOD1	biorientation of chromosomes in cell division 1	0.96343	0.842936	1.0442698
91283	C9orf30	chromosome 9 open reading frame 30	0.98586	0.988407	1.0189455
91289	LMF2	lipase maturation factor 2	0.97208	0.9954	1.0003394
91298	C12orf29	chromosome 12 open reading frame 29	0.94994	0.984339	1.0207479
91300	C19orf22	chromosome 19 open reading frame 22	0.94517	0.92303	0.940108
91304	C19orf6	chromosome 19 open reading frame 6	0.97085	1.034203	1.0578356
91316	LOC91316	glucuronidase, beta/immunoglobulin lambda-like polypeptide 1 pseudogene	0.99234	1.066809	0.9743454

91319	DERL3	Der1-like domain family, member 3	0.9822	1.021438	1.0695047
91351	DDX60L	DEAD (Asp-Glu-Ala-Asp) box polypeptide 60-like	1.01757	0.921612	0.9547788
91355	LRP5L	low density lipoprotein receptor-related protein 5-like	1.00257	1.010146	0.9745592
91368	CDKN2AIPN	CDKN2A interacting protein N-terminal like	1.00162	0.977633	1.0212308
91369	ANKRD40	ankyrin repeat domain 40	0.91074	0.997559	1.0342731
91373	UAP1L1	UDP-N-acteylglucosamine pyrophosphorylase 1-like 1	0.97106	0.961925	1.0546251
91392	ZNF502	zinc finger protein 502	0.9643	0.954545	1.0243757
91404	SESTD1	SEC14 and spectrin domains 1	1.00034	1.009275	1.009424
91408	BTF3L4	basic transcription factor 3-like 4	1.03246	1.014522	1.044999
91409	CCDC74B	coiled-coil domain containing 74B	1.01074	0.948292	1.0785485
91419	XRCC6BP1	XRCC6 binding protein 1	0.97528	0.974686	1.0136986
91431	[No Symbo	[No Name]	1.01251	0.989015	1.0118805
91433	RCCD1	RCC1 domain containing 1	0.9976	0.98221	1.0298501
91442	C19orf40	chromosome 19 open reading frame 40	0.95192	0.957812	1.0471797
91445	RNF185	ring finger protein 185	0.97234	0.96313	1.0527801
91452	ACBD5	acyl-CoA binding domain containing 5	0.94072	1.066976	1.0483736
91461	PKDCC	protein kinase domain containing, cytoplasmic homolog (mouse)	1.00896	0.985846	0.9904044
91464	ISX	intestine-specific homeobox	1.00468	0.976405	0.99843
91522	COL23A1	collagen, type XXIII, alpha 1	0.99627	1.010783	1.0215379
91523	FAM113B	family with sequence similarity 113, member B	0.95273	1.026717	0.8562224
91526	ANKRD44	ankyrin repeat domain 44	0.97355	0.926926	0.8545863
91543	RSAD2	radical S-adenosyl methionine domain containing 2	0.96281	0.961129	1.0675046
91544	UBXN11	UBX domain protein 11	1.00476	0.943728	1.0184217
91561	RPS2P40	ribosomal protein S2 pseudogene 40	0.92907	1.057417	1.0883292
91574	C12orf65	chromosome 12 open reading frame 65	0.9368	0.997241	0.9486821
91582	RPS19BP1	ribosomal protein S19 binding protein 1	0.91262	1.001551	1.090524
91584	PLXNA4	plexin A4	1.01098	0.996842	1.0151257
91603	ZNF830	zinc finger protein 830	0.86071	0.923833	0.9843456
91607	SLFN11	schlafen family member 11	0.97851	1.258507	1.0328938
91608	RASL10B	RAS-like, family 10, member B	0.99331	1.003027	1.071865
91612	CHURC1	churchill domain containing 1	0.91806	0.875988	0.9622951
91614	DEPDC7	DEP domain containing 7	0.97567	0.987876	0.9915864
91624	NEXN	nexilin (F actin binding protein)	0.92629	0.895205	0.9678659

91646	TDRD12	tudor domain containing 12	0.99305	1.001526	0.999335
91647	ATPAF2	ATP synthase mitochondrial F1 complex assembly factor 2	0.96035	0.985417	1.0822298
91653	BOC	Boc homolog (mouse)	1.00813	1.007369	1.0175143
91661	ZNF765	zinc finger protein 765	0.87244	1.010807	1.0854584
91663	MYADM	myeloid-associated differentiation marker	0.95849	0.987732	1.0410681
91664	ZNF845	zinc finger protein 845	0.90355	1.012206	1.0753964
91683	SYT12	synaptotagmin XII	1.0006	1.00035	1.0132964
91687	CENPL	centromere protein L	0.99567	0.960166	1.0556633
91689	C22orf32	chromosome 22 open reading frame 32	0.93474	0.857653	1.04719
91694	LONRF1	LON peptidase N-terminal domain and ring finger 1	0.97806	1.0428	0.9555479
91695	RRP7B	ribosomal RNA processing 7 homolog B (S. cerevisiae)	0.98654	0.96211	0.9995756
91703	ACY3	aspartoacylase (aminocyclase) 3	0.98654	0.973948	1.0359699
91734	IDI2	isopentenyl-diphosphate delta isomerase 2	0.99647	0.999587	0.9465616
91746	YTHDC1	YTH domain containing 1	0.99609	0.993492	0.9990725
91748	C14orf43	chromosome 14 open reading frame 43	0.97859	0.974924	0.995956
91749	KIAA1919	KIAA1919	1.02327	1.009615	0.9581771
91750	LIN52	lin-52 homolog (C. elegans)	0.99293	0.959652	0.9979573
91752	ZNF804A	zinc finger protein 804A	0.99053	0.994949	1.0201065
91754	NEK9	NIMA (never in mitosis gene a)- related kinase 9	0.95996	0.943719	0.991219
91768	CABLES1	Cdk5 and Abl enzyme substrate 1	1.001	0.979787	1.0024838
91775	FAM55C	family with sequence similarity 55, member C	0.9871	0.920539	1.0273304
91782	CHMP7	CHMP family, member 7	0.97524	0.884392	0.925718
91801	ALKBH8	alkB, alkylation repair homolog 8 (E. coli)	0.95505	0.945691	1.0651514
91807	MYLK3	myosin light chain kinase 3	1.00064	1.002559	1.0167284
91828	EXOC3L4	exocyst complex component 3-like 4	0.99077	0.976579	1.0431835
91833	WDR20	WD repeat domain 20	0.99457	0.998506	1.0138171
91851	CHRDL1	chordin-like 1	1.00236	0.995461	0.9999558
91860	CALML4	calmodulin-like 4	0.97175	0.957527	1.0431088
91862	MARVELD3	MARVEL domain containing 3	0.99369	0.990311	0.9978424
91869	RFT1	RFT1 homolog (S. cerevisiae)	0.98135	0.932797	1.0203072
91875	TTC5	tetratricopeptide repeat domain 5	0.93164	0.938377	1.0456995
91893	FDXACB1	ferredoxin-fold anticodon binding domain containing 1	0.95038	0.983843	1.0341232
91894	C11orf52	chromosome 11 open reading frame 52	0.9788	0.968753	1.0214778

91937	TIMD4	T-cell immunoglobulin and mucin domain containing 4	0.97832	0.997914	0.9449968
91942	NDUFAF2	NADH dehydrogenase (ubiquinone) 1 alpha subcomplex, assembly factor 2	0.99365	0.966661	0.9752437
91947	ARRDC4	arrestin domain containing 4	0.95872	0.911427	0.9839587
91948	LOC91948	hypothetical LOC91948	1.00009	1.012588	1.0303568
91949	COG7	component of oligomeric golgi complex 7	0.9782	0.983339	0.9839193
91966	CXorf40A	chromosome X open reading frame 40A	0.99062	0.969426	1.0215208
91975	ZNF300	zinc finger protein 300	0.98101	0.943138	0.9784662
91977	MYOZ3	myozenin 3	0.9944	0.976201	1.0003596
91978	C19orf20	chromosome 19 open reading frame 20	1.0055	0.981696	1.0338199
92002	FAM58A	family with sequence similarity 58, member A	0.98791	1.003088	1.069973
92014	MCART1	mitochondrial carrier triple repeat 1	0.9573	1.072254	1.0278893
92017	SNX29	sorting nexin 29	0.98503	0.911906	1.0487825
92070	C4orf42	chromosome 4 open reading frame 42	1.04007	0.961985	0.8969059
92086	GGTLC1	gamma-glutamyltransferase light chain 1	0.98641	1.049253	1.0326405
92092	ZC3HAV1L	zinc finger CCCH-type, antiviral 1-like	1.02544	0.916119	1.0637135
92105	INTS4	integrator complex subunit 4	0.92932	0.974596	1.0005021
92106	OXNAD1	oxidoreductase NAD-binding domain containing 1	0.95449	0.876925	0.829988
92126	DSEL	dermatan sulfate epimerase-like	0.9761	0.939208	0.98989
92129	RIPPLY1	ripply1 homolog (zebrafish)	0.99915	1.009402	0.9421495
92140	MTDH	metadherin	1.00578	0.923439	1.0224478
92154	MTSS1L	metastasis suppressor 1-like	1.00266	1.003091	0.9801307
92162	TMEM88	transmembrane protein 88	1	1.058585	0.9783816
92170	MTG1	mitochondrial GTPase 1 homolog (S. cerevisiae)	0.98296	1.055537	1.0139888
92181	UBTD2	ubiquitin domain containing 2	0.99244	0.996479	1.0002245
92196	DAPL1	death associated protein-like 1	0.99373	1.021496	1.0223035
92211	CDHR1	cadherin-related family member 1	0.99381	0.986357	1.021132
92235	DUSP27	dual specificity phosphatase 27 (putative)	1.00047	1.025839	1.0139668
92241	RCSD1	RCSD domain containing 1	1.01492	0.95084	0.936608
92255	LMBRD2	LMBR1 domain containing 2	0.98356	0.994057	1.0675491
92259	MRPS36	mitochondrial ribosomal protein S36	0.97708	0.965366	0.9871561
92270	ATP6AP1L	ATPase, H+ transporting, lysosomal accessory protein 1-like	1.00905	1.028044	0.9856388
92283	ZNF461	zinc finger protein 461	0.92232	0.990656	1.0459211
92285	ZNF585B	zinc finger protein 585B	0.9263	0.98296	0.9609899

92291	CAPN13	calpain 13	0.99953	1.006309	1.0129666
92292	GLYATL1	glycine-N-acyltransferase-like 1	0.99249	0.993845	0.9993777
92293	TMEM132(	transmembrane protein 132C	0.99955	0.989168	0.9991335
92304	SCGB3A1	secretoglobin, family 3A, member 1	1.02908	1.062375	1.0517759
92305	TMEM129	transmembrane protein 129	1.00096	0.99731	1.0163687
92312	MEX3A	mex-3 homolog A (C. elegans)	0.99655	0.995333	1.0290854
92335	STRADA	STE20-related kinase adaptor alpha	0.93654	1.053294	0.9376351
92340	C17orf72	chromosome 17 open reading frame 72	0.98542	1.029976	1.0775451
92342	METTL18	methyltransferase like 18	0.92959	1.019238	1.1577396
92344	GORAB	golgin, RAB6-interacting	0.98234	1.001051	0.9868764
92345	NAF1	nuclear assembly factor 1 homolog (S. cerevisiae)	0.99924	0.939568	0.9655457
92346	C1orf105	chromosome 1 open reading frame 105	1.01797	1.038183	1.0045418
92359	CRB3	crumbs homolog 3 (Drosophila)	0.98212	0.959041	1.0796913
92369	SPSB4	splA/ryanodine receptor domain and SOCS box containing 4	1.00323	1.016533	0.997741
92370	ACPL2	acid phosphatase-like 2	0.97935	0.999298	0.9476313
92399	MRRF	mitochondrial ribosome recycling factor	0.9619	0.988602	1.0079771
92400	RBM18	RNA binding motif protein 18	0.92633	0.96978	1.1411836
92421	CHMP4C	chromatin modifying protein 4C	0.99982	0.986887	1.0002597
92427	RPS2P4	ribosomal protein S2 pseudogene 4	0.95431	0.986336	1.0585822
92482	BBIP1	BBSome interacting protein 1	0.96206	0.998939	0.9891535
92483	LDHAL6B	lactate dehydrogenase A-like 6B	1.00235	0.969602	1.0270477
92507	RPS4XP2	ribosomal protein S4X pseudogene 2	0.93729	0.968061	1.017789
92521	SPECC1	sperm antigen with calponin homology and coiled-coil domains 1	1.01093	0.977406	1.0052101
92552	ATXN3L	ataxin 3-like	1.0176	0.958999	1.0218319
92558	CCDC64	coiled-coil domain containing 64	0.97703	1.05346	1.013175
92565	FANK1	fibronectin type III and ankyrin repeat domains 1	1.00147	0.99424	1.0079093
92579	G6PC3	glucose 6 phosphatase, catalytic, 3	0.93751	0.893165	1.0683317
92591	ASB16	ankyrin repeat and SOCS box containing 16	0.99603	1.01937	1.0005407
92595	ZNF764	zinc finger protein 764	0.97291	0.95775	0.9547939
92597	MOBKL1A	MOB1, Mps One Binder kinase activator-like 1A (yeast)	1.01943	0.95568	1.041859
92609	TIMM50	translocase of inner mitochondrial membrane 50 homolog (S. cerevisiae)	0.94255	0.924797	1.0762038
92610	TIFA	TRAF-interacting protein with forkhead-associated domain	0.93813	0.986746	1.0424764
92667	C20orf72	chromosome 20 open reading frame 72	0.95617	1.002082	1.0190464

92675	DTD1	D-tyrosyl-tRNA deacylase 1 homolog (S. cerevisiae)	0.97837	0.994099	1.0166172
92689	FAM114A1	family with sequence similarity 114, member A1	1.00649	1.008028	1.0266422
92691	TMEM169	transmembrane protein 169	1.00451	1.017699	1.0243754
92703	TMEM183A	transmembrane protein 183A	1.01068	0.897524	1.010444
92714	ARRDC1	arrestin domain containing 1	0.98622	0.999852	1.0275222
92715	WDR85	WD repeat domain 85	0.95209	1.004675	1.013163
92736	OTOP2	otopetrin 2	0.99655	0.953285	0.9818118
92737	DNER	delta/notch-like EGF repeat containing	1.01239	0.997826	1.0116029
92745	SLC38A5	solute carrier family 38, member 5	0.96293	0.949119	1.1130072
92747	C20orf114	chromosome 20 open reading frame 114	0.99801	1.001451	0.9920412
92749	CCDC164	coiled-coil domain containing 164	1.00681	0.987983	1.0071608
92755	TUBBP1	tubulin, beta pseudogene 1	1.00113	0.986769	0.993818
92797	HELB	helicase (DNA) B	0.93117	0.993049	0.9162749
92799	SHKBP1	SH3KBP1 binding protein 1	0.9855	1.035667	0.9581871
92806	CENPBD1	CENPB DNA-binding domains containing 1	0.88786	0.992398	0.965308
92815	HIST3H2A	histone cluster 3, H2a	0.9821	0.997538	0.9536692
92822	ZNF276	zinc finger protein 276	0.94127	0.978177	0.9692389
92840	REEP6	receptor accessory protein 6	0.96259	0.979678	1.0300501
92856	IMP4	IMP4, U3 small nucleolar ribonucleoprotein, homolog (yeast)	0.97985	0.964168	1.0847593
92906	HNRPLL	heterogeneous nuclear ribonucleoprotein L-like	0.99405	1.014499	0.9724122
92912	UBE2Q2	ubiquitin-conjugating enzyme E2Q family member 2	0.94387	0.916649	1.0170039
92922	CCDC102A	coiled-coil domain containing 102A	0.96451	0.955529	1.0920958
92935	MARS2	methionyl-tRNA synthetase 2, mitochondrial	0.96398	1.003834	1.0483374
92949	ADAMTSL1	ADAMTS-like 1	1.00428	0.997725	1.0172091
92960	PEX11G	peroxisomal biogenesis factor 11 gamma	0.96884	0.959126	0.971625
92973	FP588	hypothetical LOC92973	0.95105	0.961919	1.0423923
92979	9-Mar	membrane-associated ring finger (C3HC4) 9	0.90451	1.100801	0.9907015
92999	ZBTB47	zinc finger and BTB domain containing 47	0.97571	1.017806	0.9951117
93010	B3GNT7	UDP-GlcNAc:betaGal beta-1,3-N-acetylglucosaminyltransferase 7	1.0102	1.035434	1.0015588
93034	NT5C1B	5'-nucleotidase, cytosolic IB	1.00069	0.988018	1.0186004
93035	PKHD1L1	polycystic kidney and hepatic disease 1 (autosomal recessive)-like 1	0.97047	0.955162	0.9185987
93058	COQ10A	coenzyme Q10 homolog A (S. cerevisiae)	0.92322	1.007044	0.9299316
93081	C13orf27	chromosome 13 open reading frame 27	0.97124	1.021251	0.9935871

93082	NEURL3	neuralized homolog 3 (Drosophila) pseudogene	1.00025	1.033468	1.018329
93099	DMKN	dermokine	0.9896	1.006963	1.0150828
93100	NAPRT1	nicotinate phosphoribosyltransferase domain containing 1	0.99424	1.016131	0.9956195
93107	KCNG4	potassium voltage-gated channel, subfamily G, member 4	1.0066	1.006252	0.9667519
93109	TMEM44	transmembrane protein 44	1.00303	1.025266	1.0050037
93129	ORAI3	ORAI calcium release-activated calcium modulator 3	0.91651	1.029174	1.0114671
93134	ZNF561	zinc finger protein 561	0.91694	0.941912	1.0735043
93144	RPS10P5	ribosomal protein S10 pseudogene 5	0.99865	1.024085	0.9827767
93145	OLFM2	olfactomedin 2	0.96665	1.010743	1.0080071
93164	HTR7P1	5-hydroxytryptamine (serotonin) receptor 7 pseudogene 1	0.94577	0.98045	0.9603374
93166	PRDM6	PR domain containing 6	1.00103	1.00149	1.0246646
93183	PIGM	phosphatidylinositol glycan anchor biosynthesis, class M	0.98843	0.907348	1.0719305
93185	IGSF8	immunoglobulin superfamily, member 8	0.98309	0.982383	1.0005334
93190	C1orf158	chromosome 1 open reading frame 158	0.99775	0.983177	0.9935496
93210	PGAP3	post-GPI attachment to proteins 3	0.93896	0.951919	1.0037343
93233	CCDC114	coiled-coil domain containing 114	0.99848	0.992645	0.9934143
93273	LEMD1	LEM domain containing 1	1.00046	0.959971	1.006915
93323	HAUS8	HAUS augmin-like complex, subunit 8	0.97005	0.952844	0.9723877
93343	FAM125A	family with sequence similarity 125, member A	1.00253	0.929107	1.0846235
93349	SP140L	SP140 nuclear body protein-like	0.98791	0.985045	0.9423135
93377	OPALIN	oligodendrocytic myelin paranodal and inner loop protein	1.00352	0.985347	1.0041972
93380	MMGT1	membrane magnesium transporter 1	0.93191	1.040259	1.1121196
93408	MYL10	myosin, light chain 10, regulatory	1.00214	0.989186	1.0418251
93426	SYCE1	synaptonemal complex central element protein 1	0.99036	0.992294	1.0204196
93432	LOC93432	maltase-glucoamylase-like pseudogene	1.02863	0.991171	0.9876945
93436	ARMC6	armadillo repeat containing 6	0.95519	0.942396	1.0179346
93474	ZNF670	zinc finger protein 670	0.9941	1.011886	0.9888557
93487	MAPK1IP1L	mitogen-activated protein kinase 1 interacting protein 1-like	0.97028	0.987976	1.0271306
93517	SDR42E1	short chain dehydrogenase/reductase family 42E, member 1	0.99372	0.967576	0.9785636
93550	ANUBL1	AN1, ubiquitin-like, homolog (Xenopus laevis)	0.94724	0.888156	0.9863388
93556	EGFEM1P	EGF-like and EMI domain containing 1, pseudogene	1.02468	1.033436	1.0072999
93587	RG9MTD2	RNA (guanine-9-) methyltransferase domain containing 2	0.98788	0.970225	1.0273756
93589	CACNA2D4	calcium channel, voltage-dependent, alpha 2/delta subunit 4	1.00198	1.005222	0.996276

93594	WDR67	WD repeat domain 67	0.97997	0.928577	0.9800276
93611	FBXO44	F-box protein 44	0.97262	0.949546	0.9925394
93621	MRFAP1	Morf4 family associated protein 1	0.92605	0.875223	0.9504505
93622	LOC93622	hypothetical LOC93622	1.00374	1.045197	1.0015225
93624	TADA2B	transcriptional adaptor 2B	0.97133	0.87678	0.9473575
93627	TBCK	TBC1 domain containing kinase	0.97982	0.979274	1.0003229
93643	TJAP1	tight junction associated protein 1 (peripheral)	0.98555	1.006158	0.9788955
93649	MYOCD	myocardin	1.00249	1.00093	1.0027776
93650	ACPT	acid phosphatase, testicular	0.99465	0.985189	1.066742
93653	ST7-AS1	ST7 antisense RNA 1 (non-protein coding)	1.00165	0.985225	1.0359991
93654	ST7-AS2	ST7 antisense RNA 2 (non-protein coding)	1.00346	1.042895	1.0617484
93661	CAPZA3	capping protein (actin filament) muscle Z-line, alpha 3	0.98796	1.003223	0.9995813
93663	ARHGAP18	Rho GTPase activating protein 18	0.9933	0.977527	1.0273931
93664	CADPS2	Ca++-dependent secretion activator 2	1.00336	0.988792	1.0068767
93948	RPS23P1	ribosomal protein S23 pseudogene 1	1.02686	1.021769	0.8685772
93953	ACRC	acidic repeat containing	0.9648	1.032227	0.9303392
93973	ACTR8	ARP8 actin-related protein 8 homolog (yeast)	0.98055	0.970343	1.0565099
93974	ATPIF1	ATPase inhibitory factor 1	0.96826	0.893277	1.0130792
93978	CLEC6A	C-type lectin domain family 6, member A	0.9175	0.893982	1.0274705
93979	CPA5	carboxypeptidase A5	1.0123	0.999446	1.0084675
93986	FOXP2	forkhead box P2	1.00009	1.01467	1.0185088
93996	NDUFB3P3	NADH dehydrogenase (ubiquinone) 1 beta subcomplex, 3, 12kDa pseudogene 3	0.98817	1.035234	1.0416806
94005	PIGS	phosphatidylinositol glycan anchor biosynthesis, class S	0.95636	1.027822	1.0818834
94009	SERHL	serine hydrolase-like	1.01381	0.98394	1.004042
94015	TTYH2	tweety homolog 2 (Drosophila)	0.95214	0.939051	0.9926755
94025	MUC16	mucin 16, cell surface associated	1.00619	1.016302	0.9889075
94026	POM121L2	POM121 membrane glycoprotein-like 2	0.9985	1.019395	1.0332165
94030	LRRC4B	leucine rich repeat containing 4B	0.97994	1.037184	0.9952903
94031	HTRA3	HtrA serine peptidase 3	0.99716	1.006092	1.0214106
94032	CAMK2N2	calcium/calmodulin-dependent protein kinase II inhibitor 2	0.98257	0.943122	1.0571149
94033	FTMT	ferritin mitochondrial	1.01491	0.979893	1.0425747
94039	ZNF101	zinc finger protein 101	0.93138	0.957086	0.8863527
94056	SYAP1	synapse associated protein 1	0.95139	1.044843	0.9766714

94059	LENG9	leukocyte receptor cluster (LRC) member 9	0.97909	1.010168	1.0860039
94081	SFXN1	sideroflexin 1	0.9968	1.005755	1.0542874
94086	HSPB9	heat shock protein, alpha-crystallin-related, B9	0.99417	1.004843	1.0575654
94097	SFXN5	sideroflexin 5	1.01689	0.98439	1.0320378
94101	ORMDL1	ORM1-like 1 (S. cerevisiae)	1.02448	0.837751	0.9791174
94103	ORMDL3	ORM1-like 3 (S. cerevisiae)	0.9534	1.002103	1.0555145
94104	GCFC1	GC-rich sequence DNA-binding factor 1	0.96387	0.991347	1.1529639
94107	TMEM203	transmembrane protein 203	0.94562	0.836752	1.0433391
94120	SYTL3	synaptotagmin-like 3	1.00716	1.099626	0.9339721
94121	SYTL4	synaptotagmin-like 4	0.94836	0.935958	0.9925368
94122	SYTL5	synaptotagmin-like 5	0.99183	1.015842	1.0251789
94134	ARHGAP12	Rho GTPase activating protein 12	0.91204	1.011345	1.0187588
94137	RP1L1	retinitis pigmentosa 1-like 1	0.99289	0.973566	1.0222034
94160	ABCC12	ATP-binding cassette, sub-family C (CFTR/MRP), member 12	0.9985	0.993462	0.9870383
94233	OPN4	opsin 4	0.99894	0.987066	0.9914438
94234	FOXQ1	forkhead box Q1	0.99601	1.004753	1.0686069
94235	GNG8	guanine nucleotide binding protein (G protein), gamma 8	0.96593	0.958203	1.0092317
94236	DNAJA1P5	DnaJ (Hsp40) homolog, subfamily A, member 1 pseudogene 5	1.02404	1.024546	0.9913388
94239	H2AFV	H2A histone family, member V	1.01334	0.928032	0.9407916
94240	EPSTI1	epithelial stromal interaction 1 (breast)	0.96996	0.96797	1.0716699
94241	TP53INP1	tumor protein p53 inducible nuclear protein 1	0.9827	0.894575	0.9675754
94274	PPP1R14A	protein phosphatase 1, regulatory (inhibitor) subunit 14A	0.97322	0.960282	0.9540751
95681	TSGA14	testis specific, 14	0.97181	0.980928	1.0048457
96459	FNIP1	folliculin interacting protein 1	0.97281	1.00689	0.9957238
96597	TBC1D27	TBC1 domain family, member 27	1.0102	0.971149	1.0149455
96764	TGS1	trimethylguanosine synthase 1	0.93654	0.868799	1.0567326
103910	MYL12B	myosin, light chain 12B, regulatory	0.9989	0.992857	0.9458137
103911	RPS27AP1	ribosomal protein S27a pseudogene 1	0.95918	0.936635	0.9987394
112398	EGLN2	egl nine homolog 2 (C. elegans)	0.95248	1.050946	0.9701521
112399	EGLN3	egl nine homolog 3 (C. elegans)	0.96593	0.962353	1.0262057
112401	BIRC8	baculoviral IAP repeat containing 8	0.95817	1.003779	0.9954604
112464	PRKCDBP	protein kinase C, delta binding protein	1.0082	1.014411	1.0870741
112476	PRRT2	proline-rich transmembrane protein 2	1.00677	1.043557	1.1403915

112479	ERI2	ERI1 exoribonuclease family member 2	0.99178	0.980159	1.0155925
112483	SAT2	spermidine/spermine N1-acetyltransferase family member 2	0.92676	1.014001	1.0290402
112487	C14orf126	chromosome 14 open reading frame 126	0.92418	0.920898	0.9860868
112495	GTF3C6	general transcription factor IIIC, polypeptide 6, alpha 35kDa	1.00298	0.92579	0.9405414
112574	SNX18	sorting nexin 18	1.04261	1.058285	0.9798782
112609	MRAP2	melanocortin 2 receptor accessory protein 2	1.00692	1.000344	0.994809
112611	RWDD2A	RWD domain containing 2A	0.98024	0.987391	1.0210134
112616	CMTM7	CKLF-like MARVEL transmembrane domain containing 7	1	0.939896	1.001119
112703	FAM71E1	family with sequence similarity 71, member E1	0.99586	0.977097	1.0096294
112724	RDH13	retinol dehydrogenase 13 (all-trans/9-cis)	0.9898	0.984275	0.9856991
112744	IL17F	interleukin 17F	0.99789	1.010258	0.9934319
112752	C14orf179	chromosome 14 open reading frame 179	0.96924	0.99247	1.0470179
112755	STX1B	syntaxin 1B	0.99384	0.997786	1.0044871
112770	C1orf85	chromosome 1 open reading frame 85	1.00673	0.864869	1.0973471
112802	KRT71	keratin 71	0.98105	1.009123	0.9993027
112812	FDX1L	ferredoxin 1-like	0.98335	0.990953	1.0292802
112817	HOGA1	4-hydroxy-2-oxoglutarate aldolase 1	0.99521	0.991505	0.9691761
112840	WDR89	WD repeat domain 89	0.94073	0.925712	0.9963121
112846	RBM8B	RNA binding motif protein 8B pseudogene	0.95401	0.983609	1.1009021
112849	C14orf149	chromosome 14 open reading frame 149	0.95719	0.987705	1.0653611
112858	TP53RK	TP53 regulating kinase	0.91037	0.974929	1.1484159
112869	CCDC101	coiled-coil domain containing 101	0.92558	0.885586	1.0189613
112885	PHF21B	PHD finger protein 21B	0.99547	0.983132	1.0152077
112936	VPS26B	vacuolar protein sorting 26 homolog B (S. pombe)	0.94807	0.970182	1.0157953
112937	GLB1L3	galactosidase, beta 1-like 3	0.98621	1.004729	0.9983003
112939	NACC1	nucleus accumbens associated 1, BEN and BTB (POZ) domain containing	0.96813	0.958816	1.0309111
112942	CCDC104	coiled-coil domain containing 104	0.97082	0.968861	1.0034791
112950	MED8	mediator complex subunit 8	0.97635	0.978168	1.0409474
113000	RPUSD1	RNA pseudouridylate synthase domain containing 1	0.94024	0.966905	1.0778967
113026	PLCD3	phospholipase C, delta 3	0.9877	1.024033	1.0148299
113091	PTH2	parathyroid hormone 2	0.9931	0.990398	1.0526997
113115	FAM54A	family with sequence similarity 54, member A	0.99866	0.968448	1.0012861
113130	CDCA5	cell division cycle associated 5	1.0169	0.980268	1.0169061

113146	AHNAK2	AHNAK nucleoprotein 2	0.98413	0.98668	0.9975111
113157	RPLP0P2	ribosomal protein, large, P0 pseudogene 2	0.99022	1.001083	1.0103518
113174	SAAL1	serum amyloid A-like 1	0.94164	0.968192	1.0668441
113177	IZUMO4	IZUMO family member 4	0.99189	0.980601	1.0125316
113178	SCAMP4	secretory carrier membrane protein 4	0.97753	0.971578	1.0578394
113189	CHST14	carbohydrate (N-acetylgalactosamine 4-0) sulfotransferase 14	0.99023	0.916661	1.1472541
113201	CASC4	cancer susceptibility candidate 4	0.96205	0.991783	1.023996
113220	KIF12	kinesin family member 12	0.98334	1.011645	1.0417726
113230	LOC11323C	hypothetical LOC113230	0.98758	0.995424	1.0201181
113235	SLC46A1	solute carrier family 46 (folate transporter), member 1	1.00255	1.007954	0.9802683
113246	C12orf57	chromosome 12 open reading frame 57	0.87146	0.920565	1.020044
113251	LARP4	La ribonucleoprotein domain family, member 4	0.94695	0.947208	1.1028853
113263	GLCCI1	glucocorticoid induced transcript 1	0.99723	0.987231	1.0187611
113277	TMEM106A	transmembrane protein 106A	0.97944	1.031923	0.9495671
113278	C20orf54	chromosome 20 open reading frame 54	0.98747	0.967494	1.0507103
113402	SFT2D1	SFT2 domain containing 1	1.05022	1.002822	1.1151802
113419	TEX261	testis expressed 261	1.03155	0.928692	1.0667551
113444	C1orf212	chromosome 1 open reading frame 212	0.98934	0.975183	1.0007885
113451	ADC	arginine decarboxylase	1.00074	0.991326	0.9898622
113452	TMEM54	transmembrane protein 54	1.00542	0.996815	0.9897033
113510	HELQ	helicase, POLQ-like	0.97638	0.982862	1.044933
113540	CMTM1	CKLF-like MARVEL transmembrane domain containing 1	0.97337	0.99409	0.9808224
113612	CYP2U1	cytochrome P450, family 2, subfamily U, polypeptide 1	0.98606	0.989005	0.9538746
113622	ADPRHL1	ADP-ribosylhydrolase like 1	1.00606	1.005268	0.9998604
113655	MFSD3	major facilitator superfamily domain containing 3	1.00538	0.999519	1.0587757
113675	SDSL	serine dehydratase-like	0.99316	0.97252	0.9920494
113691	MGC16703	tubulin, alpha pseudogene	1.00797	1.000088	1.0135991
113730	KLHDC7B	kelch domain containing 7B	0.98941	0.910634	0.9979808
113746	ODF3	outer dense fiber of sperm tails 3	0.98813	0.984949	0.9844031
113791	PIK3IP1	phosphoinositide-3-kinase interacting protein 1	0.92262	0.877384	0.9539808
113802	HENMT1	HEN1 methyltransferase homolog 1 (Arabidopsis)	0.99306	0.950264	0.9369152
113828	FAM83F	family with sequence similarity 83, member F	1.00061	0.979447	0.9929047
113829	SLC35A4	solute carrier family 35, member A4	0.98378	0.845059	1.0813406

113835	ZNF257	zinc finger protein 257	0.98398	1.05284	1.0256365
113878	DTX2	deltex homolog 2 (Drosophila)	0.97498	0.905192	0.9053078
114026	ZIM3	zinc finger, imprinted 3	0.99718	1.001972	1.0035103
114034	TOE1	target of EGR1, member 1 (nuclear)	1.0108	0.924944	0.9315359
114038	NCRNA003	non-protein coding RNA 313	0.99477	0.973149	1.0024094
114041	C21orf88	chromosome 21 open reading frame 88	0.98065	1.012045	1.0077259
114042	C21orf89	chromosome 21 open reading frame 89	0.9485	1.013678	1.0628427
114043	C21orf90	chromosome 21 open reading frame 90	0.98863	1.004228	0.9242112
114044	MCM3AP-A	MCM3AP antisense RNA 1 (non-protein coding)	0.96993	0.971544	1.0134434
114049	WBSCR22	Williams Beuren syndrome chromosome region 22	0.99819	0.919861	1.0212568
114088	TRIM9	tripartite motif containing 9	0.9903	0.988887	0.9978245
114112	TXNRD3	thioredoxin reductase 3	0.99783	0.981025	0.9922643
114131	UCN3	urocortin 3 (stresscopin)	0.99565	1.060174	1.019484
114132	SIGLEC11	sialic acid binding Ig-like lectin 11	1.02455	0.992133	0.9721508
114134	SLC2A13	solute carrier family 2 (facilitated glucose transporter), member 13	0.97546	0.967139	0.9922188
114182	FAM8A6P	family with sequence similarity 8, member A1 pseudogene	0.99683	0.993444	1.1001242
114186	SIGLEC27P	sialic acid binding Ig-like lectin 27, pseudogene	0.97865	1.03757	1.0604458
114188	SIGLEC29P	sialic acid binding Ig-like lectin 29, pseudogene	0.99172	0.948624	0.9922729
114190	SIGLEC30P	sialic acid binding Ig-like lectin 30, pseudogene	0.98555	1.051266	0.9810567
114192	SIGLEC20P	sialic acid binding Ig-like lectin 20, pseudogene	1.01485	1.010041	1.0340282
114195	SIGLEC22P	sialic acid binding Ig-like lectin 22, pseudogene	0.96222	0.866515	0.9639833
114196	SIGLEC24P	sialic acid binding Ig-like lectin 24, pseudogene	0.995	0.979849	0.9698462
114294	LACTB	lactamase, beta	1.00617	1.057517	0.996309
114299	PALM2	paralemmin 2	0.99579	1.024667	1.0033814
114327	EFHC1	EF-hand domain (C-terminal) containing 1	1.00083	0.915107	1.0152925
114569	MAL2	mal, T-cell differentiation protein 2 (gene/pseudogene)	1.01143	1.036954	1.0128079
114571	SLC22A9	solute carrier family 22 (organic anion transporter), member 9	0.98763	0.966038	1.0171662
114609	TIRAP	toll-interleukin 1 receptor (TIR) domain containing adaptor protein	0.9469	1.004132	1.0247468
114614	MIR155HG	MIR155 host gene (non-protein coding)	0.9062	1.039472	0.9416087
114625	ERMAP	erythroblast membrane-associated protein (Scianna blood group)	1.01174	0.971016	1.0208415
114659	LRRC37B	leucine rich repeat containing 37B	0.97331	0.939467	0.9607652
114757	CYGB	cytoglobin	0.99405	1.020962	0.9989247
114769	CARD16	caspase recruitment domain family, member 16	1.03799	1.206925	0.9622174

114770	PGLYRP2	peptidoglycan recognition protein 2	1.00008	1.002273	0.9998168
114771	PGLYRP3	peptidoglycan recognition protein 3	1.01663	0.991313	1.0107594
114780	PKD1L2	polycystic kidney disease 1-like 2	0.99257	0.982696	1.0081462
114781	BTBD9	BTB (POZ) domain containing 9	0.98258	0.955788	0.9895316
114783	LMTK3	lemur tyrosine kinase 3	0.96773	1.009796	1.0092848
114784	CSMD2	CUB and Sushi multiple domains 2	1.00647	0.998389	1.0123164
114785	MBD6	methyl-CpG binding domain protein 6	0.98341	0.981053	0.9363591
114786	XKR4	XK, Kell blood group complex subunit-related family, member 4	1.00243	1.007379	1.0102386
114787	GPRIN1	G protein regulated inducer of neurite outgrowth 1	1.00379	1.000944	1.0349126
114788	CSMD3	CUB and Sushi multiple domains 3	0.99472	0.997353	0.9994515
114789	SLC25A25	solute carrier family 25 (mitochondrial carrier; phosphate carrier), member 25	1.00802	1.005818	1.0208926
114790	STK11IP	serine/threonine kinase 11 interacting protein	1.01304	0.981384	1.0798564
114791	TUBGCP5	tubulin, gamma complex associated protein 5	0.93725	0.887384	1.0171677
114792	KLHL32	kelch-like 32 (Drosophila)	0.99523	0.970628	0.9987969
114793	FMNL2	formin-like 2	1.01577	1.006939	1.0393213
114794	ELFN2	extracellular leucine-rich repeat and fibronectin type III domain containing 2	0.99948	0.972674	1.0071003
114795	TMEM132E	transmembrane protein 132B	1.00979	1.005558	1.011091
114796	KIAA1908	hypothetical LOC114796	1.02353	0.984885	0.9467942
114798	SLITRK1	SLIT and NTRK-like family, member 1	1.01989	1.027858	1.0157226
114799	ESCO1	establishment of cohesion 1 homolog 1 (S. cerevisiae)	0.91705	1.010886	0.9454745
114800	CCDC85A	coiled-coil domain containing 85A	1.00181	1.022169	1.0204504
114801	TMEM200A	transmembrane protein 200A	0.99967	1.029404	1.0340429
114803	MYSM1	Myb-like, SWIRM and MPN domains 1	1.02949	0.961987	1.0377086
114804	RNF157	ring finger protein 157	0.90517	0.966675	1.0381789
114805	GALNT13	UDP-N-acetyl-alpha-D-galactosamine:polypeptide N-acetylgalactosaminyltransferase 13 (GalNAc-T13)	1.00311	1.016009	1.0128271
114814	GNRHR2	gonadotropin-releasing hormone (type 2) receptor 2	0.99148	1.025013	1.0239374
114815	SORCS1	sortilin-related VPS10 domain containing receptor 1	1.0039	0.994768	1.0036346
114817	CSPG4P5	chondroitin sulfate proteoglycan 4 pseudogene 5	1.00939	1.036193	0.9915353
114818	KLHL29	kelch-like 29 (Drosophila)	1.00208	1.005751	1.0113054
114819	CROCCP3	ciliary rootlet coiled-coil, rootletin pseudogene 3	0.99773	0.995435	0.9898775
114821	SCAND3	SCAN domain containing 3	1.00176	1.017531	1.0117647
114822	RHPN1	rhophilin, Rho GTPase binding protein 1	0.98597	1.005479	1.0095929
114823	LENG8	leukocyte receptor cluster (LRC) member 8	0.99594	1.056517	0.9449448

114824	PNMA5	paraneoplastic antigen like 5	0.99095	1.007113	1.0154042
114825	PWWP2A	PWWP domain containing 2A	1.00517	0.958624	1.0202729
114826	SMYD4	SET and MYND domain containing 4	0.92904	0.959917	0.9943162
114827	FHAD1	forkhead-associated (FHA) phosphopeptide binding domain 1	1.01334	1.004028	1.0117591
114836	SLAMF6	SLAM family member 6	0.95078	0.891599	0.9239656
114876	OSBPL1A	oxysterol binding protein-like 1A	0.96484	0.970151	0.999407
114879	OSBPL5	oxysterol binding protein-like 5	0.97547	1.001452	0.9429295
114880	OSBPL6	oxysterol binding protein-like 6	1.00374	1	1.005389
114881	OSBPL7	oxysterol binding protein-like 7	0.98041	1.039952	1.0187272
114882	OSBPL8	oxysterol binding protein-like 8	0.94571	0.98916	1.0422106
114883	OSBPL9	oxysterol binding protein-like 9	1.00362	0.981678	0.929842
114885	OSBPL11	oxysterol binding protein-like 11	1.01748	0.921842	1.036349
114897	C1QTNF1	C1q and tumor necrosis factor related protein 1	0.99441	1.024399	0.992716
114898	C1QTNF2	C1q and tumor necrosis factor related protein 2	1.00909	0.984051	1.0466579
114899	C1QTNF3	C1q and tumor necrosis factor related protein 3	0.98932	0.934591	0.9543301
114900	C1QTNF4	C1q and tumor necrosis factor related protein 4	1.00279	0.944495	1.0882296
114902	C1QTNF5	C1q and tumor necrosis factor related protein 5	0.99565	1.036937	0.9835022
114904	C1QTNF6	C1q and tumor necrosis factor related protein 6	0.9935	0.94444	0.9628876
114905	C1QTNF7	C1q and tumor necrosis factor related protein 7	1.00307	1.006463	0.9943996
114907	FBXO32	F-box protein 32	0.95866	0.968144	0.9380162
114908	TMEM123	transmembrane protein 123	0.95587	0.972825	1.0298695
114915	NCRNA002	non-protein coding RNA 219	0.95129	0.967407	0.95353
114926	C8orf40	chromosome 8 open reading frame 40	0.97476	0.980384	1.0142717
114928	GPRASP2	G protein-coupled receptor associated sorting protein 2	1.01023	0.980963	0.9448785
114932	MRFAP1L1	Morf4 family associated protein 1-like 1	0.94739	0.962677	1.0677898
114960	TSGA13	testis specific, 13	0.99767	0.998997	1.0390068
114971	PTPMT1	protein tyrosine phosphatase, mitochondrial 1	0.97737	1.026358	1.0957739
114984	FLYWCH2	FLYWCH family member 2	0.98488	1.022892	1.0110812
114987	WDR31	WD repeat domain 31	0.98614	0.99585	1.0176791
114990	VASN	vasorin	0.99809	1.010793	1.0069786
114991	ZNF618	zinc finger protein 618	0.98643	0.989994	1.0028287
115004	MB21D1	Mab-21 domain containing 1	1.01312	0.907622	1.0765766
115019	SLC26A9	solute carrier family 26, member 9	1.01061	1.02355	1.0036251

115024	NT5C3L	5'-nucleotidase, cytosolic III-like	0.96214	0.86224	0.9714614
115098	CCDC124	coiled-coil domain containing 124	0.9764	0.993844	1.0784299
115106	HAUS1	HAUS augmin-like complex, subunit 1	0.95272	0.995825	1.0088034
115111	SLC26A7	solute carrier family 26, member 7	1.01384	1.014351	1.0307263
115123	3-Mar	membrane-associated ring finger (C3HC4) 3	0.99324	0.953955	0.9976294
115196	ZNF554	zinc finger protein 554	0.94552	1.033448	1.0204448
115201	ATG4A	ATG4 autophagy related 4 homolog A (S. cerevisiae)	0.93943	0.999771	1.0590092
115207	KCTD12	potassium channel tetramerisation domain containing 12	0.98977	0.863546	1.0441937
115209	OMA1	OMA1 homolog, zinc metallopeptidase (S. cerevisiae)	1.00078	1.074605	1.0241471
115265	DDIT4L	DNA-damage-inducible transcript 4-like	0.98339	0.998391	1.0470434
115273	RAB42	RAB42, member RAS oncogene family	0.9865	0.998331	1.0212426
115286	SLC25A26	solute carrier family 25, member 26	1.00535	0.898141	0.9973753
115290	FBXO17	F-box protein 17	0.9905	1.006815	1.0197027
115294	PCMTD1	protein-L-isoaspartate (D-aspartate) O-methyltransferase domain containing 1	0.95573	0.981658	0.9633535
115330	GPR146	G protein-coupled receptor 146	0.95645	0.956434	1.1542003
115352	FCRL3	Fc receptor-like 3	1.03826	1.047373	0.9008145
115353	LRRC42	leucine rich repeat containing 42	0.9875	0.9975	1.0244912
115361	GBP4	guanylate binding protein 4	1.02236	0.983107	0.9889415
115362	GBP5	guanylate binding protein 5	1.01966	1.073549	0.8487395
115399	LRRC56	leucine rich repeat containing 56	0.98935	0.995255	1.0074562
115416	C7orf30	chromosome 7 open reading frame 30	0.98874	0.88626	0.9753416
115426	UHRF2	ubiquitin-like with PHD and ring finger domains 2	0.95383	0.972551	1.0545512
115509	ZNF689	zinc finger protein 689	0.94977	0.934584	1.0853664
115548	FCHO2	FCH domain only 2	0.98976	0.907495	1.0091403
115557	ARHGEF25	Rho guanine nucleotide exchange factor (GEF) 25	0.9946	0.975629	1.0038733
115560	ZNF501	zinc finger protein 501	0.97276	0.984527	1.0177504
115572	FAM46B	family with sequence similarity 46, member B	1.02138	0.969525	1.0248153
115584	SLC5A11	solute carrier family 5 (sodium/glucose cotransporter), member 11	1.00505	1.012021	1.006389
115653	KIR3DL3	killer cell immunoglobulin-like receptor, three domains, long cytoplasmic tail, 3	0.97906	0.967832	0.9800893
115669	[No Symbo	[No Name]	0.98896	1.044985	0.9975981
115677	NOSTRIN	nitric oxide synthase trafficker	1.00402	1.002222	0.9966212
115701	ALPK2	alpha-kinase 2	0.99459	1.010596	1.0003746
115703	ARHGAP33	Rho GTPase activating protein 33	0.99497	1.003721	1.0586051

115704	EVI5L	ecotropic viral integration site 5-like	0.96641	0.975334	1.0375259
115708	TRMT61A	tRNA methyltransferase 61 homolog A (S. cerevisiae)	0.97194	0.967182	1.058782
115727	RASGRP4	RAS guanyl releasing protein 4	0.95601	0.863126	1.0171504
115749	C12orf56	chromosome 12 open reading frame 56	0.98829	0.988005	1.0293637
115752	DIS3L	DIS3 mitotic control homolog (S. cerevisiae)-like	0.92574	0.923571	0.9774573
115761	ARL11	ADP-ribosylation factor-like 11	0.98563	1.022297	1.0278791
115795	C3orf24	chromosome 3 open reading frame 24	1.01608	1.015154	0.9931476
115811	IQCD	IQ motif containing D	0.98917	1.014564	1.0044755
115817	DHRS1	dehydrogenase/reductase (SDR family) member 1	1.00572	1.041634	0.9586653
115825	WDFY2	WD repeat and FYVE domain containing 2	0.99384	0.976132	1.0293959
115861	NXNL1	nucleoredoxin-like 1	0.97404	1.023472	1.0996873
115908	CTHRC1	collagen triple helix repeat containing 1	0.98726	1.028832	1.0157575
115939	C16orf42	chromosome 16 open reading frame 42	0.97106	0.935533	1.1086391
115948	CCDC151	coiled-coil domain containing 151	0.99725	0.985369	1.0150386
115950	ZNF653	zinc finger protein 653	0.95757	0.969936	1.047208
115992	RNF166	ring finger protein 166	0.96156	1.071391	0.9585775
116028	C16orf75	chromosome 16 open reading frame 75	0.97322	1.004677	1.1082431
116039	OSR2	odd-skipped related 2 (Drosophila)	0.98614	0.988264	1.0288466
116064	LRRC58	leucine rich repeat containing 58	0.968	1.000344	1.0489758
116068	LYSMD3	LysM, putative peptidoglycan-binding, domain containing 3	0.95328	0.964734	0.9212032
116071	BATF2	basic leucine zipper transcription factor, ATF-like 2	0.99419	0.997499	0.9965769
116085	SLC22A12	solute carrier family 22 (organic anion/urate transporter), member 12	1.01138	1.007846	1.0072324
116092	DNTTIP1	deoxynucleotidyltransferase, terminal, interacting protein 1	0.92531	1.030259	0.9593725
116093	DIRC1	disrupted in renal carcinoma 1	1.01308	0.983212	0.9994894
116113	FOXP4	forkhead box P4	0.99207	0.971812	0.984753
116115	ZNF526	zinc finger protein 526	0.95105	1.033822	0.9998441
116123	FMO9P	flavin containing monooxygenase 9 pseudogene	1.01444	1.009456	0.9865954
116135	LRRC3B	leucine rich repeat containing 3B	1.00009	0.99991	1.0398926
116138	KLHDC3	kelch domain containing 3	0.97018	0.911888	1.0905901
116143	WDR92	WD repeat domain 92	0.99919	0.956699	1.0002994
116150	NUS1	nuclear undecaprenyl pyrophosphate synthase 1 homolog (S. cerevisiae)	0.98192	0.957767	1.0018059
116151	C20orf108	chromosome 20 open reading frame 108	0.98756	0.973464	1.0046553
116154	PHACTR3	phosphatase and actin regulator 3	0.99321	0.99382	0.9816397

116159	CYYR1	cysteine/tyrosine-rich 1	1.01497	0.995593	0.9925083
116173	CMTM5	CKLF-like MARVEL transmembrane domain containing 5	0.93331	1.000226	0.9869768
116179	TGM7	transglutaminase 7	1.00066	1.009859	0.9928496
116211	TM4SF19	transmembrane 4 L six family member 19	1.03092	1.027838	0.983624
116225	ZMYND19	zinc finger, MYND-type containing 19	0.97104	0.97027	1.0364676
116228	FAM36A	family with sequence similarity 36, member A	0.97095	0.943627	0.9769258
116236	ABHD15	abhydrolase domain containing 15	0.94054	0.968214	0.9784626
116238	TLCD1	TLC domain containing 1	1.01719	0.992504	1.0236954
116254	C6orf72	chromosome 6 open reading frame 72	1.01723	0.840317	0.889208
116255	MOGAT1	monoacylglycerol O-acyltransferase 1	0.99339	1.001901	1.0403493
116285	ACSM1	acyl-CoA synthetase medium-chain family member 1	0.98566	0.981173	0.9849732
116337	PANX3	pannexin 3	0.99278	0.968297	0.9443574
116349	C5orf55	chromosome 5 open reading frame 55	0.9973	1.014357	0.9791164
116369	SLC26A8	solute carrier family 26, member 8	1.01419	1.011067	1.0082897
116372	LYPD1	LY6/PLAUR domain containing 1	0.99236	1.024666	1.0533885
116379	IL22RA2	interleukin 22 receptor, alpha 2	1.0092	1.001191	1.0199185
116412	ZNF837	zinc finger protein 837	0.98834	1.02107	0.9946604
116437	LOC116437	hypothetical LOC116437	1.01272	0.936682	0.9954691
116441	TM4SF18	transmembrane 4 L six family member 18	1.01258	1.002519	0.9738125
116442	RAB39B	RAB39B, member RAS oncogene family	0.9128	1.018603	0.991514
116443	GRIN3A	glutamate receptor, ionotropic, N-methyl-D-aspartate 3A	1.00385	0.998788	1.0027642
116444	GRIN3B	glutamate receptor, ionotropic, N-methyl-D-aspartate 3B	1.01694	1.034468	1.053629
116447	TOP1MT	topoisomerase (DNA) I, mitochondrial	0.93997	1.056452	1.0005318
116448	OLIG1	oligodendrocyte transcription factor 1	0.9745	0.996547	1.0733786
116449	CLNK	cytokine-dependent hematopoietic cell linker	1.01801	0.999537	1.0184887
116461	TSEN15	tRNA splicing endonuclease 15 homolog (S. cerevisiae)	0.98641	0.967012	1.1558093
116496	FAM129A	family with sequence similarity 129, member A	0.99712	1.079027	1.0012984
116511	MAS1L	MAS1 oncogene-like	1.01776	0.962301	1.0029733
116512	MRGPRD	MAS-related GPR, member D	0.97227	1.039503	1.018047
116519	APOA5	apolipoprotein A-V	1.00165	0.969007	0.9966436
116534	MRGPRE	MAS-related GPR, member E	0.9937	0.948516	0.9578047
116540	MRPL53	mitochondrial ribosomal protein L53	0.94227	0.902645	1.1784437
116541	MRPL54	mitochondrial ribosomal protein L54	0.95677	1.032779	1.060093

116729	DYSFIP1	dysferlin interacting protein 1	0.99618	0.996792	1.0073977
116832	RPL39L	ribosomal protein L39-like	0.9737	0.976127	0.9880781
116835	HSPA12B	heat shock 70kD protein 12B	0.96728	0.988726	1.0533159
116840	CNTROB	centrobin, centrosomal BRCA2 interacting protein	0.96615	1.050381	0.9764772
116841	SNAP47	synaptosomal-associated protein, 47kDa	0.99695	0.989694	1.0831819
116842	LEAP2	liver expressed antimicrobial peptide 2	1.01421	1.016469	0.9772341
116843	C6orf192	chromosome 6 open reading frame 192	0.97218	0.94189	0.9436546
116844	LRG1	leucine-rich alpha-2-glycoprotein 1	1.00195	1.030211	0.9410629
116931	MED12L	mediator complex subunit 12-like	1.00059	0.965311	1.0034431
116966	WDR17	WD repeat domain 17	0.99145	0.983475	0.997512
116969	ART5	ADP-ribosyltransferase 5	0.98455	1.020873	1.0066815
116983	ACAP3	ArfGAP with coiled-coil, ankyrin repeat and PH domains 3	0.98667	0.989774	1.0670782
116984	ARAP2	ArfGAP with RhoGAP domain, ankyrin repeat and PH domain 2	1.03671	1.101569	1.0361413
116985	ARAP1	ArfGAP with RhoGAP domain, ankyrin repeat and PH domain 1	0.97195	1.074194	0.9276437
116986	AGAP2	ArfGAP with GTPase domain, ankyrin repeat and PH domain 2	0.94365	1.003288	1.0238076
116987	AGAP1	ArfGAP with GTPase domain, ankyrin repeat and PH domain 1	1.009	0.996642	1.0096831
116988	AGAP3	ArfGAP with GTPase domain, ankyrin repeat and PH domain 3	0.98876	1.000863	0.9843378
117143	TADA1	transcriptional adaptor 1	0.91608	0.811267	1.0829579
117145	THEM4	thioesterase superfamily member 4	1.00522	0.945992	0.9343023
117153	MIA2	melanoma inhibitory activity 2	0.98304	0.99273	1.0142146
117154	DACH2	dachshund homolog 2 (Drosophila)	1.00257	1.009596	0.9876521
117155	CATSPER2	cation channel, sperm associated 2	0.97524	0.991831	0.985411
117156	SCGB3A2	secretoglobin, family 3A, member 2	0.97263	1.042464	0.9644478
117159	DCD	dermcidin	1.01433	0.998182	1.0132445
117166	WFIKKN1	WAP, follistatin/kazal, immunoglobulin, kunitz and netrin domain containing 1	0.97821	0.956749	1.0270825
117177	RAB3IP	RAB3A interacting protein (rabin3)	0.93891	1.000558	1.0754448
117178	SSX2IP	synovial sarcoma, X breakpoint 2 interacting protein	0.96519	0.929646	0.941905
117194	MRGPRX2	MAS-related GPR, member X2	1.01472	1.03704	0.9779544
117196	MRGPRX4	MAS-related GPR, member X4	1.005	1.035409	0.9926218
117245	HRASLS5	HRAS-like suppressor family, member 5	0.97963	1.043498	0.9882538
117246	FTSJ3	FtsJ homolog 3 (E. coli)	0.90967	0.963609	1.0852205
117247	SLC16A10	solute carrier family 16, member 10 (aromatic amino acid transporter)	0.98469	0.979841	0.951758
117248	GALNTL2	UDP-N-acetyl-alpha-D-galactosamine:polypeptide N-acetylgalactosaminyltransferase-like 2	1.01063	1.013114	1.0194312

117283	IP6K3	inositol hexakisphosphate kinase 3	1.02047	1.038856	1.0359479
117285	DEFB118	defensin, beta 118	1.0145	0.992302	1.0483184
117286	CIB3	calcium and integrin binding family member 3	1.00284	1.01157	0.9667989
117289	TAGAP	T-cell activation RhoGTPase activating protein	0.96637	0.962982	0.9296711
117531	TMC1	transmembrane channel-like 1	0.99954	0.983578	0.9982358
117532	TMC2	transmembrane channel-like 2	0.99928	0.996062	1.0300042
117579	RLN3	relaxin 3	0.96468	1.005084	1.047277
117583	PARD3B	par-3 partitioning defective 3 homolog B (C. elegans)	1.00815	0.996075	1.0096112
117584	RFFL	ring finger and FYVE-like domain containing 1	0.95966	0.988138	0.9650576
117608	ZNF354B	zinc finger protein 354B	0.93828	0.89956	1.0137058
117852	TRIM78P	tripartite motif containing 78, pseudogene	0.96426	1.00087	1.047359
117854	TRIM6	tripartite motif containing 6	0.99093	1.008511	1.1075669
118424	UBE2J2	ubiquitin-conjugating enzyme E2, J2	1.00037	1.010009	1.033175
118426	LOH12CR1	loss of heterozygosity, 12, chromosomal region 1	0.99061	0.991009	0.953171
118427	OLFM3	olfactomedin 3	1.00515	0.98252	1.0068352
118429	ANTXR2	anthrax toxin receptor 2	1.00745	0.95367	1.0139416
118430	MUCL1	mucin-like 1	0.99224	1.017717	1.0484651
118442	GPR62	G protein-coupled receptor 62	1.02053	0.984105	0.9918863
118460	EXOSC6	exosome component 6	0.95391	0.886468	1.1381549
118461	C10orf71	chromosome 10 open reading frame 71	1.00762	1.007498	0.9935071
118471	PRAP1	proline-rich acidic protein 1	0.98715	1.029402	1.0172196
118472	ZNF511	zinc finger protein 511	0.94325	0.993363	1.0515635
118487	CHCHD1	coiled-coil-helix-coiled-coil-helix domain containing 1	0.92381	1.097254	1.0619511
118490	ZMYND17	zinc finger, MYND-type containing 17	1.01324	0.996784	0.9974729
118491	TTC18	tetratricopeptide repeat domain 18	0.99345	0.993248	0.9704774
118611	C10orf90	chromosome 10 open reading frame 90	1.00713	1.002563	0.9951015
118663	BTBD16	BTB (POZ) domain containing 16	1.01038	0.989892	1.0004542
118670	FAM24A	family with sequence similarity 24, member A	1.04656	1.057101	1.01233
118672	PSTK	phosphoseryl-tRNA kinase	0.97262	0.975049	0.9731213
118738	ZNF488	zinc finger protein 488	1.01773	0.974868	1.0438767
118788	PIK3AP1	phosphoinositide-3-kinase adaptor protein 1	0.9735	1.119304	0.9879979
118812	MORN4	MORN repeat containing 4	0.97811	0.993323	0.9914975
118813	ZFYVE27	zinc finger, FYVE domain containing 27	0.96837	0.973612	0.9848947

118856	MMP21	matrix metallopeptidase 21	1.00188	1.049697	1.0352131
118881	COMTD1	catechol-O-methyltransferase domain containing 1	0.9979	1.042236	1.0666493
118924	FRA10AC1	fragile site, folic acid type, rare, fra(10)(q23.3) or fra(10)(q24.2) candidate 1	0.90733	0.991023	1.0091073
118932	ANKRD22	ankyrin repeat domain 22	0.97769	1.057992	0.9846781
118980	SFXN2	sideroflexin 2	0.96778	1.023904	1.0619652
118987	PDZD8	PDZ domain containing 8	0.98747	0.989916	1.0500247
119032	C10orf32	chromosome 10 open reading frame 32	0.93092	0.855921	1.0377276
119180	LYZL2	lysozyme-like 2	0.96547	1.042122	1.0604359
119358	SNRPD2P1	small nuclear ribonucleoprotein D2 pseudogene 1	0.99083	1.021228	1.0467184
119369	NUDT9P1	nudix (nucleoside diphosphate linked moiety X)-type motif 9 pseudogene 1	0.99199	0.965815	0.985193
119385	AGAP11	ankyrin repeat and GTPase domain Arf GTPase activating protein 11	0.99525	0.988583	1.0151108
119391	GSTO2	glutathione S-transferase omega 2	0.98445	0.988177	0.9733948
119392	MEIR5	MEI5 meiotic recombination protein homolog (S. cerevisiae)	0.96481	0.991576	0.9713044
119395	CALHM3	calcium homeostasis modulator 3	0.97694	0.961833	1.0043183
119437	CTAGE7P	CTAGE family, member 7, pseudogene	0.9843	0.941345	1.0239106
119467	CLRN3	clarin 3	1.00582	1.008883	0.9988947
119504	ANAPC16	anaphase promoting complex subunit 16	0.95429	0.965791	1.0406975
119548	PNLIPRP3	pancreatic lipase-related protein 3	0.99604	1.014006	0.9963912
119559	SFXN4	sideroflexin 4	0.95587	0.970475	1.019403
119587	CPXM2	carboxypeptidase X (M14 family), member 2	1.01048	1.021889	0.9994413
119678	OR52E2	olfactory receptor, family 52, subfamily E, member 2	1.00999	1.03546	1.0418302
119679	OR52J3	olfactory receptor, family 52, subfamily J, member 3	0.98388	0.958362	1.0398532
119682	OR51L1	olfactory receptor, family 51, subfamily L, member 1	1.03781	0.960092	0.9513618
119687	OR51A7	olfactory receptor, family 51, subfamily A, member 7	1.01495	1.050191	1.0341014
119694	OR51F2	olfactory receptor, family 51, subfamily F, member 2	1.00737	1.001382	1.0027093
119695	OR52R1	olfactory receptor, family 52, subfamily R, member 1	0.9928	1.02446	0.963268
119710	C11orf74	chromosome 11 open reading frame 74	0.99216	1.019401	1.0305642
119722	KRT18P14	keratin 18 pseudogene 14	1.00123	1.022793	0.9309297
119750	OR4C2P	olfactory receptor, family 4, subfamily C, member 2 pseudogene	1.04993	1.040482	0.939519
119764	OR4X2	olfactory receptor, family 4, subfamily X, member 2	1.01229	0.998853	1.016557
119765	OR4B1	olfactory receptor, family 4, subfamily B, member 1	0.98396	0.958151	0.9957965
119772	OR52M1	olfactory receptor, family 52, subfamily M, member 1	0.97996	0.972156	1.0360907
119774	OR52K2	olfactory receptor, family 52, subfamily K, member 2	0.97034	1.004776	1.0341873

119775	OR52P2P	olfactory receptor, family 52, subfamily P, member 2 pseudogene	1.01361	0.994574	0.9682986
120065	OR5P2	olfactory receptor, family 5, subfamily P, member 2	1.00529	1.015888	0.9874876
120066	OR5P3	olfactory receptor, family 5, subfamily P, member 3	1.00834	0.940539	0.9641097
120071	GYLTL1B	glycosyltransferase-like 1B	0.96714	1.039516	0.9646215
120103	SLC36A4	solute carrier family 36 (proton/amino acid symporter), member 4	0.98309	1.121888	0.9505522
120114	FAT3	FAT tumor suppressor homolog 3 (Drosophila)	1.00495	1.00635	0.9949349
120224	TMEM45B	transmembrane protein 45B	0.96679	0.905308	0.9462992
120227	CYP2R1	cytochrome P450, family 2, subfamily R, polypeptide 1	0.9345	0.949977	1.0979874
120237	DBX1	developing brain homeobox 1	0.98152	0.994738	1.0537419
120318	RPS6P17	ribosomal protein S6 pseudogene 17	0.98181	0.881998	1.0333695
120321	CSNK1A1P2	casein kinase 1, alpha 1 pseudogene 2	1.00095	1.049449	1.0090917
120329	[No Symbo	[No Name]	0.99403	0.988269	1.0236341
120364	LOC120364	heterogeneous nuclear ribonucleoprotein A1 pseudogene	1.00367	1.001072	0.997744
120376	C11orf93	chromosome 11 open reading frame 93	0.99697	1.007912	0.9915626
120379	PIH1D2	PIH1 domain containing 2	1.00644	0.981341	1.0102934
120400	FAM55A	family with sequence similarity 55, member A	1.00454	0.983186	0.9902717
120406	FAM55B	family with sequence similarity 55, member B	0.99957	1.001082	1.0085983
120425	AMICA1	adhesion molecule, interacts with CXADR antigen 1	0.98547	0.856994	0.9428766
120449	LOC120449	C-terminal binding protein 2 pseudogene	0.98952	0.97592	1.0274878
120526	DNAJC24	DnaJ (Hsp40) homolog, subfamily C, member 24	0.96059	0.951515	1.0203999
120534	C11orf46	chromosome 11 open reading frame 46	0.9602	1.017774	1.0335805
120586	OR8I2	olfactory receptor, family 8, subfamily I, member 2	0.97933	0.982674	1.017146
120775	OR2D3	olfactory receptor, family 2, subfamily D, member 3	1.00927	1.025392	1.0142336
120776	OR2D2	olfactory receptor, family 2, subfamily D, member 2	1.04522	1.01897	1.1037748
120793	OR56A4	olfactory receptor, family 56, subfamily A, member 4	1.03979	1.005721	0.9754994
120796	OR56A1	olfactory receptor, family 56, subfamily A, member 1	1.03938	1.029828	1.004963
120824	LOC120824	tripartite motif-containing protein ENSP00000309378-like	1.00708	0.998258	0.9736269
120863	DEPDC4	DEP domain containing 4	0.97102	0.993157	1.0257222
120872	RPL7P38	ribosomal protein L7 pseudogene 38	0.947	0.986436	1.0168302
120892	LRRK2	leucine-rich repeat kinase 2	0.99119	0.862708	1.1566351
120939	C12orf59	chromosome 12 open reading frame 59	0.99236	1.002843	1.0047929
121006	FAM186A	family with sequence similarity 186, member A	0.99099	1.01275	0.9709835
121014	LOC121014	HSA12cenp11 beta-tubulin 4Q (TUBB4Q) pseudogene	1.00529	0.9818	1.0258898

121053	C12orf45	chromosome 12 open reading frame 45	0.97073	0.973906	1.0074272
121054	KRT18P20	keratin 18 pseudogene 20	0.96311	0.967341	0.9695774
121129	OR2AP1	olfactory receptor, family 2, subfamily AP, member 1	1.01126	0.983311	0.946575
121130	OR10P1	olfactory receptor, family 10, subfamily P, member 1	0.9993	0.974395	0.9946296
121131	PSMB3P	proteasome (prosome, macropain) subunit, beta type, 3 pseudogene	0.9894	1.020825	1.0666825
121214	SDR9C7	short chain dehydrogenase/reductase family 9C, member 7	0.97794	0.994928	0.998798
121227	LRIG3	leucine-rich repeats and immunoglobulin-like domains 3	0.99991	0.989864	1.0168067
121256	TMEM132D	transmembrane protein 132D	1.00027	1.002998	1.0078307
121260	SLC15A4	solute carrier family 15, member 4	0.99161	1.038717	1.0195061
121268	RHEBL1	Ras homolog enriched in brain like 1	1.00818	0.967247	1.0152864
121270	OR11M1P	olfactory receptor, family 11, subfamily M, member 1 pseudogene	1.07358	1.009106	0.9841628
121273	C12orf54	chromosome 12 open reading frame 54	0.99378	0.968391	0.9999082
121274	ZNF641	zinc finger protein 641	0.90079	0.990059	0.8966638
121275	OR10AD1	olfactory receptor, family 10, subfamily AD, member 1	0.99275	1.010379	1.0214116
121278	TPH2	tryptophan hydroxylase 2	1.00149	0.993996	1.0063362
121296	LOC121296	transmembrane protein 132B pseudogene	1.02198	1.002068	1.0421194
121328	GGTA2P	glycoprotein, alpha-galactosyltransferase 2, pseudogene	1.05048	1.072034	0.9271895
121340	SP7	Sp7 transcription factor	0.99245	1.008088	1.0081117
121355	GTSF1	gametocyte specific factor 1	0.95007	0.997767	1.0322027
121360	OR9K1P	olfactory receptor, family 9, subfamily K, member 1 pseudogene	0.99543	1.015804	1.0200767
121364	OR10A7	olfactory receptor, family 10, subfamily A, member 7	1.01109	1.01563	0.9813371
121391	KRT74	keratin 74	1.01854	0.960033	0.9908484
121441	NEDD1	neural precursor cell expressed, developmentally down-regulated 1	0.91982	0.874473	1.0495719
121456	LOC121456	solute carrier family 9 (sodium/hydrogen exchanger), member 7 pseudogene	1.00639	0.932799	1.055934
121457	IKBIP	IKBKB interacting protein	0.95805	1.009679	1.0095685
121498	LOC121498	lactate dehydrogenase A-like 6B pseudogene	1.00281	0.974415	0.959139
121504	HIST4H4	histone cluster 4, H4	0.94963	0.896154	0.9880948
121506	ERP27	endoplasmic reticulum protein 27	0.98464	1.114754	0.9915785
121512	FGD4	FYVE, RhoGEF and PH domain containing 4	0.99042	1.061911	0.9833602
121520	GOT2P4	glutamic-oxaloacetic transaminase 2 pseudogene 4	1.00654	1.010049	0.9860871
121536	AEBP2	AE binding protein 2	0.94114	0.993166	1.0398348
121549	ASCL4	achaete-scute complex homolog 4 (Drosophila)	0.99343	0.943426	0.9812874
121551	BTBD11	BTB (POZ) domain containing 11	0.95757	0.952675	0.9483538

121599	SPIC	Spi-C transcription factor (Spi-1/PU.1 related)	1.02172	0.97534	1.0450555
121601	ANO4	anoctamin 4	1.00783	0.99312	1.0004755
121642	ALKBH2	alkB, alkylation repair homolog 2 (E. coli)	0.94927	0.949416	0.9942715
121643	FOXN4	forkhead box N4	0.9911	1.024156	1.0284679
121665	SPPL3	signal peptide peptidase 3	0.98247	1.014167	0.9865769
121727	PEX12P1	peroxisomal biogenesis factor 12 pseudogene 1	0.99439	0.97471	0.9516912
121793	C13orf16	chromosome 13 open reading frame 16	0.99011	1.01552	1.0018697
121838	NCRNA002	non-protein coding RNA 284	1.01199	0.998164	1.015722
121906	PSMA6P4	proteasome (prosome, macropain) subunit, alpha type, 6 pseudogene 4	0.965	0.997799	1.010988
121916	CYCSP35	cytochrome c, somatic pseudogene 35	0.96627	0.95005	1.0240765
121952	LOC121952	hypothetical LOC121952	0.96797	1.005646	1.0219412
121981	PPIAP26	peptidylprolyl isomerase A (cyclophilin A) pseudogene 26	1.05457	1.026686	0.9208218
122011	CSNK1A1L	casein kinase 1, alpha 1-like	1.0151	0.991675	1.0758383
122038	TOMM22P3	translocase of outer mitochondrial membrane 22 homolog (yeast) pseudogene 3	0.95657	1.031684	1.0834992
122042	RXFP2	relaxin/insulin-like family peptide receptor 2	0.98274	0.975299	1.0094901
122046	C13orf26	chromosome 13 open reading frame 26	1.00108	0.991882	1.0271295
122060	SLAIN1	SLAIN motif family, member 1	0.91989	0.947879	1.0035704
122145	RIOK3P1	RIO kinase 3 (yeast) pseudogene 1	0.98808	1.055841	1.002924
122199	LGMNP1	legumain pseudogene 1	1.01182	1.006378	0.9998204
122258	SPACA7	sperm acrosome associated 7	0.99922	1.000812	0.9937626
122335	PPIAP24	peptidylprolyl isomerase A (cyclophilin A) pseudogene 24	0.9978	0.983929	1.0291904
122402	TDRD9	tudor domain containing 9	0.99536	0.945414	1.0144599
122409	RPL21P12	ribosomal protein L21 pseudogene 12	0.98071	1.016713	1.0445364
122416	ANKRD9	ankyrin repeat domain 9	0.98186	1.008761	1.0149828
122449	RPS29P1	ribosomal protein S29 pseudogene 1	0.98815	0.996304	0.9982887
122481	AK7	adenylate kinase 7	0.99945	1.014877	1.0079339
122509	IFI27L1	interferon, alpha-inducible protein 27-like 1	0.99509	0.992242	0.9988293
122525	C14orf28	chromosome 14 open reading frame 28	0.90538	0.866282	0.9435294
122552	PPIAP4	peptidylprolyl isomerase A (cyclophilin A) pseudogene 4	0.96918	0.97779	1.0310015
122553	TRAPPC6B	trafficking protein particle complex 6B	0.95161	0.949461	1.0563375
122589	RPLP0P3	ribosomal protein, large, P0 pseudogene 3	0.95014	1.030065	0.9375437
122592	KRT18P6	keratin 18 pseudogene 6	0.99757	1.003835	1.0094419
122616	C14orf79	chromosome 14 open reading frame 79	0.99421	0.961575	1.0099487

122618	PLD4	phospholipase D family, member 4	0.99713	0.907877	1.0278833
122622	ADSSL1	adenylosuccinate synthase like 1	0.97838	0.985331	0.969376
122651	RNASE11	ribonuclease, RNase A family, 11 (non-active)	0.98787	0.999122	1.0348117
122664	TPPP2	tubulin polymerization-promoting protein family member 2	0.98879	0.994892	0.992017
122665	RNASE8	ribonuclease, RNase A family, 8	0.97721	1.008589	0.9831851
122704	MRPL52	mitochondrial ribosomal protein L52	0.97995	1.077475	1.0666116
122706	PSMB11	proteasome (prosome, macropain) subunit, beta type, 11	1.00587	0.988824	0.9820444
122740	OR4K14	olfactory receptor, family 4, subfamily K, member 14	0.99654	1.011392	1.0866344
122748	OR11H6	olfactory receptor, family 11, subfamily H, member 6	1.05128	1.043706	0.9265431
122769	LRR1	leucine rich repeat protein 1	0.95807	1.00399	1.0445253
122773	KLHDC1	kelch domain containing 1	0.93961	0.935429	0.9391939
122786	FRMD6	FERM domain containing 6	0.9878	1.008357	1.0111457
122809	SOCS4	suppressor of cytokine signaling 4	0.93091	0.989588	1.125
122830	NAA30	N(alpha)-acetyltransferase 30, NatC catalytic subunit	0.92841	0.94509	1.05976
122837	UBA52P3	ubiquitin A-52 residue ribosomal protein fusion product 1 pseudogene 3	1.01384	0.976617	0.9891568
122876	GPHB5	glycoprotein hormone beta 5	1.01869	1.038623	1.0025789
122888	PTBP1P	polypyrimidine tract binding protein 1 pseudogene	0.96649	0.923949	1.0801829
122942	FRDAP	Friedreich ataxia pseudogene	0.99419	0.93187	0.9762897
122945	C14orf148	chromosome 14 open reading frame 148	0.98828	1.025779	1.006333
122950	CYCSP1	cytochrome c, somatic pseudogene 1	1.01542	1.032616	0.9549199
122953	JDP2	Jun dimerization protein 2	1.00586	0.909105	0.9855214
122961	ISCA2	iron-sulfur cluster assembly 2 homolog (S. cerevisiae)	0.94264	0.949477	1.0940193
122970	ACOT4	acyl-CoA thioesterase 4	0.97647	1.015158	1.0765739
123016	TTC8	tetratricopeptide repeat domain 8	0.99384	1.000183	1.0281769
123036	TC2N	tandem C2 domains, nuclear	0.92275	0.899671	0.9469979
123041	SLC24A4	solute carrier family 24 (sodium/potassium/calcium exchanger), member 4	0.97519	0.918904	0.9900751
123096	SLC25A29	solute carrier family 25, member 29	1.01825	1.028735	0.9729984
123099	DEGS2	degenerative spermatocyte homolog 2, lipid desaturase (Drosophila)	0.99179	0.963793	0.9999569
123103	KLHL33	kelch-like 33 (Drosophila)	1.01286	1.036626	1.026581
123169	LEO1	Leo1, Paf1/RNA polymerase II complex component, homolog (S. cerevisiae)	0.91252	0.975624	1.0007614
123207	C15orf40	chromosome 15 open reading frame 40	0.94346	0.98685	0.9967208
123228	SENP8	SUMO/sentrin specific peptidase family member 8	0.97728	1.0063	1.0514358
123263	MTFMT	mitochondrial methionyl-tRNA formyltransferase	0.91457	0.980023	1.1551256

123264	OSTBETA	organic solute transporter beta	0.9749	1.016582	1.0020839
123283	TARSL2	threonyl-tRNA synthetase-like 2	0.93499	0.973367	1.0153462
123346	HIGD2B	HIG1 hypoxia inducible domain family, member 2B (pseudogene)	0.99694	1.032857	1.024047
123355	LRRC28	leucine rich repeat containing 28	0.94471	0.989524	1.0030988
123591	C15orf27	chromosome 15 open reading frame 27	1.00175	1.003117	0.9955389
123606	NIPA1	non imprinted in Prader-Willi/Angelman syndrome 1	0.98229	0.982562	0.9910256
123624	AGBL1	ATP/GTP binding protein-like 1	1.0014	0.996127	1.0053157
123688	AGPHD1	aminoglycoside phosphotransferase domain containing 1	0.98762	0.975407	0.9804818
123720	WHAMM	WAS protein homolog associated with actin, golgi membranes and microtubules	0.95502	0.999446	0.9592768
123722	FSD2	fibronectin type III and SPRY domain containing 2	1.00165	0.979906	1.0013868
123745	PLA2G4E	phospholipase A2, group IVE	1.01205	0.994973	0.9906041
123775	C16orf46	chromosome 16 open reading frame 46	0.98931	0.989946	1.0088104
123787	PRSS29P	protease, serine, 29 pseudogene	1.00115	1.004602	1.0428318
123803	NTAN1	N-terminal asparagine amidase	0.9542	0.929994	1.069927
123811	FOPNL	FGFR1OP N-terminal like	0.93425	0.908234	1.1526255
123855	LOC123855	hypothetical protein LOC123855	0.98774	0.98798	1.0347163
123862	LOC123862	interferon induced transmembrane protein pseudogene	0.94634	0.93227	0.9975566
123872	LRRC50	leucine rich repeat containing 50	1.00597	1.00753	0.9928183
123876	ACSM2A	acyl-CoA synthetase medium-chain family member 2A	1.00142	1.025512	1.0501706
123879	DCUN1D3	DCN1, defective in cullin neddylation 1, domain containing 3 (S. cerevisiae)	0.98857	1.039355	0.982968
123904	NRN1L	neuritin 1-like	0.98677	1.015236	0.9820963
123920	CMTM3	CKLF-like MARVEL transmembrane domain containing 3	0.95699	0.941667	1.012155
123970	C16orf78	chromosome 16 open reading frame 78	0.98218	1.039202	1.0290882
124044	SPATA2L	spermatogenesis associated 2-like	0.99534	0.998627	1.0665737
124045	C16orf55	chromosome 16 open reading frame 55	0.98765	1.028103	1.047702
124056	NOXO1	NADPH oxidase organizer 1	0.968	0.956497	1.0380652
124093	CCDC78	coiled-coil domain containing 78	0.99408	1.007246	1.0221043
124149	ANKRD26P	ankyrin repeat domain 26 pseudogene 1	0.99603	0.984319	1.0292464
124152	IQCK	IQ motif containing K	0.98485	0.994623	0.9971886
124199	EEF1A1P38	eukaryotic translation elongation factor 1 alpha 1 pseudogene 38	0.9735	0.946449	1.0123197
124220	ZG16B	zymogen granule protein 16 homolog B (rat)	1.01204	0.967902	1.0076454
124221	PRSS30P	protease, serine, 30 homolog (mouse), pseudogene	0.98191	0.993051	0.9775781
124222	PAQR4	progestin and adipoQ receptor family member IV	0.99013	0.964286	1.0711584

124245	ZC3H18	zinc finger CCCH-type containing 18	0.95495	0.997878	0.9919699
124274	GPR139	G protein-coupled receptor 139	0.99191	0.936165	0.9540322
124359	CDYL2	chromodomain protein, Y-like 2	0.99615	0.968343	0.9974141
124401	ANKS3	ankyrin repeat and sterile alpha motif domain containing 3	0.98214	0.991249	1.032972
124402	FAM100A	family with sequence similarity 100, member A	0.97163	1.008005	1.0540034
124404	12-Sep	septin 12	0.97948	0.971294	1.0086703
124446	TMEM219	transmembrane protein 219	0.94698	1.030054	0.9950739
124454	EARS2	glutamyl-tRNA synthetase 2, mitochondrial (putative)	0.95582	0.951667	1.0069251
124460	SNX20	sorting nexin 20	0.93339	1.008438	0.9987481
124491	TMEM170A	transmembrane protein 170A	0.90903	0.98044	1.026959
124496	ATP5F1P7	ATP synthase, H+ transporting, mitochondrial Fo complex, subunit B1 pseudogene 7	1.02891	1.065914	0.9899793
124512	METTL23	methyltransferase like 23	0.91849	0.91876	0.9760589
124535	HSF5	heat shock transcription factor family member 5	0.95681	0.973751	1.0114081
124538	OR4D2	olfactory receptor, family 4, subfamily D, member 2	0.99251	1.020571	0.9766451
124540	MSI2	musashi homolog 2 (Drosophila)	0.98477	1.018512	1.0479952
124565	SLC38A10	solute carrier family 38, member 10	0.98358	0.965053	1.0881911
124583	CANT1	calcium activated nucleotidase 1	0.95164	0.980192	1.0090174
124590	USH1G	Usher syndrome 1G (autosomal recessive)	0.98295	0.938547	1.0085736
124599	CD300LB	CD300 molecule-like family member b	0.97713	1.129434	0.8882339
124602	KIF19	kinesin family member 19	0.98926	0.997677	1.0209328
124626	ZPBP2	zona pellucida binding protein 2	0.98708	1.011336	1.0302135
124637	CYB5D1	cytochrome b5 domain containing 1	0.95018	0.990154	1.0376687
124685	LOC124685	hCG1644301	0.99355	1.032861	1.0491788
124739	USP43	ubiquitin specific peptidase 43	0.99134	0.995242	1.0244191
124751	KRBA2	KRAB-A domain containing 2	0.98034	1.060846	1.0267743
124773	C17orf64	chromosome 17 open reading frame 64	1.01166	1.011232	0.9866667
124783	C17orf46	chromosome 17 open reading frame 46	0.99151	1.005419	1.0324259
124790	HEXIM2	hexamthylene bis-acetamide inducible 2	0.97819	0.997553	1.0480987
124801	LSM12	LSM12 homolog (S. cerevisiae)	1.01946	1.027815	1.062881
124808	CCDC43	coiled-coil domain containing 43	0.9526	0.983289	1.0648192
124817	CNTD1	cyclin N-terminal domain containing 1	0.99893	0.998477	0.9941904
124842	TMEM132E	transmembrane protein 132E	0.9957	1.00469	1.0275709
124857	WFIKKN2	WAP, follistatin/kazal, immunoglobulin, kunitz and netrin domain containing 2	0.99272	0.980564	1.0163553

124865	LOC124865	mRNA turnover 4 homolog (S. cerevisiae) pseudogene	0.99839	0.970508	1.0460227
124871	FLJ40194	hypothetical FLJ40194	1.01313	1.023588	1.0001356
124872	B4GALNT2	beta-1,4-N-acetyl-galactosaminyl transferase 2	0.99328	0.976958	0.9883392
124912	SPACA3	sperm acrosome associated 3	0.99493	0.987914	1.0019061
124923	SGK494	uncharacterized serine/threonine-protein kinase SgK494	1.00339	0.99011	0.9917308
124925	SEZ6	seizure related 6 homolog (mouse)	0.99513	1.005232	0.9995315
124930	ANKRD13B	ankyrin repeat domain 13B	0.99055	1.012004	1.0900538
124935	SLC43A2	solute carrier family 43, member 2	0.99019	1.050365	0.9414109
124936	CYB5D2	cytochrome b5 domain containing 2	0.96009	0.929895	1.0275851
124944	C17orf49	chromosome 17 open reading frame 49	0.94516	0.897514	1.0356226
124961	ZFP3	zinc finger protein 3 homolog (mouse)	0.94402	0.964883	0.9823618
124974	LOC124974	thioredoxin 1 pseudogene 4	0.9764	0.999074	1.0437753
124975	GGT6	gamma-glutamyltransferase 6	0.97692	0.972454	0.9954775
124976	SPNS2	spinster homolog 2 (Drosophila)	0.98938	1.002181	1.0392648
124989	C17orf57	chromosome 17 open reading frame 57	0.95116	0.951973	1.0193838
124995	MRPL10	mitochondrial ribosomal protein L10	0.97711	0.961501	1.0284695
124997	WDR81	WD repeat domain 81	0.95196	0.901857	1.0392623
125058	TBC1D16	TBC1 domain family, member 16	0.98624	0.996595	1.0254117
125061	AFMID	arylformamidase	0.97245	0.945281	1.0400482
125111	GJD3	gap junction protein, delta 3, 31.9kDa	0.98658	0.96861	1.0885808
125113	KRT222	keratin 222	1.00032	1.039493	1.0053996
125115	KRT40	keratin 40	1.01015	0.999641	1.0084765
125144	NCRNA001	non-protein coding RNA 188	0.95348	0.957424	1.0147416
125150	ZSWIM7	zinc finger, SWIM-type containing 7	0.97009	1.002606	0.9747107
125170	SMCR7	Smith-Magenis syndrome chromosome region, candidate 7	0.97938	0.97649	1.0236335
125206	SLC5A10	solute carrier family 5 (sodium/glucose cotransporter), member 10	1.00401	0.985511	1.014289
125228	C18orf19	chromosome 18 open reading frame 19	0.9503	0.989384	0.9975357
125336	LOXHD1	lipoxygenase homology domains 1	1.00353	0.995484	1.0021028
125476	INO80C	INO80 complex subunit C	0.95876	0.943711	1.0284296
125488	TTC39C	tetratricopeptide repeat domain 39C	0.97166	0.989824	0.9841906
125704	FAM69C	family with sequence similarity 69, member C	0.99902	0.982645	1.0079785
125875	CLDND2	claudin domain containing 2	1.00022	1.078809	1.022937
125893	ZNF816	zinc finger protein 816	0.92557	0.996405	1.0029603

125919	ZNF543	zinc finger protein 543	0.91922	0.981049	1.0363526
125931	CEACAM20	carcinoembryonic antigen-related cell adhesion molecule 20	1.02543	1.002345	0.9494719
125950	RAVER1	ribonucleoprotein, PTB-binding 1	0.97184	1.000227	1.0623037
125962	OR7G1	olfactory receptor, family 7, subfamily G, member 1	1.00898	1.033468	0.9784615
125965	COX6B2	cytochrome c oxidase subunit VIb polypeptide 2 (testis)	0.98001	1.027551	1.0393751
125972	CALR3	calreticulin 3	0.99512	1.024825	1.0308639
125988	C19orf70	chromosome 19 open reading frame 70	0.94656	0.99695	1.0345443
126003	TRAPPC5	trafficking protein particle complex 5	0.96253	0.904773	1.1884653
126006	PCP2	Purkinje cell protein 2	0.98165	0.986184	0.9904722
126014	OSCAR	osteoclast associated, immunoglobulin-like receptor	0.97538	0.957817	1.0715133
126017	ZNF813	zinc finger protein 813	0.94667	0.953811	1.1141163
126037	EEF1DP1	eukaryotic translation elongation factor 1 delta pseudogene 1	0.92515	0.945661	1.0165139
126052	TGIF1P1	TGFB-induced factor homeobox 1 pseudogene 1	0.99515	1.001617	0.9462758
126068	ZNF441	zinc finger protein 441	0.89669	0.996304	1.019185
126069	ZNF491	zinc finger protein 491	0.97864	1.030031	0.9867229
126070	ZNF440	zinc finger protein 440	0.94462	0.928776	1.0735967
126074	C19orf39	chromosome 19 open reading frame 39	0.94291	0.967982	1.0606745
126075	CCDC159	coiled-coil domain containing 159	0.99316	0.982149	1.01875
126119	JOSD2	Josephin domain containing 2	0.98837	1.017589	1.0212197
126123	IZUMO2	IZUMO family member 2	0.99802	1.005764	1.0302755
126129	CPT1C	carnitine palmitoyltransferase 1C	1.00194	0.986849	0.9936468
126133	ALDH16A1	aldehyde dehydrogenase 16 family, member A1	0.96999	0.931923	1.062435
126147	NTN5	netrin 5	0.98031	1.003187	1.0473119
126170	LOC126170	peptidylprolyl isomerase A pseudogene	0.98843	1.026398	0.9288697
126204	NLRP13	NLR family, pyrin domain containing 13	0.99609	1.033037	1.01141
126205	NLRP8	NLR family, pyrin domain containing 8	1.00026	0.984953	1.01769
126206	NLRP5	NLR family, pyrin domain containing 5	0.99409	0.991596	0.9932562
126208	ZNF787	zinc finger protein 787	0.97321	0.970729	1.0384122
126231	ZNF573	zinc finger protein 573	0.96819	0.991835	1.0181789
126235	RPS4XP21	ribosomal protein S4X pseudogene 21	1.00724	1.036029	0.9646647
126242	AKR1B1P7	aldo-keto reductase family 1, member B1 pseudogene 7	0.9971	1.038738	1.012523
126248	WDR88	WD repeat domain 88	1.00349	1.002128	0.9968051
126259	TMIGD2	transmembrane and immunoglobulin domain containing 2	0.91676	0.930981	0.968373

126272	EID2B	EP300 interacting inhibitor of differentiation 2B	0.9417	0.927459	1.0550206
126282	TNFAIP8L1	tumor necrosis factor, alpha-induced protein 8-like 1	0.96985	0.983362	1.0008075
126295	ZNF57	zinc finger protein 57	0.943	1.012523	0.9973405
126298	IRGQ	immunity-related GTPase family, Q	0.98391	1.037314	0.9473403
126299	ZNF428	zinc finger protein 428	0.95894	0.974475	1.0024444
126306	JSRP1	junctional sarcoplasmic reticulum protein 1	0.98307	0.993888	1.0347037
126308	MOBKL2A	MOB1, Mps One Binder kinase activator-like 2A (yeast)	1.00793	1.026818	1.0381875
126321	C19orf28	chromosome 19 open reading frame 28	0.96723	1.002246	0.9285871
126326	GIPC3	GIPC PDZ domain containing family, member 3	0.94716	0.952543	1.0493315
126328	NDUFA11	NADH dehydrogenase (ubiquinone) 1 alpha subcomplex, 11, 14.7kDa	0.96202	0.970819	1.0954533
126353	C19orf21	chromosome 19 open reading frame 21	0.99664	1.041962	0.9835047
126364	LRRC25	leucine rich repeat containing 25	1.00988	1.058173	0.9380684
126370	OR1I1	olfactory receptor, family 1, subfamily I, member 1	1.00786	0.975621	0.891386
126374	WTIP	Wilms tumor 1 interacting protein	0.99162	1.003642	1.0417684
126375	ZNF792	zinc finger protein 792	0.87903	0.900642	0.9652685
126382	NR2C2AP	nuclear receptor 2C2-associated protein	0.92324	0.940003	1.0504173
126393	HSPB6	heat shock protein, alpha-crystallin-related, B6	0.97709	0.980861	1.0515359
126402	CCDC105	coiled-coil domain containing 105	0.99768	0.996498	1.0352921
126410	CYP4F22	cytochrome P450, family 4, subfamily F, polypeptide 22	0.97418	0.928456	1.0541162
126432	RINL	Ras and Rab interactor-like	0.95174	0.952888	1.0202896
126433	FBXO27	F-box protein 27	1.00201	1.065279	1.0130437
126520	PLK5P	polo-like kinase 5, pseudogene	0.97238	1.046738	1.0863386
126526	C19orf47	chromosome 19 open reading frame 47	0.99693	0.991269	1.0121719
126536	LOC126536	hypothetical LOC126536	1.01342	0.922605	1.0008887
126549	ANKLE1	ankyrin repeat and LEM domain containing 1	0.98747	1.044025	1.0577845
126567	C2CD4C	C2 calcium-dependent domain containing 4C	0.9891	0.984934	1.0711775
126581	MRP63P1	mitochondrial ribosomal protein 63 pseudogene 1	0.99278	0.999926	1.0451712
126626	GABPB2	GA binding protein transcription factor, beta subunit 2	0.9676	0.98792	0.967978
126637	TCHHL1	trichohyalin-like 1	1.01088	0.980975	1.0509772
126638	RPTN	repetin	1.01304	1.021962	0.9954568
126661	CCDC163P	coiled-coil domain containing 163, pseudogene	1.045	0.928365	1.0420154
126668	TDRD10	tudor domain containing 10	1.02009	1.022339	0.981146
126669	SHE	Src homology 2 domain containing E	1.01568	0.99522	1.0069773

126695	C1orf172	chromosome 1 open reading frame 172	1.01362	0.99626	1.0139841
126731	C1orf96	chromosome 1 open reading frame 96	1.02394	0.994244	1.0636832
126755	LRRC38	leucine rich repeat containing 38	1.00491	1.015427	1.0421529
126767	AADACL3	arylacetamide deacetylase-like 3	1.02249	1.032132	1.0336443
126789	PUSL1	pseudouridylate synthase-like 1	0.99176	0.96339	1.0111467
126792	B3GALT6	UDP-Gal:betaGal beta 1,3-galactosyltransferase polypeptide 6	0.96617	1.005645	1.0486431
126811	KRT8P21	keratin 8 pseudogene 21	1.00812	0.978681	1.0145072
126820	WDR63	WD repeat domain 63	1.0035	1.035252	1.0095382
126823	KLHDC9	kelch domain containing 9	1.0006	1.011502	0.9856783
126826	TGIF2P1	TGFB-induced factor homeobox 2 pseudogene 1	1.02006	1.056851	1.0195357
126859	AXDND1	axonemal dynein light chain domain containing 1	1.00817	1.007996	1.0060825
126860	LOC126860	ArfGAP with GTPase domain, ankyrin repeat and PH domain 1 pseudogene	1.02033	0.987044	1.0110824
126868	MAB21L3	mab-21-like 3 (C. elegans)	0.99611	0.969887	1.0270346
126917	IFFO2	intermediate filament family orphan 2	0.97209	1.021526	0.9030343
126969	SLC44A3	solute carrier family 44, member 3	1.00911	0.988617	1.0223176
126987	LOC126987	tight junction protein 3 (zona occludens 3) pseudogene	0.94021	1.012179	1.032872
127002	ATXN7L2	ataxin 7-like 2	0.99229	0.964886	0.994779
127003	C1orf194	chromosome 1 open reading frame 194	1.00467	0.962409	1.0049756
127011	LOC127011	ATPase, H+ transporting, lysosomal accessory protein 2 pseudogene	0.99458	0.968659	0.9623165
127018	LYPLAL1	lysophospholipase-like 1	0.96715	0.943807	0.9349653
127066	OR14C36	olfactory receptor, family 14, subfamily C, member 36	1.01996	0.950286	0.9865783
127069	OR2T10	olfactory receptor, family 2, subfamily T, member 10	0.99444	1.012832	1.0183797
127074	OR2T4	olfactory receptor, family 2, subfamily T, member 4	0.99697	0.991882	1.0108101
127086	LOC127086	X-ray repair complementing defective repair in Chinese hamster cells 6 pseudogene	0.98968	1.036703	0.9996995
127124	ATP6V1G3	ATPase, H+ transporting, lysosomal 13kDa, V1 subunit G3	1.02098	1.049816	1.0188632
127247	ASB17	ankyrin repeat and SOCS box containing 17	1.00142	1.022533	1.0235183
127253	TYW3	tRNA-yW synthesizing protein 3 homolog (S. cerevisiae)	0.97958	0.945869	1.0282075
127254	C1orf173	chromosome 1 open reading frame 173	0.99594	1.025721	1.0073387
127255	LRRIQ3	leucine-rich repeats and IQ motif containing 3	0.99377	0.983719	1.055274
127262	TPRG1L	tumor protein p63 regulated 1-like	0.93798	0.910227	1.0654077
127281	C1orf93	chromosome 1 open reading frame 93	0.98258	1.011277	1.0554398
127294	MYOM3	myomesin family, member 3	1.00642	1.003105	1.0233898
127295	RPL36P5	ribosomal protein L36 pseudogene 5	1.01227	0.956273	0.9692732

127343	DMBX1	diencephalon/mesencephalon homeobox 1	1.00712	1.000599	1.0095417
127385	OR10J5	olfactory receptor, family 10, subfamily J, member 5	0.97386	1.001292	1.0085575
127391	TMCO2	transmembrane and coiled-coil domains 2	0.97462	1.037205	1.0009518
127396	ZNF684	zinc finger protein 684	0.97941	0.984101	1.0207629
127406	RPSAP20	ribosomal protein SA pseudogene 20	0.98198	1.073994	1.0052001
127428	TCEANC2	transcription elongation factor A (SII) N-terminal and central domain containing 2	0.97747	0.961031	0.9825074
127435	PODN	podocan	1.01356	0.995835	1.0343934
127495	LRRC39	leucine rich repeat containing 39	1.00136	0.999822	0.9922628
127534	GJB4	gap junction protein, beta 4, 30.3kDa	1.01241	1.004477	1.0616105
127540	HMGB4	high mobility group box 4	1.00296	0.968703	0.9751679
127544	RNF19B	ring finger protein 19B	0.96983	1.001468	0.9897927
127550	A3GALT2P	alpha 1,3-galactosyltransferase 2, pseudogene	0.99449	1.034452	1.0248569
127579	DCST2	DC-STAMP domain containing 2	1.00472	0.992317	0.9983581
127602	DNAH14	dynein, axonemal, heavy chain 14	0.99981	1.001518	1.0091119
127608	OR2AJ1	olfactory receptor, family 2, subfamily AJ, member 1	1.04896	0.987581	1.0504239
127617	OR14L1P	olfactory receptor, family 14, subfamily L, member 1 pseudogene	0.99195	0.997375	1.0209484
127623	OR2B11	olfactory receptor, family 2, subfamily B, member 11	0.99458	0.975668	0.9845823
127665	ZNF648	zinc finger protein 648	1.01904	1.004807	1.0006914
127670	TEDDM1	transmembrane epididymal protein 1	1.01949	1.003449	0.9845411
127671	LOC127671	transmembrane epididymal protein 1-like	1.01916	0.999912	1.0226487
127687	C1orf122	chromosome 1 open reading frame 122	1.00545	1.01591	1.0380355
127700	OSCP1	organic solute carrier partner 1	1.00205	1.022615	0.9965186
127703	C1orf216	chromosome 1 open reading frame 216	1.01074	0.981419	1.069685
127707	KLHDC7A	kelch domain containing 7A	1.00602	0.971315	1.0129344
127731	VWA5B1	von Willebrand factor A domain containing 5B1	1.00257	1.034535	1.0133235
127733	UBXN10	UBX domain protein 10	0.99415	0.988203	0.9774621
127795	C1orf87	chromosome 1 open reading frame 87	0.99956	0.952331	0.9907556
127829	ARL8A	ADP-ribosylation factor-like 8A	1.02286	1.030411	0.9903577
127833	SYT2	synaptotagmin II	1.00206	1.015017	1.0173517
127845	GOLT1A	golgi transport 1A	0.99717	0.994448	1.025909
127933	UHMK1	U2AF homology motif (UHM) kinase 1	1.04274	0.934458	1.0944703
127943	FCRLB	Fc receptor-like B	0.98847	0.988548	1.0343603
128025	WDR64	WD repeat domain 64	0.99741	1.005977	0.9951708

128061	C1orf131	chromosome 1 open reading frame 131	0.99455	1.018312	1.0235696
128077	LIX1L	Lix1 homolog (mouse)-like	1.02095	0.981037	1.1301391
128136	LOC128136	3-hydroxy-3-methylglutaryl-Coenzyme A synthase 1 (soluble) pseudogene	0.99793	1.01909	1.038857
128153	SPATA17	spermatogenesis associated 17	1.01279	1.008447	1.0261218
128178	EDARADD	EDAR-associated death domain	0.9851	0.989971	1.0109322
128209	KLF17	Kruppel-like factor 17	1.00928	1.001099	1.0228544
128218	TMEM125	transmembrane protein 125	1.00432	1.034819	1.0490707
128229	C1orf182	chromosome 1 open reading frame 182	0.99188	1.037517	1.0275884
128240	APOA1BP	apolipoprotein A-I binding protein	0.96153	0.974857	1.0427486
128272	ARHGEF19	Rho guanine nucleotide exchange factor (GEF) 19	0.98572	0.982684	1.0231132
128308	MRPL55	mitochondrial ribosomal protein L55	0.96125	1.006158	0.9564793
128312	HIST3H2BB	histone cluster 3, H2bb	0.97953	0.901757	0.9191632
128322	LOC128322	nuclear transport factor 2-like	0.96497	0.933428	1.0870182
128338	DRAM2	DNA-damage regulated autophagy modulator 2	1.02578	1.032955	1.0379738
128344	C1orf88	chromosome 1 open reading frame 88	0.98952	0.997622	0.9857475
128360	OR10T2	olfactory receptor, family 10, subfamily T, member 2	1.00961	1.026402	1.0682772
128366	OR6P1	olfactory receptor, family 6, subfamily P, member 1	0.97427	1.053479	1.035666
128367	OR10X1	olfactory receptor, family 10, subfamily X, member 1	0.99178	0.985433	1.0242466
128368	OR10Z1	olfactory receptor, family 10, subfamily Z, member 1	1.04865	0.98347	1.0433729
128370	OR6K4P	olfactory receptor, family 6, subfamily K, member 4 pseudogene	1.00127	1.044533	1.0463335
128371	OR6K6	olfactory receptor, family 6, subfamily K, member 6	0.97133	0.994576	0.9576964
128372	OR6N1	olfactory receptor, family 6, subfamily N, member 1	1.02653	0.951575	1.0604848
128387	TATDN3	TatD DNase domain containing 3	0.98417	0.962503	1.0066859
128408	BHLHE23	basic helix-loop-helix family, member e23	0.9921	1.024394	1.0878944
128414	NKAIN4	Na+/K+ transporting ATPase interacting 4	0.99422	1.008909	1.0037346
128434	VSTM2L	V-set and transmembrane domain containing 2 like	0.99617	0.961635	0.9965141
128439	SNHG11	small nucleolar RNA host gene 11 (non-protein coding)	0.9642	0.994778	1.0403247
128467	RPL12P11	ribosomal protein L12 pseudogene 11	1.01132	0.960337	1.0241909
128481	RPL27AP	ribosomal protein L27a pseudogene	0.98699	0.958363	1.0109794
128486	FITM2	fat storage-inducing transmembrane protein 2	0.94878	1.000612	1.0033406
128488	WFDC12	WAP four-disulfide core domain 12	1.0056	1.014643	0.9847682
128497	C20orf165	chromosome 20 open reading frame 165	0.98574	0.989614	1.0218759
128500	RPL13P2	ribosomal protein L13 pseudogene 2	0.98675	1.013144	1.0441964

128506	C20orf123	chromosome 20 open reading frame 123	0.99639	1.007293	0.9881905
128553	TSHZ2	teashirt zinc finger homeobox 2	0.97542	0.864413	0.8751436
128602	C20orf85	chromosome 20 open reading frame 85	0.98384	0.957555	1.0125592
128609	MRPS16P	mitochondrial ribosomal protein S16 pseudogene	1.01713	0.981166	1.0228003
128611	ZNF831	zinc finger protein 831	0.94519	0.990931	0.9272016
128637	TBC1D20	TBC1 domain family, member 20	0.93107	1.030735	1.0285218
128646	SIRPD	signal-regulatory protein delta	1.01203	0.979836	0.9798317
128653	C20orf141	chromosome 20 open reading frame 141	1.00479	0.999825	0.9744843
128668	RPL7AP12	ribosomal protein L7a pseudogene 12	1.0147	1.025899	1.0329161
128674	PROKR2	prokineticin receptor 2	0.98646	0.994948	0.9995485
128676	RPS18P1	ribosomal protein S18 pseudogene 1	0.95715	0.991139	1.0017208
128710	C20orf94	chromosome 20 open reading frame 94	0.98694	0.9375	0.9773934
128718	RPS11P1	ribosomal protein S11 pseudogene 1	0.96679	0.96887	0.9597073
128745	RPLP0P1	ribosomal protein, large, P0 pseudogene 1	0.97112	1.067111	0.977999
128756	RPL15P1	ribosomal protein L15 pseudogene 1	0.99285	0.962174	1.0206793
128770	EIF4E2P1	eukaryotic translation initiation factor 4E family member 2 pseudogene 1	1.00456	1.040147	1.0290224
128774	MRPS11P1	mitochondrial ribosomal protein S11 pseudogene 1	1.01228	0.94685	1.0123267
128790	SLC25A6P1	solute carrier family 25 (mitochondrial carrier; adenine nucleotide translocator), member 6, pseudogene 1	1.02188	1.013911	0.9863981
128817	CSTL1	cystatin-like 1	1.00675	0.97716	0.9715012
128820	CST9LP1	cystatin 9-like pseudogene 1	1.03267	1.007616	0.9963209
128821	CST9L	cystatin 9-like	1.02975	0.983339	0.9484706
128822	CST9	cystatin 9 (testatin)	0.99097	0.956787	0.9866993
128826	C20orf166	chromosome 20 open reading frame 166	0.9875	1.018171	1.0278159
128848	DKKL1P1	dickkopf-like 1 pseudogene 1	1.01504	0.971568	1.0258752
128853	DUSP15	dual specificity phosphatase 15	0.98796	0.98707	1.0084078
128854	TSPY26P	testis specific protein, Y-linked 26, pseudogene	0.96624	1.01985	0.9900248
128859	BPIL3	bactericidal/permeability-increasing protein-like 3	1.01151	0.986422	0.979163
128861	C20orf71	chromosome 20 open reading frame 71	1.01658	1.016208	1.0041611
128864	C20orf144	chromosome 20 open reading frame 144	0.95875	1.020144	1.089123
128866	CHMP4B	chromatin modifying protein 4B	0.98583	1.031424	0.9979451
128869	PIGU	phosphatidylinositol glycan anchor biosynthesis, class U	0.96518	0.981633	1.019291
128872	HMGB3P1	high mobility group box 3 pseudogene 1	0.9993	1.027341	1.0129825
128876	FAM83C	family with sequence similarity 83, member C	0.99564	1.016089	1.0740826

128879	HMGB3P2	high mobility group box 3 pseudogene 2	0.98988	1.035857	1.0001485
128954	GAB4	GRB2-associated binding protein family, member 4	0.9976	1.038779	0.9492321
128977	C22orf39	chromosome 22 open reading frame 39	0.94453	0.973415	0.952591
128989	C22orf25	chromosome 22 open reading frame 25	0.99056	0.902127	1.002072
128997	SLC9A3P2	solute carrier family 9 (sodium/hydrogen exchanger), member 3 pseudogene 2	0.99372	0.933361	0.9901545
129025	ZNF280A	zinc finger protein 280A	0.99436	0.985929	1.032911
129049	SGSM1	small G protein signaling modulator 1	1.00017	0.982612	0.9836022
129080	EMID1	EMI domain containing 1	0.99105	1.003993	1.0026727
129099	CPSF1P1	cleavage and polyadenylation specific factor 1, 160kDa pseudogene 1	1.00016	0.987726	0.9547805
129138	ANKRD54	ankyrin repeat domain 54	0.97456	1.016269	0.9969051
129285	KLRAQ1	KLRAQ motif containing 1	0.96745	1.008266	1.0120437
129293	C2orf89	chromosome 2 open reading frame 89	0.92579	0.941814	0.9265978
129303	TMEM150A	transmembrane protein 150A	0.97871	0.964591	1.0321776
129401	NUP35	nucleoporin 35kDa	0.95474	0.951585	1.0228838
129402	LIN28AP1	lin-28 homolog A (C. elegans) pseudogene 1	1.01643	0.968239	0.985505
129450	TYW5	tRNA-yW synthesizing protein 5	0.98541	0.98748	1.0640288
129521	NMS	neuromedin S	1.00954	0.979909	1.0001334
129522	LOC129522	ralA binding protein 1 pseudogene	0.986	0.931516	0.9872601
129530	LYG1	lysozyme G-like 1	0.9947	0.98833	1.0571981
129531	MITD1	MIT, microtubule interacting and transport, domain containing 1	0.98007	0.983946	1.0173567
129560	LOC129560	adenosylhomocysteinase pseudogene	0.99437	0.970901	0.9852208
129563	DIS3L2	DIS3 mitotic control homolog (S. cerevisiae)-like 2	1.00024	0.922577	0.9732715
129607	CMPK2	cytidine monophosphate (UMP-CMP) kinase 2, mitochondrial	0.95718	0.951649	1.0608767
129642	MBOAT2	membrane bound O-acyltransferase domain containing 2	0.9962	0.93913	0.9935031
129656	LOC129656	CREB regulated transcription coactivator 1 pseudogene	1.01052	1.017812	1.0108498
129684	CNTNAP5	contactin associated protein-like 5	1.01081	1.00826	1.025044
129685	TAF8	TAF8 RNA polymerase II, TATA box binding protein (TBP)-associated factor, 43kDa	0.97945	0.989598	1.0581276
129787	TMEM18	transmembrane protein 18	0.99836	0.925572	1.0817335
129790	C7orf13	chromosome 7 open reading frame 13	1.01209	1.033598	1.0243442
129804	FBLN7	fibulin 7	1.01022	1.01003	0.9806372
129807	NEU4	sialidase 4	0.9919	1.022403	1.008651
129831	RBM45	RNA binding motif protein 45	0.99688	0.919851	1.071701
129841	RPSAP25	ribosomal protein SA pseudogene 25	0.9602	0.968087	1.0692781

129852	C2orf73	chromosome 2 open reading frame 73	1.02444	0.983958	1.0209923
129880	BBS5	Bardet-Biedl syndrome 5	0.98771	0.974749	1.0149225
129881	C2orf77	chromosome 2 open reading frame 77	0.99499	0.971595	0.9409571
130013	ACMSD	aminocarboxymuconate semialdehyde decarboxylase	1.02001	0.992804	0.9974555
130026	ICA1L	islet cell autoantigen 1,69kDa-like	0.99754	0.973842	0.965523
130074	FAM168B	family with sequence similarity 168, member B	1.00069	0.944777	0.9524522
130075	OR9A4	olfactory receptor, family 9, subfamily A, member 4	0.9892	0.999073	0.9863438
130106	CIB4	calcium and integrin binding family member 4	1.00299	1.002499	1.0073103
130132	RFTN2	raftlin family member 2	1.00597	1.011056	0.9973664
130162	C2orf63	chromosome 2 open reading frame 63	1.01564	0.994699	1.0587975
130195	LOC130195	heat shock 70kDa protein 8 pseudogene	0.98153	1.02606	0.9735164
130271	PLEKHH2	pleckstrin homology domain containing, family H (with MyTH4 domain) member 2	1.00728	1.008137	0.9671296
130340	AP1S3	adaptor-related protein complex 1, sigma 3 subunit	1.02796	1.046095	0.9976792
130355	C2orf76	chromosome 2 open reading frame 76	0.97984	0.94083	0.9948454
130367	SGPP2	sphingosine-1-phosphate phosphatase 2	0.99717	0.974564	0.9960276
130399	ACVR1C	activin A receptor, type IC	0.97845	0.928123	0.9325352
130429	RPSAP27	ribosomal protein SA pseudogene 27	0.96027	0.975436	1.0552518
130497	OSR1	odd-skipped related 1 (Drosophila)	0.98618	0.990049	1.0276116
130500	CISD1P1	CDGSH iron sulfur domain 1 pseudogene 1	1.03675	1.021954	1.0958385
130502	TTC32	tetratricopeptide repeat domain 32	0.9804	0.949763	0.9270916
130507	UBR3	ubiquitin protein ligase E3 component n-recognin 3 (putative)	0.997	0.874644	1.0660875
130535	KCTD18	potassium channel tetramerisation domain containing 18	1.0042	0.984223	0.9737839
130540	ALS2CR12	amyotrophic lateral sclerosis 2 (juvenile) chromosome region, candidate 12	0.99659	0.960494	0.9923028
130557	ZNF513	zinc finger protein 513	1.01465	0.977284	0.9857637
130560	SPATA3	spermatogenesis associated 3	1.00664	1.024516	1.0253357
130574	LYPD6	LY6/PLAUR domain containing 6	1.01152	0.999355	1.0220472
130576	LYPD6B	LY6/PLAUR domain containing 6B	1.01681	1.004833	1.0064491
130589	GALM	galactose mutarotase (aldose 1-epimerase)	0.97684	0.987202	0.9751948
130612	TMEM198	transmembrane protein 198	0.99632	1.006619	1.021286
130617	ZFAND2B	zinc finger, AN1-type domain 2B	0.96256	0.955091	1.0007317
130678	RPLP1P5	ribosomal protein, large, P1 pseudogene 5	1.01197	1.039575	0.9470253
130700	PSMB3P2	proteasome (prosome, macropain) subunit, beta type, 3 pseudogene 2	1.00326	1.00811	1.0384685
130728	RPL7P13	ribosomal protein L7 pseudogene 13	1.00735	1.014341	1.0078938

130733	TMEM178	transmembrane protein 178	1.0155	1.015399	1.0549183
130749	CPO	carboxypeptidase O	1.01169	1.005831	0.9901772
130752	MDH1B	malate dehydrogenase 1B, NAD (soluble)	1.00204	0.982367	1.0355496
130802	DNMT3AP1	DNA methyltransferase 3A pseudogene 1	1.01938	1.006121	1.0260139
130813	C2orf50	chromosome 2 open reading frame 50	0.97137	0.990411	0.9940707
130814	PQLC3	PQ loop repeat containing 3	1.00749	0.969046	0.979132
130827	TMEM182	transmembrane protein 182	1.015	0.974518	1.0121446
130865	RPL26P13	ribosomal protein L26 pseudogene 13	1.0124	0.998343	1.1243769
130872	AHSA2	AHA1, activator of heat shock 90kDa protein ATPase homolog 2 (yeast)	1.04812	0.990983	0.9542239
130888	FBXO36	F-box protein 36	0.98406	0.995942	1.0287157
130916	MTERFD2	MTERF domain containing 2	0.98518	0.960899	1.0179211
130940	CCDC148	coiled-coil domain containing 148	1.00267	1.007959	1.0124435
130951	C2orf65	chromosome 2 open reading frame 65	1.00365	1.008403	1.0081301
131034	CPNE4	copine IV	1.00554	0.985536	1.0063573
131054	LOC131054	ralA binding protein 1 pseudogene	1.00366	0.967168	1.0365613
131076	CCDC58	coiled-coil domain containing 58	0.99451	0.978002	1.0208205
131096	KCNH8	potassium voltage-gated channel, subfamily H (eag-related), member 8	0.9988	0.998964	0.9886282
131118	DNAJC19	DnaJ (Hsp40) homolog, subfamily C, member 19	0.96427	0.923873	0.9668607
131149	OTOL1	otolin 1	0.9857	0.984769	0.9445292
131177	FAM3D	family with sequence similarity 3, member D	0.98864	1.014204	1.0055249
131185	LOC131185	RAD23 homolog B (S. cerevisiae) pseudogene	0.96578	0.989861	1.0076583
131368	ZPLD1	zona pellucida-like domain containing 1	1.02129	1.021034	1.0046935
131375	LYZL4	lysozyme-like 4	1.02731	0.978275	0.9547964
131377	KBTBD5	kelch repeat and BTB (POZ) domain containing 5	1.02708	0.988219	1.0064404
131405	TRIM71	tripartite motif containing 71	1.00282	1.010265	1.0224981
131408	FAM131A	family with sequence similarity 131, member A	1.00653	0.991196	1.0210167
131450	CD200R1	CD200 receptor 1	0.94158	0.964998	1.0777929
131474	CHCHD4	coiled-coil-helix-coiled-coil-helix domain containing 4	0.99693	0.970526	0.9959299
131540	ZDHHC19	zinc finger, DHHC-type containing 19	1.00544	1.005646	1.012438
131544	CRYBG3	beta-gamma crystallin domain containing 3	0.98619	0.910479	1.0212998
131566	DCBLD2	discoidin, CUB and LCCL domain containing 2	0.99279	0.977642	1.0034031
131572	MESTP4	mesoderm specific transcript homolog (mouse) pseudogene 4	0.97116	1.005677	1.0196889
131578	LRRC15	leucine rich repeat containing 15	1.02492	1.034911	1.0286791

131583	FAM43A	family with sequence similarity 43, member A	0.94575	0.938384	1.096319
131601	TPRA1	transmembrane protein, adipocyte asscociated 1	0.98612	0.986221	1.0147
131616	TMEM42	transmembrane protein 42	0.96435	0.931369	1.0614249
131669	UROC1	urocanase domain containing 1	1.00669	1.007284	1.0178075
131831	FAM194A	family with sequence similarity 194, member A	1.0085	1.010351	1.0206316
131870	NUDT16	nudix (nucleoside diphosphate linked moiety X)-type motif 16	0.99854	0.924893	1.1167414
131873	COL6A6	collagen, type VI, alpha 6	0.99292	1.020494	1.0181077
131890	GRK7	G protein-coupled receptor kinase 7	1.00328	1.021544	1.0092554
131909	FAM172BP	family with sequence similarity 172, member B pseudogene	1.01086	1.034614	1.0001281
131920	TMEM207	transmembrane protein 207	0.9993	0.973481	0.9747298
131961	IMPDH1P8	IMP (inosine monophosphate) dehydrogenase 1 pseudogene 8	0.96435	0.971099	0.9602101
131965	METTL6	methyltransferase like 6	0.99879	0.967544	0.978643
131973	LOC131973	rhomboid 5 homolog 1 (Drosophila) pseudogene	0.99096	1.001397	1.0662466
132001	C3orf31	chromosome 3 open reading frame 31	1.00081	0.979372	1.0319384
132014	IL17RE	interleukin 17 receptor E	0.99495	0.978597	0.9914348
132112	RTP1	receptor (chemosensory) transporter protein 1	1.01868	1.031788	0.9987971
132141	IQCF1	IQ motif containing F1	1.0036	1.012511	0.9815527
132158	GLYCTK	glycerate kinase	0.9866	0.970408	1.0044274
132160	PPM1M	protein phosphatase, Mg2+/Mn2+ dependent, 1M	1.01717	1.017093	0.9972947
132200	C3orf49	chromosome 3 open reading frame 49	1.00164	1.032182	1.0073665
132203	SNTN	sentan, cilia apical structure protein	1.05309	1.016327	1.0015045
132205	[No Symbo	[No Name]	1.00204	1.01729	1.0115392
132228	C3orf45	chromosome 3 open reading frame 45	0.99469	1.004364	1.0207239
132241	RPL32P3	ribosomal protein L32 pseudogene 3	0.99627	0.932052	1.1208268
132243	H1FOO	H1 histone family, member O, oocyte-specific	1.0142	1.030986	1.0243837
132299	OCIAD2	OCIA domain containing 2	0.9741	0.941766	1.0233323
132320	SCLT1	sodium channel and clathrin linker 1	0.99454	0.95532	1.0166217
132321	C4orf33	chromosome 4 open reading frame 33	1.03215	1.00357	0.9757463
132332	TMEM155	transmembrane protein 155	1.01144	0.980608	1.002172
132386	LOC132386	tumor-associated calcium signal transducer 1 pseudogene	0.9508	1.019143	1.0617479
132391	KRT18P21	keratin 18 pseudogene 21	0.99275	0.994676	1.034798
132430	PABPC4L	poly(A) binding protein, cytoplasmic 4-like	1.01317	0.996079	0.9608544
132612	ADAD1	adenosine deaminase domain containing 1 (testis-specific)	0.99747	0.98672	1.0200009

132625	ZFP42	zinc finger protein 42 homolog (mouse)	1.00303	0.992462	1.0220058
132660	LIN54	lin-54 homolog (C. elegans)	0.9801	1.017949	1.0131922
132671	SPATA18	spermatogenesis associated 18 homolog (rat)	0.99991	1.00037	0.9977612
132719	LOC132719	tubulin, beta 8 pseudogene	1.01562	1.046354	1.0279584
132720	C4orf32	chromosome 4 open reading frame 32	1.0213	0.966581	0.9700895
132724	TMPRSS11I	transmembrane protease, serine 11B	1.00421	1.003046	1.0213067
132789	GNPDA2	glucosamine-6-phosphate deaminase 2	0.9637	0.990375	1.0801081
132817	LOC132817	chromosome 7 open reading frame 64 pseudogene	1.0059	0.996467	1.0194649
132851	SPATA4	spermatogenesis associated 4	1.00706	0.967039	1.0002735
132864	CPEB2	cytoplasmic polyadenylation element binding protein 2	0.98206	0.946509	0.9715627
132884	EVC2	Ellis van Creveld syndrome 2	0.999	1.008124	1.0129325
132946	ARL9	ADP-ribosylation factor-like 9	0.99549	0.990655	1.0043334
132949	AASDH	aminoadipate-semialdehyde dehydrogenase	0.98564	0.99622	1.0490285
132954	PDCL2	phosducin-like 2	0.99797	1.028648	0.9969975
132989	C4orf36	chromosome 4 open reading frame 36	0.98938	0.980577	1.0245292
133015	PACRGL	PARK2 co-regulated-like	1.00061	0.913104	1.0456914
133022	TRAM1L1	translocation associated membrane protein 1-like 1	0.99147	1.054795	1.0174062
133060	OTOP1	otopetrin 1	0.97523	1.002261	1.0189595
133083	LOC133083	peptidase (mitochondrial processing) alpha pseudogene	1.00534	1.031194	1.0409148
133121	ENPP6	ectonucleotide pyrophosphatase/phosphodiesterase 6	0.99072	1.007391	1.0204331
133150	SULT1D1P	sulfotransferase family, cytosolic, 1D, member 1, pseudogene	1.00158	1.030563	1.0036933
133308	NHEDC2	Na+/H+ exchanger domain containing 2	0.99438	0.992931	0.9662921
133332	LOC133332	mitochondrial ribosomal protein S5 pseudogene	1.02831	1.053224	0.9876533
133383	C5orf35	chromosome 5 open reading frame 35	0.95629	0.988706	1.0067464
133396	IL31RA	interleukin 31 receptor A	1.00187	1.059766	0.9419922
133418	EMB	embigin	0.98677	1.015517	1.0172596
133482	SLCO6A1	solute carrier organic anion transporter family, member 6A1	1.01246	1.002715	1.0245878
133491	C5orf47	chromosome 5 open reading frame 47	0.98921	0.980336	1.020334
133522	PPARGC1B	peroxisome proliferator-activated receptor gamma, coactivator 1 beta	0.99951	0.964043	1.0519053
133558	HEATR7B2	HEAT repeat family member 7B2	1.01044	1.006205	1.008885
133569	RPS10P12	ribosomal protein S10 pseudogene 12	1.00444	0.963973	0.9686614
133584	EGFLAM	EGF-like, fibronectin type III and laminin G domains	1.00639	0.995805	1.0017052
133609	RPLP1P7	ribosomal protein, large, P1 pseudogene 7	0.9765	1.064824	0.9587612

133615	MRPS5P3	mitochondrial ribosomal protein S5 pseudogene 3	0.99983	0.996052	1.0516003
133619	PRRC1	proline-rich coiled-coil 1	1.02222	1.028141	1.0909855
133629	RPL11P2	ribosomal protein L11 pseudogene 2	0.93259	0.950362	1.0002667
133686	C5orf33	chromosome 5 open reading frame 33	0.98947	1.007938	0.8995799
133688	UGT3A1	UDP glycosyltransferase 3 family, polypeptide A1	1.01371	1.001416	1.0175835
133690	CAPSL	calcyphosine-like	1.00501	0.972892	1.0328083
133746	JMY	junction mediating and regulatory protein, p53 cofactor	1.0133	0.932788	0.9174195
133748	RPL7AP32	ribosomal protein L7a pseudogene 32	1.00387	1.01833	1.0126923
133789	LOC13378[illegible]	RAB5C, member RAS oncogene family pseudogene	1.01326	0.988336	0.9812769
133790	RPS18P7	ribosomal protein S18 pseudogene 7	0.99547	1.001289	0.9809659
133874	C5orf58	chromosome 5 open reading frame 58	0.98534	0.993526	0.9937989
133923	ZNF474	zinc finger protein 474	1.0263	1.018296	1.0254361
133957	CCDC127	coiled-coil domain containing 127	0.94423	0.933561	1.0350402
134008	MRPL11P2	mitochondrial ribosomal protein L11 pseudogene 2	0.99437	0.994538	1.0780245
134082	OR2AI1P	olfactory receptor, family 2, subfamily AI, member 1 pseudogene	0.99666	0.987501	0.9869641
134083	OR2Y1	olfactory receptor, family 2, subfamily Y, member 1	1.02073	0.980496	0.981409
134111	UBE2QL1	ubiquitin-conjugating enzyme E2Q family-like 1	1.0039	0.969886	1.0178146
134121	C5orf49	chromosome 5 open reading frame 49	1.01814	1.042437	0.9881946
134145	FAM173B	family with sequence similarity 173, member B	0.97664	0.953954	1.0214855
134147	CMBL	carboxymethylenebutenolidase homolog (Pseudomonas)	1.01391	1.00475	1.0235999
134218	DNAJC21	DnaJ (Hsp40) homolog, subfamily C, member 21	0.95889	0.897335	1.0031386
134265	AFAP1L1	actin filament associated protein 1-like 1	1.00918	0.992882	0.9995434
134266	GRPEL2	GrpE-like 2, mitochondrial (E. coli)	1.01622	0.983261	1.0196947
134285	TMEM171	transmembrane protein 171	0.98883	0.999399	1.0102582
134288	TMEM174	transmembrane protein 174	1.0174	1.014468	0.9827825
134353	LSM11	LSM11, U7 small nuclear RNA associated	0.98168	0.982441	1.0379804
134359	POC5	POC5 centriolar protein homolog (Chlamydomonas)	0.99019	0.977295	1.0389556
134391	GPR151	G protein-coupled receptor 151	0.99897	0.985349	1.082982
134429	STARD4	StAR-related lipid transfer (START) domain containing 4	1.00878	0.990304	1.0240523
134430	WDR36	WD repeat domain 36	0.95965	0.875653	1.1119299
134466	ZNF300P1	zinc finger protein 300 pseudogene 1	0.9938	1.01288	1.0336661
134490	MRP63P6	mitochondrial ribosomal protein 63 pseudogene 6	1.01095	0.943866	1.0098342
134492	NUDCD2	NudC domain containing 2	0.97419	0.874552	1.0235825

134505	LOC134505	eukaryotic translation initiation factor 3 subunit K pseudogene	1.00429	1.048502	1.0797365
134510	UBLCP1	ubiquitin-like domain containing CTD phosphatase 1	0.98259	0.903788	0.9509895
134526	ACOT12	acyl-CoA thioesterase 12	1.00493	1.009141	1.0009371
134548	ANKRD43	ankyrin repeat domain 43	1.00079	1.003877	1.0660714
134549	SHROOM1	shroom family member 1	0.99619	1.007253	1.0269553
134553	C5orf24	chromosome 5 open reading frame 24	0.99714	1.011543	0.9835945
134637	ADAT2	adenosine deaminase, tRNA-specific 2	1.02029	0.897144	0.9980856
134701	RIPPLY2	ripply2 homolog (zebrafish)	0.99261	0.982001	1.0564416
134728	IRAK1BP1	interleukin-1 receptor-associated kinase 1 binding protein 1	0.98969	0.936979	1.0273204
134829	CLVS2	clavesin 2	0.99005	1.023198	0.999411
134860	TAAR9	trace amine associated receptor 9 (gene/pseudogene)	1.03777	0.997992	0.941503
134864	TAAR1	trace amine associated receptor 1	0.97392	1.007185	1.0614287
134957	STXBP5	syntaxin binding protein 5 (tomosyn)	0.98153	0.959176	0.9655148
135112	NCOA7	nuclear receptor coactivator 7	1.00384	1.023223	1.0445284
135114	HINT3	histidine triad nucleotide binding protein 3	0.99282	0.97184	1.0683196
135138	PACRG	PARK2 co-regulated	0.99877	0.996211	1.0005768
135152	B3GAT2	beta-1,3-glucuronyltransferase 2 (glucuronosyltransferase S)	0.99895	0.999817	1.019268
135154	C6orf57	chromosome 6 open reading frame 57	1.01793	1.009883	1.0391543
135228	CD109	CD109 molecule	0.99541	1.025184	0.9911288
135250	RAET1E	retinoic acid early transcript 1E	1.02767	1.018785	1.0401788
135270	COPS5P	COP9 constitutive photomorphogenic homolog subunit 5 (Arabidopsis) pseudogene	0.9943	1.006055	0.9935906
135293	PM20D2	peptidase M20 domain containing 2	0.98568	0.86566	0.9418587
135295	SRSF12	serine/arginine-rich splicing factor 12	1.00159	1.010314	0.9829417
135398	C6orf141	chromosome 6 open reading frame 141	1.00093	1.001445	0.9887043
135644	TRIM40	tripartite motif containing 40	0.95473	0.949716	1.0178083
135656	DPCR1	diffuse panbronchiolitis critical region 1	1.00836	0.988031	1.007923
135886	WBSCR28	Williams-Beuren syndrome chromosome region 28	1.00895	1.006535	1.0419571
135892	TRIM50	tripartite motif containing 50	1.02064	0.982279	1.059456
135924	OR9A2	olfactory receptor, family 9, subfamily A, member 2	1	1.043694	1.0189594
135927	C7orf34	chromosome 7 open reading frame 34	1.01217	1.001735	0.9915445
135932	TMEM139	transmembrane protein 139	1.00336	0.950294	0.9827167
135935	NOBOX	NOBOX oogenesis homeobox	1.01889	0.98811	0.9762076
135941	OR2A14	olfactory receptor, family 2, subfamily A, member 14	0.97723	1.049636	1.0601998

135942	OR2A15P	olfactory receptor, family 2, subfamily A, member 15 pseudogene	0.98837	1.044461	0.9890708
135946	OR6B1	olfactory receptor, family 6, subfamily B, member 1	1.02817	1.039478	0.9230997
135948	OR2F2	olfactory receptor, family 2, subfamily F, member 2	1.00341	0.983276	1.0502426
136051	ZNF786	zinc finger protein 786	0.97684	0.956928	0.9691905
136227	EMID2	EMI domain containing 2	1.01371	1.010956	1.0172098
136259	KLF14	Kruppel-like factor 14	0.98296	1.048785	1.0442996
136263	C7orf45	chromosome 7 open reading frame 45	0.99699	0.97975	0.9965833
136288	C7orf57	chromosome 7 open reading frame 57	1.01911	0.996984	1.0026696
136306	SVOPL	SVOP-like	1.0069	0.994179	1.0192308
136307	IMPDH1P3	IMP (inosine monophosphate) dehydrogenase 1 pseudogene 3	0.98103	1.020814	1.0024302
136319	MTPN	myotrophin	1.01018	0.972002	1.0148993
136332	LRGUK	leucine-rich repeats and guanylate kinase domain containing	1.00973	0.977434	0.9838015
136371	ASB10	ankyrin repeat and SOCS box containing 10	1.0076	1.024252	1.0411937
136540	TRY3	trypsinogen-like pseudogene	1.01702	1.03497	1.0430036
136541	PRSS58	protease, serine, 58	1.02003	1.022165	1.028061
136542	[No Symbo	[No Name]	1.00805	0.977127	1.0073232
136647	C7orf11	chromosome 7 open reading frame 11	0.97433	0.905807	1.0110452
136853	SRCRB4D	scavenger receptor cysteine rich domain containing, group B (4 domains)	1.00123	1.01545	1.0124965
136895	C7orf31	chromosome 7 open reading frame 31	0.96662	0.921599	0.9698231
136991	ASZ1	ankyrin repeat, SAM and basic leucine zipper domain containing 1	0.98769	0.993249	0.9924005
137012	PPM1AP1	protein phosphatase, Mg2+/Mn2+ dependent 1A, pseudogene 1	1.00425	0.974553	1.0096042
137075	CLDN23	claudin 23	0.99639	1.003009	1.0699581
137107	RPL10AP3	ribosomal protein L10a pseudogene 3	0.99278	0.959095	0.9694569
137133	PSAT1P1	phosphoserine aminotransferase 1 pseudogene 1	0.98281	1.0786	1.0292175
137209	ZNF572	zinc finger protein 572	0.98887	1.011439	0.9601419
137290	MRPL9P1	mitochondrial ribosomal protein L9 pseudogene 1	1.01887	0.956976	0.9843062
137362	GOT1L1	glutamic-oxaloacetic transaminase 1-like 1	0.98658	1.008941	1.0040978
137392	FAM92A1	family with sequence similarity 92, member A1	1.01002	1.011202	0.9998081
137406	MRPS16P1	mitochondrial ribosomal protein S16 pseudogene 1	0.98943	0.931114	1.0572751
137492	VPS37A	vacuolar protein sorting 37 homolog A (S. cerevisiae)	0.99695	1.019462	1.0682925
137682	C8orf38	chromosome 8 open reading frame 38	0.97599	0.93608	0.9925291
137695	TMEM68	transmembrane protein 68	0.96225	0.999693	1.0625973
137735	ABRA	actin-binding Rho activating protein	1.01433	0.998558	0.9981976

137797	LYPD2	LY6/PLAUR domain containing 2	1.0341	0.939788	1.0481305
137814	NKX2-6	NK2 homeobox 6	0.99823	0.974184	1.0321904
137835	TMEM71	transmembrane protein 71	0.91785	1.12821	0.9713341
137868	SGCZ	sarcoglycan, zeta	0.99936	1.004825	1.0138441
137872	ADHFE1	alcohol dehydrogenase, iron containing, 1	1.00946	0.977453	0.9965041
137886	UBXN2B	UBX domain protein 2B	1.0058	0.985585	0.9768982
137902	PXDNL	peroxidasin homolog (Drosophila)-like	0.99821	0.997709	0.9960703
137964	AGPAT6	1-acylglycerol-3-phosphate O-acyltransferase 6 (lysophosphatidic acid acyltransferase, zeta)	1.03927	1.001038	0.9683407
137970	UNC5D	unc-5 homolog D (C. elegans)	1.00878	0.992959	0.9996224
137994	LETM2	leucine zipper-EF-hand containing transmembrane protein 2	1.01044	0.993047	0.9621001
138009	DCAF4L2	DDB1 and CUL4 associated factor 4-like 2	1.00167	1.015236	1.0148908
138046	RALYL	RALY RNA binding protein-like	1.01294	1.005119	1.0098744
138050	HGSNAT	heparan-alpha-glucosaminide N-acetyltransferase	0.99575	1.050287	0.9278693
138065	RNF183	ring finger protein 183	1.01383	1.010829	1.0030395
138151	NACC2	NACC family member 2, BEN and BTB (POZ) domain containing	0.99234	0.981498	1.0365101
138159	LOC138159	beta-lactoglobulin pseudogene	0.99409	0.986074	1.0266029
138162	C9orf116	chromosome 9 open reading frame 116	0.98618	0.994373	0.991884
138199	C9orf41	chromosome 9 open reading frame 41	0.96648	0.990388	1.0206406
138240	C9orf57	chromosome 9 open reading frame 57	1.00068	1.003185	1.0177217
138241	C9orf85	chromosome 9 open reading frame 85	0.97723	1.015961	1.0379242
138255	C9orf135	chromosome 9 open reading frame 135	0.99564	0.968847	1.0259574
138307	LCN8	lipocalin 8	0.9943	0.994422	1.0250215
138311	FAM69B	family with sequence similarity 69, member B	0.96937	0.987769	1.0411077
138428	PTRH1	peptidyl-tRNA hydrolase 1 homolog (S. cerevisiae)	0.96998	0.92566	1.0142563
138429	PIP5KL1	phosphatidylinositol-4-phosphate 5-kinase-like 1	0.97229	1.003538	1.0482458
138474	TAF1L	TAF1 RNA polymerase II, TATA box binding protein (TBP)-associated factor, 210kDa-like	0.97429	0.995854	1.015478
138639	PTPDC1	protein tyrosine phosphatase domain containing 1	0.98674	0.999728	1.0353686
138649	ANKRD19P	ankyrin repeat domain 19, pseudogene	0.98225	0.953625	0.9735525
138652	PRSS47	protease, serine, 47	0.99169	0.997251	1.0036719
138715	ARID3C	AT rich interactive domain 3C (BRIGHT-like)	0.98241	1.041045	0.9926396
138716	C9orf23	chromosome 9 open reading frame 23	0.94123	0.973325	0.9723477
138724	C9orf131	chromosome 9 open reading frame 131	0.99386	1.033655	0.9551091
138799	OR13C5	olfactory receptor, family 13, subfamily C, member 5	1.02589	1.009444	0.931344

138802	OR13C8	olfactory receptor, family 13, subfamily C, member 8	1.00535	1.01537	1.0729673
138803	OR13C3	olfactory receptor, family 13, subfamily C, member 3	0.99178	1.052655	0.9390766
138805	OR13F1	olfactory receptor, family 13, subfamily F, member 1	1.03391	1.002371	0.9198466
138864	LOC138864	ATP synthase, H+ transporting, mitochondrial Fo complex, subunit d pseudogene	1.00382	1.010296	0.9922689
138882	OR1N2	olfactory receptor, family 1, subfamily N, member 2	0.99466	0.973624	0.9804809
138916	SYNGR2P2	synaptogyrin 2 pseudogene 2	0.93599	0.946797	1.0928125
138948	NCRNA001	non-protein coding RNA 118	0.97173	1.018207	1.0022584
138971	LOC138971	dipeptidyl-peptidase 3 pseudogene	0.98174	0.997083	1.0673204
139060	LOC139060	keratin, type I cytoskeletal 18-like	1.03125	0.946819	0.9854502
139061	LOC139061	plasmolipin pseudogene	0.99056	1.048324	1.0368296
139065	SLITRK4	SLIT and NTRK-like family, member 4	1.01074	0.939103	0.9867393
139067	SPANXN3	SPANX family, member N3	1.03425	0.999229	0.9725008
139105	BEND2	BEN domain containing 2	0.85902	1.004668	0.9855611
139135	PASD1	PAS domain containing 1	0.99234	0.996647	1.0125523
139163	SALL1P1	sal-like 1 (Drosophila) pseudogene 1	0.99855	0.967826	0.984387
139170	DCAF12L1	DDB1 and CUL4 associated factor 12-like 1	0.9965	0.98669	1.0284324
139189	DGKK	diacylglycerol kinase, kappa	0.98837	0.98866	0.9873442
139201	LOC139201	mitogen-activated protein kinase kinase 4 pseudogene	1.00513	1.04173	1.0653664
139221	MUM1L1	melanoma associated antigen (mutated) 1-like 1	1.01144	1.031483	1.0106917
139231	FAM199X	family with sequence similarity 199, X-linked	0.94396	0.990985	0.9893025
139249	FAM47DP	family with sequence similarity 47, member D (pseudogene)	1.00335	0.993151	0.9621607
139285	FAM123B	family with sequence similarity 123B	0.97589	0.91496	1.020265
139302	[No Symbo	[No Name]	1.0771	1.073493	1.0728985
139322	APOOL	apolipoprotein O-like	0.96076	0.953986	1.0320714
139334	IMPDH1P4	IMP (inosine monophosphate) dehydrogenase 1 pseudogene 4	1.00101	1.025825	1.0162294
139341	FUNDC1	FUN14 domain containing 1	0.9384	0.945109	1.0683775
139363	MAGEA13P	melanoma antigen family A, 13 pseudogene	0.98155	1.029841	1.0142677
139378	GPR112	G protein-coupled receptor 112	1.00083	1.025593	1.0116218
139411	PTCHD1	patched domain containing 1	1.01177	0.997598	1.0104311
139420	SMEK3P	SMEK homolog 3, suppressor of mek1 (Dictyostelium) pseudogene	0.99885	0.990179	0.9833401
139422	MAGEB10	melanoma antigen family B, 10	0.97987	0.985889	0.9865495
139425	DCAF8L1	DDB1 and CUL4 associated factor 8-like 1	1.00792	1.025166	1.0174644
139431	FTH1P14	ferritin, heavy polypeptide 1 pseudogene 14	1.00623	0.993326	1.0173902

139452	RPL6P30	ribosomal protein L6 pseudogene 30	0.95135	0.966052	1.0785153
139466	[No Symbo	[No Name]	0.97932	1.024674	1.0048297
139516	AKAP16BP	A kinase (PRKA) anchor protein 16B, pseudogene	0.99162	0.997582	1.0186246
139562	OTUD6A	OTU domain containing 6A	0.98333	1.023523	0.9779679
139596	UPRT	uracil phosphoribosyltransferase (FUR1) homolog (S. cerevisiae)	0.93393	0.923072	0.9840322
139599	MAGEE2	melanoma antigen family E, 2	1.00073	0.988042	1.0115736
139604	MAGEB16	melanoma antigen family B, 16	1.0167	0.958588	0.9950365
139628	FOXR2	forkhead box R2	0.99575	1.023041	1.0066204
139629	XAGE-4	XAGE-4 protein	1.02512	1.053368	0.9851448
139716	GAB3	GRB2-associated binding protein 3	0.9528	1.098815	0.9525085
139728	PNCK	pregnancy up-regulated non-ubiquitously expressed CaM kinase	0.99668	1.037331	1.0482237
139735	ZFP92	zinc finger protein 92 homolog (mouse)	1.00216	1.011775	0.9953977
139741	ACTRT1	actin-related protein T1	1.00227	0.966773	0.9885466
139748	KRT18P44	keratin 18 pseudogene 44	0.99609	1.003322	0.9959533
139760	GPR119	G protein-coupled receptor 119	0.99018	1.028279	1.0027417
139793	PAGE3	P antigen family, member 3 (prostate associated)	0.99205	1.061503	0.9903012
139804	RBMXL3	RNA binding motif protein, X-linked-like 3	0.99593	0.988401	1.0014702
139818	DOCK11	dedicator of cytokinesis 11	0.95961	0.94968	0.9039342
139886	SPIN4	spindlin family, member 4	0.95487	0.994548	1.0732127
139952	LOC139952	LIM domain kinase 2 pseudogene	1.02361	1.002563	1.0044291
139957	LOC139957	platelet-activating factor acetylhydrolase, isoform Ib, beta subunit 30kDa pseudogene	0.97164	0.95592	1.0515162
140028	GAPDHP19	glyceraldehyde 3 phosphate dehydrogenase pseudogene 19	0.9734	1.032346	0.9765101
140032	RPS4Y2	ribosomal protein S4, Y-linked 2	0.9985	1.015324	1.022752
140043	CDY10P	chromodomain protein, Y-linked 10 pseudogene	1.02089	0.983735	0.9883019
140100	RBMY2KP	RNA binding motif protein, Y-linked, family 2, member K pseudogene	1.0064	0.990455	0.9626402
140101	RBMY2MP	RNA binding motif protein, Y-linked, family 2, member M pseudogene	1.00106	1.062171	1.0364561
140258	KRTAP13-1	keratin associated protein 13-1	1.0226	0.942322	1.0505356
140290	TCP10L	t-complex 10 (mouse)-like	0.9872	1.064382	1.0146777
140432	RNF113B	ring finger protein 113B	0.96743	1.014984	1.0107132
140453	MUC17	mucin 17, cell surface associated	1.01257	0.996599	1.0346113
140456	ASB11	ankyrin repeat and SOCS box containing 11	0.98752	0.997817	0.9842225
140458	ASB5	ankyrin repeat and SOCS box containing 5	0.9902	1.018952	0.9999564
140459	ASB6	ankyrin repeat and SOCS box containing 6	0.95114	0.94787	1.0516547

140460	ASB7	ankyrin repeat and SOCS box containing 7	0.96634	1.023912	1.046793
140461	ASB8	ankyrin repeat and SOCS box containing 8	0.96475	0.972164	0.9229991
140462	ASB9	ankyrin repeat and SOCS box containing 9	1	1.049222	1.0052035
140465	MYL6B	myosin, light chain 6B, alkali, smooth muscle and non-muscle	0.9875	0.978016	1.0157495
140467	ZNF358	zinc finger protein 358	0.96892	0.996763	1.017438
140468	COX11P1	COX11 homolog, cytochrome c oxidase assembly protein (yeast) pseudogene 1	1.07524	1.07894	0.9480148
140469	MYO3B	myosin IIIB	1.00179	1.015906	1.0269676
140545	RNF32	ring finger protein 32	1.00962	1.007873	1.0223983
140564	APOBEC3D	apolipoprotein B mRNA editing enzyme, catalytic polypeptide-like 3D	0.97528	1.011028	0.9848988
140565	[No Symbo	[No Name]	0.99507	0.982327	0.9544319
140576	S100A16	S100 calcium binding protein A16	1.0254	1.023174	1.0262242
140578	CHODL	chondrolectin	0.99228	0.99892	0.9797701
140597	TCEAL2	transcription elongation factor A (SII)-like 2	0.98996	0.983134	0.9724265
140603	COX6B1P1	cytochrome c oxidase subunit VIb polypeptide 1 (ubiquitous) pseudogene 1	0.9806	0.971867	1.051281
140606	SELM	selenoprotein M	0.94491	0.952353	0.9503081
140609	NEK7	NIMA (never in mitosis gene a)-related kinase 7	1.0054	1.076682	0.9868115
140612	ZFP28	zinc finger protein 28 homolog (mouse)	0.94843	0.988664	0.958972
140625	ACTRT2	actin-related protein T2	0.98642	0.985801	1.0747644
140628	GATA5	GATA binding protein 5	1.00977	1.022727	1.0155865
140679	SLC32A1	solute carrier family 32 (GABA vesicular transporter), member 1	0.98173	1.009277	1.01143
140680	C20orf96	chromosome 20 open reading frame 96	0.99626	0.969433	1.0161605
140683	C20orf70	chromosome 20 open reading frame 70	0.99209	1.008828	1.0237532
140685	ZBTB46	zinc finger and BTB domain containing 46	1.00306	1.023739	0.9807214
140686	WFDC3	WAP four-disulfide core domain 3	1.01269	1.005252	0.9972664
140687	GCNT7	glucosaminyl (N-acetyl) transferase family member 7	0.98825	1.019661	0.9716563
140688	C20orf112	chromosome 20 open reading frame 112	0.98069	1.00511	0.9717748
140689	CBLN4	cerebellin 4 precursor	1.01533	1.004494	1.016518
140691	TRIM69	tripartite motif containing 69	1.01374	1.010981	0.9989066
140696	RPL37P1	ribosomal protein L37 pseudogene 1	0.95225	1.015566	1.048136
140697	RPL36P1	ribosomal protein L36 pseudogene 1	0.99622	0.983293	1.0625665
140699	C20orf132	chromosome 20 open reading frame 132	0.99341	1.003522	1.0308244
140700	SAMD10	sterile alpha motif domain containing 10	0.97853	1.010654	1.0810742
140701	ABHD16B	abhydrolase domain containing 16B	1.01304	1.020767	1.042496

140706	C20orf160	chromosome 20 open reading frame 160	0.98759	0.9797	1.048954
140710	KIAA0889	KIAA0889	0.9841	0.990394	1.0221755
140711	C20orf118	chromosome 20 open reading frame 118	0.97801	1.009149	1.0284824
140715	RPL21P2	ribosomal protein L21 pseudogene 2	1.01122	0.94003	0.9692869
140716	RPL35AP	ribosomal protein L35a pseudogene	0.94119	1.047525	1.0101663
140718	RPL31P3	ribosomal protein L31 pseudogene 3	1.00877	1.046039	0.9910382
140730	RIMS4	regulating synaptic membrane exocytosis 4	1.0064	1.010628	1.0250893
140731	ANKRD60	ankyrin repeat domain 60	0.97981	1.008557	1.0535425
140732	SUN5	Sad1 and UNC84 domain containing 5	0.99982	0.994649	1.0183633
140733	MACROD2	MACRO domain containing 2	0.9962	1.012723	1.0078778
140735	DYNLL2	dynein, light chain, LC8-type 2	0.94579	0.916295	0.9566391
140738	TMEM37	transmembrane protein 37	1.01494	1.04315	1.0465602
140739	UBE2F	ubiquitin-conjugating enzyme E2F (putative)	1.00257	1.002559	1.0398605
140748	RPL17P1	ribosomal protein L17 pseudogene 1	1.02072	1.050871	0.9748815
140749	RPL5P2	ribosomal protein L5 pseudogene 2	0.97759	0.978093	1.0539125
140750	RPL36P4	ribosomal protein L36 pseudogene 4	0.98007	0.95537	1.028691
140751	RPL36P2	ribosomal protein L36 pseudogene 2	1.03775	0.984092	0.9327218
140752	RPS27AP2	ribosomal protein S27a pseudogene 2	0.99888	1.004749	0.9915226
140755	RPS2P1	ribosomal protein S2 pseudogene 1	0.98721	0.90878	1.1058662
140756	RPL7AP14	ribosomal protein L7a pseudogene 14	1.01792	1.018232	0.9734669
140757	RSL24D1P6	ribosomal L24 domain containing 1 pseudogene 6	0.98381	1.082701	0.9720102
140760	RPL24P2	ribosomal protein L24 pseudogene 2	0.94972	1.078106	1.0397073
140766	ADAMTS14	ADAM metallopeptidase with thrombospondin type 1 motif, 14	0.99167	0.973479	1.0026199
140767	NRSN1	neurensin 1	1.00421	0.9955	1.0181177
140771	SMCR5	Smith-Magenis syndrome chromosome region, candidate 5 (non-protein coding)	1.01386	0.989147	1.001083
140775	SMCR8	Smith-Magenis syndrome chromosome region, candidate 8	0.94014	0.915159	0.9912801
140801	RPL10L	ribosomal protein L10-like	0.96698	1.001658	1.0981524
140803	TRPM6	transient receptor potential cation channel, subfamily M, member 6	0.98624	0.970533	0.9640727
140807	KRT72	keratin 72	0.9778	1.01997	0.8652756
140809	SRXN1	sulfiredoxin 1	0.93075	1.031471	0.9946151
140823	ROMO1	reactive oxygen species modulator 1	0.92983	0.990981	1.0557761
140825	NEURL2	neuralized homolog 2 (Drosophila)	1.00294	0.991094	0.9920192
140828	NCRNA002	non-protein coding RNA 261	0.98673	1.004732	1.0271285

140831	ZSWIM3	zinc finger, SWIM-type containing 3	0.95643	0.991729	0.9794567
140832	WFDC10A	WAP four-disulfide core domain 10A	1.01258	1.02367	1.0096513
140836	BANF2	barrier to autointegration factor 2	1.00238	1.006107	1.0469327
140838	NANP	N-acetylneuraminic acid phosphatase	0.97374	0.962264	1.0131267
140850	DEFB127	defensin, beta 127	0.99147	0.982182	1.008246
140856	C20orf79	chromosome 20 open reading frame 79	1.00034	1.029377	0.9879488
140857	RPL7P3	ribosomal protein L7 pseudogene 3	0.98696	0.995826	1.0143384
140862	ISM1	isthmin 1 homolog (zebrafish)	0.96771	0.98609	1.0051699
140870	WFDC6	WAP four-disulfide core domain 6	0.98712	0.9903	0.9829655
140873	C20orf173	chromosome 20 open reading frame 173	0.98035	1.014826	1.0333839
140876	FAM65C	family with sequence similarity 65, member C	0.99043	0.992401	0.9893437
140880	CST11	cystatin 11	1.00584	0.995923	0.9579106
140881	DEFB129	defensin, beta 129	1.03987	1.052383	0.9339526
140883	ZNF280B	zinc finger protein 280B	0.95601	0.964843	1.0283694
140885	SIRPA	signal-regulatory protein alpha	1.01116	1.03359	1.0015271
140886	PABPC5	poly(A) binding protein, cytoplasmic 5	0.97005	0.995298	0.9793164
140890	SREK1	splicing regulatory glutamine/lysine-rich protein 1	1.03408	0.855394	0.9868193
140893	C20orf151	chromosome 20 open reading frame 151	1.0038	0.982604	1.0098005
140894	C20orf152	chromosome 20 open reading frame 152	0.98811	0.993767	1.0216372
140901	STK35	serine/threonine kinase 35	0.98829	1.008246	1.0488282
140902	R3HDML	R3H domain containing-like	0.99316	1.018657	0.9973038
140947	C5orf20	chromosome 5 open reading frame 20	1.00462	1.020475	0.9868302
142678	MIB2	mindbomb homolog 2 (Drosophila)	0.97257	0.996817	1.0552857
142679	DUSP19	dual specificity phosphatase 19	0.98987	0.899934	1.0565783
142680	SLC34A3	solute carrier family 34 (sodium phosphate), member 3	1.01324	1.037212	1.0457107
142683	ITLN2	intelectin 2	1.00578	1.027054	1.0332492
142684	RAB40A	RAB40A, member RAS oncogene family	0.98635	1.037109	1.0121022
142685	ASB15	ankyrin repeat and SOCS box containing 15	1.01088	0.976557	1.0054711
142686	ASB14	ankyrin repeat and SOCS box containing 14	0.99341	1.015663	1.0072979
142689	ASB12	ankyrin repeat and SOCS box containing 12	1.01646	0.982865	0.9949793
142827	C10orf129	chromosome 10 open reading frame 129	1.01592	1.023447	1.0093289
142891	SAMD8	sterile alpha motif domain containing 8	0.96517	0.997657	1.0181916
142910	LIPJ	lipase, family member J	0.99357	1.011807	0.9700335

142913	CFL1P1	cofilin 1 (non-muscle) pseudogene 1	0.98978	0.988124	0.9826533
142940	TRUB1	TruB pseudouridine (psi) synthase homolog 1 (E. coli)	0.9166	0.974667	1.0649543
143098	MPP7	membrane protein, palmitoylated 7 (MAGUK p55 subfamily member 7)	0.96833	0.98605	0.9305416
143162	FRMPD2	FERM and PDZ domain containing 2	0.99853	1.00364	0.9936617
143187	VTI1A	vesicle transport through interaction with t-SNAREs homolog 1A (yeast)	0.97477	0.924167	1.0210641
143188	LOC143188	hypothetical LOC143188	0.99865	0.975498	0.9936535
143241	DYDC1	DPY30 domain containing 1	1.00605	1.040332	1.0000885
143279	HECTD2	HECT domain containing 2	0.97698	0.982051	0.993247
143282	FGFBP3	fibroblast growth factor binding protein 3	0.97551	1.03169	1.0090782
143341	EIF3LP2	eukaryotic translation initiation factor 3, subunit L pseudogene 2	0.94734	0.90243	1.0355187
143379	C10orf82	chromosome 10 open reading frame 82	1.00215	1.012297	1.0157704
143384	C10orf46	chromosome 10 open reading frame 46	0.98576	0.984877	1.0202023
143425	SYT9	synaptotagmin IX	0.99633	0.99752	1.0160084
143458	LDLRAD3	low density lipoprotein receptor class A domain containing 3	0.99804	0.989332	0.9957491
143471	PSMA8	proteasome (prosome, macropain) subunit, alpha type, 8	0.98238	0.9967	1.0232986
143501	C11orf40	chromosome 11 open reading frame 40	1.0042	0.97831	0.9435641
143503	OR51E1	olfactory receptor, family 51, subfamily E, member 1	1.01379	1.044429	1.0415777
143570	XRRA1	X-ray radiation resistance associated 1	0.95937	1.009054	1.0320044
143630	UBQLNL	ubiquilin-like	1.03562	0.998976	1.0194475
143662	MUC15	mucin 15, cell surface associated	0.99233	1.015176	1.0312418
143666	LOC143666	hypothetical LOC143666	1.00212	1.049576	1.0157477
143678	C11orf94	chromosome 11 open reading frame 94	0.99392	1.026288	1.0144714
143684	FAM76B	family with sequence similarity 76, member B	0.96889	0.936358	1.0349038
143686	SESN3	sestrin 3	0.91034	0.89915	0.9717933
143689	PIWIL4	piwi-like 4 (Drosophila)	0.96513	1.04129	1.0208349
143872	ARHGAP42	Rho GTPase activating protein 42	0.99204	0.988941	1.0093938
143879	KBTBD3	kelch repeat and BTB (POZ) domain containing 3	0.97851	0.971127	0.9642827
143884	CWF19L2	CWF19-like 2, cell cycle control (S. pombe)	0.92203	0.953876	1.0658397
143888	KDELC2	KDEL (Lys-Asp-Glu-Leu) containing 2	0.9047	0.884165	0.9651155
143903	LAYN	layilin	0.99904	1.021316	0.996978
143941	TTC36	tetratricopeptide repeat domain 36	0.98936	1.034618	1.0667848
144097	C11orf84	chromosome 11 open reading frame 84	0.96529	1.048525	1.0520866
144100	PLEKHA7	pleckstrin homology domain containing, family A member 7	0.9954	1.002626	1.0230846

144108	SPTY2D1	SPT2, Suppressor of Ty, domain containing 1 (S. cerevisiae)	0.90906	0.992206	1.0174207
144110	TMEM86A	transmembrane protein 86A	0.99825	1.03753	0.961039
144124	OR10A5	olfactory receptor, family 10, subfamily A, member 5	1.01703	1.008302	1.0109154
144125	OR2AG1	olfactory receptor, family 2, subfamily AG, member 1	1.01555	0.996091	1.046773
144132	DNHD1	dynein heavy chain domain 1	0.97261	1.028159	1.0026689
144165	PRICKLE1	prickle homolog 1 (Drosophila)	0.95399	1.04631	1.0449074
144193	AMDHD1	amidohydrolase domain containing 1	0.99008	0.971737	0.9721987
144195	SLC2A14	solute carrier family 2 (facilitated glucose transporter), member 14	0.98076	1.009854	0.988256
144203	OVOS2	ovostatin 2	1.01444	0.979354	0.9736853
144233	BCDIN3D	BCDIN3 domain containing	0.94207	0.985819	1.0473533
144245	ALG10B	asparagine-linked glycosylation 10, alpha-1,2-glucosyltransferase homolog B (yeast)	0.99521	1.004228	0.9863914
144321	GLIPR1L2	GLI pathogenesis-related 1 like 2	0.99539	0.983835	0.9967195
144347	FAM101A	family with sequence similarity 101, member A	0.98664	0.980439	1.0621829
144348	ZNF664	zinc finger protein 664	0.97055	0.99373	1.0696369
144360	C12orf67	chromosome 12 open reading frame 67	0.98798	0.968141	1.0260892
144363	LYRM5	LYR motif containing 5	0.9269	0.939235	0.9431708
144383	LOC144383	interferon induced transmembrane protein pseudogene	0.98737	1.000633	1.0849697
144402	CPNE8	copine VIII	0.94299	0.90972	1.0770353
144404	TMEM120E	transmembrane protein 120B	0.98867	0.9845	1.0067223
144423	GLT1D1	glycosyltransferase 1 domain containing 1	0.95116	0.988575	1.000601
144448	TSPAN19	tetraspanin 19	1.01071	1.003095	1.0089883
144453	BEST3	bestrophin 3	0.99929	0.985976	1.0138967
144455	E2F7	E2F transcription factor 7	0.99163	0.987755	1.0155601
144481	LOC144481	hypothetical protein LOC144481	0.98692	1.036399	1.0547154
144501	KRT80	keratin 80	1.01417	0.993123	1.0152265
144535	C12orf55	chromosome 12 open reading frame 55	0.99918	0.984058	1.0008689
144568	A2ML1	alpha-2-macroglobulin-like 1	0.99752	0.995367	1.0041071
144577	C12orf66	chromosome 12 open reading frame 66	0.94892	0.942398	1.0547336
144581	RPL14P1	ribosomal protein L14 pseudogene 1	1.04011	0.983968	1.0250203
144608	C12orf60	chromosome 12 open reading frame 60	0.98502	1.004769	0.9392525
144638	ST13P3	suppression of tumorigenicity 13 (colon carcinoma) (Hsp70 interacting protein) pseudogene 3	0.97445	1.010282	1.0309295
144699	FBXL14	F-box and leucine-rich repeat protein 14	0.9892	0.994254	0.9854621
144712	HMGA1P3	high mobility group AT-hook 1 pseudogene 3	1.0224	1.020038	0.9135266

144715	RAD9B	RAD9 homolog B (S. pombe)	0.95365	0.988213	1.0172875
144717	FAM109A	family with sequence similarity 109, member A	0.98227	1.029652	1.0883244
144742	LOC144742	hypothetical LOC144742	1.02923	1.032457	0.9708196
144809	C13orf30	chromosome 13 open reading frame 30	0.97607	1.000951	1.0123648
144811	C13orf31	chromosome 13 open reading frame 31	0.95691	0.933566	0.9984279
144817	LOC144817	hypothetical protein LOC144817	1.00304	0.989773	0.9730562
144832	ESRRAP2	estrogen-related receptor alpha pseudogene 2	0.97826	1.048832	0.9760157
144983	HNRNPA1L	heterogeneous nuclear ribonucleoprotein A1-like 2	0.96886	0.936544	1.052532
145165	ST13P4	suppression of tumorigenicity 13 (colon carcinoma) (Hsp70 interacting protein) pseudogene 4	1.04266	1.002718	1.0012612
145173	B3GALTL	beta 1,3-galactosyltransferase-like	0.93796	0.955609	0.9677643
145200	NCRNA002	non-protein coding RNA 239	0.99721	0.992371	0.9580678
145216	LOC145216	hypothetical LOC145216	0.99512	1.066119	0.9835453
145226	RDH12	retinol dehydrogenase 12 (all-trans/9-cis/11-cis)	1.00538	0.978212	0.9573249
145235	RPL24P3	ribosomal protein L24 pseudogene 3	0.99698	1.010314	0.9267572
145241	ADAM21P1	ADAM metallopeptidase domain 21 pseudogene 1	1.01635	1.008049	0.9826299
145258	GSC	goosecoid homeobox	0.97247	1.021529	1.0577833
145259	RPSAP4	ribosomal protein SA pseudogene 4	1.04272	1.079057	1.0303076
145264	SERPINA12	serpin peptidase inhibitor, clade A (alpha-1 antiproteinase, antitrypsin), member 12	0.99337	0.983306	1.027875
145270	PRIMA1	proline rich membrane anchor 1	1.00797	1.004243	1.0192134
145282	MIPOL1	mirror-image polydactyly 1	0.98946	1.003113	1.0083452
145376	C14orf50	chromosome 14 open reading frame 50	1.0011	0.996531	1.0137037
145389	SLC38A6	solute carrier family 38, member 6	0.95794	0.949496	0.9619034
145407	C14orf37	chromosome 14 open reading frame 37	1.00522	0.986773	1.0234994
145438	C14orf82	chromosome 14 open reading frame 82	1.0041	0.977486	1.0297443
145447	ABHD12B	abhydrolase domain containing 12B	0.99584	0.980263	0.9868445
145482	PTGR2	prostaglandin reductase 2	0.95593	0.918466	0.9982111
145483	FAM161B	family with sequence similarity 161, member B	0.95983	1.013477	1.007841
145497	C14orf166B	chromosome 14 open reading frame 166B	0.99686	0.992582	1.019117
145499	RPLP1P1	ribosomal protein, large, P1 pseudogene 1	0.95872	0.959014	1.0599701
145501	ISM2	isthmin 2 homolog (zebrafish)	0.98111	0.997689	1.0597228
145508	CEP128	centrosomal protein 128kDa	0.96286	0.999267	1.0318095
145553	MDP1	magnesium-dependent phosphatase 1	0.93599	1.018196	1.0590655
145567	TTC7B	tetratricopeptide repeat domain 7B	0.97929	0.977535	0.9975192

145581	LRFN5	leucine rich repeat and fibronectin type III domain containing 5	0.99183	1.02378	1.0062265
145645	C15orf43	chromosome 15 open reading frame 43	1.01363	1.003421	1.0108202
145741	C2CD4A	C2 calcium-dependent domain containing 4A	1.0101	0.951091	1.0423674
145748	LYSMD4	LysM, putative peptidoglycan-binding, domain containing 4	0.96301	0.928225	0.9695993
145757	LOC145757	hypothetical LOC145757	0.97147	0.98787	0.990548
145773	FAM81A	family with sequence similarity 81, member A	1.00423	0.987828	1.0227283
145781	GCOM1	GRINL1A complex locus	0.95759	0.968464	0.9504298
145788	FLJ27352	hypothetical LOC145788	0.99948	1.00908	0.9630216
145814	PGPEP1L	pyroglutamyl-peptidase I-like	0.99438	1.026335	0.9926362
145837	LOC145837	hypothetical LOC145837	1.00927	1.010717	1.0226134
145845	LOC145845	hypothetical LOC145845	0.99134	0.97775	1.0245184
145853	C15orf61	chromosome 15 open reading frame 61	0.96878	0.948396	0.9835642
145858	C15orf32	chromosome 15 open reading frame 32	1.02423	0.960676	0.9805593
145864	HAPLN3	hyaluronan and proteoglycan link protein 3	0.94459	0.891577	1.02769
145873	MESP2	mesoderm posterior 2 homolog (mouse)	0.98249	1.065729	1.0326033
145942	TMCO5A	transmembrane and coiled-coil domains 5A	1.00514	0.99241	1.0085899
145946	SPATA8	spermatogenesis associated 8	0.99698	1.013208	0.9877452
145957	NRG4	neuregulin 4	0.98453	0.985919	0.9945269
145989	LOC145989	fascin homolog 1, actin-bundling protein (Strongylocentrotus purpuratus) pseudogene	0.96917	0.938342	1.1160781
146050	ZSCAN29	zinc finger and SCAN domain containing 29	0.92235	0.935398	0.9742632
146057	TTBK2	tau tubulin kinase 2	0.95779	0.955706	0.9855894
146059	CDAN1	congenital dyserythropoietic anemia, type I	0.95121	0.964174	1.0522781
146167	SLC38A8	solute carrier family 38, member 8	0.99355	0.970546	1.0458809
146177	VWA3A	von Willebrand factor A domain containing 3A	0.9952	1.004003	0.9980626
146183	OTOA	otoancorin	1.00037	0.985113	0.9605983
146198	ZFP90	zinc finger protein 90 homolog (mouse)	0.93855	1.038148	0.9411722
146206	RLTPR	RGD motif, leucine rich repeats, tropomodulin domain and proline-rich containing	0.96894	1.021609	1.0247619
146212	KCTD19	potassium channel tetramerisation domain containing 19	1.00159	1.010541	1.0030049
146223	CMTM4	CKLF-like MARVEL transmembrane domain containing 4	0.98158	0.968701	1.0088063
146227	BEAN1	brain expressed, associated with NEDD4, 1	0.98844	0.988118	1.014466
146253	LOC146253	tropomyosin-like	0.96001	0.902563	1.0330901
146279	TEKT5	tektin 5	1.01289	1.023207	1.0063167
146310	RNF151	ring finger protein 151	1.01348	1.000665	0.9762122

146325	C16orf11	chromosome 16 open reading frame 11	1.0035	0.983358	1.0149949
146330	FBXL16	F-box and leucine-rich repeat protein 16	0.95478	0.969423	1.0716869
146336	LOC146336	hypothetical LOC146336	0.97413	0.976712	1.0524179
146378	C16orf92	chromosome 16 open reading frame 92	1.00281	0.999495	1.0539304
146395	GSG1L	GSG1-like	1.00848	0.991089	1.0015147
146429	LOC146429	putative solute carrier family 22 member ENSG00000182157	0.99446	0.997401	1.0243775
146433	IL34	interleukin 34	0.98261	1.022224	1.0019185
146434	ZNF597	zinc finger protein 597	0.90979	0.992631	1.0760591
146439	CCDC64B	coiled-coil domain containing 64B	0.99063	0.990634	0.9912764
146456	TMED6	transmembrane emp24 protein transport domain containing 6	0.98329	1.052536	1.0099695
146481	LOC146481	FSHD region gene 2 family member pseudogene	1.00024	0.967638	0.9957923
146512	FLJ30679	hypothetical protein FLJ30679	0.98228	0.97442	0.9957606
146540	ZNF785	zinc finger protein 785	0.97576	0.939342	0.9984916
146542	ZNF688	zinc finger protein 688	0.96718	0.954449	0.9935384
146547	PRSS36	protease, serine, 36	0.9874	1.00163	1.016095
146556	C16orf89	chromosome 16 open reading frame 89	1.01894	1.020697	0.9800689
146562	C16orf71	chromosome 16 open reading frame 71	1.00576	0.991537	1.0109023
146664	MGAT5B	mannosyl (alpha-1,6-)-glycoprotein beta-1,6-N-acetyl-glucosaminyltransferase, isozyme B	0.98859	0.998037	1.0190808
146691	TOM1L2	target of myb1-like 2 (chicken)	0.96339	0.980086	1.0041579
146705	C17orf56	chromosome 17 open reading frame 56	0.97255	1.000384	1.0374924
146712	B3GNTL1	UDP-GlcNAc:betaGal beta-1,3-N-acetylglucosaminyltransferase-like 1	0.991	1.013837	1.0333728
146722	CD300LF	CD300 molecule-like family member f	1.01012	0.849485	1.0141408
146723	C17orf77	chromosome 17 open reading frame 77	0.99195	0.945708	0.9366
146754	DNAH2	dynein, axonemal, heavy chain 2	0.99392	0.999809	1.0007653
146760	RTN4RL1	reticulon 4 receptor-like 1	1.00532	1.00937	1.0337049
146771	TCAM1P	testicular cell adhesion molecule 1 homolog (mouse), pseudogene	1.00463	1.023521	1.0171786
146779	EFCAB3	EF-hand calcium binding domain 3	0.97034	0.953739	1.0067085
146802	SLC47A2	solute carrier family 47, member 2	0.99641	0.991569	0.995887
146845	WDR16	WD repeat domain 16	0.99682	0.99332	0.9880602
146849	CCDC42	coiled-coil domain containing 42	0.99976	1.010686	0.9687822
146850	PIK3R6	phosphoinositide-3-kinase, regulatory subunit 6	0.99107	0.991321	1.0205795
146852	ODF4	outer dense fiber of sperm tails 4	1.00979	1.018999	0.9743283
146853	C17orf50	chromosome 17 open reading frame 50	0.98012	0.968457	0.9953802

146857	SLFN13	schlafen family member 13	0.95918	0.933054	1.0978487
146862	UNC45B	unc-45 homolog B (C. elegans)	0.97987	0.97853	0.9877551
146880	LOC14688C	hypothetical LOC146880	0.98021	1.009017	1.0098256
146894	CD300LG	CD300 molecule-like family member g	0.99817	0.974647	0.9945508
146909	KIF18B	kinesin family member 18B	0.99471	0.991712	1.0036759
146923	RUNDC1	RUN domain containing 1	0.94989	1.005969	1.0901294
146956	EME1	essential meiotic endonuclease 1 homolog 1 (S. pombe)	0.99414	1.012745	1.0101106
147007	TMEM199	transmembrane protein 199	0.96926	1.004191	1.0239487
147011	PROCA1	protein interacting with cyclin A1	1.0072	0.994709	0.9854464
147015	DHRS13	dehydrogenase/reductase (SDR family) member 13	0.96069	1.013567	1.0848908
147040	KCTD11	potassium channel tetramerisation domain containing 11	0.9859	0.953913	1.014749
147081	C17orf69	chromosome 17 open reading frame 69	1.0166	0.997545	1.0146285
147111	NOTUM	notum pectinacetylesterase homolog (Drosophila)	0.97717	1.036344	1.0673347
147138	TMC8	transmembrane channel-like 8	0.97469	1.031271	0.8988405
147172	LRRC37BP1	leucine rich repeat containing 37B pseudogene 1	1.00372	0.937342	1.0106598
147179	WIPF2	WAS/WASL interacting protein family, member 2	0.95885	1.024524	0.9784526
147183	KRT25	keratin 25	0.97726	1.038223	1.0093944
147184	TMEM99	transmembrane protein 99	0.98517	0.987718	1.0169763
147199	SCGB1C1	secretoglobin, family 1C, member 1	0.90622	0.993447	0.9943444
147323	STARD6	StAR-related lipid transfer (START) domain containing 6	0.97255	0.976939	1.0122249
147339	C18orf25	chromosome 18 open reading frame 25	0.96734	1.00502	0.9738916
147372	CCBE1	collagen and calcium binding EGF domains 1	1.00518	0.997905	1.0136769
147381	CBLN2	cerebellin 2 precursor	1.0064	1.016574	1.0172399
147407	MCART2	mitochondrial carrier triple repeat 2	0.9835	0.992979	1.0897402
147409	DSG4	desmoglein 4	0.98493	1.021377	0.9915677
147429	CHST9-AS1	CHST9 antisense RNA 1 (non-protein coding)	0.97458	0.988504	0.9811576
147463	ANKRD29	ankyrin repeat domain 29	0.9895	1.022713	1.0180414
147468	WBP2P1	WW domain binding protein 2 pseudogene 1	1.02689	1.040071	1.0276327
147495	APCDD1	adenomatosis polyposis coli down-regulated 1	0.9996	1.01156	1.0060395
147525	C18orf18	chromosome 18 open reading frame 18	0.93724	0.976546	1.0546365
147645	VSIG10L	V-set and immunoglobulin domain containing 10 like	0.99586	1.007375	0.9852048
147646	LOC147646	hypothetical protein LOC147646	1.00341	1.036082	1.0274735
147650	NCRNA000	non-protein coding RNA 85	0.96232	1.035294	0.9751708

147657	ZNF480	zinc finger protein 480	0.88561	0.93112	1.0753099
147658	ZNF534	zinc finger protein 534	0.99092	0.989238	0.9985793
147660	ZNF578	zinc finger protein 578	0.98432	0.974437	0.9811735
147670	LOC147670	hypothetical protein LOC147670	1.00719	1.027194	0.9901452
147685	C19orf18	chromosome 19 open reading frame 18	1.00541	0.976247	1.0081026
147686	ZNF418	zinc finger protein 418	0.96313	1.005289	1.0316189
147687	ZNF417	zinc finger protein 417	0.9501	0.950154	1.0337776
147694	ZNF548	zinc finger protein 548	0.93729	0.927732	1.0381679
147699	PPM1N	protein phosphatase, Mg2+/Mn2+ dependent, 1N (putative)	0.97689	0.986375	0.9850297
147700	KLC3	kinesin light chain 3	1.00181	0.962872	1.041314
147710	IGSF23	immunoglobulin superfamily, member 23	1.00811	1.046512	0.9647388
147711	ZNF285B	zinc finger protein 285B, pseudogene	1.0132	1.024063	1.0561045
147719	LYPD4	LY6/PLAUR domain containing 4	0.97927	1.02579	1.0151293
147727	LOC147727	hypothetical LOC147727	0.88118	0.879495	0.8934777
147741	ZNF560	zinc finger protein 560	0.99429	1.005418	1.0224166
147744	TMEM190	transmembrane protein 190	0.99219	0.949415	1.0751044
147746	HIPK4	homeodomain interacting protein kinase 4	0.98705	0.995568	1.0414871
147798	TMC4	transmembrane channel-like 4	1.00385	0.957362	1.0100317
147804	LOC147804	tropomyosin 3 pseudogene	0.99686	0.913958	1.0924713
147807	ZNF524	zinc finger protein 524	0.97375	1.00619	1.0433748
147808	ZNF784	zinc finger protein 784	0.96359	0.968213	1.1006689
147837	ZNF563	zinc finger protein 563	0.98131	0.956307	0.9534756
147841	SPC24	SPC24, NDC80 kinetochore complex component, homolog (S. cerevisiae)	0.96889	0.993621	1.0136811
147872	CCDC155	coiled-coil domain containing 155	1.00163	1.004832	1.0121447
147906	DACT3	dapper, antagonist of beta-catenin, homolog 3 (Xenopus laevis)	0.97368	1.036908	1.0109962
147912	SIX5	SIX homeobox 5	1.0067	1.02265	1.083083
147920	IGFL2	IGF-like family member 2	1.00395	1.024044	0.998358
147923	ZNF420	zinc finger protein 420	0.90236	1.03449	1.0519378
147929	ZNF565	zinc finger protein 565	0.9601	0.994592	0.9963493
147945	NLRP4	NLR family, pyrin domain containing 4	1.01115	0.986974	1.0281562
147948	ZNF582	zinc finger protein 582	0.93865	0.995932	1.0025895
147949	ZNF583	zinc finger protein 583	0.92912	0.987799	1.0416122
147965	FAM98C	family with sequence similarity 98, member C	0.96602	0.928534	1.0870563

147968	CAPN12	calpain 12	0.99878	1.035526	1.0270874
147991	DPY19L3	dpy-19-like 3 (C. elegans)	0.96861	0.963352	1.0701131
148003	LGALS16	beta-galactoside-binding lectin	1.042	1.064263	1.0526685
148014	TTC9B	tetratricopeptide repeat domain 9B	0.98254	0.981309	1.0813909
148022	TICAM1	toll-like receptor adaptor molecule 1	0.97313	1.014895	0.9927103
148046	C19orf23	chromosome 19 open reading frame 23	0.99541	1.009603	0.9969896
148066	ZNRF4	zinc and ring finger 4	1.00159	1.001213	1.011249
148103	ZNF599	zinc finger protein 599	0.96288	0.983917	0.9978321
148109	FAM187B	family with sequence similarity 187, member B	0.97918	0.943522	1.0884483
148113	CILP2	cartilage intermediate layer protein 2	0.98837	1.03055	1.0378854
148137	C19orf55	chromosome 19 open reading frame 55	0.98445	1.018969	1.0003825
148156	ZNF558	zinc finger protein 558	0.97477	0.957203	1.0640701
148170	CDC42EP5	CDC42 effector protein (Rho GTPase binding) 5	1.00146	0.981434	1.0317888
148203	ZNF738	zinc finger protein 738	0.94105	0.964759	1.0908131
148206	ZNF714	zinc finger protein 714	0.99091	0.982973	1.0405986
148213	ZNF681	zinc finger protein 681	0.93278	0.979389	0.9900942
148223	C19orf25	chromosome 19 open reading frame 25	0.97453	1.021116	1.0599863
148229	ATP8B3	ATPase, aminophospholipid transporter, class I, type 8B, member 3	0.98158	0.982546	1.0759924
148252	DIRAS1	DIRAS family, GTP-binding RAS-like 1	1.01044	0.982	0.9974829
148254	ZNF555	zinc finger protein 555	0.8862	1.014603	1.013829
148266	ZNF569	zinc finger protein 569	0.95076	1.009821	1.0554615
148268	ZNF570	zinc finger protein 570	0.90364	1.002988	1.0681395
148300	SONP1	SON pseudogene 1	0.9528	1.028358	1.0169413
148304	C1orf74	chromosome 1 open reading frame 74	0.98336	1.000179	0.969812
148327	CREB3L4	cAMP responsive element binding protein 3-like 4	1.00067	0.986034	1.0474229
148345	C1orf127	chromosome 1 open reading frame 127	1.02397	0.993385	1.0162656
148362	BROX	BRO1 domain and CAAX motif containing	1.01644	0.919353	1.0060578
148398	SAMD11	sterile alpha motif domain containing 11	0.98605	1.01095	1.0594047
148413	LOC148413	hypothetical LOC148413	0.98277	1.003998	0.9976628
148418	SAMD13	sterile alpha motif domain containing 13	0.99727	0.975239	1.0017088
148423	C1orf52	chromosome 1 open reading frame 52	1.01343	0.888748	0.9156879
148430	LOC148430	ribosomal protein S2 pseudogene	0.95678	0.988992	1.096832
148479	PHF13	PHD finger protein 13	0.99602	0.937593	1.0333333

148523	C1orf51	chromosome 1 open reading frame 51	1.003	0.955956	0.9780487
148534	TMEM56	transmembrane protein 56	1.00807	0.929872	1.030729
148581	UBE2U	ubiquitin-conjugating enzyme E2U (putative)	0.98838	0.999726	1.0226215
148641	SLC35F3	solute carrier family 35, member F3	1.01675	1.000744	1.0293035
148645	C1orf211	chromosome 1 open reading frame 211	0.99579	1.031263	0.9928684
148696	LOC148696	hypothetical LOC148696	1.01772	0.998029	0.9292571
148709	LOC148709	actin pseudogene	1.01185	1.016725	1.0291756
148713	PTPRVP	protein tyrosine phosphatase, receptor type, V, pseudogene	1.00367	1.014164	1.0032615
148738	HFE2	hemochromatosis type 2 (juvenile)	0.98803	0.990777	1.0309407
148741	ANKRD35	ankyrin repeat domain 35	0.99663	1.0078	1.017308
148753	FAM163A	family with sequence similarity 163, member A	0.99585	1.00037	0.9914684
148766	[No Symbo	[No Name]	1.0169	1.001733	1.0377595
148789	B3GALNT2	beta-1,3-N-acetylgalactosaminyltransferase 2	1.01105	0.95986	1.0544245
148808	MFSD4	major facilitator superfamily domain containing 4	0.99446	0.987207	1.009273
148811	PM20D1	peptidase M20 domain containing 1	0.98191	1.005167	0.9571351
148823	C1orf150	chromosome 1 open reading frame 150	0.95119	0.928269	1.0091869
148867	SLC30A7	solute carrier family 30 (zinc transporter), member 7	0.97507	0.951651	0.9247111
148870	CCDC27	coiled-coil domain containing 27	0.99926	1.011537	1.0213039
148898	C1orf213	chromosome 1 open reading frame 213	0.9767	1.019811	1.0143301
148930	KNCN	kinocilin	1.02147	1.031683	0.9869985
148932	MOBKL2C	MOB1, Mps One Binder kinase activator-like 2C (yeast)	0.97958	0.959692	1.0100443
148979	GLIS1	GLIS family zinc finger 1	1.00374	1.002858	1.0227496
149018	LELP1	late cornified envelope-like proline-rich 1	1.04643	1.014854	0.9819471
149041	RC3H1	ring finger and CCCH-type domains 1	0.99065	1.078519	1.0647277
149069	DCDC2B	doublecortin domain containing 2B	1.00961	1.016164	1.0071633
149076	ZNF362	zinc finger protein 362	0.97519	0.944392	0.9891919
149086	LOC149086	ornithine decarboxylase 1 pseudogene	1.01857	0.957945	0.9974409
149095	DCST1	DC-STAMP domain containing 1	0.99568	1.008132	0.9940372
149111	CNIH3	cornichon homolog 3 (Drosophila)	1.02862	1.015689	1.0117264
149134	LOC149134	hypothetical LOC149134	0.98246	1.027043	1.0116264
149157	[No Symbo	[No Name]	1.02562	0.998205	0.9918366
149175	MANEAL	mannosidase, endo-alpha-like	1.00147	1.024614	0.9982441
149233	IL23R	interleukin 23 receptor	1.00017	1.015948	1.0029112

149281	METTL11B	methyltransferase like 11B	1.00664	1.004134	1.0481101
149297	FAM78B	family with sequence similarity 78, member B	1.01049	0.995525	1.0267658
149345	SHISA4	shisa homolog 4 (Xenopus laevis)	0.98532	0.984171	1.0125873
149351	LOC149351	hypothetical protein LOC149351	0.99431	1.015002	0.9923468
149371	EXOC8	exocyst complex component 8	0.99885	0.971003	0.9583985
149373	FLJ30430	hypothetical protein FLJ30430	1.01017	0.980109	1.0115056
149420	PDIK1L	PDLIM1 interacting kinase 1 like	0.96826	0.943142	1.0564119
149421	[No Symbo	[No Name]	1.01423	1.003327	0.9883469
149428	BNIPL	BCL2/adenovirus E1B 19kD interacting protein like	1.00017	1.009986	1.0223239
149461	CLDN19	claudin 19	1.03104	1.003327	0.9735252
149465	WDR65	WD repeat domain 65	1.0033	0.9753	1.023082
149466	C1orf210	chromosome 1 open reading frame 210	0.98104	0.999575	0.9770292
149469	[No Symbo	[No Name]	1.00882	0.975507	0.9875147
149473	CCDC24	coiled-coil domain containing 24	1.00585	1.002238	1.0239606
149478	BTBD19	BTB (POZ) domain containing 19	1.02406	1.042343	0.9640438
149483	CCDC17	coiled-coil domain containing 17	1.00287	0.983446	0.9794648
149499	LRRC71	leucine rich repeat containing 71	1.00578	0.974081	1.0107219
149501	KRT8P45	keratin 8 pseudogene 45	0.97634	0.996316	1.0413732
149563	C1orf64	chromosome 1 open reading frame 64	0.99459	1.009896	0.9390767
149603	RNF187	ring finger protein 187	0.98062	1.009457	1.0215633
149620	RP11-165H	CHIA-like pseudogene	1.01167	0.967382	1.0043413
149628	PYHIN1	pyrin and HIN domain family, member 1	0.98454	1.050375	0.8844409
149643	C1orf227	chromosome 1 open reading frame 227	1.00531	0.969975	1.0027276
149647	FAM71A	family with sequence similarity 71, member A	1.02588	0.989355	0.9899686
149650	FLJ32154	hypothetical protein FLJ32154	0.96815	0.962218	0.9880279
149685	ADIG	adipogenin	1.00696	0.996564	0.9920887
149699	GTSF1L	gametocyte specific factor 1-like	0.9977	0.985401	1.0574415
149708	WFDC5	WAP four-disulfide core domain 5	1.00202	0.963722	0.9670632
149773	LOC149773	hypothetical LOC149773	0.97256	1.046216	0.9801534
149775	GNAS-AS1	GNAS antisense RNA 1 (non-protein coding)	0.98326	1.012544	1.0239786
149830	PRNT	prion protein (testis specific)	1.00685	1.068426	0.9756284
149837	LOC149837	hypothetical LOC149837	1.01443	1.007652	1.0223174
149840	C20orf196	chromosome 20 open reading frame 196	0.96406	0.965926	0.9905843

149844	LOC149844	synaptotagmin binding, cytoplasmic RNA interacting protein pseudogene	1.03291	1.046301	1.0004592
149950	LOC149950	hypothetical LOC149950	0.98485	1.056415	0.9827004
149951	COMMD7	COMM domain containing 7	0.96374	1.059137	1.0629249
149954	C20orf186	chromosome 20 open reading frame 186	1.01355	0.993628	0.9913954
149986	LSM14B	LSM14B, SCD6 homolog B (S. cerevisiae)	0.95382	1.017034	1.005384
149998	LIPI	lipase, member I	0.98149	1.025925	1.0381308
150000	ABCC13	ATP-binding cassette, sub-family C (CFTR/MRP), member 13, pseudogene	0.98416	1.027391	0.9919857
150051	LOC150051	hypothetical LOC150051	0.99699	1.006871	1.0412925
150082	LCA5L	Leber congenital amaurosis 5-like	0.98773	1.035637	1.0170801
150084	IGSF5	immunoglobulin superfamily, member 5	1.0039	1.018557	1.0121804
150094	SIK1	salt-inducible kinase 1	1.00464	0.991334	0.9923109
150135	C21orf129	chromosome 21 open reading frame 129	0.97613	0.967346	1.0366339
150142	NCRNA003	non-protein coding RNA 318	0.97962	0.99504	1.0406219
150147	C21orf128	chromosome 21 open reading frame 128	1.00634	0.977246	0.9764943
150159	NHEDC1	Na+/H+ exchanger domain containing 1	0.99737	0.989349	0.9938864
150165	XKR3	XK, Kell blood group complex subunit-related family, member 3	0.99569	0.994138	1.0186899
150185	LOC150185	hypothetical LOC150185	1.06047	1.060497	0.9878302
150197	LOC150197	hypothetical LOC150197	1.00794	0.981954	0.9619223
150209	AIFM3	apoptosis-inducing factor, mitochondrion-associated, 3	0.98283	0.991668	1.0333025
150223	YDJC	YdjC homolog (bacterial)	0.97767	0.931967	1.1013937
150244	ZDHHC8P1	zinc finger, DHHC-type containing 8 pseudogene 1	0.97203	0.995232	0.9801209
150248	C22orf15	chromosome 22 open reading frame 15	1.00382	0.957971	0.9993816
150274	HSCB	HscB iron-sulfur cluster co-chaperone homolog (E. coli)	0.97508	0.991322	1.0625867
150275	CCDC117	coiled-coil domain containing 117	0.92126	1.014351	1.0612011
150280	HORMAD2	HORMA domain containing 2	0.9768	1.035945	1.0016446
150290	DUSP18	dual specificity phosphatase 18	0.96638	0.9224	1.0055237
150291	MORC2-AS	MORC2 antisense RNA 1 (non-protein coding)	0.96877	0.997669	1.061512
150297	C22orf42	chromosome 22 open reading frame 42	1.00555	0.94003	0.9942783
150350	ENTHD1	ENTH domain containing 1	0.99268	1.006172	1.0176627
150353	DNAJB7	DnaJ (Hsp40) homolog, subfamily B, member 7	1.007	0.965191	0.970616
150365	MEI1	meiosis inhibitor 1	0.98767	0.994296	1.0023506
150368	FAM109B	family with sequence similarity 109, member B	0.97541	0.977564	1.008094
150372	NFAM1	NFAT activating protein with ITAM motif 1	0.98647	0.981168	0.9994199

150379	PNPLA5	patatin-like phospholipase domain containing 5	0.99884	0.956783	0.9867365
150383	C22orf40	chromosome 22 open reading frame 40	0.97709	0.957876	1.0165354
150384	CN5H6.4	hypothetical LOC150384	0.99498	1.027128	0.9882247
150465	TTL	tubulin tyrosine ligase	1.00521	0.993648	1.0739857
150468	CKAP2L	cytoskeleton associated protein 2-like	0.98837	0.978055	1.0273024
150483	TEKT4	tektin 4	0.97061	0.951343	1.0471715
150519	LOC150519	hypothetical LOC150519	1.00208	1.043764	1.0123677
150538	FLJ32063	hypothetical LOC150538	0.9673	0.999756	1.0188924
150554	LOC150554	tyrosine 3-monooxygenase/tryptophan 5-monooxygenase activation protein, zeta pseudogene	0.96604	0.988659	0.9861627
150572	SMYD1	SET and MYND domain containing 1	0.99756	0.997539	1.030823
150577	LOC150577	hypothetical LOC150577	1.00753	0.979853	1.0260135
150590	C2orf15	chromosome 2 open reading frame 15	0.97203	1.009845	1.0449922
150622	LOC150622	hypothetical LOC150622	1.02324	0.973159	0.9579637
150677	OTOS	otospiralin	0.98555	1.003751	0.9665664
150678	MYEOV2	myeloma overexpressed 2	0.92842	0.931302	1.0945028
150684	COMMD1	copper metabolism (Murr1) domain containing 1	1.01532	0.964263	1.0387238
150696	PROM2	prominin 2	1.00245	1.024001	1.0304354
150709	ANKAR	ankyrin and armadillo repeat containing	0.9873	0.969314	0.9567172
150726	FBXO41	F-box protein 41	0.97556	0.97888	1.0714467
150737	TTC30B	tetratricopeptide repeat domain 30B	1.00128	1.005876	1.0500023
150763	GPAT2	glycerol-3-phosphate acyltransferase 2, mitochondrial	0.98366	0.985348	1.0656515
150771	ITPRIPL1	inositol 1,4,5-trisphosphate receptor interacting protein-like 1	1.02076	1.012541	1.0984835
150776	LOC150776	sphingomyelin phosphodiesterase 4, neutral membrane pseudogene	0.98336	1.004516	0.9709557
150786	WTH3DI	RAB6C-like	1.02104	0.985434	1.0048132
150864	FAM117B	family with sequence similarity 117, member B	0.957	0.915125	0.905397
150921	TCF23	transcription factor 23	1.00333	1.031043	1.0275252
150935	LOC150935	hypothetical LOC150935	1.00287	0.998984	1.0035081
150946	FAM59B	family with sequence similarity 59, member B	1.00531	1.035685	1.0522451
150947	PPIL1P1	peptidylprolyl isomerase (cyclophilin)-like 1 pseudogene 1	0.99451	0.993279	1.0127893
150962	PUS10	pseudouridylate synthase 10	0.99675	1.005146	0.9902592
151011	10-Sep	septin 10	1.00609	0.967673	0.992296
151050	C2orf67	chromosome 2 open reading frame 67	0.9787	1.003219	0.9803599
151056	PLB1	phospholipase B1	0.99793	1.00515	1.0078712

151112	ZSWIM2	zinc finger, SWIM-type containing 2	0.98993	0.999913	1.0498731
151126	ZNF385B	zinc finger protein 385B	1.00584	1.003551	1.0193797
151174	LOC151174	hypothetical LOC151174	1.00395	0.999287	1.021357
151176	FAM132B	family with sequence similarity 132, member B	1.0049	1.003721	1.0637919
151188	ARL6IP6	ADP-ribosylation-like factor 6 interacting protein 6	1.00797	0.968483	1.0557887
151194	METTL21A	methyltransferase like 21A	0.99505	0.977646	0.9214222
151195	CCNYL1	cyclin Y-like 1	1.00283	0.943393	0.9807196
151230	KLHL23	kelch-like 23 (Drosophila)	0.99348	0.989395	1.0124787
151234	SULT1C2P1	sulfotransferase family, cytosolic, 1C, member 2 pseudogene 1	0.99973	0.978314	1.0204082
151242	PPP1R1C	protein phosphatase 1, regulatory (inhibitor) subunit 1C	1.00186	1.026751	1.0180105
151246	SGOL2	shugoshin-like 2 (S. pombe)	0.99898	1.000778	1.0137333
151254	ALS2CR11	amyotrophic lateral sclerosis 2 (juvenile) chromosome region, candidate 11	1.00415	0.994414	1.0202229
151258	SLC38A11	solute carrier family 38, member 11	0.98748	1.0452	0.9601764
151276	LOC151276	thioredoxin 1 pseudogene 1	0.98926	1.035082	0.9706619
151278	CCDC140	coiled-coil domain containing 140	0.99536	1.011122	1.0323826
151295	SLC23A3	solute carrier family 23 (nucleobase transporters), member 3	1.02043	1.006918	1.0094
151300	LOC151300	hypothetical LOC151300	1.00316	0.977525	1.0058644
151306	GPBAR1	G protein-coupled bile acid receptor 1	0.99631	0.967345	1.0551484
151313	FAHD2B	fumarylacetoacetate hydrolase domain containing 2B	0.9775	0.971709	0.9138555
151325	MYADML	myeloid-associated differentiation marker-like	0.99513	0.957669	1.0263039
151354	FAM84A	family with sequence similarity 84, member A	1.00577	1.03324	1.0547662
151393	FAM82A1	family with sequence similarity 82, member A1	1.00055	0.947562	0.9861334
151449	GDF7	growth differentiation factor 7	0.98444	0.978701	1.0185323
151457	LOC151457	developmentally regulated GTP binding protein 1 pseudogene	0.96171	0.946598	0.9704227
151473	SLC16A14	solute carrier family 16, member 14 (monocarboxylic acid transporter 14)	1.00018	0.994765	1.004036
151477	C2orf52	chromosome 2 open reading frame 52	1.01632	1.050444	1.0144445
151507	MSL3P1	male-specific lethal 3 homolog (Drosophila) pseudogene 1	0.99477	1.018411	1.0673594
151525	WDSUB1	WD repeat, sterile alpha motif and U-box domain containing 1	1.01643	0.994748	1.0662082
151531	UPP2	uridine phosphorylase 2	1.00085	1.022022	1.0197628
151534	LOC151534	hypothetical LOC151534	1.00097	1.016287	1.0424964
151556	GPR155	G protein-coupled receptor 155	0.97867	0.918072	0.8691312
151613	TTC14	tetratricopeptide repeat domain 14	0.99485	0.962354	0.922792
151636	DTX3L	deltex 3-like (Drosophila)	0.97854	0.940549	1.0139393

151647	FAM19A4	family with sequence similarity 19 (chemokine (C-C motif)-like), member A4	0.99935	0.999724	1.0137403
151648	SGOL1	shugoshin-like 1 (S. pombe)	0.98132	1.021636	1.015764
151649	C3orf48	chromosome 3 open reading frame 48	0.9946	0.994296	1.0302188
151651	EFHB	EF-hand domain family, member B	1.02277	0.98685	0.9946108
151742	PPM1L	protein phosphatase, Mg2+/Mn2+ dependent, 1L	1.02998	0.971042	0.9560835
151760	LOC151760	hypothetical LOC151760	1.00458	0.98277	1.0095297
151790	WDR49	WD repeat domain 49	1.01729	0.970552	1.0338849
151825	KRT18P43	keratin 18 pseudogene 43	0.97857	0.99628	0.9892876
151827	LRRC34	leucine rich repeat containing 34	1.0107	0.97908	0.9926049
151835	CPNE9	copine family member IX	1.00247	1.01569	1.0211316
151842	PP1P	pyrophosphatase (inorganic) 1 pseudogene	0.96034	1.008724	1.0293956
151871	DPPA2	developmental pluripotency associated 2	0.99671	1.000861	0.9937389
151887	CCDC80	coiled-coil domain containing 80	1.00997	1.004096	1.0146332
151888	BTLA	B and T lymphocyte associated	0.97614	0.989215	0.9709718
151903	CCDC12	coiled-coil domain containing 12	1.02688	1.005836	1.0655581
151963	MB21D2	Mab-21 domain containing 2	1.00889	0.984089	1.022526
151987	PPP4R2	protein phosphatase 4, regulatory subunit 2	1.00581	0.991462	0.9290608
152002	C3orf21	chromosome 3 open reading frame 21	1.00678	0.992068	1.0181378
152006	RNF38	ring finger protein 38	0.94719	0.899631	0.9390156
152007	GLIPR2	GLI pathogenesis-related 2	0.9589	0.926883	0.9454228
152078	C3orf55	chromosome 3 open reading frame 55	1.02863	0.99946	1.0015277
152098	ZCWPW2	zinc finger, CW type with PWWP domain 2	0.97649	1.024404	1.0290221
152100	CMC1	COX assembly mitochondrial protein homolog (S. cerevisiae)	0.9836	0.991562	0.9904951
152101	VENTXP4	VENT homeobox pseudogene 4	0.99041	0.944213	0.9377492
152110	NEK10	NIMA (never in mitosis gene a)- related kinase 10	1.00183	0.972317	1.008617
152118	C3orf79	chromosome 3 open reading frame 79	1.0089	1.033849	1.0362134
152137	CCDC50	coiled-coil domain containing 50	1.0307	0.927505	1.0929377
152185	SPICE1	spindle and centriole associated protein 1	0.96262	0.945466	1.016987
152189	CMTM8	CKLF-like MARVEL transmembrane domain containing 8	0.99315	0.984949	0.9446739
152195	NUDT16P1	nudix (nucleoside diphosphate linked moiety X)-type motif 16 pseudogene 1	1.00126	0.951762	1.0673745
152206	CCDC13	coiled-coil domain containing 13	0.98949	1.029143	0.99895
152217	LOC152217	hypothetical LOC152217	0.96682	0.919516	0.9151565
152225	LOC152225	hypothetical LOC152225	1.01528	0.993702	1.0448379

152273	FGD5	FYVE, RhoGEF and PH domain containing 5	1.00054	0.992912	0.9901046
152302	CIDECP	cell death-inducing DFFA-like effector c pseudogene	0.97033	1.015875	0.9676704
152330	CNTN4	contactin 4	1.00748	0.990159	1.0184385
152405	C3orf30	chromosome 3 open reading frame 30	0.99285	0.935721	0.9947928
152485	ZNF827	zinc finger protein 827	0.99062	1.042999	1.0196766
152503	SH3D19	SH3 domain containing 19	1.00703	0.995592	0.986458
152518	NFXL1	nuclear transcription factor, X-box binding-like 1	1.00281	0.988381	0.9879123
152519	NIPAL1	NIPA-like domain containing 1	1.02412	1.019342	1.0408191
152559	PAQR3	progestin and adipoQ receptor family member III	0.99301	0.942137	0.9457628
152573	SHISA3	shisa homolog 3 (Xenopus laevis)	0.99417	1.016042	1.0315974
152579	SCFD2	sec1 family domain containing 2	0.99086	0.947507	0.9941583
152586	LOC152586	glycosyltransferase 54 domain-containing protein	1.00742	0.997148	1.0050718
152594	LOC152594	NIN1/RPN12 binding protein 1 pseudogene	1.01457	0.99773	1.0427839
152663	RPL7AP27	ribosomal protein L7a pseudogene 27	0.9754	0.977838	1.0064375
152667	FAM192BP	family with sequence similarity 192, member B pseudogene	1.02877	1.005891	0.961814
152687	ZNF595	zinc finger protein 595	1.02243	0.995284	0.9447822
152742	LOC152742	hypothetical LOC152742	1.00976	0.975431	1.0075391
152756	C4orf39	chromosome 4 open reading frame 39	1.021	0.934604	1.0272663
152789	JAKMIP1	janus kinase and microtubule interacting protein 1	1.00284	1.082895	1.0479075
152815	THAP6	THAP domain containing 6	0.9498	0.942134	1.0408793
152831	KLB	klotho beta	1.007	1.007322	1.0216069
152845	LOC152845	pleiomorphic adenoma gene-like 2 pseudogene	1.02161	0.956525	0.9495551
152877	FAM53A	family with sequence similarity 53, member A	1.00918	1.00384	1.0169267
152926	PPM1K	protein phosphatase, Mg2+/Mn2+ dependent, 1K	1.00988	0.967407	0.9605274
152940	C4orf45	chromosome 4 open reading frame 45	1.00353	0.997569	0.9802455
152992	METTL19	methyltransferase like 19	0.99302	0.953274	1.0025246
153020	RASGEF1B	RasGEF domain family, member 1B	0.99455	1.117247	0.9461747
153090	DAB2IP	DAB2 interacting protein	0.99871	0.984492	1.0164481
153129	SLC38A9	solute carrier family 38, member 9	0.98412	0.958857	0.9640353
153201	SLC36A2	solute carrier family 36 (proton/amino acid symporter), member 2	1.00819	1.008072	0.9954726
153218	SPINK13	serine peptidase inhibitor, Kazal type 13 (putative)	1.01109	1.028708	1.0354616
153222	C5orf41	chromosome 5 open reading frame 41	0.96952	0.969026	0.9933721
153241	CEP120	centrosomal protein 120kDa	0.96936	0.99577	0.9451331

153328	SLC25A48	solute carrier family 25, member 48	1.0031	1.008273	1.029694
153339	TMEM167A	transmembrane protein 167A	1.03031	0.902154	1.0598106
153364	MBLAC2	metallo-beta-lactamase domain containing 2	0.97105	0.967455	0.9324614
153396	TMEM161B	transmembrane protein 161B	0.9672	0.977912	1.0313367
153443	SRFBP1	serum response factor binding protein 1	0.94227	0.932513	1.0660422
153469	LOC153469	hypothetical protein LOC153469	1.01041	0.991883	1.0132203
153478	PLEKHG4B	pleckstrin homology domain containing, family G (with RhoGef domain) member 4B	1.00899	0.997458	1.0138325
153527	ZMAT2	zinc finger, matrin-type 2	0.98886	0.852261	0.9824418
153562	MARVELD2	MARVEL domain containing 2	0.99669	0.993202	0.9917684
153571	C5orf38	chromosome 5 open reading frame 38	1.01954	1.043831	1.0118391
153572	IRX2	iroquois homeobox 2	0.98394	1.034225	1.0871959
153579	BTNL9	butyrophilin-like 9	1.00544	1.004337	1.0227728
153642	ARSK	arylsulfatase family, member K	0.96865	0.943754	1.0622518
153643	FAM81B	family with sequence similarity 81, member B	0.98392	0.964035	0.9873305
153657	TTC23L	tetratricopeptide repeat domain 23-like	1.00826	0.98434	1.0059386
153684	LOC153684	hypothetical LOC153684	0.98014	0.945896	0.9522821
153733	CCDC112	coiled-coil domain containing 112	1.0015	0.976905	1.0119626
153743	PPP1R2P3	protein phosphatase 1, regulatory (inhibitor) subunit 2 pseudogene 3	0.98819	0.955876	1.0008128
153745	FAM71B	family with sequence similarity 71, member B	0.98429	0.996514	1.0100244
153768	PRELID2	PRELI domain containing 2	0.99813	0.948571	0.9986238
153769	SH3RF2	SH3 domain containing ring finger 2	1.00821	1.006121	0.987364
153770	PLAC8L1	PLAC8-like 1	0.98613	0.992513	1.0281588
153830	RNF145	ring finger protein 145	1.02038	0.989846	0.9306758
153893	LOC153893	DCN1, defective in cullin neddylation 1, domain containing 1 (S. cerevisiae) pseudogene	0.97186	0.943487	1.0349446
153918	C6orf94	chromosome 6 open reading frame 94	0.98872	1.06476	1.0452889
153932	LOC153932	signal recognition particle 72kD pseudogene	1.01872	1.053268	0.9462864
154007	SNRNP48	small nuclear ribonucleoprotein 48kDa (U11/U12)	1.00939	0.920647	1.0509165
154043	CNKSR3	CNKSR family member 3	1.00195	1.001776	1.001636
154064	RAET1L	retinoic acid early transcript 1L	1.02339	1.041725	0.9841346
154075	SAMD3	sterile alpha motif domain containing 3	0.97442	1.07142	0.9135868
154091	SLC2A12	solute carrier family 2 (facilitated glucose transporter), member 12	0.99982	1.012269	1.0519424
154141	MBOAT1	membrane bound O-acyltransferase domain containing 1	1.00865	0.971671	1.0490293
154150	HDGFL1	hepatoma derived growth factor-like 1	0.98788	0.983705	1.0078026

154197	PNLDC1	poly(A)-specific ribonuclease (PARN)-like domain containing 1	1.01052	1.016629	1.0159261
154214	RNF217	ring finger protein 217	1.01004	0.972113	0.9946272
154215	NKAIN2	Na+/K+ transporting ATPase interacting 2	1.00599	1.004705	1.0105482
154288	C6orf221	chromosome 6 open reading frame 221	1.00635	1.008661	1.0092064
154313	C6orf165	chromosome 6 open reading frame 165	1.00073	1.002685	1.0004243
154386	C6orf195	chromosome 6 open reading frame 195	0.98401	0.976801	0.9677447
154449	LOC154449	hypothetical LOC154449	1.03051	1.069327	0.9794969
154467	C6orf129	chromosome 6 open reading frame 129	0.98091	1.003445	1.0224584
154661	RUNDC3B	RUN domain containing 3B	0.98677	1.020504	1.037448
154664	ABCA13	ATP-binding cassette, sub-family A (ABC1), member 13	1.00679	0.997733	1.0095951
154743	C7orf60	chromosome 7 open reading frame 60	0.97332	1.026526	1.086262
154790	CLEC2L	C-type lectin domain family 2, member L	0.99581	0.947208	1.0087468
154791	C7orf55	chromosome 7 open reading frame 55	0.99694	0.947702	1.0307983
154796	AMOT	angiomotin	0.99033	0.986205	1.0010868
154807	VKORC1L1	vitamin K epoxide reductase complex, subunit 1-like 1	0.99291	0.965415	1.0153768
154810	AMOTL1	angiomotin like 1	0.98657	1.005176	1.0031173
154860	LOC154860	hypothetical LOC154860	1.01477	1.000525	1.0961758
154865	IQUB	IQ motif and ubiquitin domain containing	0.99154	1.011979	0.9849583
154872	LOC154872	hypothetical protein LOC154872	1.01555	1.020868	0.9856307
154881	KCTD7	potassium channel tetramerisation domain containing 7	0.96874	0.968977	0.8808038
154907	C7orf66	chromosome 7 open reading frame 66	1.04065	0.989394	0.9434379
155006	TMEM213	transmembrane protein 213	0.99528	1.009607	1.0308946
155019	ST13P7	suppression of tumorigenicity 13 (colon carcinoma) (Hsp70 interacting protein) pseudogene 7	1.00397	0.977954	1.0449951
155038	GIMAP8	GTPase, IMAP family member 8	1.00622	0.895768	0.9863509
155051	CRYGN	crystallin, gamma N	0.9861	0.968941	1.0429792
155054	ZNF425	zinc finger protein 425	0.98454	1.022927	1.0269843
155060	LOC155060	AI894139 pseudogene	0.99269	0.964031	0.9892874
155061	ZNF746	zinc finger protein 746	0.9708	0.987964	0.9934427
155066	ATP6V0E2	ATPase, H+ transporting V0 subunit e2	0.98015	0.984578	1.0192
155184	SLC2A7	solute carrier family 2 (facilitated glucose transporter), member 7	0.98652	1.00774	1.0192097
155185	AMZ1	archaelysin family metallopeptidase 1	1.01304	1.009586	1.0395891
155368	WBSCR27	Williams Beuren syndrome chromosome region 27	0.99324	0.980529	1.0227102
155370	SBDSP1	Shwachman-Bodian-Diamond syndrome pseudogene 1	0.98457	0.969651	0.9425594

155382	VPS37D	vacuolar protein sorting 37 homolog D (S. cerevisiae)	0.98068	1.031748	1.0784387
155435	RBM33	RNA binding motif protein 33	0.99686	0.939712	0.9135268
155465	AGR3	anterior gradient homolog 3 (Xenopus laevis)	0.98109	1.001048	1.0132619
157285	SGK223	homolog of rat pragma of Rnd2	0.96503	0.949204	1.0363854
157310	PEBP4	phosphatidylethanolamine-binding protein 4	0.99617	1.005515	1.001108
157313	CDCA2	cell division cycle associated 2	1.0023	0.991274	1.021668
157317	CYCSP55	cytochrome c, somatic pseudogene 55	0.99247	1.007356	0.9334843
157378	TMEM65	transmembrane protein 65	0.93214	1.008674	0.9935432
157489	SDAD1P1	SDA1 domain containing 1 pseudogene 1	0.95454	0.932874	0.9789339
157506	RDH10	retinol dehydrogenase 10 (all-trans)	0.98643	1.04462	0.9867994
157530	ST13P6	suppression of tumorigenicity 13 (colon carcinoma) (Hsp70 interacting protein) pseudogene 6	0.99307	0.992873	0.9627124
157567	ANKRD46	ankyrin repeat domain 46	0.93786	1.028264	1.0274503
157570	ESCO2	establishment of cohesion 1 homolog 2 (S. cerevisiae)	1.01869	1.014696	1.0166764
157574	FBXO16	F-box protein 16	0.99818	1.013774	1.0037855
157638	FAM84B	family with sequence similarity 84, member B	0.93343	0.990299	1.0146132
157657	C8orf37	chromosome 8 open reading frame 37	0.95296	0.974223	1.0486687
157667	LOC157667	myosin, light chain 12A, regulatory, non-sarcomeric pseudogene	0.91629	0.93356	1.0433091
157680	VPS13B	vacuolar protein sorting 13 homolog B (yeast)	1.01648	0.881798	0.9596478
157695	C8orf42	chromosome 8 open reading frame 42	0.97793	0.966943	1.0046166
157697	ERICH1	glutamate-rich 1	1.01938	1.023263	0.9961257
157708	SEC11B	SEC11 homolog B (S. cerevisiae)	1.00971	0.987511	1.0097968
157713	LOC157713	lysophospholipase I-like	0.98925	1.036474	1.0201819
157724	SLC7A13	solute carrier family 7, (cationic amino acid transporter, y+ system) member 13	0.99489	0.968505	1.0227049
157739	TDH	L-threonine dehydrogenase	1.01645	0.99011	1.025759
157740	LOC157740	hypothetical protein C8orf9	0.98225	1.018414	1.0589998
157753	TMEM74	transmembrane protein 74	0.99833	0.984655	1.0189323
157769	FAM91A1	family with sequence similarity 91, member A1	1.03326	0.906865	1.0684555
157773	C8orf48	chromosome 8 open reading frame 48	1.01936	0.957961	0.9838885
157777	C8orf45	chromosome 8 open reading frame 45	0.98018	0.981394	1.0266807
157848	NKX6-3	NK6 homeobox 3	0.98605	0.969779	1.064468
157855	KCNU1	potassium channel, subfamily U, member 1	1.01002	0.992929	1.0086011
157869	C8orf84	chromosome 8 open reading frame 84	0.98923	1.005958	1.0014561
157916	IL6RP1	interleukin 6 receptor pseudogene 1	1.02344	1.027967	0.9990901

157922	CAMSAP1	calmodulin regulated spectrin-associated protein 1	0.95342	0.989971	1.0710071
157927	C9orf62	chromosome 9 open reading frame 62	1.0208	0.992377	0.9847105
157956	CDC20P1	cell division cycle 20 homolog (S. cerevisiae) pseudogene 1	1.01212	1.020914	1.0355004
157983	C9orf66	chromosome 9 open reading frame 66	0.98185	0.962699	1.0436001
158038	LINGO2	leucine rich repeat and Ig domain containing 2	0.98808	0.994037	0.9806409
158046	NXNL2	nucleoredoxin-like 2	1.00674	1.030783	0.9955291
158055	C9orf163	chromosome 9 open reading frame 163	0.9748	1.01731	1.0248595
158056	MAMDC4	MAM domain containing 4	0.99509	1.020205	1.0258467
158062	LCN6	lipocalin 6	0.98872	0.996315	1.008557
158067	AK8	adenylate kinase 8	0.99237	0.984814	1.0069983
158104	RPS10P3	ribosomal protein S10 pseudogene 3	0.95609	1.044455	1.1286032
158131	OR1Q1	olfactory receptor, family 1, subfamily Q, member 1	1.00968	0.939264	0.9768173
158135	TTLL11	tubulin tyrosine ligase-like family, member 11	0.98739	0.983377	1.0309033
158158	RASEF	RAS and EF-hand domain containing	0.99274	1.00721	1.0104029
158219	TTC39B	tetratricopeptide repeat domain 39B	0.95324	0.963695	0.943524
158222	LDHAP4	lactate dehydrogenase A pseudogene 4	0.97729	1.008054	1.0198011
158234	RG9MTD3	RNA (guanine-9-) methyltransferase domain containing 3	0.92913	0.999519	1.0352323
158248	TTC16	tetratricopeptide repeat domain 16	0.98125	0.991392	0.9914558
158257	LOC158257	hypothetical LOC158257	0.99541	1.047184	1.0099107
158293	FAM120AC	family with sequence similarity 120A opposite strand	0.9386	0.938946	0.9431586
158297	FAM154A	family with sequence similarity 154, member A	0.99001	1.002354	0.9955771
158326	FREM1	FRAS1 related extracellular matrix 1	0.99972	0.996175	1.0018168
158345	RPL4P5	ribosomal protein L4 pseudogene 5	0.95462	0.957096	1.0775608
158358	KIAA2026	KIAA2026	0.94025	1.023098	0.9593638
158373	BA327L3.4	nuclease sensitive element binding protein 1 pseudogene	0.97121	0.958267	1.1454884
158376	LOC158376	hypothetical LOC158376	1.00737	0.983108	1.0408364
158381	ATP8B5P	ATPase, class I, type 8B, member 5, pseudogene	1	0.993139	1.0021513
158399	ZNF483	zinc finger protein 483	0.93816	0.928752	0.9457825
158401	C9orf84	chromosome 9 open reading frame 84	0.98093	0.997291	0.9975771
158405	KIAA1958	KIAA1958	1.00124	0.979558	1.0390603
158427	TSTD2	thiosulfate sulfurtransferase (rhodanese)-like domain containing 2	0.95899	1.000231	1.0004213
158431	ZNF782	zinc finger protein 782	0.94364	1.017129	1.0259387
158452	ASS1P3	argininosuccinate synthetase 1 pseudogene 3	0.99394	1.01031	1.0453243

158506	ZNF645	zinc finger protein 645	0.96392	0.984324	0.9870071
158511	CSAG1	chondrosarcoma associated gene 1	1.02766	1.088606	0.9856215
158521	FMR1NB	fragile X mental retardation 1 neighbor	0.98777	0.999199	0.9855303
158584	FAAH2	fatty acid amide hydrolase 2	0.97211	0.979881	0.954625
158586	ZXDB	zinc finger, X-linked, duplicated B	0.93749	0.877191	1.021288
158674	RPSAP3	ribosomal protein SA pseudogene 3	0.95585	0.975336	0.9712858
158714	[No Symbo	[No Name]	0.9723	0.922423	0.9877392
158724	FAM47A	family with sequence similarity 47, member A	0.98607	1.00017	1.0026612
158747	MOSPD2	motile sperm domain containing 2	0.96989	0.906161	1.0164542
158763	ARHGAP36	Rho GTPase activating protein 36	0.99793	0.997142	1.0177704
158787	RIBC1	RIB43A domain with coiled-coils 1	0.97502	0.994797	0.9642581
158798	AKAP14	A kinase (PRKA) anchor protein 14	1.00843	0.977485	1.0015675
158800	RHOXF1	Rhox homeobox family, member 1	1.0013	1.005134	1.0165887
158801	NKAPP1	NFKB activating protein pseudogene 1	0.98689	0.945435	1.035585
158809	MAGEB6	melanoma antigen family B, 6	1.01773	1.014149	1.0030648
158830	CXorf65	chromosome X open reading frame 65	1.00095	1.058832	0.8792714
158833	AWAT1	acyl-CoA wax alcohol acyltransferase 1	1.01272	1.030254	1.0130529
158835	AWAT2	acyl-CoA wax alcohol acyltransferase 2	1.00711	0.979426	1.0276951
158866	ZDHHC15	zinc finger, DHHC-type containing 15	0.99119	0.977597	0.9686661
158880	USP51	ubiquitin specific peptidase 51	0.96766	0.962266	1.0140533
158905	PHF10P1	PHD finger protein 10 pseudogene 1	0.96309	0.964789	0.9533423
158931	TCEAL6	transcription elongation factor A (SII)-like 6	1.00428	0.987789	1.0384412
158948	LOC158948	protein kinase C, iota pseudogene	0.94095	0.969379	1.0907314
158983	H2BFWT	H2B histone family, member W, testis-specific	0.97313	0.947588	0.9794438
159007	LOC159007	aldo-keto reductase family 7, member A2 (aflatoxin aldehyde reductase) pseudogene	1.00679	0.994237	1.0092655
159013	CXorf38	chromosome X open reading frame 38	0.94388	0.931149	1.0259639
159090	FAM122B	family with sequence similarity 122B	0.98417	0.95701	1.0052736
159091	FAM122C	family with sequence similarity 122C	0.95706	0.968695	1.0093365
159125	RBMY2EP	RNA binding motif protein, Y-linked, family 2, member E pseudogene	0.98799	0.975171	1.0152885
159162	RBMY2FP	RNA binding motif protein, Y-linked, family 2, member F pseudogene	1.00965	1.044271	0.9955726
159195	USP54	ubiquitin specific peptidase 54	0.97807	1.001753	1.0198614
159296	NKX2-3	NK2 homeobox 3	0.98635	1.017783	1.0613541
159371	TMEM20	transmembrane protein 20	0.98341	0.996798	1.0136416

159491	[No Symbo	[No Name]	1.00158	1.04287	0.9796698
159686	CCDC147	coiled-coil domain containing 147	0.98542	1.00407	0.9728206
159770	CBX3P1	chromobox homolog 3 pseudogene 1	0.98575	0.98991	1.0358137
159963	SLC5A12	solute carrier family 5 (sodium/glucose cotransporter), member 12	1.00569	1.00544	1.0226724
159989	CCDC67	coiled-coil domain containing 67	0.99872	0.993861	1.0076558
160065	PATE1	prostate and testis expressed 1	1.00628	1.018594	1.0067843
160140	C11orf65	chromosome 11 open reading frame 65	0.98102	0.992107	1.049782
160287	LDHAL6A	lactate dehydrogenase A-like 6A	0.99888	1.001789	1.0338944
160298	C11orf42	chromosome 11 open reading frame 42	0.98008	0.979753	1.0113806
160335	TMTC2	transmembrane and tetratricopeptide repeat containing 2	0.9914	0.999726	1.0497095
160364	CLEC12A	C-type lectin domain family 12, member A	0.98011	0.976371	1.0440434
160365	CLECL1	C-type lectin-like 1	0.9756	1.036751	0.995608
160410	PSMC6P2	proteasome 26S subunit, ATPase, 6 pseudogene 2	1.00917	0.972942	0.9826455
160418	TMTC3	transmembrane and tetratricopeptide repeat containing 3	0.90945	1.012204	1.0909578
160419	C12orf50	chromosome 12 open reading frame 50	0.97674	1.001411	0.9965598
160428	ALDH1L2	aldehyde dehydrogenase 1 family, member L2	0.99032	0.996782	1.0014984
160492	IFLTD1	intermediate filament tail domain containing 1	1.0006	1.006994	1.0017233
160518	DENND5B	DENN/MADD domain containing 5B	0.96918	0.988686	0.9953804
160622	GRASP	GRP1 (general receptor for phosphoinositides 1)-associated scaffold protein	0.99683	1.012959	0.9909693
160728	SLC5A8	solute carrier family 5 (iodide transporter), member 8	0.98318	1.005831	0.9947973
160760	PPTC7	PTC7 protein phosphatase homolog (S. cerevisiae)	0.95977	0.922625	1.0876924
160762	CCDC63	coiled-coil domain containing 63	0.98588	0.997659	0.9889945
160777	CCDC60	coiled-coil domain containing 60	0.99824	0.984736	1.0077846
160824	SP3P	Sp3 transcription factor pseudogene	0.98403	1.012829	1.0041776
160851	DGKH	diacylglycerol kinase, eta	0.95403	0.962762	1.0048441
160857	CCDC122	coiled-coil domain containing 122	0.98575	0.930659	0.98181
160897	GPR180	G protein-coupled receptor 180	0.95481	0.963833	1.0863207
161003	STOML3	stomatin (EPB72)-like 3	1.01238	0.984621	1.0127352
161142	FAM71D	family with sequence similarity 71, member D	0.98326	1.005533	1.0096674
161145	TMEM229E	transmembrane protein 229B	0.98372	0.973439	0.9553631
161176	C14orf49	chromosome 14 open reading frame 49	0.96184	0.969527	1.0246147
161247	FITM1	fat storage-inducing transmembrane protein 1	0.99961	1.005645	0.9882881
161253	REM2	RAS (RAD and GEM)-like GTP binding 2	0.98415	1.038636	0.9747041

161291	TMEM30B	transmembrane protein 30B	0.95249	0.892691	0.9313012
161357	MDGA2	MAM domain containing glycosylphosphatidylinositol anchor 2	0.98374	1.010068	0.9856892
161394	SAMD15	sterile alpha motif domain containing 15	0.99179	0.966978	0.9713775
161424	C14orf21	chromosome 14 open reading frame 21	0.96898	0.959191	1.0122273
161436	EML5	echinoderm microtubule associated protein like 5	0.96348	0.975618	0.9239072
161497	STRC	stereocilin	0.99234	0.975897	1.124964
161502	C15orf26	chromosome 15 open reading frame 26	0.95431	0.961686	0.9252933
161514	TBC1D21	TBC1 domain family, member 21	1.01329	0.992952	1.0009644
161582	DYX1C1	dyslexia susceptibility 1 candidate 1	1.00638	0.998021	1.0236009
161725	OTUD7A	OTU domain containing 7A	0.98971	1.001656	1.0380284
161742	SPRED1	sprouty-related, EVH1 domain containing 1	1.02692	0.966587	1.0502438
161753	ODF3L1	outer dense fiber of sperm tails 3-like 1	0.99381	1.000611	1.0527244
161779	PGBD4	piggyBac transposable element derived 4	0.93846	0.89471	1.0406478
161823	ADAL	adenosine deaminase-like	0.95305	1.01619	1.0018957
161829	EXD1	exonuclease 3'-5' domain containing 1	1.00655	0.994626	0.9852478
161835	FSIP1	fibrous sheath interacting protein 1	1.00072	0.999906	1.0046401
161882	ZFPM1	zinc finger protein, multitype 1	0.98983	1.028818	0.9552481
161931	ADAD2	adenosine deaminase domain containing 2	0.99673	0.990785	1.0859713
162073	ITPRIPL2	inositol 1,4,5-trisphosphate receptor interacting protein-like 2	0.95698	1.049463	1.0589311
162083	C16orf82	chromosome 16 open reading frame 82	0.99135	0.976153	1.0048313
162137	MGC34800	hypothetical protein MGC34800	1.00021	1.038007	1.0732866
162239	ZFP1	zinc finger protein 1 homolog (mouse)	0.9171	0.986991	1.1159332
162282	ANKFN1	ankyrin-repeat and fibronectin type III domain containing 1	1.00424	0.9891	0.9936947
162333	10-Mar	membrane-associated ring finger (C3HC4) 10	0.99945	0.993558	0.9942705
162387	MFSD6L	major facilitator superfamily domain containing 6-like	0.99572	1.023135	1.0050395
162394	SLFN5	schlafen family member 5	0.95653	1.07858	0.9047322
162417	NAGS	N-acetylglutamate synthase	0.96183	1.053927	1.0640568
162427	FAM134C	family with sequence similarity 134, member C	0.96632	0.92952	1.0554965
162461	TMEM92	transmembrane protein 92	0.99699	1.069776	1.0440737
162466	PHOSPHO1	phosphatase, orphan 1	0.97842	0.920939	0.9763684
162494	RHBDL3	rhomboid, veinlet-like 3 (Drosophila)	1.01384	0.942073	0.9533112
162514	TRPV3	transient receptor potential cation channel, subfamily V, member 3	1.00868	0.973453	0.9918814
162517	FBXO39	F-box protein 39	1.00768	0.974899	1.0403552

162540	IMP5	intramembrane protease 5	1.00662	0.959157	0.9764417
162605	KRT28	keratin 28	0.99571	1.0367	1.007027
162655	ZNF519	zinc finger protein 519	0.96841	0.996787	1.0418879
162681	C18orf54	chromosome 18 open reading frame 54	0.96604	0.975431	1.0268441
162962	ZNF836	zinc finger protein 836	0.93701	0.958489	0.9926592
162963	ZNF610	zinc finger protein 610	0.9916	1.011729	0.9912642
162966	ZNF600	zinc finger protein 600	0.98826	0.969673	0.9942291
162967	ZNF320	zinc finger protein 320	0.95317	0.973085	0.9686143
162968	ZNF497	zinc finger protein 497	0.99789	1.009297	1.0500668
162972	ZNF550	zinc finger protein 550	0.91241	0.978941	0.9775021
162979	ZNF296	zinc finger protein 296	0.96908	0.921377	1.0842505
162989	DEDD2	death effector domain containing 2	0.97902	0.984505	0.9953221
162993	ZNF846	zinc finger protein 846	0.93231	0.952978	0.9056264
162998	OR7D2	olfactory receptor, family 7, subfamily D, member 2	0.97804	1.017605	1.0253132
163033	ZNF579	zinc finger protein 579	0.9746	1.004363	1.0828794
163049	ZNF791	zinc finger protein 791	0.88735	1.009853	1.077998
163050	ZNF564	zinc finger protein 564	0.95053	0.992643	1.0438919
163051	ZNF709	zinc finger protein 709	0.94581	0.950174	1.0248468
163059	ZNF433	zinc finger protein 433	0.98694	0.983216	0.9747747
163071	ZNF114	zinc finger protein 114	0.99132	0.990614	1.000045
163081	ZNF567	zinc finger protein 567	0.93215	1.052277	1.0072042
163087	ZNF383	zinc finger protein 383	0.93109	1.032854	1.0510148
163088	TAF9P3	TAF9 RNA polymerase II, TATA box binding protein (TBP)-associated factor, 32kDa pseudogene 3	0.97882	0.972965	1.0596062
163115	ZNF781	zinc finger protein 781	0.96701	1.026407	0.9569222
163126	EID2	EP300 interacting inhibitor of differentiation 2	0.98448	0.903713	0.9997338
163131	ZNF780B	zinc finger protein 780B	0.89685	1.000503	0.9120801
163154	PRR22	proline rich 22	0.98408	1.063762	0.9973048
163175	LGI4	leucine-rich repeat LGI family, member 4	1.00764	1.032106	0.9909643
163183	C19orf46	chromosome 19 open reading frame 46	1.0298	0.993782	1.0324421
163223	ZNF676	zinc finger protein 676	0.9734	0.972625	1.0172334
163227	ZNF100	zinc finger protein 100	0.99163	1.009291	0.974803
163255	ZNF540	zinc finger protein 540	0.96861	1.001055	0.9769237
163259	DENND2C	DENN/MADD domain containing 2C	0.9644	0.902472	0.9906515

163351	GBP6	guanylate binding protein family, member 6	0.99335	1.010045	1.0197009
163404	LPPR5	lipid phosphate phosphatase-related protein type 5	0.99135	1.025944	1.0226419
163479	FNDC7	fibronectin type III domain containing 7	1.01606	1.021367	0.9725089
163486	DENND1B	DENN/MADD domain containing 1B	0.97069	0.963003	0.9847423
163589	TDRD5	tudor domain containing 5	0.98885	0.987684	1.011597
163590	TOR1AIP2	torsin A interacting protein 2	0.9599	0.99553	1.008536
163688	CALML6	calmodulin-like 6	0.99047	0.97151	0.9849826
163702	IL28RA	interleukin 28 receptor, alpha (interferon, lambda receptor)	0.99701	1.012545	1.006394
163720	CYP4Z2P	cytochrome P450, family 4, subfamily Z, polypeptide 2 pseudogene	1.04405	1.020129	1.0002259
163732	CITED4	Cbp/p300-interacting transactivator, with Glu/Asp-rich carboxy-terminal domain, 4	0.96791	1.006449	0.9237794
163742	FLJ40434	hypothetical FLJ40434	0.98138	0.971288	1.0419119
163747	C1orf177	chromosome 1 open reading frame 177	0.98857	0.988937	1.032505
163778	SPRR4	small proline-rich protein 4	1.05228	1.022858	1.078806
163782	KANK4	KN motif and ankyrin repeat domains 4	0.99704	0.990746	1.0036753
163786	SASS6	spindle assembly 6 homolog (C. elegans)	0.9744	1.052733	1.0053089
163859	C1orf55	chromosome 1 open reading frame 55	0.94908	0.847574	0.8934007
163882	CNST	consortin, connexin sorting protein	0.97903	0.916742	0.9502998
163933	FAM43B	family with sequence similarity 43, member B	1.00919	1.025978	1.016894
164045	HFM1	HFM1, ATP-dependent DNA helicase homolog (S. cerevisiae)	1.00035	0.996521	0.9986493
164091	PAQR7	progestin and adipoQ receptor family member VII	1.03824	0.984389	1.0361394
164118	TTC24	tetratricopeptide repeat domain 24	0.99953	1.038486	1.0063705
164127	C1orf65	chromosome 1 open reading frame 65	1.02053	0.986568	0.9528071
164153	UBL4B	ubiquitin-like 4B	0.99947	0.987429	1.0304926
164237	WFDC13	WAP four-disulfide core domain 13	1.03344	1.017289	1.0143652
164284	APCDD1L	adenomatosis polyposis coli down-regulated 1-like	1.00072	0.985352	1.0263336
164312	LRRN4	leucine rich repeat neuronal 4	1.00053	1.010405	1.0573099
164380	CSTT	cystatin pseudogene	0.99578	1.014461	1.0239726
164395	TTLL9	tubulin tyrosine ligase-like family, member 9	0.99801	0.982894	1.0215467
164633	CABP7	calcium binding protein 7	0.99933	0.965456	1.0505652
164656	TMPRSS6	transmembrane protease, serine 6	0.98845	1.011295	1.0160646
164668	APOBEC3H	apolipoprotein B mRNA editing enzyme, catalytic polypeptide-like 3H	0.96624	1.040144	1.0321726
164684	WBP2NL	WBP2 N-terminal like	0.98949	1.004804	1.0430861
164714	TTLL8	tubulin tyrosine ligase-like family, member 8	0.99565	1.008057	1.0414592

164781	WDR69	WD repeat domain 69	1.00093	0.995652	0.9959514
164832	LONRF2	LON peptidase N-terminal domain and ring finger 2	1.01078	0.985238	1.0181869
165055	CCDC138	coiled-coil domain containing 138	0.98864	1.054229	1.0574543
165057	FLJ39061	hypothetical protein FLJ39061	0.99871	1.013045	1.0227422
165082	GPR113	G protein-coupled receptor 113	1.00955	1.006936	1.0147072
165100	C2orf57	chromosome 2 open reading frame 57	0.9938	1.042737	0.9993951
165140	OXER1	oxoeicosanoid (OXE) receptor 1	1.03046	0.970294	1.0291204
165186	FAM179A	family with sequence similarity 179, member A	1.02433	1.035548	0.9854422
165215	FAM171B	family with sequence similarity 171, member B	0.99292	0.995656	1.0291443
165257	C1QL2	complement component 1, q subcomponent-like 2	1.00528	1.006254	0.963009
165324	UBXN2A	UBX domain protein 2A	1.00506	0.867984	1.003391
165530	CLEC4F	C-type lectin domain family 4, member F	1.01211	0.992243	1.0086229
165545	DQX1	DEAQ box RNA-dependent ATPase 1	1.02416	1.02319	1.0231829
165631	PARP15	poly (ADP-ribose) polymerase family, member 15	1.02593	1.092774	0.9815358
165679	C3orf57	chromosome 3 open reading frame 57	0.98483	0.948999	1.0056692
165721	DNAJB8	DnaJ (Hsp40) homolog, subfamily B, member 8	1.00018	1.011234	0.9924514
165829	GPR156	G protein-coupled receptor 156	1.01242	1.00127	0.9946644
165904	XIRP1	xin actin-binding repeat containing 1	0.99229	0.978457	1.0341513
165918	RNF168	ring finger protein 168	0.97105	1.003665	1.0864807
166012	CHST13	carbohydrate (chondroitin 4) sulfotransferase 13	0.99704	0.980181	1.0550209
166070	DDX50P2	DEAD/H (Asp-Glu-Ala-Asp/His) box polypeptide 50 pseudogene 2	1.01415	1.060199	1.0009974
166336	PRICKLE2	prickle homolog 2 (Drosophila)	1.01142	0.981929	1.0109282
166348	KBTBD12	kelch repeat and BTB (POZ) domain containing 12	0.9945	0.99634	1.0085509
166378	SPATA5	spermatogenesis associated 5	0.97858	0.94465	1.0163417
166379	BBS12	Bardet-Biedl syndrome 12	0.9841	0.945886	0.961441
166614	DCLK2	doublecortin-like kinase 2	0.99092	0.99877	1.0088714
166647	GPR125	G protein-coupled receptor 125	0.99756	0.968444	1.0085976
166655	TRIM60	tripartite motif containing 60	0.98452	0.992071	1.0249406
166752	FREM3	FRAS1 related extracellular matrix 3	0.99701	0.982298	1.0143647
166785	MMAA	methylmalonic aciduria (cobalamin deficiency) cblA type	0.98764	0.909405	1.061316
166793	ZBTB49	zinc finger and BTB domain containing 49	0.98713	1.016841	1.0481837
166815	TIGD2	tigger transposable element derived 2	0.96534	0.912816	1.0591673
166824	RASSF6	Ras association (RalGDS/AF-6) domain family member 6	1.00035	0.96896	0.9996177

166863	RBM46	RNA binding motif protein 46	1.00584	1.004863	1.0295932
166929	SGMS2	sphingomyelin synthase 2	0.95137	0.880182	0.9823039
166968	MIER3	mesoderm induction early response 1, family member 3	0.95908	0.865023	0.9822493
166979	CDC20B	cell division cycle 20 homolog B (S. cerevisiae)	1.00939	1.021419	1.016484
167127	UGT3A2	UDP glycosyltransferase 3 family, polypeptide A2	1.01746	0.990324	1.0087064
167153	PAPD4	PAP associated domain containing 4	0.9979	0.914248	0.9535372
167227	DCP2	DCP2 decapping enzyme homolog (S. cerevisiae)	0.9952	0.918494	1.0387304
167359	NIM1	serine/threonine-protein kinase NIM1	1.0133	1.023935	1.0253437
167410	LIX1	Lix1 homolog (chicken)	1.00822	1.032494	1.0043522
167465	ZNF366	zinc finger protein 366	1.00753	1.015225	1.0230891
167555	FAM151B	family with sequence similarity 151, member B	1.01618	0.993343	0.992483
167681	PRSS35	protease, serine, 35	1.00126	1.016846	1.0537204
167691	LCA5	Leber congenital amaurosis 5	0.97762	1.009016	1.0083527
167826	OLIG3	oligodendrocyte transcription factor 3	0.99032	0.979729	1.0002436
167838	TXLNB	taxilin beta	0.99935	0.997793	1.0293954
168002	DACT2	dapper, antagonist of beta-catenin, homolog 2 (Xenopus laevis)	1.0113	1.01677	1.0411459
168090	C6orf118	chromosome 6 open reading frame 118	0.99009	0.990495	1.046057
168374	ZNF92	zinc finger protein 92	0.96859	0.896485	1.047884
168391	GALNTL5	UDP-N-acetyl-alpha-D-galactosamine:polypeptide N-acetylgalactosaminyltransferase-like 5	1.01279	1.005118	0.9999539
168400	DDX53	DEAD (Asp-Glu-Ala-Asp) box polypeptide 53	0.99511	1.043609	1.0251656
168417	ZNF679	zinc finger protein 679	1.01828	1.007472	1.024675
168451	THAP5	THAP domain containing 5	0.94491	0.902273	0.9126039
168455	FLJ36031	hypothetical protein FLJ36031	0.94893	1.028287	0.9592057
168474	SEPHS1P	selenophosphate synthetase pseudogene	1.00663	0.983369	0.9514478
168507	PKD1L1	polycystic kidney disease 1 like 1	1.01502	0.996926	1.0021014
168537	GIMAP7	GTPase, IMAP family member 7	0.95847	0.878508	0.889238
168544	ZNF467	zinc finger protein 467	0.99595	1.006652	0.9703574
168620	BHLHA15	basic helix-loop-helix family, member a15	1.02586	1.036382	1.0312999
168741	PER4	period homolog 3 (Drosophila) pseudogene	1.01901	1.034809	1.0264976
168850	ZNF800	zinc finger protein 800	0.98755	0.902854	1.0388714
168975	CNBD1	cyclic nucleotide binding domain containing 1	1.00568	1.003291	1.028523
169026	SLC30A8	solute carrier family 30 (zinc transporter), member 8	0.99929	0.983763	0.9991886
169044	COL22A1	collagen, type XXII, alpha 1	1.01313	0.994392	0.9903864

169166	SNX31	sorting nexin 31	0.98974	1.010112	1.018163
169200	TMEM64	transmembrane protein 64	0.9338	1.023442	1.0514012
169270	ZNF596	zinc finger protein 596	0.9909	1.013561	1.0130696
169355	IDO2	indoleamine 2,3-dioxygenase 2	1.01013	1.01495	1.0021491
169436	C9orf96	chromosome 9 open reading frame 96	0.98848	1.000837	1.0007354
169522	KCNV2	potassium channel, subfamily V, member 2	0.98597	0.996381	1.0128222
169611	OLFML2A	olfactomedin-like 2A	0.99121	1.016431	1.0158573
169693	C9orf71	chromosome 9 open reading frame 71	1.0378	1.006669	0.986152
169714	QSOX2	quiescin Q6 sulfhydryl oxidase 2	0.94145	0.971663	1.0539567
169792	GLIS3	GLIS family zinc finger 3	0.99574	0.990887	1.0083829
169834	ZNF883	zinc finger protein 883	0.99724	1.021697	1.0059118
169841	ZNF169	zinc finger protein 169	0.98661	0.975229	0.9978807
169966	FAM46D	family with sequence similarity 46, member D	0.99123	1.010779	0.9888395
169981	SPIN3	spindlin family, member 3	0.92363	0.854872	1.0397525
170062	FAM47B	family with sequence similarity 47, member B	0.96649	0.979129	1.0570616
170063	CXorf22	chromosome X open reading frame 22	0.985	1.010715	1.0058736
170067	FAM48B2	family with sequence similarity 48, member B2	1.0003	1.021654	0.9762856
170082	TCEANC	transcription elongation factor A (SII) N-terminal and central domain containing	0.89772	1.026958	0.9641835
170261	ZCCHC12	zinc finger, CCHC domain containing 12	0.9983	0.996665	1.0043092
170302	ARX	aristaless related homeobox	0.98118	0.969376	1.0801552
170370	FAM170B	family with sequence similarity 170, member B	1.03211	1.028176	0.9971778
170371	C10orf128	chromosome 10 open reading frame 128	0.9827	1.005949	0.9523478
170384	FUT11	fucosyltransferase 11 (alpha (1,3) fucosyltransferase)	0.93243	1.076127	1.0188885
170392	OIT3	oncoprotein induced transcript 3	0.99826	1.026753	1.0144177
170393	C10orf91	chromosome 10 open reading frame 91	1.00102	1.00778	0.973386
170394	PWWP2B	PWWP domain containing 2B	0.98228	1.039733	0.9703799
170463	SSBP4	single stranded DNA binding protein 4	0.985	0.977703	1.1208425
170482	CLEC4C	C-type lectin domain family 4, member C	1.08086	0.955666	0.977925
170485	ARF4P2	ADP-ribosylation factor 4 pseudogene 2	1.03033	1.011606	0.9354637
170487	C20orf134	chromosome 20 open reading frame 134	0.97966	1.009371	1.0547464
170506	DHX36	DEAH (Asp-Glu-Ala-His) box polypeptide 36	0.97628	1.039529	0.9282661
170513	FAT1P1	FAT tumor suppressor homolog 1 (Drosophila) pseudogene 1	1.00478	0.99772	1.0289184
170514	FTLP1	ferritin, light polypeptide pseudogene 1	0.98089	0.960356	0.9756509

170516	GAPDHP2	glyceraldehyde-3-phosphate dehydrogenase pseudogene 2	1.01491	0.974771	0.9509921
170523	GSTM3P1	glutathione S-transferase mu 3 pseudogene 1	0.97981	1.018721	1.0237976
170526	HNRNPA3P	heterogeneous nuclear ribonucleoprotein A3 pseudogene 2	0.96952	1.043486	0.8804997
170527	KRT18P3	keratin 18 pseudogene 3	0.99482	1.022725	1.0497198
170529	RPSAP1	ribosomal protein SA pseudogene 1	1.02592	1.004146	0.9663939
170533	PA2G4P2	proliferation-associated 2G4 pseudogene 2	0.97581	0.978919	1.03534
170535	PGAM3P	phosphoglycerate mutase 3, pseudogene	0.99198	1.022306	1.054723
170536	PPIAP21	peptidylprolyl isomerase A (cyclophilin A) pseudogene 21	0.97277	0.998607	1.0621629
170539	PPIAP3	peptidylprolyl isomerase A (cyclophilin A) pseudogene 3	1.00647	0.85909	1.019639
170541	PSMD10P1	proteasome 26S subunit, non-ATPase, 10 pseudogene 1	0.97103	1.042519	0.9192189
170548	SF3A3P1	splicing factor 3a, subunit 3 pseudogene 1	0.94628	1.017895	1.0381482
170550	SNRPFP1	small nuclear ribonucleoprotein polypeptide F pseudogene 1	0.99116	1.012514	0.9190839
170552	SRMP1	spermidine synthase pseudogene 1	1.03234	0.972571	0.9819455
170555	TPM3P2	tropomyosin 3 pseudogene 2	0.99214	0.965056	1.0566312
170559	XPOTP1	exportin, tRNA (nuclear export receptor for tRNAs) pseudogene 1	0.95617	0.903362	1.0339739
170572	HTR3C	5-hydroxytryptamine (serotonin) receptor 3, family member C	0.98938	1.020921	1.0069421
170575	GIMAP1	GTPase, IMAP family member 1	0.96618	0.76367	0.8976035
170589	GPHA2	glycoprotein hormone alpha 2	0.97529	1.047859	1.0326506
170591	S100Z	S100 calcium binding protein Z	1.0235	0.9943	0.936994
170622	COMMD6	COMM domain containing 6	0.94475	0.97352	1.0226813
170626	XAGE3	X antigen family, member 3	0.97642	1.032606	1.015975
170627	XAGE5	X antigen family, member 5	1.00904	1.02887	0.9642707
170679	PSORS1C1	psoriasis susceptibility 1 candidate 1	1.00343	0.977901	1.001717
170680	PSORS1C2	psoriasis susceptibility 1 candidate 2	1	0.994255	0.9942299
170685	NUDT10	nudix (nucleoside diphosphate linked moiety X)-type motif 10	0.99541	1.060716	1.0607607
170689	ADAMTS15	ADAM metallopeptidase with thrombospondin type 1 motif, 15	0.99179	0.961616	1.0075236
170690	ADAMTS16	ADAM metallopeptidase with thrombospondin type 1 motif, 16	1.01237	1.016563	1.0308613
170691	ADAMTS17	ADAM metallopeptidase with thrombospondin type 1 motif, 17	0.97724	0.989468	0.984051
170692	ADAMTS18	ADAM metallopeptidase with thrombospondin type 1 motif, 18	1.00952	0.98873	1.0157014
170712	COX7B2	cytochrome c oxidase subunit VIIb2	0.99125	1.027721	1.0300968
170825	GSX2	GS homeobox 2	1.00459	0.99456	1.014855
170850	KCNG3	potassium voltage-gated channel, subfamily G, member 3	0.99473	1.006264	1.0255654
170954	KIAA1949	KIAA1949	0.96558	0.990793	0.9257856

170958	ZNF525	zinc finger protein 525	0.90156	0.974359	0.9848468
170959	ZNF431	zinc finger protein 431	0.95226	0.910677	0.9997706
170960	ZNF721	zinc finger protein 721	0.98519	0.932233	0.9858423
170961	ANKRD24	ankyrin repeat domain 24	0.99672	1.003425	1.0526709
171017	ZNF384	zinc finger protein 384	0.96949	1.014042	1.0635891
171019	ADAMTS19	ADAM metallopeptidase with thrombospondin type 1 motif, 19	1.0123	1.009641	1.0137856
171023	ASXL1	additional sex combs like 1 (Drosophila)	0.9447	0.982402	1.0324743
171024	SYNPO2	synaptopodin 2	1.01036	1.008258	1.0216177
171169	SPACA4	sperm acrosome associated 4	0.9752	1.030715	0.9830969
171177	RHOV	ras homolog gene family, member V	0.98296	1.03079	1.0439832
171221	DNAJB1P1	DnaJ (Hsp40) homolog, subfamily B, member 1 pseudogene 1	0.9955	1.007167	0.9934759
171222	UBE2L5P	ubiquitin-conjugating enzyme E2L 5, pseudogene	0.94146	1.010124	1.0628795
171389	NLRP6	NLR family, pyrin domain containing 6	1.01199	0.972261	0.9669406
171391	NS3BP	NS3BP	0.98385	1.032938	1.0099922
171392	ZNF675	zinc finger protein 675	0.91271	0.971046	1.068219
171421	EPF8	heat shock 10kDa protein 1 (chaperonin 10) pseudogene	0.99976	1.003122	1.0027144
171423	PDIA3P	protein disulfide isomerase family A, member 3 pseudogene	0.97269	1.03243	0.9921447
171424	SPA17P1	sperm autoantigenic protein 17 pseudogene 1	0.99452	0.956545	1.067279
171425	CLYBL	citrate lyase beta like	0.99253	0.955845	1.0185452
171427	CYP2F1P	cytochrome P450, family 2, subfamily F, polypeptide 1 pseudogene	0.9895	1.049113	1.0154639
171472	SRSF10P2	serine/arginine-rich splicing factor 10 pseudogene 2	0.98129	1.054254	0.9188848
171482	FAM9A	family with sequence similarity 9, member A	0.98791	0.970601	1.0070149
171483	FAM9B	family with sequence similarity 9, member B	0.98314	1.013321	1.0159948
171484	FAM9C	family with sequence similarity 9, member C	1.00427	0.983045	0.9929874
171523	CYP2T2P	cytochrome P450, family 2, subfamily T, polypeptide 2, pseudogene	0.99195	1.032488	1.0115362
171546	C14orf147	chromosome 14 open reading frame 147	0.98921	1.010126	1.0268212
171558	PTCRA	pre T-cell antigen receptor alpha	1.00388	0.993118	0.9931247
171560	AF357530	thioredoxin 1 pseudogene 3	0.98179	0.964707	1.0575521
171568	POLR3H	polymerase (RNA) III (DNA directed) polypeptide H (22.9kD)	0.94414	0.951856	0.9997557
171586	ABHD3	abhydrolase domain containing 3	0.96202	0.983895	0.9531363
191585	PLAC4	placenta-specific 4	0.9908	0.987639	0.9861286
192111	PGAM5	phosphoglycerate mutase family member 5	0.94876	1.003233	1.043559
192127	SLC20A1P1	solute carrier family 20 (phosphate transporter), member 1 pseudogene 1	1.05381	0.888409	1.0271279

192134	B3GNT6	UDP-GlcNAc:betaGal beta-1,3-N-acetylglucosaminyltransferase 6 (core 3 synthase)	0.98381	0.986632	1.0323174
192286	HIGD2A	HIG1 hypoxia inducible domain family, member 2A	0.95342	0.934182	1.1060906
192666	KRT24	keratin 24	0.98044	1.007513	0.9861269
192668	CYS1	cystin 1	0.99699	0.999506	1.0643833
192669	EIF2C3	eukaryotic translation initiation factor 2C, 3	1.00434	1.001527	1.0056042
192670	EIF2C4	eukaryotic translation initiation factor 2C, 4	1.01138	0.934964	0.9778016
192683	SCAMP5	secretory carrier membrane protein 5	1.0061	1.019467	0.9962038
195814	SDR16C5	short chain dehydrogenase/reductase family 16C, member 5	0.99201	0.987745	0.9955039
195827	C9orf21	chromosome 9 open reading frame 21	0.96391	0.955277	0.9696739
195828	ZNF367	zinc finger protein 367	0.94317	0.954082	0.9793894
195977	ANTXRL	anthrax toxin receptor-like	0.98621	0.996269	0.9594
196047	EMX2OS	EMX2 opposite strand (non-protein coding)	1.00289	0.982851	1.0117338
196051	PPAPDC1A	phosphatidic acid phosphatase type 2 domain containing 1A	1.00267	0.993712	1.0007391
196074	METTL15	methyltransferase like 15	0.97967	0.954996	0.9856519
196188	RPS2P38	ribosomal protein S2 pseudogene 38	0.99032	1.026859	0.9900049
196264	MPZL3	myelin protein zero-like 3	0.93946	0.998895	0.9515297
196294	IMMP1L	IMP1 inner mitochondrial membrane peptidase-like (S. cerevisiae)	0.97559	0.982532	1.0319894
196296	[No Symbo	[No Name]	0.99558	1	0.9954644
196335	OR56B4	olfactory receptor, family 56, subfamily B, member 4	1.00428	0.950166	1.0377191
196346	[No Symbo	[No Name]	1.009	1.065134	0.9605887
196374	KRT78	keratin 78	0.99698	0.987744	1.0053901
196383	RILPL2	Rab interacting lysosomal protein-like 2	0.96997	0.977813	1.0460895
196385	DNAH10	dynein, axonemal, heavy chain 10	0.99661	0.98953	0.9989387
196394	AMN1	antagonist of mitotic exit network 1 homolog (S. cerevisiae)	0.93212	1.001976	0.9915071
196403	DTX3	deltex homolog 3 (Drosophila)	0.94895	0.950487	1.0459277
196410	METTL7B	methyltransferase like 7B	0.99442	1.033618	0.9842263
196415	C12orf77	chromosome 12 open reading frame 77	1.01971	1.036156	1.0208748
196441	ZFC3H1	zinc finger, C3H1-type containing	0.9533	1.02368	1.0685772
196446	C12orf28	chromosome 12 open reading frame 28	0.98913	1.011544	0.9967672
196463	PLBD2	phospholipase B domain containing 2	0.96841	0.971616	0.9683478
196472	FAM71C	family with sequence similarity 71, member C	0.99292	1.075206	1.0048735
196477	C12orf12	chromosome 12 open reading frame 12	1.01057	0.978913	0.9975654
196483	FAM86A	family with sequence similarity 86, member A	0.95955	0.974794	1.0312408

196500	C12orf53	chromosome 12 open reading frame 53	1.00562	1.007417	1.0098154
196513	DCP1B	DCP1 decapping enzyme homolog B (S. cerevisiae)	0.97252	1.001966	1.0099392
196527	ANO6	anoctamin 6	0.96146	0.961853	1.0947164
196528	ARID2	AT rich interactive domain 2 (ARID, RFX-like)	0.96297	1.069522	0.9375088
196541	METTL21C	methyltransferase like 21C	1.01125	1.010049	0.9814773
196549	EEF1DP3	eukaryotic translation elongation factor 1 delta pseudogene 3	0.96341	0.947821	1.0451035
196707	[No Symbo	[No Name]	1.01223	1.033406	0.9022358
196740	C10orf72	chromosome 10 open reading frame 72	0.98972	1.018154	1.0224535
196743	PAOX	polyamine oxidase (exo-N4-amino)	0.9781	0.968784	1.0316676
196792	FAM24B	family with sequence similarity 24, member B	1.00569	0.989458	1.1264983
196883	ADCY4	adenylate cyclase 4	1.0092	1.025587	0.9733333
196913	C14orf183	chromosome 14 open reading frame 183	0.98233	1.02632	1.0024679
196951	C15orf33	chromosome 15 open reading frame 33	0.97779	0.982661	1.0243072
196993	CT62	cancer/testis antigen 62	0.99471	1.015661	0.9992714
196996	GRAMD2	GRAM domain containing 2	1.01026	1.007195	0.9972437
197003	MGC15885	hypothetical protein MGC15885	1.00154	1.014608	1.0040386
197021	LCTL	lactase-like	0.99892	1.008212	0.9832791
197131	UBR1	ubiquitin protein ligase E3 component n-recognin 1	0.93792	0.96841	1.0575569
197135	PATL2	protein associated with topoisomerase II homolog 2 (yeast)	0.99361	1.098592	0.9948753
197196	NCRNA003	non-protein coding RNA 311	0.98413	0.975835	1.0098936
197257	LDHD	lactate dehydrogenase D	1.0003	0.990975	1.0355083
197258	FUK	fucokinase	0.97609	0.967333	1.0233125
197259	MLKL	mixed lineage kinase domain-like	1.00606	0.972019	1.0053568
197320	ZNF778	zinc finger protein 778	0.94718	1.005931	1.0591087
197322	ACSF3	acyl-CoA synthetase family member 3	0.97279	0.962484	0.9677947
197335	WDR90	WD repeat domain 90	0.99321	1.014564	1.0488771
197342	EME2	essential meiotic endonuclease 1 homolog 2 (S. pombe)	1.0028	0.984933	1.0468342
197350	CASP14L	putative caspase-14-like protein	1.00058	0.971176	1.0288073
197358	NLRC3	NLR family, CARD domain containing 3	0.95834	0.982121	0.9727133
197370	NSMCE1	non-SMC element 1 homolog (S. cerevisiae)	0.9599	0.961825	0.9973096
197407	ZNF48	zinc finger protein 48	0.94637	0.942151	1.0365586
198437	C20orf201	chromosome 20 open reading frame 201	0.99894	1.01058	1.1225173
199221	DZIP1L	DAZ interacting protein 1-like	1.00531	1.012962	0.9946242

199223	TTC21A	tetratricopeptide repeat domain 21A	1.00792	0.981608	1.0086381
199692	ZNF627	zinc finger protein 627	0.94466	0.989357	0.944176
199699	DAND5	DAN domain family, member 5	1.00254	1.017471	1.0144014
199704	ZNF585A	zinc finger protein 585A	0.9277	0.982539	1.0492174
199713	NLRP7	NLR family, pyrin domain containing 7	1.02506	1.040558	1.0248761
199720	GGN	gametogenetin	1.00336	1.020191	1.0054225
199731	CADM4	cell adhesion molecule 4	1.0021	1.014503	1.0001781
199745	THAP8	THAP domain containing 8	0.96348	0.997904	1.0334009
199746	U2AF1L4	U2 small nuclear RNA auxiliary factor 1-like 4	0.91213	0.901755	0.9265937
199777	ZNF626	zinc finger protein 626	0.94967	0.945395	0.9370047
199800	C19orf76	chromosome 19 open reading frame 76	0.93924	0.963681	1.1225876
199827	RNASEH1P3	ribonuclease H1 pseudogene 3	1.00726	0.970172	1.0262472
199834	LCE4A	late cornified envelope 4A	1.05837	1.023754	1.0975155
199857	ALG14	asparagine-linked glycosylation 14 homolog (S. cerevisiae)	1.00714	0.982209	1.0444574
199870	FAM76A	family with sequence similarity 76, member A	1.00007	0.962601	1.0263762
199897	LOC199897	hypothetical protein LOC199897	0.98148	1.002251	1.0214858
199920	C1orf168	chromosome 1 open reading frame 168	1.00684	1.001426	1.0097529
199953	TMEM201	transmembrane protein 201	0.98355	0.952574	1.057653
199964	TMEM61	transmembrane protein 61	1.02383	1.03205	1.055934
199974	CYP4Z1	cytochrome P450, family 4, subfamily Z, polypeptide 1	0.99447	1.017068	0.9909483
199990	C1orf86	chromosome 1 open reading frame 86	0.99856	1.002291	0.9926014
200008	CDCP2	CUB domain containing protein 2	1.00261	0.969931	1.0374151
200010	SLC5A9	solute carrier family 5 (sodium/glucose cotransporter), member 9	1.02455	0.987001	1.0027397
200014	CC2D1B	coiled-coil and C2 domain containing 1B	0.98257	0.987579	0.933708
200035	NUDT17	nudix (nucleoside diphosphate linked moiety X)-type motif 17	1.00855	1.05315	1.0467612
200081	TXLNA	taxilin alpha	0.94811	0.918186	1.0503286
200095	[No Symbo	[No Name]	0.98968	0.950104	1.0073921
200132	TCTEX1D1	Tctex1 domain containing 1	0.99146	0.973948	1.0033255
200149	LOC200149	tubulin, beta 8 pseudogene	1.00134	0.991534	1.0650666
200150	PLD5	phospholipase D family, member 5	1.01059	1.004788	1.0226782
200159	C1orf100	chromosome 1 open reading frame 100	0.99541	1.024336	0.961261
200162	SPAG17	sperm associated antigen 17	1.0084	0.998195	1.0136665
200172	SLFNL1	schlafen-like 1	1.01682	0.990525	1.0179641

200185	KRTCAP2	keratinocyte associated protein 2	0.97184	0.961239	1.0811888
200186	CRTC2	CREB regulated transcription coactivator 2	1.02491	1.065774	1.0095997
200205	IBA57	IBA57, iron-sulfur cluster assembly homolog (S. cerevisiae)	0.99253	1.00739	0.995223
200232	C20orf106	chromosome 20 open reading frame 106	1.00737	0.972416	1.0229572
200261	LOC200261	hypothetical LOC200261	1.03128	0.952696	0.9555171
200298	C22orf37	chromosome 22 open reading frame 37	0.97072	1.064856	0.8519964
200312	RNF215	ring finger protein 215	0.98366	1.002436	1.0556135
200315	APOBEC3A	apolipoprotein B mRNA editing enzyme, catalytic polypeptide-like 3A	1.00413	1.144165	0.9668153
200316	APOBEC3F	apolipoprotein B mRNA editing enzyme, catalytic polypeptide-like 3F	0.96101	0.996564	1.0558363
200317	FLJ23865	hypothetical protein FLJ23865	1.06859	1.003573	1.0360027
200373	PCDP1	primary ciliary dyskinesia protein 1	1.01052	1.011865	1.0214432
200403	VWA3B	von Willebrand factor A domain containing 3B	1.01459	1.014486	1.0139624
200407	CREG2	cellular repressor of E1A-stimulated genes 2	0.99769	1.004761	1.027539
200420	ALMS1P	Alstrom syndrome 1 pseudogene	1.00436	0.960519	1.0121731
200424	TET3	tet oncogene family member 3	1.01026	0.962895	1.0319457
200504	GKN2	gastrokine 2	1.01095	1.020181	1.023166
200523	C2orf51	chromosome 2 open reading frame 51	1.02187	0.997165	1.0090507
200539	ANKRD23	ankyrin repeat domain 23	1.00854	1.00315	1.0096
200558	APLF	aprataxin and PNKP like factor	0.97791	0.987815	1.0056721
200576	PIKFYVE	phosphoinositide kinase, FYVE finger containing	0.98417	0.977591	0.9114894
200634	KRTCAP3	keratinocyte associated protein 3	0.99845	0.982053	1.0220872
200726	LOC200726	hCG1657980	0.97355	0.990994	0.9886241
200728	TMEM17	transmembrane protein 17	0.97024	0.94239	1.0605356
200734	SPRED2	sprouty-related, EVH1 domain containing 2	0.99856	1.012105	0.9989138
200765	TIGD1	tigger transposable element derived 1	1.02186	0.930658	0.9285981
200810	ALG1L	asparagine-linked glycosylation 1-like	0.99372	0.988741	0.9601449
200844	C3orf67	chromosome 3 open reading frame 67	1.00559	1.004977	1.01225
200845	KCTD6	potassium channel tetramerisation domain containing 6	1.00602	0.964482	0.9852206
200879	LIPH	lipase, member H	0.97832	0.92581	1.0593763
200894	ARL13B	ADP-ribosylation factor-like 13B	0.98208	0.993733	1.0794359
200895	DHFRL1	dihydrofolate reductase-like 1	0.936	0.968938	1.0548686
200909	HTR3D	5-hydroxytryptamine (serotonin) receptor 3 family member D	1.01634	1.006204	0.994277
200916	RPL22L1	ribosomal protein L22-like 1	0.98171	1.026919	1.0813694

200931	OSTalpha	organic solute transporter alpha	1.01671	1.014092	0.9913432
200933	FBXO45	F-box protein 45	0.98816	0.940225	1.0911175
200942	KLHDC8B	kelch domain containing 8B	0.98669	1.005668	1.0182396
200958	MUC20	mucin 20, cell surface associated	1.03455	0.992916	0.9591945
200959	GABRR3	gamma-aminobutyric acid (GABA) receptor, rho 3	1.00736	1.013272	1.0172184
201134	CEP112	centrosomal protein 112kDa	0.99295	0.990489	0.9997603
201140	DHRS7C	dehydrogenase/reductase (SDR family) member 7C	1.00176	1.017689	0.9974728
201158	FAM18B2	family with sequence similarity 18, member B2	0.97519	0.970332	1.0043133
201161	CENPV	centromere protein V	0.97421	0.995578	1.0043656
201163	FLCN	folliculin	0.97102	0.976864	1.0010189
201164	PLD6	phospholipase D family, member 6	0.94646	0.974657	1.036245
201175	[No Symbo	[No Name]	0.99786	1.006212	0.9894081
201176	ARHGAP27	Rho GTPase activating protein 27	0.98193	0.985595	1.0247828
201181	ZNF385C	zinc finger protein 385C	0.98205	1.000784	0.9963867
201191	SAMD14	sterile alpha motif domain containing 14	0.97056	0.950493	1.055012
201229	C17orf108	chromosome 17 open reading frame 108	0.98662	0.972796	0.9403654
201232	SLC16A13	solute carrier family 16, member 13 (monocarboxylic acid transporter 13)	0.98217	0.940059	0.968958
201243	C17orf74	chromosome 17 open reading frame 74	1.0051	1.001062	1.0418347
201254	STRA13	stimulated by retinoic acid 13 homolog (mouse)	1.0084	0.982433	1.0806738
201255	LRRC45	leucine rich repeat containing 45	0.98171	0.976456	1.0720503
201259	RPL32P31	ribosomal protein L32 pseudogene 31	0.94385	1.01864	1.0439866
201266	SLC39A11	solute carrier family 39 (metal ion transporter), member 11	0.99882	0.948388	1.0078727
201283	AMZ2P1	archaelysin family metallopeptidase 2 pseudogene 1	0.96035	1.077881	0.9033636
201292	TRIM65	tripartite motif containing 65	1.00195	0.921396	1.0999675
201294	UNC13D	unc-13 homolog D (C. elegans)	0.93318	1.004884	1.0345566
201299	RDM1	RAD52 motif 1	0.99357	0.948734	1.0564205
201305	SPNS3	spinster homolog 3 (Drosophila)	1.00775	1.009	1.0428951
201456	FBXO15	F-box protein 15	0.98861	1.005039	0.9979364
201475	RAB12	RAB12, member RAS oncogene family	0.96494	1.144715	0.9672915
201501	ZBTB7C	zinc finger and BTB domain containing 7C	1.00916	1.015033	0.9906162
201514	ZNF584	zinc finger protein 584	0.96605	1.0289	1.0658771
201516	ZSCAN4	zinc finger and SCAN domain containing 4	0.99396	0.979605	1.0006232
201562	PTPLB	protein tyrosine phosphatase-like (proline instead of catalytic arginine), member b	0.9928	0.894246	1.068029

201595	STT3B	STT3, subunit of the oligosaccharyltransferase complex, homolog B (S. cerevisiae)	1.02946	0.990884	1.0219057
201626	PDE12	phosphodiesterase 12	0.95015	0.907336	0.9271924
201627	FAM116A	family with sequence similarity 116, member A	1.00605	1.026954	0.9641002
201633	TIGIT	T cell immunoreceptor with Ig and ITIM domains	0.97906	0.938648	0.9390189
201651	LOC201651	arylacetamide deacetylase (esterase) pseudogene	0.9872	1.015133	1.0202002
201725	C4orf46	chromosome 4 open reading frame 46	0.987	1.024294	1.0557192
201780	SLC10A4	solute carrier family 10 (sodium/bile acid cotransporter family), member 4	0.97665	0.978662	1.0523987
201798	TIGD4	tigger transposable element derived 4	0.96659	0.982405	1.032908
201799	TMEM154	transmembrane protein 154	0.9725	1.004964	0.9373404
201895	C4orf34	chromosome 4 open reading frame 34	1.03999	1.009316	0.9982432
201931	TMEM192	transmembrane protein 192	0.98336	0.928545	0.9593065
201965	RWDD4	RWD domain containing 4	1.01127	0.910784	0.9939652
201973	CCDC111	coiled-coil domain containing 111	0.98709	1.035976	1.050138
201989	GK6P	glycerol kinase 6 pseudogene	0.98586	0.919114	1.0471904
202018	TAPT1	transmembrane anterior posterior transformation 1	0.98699	0.902988	1.0340529
202020	FLJ39653	hypothetical FLJ39653	0.9902	0.970438	0.9466014
202051	SPATA24	spermatogenesis associated 24	0.98819	1.013001	1.0194104
202052	DNAJC18	DnaJ (Hsp40) homolog, subfamily C, member 18	0.99991	1.001061	1.0102564
202122	RPL27P10	ribosomal protein L27 pseudogene 10	1.0331	1.020756	1.0181803
202151	RANBP3L	RAN binding protein 3-like	0.99125	1.011897	1.0255246
202181	LOC202181	hypothetical LOC202181	0.97052	0.984415	0.9599264
202243	CCDC125	coiled-coil domain containing 125	0.98697	0.945324	1.0225326
202299	C5orf27	chromosome 5 open reading frame 27	1.00341	0.992812	1.0304221
202309	GAPT	GRB2-binding adaptor protein, transmembrane	0.99898	0.971314	0.983093
202319	LOC202319	peptidylprolyl cis-trans isomerase A-like 4G-like	0.99122	0.920053	1.0761069
202333	CMYA5	cardiomyopathy associated 5	1.01134	0.984033	0.9908306
202374	STK32A	serine/threonine kinase 32A	1.00055	0.980198	1.0124836
202459	OSTCL	oligosaccharyltransferase complex subunit-like	1.03309	0.974641	0.9913043
202500	TCTE1	t-complex-associated-testis-expressed 1	1.01398	1.020509	0.9780642
202559	KHDRBS2	KH domain containing, RNA binding, signal transduction associated 2	0.9898	0.990943	0.9755968
202781	LOC202781	hypothetical LOC202781	0.98505	0.970962	1.013157
202861	OR2A3P	olfactory receptor, family 2, subfamily A, member 3 pseudogene	1.01216	0.998647	1.0040003
202865	C7orf33	chromosome 7 open reading frame 33	1.02408	1.007032	1.0029359

202915	TMEM184A	transmembrane protein 184A	0.98104	0.997116	1.0095229
203054	ADCK5	aarF domain containing kinase 5	0.99044	1.001629	1.0450609
203062	TSNARE1	t-SNARE domain containing 1	0.98363	1.028476	1.031937
203068	TUBB	tubulin, beta	0.99466	1.095736	1.1024781
203069	R3HCC1	R3H domain and coiled-coil containing 1	1.00262	0.937841	1.0518531
203074	PRSS55	protease, serine, 55	1.0031	1.005227	1.0107181
203076	C8orf74	chromosome 8 open reading frame 74	1.00468	1.018224	0.9805359
203102	ADAM32	ADAM metallopeptidase domain 32	0.98776	0.995568	0.9845904
203111	C8orf47	chromosome 8 open reading frame 47	1.00191	1.022845	1.0267853
203190	LGI3	leucine-rich repeat LGI family, member 3	0.987	0.999453	1.0072577
203197	C9orf91	chromosome 9 open reading frame 91	0.96793	0.978449	1.0581539
203228	C9orf72	chromosome 9 open reading frame 72	1.03399	1.000483	0.9558895
203235	C9orf141	chromosome 9 open reading frame 141	0.97927	1.037031	1.0222433
203238	C9orf93	chromosome 9 open reading frame 93	0.99922	0.991656	1.0036593
203245	NAIF1	nuclear apoptosis inducing factor 1	0.96605	0.928706	1.0597204
203259	C9orf25	chromosome 9 open reading frame 25	0.96701	0.9365	0.9837017
203260	CCDC107	coiled-coil domain containing 107	0.95476	1.044607	1.0937294
203286	ANKS6	ankyrin repeat and sterile alpha motif domain containing 6	0.98185	0.953556	0.9636371
203328	SUSD3	sushi domain containing 3	0.94562	0.983132	1.0490677
203413	CXorf61	chromosome X open reading frame 61	1.01701	1.022629	1.0048253
203427	SLC25A43	solute carrier family 25, member 43	0.97573	0.974204	1.0138357
203430	ZCCHC5	zinc finger, CCHC domain containing 5	1.00235	0.982668	1.0207642
203447	NRK	Nik related kinase	1.00384	1.011926	1.0004236
203477	HMGA1P1	high mobility group AT-hook 1 pseudogene 1	1.00429	1.032087	1.0401623
203510	HMGB3P30	high mobility group box 3 pseudogene 30	0.99504	0.968984	1.023062
203522	DDX26B	DEAD/H (Asp-Glu-Ala-Asp/His) box polypeptide 26B	0.95331	1.010072	1.0338831
203523	ZNF449	zinc finger protein 449	0.95684	0.975874	1.0395444
203547	VMA21	VMA21 vacuolar H+-ATPase homolog (S. cerevisiae)	0.94253	0.860582	1.1287355
203562	TMEM31	transmembrane protein 31	1.0267	0.960083	1.0558471
203859	ANO5	anoctamin 5	0.99193	0.995763	1.0244303
203930	LOC203930	sorting nexin 7 pseudogene	0.97646	0.955542	1.0222203
204010	RPSAP52	ribosomal protein SA pseudogene 52	0.99193	1.021677	1.0110688
204219	LASS3	LAG1 homolog, ceramide synthase 3	0.98181	0.994287	0.965645

204474	PDILT	protein disulfide isomerase-like, testis expressed	1.00596	0.981522	1.0178271
204777	IGLJCOR18	immunoglobulin lambda joining-constant/OR18 (pseudogene)	1.04165	1.011076	0.9044834
204800	LOC204800	actin related protein 2/3 complex subunit 1A pseudogene	1.00613	0.958709	1.0247261
204801	NLRP11	NLR family, pyrin domain containing 11	0.98013	1.013855	1.0108469
204851	HIPK1	homeodomain interacting protein kinase 1	1.03391	0.946666	0.9521706
204962	SLC44A5	solute carrier family 44, member 5	1.01894	0.977975	1.0205556
205147	FAM123C	family with sequence similarity 123C	1.01114	1.03618	0.983772
205251	NCRNA001	non-protein coding RNA 116	0.97857	1.005593	1.0229963
205272	CNN2P8	calponin 2 pseudogene 8	0.99274	0.984177	0.9619927
205327	C2orf69	chromosome 2 open reading frame 69	0.97813	0.961139	1.006676
205428	C3orf58	chromosome 3 open reading frame 58	1.01408	0.954292	1.0654616
205564	SENP5	SUMO1/sentrin specific peptidase 5	0.97444	0.93928	0.9940116
205655	NBPF21P	neuroblastoma breakpoint family, member 21, pseudogene	1.01478	1.001791	0.9870283
205717	KIAA2018	KIAA2018	1.01988	0.889152	1.0197697
205860	TRIML2	tripartite motif family-like 2	1.0226	1.010152	0.9867307
206338	AQPEP	laeverin	1.0087	0.979478	1.0149101
206358	SLC36A1	solute carrier family 36 (proton/amino acid symporter), member 1	1.02498	0.938242	1.0321583
206412	C6orf163	chromosome 6 open reading frame 163	1.00631	0.969122	1.0648084
206426	PIP5K1P1	phosphatidylinositol-4-phosphate 5-kinase, type I, pseudogene 1	1.01162	0.965372	1.0474844
207032	RPL36AP52	ribosomal protein L36a pseudogene 52	0.99672	0.977228	1.0451411
207147	TRY2P	trypsinogen-like pseudogene	1.02266	0.995394	0.9932137
219285	SAMD9L	sterile alpha motif domain containing 9-like	1.00594	0.943295	1.0577336
219287	FAM123A	family with sequence similarity 123A	0.98492	0.978596	1.0059227
219293	ATAD3C	ATPase family, AAA domain containing 3C	0.98264	1.041611	1.0052739
219333	USP12	ubiquitin specific peptidase 12	0.99097	1.049781	0.9445076
219348	PLAC9	placenta-specific 9	0.97285	1.013759	1.0500062
219402	MTIF3	mitochondrial translational initiation factor 3	0.93767	0.96073	0.9352463
219409	GSX1	GS homeobox 1	1.00456	0.978845	1.0862872
219417	OR8U1	olfactory receptor, family 8, subfamily U, member 1	1.01952	1.099718	0.9710196
219429	OR4C11	olfactory receptor, family 4, subfamily C, member 11	1.01258	1.057498	0.9866274
219431	OR4S2	olfactory receptor, family 4, subfamily S, member 2	0.99578	1.053011	0.9938898
219432	OR4C6	olfactory receptor, family 4, subfamily C, member 6	0.95972	1.007264	1.0499699
219436	OR5D14	olfactory receptor, family 5, subfamily D, member 14	0.99126	0.984315	0.9904534

219437	OR5L1	olfactory receptor, family 5, subfamily L, member 1	1.03407	1.0303	1.0227246
219438	OR5D18	olfactory receptor, family 5, subfamily D, member 18	1.01271	1.004589	1.0108832
219445	OR7E5P	olfactory receptor, family 7, subfamily E, member 5 pseudogene	1.00514	0.983169	1.0120215
219453	OR8K5	olfactory receptor, family 8, subfamily K, member 5	1.00033	1.011293	1.0192267
219464	OR5T2	olfactory receptor, family 5, subfamily T, member 2	0.9917	1.056612	0.9287843
219469	OR8H1	olfactory receptor, family 8, subfamily H, member 1	1.00618	0.996745	0.9659451
219473	OR8K3	olfactory receptor, family 8, subfamily K, member 3	0.97696	0.934037	0.9945481
219477	OR8J1	olfactory receptor, family 8, subfamily J, member 1	0.98468	0.997241	1.008911
219479	OR5R1	olfactory receptor, family 5, subfamily R, member 1	1.00288	0.988512	1.0888139
219484	OR5M8	olfactory receptor, family 5, subfamily M, member 8	1.03788	1.001129	0.9763439
219487	OR5M11	olfactory receptor, family 5, subfamily M, member 11	1.01229	1.028788	0.9861398
219493	OR5AR1	olfactory receptor, family 5, subfamily AR, member 1	1.01157	0.983593	0.9895416
219525	OR5AK4P	olfactory receptor, family 5, subfamily AK, member 4 pseudogene	0.99827	1.007019	0.9435626
219527	LRRC55	leucine rich repeat containing 55	0.99911	1.006139	1.007879
219537	SMTNL1	smoothelin-like 1	1.02427	0.984716	0.9563516
219539	YPEL4	yippee-like 4 (Drosophila)	0.99576	1.019585	0.9585857
219541	MED19	mediator complex subunit 19	0.97592	0.990598	1.0285884
219557	C7orf62	chromosome 7 open reading frame 62	0.99976	0.983608	1.0724393
219578	ZNF804B	zinc finger protein 804B	1.00482	1.008626	1.0103034
219595	FOLH1B	folate hydrolase 1B	0.99499	1.013693	1.032598
219621	C10orf107	chromosome 10 open reading frame 107	1.00044	1.000184	1.0093636
219623	TMEM26	transmembrane protein 26	0.97177	1.026128	0.9974446
219670	ENKUR	enkurin, TRPC channel interacting protein	0.8702	0.85867	1.0150324
219681	ARMC3	armadillo repeat containing 3	0.95956	0.962453	0.9586701
219699	UNC5B	unc-5 homolog B (C. elegans)	0.98586	1.0233	1.0453613
219736	STOX1	storkhead box 1	0.98388	0.998827	1.0080466
219738	C10orf35	chromosome 10 open reading frame 35	1.00958	0.982335	1.0024016
219743	TYSND1	trypsin domain containing 1	0.96199	0.974844	1.0192531
219749	ZNF25	zinc finger protein 25	0.96375	0.964268	0.9343407
219752	LOC219752	transforming, acidic coiled-coil containing protein 1 pseudogene	1.01464	0.95136	1.0012986
219770	GJD4	gap junction protein, delta 4, 40.1kDa	0.99535	1.012502	1.0303771
219771	CCNY	cyclin Y	0.98873	0.960954	0.9845702
219790	RTKN2	rhotekin 2	0.96747	0.983021	0.9899832

219793	C10orf27	chromosome 10 open reading frame 27	1.00731	0.970075	1.0015466
219833	C11orf45	chromosome 11 open reading frame 45	0.97556	1.013225	1.0421501
219844	HYLS1	hydrolethalus syndrome 1	0.93908	0.993583	1.0333723
219848	[No Symbo	[No Name]	1.01775	0.964608	0.9611546
219854	TMEM218	transmembrane protein 218	0.92348	0.918382	1.015173
219855	SLC37A2	solute carrier family 37 (glycerol-3-phosphate transporter), member 2	0.99204	0.962201	1.0245001
219858	OR8B12	olfactory receptor, family 8, subfamily B, member 12	0.99992	0.966319	0.9628884
219869	OR10G8	olfactory receptor, family 10, subfamily G, member 8	1.00823	1.01279	0.9765991
219873	OR10S1	olfactory receptor, family 10, subfamily S, member 1	0.99899	1.026393	0.8777082
219874	OR6T1	olfactory receptor, family 6, subfamily T, member 1	1.04498	0.945259	1.0016264
219875	OR4D5	olfactory receptor, family 4, subfamily D, member 5	1.02236	1.004532	1.0452118
219899	TBCEL	tubulin folding cofactor E-like	0.91546	0.933271	1.084268
219902	TMEM136	transmembrane protein 136	1.00278	1.015756	0.9905945
219927	MRPL21	mitochondrial ribosomal protein L21	0.97156	0.951925	1.0009557
219928	[No Symbo	[No Name]	1.00229	0.99931	1.0237351
219931	TPCN2	two pore segment channel 2	0.99903	1.071797	0.9797664
219938	SPATA19	spermatogenesis associated 19	1.0049	0.968264	1.0136006
219954	OR9I1	olfactory receptor, family 9, subfamily I, member 1	1.00496	1.016253	0.9929635
219956	OR9Q1	olfactory receptor, family 9, subfamily Q, member 1	1.00258	0.993544	1.0080325
219957	OR9Q2	olfactory receptor, family 9, subfamily Q, member 2	0.99283	1.010108	1.0288967
219958	OR1S2	olfactory receptor, family 1, subfamily S, member 2	1.0042	0.993043	0.9480853
219959	OR1S1	olfactory receptor, family 1, subfamily S, member 1	1.00719	1.060705	0.983541
219960	OR10Q1	olfactory receptor, family 10, subfamily Q, member 1	0.9705	1.0118	1.0583605
219965	OR5B17	olfactory receptor, family 5, subfamily B, member 17	1.00252	0.964093	1.0645292
219968	OR5B21	olfactory receptor, family 5, subfamily B, member 21	0.99771	1.036773	1.0045049
219970	GLYATL2	glycine-N-acyltransferase-like 2	0.9565	0.993289	0.9905744
219972	MPEG1	macrophage expressed 1	0.99099	0.903757	0.9621553
219981	OR5A2	olfactory receptor, family 5, subfamily A, member 2	1.05667	1.033246	0.9906119
219982	OR5A1	olfactory receptor, family 5, subfamily A, member 1	1.02451	1.00632	1.0334313
219983	OR4D6	olfactory receptor, family 4, subfamily D, member 6	1.03477	1.000799	0.9713603
219986	OR4D11	olfactory receptor, family 4, subfamily D, member 11	1.0066	1.024781	0.9844015
219988	PATL1	protein associated with topoisomerase II homolog 1 (yeast)	0.97473	1.072514	1.0752169
219990	PLAC1L	placenta-specific 1-like	0.99688	0.991573	1.0437687

219995	MS4A15	membrane-spanning 4-domains, subfamily A, member 15	0.99392	1.015563	1.0306902
220001	VWCE	von Willebrand factor C and EGF domains	0.99719	1.000939	1.0160492
220002	CYBASC3	cytochrome b, ascorbate dependent 3	0.93505	1.002757	0.9948507
220004	C11orf66	chromosome 11 open reading frame 66	1.01207	1.004998	1.0084746
220032	GDPD4	glycerophosphodiester phosphodiesterase domain containing 4	1.02866	1.042596	0.9925985
220042	C11orf82	chromosome 11 open reading frame 82	0.9376	0.990424	1.002248
220047	CCDC83	coiled-coil domain containing 83	1.00552	0.992923	0.9938036
220064	ORAOV1	oral cancer overexpressed 1	0.96113	1.002205	1.1002072
220070	SHANK2-AS	SHANK2 antisense RNA 3 (non-protein coding)	1.01237	1.007755	1.0274329
220077	LOC220077	dedicator of cytokinesis 1 pseudogene	0.98821	0.967097	1.0854605
220081	FAM194B	family with sequence similarity 194, member B	1.00702	1.009748	0.9904018
220082	SPERT	spermatid associated	0.97758	1.033625	1.0179376
220107	DLEU7	deleted in lymphocytic leukemia, 7	0.99731	1.019042	1.0044263
220108	FAM124A	family with sequence similarity 124A	1.00672	0.99683	0.972988
220112	CTAGE3P	CTAGE family, member 3, pseudogene	0.96078	0.987117	0.9296693
220134	SKA1	spindle and kinetochore associated complex subunit 1	0.98819	1.004062	1.0256532
220136	CCDC11	coiled-coil domain containing 11	0.97684	0.981478	0.9668395
220147	LOC220147	C-terminal binding protein 2 pseudogene	0.9842	0.98459	1.0149869
220164	DOK6	docking protein 6	0.99343	0.98669	0.9989435
220202	ATOH7	atonal homolog 7 (Drosophila)	0.99245	1.041049	1.1747484
220213	OTUD1	OTU domain containing 1	0.94318	0.969952	0.9465037
220296	HEPACAM	hepatic and glial cell adhesion molecule	1.00262	1.001418	0.9883806
220323	OAF	OAF homolog (Drosophila)	0.99276	0.964524	1.0378897
220359	TIGD3	tigger transposable element derived 3	1.0122	1.08346	1.0112402
220382	FAM181B	family with sequence similarity 181, member B	0.98617	0.989465	1.0532807
220388	CCDC89	coiled-coil domain containing 89	1.0276	1.048891	0.9435391
220416	LRRC63	leucine rich repeat containing 63	0.9841	0.993617	1.0295688
220441	RNF152	ring finger protein 152	0.99585	0.998236	0.9697053
220717	RPLP0P6	ribosomal protein, large, P0 pseudogene 6	0.97799	0.962666	1.1252244
220786	CDK7PS	cyclin-dependent kinase 7 pseudogene	0.9992	0.956552	0.9857775
220885	RPSAP15	ribosomal protein SA pseudogene 15	1.00914	1.034094	1.0375005
220906	LOC220906	hypothetical LOC220906	0.95219	0.986463	0.9714886
220929	ZNF438	zinc finger protein 438	0.98171	0.951908	0.9968096

220963	SLC16A9	solute carrier family 16, member 9 (monocarboxylic acid transporter 9)	0.99596	0.991797	1.0075293
220965	FAM13C	family with sequence similarity 13, member C	0.98651	1.016914	1.0163146
220972	8-Mar	membrane-associated ring finger (C3HC4) 8	0.95348	1.021053	0.9710644
220974	OR6D1P	olfactory receptor, family 6, subfamily D, member 1 pseudogene	0.972	0.965958	0.9846049
220979	C10orf25	chromosome 10 open reading frame 25	0.9859	0.981676	1.0633717
220988	HNRNPA3	heterogeneous nuclear ribonucleoprotein A3	1.05241	1.025195	0.9378444
220992	ZNF485	zinc finger protein 485	0.94675	0.978481	1.0183552
221002	RASGEF1A	RasGEF domain family, member 1A	0.9742	1.024726	1.0406423
221016	CCDC7	coiled-coil domain containing 7	0.91982	0.955497	0.9840633
221035	REEP3	receptor accessory protein 3	0.96373	1.020837	1.0040972
221037	JMJD1C	jumonji domain containing 1C	0.97832	1.035562	0.9449301
221044	UCMA	upper zone of growth plate and cartilage matrix associated	0.99074	1.012662	1.0253277
221060	C10orf111	chromosome 10 open reading frame 111	0.98151	0.961944	0.9830465
221061	FAM171A1	family with sequence similarity 171, member A1	0.98383	0.985556	1.0039123
221074	SLC39A12	solute carrier family 39 (zinc transporter), member 12	0.99329	1.004982	1.0060258
221078	NSUN6	NOP2/Sun domain family, member 6	0.92037	0.987466	1.0114809
221079	ARL5B	ADP-ribosylation factor-like 5B	0.94418	1.075143	1.0655311
221091	LRRN4CL	LRRN4 C-terminal like	1.00263	1.006158	1.0323955
221092	HNRNPUL2	heterogeneous nuclear ribonucleoprotein U-like 2	0.95478	0.949228	1.0403796
221120	ALKBH3	alkB, alkylation repair homolog 3 (E. coli)	0.91657	0.975413	0.9948018
221143	N6AMT2	N-6 adenine-specific DNA methyltransferase 2 (putative)	0.96877	1.00352	1.0148595
221154	EFHA1	EF-hand domain family, member A1	0.95107	0.933282	1.0121804
221178	SPATA13	spermatogenesis associated 13	0.97739	0.979983	0.9544473
221188	GPR114	G protein-coupled receptor 114	0.97842	0.999529	1.0497353
221191	PRSS54	protease, serine, 54	1.01897	1.042315	0.984268
221223	CES5A	carboxylesterase 5A	0.99919	0.9977	0.9565954
221241	NCRNA003	non-protein coding RNA 305	0.9992	0.989727	0.9952647
221261	[No Symbo	[No Name]	1.02949	0.991292	1.0076036
221262	CCDC162P	coiled-coil domain containing 162, pseudogene	1.02302	1.007548	1.0381849
221264	AKD1	adenylate kinase domain containing 1	0.98719	0.965257	0.9833786
221294	NT5DC1	5'-nucleotidase domain containing 1	0.98426	0.925978	0.9987926
221301	FAM26D	family with sequence similarity 26, member D	1.00282	1.020887	1.0345783
221302	ZUFSP	zinc finger with UFM1-specific peptidase domain	0.97306	0.957214	1.0367053

221303	FAM162B	family with sequence similarity 162, member B	0.98018	1.015432	1.0561449
221322	C6orf170	chromosome 6 open reading frame 170	0.97395	0.94061	1.008547
221336	BEND6	BEN domain containing 6	0.99747	1.005599	1.0202356
221357	GSTA5	glutathione S-transferase alpha 5	1.03159	0.966523	0.9876866
221391	OPN5	opsin 5	1.01486	1.00487	0.9879583
221393	GPR115	G protein-coupled receptor 115	1.01015	0.99497	0.975203
221395	GPR116	G protein-coupled receptor 116	1.00479	0.992111	0.9966994
221400	TDRD6	tudor domain containing 6	1.00751	1.000268	1.0414162
221409	SPATS1	spermatogenesis associated, serine-rich 1	1.00864	1.048483	0.9883999
221416	C6orf223	chromosome 6 open reading frame 223	1.00933	0.976028	0.9976886
221421	RSPH9	radial spoke head 9 homolog (Chlamydomonas)	1.00753	0.998739	1.0142119
221424	C6orf154	chromosome 6 open reading frame 154	0.99131	0.964076	1.0352202
221442	LOC221442	adenylate cyclase 10 pseudogene	1.00139	1.024417	0.9831154
221443	C6orf130	chromosome 6 open reading frame 130	1.01033	0.881407	0.9519895
221458	KIF6	kinesin family member 6	1.00336	1.011299	1.0078748
221468	TMEM217	transmembrane protein 217	1.02186	1.019702	1.031053
221472	FGD2	FYVE, RhoGEF and PH domain containing 2	1.05082	0.950339	1.1502806
221476	PI16	peptidase inhibitor 16	0.9957	0.957591	0.9987538
221477	C6orf89	chromosome 6 open reading frame 89	0.98663	0.873217	1.0327482
221481	C6orf81	chromosome 6 open reading frame 81	1.02626	1.018458	1.0110666
221491	C6orf1	chromosome 6 open reading frame 1	0.97587	0.982535	0.9590427
221496	LEMD2	LEM domain containing 2	0.99245	1.049476	0.9926807
221504	ZBTB9	zinc finger and BTB domain containing 9	0.98424	0.918541	0.9899596
221527	ZBTB12	zinc finger and BTB domain containing 12	1.00129	1.013754	1.0925911
221545	C6orf136	chromosome 6 open reading frame 136	0.98798	1.033659	1.021603
221547	RANP1	RAN, member RAS oncogene family pseudogene 1	1.00984	0.89356	1.0447257
221613	HIST1H2AA	histone cluster 1, H2aa	1.01372	0.992615	0.9010499
221656	KDM1B	lysine (K)-specific demethylase 1B	1.01411	1.018081	0.9746641
221662	RBM24	RNA binding motif protein 24	0.99667	1.004718	0.9609413
221687	RNF182	ring finger protein 182	1.00291	0.923667	1.033112
221692	PHACTR1	phosphatase and actin regulator 1	1.00607	0.994497	0.9896587
221710	LOC221710	hypothetical protein LOC221710	1.02715	0.929557	1.1775283
221711	SYCP2L	synaptonemal complex protein 2-like	1.03114	0.998509	1.04617

221717	MRPL48P1	mitochondrial ribosomal protein L48 pseudogene 1	1.02626	1.09055	0.9591851
221718	C6orf218	chromosome 6 open reading frame 218	1.00724	1.034776	1.0365396
221749	C6orf145	chromosome 6 open reading frame 145	0.98279	1.006713	0.9599185
221756	MGC39372	serpin peptidase inhibitor, clade B (ovalbumin), member 9 pseudogene	1.02536	1.079151	1.0139523
221785	ZNF498	zinc finger protein 498	0.98259	0.912308	1.0331852
221786	FAM200A	family with sequence similarity 200, member A	0.93434	0.984701	1.0539735
221806	VWDE	von Willebrand factor D and EGF domains	0.9924	0.942422	0.9943513
221823	PRPS1L1	phosphoribosyl pyrophosphate synthetase 1-like 1	1.01569	1.012664	1.0027368
221830	TWISTNB	TWIST neighbor	0.97142	0.86252	0.9577215
221833	SP8	Sp8 transcription factor	1.02944	1.0204	1.0360674
221883	HOXA11-AS	HOXA11 antisense RNA 1 (non-protein coding)	0.9979	1.005071	1.0345277
221895	JAZF1	JAZF zinc finger 1	1.01004	0.957141	0.9626216
221908	C7orf47	chromosome 7 open reading frame 47	0.98844	0.990334	1.0585758
221914	GPC2	glypican 2	0.98233	0.986818	1.0249136
221927	BRAT1	BRCA1-associated ATM activator 1	0.99515	1.088472	1.0033686
221935	SDK1	sidekick homolog 1, cell adhesion molecule (chicken)	1.00367	0.998967	1.0105922
221937	FOXK1	forkhead box K1	1.00861	0.940103	0.9638101
221938	MMD2	monocyte to macrophage differentiation-associated 2	0.98975	1.019752	0.9882691
221955	DAGLB	diacylglycerol lipase, beta	1.01071	0.947068	1.0184553
221981	THSD7A	thrombospondin, type I, domain containing 7A	1.00611	0.979508	0.9995271
222005	SLC25A5P3	solute carrier family 25 (mitochondrial carrier; adenine nucleotide translocator), member 5 pseudogene 3	0.98954	1.015614	1.0529017
222008	VSTM2A	V-set and transmembrane domain containing 2A	1.00027	1.014473	1.0190017
222052	TTC4P1	tetratricopeptide repeat domain 4 pseudogene 1	0.9971	0.920058	1.0193727
222068	TMED4	transmembrane emp24 protein transport domain containing 4	0.96719	0.991503	0.9994723
222161	DKFZP586I1	hypothetical protein DKFZp586I1420	0.99081	1.054585	1.0118519
222166	C7orf41	chromosome 7 open reading frame 41	1.01057	0.920119	0.954647
222171	PRR15	proline rich 15	1.00748	0.998114	1.0203058
222183	SRRM3	serine/arginine repetitive matrix 3	0.99315	0.982781	1.0409931
222194	RSBN1L	round spermatid basic protein 1-like	0.97966	0.939962	1.0167792
222223	KIAA1324L	KIAA1324-like	0.95458	0.972081	1.0488563
222229	LRWD1	leucine-rich repeats and WD repeat domain containing 1	0.99348	0.995191	1.0140171
222234	FAM185A	family with sequence similarity 185, member A	1.00236	0.933824	0.981137
222235	FBXL13	F-box and leucine-rich repeat protein 13	1.00008	0.991908	0.9981733

222236	NAPEPLD	N-acyl phosphatidylethanolamine phospholipase D	0.98863	0.970198	0.9839553
222255	ATXN7L1	ataxin 7-like 1	0.99777	0.947057	0.9428501
222256	CDHR3	cadherin-related family member 3	1.00047	1.001724	0.984145
222389	BEND7	BEN domain containing 7	1.01715	0.983542	0.9798287
222484	LNX2	ligand of numb-protein X 2	0.93879	1.003844	1.0490074
222487	GPR97	G protein-coupled receptor 97	1.01311	1.016314	0.9471231
222521	[No Symbo	[No Name]	1.00434	0.955837	0.9896552
222537	HS3ST5	heparan sulfate (glucosamine) 3-O-sulfotransferase 5	1.01968	1.009194	1.0217079
222545	GPRC6A	G protein-coupled receptor, family C, group 6, member A	1.00524	0.990353	1.0290115
222546	RFX6	regulatory factor X, 6	0.99847	0.992989	1.0383766
222553	SLC35F1	solute carrier family 35, member F1	1.0189	1.000656	1.0125196
222584	FAM83B	family with sequence similarity 83, member B	1.01713	1.005022	1.0080593
222611	GPR111	G protein-coupled receptor 111	1.00484	0.999271	0.9805241
222642	TSPO2	translocator protein 2	1.01003	0.977705	1.0083547
222643	UNC5CL	unc-5 homolog C (C. elegans)-like	1.01874	1.014275	1.0024309
222658	KCTD20	potassium channel tetramerisation domain containing 20	1.01847	1.052729	1.0301766
222659	PXT1	peroxisomal, testis specific 1	0.99833	0.978864	1.0217661
222662	LHFPL5	lipoma HMGIC fusion partner-like 5	1.00233	0.975015	1.0192616
222663	SCUBE3	signal peptide, CUB domain, EGF-like 3	0.9985	1.011691	1.0022531
222696	ZSCAN23	zinc finger and SCAN domain containing 23	0.98412	0.995045	1.0030296
222698	NKAPL	NFKB activating protein-like	0.9873	0.951534	0.9922426
222699	LOC222699	transducer of ERBB2, 2 pseudogene	0.99933	0.988664	1.0093597
222701	LOC222701	similar to zinc finger protein 91	1.03174	0.991036	0.9920372
222826	C6orf146	chromosome 6 open reading frame 146	0.98181	0.983855	1.038893
222865	TMEM130	transmembrane protein 130	1.01362	1.010179	1.0257035
222894	FERD3L	Fer3-like (Drosophila)	1.00828	1.026619	1.0579759
222950	C7orf51	chromosome 7 open reading frame 51	1.00114	1.009653	1.0247869
222962	SLC29A4	solute carrier family 29 (nucleoside transporters), member 4	0.97331	1.024056	1.0390103
223075	CCDC129	coiled-coil domain containing 129	1.00054	1.002894	0.9949848
223082	ZNRF2	zinc and ring finger 2	1.01582	0.963324	0.9609979
223117	SEMA3D	sema domain, immunoglobulin domain (Ig), short basic domain, secreted, (semaphorin) 3D	0.99972	1.014409	1.007255
225689	MAPK15	mitogen-activated protein kinase 15	0.99993	1.061651	1.0725132
245711	SPDYA	speedy homolog A (Xenopus laevis)	1.0047	0.995193	1.0149294

245802	MS4A6E	membrane-spanning 4-domains, subfamily A, member 6E	1.01249	1.033816	1.0097319
245806	VGLL2	vestigial like 2 (Drosophila)	1.01681	1.060072	1.0091843
245812	CNPY4	canopy 4 homolog (zebrafish)	0.96845	0.996699	1.0223479
245913	DEFB110	defensin, beta 110 locus	0.96947	1.015444	0.9844568
245915	DEFB112	defensin, beta 112	1.00477	0.966872	1.0309932
245929	DEFB115	defensin, beta 115	1.00604	1.065922	0.9855569
245930	DEFB116	defensin, beta 116	1.00185	1.003482	1.0921023
245932	DEFB119	defensin, beta 119	0.99895	1.02676	1.0101664
245934	DEFB121	defensin, beta 121	1.02227	1.01447	0.9134612
245936	DEFB123	defensin, beta 123	0.99518	1.012658	0.995667
245937	DEFB124	defensin, beta 124	0.99065	0.949875	0.9146854
245938	DEFB125	defensin, beta 125	0.99541	1.028459	0.9906998
245939	DEFB128	defensin, beta 128	1.03505	0.903105	1.017391
245972	ATP6V0D2	ATPase, H+ transporting, lysosomal 38kDa, V0 subunit d2	1.00928	1.021493	1.0293109
245973	ATP6V1C2	ATPase, H+ transporting, lysosomal 42kDa, V1 subunit C2	1.00739	0.991226	1.0312753
246119	TTTY10	testis-specific transcript, Y-linked 10 (non-protein coding)	1.00169	1.007649	1.007377
246126	CYorf15A	chromosome Y open reading frame 15A	0.99311	0.959874	1.0183516
246135	TAF9BP1	TAF9B RNA polymerase II, TATA box binding protein (TBP)-associated factor, 31kDa pseudogene 1	1.0139	1.010682	1.0043241
246175	CNOT6L	CCR4-NOT transcription complex, subunit 6-like	0.99585	1.024601	0.9973303
246176	GAS2L2	growth arrest-specific 2 like 2	0.99861	1.018557	1.0333242
246181	AKR7L	aldo-keto reductase family 7-like	1.01334	0.982229	0.9928797
246182	AKR7A2P1	aldo-keto reductase family 7, member A2 pseudogene 1	0.99801	1.009984	1.0499557
246184	CDC26	cell division cycle 26 homolog (S. cerevisiae)	0.95846	0.996575	0.9878962
246213	SLC17A8	solute carrier family 17 (sodium-dependent inorganic phosphate cotransporter), member 8	0.98379	0.98407	1.0082202
246243	RNASEH1	ribonuclease H1	1.01151	0.934973	1.0398424
246269	LACE1	lactation elevated 1	1.00588	0.990827	1.0088263
246329	STAC3	SH3 and cysteine rich domain 3	0.96618	0.896344	1.0686982
246330	PELI3	pellino homolog 3 (Drosophila)	0.95109	0.982419	1.0199317
246704	NCRNA003	non-protein coding RNA 315	0.98452	1.080039	1.0235557
246716	YWHAQP2	YWHAQ pseudogene 2	0.98993	1.009417	0.9288486
246717	TCEB2P1	transcription elongation factor B (SIII), polypeptide 2 (18kDa, elongin B) pseudogene 1	0.98641	0.990864	1.0111302
246720	DYNLL1P1	dynein, cytoplasmic, light polypeptide pseudogene	1.00069	0.943547	0.9676385
246723	LOC246723	ATP synthase, H+ transporting, mitochondrial F0 complex, subunit d pseudogene	0.89348	1.034283	1.0939168

246731	PPIHP1	peptidylprolyl isomerase H (cyclophilin H) pseudogene 1	0.98651	1.057924	1.0668955
246737	YWHAZP4	tyrosine 3-monooxygenase/tryptophan 5-monooxygenase activation protein, zeta pseudogene 4	0.99574	0.962363	1.0370687
246778	IL27	interleukin 27	0.99179	0.995308	1.0044093
246784	LOC246784	homolog of C. elegans smu-1 pseudogene	0.95019	0.935362	1.1602541
246785	LOC246785	Obg-like ATPase 1 pseudogene	0.98262	1.06564	0.9483148
252839	TMEM9	transmembrane protein 9	0.98282	0.970254	0.9928652
252884	ZNF396	zinc finger protein 396	0.98027	0.985031	1.0078262
252948	TTTY16	testis-specific transcript, Y-linked 16 (non-protein coding)	1.02734	0.990024	1.0809683
252950	TTTY18	testis-specific transcript, Y-linked 18 (non-protein coding)	1.02984	1.050388	0.9620713
252952	TTTY19	testis-specific transcript, Y-linked 19 (non-protein coding)	1.00202	0.96241	0.9918956
252954	TTTY22	testis-specific transcript, Y-linked 22 (non-protein coding)	1.01087	0.995141	0.9454757
252956	TPM3P1	tropomyosin 3 pseudogene 1	1.02023	0.954202	1.0388237
252969	NEIL2	nei endonuclease VIII-like 2 (E. coli)	0.99103	0.945824	1.0564456
252983	STXBP4	syntaxin binding protein 4	0.97376	0.957877	1.0296952
252995	FNDC5	fibronectin type III domain containing 5	1.00271	1.003544	0.993655
253012	HEPACAM2	HEPACAM family member 2	1.00359	1.008674	0.9987668
253017	TECRL	trans-2,3-enoyl-CoA reductase-like	1.00203	1.034083	1.0144666
253018	HCG27	HLA complex group 27	1.02067	0.895343	0.9395167
253039	LOC253039	hypothetical LOC253039	0.89208	0.951902	0.9109487
253128	C12orf33	chromosome 12 open reading frame 33	0.99071	1.034432	1.0160028
253143	PRR14L	proline rich 14-like	0.94561	0.916603	1.0017148
253152	EPHX4	epoxide hydrolase 4	1.00138	1.045684	1.0408219
253190	SERHL2	serine hydrolase-like 2	0.97576	0.987674	1.032079
253260	RICTOR	RPTOR independent companion of MTOR, complex 2	0.9865	1.0197	0.9197128
253314	EIF4E1B	eukaryotic translation initiation factor 4E family member 1B	1.00409	0.991068	1.0201538
253430	IPMK	inositol polyphosphate multikinase	0.94243	1.064733	1.0024703
253461	ZBTB38	zinc finger and BTB domain containing 38	1.02535	0.991345	0.9943145
253482	[No Symbo	[No Name]	0.96785	0.976368	0.9616137
253512	SLC25A30	solute carrier family 25, member 30	0.95062	0.964003	1.0856372
253558	LCLAT1	lysocardiolipin acyltransferase 1	0.95882	0.975865	1.0467599
253559	CADM2	cell adhesion molecule 2	1.00034	1.007283	0.9838212
253582	C6orf191	chromosome 6 open reading frame 191	1.00688	1.020053	0.962089
253635	CCDC75	coiled-coil domain containing 75	0.99324	1.044792	1.1239928

253639	ZNF620	zinc finger protein 620	1.00056	1.021492	0.9957304
253650	ANKRD18A	ankyrin repeat domain 18A	1.01849	0.959328	1.0058003
253665	TCEB2P2	transcription elongation factor B (SIII), polypeptide 2 (18kDa, elongin B) pseudogene 2	0.94716	1.006593	1.1339993
253714	MMS22L	MMS22-like, DNA repair protein	0.99891	0.991949	1.0091988
253724	GNN	Grp94 neighboring nucleotidase pseudogene	1.00083	1.012975	1.0122825
253738	EBF3	early B-cell factor 3	0.99642	1.012483	1.0181277
253769	WDR27	WD repeat domain 27	0.99836	1.001051	1.0247645
253782	LASS6	LAG1 homolog, ceramide synthase 6	0.98075	0.927129	1.0725563
253820	[No Symbo	[No Name]	1.00815	1.008915	1.0502743
253827	MSRB3	methionine sulfoxide reductase B3	0.99413	0.997213	1.0221903
253832	ZDHHC20	zinc finger, DHHC-type containing 20	0.98125	0.990344	1.0140324
253868	C20orf200	chromosome 20 open reading frame 200	0.99587	1.000621	1.0247271
253933	OR7E39P	olfactory receptor, family 7, subfamily E, member 39 pseudogene	1.05666	0.992562	0.9828311
253935	ANGPTL5	angiopoietin-like 5	0.99958	1.015301	1.0175136
253943	YTHDF3	YTH domain family, member 3	0.98868	0.936788	1.0449629
253959	RALGAPA1	Ral GTPase activating protein, alpha subunit 1 (catalytic)	0.97453	0.965555	0.917281
253970	SFTA3	surfactant associated 3	1.00018	1.007545	0.9963513
253980	KCTD13	potassium channel tetramerisation domain containing 13	0.98422	0.959133	1.0617924
253982	ASPHD1	aspartate beta-hydroxylase domain containing 1	0.99952	1.023389	1.026747
254013	METTL20	methyltransferase like 20	1.00088	0.970693	0.9795287
254028	LOC254028	hypothetical LOC254028	1	1.074482	1.0531981
254042	METAP1D	methionyl aminopeptidase type 1D (mitochondrial)	0.99906	0.959527	1.0054923
254048	UBN2	ubinuclein 2	1.01398	1.034612	1.0007955
254050	LRRC43	leucine rich repeat containing 43	0.9832	0.987113	0.9942207
254065	BRWD3	bromodomain and WD repeat domain containing 3	0.94695	1.02293	1.1384121
254102	EHBP1L1	EH domain binding protein 1-like 1	0.97141	1.065356	1.0315298
254122	SNX32	sorting nexin 32	0.99131	1.004344	1.0140921
254158	CXorf58	chromosome X open reading frame 58	0.97211	1	1.0085841
254170	FBXO33	F-box protein 33	0.93724	0.933937	0.9591689
254173	TTLL10	tubulin tyrosine ligase-like family, member 10	1.00032	0.970039	0.9956934
254187	TSGA10IP	testis specific, 10 interacting protein	0.99507	0.986737	0.9813303
254225	RNF169	ring finger protein 169	0.95437	0.971856	1.0270629
254228	FAM26E	family with sequence similarity 26, member E	0.96418	0.973567	0.9631876

254240	BPIL2	bactericidal/permeability-increasing protein-like 2	0.9944	1.004186	0.9914815
254251	LCORL	ligand dependent nuclear receptor corepressor-like	0.99215	1.019212	0.9889577
254263	CNIH2	cornichon homolog 2 (Drosophila)	1.01061	1.039505	0.9912051
254268	AKNAD1	AKNA domain containing 1	1.00891	1.010138	1.0275415
254295	PHYHD1	phytanoyl-CoA dioxygenase domain containing 1	0.99599	1.017555	0.9888521
254312	LOC254312	hypothetical LOC254312	1.00447	0.99853	1.0237618
254359	ZDHHC24	zinc finger, DHHC-type containing 24	1.00287	0.949274	0.978141
254394	MCM9	minichromosome maintenance complex component 9	0.96554	0.910125	1.0536614
254427	C10orf47	chromosome 10 open reading frame 47	0.96218	0.919631	0.9765433
254428	SLC41A1	solute carrier family 41, member 1	0.98169	1.021841	0.9455296
254439	C11orf86	chromosome 11 open reading frame 86	0.99069	1.005829	1.0300719
254528	C16orf73	chromosome 16 open reading frame 73	0.99754	1.046746	1.0464342
254531	LPCAT4	lysophosphatidylcholine acyltransferase 4	0.97027	1.039685	1.0071593
254552	NUDT8	nudix (nucleoside diphosphate linked moiety X)-type motif 8	0.99562	0.962548	1.0915385
254664	MAPK6PS2	mitogen-activated protein kinase 6 pseudogene 2	0.98775	1.014805	1.0302372
254773	LYG2	lysozyme G-like 2	0.99233	1.005418	0.9866482
254778	C8orf46	chromosome 8 open reading frame 46	1.0151	1.017042	0.990144
254783	OR6C74	olfactory receptor, family 6, subfamily C, member 74	1.00764	0.971654	1.0495679
254827	NAALADL2	N-acetylated alpha-linked acidic dipeptidase-like 2	1.00833	1.013657	1.0123244
254863	C17orf61	chromosome 17 open reading frame 61	0.96557	0.965848	1.0411523
254879	OR2T6	olfactory receptor, family 2, subfamily T, member 6	1.0438	0.989444	1.0423822
254887	ZDHHC23	zinc finger, DHHC-type containing 23	0.99289	0.979006	1.0403413
254910	LCE5A	late cornified envelope 5A	1.02903	1.027496	0.9744081
254950	KRTAP15-1	keratin associated protein 15-1	1.02774	1.01284	1.0089081
254956	MORN5	MORN repeat containing 5	1.00548	0.980643	0.989
255022	CALHM1	calcium homeostasis modulator 1	0.99328	1.01253	1.0080262
255027	MPV17L	MPV17 mitochondrial membrane protein-like	0.94834	1.035312	1.1146757
255043	TMEM86B	transmembrane protein 86B	0.99271	1.003806	1.007598
255057	C19orf26	chromosome 19 open reading frame 26	0.98292	1.033092	1.0492019
255061	TAC4	tachykinin 4 (hemokinin)	1.02369	0.981873	0.96149
255082	CASC2	cancer susceptibility candidate 2	0.99544	1.031193	0.9986847
255101	CCDC108	coiled-coil domain containing 108	1.00842	1.00147	1.0200483
255104	TMCO4	transmembrane and coiled-coil domains 4	0.99703	0.999453	0.9768063

255119	C4orf22	chromosome 4 open reading frame 22	1.00897	1.008382	1.0100914
255130	LOC255130	hypothetical LOC255130	0.9959	1.023065	1.0179337
255167	LOC255167	hypothetical LOC255167	1.04679	0.935865	1.0178722
255180	FLJ38723	hypothetical FLJ38723	1.01215	1.028396	0.9919012
255189	PLA2G4F	phospholipase A2, group IVF	0.9935	1.00385	1.0154069
255193	C19orf34	chromosome 19 open reading frame 34	0.98151	1.020724	1.0100352
255220	TXNDC8	thioredoxin domain containing 8 (spermatozoa)	1.00957	1.02679	1.0286986
255231	MCOLN2	mucolipin 2	1.01768	1.140711	1.0050167
255239	ANKK1	ankyrin repeat and kinase domain containing 1	0.99715	0.996024	0.985984
255252	LRRC57	leucine rich repeat containing 57	0.96032	0.922938	1.0159902
255275	MYADML2	myeloid-associated differentiation marker-like 2	0.99547	1.000823	1.0239502
255308	LOC255308	eukaryotic translation initiation factor 2, subunit 3 gamma, 52kDa pseudogene	1.00285	1.002984	1.0069013
255324	EPGN	epithelial mitogen homolog (mouse)	0.993	0.971939	1.0160498
255330	C3orf46	nucleoporin 210kDa pseudogene	1.00708	1.016373	1.0518642
255349	TMEM211	transmembrane protein 211	0.97103	0.990809	1.0201408
255352	C10orf93	chromosome 10 open reading frame 93	1.00456	0.990351	1.0196346
255374	MBLAC1	metallo-beta-lactamase domain containing 1	0.98757	1.068154	0.9932846
255394	TCP11L2	t-complex 11 (mouse)-like 2	0.93259	0.95963	1.072486
255403	ZNF718	zinc finger protein 718	1.01621	0.984616	1.0102197
255411	LOC255411	hypothetical LOC255411	0.98273	1.02983	1.0265073
255426	RASGEF1C	RasGEF domain family, member 1C	1.00135	1.026502	0.9944278
255480	LOC255480	hypothetical LOC255480	1.00675	0.954479	0.9954524
255488	RNF144B	ring finger protein 144B	1.0489	1.113688	0.9944593
255519	LOC255519	high mobility group protein B3-like	1.01183	1.023674	0.9477322
255520	ELMOD2	ELMO/CED-12 domain containing 2	0.9951	0.993313	1.0005603
255631	COL24A1	collagen, type XXIV, alpha 1	1	1.002598	1.0083993
255725	OR52B2	olfactory receptor, family 52, subfamily B, member 2	1.02565	1.022749	1.0247934
255738	PCSK9	proprotein convertase subtilisin/kexin type 9	0.99658	1.004239	1.0236787
255743	NPNT	nephronectin	1.00135	0.981211	1.0092809
255758	TCTEX1D2	Tctex1 domain containing 2	1.00995	0.990475	1.0273814
255762	PDZD9	PDZ domain containing 9	0.98485	0.993239	0.9807786
255783	PRR24	proline rich 24	0.96413	0.979489	1.0331494
255798	C3orf43	chromosome 3 open reading frame 43	1.00889	1.042661	1.040926

255809	C19orf38	chromosome 19 open reading frame 38	0.97672	0.936729	1.0253879
255877	BCL6B	B-cell CLL/lymphoma 6, member B	0.99354	1.022769	1.0108091
255919	TMEM188	transmembrane protein 188	0.98159	0.945915	0.9279724
255926	ADAM5P	ADAM metallopeptidase domain 5, pseudogene	0.99003	1.0124	0.9897263
255928	SYT14	synaptotagmin XIV	1.00131	1.004477	1.0079471
255967	PAN3	PAN3 poly(A) specific ribonuclease subunit homolog (S. cerevisiae)	0.98104	0.994465	0.9885947
256006	ANKRD31	ankyrin repeat domain 31	0.98475	0.996526	0.9642397
256051	ZNF549	zinc finger protein 549	0.93632	0.989695	1.0640357
256076	COL6A5	collagen, type VI, alpha 5	1.02202	0.974761	0.9826836
256085	LOC256085	tryptophan rich basic protein pseudogene	1.04465	1.023517	1.0322888
256112	[No Symbo	[No Name]	0.97318	0.950313	1.0959507
256126	SYCE2	synaptonemal complex central element protein 2	0.98164	1.034981	0.9664836
256130	TMEM196	transmembrane protein 196	1.00668	1.014152	0.9976558
256144	OR4C3	olfactory receptor, family 4, subfamily C, member 3	1.00358	0.978655	1.0337799
256148	OR4S1	olfactory receptor, family 4, subfamily S, member 1	0.96938	1.092068	1.0129676
256158	HMCN2	hemicentin 2	0.99379	0.987456	1.030497
256190	OR4C50P	olfactory receptor, family 4, subfamily C, member 50 pseudogene	0.99425	1.029727	1.0206573
256227	STEAP1B	STEAP family member 1B	1.01929	1.026842	1.0243942
256236	NAPSB	napsin B aspartic peptidase pseudogene	0.99701	0.994671	1.0232774
256281	NUDT14	nudix (nucleoside diphosphate linked moiety X)-type motif 14	0.97775	1.079146	1.0613225
256297	PTF1A	pancreas specific transcription factor, 1a	0.98508	0.900977	1.0850038
256302	C17orf103	chromosome 17 open reading frame 103	0.96497	0.986743	1.051433
256309	CCDC110	coiled-coil domain containing 110	0.99056	0.998892	1.0302263
256329	C11orf35	chromosome 11 open reading frame 35	0.97863	0.951019	1.0821387
256355	RPS2P32	ribosomal protein S2 pseudogene 32	0.98586	1.019536	1.0248129
256356	GK5	glycerol kinase 5 (putative)	1.01572	1.031311	1.0101449
256364	EML3	echinoderm microtubule associated protein like 3	0.9606	1.040993	0.970287
256374	LOC256374	peptidyl-prolyl cis-trans isomerase A-like	1.00805	0.994988	1.0074057
256380	SCML4	sex comb on midleg-like 4 (Drosophila)	0.96636	1.022684	0.941321
256394	SERPINA11	serpin peptidase inhibitor, clade A (alpha-1 antiproteinase, antitrypsin), member 11	1.01508	1.046179	0.950517
256435	ST6GALNAC	ST6 (alpha-N-acetyl-neuraminyl-2,3-beta-galactosyl-1,3)-N-acetylgalactosaminide alpha-2,6-sialyltransferase	0.99783	1.023679	0.9773637
256471	MFSD8	major facilitator superfamily domain containing 8	0.99543	0.975436	1.0731183
256472	TMEM151A	transmembrane protein 151A	0.99391	0.974271	1.1630732

256483	LOC256483	hypothetical protein LOC256483	1.00933	0.988991	1.0252536
256536	TCERG1L	transcription elongation regulator 1-like	0.99028	1.005293	0.9999541
256586	LYSMD2	LysM, putative peptidoglycan-binding, domain containing 2	0.94317	1.078417	1.0129791
256643	CXorf23	chromosome X open reading frame 23	0.95461	1.045726	1.0091188
256646	C15orf55	chromosome 15 open reading frame 55	1.00097	0.997246	1.0056888
256691	MAMDC2	MAM domain containing 2	1.01346	0.995142	1.0080296
256710	GLIPR1L1	GLI pathogenesis-related 1 like 1	0.99652	1.007535	1.0370844
256714	MAP7D2	MAP7 domain containing 2	0.9851	1.033674	1.0100381
256764	WDR72	WD repeat domain 72	0.99135	1.011179	0.9839838
256815	C10orf67	chromosome 10 open reading frame 67	0.99949	0.99358	1.0143451
256892	OR51F1	olfactory receptor, family 51, subfamily F, member 1	1.02149	0.955975	0.9819556
256933	NPB	neuropeptide B	0.99797	1.04929	1.0942358
256949	KANK3	KN motif and ankyrin repeat domains 3	0.99779	0.986095	1.0366567
256957	C17orf66	chromosome 17 open reading frame 66	1.03254	1.06834	0.9182281
256979	SUN3	Sad1 and UNC84 domain containing 3	1.0125	0.996483	1.0046978
256987	SERINC5	serine incorporator 5	0.96993	0.922998	0.9172469
257000	PLAC2	placenta-specific 2 (non-protein coding)	1.00824	1.048596	0.9710452
257019	FRMD3	FERM domain containing 3	0.98642	0.985289	0.9778171
257039	[No Symbo	[No Name]	0.9923	1.01192	1.014503
257044	C1orf101	chromosome 1 open reading frame 101	1.00142	0.998816	1.0055629
257062	TMEM146	transmembrane protein 146	0.99252	0.985856	0.9986961
257068	PLCXD2	phosphatidylinositol-specific phospholipase C, X domain containing 2	1.0025	1.022825	0.9931554
257093	PSME2P1	proteasome activator subunit 2 pseudogene 1	0.98738	0.996798	1.0694549
257101	ZNF683	zinc finger protein 683	1.0175	1.04308	0.9800914
257103	NCRNA002	non-protein coding RNA 205	0.97416	0.948393	0.9989208
257106	ARHGAP30	Rho GTPase activating protein 30	0.99654	0.955362	0.9585527
257144	GCET2	germinal center expressed transcript 2	0.94568	1.011627	0.9871137
257160	RNF214	ring finger protein 214	0.93143	0.917581	0.9891917
257169	C9orf43	chromosome 9 open reading frame 43	0.99763	1.019225	1.0024046
257177	C1orf192	chromosome 1 open reading frame 192	1.01189	0.971583	1.0004516
257194	NEGR1	neuronal growth regulator 1	0.99939	0.986841	1.0000928
257202	GPX6	glutathione peroxidase 6 (olfactory)	1.00481	0.988448	1.0111275
257203	DSCR9	Down syndrome critical region gene 9 (non-protein coding)	0.98908	1.000827	1.0128586

257218	SHPRH	SNF2 histone linker PHD RING helicase	0.98114	0.965522	1.0836659
257236	CCDC96	coiled-coil domain containing 96	0.98122	0.955595	1.0630805
257240	KLHL34	kelch-like 34 (Drosophila)	0.97535	1.002706	1.0914262
257313	UTS2D	urotensin 2 domain containing	1.01405	0.990163	1.0252489
257357	NCRNA002	non-protein coding RNA 257	0.96414	1.052203	0.9513574
257364	SNX33	sorting nexin 33	0.98374	0.9647	1.0439355
257396	LOC257396	hypothetical LOC257396	0.96892	0.979745	1.0140918
257397	TAB3	TGF-beta activated kinase 1/MAP3K7 binding protein 3	0.98324	1.045473	0.9572301
257407	C2orf72	chromosome 2 open reading frame 72	1.00718	0.963136	1.002702
257415	FAM133B	family with sequence similarity 133, member B	1.02226	0.943107	1.0111587
257629	ANKS4B	ankyrin repeat and sterile alpha motif domain containing 4B	0.98558	1.01397	0.9748183
258010	SVIP	small VCP/p97-interacting protein	0.91615	0.824755	0.9146179
259173	ALS2CL	ALS2 C-terminal like	0.97923	0.979005	0.9624326
259197	NCR3	natural cytotoxicity triggering receptor 3	0.97991	0.970337	1.0075462
259215	LY6G6F	lymphocyte antigen 6 complex, locus G6F	0.91456	0.898438	0.9468258
259217	HSPA12A	heat shock 70kDa protein 12A	0.98684	0.972331	1.0002696
259230	SGMS1	sphingomyelin synthase 1	0.97655	0.970405	1.0446142
259232	NALCN	sodium leak channel, non-selective	0.99574	1.006303	1.0016132
259234	DSCR10	Down syndrome critical region gene 10 (non-protein coding)	1.01093	1.046119	0.9904068
259236	TMIE	transmembrane inner ear	0.9873	1.026808	1.0504729
259239	WFDC11	WAP four-disulfide core domain 11	0.98238	1.093409	1.035329
259240	WFDC9	WAP four-disulfide core domain 9	0.98846	0.986019	1.0189056
259249	MRGPRX1	MAS-related GPR, member X1	0.96154	0.965119	1.0409441
259266	ASPM	asp (abnormal spindle) homolog, microcephaly associated (Drosophila)	0.99593	0.986565	1.032375
259282	BOD1L	biorientation of chromosomes in cell division 1-like	1.01846	0.960198	1.0031958
259286	TAS2R40	taste receptor, type 2, member 40	0.94698	1.043877	1.0770372
259287	TAS2R41	taste receptor, type 2, member 41	1.02755	0.933879	0.9141621
259307	IL4I1	interleukin 4 induced 1	0.97419	1.007629	0.9656381
259308	C9orf144B	transmembrane protein C9orf144B	0.99191	0.958393	1.0189178
260293	CYP4X1	cytochrome P450, family 4, subfamily X, polypeptide 1	1.00439	1.008384	1.0279172
260332	ECEL1P3	endothelin converting enzyme-like 1, pseudogene 3	1.02786	1.042957	1.056639
260335	ELF2P2	E74-like factor 2 pseudogene 2	0.99611	1.010929	1.0592026
260337	UHRF2P1	ubiquitin-like with PHD and ring finger domains 2 pseudogene 1	0.99109	1.027686	1.0396541

260338	LOC260338	HSA12cenq11 beta-tubulin 4Q (TUBB4Q) pseudogene	0.97175	1.027261	1.0511787
260339	LOC260339	transcription factor A, mitochondrial pseudogene	1.01697	0.97463	0.9932664
260340	TFAMP2	transcription factor A, mitochondrial pseudogene 2	0.96839	0.980806	0.9963913
260341	TFAMP1	transcription factor A, mitochondrial pseudogene 1	0.98193	0.970823	0.9983786
260342	BA345E19.1	COPS8 pseudogene	1.02526	1.041541	1.1081477
260422	LOC260422	actin related protein 2/3 complex subunit 1A pseudogene	1.01491	1.020298	0.9844091
260425	MAGI3	membrane associated guanylate kinase, WW and PDZ domain containing 3	0.99719	0.987203	0.9723461
260429	PRSS33	protease, serine, 33	1.0053	1.01552	1.0169446
260434	PYDC1	PYD (pyrin domain) containing 1	1.01446	0.960855	1.0480024
260436	C4orf7	chromosome 4 open reading frame 7	1.00701	0.994428	1.0336996
261726	TIPRL	TIP41, TOR signaling pathway regulator-like (S. cerevisiae)	0.99821	0.959331	0.9879613
261729	STEAP2	six transmembrane epithelial antigen of the prostate 2	1.0183	1.019386	1.0018269
261734	NPHP4	nephronophthisis 4	1.00952	1.015776	1.0066619
266629	SEC14L3	SEC14-like 3 (S. cerevisiae)	0.98439	1.01615	1.0315933
266655	NCRNA000	non-protein coding RNA 94	0.98755	1.005942	1.1107321
266673	ZNF402P	zinc finger protein 402, pseudogene	0.99141	0.980637	0.9695972
266675	BEST4	bestrophin 4	1.00432	0.998743	1.0005476
266683	LOC266683	dendritic cell protein pseudogene	1.00027	0.977046	1.098666
266695	PHF2P1	PHD finger protein 2 pseudogene 1	0.98979	1.013795	1.0096004
266697	POM121L4	POM121 membrane glycoprotein-like 4 pseudogene	1.00187	0.992131	0.958549
266722	HS6ST3	heparan sulfate 6-O-sulfotransferase 3	1.00155	1.012643	1.00869
266724	HSPA9P1	heat shock 70kDa protein 9 pseudogene 1	1.03009	1.034791	1.0416511
266727	MDGA1	MAM domain containing glycosylphosphatidylinositol anchor 1	0.99771	0.987319	1.042134
266743	NPAS4	neuronal PAS domain protein 4	1.00342	0.993515	1.0258621
266747	RGL4	ral guanine nucleotide dissociation stimulator-like 4	0.99714	0.988847	0.9849468
266783	PSMD2P1	proteasome 26S subunit, non-ATPase, 2 pseudogene 1	1.00179	1.017349	1.0143786
266786	NUP50P3	nucleoporin 50 pseudogene 3	0.98337	0.90519	1.0314395
266812	NAP1L5	nucleosome assembly protein 1-like 5	0.99819	0.906099	0.9040471
266954	CABYRP1	calcium binding tyrosine-(Y)-phosphorylation regulated pseudogene 1	0.98959	0.969712	0.9911696
266977	GPR110	G protein-coupled receptor 110	1.01249	1.019773	0.9931657
267002	PGBD2	piggyBac transposable element derived 2	0.99545	0.979662	1.025947
267005	PGBD3P1	piggyBac transposable element derived 3 pseudogene 1	0.99086	1.020805	0.9627646
267007	PGBD3P3	piggyBac transposable element derived 3 pseudogene 3	1.00981	0.988193	1.0050861

267008	PGBD3P4	piggyBac transposable element derived 3 pseudogene 4	1.01254	1.003027	0.9901682
267009	RPS9P2	ribosomal protein S9 pseudogene 2	0.96357	1.003776	1.0418423
267012	DAOA	D-amino acid oxidase activator	1.00899	1.041992	1.0457372
276719	SOCS2P1	suppressor of cytokine signaling 2 pseudogene 1	1.0024	0.994477	1.0042677
280636	C11orf31	chromosome 11 open reading frame 31	0.94574	0.996965	1.0626215
280637	PSMD7P1	proteasome (prosome, macropain) 26S subunit, non-ATPase, 7 pseudogene 1	0.99038	0.998295	1.0182257
280644	PSMD10P2	proteasome 26S subunit, non-ATPase, 10 pseudogene 2	0.99229	1.014179	1.0632167
280655	C14orf19	immunoglobulin (CD79A) binding protein 1 pseudogene	1.00258	0.973858	1.0786157
280657	SSX6	synovial sarcoma, X breakpoint 6 (pseudogene)	0.97467	1.014778	1.0064122
280658	SSX7	synovial sarcoma, X breakpoint 7	1.04926	1.071625	1.0140314
280664	WFDC10B	WAP four-disulfide core domain 10B	1.01335	0.981529	0.9791016
282618	IL29	interleukin 29 (interferon, lambda 1)	0.96446	0.975352	0.9791005
282679	AQP11	aquaporin 11	0.96516	1.002903	1.0211593
282694	NASPP1	nuclear autoantigenic sperm protein pseudogene 1	0.98155	1.027724	1.0194661
282756	OR7E149P	olfactory receptor, family 7, subfamily E, member 149 pseudogene	0.99361	1.052806	1.037143
282763	OR51B5	olfactory receptor, family 51, subfamily B, member 5	1.01832	0.93312	1.0315405
282770	OR10AG1	olfactory receptor, family 10, subfamily AG, member 1	0.98522	1.005996	1.0070536
282775	OR5J2	olfactory receptor, family 5, subfamily J, member 2	0.99867	1.002357	1.0010005
282776	OR8V1P	olfactory receptor, family 8, subfamily V, member 1 pseudogene	1.00329	1.009887	1.0279085
282786	OR8G7P	olfactory receptor, family 8, subfamily G, member 7 pseudogene	0.99111	0.988379	0.997528
282790	OR5BT1P	olfactory receptor, family 5, subfamily BT, member 1 pseudogene	1.03485	1.00377	0.9664743
282791	OR8T1P	olfactory receptor, family 8, subfamily T, member 1 pseudogene	1.01089	1.030272	0.973579
282801	OR7E148P	olfactory receptor, family 7, subfamily E, member 148 pseudogene	0.98518	1.05751	0.9405929
282808	RAB40AL	RAB40A, member RAS oncogene family-like	0.97497	0.9438	0.9847012
282809	POC1B	POC1 centriolar protein homolog B (Chlamydomonas)	0.93309	1.03674	1.0711852
282890	ZNF311	zinc finger protein 311	0.99405	0.97228	0.9848842
282966	C10orf53	chromosome 10 open reading frame 53	0.99154	1.001915	1.0433555
282967	MAPK6PS6	mitogen-activated protein kinase 6 pseudogene 6	1.01691	1.031632	1.0416599
282969	C10orf125	chromosome 10 open reading frame 125	0.96236	0.98989	1.1028508
282973	JAKMIP3	Janus kinase and microtubule interacting protein 3	0.99672	0.998583	1.0083995
282974	STK32C	serine/threonine kinase 32C	0.98462	1.012969	1.0151771
282991	BLOC1S2	biogenesis of lysosomal organelles complex-1, subunit 2	0.9375	1.027374	0.9741606
282992	[No Symbo	[No Name]	0.98532	1.033394	1.0329106

282996	RBM20	RNA binding motif protein 20	0.99772	0.992134	1.0134479
282997	LOC282997	hypothetical LOC282997	0.896	0.971965	0.9026655
283011	FLJ37201	tigger transposable element derived 2 pseudogene	1.04117	1.011777	1.0319615
283028	LOC283028	hypothetical protein LOC283028	0.99067	1.02381	1.0335174
283078	MKX	mohawk homeobox	0.99342	1.031579	1.0239666
283093	OR4C12	olfactory receptor, family 4, subfamily C, member 12	0.9645	1.050658	0.9561913
283102	KRT8P41	keratin 8 pseudogene 41	0.9989	1.004815	1.0303937
283104	LOC283104	hypothetical LOC283104	1.0052	0.984881	1.0034302
283110	OR52Z1	olfactory receptor, family 52, subfamily Z, member 1 (gene/pseudogene)	0.97909	0.982085	1.0439584
283111	OR51V1	olfactory receptor, family 51, subfamily V, member 1	1.01546	1.100883	0.9557112
283120	H19	H19, imprinted maternally expressed transcript (non-protein coding)	0.97375	0.974262	1.0136242
283129	C11orf85	chromosome 11 open reading frame 85	1.00346	1.018114	1.0023831
283130	SLC25A45	solute carrier family 25, member 45	0.95356	0.950176	0.9767106
283135	[No Symbo	[No Name]	0.98626	1.019058	1.0003215
283143	LOC283143	hypothetical LOC283143	0.98064	1.007002	1.0155789
283149	BCL9L	B-cell CLL/lymphoma 9-like	0.95663	1.048215	0.9735978
283152	CCDC153	coiled-coil domain containing 153	1.01077	0.956197	0.9682433
283155	[No Symbo	[No Name]	0.96256	1.026395	1.1161061
283159	OR8D1	olfactory receptor, family 8, subfamily D, member 1	0.96911	1.004965	1.0068736
283160	OR8D2	olfactory receptor, family 8, subfamily D, member 2	0.97546	1.028699	1.0364153
283162	OR8B4	olfactory receptor, family 8, subfamily B, member 4	1.01922	0.986981	1.0204175
283165	NCRNA002	non-protein coding RNA 288	0.98019	0.987248	1.0332467
283171	C11orf44	chromosome 11 open reading frame 44	1.00084	0.956397	1.0026425
283174	LOC283174	hypothetical LOC283174	0.96953	1.003358	1.0581634
283189	OR9G4	olfactory receptor, family 9, subfamily G, member 4	0.97664	0.977805	0.9969414
283193	OR5AZ1P	olfactory receptor, family 5, subfamily AZ, member 1 pseudogene	1.00468	1.004957	1.0279833
283197	NCRNA003	non-protein coding RNA 301	1.00307	0.987367	0.9471735
283208	P4HA3	prolyl 4-hydroxylase, alpha polypeptide III	0.99531	0.990625	0.9957106
283209	PGM2L1	phosphoglucomutase 2-like 1	0.91975	0.904982	1.0556109
283212	KLHL35	kelch-like 35 (Drosophila)	1.00459	1.018365	1.0019151
283218	DKFZp434E	hypothetical DKFZp434E1119	1.00697	0.968457	1.0544888
283219	KCTD21	potassium channel tetramerisation domain containing 21	0.97012	0.966872	0.9794156
283226	FNTAP1	farnesyltransferase, CAAX box, alpha pseudogene 1	0.97755	1.010922	1.1028105

283229	EFCAB4A	EF-hand calcium binding domain 4A	0.98387	0.974025	1.0596228
283232	TMEM80	transmembrane protein 80	0.99084	0.982479	1.0033595
283234	CCDC88B	coiled-coil domain containing 88B	0.96543	1.069967	1.0410969
283237	TTC9C	tetratricopeptide repeat domain 9C	0.92152	0.972602	1.0847694
283238	SLC22A24	solute carrier family 22, member 24	1.00236	0.984931	1.0101246
283248	RCOR2	REST corepressor 2	0.99073	0.972516	1.0592397
283254	HARBI1	harbinger transposase derived 1	0.93002	0.931985	1.0211062
283257	TRIM49B	tripartite motif-containing protein 49B-like	0.99556	1.026798	1.0223275
283284	IGSF22	immunoglobulin superfamily, member 22	1	1.002837	0.9841997
283294	[No Symbo	[No Name]	0.97397	1.006425	0.9308841
283298	OLFML1	olfactomedin-like 1	1.01688	1.005773	1.0084926
283303	C11orf36	chromosome 11 open reading frame 36	1.00381	1.026793	1.025819
283310	OTOGL	otogelin-like	1.0067	1.012625	1.0034486
283314	MATL2963	hypothetical LOC283314	1.00394	1.020413	0.99389
283316	CD163L1	CD163 molecule-like 1	0.99831	0.999173	1.005224
283332	LOC283332	hypothetical LOC283332	0.99069	0.929338	1.0187305
283337	ZNF740	zinc finger protein 740	0.94693	0.962469	1.2017471
283345	RPL13P5	ribosomal protein L13 pseudogene 5	0.9611	1.016207	1.0506178
283349	RASSF3	Ras association (RalGDS/AF-6) domain family member 3	0.96547	1.028474	0.9346928
283365	OR6C6	olfactory receptor, family 6, subfamily C, member 6	0.97616	1.015532	1.0349265
283373	ANKRD52	ankyrin repeat domain 52	0.97794	0.999699	1.0952186
283375	SLC39A5	solute carrier family 39 (metal ion transporter), member 5	1.00092	0.974869	1.0131265
283377	SPRYD4	SPRY domain containing 4	0.98373	0.962077	1.0133143
283383	GPR133	G protein-coupled receptor 133	1.00194	0.998294	1.0260134
283385	MORN3	MORN repeat containing 3	0.97029	1.034779	0.9499792
283392	LOC283392	hypothetical LOC283392	0.98743	1.004075	0.993645
283398	LOC283398	succinyl-CoA ligase [GDP-forming] subunit beta, mitochondrial pseudogene	1.0405	1.028117	0.9710964
283416	C12orf61	chromosome 12 open reading frame 61	0.97797	0.957413	0.9567928
283417	DPY19L2	dpy-19-like 2 (C. elegans)	1.00751	1.013407	1.0195506
283420	CLEC9A	C-type lectin domain family 9, member A	0.99332	0.993907	1.0072236
283422	C12orf36	chromosome 12 open reading frame 36	1.01035	0.999654	1.0081584
283431	GAS2L3	growth arrest-specific 2 like 3	0.96537	0.935055	0.9980824
283440	LOC283440	similar to hCG2045487	1.01378	1.029412	0.9522426

283446	MYO1H	myosin IH	0.99909	0.998682	0.9903094
283450	C12orf51	chromosome 12 open reading frame 51	0.97526	0.964112	0.9988638
283455	KSR2	kinase suppressor of ras 2	1.00652	1.001367	0.9931552
283459	GATC	glutamyl-tRNA(Gln) amidotransferase, subunit C homolog (bacterial)	0.97339	0.976433	1.0753236
283460	HNF1A-AS1	HNF1A antisense RNA 1 (non-protein coding)	0.97494	0.983927	1.0390718
283461	C12orf40	chromosome 12 open reading frame 40	0.99408	1.023374	1.0080717
283463	MUC19	mucin 19, oligomeric	1.00621	0.997666	1.009206
283464	GXYLT1	glucoside xylosyltransferase 1	0.93533	1.012308	1.0618247
283471	TMPRSS12	transmembrane (C-terminal) protease, serine 12	1.00563	0.971938	1.0296752
283481	LOC283481	hypothetical LOC283481	0.98325	1.005871	1.0275177
283487	C13orf29	chromosome 13 open reading frame 29	1.0005	1.035013	0.9774344
283489	ZNF828	zinc finger protein 828	0.92325	0.96491	1.1306224
283491	OR7E156P	olfactory receptor, family 7, subfamily E, member 156 pseudogene	1.02095	0.998067	1.0175193
283507	SUGT1P3	suppressor of G2 allele of SKP1 (S. cerevisiae) pseudogene 3	0.95699	0.95908	0.9412116
283514	SIAH3	seven in absentia homolog 3 (Drosophila)	0.97405	0.989884	0.9919115
283518	KCNRG	potassium channel regulator	0.98821	1.034038	0.94015
283521	NCRNA002	non-protein coding RNA 282	0.97032	0.966108	0.920599
283537	SLC46A3	solute carrier family 46, member 3	0.96811	0.878109	0.9012681
283547	LOC283547	hypothetical LOC283547	1.00408	0.981927	1.0238933
283551	C14orf182	chromosome 14 open reading frame 182	0.98577	0.987046	0.9878758
283553	LOC283553	hypothetical LOC283553	1.00793	0.995493	1.0158962
283554	GPR137C	G protein-coupled receptor 137C	0.99265	1.021781	1.0166086
283562	RPL21P9	ribosomal protein L21 pseudogene 9	1.01194	1.000163	1.020148
283571	PROX2	prospero homeobox 2	1.00245	0.99845	0.9778034
283576	ZDHHC22	zinc finger, DHHC-type containing 22	0.99694	1.014623	1.0166615
283578	TMED8	transmembrane emp24 protein transport domain containing 8	0.97419	0.96618	1.1512786
283579	C14orf178	chromosome 14 open reading frame 178	0.9863	0.987762	0.9789837
283584	[No Symbo	[No Name]	0.98962	1.015876	1.0163643
283585	LOC283585	hypothetical LOC283585	0.99014	1.010493	0.9688363
283588	LOC283588	hypothetical LOC283588	0.98278	0.952199	0.8916596
283596	SNHG10	small nucleolar RNA host gene 10 (non-protein coding)	0.93353	0.976866	1.0653951
283598	C14orf177	chromosome 14 open reading frame 177	0.99155	1.017709	0.9951049
283600	SLC25A47	solute carrier family 25, member 47	0.97129	0.958523	0.973005

283617	OR4K3	olfactory receptor, family 4, subfamily K, member 3 (gene/pseudogene)	1.04067	1.063265	0.9456625
283629	TSSK4	testis-specific serine kinase 4	1.01209	1.029065	0.988607
283631	BTF3P2	basic transcription factor 3, pseudogene 2	0.9861	0.983535	1.0899157
283635	FAM177A1	family with sequence similarity 177, member A1	0.979	1.05061	1.0044213
283638	KIAA0284	KIAA0284	0.99884	1.000354	1.009188
283643	C14orf80	chromosome 14 open reading frame 80	1.01205	0.995791	0.9922771
283651	HMGN2P46	high mobility group nucleosomal binding domain 2 pseudogene 46	1.00812	1.018341	0.9873901
283652	SLC24A5	solute carrier family 24, member 5	1.00087	1.00348	1.0039372
283659	PRTG	protogenin	0.99212	1.000546	0.9891403
283677	C15orf60	chromosome 15 open reading frame 60	1.00137	1.002564	1.0070989
283683	LOC283683	hypothetical LOC283683	0.97532	0.94454	0.8943312
283685	GOLGA6L2	golgin A6 family-like 2	0.98644	1.020962	0.9961629
283687	C15orf37	chromosome 15 open reading frame 37	0.99435	1.024742	1.0247133
283688	LOC283688	hypothetical LOC283688	0.9973	0.994767	1.0381035
283710	LOC283710	hypothetical protein LOC283710	0.96725	1.027884	1.0313126
283711	LOC283711	ubiquitin-conjugating enzyme E2C pseudogene	1.01498	1.034869	1.0032489
283726	FAM154B	family with sequence similarity 154, member B	1.00898	1.01758	0.9640116
283742	FAM98B	family with sequence similarity 98, member B	0.96933	0.962918	1.0368357
283747	TCEB1P2	transcription elongation factor B (SIII), polypeptide 1 pseudogene 2	0.98233	0.980253	1.0067892
283755	HERC2P3	hect domain and RLD 2 pseudogene 3	1.01929	1.019868	0.9187726
283777	FAM169B	family with sequence similarity 169, member B	1.01554	1.00713	0.9862452
283807	FBXL22	F-box and leucine-rich repeat protein 22	0.99191	0.982955	1.0364622
283847	CCDC79	coiled-coil domain containing 79	0.99403	0.988293	0.9996338
283848	CES4A	carboxylesterase 4A	0.9934	0.973176	1.0635174
283849	EXOC3L1	exocyst complex component 3-like 1	0.99932	1.02761	1.0404286
283860	NCRNA003	non-protein coding RNA 304	1.00838	0.975634	0.9981541
283867	LOC283867	hypothetical LOC283867	0.99759	1.008913	1.0012308
283869	NPW	neuropeptide W	0.98824	1.039746	1.0250342
283870	C16orf79	chromosome 16 open reading frame 79	1.00425	0.980212	1.0457108
283871	PGP	phosphoglycolate phosphatase	0.96082	0.981959	1.0209593
283876	FLJ39639	hypothetical protein FLJ39639	0.99332	0.882338	0.9012101
283897	C16orf54	chromosome 16 open reading frame 54	0.93201	0.958854	0.9466549
283899	INO80E	INO80 complex subunit E	0.96647	0.988779	1.0435165

283902	HTA	hypothetical LOC283902	1.00775	1.02345	1.0359188
283904	[No Symbo	[No Name]	0.99586	0.993106	1.0565905
283922	LOC283922	pyruvate dehydrogenase phosphatase regulatory subunit pseudogene	0.97284	0.99381	1.0391695
283927	NUDT7	nudix (nucleoside diphosphate linked moiety X)-type motif 7	0.9711	1.015158	1.0175391
283932	FBXL19-AS1	FBXL19 antisense RNA 1 (non-protein coding)	0.98618	1.011487	1.0255691
283933	ZNF843	zinc finger protein 843	0.99744	0.994168	1.0341068
283948	NHLRC4	NHL repeat containing 4	0.98256	0.935028	0.9770418
283951	C16orf91	chromosome 16 open reading frame 91	0.96253	1.018793	1.0058658
283953	TMEM114	transmembrane protein 114	0.98954	0.969358	0.9465863
283970	PDXDC2P	pyridoxal-dependent decarboxylase domain containing 2, pseudogene	1.00356	1.080162	0.8078254
283985	FADS6	fatty acid desaturase domain family, member 6	0.99865	1.006815	0.9876286
283987	C17orf28	chromosome 17 open reading frame 28	0.97891	0.967029	1.0070755
283989	TSEN54	tRNA splicing endonuclease 54 homolog (S. cerevisiae)	0.93547	1.056845	0.9836633
283991	FAM100B	family with sequence similarity 100, member B	0.9509	1.028161	1.058158
283999	TMEM235	transmembrane protein 235	0.99345	0.996472	1.0169448
284001	CCDC57	coiled-coil domain containing 57	0.98261	1.015541	0.9948095
284004	HEXDC	hexosaminidase (glycosyl hydrolase family 20, catalytic domain) containing	0.93678	0.923814	1.0262891
284009	LOC284009	hypothetical LOC284009	1.01802	0.943122	0.8899511
284013	VMO1	vitelline membrane outer layer 1 homolog (chicken)	1.0044	1.040573	0.990486
284018	C17orf58	chromosome 17 open reading frame 58	0.9982	0.941246	1.0395175
284021	C17orf60	chromosome 17 open reading frame 60	0.95405	0.998607	1.0048464
284023	LOC284023	hypothetical LOC284023	0.95221	0.993155	1.0596873
284040	CDRT4	CMT1A duplicated region transcript 4	0.98775	0.972075	1.0052203
284058	KIAA1267	KIAA1267	0.95955	1.020832	0.987079
284067	C17orf105	chromosome 17 open reading frame 105	0.97001	0.986263	1.0324179
284069	FAM171A2	family with sequence similarity 171, member A2	1.01319	0.987656	1.0335501
284071	C17orf104	chromosome 17 open reading frame 104	1.00159	1.00686	0.9770536
284076	TTLL6	tubulin tyrosine ligase-like family, member 6	1.00421	1.001617	0.9875935
284083	C17orf47	chromosome 17 open reading frame 47	1.0172	1.057428	0.9700396
284085	FLJ40504	keratin 18 pseudogene	0.99733	0.960947	1.0527995
284086	NEK8	NIMA (never in mitosis gene a)- related kinase 8	0.97085	0.999733	0.9945692
284098	PIGW	phosphatidylinositol glycan anchor biosynthesis, class W	0.9275	0.940928	1.0664152
284099	C17orf78	chromosome 17 open reading frame 78	0.98661	0.972471	1.0220694

284100	LOC284100	tyrosine 3-monooxygenase/tryptophan 5-monooxygenase activation protein, epsilon polypeptide pseudogen	0.99035	1.01643	0.9902351
284106	CISD3	CDGSH iron sulfur domain 3	0.9623	1.044992	1.0060747
284110	GSDMA	gasdermin A	1.01511	0.970967	0.9640431
284111	SLC13A5	solute carrier family 13 (sodium-dependent citrate transporter), member 5	0.99087	1.027565	1.0179979
284114	TMEM102	transmembrane protein 102	0.93627	0.942773	1.1265221
284116	KRT42P	keratin 42 pseudogene	0.98259	0.984514	0.9971033
284119	PTRF	polymerase I and transcript release factor	1.00805	0.970551	1.044581
284120	[No Symbo	[No Name]	1.01756	1.030479	0.9830074
284123	FAM27L	family with sequence similarity 27-like	1.015	1.016411	0.9755975
284129	SLC26A11	solute carrier family 26, member 11	0.98667	0.985659	0.9688717
284131	ENDOV	endonuclease V	0.98132	1.016081	1.010555
284161	GDPD1	glycerophosphodiester phosphodiesterase domain containing 1	0.98392	0.975406	0.9687514
284167	POLRMTP1	polymerase (RNA) mitochondrial (DNA directed) pseudogene 1	0.98335	0.99239	1.0384344
284184	C17orf89	chromosome 17 open reading frame 89	0.95239	0.966518	1.0715299
284185	C17orf55	chromosome 17 open reading frame 55	0.99901	0.987857	1.0194583
284186	TMEM105	transmembrane protein 105	0.99969	1.019057	1.0101354
284207	METRNL	meteorin, glial cell differentiation regulator-like	1.01202	1.056241	0.9651496
284215	LOC284215	hypothetical LOC284215	0.99452	0.994616	1.0148222
284217	LAMA1	laminin, alpha 1	0.99991	1.005541	1.0051202
284232	ANKRD20A	ankyrin repeat domain 20 family, member A9, pseudogene	0.99275	1.050658	1.033556
284252	KCTD1	potassium channel tetramerisation domain containing 1	1.00045	1.004853	1.018329
284254	C18orf26	chromosome 18 open reading frame 26	1.00237	0.960222	1.0526986
284257	BOD1P	biorientation of chromosomes in cell division 1 pseudogene	0.98167	1.097263	1.1092517
284260	LOC284260	hypothetical LOC284260	0.9979	0.996078	1.002703
284269	LOC284269	chromosome 9 open reading frame 86 pseudogene	0.99913	0.995541	0.9890316
284273	ZADH2	zinc binding alcohol dehydrogenase domain containing 2	0.98943	0.959852	1.0567886
284274	C18orf62	chromosome 18 open reading frame 62	0.99106	0.969671	1.0170432
284293	HMSD	histocompatibility (minor) serpin domain containing	0.98055	0.993468	1.0131459
284296	[No Symbo	[No Name]	1.02081	0.991004	0.9873948
284297	SSC5D	scavenger receptor cysteine rich domain containing (5 domains)	0.98405	0.996288	1.0318846
284306	ZNF547	zinc finger protein 547	0.98533	0.965778	1.0268501
284307	ZIK1	zinc finger protein interacting with K protein 1 homolog (mouse)	0.92387	0.963267	1.003927
284309	ZNF776	zinc finger protein 776	0.90215	0.958758	1.0070402

284312	ZSCAN1	zinc finger and SCAN domain containing 1	0.99947	0.980117	1.0091653
284313	[No Symbo	[No Name]	1.01214	1.013461	1.0329724
284323	ZNF780A	zinc finger protein 780A	0.9415	1.09826	1.1101929
284325	C19orf54	chromosome 19 open reading frame 54	0.99868	0.963723	0.9934953
284338	PRR19	proline rich 19	1.00808	1.005172	1.0019881
284339	TMEM145	transmembrane protein 145	0.99138	1.02947	1.0692356
284340	CXCL17	chemokine (C-X-C motif) ligand 17	1.00114	0.988746	1.015767
284346	ZNF575	zinc finger protein 575	0.97946	1.011779	1.0823244
284348	LYPD5	LY6/PLAUR domain containing 5	0.98083	1.025233	1.0044305
284349	ZNF283	zinc finger protein 283	0.93187	0.994	1.0248736
284352	LRRC68	leucine rich repeat containing 68	0.99353	0.985756	1.0200324
284353	NKPD1	NTPase, KAP family P-loop domain containing 1	0.99458	0.979276	1.0432992
284355	TPRX1	tetra-peptide repeat homeobox 1	1.02104	0.92839	0.9904477
284358	MAMSTR	MEF2 activating motif and SAP domain containing transcriptional regulator	1.00635	1.059544	1.0988596
284359	IZUMO1	izumo sperm-egg fusion 1	0.98848	1.010363	1.0315751
284361	C19orf63	chromosome 19 open reading frame 63	0.929	0.941369	1.028
284365	MGC45922	hypothetical LOC284365	1.03015	1.058876	1.105687
284366	KLK9	kallikrein-related peptidase 9	1.00425	1.027071	1.0055516
284367	SIGLECP3	sialic acid binding Ig-like lectin, pseudogene 3	1.02308	0.942621	0.9749478
284369	C19orf75	chromosome 19 open reading frame 75	0.99244	1.040215	0.9716882
284370	ZNF615	zinc finger protein 615	0.96208	0.92296	1.0753874
284371	ZNF841	zinc finger protein 841	0.97883	0.992337	1.0591331
284379	LOC284379	solute carrier family 7 (cationic amino acid transporter, y+ system), member 3 pseudogene	1.00541	1.052968	0.9832811
284382	ACTL9	actin-like 9	0.979	1.013353	1.0499397
284383	OR2Z1	olfactory receptor, family 2, subfamily Z, member 1	1.01697	0.934026	0.8991826
284390	ZNF763	zinc finger protein 763	0.94189	0.985627	0.9547965
284391	ZNF844	zinc finger protein 844	0.91894	0.936938	0.9443083
284395	LOC284395	hypothetical LOC284395	0.98703	0.999191	1.0014717
284402	SCGBL	secretoglobin-like	0.97091	1.008605	1.0277117
284403	WDR62	WD repeat domain 62	0.9984	0.975216	1.0158304
284406	ZFP82	zinc finger protein 82 homolog (mouse)	0.92907	1.013618	1.0424552
284415	VSTM1	V-set and transmembrane domain containing 1	0.97557	0.726576	1.0609685
284417	TMEM150B	transmembrane protein 150B	0.99449	0.947157	0.9563107

284418	FAM71E2	family with sequence similarity 71, member E2	0.98073	1.003268	1.0189792
284422	C19orf77	chromosome 19 open reading frame 77	1.00559	1.025385	1.0149345
284424	NCRNA003	non-protein coding RNA 306	1.00759	0.981085	0.9870294
284427	SLC25A41	solute carrier family 25, member 41	1.00121	1.000089	1.0078841
284434	NWD1	NACHT and WD repeat domain containing 1	0.99045	0.983581	1.0038293
284439	SLC25A42	solute carrier family 25, member 42	0.99457	1.010027	1.0003965
284440	LOC284440	hypothetical LOC284440	0.96313	1.0452	0.9206951
284441	LOC284441	actin-related protein 2 pseudogene	1.01975	0.953571	1.0671397
284443	ZNF493	zinc finger protein 493	0.91094	0.986584	0.9630471
284451	ODF3L2	outer dense fiber of sperm tails 3-like 2	1.00944	1.022476	1.0332739
284459	HKR1	HKR1, GLI-Kruppel zinc finger family member	0.97295	1.019343	0.9904264
284467	FAM19A3	family with sequence similarity 19 (chemokine (C-C motif)-like), member A3	1.02067	1.029367	1.0188832
284485	RIIAD1	regulatory subunit of type II PKA R-subunit (RIIa) domain containing 1	1.01336	0.974437	1.021975
284486	THEM5	thioesterase superfamily member 5	1.02114	0.99654	0.966683
284498	C1orf167	chromosome 1 open reading frame 167	0.99969	1.022635	1.0165275
284521	OR2L13	olfactory receptor, family 2, subfamily L, member 13	1.01169	0.976224	0.9787974
284525	SLC9A11	solute carrier family 9, member 11	1.00531	0.998309	1.0082053
284532	OR14A16	olfactory receptor, family 14, subfamily A, member 16	1.03228	1.029181	1.0482867
284541	CYP4A22	cytochrome P450, family 4, subfamily A, polypeptide 22	1.00358	0.99519	1.0572821
284546	C1orf185	chromosome 1 open reading frame 185	1.02347	1.022478	1.0646849
284573	NCRNA003	non-protein coding RNA 303	1.00542	0.999822	1.0162392
284577	LOC284577	hypothetical LOC284577	0.99569	1.037573	1.0160973
284611	FAM102B	family with sequence similarity 102, member B	1.02279	1.014216	1.0529572
284612	SYPL2	synaptophysin-like 2	1.02902	1.030894	1.0108954
284613	CYB561D1	cytochrome b-561 domain containing 1	0.96862	0.982962	1.0922215
284615	ANKRD34A	ankyrin repeat domain 34A	1.0116	1.008415	1.0005191
284618	RUSC1-AS1	RUSC1 antisense RNA 1 (non-protein coding)	0.99055	0.968947	1.0254161
284648	LOC284648	hypothetical LOC284648	1.00965	1.031078	1.0139693
284654	RSPO1	R-spondin homolog (Xenopus laevis)	1.00363	1.02389	0.9915944
284656	EPHA10	EPH receptor A10	0.99422	1.019722	1.0280975
284672	PTGES3P	prostaglandin E synthase 3 (cytosolic) pseudogene	0.98793	1.180943	0.9542097
284680	C1orf111	chromosome 1 open reading frame 111	1.00378	1.000455	1.0316158
284685	LOC284685	Ewing sarcoma breakpoint region 1 pseudogene	1.00248	0.988913	1.0146604

284688	LOC284688	hypothetical LOC284688	1.03348	1.005227	1.0109372
284695	ZNF326	zinc finger protein 326	1.0486	0.884953	0.9247741
284697	BTBD8	BTB (POZ) domain containing 8	0.99155	0.954549	0.9437412
284716	RIMKLA	ribosomal modification protein rimK-like family member A	1	0.985965	0.9928766
284723	SLC25A34	solute carrier family 25, member 34	0.99782	0.971121	0.960144
284729	ESPNP	espin pseudogene	1.0161	0.983284	1.0503401
284739	NCRNA001	non-protein coding RNA 176	0.9876	0.991105	1.0174019
284756	C20orf197	chromosome 20 open reading frame 197	0.97661	0.987743	1.0661454
284759	SIRPB2	signal-regulatory protein beta 2	0.98721	0.90724	1.0481095
284764	FTLP3	ferritin, light polypeptide pseudogene 3	0.99888	1.021597	1.0383437
284800	FAM182A	family with sequence similarity 182, member A	1.0535	1.128131	0.9260104
284801	LOC284801	hypothetical protein LOC284801	0.96646	1.0605	0.9616602
284805	C20orf203	chromosome 20 open reading frame 203	1.01171	1.042521	0.9230443
284821	RPL13AP7	ribosomal protein L13a pseudogene 7	0.95603	0.974125	1.0201954
284827	KRTAP13-4	keratin associated protein 13-4	0.98803	1.010071	0.9663317
284836	NCRNA003	non-protein coding RNA 319	1.02195	1.010463	1.0174455
284837	LOC284837	hypothetical LOC284837	1.00227	0.941089	1.0001106
284904	SEC14L4	SEC14-like 4 (S. cerevisiae)	0.99453	0.998995	1.016614
284912	LOC284912	hypothetical LOC284912	0.97874	0.985998	0.9946716
284942	RPL23AP82	ribosomal protein L23a pseudogene 82	1.01551	1.052628	0.9785043
284944	[No Symbo	[No Name]	0.99802	0.98786	1.0003632
284948	SH2D6	SH2 domain containing 6	0.99433	1.019127	1.0148441
284952	LOC284952	hypothetical protein LOC284952	0.98144	1.046375	1.0311252
284958	NT5DC4	5'-nucleotidase domain containing 4	1.00614	0.988305	0.9991003
284992	CCDC150	coiled-coil domain containing 150	1.0095	0.981604	1.0258709
284996	RNF149	ring finger protein 149	1.05615	1.131821	0.9383534
284998	LOC284998	hypothetical LOC284998	1.01433	1.021216	1.023821
285016	FAM150B	family with sequence similarity 150, member B	1.00569	1.003997	1.003485
285025	CCDC141	coiled-coil domain containing 141	0.9354	0.910785	0.9843519
285033	LOC285033	hypothetical protein LOC285033	1.00573	0.996628	1.0537527
285051	C2orf61	chromosome 2 open reading frame 61	0.98703	1.01665	1.0097729
285093	C2orf85	chromosome 2 open reading frame 85	1.00712	0.988125	1.0316302
285095	LOC285095	hypothetical protein LOC285095	1.02281	1.001117	1.0698096

285097	FLJ38379	hypothetical FLJ38379	1.02034	0.95936	1.004112
285116	AHCTF1P1	AT hook containing transcription factor 1 pseudogene 1	0.98177	0.953173	0.9505969
285126	DNAJC5G	DnaJ (Hsp40) homolog, subfamily C, member 5 gamma	1.00141	0.990484	0.9901052
285141	LOC285141	hypothetical protein LOC285141	0.99792	0.976195	1.0113333
285148	IAH1	isoamyl acetate-hydrolyzing esterase 1 homolog (S. cerevisiae)	0.9714	0.981776	1.0238017
285150	FLJ33534	hypothetical LOC285150	0.97976	1.040694	1.0288894
285154	CYP1B1-AS	CYP1B1 antisense RNA 1 (non-protein coding)	1.02149	1.024	1.0430149
285172	FAM126B	family with sequence similarity 126, member B	0.97952	1.034534	0.9780018
285175	UNC80	unc-80 homolog (C. elegans)	1.0138	1.016691	1.0315516
285180	RUFY4	RUN and FYVE domain containing 4	1.04282	0.984757	1.0130841
285193	DUSP28	dual specificity phosphatase 28	0.99784	1.000988	0.979857
285194	LSAMP-AS1	LSAMP antisense RNA 1 (non-protein coding)	1.0197	0.995367	1.0035387
285195	SLC9A9	solute carrier family 9 (sodium/hydrogen exchanger), member 9	0.99794	0.948949	0.9925194
285203	C3orf64	chromosome 3 open reading frame 64	0.99681	0.938129	1.0130341
285205	LOC285205	hypothetical LOC285205	1.0775	0.977808	1.0002787
285216	[No Symbo	[No Name]	1.02022	0.99488	1.0326964
285220	EPHA6	EPH receptor A6	1.01085	1.004145	1.0040111
285226	[No Symbo	[No Name]	0.98072	0.991589	1.0151147
285231	FBXW12	F-box and WD repeat domain containing 12	1.02981	0.972401	1.0079676
285237	C3orf38	chromosome 3 open reading frame 38	0.97633	0.883626	1.059538
285242	HTR3E	5-hydroxytryptamine (serotonin) receptor 3, family member E	1.01333	0.998501	0.997919
285264	[No Symbo	[No Name]	1.03344	1.011635	0.9988071
285267	ZNF619	zinc finger protein 619	0.97849	0.974015	1.0332852
285268	ZNF621	zinc finger protein 621	0.98725	1.054854	0.9880823
285282	RABL3	RAB, member of RAS oncogene family-like 3	0.97424	0.907904	1.0424632
285303	LOC285303	family with sequence similarity 151, member A pseudogene	0.99746	0.977418	1.0267404
285311	C3orf56	chromosome 3 open reading frame 56	0.99573	1.004117	1.0248258
285313	IGSF10	immunoglobulin superfamily, member 10	1.00782	1.014025	1.0223523
285315	C3orf33	chromosome 3 open reading frame 33	0.9903	0.943049	1.0006287
285322	[No Symbo	[No Name]	0.98539	1.034759	1.0302571
285331	CCDC66	coiled-coil domain containing 66	0.97361	0.946719	0.9598811
285335	SLC9A10	solute carrier family 9, member 10	0.99461	1.001308	1.0043397
285343	C3orf23	chromosome 3 open reading frame 23	0.97604	0.947766	1.0153758

285346	ZNF852	zinc finger protein 852	1.02113	0.983852	1.1029805
285349	ZNF660	zinc finger protein 660	1.02573	1.039861	1.0237615
285359	LOC285359	phosducin-like 3 pseudogene	0.9464	0.95955	0.987055
285362	SUMF1	sulfatase modifying factor 1	0.98791	0.970229	0.9750423
285367	RPUSD3	RNA pseudouridylate synthase domain containing 3	0.99242	0.994891	1.0297384
285368	PRRT3	proline-rich transmembrane protein 3	0.98643	0.993552	1.0053532
285381	DPH3	DPH3, KTI11 homolog (S. cerevisiae)	0.9555	0.987185	0.9939716
285382	C3orf70	chromosome 3 open reading frame 70	1.01456	1.01342	1.0204775
285386	TPRG1	tumor protein p63 regulated 1	1.01726	1.025073	1.0160403
285412	LOC285412	TOX high mobility group box family member 4 pseudogene	0.98612	1.001042	1.0625223
285415	MRPL42P1	mitochondrial ribosomal protein L42 pseudogene 1	0.97197	0.941181	0.9835773
285423	LOC285423	hypothetical LOC285423	1.01572	0.98979	1.0267012
285429	DCAF4L1	DDB1 and CUL4 associated factor 4-like 1	0.99557	1.066773	1.0554983
285440	CYP4V2	cytochrome P450, family 4, subfamily V, polypeptide 2	0.99964	1.025546	0.970198
285453	LOC285453	TXNDC9 pseudogene	0.9784	1.014968	0.9643627
285456	LOC285456	hypothetical LOC285456	1.0053	0.992221	1.0022535
285464	CRIPAK	cysteine-rich PAK1 inhibitor	1.02122	1.026972	0.9941355
285484	LOC285484	hypothetical LOC285484	0.97969	1.015643	0.9822522
285489	DOK7	docking protein 7	1.00715	1.001656	1.039541
285492	FLJ35424	hypothetical FLJ35424	1.01357	0.989931	1.0543385
285498	RNF212	ring finger protein 212	0.98897	0.992782	1.0090651
285501	LOC285501	hypothetical LOC285501	1.00893	0.980183	1.0109538
285512	FAM13AOS	FAM13A opposite strand (non-protein coding)	1.05315	1.001097	0.8750246
285513	GPRIN3	GPRIN family member 3	1.00711	1.038242	1.0039652
285521	COX18	COX18 cytochrome c oxidase assembly homolog (S. cerevisiae)	0.99192	1.029114	1.0039166
285525	YIPF7	Yip1 domain family, member 7	1.01266	0.981694	0.9852513
285527	FRYL	FRY-like	0.97857	0.908334	1.024965
285533	RNF175	ring finger protein 175	0.98928	0.966918	0.9615969
285548	LOC285548	hypothetical LOC285548	0.99522	0.972648	1.0066855
285550	FAM200B	family with sequence similarity 200, member B	0.98966	0.940765	0.9467382
285555	C4orf37	chromosome 4 open reading frame 37	1.00845	1.001389	1.0016735
285556	LOC285556	hypothetical protein LOC285556	0.99551	0.99329	1.007659
285588	EFCAB9	EF-hand calcium binding domain 9	1.00903	0.995457	1.0447076

285590	SH3PXD2B	SH3 and PX domains 2B	1.00191	1.002966	1.0025864
285591	LOC285591	inorganic pyrophosphatase pseudogene	0.98919	0.974554	1.0905029
285598	ARL10	ADP-ribosylation factor-like 10	1.01456	0.975528	1.032671
285600	KIAA0825	KIAA0825	1.0022	1.012536	1.0170884
285601	GPR150	G protein-coupled receptor 150	1.00287	0.980022	1.1149366
285605	DTWD2	DTW domain containing 2	0.98215	0.971323	0.9481112
285613	RELL2	RELT-like 2	1.00124	1.017704	1.0106647
285634	LOC285634	integrator complex subunit 6 pseudogene	1.0013	1.010945	1.048114
285636	C5orf51	chromosome 5 open reading frame 51	0.95637	0.888048	0.9121
285641	SLC36A3	solute carrier family 36 (proton/amino acid symporter), member 3	1.00401	1	1.0082674
285643	KIF4B	kinesin family member 4B	1.01699	1.000226	1.0177447
285647	LOC285647	suppressor of defective silencing 3 pseudogene	1.01199	1.03665	1.0590414
285668	C5orf64	chromosome 5 open reading frame 64	1.00916	0.990977	0.9953309
285671	RNF180	ring finger protein 180	0.98062	1.033898	0.9893001
285672	SREK1IP1	SREK1-interacting protein 1	1.00511	0.926496	1.0755393
285676	ZNF454	zinc finger protein 454	1.00703	1.004613	1.00801
285679	C5orf60	chromosome 5 open reading frame 60	1.00878	1.010363	1.0115436
285696	LOC285696	hypothetical LOC285696	1.00838	1.016264	0.9876904
285704	RGMB	RGM domain family, member B	0.97611	0.907318	0.9861255
285706	LOC285706	cytokine receptor-like factor 3 pseudogene	0.97452	0.982737	0.9438219
285733	LOC285733	hypothetical LOC285733	1.02135	0.974516	1.0242532
285735	NCRNA003	non-protein coding RNA 326	1.01128	1.000617	1.018146
285741	LOC285741	tumor protein, translationally-controlled 1 pseudogene	0.96373	1.058815	0.947704
285753	CEP57L1	centrosomal protein 57kDa-like 1	1.00381	0.985437	1.0106952
285754	FLJ37396	hypothetical protein FLJ37396	0.99154	1.010358	1.0085364
285755	PPIL6	peptidylprolyl isomerase (cyclophilin)-like 6	0.99434	0.98918	0.9877418
285759	FLJ34503	hypothetical FLJ34503	1.00906	1.001493	1.0106425
285761	DCBLD1	discoidin, CUB and LCCL domain containing 1	0.98841	1.026743	1.0362519
285782	CAGE1	cancer antigen 1	1.00616	0.989233	1.0169538
285800	PRR18	proline rich 18	0.97707	1.05564	1.0798148
285804	LOC285804	hypothetical LOC285804	1.01164	0.991831	1.0134417
285829	SUMO2P1	SMT3 suppressor of mif two 3 homolog 2 (S. cerevisiae) pseudogene 1	0.97276	0.960319	0.9764496
285830	HLA-F-AS1	HLA-F antisense RNA 1 (non-protein coding)	1.02531	0.978443	1.0173128

285834	HCG22	HLA complex group 22	1.00094	0.970168	0.9913902
285848	PNPLA1	patatin-like phospholipase domain containing 1	0.99122	1.010501	0.9821143
285852	TREML4	triggering receptor expressed on myeloid cells-like 4	1.02071	0.996072	1.0717899
285855	RPL7L1	ribosomal protein L7-like 1	1.01468	0.990343	0.9409013
285872	LOC285872	THAP5 pseudogene	0.966	1.087033	1.0361986
285877	POM121L1	POM121 membrane glycoprotein-like 12	0.97749	0.979323	0.9781984
285888	CNPY1	canopy 1 homolog (zebrafish)	1.00157	1.016436	1.0185185
285900	RPL6P20	ribosomal protein L6 pseudogene 20	0.98331	0.964475	1.0061705
285908	NCRNA001	non-protein coding RNA 174	1.01108	1.025436	1.026456
285941	C7orf71	chromosome 7 open reading frame 71	0.99846	1.002227	0.9817268
285943	LOC285943	hypothetical LOC285943	0.99097	1.007952	1.0039446
285958	C7orf40	chromosome 7 open reading frame 40	0.98219	1.035817	1.0327017
285961	SEPT7L	septin 7-like	0.96288	0.98135	1.0459997
285971	ZNF775	zinc finger protein 775	1.00931	1.009823	1.0587025
285973	ATG9B	ATG9 autophagy related 9 homolog B (S. cerevisiae)	0.9873	1.011344	1.0167996
285987	DLX6-AS1	DLX6 antisense RNA 1 (non-protein coding)	1.02918	0.994851	1.0458884
285989	ZNF789	zinc finger protein 789	1.00515	1.014668	1.0100931
286002	LOC286002	hypothetical LOC286002	0.99969	1.022185	1.0168918
286006	C7orf53	chromosome 7 open reading frame 53	0.99873	0.9947	0.9758236
286016	TPI1P2	triosephosphate isomerase 1 pseudogene 2	0.98617	1.003268	0.9757435
286023	FLJ40288	hypothetical FLJ40288	1.00837	1.006822	1.017753
286042	FLJ10661	family with sequence similarity 86, member A pseudogene	1.01777	1.037782	1.0464227
286046	XKR6	XK, Kell blood group complex subunit-related family, member 6	0.99671	0.987552	0.9873746
286053	NSMCE2	non-SMC element 2, MMS21 homolog (S. cerevisiae)	0.98789	0.996432	0.9458713
286065	MAPK6PS4	mitogen-activated protein kinase 6 pseudogene 4	0.99196	0.990454	1.0073728
286075	ZNF707	zinc finger protein 707	1.00251	1.006909	1.0321549
286076	BREA2	breast cancer estrogen-induced apoptosis 2	1.0075	1.047424	1.0506948
286077	FAM83H	family with sequence similarity 83, member H	0.98108	0.991462	1.0480876
286094	LOC286094	hypothetical LOC286094	1.02312	1.000371	1.0101729
286097	EFHA2	EF-hand domain family, member A2	0.98238	0.983389	0.9658843
286101	ZNF252	zinc finger protein 252	0.91793	0.96747	0.9792322
286102	TMED10P1	transmembrane emp24-like trafficking protein 10 (yeast) pseudogene 1	0.97193	0.967712	0.9777415
286103	C8orf77	chromosome 8 open reading frame 77	1.01002	1.055639	1.0216569

286122	C8orf31	chromosome 8 open reading frame 31	1.01862	1.012568	1.014518
286128	ZFP41	zinc finger protein 41 homolog (mouse)	0.9995	0.982592	1.0180011
286133	SCARA5	scavenger receptor class A, member 5 (putative)	0.99412	1.008962	0.9984484
286140	RNF5P1	ring finger protein 5 pseudogene 1	0.97144	1.018333	1.0570042
286144	C8orf83	chromosome 8 open reading frame 83	0.96778	0.94022	1.0245024
286148	DPY19L4	dpy-19-like 4 (C. elegans)	0.99075	0.962109	0.9959745
286150	RPS2P33	ribosomal protein S2 pseudogene 33	0.97218	0.954302	1.0396438
286151	FBXO43	F-box protein 43	0.984	0.977511	1.020862
286157	PCBP2P2	poly(rC) binding protein 2 pseudogene 2	0.94323	0.988332	1.0229777
286177	LOC286177	hypothetical LOC286177	0.98892	0.985209	1.024578
286183	NKAIN3	Na+/K+ transporting ATPase interacting 3	1.00627	1.009796	1.0181887
286187	LRRC67	leucine rich repeat containing 67	0.97452	0.995118	0.997147
286204	CRB2	crumbs homolog 2 (Drosophila)	0.99451	0.996252	1.0259949
286205	SCAI	suppressor of cancer cell invasion	0.93169	0.929617	1.0420135
286207	C9orf117	chromosome 9 open reading frame 117	0.99173	1.009276	0.9560814
286223	C9orf47	chromosome 9 open reading frame 47	0.95827	0.952172	1.1256538
286234	C9orf79	chromosome 9 open reading frame 79	0.99553	1.021456	1.0078132
286238	LOC286238	hypothetical protein LOC286238	1.01455	0.998232	1.0060158
286239	LOC286239	malic enzyme 2, NAD(+)-dependent, mitochondrial pseudogene	0.98015	0.969154	1.0457406
286256	LCN12	lipocalin 12	0.99307	1.002795	1.0107749
286257	C9orf142	chromosome 9 open reading frame 142	0.94406	1.077394	1.0800667
286262	TPRN	taperin	0.97477	0.9932	1.043222
286319	TUSC1	tumor suppressor candidate 1	0.99256	0.959789	1.0889517
286336	FAM78A	family with sequence similarity 78, member A	0.96983	0.936302	0.8809452
286343	C9orf150	chromosome 9 open reading frame 150	0.98917	0.981216	0.9843108
286348	RPL7P33	ribosomal protein L7 pseudogene 33	0.95489	0.957047	1.018978
286359	LOC286359	hypothetical LOC286359	1.01372	1.022347	0.9965664
286362	OR13C9	olfactory receptor, family 13, subfamily C, member 9	0.98216	1.01406	0.9881597
286410	ATP11C	ATPase, class VI, type 11C	0.91326	0.944227	1.0620302
286411	RP1-177G6	hypothetical LOC286411	1.00393	0.965717	0.9943024
286423	MRRFP1	mitochondrial ribosome recycling factor pseudogene 1	0.99714	1.031846	0.9988949
286430	NLRP2P	NLR family, pyrin domain containing 2 pseudogene	0.99322	0.97637	1.0284343
286436	H2BFM	H2B histone family, member M	0.97557	0.982961	1.0552089

286451	YIPF6	Yip1 domain family, member 6	0.95216	1.010114	1.0988906
286453	dJ341D10.1	chondroitin beta1,4 N-acetylgalactosaminyltransferase (ChGn) pseudogene	0.97394	1.063247	0.9760197
286456	LOC286456	NGFI-A binding protein 1 (EGR1 binding protein 1) pseudogene	0.99597	1.013105	1.069204
286464	CXorf59	chromosome X open reading frame 59	1.00418	0.984969	0.9910322
286467	LOC286467	hypothetical LOC286467	0.97034	0.981318	1.0377752
286472	RAC1P4	ras-related C3 botulinum toxin substrate 1 pseudogene 4	1.02914	1.040543	1.0582827
286480	UBE2E4P	ubiquitin-conjugating enzyme E2E 4 pseudogene	0.95622	0.997262	0.9731218
286494	BRAFP1	v-raf murine sarcoma viral oncogene homolog B1 pseudogene 1	1.00663	0.961146	1.0076086
286495	TTC3P1	tetratricopeptide repeat domain 3 pseudogene 1	0.95908	0.934344	1.0324234
286499	FAM133A	family with sequence similarity 133, member A	1.0054	1.019804	1.0142618
286512	LOC286512	eukaryotic translation initiation factor 4A2 pseudogene	1.00776	0.981606	1.0404778
286514	MAGEB18	melanoma antigen family B, 18	0.9984	1.022912	1.0150894
286544	MXRA5P1	matrix-remodelling associated 5 pseudogene 1	1.01112	1.001223	1.024051
286550	CYCSP46	cytochrome c, somatic pseudogene 46	0.99503	0.962923	0.935738
286554	BCORP1	BCL6 corepressor pseudogene 1	0.99499	0.96246	1.0010788
286555	TBL1YP1	transducin (beta)-like 1, Y-linked pseudogene 1	1.01933	0.99239	1.0052671
286676	ILDR1	immunoglobulin-like domain containing receptor 1	1.00459	1.039	1.0263082
286749	STON1-GTF	STON1-GTF2A1L readthrough	1.01821	0.98438	0.972738
286753	TUSC5	tumor suppressor candidate 5	0.99732	0.98098	1.0154518
286826	LIN9	lin-9 homolog (C. elegans)	0.98217	0.973472	1.026111
286827	TRIM59	tripartite motif containing 59	0.93883	0.834412	1.1134891
286828	CSN1S2AP	casein alpha s2-like A, pseudogene	1.00865	1.045527	1.0427755
287015	TRIM42	tripartite motif containing 42	1.00759	0.993438	1.0093707
317649	EIF4E3	eukaryotic translation initiation factor 4E family member 3	1.00981	0.968577	0.9836835
317662	FAM149B1	family with sequence similarity 149, member B1	0.98095	0.967027	1.0418138
317671	RFESD	Rieske (Fe-S) domain containing	0.98969	0.983415	0.9975315
317684	MAPK6PS3	mitogen-activated protein kinase 6 pseudogene 3	0.99335	1.007008	1.036639
317686	MAPK6PS1	mitogen-activated protein kinase 6 pseudogene 1	1.00767	0.98282	1.0345201
317701	VN1R2	vomeronasal 1 receptor 2	1.01931	1.041354	1.0086603
317702	VN1R3	vomeronasal 1 receptor 3 (gene/pseudogene)	1.03863	1.018553	0.9949037
317705	VN1R5	vomeronasal 1 receptor 5 (gene/pseudogene)	1.01108	1.042598	1.0403812
317716	BASE	breast cancer and salivary gland expression gene	0.99513	0.986592	1.011866
317719	KLHL10	kelch-like 10 (Drosophila)	0.99581	0.954854	1.0107546

317727	LOC317727	ataxin 2 related protein pseudogene	0.99646	0.980755	0.9946207
317729	BLZF2P	basic leucine zipper nuclear factor 2 pseudogene	0.99723	1.06101	0.9808696
317730	C14orf99	chromosome 14 open reading frame 99	0.99515	1.044079	1.0320948
317753	PSMD12P	proteasome 26S subunit, non-ATPase, 12 pseudogene	0.98361	1.025093	0.9972208
317760	C14orf55	metalloproteinase-disintegrin 1-2 pseudogene	0.96204	0.995114	1.010403
317761	C14orf39	chromosome 14 open reading frame 39	0.98352	1.018047	0.9878732
317762	CCDC85C	coiled-coil domain containing 85C	0.99045	0.983025	1.0088856
317772	HIST2H2AB	histone cluster 2, H2ab	0.93609	0.907935	1.0635342
317774	SEPT7P1	septin 7 pseudogene 1	0.96903	1.013934	1.0346968
317775	CHORDC2P	cysteine and histidine-rich domain (CHORD) containing 2 pseudogene	0.98416	0.995138	0.9981976
317781	DDX51	DEAD (Asp-Glu-Ala-Asp) box polypeptide 51	0.98395	0.991512	1.0569693
317784	COILP1	coilin pseudogene 1	0.95716	1.048194	1.0685171
319085	ITPK1-AS1	ITPK1 antisense RNA 1 (non-protein coding)	1.00769	1.030114	0.97424
319087	COX5AP2	cytochrome c oxidase subunit Va pseudogene 2	0.97464	1.045024	1.033181
319099	DDX18P1	DEAD (Asp-Glu-Ala-Asp) box polypeptide 18 pseudogene 1	1.01479	1.008871	0.9819198
319101	KRT73	keratin 73	0.94994	1.032021	0.9244104
319102	ARHGAP16	Rho GTPase activating protein 16 pseudogene	0.99275	0.995432	1.0479051
319111	DNAJC8P1	DnaJ (Hsp40) homolog, subfamily C, member 8 pseudogene 1	0.99067	0.979852	1.0845771
319112	EEF1A1P2	eukaryotic translation elongation factor 1 alpha 1 pseudogene 2	1.03632	0.985085	1.0226981
319115	EIF2S2P1	eukaryotic translation initiation factor 2, subunit 2 beta pseudogene 1	0.97378	0.9446	1.014513
319117	EIF3LP1	eukaryotic translation initiation factor 3, subunit L pseudogene 1	0.98589	0.961199	1.0469097
319118	EIF4BP1	eukaryotic translation initiation factor 4B pseudogene 1	0.97713	1.007206	1.0270564
319120	YWHAZP1	tyrosine 3-monooxygenase/tryptophan 5-monooxygenase activation protein, zeta pseudogene 1	1.01367	1.028265	0.9911094
319121	YWHAQP1	YWHAQ pseudogene 1	1.00883	0.98963	1.0248691
319122	UNGP3	uracil-DNA glycosylase pseudogene 3	0.99523	1.025489	1.0505702
319123	ZFP64P1	zinc finger protein 64 homolog (mouse) pseudogene 1	1.00669	0.969019	0.9780936
319128	PPIAP6	peptidylprolyl isomerase A (cyclophilin A) pseudogene 6	0.92831	0.985443	1.0069995
319129	RPSAP5	ribosomal protein SA pseudogene 5	0.95067	0.97948	1.0365105
319130	HMGB1P14	high mobility group box 1 pseudogene 14	1.01413	1.033907	1.0061093
319134	HNRPUP	heterogeneous nuclear ribonucleoprotein U pseudogene	0.98936	1.030937	1.1462089
319135	RPL10AP1	ribosomal protein L10a pseudogene 1	0.98967	1.030319	0.9965504
319140	GCATP1	glycine C-acetyltransferase pseudogene 1	0.96576	1.011029	0.9595761
319142	RPL12P5	ribosomal protein L12 pseudogene 5	1.01278	1.005852	0.9785056

319144	RPL21P13	ribosomal protein L21 pseudogene 13	0.97008	0.876311	0.9937401
319145	RPL21P8	ribosomal protein L21 pseudogene 8	0.98125	1.009991	1.0403848
326105	UBE2CP1	ubiquitin-conjugating enzyme E2C pseudogene 1	0.9974	1.021383	0.9744439
326106	TUBBP3	tubulin, beta pseudogene 3	0.99012	1.005416	1.036772
326107	STK16P1	serine/threonine kinase 16 pseudogene 1	1.01152	0.998623	1.0288375
326268	ABI1P1	abl-interactor 1 pseudogene 1	0.99202	1.043275	1.0046333
326269	SRMP2	spermidine synthase pseudogene 2	1.0123	1.001606	0.9769492
326272	SNRPGP1	small nuclear ribonucleoprotein polypeptide G pseudogene 1	1.03594	0.995754	0.9613892
326275	RPL12P7	ribosomal protein L12 pseudogene 7	1.00338	1.044511	1.0126112
326277	SETP2	SET pseudogene 2	0.96834	0.955118	1.0335901
326279	RPL15P2	ribosomal protein L15 pseudogene 2	0.97327	0.972558	1.0106728
326286	RPL17P3	ribosomal protein L17 pseudogene 3	1.00386	0.973236	0.9829853
326288	RPL17P4	ribosomal protein L17 pseudogene 4	1.04546	0.996066	1.0458053
326289	RPL18AP1	ribosomal protein L18a pseudogene 1	0.92041	0.917596	1.0244862
326291	RPL18P1	ribosomal protein L18 pseudogene 1	0.99604	1.011008	0.9849902
326293	RPL23AP10	ribosomal protein L23a pseudogene 10	0.98622	1.045186	1.0069766
326294	RPL23AP11	ribosomal protein L23a pseudogene 11	0.99942	1.010276	1.0402868
326297	RPL26P2	ribosomal protein L26 pseudogene 2	1.0014	1.035926	1.0108053
326298	RPL26P3	ribosomal protein L26 pseudogene 3	0.99444	1.027217	1.0337504
326299	RPL27P1	ribosomal protein L27 pseudogene 1	0.9817	0.98091	1.0483108
326301	RPL36AP2	ribosomal protein L36a pseudogene 2	0.99073	0.989324	1.0203827
326303	RPL36P3	ribosomal protein L36 pseudogene 3	0.96702	0.928577	1.1009678
326305	MOCS3P1	molybdenum cofactor synthesis 3 pseudogene 1	0.98315	0.998207	1.0117075
326306	RPL39P2	ribosomal protein L39 pseudogene 2	1.00932	0.968916	1.050312
326308	RPL41P4	ribosomal protein L41 pseudogene 4	0.95833	0.920936	1.0550901
326309	RPL7AP5	ribosomal protein L7a pseudogene 5	1.00223	1.000639	1.001889
326310	RPL7AP6	ribosomal protein L7a pseudogene 6	0.92074	0.944937	1.0388372
326311	RPL9P6	ribosomal protein L9 pseudogene 6	1.024	1.014532	0.9996832
326312	RPS12P1	ribosomal protein S12 pseudogene 1	0.97093	0.98966	1.0600465
326317	RPS15AP3	ribosomal protein S15a pseudogene 3	0.97955	0.962951	1.0182756
326320	RPS18P2	ribosomal protein S18 pseudogene 2	0.95843	1.07501	1.1170244
326321	RPS24P2	ribosomal protein S24 pseudogene 2	1.01792	0.992035	1.0440006
326323	RPS27AP4	ribosomal protein S27a pseudogene 4	1.00219	1.011201	0.9759544

326325	RPS2P2	ribosomal protein S2 pseudogene 2	0.96975	1.04823	1.04399
326326	RPS2P3	ribosomal protein S2 pseudogene 3	1.00109	1.009218	0.9978843
326337	SSXP4	SSX family pseudogene 4	1.03296	1.035021	1.0071813
326340	ZAR1	zygote arrest 1	1.01139	0.982046	1.0297086
326342	EMR4P	egf-like module containing, mucin-like, hormone receptor-like 4 pseudogene	1.03065	0.839193	0.941798
326343	MT1DP	metallothionein 1D, pseudogene	1.00077	1.010246	1.0324929
326590	SSXP7	SSX family pseudogene 7	1.01841	1.002919	1.0231947
326593	MORF4L1P	mortality factor 4 like 1 pseudogene 3	0.97121	0.999916	0.958828
326603	MTCO1P2	MT-CO1 pseudogene 2	1.01087	0.912212	1.0813161
326605	NCOA4P1	nuclear receptor coactivator 4 pseudogene 1	1.01163	0.972873	1.0081187
326606	NDUFB8P1	NADH dehydrogenase (ubiquinone) 1 beta subcomplex, 8, pseudogene 1	0.95887	1.051685	1.0368295
326608	NPM1P20	nucleophosmin 1 (nucleolar phosphoprotein B23, numatrin) pseudogene 20	1.01192	1.053916	0.958064
326613	NUTF2P2	nuclear transport factor 2 pseudogene 2	0.94913	0.959711	1.0405419
326614	PEBP1P1	phosphatidylethanolamine binding protein 1 pseudogene 1	0.98147	0.980684	0.9922167
326616	PDLIM1P	PDZ and LIM domain 1 pseudogene	0.97264	0.970598	1.0742621
326617	PSMA3P	proteasome (prosome, macropain) subunit, alpha type, 3 pseudogene	0.96359	1.0045	1.0739524
326624	RAB37	RAB37, member RAS oncogene family	0.96096	0.903756	1.02027
326625	MMAB	methylmalonic aciduria (cobalamin deficiency) cblB type	0.9704	0.984464	1.0156961
326626	PTMAP7	prothymosin, alpha pseudogene 7	0.95422	0.976788	1.0332374
326627	RANBP20P	RAN binding protein 20 pseudogene	0.99138	0.974357	0.970785
326628	RPA2P	replication protein A2 pseudogene	1.00215	0.95125	0.9809393
327657	SERPINA9	serpin peptidase inhibitor, clade A (alpha-1 antiproteinase, antitrypsin), member 9	1.00703	0.989804	1.0031368
327659	HDHD1P2	haloacid dehalogenase-like hydrolase domain containing 1 pseudogene 2	1.01845	1.00327	1.0612171
333926	PPM1J	protein phosphatase, Mg2+/Mn2+ dependent, 1J	0.99722	1.016736	0.9815492
333929	SNAI3	snail homolog 3 (Drosophila)	0.99619	1.026838	0.9489982
337867	UBAC2	UBA domain containing 2	0.98912	1.06163	0.979265
337872	HIST3H2BA	histone cluster 3, H2ba, pseudogene	1.024	0.991438	0.9899914
337873	HIST2H2BC	histone cluster 2, H2bc	1.01002	0.915793	0.8761747
337874	HIST2H2BD	histone cluster 2, H2bd (pseudogene)	1.04295	0.873497	1.0007087
337876	CHSY3	chondroitin sulfate synthase 3	1.01275	1.012294	1.0259884
337878	KRTAP7-1	keratin associated protein 7-1 (gene/pseudogene)	0.97405	1.015553	1.0208306
337879	KRTAP8-1	keratin associated protein 8-1	1.01038	1.107037	1.0447177
337880	KRTAP11-1	keratin associated protein 11-1	1.00061	0.971672	1.0279479

337882	KRTAP19-1	keratin associated protein 19-1	1.0174	1.020198	1.0409611
337959	KRTAP13-2	keratin associated protein 13-2	0.9961	1.023449	1.0401047
337963	KRTAP23-1	keratin associated protein 23-1	0.98524	1.002282	0.9685806
337964	KRTAP13-5	keratin associated protein 13-5, pseudogene	1.00417	0.986103	0.9966479
337966	KRTAP6-1	keratin associated protein 6-1	0.99121	0.961817	0.9661543
337967	KRTAP6-2	keratin associated protein 6-2	0.98292	0.974463	0.9885303
337968	KRTAP6-3	keratin associated protein 6-3	1.06696	0.981686	0.9888997
337971	KRTAP19-4	keratin associated protein 19-4	0.96822	1.057867	1.0040549
337972	KRTAP19-5	keratin associated protein 19-5	0.99938	0.933544	0.9465121
337973	KRTAP19-6	keratin associated protein 19-6	0.99732	0.990902	1.0443447
337976	KRTAP20-2	keratin associated protein 20-2	1.02977	1.017511	1.0132372
337978	KRTAP21-2	keratin associated protein 21-2	1.0453	0.970428	1.0679809
337979	KRTAP22-1	keratin associated protein 22-1	1.00675	1.070075	1.0120876
337985	KRTAP20-3	keratin associated protein 20-3	1.01178	1.019145	0.9263475
338017	KRT8P1	keratin 8 pseudogene 1	1.0115	1.000972	0.9962789
338094	FAM151A	family with sequence similarity 151, member A	1.02732	1.051154	1.009293
338096	PSME2P4	proteasome activator subunit 2 pseudogene 4	1.0193	0.995582	0.9173431
338097	PSME2P5	proteasome activator subunit 2 pseudogene 5	0.98222	0.984496	1.0506796
338098	PSME2P6	proteasome activator subunit 2 pseudogene 6	1.00705	0.96318	1.0367428
338099	PSME2P2	proteasome activator subunit 2 pseudogene 2	0.96529	1.009014	1.1511868
338321	NLRP9	NLR family, pyrin domain containing 9	0.98567	1.032018	1.0086425
338322	NLRP10	NLR family, pyrin domain containing 10	0.98071	1.022116	1.0133056
338323	NLRP14	NLR family, pyrin domain containing 14	0.99359	1.0015	1.0311006
338324	S100A7A	S100 calcium binding protein A7A	1.03363	0.977988	0.9955359
338328	GPIHBP1	glycosylphosphatidylinositol anchored high density lipoprotein binding protein 1	0.99579	1.013007	0.9996724
338339	CLEC4D	C-type lectin domain family 4, member D	0.9739	1.055993	1.1020965
338376	IFNE	interferon, epsilon	0.97765	1.004866	0.9964803
338389	MRPS24P1	mitochondrial ribosomal protein S24 pseudogene 1	0.96723	0.935553	1.0376356
338390	MRPS25P1	mitochondrial ribosomal protein S25 pseudogene 1	0.98787	0.894166	0.9110848
338396	TAS2R2	taste receptor, type 2, member 2 pseudogene	1.00517	0.951734	1.0144373
338398	TAS2R60	taste receptor, type 2, member 60	1.00163	1.090154	0.914218
338440	ANO9	anoctamin 9	0.95739	0.987865	1.0351078
338470	MESTP1	mesoderm specific transcript homolog (mouse) pseudogene 1	1.00194	1	1.0177037

338557	O3FAR1	omega-3 fatty acid receptor 1	1.00972	0.972133	0.9802905
338567	KCNK18	potassium channel, subfamily K, member 18	0.96798	1.045076	0.9908202
338588	LOC338588	hypothetical LOC338588	0.98236	0.99638	1.0399499
338591	LOC338591	coiled-coil-helix-coiled-coil-helix domain containing 3 pseudogene	1.00546	1.063075	0.9935693
338596	ST8SIA6	ST8 alpha-N-acetyl-neuraminide alpha-2,8-sialyltransferase 6	0.97212	0.971138	1.0099307
338599	DUPD1	dual specificity phosphatase and pro isomerase domain containing 1	0.99929	0.982729	1.0187308
338611	RPS26P40	ribosomal protein S26 pseudogene 40	1.01776	1.022599	0.9738852
338637	OR52M2P	olfactory receptor, family 52, subfamily M, member 2 pseudogene	0.99027	0.979808	1.0543501
338645	LUZP2	leucine zipper protein 2	1	1.007527	1.0143339
338657	CCDC84	coiled-coil domain containing 84	1.00006	1.020099	0.9206783
338661	TMEM225	transmembrane protein 225	1.0068	1.00935	0.9978741
338662	OR8D4	olfactory receptor, family 8, subfamily D, member 4	1.00719	1.041307	0.9820674
338667	LOC338667	hypothetical protein LOC338667	0.97509	1.002099	1.0103456
338674	OR5F1	olfactory receptor, family 5, subfamily F, member 1	0.99963	0.965243	0.9610763
338675	OR5AP2	olfactory receptor, family 5, subfamily AP, member 2	1.00563	0.980961	1.0001253
338692	ANKRD13D	ankyrin repeat domain 13 family, member D	0.98152	1.011577	1.0139868
338699	ANKRD42	ankyrin repeat domain 42	0.9434	0.99076	0.9892402
338707	B4GALNT4	beta-1,4-N-acetyl-galactosaminyl transferase 4	1.00893	0.990118	1.0992701
338739	LOC338739	hypothetical LOC338739	0.9877	0.985727	1.049789
338751	OR52L1	olfactory receptor, family 52, subfamily L, member 1	1.03988	1.043163	0.9962443
338756	[No Symbo	[No Name]	0.98893	1.03369	0.9764097
338758	LOC338758	hypothetical LOC338758	0.97368	1.091501	0.9705761
338761	C1QL4	complement component 1, q subcomponent-like 4	1.00512	1.013054	1.0240047
338773	TMEM119	transmembrane protein 119	0.99913	1.005669	1.0299548
338785	KRT79	keratin 79	1.00803	0.988377	0.9945951
338799	LOC338799	hypothetical LOC338799	1.00238	0.97516	0.8773864
338805	ST13P8	suppression of tumorigenicity 13 (colon carcinoma) (Hsp70 interacting protein) pseudogene 8	1.00081	0.987045	1.0033022
338809	C12orf74	chromosome 12 open reading frame 74	0.98646	0.993406	1.0235512
338811	FAM19A2	family with sequence similarity 19 (chemokine (C-C motif)-like), member A2	0.98884	1.016283	0.9957324
338821	LST-3TM12	organic anion transporter LST-3b	1.00298	0.98832	1.0219962
338870	RPS12P23	ribosomal protein S12 pseudogene 23	0.98239	1.007645	1.0264463
338872	C1QTNF9	C1q and tumor necrosis factor related protein 9	0.98985	1.030188	0.9935228
338879	RNASE10	ribonuclease, RNase A family, 10 (non-active)	0.99387	1.009134	1.0120549

338917	VSX2	visual system homeobox 2	0.96921	1.021115	1.0436103
338949	TMEM202	transmembrane protein 202	0.98963	1.010956	0.9922321
338963	LOC338963	epididymal protein pseudogene	1.00032	1.013673	0.987992
339059	FLJ40448	hypothetical protein FLJ40448	0.98594	0.933442	1.056935
339105	PRSS53	protease, serine, 53	0.98713	1.052646	0.9853403
339122	RAB43	RAB43, member RAS oncogene family	1.03438	0.988941	0.9320618
339123	JMJD8	jumonji domain containing 8	0.93628	0.883076	1.0820477
339145	FAM92B	family with sequence similarity 92, member B	1.01021	1.064604	0.9814798
339168	TMEM95	transmembrane protein 95	0.99575	0.999643	0.9561966
339175	METTL2A	methyltransferase like 2A	1.00343	0.890887	1.0550502
339192	LOC339192	hypothetical LOC339192	0.94897	1.064372	0.9310691
339201	C17orf65	chromosome 17 open reading frame 65	0.99097	0.975969	0.9742328
339209	[No Symbo	[No Name]	0.99619	0.978914	0.9951177
339210	C17orf67	chromosome 17 open reading frame 67	0.97514	0.998452	0.9839336
339221	ENPP7	ectonucleotide pyrophosphatase/phosphodiesterase 7	0.98319	0.95303	1.0270752
339229	C17orf90	chromosome 17 open reading frame 90	0.94006	0.982278	1.0494888
339230	CCDC137	coiled-coil domain containing 137	0.99523	1.027637	1.0669118
339231	ARL16	ADP-ribosylation factor-like 16	0.97811	1.010141	1.0700204
339263	C17orf51	chromosome 17 open reading frame 51	0.97144	1.013368	0.986871
339287	MSL1	male-specific lethal 1 homolog (Drosophila)	0.95458	1.005252	1.0025138
339290	LOC339290	hypothetical LOC339290	0.88484	0.982952	0.9623994
339302	CPLX4	complexin 4	0.98957	1.023256	1.0397968
339318	ZNF181	zinc finger protein 181	0.91836	0.96095	0.9923542
339324	ZNF260	zinc finger protein 260	0.90233	1.096734	1.0164474
339327	ZNF546	zinc finger protein 546	0.93588	1.063679	1.0500813
339344	MYPOP	Myb-related transcription factor, partner of profilin	0.97119	1.029183	1.0027954
339345	NANOS2	nanos homolog 2 (Drosophila)	1.00628	1.052157	0.9977977
339352	LOC339352	putative ATP-binding domain-containing protein 3-like protein-like	1.00331	1.01156	1.0477202
339366	ADAMTSL5	ADAMTS-like 5	0.98537	0.974813	1.0125891
339390	CLEC4G	C-type lectin domain family 4, member G	0.98418	1.002918	1.0106092
339396	NEFHP1	neurofilament, heavy polypeptide pseudogene 1	0.99808	1.006609	1.01058
339398	LINGO4	leucine rich repeat and Ig domain containing 4	1.00772	0.990618	1.0022116
339403	RXFP4	relaxin/insulin-like family peptide receptor 4	1.00046	1.006573	1.0035443

339416	ANKRD45	ankyrin repeat domain 45	0.99253	1.000462	1.0062557
339448	C1orf174	chromosome 1 open reading frame 174	0.98386	0.942187	1.0757267
339451	KLHL17	kelch-like 17 (Drosophila)	0.97243	1.028031	1.0941272
339453	C1orf70	chromosome 1 open reading frame 70	1.00827	1.017859	1.0447531
339456	TMEM52	transmembrane protein 52	1.00007	1.083733	1.0677278
339457	C1orf222	chromosome 1 open reading frame 222	1.0067	1.007978	0.9933818
339479	FAM5C	family with sequence similarity 5, member C	0.99973	1.001672	1.0036064
339483	MTMR9LP	myotubularin related protein 9-like, pseudogene	0.9991	0.996508	0.9967772
339487	ZBTB8OS	zinc finger and BTB domain containing 8 opposite strand	0.98852	0.965476	1.0275243
339488	TFAP2E	transcription factor AP-2 epsilon (activating enhancer binding protein 2 epsilon)	1.00512	1.016103	0.9889724
339500	ZNF678	zinc finger protein 678	0.97138	0.966771	0.9774344
339501	PRSS38	protease, serine, 38	1.01087	0.992025	1.045765
339512	C1orf110	chromosome 1 open reading frame 110	1.01723	0.973278	1.0167815
339524	LOC339524	hypothetical LOC339524	0.99163	0.989823	1.0393549
339541	C1orf228	chromosome 1 open reading frame 228	0.99189	0.991731	1.0129998
339559	ZNF642	zinc finger protein 642	0.97745	0.967636	0.9951428
339562	IGKV1OR2-	immunoglobulin kappa variable 1/OR2-118 (pseudogene)	0.98676	1.109075	0.9748459
339665	SLC35E4	solute carrier family 35, member E4	0.99526	0.939738	0.9798794
339669	C22orf33	chromosome 22 open reading frame 33	0.98477	0.978052	0.9748888
339674	BK250D10.	hypothetical LOC339674	0.97191	1.077293	1.0433036
339736	AK2P2	adenylate kinase 2 pseudogene 2	0.9548	0.951831	1.0303929
339745	SPOPL	speckle-type POZ protein-like	1.02955	0.991614	1.0590093
339760	LOC339760	hypothetical protein LOC339760	1.0087	0.998119	0.9896054
339761	CYP27C1	cytochrome P450, family 27, subfamily C, polypeptide 1	1.00143	1.012123	1.0310895
339766	HEATR7B1	HEAT repeat containing 7B1	1.00175	1.0074	1.0167948
339768	ESPNL	espin-like	1.00742	1.017339	1.0132056
339778	C2orf70	chromosome 2 open reading frame 70	0.98506	1.001916	1.0074214
339779	C2orf53	chromosome 2 open reading frame 53	1.0073	0.968219	1.05012
339781	KRT18P19	keratin 18 pseudogene 19	1.01083	1.004515	0.9762832
339789	NCRNA002	non-protein coding RNA 299	0.99825	0.978925	1.0198024
339799	EIF3FP3	eukaryotic translation initiation factor 3, subunit F pseudogene 3	0.9746	1.025787	1.0359276
339804	C2orf74	chromosome 2 open reading frame 74	0.96963	0.972531	1.001614
339829	CCDC39	coiled-coil domain containing 39	1.00549	0.986346	1.02428

339834	CCDC36	coiled-coil domain containing 36	1.01142	1.018231	0.9928481
339843	LOC339843	WW domain containing E3 ubiquitin protein ligase 1 pseudogene	0.99419	0.930461	0.9770627
339845	ASS1P7	argininosuccinate synthetase 1 pseudogene 7	1.01483	0.948478	0.9861943
339855	KY	kyphoscoliosis peptidase	1.02014	1.002044	1.0148855
339879	LOC339879	asparagine-linked glycosylation 1 homolog pseudogene	1.01551	1.01424	1.0190127
339881	EIF4BP8	eukaryotic translation initiation factor 4B pseudogene 8	1.01354	0.977231	1.0308048
339883	C3orf35	chromosome 3 open reading frame 35	1.01012	1.042183	0.9416224
339896	GADL1	glutamate decarboxylase-like 1	1.01871	1.018586	1.0065299
339897	CNN2P6	calponin 2 pseudogene 6	0.98005	1.017446	1.0481966
339902	LOC339902	hCG1813818	1.02687	1.03942	0.9459928
339906	PRSS42	protease, serine, 42	1.00205	1.012286	1.0430959
339942	C3orf47	chromosome 3 open reading frame 47	1.04402	0.987227	1.0117501
339965	CCDC158	coiled-coil domain containing 158	0.99383	1.000091	1.005465
339966	LOC339966	ubiquitin domain-containing protein UBFD1-like	0.9765	0.943928	1.0483703
339967	TMPRSS11A	transmembrane protease, serine 11A	1.01259	1.019827	1.0084517
339976	TRIML1	tripartite motif family-like 1	0.99819	0.977037	1.0307397
339977	LRRC66	leucine rich repeat containing 66	1.01396	0.966984	1.0493904
339983	NAT8L	N-acetyltransferase 8-like (GCN5-related, putative)	1.05147	1.027597	1.0717743
340024	SLC6A19	solute carrier family 6 (neutral amino acid transporter), member 19	1.0062	1.00075	0.9882787
340061	TMEM173	transmembrane protein 173	1.00327	0.901682	0.992686
340069	FAM170A	family with sequence similarity 170, member A	1.00545	0.983114	0.9904832
340075	ARSI	arylsulfatase family, member I	0.98178	1.014353	0.9449554
340094	LOC340094	hypothetical LOC340094	1.01664	0.989427	0.9963423
340096	H3.X	histone cluster 2, H3d pseudogene	1.00016	1.028822	0.9970736
340113	LOC340113	hypothetical LOC340113	0.98887	0.990488	1.0073677
340120	ANKRD34B	ankyrin repeat domain 34B	0.98722	1.019417	1.0391762
340146	SLC35D3	solute carrier family 35, member D3	0.96545	0.938114	0.9924846
340152	ZC3H12D	zinc finger CCCH-type containing 12D	0.99278	1.016672	1.0363151
340156	MYLK4	myosin light chain kinase family, member 4	1.00427	1.008982	1.0141153
340168	DPPA5	developmental pluripotency associated 5	0.99433	1.049617	1.0373676
340192	IQCB2P	IQ motif containing B2 pseudogene	1.01634	0.981949	1.0185598
340204	C6orf127	chromosome 6 open reading frame 127	0.99565	0.954943	1.048241
340205	TREML1	triggering receptor expressed on myeloid cells-like 1	0.9502	0.933952	1.0209325

340206	TREML3	triggering receptor expressed on myeloid cells-like 3	0.99595	1.006122	0.921129
340252	ZNF680	zinc finger protein 680	0.97279	0.959878	1.0000799
340260	UNCX	UNC homeobox	1.01269	1.022448	1.0670157
340267	COL28A1	collagen, type XXVIII, alpha 1	1.00383	0.988886	1.0037972
340268	LOC340268	C3 and PZP-like, alpha-2-macroglobulin domain containing 8 pseudogene	0.99495	1.010008	1.0183741
340273	ABCB5	ATP-binding cassette, sub-family B (MDR/TAP), member 5	1.00453	1.002409	1.0282039
340274	ASS1P11	argininosuccinate synthetase 1 pseudogene 11	1.01965	0.995645	1.0584706
340277	C7orf46	chromosome 7 open reading frame 46	1.00018	0.963924	0.9993689
340286	FAM183B	acyloxyacyl hydrolase (neutrophil)	0.99072	0.949637	0.9583025
340348	TSPAN33	tetraspanin 33	0.95425	1.022602	1.0528136
340351	AGBL3	ATP/GTP binding protein-like 3	0.988	0.99566	0.9951799
340359	KLHL38	kelch-like 38 (Drosophila)	0.99927	0.966247	1.0368412
340371	NRBP2	nuclear receptor binding protein 2	0.97122	0.976789	1.0029819
340385	ZNF517	zinc finger protein 517	0.9702	0.990759	1.02165
340390	KIAA1875	KIAA1875	1.01186	1.008993	1.0151522
340419	RSPO2	R-spondin 2 homolog (Xenopus laevis)	0.9967	1.011563	0.9948173
340441	POTEA	POTE ankyrin domain family, member A	0.99344	0.992984	1.0390715
340443	RPS5P5	ribosomal protein S5 pseudogene 5	0.98551	0.969657	1.0918425
340460	KRT18P24	keratin 18 pseudogene 24	0.99735	0.979504	0.9858541
340481	ZDHHC21	zinc finger, DHHC-type containing 21	0.94725	0.942559	0.9872027
340485	ACER2	alkaline ceramidase 2	0.98518	0.986587	0.985631
340499	IMPDH1P1	IMP (inosine monophosphate) dehydrogenase 1 pseudogene 1	0.98807	0.970078	1.0394765
340501	LOC340501	transcription elongation factor A (SII), 1 pseudogene	1.00522	0.993446	1.0508326
340502	[No Symbo	[No Name]	0.99386	1.011933	1.0007537
340515	LOC340515	hypothetical LOC340515	1.0052	0.95254	0.9816522
340526	RGAG4	retrotransposon gag domain containing 4	0.99084	0.975703	0.9774922
340527	NHSL2	NHS-like 2	0.90996	0.879961	0.9358703
340533	KIAA2022	KIAA2022	1.00289	0.974767	1.0068117
340542	BEX5	brain expressed, X-linked 5	0.94394	0.982444	0.890596
340543	TCEAL5	transcription elongation factor A (SII)-like 5	1.00876	1.006593	1.0346304
340547	VSIG1	V-set and immunoglobulin domain containing 1	0.88429	0.923814	0.8689903
340554	ZC3H12B	zinc finger CCCH-type containing 12B	0.9715	0.929671	0.9035211
340571	SIAH1P1	seven in absentia homolog 1 (Drosophila) pseudogene 1	0.99302	1.032931	1.0735114

340578	DCAF12L2	DDB1 and CUL4 associated factor 12-like 2	0.98848	0.952178	1.0437565
340591	CA5BP1	carbonic anhydrase VB pseudogene 1	0.96812	1.024025	0.963822
340595	ZCCHC16	zinc finger, CCHC domain containing 16	1.00416	0.982989	0.9754041
340596	LHFPL1	lipoma HMGIC fusion partner-like 1	0.98848	1.020352	1.0270074
340598	KRT18P48	keratin 18 pseudogene 48	0.98469	0.993414	1.0134103
340602	CXorf67	chromosome X open reading frame 67	0.94003	0.978659	0.9820754
340654	LIPM	lipase, family member M	0.99556	1.015325	0.999816
340665	CYP26C1	cytochrome P450, family 26, subfamily C, polypeptide 1	1.00246	1.051356	1.0384533
340706	VWA2	von Willebrand factor A domain containing 2	1.00805	1.010686	1.0145931
340719	NANOS1	nanos homolog 1 (Drosophila)	0.97982	0.967289	1.0514929
340745	LRIT2	leucine-rich repeat, immunoglobulin-like and transmembrane domains 2	0.98523	0.995714	1.0323781
340749	RPL7AP8	ribosomal protein L7a pseudogene 8	0.96406	1.00407	1.0090308
340780	IMPDH1P5	IMP (inosine monophosphate) dehydrogenase 1 pseudogene 5	1.00023	0.99063	1.0258086
340784	HMX3	H6 family homeobox 3	0.98231	1.053443	1.1139699
340811	AKR1CL1	aldo-keto reductase family 1, member C-like 1	0.99361	0.990917	1.0338173
340844	LOC340844	adenosylhomocysteinase pseudogene	0.99523	1.01196	1.0169171
340888	LOC340888	aldo-keto reductase family 1, member B10 (aldose reductase) pseudogene	0.96784	1.022606	1.0729595
340895	C10orf112	chromosome 10 open reading frame 112	1.01531	1.009162	0.9903256
340947	LOC340947	eukaryotic translation initiation factor 3, subunit L pseudogene	1.00524	0.974374	0.9688908
340980	OR52B6	olfactory receptor, family 52, subfamily B, member 6	1.02553	0.983747	0.9928703
340990	OTOG	otogelin	0.99537	0.985586	1.001745
341019	DCDC1	doublecortin domain containing 1	0.99496	0.980811	1.032125
341032	C11orf53	chromosome 11 open reading frame 53	0.99539	0.991295	0.9989815
341056	LOC341056	SUMO-1 activating enzyme subunit 1 pseudogene	0.9659	1.081673	1.0474064
341112	LOC341112	tryptophanyl-tRNA synthetase, cytoplasmic-like	1.00558	1.051571	0.9956763
341116	MS4A10	membrane-spanning 4-domains, subfamily A, member 10	1.0276	0.982971	0.9533895
341148	CYCSP27	cytochrome c, somatic pseudogene 27	0.96451	1.083776	1.0722287
341152	OR2AT4	olfactory receptor, family 2, subfamily AT, member 4	0.98294	1.022516	1.0150497
341208	HEPHL1	hephaestin-like 1	0.9974	1.000713	0.9944906
341230	ASS1P13	argininosuccinate synthetase 1 pseudogene 13	0.98887	1.026185	0.9546068
341277	OVCH2	ovochymase 2 (gene/pseudogene)	1.0078	0.97354	1.0479963
341315	PABPC1P4	poly(A) binding protein, cytoplasmic 1 pseudogene 4	0.97401	1.022481	1.008276
341333	LOC341333	heterogeneous nuclear ribonucleoprotein A1 pseudogene	0.93562	0.945916	0.9506546

341346	C12orf70	chromosome 12 open reading frame 70	1.00516	0.98551	0.9812185
341350	OVCH1	ovochymase 1	1.0038	0.99062	1.0001395
341356	RPL31P50	ribosomal protein L31 pseudogene 50	1.00445	1.013836	1.0455698
341359	SYT10	synaptotagmin X	0.9938	1.014566	0.9918151
341378	LOC341378	thyroid hormone receptor interactor 11 pseudogene	1.01918	0.973246	1.0378297
341392	ACSM4	acyl-CoA synthetase medium-chain family member 4	0.99398	0.99371	1.0050013
341405	ANKRD33	ankyrin repeat domain 33	0.9732	0.977283	1.0033294
341412	[No Symbo	[No Name]	0.99083	1.001811	0.956533
341416	OR6C2	olfactory receptor, family 6, subfamily C, member 2	0.9876	0.946235	1.0139158
341418	OR6C4	olfactory receptor, family 6, subfamily C, member 4	1.02027	0.943114	1.0208282
341457	PPIAP8	peptidylprolyl isomerase A (cyclophilin A) pseudogene 8	0.98891	0.906017	1.0378788
341469	MRPS7P2	mitochondrial ribosomal protein S7 pseudogene 2	1.00657	0.973998	0.9910481
341511	RPL23AP14	ribosomal protein L23a pseudogene 14	0.99244	0.993313	1.053045
341567	H1FNT	H1 histone family, member N, testis-specific	1.01051	0.93058	1.0302147
341568	OR8S1	olfactory receptor, family 8, subfamily S, member 1	1.01056	1.000957	0.9928249
341604	RPL35P7	ribosomal protein L35 pseudogene 7	0.97644	0.97783	1.0069868
341640	FREM2	FRAS1 related extracellular matrix protein 2	1.00805	0.987994	0.9793283
341674	RPL5P31	ribosomal protein L5 pseudogene 31	0.98753	0.95441	1.1183027
341676	NEK5	NIMA (never in mitosis gene a)-related kinase 5	0.99471	1.014197	0.9868983
341689	CTAGE16P	CTAGE family, member 16, pseudogene	1.00523	1.016318	0.9990933
341757	MRPS31P2	mitochondrial ribosomal protein S31 pseudogene 2	0.9947	1.012192	1.0855472
341784	EIF4A1P7	eukaryotic translation initiation factor 4A1 pseudogene 7	0.97604	0.997821	1.045309
341799	OR6S1	olfactory receptor, family 6, subfamily S, member 1	0.9924	0.958669	1.0009231
341880	SLC35F4	solute carrier family 35, member F4	1.01648	1.026942	0.9983163
341883	LRRC9	leucine rich repeat containing 9	0.99188	1.001423	1.0190847
341965	LOC341965	voltage-dependent anion channel 3 pseudogene	0.97242	0.952906	1.0387016
342035	GLDN	gliomedin	0.98968	1.006173	1.0247926
342125	TMC3	transmembrane channel-like 3	0.99282	1.013188	0.9889238
342132	ZNF774	zinc finger protein 774	0.98917	1.005125	1.0297155
342184	FMN1	formin 1	0.98695	0.982871	0.9770415
342293	LOC342293	ATP-binding cassette, sub-family A (ABC1), member 3 pseudogene	1.00041	0.993524	1.0104548
342346	LOC342346	hypothetical protein LOC342346	0.98955	1.024742	1.0252604
342357	ZKSCAN2	zinc finger with KRAB and SCAN domains 2	0.97014	0.95068	1.0079392

342372	PKD1L3	polycystic kidney disease 1-like 3	0.99356	1.015222	0.9918737
342374	KRT18P18	keratin 18 pseudogene 18	0.97549	1.01285	1.0065154
342419	KRT8P22	keratin 8 pseudogene 22	0.98936	1.009246	0.9558367
342426	ZNF720P1	zinc finger protein 720 pseudogene 1	1.02266	1.081687	1.0407258
342443	LOC342443	ADAM metallopeptidase domain 21 pseudogene	1.02636	0.993961	1.0378812
342510	CD300E	CD300e molecule	1.01173	1.039489	1.0293
342527	SMTNL2	smoothelin-like 2	1.00015	1.016679	1.0392616
342531	ALOX15P1	arachidonate 15-lipoxygenase pseudogene 1	0.97985	0.96641	0.9625011
342541	LOC342541	peptidylprolyl isomerase A (cyclophilin A) pseudogene	1.00052	0.902806	1.018577
342574	KRT27	keratin 27	0.99873	1.005052	1.0226288
342615	[No Symbo	[No Name]	0.92984	0.979611	0.9017006
342618	SLFN14	schlafen family member 14	0.88197	0.85761	1.0949283
342666	FLJ43826	FLJ43826 protein	0.98441	0.999146	1.0205504
342667	STAC2	SH3 and cysteine rich domain 2	0.98654	0.997091	1.0006711
342705	PGDP1	phosphogluconate dehydrogenase pseudogene 1	1.01066	0.923921	1.0282908
342732	KRT8P5	keratin 8 pseudogene 5	0.99896	1.001871	1.0047908
342776	MRPS17P7	mitochondrial ribosomal protein S17 pseudogene 7	1.0137	1.066181	1.02946
342784	LOC342784	general transcription factor IIH, polypeptide 1, 62kDa pseudogene	0.9846	1.037025	1.028043
342808	RPS2P6	ribosomal protein S2 pseudogene 6	1.00503	0.998487	1.0020781
342850	ANKRD62	ankyrin repeat domain 62	0.99527	1.004191	0.9921751
342865	VSTM2B	V-set and transmembrane domain containing 2B	1.00694	0.989016	1.0348033
342892	ZNF850	zinc finger protein 850	0.96246	0.996278	1.0188209
342897	NCCRP1	non-specific cytotoxic cell receptor protein 1 homolog (zebrafish)	0.9959	1.022612	1.0460789
342898	SYCN	syncollin	1.01078	1.044941	1.0408092
342908	ZNF404	zinc finger protein 404	0.92721	1.037376	0.9771297
342909	ZNF284	zinc finger protein 284	0.95828	0.995532	0.9991659
342918	LOC342918	hypothetical protein LOC342918	0.97892	1.042279	1.0317982
342926	ZNF677	zinc finger protein 677	0.94737	0.980354	0.9680199
342933	ZSCAN5B	zinc finger and SCAN domain containing 5B	1.0088	1.003643	0.9965359
342945	ZSCAN22	zinc finger and SCAN domain containing 22	0.94912	1.013586	1.0192646
342977	NANOS3	nanos homolog 3 (Drosophila)	0.9808	0.996108	1.0653088
342979	PALM3	paralemmin 3	0.97692	0.973791	1.0428971
342994	RPL34P34	ribosomal protein L34 pseudogene 34	0.9812	0.96863	1.0144747

343035	RD3	retinal degeneration 3	1.00513	1.014758	0.9758101
343052	LOC343052	similar to hCG1996527	1.02532	1.02233	1.01588
343066	AADACL4	arylacetamide deacetylase-like 4	0.98702	0.989145	1.024054
343069	HNRNPCL1	heterogeneous nuclear ribonucleoprotein C-like 1	1.01249	1.059499	1.0703834
343099	CCDC18	coiled-coil domain containing 18	1.00008	0.983152	1.0341776
343165	LOC343165	heat shock 70kDa protein 8 pseudogene	0.99728	0.985098	1.0254819
343169	OR6F1	olfactory receptor, family 6, subfamily F, member 1	0.97453	1.009133	1.0343675
343171	OR2W3	olfactory receptor, family 2, subfamily W, member 3	1.01036	0.920687	0.9967351
343184	RPS2P11	ribosomal protein S2 pseudogene 11	0.95424	1.003664	0.9764246
343263	MYBPHL	myosin binding protein H-like	0.99249	1.010703	1.016873
343296	ADH5P2	alcohol dehydrogenase 5 (class III), chi polypeptide, pseudogene 2	1.0093	1.018584	0.9927061
343301	RPL10AP4	ribosomal protein L10a pseudogene 4	0.96221	0.977712	1.0274328
343326	KRT18P28	keratin 18 pseudogene 28	1.02516	1.016912	0.9826441
343332	FDPSP1	farnesyl diphosphate synthase pseudogene 1	0.99368	1.05337	1.0496004
343338	GAPDHP20	glyceraldehyde 3 phosphate dehydrogenase pseudogene 20	0.98697	1.029497	1.0307369
343406	OR10R2	olfactory receptor, family 10, subfamily R, member 2	1.0307	0.961535	1.0284791
343409	OR10J8P	olfactory receptor, family 10, subfamily J, member 8 pseudogene	1.00371	0.968698	1.0291834
343413	FCRL6	Fc receptor-like 6	1.04043	1.037491	0.9311649
343450	KCNT2	potassium channel, subfamily T, member 2	1.0098	1.001595	1.0017044
343472	BARHL2	BarH-like homeobox 2	1.0091	0.986468	1.0029421
343495	RPL6P2	ribosomal protein L6 pseudogene 2	1.01952	1.011551	0.9855813
343505	NBPF7	neuroblastoma breakpoint family, member 7	0.98309	0.991588	0.9872171
343508	LOC343508	aconitate hydratase, mitochondrial-like	1.01393	1.004947	1.042762
343515	ATP6V1E1P	ATPase, H+ transporting, lysosomal 31kDa, V1 subunit E1 pseudogene 1	1.00293	1.004563	1.0094965
343521	TCTEX1D4	Tctex1 domain containing 4	0.998	1.010206	0.9636287
343578	ARHGAP40	Rho GTPase activating protein 40	0.99587	1.000275	1.0283722
343629	[No Symbo	[No Name]	0.99956	0.987263	0.9794874
343637	RSPO4	R-spondin family, member 4	1.0067	0.993712	0.9779849
343641	TGM6	transglutaminase 6	0.99991	0.98491	0.9905585
343702	XKR7	XK, Kell blood group complex subunit-related family, member 7	0.98606	0.992206	1.0063516
343727	CYCSP42	cytochrome c, somatic pseudogene 42	0.99772	0.992164	1.0114084
343930	MSGN1	mesogenin 1	1.01799	1.034454	1.0734037
343981	GLULP6	glutamate-ammonia ligase (glutamine synthetase) pseudogene 6	1.01578	0.969209	0.9982117

343990	C2orf55	chromosome 2 open reading frame 55	1.00969	1.01033	1.0081225
344018	FIGLA	folliculogenesis specific basic helix-loop-helix	0.97346	0.989011	1.0583189
344022	NOTO	notochord homeobox	0.99539	0.982326	0.9826007
344065	LOC344065	zinc finger protein 184 pseudogene	0.97672	1.008505	1.0528681
344148	NCKAP5	NCK-associated protein 5	1.01403	0.994209	1.0106124
344167	FOXI3	forkhead box I3	0.99633	1.003867	1.0065807
344191	EVX2	even-skipped homeobox 2	0.99518	0.990685	0.9936892
344320	KRT8P10	keratin 8 pseudogene 10	0.99493	0.988393	1.0181906
344328	ST13P2	suppression of tumorigenicity 13 (colon carcinoma) (Hsp70 interacting protein) pseudogene 2	0.97653	0.992988	1.0168891
344332	FABP5P10	fatty acid binding protein 5 pseudogene 10	0.98688	1.006753	1.0670869
344371	SLC25A5P2	solute carrier family 25 (mitochondrial carrier; adenine nucleotide translocator), member 5 pseudogene 2	1.00063	1.020826	1.0310622
344382	LOC344382	serine/threonine kinase receptor associated protein pseudogene	0.99937	0.96232	1.0032606
344387	CDKL4	cyclin-dependent kinase-like 4	0.9954	0.949305	1.0263385
344405	PRORSD1P	prolyl-tRNA synthetase associated domain containing 1, pseudogene	0.99385	0.964362	1.0227433
344454	AOX2P	aldehyde oxidase 2 pseudogene	1.00361	0.98622	1.0065956
344462	KRT18P39	keratin 18 pseudogene 39	1.00404	1.014554	1.0251589
344558	SH3RF3	SH3 domain containing ring finger 3	0.99905	1.03522	0.9762087
344593	LOC344593	protein tyrosine phosphatase, non-receptor type 11 pseudogene	0.98733	0.954912	1.0214132
344595	LOC344595	hypothetical LOC344595	1.00345	1.023741	1.0118639
344653	LOC344653	ATP-binding cassette, sub-family F (GCN20), member 2 pseudogene	0.99834	0.999656	0.9993653
344657	LRRIQ4	leucine-rich repeats and IQ motif containing 4	0.98769	0.977638	1.0130638
344658	SAMD7	sterile alpha motif domain containing 7	1.00035	0.985867	1.0000479
344709	LOC344709	coiled-coil domain containing 75 pseudogene	0.99939	0.998435	1.0366698
344741	HNRNPA1P	heterogeneous nuclear ribonucleoprotein A1 pseudogene 20	1.01678	0.945046	0.9846787
344752	AADACL2	arylacetamide deacetylase-like 2	1.01189	1.005433	1.0059468
344758	GPR149	G protein-coupled receptor 149	1.02258	1.00713	1.0275675
344760	VN2R1P	vomeronasal 2 receptor 1 pseudogene	1.01223	1.03248	1.029703
344787	ZNF860	zinc finger protein 860	0.99695	1.000147	0.94428
344805	TMPRSS7	transmembrane protease, serine 7	1.03544	0.990475	0.9982838
344807	CD200R1L	CD200 receptor 1-like	1.01655	1.004048	1.0251665
344813	GSTO3P	glutathione S-transferase omega 3, pseudogene	0.98616	0.983056	1.0296669
344838	PAQR9	progestin and adipoQ receptor family member IX	0.99839	0.970396	0.9956596
344866	KRT18P17	keratin 18 pseudogene 17	1.01824	0.986408	1.0091247

344887	LOC344887	NmrA-like family domain containing 1 pseudogene	0.99618	0.977445	0.9887202
344892	RTP2	receptor (chemosensory) transporter protein 2	0.99756	0.980764	0.9956324
344901	OSTN	osteocrin	0.99729	0.980259	1.0098022
344905	ATP13A5	ATPase type 13A5	1.01134	1.005928	1.0085202
344967	LOC344967	acyl-CoA thioesterase 7 pseudogene	1.00704	0.992065	1.0383442
344978	LOC344978	actinin, alpha 4 pseudogene	0.96461	0.957991	1.0410076
344988	SAR1AP3	SAR1 homolog A (S. cerevisiae) pseudogene 3	1.00581	0.980687	0.9794054
345016	LOC345016	pescadillo homolog 1, containing BRCT domain (zebrafish) pseudogene	0.99635	0.992186	0.9917754
345041	HSPD1P5	heat shock 60kDa protein 1 (chaperonin) pseudogene 5	0.97845	0.997478	1.0208343
345051	LOC345051	hCG38984	0.99798	1.025551	0.9861067
345062	PRSS48	protease, serine, 48	1.01721	1.004215	1.0020921
345079	ANKRD56	ankyrin repeat domain 56	0.99379	1.009381	1.0229139
345193	LRIT3	leucine-rich repeat, immunoglobulin-like and transmembrane domains 3	1.0022	0.992691	1.0223254
345222	C4orf44	chromosome 4 open reading frame 44	0.99538	0.9966	1.0100647
345258	LOC345258	bridging integrator 2 pseudogene	0.97693	0.984115	0.9953694
345274	SLC10A6	solute carrier family 10 (sodium/bile acid cotransporter family), member 6	1.01659	0.957522	0.9936409
345275	HSD17B13	hydroxysteroid (17-beta) dehydrogenase 13	1.01336	1.021188	0.9922208
345430	KRT18P41	keratin 18 pseudogene 41	0.98737	1.008619	0.9319228
345456	PFN3	profilin 3	1.02821	1.069149	1.1042649
345462	ZNF879	zinc finger protein 879	0.96824	0.994193	0.936397
345471	LOC345471	cell division cycle 37 homolog (S. cerevisiae) pseudogene	1.01318	0.987876	1.0517271
345557	PLCXD3	phosphatidylinositol-specific phospholipase C, X domain containing 3	1.00562	1.001124	1.0087013
345571	LOC345571	RUN and FYVE domain containing 3 pseudogene	1.00436	1.017037	1.0235536
345576	LOC345576	ubiquitin specific peptidase 7 (herpes virus-associated) pseudogene	0.99306	0.964574	1.0362485
345611	IRGM	immunity-related GTPase family, M	0.97296	1.033703	0.9891444
345630	FBLL1	fibrillarin-like 1	0.95441	0.983759	1.0351159
345643	LOC345643	hypothetical LOC345643	1.00098	0.979362	1.0299991
345645	PSMC1P4	proteasome (prosome, macropain) 26S subunit, ATPase, 1 pseudogene 4	1.0034	0.948039	1.0383627
345651	ACTBL2	actin, beta-like 2	1.01545	1.007805	0.996447
345757	FAM174A	family with sequence similarity 174, member A	0.99094	0.986011	1.0808863
345778	MTX3	metaxin 3	1.00997	0.906919	0.9808266
345829	RAET1J	retinoic acid early transcript 1J pseudogene	0.98665	1.030983	1.0181454
345895	RSPH4A	radial spoke head 4 homolog A (Chlamydomonas)	1.0104	0.975011	1.0077645

345930	ECT2L	epithelial cell transforming sequence 2 oncogene-like	1.00929	1.023054	1.0194157
346007	EYS	eyes shut homolog (Drosophila)	1.0018	0.994489	1.0056285
346085	GAPDHP72	glyceraldehyde-3-phosphate dehydrogenase pseudogene 72	1.03411	1.024334	1.0480517
346157	ZNF391	zinc finger protein 391	0.97915	1.015983	1.0188491
346171	ZFP57	zinc finger protein 57 homolog (mouse)	0.98636	0.962582	1.0101926
346288	14-Sep	septin 14	0.98879	1.032095	1.012648
346296	LOC346296	insulin-like growth factor 2 mRNA binding protein 3	0.98787	0.93379	1.0478987
346329	LOC346329	guanine nucleotide binding protein (G protein), alpha 11 (Gq class) pseudogene	0.99073	0.99632	1.0190827
346389	MACC1	metastasis associated in colon cancer 1	0.99692	1.015978	1.0084346
346470	MRPL42P4	mitochondrial ribosomal protein L42 pseudogene 4	0.99096	0.95563	1.0689714
346517	OR6V1	olfactory receptor, family 6, subfamily V, member 1	1.03557	1.021572	1.0460003
346524	OR2Q1P	olfactory receptor, family 2, subfamily Q, member 1 pseudogene	1.00729	1.020728	1.0099811
346525	OR2A12	olfactory receptor, family 2, subfamily A, member 12	0.97806	1.038204	0.992083
346547	ERVFC1-1	endogenous retrovirus group FC1, member 1	1.02072	0.977968	0.9987224
346562	GNAT3	guanine nucleotide binding protein, alpha transducing 3	1.01231	1.000527	1.0118969
346606	MOGAT3	monoacylglycerol O-acyltransferase 3	1.00868	0.991257	0.9855842
346653	FAM71F2	family with sequence similarity 71, member F2	1.03604	1.013379	0.9755098
346673	STRA8	stimulated by retinoic acid gene 8 homolog (mouse)	1.00439	0.992938	0.9953121
346689	KLRG2	killer cell lectin-like receptor subfamily G, member 2	1.00347	0.995834	1.0310434
346702	LOC346702	protease, serine, 51-like	1.01593	0.986716	0.991085
346711	MRPL49P2	mitochondrial ribosomal protein L49 pseudogene 2	1.03603	1.000336	1.0329039
346990	H2AFZP2	H2A histone family, member Z pseudogene 2	0.99525	1.068875	1.0349198
347028	AFG3L2P1	AFG3 ATPase family gene 3-like 2 (S. cerevisiae) pseudogene 1	0.98728	1.030195	1.0091422
347051	SLC10A5	solute carrier family 10 (sodium/bile acid cotransporter family), member 5	0.96203	1.022036	1.0112922
347088	GPR144	G protein-coupled receptor 144	0.9969	0.989909	1.0308154
347097	LOC347097	dermatan sulfate epimerase pseudogene	1.0021	1.005842	1.0853857
347119	LOC347119	protein C15orf2-like	1.00887	1.01858	1.0094005
347127	FAM75D5	FAM75-like protein FLJ46321 pseudogene	0.997	0.98393	0.9608168
347168	OR1J1	olfactory receptor, family 1, subfamily J, member 1	0.9937	0.974866	1.0316746
347193	LOC347193	pescadillo homolog 1, containing BRCT domain (zebrafish) pseudogene	1.0134	0.989584	1.0233584
347240	KIF24	kinesin family member 24	0.98887	1.012012	1.0113481
347252	IGFBPL1	insulin-like growth factor binding protein-like 1	1.00951	1.013379	1.0221571
347265	KRT8P11	keratin 8 pseudogene 11	0.97976	1.041348	1.0178464

347273	MURC	muscle-related coiled-coil protein	0.99438	0.955393	1.0357932
347275	BAATP1	bile acid CoA: amino acid N-acyltransferase pseudogene 1	0.99303	1.001684	1.0287446
347281	LOC347281	topoisomerase I binding, arginine/serine-rich pseudogene	0.9991	0.981039	1.0101709
347333	KRT8P14	keratin 8 pseudogene 14	1.01987	1.003342	1.0305817
347344	ZNF81	zinc finger protein 81	0.9607	0.944297	1.0319882
347359	BMP2KL	BMP2 inducible kinase-like	0.9934	1.063187	1.0249961
347363	KIF4CP	kinesin family member 4C pseudogene	0.99793	1.032958	0.9787644
347365	ITIH5L	inter-alpha (globulin) inhibitor H5-like	1.00213	0.987618	0.9854011
347402	NLRP3P	NLR family, pyrin domain containing 3 pseudogene	1.00761	0.956304	1.0394107
347404	LANCL3	LanC lantibiotic synthetase component C-like 3 (bacterial)	0.98232	0.981077	0.9789203
347411	LOC347411	brain protein 44-like protein 2	0.99422	0.980963	1.007401
347422	LOC347422	TRM1 tRNA methyltransferase 1 homolog (S. cerevisiae) pseudogene	0.97416	1.013466	1.04744
347442	DCAF8L2	DDB1 and CUL4 associated factor 8-like 2	0.99259	1.010335	0.9719354
347454	ANKRD58	ankyrin repeat domain 58	0.94168	1.002659	1.0489411
347468	OR13H1	olfactory receptor, family 13, subfamily H, member 1	1.02643	0.980287	0.995499
347475	CCDC160	coiled-coil domain containing 160	0.9673	1.001223	1.0233166
347487	CXorf66	chromosome X open reading frame 66	0.99843	1.006	1.0151509
347516	DGAT2L6	diacylglycerol O-acyltransferase 2-like 6	1.00654	0.990112	0.9834153
347517	RAB41	RAB41, member RAS oncogene family	1.00512	0.970404	1.0402556
347527	ARSH	arylsulfatase family, member H	1.02038	1.023417	0.9984419
347541	MAGEB5	melanoma antigen family B, 5	1.00414	0.948991	0.9553884
347544	RPL18AP16	ribosomal protein L18a pseudogene 16	0.95665	0.951045	1.1034863
347549	LOC347549	centromere protein V pseudogene	1.01405	1.06777	1.0778898
347587	MRP63P10	mitochondrial ribosomal protein 63 pseudogene 10	1.01619	0.988943	0.9698719
347613	PARP4P1	poly (ADP-ribose) polymerase family, member 4 pseudogene 1	0.96588	0.970801	1.0380703
347675	EIF2S2P5	eukaryotic translation initiation factor 2, subunit 2 beta pseudogene 5	0.99673	1.048378	1.0474173
347687	GNG5P2	guanine nucleotide binding protein (G protein), gamma 5 pseudogene 2	0.93367	1.038613	1.03715
347688	TUBB8	tubulin, beta 8	1.00861	0.982814	1.0522626
347694	ECEL1P2	endothelin converting enzyme-like 1, pseudogene 2	1.00998	0.952423	1.0387253
347705	MRPS36P1	mitochondrial ribosomal protein S36 pseudogene 1	0.98534	0.919543	1.0045996
347706	MRPS36P2	mitochondrial ribosomal protein S36 pseudogene 2	1.02103	0.960454	0.9800866
347720	COTL1P2	coactosin-like 1 (Dictyostelium) pseudogene 2	0.9692	1.03257	1.0715566
347730	LRRTM1	leucine rich repeat transmembrane neuronal 1	1.01958	0.995937	1.0089711

347731	LRRTM3	leucine rich repeat transmembrane neuronal 3	0.993	1.001678	1.0153033
347732	CATSPER3	cation channel, sperm associated 3	0.98419	0.957815	0.9935777
347733	TUBB2B	tubulin, beta 2B	0.98842	1.005732	1.0210756
347734	SLC35B2	solute carrier family 35, member B2	1.0068	1.065352	1.0404801
347735	SERINC2	serine incorporator 2	0.99479	1.034885	1.0390275
347736	TXNDC6	thioredoxin domain containing 6	1.00926	0.998514	0.9868378
347741	OTOP3	otopetrin 3	0.99722	1.029694	1.0578414
347744	C6orf52	chromosome 6 open reading frame 52	1.01049	1.012209	0.9836023
347853	TBX10	T-box 10	1.01619	0.998546	0.9892248
347862	PDDC1	Parkinson disease 7 domain containing 1	0.97066	0.902004	1.0415279
347894	MRPL2P1	mitochondrial ribosomal protein L2 pseudogene 1	0.99049	1.026338	1.0212803
347902	AMIGO2	adhesion molecule with Ig-like domain 2	0.92546	1.018887	0.9899779
347918	EP400NL	EP400 N-terminal like	0.9947	0.996485	1.0088879
347933	NLRP9P	NLR family, pyrin domain containing 9 pseudogene	0.98365	1.023066	0.9482362
348013	FAM70B	family with sequence similarity 70, member B	0.97756	1.000541	0.9924792
348021	LOC348021	hypothetical LOC348021	1.02588	0.982591	0.9484417
348094	ANKDD1A	ankyrin repeat and death domain containing 1A	1.03269	1.030837	0.9539698
348110	C15orf38	chromosome 15 open reading frame 38	0.96565	1.006832	1.035664
348158	ACSM2B	acyl-CoA synthetase medium-chain family member 2B	1.01912	1.047555	1.054376
348180	CTU2	cytosolic thiouridylase subunit 2 homolog (S. pombe)	0.98454	0.982426	1.0220962
348235	SKA2	spindle and kinetochore associated complex subunit 2	0.93115	0.983186	1.0040569
348303	SELV	selenoprotein V	1.01568	1.001773	1.0211301
348327	ZNF530	zinc finger protein 530	0.9694	1.012038	1.002702
348378	FAM159A	family with sequence similarity 159, member A	1.01535	0.959947	0.9957492
348487	FAM131C	family with sequence similarity 131, member C	1.00463	0.986037	1.0167966
348645	C22orf34	chromosome 22 open reading frame 34	0.98029	0.938279	0.9959822
348654	GEN1	Gen homolog 1, endonuclease (Drosophila)	1.01398	0.946524	0.944293
348751	FONG	hypothetical LOC348751	1.00471	0.998706	1.0129204
348793	WDR53	WD repeat domain 53	0.99689	0.868114	1.0037095
348801	LNP1	leukemia NUP98 fusion partner 1	0.99741	1.014251	1.0071451
348807	CCDC37	coiled-coil domain containing 37	1.00627	0.997746	0.9971576
348808	NCRNA001	non-protein coding RNA 119	1.01929	0.995376	0.9811372
348825	TPRXL	tetra-peptide repeat homeobox-like	0.98769	0.989794	0.965038

348840	LOC348840	hypothetical LOC348840	0.99967	0.997183	1.0182063
348910	RAF1P1	v-raf-1 murine leukemia viral oncogene homolog 1 pseudogene 1	1.01065	1.041125	0.9866983
348926	LOC348926	family with sequence similarity 86, member A pseudogene	1.0137	0.998102	1.0130312
348932	SLC6A18	solute carrier family 6, member 18	1.02154	0.988096	0.9991022
348938	NIPAL4	NIPA-like domain containing 4	1.00023	1.011768	0.9837972
348958	LOC348958	mitochondrial ribosomal protein L10 pseudogene	1.03662	0.97565	0.9897988
348980	HCN1	hyperpolarization activated cyclic nucleotide-gated potassium channel 1	1.0014	1.011696	1.0222305
348995	NUP43	nucleoporin 43kDa	1.0003	1.007259	0.9742559
349075	ZNF713	zinc finger protein 713	1.00239	0.978787	1.0031771
349136	WDR86	WD repeat domain 86	1.00086	1.04537	1.0449498
349149	GJC3	gap junction protein, gamma 3, 30.2kDa	1.00035	0.992269	1.0500316
349152	DPY19L2P2	dpy-19-like 2 pseudogene 2 (C. elegans)	0.98309	1.017518	1.0093466
349158	CYCSP20	cytochrome c, somatic pseudogene 20	1.00896	0.965418	0.997738
349236	[No Symbo	[No Name]	0.93539	1.065575	0.9350769
349372	NANOGP10	Nanog homeobox pseudogene 10	1.01666	0.973797	1.006526
349386	NANOGP9	Nanog homeobox pseudogene 9	0.97216	0.95579	1.0327721
349391	CYCSP44	cytochrome c, somatic pseudogene 44	1.00718	1.026168	1.035528
349565	NMNAT3	nicotinamide nucleotide adenylyltransferase 3	0.99402	1.002182	0.9874913
349633	C11orf34	chromosome 11 open reading frame 34	0.99628	1.034013	0.9792819
349667	RTN4RL2	reticulon 4 receptor-like 2	0.99333	1.011348	1.0634828
350383	GPR142	G protein-coupled receptor 142	0.98905	1.011497	0.9721997
352852	CYCSP45	cytochrome c, somatic pseudogene 45	0.98085	0.920419	1.0581976
352887	GYG2P1	glycogenin 2 pseudogene 1	0.99593	1.030964	0.9094327
352905	CTBP2P1	C-terminal binding protein 2 pseudogene 1	0.95863	0.990867	1.0163611
352909	C19orf51	chromosome 19 open reading frame 51	0.9788	0.993144	0.981367
352954	GATS	GATS, stromal antigen 3 opposite strand	1.00699	0.941993	0.9972704
352966	HLA-W	major histocompatibility complex, class I, W (pseudogene)	1.00775	0.946639	0.9676126
352993	HCP5P6	HLA complex P5 pseudogene 6	1.04	0.967403	1.0216538
352999	C6orf58	chromosome 6 open reading frame 58	0.996	1.020018	1.0119291
353006	HCG2P6	HLA complex group 2 pseudogene 6	0.98382	0.947368	0.9881223
353013	RPL7AP7	ribosomal protein L7a pseudogene 7	1.03645	0.997083	0.9988905
353088	ZNF429	zinc finger protein 429	0.9599	1.011485	1.0132101
353091	RAET1G	retinoic acid early transcript 1G	0.99875	0.997155	1.0023186

353116	RILPL1	Rab interacting lysosomal protein-like 1	0.99974	0.975368	0.9861061
353132	LCE1B	late cornified envelope 1B	1.00129	0.944268	0.9978392
353133	LCE1C	late cornified envelope 1C	1.02504	1.022857	0.9889431
353135	LCE1E	late cornified envelope 1E	1.01945	1.001422	1.0068811
353139	LCE2A	late cornified envelope 2A	0.98879	1.023107	1.0587987
353141	LCE2D	late cornified envelope 2D	1.04347	0.977905	0.9456543
353142	LCE3A	late cornified envelope 3A	0.99117	1.022523	0.9598199
353143	LCE3B	late cornified envelope 3B	1.02241	0.962858	1.0248703
353144	LCE3C	late cornified envelope 3C	1.02259	1.015427	1.0139507
353145	LCE3E	late cornified envelope 3E	1.00578	0.987553	1.0147232
353149	TBC1D26	TBC1 domain family, member 26	0.99634	0.963338	1.012857
353164	TAS2R42	taste receptor, type 2, member 42	1.00894	0.983592	1.0198952
353174	ZACN	zinc activated ligand-gated ion channel	0.98088	0.995996	0.9738803
353189	SLCO4C1	solute carrier organic anion transporter family, member 4C1	0.99665	0.97822	0.9920513
353194	LOC353194	keratin pseudogene	1.03963	1.001764	0.9986475
353219	KAAG1	kidney associated antigen 1	0.97262	1.066403	1.0426907
353238	PADI6	peptidyl arginine deiminase, type VI	0.99824	1.002076	1.0012227
353274	ZNF445	zinc finger protein 445	0.9741	0.9288	0.9552964
353288	KRT26	keratin 26	0.998	1.031025	0.9878524
353299	RGSL1	regulator of G-protein signaling like 1	1.0076	1.011565	1.0032887
353322	ANKRD37	ankyrin repeat domain 37	0.98727	0.977909	1.0092142
353324	SPATA12	spermatogenesis associated 12	0.99158	0.974175	0.9794386
353329	PSIP1P1	PC4 and SFRS1 interacting protein 1 pseudogene 1	0.94923	0.892623	0.9859613
353333	KRTAP10-1	keratin associated protein 10-10	1.00407	1.01546	0.9568032
353355	ZNF233	zinc finger protein 233	0.99245	1.011814	1.0071853
353376	TICAM2	toll-like receptor adaptor molecule 2	1.02316	0.976017	1.0507095
353497	POLN	polymerase (DNA directed) nu	0.97826	0.994978	0.9886219
353500	BMP8A	bone morphogenetic protein 8a	1.01539	0.989199	0.9794089
353511	PKD1P6	polycystic kidney disease 1 (autosomal dominant) pseudogene 6	0.9671	0.994426	1.016073
353514	LILRA5	leukocyte immunoglobulin-like receptor, subfamily A (with TM domain), member 5	1.00042	1.051299	0.8929829
359710	C20orf185	chromosome 20 open reading frame 185	0.99771	0.996997	0.9775306
359724	RN18S2P	RNA, 18S ribosomal 2, pseudogene	1.03604	1.065366	1.010795
359731	MRP63P7	mitochondrial ribosomal protein 63 pseudogene 7	1.01651	1.036059	0.9727185

359732	MRP63P8	mitochondrial ribosomal protein 63 pseudogene 8	1.0053	1.033054	0.9877334
359736	MRPL15P1	mitochondrial ribosomal protein L15 pseudogene 1	1.00964	1.005063	1.0320107
359737	MRPL20P1	mitochondrial ribosomal protein L20 pseudogene 1	0.96002	0.99706	1.0571373
359739	MRPL3P1	mitochondrial ribosomal protein L3 pseudogene 1	1.00793	1.005109	1.0162497
359740	MRPL30P1	mitochondrial ribosomal protein L30 pseudogene 1	0.96196	1.05818	0.9830874
359743	MRPL35P2	mitochondrial ribosomal protein L35 pseudogene 2	0.97188	0.977798	1.0876949
359745	MRPL35P4	mitochondrial ribosomal protein L35 pseudogene 4	0.98989	1.029705	0.9924113
359747	MRPL45P1	mitochondrial ribosomal protein L45 pseudogene 1	0.97009	0.950746	1.0289027
359748	MRPL49P1	mitochondrial ribosomal protein L49 pseudogene 1	1.00411	1.001203	1.0436484
359755	MRPS15P1	mitochondrial ribosomal protein S15 pseudogene 1	1.03281	1.021877	0.9960845
359757	MRPS17P3	mitochondrial ribosomal protein S17 pseudogene 3	1.03479	0.996942	1.0179596
359763	MRPS18BP	mitochondrial ribosomal protein S18B pseudogene 2	1.01578	1.054499	1.0903735
359764	MRPS18CP	mitochondrial ribosomal protein S18C pseudogene 3	1.02767	1.017099	1.0558762
359774	MRPS29P2	mitochondrial ribosomal protein S29 pseudogene 2	0.99453	1.041446	1.0121907
359776	MRPS33P3	mitochondrial ribosomal protein S33 pseudogene 3	1.02297	1.078557	0.9348415
359791	MED14P1	mediator complex subunit 14 pseudogene 1	0.97439	1.056836	0.9586749
359803	MRPS17P1	mitochondrial ribosomal protein S17 pseudogene 1	1.03028	1.024308	1.0827826
359804	MRPS29P1	mitochondrial ribosomal protein S29 pseudogene 1	1.02737	1.049016	1.0594232
359821	MRPL42P5	mitochondrial ribosomal protein L42 pseudogene 5	0.95494	0.961021	0.9894404
359844	PRDX2P1	peroxiredoxin 2 pseudogene 1	0.9822	1.026525	1.0575871
359845	FAM101B	family with sequence similarity 101, member B	0.9471	1.076934	0.997835
359948	IRF2BP2	interferon regulatory factor 2 binding protein 2	0.99477	0.980056	0.9650207
360008	GAPDHP17	glyceraldehyde-3-phosphate dehydrogenase pseudogene 17	1.02759	1.002133	0.9878674
360009	CYCSP49	cytochrome c, somatic pseudogene 49	1.00532	0.995846	0.9763408
360015	RFTN1P1	raftlin, lipid raft linker 1 pseudogene 1	0.9802	0.981014	1.0020525
360021	PPP1R12BP	protein phosphatase 1, regulatory (inhibitor) subunit 12B pseudogene, Y-linked 1	1.01307	0.968816	0.9715771
360023	ZBTB41	zinc finger and BTB domain containing 41	0.94545	0.975841	1.0312597
360030	NANOGNB	NANOG neighbor homeobox	0.99624	0.978452	0.98515
360159	CYCSP6	cytochrome c, somatic pseudogene 6	1.0391	1.028763	0.9875276
360160	CYCSP8	cytochrome c, somatic pseudogene 8	0.98188	0.994342	0.9729466
360166	CYCSP10	cytochrome c, somatic pseudogene 10	0.96716	0.990665	0.974617
360167	CYCSP11	cytochrome c, somatic pseudogene 11	0.97289	0.947385	1.0396022
360174	CYCSP19	cytochrome c, somatic pseudogene 19	1.02084	0.951658	1.0329661

360200	TMPRSS9	transmembrane protease, serine 9	1.00053	1.011773	1.0599453
360203	GLT6D1	glycosyltransferase 6 domain containing 1	0.98949	0.98229	0.9942047
360226	PRSS41	protease, serine, 41	0.99708	1.005089	1.0194357
360227	HIN1L	OTU domain containing 4 pseudogene	0.98812	0.966314	1.0214603
373156	GSTK1	glutathione S-transferase kappa 1	0.99719	0.901299	1.0722869
373509	USP50	ubiquitin specific peptidase 50	0.98214	1.029772	0.9937779
373861	HILS1	histone linker H1 domain, spermatid-specific 1	1.01596	0.949885	1.0048837
373863	DND1	dead end homolog 1 (zebrafish)	1.02857	1.025341	0.987225
374286	CDRT1	CMT1A duplicated region transcript 1	0.99776	1.00091	0.9887398
374291	NDUFS7	NADH dehydrogenase (ubiquinone) Fe-S protein 7, 20kDa (NADH-coenzyme Q reductase)	0.97097	0.978541	1.0494883
374308	PTCHD3	patched domain containing 3	0.97513	1.025435	0.9897243
374354	NHLRC2	NHL repeat containing 2	0.94405	0.945014	1.0455867
374355	C10orf96	chromosome 10 open reading frame 96	0.98953	1.025964	1.0192211
374378	GALNTL4	UDP-N-acetyl-alpha-D-galactosamine:polypeptide N-acetylgalactosaminyltransferase-like 4	1.00148	0.99972	1.0052838
374383	B7H6	B7 homolog 6	1.0026	0.989615	0.9723757
374387	DKFZp779N	hypothetical DKFZp779M0652	0.98509	0.983758	1.0467347
374393	FAM111B	family with sequence similarity 111, member B	0.99182	0.950967	1.1168774
374395	TMEM179E	transmembrane protein 179B	0.96548	0.97992	1.0074851
374403	TBC1D10C	TBC1 domain family, member 10C	0.93005	0.975053	0.9531424
374407	DNAJB13	DnaJ (Hsp40) homolog, subfamily B, member 13	1.00115	1.012185	1.0112233
374443	LOC374443	CLR pseudogene	0.93334	0.865515	0.9862756
374454	KRT77	keratin 77	1.00135	1.018892	0.9931104
374462	PTPRQ	protein tyrosine phosphatase, receptor type, Q	0.9909	0.995205	1.0197136
374467	C12orf63	chromosome 12 open reading frame 63	0.99412	1.029808	1.0082249
374470	C12orf42	chromosome 12 open reading frame 42	0.97805	0.996193	0.9964226
374491	LOC374491	TPTE and PTEN homologous inositol lipid phosphatase pseudogene	1.04517	1.013178	1.0384234
374500	THSD1P1	thrombospondin, type I, domain containing 1 pseudogene 1	0.96264	0.922883	0.9715879
374569	ASPG	asparaginase homolog (S. cerevisiae)	1.00454	0.984615	1.0071497
374618	TEX9	testis expressed 9	0.95925	0.96868	0.98954
374654	KIF7	kinesin family member 7	0.97949	0.990213	1.0445084
374655	ZNF710	zinc finger protein 710	0.99623	0.972965	0.9932933
374659	HDDC3	HD domain containing 3	0.96529	0.950113	1.0378543
374739	TEPP	testis, prostate and placenta expressed	0.97142	0.972252	1.032621

374768	SPEM1	spermatid maturation 1	1.00219	0.975582	0.9910202
374786	EFCAB5	EF-hand calcium binding domain 5	1.00088	1.023964	0.9985185
374819	LRRC37A3	leucine rich repeat containing 37, member A3	1.02433	1.013171	0.9632575
374860	ANKRD30B	ankyrin repeat domain 30B	0.98614	0.997971	0.9954847
374864	C18orf34	chromosome 18 open reading frame 34	1.00416	1.013115	0.9995792
374868	ATP9B	ATPase, class II, type 9B	0.95116	0.950188	0.9356174
374872	C19orf35	chromosome 19 open reading frame 35	0.98555	0.97746	0.9129719
374875	HSD11B1L	hydroxysteroid (11-beta) dehydrogenase 1-like	1.0033	0.992919	1.051382
374877	C19orf45	chromosome 19 open reading frame 45	0.99991	1.01125	0.9548166
374879	ZNF699	zinc finger protein 699	0.92793	0.966538	0.9652154
374882	TMEM205	transmembrane protein 205	0.91241	1.040172	1.0237337
374887	YJEFN3	YjeF N-terminal domain containing 3	0.99872	1.005059	1.0439029
374897	SBSN	suprabasin	0.99236	0.971826	1.0178129
374899	ZNF829	zinc finger protein 829	0.97648	0.967504	1.0306908
374900	ZNF568	zinc finger protein 568	0.88892	0.958525	1.0067279
374907	B3GNT8	UDP-GlcNAc:betaGal beta-1,3-N-acetylglucosaminyltransferase 8	0.99036	0.979333	0.974873
374918	IGFL1	IGF-like family member 1	1.00501	0.981836	0.9911165
374920	C19orf68	chromosome 19 open reading frame 68	0.97015	0.993602	1.0165069
374928	ZNF773	zinc finger protein 773	0.97915	0.97103	1.0075813
374946	C1orf187	chromosome 1 open reading frame 187	1.01586	1.003884	1.0319885
374955	SPATA21	spermatogenesis associated 21	1.02184	1.034423	0.9933673
374969	CCDC23	coiled-coil domain containing 23	0.9419	0.927459	1.0024037
374973	ATPAF1-AS	ATPAF1 antisense RNA 1 (non-protein coding)	0.99758	1.00903	0.9449612
374977	HEATR8	HEAT repeat containing 8	0.99652	0.996848	0.9865909
374986	FAM73A	family with sequence similarity 73, member A	0.97798	0.970543	0.984641
374987	C1orf118	chromosome 1 open reading frame 118	1.00349	0.99954	1.0098708
375033	PEAR1	platelet endothelial aggregation receptor 1	1.00768	0.976945	0.9658533
375035	SFT2D2	SFT2 domain containing 2	1.03704	0.857346	1.042433
375056	MIA3	melanoma inhibitory activity family, member 3	0.9816	0.99975	0.9643651
375057	C1orf95	chromosome 1 open reading frame 95	1.01198	1.019252	1.010241
375061	FAM89A	family with sequence similarity 89, member A	0.98359	0.999473	0.9929102
375189	PFN4	profilin family, member 4	1.01935	1.015914	0.9811819
375190	LOC375190	hypothetical protein LOC375190	0.99119	0.963022	1.0071435

375287	RBM43	RNA binding motif protein 43	0.95863	0.947843	0.9335196
375295	LOC375295	hypothetical LOC375295	1.00225	0.978288	0.9961355
375298	CERKL	ceramide kinase-like	1.01145	1.008409	1.0153519
375307	C2orf62	chromosome 2 open reading frame 62	1.01096	1.003283	0.9790398
375316	RBM44	RNA binding motif protein 44	1.0011	1.016934	0.9448521
375323	LHFPL4	lipoma HMGIC fusion partner-like 4	1.00526	0.997119	1.0117162
375337	C3orf77	chromosome 3 open reading frame 77	1.01243	1.005226	1.0053537
375341	C3orf62	chromosome 3 open reading frame 62	0.98723	1.021868	0.9955359
375346	TMEM110	transmembrane protein 110	1.0159	0.921025	0.9735236
375347	[No Symbo	[No Name]	1.00438	1.001868	1.0165933
375387	LRRC33	leucine rich repeat containing 33	0.97186	1.006715	1.0178404
375444	C5orf34	chromosome 5 open reading frame 34	1.00008	0.965012	1.0118103
375449	MAST4	microtubule associated serine/threonine kinase family member 4	0.9939	0.962337	0.9804351
375484	C5orf25	chromosome 5 open reading frame 25	0.98387	1.003368	0.9516233
375513	GUSBP4	glucuronidase, beta pseudogene 4	1.00248	0.954498	1.0025964
375519	GJB7	gap junction protein, beta 7, 25kDa	1.01051	1.051049	0.9632863
375567	VWC2	von Willebrand factor C domain containing 2	0.9948	1.012592	1.0367111
375607	C7orf52	chromosome 7 open reading frame 52	0.98277	0.991845	1.0305405
375611	SLC26A5	solute carrier family 26, member 5 (prestin)	1.01809	1.028246	1.0062231
375612	LHFPL3	lipoma HMGIC fusion partner-like 3	1.00636	1.002546	1.0159506
375686	SPATC1	spermatogenesis and centriole associated 1	1.01573	1.002096	1.0248705
375704	ENHO	energy homeostasis associated	0.98443	1.013708	1.030194
375743	PTAR1	protein prenyltransferase alpha subunit repeat containing 1	0.9487	0.965952	1.0151879
375748	C9orf102	chromosome 9 open reading frame 102	0.92631	0.950036	1.0468117
375757	C9orf119	chromosome 9 open reading frame 119	0.9181	0.979763	1.0401979
375759	C9orf50	chromosome 9 open reading frame 50	0.98953	1.013605	0.9999189
375775	PNPLA7	patatin-like phospholipase domain containing 7	0.98666	0.991411	1.0280112
375790	AGRN	agrin	0.98399	1.022899	1.0205079
375940	FLJ46361	deleted in malignant brain tumors 1 pseudogene	1.00542	1.001297	0.9917493
376132	LRRC10	leucine rich repeat containing 10	0.99231	0.993604	0.9893774
376267	RAB15	RAB15, member RAS onocogene family	0.97949	1.013302	0.9928384
376412	RNF126P1	ring finger protein 126 pseudogene 1	0.95911	1.046763	1.0585944
376497	SLC27A1	solute carrier family 27 (fatty acid transporter), member 1	0.98383	0.907966	1.050887

376693	RPS10P7	ribosomal protein S10 pseudogene 7	1.01576	0.99537	1.0451285
376844	SDC4P	syndecan 4 pseudogene	0.99269	1.079156	1.0251822
376940	ZC3H6	zinc finger CCCH-type containing 6	0.96348	0.890869	0.9394598
377007	KLHL30	kelch-like 30 (Drosophila)	1.01411	1.010198	1.0106282
377047	PRSS45	protease, serine, 45	1.00529	1.025521	1.0168837
377677	CA13	carbonic anhydrase XIII	0.95677	0.915491	1.0006479
377841	ENTPD8	ectonucleoside triphosphate diphosphohydrolase 8	0.98717	1.0138	1.0351818
377997	TSPY12P	testis specific protein, Y-linked 12, pseudogene	0.98053	1.037615	1.0208307
378013	TSPY14P	testis specific protein, Y-linked 14, pseudogene	1.00087	1.02038	0.989751
378708	APITD1	apoptosis-inducing, TAF9-like domain 1	0.98723	1.008828	1.0294275
378807	CATSPER4	cation channel, sperm associated 4	1.00662	0.994361	1.0113019
378818	RPL37P3	ribosomal protein L37 pseudogene 3	1.00607	0.991067	0.9603124
378832	NCRNA001	non-protein coding RNA 175	0.98809	0.985516	1.0083398
378884	NHLRC1	NHL repeat containing 1	0.96355	0.996965	1.1114406
378938	MALAT1	metastasis associated lung adenocarcinoma transcript 1 (non-protein coding)	1.07348	1.042437	0.8755365
378953	RBMY2HP	RNA binding motif protein, Y-linked, family 2, member H pseudogene	0.99788	1.005811	1.0156684
378955	RBMY2JP	RNA binding motif protein, Y-linked, family 2, member J pseudogene	1.01167	1.0139	0.9619637
379005	RBMY2OP	RNA binding motif protein, Y-linked, family 2, member O pseudogene	0.97924	1.017685	0.9700745
379006	RBMY2QP	RNA binding motif protein, Y-linked, family 2, member Q pseudogene	0.99977	1.013622	1.0564513
379007	RBMY2SP	RNA binding motif protein, Y-linked, family 2, member S pseudogene	0.96694	0.994219	0.9857866
379025	FLJ31306	hypothetical LOC379025	0.95715	0.924601	0.9804204
379027	TSPY5P	testis specific protein, Y-linked 5, pseudogene	1.0005	1.032021	1.0025472
379034	FLJ10489	hypothetical protein FLJ10489	1.00058	1.015346	1.0075022
386608	RNASEH2CI	ribonuclease H2, subunit C pseudogene 1	1.00974	0.999314	0.9924227
386617	KCTD8	potassium channel tetramerisation domain containing 8	0.99682	1.034053	1.0164545
386618	KCTD4	potassium channel tetramerisation domain containing 4	1.0146	1.015403	0.9834686
386653	IL31	interleukin 31	1.01832	1.042155	1.0046035
386654	OSTF1P1	osteoclast stimulating factor 1 pseudogene 1	0.9855	1.000074	0.9951811
386672	KRTAP10-4	keratin associated protein 10-4	0.97769	1.025615	0.9670732
386675	KRTAP10-7	keratin associated protein 10-7	1.02301	1.035103	1.0893454
386676	KRTAP10-9	keratin associated protein 10-9	1.00679	1.075072	0.9938174
386678	KRTAP10-1	keratin associated protein 10-11	0.99027	0.900193	0.994458
386683	KRTAP12-3	keratin associated protein 12-3	1.02303	1.016016	0.9900835

386685	KRTAP10-1	keratin associated protein 10-12	0.97836	0.966387	1.1429024
386687	OFD1P3Y	OFD1 pseudogene 3, Y-linked	0.97393	0.966672	1.0189831
386690	OFD1P6Y	OFD1 pseudogene 6, Y-linked	0.99521	0.978924	0.9896101
386724	AMIGO3	adhesion molecule with Ig-like domain 3	1.00226	1.032018	0.9361755
386726	FAM8A4P	family with sequence similarity 8, member A1 pseudogene	0.99193	0.989611	0.9497146
386729	CDY4P	chromodomain protein, Y-linked 4 pseudogene	1.00848	0.94219	1.0288873
386731	CDY7P	chromodomain protein, Y-linked 7 pseudogene	0.99972	0.955933	1.0027731
386746	MRGPRG	MAS-related GPR, member G	0.99246	0.961059	1.0284632
387032	ZKSCAN4	zinc finger with KRAB and SCAN domains 4	0.96665	1.002709	1.0422346
387066	SNHG5	small nucleolar RNA host gene 5 (non-protein coding)	0.97072	0.958103	1.0409823
387103	CENPW	centromere protein W	1.00781	1.004784	1.0391887
387104	C6orf174	chromosome 6 open reading frame 174	0.9995	0.972026	1.0509154
387119	C6orf204	chromosome 6 open reading frame 204	0.97761	0.971056	0.9866227
387129	NPSR1	neuropeptide S receptor 1	1.00939	1.017899	1.0154488
387263	C6orf120	chromosome 6 open reading frame 120	1.00422	0.897868	0.9911965
387264	KRTAP5-1	keratin associated protein 5-1	1.00909	1.07144	0.9311641
387266	KRTAP5-3	keratin associated protein 5-3	1.0075	1.002697	0.9584672
387332	TBPL2	TATA box binding protein like 2	1.01063	1.017149	0.9778837
387338	NSUN4	NOP2/Sun domain family, member 4	0.97965	0.915067	1.0059095
387357	THEMIS	thymocyte selection associated	0.96451	1.038559	0.9313566
387496	RASL11A	RAS-like, family 11, member A	0.98877	0.997401	1.0076826
387509	GPR153	G protein-coupled receptor 153	1.01753	0.997075	1.0355821
387522	TMEM189-	TMEM189-UBE2V1 readthrough	0.98962	0.93926	1.0138504
387535	HCRP1	hepatocellular carcinoma-related HCRP1	1.02583	1.015831	1.0578469
387590	psiTPTE22	TPTE pseudogene	0.95079	0.96766	0.9951716
387597	ILDR2	immunoglobulin-like domain containing receptor 2	1.02945	0.983157	1.0215636
387601	SLC22A25	solute carrier family 22, member 25	0.98159	1.030603	1.0142549
387640	C10orf140	chromosome 10 open reading frame 140	0.99597	0.976195	1.0297869
387642	C10orf115	chromosome 10 open reading frame 115	1.01133	0.986373	0.9964322
387644	NCRNA002	non-protein coding RNA 202	0.98455	1.012739	0.961601
387646	LOC387646	leucine rich repeat containing 37, member A family pseudogene	1.00577	0.986005	0.9997817
387647	LOC387647	patched domain containing 3 pseudogene	0.94667	0.898428	0.9916069
387654	[No Symbo	[No Name]	0.94662	0.959073	0.9632793

387694	SH2D4B	SH2 domain containing 4B	1.01733	1.005848	1.0017473
387695	C10orf99	chromosome 10 open reading frame 99	1.0219	0.993173	1.0218964
387700	SLC16A12	solute carrier family 16, member 12 (monocarboxylic acid transporter 12)	0.99928	1.012811	1.0052462
387703	LOC387703	x-ray repair cross-complementing protein 6-like	0.99793	1.003965	1.0081043
387707	CC2D2B	coiled-coil and C2 domain containing 2B	1.00464	1.022873	1.0421232
387712	ENO4	enolase family member 4	0.99389	0.992796	0.9964381
387715	ARMS2	age-related maculopathy susceptibility 2	0.99423	0.982726	0.9872004
387720	LOC387720	hypothetical LOC387720	1.0084	0.968156	1.0271255
387723	LOC387723	hypothetical LOC387723	0.96142	0.961627	0.9717292
387745	LOC387745	family with sequence similarity 86, member A pseuodgene	1.00295	0.953654	1.0200645
387747	OR52B3P	olfactory receptor, family 52, subfamily B, member 3 pseudogene	1.02183	0.972797	0.94663
387748	OR56B1	olfactory receptor, family 56, subfamily B, member 1	0.9776	0.992562	0.9227338
387755	INSC	inscuteable homolog (Drosophila)	0.99678	1.006383	1.0161782
387758	FIBIN	fin bud initiation factor homolog (zebrafish)	0.98612	0.979501	0.9840414
387761	[No Symbo	[No Name]	0.99644	1.001965	1.0352649
387763	C11orf96	chromosome 11 open reading frame 96	0.99549	0.977938	1.0330116
387775	SLC22A10	solute carrier family 22, member 10	1.00862	0.978707	1.0104429
387778	SPDYC	speedy homolog C (Xenopus laevis)	0.99848	0.92569	1.0289294
387787	LIPT2	lipoyl(octanoyl) transferase 2 (putative)	0.97525	0.95609	1.0030028
387791	LOC387791	mitochondrial import receptor subunit TOM20 homolog	0.97195	1.013387	0.9594706
387804	C11orf90	chromosome 11 open reading frame 90	0.99349	0.985016	1.003212
387815	OR8G3P	olfactory receptor, family 8, subfamily G, member 3 pseudogene	1.01713	1.024334	0.9640115
387825	[No Symbo	[No Name]	0.98698	1.015423	1.0040237
387826	[No Symbo	[No Name]	0.99563	0.983278	0.9776386
387837	CLEC12B	C-type lectin domain family 12, member B	1.01026	1.036061	1.0765059
387841	RPL13AP20	ribosomal protein L13a pseudogene 20	0.99306	1.051491	0.9615351
387845	EEF1A1P16	eukaryotic translation elongation factor 1 alpha 1 pseudogene 16	0.96983	0.93008	1.0705361
387849	REP15	RAB15 effector protein	1.00742	1.000678	0.9793917
387856	C12orf68	chromosome 12 open reading frame 68	0.95539	0.982805	1.0310374
387869	LOC387869	microtubule-associated proteins 1A/1B light chain 3B-like	0.98228	0.968454	0.9792817
387876	[No Symbo	[No Name]	0.98465	0.9846	1.0141408
387882	C12orf75	chromosome 12 open reading frame 75	0.99939	1.062559	1.018985
387885	CCDC42B	coiled-coil domain containing 42B	1.0091	1.000882	0.9907206

387890	TMEM233	transmembrane protein 233	0.98968	1.005914	1.0170045
387893	SETD8	SET domain containing (lysine methyltransferase) 8	1.00718	1.002898	1.0540115
387914	SHISA2	shisa homolog 2 (Xenopus laevis)	1.00425	1.01652	1.0017746
387921	NHLRC3	NHL repeat containing 3	0.98452	0.940653	1.0775926
387923	SERP2	stress-associated endoplasmic reticulum protein family member 2	0.99714	0.99963	1.0021243
387924	OGFOD1P1	2-oxoglutarate and iron-dependent oxygenase domain containing 1 pseudogene 1	0.97887	1.060756	0.9925161
387933	HNRNPA3P	heterogeneous nuclear ribonucleoprotein A3 pseudogene 5	0.97739	1.001258	1.0450015
387939	LOC387939	hCG2045417	1.00362	1.027343	0.9919503
387941	[No Symbo	[No Name]	0.9916	0.9917	1.1249459
387978	C14orf23	chromosome 14 open reading frame 23	0.98831	1.042609	1.0174716
387990	TOMM20L	translocase of outer mitochondrial membrane 20 homolog (yeast)-like	0.96648	0.948052	0.9604587
388002	[No Symbo	[No Name]	1.01138	1.009722	0.9995363
388007	SERPINA13	serpin peptidase inhibitor, clade A (alpha-1 antiproteinase, antitrypsin), member 13 (pseudogene)	0.98314	0.991623	0.9888538
388021	TMEM179	transmembrane protein 179	0.99948	1.001931	0.9740827
388104	LOC388104	transmembrane protein 183A pseudogene	0.99774	1.000576	1.0518969
388115	C15orf52	chromosome 15 open reading frame 52	0.99394	0.985398	1.0002769
388121	TNFAIP8L3	tumor necrosis factor, alpha-induced protein 8-like 3	0.99912	0.992192	0.9993173
388122	RPSAP55	ribosomal protein SA pseudogene 55	0.99533	1.022755	1.0315505
388125	C2CD4B	C2 calcium-dependent domain containing 4B	0.9834	0.996156	1.0857275
388135	C15orf59	chromosome 15 open reading frame 59	0.99225	0.995428	0.9943574
388177	[No Symbo	[No Name]	0.98946	0.946835	1.0283228
388181	LOC388181	protein FAM149B1-like	0.99183	1.032297	1.0363403
388182	FLJ42289	hypothetical LOC388182	0.96528	1.040078	0.9384303
388199	PRR25	proline rich 25	0.98214	0.999123	1.0360084
388210	LOC388210	hypothetical protein LOC388210	1.00668	0.986209	0.9661249
388228	SBK1	SH3-binding domain kinase 1	0.98066	1.004682	0.9485543
388272	C16orf87	chromosome 16 open reading frame 87	0.96429	0.94488	1.0083513
388276	LOC388276	hCG2045437	1.01677	1.001143	1.0032731
388279	[No Symbo	[No Name]	0.99415	0.990429	1.0167397
388282	LOC388282	hypothetical LOC388282	1.00042	0.97633	0.9791612
388284	C16orf86	chromosome 16 open reading frame 86	0.98379	1.00674	1.0433863
388323	GLTPD2	glycolipid transfer protein domain containing 2	0.95928	1.003584	1.0535422
388324	INCA1	inhibitor of CDK, cyclin A1 interacting protein 1	0.98206	0.99445	0.9910322

388325	C17orf87	chromosome 17 open reading frame 87	1.01705	0.911418	1.0101145
388327	C17orf100	chromosome 17 open reading frame 100	0.98869	0.964194	1.0568596
388333	SPDYE4	speedy homolog E4 (Xenopus laevis)	1.01156	0.991131	0.9952402
388335	TMEM220	transmembrane protein 220	1.00788	1.001519	0.9859327
388336	SHISA6	shisa homolog 6 (Xenopus laevis)	0.99869	0.998075	0.9961591
388341	C17orf76	chromosome 17 open reading frame 76	0.99458	1.032445	0.9916752
388344	RPL13P12	ribosomal protein L13 pseudogene 12	0.98142	1.017993	1.0904228
388363	RPS7P1	ribosomal protein S7 pseudogene 1	1.00203	1.021619	0.9587806
388364	TMIGD1	transmembrane and immunoglobulin domain containing 1	0.99764	1.037345	0.9736714
388381	C17orf98	chromosome 17 open reading frame 98	0.9933	1.050754	0.978155
388387	LOC388387	hypothetical LOC388387	1.01693	0.977562	0.9727029
388389	CCDC103	coiled-coil domain containing 103	0.97343	0.989675	1.0483068
388394	RPRML	reprimo-like	0.99146	1.004425	1.0874194
388401	RPL7P48	ribosomal protein L7 pseudogene 48	0.95479	1.020014	1.0392977
388403	YPEL2	yippee-like 2 (Drosophila)	0.97871	0.953588	1.0617745
388407	C17orf82	chromosome 17 open reading frame 82	0.9866	0.930705	0.9887441
388419	BTBD17	BTB (POZ) domain containing 17	1.00907	1.038771	0.9894196
388460	RPL6P22	ribosomal protein L6 pseudogene 22	0.98351	1.017868	0.9687341
388474	RPL7AP66	ribosomal protein L7a pseudogene 66	1.01173	0.998584	1.012169
388478	SDCCAG3P1	serologically defined colon cancer antigen 3 pseudogene 1	0.97869	0.981629	1.0741188
388499	LOC388499	hCG2003956	0.98849	0.970499	1.1015895
388503	C3P1	complement component 3 precursor pseudogene	0.99463	0.992422	0.9866593
388507	ZNF788	zinc finger family member 788	0.98321	0.994002	1.0033923
388514	CYP4F24P	cytochrome P450, family 4, subfamily F, polypeptide 24 pseudogene	1.00567	0.988154	0.9838107
388523	ZNF728	zinc finger protein 728	0.99481	0.981128	0.9776465
388531	RGS9BP	regulator of G protein signaling 9 binding protein	0.9656	1.035682	1.0558472
388533	KRTDAP	keratinocyte differentiation-associated protein	1.0141	0.950698	0.9791013
388536	ZNF790	zinc finger protein 790	0.9405	1.02526	0.971781
388550	CEACAM22	carcinoembryonic antigen-related cell adhesion molecule 2, pseudogene	1.00239	0.976308	1.001352
388551	CEACAM16	carcinoembryonic antigen-related cell adhesion molecule 16	0.97672	1.003814	0.990246
388552	BLOC1S3	biogenesis of lysosomal organelles complex-1, subunit 3	0.96396	0.978963	1.0837406
388553	LOC388553	hypothetical LOC388553	0.98309	1.00623	0.990007
388555	IGFL3	IGF-like family member 3	1.01001	1.003743	1.0575921

388556	RPS9P4	ribosomal protein S9 pseudogene 4	0.94309	0.980963	1.1269229
388558	ZNF808	zinc finger protein 808	0.97513	0.998489	1.049676
388559	ZNF888	zinc finger protein 888	0.92249	0.902104	1.1961183
388561	ZNF761	zinc finger protein 761	0.92751	0.9774	0.9455005
388564	LOC388564	hypothetical protein LOC388564	0.94922	0.991363	1.1001652
388566	ZNF470	zinc finger protein 470	0.97462	0.974002	1.0328441
388567	ZNF749	zinc finger protein 749	0.95253	1.028262	1.0311676
388569	ZNF324B	zinc finger protein 324B	0.96835	0.958154	1.0629113
388581	FAM132A	family with sequence similarity 132, member A	0.97799	1.042756	1.0616379
388585	HES5	hairy and enhancer of split 5 (Drosophila)	0.98531	0.956654	1.1239249
388588	LOC388588	hypothetical protein LOC388588	0.99614	0.975456	0.9977415
388591	RNF207	ring finger protein 207	1.00185	1.001265	1.0209708
388595	TMEM82	transmembrane protein 82	1.00633	0.978942	1.0870471
388610	TRNP1	TMF1-regulated nuclear protein 1	1.00978	0.990226	0.9761775
388611	CD164L2	CD164 sialomucin-like 2	0.99555	1.025191	0.9744286
388630	LOC388630	UPF0632 protein A	1.00596	1.008651	1.0320666
388646	GBP7	guanylate binding protein 7	0.99109	1.022214	1.0083823
388649	C1orf146	chromosome 1 open reading frame 146	0.97648	1.008926	1.0308681
388650	FAM69A	family with sequence similarity 69, member A	0.99805	1.021029	0.954446
388662	SLC6A17	solute carrier family 6, member 17	0.99387	0.99548	1.0185783
388695	LYSMD1	LysM, putative peptidoglycan-binding, domain containing 1	0.98613	1.003905	1.0013597
388697	HRNR	hornerin	1.0107	1.020997	0.9882156
388698	FLG2	filaggrin family member 2	1.02514	0.959568	0.988908
388701	C1orf189	chromosome 1 open reading frame 189	1.01419	0.977441	1.0001745
388720	[No Symbo	[No Name]	0.99038	1.018941	1.0334597
388722	C1orf53	chromosome 1 open reading frame 53	0.99809	1.026443	1.0453569
388730	TMEM81	transmembrane protein 81	1.01376	0.95916	1.0145049
388734	LOC388734	PRELI domain containing 1 pseudogene	0.99891	0.951627	1.1692658
388743	CAPN8	calpain 8	1.02483	0.999025	0.9735583
388753	C1orf31	chromosome 1 open reading frame 31	0.946	0.931577	1.0211835
388759	C1orf229	chromosome 1 open reading frame 229	0.98665	0.968366	1.0213503
388761	OR14A2	olfactory receptor, family 14, subfamily A, member 2	0.98291	0.985105	1.0162245
388780	LOC388780	hypothetical LOC388780	0.98315	1.079729	0.9404314

388789	LOC388789	hypothetical LOC388789	0.94219	0.983992	1.1305758
388795	EFCAB8	EF-hand calcium binding domain 8	0.98998	1.00367	0.9657606
388796	LOC388796	hypothetical LOC388796	1.02246	0.901531	1.0561534
388813	LOC388813	uncharacterized protein ENSP00000383407-like	0.9909	0.978107	0.9846567
388815	C21orf34	chromosome 21 open reading frame 34	1.00037	0.982847	1.0020343
388818	KRTAP26-1	keratin associated protein 26-1	1.01489	1.078709	1.0315807
388820	LOC388820	hypothetical LOC388820	1.00203	0.99869	0.9782076
388849	LOC388849	hypothetical protein LOC388849	0.98976	1.029569	1.0370307
388882	LOC388882	hypothetical LOC388882	1.00586	1.01431	1.0037258
388885	RPS10P29	ribosomal protein S10 pseudogene 29	0.93214	0.941951	1.1000068
388886	C22orf36	chromosome 22 open reading frame 36	1.01758	1.014869	1.0144075
388906	LOC388906	hypothetical LOC388906	0.98949	1.041296	1.0518081
388907	RPL5P34	ribosomal protein L5 pseudogene 34	0.88287	0.984193	0.9818224
388910	NCRNA002	non-protein coding RNA 207	0.99833	1.030943	1.0356608
388923	[No Symbo	[No Name]	1.01993	0.969047	1.0363935
388925	PSMC1P10	proteasome (prosome, macropain) 26S subunit, ATPase, 1 pseudogene 10	0.96942	0.995928	1.0281982
388931	MFSD2B	major facilitator superfamily domain containing 2B	0.98192	0.965847	0.979727
388939	C2orf71	chromosome 2 open reading frame 71	1.00075	0.986069	0.995382
388942	LOC388942	hypothetical LOC388942	1.00882	1.018046	1.0394502
388946	LOC388946	transmembrane protein ENSP00000343375	1.00411	1.008099	1.0016635
388948	LOC388948	hypothetical LOC388948	1.0021	1.012604	1.0309503
388951	TSPYL6	TSPY-like 6	0.98832	0.995268	1.0047934
388955	LOC388955	PRELI domain-containing protein 1, mitochondrial pseudogene	1.02797	0.871276	1.2400906
388960	C2orf78	chromosome 2 open reading frame 78	1.00439	0.953984	1.0264126
388962	BOLA3	bolA homolog 3 (E. coli)	1.00292	1.007895	1.0837518
388963	C2orf81	chromosome 2 open reading frame 81	0.98644	1.04428	1.0418469
388965	FUNDC2P2	FUN14 domain containing 2 pseudogene 2	0.98454	1.00333	1.0502511
388969	C2orf68	chromosome 2 open reading frame 68	0.98809	0.950765	1.1075047
388996	LOC388996	otopetrin 1 pseudogene	1.00974	0.93781	0.9882929
389000	NHEDC1P2	Na+/H+ exchanger domain containing 1 pseudogene 2	0.98435	1.013	1.0464809
389012	[No Symbo	[No Name]	0.99983	1.023889	0.9808214
389015	SLC9A4	solute carrier family 9 (sodium/hydrogen exchanger), member 4	1.01489	0.981915	0.9943849
389023	LOC389023	hypothetical LOC389023	1.03358	1.0818	1.0558273

389033	LOC389033	placenta-specific 9 pseudogene	1.00659	1.053734	1.0301779
389043	LOC389043	hypothetical LOC389043	0.99773	0.958093	0.983206
389049	[No Symbo	[No Name]	0.97829	1.022188	1.0226157
389053	LOC389053	heterogeneous nuclear ribonucleoprotein K pseudogene	1.04731	0.998863	1.0263029
389058	SP5	Sp5 transcription factor	0.98036	1.07963	1.0666217
389072	PLEKHM3	pleckstrin homology domain containing, family M, member 3	1.01	0.962394	0.9958433
389073	C2orf80	chromosome 2 open reading frame 80	1.0107	0.988139	1.0045253
389075	RESP18	regulated endocrine-specific protein 18 homolog (rat)	0.98887	1.008634	0.9953047
389084	C2orf82	chromosome 2 open reading frame 82	0.99579	0.984325	1.0939641
389101	RPL23AP43	ribosomal protein L23a pseudogene 43	0.98784	1.022811	0.9990889
389102	YPLR6490	hypothetical LOC389102	0.96537	0.980049	1.0438612
389114	ZNF662	zinc finger protein 662	1.00177	0.988312	0.9818917
389118	CDHR4	cadherin-related family member 4	1.01787	0.981472	1.0188628
389119	C3orf54	chromosome 3 open reading frame 54	1.00299	0.964864	1.0451782
389120	LOC389120	actin, beta pseudogene	1.02668	1.023267	1.0231966
389123	IQCF2	IQ motif containing F2	1.00379	1.013835	1.0119109
389124	IQCF5	IQ motif containing F5	0.96657	0.965672	1.0069498
389125	MUSTN1	musculoskeletal, embryonic nuclear protein 1	1.00052	0.976668	0.9929334
389127	RPS10P10	ribosomal protein S10 pseudogene 10	1.01315	1.046378	0.9682016
389136	VGLL3	vestigial like 3 (Drosophila)	1.01117	1.000736	1.0302889
389141	RPSAP29	ribosomal protein SA pseudogene 29	0.95409	1.094846	1.0952827
389144	OR7E130P	olfactory receptor, family 7, subfamily E, member 130 pseudogene	1.02701	1.02277	1.0069277
389151	PRR23B	proline rich 23B	1.00022	0.989172	1.0664145
389152	PRR23C	proline rich 23C	0.98736	0.998048	1.1010463
389158	PLSCR5	phospholipid scramblase family, member 5	1.00645	1.007575	1.0344859
389160	CPHL1P	ceruloplasmin and hephaestin-like 1 pseudogene	0.99399	1.001376	1.0273858
389161	ANKUB1	ankyrin repeat and ubiquitin domain containing 1	1.01181	0.986792	1.0026475
389170	LEKR1	leucine, glutamate and lysine rich 1	0.99741	0.970921	1.022536
389174	[No Symbo	[No Name]	1.00578	1.009599	1.0167309
389179	EEF1A1P8	eukaryotic translation elongation factor 1 alpha 1 pseudogene 8	0.94639	1.013144	1.107351
389180	5-HT3C2	5-HT3c2 serotonin receptor-like protein pseudogene	1.00793	1.027271	1.0197316
389197	C4orf50	chromosome 4 open reading frame 50	1.00296	1.025984	1.0034231
389199	LOC389199	hypothetical LOC389199	1.02043	1.049859	0.9705006

389203	C4orf52	chromosome 4 open reading frame 52	0.98421	0.979964	0.9724244
389206	BEND4	BEN domain containing 4	0.99885	0.963866	1.0064504
389207	GRXCR1	glutaredoxin, cysteine rich 1	1.01355	1.007107	1.0380996
389208	TMPRSS11I	transmembrane protease, serine 11F	1.0044	1.005009	0.9907071
389217	LOC389217	protein SET-like	0.98141	0.980558	1.0459695
389223	EEF1A1P35	eukaryotic translation elongation factor 1 alpha 1 pseudogene 35	0.95223	1.003677	0.950371
389240	NACA3P	NACA family member 3 pseudogene	1.02251	0.935519	1.0264864
389249	LOC389249	peroxiredoxin 6 pseudogene	1.01518	0.982856	1.0113749
389257	LRRC14B	leucine rich repeat containing 14B	0.96448	0.968367	0.9776054
389289	C5orf39	chromosome 5 open reading frame 39	0.96116	1.007921	0.9469351
389300	LOC389300	hypothetical protein LOC389300	1.01703	0.967231	0.9805907
389320	C5orf48	chromosome 5 open reading frame 48	0.99613	0.953219	1.0405929
389322	LOC389322	heterogeneous nuclear ribonucleoprotein K pseudogene	1.04397	1.011126	1.0462462
389328	[No Symbo	[No Name]	1.00301	1.005601	1.0332683
389332	LOC389332	hypothetical LOC389332	1.01361	1.01848	1.1044556
389333	LOC389333	hypothetical protein LOC389333	0.97487	1.015418	1.0381221
389336	C5orf46	chromosome 5 open reading frame 46	0.95976	0.965437	1.0164056
389337	ARHGEF37	Rho guanine nucleotide exchange factor (GEF) 37	1.00715	1.016894	1.0145858
389362	PSMG4	proteasome (prosome, macropain) assembly chaperone 4	0.99944	0.958448	1.0463209
389365	[No Symbo	[No Name]	0.99599	0.977311	1.0261563
389369	[No Symbo	[No Name]	1.01905	1.035343	1.0280238
389370	[No Symbo	[No Name]	0.99867	0.988963	1.0108231
389376	SFTA2	surfactant associated 2	1.02259	1.006387	1.0197144
389383	C6orf126	chromosome 6 open reading frame 126	1.00154	1.000232	1.0824349
389384	C6orf222	chromosome 6 open reading frame 222	1.00111	1.020858	1.0291587
389386	LOC389386	leucine aminopeptidase 3 pseudogene	0.99918	1.032728	1.1055298
389395	HNRNPA3P	heterogeneous nuclear ribonucleoprotein A3 pseudogene 4	0.9942	1.020507	1.0001223
389400	GFRAL	GDNF family receptor alpha like	1.01191	0.976379	1.0086949
389404	RPL9P18	ribosomal protein L9 pseudogene 18	0.99621	0.987177	1.0959983
389416	BDH2P1	3-hydroxybutyrate dehydrogenase, type 2 pseudogene 1	0.96836	0.990541	1.0685678
389419	[No Symbo	[No Name]	1.00943	1.04071	1.0581105
389421	LIN28B	lin-28 homolog B (C. elegans)	1.00624	0.990897	1.0018372
389424	PA2G4P5	proliferation-associated 2G4 pseudogene 5	0.98277	0.958024	1.0328433

389428	RPL5P18	ribosomal protein L5 pseudogene 18	1.05435	0.959045	1.0362575
389432	SAMD5	sterile alpha motif domain containing 5	1.00757	1.010809	1.0078839
389434	IYD	iodotyrosine deiodinase	1.03107	1	0.9948239
389435	RPL27AP6	ribosomal protein L27a pseudogene 6	1.00046	1.069603	1.1407599
389458	LOC389458	hypothetical LOC389458	0.97217	0.985578	0.9830635
389465	LOC389465	Sjogren syndrome antigen B pseudogene	0.99443	0.983699	1.0399332
389493	LOC389493	hypothetical protein LOC389493	1.01663	1.002676	1.0189077
389538	MGC72080	MGC72080 pseudogene	1.03809	0.928984	0.9375488
389541	C7orf59	chromosome 7 open reading frame 59	1.0036	0.99885	0.9819579
389549	FEZF1	FEZ family zinc finger 1	0.98272	1.022755	1.0349461
389558	FAM180A	family with sequence similarity 180, member A	1.00822	1.003548	1.0242878
389599	STRADBP1	STE20-related kinase adaptor beta pseudogene 1	1.00281	1.00805	1.0355321
389602	LOC389602	hypothetical LOC389602	1.01861	0.922617	0.988765
389610	XKR5	XK, Kell blood group complex subunit-related family, member 5	1.00533	1.000991	1.0174628
389626	OR7E161P	olfactory receptor, family 7, subfamily E, member 161 pseudogene	0.99416	1.025759	0.9931555
389634	LOC389634	hypothetical LOC389634	1.01966	1.051867	1.0264738
389643	C8orf80	chromosome 8 open reading frame 80	0.9918	1.061558	1.0267003
389649	C8orf86	chromosome 8 open reading frame 86	0.98227	0.968205	1.0309016
389652	ASNSP1	asparagine synthetase pseudogene 1	0.96445	1.009893	1.0011467
389655	RPL29P19	ribosomal protein L29 pseudogene 19	0.99521	1.050374	1.0934392
389658	FAM150A	family with sequence similarity 150, member A	1.00484	1.030696	0.9942461
389662	SEPT10P1	septin 10 pseudogene 1	1.00819	0.991843	0.994075
389668	XKR9	XK, Kell blood group complex subunit-related family, member 9	1.01573	0.95563	1.0091465
389672	RPSAP47	ribosomal protein SA pseudogene 47	0.94646	1.11011	1.0061412
389676	LOC389676	hypothetical LOC389676	1.00429	1.003085	1.0406833
389677	RBM12B	RNA binding motif protein 12B	0.9563	0.929122	1.0442675
389690	FLJ43860	FLJ43860 protein	0.99983	0.98467	1.0105564
389692	MAFA	v-maf musculoaponeurotic fibrosarcoma oncogene homolog A (avian)	1.00007	0.967644	1.1097353
389705	LOC389705	chromosome 4 open reading frame 27 pseudogene	0.98546	1.007059	1.0423338
389715	C9orf144	transmembrane protein C9orf144B pseudogene	0.99918	1.064484	0.943693
389762	FAM75D3	family with sequence similarity 75, member D3	1.00453	0.986692	1.0003789
389763	FAM75D1	family with sequence similarity 75, member D1	1.00212	1.045607	0.9736007
389765	LOC389765	kinesin family member 27 pseudogene	0.96738	0.996191	0.9720313

389766	C9orf153	chromosome 9 open reading frame 153	0.99117	1.023716	0.9776562
389768	LOC389768	potassium channel tetramerisation domain containing 1 pseudogene	1.00576	1.040439	1.0491318
389787	LOC389787	tumor protein, translationally-controlled 1 pseudogene	0.97101	1.041273	1.0127937
389791	LOC389791	hypothetical LOC389791	1.0114	1.027351	0.9483181
389792	IER5L	immediate early response 5-like	0.98595	1.055314	1.0500649
389799	C9orf171	chromosome 9 open reading frame 171	0.98563	0.987155	1.0001377
389812	LCN15	lipocalin 15	0.97906	1.009626	1.0457018
389813	C9orf172	chromosome 9 open reading frame 172	0.99944	0.956193	1.021457
389816	LRRC26	leucine rich repeat containing 26	1.0068	0.970556	1.1103639
389827	TMEM8C	transmembrane protein 8C	0.97402	1.009649	1.0369622
389840	MAP3K15	mitogen-activated protein kinase kinase kinase 15	0.99464	1.027669	1.0113652
389844	FTH1P19	ferritin, heavy polypeptide 1 pseudogene 19	1.00499	1.007248	0.9807291
389849	GAPDHP65	glyceraldehyde 3 phosphate dehydrogenase pseudogene 65	0.97034	1.033596	1.053192
389856	USP27X	ubiquitin specific peptidase 27, X-linked	0.97908	0.944132	1.0828931
389860	PAGE2B	P antigen family, member 2B	0.99066	0.960784	1.0418906
389866	SERBP1P1	SERPINE1 mRNA binding protein 1 pseudogene 1	0.94113	1.024399	0.9887265
389873	SEPHS1P4	selenophosphate synthetase 1 pseudogene 4	0.98939	0.98271	1.0186118
389884	PA2G4P1	proliferation-associated 2G4 pseudogene 1	0.99437	0.982103	1.0120974
389895	LOC389895	chromosome 16 open reading frame 72 pseudogene	0.98795	0.959485	1.0286154
389898	UBE2NL	ubiquitin-conjugating enzyme E2N-like	0.97193	0.951851	1.097173
389901	LOC389901	x-ray repair cross-complementing protein 6-like	0.97445	1.018522	1.0200094
389904	[No Symbo	[No Name]	0.99314	1.039574	1.0061065
389906	LOC389906	hypothetical LOC389906	0.94862	1.033065	1.0348649
389932	tAKR	aldo-keto reductase, truncated	1.00665	0.964813	0.9916978
389935	LOC389935	PRP38 pre-mRNA processing factor 38 (yeast) domain containing A pseudogene	1.00958	0.980732	1.0298202
389936	[No Symbo	[No Name]	1.01169	1.08061	0.9722509
389941	C1QL3	complement component 1, q subcomponent-like 3	0.97153	0.994919	1.0651344
389948	LOC389948	MAP kinase interacting serine/threonine kinase 2 pseudogene	0.98018	1.040961	1.0416372
389970	GAPDHP21	glyceraldehyde 3 phosphate dehydrogenase pseudogene 21	1.03304	1.063258	0.9945468
389976	LOC389976	actin, gamma 1 pseudogene	0.9803	0.951028	1.050318
389990	LOC389990	replication protein A2, 32kDa pseudogene	1.00679	1.002992	0.9585061
389992	RPL7P34	ribosomal protein L7 pseudogene 34	0.9934	1.034399	0.9968839
389997	LOC389997	nuclear import 7 homolog (S. cerevisiae) pseudogene	0.99625	0.994674	1.0081074

390003	GUCY2GP	guanylate cyclase 2G homolog (mouse), pseudogene	1.0061	1.026628	0.9723695
390006	PPIAP19	peptidylprolyl isomerase A (cyclophilin A) pseudogene 19	1.00146	1.016519	1.030371
390007	TOMM22P!	translocase of outer mitochondrial membrane 22 homolog (yeast) pseudogene 5	1.00224	1.018385	1.0143549
390010	NKX1-2	NK1 homeobox 2	1.0017	0.969505	1.0403215
390029	LOC390029	actin, beta pseudogene	0.99118	0.974903	1.0489822
390030	OR55B1P	olfactory receptor, family 55, subfamily B, member 1 pseudogene	1.03106	1.022678	0.9776319
390034	OR51R1P	olfactory receptor, family 51, subfamily R, member 1 pseudogene	1.07864	1.046591	0.9878708
390035	OR52K3P	olfactory receptor, family 52, subfamily K, member 3 pseudogene	0.99867	0.947069	0.9751012
390036	OR52K1	olfactory receptor, family 52, subfamily K, member 1	0.99249	1.045138	1.0044365
390038	OR51D1	olfactory receptor, family 51, subfamily D, member 1	0.99073	1.070351	1.0072863
390039	OR51A9P	olfactory receptor, family 51, subfamily A, member 9 pseudogene	1.03853	1.045334	0.9974903
390040	OR51F5P	olfactory receptor, family 51, subfamily F, member 5 pseudogene	1.00143	0.970192	1.0133713
390041	OR51C4P	olfactory receptor, family 51, subfamily C, member 4 pseudogene	1.00704	0.987868	0.9874394
390043	OR51F3P	olfactory receptor, family 51, subfamily F, member 3 pseudogene	1.00703	0.990704	0.9737026
390044	OR51F4P	olfactory receptor, family 51, subfamily F, member 4 pseudogene	0.97658	1.010614	0.9959507
390046	OR51A6P	olfactory receptor, family 51, subfamily A, member 6 pseudogene	0.99677	1.00886	0.9794804
390053	OR52A4	olfactory receptor, family 52, subfamily A, member 4	0.99147	1.013097	0.972119
390054	OR52A5	olfactory receptor, family 52, subfamily A, member 5	1.0257	1.012866	1.055141
390058	OR51B6	olfactory receptor, family 51, subfamily B, member 6	0.98225	1.003381	0.9952878
390059	OR51M1	olfactory receptor, family 51, subfamily M, member 1	0.99858	1.009734	1.0389256
390063	OR51I1	olfactory receptor, family 51, subfamily I, member 1	1.02966	0.910421	0.9418537
390064	OR51I2	olfactory receptor, family 51, subfamily I, member 2	1.04839	0.996117	0.9981161
390066	OR52D1	olfactory receptor, family 52, subfamily D, member 1	0.98957	0.98195	1.0340757
390067	OR52H1	olfactory receptor, family 52, subfamily H, member 1	0.97801	0.974172	1.0735931
390072	OR52N4	olfactory receptor, family 52, subfamily N, member 4	1.00115	0.992143	0.9332164
390075	OR52N5	olfactory receptor, family 52, subfamily N, member 5	1.00531	0.963481	1.0174691
390077	OR52N2	olfactory receptor, family 52, subfamily N, member 2	1.01108	0.936289	1.0255998
390078	OR52E6	olfactory receptor, family 52, subfamily E, member 6	1.00289	1.042831	1.0486681
390079	OR52E8	olfactory receptor, family 52, subfamily E, member 8	1.02492	1.000566	1.0252282
390081	OR52E4	olfactory receptor, family 52, subfamily E, member 4	1.00139	1.023451	1.0327896
390082	OR52E5	olfactory receptor, family 52, subfamily E, member 5	1.0237	1.011812	1.0338735
390083	OR56A3	olfactory receptor, family 56, subfamily A, member 3	0.99992	1.014422	0.9720354
390084	OR56A5	olfactory receptor, family 56, subfamily A, member 5	1.02134	1.047283	0.9633659

390091	OR10AB1P	olfactory receptor, family 10, subfamily AB, member 1 pseudogene	1.04659	1.018112	0.948307
390093	OR10A6	olfactory receptor, family 10, subfamily A, member 6	0.97699	0.980926	1.0261446
390098	LOC390098	MAS-related GPR, member X1 pseudogene	1.01821	0.998802	1.0161521
390099	LOC390099	MAS-related GPR, member X3 pseudogene	1.03918	1.118904	1.0083699
390101	LOC390101	mas-related G-protein coupled receptor member X3-like	1.01365	1.012021	1.0181153
390102	LOC390102	proliferating cell nuclear antigen pseudogene	1.01717	1.071263	0.9943376
390107	RPS24P15	ribosomal protein S24 pseudogene 15	1.02038	1.031379	1.0410528
390110	ACCSL	1-aminocyclopropane-1-carboxylate synthase homolog (Arabidopsis)(non-functional)-like	0.97346	1.016213	1.0371065
390113	OR4X1	olfactory receptor, family 4, subfamily X, member 1	1.02302	1.042351	0.9746185
390117	OR4A43P	olfactory receptor, family 4, subfamily A, member 43 pseudogene	1.00108	0.983208	1.046523
390136	OR4A11P	olfactory receptor, family 4, subfamily A, member 11 pseudogene	1.03119	1.05424	0.9861773
390137	OR4A9P	olfactory receptor, family 4, subfamily A, member 9 pseudogene	1.03339	1.031491	0.9902057
390138	OR4A10P	olfactory receptor, family 4, subfamily A, member 10 pseudogene	0.98889	1.072575	1.0438791
390139	OR4A17P	olfactory receptor, family 4, subfamily A, member 17 pseudogene	1.01229	0.996836	1.0485616
390142	OR5D13	olfactory receptor, family 5, subfamily D, member 13	0.98811	1.024426	1.021166
390144	OR5D16	olfactory receptor, family 5, subfamily D, member 16	0.98434	1.015751	1.0748829
390148	OR5W2	olfactory receptor, family 5, subfamily W, member 2	1.00739	0.945129	1.0053426
390154	OR5T3	olfactory receptor, family 5, subfamily T, member 3	0.96392	1.054014	1.0095498
390155	OR5T1	olfactory receptor, family 5, subfamily T, member 1	0.96601	1.025148	1.0308944
390157	OR8K1	olfactory receptor, family 8, subfamily K, member 1	1.00033	0.984717	1.0133245
390158	RPL5P29	ribosomal protein L5 pseudogene 29	0.96796	1.008854	0.9702192
390162	OR5M9	olfactory receptor, family 5, subfamily M, member 9	1.0262	1.023379	0.9958396
390166	OR5M5P	olfactory receptor, family 5, subfamily M, member 5 pseudogene	0.99431	0.968676	1.0309825
390174	OR9G1	olfactory receptor, family 9, subfamily G, member 1	0.99463	0.949899	0.9829323
390183	RPS4XP13	ribosomal protein S4X pseudogene 13	0.91793	0.986861	1.0120311
390186	OR5B10P	olfactory receptor, family 5, subfamily B, member 10 pseudogene	0.98507	1.058169	1.0533805
390190	OR5B2	olfactory receptor, family 5, subfamily B, member 2	1.00664	1.045969	1.0634229
390191	OR5B12	olfactory receptor, family 5, subfamily B, member 12	0.95708	1.027904	1.0152573
390195	OR5AN1	olfactory receptor, family 5, subfamily AN, member 1	0.99771	1.012737	0.9818012
390197	OR4D10	olfactory receptor, family 4, subfamily D, member 10	0.97638	0.961452	1.0878756
390199	OR4D9	olfactory receptor, family 4, subfamily D, member 9	1.01503	0.938093	1.0064398
390201	OR10V1	olfactory receptor, family 10, subfamily V, member 1	1.01681	0.962874	0.9390313
390203	LOC390203	probable polyprenol reductase-like	0.97965	0.996891	0.9614109

390205	LRRC10B	leucine rich repeat containing 10B	0.99582	0.970043	1.0682586
390206	RPS2P37	ribosomal protein S2 pseudogene 37	0.9908	0.988271	0.984971
390211	KRT8P26	keratin 8 pseudogene 26	0.97961	0.968214	0.9949952
390212	GPR152	G protein-coupled receptor 152	1.00054	0.996905	0.9429779
390213	DOC2GP	double C2-like domains, gamma, pseudogene	0.98206	0.994938	1.0090024
390218	IFITM9P	interferon induced transmembrane protein 9 pseudogene	0.97501	0.975782	1.0009676
390224	OR2AT2P	olfactory receptor, family 2, subfamily AT, member 2 pseudogene	0.99234	0.985083	1.0417287
390225	OR2AT1P	olfactory receptor, family 2, subfamily AT, member 1 pseudogene	0.99347	1.006248	0.9921565
390226	GUCY2E	guanylate cyclase 2E	0.99033	1.021849	0.9921868
390228	RPL7AP54	ribosomal protein L7a pseudogene 54	0.99385	1.005831	0.9882801
390239	NDUFB11P	NADH dehydrogenase (ubiquinone) 1 beta subcomplex, 11, pseudogene 1	1.01223	0.998953	1.0442971
390243	FOLR4	folate receptor 4 (delta) homolog (mouse)	1.01082	1.008142	0.9828531
390245	KDM4DL	lysine (K)-specific demethylase 4D-like	1.01464	1.030871	0.9883835
390250	LOC390250	polymerase (RNA) II (DNA directed) polypeptide E, 25kDa pseudogene	0.9569	0.96542	1.0993882
390251	LOC390251	SH3-domain GRB2-like 1 pseudogene	0.96376	1.033963	1.0559286
390255	LOC390255	DEAD (Asp-Glu-Ala-Asp) box polypeptide 39B pseudogene	1.00825	0.937946	1.0210936
390259	BSX	brain-specific homeobox	1.00288	1.011586	0.9920387
390260	OR6X1	olfactory receptor, family 6, subfamily X, member 1	0.99274	1.004468	0.9895021
390261	OR6M1	olfactory receptor, family 6, subfamily M, member 1	0.9631	1.041915	1.0537906
390265	OR10G7	olfactory receptor, family 10, subfamily G, member 7	1.00955	1.044381	0.9915396
390267	OR10N1P	olfactory receptor, family 10, subfamily N, member 1 pseudogene	1.02399	0.943531	1.060911
390269	OR8F1P	olfactory receptor, family 8, subfamily F, member 1 pseudogene	0.99388	1.017116	1.0085171
390273	OR8A2P	olfactory receptor, family 8, subfamily A, member 2 pseudogene	1.03715	0.985704	1.0371305
390274	OR8B10P	olfactory receptor, family 8, subfamily B, member 10 pseudogene	1.00806	1.000242	0.9456359
390275	OR8A1	olfactory receptor, family 8, subfamily A, member 1	1.02848	1.048479	0.9710438
390282	LOC390282	eukaryotic translation initiation factor 3, subunit F pseudogene	0.93012	1.04055	1.0439361
390283	RPL31P10	ribosomal protein L31 pseudogene 10	0.96756	0.951706	1.0479624
390284	SRP14P1	signal recognition particle 14kDa (homologous Alu RNA binding protein) pseudogene 1	0.95248	0.922222	1.0166156
390294	LOC390294	heterogeneous nuclear ribonucleoprotein A/B pseudogene	0.98505	0.979417	0.9927316
390297	PSMC1P8	proteasome (prosome, macropain) 26S subunit, ATPase, 1 pseudogene 8	0.97346	1.006381	1.0062393
390298	LOC390298	mitochondrial import inner membrane translocase subunit Tim17-B-like	0.99742	1.007473	1.0556917
390301	ST13P9	suppression of tumorigenicity 13 (colon carcinoma) (Hsp70 interacting protein) pseudogene 9	0.99529	0.973086	0.9793375
390306	LOC390306	ribosomal protein L7a pseudogene	1.01436	1.011597	0.9922447

390311	LOC390311	prohibitin pseudogene	0.97445	0.933448	1.0632609
390313	OR5BS1P	olfactory receptor, family 5, subfamily BS, member 1 pseudogene	0.99677	0.95747	0.9728037
390314	LOC390314	chromosome 20 open reading frame 27 pseudogene	1.01075	0.991042	1.0544902
390318	OR10U1P	olfactory receptor, family 10, subfamily U, member 1 pseudogene	1.00452	1.04085	0.9951369
390320	OR6C5P	olfactory receptor, family 6, subfamily C, member 5 pseudogene	0.99103	1.015948	1.0257981
390321	OR6C1	olfactory receptor, family 6, subfamily C, member 1	1.01516	0.995333	0.8907675
390322	OR6C7P	olfactory receptor, family 6, subfamily C, member 7 pseudogene	1.00226	1.05256	0.9723267
390323	OR6C75	olfactory receptor, family 6, subfamily C, member 75	1.01456	0.995978	1.0337308
390324	OR6C71P	olfactory receptor, family 6, subfamily C, member 71 pseudogene	0.99096	1.032328	1.0060425
390326	OR6C76	olfactory receptor, family 6, subfamily C, member 76	0.99717	1.078126	0.9533175
390327	OR6C70	olfactory receptor, family 6, subfamily C, member 70	1.00896	1.000425	1.0241632
390329	OR6U2P	olfactory receptor, family 6, subfamily U, member 2 pseudogene	1.02852	1.006545	1.0598463
390332	LOC390332	retinol dehydrogenase 16-like	1.00377	1.014639	0.974068
390340	LOC390340	phosducin-like protein 3-like	0.96162	0.997815	1.0347798
390352	EEF1A1P33	eukaryotic translation elongation factor 1 alpha 1 pseudogene 33	0.99402	0.950162	1.0084746
390358	LOC390358	ret finger protein-like pseudogene	1.02058	1.026526	0.9749715
390363	LOC390363	chloride intracellular channel 1 pseudogene	0.97045	1.018654	0.9891174
390378	LOC390378	peptidylprolyl isomerase A (cyclophilin A) pseudogene	0.98179	1.051993	0.947154
390394	EIF4A1P5	eukaryotic translation initiation factor 4A1 pseudogene 5	0.97706	0.987116	1.0113193
390402	AKR1B1P4	aldo-keto reductase family 1, member B1 pseudogene 4	1.02782	0.99162	0.9917247
390403	LOC390403	chromosome 9 open reading frame 6 pseudogene	0.99953	0.980121	0.9775348
390407	EIF4A1P6	eukaryotic translation initiation factor 4A1 pseudogene 6	1.00329	0.975629	1.0070425
390411	NPM1P22	nucleophosmin 1 (nucleolar phosphoprotein B23, numatrin) pseudogene 22	0.9944	1.010547	1.060517
390413	RPL7P44	ribosomal protein L7 pseudogene 44	0.94665	0.993153	1.0232499
390415	CCT5P2	chaperonin containing TCP1, subunit 5 (epsilon) pseudogene 2	0.99551	1.040208	1.04967
390418	KRT18P27	keratin 18 pseudogene 27	0.98143	0.964647	1.065364
390419	PPIAP23	peptidylprolyl isomerase A (cyclophilin A) pseudogene 23	0.9742	0.991591	1.0166452
390423	ARF4P3	ADP-ribosylation factor 4 pseudogene 3	0.97781	0.979963	0.9969501
390424	ATP5G1P5	ATP synthase, H+ transporting, mitochondrial Fo complex, subunit C1 (subunit 9) pseudogene 5	0.99039	0.956475	1.0438086
390431	OR4K2	olfactory receptor, family 4, subfamily K, member 2	1.00925	1.059003	1.0276725
390432	OR4Q2	olfactory receptor, family 4, subfamily Q, member 2 (gene/pseudogene)	0.9873	1.003911	0.9827905
390433	OR4K13	olfactory receptor, family 4, subfamily K, member 13	0.99881	1.020567	0.9784524
390435	OR4T1P	olfactory receptor, family 4, subfamily T, member 1 pseudogene	1.03102	0.977707	0.9621304

390436	OR4K17	olfactory receptor, family 4, subfamily K, member 17	1.01857	1.040864	1.0431166
390437	OR4N5	olfactory receptor, family 4, subfamily N, member 5	0.96978	0.991996	1.0511855
390438	OR11G1P	olfactory receptor, family 11, subfamily G, member 1 pseudogene	1.01936	0.997642	0.978042
390439	OR11G2	olfactory receptor, family 11, subfamily G, member 2	0.99153	1.005505	0.9810026
390440	OR11H5P	olfactory receptor, family 11, subfamily H, member 5 pseudogene	0.97235	1.00536	1.0597663
390441	OR11H7	olfactory receptor, family 11, subfamily H, member 7 (gene/pseudogene)	1.01418	0.973992	0.9862667
390442	OR11H4	olfactory receptor, family 11, subfamily H, member 4	1.00041	0.978432	1.0186655
390445	OR5AU1	olfactory receptor, family 5, subfamily AU, member 1	1.02363	1.02339	0.9812053
390472	KRT8P2	keratin 8 pseudogene 2	0.98239	0.964802	0.9758184
390482	AKR1B1P5	aldo-keto reductase family 1, member B1 pseudogene 5	1.0168	0.957263	0.9745637
390483	LOC390483	sal-like 4 (Drosophila) pseudogene	0.98908	1.022001	0.9909271
390501	LOC390501	metallophosphoesterase 1 pseudogene	1.00444	0.980032	1.0200035
390502	SERPINA2	serpin peptidase inhibitor, clade A (alpha-1 antiproteinase, antitrypsin), member 2	1.04587	0.933837	0.9347313
390503	LOC390503	adiponectin receptor 1 pseudogene	0.96999	0.989436	1.0208727
390538	OR4M2	olfactory receptor, family 4, subfamily M, member 2	0.9954	1.08332	0.9880442
390539	OR4N3P	olfactory receptor, family 4, subfamily N, member 3 pseudogene	0.95305	0.983488	1.0227425
390557	[No Symbo	[No Name]	0.97068	1.012241	1.033712
390561	LOC390561	similar to hect domain and RLD 2	0.99311	0.965993	1.0300682
390572	LOC390572	PRELI domain containing 1 pseudogene	0.97276	0.915378	1.0030605
390576	RPS15P8	ribosomal protein S15 pseudogene 8	0.9322	0.954614	1.1126983
390578	RPLP0P10	ribosomal protein, large, P0 pseudogene 10	0.93588	1.009075	1.0652104
390581	ATP5HP1	ATP synthase, H+ transporting, mitochondrial Fo complex, subunit d, pseudogene 1	0.99022	1.023797	1.0410259
390588	[No Symbo	[No Name]	1.03689	1.023645	0.9818947
390594	KBTBD13	kelch repeat and BTB (POZ) domain containing 13	0.99057	0.961794	1.0278043
390595	LOC390595	ubiquitin-associated protein 1-like	0.97745	1.017263	1.0271627
390597	LETM1P1	leucine zipper-EF-hand containing transmembrane protein 1, pseudogene 1	1.00757	1.017173	1.0159897
390598	SKOR1	SKI family transcriptional corepressor 1	0.98456	0.972994	1.0398706
390600	LOC390600	cytokine induced apoptosis inhibitor 1 pseudogene	0.99077	0.994646	1.0603456
390601	KRT8P9	keratin 8 pseudogene 9	1.00404	0.942673	0.976311
390610	KRT8P23	keratin 8 pseudogene 23	1.00026	1.011437	0.9789581
390612	RPL18P11	ribosomal protein L18 pseudogene 11	0.98569	0.994619	1.0730954
390614	LOC390614	relaxin/insulin-like family peptide receptor 3 pseudogene	0.96686	1.016129	1.0161657
390616	ANKRD34C	ankyrin repeat domain 34C	0.983	0.99735	1.0233314

390617	LOC390617	transcription factor Dp-1 pseudogene	0.97077	1.037173	1.0653679
390618	LOC390618	ribosomal protein L7-like 1 pseudogene	1.0115	0.989808	0.9799637
390634	KRT18P47	keratin 18 pseudogene 47	1.00754	1.056176	0.9833828
390637	C15orf58	chromosome 15 open reading frame 58	0.97926	0.995842	0.9944008
390638	LOC390638	Golgin subfamily A member 2-like	0.9766	0.982902	1.0474256
390643	LOC390643	phosphoglycerate mutase 1 (brain) pseudogene	0.9641	1.015395	1.0168387
390648	OR4F6	olfactory receptor, family 4, subfamily F, member 6	0.98429	1.03611	0.9733175
390649	OR4F15	olfactory receptor, family 4, subfamily F, member 15	1.00835	1.009015	1.0343733
390650	OR4F14P	olfactory receptor, family 4, subfamily F, member 14 pseudogene	0.98874	0.97093	1.023444
390651	OR4F13P	olfactory receptor, family 4, subfamily F, member 13 pseudogene	1.01995	1.001769	0.99006
390664	C1QTNF8	C1q and tumor necrosis factor related protein 8	0.96492	0.950569	1.0326399
390667	PTX4	pentraxin 4, long	0.95739	0.956649	1.034772
390671	RPL18P12	ribosomal protein L18 pseudogene 12	0.94073	0.996923	1.0495042
390730	LOC390730	prohibitin pseudogene	0.98557	0.973194	1.0319518
390732	CES1P2	carboxylesterase 1 pseudogene 2	0.98302	1.025096	0.9764967
390735	RPS15AP34	ribosomal protein S15a pseudogene 34	1.01987	0.997831	0.9349057
390738	[No Symbo	[No Name]	1.00152	0.991673	1.0128482
390739	LOC390739	c-myc binding protein pseudogene	0.97685	1.001568	0.9993363
390748	PABPN1L	poly(A) binding protein, nuclear 1-like (cytoplasmic)	1.00302	0.978425	1.0123986
390756	OR3A4	olfactory receptor, family 3, subfamily A, member 4	0.98319	1.023985	0.9520323
390760	LOC390760	protein phosphatase inhibitor 2-like	1.01521	0.962914	0.9918424
390791	LOC390791	peptidyl-prolyl cis-trans isomerase A-like	1.00135	1.006426	1.0398887
390792	KRT39	keratin 39	0.99911	1.025931	1.0065734
390800	RPL7L1P5	ribosomal protein L7-like 1 pseudogene 5	0.98451	0.99193	1.0122439
390806	LOC390806	protein FAM136A-like	1.02312	0.963432	1.0121055
390816	THA1P	threonine aldolase 1, pseudogene	1.00931	1.018218	1.0215992
390843	LOC390843	eukaryotic translation initiation factor 4A3 pseudogene	0.99758	1.009509	0.9638755
390844	ARIH2P1	ariadne homolog 2 pseudogene 1	1.01641	0.999643	1.0056672
390858	LOC390858	ACYL3 pseudogene	1.01033	0.991796	0.977051
390860	LOC390860	ribosomal protein, large, P0 pseudogene	0.96453	1.013059	1.0325169
390861	[No Symbo	[No Name]	1.00094	0.985331	0.9484007
390874	ONECUT3	one cut homeobox 3	0.99502	1.027603	1.0384099
390877	LOC390877	adenylate kinase isoenzyme 5-like	0.98081	1.007142	1.0098203

390882	OR7G2	olfactory receptor, family 7, subfamily G, member 2	1.00388	1.089004	0.9298219
390883	OR7G3	olfactory receptor, family 7, subfamily G, member 3	1.02865	0.986334	1.0002308
390886	OR7H1P	olfactory receptor, family 7, subfamily H, member 1 pseudogene	0.97208	0.970664	1.018485
390892	OR7A10	olfactory receptor, family 7, subfamily A, member 10	0.98417	0.988932	0.995314
390894	OR7A2P	olfactory receptor, family 7, subfamily A, member 2 pseudogene	1.00576	1.0057	1.0023696
390895	OR7A1P	olfactory receptor, family 7, subfamily A, member 1 pseudogene	1.00771	0.994183	1.0227222
390904	KRT18P40	keratin 18 pseudogene 40	1.01165	1.019818	0.9956236
390916	NUDT19	nudix (nucleoside diphosphate linked moiety X)-type motif 19	0.96526	0.931501	1.1397545
390924	EEF1A1P7	eukaryotic translation elongation factor 1 alpha 1 pseudogene 7	0.9496	0.996999	0.9822359
390927	ZNF793	zinc finger protein 793	0.96985	1.004251	0.9877761
390928	PAPL	iron/zinc purple acid phosphatase-like protein	0.9904	0.997469	0.9881008
390930	[No Symbo	[No Name]	0.99926	0.944817	1.031424
390933	LOC390933	protein associated with topoisomerase II homolog 1 (yeast) pseudogene	0.90225	0.957739	0.9476749
390937	LOC390937	Ets2 repressor factor-like	0.99478	0.962488	0.9981014
390940	LOC390940	hypothetical protein LOC390940	1.01158	1.006044	0.9861167
390948	LOC390948	heterogeneous nuclear ribonucleoprotein M pseudogene	1.01975	0.962823	1.0323996
390949	RPL23AP80	ribosomal protein L23a pseudogene 80	0.96308	1.037865	0.9611959
390963	ZNF818P	zinc finger protein 818, pseudogene	0.99027	1.021046	0.9697018
390980	ZNF805	zinc finger protein 805	0.89427	0.806732	0.9271084
390988	[No Symbo	[No Name]	1.00145	1.006033	1.0476024
390992	HES3	hairy and enhancer of split 3 (Drosophila)	1.01224	0.991206	1.0727565
390993	RPL7AP18	ribosomal protein L7a pseudogene 18	1.01158	0.988271	1.0032941
390996	LOC390996	cofilin-1-like	0.96485	0.948289	1.0477293
390997	LOC390997	SET binding factor 1 pseudogene	1.01093	1.027795	0.9997392
390998	LOC390998	ribosomal protein L10 pseudogene	0.9716	0.988434	1.0519374
390999	PRAMEF12	PRAME family member 12	1.02439	1.051646	0.9884057
391012	LOC391012	dynein, cytoplasmic, light polypeptide 1 pseudogene	1.00523	1.037615	1.0334559
391013	PLA2G2C	phospholipase A2, group IIC	1.01465	0.961518	1.0154887
391019	RPL29P6	ribosomal protein L29 pseudogene 6	1.00419	0.991155	1.0112782
391020	LOC391020	interferon induced transmembrane protein pseudogene	0.98873	0.98768	1.0896876
391026	LOC391026	alpha-actinin-4-like	1.01735	1.007241	1.008884
391033	MKRN8P	makorin ring finger protein 8, pseudogene	1.016	0.94068	0.9963088
391037	SKINTL	Skint-like, pseudogene	1.01	1.010507	0.9857209

391039	CFL1P2	cofilin 1 (non-muscle) pseudogene 2	1.00079	0.952718	1.0494415
391040	LOC391040	hypothetical LOC391040	1.00789	0.987239	1.0285818
391044	LOC391044	prohibitin pseudogene	1.01235	0.984384	1.0009402
391045	SLC2A3P2	solute carrier family 2 (facilitated glucose transporter), member 3 pseudogene 2	1.01235	0.978629	0.9964289
391048	LOC391048	cysteine and histidine-rich domain (CHORD)-containing 1 pseudogene	1.00307	0.977547	0.96569
391051	UOX	urate oxidase, pseudogene	1.00748	1.00009	1.015481
391053	CAPNS1P1	calpain, small subunit 1 pseudogene 1	1.02186	1.007562	1.0020598
391059	FRRS1	ferric-chelate reductase 1	1.02966	1.011307	0.9995389
391069	GAPDHP64	glyceraldehyde-3-phosphate dehydrogenase pseudogene 64	1.01503	0.989399	1.0041095
391073	LOC391073	glyceraldehyde-3-phosphate dehydrogenase pseudogene	1.00715	1.018083	1.0277958
391075	GAPDHP23	glyceraldehyde 3 phosphate dehydrogenase pseudogene 23	0.99532	0.985913	0.9995781
391076	HSD3BP1	hydroxy-delta-5-steroid dehydrogenase, 3 beta, pseudogene 1	1.00992	0.979635	0.9921591
391077	GAPDHP58	glyceraldehyde 3 phosphate dehydrogenase pseudogene 58	1.02296	1.001289	1.0380795
391081	HSD3BP5	hydroxy-delta-5-steroid dehydrogenase, 3 beta, pseudogene 5	1.012	1.015837	1.020543
391092	LOC391092	PRAME family member 17 pseudogene	1.02222	1.005257	0.9964418
391099	KRT8P28	keratin 8 pseudogene 28	1.02155	0.977516	1.0048025
391102	RPLP0P4	ribosomal protein, large, P0 pseudogene 4	0.97792	0.971311	1.0547596
391104	VHLL	von Hippel-Lindau tumor suppressor-like	1.05895	1.000806	0.9928892
391106	VDAC1P9	voltage-dependent anion channel 1 pseudogene 9	0.98989	0.97264	0.9455646
391107	OR10K2	olfactory receptor, family 10, subfamily K, member 2	1.06313	0.994309	0.9797512
391109	OR10K1	olfactory receptor, family 10, subfamily K, member 1	1.02237	0.960382	0.973692
391110	OR10R3P	olfactory receptor, family 10, subfamily R, member 3 pseudogene	1.0139	0.981493	0.9766804
391112	OR6Y1	olfactory receptor, family 6, subfamily Y, member 1	0.99945	0.968041	0.9707246
391115	OR6K5P	olfactory receptor, family 6, subfamily K, member 5 pseudogene	1.00871	1.009035	0.9677837
391117	OR10J2P	olfactory receptor, family 10, subfamily J, member 2 pseudogene	1.00267	1.022192	1.0315536
391119	OR10J7P	olfactory receptor, family 10, subfamily J, member 7 pseudogene	1.0172	1.024731	1.0481
391121	OR10J4	olfactory receptor, family 10, subfamily J, member 4 (gene/pseudogene)	1.00276	1.038339	1.0304272
391123	VSIG8	V-set and immunoglobulin domain containing 8	1.00248	1.01519	1.0477411
391124	RPL27AP2	ribosomal protein L27a pseudogene 2	0.98445	1.042654	1.0429245
391126	RPL26P12	ribosomal protein L26 pseudogene 12	0.96181	1.024771	1.0194475
391130	RPS17P6	ribosomal protein S17 pseudogene 6	0.99646	1.046159	1.0301335
391132	[No Symbo	[No Name]	1.00602	0.995165	1.0278468
391135	RPL4P3	ribosomal protein L4 pseudogene 3	0.97904	0.937369	1.0820275

391136	LOC391136	RNA binding motif protein 3 pseudogene	0.97901	1.029804	1.0380118
391137	RPS15P3	ribosomal protein S15 pseudogene 3	1.01563	1.015621	1.0066867
391139	GOT2P2	glutamic-oxaloacetic transaminase 2 pseudogene 2	1.01018	0.999382	1.0107934
391140	RPL13P7	ribosomal protein L13 pseudogene 7	1.02742	0.958442	0.9914141
391142	[No Symbo	[No Name]	1.00879	1.043042	0.9878315
391155	KRT8P29	keratin 8 pseudogene 29	0.97455	1.046722	0.956272
391158	TFDP1P	transcription factor Dp-1 pseudogene	0.99415	1.006794	0.9966201
391160	ARPC3P2	actin related protein 2/3 complex, subunit 3 pseudogene 2	1.05456	0.994112	0.9899237
391162	GAPDHP24	glyceraldehyde 3 phosphate dehydrogenase pseudogene 24	0.97659	0.961396	1.0208449
391168	RPS3AP7	ribosomal protein S3a pseudogene 7	0.96954	1.033413	1.0325672
391169	CDKN2AIPN	CDKN2A interacting protein N-terminal like pseudogene 1	0.98661	1.02505	1.0928998
391174	LOC391174	family with sequence similarity 96, member A pseudogene	0.99879	0.945617	1.0388545
391175	RPL23AP15	ribosomal protein L23a pseudogene 15	0.97849	1.04235	1.0253218
391179	KRT18P32	keratin 18 pseudogene 32	1.01673	1.008828	1.0097875
391183	RSL24D1P4	ribosomal L24 domain containing 1 pseudogene 4	1.01748	1.006821	1.0200549
391189	OR11L1	olfactory receptor, family 11, subfamily L, member 1	1.00365	1.017431	1.0925593
391194	OR2M2	olfactory receptor, family 2, subfamily M, member 2	1.01873	1.103513	0.9795201
391196	OR2M7	olfactory receptor, family 2, subfamily M, member 7	1.02754	1.061973	0.9507541
391205	[No Symbo	[No Name]	1.00961	1.047329	1.0275675
391211	OR2G6	olfactory receptor, family 2, subfamily G, member 6	1.01824	1.03753	1.0101787
391230	LOC391230	KN motif and ankyrin repeat domain-containing protein 1-like	0.99604	0.996191	1.0025562
391239	LOC391239	V-set domain containing T cell activation inhibitor 1 pseudogene	1.02467	1.012235	1.0640526
391242	HDHD1P3	haloacid dehalogenase-like hydrolase domain containing 1 pseudogene 3	1.02967	0.951235	0.9914603
391247	LOC391247	GINS complex subunit 2 (Psf2 homolog) pseudogene	0.99668	0.999583	1.0287441
391253	SPINT4	serine peptidase inhibitor, Kunitz type 4	0.9912	1.014106	0.9790338
391256	KRT18P4	keratin 18 pseudogene 4	0.96235	1.043573	0.955649
391257	SUMO1P1	SUMO1 pseudogene 1	0.97848	0.976843	0.9595186
391266	CNN2P7	calponin 2 pseudogene 7	0.98757	1.017352	0.9187563
391267	C21orf81	ankyrin repeat domain 20 family, member A3 pseudogene	1.03255	0.901697	0.932275
391271	[No Symbo	[No Name]	0.98206	1.063387	1.006466
391276	THUMPD1P	THUMP domain containing 1 pseudogene 1	0.98593	1.00495	1.059217
391279	OR7E23P	olfactory receptor, family 7, subfamily E, member 23 pseudogene	0.99468	0.990828	1.0943125
391322	LOC391322	D-dopachrome tautomerase-like	0.98904	0.984792	1.0583566

391334	LOC391334	actin, gamma 2, smooth muscle, enteric pseudogene	0.96705	0.973856	1.016012
391349	LOC391349	phosphoinositide-3-kinase, catalytic, delta polypeptide pseudogene	1.01724	1.022118	0.9882558
391356	PTRHD1	peptidyl-tRNA hydrolase domain containing 1	0.96741	0.990357	1.0860364
391358	LOC391358	tRNA methyltransferase 11-2 homolog (S. cerevisiae) pseudogene	1.00462	0.950667	1.0702875
391359	LOC391359	epithelial membrane protein 2 pseudogene	1.00549	1.055018	1.0251428
391365	SULT6B1	sulfotransferase family, cytosolic, 6B, member 1	0.99597	0.96541	1.0074217
391367	GAPDHP25	glyceraldehyde 3 phosphate dehydrogenase pseudogene 25	0.99298	0.998862	1.0554685
391378	LOC391378	heterogeneous nuclear ribonucleoprotein A1 pseudogene	1.00294	0.973406	0.9911437
391383	LOC391383	WD repeat domain 4 pseudogene	1.02203	1.034625	0.9963522
391387	RPSAP28	ribosomal protein SA pseudogene 28	1.00324	1.013878	1.0129186
391428	SEPHS1P7	selenophosphate synthetase 1 pseudogene 7	1.00333	1.015403	1.0018167
391436	LOC391436	mediator complex subunit 15 pseudogene	1.01629	0.995004	0.9568186
391448	LOC391448	mannosidase, endo-alpha-like pseudogene	0.9736	1.011149	1.0482028
391453	LOC391453	similar to hCG2007338	1	0.978434	1.0217663
391458	KRT18P46	keratin 18 pseudogene 46	1.02341	0.996246	1.0208148
391462	RPSAP24	ribosomal protein SA pseudogene 24	1.01305	1.004334	1.0278452
391465	LOC391465	STIP1 homology and U-box containing protein 1 pseudogene	1.02858	1.014815	1.0694187
391467	IMPDH1P7	IMP (inosine monophosphate) dehydrogenase 1 pseudogene 7	1.01715	1.007071	1.0691398
391470	LOC391470	adenosylhomocysteinase pseudogene	1.01611	1.029348	1.0217126
391472	LOC391472	bicaudal D homolog 1 (Drosophila) pseudogene	1.00009	1.029958	1.02347
391475	DYTN	dystrotelin	0.99157	0.987276	1.0048327
391485	KRT8P30	keratin 8 pseudogene 30	1.02229	0.963806	1.0333188
391490	RPL23AP26	ribosomal protein L23a pseudogene 26	0.99106	1.069188	1.0024408
391491	GAMTP1	guanidinoacetate N-methyltransferase pseudogene 1	1.01035	0.971484	0.9992848
391496	OR5S1P	olfactory receptor, family 5, subfamily S, member 1 pseudogene	0.9879	1.000337	1.0066834
391508	LOC391508	phosphoglycerate mutase 1 (brain) pseudogene	0.9824	0.900807	1.0477013
391518	VENTXP7	VENT homeobox pseudogene 7	1.0197	1.032191	1.0484308
391524	THRAP3P1	thyroid hormone receptor associated protein 3 pseudogene 1	1.00275	1.006144	0.990882
391526	LOC391526	immunoglobulin (CD79A) binding protein 1 pseudogene	0.97879	0.970205	1.0500856
391530	SALL4P6	sal-like 4 (Drosophila) pseudogene 6	1.01824	0.995622	0.9651049
391532	LOC391532	peptidyl-prolyl cis-trans isomerase A-like	0.99043	0.895702	1.0136924
391533	LOC391533	fms-related tyrosine kinase 1 (vascular endothelial growth factor/vascular permeability factor receptor) pseu	1.02319	1.007454	1.0226645
391538	ALDOAP1	aldolase A, fructose-bisphosphate pseudogene 1	1.03263	1.002818	1.0073333

391539	RPS25P4	ribosomal protein S25 pseudogene 4	0.98998	1.032845	1.020081
391552	AGGF1P3	angiogenic factor with G patch and FHA domains 1 pseudogene 3	0.96097	1.045003	1.0535129
391556	LOC391556	retinoblastoma binding protein 4 pseudogene	1.0028	0.965211	0.9254156
391559	LOC391559	vesicle transport through interaction with t-SNAREs homolog 1B (yeast) pseudogene	0.98642	1.033503	1.0587452
391560	RPL32P7	ribosomal protein L32 pseudogene 7	0.97357	1.001269	0.9889073
391566	LOC391566	histone H2B type 1-D-like	0.96433	0.940781	1.0162269
391578	LOC391578	MAF1 homolog (S. cerevisiae) pseudogene	1.03009	1.008083	1.0203305
391587	LOC391587	peptidyl-prolyl cis-trans isomerase A-like	1.00697	1.035282	1.0318366
391589	KRT18P34	keratin 18 pseudogene 34	1.01783	1.004036	1.0097615
391592	BZW1P1	basic leucine zipper and W2 domains 1 pseudogene 1	0.99256	0.966754	1.020299
391598	RPSAP31	ribosomal protein SA pseudogene 31	0.94503	1.007055	1.0178034
391600	LOC391600	transmembrane epididymal protein 1 pseudogene	1.04583	0.972874	0.970601
391603	LOC391603	nicotinamide nucleotide adenylyltransferase 1 pseudogene	1.00099	0.976466	1.0615335
391632	OR7E35P	olfactory receptor, family 7, subfamily E, member 35 pseudogene	1.01428	1.030999	1.0849994
391634	HSP90AB2P	heat shock protein 90kDa alpha (cytosolic), class B member 2, pseudogene	1.00222	0.976853	1.0329033
391636	LOC391636	uncharacterized protein C9orf78-like	0.99299	0.948635	1.0013484
391640	LOC391640	heterogeneous nuclear ribonucleoprotein A1-like	0.99112	0.98118	1.020993
391642	LOC391642	immunoglobulin (CD79A) binding protein 1 pseudogene pseudogene	0.97116	0.983049	1.0081479
391647	KRT18P25	keratin 18 pseudogene 25	1.01625	1.029489	1.0828126
391655	RPL17P20	ribosomal protein L17 pseudogene 20	0.99544	1.068237	1.0317741
391656	RPS15AP17	ribosomal protein S15a pseudogene 17	0.9896	0.938987	1.0747554
391657	LOC391657	dipeptidyl-peptidase 3 pseudogene	1.01579	1.003994	1.0197827
391670	LOC391670	heterogeneous nuclear ribonucleoprotein A1 pseudogene	0.97501	0.998671	1.0231977
391672	LOC391672	immunoglobulin (CD79A) binding protein 1 pseudogene	0.99411	0.967185	1.012217
391674	LOC391674	aminomethyltransferase pseudogene	1.03478	0.970334	1.0766009
391681	LOC391681	exocyst complex component 7 pseudogene	1.02266	1.017037	1.0064399
391686	LOC391686	lysophospholipase I pseudogene	0.97104	0.978384	1.0298272
391696	LOC391696	synaptic glycoprotein SC2-like	1.00979	1.01788	1.0161995
391697	LOC391697	zinc finger, SWIM-type containing 5 pseudogene	1.0119	1.011939	0.9768034
391698	LOC391698	eukaryotic translation elongation factor 1 gamma pseudogene	1.00099	0.975809	1.0320567
391701	RPS23P2	ribosomal protein S23 pseudogene 2	0.95551	1.031172	1.0213261
391706	RPS3AP18	ribosomal protein S3a pseudogene 18	1.00309	1.03861	0.9905431
391707	LOC391707	chromatin accessibility complex protein 1-like	1.0077	1.060116	1.0309707

391710	LOC391710	tripartite motif-containing 75 pseudogene	1.02032	0.954108	0.996903
391711	LOC391711	tripartite motif-containing protein 60-like	1.00313	1.079639	0.9975391
391712	TRIM61	tripartite motif containing 61	0.9975	0.981846	1.0233003
391713	LOC391713	tripartite motif-containing 75 pseudogene	1.00497	1.038896	0.99534
391714	TRIM75	tripartite motif containing 75	1.01695	1.047377	0.9607261
391718	LOC391718	tRNA splicing endonuclease 2 homolog pseudogene	1.00245	0.994382	1.0289276
391719	RPL19P8	ribosomal protein L19 pseudogene 8	0.95894	1.00867	1.0496205
391722	LOC391722	calcium-dependent protein kinase 7-like	1.00033	1.013423	1.0100346
391723	HELT	HES/HEY-like transcription factor	0.99091	1.000803	0.9852239
391738	RPL29P13	ribosomal protein L29 pseudogene 13	0.99888	1.065333	1.0576065
391739	CCT6P2	chaperonin containing TCP1, subunit 6 (zeta) pseudogene 2	1.02196	1.009055	1.0518791
391741	LOC391741	actin, beta-like 2 pseudogene	1.00399	0.934203	1.0622552
391744	LOC391744	histone H3.3 type 2-like	1.02825	0.997489	1.0429769
391765	LOC391765	histone H3.3 type 2-like	1.00646	0.980255	0.9771337
391767	LOC391767	putative TAF11-like protein ENSP00000332601-like	1.02052	1.018529	0.944476
391768	LOC391768	putative TAF11-like protein ENSP00000332601-like	1.00384	0.98015	1.1223346
391769	H3.Y	histone cluster 2, H3c pseudogene	1.00373	0.986625	0.9617931
391771	LOC391771	protein tyrosine phosphatase, non-receptor type 11 pseudogene	0.99122	0.98187	1.0505539
391774	[No Symbo	[No Name]	1.0286	1.017164	0.990585
391777	RPS4XP6	ribosomal protein S4X pseudogene 6	0.97098	1.021045	1.0712539
391798	LOC391798	karyopherin alpha 7 (importin alpha 8) pseudogene	1.00157	0.956278	1.0306437
391803	KRT18P45	keratin 18 pseudogene 45	0.986	1.000427	1.0033788
391804	RBMX2P5	RNA binding motif protein, X-linked 2 pseudogene 5	0.9876	0.991314	0.9497834
391807	[No Symbo	[No Name]	0.99565	0.962178	0.9832867
391810	[No Symbo	[No Name]	1.01845	1.011077	0.9957566
391811	POLD2P1	polymerase (DNA directed), delta 2, regulatory subunit 50kDa pseudogene 1	0.99961	1.00043	1.0052788
391813	LOC391813	zinc finger, DHHC-type containing 3 pseudogene	1.00227	0.947762	1.0555348
391819	KRT18P42	keratin 18 pseudogene 42	0.97852	0.998987	0.996852
391827	KRT18P16	keratin 18 pseudogene 16	0.98855	0.998179	1.0137399
391828	RPL28P3	ribosomal protein L28 pseudogene 3	0.92284	0.984395	1.0329092
391833	RPS10P11	ribosomal protein S10 pseudogene 11	0.98066	0.925667	1.0009149
391836	RPL10AP10	ribosomal protein L10a pseudogene 10	1.02487	0.938563	0.9821844
391844	[No Symbo	[No Name]	0.99812	0.988308	0.9430111

391845	RPS15P6	ribosomal protein S15 pseudogene 6	0.96615	1.011064	1.1564229
391847	[No Symbo	[No Name]	0.9976	0.999166	1.0173905
392005	[No Symbo	[No Name]	0.99565	1.015791	1.0359574
392008	LOC392008	similar to hCG2010255	1.02517	0.978626	1.0463071
392027	LOC392027	ribosome-binding protein 1 pseudogene	1.02219	1.001791	0.9489149
392030	[No Symbo	[No Name]	0.98937	1.036777	0.9979619
392100	PSMC1P3	proteasome (prosome, macropain) 26S subunit, ATPase, 1 pseudogene 3	0.98346	0.969084	1.0265225
392106	OR9A3P	olfactory receptor, family 9, subfamily A, member 3 pseudogene	0.96739	1.01565	1.0034452
392132	OR2R1P	olfactory receptor, family 2, subfamily R, member 1, pseudogene	0.98682	0.978601	0.9721252
392133	OR10AC1P	olfactory receptor, family 10, subfamily AC, member 1 pseudogene	1.01188	1.011008	1.0581856
392138	OR2A25	olfactory receptor, family 2, subfamily A, member 25	0.95889	1.01612	0.9774597
392140	OR2A13P	olfactory receptor, family 2, subfamily A, member 13 pseudogene	1.00991	1.044782	0.9816762
392145	LOC392145	exosome component 6 pseudogene	1.00559	1.002591	1.0405935
392180	LOC392180	transcriptional adaptor 1 pseudogene	1.02288	1.047941	0.9447114
392181	LOC392181	ribosomal protein L23a pseudogene	0.94301	0.977993	1.0258098
392194	OR7E158P	olfactory receptor, family 7, subfamily E, member 158 pseudogene	1.05649	1.087388	0.9623481
392206	RPL10AP11	ribosomal protein L10a pseudogene 11	0.96109	0.942589	1.0643143
392209	LOC392209	heat shock 70kDa protein 8 pseudogene	1.00352	0.984619	1.0593792
392217	LOC392217	ig lambda chain V region 4A-like	1.02369	1.036938	0.974906
392221	CHCHD2P1	coiled-coil-helix-coiled-coil-helix domain containing 2 pseudogene 10	1.00049	1.039777	1.0255606
392225	LOC392225	phosducin-like 3 pseudogene	0.99157	0.986502	0.9994141
392226	LOC392226	serine/threonine-protein kinase PLK1-like	0.97513	0.982078	1.0425751
392232	LOC392232	transient receptor potential cation channel, subfamily A, member 1 pseudogene	0.97371	1.000847	1.0003608
392242	REXO1L8	REX1, RNA exonuclease 1 homolog (S. cerevisiae)-like 8	0.9437	0.916731	1.0376388
392255	GDF6	growth differentiation factor 6	1.0021	1.013715	1.0208794
392262	RPSAP48	ribosomal protein SA pseudogene 48	0.99905	0.950081	0.992
392264	LOC392264	zinc finger protein 532 pseudogene	0.97007	1.016326	1.0082811
392265	LOC392265	cyclin-dependent kinase 5 pseudogene	1.00825	0.998919	1.0294053
392266	LOC392266	17beta-hydroxysteroid dehydrogenase pseudogene	1.01732	0.999833	1.0468213
392267	IMPDH1P6	IMP (inosine monophosphate) dehydrogenase 1 pseudogene 6	0.98426	1.034498	1.0243016
392268	LOC392268	neuropilin (NRP) and tolloid (TLL)-like 2 pseudogene	0.98835	1.028611	0.9966324
392271	RPL32P20	ribosomal protein L32 pseudogene 20	0.96968	1.018937	1.0198151
392275	SMPD5	sphingomyelin phosphodiesterase 5	0.9809	1.00506	1.0526192

392281	LOC392281	G protein pathway suppressor 2 pseudogene	1.05734	0.963125	0.9742717
392282	RPS5P6	ribosomal protein S5 pseudogene 6	1.02857	0.945294	1.0420362
392285	LOC392285	peptidyl-prolyl cis-trans isomerase A-like	0.97972	1.005122	1.0295176
392288	LOC392288	microtubule-associated proteins 1A/1B light chain 3B-like	1.0245	1.020931	1.0161743
392298	CTAGE12P	CTAGE family, member 12, pseudogene	0.98928	0.98208	1.036338
392301	SLC25A5P8	solute carrier family 25 (mitochondrial carrier; adenine nucleotide translocator), member 5 pseudogene 8	1.02516	0.996372	1.039793
392302	ASS1P12	argininosuccinate synthetase 1 pseudogene 12	0.98591	1.013806	0.9567406
392305	GLULP4	glutamate-ammonia ligase (glutamine synthetase) pseudogene 4	0.96792	1.039366	0.9543332
392307	C9orf128	chromosome 9 open reading frame 128	1.01309	1.021916	0.956944
392308	OR13E1P	olfactory receptor, family 13, subfamily E, member 1 pseudogene	1.01262	1.005321	1.0030624
392310	LOC392310	phosphoglycerate mutase 1 (brain) pseudogene	1.01971	0.995121	1.0355039
392313	OR2S1P	olfactory receptor, family 2, subfamily S, member 1 pseudogene	1.00799	0.976022	1.0111521
392350	BTF3P4	basic transcription factor 3 pseudogene 4	0.98542	0.969854	1.0473122
392352	LOC392352	peptidyl-prolyl cis-trans isomerase A-like	0.98368	0.985518	1.0078699
392358	RPS6P13	ribosomal protein S6 pseudogene 13	0.97241	0.995668	1.0754616
392360	CTSL3	cathepsin L family member 3	0.98592	0.974236	1.0167528
392364	LOC392364	chromosome 15 open reading frame 2 pseudogene	1.01154	0.977848	0.9720506
392368	LOC392368	BEN domain containing 3 pseudogene	1.0033	1.03279	1.0202894
392371	KRT18P13	keratin 18 pseudogene 13	0.94948	0.997633	0.9392459
392374	GAPDHP26	glyceraldehyde 3 phosphate dehydrogenase pseudogene 26	0.97866	0.982571	1.0215499
392375	OR13D2P	olfactory receptor, family 13, subfamily D, member 2 pseudogene	1.02902	0.944431	1.0175671
392377	OR13C1P	olfactory receptor, family 13, subfamily C, member 1 pseudogene	1.0205	1.039639	0.9428001
392382	RPL31P43	ribosomal protein L31 pseudogene 43	1.01219	1.078982	1.0120368
392387	LOC392387	adenosylhomocysteinase pseudogene	1.01517	0.953396	1.0628224
392391	OR5C1	olfactory receptor, family 5, subfamily C, member 1	1.00995	0.993088	1.0049408
392392	OR1K1	olfactory receptor, family 1, subfamily K, member 1	1.0101	0.991784	1.069084
392395	[No Symbo	[No Name]	0.99466	1.00514	0.9706576
392399	LCN9	lipocalin 9	1.00433	0.964371	0.9878633
392424	RPS5P8	ribosomal protein S5 pseudogene 8	0.95811	0.956862	1.0182557
392425	RPS27AP17	ribosomal protein S27a pseudogene 17	0.98798	1.0227	1.0405389
392435	LOC392435	melanoma antigen family B, 3 pseudogene	0.97155	0.931415	0.9624511
392436	LOC392436	melanoma antigen family B, 4 pseudogene	0.99711	0.998577	1.0102003
392437	FTLP2	ferritin, light polypeptide pseudogene 2	0.95804	0.926653	1.0276698

392439	LOC392439	splicing factor, arginine/serine-rich 2 pseudogene	1.00855	0.94466	1.0624789
392440	LOC392440	melanoma antigen family B, 18 pseudogene	1.01451	1.01801	0.9974295
392442	LOC392442	BCL2-associated athanogene pseudogene	1.02245	1.010674	1.0163626
392443	[No Symbol	[No Name]	0.99446	0.9837	0.9752304
392447	RPL32P36	ribosomal protein L32 pseudogene 36	1.00146	0.964828	0.992317
392451	RPL19P20	ribosomal protein L19 pseudogene 20	0.96527	1.023139	0.9899057
392452	LOC392452	mitochondrial fission factor pseudogene	0.98826	0.987063	1.000041
392454	LOC392454	proliferating cell nuclear antigen pseudogene	1.00938	1.024289	1.0484841
392456	LOC392456	catenin, beta like 1 pseudogene	0.97658	0.986539	0.9970089
392459	CXXC1P1	CXXC finger protein 1 pseudogene 1	1.00518	0.972847	0.9814815
392465	GLOD5	glyoxalase domain containing 5	0.99627	1.023514	0.984926
392473	[No Symbol	[No Name]	0.99428	1.044037	0.9972559
392479	KRT8P17	keratin 8 pseudogene 17	0.98325	1.029474	1.0253091
392481	[No Symbol	[No Name]	1.01431	1.027208	0.9865761
392485	LOC392485	ataxin 7-like 3 pseudogene	1.0174	1.03399	1.0116662
392486	GPR165P	G protein-coupled receptor 165 pseudogene	1.01679	0.9985	1.0006607
392489	LOC392489	ras homolog gene family, member G (rho G) pseudogene	0.97028	1.04238	0.9488139
392490	FLJ44635	TPT1-like protein	0.96035	0.924364	0.9810566
392497	RPS6P26	ribosomal protein S6 pseudogene 26	0.94197	1.025475	0.9758858
392505	RPL6P29	ribosomal protein L6 pseudogene 29	0.99	1.005683	1.0738693
392508	LOC392508	RAD21 homolog (S. pombe) pseudogene	0.99699	0.94328	1.0403839
392509	ARL13A	ADP-ribosylation factor-like 13A	0.9888	1.00778	1.0126663
392510	YWHAQP8	YWHAQ pseudogene 8	1.0164	1.006494	1.0103755
392516	KRT18P49	keratin 18 pseudogene 49	1.02641	1.009796	1.0380956
392520	RPS5P7	ribosomal protein S5 pseudogene 7	1.00356	1.021946	0.9823386
392522	RPL18AP15	ribosomal protein L18a pseudogene 15	0.9289	0.941992	1.1703297
392525	AKR1B1P8	aldo-keto reductase family 1, member B1 pseudogene 8	0.98989	1.014293	1.0046994
392529	LOC392529	cordon-bleu homolog (mouse) pseudogene	1.02406	1.003452	1.0378598
392536	LOC392536	filamin binding LIM protein 1 pseudogene	0.99189	1.028348	1.0513527
392538	LOC392538	RuvB-like 2 (E. coli) pseudogene	1.00289	1.01601	0.9481648
392539	LOC392539	tight junction associated protein 1 pseudogene	1.00544	0.993053	1.0098699
392540	OR11N1P	olfactory receptor, family 11, subfamily N, member 1 pseudogene	0.99518	0.993505	0.9569813
392543	LOC392543	acylglycerol kinase pseudogene	1.01584	0.97681	0.9833607

392545	HMGB3P31	high mobility group box 3 pseudogene 31	0.97702	1.009035	0.998635
392547	CROCCP1	ciliary rootlet coiled-coil, rootletin pseudogene 1	0.98181	0.965908	1.083582
392552	KRT8P6	keratin 8 pseudogene 6	0.98297	1.0526	1.0342968
392555	LOC392555	melanoma antigen family C, 2 pseudogene	1.00251	0.993949	1.0057616
392557	RPL19P21	ribosomal protein L19 pseudogene 21	0.9666	1.04672	0.9915918
392561	OR3B1P	olfactory receptor, family 3, subfamily B, member 1 pseudogene	0.98018	0.993895	1.0251008
392617	ELFN1	extracellular leucine-rich repeat and fibronectin type III domain containing 1	1.01989	1.002302	1.0124499
392636	AGMO	alkylglycerol monooxygenase	1.00127	0.998061	0.9987796
392781	RPS2P31	ribosomal protein S2 pseudogene 31	0.96538	0.981462	1.0332733
392787	LOC392787	filamin C pseudogene	0.99744	1.049546	1.028191
392843	IQCA1P1	IQ motif containing with AAA domain 1 pseudogene 1	1.00044	0.982553	1.00441
392862	GRID2IP	glutamate receptor, ionotropic, delta 2 (Grid2) interacting protein	1.00364	1.004813	1.0319577
392872	RPL12P10	ribosomal protein L12 pseudogene 10	1.00542	0.944016	1.0200951
392979	RPL18P4	ribosomal protein L18 pseudogene 4	0.97237	0.989615	1.027761
393046	OR2A5	olfactory receptor, family 2, subfamily A, member 5	1.01034	1.089918	1.0408588
393078	LOC393078	hypothetical LOC393078	1.01426	1.028234	0.9614057
394263	MUC21	mucin 21, cell surface associated	0.99762	1.005466	0.9781594
394269	BRCA1P1	BRCA1 pseudogene 1	0.98482	0.97253	1.053925
399473	SPRED3	sprouty-related, EVH1 domain containing 3	0.98354	1.041838	1.0010993
399474	TMEM200E	transmembrane protein 200B	0.97964	0.987766	1.0656546
399512	SLC25A35	solute carrier family 25, member 35	0.97113	0.931545	0.9708628
399664	MEX3D	mex-3 homolog D (C. elegans)	0.98102	0.988911	1.1475052
399665	FAM102A	family with sequence similarity 102, member A	0.94775	0.952969	0.9985335
399668	NCRNA000	non-protein coding RNA 86	0.99653	0.950851	1.0319268
399669	ZNF321P	zinc finger protein 321, pseudogene	0.95988	1.00527	1.112567
399670	RPL13AP17	ribosomal protein L13a pseudogene 17	1.02429	0.999365	1.0224276
399671	HEATR4	HEAT repeat containing 4	0.99181	1.003687	0.9924403
399687	MYO18A	myosin XVIIIA	0.97411	0.967032	0.9948052
399694	SHC4	SHC (Src homology 2 domain containing) family, member 4	1.00307	1.005781	1.0043382
399706	NCRNA002	non-protein coding RNA 200	1.02277	0.965938	0.9810142
399715	LOC399715	hypothetical LOC399715	0.9885	1.085273	0.9630332
399717	FLJ45983	hypothetical LOC399717	0.96713	0.981607	0.995443
399726	C10orf114	chromosome 10 open reading frame 114	0.97864	1.043034	1.069592

399744	LOC399744	hypothetical LOC399744	0.97276	1.023017	1.0884186
399748	LOC399748	CAP, adenylate cyclase-associated protein 1 (yeast) pseudogene	0.94355	0.852622	1.0534836
399774	PRKRIRP3	protein-kinase, interferon-inducible double stranded RNA dependent inhibitor, repressor of (P58 repressor) p	0.98282	0.948882	1.0584169
399783	LOC399783	zinc finger protein 532 pseudogene	1.00017	1.008114	1.014919
399814	C10orf120	chromosome 10 open reading frame 120	0.99845	0.998529	1.0205506
399815	LOC399815	chromosome 10 open reading frame 88 pseudogene	0.95888	0.998915	1.0232245
399818	METTL10	methyltransferase like 10	0.93568	0.899933	1.0266932
399823	FOXI2	forkhead box I2	0.98288	1.093326	1.1284298
399827	FLJ46300	FLJ46300 protein	1.01604	0.974323	0.968896
399879	C11orf55	chromosome 11 open reading frame 55	1.01157	0.948009	1.028145
399881	LOC399881	heterogeneous nuclear ribonucleoprotein K pseudogene	0.99148	0.968178	0.9983069
399886	FLJ41423	hypothetical LOC399886	0.98272	1.04804	1.0281118
399888	FAM180B	family with sequence similarity 180, member B	1.01629	1.01064	0.9789073
399909	PCNXL3	pecanex-like 3 (Drosophila)	0.98564	1.065687	1.020571
399942	LOC399942	hypothetical gene supported by AF081484; NM_006082	1.03366	0.946506	0.9657358
399947	C11orf87	chromosome 11 open reading frame 87	1.00837	1.029953	1.0316248
399948	C11orf92	chromosome 11 open reading frame 92	0.97424	0.966467	1.0230341
399949	C11orf88	chromosome 11 open reading frame 88	0.99329	0.986801	1.0146832
399950	ST13P10	suppression of tumorigenicity 13 (colon carcinoma) (Hsp70 interacting protein) pseudogene 10	0.95561	0.963029	0.9982584
399965	LOC399965	hypothetical gene supported by AK128036	0.99467	0.979263	0.9956393
399967	PATE2	prostate and testis expressed 2	0.9947	0.990183	0.993957
399968	PATE4	prostate and testis expressed 4	1.02137	0.991832	1.0163343
399978	[No Symbo	[No Name]	0.9957	0.990577	0.9507599
399979	SNX19	sorting nexin 19	0.93809	0.960011	0.9814931
399980	[No Symbo	[No Name]	1.02057	1.015218	1.0294082
399988	LOC399988	heat shock 70kDa protein 8 pseudogene	0.96978	1.009359	0.9985918
399994	HADHAP2	hydroxyacyl-CoA dehydrogenase/3-ketoacyl-CoA thiolase/enoyl-CoA hydratase (trifunctional protein), alpha	1.00608	0.98516	0.9900459
400011	SUPT16HP	suppressor of Ty 16 homolog (S. cerevisiae) pseudogene	0.96377	0.960754	1.0466551
400013	TCP1P3	t-complex 1 pseudogene 3	0.978	0.921889	1.0212418
400026	LOC400026	protein-kinase, interferon-inducible double stranded RNA dependent inhibitor, repressor of (P58 repressor) p	0.99546	0.981783	0.9978954
400027	LOC400027	hypothetical LOC400027	0.98065	0.870518	1.0463831
400036	LOC400036	DEAD (Asp-Glu-Ala-Asp) box polypeptide 23 pseudogene	0.98052	0.994252	1.0017947
400046	FLJ41278	hypothetical LOC400046	0.97943	1.030369	0.9971979

400058	MKRN9P	makorin ring finger protein 9, pseudogene	0.99075	0.998703	0.9536279
400061	LOC400061	NSA2 ribosome biogenesis homolog (S. cerevisiae) pseudogene	0.98831	1.065921	0.9353076
400064	RPS4XP1	ribosomal protein S4X pseudogene 1	0.93918	0.940918	0.9991732
400073	C12orf76	chromosome 12 open reading frame 76	1.00244	1	1.0426067
400077	[No Symbo	[No Name]	1.05976	0.999041	0.9635364
400094	SNX19P2	sorting nexin 19 pseudogene 2	1.01644	0.945474	1.0379141
400099	LOC400099	hypothetical LOC400099	0.98064	0.940937	1.0721052
400110	FLJ46358	FLJ46358 protein	0.98708	0.986303	1.025446
400120	C13orf36	chromosome 13 open reading frame 36	0.96993	1.012128	1.0334807
400126	DGKZP1	diacylglycerol kinase, zeta pseudogene 1	0.99097	0.988408	1.1196075
400129	SMARCE1P	SWI/SNF related, matrix associated, actin dependent regulator of chromatin, subfamily e, member 1 pseudog	1.03288	1.030681	1.0183642
400141	RPSAP53	ribosomal protein SA pseudogene 53	0.95689	1.035604	1.0390264
400165	C13orf35	chromosome 13 open reading frame 35	0.95073	0.996212	0.9634069
400221	FLJ22447	hypothetical LOC400221	0.98538	1.010195	0.9059864
400223	[No Symbo	[No Name]	0.96757	0.971413	0.9415078
400224	PLEKHD1	pleckstrin homology domain containing, family D (with coiled-coil domains) member 1	1.00748	1.000717	1.0123943
400236	LOC400236	hypothetical LOC400236	0.99501	1.000448	1.0521634
400258	C14orf180	chromosome 14 open reading frame 180	0.98638	0.947443	1.0228778
400347	LOC400347	REX4, RNA exonuclease 4 homolog (S. cerevisiae) pseudogene	1.02683	1.080643	1.0382923
400359	C15orf53	chromosome 15 open reading frame 53	1.02701	1.104904	1.017093
400360	C15orf54	chromosome 15 open reading frame 54	0.82598	0.878101	0.84389
400385	RPL5P3	ribosomal protein L5 pseudogene 3	0.95674	0.998938	1.0473865
400403	DNM1P9	DNM1 pseudogene 9	0.99949	1.050063	1.0013827
400406	LOC400406	ADAM metallopeptidase with thrombospondin type 1 motif, 7 pseudogene	0.99332	1.007081	1.0250098
400410	ST20	suppressor of tumorigenicity 20	0.99799	1.051385	0.9608685
400446	LOC400446	PERP, TP53 apoptosis effector pseudogene	0.99403	1.002198	1.0205209
400451	FAM174B	family with sequence similarity 174, member B	0.99371	0.998144	0.9971548
400454	UNQ9370	IFMQ9370	0.9912	0.92048	0.9638281
400456	LOC400456	hypothetical LOC400456	1.00726	1.017186	1.0069635
400499	LOC400499	hypothetical protein LOC400499	0.98969	1.038814	0.9754911
400506	C16orf88	chromosome 16 open reading frame 88	0.96581	0.97707	0.9934391
400508	NCRNA001	non-protein coding RNA 169	1.00649	1.005741	1.0483303
400509	RUNDC2B	RUN domain containing 2B	0.95894	1.07013	1.0282364

400511	FLJ45256	hypothetical LOC400511	1.01463	1.021419	0.9761582
400533	FLJ26245	hypothetical LOC400533	0.98532	0.98933	0.9917127
400535	FLJ44674	FLJ44674 protein	1.00759	1.003291	1.0179556
400539	FLJ27243	FLJ27243 protein	1.0181	1.111337	1.0046674
400555	C16orf85	chromosome 16 open reading frame 85	0.97285	0.951753	1.0129427
400566	C17orf97	chromosome 17 open reading frame 97	0.98186	1.016421	0.9787993
400569	MED11	mediator complex subunit 11	0.90747	1.001613	0.9567795
400576	FLJ45831	hypothetical FLJ45831	0.97633	1.003019	1.0128422
400591	C17orf102	chromosome 17 open reading frame 102	0.98749	0.98357	1.0171882
400593	FLJ43944	FLJ43944 protein	0.96434	0.987353	0.9747517
400617	FLJ36644	hypothetical LOC400617	0.97497	0.969423	0.9932858
400618	FLJ37644	hypothetical LOC400618	0.98364	1.035239	1.0209911
400624	FLJ45079	FLJ45079 protein	1.01017	1.024617	0.9816633
400629	TEX19	testis expressed 19	0.98903	1.008922	1.0126176
400643	LOC400643	hypothetical LOC400643	0.99317	1.00339	1.0243925
400645	RBM22P1	RNA binding motif protein 22 pseudogene 1	1.00036	0.991014	0.9926399
400650	TPMTP1	thiopurine S-methyltransferase pseudogene 1	1.01713	0.991805	0.9519772
400652	RPS3AP49	ribosomal protein S3a pseudogene 49	0.94601	0.958671	0.9542227
400657	LOC400657	hypothetical LOC400657	0.98393	0.932312	0.9717639
400658	FLJ44313	FLJ44313 protein	0.99356	1.010132	1.0081223
400661	FLJ44881	FLJ44881	1.00886	0.9843	1.0703647
400668	PRSS57	protease, serine, 57	0.9914	0.965073	1.0336078
400673	VMAC	vimentin-type intermediate filament associated coiled-coil protein	0.94332	0.942857	1.0161562
400682	LOC400682	zinc finger protein 100-like	0.9972	0.994946	0.9475141
400696	LGALS17A	lectin, galactoside-binding, soluble, 14 pseudogene	0.97097	1.001474	1.0005908
400706	LOC400706	hypothetical LOC400706	1.0276	0.91678	1.0050191
400710	FLJ26850	FLJ26850 protein	0.99264	1.049865	1.0210357
400713	ZNF880	zinc finger protein 880	0.9477	0.952391	1.0493618
400720	ZNF772	zinc finger protein 772	0.90418	0.963228	1.0580151
400745	SH2D5	SH2 domain containing 5	0.99746	1.020385	1.0460434
400746	C1orf130	chromosome 1 open reading frame 130	1.00106	1.015116	0.9738139
400750	LOC400750	heat shock 70kDa protein 5 (glucose-regulated protein, 78kDa) pseudogene	1.01267	0.993518	1.0167159
400752	LOC400752	hypothetical LOC400752	0.98638	1.005705	1.0453095

400757	C1orf141	chromosome 1 open reading frame 141	0.99454	1.001277	1.0339579
400765	FLJ35409	FLJ35409 protein	0.97931	0.963512	0.9937936
400769	LOC400769	heterogeneous nuclear ribonucleoprotein A1 pseudogene	0.98599	1.020255	1.0037076
400793	C1orf226	chromosome 1 open reading frame 226	0.99165	0.986715	0.9729266
400796	[No Symbo	[No Name]	1.01042	1.000923	1.0038009
400797	NCRNA000	non-protein coding RNA 83	0.99077	1.035739	0.968241
400798	C1orf220	chromosome 1 open reading frame 220	0.98259	0.978058	0.977378
400823	FAM177B	family with sequence similarity 177, member B	0.99629	1.022698	0.9992814
400830	DEFB132	defensin, beta 132	0.99377	0.977759	0.9971831
400831	C20orf202	chromosome 20 open reading frame 202	0.99347	1.020815	1.0445966
400836	LOC400836	SDA1 domain containing 1 pseudogene	1.00701	1.011665	1.0803211
400863	FLJ46020	FLJ46020 protein	0.9837	0.986968	0.9222564
400866	NCRNA001	non-protein coding RNA 114	1.02821	1.029463	1.0128357
400867	FLJ45139	FLJ45139 protein	1.00411	0.998949	0.9804878
400891	LOC400891	chromosome 14 open reading frame 166B pseudogene	0.98005	0.97924	0.9940258
400892	BCRP2	breakpoint cluster region pseudogene 2	0.95914	1.041551	1.0367044
400916	CHCHD10	coiled-coil-helix-coiled-coil-helix domain containing 10	0.97444	1.00064	1.1188931
400926	FLJ90680	FLJ90680 protein	0.99721	1.009861	0.9759994
400927	LOC400927	TPTE and PTEN homologous inositol lipid phosphatase pseudogene	0.97176	1.050808	1.0388233
400931	MIRLET7BH	MIRLET7B host gene (non-protein coding)	0.98479	0.990891	1.0190738
400932	FLJ46257	hypothetical LOC400932	1.00744	0.968742	0.9799777
400934	FLJ44385	hypothetical FLJ44385	1.02614	1.064368	1.0068515
400935	IL17REL	interleukin 17 receptor E-like	1.00997	0.994477	1.038073
400940	LOC400940	hypothetical LOC400940	0.99714	1.027057	1.0264932
400941	FLJ42418	hypothetical LOC400941	1.01172	1.002928	1.0100557
400943	LOC400943	hypothetical LOC400943	1.02446	1.023152	1.0086054
400945	FLJ41481	hypothetical LOC400945	1.02648	1.005724	0.9739761
400950	LOC400950	hypothetical LOC400950	1.00867	1.002306	1.0092138
400954	EML6	echinoderm microtubule associated protein like 6	1.01419	1.0177	0.9774887
400958	LOC400958	hypothetical LOC400958	0.9971	1.026397	1.0492352
400961	PAIP2B	poly(A) binding protein interacting protein 2B	1.02355	0.958949	0.9604404
400963	RPS2P17	ribosomal protein S2 pseudogene 17	1.02144	0.915959	1.0674993
400984	OR7E102P	olfactory receptor, family 7, subfamily E, member 102 pseudogene	0.93117	1.048344	0.9913902

400986	LOC400986	ankyrin repeat domain-containing protein 36C	0.94396	0.993295	0.950998
401007	NF1P8	neurofibromin 1 pseudogene 8	0.98083	0.917467	1.0691804
401010	LOC401010	nucleolar complex associated 2 homolog (S. cerevisiae) pseudogene	0.9905	1.012162	0.9912598
401018	CTAGE14P	CTAGE family, member 14, pseudogene	0.97478	1.031633	1.0076078
401021	LOC401021	hypothetical LOC401021	1.00498	1.01738	1.0122041
401024	FSIP2	fibrous sheath interacting protein 2	0.99699	0.993479	1.0099924
401027	C2orf66	chromosome 2 open reading frame 66	1.01379	1.003068	0.9696944
401036	ASB18	ankyrin repeat and SOCS box containing 18	1.01381	1.005153	1.0203348
401039	FLJ43879	FLJ43879 protein	0.99207	1.026139	0.9731458
401040	FLJ45964	hypothetical FLJ45964	0.9988	0.980638	1.0612404
401044	FLJ40712	FLJ40712 protein	0.99463	0.985014	0.9975293
401045	FLJ41327	FLJ41327 protein	0.99067	1.119423	1.0200462
401052	LOC401052	hypothetical LOC401052	0.99139	0.995571	0.9926067
401067	IQCF3	IQ motif containing F3	1.01965	0.981115	1.0228431
401074	LOC401074	hypothetical LOC401074	1.00789	1.03186	1.0427153
401076	LOC401076	CAP, adenylate cyclase-associated protein 1 (yeast) pseudogene	0.97505	0.856181	1.0024479
401081	FLJ22763	hypothetical LOC401081	1.0228	0.999147	1.0444347
401082	FLJ25363	hypothetical LOC401082	1.00334	1.033132	0.9844749
401089	C3orf72	chromosome 3 open reading frame 72	1.02775	1.014171	0.9761482
401093	LOC401093	hypothetical LOC401093	0.98479	0.923059	0.9267079
401097	LOC401097	hypothetical protein LOC401097	0.98368	0.978103	1.0572941
401098	LOC401098	hypothetical LOC401098	1.02768	0.999042	1.0180304
401105	FLJ42393	hypothetical LOC401105	1.01086	1.018766	0.942863
401114	FLJ35816	FLJ35816 protein	0.98452	0.974601	0.9923578
401115	C4orf48	chromosome 4 open reading frame 48	0.99429	1.028668	0.981395
401123	FLJ45721	hypothetical LOC401123	1.02235	1.004301	1.014847
401124	DTHD1	death domain containing 1	1.06923	1.073961	0.9132434
401127	LOC401127	WD repeat domain 5 pseudogene	0.99444	1.045868	1.04156
401137	C4orf40	chromosome 4 open reading frame 40	1.0045	0.99954	1.0303198
401138	AMTN	amelotin	0.99894	1.009403	1.0026244
401145	FAM190A	family with sequence similarity 190, member A	0.99416	1.016058	1.0045266
401152	C4orf3	chromosome 4 open reading frame 3	0.98259	0.935043	0.9568275
401166	FLJ44896	FLJ44896 protein	1.02152	0.957038	1.0167942

401172	FLJ33360	FLJ33360 protein	1.01232	1.037538	0.9944019
401180	LOC401180	hypothetical protein LOC401180	1.00135	0.999522	1.014307
401190	RGS7BP	regulator of G-protein signaling 7 binding protein	0.99842	0.99651	0.991814
401191	FLJ46010	FLJ46010 protein	1.0098	1.004867	1.0106113
401207	C5orf63	chromosome 5 open reading frame 63	0.95563	0.96053	0.9940335
401230	FLJ46552	FLJ46552 protein	0.98345	1.015803	1.0506032
401232	DKFZP686I1	hypothetical LOC401232	1.06372	1.055147	0.9447638
401233	LOC401233	HIV-1 Tat specific factor 1 pseudogene	1.0333	0.94868	1.1016039
401236	FLJ23152	hypothetical protein LOC401236	0.98232	0.987983	1.0183847
401242	LOC401242	hypothetical LOC401242	0.97256	0.968291	0.9934817
401251	C6orf26	chromosome 6 open reading frame 26	1.02229	1.062546	1.0184822
401253	C6orf227	chromosome 6 open reading frame 227	0.99937	0.993398	1.0318369
401258	RAB44	RAB44, member RAS oncogene family	1.02277	1.013826	0.9959956
401260	FLJ41649	hypothetical LOC401260	1.00816	1.014997	1.001574
401261	FLJ38717	FLJ38717 protein	0.95121	0.928844	0.8986286
401262	CRIP3	cysteine-rich protein 3	0.99371	0.988105	1.0181234
401265	KLHL31	kelch-like 31 (Drosophila)	1.01708	0.96618	1.0087631
401271	[No Symbo	[No Name]	0.99253	0.975765	0.9943703
401285	TCP10L2	t-complex 10-like 2 (mouse)	0.97063	1.028364	1.0318927
401286	LOC401286	hypothetical LOC401286	1.0092	1.015577	1.0100737
401288	NCRNA002	non-protein coding RNA 242	0.99607	1.027556	1.0603428
401296	LOC401296	hypothetical LOC401296	1.01616	1.029617	0.9835092
401303	ZNF815	zinc finger protein 815	0.97934	0.967405	0.8868336
401321	LOC401321	hypothetical LOC401321	1.01377	0.940351	1.0058791
401335	C7orf65	chromosome 7 open reading frame 65	0.99258	0.952417	1.0270055
401337	FLJ45974	hypothetical LOC401337	1.00173	1.056508	1.0522194
401388	FLJ42280	hypothetical LOC401388	1.02591	0.994412	1.0182296
401397	LOC401397	hypothetical LOC401397	1.00727	0.933123	1.0440048
401399	PRRT4	proline-rich transmembrane protein 4	1.01461	0.995864	1.0602951
401409	RAB19	RAB19, member RAS oncogene family	0.99142	0.983852	0.9668523
401431	LOC401431	hypothetical LOC401431	0.95282	0.911775	1.0304699
401433	LOC401433	hypothetical LOC401433	1.03823	1.044557	0.9819046
401459	FLJ46365	hypothetical LOC401459	0.99043	1.015195	0.9857184

401466	C8orf59	chromosome 8 open reading frame 59	0.94724	0.915891	1.0134832
401474	SAMD12	sterile alpha motif domain containing 12	0.99302	0.981422	0.9821744
401478	FLJ45872	FLJ45872 protein	0.99689	1.007939	1.0155343
401491	FLJ35024	hypothetical LOC401491	0.99177	1.023109	1.0431262
401492	FLJ41200	hypothetical LOC401492	1.00008	0.946535	1.0560028
401494	PTPLAD2	protein tyrosine phosphatase-like A domain containing 2	0.94575	0.838283	1.013902
401498	TMEM215	transmembrane protein 215	0.98299	0.997187	0.9959103
401505	TOMM5	translocase of outer mitochondrial membrane 5 homolog (yeast)	0.94868	1.007357	1.0621869
401533	LOC401533	DEAD (Asp-Glu-Ala-Asp) box polypeptide 10 pseudogene	1.0144	0.998347	0.991878
401535	C9orf170	chromosome 9 open reading frame 170	0.99447	1.023966	1.014186
401537	RPSAP49	ribosomal protein SA pseudogene 49	1.05257	1.009711	1.0454213
401541	CENPP	centromere protein P	0.98945	0.987411	0.9971938
401546	C9orf152	chromosome 9 open reading frame 152	0.99959	0.96198	0.9646432
401548	SNX30	sorting nexin family member 30	0.97969	1.059962	0.9798829
401551	WDR38	WD repeat domain 38	1.00297	0.972094	0.9707741
401554	FLJ46836	FLJ46836 protein	0.99526	1.053094	1.0434019
401557	LOC401557	hypothetical LOC401557	0.99977	1.003568	0.9958605
401562	LCNL1	lipocalin-like 1	1.00804	0.985893	1.0520847
401563	C9orf139	chromosome 9 open reading frame 139	0.98245	0.992132	1.0242191
401565	FAM166A	family with sequence similarity 166, member A	0.99099	0.995285	1.0186945
401588	LOC401588	hypothetical LOC401588	0.9294	1.024159	0.9797742
401602	LOC401602	adaptor-related protein complex 2, beta 1 subunit pseudogene	0.95777	0.947881	1.0330193
401612	MCART6	mitochondrial carrier triple repeat 6	0.96954	1.042473	0.9634705
401637	OR7E26P	olfactory receptor, family 7, subfamily E, member 26 pseudogene	0.99836	0.969257	1.0440006
401638	RPSAP10	ribosomal protein SA pseudogene 10	1.0037	1.030644	1.0219124
401646	LOC401646	guanine nucleotide-binding protein G(i) subunit alpha-2-like	1.01205	1.046982	1.0181066
401647	GOLGA7B	golgin A7 family, member B	0.9716	0.986181	0.9763529
401648	ST13P13	suppression of tumorigenicity 13 (colon carcinoma) (Hsp70 interacting protein) pseudogene 13	1.00476	0.936448	1.0218311
401662	OR51A8P	olfactory receptor, family 51, subfamily A, member 8 pseudogene	0.99935	1.053927	1.0176279
401663	OR51H1P	olfactory receptor, family 51, subfamily H, member 1 pseudogene	1.02423	1.018067	1.0138664
401664	OR51H2P	olfactory receptor, family 51, subfamily H, member 2 pseudogene	1.00655	0.95021	1.0458267
401665	OR51T1	olfactory receptor, family 51, subfamily T, member 1	0.99212	1.057399	1.075411
401675	OR56B3P	olfactory receptor, family 56, subfamily B, member 3 pseudogene	0.96893	0.947423	0.9811932

401676	LOC401676	60S ribosomal protein L13a-like	0.98388	0.999474	1.0046055
401677	LOC401677	eukaryotic translation elongation factor 1 alpha 2 pseudogene	1.01079	1.006956	1.0289216
401679	LOC401679	filamin binding LIM protein 1 pseudogene	0.98905	1.044791	0.9882179
401687	OR5J1P	olfactory receptor, family 5, subfamily J, member 1 pseudogene	0.98453	0.990415	1.0657032
401692	OR5B1P	olfactory receptor, family 5, subfamily B, member 1 pseudogene	1.01344	1.050454	0.9746508
401696	OR4D8P	olfactory receptor, family 4, subfamily D, member 8 pseudogene	1.01216	1.023115	0.983682
401703	LOC401703	splicing factor U2AF 35 kDa subunit-like	0.9532	1.030626	1.0197114
401705	[No Symbo	[No Name]	0.97443	0.98909	0.9925391
401708	GLULP3	glutamate-ammonia ligase (glutamine synthetase) pseudogene 3	0.9665	1.007168	0.8898546
401717	EEF1A1P17	eukaryotic translation elongation factor 1 alpha 1 pseudogene 17	0.96585	0.991717	1.0168932
401718	OR5BK1P	olfactory receptor, family 5, subfamily BK, member 1 pseudogene	1.04282	1.071158	1.0304739
401720	FIGNL2	fidgetin-like 2	1.00256	0.983118	1.0252326
401725	RPL6P25	ribosomal protein L6 pseudogene 25	1.0177	0.969389	1.0291478
401730	IPMKP1	inositol polyphosphate multikinase pseudogene 1	0.98331	0.96278	1.0599926
401742	OR11K2P	olfactory receptor, family 11, subfamily K, member 2 pseudogene	1.01997	0.935105	1.0244936
401744	SMARCE1P	SWI/SNF related, matrix associated, actin dependent regulator of chromatin, subfamily e, member 1 pseudog	0.97811	1.017971	1.0010034
401767	LOC401767	1-acylglycerol-3-phosphate O-acyltransferase 6 (lysophosphatidic acid acyltransferase, zeta) pseudogene	0.98877	0.993572	1.0012697
401770	LOC401770	DET1 and DDB1 associated 1 pseudogene	0.9217	1.014758	1.0783699
401777	LOC401777	splicing factor, arginine/serine-rich 13B pseudogene	1.02209	0.947948	0.9934228
401817	RPS10P22	ribosomal protein S10 pseudogene 22	0.97851	0.899653	1.0518094
401822	FDPSP9	farnesyl diphosphate synthase pseudogene 9	0.98698	0.973176	1.0675341
401825	LOC401825	MKI67 (FHA domain) interacting nucleolar phosphoprotein pseudogene	1.01549	1.017292	1.0233266
401827	MSLNL	mesothelin-like	1.00249	0.996074	1.038024
401847	LOC401847	hypothetical protein LOC401847	1.00822	0.944732	1.0515907
401863	RPL10AP12	ribosomal protein L10a pseudogene 12	0.94471	1.038611	1.0748268
401864	LOC401864	chloride intracellular channel 1 pseudogene	1.00306	0.996371	0.992592
401865	RPL7AP63	ribosomal protein L7a pseudogene 63	1.00715	0.996848	1.038733
401875	LOC401875	differentially expressed in FDCP 8 homolog (mouse) pseudogene	0.9959	0.966449	0.99963
401884	MGC57346	hypothetical LOC401884	0.94703	0.926494	0.9811868
401895	RPS15P9	ribosomal protein S15 pseudogene 9	0.98256	0.944228	1.0466951
401898	ZNF833P	zinc finger protein 833, pseudogene	1.01375	1.013064	0.9948092
401903	OR10B1P	olfactory receptor, family 10, subfamily B, member 1 pseudogene	0.99064	0.975787	1.0000377
401911	RPL29P33	ribosomal protein L29 pseudogene 33	0.98927	1.006882	1.0260826

401914	LOC401914	C-terminal binding protein 2 pseudogene	0.99132	1.069957	1.0319675
401934	RNF223	ring finger protein 223	1.00029	1.033036	1.0293146
401944	LDLRAD2	low density lipoprotein receptor class A domain containing 2	0.99617	1.051989	1.0290229
401957	LOC401957	matrin 3 pseudogene	1	0.921089	0.9976172
401959	LOC401959	voltage-dependent anion channel 2 pseudogene	1.02683	1.068536	1.0161219
401960	GAPDHP27	glyceraldehyde 3 phosphate dehydrogenase pseudogene 27	0.99673	1.017045	1.1027331
401973	OR10J6P	olfactory receptor, family 10, subfamily J, member 6 pseudogene	1.00661	1.018423	1.0602084
401974	RPS2P10	ribosomal protein S2 pseudogene 10	0.96418	1.011502	1.1205461
401975	RPS3AP10	ribosomal protein S3a pseudogene 10	0.98755	0.980281	0.9511474
401980	LOC401980	4933406M09Rik pseudogene	1.02195	1.024501	0.9985101
401981	FDPSP8	farnesyl diphosphate synthase pseudogene 8	1.0024	1.085358	1.033556
401982	AKR1B1P1	aldehyde reductase family 1, member B1 pseudogene 1	1.01574	0.956876	1.0266637
401994	OR14I1	olfactory receptor, family 14, subfamily I, member 1	1.01221	0.994878	0.9590943
402036	LOC402036	hCG1646661-like	1.02442	0.975976	0.9814455
402055	SRRD	SRR1 domain containing	0.88177	0.957069	1.0302581
402057	RPS17P16	ribosomal protein S17 pseudogene 16	0.96457	0.977904	0.9854937
402069	RPS16P2	ribosomal protein S16 pseudogene 2	1.02638	0.997929	0.9799726
402076	LOC402076	F-box and leucine-rich repeat protein 12 pseudogene	1.00265	0.982775	1.0345461
402093	LOC402093	adenosylhomocysteinase pseudogene	1.01858	1.009529	0.9575372
402096	LOC402096	uncharacterized protein C6orf106-like	1.04797	1.016921	1.0232153
402112	LOC402112	heterogeneous nuclear ribonucleoprotein A1 pseudogene	1.02491	1.039619	0.9901812
402116	LOC402116	plakophilin 4 pseudogene	0.98461	1.003921	1.031424
402117	VWC2L	von Willebrand factor C domain containing protein 2-like	0.99752	1.001215	1.0162903
402125	LOC402125	heat shock cognate 71 kDa protein-like	0.98815	0.987383	1.005662
402134	KRT8P25	keratin 8 pseudogene 25	0.99887	1.030146	0.9999572
402135	OR5K2	olfactory receptor, family 5, subfamily K, member 2	1.03674	1.011715	1.0515719
402143	LOC402143	heat shock 70kDa protein 8 pseudogene	0.9509	0.998007	1.0708727
402149	RPL28P1	ribosomal protein L28 pseudogene 1	0.9985	0.967306	1.0278585
402150	RPL8P4	ribosomal protein L8 pseudogene 4	0.96674	0.970672	1.1460623
402152	RPL7L1P8	ribosomal protein L7-like 1 pseudogene 8	1.00634	1.009528	1.0249475
402160	LOC402160	hypothetical protein LOC402160	0.99296	1.026465	1.0712112
402175	LOC402175	hypothetical gene supported by AF044957; NM_004547	0.98164	0.950342	1.0414416
402176	RPL21P44	ribosomal protein L21 pseudogene 44	1.00483	1.065801	0.9519492

402182	LOC402182	family with sequence similarity 64, member A pseudogene	0.99282	1.001276	1.0255741
402191	LOC402191	nucleolar protein 8 pseudogene	1.00066	0.974929	0.982229
402192	LOC402192	chloride channel, nucleotide-sensitive, 1A pseudogene	0.99428	1.040176	0.983841
402193	RPL5P11	ribosomal protein L5 pseudogene 11	0.95317	0.957673	1.0524849
402198	[No Symbo	[No Name]	1.01134	0.989801	1.0098536
402213	LOC402213	peptidyl-prolyl cis-trans isomerase A-like	1.01062	1.025566	1.0817117
402214	RPL13AP13	ribosomal protein L13a pseudogene 13	1.00124	1.031041	1.1375896
402216	LOC402216	heterogeneous nuclear ribonucleoprotein H1 (H) pseudogene	1.01578	0.97295	0.9668258
402219	[No Symbo	[No Name]	0.9823	1.12562	0.9903549
402220	YTHDF1P1	YTH domain family, member 1 pseudogene 1	1.00426	1.057968	1.0389156
402221	LOC402221	actin, beta pseudogene	0.97306	0.956222	1.0339393
402222	DDX18P4	DEAD (Asp-Glu-Ala-Asp) box polypeptide 18 pseudogene 4	0.9947	0.97619	1.0142272
402226	CCT5P1	chaperonin containing TCP1, subunit 5 (epsilon) pseudogene 1	1.00936	1.002828	1.0145549
402229	LOC402229	splicing factor 4 pseudogene	0.98396	0.987369	1.0175311
402230	LOC402230	small nuclear ribonucleoprotein 27kDa (U4/U6.U5) pseudogene	1.02087	0.927314	1.0192884
402233	[No Symbo	[No Name]	1.00559	1.014621	0.9726088
402246	OR10AH1P	olfactory receptor, family 10, subfamily AH, member 1 pseudogene	0.99004	1.042202	1.0766388
402269	LOC402269	hypothetical protein LOC402269	0.97708	1.013555	1.0640707
402273	VN1R37P	vomeronasal 1 receptor 37 pseudogene	1.00845	1.013402	0.9676859
402333	OR7E160P	olfactory receptor, family 7, subfamily E, member 160 pseudogene	0.99025	0.986415	1.1023086
402342	RPL13AP18	ribosomal protein L13a pseudogene 18	0.97026	1.031021	1.0873639
402359	KHSRPP1	KH-type splicing regulatory protein pseudogene 1	0.98609	0.998539	1.0295625
402360	LOC402360	NOP56 ribonucleoprotein homolog (yeast) pseudogene	1.01474	0.934343	0.9672345
402374	OR13D3P	olfactory receptor, family 13, subfamily D, member 3 pseudogene	0.98679	0.970995	0.9675745
402375	LOC402375	nuclease-sensitive element-binding protein 1-like	0.99116	0.989024	1.0493673
402381	SOHLH1	spermatogenesis and oogenesis specific basic helix-loop-helix 1	1.00784	0.980852	1.0304401
402394	IMPDH1P2	IMP (inosine monophosphate) dehydrogenase 1 pseudogene 2	1.00246	1.04362	1.038598
402408	LOC402408	pyruvate kinase, muscle pseudogene	1.00576	0.975206	1.004095
402415	XKRX	XK, Kell blood group complex subunit-related, X-linked	0.98429	0.967414	0.964653
402420	EEF1A1P30	eukaryotic translation elongation factor 1 alpha 1 pseudogene 30	0.98005	0.947551	1.0422304
402422	LOC402422	tubulin, beta 4 pseudogene	0.9917	0.979949	0.9864309
402424	OR13K1P	olfactory receptor, family 13, subfamily K, member 1 pseudogene	0.99725	1.03531	1.0216537
402425	LOC402425	5'-nucleotidase domain containing 1 pseudogene	0.99984	1.018745	0.9979615

402429	KRT8P8	keratin 8 pseudogene 8	1.01059	0.976024	1.0243923
402481	PP13004	hypothetical LOC402481	0.99041	0.995399	1.0093287
402562	HNRNPA1P	heterogeneous nuclear ribonucleoprotein A1 pseudogene 8	1.00367	0.959843	0.9756666
402569	KPNA7	karyopherin alpha 7 (importin alpha 8)	1.00789	0.985015	0.9947444
402573	C7orf61	chromosome 7 open reading frame 61	1.01755	1.02082	0.9699821
402634	LOC402634	neugrin, neurite outgrowth associated-like pseudogene	0.96587	0.985783	1.0556176
402635	GRIFIN	galectin-related inter-fiber protein	1.00393	0.995701	0.9964739
402641	LOC402641	v-ral simian leukemia viral oncogene homolog A (ras related) pseudogene	0.98144	0.978334	1.0293457
402643	TPM3P4	tropomyosin 3 pseudogene 4	0.99835	0.989288	0.9462234
402644	LOC402644	peptidylprolyl isomerase A (cyclophilin A) pseudogene	0.99878	1	1.0166323
402665	IGLON5	IgLON family member 5	0.97727	1.011653	1.021585
402679	MARK2P10	MAP/microtubule affinity-regulating kinase 2 pseudogene 10	1.00619	0.990171	1.0220848
402682	UFSP1	UFM1-specific peptidase 1 (non-functional)	1.05176	1.083353	0.9847454
402694	RPL15P11	ribosomal protein L15 pseudogene 11	1.01788	0.966584	1.034331
402715	LOC402715	karyopherin alpha-2 pseudogene	1.00679	0.976546	1.0352261
402716	RPL32P17	ribosomal protein L32 pseudogene 17	1.01271	1.072797	1.0458115
403227	OR11Q1P	olfactory receptor, family 11, subfamily Q, member 1 pseudogene	1.00962	0.938585	1.0201549
403235	OR2AO1P	olfactory receptor, family 2, subfamily AO, member 1 pseudogene	1.00168	0.980112	1.0862571
403247	OR4A41P	olfactory receptor, family 4, subfamily A, member 41 pseudogene	0.98791	0.979312	1.013514
403248	OR4A42P	olfactory receptor, family 4, subfamily A, member 42 pseudogene	1.02018	0.977978	1.0113024
403251	OR4A45P	olfactory receptor, family 4, subfamily A, member 45 pseudogene	0.97357	1.010504	1.0063993
403256	OR4A50P	olfactory receptor, family 4, subfamily A, member 50 pseudogene	0.96381	0.978956	1.0260589
403266	OR51B8P	olfactory receptor, family 51, subfamily B, member 8 pseudogene	1.01214	1.052081	0.9758948
403271	OR5AC4P	olfactory receptor, family 5, subfamily AC, member 4 pseudogene	0.99239	1.009282	0.9962341
403274	OR5H15	olfactory receptor, family 5, subfamily H, member 15	1.03168	1.029572	1.072834
403275	OR9S24P	olfactory receptor, family 9, subfamily S, member 24 pseudogene	1.00087	0.977029	0.9352084
403277	OR5K3	olfactory receptor, family 5, subfamily K, member 3	1.01587	0.982503	0.9516603
403278	OR5K4	olfactory receptor, family 5, subfamily K, member 4	1.00249	1.000266	1.0048105
403281	OR6C64P	olfactory receptor, family 6, subfamily C, member 64 pseudogene	1.01286	0.998519	1.0249761
403282	OR6C65	olfactory receptor, family 6, subfamily C, member 65	1.00867	0.991904	0.9542338
403283	OR6C66P	olfactory receptor, family 6, subfamily C, member 66 pseudogene	1.00669	0.992502	1.0349716
403284	OR6C68	olfactory receptor, family 6, subfamily C, member 68	1.00074	1.024931	1.0288353
403285	OR6C69P	olfactory receptor, family 6, subfamily C, member 69 pseudogene	0.98546	1.096117	0.9791601

403288	OR6C72P	olfactory receptor, family 6, subfamily C, member 72 pseudogene	0.97523	1.048338	1.095686
403297	OR7E155P	olfactory receptor, family 7, subfamily E, member 155 pseudogene	1.0572	0.993737	0.9359685
403301	OR7H2P	olfactory receptor, family 7, subfamily H, member 2 pseudogene	1.00066	0.96107	1.0034583
403302	OR8A3P	olfactory receptor, family 8, subfamily A, member 3 pseudogene	1.01711	1.021792	1.0932983
403312	MGC39545	hypothetical LOC403312	0.98886	0.990395	1.036599
403313	PPAPDC2	phosphatidic acid phosphatase type 2 domain containing 2	0.93372	0.897908	0.964587
403314	APOBEC4	apolipoprotein B mRNA editing enzyme, catalytic polypeptide-like 4 (putative)	1.00973	1.005081	1.0166012
403315	FAM92A3	family with sequence similarity 92, member A3	0.97586	0.971964	0.9914823
403341	ZBTB34	zinc finger and BTB domain containing 34	0.98751	0.957299	1.0186066
404037	HAPLN4	hyaluronan and proteoglycan link protein 4	0.9888	0.975018	1.0067193
404093	CUEDC1	CUE domain containing 1	0.99518	0.968095	1.0154767
404201	NCRNA002	non-protein coding RNA 247	1.00331	1.054932	1.0100613
404203	SPINK6	serine peptidase inhibitor, Kazal type 6	1.01812	0.997406	0.9729684
404216	C10orf85	chromosome 10 open reading frame 85	0.99825	1.022107	0.9725709
404217	CTXN1	cortexin 1	0.97568	1.021881	1.0836851
404220	C6orf201	chromosome 6 open reading frame 201	1.00078	1.007803	1.0121439
404266	LOC40426€	hypothetical LOC404266	0.9713	1.012636	1.0222387
404544	COX8BP	cytochrome c oxidase subunit VIIIB, pseudogene	0.9944	0.96565	1.0166118
404550	C16orf74	chromosome 16 open reading frame 74	0.97499	0.990417	1.00792
404552	SCGB1D4	secretoglobin, family 1D, member 4	1.00756	0.993824	1.0129448
404635	NANOGP1	Nanog homeobox pseudogene 1	0.96202	0.96974	1.0065842
404636	FAM45A	family with sequence similarity 45, member A	1.01409	1.006771	1.0227351
404665	C19orf29O:	chromosome 19 open reading frame 29 opposite strand	1.01215	0.97984	1.0112116
404672	GTF2H5	general transcription factor IIH, polypeptide 5	0.95358	1.001415	1.0080718
404734	ANKHD1-EI	ANKHD1-EIF4EBP3 readthrough	1.0307	0.988783	0.9355066
404770	FAM75B	family with sequence similarity 75, member B	0.96146	1.016704	1.0413923
405753	DUOXA2	dual oxidase maturation factor 2	0.99018	1.002543	1.0502809
405754	ERVFRD-1	endogenous retrovirus group FRD, member 1	1.02676	1.053933	1.0162981
406875	FXYD8	FXYD domain containing ion transport regulator 8	1.02436	0.986783	0.9877697
406884	MIRLET7B	microRNA let-7b	1.00946	1.000818	0.9774492
406886	MIRLET7D	microRNA let-7d	1.02194	0.990759	0.9916847
406891	MIRLET7I	microRNA let-7i	0.94434	0.953538	0.8982617
406893	MIR101-1	microRNA 101-1	1.0018	0.956111	0.9571845

406902	MIR10A	microRNA 10a	0.98304	1.021685	0.9544336
406903	MIR10B	microRNA 10b	1.01771	0.998746	1.0693031
406906	MIR122	microRNA 122	0.99826	0.976815	0.9917468
406907	MIR124-1	microRNA 124-1	1.00833	0.959135	0.9718395
406911	MIR125B1	microRNA 125b-1	0.98542	0.979807	0.9575893
406914	MIR127	microRNA 127	1.0023	0.988238	1.0027493
406919	MIR130A	microRNA 130a	1.01897	0.942845	0.9973901
406922	MIR133A1	microRNA 133a-1	1.00262	0.960866	0.979688
406925	MIR135A1	microRNA 135a-1	0.98968	1.033657	1.023806
406926	MIR135A2	microRNA 135a-2	0.99258	1.01567	0.9693023
406928	MIR137	microRNA 137	0.98785	0.991612	1.0324228
406929	MIR138-1	microRNA 138-1	0.97624	1.05917	1.0406701
406930	MIR138-2	microRNA 138-2	0.98153	1.022414	1.0734431
406934	MIR142	microRNA 142	1.04696	0.955511	0.9857833
406940	MIR148A	microRNA 148a	1.03729	1.078062	0.9717495
406957	MIR181C	microRNA 181c	1.00241	0.998275	1.0497589
406958	MIR182	microRNA 182	0.97603	1.006826	0.9988187
406960	MIR184	microRNA 184	0.97022	1.027706	0.9765474
406963	MIR187	microRNA 187	0.97155	1.038753	0.9803258
406968	MIR193A	microRNA 193a	0.9678	1.068671	0.979816
406969	MIR194-1	microRNA 194-1	0.97712	1.010641	1.0623282
406970	MIR194-2	microRNA 194-2	1.01734	0.983074	0.9339269
406973	MIR196A2	microRNA 196a-2	1.00842	0.986201	0.9026047
406974	MIR197	microRNA 197	1.02182	0.94474	1.0050611
406984	MIR200B	microRNA 200b	1.0187	1.006511	1.0197079
406986	MIR203	microRNA 203	0.98457	1.01724	1.1147699
406989	MIR206	microRNA 206	0.99363	0.932073	1.0324395
406992	MIR210	microRNA 210	0.98737	0.996355	0.9878203
407003	MIR219-2	microRNA 219-2	1.01885	1.033833	0.988008
407007	MIR222	microRNA 222	1.00152	0.969975	0.9305075
407008	MIR223	microRNA 223	1.04654	1.079705	1.0553955
407023	MIR299	microRNA 299	0.98922	0.966469	0.9761728
407024	MIR29B1	microRNA 29b-1	0.94464	0.97812	0.9770594

407025	MIR29B2	microRNA 29b-2	0.99612	0.937433	0.8731134
407033	MIR30D	microRNA 30d	1.02749	1.063585	1.0366559
407035	MIR31	microRNA 31	0.99985	0.976194	1.0067644
407037	MIR320A	microRNA 320a	0.95954	0.936135	0.9944346
407040	MIR34A	microRNA 34a	1.00734	1.03715	1.0281169
407044	MIR7-2	microRNA 7-2	0.98495	0.996804	0.9395502
407051	MIR9-3	microRNA 9-3	0.98812	1.033999	1.0613832
407738	FAM19A1	family with sequence similarity 19 (chemokine (C-C motif)-like), member A1	1.00605	0.933479	0.9616313
407835	LOC407835	mitogen-activated protein kinase kinase 2 pseudogene	0.97497	1.024349	1.0474532
407975	MIR17HG	miR-17-92 cluster host gene (non-protein coding)	0.96243	1.026743	0.930182
408029	C2orf27B	chromosome 2 open reading frame 27B	1.0281	1.008058	1.0009603
408186	OVOS	ovostatin	0.99633	0.909864	1.0025428
408187	SPINK14	serine peptidase inhibitor, Kazal type 14 (putative)	1.01746	0.955966	0.9897046
408263	FNDC9	fibronectin type III domain containing 9	0.97448	1.041924	0.9867193
414061	DNAJB3	DnaJ (Hsp40) homolog, subfamily B, member 3	1.00056	0.995613	0.9756722
414131	NANOGP2	Nanog homeobox pseudogene 2	1.07204	1.062107	0.9589541
414149	ACBD7	acyl-CoA binding domain containing 7	0.98914	1.006372	1.0033925
414152	C10orf105	chromosome 10 open reading frame 105	1.00294	1.045557	0.9853167
414194	CCNYL2	cyclin Y-like 2	0.9974	0.99258	0.9782257
414196	C10orf31	chromosome 10 open reading frame 31	1.01017	0.957154	0.9459035
414235	C10orf108	chromosome 10 open reading frame 108	1.00828	0.955936	0.9789568
414236	C10orf55	chromosome 10 open reading frame 55	0.99894	1.001879	0.9816349
414260	C10orf136	chromosome 10 open reading frame 136	0.96644	1.036508	0.986285
414301	DDI1	DNA-damage inducible 1 homolog 1 (S. cerevisiae)	0.98612	0.974933	1.0451131
414318	C9orf106	chromosome 9 open reading frame 106	1.03849	0.979838	0.9672494
414328	C9orf103	chromosome 9 open reading frame 103	0.94448	1.001308	1.0096975
414332	LCN10	lipocalin 10	0.98022	1.004941	1.0311148
414355	VKORC1P1	VKORC1 pseudogene 1	0.97675	1.007084	0.9725188
414899	BLID	BH3-like motif containing, cell death inducer	0.99008	0.998899	0.9855714
414918	FAM116B	family with sequence similarity 116, member B	0.98371	1.019174	1.0539227
414919	C8orf82	chromosome 8 open reading frame 82	1.00873	0.956485	1.0914552
414927	MGC34796	SPR pseudogene	1.01582	1.025166	0.9744482
415116	PIM3	pim-3 oncogene	0.98362	1.056113	0.9488593

415117	STX19	syntaxin 19	0.99819	1.059043	1.0468575
431704	RGS21	regulator of G-protein signaling 21	1.00401	1.000338	1.0195975
431705	ASTL	astacin-like metallo-endopeptidase (M12 family)	1.01782	1.065764	0.9632622
431707	LHX8	LIM homeobox 8	0.99853	1.012549	1.0353515
439915	KRTAP5-5	keratin associated protein 5-5	1.05347	1.037649	1.0023923
439916	C12orf37	chromosome 12 open reading frame 37	1.02065	1.001645	0.9945043
439921	MXRA7	matrix-remodelling associated 7	0.98897	1.045575	1.0023038
439933	MGC42157	hypothetical locus MGC42157	0.97949	1.071865	1.0298365
439945	IL9RP2	interleukin 9 receptor pseudogene 2	0.98987	1.031415	0.9844396
439949	LOC439949	hypothetical LOC439949	0.86527	0.871588	0.9154942
439950	LOC439950	hypothetical LOC439950	1.00203	1.052083	0.9151745
439951	LOC439951	hypothetical LOC439951	0.99319	0.956628	1.0701997
439954	RPL7P37	ribosomal protein L7 pseudogene 37	0.98887	0.981489	0.9454603
439985	[No Symbo	[No Name]	0.9823	1.049242	0.9680916
439992	RPS3AP5	ribosomal protein S3A pseudogene 5	0.86136	0.95453	1.0485057
439996	IFIT1B	interferon-induced protein with tetratricopeptide repeats 1B	1.01364	0.898119	1.0150843
440015	[No Symbo	[No Name]	0.99587	0.958509	1.0015131
440021	KRTAP5-2	keratin associated protein 5-2	1.05075	0.971533	1.0201452
440023	KRTAP5-6	keratin associated protein 5-6	1.00791	0.973893	0.9108288
440026	TMEM41B	transmembrane protein 41B	0.96692	0.955803	0.945594
440031	LOC440031	hypothetical LOC440031	0.98184	0.941683	0.9967156
440040	LOC440040	glutamate receptor, metabotropic 5 pseudogene	0.99715	0.998858	1.032062
440044	SLC22A20	solute carrier family 22, member 20	1.01014	1.020556	1.0257218
440049	FLJ42258	FLJ42258 protein	1.00719	1.013044	0.9584517
440051	KRTAP5-11	keratin associated protein 5-11	1.01761	1.105874	1.0232305
440063	LOC440063	peptidyl-prolyl cis-trans isomerase A-like	0.95353	0.883032	1.0996098
440067	LOC440067	caspase-1-like	0.93003	0.896622	0.9865268
440068	CARD17	caspase recruitment domain family, member 17	1.00547	1.048081	0.9669938
440072	NCRNA001	non-protein coding RNA 167	0.98692	0.991975	0.926278
440073	IQSEC3	IQ motif and Sec7 domain 3	0.97853	0.998634	1.0165746
440077	ZNF705A	zinc finger protein 705A	0.97142	0.965862	0.9784345
440081	DDX12	DEAD/H (Asp-Glu-Ala-Asp/His) box polypeptide 12	0.97923	0.970382	1.0369341
440084	LOC440084	hCG1655019	1.01211	0.989497	1.0511801

440086	RPS6P1	ribosomal protein S6 pseudogene 1	0.96337	1.020777	1.0305952
440087	C12orf69	chromosome 12 open reading frame 69	1.01903	0.994893	0.9663125
440097	DBX2	developing brain homeobox 2	0.98614	0.988245	1.0871748
440104	LOC440104	1110012D08Rik pseudogene	0.97536	0.948923	0.9427531
440107	PLEKHG7	pleckstrin homology domain containing, family G (with RhoGef domain) member 7	0.99308	1.007683	0.9965961
440119	FLJ31485	hypothetical LOC440119	0.99799	1.0313	1.0593663
440122	ZNF891	zinc finger protein 891	0.94513	1.012346	0.950272
440125	HNRNPA1P	heterogeneous nuclear ribonucleoprotein A1 pseudogene 30	1.01814	1.001754	1.0267663
440132	LOC440132	hCG1815504	1.00596	1.000613	1.0156382
440137	FLJ31945	hypothetical LOC440137	1.01053	0.986422	1.0005829
440138	ALG11	asparagine-linked glycosylation 11, alpha-1,2-mannosyltransferase homolog (yeast)	0.94767	0.912636	1.0691847
440142	TARDBPP2	TAR DNA binding protein pseudogene 2	1.02754	0.981974	1.0312539
440145	MZT1	mitotic spindle organizing protein 1	0.91854	0.999532	1.0269058
440163	RNASE13	ribonuclease, RNase A family, 13 (non-active)	0.98282	1.00273	1.0060204
440180	LOC440180	zinc finger, CCHC domain containing 7 pseudogene	0.98706	1.004871	1.0026242
440181	LOC440181	transmembrane protein 98 pseudogene	0.9931	0.993168	1.0231144
440184	NCRNA002	non-protein coding RNA 238	0.98467	0.972405	1.0076536
440193	CCDC88C	coiled-coil domain containing 88C	0.95539	1.0267	1.0023454
440200	FLJ41170	hypothetical LOC440200	1.00314	1.028947	0.9876543
440225	NF1P1	neurofibromin 1 pseudogene 1	0.99903	1	1.0105497
440253	WHAMML2	WAS protein homolog associated with actin, golgi membranes and microtubules-like 2 (pseudogene)	0.98104	0.928749	1.1474658
440268	TMCO5B	transmembrane and coiled-coil domains 5B, pseudogene	1.00247	0.990745	1.0510962
440275	EIF2AK4	eukaryotic translation initiation factor 2 alpha kinase 4	0.94992	0.957187	1.0175748
440278	CATSPER2P	cation channel, sperm associated 2 pseudogene 1	0.96087	1.030499	0.9521102
440279	UNC13C	unc-13 homolog C (C. elegans)	0.9977	1.005175	1.0287199
440287	[No Symbo	[No Name]	1.01193	0.985918	0.996483
440288	LOC440288	similar to FLJ16518 protein	1.0017	0.987742	1.0258521
440292	LOC440292	hypothetical protein LOC440292	1.00224	1.01645	0.9792451
440307	TTLL13	tubulin tyrosine ligase-like family, member 13	1.0015	0.999547	0.9686664
440311	LOC440311	glioma tumor suppressor candidate region gene 2 pseudogene	0.97112	0.963716	0.9898285
440330	LOC440330	hypothetical protein LOC440330	0.98579	0.989397	0.9991334
440335	LOC440335	hypothetical LOC440335	0.9986	0.92399	0.976665
440338	[No Symbo	[No Name]	1.00016	0.996873	1.0619756

440356	LOC440356	hypothetical LOC440356	1.02742	0.990303	1.029935
440359	CSDAP1	cold shock domain protein A pseudogene 1	0.98027	0.954647	1.1295364
440387	CTRB2	chymotrypsinogen B2	1.01301	1.012013	1.0087819
440389	[No Symbo	[No Name]	0.99704	0.984051	1.0174911
440400	RNASEK	ribonuclease, RNase K	0.98925	1.069189	0.9712525
440419	LOC440419	TBC1 domain family, member 3H pseudogene	0.99429	0.983906	0.9811665
440423	SUZ12P	suppressor of zeste 12 homolog pseudogene	0.98546	1.062843	1.0535356
440435	GPR179	G protein-coupled receptor 179	1.00046	1.004201	0.999308
440456	PLEKHM1P	pleckstrin homology domain containing, family M (with RUN domain) member 1 pseudogene	0.99427	1.013542	0.9614063
440459	LOC440459	solute carrier family 16, member 6 pseudogene	1.00539	1.016626	0.9796514
440461	LOC440461	SH3 domain containing 20 pseudogene	0.98855	0.949247	1.0337825
440465	FLJ90757	hypothetical LOC440465	0.98842	0.964143	1.0704619
440489	LOC440489	ubiquitin fusion degradation 1-like pseudogene	1.00324	0.984886	0.96576
440491	[No Symbo	[No Name]	0.93541	0.929921	0.9766704
440503	PLIN5	perilipin 5	1.00277	1.050759	0.9332489
440508	CLEC4GP1	C-type lectin domain family 4, member G pseudogene 1	0.99006	1.028842	1.1156904
440515	ZNF506	zinc finger protein 506	0.93712	0.900717	0.8980823
440518	LOC440518	golgin A2 pseudogene	1.00044	1.006306	1.0207508
440519	ZNF724P	zinc finger protein 724, pseudogene	0.98273	0.94562	1.0183329
440525	LOC440525	proline rich 13 pseudogene	0.9827	1.053834	1.0794347
440533	PSG8	pregnancy specific beta-1-glycoprotein 8	0.96894	0.991354	1.0377759
440574	C1orf151	chromosome 1 open reading frame 151	0.97972	0.915886	0.9218483
440575	RPL21P29	ribosomal protein L21 pseudogene 29	1.00014	0.958802	1.0045865
440577	LOC440577	nucleophosmin (nucleolar phosphoprotein B23, numatrin) pseudogene	1.00976	0.969438	0.9525511
440585	FAM183A	family with sequence similarity 183, member A	1.01041	0.99946	0.9929533
440587	RPL6P1	ribosomal protein L6 pseudogene 1	0.97641	1.019896	0.987369
440589	RPS2P8	ribosomal protein S2 pseudogene 8	0.99732	1.011761	1.0005293
440590	ZYG11A	zyg-11 homolog A (C. elegans)	0.99982	0.981209	1.0165035
440595	EEF1A1P11	eukaryotic translation elongation factor 1 alpha 1 pseudogene 11	0.99333	0.996048	1.026831
440597	[No Symbo	[No Name]	1.00554	0.977912	0.9989693
440603	BCL2L15	BCL2-like 15	1.01851	1.042306	1.0824082
440606	HSD3BP2	hydroxy-delta-5-steroid dehydrogenase, 3 beta, pseudogene 2	1.0015	0.999045	1.0345944
440675	NBPF12	neuroblastoma breakpoint family, member 12	0.98346	0.918951	0.9874783

440695	ETV3L	ets variant 3-like	1.00453	1.039459	1.0508841
440699	LRRC52	leucine rich repeat containing 52	1.02945	1.015454	0.9824998
440700	LOC440700	carbonic anhydrase XIV (CA14) pseudogene	0.99707	1.035374	1.0537929
440706	[No Symbo	[No Name]	1.00758	1.002142	1.0174791
440730	TRIM67	tripartite motif containing 67	0.99769	1.003788	1.0082493
440731	[No Symbo	[No Name]	1.0222	0.924308	1.0221859
440733	[No Symbo	[No Name]	0.94499	0.975659	1.0579595
440737	RPL35P1	ribosomal protein L35 pseudogene 1	0.97266	1.00731	1.043039
440742	LOC440742	hypothetical LOC440742	1.01189	0.97412	0.9991236
440757	MGC44328	hypothetical LOC440757	1.00665	0.975659	0.9338931
440786	LOC440786	hypothetical LOC440786	0.97708	0.984233	1.0220988
440792	LOC440792	proline dehydrogenase (oxidase) 1 pseudogene	0.98046	0.989386	1.0124205
440822	PIWIL3	piwi-like 3 (Drosophila)	0.99423	1.000918	1.0047341
440823	MIAT	myocardial infarction associated transcript (non-protein coding)	1.00849	0.979438	0.9635996
440829	SHISA8	shisa homolog 8 (Xenopus laevis)	0.99075	1.005192	1.1147871
440836	ODF3B	outer dense fiber of sperm tails 3B	0.98611	0.984171	1.1221699
440854	CAPN14	calpain 14	1.01513	1.015011	0.9954577
440865	FLJ46838	FLJ46838 protein	0.98939	0.982313	1.0249844
440895	LOC440895	two pore channel 3 pseudogene	1.00502	0.943986	1.0611916
440900	LOC440900	hypothetical LOC440900	0.9856	1.040683	0.9569797
440902	LOC440902	nucleophosmin (nucleolar phosphoprotein B23, numatrin) pseudogene	1.0005	0.980981	1.053593
440915	POTEKP	POTE ankyrin domain family, member K, pseudogene	1.02677	0.984065	1.0600513
440917	LOC440917	tyrosine 3-monooxygenase/tryptophan 5-monooxygenase activation protein, epsilon polypeptide pseudogen	1.02472	0.997715	1.0581469
440934	LOC440934	hypothetical LOC440934	0.98961	1.001604	1.0343956
440944	LOC440944	hypothetical LOC440944	0.97144	1.024472	0.9141193
440955	TMEM89	transmembrane protein 89	1.02143	1.034454	1.0247841
440956	IQCF6	IQ motif containing F6	1.00193	0.940659	1.009009
440957	C3orf78	chromosome 3 open reading frame 78	1.01195	0.970053	1.0430752
440973	LOC440973	nuclear transcription factor Y, beta pseudogene	0.99899	0.995347	1.0063003
440981	LOC440981	similar to hCG1794790	0.99641	1.005101	1.0313101
440982	FLJ30375	hypothetical LOC440982	0.99491	0.996097	1.0152407
440991	RPS3P3	ribosomal protein S3 pseudogene 3	0.93855	0.994127	1.0990689
440993	LOC440993	hypothetical LOC440993	0.9955	0.993231	1.0081023

441005	[No Symbo	[No Name]	0.98946	0.992896	1.0220739
441009	LOC441009	hypothetical protein LOC441009	1.00725	1.029724	0.9960861
441013	RPS2P21	ribosomal protein S2 pseudogene 21	0.97094	1.013055	1.0643135
441024	MTHFD2L	methylenetetrahydrofolate dehydrogenase (NADP+ dependent) 2-like	0.9883	0.947713	1.0063202
441026	LOC441026	heterogeneous nuclear ribonucleoprotein A3 pseudogene	1.01259	0.958577	0.9894671
441027	TMEM150C	transmembrane protein 150C	1.00368	1.000834	1.0244104
441032	EEF1A1P9	eukaryotic translation elongation factor 1 alpha 1 pseudogene 9	1.05256	0.952872	1.1020421
441034	RPL7AP30	ribosomal protein L7a pseudogene 30	1.01433	0.96331	0.9978237
441046	GYPELOC44	glucuronidase, beta pseudogene	0.97145	0.937495	0.9832478
441051	HSP90AA6P	heat shock protein 90kDa alpha (cytosolic), class A member 6, pseudogene	1.00137	0.999018	1.0404453
441054	C4orf47	chromosome 4 open reading frame 47	0.99894	0.972079	0.9980036
441058	MGC39584	hypothetical LOC441058	1.05192	1.016191	1.0187348
441061	11-Mar	membrane-associated ring finger (C3HC4) 11	0.99892	0.981284	1.0104596
441073	RPL26P19	ribosomal protein L26 pseudogene 19	1.00681	0.942232	0.9834465
441097	[No Symbo	[No Name]	1.01475	1.006618	1.0169476
441108	C5orf56	chromosome 5 open reading frame 56	0.99966	0.929282	0.8239076
441131	ARPC3P5	actin related protein 2/3 complex, subunit 3 pseudogene 5	1.00145	0.981965	1.0271601
441132	IMPDH1P9	IMP (inosine monophosphate) dehydrogenase 1 pseudogene 9	1.00971	1.002199	0.9940572
441133	KRT18P38	keratin 18 pseudogene 38	0.99015	1.015822	1.0226555
441150	C6orf226	chromosome 6 open reading frame 226	0.96049	1.020842	1.0113502
441151	TMEM151B	transmembrane protein 151B	0.99725	0.972774	0.997571
441154	RPL7AP34	ribosomal protein L7a pseudogene 34	1.01448	0.994469	1.0088113
441160	KRT19P1	keratin 19 pseudogene 1	0.99088	1.022408	1.0509126
441161	OOEP	oocyte expressed protein homolog (dog)	0.99063	1.030511	0.9992033
441166	CNN2P9	calponin 2 pseudogene 9	0.99138	0.996561	1.0337727
441167	LOC441167	hCG1820801	1.01005	1.008163	0.9982195
441168	FAM26F	family with sequence similarity 26, member F	1.01422	0.966707	1.0827596
441172	FLJ46906	hypothetical LOC441172	1.02206	1.058601	1.0313432
441178	LOC441178	hypothetical LOC441178	1.00429	0.982147	1.0279665
441179	LOC441179	hypothetical LOC441179	1.00235	0.980686	1.0223843
441194	PMS2CL	PMS2 C-terminal like pseudogene	0.9657	1.006998	0.9986846
441208	LOC441208	zinc and ring finger 2 pseudogene	0.9325	1.026147	1.1160281
441212	RP9P	retinitis pigmentosa 9 pseudogene	1.00367	0.99571	1.0672556

441218	KRT8P20	keratin 8 pseudogene 20	1.00373	0.960789	0.9944875
441228	LOC441228	exportin, tRNA (nuclear export receptor for tRNAs) pseudogene	1.01184	1.004169	0.9962419
441234	ZNF716	zinc finger protein 716	1.00581	0.990385	0.9980944
441238	NMD3P1	NMD3 homolog (S. cerevisiae) pseudogene 1	1.00057	0.951932	1.0130064
441239	LOC441239	hypothetical protein LOC441239	1.01281	1.00243	1.0228564
441241	LOC441241	vitamin K epoxide reductase complex, subunit 1-like 1 pseudogene	0.97953	1.055192	1.046269
441242	LOC441242	hypothetical LOC441242	0.98665	0.998864	1.0419847
441246	RPL35P5	ribosomal protein L35 pseudogene 5	0.92449	0.965333	1.0988493
441250	TYW1B	tRNA-yW synthesizing protein 1 homolog B (S. cerevisiae)	0.96706	1.027741	0.9709888
441272	SPDYE3	speedy homolog E3 (Xenopus laevis)	1.02243	0.931898	1.0416695
441282	AKR1B15	aldo-keto reductase family 1, member B15	0.99229	1.014069	1.0164706
441355	FLJ39080	hypothetical LOC441355	1.01684	0.996594	1.0054574
441363	REXO1L7P	REX1, RNA exonuclease 1 homolog (S. cerevisiae)-like 7, pseudogene	1.00304	0.952668	1.0226769
441369	FLJ46284	hypothetical LOC441369	0.9911	0.991474	1.0224955
441376	C8orf85	chromosome 8 open reading frame 85	1.0203	0.967345	1.0404378
441377	RPS26P35	ribosomal protein S26 pseudogene 35	0.90178	1.004506	0.8967195
441381	LRRC24	leucine rich repeat containing 24	0.97236	1.032056	1.1089898
441391	RBMXP2	RNA binding motif protein, X-linked pseudogene 2	0.96194	0.98177	1.0123111
441400	RAB1C	RAB1C, member RAS oncogene family pseudogene	1.00767	0.99695	1.0802185
441455	LOC441455	makorin ring finger protein 1 pseudogene	0.98685	0.925268	1.0094399
441459	ANKRD18B	ankyrin repeat domain 18B	0.98122	0.986957	1.0658935
441461	LOC441461	hypothetical LOC441461	0.983	0.990666	1.0216705
441478	NRARP	NOTCH-regulated ankyrin repeat protein	0.99052	0.98538	1.0134677
441481	LOC441481	glutathione peroxidase 1 pseudogene	0.9913	1.039831	0.9628592
441484	LOC441484	coiled-coil domain containing 5 (spindle associated) pseudogene	0.98751	1.02067	0.9902495
441487	LOC441487	methyltransferase like 1 pseudogene	0.97298	0.985015	0.9356502
441493	LOC441493	hypothetical LOC441493	0.96187	0.979908	1.0109772
441502	RPS26P11	ribosomal protein S26 pseudogene 11	0.88196	1.000817	0.9728118
441505	LOC441505	stress-induced-phosphoprotein 1 pseudogene	1.00764	1.027056	1.0103188
441506	[No Symbo	[No Name]	0.91898	0.961261	1.0841529
441509	GLRA4	glycine receptor, alpha 4	0.99982	1.0132	1.00118
441511	LOC441511	mitofusin-1-like	1.00982	0.989007	1.0330062
441518	FAM127C	family with sequence similarity 127, member C	0.97935	1.012786	0.9787252

441525	SPANXN4	SPANX family, member N4	1.004	0.956865	0.9716027
441531	PGAM4	phosphoglycerate mutase family member 4	0.96033	0.889724	1.0272193
441548	LOC441548	BTB (POZ) domain containing 7 pseudogene	0.97422	0.972266	0.9816693
441549	CDNF	cerebral dopamine neurotrophic factor	0.98668	0.931572	0.9299768
441550	RPS4XP11	ribosomal protein S4X pseudogene 11	0.95893	0.95246	1.0738997
441553	[No Symbo	[No Name]	0.98925	1.024767	0.9854572
441572	GAPDHP28	glyceraldehyde 3 phosphate dehydrogenase pseudogene 28	0.98712	1.015624	1.0126745
441592	LOC441592	mas-related G-protein coupled receptor member X1-like	0.9922	0.965975	0.9161428
441601	LOC441601	septin 7 pseudogene	0.99102	1.016974	1.0103426
441608	OR5B3	olfactory receptor, family 5, subfamily B, member 3	0.9852	1.025438	0.9602477
441609	YWHAZP9	tyrosine 3-monooxygenase/tryptophan 5-monooxygenase activation protein, zeta pseudogene 9	0.99196	0.995516	0.9938377
441617	LOC441617	hypothetical LOC441617	1.00944	1.048521	1.0754207
441623	FTLP7	ferritin, light polypeptide pseudogene 7	1.00484	0.931122	0.9947252
441631	TSPAN11	tetraspanin 11	0.99346	0.989274	0.9986924
441632	RPL13AP22	ribosomal protein L13a pseudogene 22	0.98782	0.995371	1.0423187
441639	OR9K2	olfactory receptor, family 9, subfamily K, member 2	0.99236	1.001652	1.029774
441641	RPL13AP23	ribosomal protein L13a pseudogene 23	0.94438	0.983006	1.1326963
441642	RPL7AP9	ribosomal protein L7a pseudogene 9	0.96679	0.998327	1.0520413
441643	LOC441643	p53 and DNA damage-regulated protein 1-like	0.98962	0.992342	0.9794754
441654	SMPD4P2	sphingomyelin phosphodiesterase 4, neutral membrane (neutral sphingomyelinase-3) pseudogene 2	1.00233	0.990745	1.0075963
441662	TET1P1	tet oncogene 1 pseudogene 1	0.99712	1.011173	0.9766396
441666	LOC441666	zinc finger protein 91 pseudogene	1.01342	1.049044	1.113598
441669	OR4Q3	olfactory receptor, family 4, subfamily Q, member 3	0.99568	0.990717	1.0060142
441687	TEX21P	testis expressed 21, pseudogene	0.98692	1.058941	1.0374186
441720	[No Symbo	[No Name]	1.02448	1.004088	1.0536757
441722	LOC441722	U2 small nuclear RNA auxiliary factor 1 pseudogene	0.93523	1.033462	0.9913342
441726	RPL28P4	ribosomal protein L28 pseudogene 4	1.03696	0.961914	0.9660434
441728	LOC441728	golgin-like	0.98779	1.053708	1.0133397
441732	[No Symbo	[No Name]	0.99522	0.996964	0.9935117
441733	PRKXP1	protein kinase, X-linked, pseudogene 1	0.96284	0.936482	0.9815149
441736	LOC441736	Golgin subfamily A member 8-like protein 1-like	1.01152	0.964801	0.9886456
441750	LOC441750	chromosome 12 open reading frame 49 pseudogene	0.98618	0.998042	0.981892
441768	LOC441768	RAB31, member RAS oncogene family pseudogene	1.01082	0.943806	0.9696294

441771	MT1CP	metallothionein 1C, pseudogene	1.01061	1.067173	0.9706517
441774	RPS4Y1P1	ribosomal protein S4Y pseudogene 1	0.9886	1.008684	1.0177453
441775	RPL18P13	ribosomal protein L18 pseudogene 13	0.92127	0.913881	1.0957367
441781	RPL23AP76	ribosomal protein L23a pseudogene 76	1.01313	0.965777	1.021854
441783	LOC441783	POTE ankyrin domain family, member I pseudogene	0.9584	0.938087	1.0547742
441784	LOC441784	TBC1 domain family, member 25 pseudogene	0.99094	0.950127	1.0269338
441795	HMGB3P27	high mobility group box 3 pseudogene 27	1.0008	1.069695	1.0336499
441811	LOC441811	uncharacterized protein C9orf172-like	0.98259	1.026928	1.0720887
441818	WBP11P1	WW domain binding protein 11 pseudogene 1	0.99881	0.992223	0.9848088
441843	ZNF209P	zinc finger protein 209, pseudogene	1.00068	0.972428	1.0032252
441849	LOC441849	methionine-R-sulfoxide reductase B1-like	0.99927	1.00403	1.0308007
441862	LOC441862	DUT pseudogene	1.00366	1.046087	1.0365872
441864	TARM1	T cell-interacting, activating receptor on myeloid cells 1	0.9597	1.043584	0.9665818
441869	LOC441869	hypothetical protein LOC441869	1.00094	1.003231	1.0538158
441878	RPS4XP4	ribosomal protein S4X pseudogene 4	1.00247	1.066733	1.0303262
441880	LOC441880	eukaryotic translation elongation factor 1 alpha 2 pseudogene	0.99752	1.038987	1.0334544
441884	CALR4P	calreticulin 4, pseudogene	1.00745	0.998547	1.0111857
441887	LOC441887	DnaJ (Hsp40) homolog, subfamily C, member 7 pseudogene	1.00985	1.02682	1.0572464
441891	WDR82P2	WD repeat domain 82 pseudogene 2	0.9967	1.005077	1.0128991
441893	GAPDHP29	glyceraldehyde 3 phosphate dehydrogenase pseudogene 29	1.01023	1.015011	1.0012836
441896	RPL7P8	ribosomal protein L7 pseudogene 8	0.92625	1.006452	1.0048811
441897	PGCP1	progastricsin (pepsinogen C) pseudogene 1	1.00495	1.01909	0.9776001
441899	LOC441899	vacuolar protein sorting 25 homolog (S. cerevisiae) pseudogene	1.00629	1.033469	1.0240412
441900	HSD3BP3	hydroxy-delta-5-steroid dehydrogenase, 3 beta, pseudogene 3	1.01108	0.953384	1.0172368
441904	LOC441904	autoantigen La-like	0.99442	0.986773	1.044574
441907	LOC441907	ribosomal protein L6 pseudogene	1.00953	1.000452	1.0466699
441908	NBPF18P	neuroblastoma breakpoint family, member 18, pseudogene	1.01139	0.982211	1.0005531
441911	OR10J3	olfactory receptor, family 10, subfamily J, member 3	1.00827	1.010934	0.9890202
441915	LOC441915	solute carrier family 25, member 38 pseudogene	1.00346	0.990799	1.0257674
441932	OR2W5	olfactory receptor, family 2, subfamily W, member 5	0.98522	1.018121	1.010388
441938	[No Symbo	[No Name]	0.99224	0.972215	0.9317498
441940	LIN28AP3	lin-28 homolog A (C. elegans) pseudogene 3	0.97395	1.009783	1.038577
441951	NCRNA002	non-protein coding RNA 275	0.99185	0.969536	1.0274165

441956	LOC441956	similar to cDNA sequence BC021523	0.98199	1.05007	1.020039
441964	[No Symbo	[No Name]	0.99256	0.996499	1.0258919
441996	LOC441996	aconitase 2, mitochondrial pseudogene	1.0056	0.981084	1.0013566
442006	LOC442006	nucleophosmin (nucleolar phosphoprotein B23, numatrin) pseudogene	0.98765	0.951488	1.0094279
442013	LDHAP3	lactate dehydrogenase A pseudogene 3	0.99407	1.0145	1.0167673
442017	LOC442017	interferon induced transmembrane protein pseudogene	1.03715	1.029765	1.0491315
442028	LOC442028	hypothetical LOC442028	1.01345	0.959551	1.0177448
442035	[No Symbo	[No Name]	1.0461	1.031288	0.9984313
442038	SULT1C3	sulfotransferase family, cytosolic, 1C, member 3	1.04156	0.992618	0.9678132
442058	LOC442058	replication protein A 14 kDa subunit-like	0.98894	0.96128	0.9899966
442060	LOC442060	Sin3A-associated protein, 18kDa pseudogene	1.01964	0.994517	1.067628
442063	LOC442063	hypothetical LOC442063	1.00667	0.986604	0.9862832
442078	KRT8P18	keratin 8 pseudogene 18	1.01448	1.005899	1.0042064
442083	HSP90AB5I	heat shock protein 90kDa alpha (cytosolic), class B member 5, pseudogene	1.01452	0.979374	0.9894834
442086	RPL10P7	ribosomal protein L10 pseudogene 7	1.00622	1.056679	1.0068578
442087	LOC442087	prostate tumor overexpressed 1 pseudogene	1.01072	0.991688	1.0797202
442097	LOC442097	actin, gamma 1 pseudogene	0.99492	0.928561	1.1175727
442098	LOC442098	methylenetetrahydrofolate dehydrogenase (NADP+ dependent) 2, methenyltetrahydrofolate cyclohydrolase	0.99143	1.005719	0.9854777
442113	LOC442113	protein tyrosine phosphatase, non-receptor type 11 pseudogene	1.01787	0.981284	0.985506
442117	GALNTL6	UDP-N-acetyl-alpha-D-galactosamine:polypeptide N-acetylgalactosaminyltransferase-like 6	1.0047	1.007104	1.0041655
442131	LOC442131	asparagine-linked glycosylation 3, alpha-1,3- mannosyltransferase homolog (S. cerevisiae) pseudogene	1.0156	0.971126	1.0527665
442142	SOX30P1	SRY (sex determining region Y)-box 30 pseudogene 1	1.0093	1.027793	1.114007
442153	PSMC1P11	proteasome (prosome, macropain) 26S subunit, ATPase, 1 pseudogene 11	1.00018	1.013512	1.0092925
442155	LOC442155	transcription factor B2, mitochondrial pseudogene	0.95941	1.069939	1.0365011
442156	LOC442156	pyruvate kinase, muscle pseudogene	1.0156	0.987591	1.0438714
442157	LOC442157	heterogeneous nuclear ribonucleoprotein L pseudogene	0.98963	0.97437	1.0108686
442160	RPL21P62	ribosomal protein L21 pseudogene 62	1.03627	0.964273	1.0206167
442161	LOC442161	ribosomal protein L7-like 1 pseudogene	1.00146	0.952915	0.9840497
442162	RPS4XP7	ribosomal protein S4X pseudogene 7	0.98925	0.951371	1.0024323
442164	DDX18P3	DEAD (Asp-Glu-Ala-Asp) box polypeptide 18 pseudogene 3	0.99926	1.047746	1.0270738
442167	ASS1P1	argininosuccinate synthetase 1 pseudogene 1	1.02794	0.992305	0.9488387
442171	RPL10P2	ribosomal protein L10 pseudogene 2	0.9716	0.921798	1.0818752
442172	LOC442172	cell division cycle associated 7 pseudogene	0.99112	1.02588	0.9749683

442175	RPLP2P1	ribosomal protein, large P2, pseudogene 1	1.04017	0.96613	1.0115542
442179	OR1F12	olfactory receptor, family 1, subfamily F, member 12	1.01771	0.995885	1.0041899
442181	RPSAP2	ribosomal protein SA pseudogene 2	0.99427	0.992454	0.9570639
442183	OR2P1P	olfactory receptor, family 2, subfamily P, member 1 pseudogene	1.0304	1.023562	1.0165187
442184	OR2B3	olfactory receptor, family 2, subfamily B, member 3	1.02648	1.014304	1.033707
442188	OR2J4P	olfactory receptor, family 2, subfamily J, member 4 pseudogene	0.97563	1.04511	1.0326893
442189	OR2H4P	olfactory receptor, family 2, subfamily H, member 4 pseudogene	1.00614	1.002873	0.9823648
442190	OR2B4P	olfactory receptor, family 2, subfamily B, member 4 pseudogene	0.98916	1.045466	0.9474506
442192	DDX6P1	DEAD (Asp-Glu-Ala-Asp) box polypeptide 6 pseudogene 1	1.0089	1.003592	0.9893127
442195	RPS17P1	ribosomal protein S17 pseudogene 1	0.98993	0.926126	1.0273043
442197	OR2I1P	olfactory receptor, family 2, subfamily I, member 1 pseudogene	0.99616	0.967557	1.0545186
442204	MYL8P	myosin, light chain 8, pseudogene	0.9775	0.927202	1.0247368
442205	KRT18P9	keratin 18 pseudogene 9	1.0046	0.993305	1.0247468
442210	LOC442210	tubulin beta chain-like	1.02028	1.022387	1.0240802
442211	ATP6V0CP3	ATPase, H+ transporting, lysosomal 16kDa, V0 subunit c pseudogene 3	0.9553	0.990603	0.9844729
442213	C6orf138	chromosome 6 open reading frame 138	1.00408	1.0226	1.013988
442215	LOC442215	eukaryotic translation elongation factor 1 alpha 2 pseudogene	1.04652	1.052	1.02568
442217	RPL31P28	ribosomal protein L31 pseudogene 28	1.01708	1.014555	0.9378206
442221	LOC442221	regulator of chromosome condensation 2 pseudogene	1.01155	1.003364	1.0919038
442225	LOC442225	keratin, type II pseudogene	1.00371	0.97625	1.0307316
442227	EEF1B2P5	eukaryotic translation elongation factor 1 beta 2 pseudogene 5	1.01304	0.983174	0.999386
442229	MCART3P	mitochondrial carrier triple repeat 3 pseudogene	1.00155	1.042813	0.9875899
442231	GAPDHP63	glyceraldehyde 3 phosphate dehydrogenase pseudogene 63	1.00753	0.907314	1.0317335
442232	RPL17P25	ribosomal protein L17 pseudogene 25	1.03304	0.997288	0.9836921
442233	SMARCE1P	SWI/SNF related, matrix associated, actin dependent regulator of chromatin, subfamily e, member 1 pseudog	1.01783	1.011244	1.0196339
442234	RCN1P1	reticulocalbin 1, EF-hand calcium binding domain pseudogene 1	1.00242	0.990015	1.032875
442236	KRT18P50	keratin 18 pseudogene 50	1.0297	0.988429	0.9837573
442237	RPS7P8	ribosomal protein S7 pseudogene 8	1.03454	0.99729	1.0375182
442239	LOC442239	peroxiredoxin 2 pseudogene	0.98256	1.097596	1.0350092
442240	ZNF259P1	zinc finger protein 259 pseudogene 1	0.96347	0.981047	1.0270317
442244	LOC442244	PDGFA associated protein 1 pseudogene	1.00941	0.96518	1.086814
442245	GSTM2P1	glutathione S-transferase mu 2 (muscle) pseudogene 1	0.99716	1.05254	1.0944475
442247	RFPL4B	ret finger protein-like 4B	0.99356	0.980203	1.0395465

442249	LOC442249	keratin 18 pseudogene	0.97467	0.973945	1.0227028
442252	KRT18P22	keratin 18 pseudogene 22	1.02014	1.064154	1.0339464
442253	NEPNP	nephrocan, pseudogene	1.01996	1.034764	0.9986566
442255	SLC25A5P7	solute carrier family 25 (mitochondrial carrier; adenine nucleotide translocator), member 5 pseudogene 7	1.02401	1	0.9589088
442256	LOC442256	hypothetical LOC442256	1.00744	0.97299	1.0252342
442257	RPS4XP9	ribosomal protein S4X pseudogene 9	1.0013	0.998864	1.0812893
442258	EEF1DP5	eukaryotic translation elongation factor 1 delta pseudogene 5	0.9648	0.977029	1.0496443
442260	RPL23AP46	ribosomal protein L23a pseudogene 46	1.03605	1.102291	1.0425346
442261	[No Symbo	[No Name]	1.0108	0.966751	1.0480976
442262	GAPDHP73	glyceraldehyde-3-phosphate dehydrogenase pseudogene 73	0.98333	0.961702	1.0772345
442263	LOC442263	protein tyrosine phosphatase, non-receptor type 11 pseudogene	1.01573	0.981618	1.0172746
442266	YAP1P1	Yes-associated protein 1 pseudogene 1	1.02483	1.007775	1.0020259
442267	CCT7P1	chaperonin containing TCP1, subunit 7 (eta) pseudogene 1	1.00786	1.002664	1.0152513
442271	LOC442271	tubulin, beta 2C pseudogene	1.00571	0.990379	1.0434427
442290	NHP2P2	NHP2 ribonucleoprotein homolog (yeast) pseudogene 2	1.01243	0.970657	1.057797
442293	LOC442293	glycine cleavage system protein H pseudogene	0.96704	1.006705	1.083666
442304	FTLP15	ferritin, light polypeptide pseudogene 15	1.01761	1.011009	1.0085425
442308	LOC442308	tubulin, beta pseudogene	0.99328	0.995092	1.0211427
442317	LOC442317	chromosome 9 open reading frame 140 pseudogene	0.99933	0.980337	1.0943252
442318	LOC442318	chromosome 9 open reading frame 140 pseudogene	0.97767	1.05202	1.0488041
442319	ZNF727	zinc finger protein 727	0.99279	1.021343	0.9915631
442320	LOC442320	chromosome 9 open reading frame 140 pseudogene	0.9757	1.02289	1.1090227
442325	EEF1DP4	eukaryotic translation elongation factor 1 delta pseudogene 4	0.98834	1.000331	1.0181101
442326	RPL31P38	ribosomal protein L31 pseudogene 38	1.0288	0.956043	0.9986107
442334	ARF1P1	ADP-ribosylation factor 1 pseudogene 1	1.02035	1.005832	0.9825608
442338	IRS3P	insulin receptor substrate 3, pseudogene	1.02048	1.014233	1.0540564
442382	LOC442382	ATPase, Ca++ transporting, plasma membrane 1 pseudogene	1.00375	1.032524	1.0145574
442388	SDR16C6P	short chain dehydrogenase/reductase family 16C, member 6, pseudogene	0.9901	1.014218	1.0313344
442396	ARF1P3	ADP-ribosylation factor 1 pseudogene 3	1.00813	1.007274	0.9951604
442405	LOC442405	keratin 18 pseudogene	0.97353	1.00953	0.9922932
442406	KRT18P36	keratin 18 pseudogene 36	1.00689	0.994895	1.017166
442425	FOXB2	forkhead box B2	0.9768	1.029636	1.0264824
442426	RPS2P34	ribosomal protein S2 pseudogene 34	0.99048	0.975866	1.0406195

442427	LOC442427	putative UPF0607 protein ENSP00000381514-like	0.95457	0.971476	1.0313531
442429	EEF1DP2	eukaryotic translation elongation factor 1 delta pseudogene 2	0.95669	0.975123	0.987604
442434	LOC442434	hypothetical protein LOC442434	0.99931	1.027678	0.9829298
442447	LOC442447	chloride intracellular channel 4 pseudogene	0.96492	1.028484	1.0366087
442455	KRT8P27	keratin 8 pseudogene 27	1.00553	1.015653	0.9965639
442459	LOC442459	X-ray repair complementing defective repair pseudogene	1.00915	1.01288	0.9978522
442461	[No Symbo	[No Name]	1.02045	0.968359	0.9867339
442465	LOC442465	sarcoma antigen 1-like	0.99414	0.987552	1.0294145
442485	OFD1P16Y	OFD1 pseudogene 16, Y-linked	0.99391	1.026219	1.0106312
442517	LOC442517	ATP-binding cassette, sub-family E (OABP), member 1 pseudogene	0.97968	1.017413	0.9738365
442523	DPY19L2P4	dpy-19-like 2 pseudogene 4 (C. elegans)	1.01312	0.946592	1.000089
442524	DPY19L2P3	dpy-19-like 2 pseudogene 3 (C. elegans)	0.97426	1.043159	0.9310494
442525	SLC25A5P5	solute carrier family 25 (mitochondrial carrier; adenine nucleotide translocator), member 5 pseudogene 5	0.98726	0.976941	0.9988954
442659	RPL23P7	ribosomal protein L23 pseudogene 7	0.96353	0.937484	1.0604235
442660	RPL7AP38	ribosomal protein L7a pseudogene 38	1.00601	0.99947	0.9790262
442661	HMGB3P20	high mobility group box 3 pseudogene 20	1.01437	1.004317	0.9932986
442663	[No Symbo	[No Name]	1.00168	1.032094	1.0057456
442664	[No Symbo	[No Name]	1.01036	1.036202	1.0317861
442668	LOC442668	NECAP endocytosis associated 1 pseudogene	1.01917	1.026302	0.9720075
442673	TUBG1P	tubulin, gamma 1 pseudogene	1.03301	1.057267	1.0205718
442676	CICP8	capicua homolog (Drosophila) pseudogene 8	0.99334	1.020426	0.9947207
442707	LOC442707	methylene tetrahydrofolate dehydrogenase 2 pseudogene	1.0034	0.943452	1.0266101
442709	EEF1A1P28	eukaryotic translation elongation factor 1 alpha 1 pseudogene 28	1.03939	0.97824	1.0305884
442710	LOC442710	EF-hand calcium-binding domain-containing protein 1-like	0.99509	1.032794	1.0170011
442712	RPL7AP40	ribosomal protein L7a pseudogene 40	0.95018	1.000322	1.0419836
442713	LOC442713	peptidylprolyl isomerase A pseudogene	0.98012	1.013759	0.9881253
442720	EIF3IP1	eukaryotic translation initiation factor 3, subunit I pseudogene 1	1.00906	1.023382	0.9926326
442721	LMOD2	leiomodin 2 (cardiac)	0.99868	1.006846	0.9908116
442723	RPL27P11	ribosomal protein L27 pseudogene 11	1.04159	0.950967	1.138342
442726	RPS17P12	ribosomal protein S17 pseudogene 12	0.98955	1.012158	0.9360313
442727	LOC442727	prothymosin alpha pseudogene	1.00126	0.989338	1.0514473
442730	ST13P17	suppression of tumorigenicity 13 (colon carcinoma) (Hsp70 interacting protein) pseudogene 17	0.99466	0.983717	1.0023396
442890	MIR133B	microRNA 133b	0.9947	1.042239	1.0300103

442898	MIR324	microRNA 324	0.97927	1.041041	0.9958694
442920	MIR196B	microRNA 196b	1.01586	1.018448	1.0458253
444882	IGFL4	IGF-like family member 4	1.02235	1.001433	0.9244294
445577	C9orf129	chromosome 9 open reading frame 129	1.00739	0.99947	0.990013
445815	PALM2-AKA	PALM2-AKAP2 readthrough	1.00399	0.989331	1.0100585
448834	KPRP	keratinocyte proline-rich protein	1.0288	1.022823	1.0405311
449491	DEFA8P	defensin, alpha 8 pseudogene	1.02115	1.004157	1.0532417
449518	LOC449518	purinergic receptor P2Y, G-protein coupled, 10 pseudogene	1.01839	0.986671	1.1052398
449645	SF3A3P2	splicing factor 3a, subunit 3 pseudogene 2	0.98699	0.93365	0.9434853
474167	HDGFP1	hepatoma-derived growth factor pseudogene 1	0.97719	1.06331	1.0152567
474171	STGC3	hypothetical STGC3	1.01538	0.998988	0.9464222
474343	SPIN2B	spindlin family, member 2B	0.99125	1.017515	1.0087819
474344	GIMAP6	GTPase, IMAP family member 6	0.99096	0.840184	1.1559566
474345	GIMAP3P	GTPase, IMAP family member 3 pseudogene	0.99074	0.97245	0.9838741
474354	LRRC18	leucine rich repeat containing 18	1.00366	1.015373	0.9918658
492295	RAC1P3	ras-related C3 botulinum toxin substrate 1 pseudogene 3	1.00771	0.992612	0.9992483
492303	GEMIN8P4	gem (nuclear organelle) associated protein 8 pseudogene 4	0.98631	1.050093	1.0104547
492307	C8orf22	chromosome 8 open reading frame 22	1.00936	1.019978	1.0184118
492311	C5orf53	chromosome 5 open reading frame 53	1.00864	1.008488	0.9649063
493753	C2orf64	chromosome 2 open reading frame 64	0.98375	0.921696	1.0095376
493754	LOC493754	RAB guanine nucleotide exchange factor (GEF) 1 pseudogene	1.00556	1.034679	0.9766687
493829	TRIM72	tripartite motif containing 72	0.98726	1.048545	1.0539348
493856	CISD2	CDGSH iron sulfur domain 2	0.97472	1.011342	1.1128253
493860	CCDC73	coiled-coil domain containing 73	0.99146	1.00247	1.0154902
493869	GPX8	glutathione peroxidase 8 (putative)	1.00617	0.98304	1.0007066
493901	RNASE12	ribonuclease, RNase A family, 12 (non-active)	0.97923	1.053134	1.060785
493913	PAPPA-AS1	PAPPA antisense RNA 1 (non-protein coding)	0.97775	0.966506	0.9988625
494024	[No Symbo	[No Name]	0.94529	1.000239	1.0361011
494119	SPANXN2	SPANX family, member N2	0.98039	1.078725	0.9935556
494127	LOC494127	NFYC pseudogene	0.99433	1.02632	0.9881385
494141	LOC494141	mitochondrial carrier triple repeat 1 pseudogene	0.98122	1.022327	1.0279196
494143	CHAC2	ChaC, cation transport regulator homolog 2 (E. coli)	1.00514	1.015747	1.0249547
494188	FBXO47	F-box protein 47	0.99158	1.010599	1.0470873

494197	SPANXN5	SPANX family, member N5	1.0348	1.002702	1.0095652
494324	MIR375	microRNA 375	0.97612	1.047247	1.1347405
494326	MIR377	microRNA 377	1.03776	1.068788	1.0004409
494329	MIR380	microRNA 380	1.03709	1.04029	1.0034944
494331	MIR382	microRNA 382	1.02993	0.956993	0.9355755
494470	RNF165	ring finger protein 165	1.01463	0.98795	0.9651445
494513	DFNB59	deafness, autosomal recessive 59	1.01181	0.978331	0.9943584
494514	C18orf56	chromosome 18 open reading frame 56	0.97339	1.021683	1.0598762
494551	WEE2	WEE1 homolog 2 (S. pombe)	0.99207	1.011133	0.9586646
497048	KU-MEL-3	KU-MEL-3	1.01136	0.9477	1.0087972
497049	FLJ25758	hypothetical locus FLJ25758	0.99587	0.956002	0.9459101
497189	TIFAB	TRAF-interacting protein with forkhead-associated domain, family member B	1.0132	0.960738	1.0409725
497258	BDNF-AS1	BDNF antisense RNA 1 (non-protein coding)	0.99021	0.980762	0.9915527
497661	C18orf32	chromosome 18 open reading frame 32	0.96971	0.946648	0.9648097
503497	MS4A13	membrane-spanning 4-domains, subfamily A, member 13	0.97583	0.989376	1.0019811
503542	SPRN	shadow of prion protein homolog (zebrafish)	1.00737	1.07727	0.9741165
503543	BTF3P9	basic transcription factor 3 pseudogene 9	0.98294	0.956352	1.1117903
503569	FLJ35946	hypothetical protein FLJ35946	0.96804	0.955638	0.9788471
503582	ARGFX	arginine-fifty homeobox	1.01788	1.032307	0.9779669
503583	ARGFXP1	arginine-fifty homeobox pseudogene 1	1.04103	1.029213	1.0342719
503612	TAAR4P	trace amine associated receptor 4, pseudogene	1.05585	0.98273	1.0009711
503627	TPRX2P	tetra-peptide repeat homeobox 2 pseudogene	0.99291	0.94722	1.0300943
503634	DUXAP5	double homeobox A pseudogene 5	1.01466	1.010774	1.0292937
503834	DPRX	divergent-paired related homeobox	0.9731	0.992013	1.0237446
503835	DUXA	double homeobox A	1.00603	0.993512	0.9866738
541466	CT45A1	cancer/testis antigen family 45, member A1	0.995	1.006879	1.0037302
541468	C1orf190	chromosome 1 open reading frame 190	0.94459	0.981156	0.9818106
541472	LOC541472	hypothetical LOC541472	0.98297	1.001562	1.0085627
541565	C8orf58	chromosome 8 open reading frame 58	0.99855	1.00895	0.9778811
541611	HSP90AB6P	heat shock protein 90kDa alpha (cytosolic), class B member 6, pseudogene	0.99991	0.988453	1.0019468
542767	PCOTH	prostate collagen triple helix	0.99563	1.131196	0.9855625
548594	KIR3DP1	killer cell immunoglobulin-like receptor, three domains, pseudogene 1	0.95224	0.999244	1.0219369
548599	PHBP7	prohibitin pseudogene 7	1.00032	0.990647	1.0475768

550112	LOC550112	hypothetical LOC550112	0.96986	0.98212	1.0634788
550631	CCDC157	coiled-coil domain containing 157	1.00407	1.0373	0.980696
550643	LOC550643	hypothetical LOC550643	0.93395	1.002933	1.0765293
550644	LOC550644	structural maintenance of chromosomes 3 pseudogene	0.9828	0.970435	1.0464072
552889	ATXN7L3B	ataxin 7-like 3B	0.92197	1.005244	1.1523468
553115	PEF1	penta-EF-hand domain containing 1	0.97737	0.954596	0.9143843
553121	TLK2P2	tousled-like kinase 2 pseudogene 2	0.97301	1.005163	1.0084318
553139	LOC553139	PAI-1 mRNA binding protein pseudogene	1.01522	0.970723	1.0380605
553148	LOC553148	PAI-1 mRNA binding protein pseudogene	0.97876	0.989828	0.9720474
553158	PRR5-ARHC	PRR5-ARHGAP8 readthrough	0.98796	1.058313	1.0322872
554045	DUX4L9	double homeobox 4 like 9	1.01405	0.958708	1.0389436
554203	JPX	JPX transcript, XIST activator (non-protein coding)	0.99644	0.9581	1.0011195
554206	LOC554206	hypothetical LOC554206	0.98159	0.965214	0.9913315
554207	LOC554207	hypothetical LOC554207	1.00771	0.935796	1.0142169
554210	MIR429	microRNA 429	0.97353	0.979647	0.9777652
554225	STRCP1	stereocilin pseudogene 1	0.95128	0.986983	0.9848082
554234	RPL36AP40	ribosomal protein L36a pseudogene 40	1.01067	1.004445	0.9932643
554235	ASPDH	aspartate dehydrogenase domain containing	0.99046	1.003974	0.9902245
554250	GDF5OS	growth differentiation factor 5 opposite strand	0.96917	1.057121	0.9982099
554251	FBXO48	F-box protein 48	0.99366	0.973446	1.0187495
574016	CLLU1OS	chronic lymphocytic leukemia up-regulated 1 opposite strand	1.00385	1.005584	1.0443807
574029	DUSP5P	dual specificity phosphatase 5 pseudogene	1.02676	0.995708	0.9845663
574036	C1orf133	chromosome 1 open reading frame 133	0.98733	0.982903	1.0605955
574080	LOC574080	uncharacterized hematopoietic stem/progenitor cells protein MDS029 pseudogene 3	1.0117	1.009785	1.04938
574406	C1orf138	chromosome 1 open reading frame 138	0.97366	1.029932	0.926616
574431	C1orf147	chromosome 1 open reading frame 147	1.03799	0.984766	0.9739165
574447	MIR146B	microRNA 146b	1.0361	1.06215	1.0016691
574448	MIR202	microRNA 202	0.98398	1.035627	1.0001286
574452	MIR494	microRNA 494	1.02587	1.085939	1.0058304
574513	MIR508	microRNA 508	1.00668	0.977495	0.9982452
594841	WDR45P	WDR45 pseudogene	1.02821	1.04246	1.0876378
594842	HAS2-AS1	HAS2 antisense RNA 1 (non-protein coding)	0.99214	1.012818	1.0727679
594855	CPLX3	complexin 3	1.02122	0.997822	1.0401616

594857	NPS	neuropeptide S	0.99767	1.016499	0.9662662
606293	KLKP1	kallikrein pseudogene 1	0.99775	0.993072	1.0244417
606495	CYB5RL	cytochrome b5 reductase-like	1.01278	0.990888	0.9997713
606551	UBE2MP1	ubiquitin-conjugating enzyme E2M pseudogene 1	0.94156	0.955313	1.0787342
613204	IFITM8P	interferon induced transmembrane protein 8 pseudogene	0.99457	1.036516	1.1214644
613209	DEFB135	defensin, beta 135	1.01726	1.00997	1.0151171
613211	DEFB134	defensin, beta 134	0.99443	1.052082	1.0092446
613212	CTXN3	cortexin 3	1.00404	0.977811	1.0180502
613227	HIGD1C	HIG1 hypoxia inducible domain family, member 1C	1.00206	1.030888	1.0211467
619189	SERINC4	serine incorporator 4	0.99869	1.000262	0.9778859
619207	LOC619207	scavenger receptor protein family member	0.99157	0.983938	1.0369882
619208	C6orf225	chromosome 6 open reading frame 225	0.98886	0.940813	0.9763007
619343	C8orf68	chromosome 8 open reading frame 68	0.99973	1.012048	1.0005765
619373	MBOAT4	membrane bound O-acyltransferase domain containing 4	1.00913	1.006004	1.0293151
619383	SCARNA9	small Cajal body-specific RNA 9	0.97994	1.044652	1.0878033
619450	ATP6V1G1I	ATPase, H+ transporting, lysosomal 13kDa, V1 subunit G1 pseudogene 2	1.00983	1.072963	1.0872396
619455	CXorf18	chromosome X open reading frame 18	1.03494	0.948055	1.0590368
619518	C1orf191	chromosome 1 open reading frame 191	0.98849	0.992817	0.9578147
641311	RPL31P11	ribosomal protein L31 pseudogene 11	1.03355	0.987724	1.0391387
641339	ZNF674	zinc finger protein 674	0.98381	0.968116	1.0192971
641367	LOC641367	cyclin Y-like pseudogene	1.01548	1.036104	1.0199043
641371	ACOT1	acyl-CoA thioesterase 1	0.99699	1.007818	1.0136843
641372	ACOT6	acyl-CoA thioesterase 6	0.99274	1.01762	0.9418934
641378	LOC641378	ADP-ribosylation factor-like 5A pseudogene	0.98768	1.034118	0.9960817
641379	LOC641379	ADP-ribosylation factor-like 5A pseudogene	1.01415	0.943428	0.9961434
641457	LOC641457	CRIP1 pseudogene	0.9293	0.960726	1.0759564
641515	LOC641515	hypothetical LOC641515	1.00052	1.01391	1.0544151
641516	KC6	keratoconus gene 6	0.98486	0.992263	0.9849428
641518	LOC641518	hypothetical LOC641518	0.95161	0.954932	0.8800457
641522	[No Symbo	[No Name]	0.94848	0.956435	0.9862222
641614	RPL23AP8	ribosomal protein L23a pseudogene 8	0.94721	0.99005	1.0940802
641638	SNHG6	small nucleolar RNA host gene 6 (non-protein coding)	0.93353	0.968591	0.9904048
641649	TMEM91	transmembrane protein 91	0.97072	0.943121	1.1080572

641654	HEPN1	hepatocellular carcinoma, down-regulated 1	0.97361	1.037924	0.9646745
641695	LOC641695	reticulon 4 interacting protein 1 pseudogene	0.99818	1.039574	1.0218138
641700	ECSCR	endothelial cell-specific chemotaxis regulator	1.00704	0.958337	0.9635053
641705	[No Symbo	[No Name]	0.95852	0.901725	1.0406371
641708	LOC641708	hydroxysteroid (17-beta) dehydrogenase 12 pseudogene	1.00633	0.960469	1.0400462
641765	[No Symbo	[No Name]	1.01212	1.015432	1.0223153
641842	SLC23A4	solute carrier family 23 (nucleobase transporters), member 4	1.00781	0.976942	1.0122622
641977	SEPT7P2	septin 7 pseudogene 2	1.0274	0.92036	0.9212628
642006	LOC642006	glucuronidase, beta pseudogene	1.01214	1.043315	1.0030193
642007	LOC642007	glucuronidase, beta pseudogene	1.00915	0.957706	1.0158993
642137	[No Symbo	[No Name]	0.98985	0.987706	1.0309321
642259	GAPDHP70	glyceraldehyde 3 phosphate dehydrogenase pseudogene 70	0.98561	0.961421	1.0315873
642262	LOC642262	nuclear receptor coactivator 4 pseudogene	0.98574	1.045173	1.0048375
642273	FAM110C	family with sequence similarity 110, member C	0.96712	1.000665	1.0682899
642280	ZNF876P	zinc finger protein 876, pseudogene	0.98702	0.96628	0.8997578
642290	LOC642290	solute carrier family 25 (mitochondrial carrier; citrate transporter), member 1 pseudogene	0.98029	1.042978	0.9890669
642305	MAPRE1P2	MAPRE1 pseudogene 2	0.99567	0.980644	1.1813515
642306	RPL19P15	ribosomal protein L19 pseudogene 15	0.96437	1.027995	1.1053224
642316	FLJ43763	hypothetical LOC642316	1.02667	0.9675	0.9783302
642319	LOC642319	transgelin 2 pseudogene	1.00378	1.024254	1.0107752
642326	CCNYL3	cyclin Y-like 3	0.98525	1.003303	1.0021155
642329	[No Symbo	[No Name]	0.98583	1.043976	1.0329622
642337	FTLP17	ferritin, light polypeptide pseudogene 17	0.99379	0.999171	0.9150582
642340	LOC642340	hypothetical LOC642340	1.00433	1.003596	0.9991114
642343	[No Symbo	[No Name]	0.9889	1.010763	0.9804733
642355	LOC642355	nucleoside diphosphate kinase, mitochondrial-like	0.98459	1.018246	1.0998986
642357	LOC642357	eukaryotic translation elongation factor 1 gamma pseudogene	0.97368	1.104757	0.9793624
642366	LOC642366	hypothetical LOC642366	0.99419	0.991276	1.0321861
642381	LOC642381	speckle-type POZ protein-like pseudogene	0.99618	0.994376	0.9981853
642394	NCRNA001	non-protein coding RNA 168	0.99519	0.959083	1.0198414
642443	ADH5P4	alcohol dehydrogenase 5 (class III), chi polypeptide, pseudogene 4	0.96952	1.002846	0.9991757
642448	KRT18P23	keratin 18 pseudogene 23	0.98513	0.995036	0.9821856
642460	ANKRD30B	ankyrin repeat domain 30B pseudogene 1	1.00666	1.029701	1.026441

642461	LOC642461	isoleucyl-tRNA synthetase, mitochondrial-like	0.99848	0.985157	1.0339685
642474	LOC642474	UDP glucuronosyltransferase 2 family, polypeptide A3 pseudogene	1.00993	0.896893	1.0159979
642475	C8orf73	chromosome 8 open reading frame 73	1.01301	0.96472	1.0438748
642484	FLJ45743	hypothetical protein LOC642484	1.0101	1.001964	0.9905584
642487	LOC642487	H1 histone family, member O, oocyte-specific pseudogene	1.00625	1.010688	1.0222761
642489	FKBP1C	FK506 binding protein 1C	1.00692	0.998437	1.0208363
642496	LOC642496	UDP glucuronosyltransferase 2 family, polypeptide A3 pseudogene	1.00355	1.008417	1.0260622
642511	LOC642511	hypothetical protein LOC642511	1.01018	1.027848	1.0077163
642513	LOC642513	BTB/POZ domain-containing protein KCTD9-like	0.97457	0.958548	0.9889202
642515	LOC642515	proline-rich transmembrane protein 1-like	1.0073	0.970736	0.9724342
642533	LOC642533	hypothetical LOC642533	0.98392	0.991792	1.0124297
642544	[No Symbo	[No Name]	1.01707	0.92691	1.0052703
642546	HK2P1	hexokinase 2 pseudogene 1	0.96031	1.002582	0.9952083
642550	LOC642550	v-ral simian leukemia viral oncogene homolog A (ras related) pseudogene	0.97735	0.955962	1.0219767
642554	LOC642554	leucine rich repeat containing 59 pseudogene	1.0117	1.008434	1.002401
642559	POU5F1P3	POU class 5 homeobox 1 pseudogene 3	0.92563	1.081498	1.1083239
642574	LOC642574	IQ motif and ankyrin repeat domain-containing protein LOC642574	1.00662	1.014356	1.0503997
642580	LOC642580	heat shock cognate 71 kDa protein-like	1.00708	0.986375	1.0283122
642585	VDAC1P1	voltage-dependent anion channel 1 pseudogene 1	0.98284	1.006123	1.0007976
642587	LOC642587	NPC-A-5	1.01308	0.992236	1.0048865
642590	LOC642590	spermine synthase pseudogene	0.98395	0.960721	0.9316901
642597	C18orf42	chromosome 18 open reading frame 42	0.99036	1.003603	1.0178765
642614	LOC642614	chromosome 2 open reading frame 69 pseudogene	1.0274	1.00672	1.0149725
642622	LOC642622	hypothetical protein LOC642622	0.98903	0.976014	1.026084
642631	TSPY15P	testis specific protein, Y-linked 15, pseudogene	0.99021	0.944367	0.9769642
642635	LOC642635	tubulin, alpha 4a pseudogene	1.00819	1.058536	1.0867445
642648	LOC642648	hypothetical LOC642648	0.97936	0.979114	1.0225227
642652	LOC642652	retinoic acid receptor responder protein 2-like	0.98784	0.994049	1.082802
642666	[No Symbo	[No Name]	0.99558	1.032287	1.0094225
642677	LOC642677	family with sequence similarity 154, member B pseudogene	0.95355	0.992186	1.0328781
642683	LOC642683	kinesin family member 18B pseudogene	0.98866	1.028116	1.0131033
642692	LOC642692	UPF0709 protein C3orf34-like	0.95998	1.081825	1.1097974
642696	FLJ32790	hCG1983896	1.01097	0.957822	0.9677472

642702	[No Symbo	[No Name]	0.99515	0.978974	0.9704492
642737	LOC642737	SET translocation pseudogene	0.99473	0.962563	1.0186054
642741	RPL3P7	ribosomal protein L3 pseudogene 7	0.98647	0.993345	0.9740194
642746	EIF4A1P9	eukaryotic translation initiation factor 4A1 pseudogene 9	0.98561	0.970249	1.0332444
642757	FLJ32756	hypothetical LOC642757	1.00575	0.961881	1.0179019
642762	LOC642762	hCG2042779	0.99937	0.946919	0.9683636
642776	LOC642776	hypothetical LOC642776	1.00203	1.017965	1.1040593
642791	LOC642791	elongation factor 1-alpha 2-like	0.98667	0.981904	0.9847811
642797	DNAH10OS	dynein, axonemal, heavy chain 10 opposite strand	0.97007	0.95834	1.0115165
642802	CCDC137P	coiled-coil domain containing 137 pseudogene	0.99538	1.017743	0.9855318
642812	API5P1	API5 pseudogene 1	1.03537	0.972496	1.0391693
642818	ST13P21	suppression of tumorigenicity 13 (colon carcinoma) (Hsp70 interacting protein) pseudogene 21	0.9925	0.935226	0.990485
642819	ZNF487P	zinc finger protein 487, pseudogene	0.97995	0.995576	0.960462
642843	CPSF4L	cleavage and polyadenylation specific factor 4-like	1.00161	0.990413	1.038675
642846	LOC642846	DEAD/H (Asp-Glu-Ala-Asp/His) box polypeptide 11-like	0.99943	0.927894	1.0702254
642851	[No Symbo	[No Name]	1.00107	0.988359	1.0358804
642852	LOC642852	hypothetical LOC642852	0.97869	0.964489	0.9732529
642864	SRG7	spermatogenesis-related protein 7	1.00655	1.053532	1.0122595
642869	LOC642869	SET translocation (myeloid leukemia-associated) pseudogene	1.00363	0.985137	1.0294655
642879	SMARCE1P	SWI/SNF related, matrix associated, actin dependent regulator of chromatin, subfamily e, member 1 pseudog	0.98314	1.00394	1.0371651
642890	LOC642890	N(alpha)-acetyltransferase 50, NatE catalytic subunit pseudogene	0.99795	1.018897	0.9744746
642891	LOC642891	hypothetical protein LOC642891	0.97674	1.023445	1.0287687
642897	LOC642897	N-6 adenine-specific DNA methyltransferase 1 (putative) pseudogene	0.98408	0.997268	1.0581558
642907	LOC642907	MKI67 (FHA domain) interacting nucleolar phosphoprotein pseudogene	1.01892	0.982333	1.0091712
642909	LOC642909	C-terminal binding protein 2 pseudogene	1.00538	1.017164	0.9972084
642934	LOC642934	hypothetical LOC642934	0.96763	1.020559	1.0263146
642935	LOC642935	ADAM metallopeptidase with thrombospondin type 1 motif, 7 pseudogene	1.00752	0.985795	1.0228086
642938	FAM196A	family with sequence similarity 196, member A	1.0035	1.00981	1.0051948
642943	LOC642943	family with sequence similarity 75, member A1 pseudogene	1.0203	1.019275	0.9993149
642946	NCRNA002	non-protein coding RNA 292	1.01351	0.996131	1.0004182
642952	SNX18P2	sorting nexin 18 pseudogene 2	1.00472	0.981402	1.0119229
642958	LOC642958	heterogeneous nuclear ribonucleoprotein A1 pseudogene	1.03442	0.986587	1.0277833
642959	ALOX15P2	arachidonate 15-lipoxygenase pseudogene 2	1.0079	1.018517	0.9746127

642968	FAM163B	family with sequence similarity 163, member B	0.99121	0.935047	0.9764502
642976	GRIK1-AS1	GRIK1 antisense RNA 1 (non-protein coding)	0.98143	1.056615	1.0049602
642987	TMEM232	transmembrane protein 232	0.99851	0.988688	1.0078227
642996	NFE2L3P1	nuclear factor (erythroid-derived 2)-like 3 pseudogene 1	0.98239	1.00244	0.9682012
643008	C17orf109	chromosome 17 open reading frame 109	0.95146	0.954096	0.9902225
643014	LOC643014	coiled-coil domain containing 90B pseudogene	0.96151	1.022459	1.0243859
643015	LOC643015	nucleolar protein 11 pseudogene	1.01669	1.006606	1.058272
643022	[No Symbo	[No Name]	1.00392	0.971984	0.9849418
643031	LOC643031	mitochondrially encoded NADH dehydrogenase 5 pseudogene	1.02199	1.019774	1.004723
643037	LOC643037	hypothetical protein LOC643037	0.99948	0.984429	1.0204173
643043	LOC643043	hypothetical LOC643043	0.99374	0.956112	1.0037904
643058	LOC643058	interferon induced transmembrane protein pseudogene	0.96636	0.892486	0.8681113
643064	LOC643064	glutamate decarboxylase 1-like	0.99168	1.00116	1.013562
643067	LOC643067	HORMA domain containing 1 pseudogene	1.01377	1.040786	1.0294316
643072	LOC643072	hypothetical LOC643072	0.98142	0.976236	0.9568139
643085	LOC643085	hypothetical LOC643085	1.00194	0.980212	1.1408269
643095	[No Symbo	[No Name]	1.01737	0.989384	1.0213781
643100	[No Symbo	[No Name]	1.00313	0.965826	1.0440887
643102	[No Symbo	[No Name]	0.98567	1.007496	1.0214973
643114	[No Symbo	[No Name]	1.00366	1.007028	1.0356559
643115	KRT223P	keratin 223 pseudogene	0.985	0.998603	1.0242443
643123	[No Symbo	[No Name]	1.0005	1.001446	0.9724241
643126	LOC643126	putative tripartite motif-containing protein 49B-like	1.00238	0.936877	0.9430924
643143	[No Symbo	[No Name]	1.00314	0.976761	1.0060751
643155	C5orf43	chromosome 5 open reading frame 43	0.97354	0.879781	1.0859788
643160	CYMP	chymosin pseudogene	0.99956	1.001759	1.0474104
643162	DKFZP779L	hypothetical LOC643162	0.92582	0.905599	0.9412156
643167	LOC643167	RNA binding motif protein 39 pseudogene	0.95286	0.994596	0.9706838
643168	LOC643168	Rho GTPase activating protein 5 pseudogene	0.9836	0.98319	0.9562971
643172	LOC643172	neurofilament, heavy polypeptide pseudogene	0.9929	1.023317	1.0476988
643181	LOC643181	fatty acid desaturase 2-like protein-like	0.99965	0.982094	1.0027357
643182	LOC643182	upstream binding transcription factor, RNA polymerase I pseudogene	1.02719	1.013842	0.9986625
643194	[No Symbo	[No Name]	1.00417	0.994969	1.0439076

643201	LOC643201	centrosomal protein 192kDa pseudogene	0.9966	1.013766	0.9908316
643220	RPL3P6	ribosomal protein L3 pseudogene 6	0.9805	1.074253	0.9754276
643226	GRXCR2	glutaredoxin, cysteine rich 2	1.00857	0.97069	1.0239362
643228	GAPDHP30	glyceraldehyde 3 phosphate dehydrogenase pseudogene 30	1.01019	0.974057	1.0044847
643232	LOC643232	nuclear receptor binding factor 2 pseudogene	0.94146	1.007818	1.0348086
643236	TMEM72	transmembrane protein 72	1.00116	0.98863	0.9979763
643244	LOC643244	radixin pseudogene	0.99339	1.015061	1.0192986
643246	MAP1LC3B	microtubule-associated protein 1 light chain 3 beta 2	0.9781	0.992243	0.9679707
643253	CCT6P1	chaperonin containing TCP1, subunit 6 (zeta) pseudogene 1	0.98776	1.106656	1.0194923
643264	RPL21P106	ribosomal protein L21 pseudogene 106	0.98202	0.967117	0.9847786
643276	LOC643276	nucleolar complex associated 2 homolog (S. cerevisiae) pseudogene	1.00464	0.958996	0.9777358
643281	[No Symbo	[No Name]	1.01504	0.970732	0.9823178
643307	LOC643307	actin, beta pseudogene	1.00177	0.974068	1.0080488
643308	LOC643308	ribosomal protein L7 pseudogene	1.0109	1.005822	1.0474619
643309	LOC643309	actin, beta pseudogene	0.99143	0.979059	1.1034742
643310	ST13P18	suppression of tumorigenicity 13 (colon carcinoma) (Hsp70 interacting protein) pseudogene 18	0.95332	1.029182	1.0313427
643311	CT47B1	cancer/testis antigen family 47, member B1	1.01298	0.996339	1.016181
643321	[No Symbo	[No Name]	0.9995	1.002281	1.0225026
643327	LOC643327	hypothetical LOC643327	1.05219	0.959603	1.0183012
643329	LOC643329	keratin 18 pseudogene	1.01785	0.986078	0.9363099
643330	[No Symbo	[No Name]	1.02242	0.996556	0.9767626
643332	ECRP	ribonuclease, RNase A family, 2 (liver, eosinophil-derived neurotoxin) pseudogene	0.9294	0.898456	1.0361003
643336	CDC42P2	cell division cycle 42 pseudogene 2	1.00473	1.027013	0.9835069
643338	C15orf62	chromosome 15 open reading frame 62	0.99959	0.979292	1.0509567
643339	LOC643339	hypothetical protein LOC643339	0.998	0.98335	1.0002332
643342	LOC643342	ATM interactor pseudogene	1.0156	1.017075	1.0096283
643348	LOC643348	ankyrin repeat domain 26 pseudogene	0.95379	0.882858	1.0294232
643353	LOC643353	C9orf140 pseudogene	1.00421	1.024679	1.0906046
643355	LOC643355	hypothetical LOC643355	1.00935	0.983805	1.0481548
643358	RPS27AP16	ribosomal protein S27a pseudogene 16	0.95063	1.062322	1.09466
643365	FLJ44054	hypothetical LOC643365	0.99673	1.00677	1.015257
643370	[No Symbo	[No Name]	1.01926	1.096723	0.9546217
643376	BTBD18	BTB (POZ) domain containing 18	0.9899	1.02371	1.0129206

643381	LOC643381	zinc finger protein 614 pseudogene	0.97899	1.054138	1.0210345
643382	C14orf176	chromosome 14 open reading frame 176	1.00495	0.991873	1.0000448
643386	[No Symbo	[No Name]	0.97254	1.024217	1.0408535
643387	LOC643387	TAR DNA binding protein pseuodgene	0.98982	0.992125	1.0582822
643394	SPINK9	serine peptidase inhibitor, Kazal type 9	0.99248	1.028194	1.0113864
643406	LOC643406	hypothetical LOC643406	1.00556	1.004097	1.046664
643414	LIPK	lipase, family member K	1.00358	0.998839	1.0076364
643418	LIPN	lipase, family member N	0.94427	1.072135	0.9696798
643421	LOC643421	tumor protein, translationally-controlled 1 pseudogene	0.97381	1.023459	0.9858705
643438	LOC643438	chromosome 15 open reading frame 63 pseudogene	1.00028	0.967674	1.1227552
643441	LOC643441	hypothetical LOC643441	1.00401	0.99232	1.0039801
643445	LOC643445	tripartite motif containing 43 pseudogene	1.04233	1.078173	1.0298921
643446	LOC643446	RNA binding protein S1, serine-rich domain pseudogene	1.02596	0.987703	0.9905039
643447	LOC643447	keratin 8 pseudogene	0.98682	1.014682	0.9818849
643449	[No Symbo	[No Name]	1.02196	1.048524	1.0211638
643459	LOC643459	dual specificity phosphatase 5 pseudogene	0.97489	0.992698	1.0745234
643470	SURF6P1	surfeit 6 pseudogene 1	0.98837	1.020212	1.027187
643471	KRT18P12	keratin 18 pseudogene 12	1.0022	1.000083	1.0418683
643486	LOC643486	bromodomain, testis-specific pseudogene	0.97345	0.990215	1.0072134
643494	LOC643494	RNA binding motif protein 4B pseudogene	0.97179	1.056322	1.0583664
643496	LOC643496	hCG1660379	1.00069	1.020249	1.0352143
643499	RPL11P5	ribosomal protein L11 pseudogene 5	0.99869	0.905884	1.0212521
643503	TARDBPP1	TAR DNA binding protein pseudogene 1	0.99679	1.018135	0.9851534
643513	LOC643513	karyopherin alpha 2 (RAG cohort 1, importin alpha 1) pseudogene	1.0111	0.981437	1.0252628
643517	[No Symbo	[No Name]	0.97384	0.974517	1.0170246
643529	LOC643529	hCG2024094	0.97182	1.00577	1.0159073
643536	VDAC1P10	voltage-dependent anion channel 1 pseudogene 10	1.01047	0.977554	1.0505429
643550	RPL12P33	ribosomal protein L12 pseudogene 33	0.95492	1.080389	1.0366576
643562	LOC643562	Rho GTPase activating protein 21 pseudogene	1.00452	0.944548	1.0117977
643563	EEF1A1P15	eukaryotic translation elongation factor 1 alpha 1 pseudogene 15	0.97541	1.027416	0.9498412
643582	LOC643582	aldo-keto reductase family 1, member B10 (aldose reductase) pseudogene	1.0289	1.000429	1.0298746
643586	LOC643586	pyruvate kinase, muscle pseudogene	1.03724	0.978244	0.9916352
643596	RNF224	ring finger protein 224	0.97357	0.980604	0.9922873

643605	LOC643605	CTD (carboxy-terminal domain, RNA polymerase II, polypeptide A) small phosphatase like 2 pseudogene	1.00068	1.059031	1.0367875
643609	NR1H5P	nuclear receptor subfamily 1, group H, member 5, pseudogene	1.00892	0.972956	1.0235983
643613	[No Symbo	[No Name]	1.01269	1.018602	1.0626023
643617	RPSAP8	ribosomal protein SA pseudogene 8	0.94769	1.00082	1.1001348
643618	[No Symbo	[No Name]	0.99783	1.008709	1.0400142
643623	LOC643623	hypothetical LOC643623	1.01601	0.966308	0.9960152
643637	GLYATL1P4	glycine-N-acyltransferase-like 1 pseudogene 4	1.00298	1.0334	1.0625989
643641	ZNF862	zinc finger protein 862	0.96953	0.93752	0.9591758
643646	HSD17B1P1	hydroxysteroid (17-beta) dehydrogenase 1 pseudogene 1	0.98152	0.978851	1.0348158
643653	RPL35P8	ribosomal protein L35 pseudogene 8	0.98534	1.016593	0.9814032
643654	[No Symbo	[No Name]	0.95794	0.976349	1.0643919
643664	AMAC1L3	acyl-malonyl condensing enzyme 1-like 3	1.00353	0.986714	0.9320479
643668	PSMC1P9	proteasome (prosome, macropain) 26S subunit, ATPase, 1 pseudogene 9	0.94325	0.919837	1.036606
643669	LOC643669	hypothetical protein LOC643669	1.02356	0.986153	0.9840894
643675	LOC643675	hCG2045751	1.01582	0.969678	1.0102873
643680	MS4A4E	membrane-spanning 4-domains, subfamily A, member 4E	1.03968	0.91557	1.0207634
643684	[No Symbo	[No Name]	1.00992	1.009544	1.0562289
643689	HNRNPA3P	heterogeneous nuclear ribonucleoprotein A3 pseudogene 3	1.01211	1.225555	1.248356
643707	LOC643707	golgi autoantigen, golgin subfamily a, 6 pseudogene	0.98511	1.049028	0.9798178
643709	LOC643709	macrophage expressed 1 pseudogene	1.03301	0.988244	0.987978
643714	LOC643714	hypothetical LOC643714	1.01124	0.998397	1.0146612
643717	[No Symbo	[No Name]	0.98981	0.985246	1.0213342
643718	[No Symbo	[No Name]	0.99662	0.990156	0.9817325
643719	LOC643719	hypothetical LOC643719	1.01483	0.969806	1.0211731
643723	LOC643723	hypothetical LOC643723	0.97768	0.896804	0.8986301
643733	LOC643733	caspase 4, apoptosis-related cysteine peptidase pseudogene	0.98588	1.017563	0.9647967
643736	LCA10	lung carcinoma-associated protein 10	0.97996	0.969281	1.0109102
643739	GAPDHP31	glyceraldehyde 3 phosphate dehydrogenase pseudogene 31	0.99983	0.99642	1.0295138
643744	HMGN2P4	high mobility group nucleosomal binding domain 2 pseudogene 4	0.99012	0.961016	1.221021
643749	TRAF3IP2-A	TRAF3IP2 antisense RNA 1 (non-protein coding)	1.01091	0.962182	0.9786156
643751	LOC643751	cell division cycle 42 pseudogene	1.00881	1.000185	1.0056454
643766	PSMC1P6	proteasome (prosome, macropain) 26S subunit, ATPase, 1 pseudogene 6	0.97232	1.014082	1.0096545
643768	[No Symbo	[No Name]	0.98419	1.000419	1.0402418

643770	LOC643770	hypothetical LOC643770	0.98677	0.949606	1.0258951
643773	PNPLA10P	patatin-like phospholipase domain containing 10 pseudogene	0.97355	1.019425	1.0151465
643779	RPLP0P5	ribosomal protein, large, P0 pseudogene 5	1.00735	0.976379	1.0813724
643783	LOC643783	hypothetical LOC643783	0.97679	0.986036	1.0969238
643797	LOC643797	AGVR6190	1.00529	1.011436	1.0808821
643802	LOC643802	u3 small nucleolar ribonucleoprotein protein MPP10-like	0.94	0.93198	1.0183255
643803	KRTAP24-1	keratin associated protein 24-1	1.01766	0.951107	0.9921159
643806	LOC643806	hypothetical protein LOC643806	1	1.004787	0.9988499
643810	LOC643810	olfactomedin-4-like	0.99649	1.006888	0.9384537
643832	GAGE10	G antigen 10	1.00797	0.972044	0.9761069
643836	ZFP62	zinc finger protein 62 homolog (mouse)	0.94916	0.972024	0.9799985
643855	LOC643855	G1 to S phase transition pseudogene	1.01521	0.981135	1.0622277
643858	KRT18P30	keratin 18 pseudogene 30	0.9938	1.040697	1.0282644
643859	FEM1AP3	fem-1 homolog a (C. elegans) pseudogene 3	0.99277	0.963675	1.0297145
643863	RPL17P34	ribosomal protein L17 pseudogene 34	0.97641	0.98939	1.084824
643865	KRT126P	keratin 126 pseudogene	1.02351	1.011473	1.012263
643866	CBLN3	cerebellin 3 precursor	1.01086	1.057704	1.0058036
643870	LOC643870	translationally-controlled tumor protein-like	0.98755	0.99924	1.0466503
643875	CNN3P1	calponin 3, acidic pseudogene 1	0.98853	0.931032	0.9793749
643877	LOC643877	t-complex protein 11 homolog	0.99924	1.017149	0.9798098
643884	LOC643884	suppressor of cytokine signaling 5 pseudogene	1.00816	1.019335	1.0181951
643898	ARL2BPP2	ADP-ribosylation factor-like 2 binding protein pseudogene 2	0.9735	0.931645	1.0120917
643904	RNF222	ring finger protein 222	0.97731	0.933985	1.0242093
643906	RPL7P27	ribosomal protein L7 pseudogene 27	1.00417	0.951301	1.0447538
643911	CRNDE	colorectal neoplasia differentially expressed (non-protein coding)	1.00235	1.006946	1.0440644
643916	LOC643916	RAB1A, member RAS oncogene family pseudogene	1.01417	1.00907	1.0768095
643926	[No Symbo	[No Name]	0.99777	1.015158	0.9740846
643932	RPS3AP20	ribosomal protein S3a pseudogene 20	0.90246	1.002037	0.9449984
643941	PHKG1P1	phosphorylase kinase, gamma 1 pseudogene 1	1.01224	1.054695	1.0183774
643949	RPLP2P3	ribosomal protein, large, P2 pseudogene 3	0.99005	0.934649	0.9533671
643951	[No Symbo	[No Name]	0.95025	0.895703	0.9968937
643954	RPSAP43	ribosomal protein SA pseudogene 43	0.95792	0.910815	0.9991352
643962	LOC643962	hypothetical LOC643962	1.00151	0.986211	0.9984265

643965	TMEM88B	transmembrane protein 88B	1.0137	1.011455	1.0133538
643972	[No Symbo	[No Name]	0.98291	0.998214	1.0161883
643974	RPL6P13	ribosomal protein L6 pseudogene 13	1.00348	0.999044	1.0517747
643976	LOC643976	forkhead box N3 pseudogene	0.96008	0.989982	1.0033693
643977	FLJ32255	hypothetical LOC643977	0.98568	0.824737	0.9103287
643981	RPS3AP36	ribosomal protein S3a pseudogene 36	0.96403	0.977091	1.0113773
643987	HSFY4P	heat shock transcription factor, Y-linked 4, pseudogene	0.96783	1.005342	0.966946
643988	LOC643988	hypothetical LOC643988	0.98779	1.020308	0.984078
643989	[No Symbo	[No Name]	1.01532	0.97168	1.0342751
643992	RPS2P26	ribosomal protein S2 pseudogene 26	0.98218	1.013118	1.0764604
643996	VDAC2P2	voltage-dependent anion channel 2 pseudogene 2	0.98932	1.25202	0.8817232
643997	LOC643997	peptidylprolyl isomerase A (cyclophilin A) pseudogene	1.01977	0.91523	1.1306721
644006	LOC644006	ring finger protein 4 pseudogene	1.02052	0.955278	1.0638567
644013	RPS6P3	ribosomal protein S6 pseudogene 3	1.01594	0.975974	0.9436163
644016	HSPD1P10	heat shock 60kDa protein 1 (chaperonin) pseudogene 10	1.00633	1.011693	1.0202973
644022	RPL22P19	ribosomal protein L22 pseudogene 19	0.9647	0.880068	0.9914377
644030	LOC644030	keratin, type I cytoskeletal 18-like	1.01031	1.032495	0.9494727
644035	NONOP1	non-POU domain containing, octamer-binding pseudogene 1	0.91132	0.891016	1.0684199
644037	HNRNPA1P	heterogeneous nuclear ribonucleoprotein A1 pseudogene 12	0.98486	1.056893	1.0697027
644041	C18orf63	chromosome 18 open reading frame 63	0.99708	1.017867	1.0009493
644042	SLC25A5P6	solute carrier family 25 (mitochondrial carrier; adenine nucleotide translocator), member 5 pseudogene 6	1.00087	1.02436	1.0405812
644050	LOC644050	hypothetical LOC644050	0.98077	0.973352	1.0290781
644051	LOC644051	isocitrate dehydrogenase pseudogene	1.02175	0.957735	1.0044355
644056	[No Symbo	[No Name]	1.0168	1.026431	0.9676474
644058	LOC644058	hCG2045048	1.02741	0.94356	1.0334991
644060	LOC644060	IGF-II mRNA-binding protein 2-like	0.96231	1.088452	1.0262092
644063	LOC644063	heterogeneous nuclear ribonucleoprotein K pseudogene	0.98114	0.951056	1.0800168
644070	LOC644070	germ cell-specific gene 1-like protein 2-like	1.02119	0.988501	1.0187466
644076	GLYCAM1	glycosylation dependent cell adhesion molecule 1 (pseudogene)	0.98424	0.996969	0.9672863
644082	RPL18AP8	ribosomal protein L18a pseudogene 8	0.97803	0.928215	1.0832251
644083	LOC644083	hypothetical LOC644083	1.03591	0.963665	1.023753
644090	LOC644090	hypothetical LOC644090	0.98943	1.006584	0.9554534
644093	LOC644093	hCG2040054	0.99201	1.002112	1.0422937

644094	PSMC1P12	proteasome (prosome, macropain) 26S subunit, ATPase, 1 pseudogene 12	1.0315	1.00106	0.9870251
644096	SDHAF1	succinate dehydrogenase complex assembly factor 1	0.96601	0.919238	1.0997982
644101	LOC644101	chromobox homolog 3 pseudogene	0.99566	1.027503	1.0719414
644109	RPSAP50	ribosomal protein SA pseudogene 50	0.99294	0.946527	1.020583
644128	RPL23AP53	ribosomal protein L23a pseudogene 53	1.04524	0.92723	0.9827812
644129	LOC644129	hypothetical protein LOC644129	1.0245	1.018745	1.0210715
644135	LOC644135	hypothetical LOC644135	0.98681	1.024903	0.978587
644138	GOT2P3	glutamic-oxaloacetic transaminase 2 pseudogene 3	0.982	1.000965	1.0044513
644139	PIRT	phosphoinositide-interacting regulator of transient receptor potential channels	1.03083	1.001339	0.9759957
644145	LOC644145	exocyst complex component 1 pseudogene	0.99151	1.046516	1.0355508
644146	RPS14P8	ribosomal protein S14 pseudogene 8	0.97453	1.045285	1.0819443
644150	WIPF3	WAS/WASL interacting protein family, member 3	1.00732	1.002992	0.9971166
644154	RPL10AP8	ribosomal protein L10a pseudogene 8	1.00873	1.021949	1.0183301
644158	LOC644158	leucine rich repeat containing 2 pseudogene	0.99383	0.965153	1.0486025
644159	LOC644159	solute carrier family 25 (mitochondrial carrier; citrate transporter), member 1 pseudogene	1.01225	1.064653	1.0557951
644160	LOC644160	actin, gamma 1 pseudogene	0.96582	0.919506	1.0429032
644168	DRGX	dorsal root ganglia homeobox	0.98229	1.019531	1.0121281
644169	LOC644169	voltage-dependent anion-selective channel protein 1-like	1.02225	0.968935	0.9175794
644171	[No Symbo	[No Name]	1.00279	0.995139	0.9788416
644173	LOC644173	hypothetical LOC644173	0.98866	1.00431	1.0607496
644186	C22orf41	chromosome 22 open reading frame 41	0.968	0.967559	0.9666295
644189	LOC644189	acyl-CoA thioesterase 4 pseudogene	0.97558	0.999099	1.0943056
644196	[No Symbo	[No Name]	1.00248	0.986875	1.0031783
644198	RPLP2P2	ribosomal protein, large, P2 pseudogene 2	0.94142	0.92646	1.0667271
644199	[No Symbo	[No Name]	0.99316	1.041304	1.025449
644202	RPL12P27	ribosomal protein L12 pseudogene 27	0.95641	0.982898	1.0345919
644203	LOC644203	glutaryl-CoA dehydrogenase, mitochondrial-like	1.00988	0.962636	1.0425006
644213	GAPDHP32	glyceraldehyde 3 phosphate dehydrogenase pseudogene 32	0.99081	0.949303	1.0185082
644214	PHBP11	prohibitin pseudogene 11	1.00483	0.989433	1.0534804
644220	ACTBP11	actin, beta pseudogene 11	0.99866	1.002991	1.0509736
644222	[No Symbo	[No Name]	1.0244	0.957892	0.9736082
644223	RPL9P4	ribosomal protein L9 pseudogene 4	1.01029	0.945576	1.0158138
644232	[No Symbo	[No Name]	0.99594	0.966959	0.9764282

644233	LOC644233	aurora kinase B pseudogene	0.98199	0.996749	1.0247043
644237	GAPDHP33	glyceraldehyde 3 phosphate dehydrogenase pseudogene 33	1.00546	1.009714	1.0200689
644248	LOC644248	hypothetical LOC644248	1.00269	1	0.9968317
644261	LOC644261	mitochondrial carrier triple repeat 6 pseudogene	0.98524	1.029627	1.1124157
644269	LOC644269	G protein pathway suppressor 1 pseudogene	0.98573	0.979308	1.0285516
644277	LOC644277	hypothetical LOC644277	1.00799	0.974366	1.0119156
644280	[No Symbo	[No Name]	0.99137	1.016981	0.9866415
644285	LOC644285	hypothetical LOC644285	0.98085	0.987827	1.0040051
644303	LOC644303	ATP-dependent RNA helicase DDX24-like	0.97536	0.956979	0.9252839
644310	LOC644310	ubiquinol-cytochrome c reductase, complex III subunit X pseudogene	0.99801	1.00891	0.9699328
644314	MT1IP	metallothionein 1I, pseudogene	1.02211	0.972351	0.9665003
644315	RPS7P11	ribosomal protein S7 pseudogene 11	0.95542	1.068487	1.0116341
644325	LOC644325	disintegrin and metalloprotease domain protein pseudogene	0.99475	0.978821	0.9853548
644330	TPM3P5	tropomyosin 3 pseudogene 5	0.93949	0.848062	1.0541826
644333	ATP5F1P3	ATP synthase, H+ transporting, mitochondrial Fo complex, subunit B1 pseudogene 3	0.99671	0.968821	1.0666216
644334	LOC644334	band 4.1-like protein 5-like	1.01649	0.996167	1.0208016
644335	LOC644335	Smg-5 homolog, nonsense mediated mRNA decay factor (C. elegans) pseudogene	1.01592	1.006477	1.0229399
644353	ZCCHC18	zinc finger, CCHC domain containing 18	0.99252	1.012099	1.0004119
644357	LOC644357	eukaryotic translation elongation factor 1 delta (guanine nucleotide exchange protein) pseudogene	0.99072	0.915367	1.0408871
644366	LOC644366	hypothetical protein LOC644366	0.986	1.032509	1.0320683
644369	[No Symbo	[No Name]	1.01075	0.984679	1.0122969
644378	GCNT6	glucosaminyl (N-acetyl) transferase 6	1.00321	0.95931	1.0454631
644383	LOC644383	heat shock transcription factor 2 pseudogene	0.97698	1.024975	0.9952447
644390	LOC644390	heterogeneous nuclear ribonucleoprotein L-like	0.9511	0.980288	1.1457772
644397	LOC644397	leucine-rich repeat-containing protein 37A2-like	0.9696	0.935121	0.9481952
644403	LOC644403	La ribonucleoprotein domain family, member 1B pseudogene	0.99668	1.052988	1.0055734
644415	[No Symbo	[No Name]	1.00134	0.996384	0.9518525
644422	LOC644422	splicing factor, arginine/serine-rich 6 pseudogene	0.99973	1.023485	1.0373214
644436	LOC644436	tubulin, alpha 4a pseudogene	1.00285	0.996639	1.0070446
644442	HMGB3P11	high mobility group box 3 pseudogene 11	0.98442	1.026068	1.0333014
644444	TMEM30C	transmembrane protein 30C	0.98558	1.005015	1.0626596
644449	LOC644449	heterogeneous nuclear ribonucleoprotein H1 (H) pseudogene	0.98569	0.959044	1.0841193
644451	LOC644451	SHQ1 homolog (S. cerevisiae) pseudogene	1.00595	1.00906	1.0239539

644456	LOC644456	IK cytokine, down-regulator of HLA II pseudogene	0.97171	0.98935	1.0105575
644459	LOC644459	BEN domain containing 3 pseudogene	0.98915	0.971661	0.9559705
644464	RPSAP61	ribosomal protein SA pseudogene 61	1.04297	1.035185	0.9522792
644467	[No Symbo	[No Name]	0.99505	1.013814	0.9650948
644489	[No Symbo	[No Name]	1.03144	0.974855	0.9848067
644491	LOC644491	NMDA receptor regulated 2 pseudogene	0.9709	1.007132	1.0467414
644495	LOC644495	heat shock protein 90kDa alpha (cytosolic), class B member 1 pseudogene	0.99802	1.00158	1.022659
644498	HMGN2P7	high mobility group nucleosomal binding domain 2 pseudogene 7	0.98867	1.032456	1.1018735
644502	HMGB3P18	high mobility group box 3 pseudogene 18	0.99711	1.039947	0.9962034
644511	RPL13AP6	ribosomal protein L13a pseudogene 6	0.90943	0.959053	1.0116709
644517	LOC644517	family with sequence similarity 86, member A pseudogene	1.0222	1.014812	1.0138565
644518	LOC644518	G antigen family D member 5-like	0.98007	1.049037	0.9982169
644522	RPL31P44	ribosomal protein L31 pseudogene 44	0.96773	1.016605	0.9875913
644524	NKX2-4	NK2 homeobox 4	0.98349	0.981854	1.087088
644538	LOC644538	hypothetical protein LOC644538	0.99844	1.013472	0.9650134
644540	TCEB1P3	transcription elongation factor B (SIII), polypeptide 1 pseudogene 3	1.01509	0.977381	1.0068994
644548	LOC644548	exocyst complex component 5 pseudogene	0.97568	0.971601	0.9797124
644554	LOC644554	hypothetical LOC644554	0.98307	1.046705	1.0404584
644563	LOC644563	general transcription factor IIIC, polypeptide 6, alpha 35kDa pseudogene	0.92508	0.958804	1.0010523
644566	LOC644566	tropomyosin 2 (beta) pseudogene	0.96086	0.939128	1.0172782
644573	LOC644573	signal peptidase complex subunit 2 homolog (S. cerevisiae) pseudogene	1.0147	0.980036	1.0271354
644578	LOC644578	hypothetical protein LOC644578	1.01365	1.004658	1.0550564
644584	LOC644584	Ewing sarcoma breakpoint region 1 pseudogene	0.97701	0.97856	0.9947693
644588	DNAJA1P3	DnaJ (Hsp40) homolog, subfamily A, member 1 pseudogene 3	0.94967	0.995887	1.0805514
644590	[No Symbo	[No Name]	0.97117	1.032588	1.0320754
644596	NCRNA000	non-protein coding RNA 87	1	0.970419	0.9616838
644600	GEMIN8P2	gem (nuclear organelle) associated protein 8 pseudogene 2	1.0165	1.082561	0.9771922
644601	LOC644601	bridging integrator 2 pseudogene	1.02078	0.993796	0.9812581
644604	EEF1A1P12	eukaryotic translation elongation factor 1 alpha 1 pseudogene 12	0.98991	0.981757	0.9979463
644616	LOC644616	interleukin enhancer binding factor 2, 45kDa pseudogene	0.99281	0.987199	0.9969621
644618	[No Symbo	[No Name]	0.99248	0.92475	0.9388014
644620	[No Symbo	[No Name]	1.00228	0.983856	1.0016772
644623	TPTE2P2	transmembrane phosphoinositide 3-phosphatase and tensin homolog 2 pseudogene 2	1.00426	0.98611	0.9862191

644624	[No Symbo	[No Name]	1.02447	1.004368	0.9791857
644638	[No Symbo	[No Name]	1.02674	1.034817	1.0268597
644649	LOC644649	apolipoprotein O pseudogene	0.98905	1.041255	1.0306567
644651	HMGB1P25	high mobility group box 1 pseudogene 25	1.01319	1.019316	1.0165433
644659	LOC644659	high-mobility group box 1 pseudogene	1.00817	1.019687	0.997646
644660	NCRNA002	non-protein coding RNA 255	1.01649	0.950363	0.9878426
644661	LOC644661	importin subunit alpha-2-like	0.99865	0.97644	0.9791087
644662	LOC644662	hypothetical protein LOC644662	1.01434	0.965135	1.0380118
644667	LOC644667	golgi autoantigen, golgin subfamily a, 7 pseudogene	1.01364	1.001717	0.9985892
644669	LOC644669	ankyrin repeat domain 30B pseudogene	0.95225	0.970921	0.9832694
644672	CLDN25	claudin 25	1.00441	1.01521	1.0115247
644681	LOC644681	synovial sarcoma, X breakpoint 2 interacting protein pseudogene	1.00447	1.038468	1.0018109
644682	[No Symbo	[No Name]	1.02567	0.98217	0.9838844
644693	[No Symbo	[No Name]	0.96742	0.955559	0.9438557
644714	LOC644714	hypothetical LOC644714	0.99524	0.988942	0.9998838
644717	LOC644717	putative SAGE1-like protein	1.00822	1.031201	0.9924707
644721	LOC644721	ribosomal protein L7-like 1 pseudogene	0.9885	0.961024	1.0787702
644727	LOC644727	hypothetical LOC644727	1.01082	0.956374	0.9372131
644728	LOC644728	glyceraldehyde-3-phosphate dehydrogenase-like	0.97448	0.994823	1.052277
644739	WASF4P	WAS protein family, member 4, pseudogene	0.98296	0.985652	0.9961565
644743	LOC644743	keratin 8 pseudogene	0.98885	0.961669	0.9963633
644745	HSPD1P4	heat shock 60kDa protein 1 (chaperonin) pseudogene 4	0.96566	1.031498	1.005405
644753	[No Symbo	[No Name]	0.98873	0.975569	0.9990211
644759	LOC644759	serine protease Desc4 pseudogene	1.02106	0.997831	1.0457402
644776	LOC644776	RAD52 homolog (S. cerevisiae) pseudogene	0.99825	0.987918	1.0441276
644780	[No Symbo	[No Name]	0.99233	0.972568	0.9870206
644790	RPS15AP11	ribosomal protein S15a pseudogene 11	1.00894	1.046854	1.0210025
644791	[No Symbo	[No Name]	0.98558	1.031105	1.011935
644800	LOC644800	Golgin subfamily A member 8A-like	0.9839	1.026303	1.0077074
644809	C15orf56	chromosome 15 open reading frame 56	0.98165	1.096434	0.9643342
644815	FAM83G	family with sequence similarity 83, member G	0.98038	0.993508	1.0224315
644817	[No Symbo	[No Name]	0.97834	0.978083	1.0192774
644838	LOC644838	hypothetical LOC644838	1.00656	0.998306	1.0181284

644841	LOC644841	hypothetical LOC644841	0.97863	1.01113	1.051822
644844	PHGR1	proline/histidine/glycine-rich 1	1.00084	0.990199	1.0287313
644852	LOC644852	hypothetical LOC644852	0.99644	1.0133	1.0543556
644861	NBPF13P	neuroblastoma breakpoint family, member 13, pseudogene	1.01604	0.99299	0.9848901
644862	RPS28P3	ribosomal protein S28 pseudogene 3	1.05051	0.998221	1.0053596
644871	LOC644871	TNF receptor-associated factor 6 pseudogene	0.97489	1.037362	1.0285289
644890	MEIG1	meiosis expressed gene 1 homolog (mouse)	1.01209	1.021146	0.9873238
644892	[No Symbo	[No Name]	0.99128	1.005467	1.0141769
644899	TERF2IPP1	telomeric repeat binding factor 2, interacting protein pseudogene 1	1.02109	1.024675	0.9522198
644903	FLJ38668	hypothetical LOC644903	0.99899	1.005834	1.0786781
644906	LOC644906	malate dehydrogenase 1, NAD (soluble) pseudogene	1.01443	1.019477	1.0327627
644909	LOC644909	ligand of numb-protein X 1 pseudogene	0.99505	0.985113	1.0093072
644911	RPL10P5	ribosomal protein L10 pseudogene 5	1.01234	0.978643	1.0857212
644915	METTL15P2	methyltransferase like 15 pseudogene 2	1.02022	1.007221	1.0541034
644924	LOC644924	glutamic-oxaloacetic transaminase 2, mitochondrial (aspartate aminotransferase 2) pseudogene	0.97236	0.992225	1.0072279
644936	LOC644936	actin, beta pseudogene	1.00979	0.897335	1.077702
644937	RPL10P8	ribosomal protein L10 pseudogene 8	0.97748	1.020679	1.0290862
644941	[No Symbo	[No Name]	1.04593	1.037372	1.0134225
644943	RASSF10	Ras association (RalGDS/AF-6) domain family (N-terminal) member 10	1.00753	1.025707	1.0791633
644949	LOC644949	hypothetical LOC644949	1.00872	0.992741	1.0905209
644950	LOC644950	histone H3.3-like	1.02514	0.942908	1.0293843
644974	ALG1L2	asparagine-linked glycosylation 1-like 2	0.99909	1.004396	1.0044223
644976	[No Symbo	[No Name]	1.00711	0.995731	0.9863257
644988	FAM108A7	family with sequence similarity 108, member A7, pseudogene	0.98314	1.082193	1.0727552
644990	LOC644990	hypothetical LOC644990	0.98293	1	1.0087752
644992	[No Symbo	[No Name]	0.97807	0.960057	1.0095489
644993	[No Symbo	[No Name]	0.98452	1.002741	0.9532141
644994	C5orf50	chromosome 5 open reading frame 50	1.01195	0.954704	1.0043928
644996	RPL32P22	ribosomal protein L32 pseudogene 22	1.00478	0.997847	0.967139
644997	C1orf200	chromosome 1 open reading frame 200	0.99368	1.009695	1.0452792
644998	KRT8P19	keratin 8 pseudogene 19	0.99037	1.005735	1.0113799
645001	LOC645001	heterogeneous nuclear ribonucleoprotein A1-like	0.99971	1.027489	0.9438761
645018	RPS2P20	ribosomal protein S2 pseudogene 20	0.97208	0.929915	1.0242384

645024	GAPDHP59	glyceraldehyde-3-phosphate dehydrogenase pseudogene 59	0.97591	1.01074	0.9763703
645027	EVPLL	envoplakin-like	1.00259	1.012083	0.9829228
645030	LOC645030	hypothetical protein LOC645030	1.01306	0.999831	0.9991927
645053	[No Symbo	[No Name]	1.00924	1.060964	1.0290465
645054	RPL6P4	ribosomal protein L6 pseudogene 4	1.0093	1.05328	1.0064488
645065	LOC645065	ribulose-5-phosphate-3-epimerase pseudogene	0.98265	1.076796	1.0341791
645086	LOC645086	chromosome 11 open reading frame 58 pseudogene	0.97145	0.984846	1.065433
645090	CXorf30	chromosome X open reading frame 30	0.98437	1.003931	0.9868421
645094	LOC645094	myosin, light chain 12B, regulatory pseudogene	0.97575	0.946351	1.0728269
645097	[No Symbo	[No Name]	1.01114	0.988323	0.9982878
645100	LOC645100	hCG1820764	0.99215	1.039092	0.982577
645101	[No Symbo	[No Name]	1.01803	1.048393	0.9610956
645104	CLRN2	clarin 2	1.01615	0.952004	1.059924
645106	[No Symbo	[No Name]	0.96727	1.037405	1.0028632
645118	[No Symbo	[No Name]	0.99936	1.012328	1.0071173
645120	LOC645120	apoptosis-inducing factor, mitochondrion-associated, 1 pseudogene	0.99443	0.965488	0.9449804
645121	CCNI2	cyclin I family, member 2	1.00066	0.951911	0.9981357
645125	[No Symbo	[No Name]	0.97631	1.002492	1.0553804
645136	LOC645136	v-myc myelocytomatosis viral oncogene homolog 1, lung carcinoma derived (avian) pseudogene	0.99538	1.002156	1.0626486
645139	LOC645139	poly(A) binding protein interacting protein 1 pseudogene	0.86056	0.841778	1.0347184
645145	ZBED1P	zinc finger, BED-type containing 1 pseudogene	1.02935	0.994067	1.0011812
645157	RPL21P61	ribosomal protein L21 pseudogene 61	0.99456	1.049453	1.0489588
645161	RPL12P8	ribosomal protein L12 pseudogene 8	0.95314	0.975314	1.0079934
645163	LOC645163	phosphatidylinositol 4-kinase type 2 beta pseudogene	0.98603	1.011915	1.037967
645175	MEMO1P2	mediator of cell motility 1 pseudogene 2	0.99162	0.94622	1.0546767
645177	LOC645177	hypothetical protein LOC645177	0.98575	1.023644	0.9980293
645180	LOC645180	KIAA1539 pseudogene	1.00025	1.010941	0.9997352
645181	LOC645181	PDGFA associated protein 1 pseudogene	1.02751	1.027284	0.9970159
645191	LINGO3	leucine rich repeat and Ig domain containing 3	0.99327	1.032797	1.0655266
645195	LOC645195	hypothetical LOC645195	0.97199	1.002833	0.9699472
645201	HMGB1P26	high mobility group box 1 pseudogene 26	1.01033	0.947559	1.0266099
645203	TMEM14D	transmembrane protein 14D	0.95534	1.017958	1.0210829
645204	LOC645204	heterogeneous nuclear ribonucleoprotein M pseudogene	0.99731	1.037075	1.0230809

645207	LOC645207	eukaryotic translation initiation factor 4E family member 2 pseudogene	1.01348	0.981945	1.0419226
645212	LOC645212	hypothetical LOC645212	0.93738	0.976192	1.0202272
645218	[No Symbo	[No Name]	0.98302	0.937398	0.974887
645220	LOC645220	adiponectin receptor 2 pseudogene	0.9427	0.957081	1.0205591
645221	LOC645221	NADH-ubiquinone oxidoreductase chain 1-like	1.00227	0.992593	1.000938
645227	[No Symbo	[No Name]	1.0295	0.998027	1.061072
645233	LOC645233	thymine-DNA glycosylase pseudogene	0.86789	0.946458	1.0159325
645249	LOC645249	hypothetical LOC645249	1.00679	0.984878	1.0198776
645251	CBX1P1	chromobox homolog 1 pseudogene 1	0.99388	0.996067	0.9942552
645256	GAPDHP66	glyceraldehyde-3-phosphate dehydrogenase pseudogene 66	1.00113	0.977731	1.0613722
645264	LOC645264	oligosaccharyltransferase complex subunit OSTC-like	1.03202	0.98292	1.0639158
645266	LOC645266	PRP4 pre-mRNA processing factor 4 homolog B (yeast) pseudogene	0.99522	0.974096	1.0431444
645272	LOC645272	S-adenosylmethionine decarboxylase pseudogene	1.00174	1.025972	0.9917808
645273	[No Symbo	[No Name]	1.03704	0.962037	1.0208121
645277	LOC645277	hypothetical LOC645277	0.97955	0.967958	1.0095655
645280	SMPD4P1	sphingomyelin phosphodiesterase 4, neutral membrane (neutral sphingomyelinase-3) pseudogene 1	0.99276	1.005091	0.9871801
645294	LOC645294	RING finger and transmembrane domain-containing protein 1-like	1.01487	0.972227	1.0079895
645297	LOC645297	MAS-related GPR, member X2 pseudogene	1.02203	0.968812	1.0434189
645301	[No Symbo	[No Name]	1.00263	1.015886	1.1001266
645311	LOC645311	eukaryotic translation elongation factor 1 gamma pseudogene	0.99115	1.02029	1.007038
645313	SCDP1	stearoyl-CoA desaturase (delta-9-desaturase) pseudogene 1	1.01218	0.969741	1.0233095
645314	LOC645314	DnaJ (Hsp40) homolog, subfamily C, member 17 pseudogene	0.99209	1.025966	1.0228142
645317	CHCHD2P6	coiled-coil-helix-coiled-coil-helix domain containing 2 pseudogene 6	0.96651	0.963506	0.9981713
645318	LOC645318	prohibitin 2 pseudogene	1.00434	0.999576	1.0301939
645319	LOC645319	MAS-related GPR, member X3 pseudogene	1.01807	0.970045	0.9522826
645321	LOC645321	hypothetical LOC645321	1.01436	0.982223	0.9752718
645323	LOC645323	hypothetical LOC645323	1.00381	0.98555	1.0221649
645324	LOC645324	GrpE-like 1, mitochondrial (E. coli) pseudogene	0.97644	1.033711	1.0320006
645326	RPSAP11	ribosomal protein SA pseudogene 11	0.97451	1.002647	1.0325166
645328	LOC645328	mitochondrial ribosomal protein L40 pseudogene	0.99927	1.011464	0.9792098
645332	LOC645332	family with sequence similarity 86, member A pseudogene	0.95148	0.935539	1.0662808
645339	SNRPD2P2	small nuclear ribonucleoprotein D2 pseudogene 2	1.01368	0.939609	1.0308876
645355	LOC645355	hypothetical LOC645355	1.00303	1.006884	0.98998

645357	LOC645357	kinesin family member pseudogene	0.98415	0.999826	0.9910216
645360	HMGB3P5	high mobility group box 3 pseudogene 5	1.04456	1.024056	1.0322239
645367	GGT8P	gamma-glutamyltransferase 8 pseudogene	1.0048	1.048633	1.0453144
645368	LOC645368	eukaryotic translation initiation factor 3, subunit K pseudogene	1.00659	1.003802	0.9716795
645369	TMEM200C	transmembrane protein 200C	0.98833	1.065371	1.0645161
645381	TLE1P1	transducin-like enhancer of split 1 pseudogene 1	0.9666	0.936593	1.0035556
645388	LOC645388	adaptor-related protein complex 1, mu 2 subunit pseudogene	1.00159	0.993612	0.9742768
645395	LOC645395	uncharacterized protein C9orf78-like	1.00206	0.994338	1.0142906
645397	LOC645397	methylenetetrahydrofolate dehydrogenase (NADP+ dependent) 1-like pseudogene	1.01517	1.028879	1.0097927
645398	LOC645398	MKI67 (FHA domain) interacting nucleolar phosphoprotein pseudogene	1.00324	0.998568	1.0914894
645412	RPS16P9	ribosomal protein S16 pseudogene 9	0.98014	0.984935	1.049033
645415	LOC645415	mas-related G-protein coupled receptor member X3-like	1.02105	0.958952	1.0555673
645422	RPL10P12	ribosomal protein L10 pseudogene 12	0.93191	1.026435	0.8631859
645423	MCART4P	mitochondrial carrier triple repeat 4 pseudogene	1.00415	0.975811	0.9794062
645431	LOC645431	hypothetical LOC645431	0.94451	1.00428	1.0774731
645432	ARRDC5	arrestin domain containing 5	0.96695	1.038011	0.8793466
645433	LOC645433	inositol-3-phosphate synthase 1 pseudogene	0.98596	1.007387	1.0296586
645434	LOC645434	hypothetical LOC645434	0.98669	1.008579	0.9934901
645436	LOC645436	heterogeneous nuclear ribonucleoprotein A1 pseudogene	0.98899	0.939524	1.0398174
645440	ATP5F1P6	ATP synthase, H+ transporting, mitochondrial Fo complex, subunit B1 pseudogene 6	0.99959	0.951331	1.0641307
645443	ATP5A1P3	ATP synthase, H+ transporting, mitochondrial F1 complex, alpha subunit 1 pseudogene 3	0.99552	0.964075	1.0312043
645446	LOC645446	hypothetical LOC645446	0.97125	0.975639	0.9629188
645452	RPL21P68	ribosomal protein L21 pseudogene 68	1.02006	0.998418	1.0077507
645453	RLIMP3	ring finger protein, LIM domain interacting pseudogene 3	0.98827	0.995935	1.0016951
645460	FLJ44342	hypothetical LOC645460	0.98755	1.136415	0.9869438
645470	[No Symbo	[No Name]	0.96104	1.003269	0.9581677
645474	S100A11P3	S100 calcium binding protein A11 pseudogene 3	1.03926	1.021389	1.0001745
645478	[No Symbo	[No Name]	1.00282	1.019132	1.0393258
645479	[No Symbo	[No Name]	1.0041	1.076219	0.9063019
645481	LOC645481	zinc finger protein 248 pseudogene	0.97774	1.039185	1.0237043
645486	LOC645486	adenosylhomocysteinase pseudogene	0.99789	1.003185	1.0045451
645490	[No Symbo	[No Name]	0.98632	1.010107	0.9905995
645513	LOC645513	hypothetical LOC645513	0.97688	0.950651	0.9776878

645528	NCRNA002	non-protein coding RNA 264	0.99574	0.966289	1.0158994
645538	GOT2P1	glutamic-oxaloacetic transaminase 2 pseudogene 1	1.01356	0.964238	0.972098
645548	HSPD1P6	heat shock 60kDa protein 1 (chaperonin) pseudogene 6	1.00121	0.961451	1.0326427
645551	LOC645551	glycoprotein-N-acetylgalactosamine 3-beta-galactosyltransferase 1-like	0.99015	1.068306	1.0735239
645560	ATP5C1P1	ATP synthase, H+ transporting, mitochondrial F1 complex, gamma polypeptide 1 pseudogene 1	0.91734	1.052427	1.0471122
645571	LOC645571	chromosome 2 open reading frame 7 pseudogene	1.03308	0.996264	0.9771485
645573	RPS4XP19	ribosomal protein S4X pseudogene 19	0.96234	0.900658	1.0905128
645581	[No Symbo	[No Name]	0.99544	0.943295	1.0212709
645586	LOC645586	hypothetical LOC645586	0.98716	1.024086	1.0125121
645598	LOC645598	widely interspaced zinc finger motifs pseudogene	1.01339	0.99929	1.0120183
645605	[No Symbo	[No Name]	0.98377	0.983727	1.0253143
645615	[No Symbo	[No Name]	0.96901	1.005674	0.9997736
645626	LOC645626	coiled-coil domain containing 29-like	1.00607	1.00434	1.0524845
645630	RPS13P8	ribosomal protein S13 pseudogene 8	0.97214	1.008406	1.0863194
645634	ANO7L1	anoctamin 7-like 1	1.00784	1.015568	1.0125295
645638	LOC645638	WDNM1-like pseudogene	0.99447	0.996153	0.995306
645641	MESTP3	mesoderm specific transcript homolog (mouse) pseudogene 3	1.00984	1.025194	0.9156842
645645	LOC645645	hypothetical protein LOC645645	0.99362	1.048003	1.0045322
645646	FAM32C	family with sequence similarity 32, member C (pseudogene)	0.97805	0.978454	0.9936007
645652	LOC645652	metallothionein-1X-like	1.02478	1.060819	0.9531184
645668	TUFMP1	Tu translation elongation factor, mitochondrial pseudogene 1	1.00686	1.066565	1.0529888
645676	LOC645676	hypothetical LOC645676	0.99681	1.016543	0.9626972
645680	LOC645680	karyopherin alpha 2 pseudogene	1.006	0.962165	1.0228275
645682	POU5F1P4	POU class 5 homeobox 1 pseudogene 4	1.02644	1.05602	0.9493273
645683	RPL13AP3	ribosomal protein L13a pseudogene 3	1.02551	1.008471	0.9850058
645685	[No Symbo	[No Name]	0.98307	0.976593	0.9399926
645687	C14orf34	chromosome 14 open reading frame 34	0.99679	0.965805	0.9940606
645688	RPL12P38	ribosomal protein L12 pseudogene 38	0.99673	0.952377	1.0956142
645693	EEF1A1P22	eukaryotic translation elongation factor 1 alpha 1 pseudogene 22	0.97035	0.94207	1.0366197
645700	ZNF890P	zinc finger protein 890, pseudogene	0.96784	0.983808	1.0613443
645715	EEF1A1P24	eukaryotic translation elongation factor 1 alpha 1 pseudogene 24	0.97893	0.892495	1.021845
645716	[No Symbo	[No Name]	0.97813	1.048557	1.0335501
645726	[No Symbo	[No Name]	1.00137	1.021358	1.0358552

645733	[No Symbo	[No Name]	1.00124	1.000988	0.9861835
645739	LOC645739	hCG1646718	0.99288	1.006349	0.9267928
645740	NOS2P1	nitric oxide synthase 2 pseudogene 1	0.97857	0.96886	1.0174531
645744	LOC645744	TAF5-like RNA polymerase II, p300/CBP-associated factor (PCAF)-associated factor, 65kDa pseudogene	0.98224	0.972725	1.0160921
645745	MT1P2	metallothionein 1 pseudogene 2	1.01128	1.009569	0.9690925
645749	LOC645749	zinc finger protein 131 pseudogene	1.01318	0.992194	1.0283559
645752	LOC645752	golgi autoantigen, golgin subfamily a, 6 pseudogene	0.95743	0.967423	1.0111661
645754	LOC645754	lectin, galactoside-binding, soluble, 9 pseudogene	1.00313	1.011091	1.0053537
645757	[No Symbo	[No Name]	1.02211	0.980294	0.9607489
645769	LOC645769	hypothetical LOC645769	1.00598	0.998177	1.0119708
645771	PARP4P2	poly (ADP-ribose) polymerase family, member 4 pseudogene 2	0.98823	0.922125	1.0117502
645773	LOC645773	tubulin, alpha 4a pseudogene	0.99063	1.088918	0.9581214
645781	[No Symbo	[No Name]	0.98078	0.966	1.0889889
645798	RPL3P3	ribosomal protein L3 pseudogene 3	0.96583	0.951011	1.0241054
645799	[No Symbo	[No Name]	0.9957	0.998972	1.0074271
645805	LOC645805	deleted in azoospermia associated protein 2 pseudogene	0.98827	0.956742	0.9632469
645811	CCDC154	coiled-coil domain containing 154	0.99362	1.015884	1.0548139
645832	SEBOX	SEBOX homeobox	1.02737	1.041091	0.9855236
645840	TXNRD3NB	thioredoxin reductase 3 neighbor	0.9907	0.992131	0.9818591
645841	LOC645841	chromosome 7 open reading frame 42 pseudogene	0.99594	0.963824	1.0430618
645842	[No Symbo	[No Name]	0.96381	1.059913	1.0315558
645843	TMEM14E	transmembrane protein 14E	1.02746	1.029791	0.9464464
645852	LOC645852	splicing factor 3a, subunit 2, 66kDa pseudogene	1.00342	1.008857	1.0229401
645853	LOC645853	apolipoprotein A-I binding protein pseudogene	0.98808	0.96993	1.028835
645857	RPS10P17	ribosomal protein S10 pseudogene 17	0.99718	0.993245	1.0442911
645862	LOC645862	coiled-coil-helix-coiled-coil-helix domain containing 4 pseudogene	0.99698	1.030537	0.9379085
645864	MAGEB17	melanoma antigen family B, 17	0.97988	0.987898	1.0290695
645874	[No Symbo	[No Name]	0.99163	0.976432	0.9748395
645877	LOC645877	acyl-CoA-binding domain-containing protein 7-like	1.03765	0.985463	1.0224524
645884	RPS7P5	ribosomal protein S7 pseudogene 5	0.99245	1.032018	0.9949672
645900	LOC645900	lysosomal protein transmembrane 4 beta pseudogene	0.98877	1.027033	1.0862259
645916	RPS11P2	ribosomal protein S11 pseudogene 2	1.02884	0.990229	1.0036124
645922	S100A7L2	S100 calcium binding protein A7-like 2	1.00953	1.077534	1.0165967

645927	MCFD2P1	multiple coagulation factor deficiency 2 pseudogene 1	1.00825	0.975972	1.0151999
645930	HMGN2P31	high mobility group nucleosomal binding domain 2 pseudogene 31	0.9741	1.022078	1.0118838
645931	FEN1P1	flap structure-specific endonuclease 1 pseudogene 1	1.01517	0.995044	1.020815
645934	[No Symbo	[No Name]	1.01123	0.998563	1.0141815
645938	LOC645938	capping protein (actin filament) muscle Z-line, alpha 1 pseudogene	1.01498	0.985348	1.0200789
645939	PRKRIRP8	protein-kinase, interferon-inducible double stranded RNA dependent inhibitor, repressor of (P58 repressor)	0.98715	1.01633	1.0212461
645940	LOC645940	heterogeneous nuclear ribonucleoprotein A1-like	0.98983	0.976345	0.9994848
645944	[No Symbo	[No Name]	0.9917	0.974703	0.9891164
645949	LOC645949	hypothetical LOC645949	0.99879	1.001088	1.0122276
645950	HNRNPA1P	heterogeneous nuclear ribonucleoprotein A1 pseudogene 1	0.98351	1.032893	0.9666986
645954	LOC645954	supervillin pseudogene	1.00165	0.991987	1.0148196
645955	[No Symbo	[No Name]	1.00544	1.023094	0.997513
645958	RPS18P9	ribosomal protein S18 pseudogene 9	0.92398	0.935584	0.8784508
645960	LOC645960	similar to hCG1644362	0.99843	0.99827	0.9969045
645967	LOC645967	hypothetical LOC645967	0.98561	0.985415	1.072658
645968	RPS3AP25	ribosomal protein S3a pseudogene 25	0.97276	0.98264	1.0108978
645969	LOC645969	selenoprotein T pseudogene	0.98391	1.062484	1.0079939
645971	LOC645971	hypothetical protein LOC645971	0.98933	0.958996	1.0017944
645973	ATP5F1P2	ATP synthase, H+ transporting, mitochondrial Fo complex, subunit B1 pseudogene 2	1.00765	1.015665	1.0310384
645978	LOC645978	alkaline ceramidase 2-like	1.00701	1.016626	0.9876942
645979	RPS26P31	ribosomal protein S26 pseudogene 31	0.99069	1.026994	1.0489593
645996	NAP1L6	nucleosome assembly protein 1-like 6	0.99023	0.949659	0.9797475
646012	LOC646012	aurora kinase A interacting protein 1 pseudogene	1.00221	1.029086	1.0274723
646013	RPS17P3	ribosomal protein S17 pseudogene 3	0.96446	1.060243	0.9791761
646019	CBY3	chibby homolog 3 (Drosophila)	0.98861	1.006152	0.9401095
646021	LOC646021	hypothetical protein LOC646021	0.97685	0.977829	0.9921004
646023	C22orf45	chromosome 22 open reading frame 45	0.99222	1.050446	0.9559297
646024	RAET1K	retinoic acid early transcript 1K pseudogene	0.98458	1.010415	1.014206
646030	LOC646030	leucine-rich repeat-containing protein 37A2-like	1.01308	0.94745	0.9968951
646034	LOC646034	hypothetical LOC646034	0.98473	1.004857	0.9945631
646044	LOC646044	single stranded DNA binding protein 4 pseudogene	0.99048	1.05481	1.0554785
646048	[No Symbo	[No Name]	0.99512	0.975772	1.0451799
646053	LOC646053	nuclear factor NF-kappa-B p100 subunit-like	0.99976	0.988926	0.9969338

646056	[No Symbo	[No Name]	0.93783	0.985862	1.026167
646057	LOC646057	ig heavy chain V region 5A-like	0.98187	0.993443	1.064106
646058	LOC646058	chromosome 4 open reading frame 27 pseudogene	1.00895	1.000916	1.007565
646080	LOC646080	Rab coupling protein pseudogene	0.99769	0.977936	1.0363009
646085	PSMC1P7	proteasome (prosome, macropain) 26S subunit, ATPase, 1 pseudogene 7	1.04728	0.991276	0.9870273
646086	LOC646086	WDR45-like pseudogene	0.97201	1.028784	1.0757931
646087	LOC646087	IMP3, U3 small nucleolar ribonucleoprotein, homolog (yeast) pseudogene	0.98308	1.017364	0.9816525
646090	RHPN2P1	rhophilin, Rho GTPase binding protein 2 pseudogene 1	0.99975	1.053949	1.0603828
646091	[No Symbo	[No Name]	0.99928	0.994256	1.0168771
646093	RPS8P7	ribosomal protein S8 pseudogene 7	1.01214	1.007087	0.9868641
646108	RPS7P14	ribosomal protein S7 pseudogene 14	0.98006	1.034369	0.9991647
646112	LOC646112	origin recognition complex, subunit 3-like (yeast) pseudogene	0.98547	0.991684	1.026936
646114	LOC646114	A kinase (PRKA) anchor protein 8 pseudogene	1.00287	0.952026	1.0770468
646118	[No Symbo	[No Name]	0.99103	1.043212	0.9567037
646119	RPS8P4	ribosomal protein S8 pseudogene 4	1.01636	0.968917	0.9914244
646140	LOC646140	ribosomal protein L7-like 1 pseudogene	0.97046	0.978985	1.0249219
646164	DDX39AP1	DEAD (Asp-Glu-Ala-Asp) box polypeptide 39A pseudogene 1	1.01479	0.961878	1.0020228
646174	C16orf90	chromosome 16 open reading frame 90	1.00472	1.002423	1.0028395
646175	RPL36AP48	ribosomal protein L36a pseudogene 48	0.98464	1.009239	0.9704939
646179	LOC646179	hCG2045614	1.02288	0.988565	0.9730579
646187	[No Symbo	[No Name]	1.01577	1.027398	1.0262042
646189	RPL18AP13	ribosomal protein L18a pseudogene 13	0.99144	1.048761	0.9873969
646193	LOC646193	C5orf44 pseudogene	1.00672	0.995794	1.0070578
646196	SMARCE1P	SWI/SNF related, matrix associated, actin dependent regulator of chromatin, subfamily e, member 1 pseudog	0.98832	0.962934	0.985839
646197	LOC646197	heat shock protein 90kDa alpha (cytosolic), class B member 1 pseudogene	0.98056	1.005453	0.9998326
646201	BASP1P1	brain abundant, membrane attached signal protein 1 pseudogene 1	1.01094	1.060814	0.90818
646203	LOC646203	mRNA turnover 4 homolog (S. cerevisiae) pseudogene	1.01112	0.960625	1.0474214
646208	NUS1P2	nuclear undecaprenyl pyrophosphate synthase 1 homolog (S. cerevisiae) pseudogene 2	0.9754	1.088895	0.9914117
646213	LOC646213	synovial sarcoma translocation gene on chromosome 18-like 2 pseudogene	0.97584	1.010821	1.0087196
646216	LOC646216	ribosomal protein S3a pseudogene	0.97079	0.948497	0.9798742
646218	LOC646218	peroxiredoxin 2 pseudogene	1.01405	0.984957	1.0207512
646227	LOC646227	cAMP-regulated phosphoprotein 19 pseudogene	1.00535	0.983285	1.0514452
646231	AGPAT5P1	1-acylglycerol-3-phosphate O-acyltransferase 5 pseudogene 1	0.98193	1.024176	1.0214498

646249	LOC646249	family with sequence similarity 113, member A pseudogene	1.00913	0.998028	1.0189849
646255	RPL19P16	ribosomal protein L19 pseudogene 16	0.97621	0.971946	0.9611678
646260	SAR1AP1	SAR1 homolog A (S. cerevisiae) pseudogene 1	0.98433	0.964403	1.0386323
646262	LACTBL1	lactamase, beta-like 1	1.01475	0.992476	0.9694679
646268	LOC646268	hCG1654703	1.01654	1.041531	1.0491442
646269	RPS4XP8	ribosomal protein S4X pseudogene 8	0.99673	1.032226	0.9671472
646270	ELL2P1	elongation factor, RNA polymerase II, 2 pseudogene 1	1.00739	1.008544	1.0134831
646272	LOC646272	cytochrome b-c1 complex subunit 8-like	0.9489	0.995104	1.0258344
646273	HSPD1P15	heat shock 60kDa protein 1 (chaperonin) pseudogene 15	0.98872	0.961769	1.0274133
646274	LOC646274	stathmin-like 2 pseudogene	1.03081	0.966372	0.9583281
646282	AZGP1P1	alpha-2-glycoprotein 1, zinc-binding pseudogene 1	0.97898	0.999787	1.0047061
646300	COL6A4P2	collagen, type VI, alpha 4 pseudogene 2	1.00648	0.981694	1.0258076
646316	TERF1P3	telomeric repeat binding factor (NIMA-interacting) 1 pseudogene 3	0.9487	1.104656	0.9882009
646324	LOC646324	hypothetical LOC646324	1.01589	1.01529	1.0326894
646329	LOC646329	hypothetical LOC646329	0.99014	1.081403	0.9302015
646344	LOC646344	similar to hCG2042391	0.94873	0.932322	0.9716037
646346	RPL7P58	ribosomal protein L7 pseudogene 58	0.92391	0.912531	1.0300617
646347	LOC646347	spermine synthase pseudogene	0.97952	0.940929	1.143729
646357	[No Symbo	[No Name]	1.02552	1.046869	0.9914761
646360	LOC646360	transmembrane protein 161B pseudogene	0.98114	0.993391	1.0196402
646362	FTH1P13	ferritin, heavy polypeptide 1 pseudogene 13	0.99751	1.00318	1.0232929
646370	IGHV1OR15	immunoglobulin heavy variable 1/OR15-3 (pseudogene)	0.99709	1.073961	1.0242417
646377	LOC646377	similar to hCG2041495	1.03451	1.110774	0.983693
646378	DDX18P5	DEAD (Asp-Glu-Ala-Asp) box polypeptide 18 pseudogene 5	0.97325	0.982512	1.0266644
646379	IGHV1OR15	immunoglobulin heavy variable 1/OR15-4 (pseudogene)	1.01072	0.979847	1.032494
646390	IRX1P1	iroquois homeobox 1 pseudogene 1	0.99617	1.035704	1.017053
646396	REREP3	arginine-glutamic acid dipeptide (RE) repeats pseudogene 3	0.96792	0.98596	1.2128292
646405	TPTE2P1	transmembrane phosphoinositide 3-phosphatase and tensin homolog 2 pseudogene 1	1.00508	0.9834	1.0177936
646409	DGAT2L7	diacylglycerol O-acyltransferase 2 like protein 7	0.95198	1.058031	1.0466672
646417	RPL29P5	ribosomal protein L29 pseudogene 5	0.9915	0.928065	1.0787707
646424	SPINK8	serine peptidase inhibitor, Kazal type 8 (putative)	1.00995	0.994684	1.0021932
646430	LOC646430	amyloid P component, serum pseudogene	0.99547	0.996812	0.981754
646433	RPL32P19	ribosomal protein L32 pseudogene 19	0.9897	0.970655	1.0148073

646446	RPL27P2	ribosomal protein L27 pseudogene 2	1.01872	1.012138	0.9608605
646450	C3orf71	chromosome 3 open reading frame 71	0.99628	1.023509	1.0465195
646457	C19orf67	chromosome 19 open reading frame 67	1.00202	1.002997	0.967465
646463	UBE2HP1	ubiquitin-conjugating enzyme E2H pseudogene 1	1.02324	0.936499	1.0181913
646467	CNN2P1	calponin 2 pseudogene 1	0.97215	0.946517	1.0073857
646471	LOC646471	hypothetical LOC646471	0.98436	1.003076	0.958096
646479	ADAM24P	ADAM metallopeptidase domain 24, pseudogene	1.00686	1.005964	0.9915063
646480	FABP9	fatty acid binding protein 9, testis	1.00071	1.032054	1.0067308
646482	LOC646482	hypothetical LOC646482	0.99768	1.003369	0.9966792
646483	RPL6P19	ribosomal protein L6 pseudogene 19	1.00969	0.982743	0.8943914
646486	FABP12	fatty acid binding protein 12	1.00017	0.977239	1.0362657
646498	LOC646498	hypothetical protein LOC646498	1.0141	0.969187	0.9964741
646506	LOC646506	lysosomal-associated membrane protein 1 pseudogene	1.02646	1.028976	0.9631459
646508	LOC646508	family with sequence similarity 90, member A1-like	0.98077	1.06641	1.037524
646509	[No Symbo	[No Name]	0.94239	0.953411	1.0444889
646510	[No Symbo	[No Name]	1.00269	1.006892	0.991888
646513	LOC646513	VLGN1945	1.05838	1.072115	0.9333527
646517	LOC646517	pre-mRNA-splicing regulator WTAP-like	0.99263	1.00795	1.0135973
646520	SUCLA2P1	succinate-CoA ligase, ADP-forming, beta subunit pseudogene 1	0.96353	1.00531	0.9327339
646522	LOC646522	hypothetical LOC646522	1.00538	0.993297	1.0258147
646531	YBX1P2	Y box binding protein 1 pseudogene 2	1.02017	0.984047	0.9893461
646532	RPL19P5	ribosomal protein L19 pseudogene 5	0.96554	1.073991	1.1132492
646535	LOC646535	60S ribosomal protein L7a-like	0.97715	0.967857	0.9812279
646538	RPL7P10	ribosomal protein L7 pseudogene 10	0.94801	0.980248	1.0054191
646543	LOC646543	hypothetical protein LOC646543	0.99158	0.998452	0.9999165
646548	LOC646548	ADAM metallopeptidase domain 20 pseudogene	0.98004	1.065368	0.9809287
646555	LOC646555	AT rich interactive domain 3B pseudogene	1.00871	1.044396	1.0670595
646560	[No Symbo	[No Name]	1.00786	1.003391	1.0285382
646567	LOC646567	oligosaccharyltransferase complex subunit OSTC-like	0.98092	0.942982	1.0575631
646572	LOC646572	coiled-coil-helix-coiled-coil-helix domain-containing protein 3, mitochondrial-like	1.01337	0.986922	1.0545781
646575	[No Symbo	[No Name]	1.00355	0.980628	0.9962441
646576	LOC646576	hypothetical LOC646576	1.01174	1.0335	1.0186128
646577	HMGB1P16	high mobility group box 1 pseudogene 16	0.97061	1.000581	1.027801

646581	IFNWP2	interferon, omega 1 pseudogene 2	1.02088	0.972437	1.0094801
646583	RPS4XP18	ribosomal protein S4X pseudogene 18	0.99165	0.96474	0.9974863
646588	LOC646588	hypothetical protein LOC646588	1.01459	0.972273	1.002601
646597	CHMP1AP1	chromatin modifying protein 1A pseudogene 1	1.02239	0.944914	1.0603231
646600	C3orf65	chromosome 3 open reading frame 65	1.03212	1.018587	1.0239653
646603	C4orf51	chromosome 4 open reading frame 51	1.02111	1.040628	0.9941927
646609	LOC646609	chloride intracellular channel 4 pseudogene	1.00642	0.996298	0.9684201
646610	LOC646610	similar to hCG36930	1.00671	0.999494	1.0117742
646612	EEF1A1P25	eukaryotic translation elongation factor 1 alpha 1 pseudogene 25	0.97123	0.943561	0.9873543
646614	LOC646614	budding uninhibited by benzimidazoles 3 pseudogene	0.9854	0.980306	1.0245126
646625	PRHOXNB	parahox cluster neighbor	0.97219	0.990478	0.9988613
646626	LOC646626	hypothetical protein LOC646626	1.01659	0.95364	0.9794654
646629	LOC646629	similar to hCG1643857	1.0088	0.994229	0.9972857
646630	CHCHD2P8	coiled-coil-helix-coiled-coil-helix domain containing 2 pseudogene 8	0.92039	0.918515	1.0752081
646639	LOC646639	translocase of outer mitochondrial membrane 40 homolog (yeast) pseudogene	1.02097	0.950396	1.0181512
646640	TTC39DP	tetratricopeptide repeat domain 39D, pseudogene	1.00656	1	0.9807167
646643	SBK2	SH3-binding domain kinase family, member 2	0.96659	0.952426	1.0643295
646658	TMEM90A	transmembrane protein 90A	1.01116	1.029072	1.0208693
646663	LOC646663	ret finger protein-like 1 pseudogene	0.98869	0.954954	1.0529762
646665	LOC646665	golgi autoantigen, golgin subfamily a, 6 pseudogene	0.98592	1.046393	0.9717431
646672	RPL15P20	ribosomal protein L15 pseudogene 20	1.03231	1.01741	1.0267733
646674	LOC646674	single stranded DNA binding protein 4 pseudogene	0.97708	1.034341	0.9810117
646688	RPL4P2	ribosomal protein L4 pseudogene 2	1.00236	0.97391	1.0256111
646693	CNN2P10	calponin 2 pseudogene 10	0.97213	1.011638	0.9873576
646696	LOC646696	MAX-like protein X pseudogene	1.00176	1.023361	1.0113382
646698	ZSCAN5D	zinc finger and SCAN domain containing 5D	0.99507	1.000866	1.0162068
646702	HLA-DPA2	major histocompatibility complex, class II, DP alpha 2 (pseudogene)	1.01802	1.038183	0.9772227
646708	LOC646708	DnaJ (Hsp40) homolog, subfamily C, member 5 pseudogene	1.01548	0.982499	0.9819219
646710	RPL19P4	ribosomal protein L19 pseudogene 4	0.9794	0.982163	1.0302224
646719	LOC646719	hypothetical LOC646719	0.99288	0.97494	0.9140316
646720	[No Symbo	[No Name]	1.00511	1.026683	1.0475657
646721	LOC646721	family with sequence similarity 60, member A pseudogene	0.98654	0.980114	1.0307329
646730	LOC646730	hypothetical protein LOC646730	0.99539	0.99019	0.9757318

646735	[No Symbo	[No Name]	0.98923	0.99133	1.0439041
646737	RPS14P6	ribosomal protein S14 pseudogene 6	0.97721	1.014846	0.987712
646745	LOC646745	general transcription factor IIIC, polypeptide 6, alpha 35kDa pseudogene	1.00711	0.955513	1.0314974
646754	TRIM64C	tripartite motif containing 64C	0.99686	1.071463	1.0412447
646755	MFAP1P1	microfibrillar-associated protein 1 pseudogene 1	0.98509	0.968386	1.0397782
646758	LOC646758	protein tyrosine phosphatase, non-receptor type 2 pseudogene	0.99102	1.042184	1.0355948
646766	RPL35P2	ribosomal protein L35 pseudogene 2	0.95727	1.047316	1.0622457
646770	LOC646770	putative tripartite motif-containing protein 77-like	0.99458	0.999516	0.974108
646778	LOC646778	hypothetical LOC646778	0.99284	0.986192	1.0030604
646784	RPL5P6	ribosomal protein L5 pseudogene 6	1.0197	0.939144	0.9549312
646787	RPL31P37	ribosomal protein L31 pseudogene 37	0.98255	1.033374	0.9994265
646791	ANP32BP1	acidic (leucine-rich) nuclear phosphoprotein 32 family, member B pseudogene 1	0.95417	0.968337	1.0398167
646794	LOC646794	SNF8, ESCRT-II complex subunit, homolog (S. cerevisiae) pseudogene	1.00475	1.005218	1.0200151
646797	LOC646797	glutamyl aminopeptidase (aminopeptidase A) pseudogene	0.96771	0.960174	1.0321391
646799	ZAR1L	zygote arrest 1-like	1.00066	1.015975	0.9568417
646801	LOC646801	pantothenate kinase 3 pseudogene	0.99441	0.996873	1.0992786
646804	LOC646804	alkylated DNA repair protein alkB homolog 8-like	0.98074	0.978221	0.9984159
646805	[No Symbo	[No Name]	0.99646	1.04297	0.9538215
646808	LOC646808	L antigen family member 3-like	1.00889	1.028905	1.0618239
646810	LOC646810	acyl-coenzyme A synthetase ACSM4, mitochondrial-like	0.99296	1.056346	1.0197491
646813	LOC646813	DEAH (Asp-Glu-Ala-His) box polypeptide 9 pseudogene	1.01439	1.018754	1.0151229
646820	RPL19P19	ribosomal protein L19 pseudogene 19	0.99105	0.955846	1.0346297
646821	[No Symbo	[No Name]	0.96416	0.944248	1.0594211
646828	LOC646828	carbonic anhydrase VA, mitochondrial pseudogene	0.97849	1.002137	1.0136331
646837	LOC646837	eukaryotic translation elongation factor 1 gamma pseudogene	1.01438	0.962525	1.0331813
646842	[No Symbo	[No Name]	0.98109	0.985456	1.0244214
646849	LOC646849	nucleophosmin (nucleolar phosphoprotein B23, numatrin) pseudogene	0.98868	0.948352	1.0532634
646851	LOC646851	hypothetical LOC646851	0.99782	0.997585	1.0069884
646862	LOC646862	hypothetical protein LOC646862	1.00548	0.976031	1.1012897
646864	LOC646864	similar to hCG36734	1.00238	0.987278	1.030336
646865	LOC646865	hCG1814936	1.01655	0.97792	0.9656108
646870	LOC646870	centrosomal protein 57kDa pseudogene	1.04646	1.012651	0.9450956
646871	[No Symbo	[No Name]	1.00333	0.978909	0.9875361

646873	LOC646873	slowmo homolog 2 (Drosophila) pseudogene	1.0278	0.999789	1.0867663
646875	RPL12P2	ribosomal protein L12 pseudogene 2	0.95591	0.995555	1.0146705
646877	GAPDHP34	glyceraldehyde 3 phosphate dehydrogenase pseudogene 34	0.99662	1.031965	1.0137931
646879	ARL2BPP3	ADP-ribosylation factor-like 2 binding protein pseudogene 3	0.98896	0.989379	1.033528
646880	[No Symbo	[No Name]	0.98579	0.945994	1.0591905
646888	FLJ45825	hypothetical protein LOC646888	0.96158	1.016018	1.0116
646890	LOC646890	axin interactor, dorsalization-associated protein-like	0.99445	1.013576	1.0411622
646891	SDCCAG3P2	serologically defined colon cancer antigen 3 pseudogene 2	1.00979	0.954833	0.9962771
646892	SH2D7	SH2 domain containing 7	0.99767	1.01334	0.9688228
646895	[No Symbo	[No Name]	0.8985	0.923857	0.9216327
646908	[No Symbo	[No Name]	0.98438	0.985223	0.9824208
646909	RPL17P33	ribosomal protein L17 pseudogene 33	1.00643	0.946185	1.0229732
646910	HSPD1P9	heat shock 60kDa protein 1 (chaperonin) pseudogene 9	0.98696	0.945584	1.0112369
646913	SEPT7P3	septin 7 pseudogene 3	1.00066	1.040721	1.0569757
646915	ZNF806	zinc finger protein 806	0.96193	0.953221	1.0255333
646929	ANKRD26P	ankyrin repeat domain 26 pseudogene 2	0.97807	0.988306	1.0251324
646934	LOC646934	putative golgin subfamily A member 6D-like	1.03018	1.022621	1.0295373
646936	LOC646936	C-terminal binding protein 2 pseudogene	1.00482	0.974736	1.0021595
646942	RPS10P14	ribosomal protein S10 pseudogene 14	1.00995	1.005953	0.9778092
646949	RPL23P6	ribosomal protein L23 pseudogene 6	0.87923	0.946077	1.0761377
646951	FAM188B2	family with sequence similarity 188, member B2	1.02154	0.991123	0.9964652
646954	YWHAQP4	YWHAQ pseudogene 4	0.9818	0.95415	1.0147149
646956	LOC646956	heterogeneous nuclear ribonucleoprotein C (C1/C2) pseudogene	1.00504	1.019321	1.014641
646960	PRSS56	protease, serine, 56	1.00698	1.010724	0.9880403
646962	HRCT1	histidine rich carboxyl terminus 1	1.00715	1.041109	1.012167
646970	LOC646970	chromosome 5 open reading frame 15 pseudogene	1.01456	1.003466	1.0315303
646973	[No Symbo	[No Name]	1.01199	1.01158	0.9949749
646976	LOC646976	hypothetical LOC646976	1.01733	0.996389	1.0052671
646980	[No Symbo	[No Name]	0.9891	1.027256	0.9958245
646981	LOC646981	protein tyrosine phosphatase, non-receptor type-like pseudogene	1.00103	0.990001	1.0492347
646985	RPS12P25	ribosomal protein S12 pseudogene 25	1.0032	0.992791	1.0302859
646993	HMGB3P24	high mobility group box 3 pseudogene 24	0.99171	0.926968	1.0152413
646995	LOC646995	tripartite motif-containing protein 38-like	0.99517	0.978448	0.9835294

647000	TUBBP2	tubulin, beta pseudogene 2	0.93249	0.980767	1.0265681
647001	GAPDHP35	glyceraldehyde 3 phosphate dehydrogenase pseudogene 35	1.0087	1.007541	1.0532198
647012	LOC647012	YY1 transcription factor pseudogene	0.99539	1.007084	1.0476877
647013	LOC647013	potassium channel tetramerisation domain containing 9 pseudogene	0.99507	0.975461	1.0140358
647016	EIF2S2P7	eukaryotic translation initiation factor 2, subunit 2 beta pseudogene 7	0.9829	0.987446	0.992112
647020	LOC647020	hypothetical protein LOC647020	0.99124	1.015644	0.9884881
647024	C6orf132	chromosome 6 open reading frame 132	1.00623	0.955579	0.991989
647030	LOC647030	eukaryotic translation elongation factor 1 beta 2-like	1.00487	0.917585	1.033407
647033	PA2G4P4	proliferation-associated 2G4 pseudogene 4	0.96866	0.988619	1.0276152
647034	RPS14P10	ribosomal protein S14 pseudogene 10	0.91753	0.987959	0.9661818
647038	[No Symbo	[No Name]	1.03645	1.029238	1.0285867
647039	[No Symbo	[No Name]	1.02489	1.059958	0.9460251
647047	TCP1P1	t-complex 1 pseudogene 1	0.9758	0.945883	1.0253672
647049	[No Symbo	[No Name]	1.01549	0.990545	0.9669374
647051	LOC647051	tyrosine 3-monooxygenase/tryptophan 5-monooxygenase activation protein, beta polypeptide pseudogene	1.00617	1.009119	0.9667534
647055	[No Symbo	[No Name]	0.99004	1.006188	0.9843975
647065	[No Symbo	[No Name]	1.03448	0.925632	0.9713906
647070	LOC647070	hypothetical LOC647070	0.97955	1.019375	1.0228544
647073	[No Symbo	[No Name]	0.98793	1.011956	1.0125961
647076	BRD7P2	bromodomain containing 7 pseudogene 2	0.99576	0.981892	0.938787
647077	LOC647077	60S ribosomal protein L14-like	1.03582	0.899442	1.0014312
647081	SDHDP2	succinate dehydrogenase complex, subunit D, integral membrane protein pseudogene 2	1.01719	0.991287	1.0706113
647085	PPIAP25	peptidylprolyl isomerase A (cyclophilin A) pseudogene 25	1.01242	1.047574	0.9825366
647086	LOC647086	UPF0687 protein C20orf27-like	1.00714	0.973133	1.0480283
647087	PL-5283	PL-5283 protein	0.98013	0.876923	1.0459472
647088	CTF2P	cardiotrophin 2, pseudogene	0.99619	1.03375	0.9833683
647094	RPLP0P11	ribosomal protein, large, P0 pseudogene 11	0.99503	1.013724	0.9982832
647099	RPL23AP42	ribosomal protein L23a pseudogene 42	1.02335	1.000793	1.0074717
647102	ELK1P1	ELK1, member of ETS oncogene family pseudogene 1	1.00214	0.98601	0.9888503
647109	[No Symbo	[No Name]	1.03129	1.022558	1.0157431
647112	RPL27P6	ribosomal protein L27 pseudogene 6	0.97626	1.014214	1.1231792
647115	FLJ36848	hypothetical LOC647115	0.9872	0.931739	0.9628078
647121	EMBP1	embigin pseudogene 1	0.98533	1.04208	1.0071173

647123	UQCRFS1P2	ubiquinol-cytochrome c reductase, Rieske iron-sulfur polypeptide 1 pseudogene 2	1.00369	1.024696	1.023325
647131	[No Symbo	[No Name]	1.00154	1.03856	0.8941588
647132	LOC647132	DEAH (Asp-Glu-Ala-His) box polypeptide 29 pseudogene	0.99806	1.023211	1.0210622
647135	SRGAP2P2	SLIT-ROBO Rho GTPase activating protein 2 pseudogene 2	1.03359	0.962384	1.0265189
647154	[No Symbo	[No Name]	1.00018	0.994588	1.0175538
647155	RPS15AP20	ribosomal protein S15a pseudogene 20	1.01923	0.992909	1.025486
647157	LOC647157	sodium/hydrogen exchanger-like domain-containing protein 1-like	1.00167	0.959184	1.0019431
647158	RPSAP33	ribosomal protein SA pseudogene 33	1.01599	0.95741	1.0819015
647162	KRT18P33	keratin 18 pseudogene 33	1.01588	1.007931	1.0038009
647163	LOC647163	hypothetical protein LOC647163	1.00823	1.012088	1.0379771
647166	LOC647166	hypothetical protein LOC647166	1.0031	1.024009	0.9995396
647167	EEF1A1P14	eukaryotic translation elongation factor 1 alpha 1 pseudogene 14	1.00469	0.975818	1.1393184
647169	LOC647169	glutathione S-transferase alpha 3 pseudogene	0.97806	1.009937	1.0057553
647174	SERPINE3	serpin peptidase inhibitor, clade E (nexin, plasminogen activator inhibitor type 1), member 3	1.00337	1.029439	1.0001339
647175	LOC647175	glutathione S-transferase A1-like	1.00254	0.979948	1.017613
647177	LOC647177	glutathione S-transferase A3-like	1.00923	1.014444	1.0038668
647188	[No Symbo	[No Name]	0.99062	0.999154	0.9904002
647202	[No Symbo	[No Name]	1.00694	0.99786	0.9823392
647209	[No Symbo	[No Name]	0.99003	0.942667	0.9536482
647212	[No Symbo	[No Name]	1.00248	0.975262	1.0279679
647215	C1orf81	chromosome 1 open reading frame 81	1.00009	0.99465	1.0313255
647219	ASCL5	achaete-scute complex homolog 5 (Drosophila)	0.99171	0.987483	1.0141518
647249	GAPDHP36	glyceraldehyde 3 phosphate dehydrogenase pseudogene 36	0.99978	0.967721	1.0746957
647252	[No Symbo	[No Name]	1.02107	0.966178	0.9523274
647253	LOC647253	ring finger protein 13 pseudogene	0.99029	0.915827	1.0214471
647259	[No Symbo	[No Name]	1.03099	1.024473	1.0892086
647265	LOC647265	cytochrome P450 2J5-like	0.99479	1.036643	1.006664
647280	[No Symbo	[No Name]	1.01439	0.998734	1.0262655
647281	[No Symbo	[No Name]	0.9968	0.979567	0.992635
647283	LOC647283	hCG1820717	0.97895	1.025371	0.9685464
647286	LOC647286	hypothetical protein LOC647286	0.99899	1.015045	0.9943755
647288	CTAGE11P	CTAGE family, member 11, pseudogene	1.02304	1.007679	0.9407652
647298	HSPD1P8	heat shock 60kDa protein 1 (chaperonin) pseudogene 8	1.01788	0.983584	1.0028282

647299	LOC647299	enolase-phosphatase 1 pseudogene	0.99204	1.002385	0.9919457
647302	LOC647302	LSM3 homolog, U6 small nuclear RNA associated (S. cerevisiae) pseudogene	0.9959	0.940009	1.0419961
647305	[No Symbo	[No Name]	1.01177	1.034156	0.9832285
647309	GEMC1	geminin coiled-coil domain-containing protein 1	1.00215	0.981716	1.0229705
647310	TEX22	testis expressed 22	0.99217	0.998715	1.0474315
647313	[No Symbo	[No Name]	1.00526	1.070771	0.9536306
647317	GYG1P2	glycogenin 1 pseudogene 2	0.98502	1.006471	1.020619
647319	VEZF1L1	vascular endothelial zinc finger 1-like 1	1.02523	0.950446	0.9869761
647503	LOC647503	NHP2-like protein 1-like	1.00464	1.031345	0.9146806
647532	LOC647532	phenylalanine-tRNA synthetase-like, beta subunit pseudogene	1.0106	0.984933	1.0194696
647580	[No Symbo	[No Name]	0.99256	0.998994	0.990783
647633	LOC647633	hypothetical LOC647633	1.00838	1.001879	1.2078969
647996	LOC647996	chromosome 9 open reading frame 172 pseudogene	1.02033	0.991457	1.0464102
648044	LOC648044	guanine nucleotide binding protein (G protein), gamma 12-like	1.01035	1.053036	0.9777481
648154	[No Symbo	[No Name]	0.96451	0.951108	0.979477
648237	[No Symbo	[No Name]	0.98558	1.039275	1.0645049
648245	[No Symbo	[No Name]	0.97363	1.075443	0.9576711
648283	TERF1P4	telomeric repeat binding factor (NIMA-interacting) 1 pseudogene 4	0.99262	0.970276	1.0199901
648315	VN2R9P	vomeronasal 2 receptor 9 pseudogene	1.00922	1.007931	0.9914274
648405	[No Symbo	[No Name]	1.0209	0.960388	1.0074738
648415	[No Symbo	[No Name]	1.01521	0.936264	0.9486067
648442	LOC648442	eukaryotic translation initiation factor 3, subunit I pseudogene	0.99091	0.985525	0.9973044
648532	LOC648532	nuclear receptor binding factor 2 pseudogene	1.00035	1.01242	1.0182714
648603	[No Symbo	[No Name]	0.99933	0.973423	1.0117967
648665	LOC648665	hypothetical LOC648665	0.99142	0.971155	0.9506648
648708	[No Symbo	[No Name]	1.01793	1.022226	1.0300982
648774	LOC648774	polo-like kinase 1 (Drosophila) pseudogene	0.98192	0.960861	1.038088
648791	PPP1R3G	protein phosphatase 1, regulatory (inhibitor) subunit 3G	0.97188	1.056362	1.1271172
648809	LOC648809	elongation factor Tu GTP-binding domain-containing protein 1 pseudogene	1.00274	0.994717	1.0343753
648822	HMGN2P18	high mobility group nucleosomal binding domain 2 pseudogene 18	0.99103	0.951519	0.9491585
648890	[No Symbo	[No Name]	1.02751	1.005421	0.9942144
648921	[No Symbo	[No Name]	0.98725	1.017659	0.9738819
648927	LOC648927	histone acetyltransferase MYST2-like	0.97186	0.959378	0.9624849

648934	LOC648934	citrate synthase, mitochondrial-like	1.00899	0.996562	1.0131867
648937	[No Symbo	[No Name]	0.99775	1.027214	0.9407083
648947	LOC648947	aldo-keto reductase family 1, member C pseudogene	0.97064	0.985443	0.997351
648987	LOC648987	hypothetical LOC648987	0.9715	0.95787	1.0140766
649024	LOC649024	citrate synthase pseudogene	0.9989	0.976327	1.0280506
649095	[No Symbo	[No Name]	0.96064	0.996102	0.9854459
649133	LOC649133	hypothetical LOC649133	1.03416	1.010895	0.9700869
649137	ZSCAN5C	zinc finger and SCAN domain containing 5C	1.01455	1.044464	0.9799581
649159	NCRNA002	non-protein coding RNA 273	0.98547	0.972117	1.0920682
649166	LOC649166	eukaryotic translation initiation factor 3, subunit J pseudogene	1.01281	1.05174	1.0176406
649179	PRAMEL	preferentially expressed antigen in melanoma-like	0.99077	0.98972	1.0137073
649186	VN2R17P	vomeronasal 2 receptor 17 pseudogene	1.01527	0.9849	0.9688155
649201	LOC649201	paraneoplastic antigen like 6A-like	0.99904	0.974518	1.0216519
649238	LOC649238	similar to hCG1645335	0.98051	1.034548	1.0184448
649264	CES5AP1	carboxylesterase 5A pseudogene 1	0.99149	1.007727	1.0404908
649288	AK4P6	adenylate kinase 4 pseudogene 6	1.06511	0.961146	1.0758391
649299	RPL36AP51	ribosomal protein L36a pseudogene 51	0.91726	0.897838	0.9492904
649445	[No Symbo	[No Name]	1.02487	1.016591	0.9435733
649446	FLJ35776	hypothetical LOC649446	0.96477	0.945186	0.8889087
649458	MPTX	mucosal pentraxin pseudogene	1.00333	0.969291	1.1067108
649489	LOC649489	protein phosphatase 1, regulatory (inhibitor) subunit 2 pseudogene	1.03343	1.013924	1.0126528
649671	LOC649671	probable ATP-dependent RNA helicase DDX56-like	1.0088	0.982536	0.9841083
649873	LOC649873	peptidyl-prolyl cis-trans isomerase A-like	0.97507	0.995598	0.9899487
650095	[No Symbo	[No Name]	1.01381	1.009388	0.9415131
650157	LOC650157	peptidyl-prolyl cis-trans isomerase A-like	1.01283	1.022086	1.0534005
650172	CCT4P1	chaperonin containing TCP1, subunit 4 (delta) pseudogene 1	1.00787	1.01479	1.0383434
650181	LOC650181	chromosome 9 open reading frame 21 pseudogene	0.99927	1.06989	1.0309776
650226	LOC650226	ankyrin repeat domain 26 pseudogene	0.97797	1.06676	1.0539683
650474	LOC650474	tryptase beta pseudogene	0.99431	0.951539	1.0206941
650531	[No Symbo	[No Name]	0.97019	0.976479	1.0153354
650560	DDX3YP3	DEAD (Asp-Glu-Ala-Asp) box polypeptide 3, Y-linked pseudogene 3	0.97737	0.968893	0.9843379
650655	ABCA17P	ATP-binding cassette, sub-family A (ABC1), member 17, pseudogene	0.9976	1.008932	0.9971505
650656	[No Symbo	[No Name]	0.98098	1.009331	1.0550109

650662	C14orf184	chromosome 14 open reading frame 184	0.9884	1.030739	0.9907307
650794	LOC650794	hypothetical LOC650794	0.99427	0.991933	1.0157779
650866	LOC650866	transient receptor potential cation channel, subfamily C, member 6 pseudogene	1.03304	0.969048	1.0087175
651033	[No Symbo	[No Name]	1.0191	0.944454	0.9834331
651053	DDX18P2	DEAD (Asp-Glu-Ala-Asp) box polypeptide 18 pseudogene 2	1.03048	1.026124	0.9972561
651118	[No Symbo	[No Name]	0.97758	1.007576	1.0417749
651144	LOC651144	nascent-polypeptide-associated complex alpha polypeptide pseudogene	1.00967	0.96662	0.9887706
651158	[No Symbo	[No Name]	0.94955	0.990964	1.0277964
651250	LOC651250	leucine rich repeat containing 37, member A3 pseudogene	0.98105	0.98454	0.9834627
651258	GAPDHP37	glyceraldehyde 3 phosphate dehydrogenase pseudogene 37	0.98053	0.958601	1.003146
651419	[No Symbo	[No Name]	0.99831	0.980546	1.0018207
651430	FLJ38576	hypothetical LOC651430	1.00574	1.050209	0.9668317
651503	[No Symbo	[No Name]	0.99966	0.993812	1.0144482
651628	LOC651628	eukaryotic translation elongation factor 1 gamma pseudogene	1.02801	0.960048	1.0156257
651644	LOC651644	LIM and senescent cell antigen-like domains 2 pseudogene	0.9749	0.982951	1.0576552
651714	LOC651714	mucin 20, cell surface associated pseudogene	0.9714	0.985902	1.035498
651746	ANKRD33B	ankyrin repeat domain 33B	0.99342	1.062816	0.9947974
651868	LOC651868	similar to hCG1643984	1.01382	0.963087	0.951295
652170	COX4I1P2	cytochrome c oxidase subunit IV isoform 1 pseudogene 2	0.98783	1.00491	1.0079167
652191	LOC652191	microtubule-associated proteins 1A/1B light chain 3B-like	0.99067	1.013043	1.0611979
652276	LOC652276	hypothetical LOC652276	1.00162	1.020606	1.034984
652411	RPSAP44	ribosomal protein SA pseudogene 44	1.01278	1.01468	1.0585534
652423	LOC652423	nucleophosmin (nucleolar phosphoprotein B23, numatrin) pseudogene	1.00252	1.018519	1.0306518
652489	SUMO2P3	SMT3 suppressor of mif two 3 homolog 2 (S. cerevisiae) pseudogene 3	0.99634	1.022948	1.01995
652549	LOC652549	SNW domain containing 1 pseudogene	0.9906	0.947412	1.0168082
652624	RPSAP46	ribosomal protein SA pseudogene 46	0.96388	0.997951	1.0084909
652959	BTF3P5	basic transcription factor 3 pseudogene 5	1.02308	0.913979	1.0980769
652960	BTF3P7	basic transcription factor 3 pseudogene 7	0.99661	0.941851	1.0343074
652968	GATSL3	GATS protein-like 3	0.96293	1.020335	0.9445857
652972	C9orf29	chromosome 9 open reading frame 29	0.99955	1.048307	0.988384
652991	SKOR2	SKI family transcriptional corepressor 2	0.9903	1.010939	1.0530552
653051	[No Symbo	[No Name]	0.99547	0.990015	1.0028266
653052	[No Symbo	[No Name]	1.05501	0.966472	0.9638361

653082	ZDHHC11B	zinc finger, DHHC-type containing 11B	1.0252	0.998908	1.0551923
653113	LOC653113	family with sequence similarity 86, member A pseudogene	0.98477	0.952246	1.0594728
653121	ZBTB8A	zinc finger and BTB domain containing 8A	1.00517	0.986471	1.0067631
653140	C2orf84	chromosome 2 open reading frame 84	0.99779	0.99991	1.0032224
653175	LOC653175	family with sequence similarity 29, member A pseudogene	0.94949	1.01746	0.9078598
653190	ABCC6P1	ATP-binding cassette, sub-family C, member 6 pseudogene 1	1.03176	1.025952	0.9420903
653194	LOC653194	KH homology domain-containing protein 1-like	1.04084	1.012677	0.9902286
653198	[No Symbo	[No Name]	0.99642	0.968098	1.0314436
653314	RPL19P9	ribosomal protein L19 pseudogene 9	0.97115	1.004136	1.0088855
653319	KIAA0895L	KIAA0895-like	0.98901	0.9679	1.0060644
653325	[No Symbo	[No Name]	0.98965	0.969357	1.0268774
653364	HSPB1P2	heat shock 27kDa protein 1 pseudogene 2	0.96204	0.939481	1.0430295
653381	SORD2	SORD pseudogene	0.99079	0.996002	0.9965913
653390	RRN3P2	RNA polymerase I transcription factor homolog (S. cerevisiae) pseudogene 2	1.02448	1.010744	0.8606248
653399	GSTTP2	glutathione S-transferase theta pseudogene 2	0.97732	1.01299	0.9826227
[truncated: 275,505 more chars]
