# Supplementary material for: Gene-expression patterns in peripheral blood classify familial breast cancer susceptibility
Source: BMC Med Genomics. 2015 Nov 4;8:72. doi: 10.1186/s12920-015-0145-6 (PMC4634735; doi:10.1186/s12920-015-0145-6)
Supplement: Additional file 10: — Summary of differentially expressed genes. Genes for which the average expression was consistently either higher or lower in FBC individuals relative to controls for the Utah and Ontario cohorts. (PDF 987 kb) [file 12920_2015_145_MOESM10_ESM.pdf]

Entrez Gene ID	Gene Symbol	Gene Name
4675	NAP1L3	nucleosome assembly protein 1-like 3
5769	PTP4A2P2	protein tyrosine phosphatase type IVA, member 2 pseudogene 2
390980	ZNF805	zinc finger protein 805
161291	TMEM30B	transmembrane protein 30B
400360	C15orf54	chromosome 15 open reading frame 54
959	CD40LG	CD40 ligand
11123	RCAN3	RCAN family member 3
56985	C17orf48	chromosome 17 open reading frame 48
9840	KIAA0748	KIAA0748
6920	TCEA3	transcription elongation factor A (SII), 3
258010	SVIP	small VCP/p97-interacting protein
158399	ZNF483	zinc finger protein 483
57191	VN1R1	vomeronasal 1 receptor 1
3957	LGALS2	lectin, galactoside-binding, soluble, 2
643162	DKFZP779L1853	hypothetical LOC643162
5920	RARRES3	retinoic acid receptor responder (tazarotene induced) 3
92106	OXNAD1	oxidoreductase NAD-binding domain containing 1
51301	GCNT4	glucosaminyl (N-acetyl) transferase 4, core 2
340554	ZC3H12B	zinc finger CCCH-type containing 12B
322	APBB1	amyloid beta (A4) precursor protein-binding, family B, member 1 (Fe65)
100132760	[No Symbol]	[No Name]
9241	NOG	noggin
1823	DSC1	desmocollin 1
56271	BEX4	brain expressed, X-linked 4
552	AVPR1A	arginine vasopressin receptor 1A
439949	LOC439949	hypothetical LOC439949
100131733	LOC100131733	hypothetical LOC100131733
147727	LOC147727	hypothetical LOC147727
8797	TNFRSF10A	tumor necrosis factor receptor superfamily, member 10a
91624	NEXN	nexilin (F actin binding protein)
54033	RBM11	RNA binding motif protein 11
7695	ZNF136	zinc finger protein 136
100101405	LOC100101405	chromosome 4 open reading frame 43 pseudogene

3399	ID3	inhibitor of DNA binding 3, dominant negative helix-loop-helix protein
128553	TSHZ2	teashirt zinc finger homeobox 2
5140	PDE3B	phosphodiesterase 3B, cGMP-inhibited
10778	ZNF271	zinc finger protein 271
940	CD28	CD28 molecule
11043	MID2	midline 2
340547	VSIG1	V-set and immunoglobulin domain containing 1
321	APBA2	amyloid beta (A4) precursor protein-binding, family A, member 2
2791	GNG11	guanine nucleotide binding protein (G protein), gamma 11
152217	LOC152217	hypothetical LOC152217
32	ACACB	acetyl-CoA carboxylase beta
1408	CRY2	cryptochrome 2 (photolyase-like)
55664	CDC37L1	cell division cycle 37 homolog (S. cerevisiae)-like 1
4050	LTB	lymphotoxin beta (TNF superfamily, member 3)
26119	LDLRAP1	low density lipoprotein receptor adaptor protein 1
130399	ACVR1C	activin A receptor, type IC
3480	IGF1R	insulin-like growth factor 1 receptor
51176	LEF1	lymphoid enhancer-binding factor 1
55837	EAPP	E2F-associated phosphoprotein
144363	LYRM5	LYR motif containing 5
168537	GIMAP7	GTPase, IMAP family member 7
120224	TMEM45B	transmembrane protein 45B
100128616	[No Symbol]	[No Name]
3337	DNAJB1	DnaJ (Hsp40) homolog, subfamily B, member 1
645958	RPS18P9	ribosomal protein S18 pseudogene 9
7294	TXK	TXK tyrosine kinase
7754	ZNF204P	zinc finger protein 204, pseudogene
100132341	KIAA0664L3	KIAA0664-like 3
54851	ANKRD49	ankyrin repeat domain 49
646895	[No Symbol]	[No Name]
10179	RBM7	RNA binding motif protein 7
3655	ITGA6	integrin, alpha 6
4054	LTBP3	latent transforming growth factor beta binding protein 3
113791	PIK3IP1	phosphoinositide-3-kinase interacting protein 1
641518	LOC641518	hypothetical LOC641518

7381	UQCRB	ubiquinol-cytochrome c reductase binding protein
30061	SLC40A1	solute carrier family 40 (iron-regulated transporter), member 1
259215	LY6G6F	lymphocyte antigen 6 complex, locus G6F
360	AQP3	aquaporin 3 (Gill blood group)
84749	USP30	ubiquitin specific peptidase 30
100133168	GPR183P1	G protein-coupled receptor 183 pseudogene 1
54494	C11orf71	chromosome 11 open reading frame 71
401093	LOC401093	hypothetical LOC401093
168451	THAP5	THAP domain containing 5
282997	LOC282997	hypothetical LOC282997
122525	C14orf28	chromosome 14 open reading frame 28
51266	CLEC1B	C-type lectin domain family 1, member B
6304	SATB1	SATB homeobox 1
80701	AKR1D1P1	aldo-keto reductase family 1, member D1 pseudogene 1
2815	GP9	glycoprotein IX (platelet)
80177	MYCT1	myc target 1
2686	GGT7	gamma-glutamyltransferase 7
100129436	[No Symbol]	[No Name]
403313	PPAPDC2	phosphatidic acid phosphatase type 2 domain containing 2
9925	ZBTB5	zinc finger and BTB domain containing 5
7644	ZNF91	zinc finger protein 91
122773	KLHDC1	kelch domain containing 1
91782	CHMP7	CHMP family, member 7
80321	CEP70	centrosomal protein 70kDa
5125	PCSK5	proprotein convertase subtilisin/kexin type 5
283897	C16orf54	chromosome 16 open reading frame 54
140606	SELM	selenoprotein M
23548	TTC33	tetratricopeptide repeat domain 33
288	ANK3	ankyrin 3, node of Ranvier (ankyrin G)
239	ALOX12	arachidonate 12-lipoxygenase
120776	OR2D2	olfactory receptor, family 2, subfamily D, member 2
687	KLF9	Kruppel-like factor 9
163778	SPRR4	small proline-rich protein 4
3081	HGD	homogentisate 1,2-dioxygenase
79048	SECISBP2	SECIS binding protein 2

2811	GP1BA	glycoprotein Ib (platelet), alpha polypeptide
644693	[No Symbol]	[No Name]
199746	U2AF1L4	U2 small nuclear RNA auxiliary factor 1-like 4
100128457	LOC100128457	similar to hCG2026341
100129866	LOC100129866	etoposide induced 2.4 mRNA pseudogene
1236	CCR7	chemokine (C-C motif) receptor 7
29851	ICOS	inducible T-cell co-stimulator
84850	C9orf70	chromosome 9 open reading frame 70
130500	CISD1P1	CDGSH iron sulfur domain 1 pseudogene 1
5578	PRKCA	protein kinase C, alpha
284391	ZNF844	zinc finger protein 844
9126	SMC3	structural maintenance of chromosomes 3
5463	POU6F1	POU class 6 homeobox 1
440603	BCL2L15	BCL2-like 15
49854	ZNF295	zinc finger protein 295
10023	FRAT1	frequently rearranged in advanced T-cell lymphomas
28984	C13orf15	chromosome 13 open reading frame 15
139302	[No Symbol]	[No Name]
129293	C2orf89	chromosome 2 open reading frame 89
100132148	[No Symbol]	[No Name]
27134	TJP3	tight junction protein 3 (zona occludens 3)
84144	SYDE2	synapse defective 1, Rho GTPase, homolog 2 (C. elegans)
3738	KCNA3	potassium voltage-gated channel, shaker-related subfamily, member 3
643058	LOC643058	interferon induced transmembrane protein pseudogene
57561	ARRDC3	arrestin domain containing 3
143686	SESN3	sestrin 3
342426	ZNF720P1	zinc finger protein 720 pseudogene 1
87	ACTN1	actinin, alpha 1
400347	LOC400347	REX4, RNA exonuclease 4 homolog (S. cerevisiae) pseudogene
5440	POLR2K	polymerase (RNA) II (DNA directed) polypeptide K, 7.0kDa
401303	ZNF815	zinc finger protein 815
55831	TMEM111	transmembrane protein 111
643723	LOC643723	hypothetical LOC643723
80741	LY6G5C	lymphocyte antigen 6 complex, locus G5C
113878	DTX2	deltex homolog 2 (Drosophila)

64407	RGS18	regulator of G-protein signaling 18
57605	PITPNM2	phosphatidylinositol transfer protein, membrane-associated 2
23086	EXPH5	exophilin 5
9737	GPRASP1	G protein-coupled receptor associated sorting protein 1
150864	FAM117B	family with sequence similarity 117, member B
23348	DOCK9	dedicator of cytokinesis 9
58473	PLEKHB1	pleckstrin homology domain containing, family B (evectins) member 1
3021	H3F3B	H3 histone, family 3B (H3.3B)
260342	BA345E19.2	COPS8 pseudogene
100129842	ZNF737	zinc finger protein 737
4240	MFGE8	milk fat globule-EGF factor 8 protein
80764	THAP7	THAP domain containing 7
7561	ZNF14	zinc finger protein 14
256987	SERINC5	serine incorporator 5
23641	LDOC1	leucine zipper, down-regulated in cancer 1
91612	CHURC1	churchill domain containing 1
3841	KPNA5	karyopherin alpha 5 (importin alpha 6)
442673	TUBG1P	tubulin, gamma 1 pseudogene
3727	JUND	jun D proto-oncogene
100129196	LOC100129196	hypothetical LOC100129196
51763	INPP5K	inositol polyphosphate-5-phosphatase K
8535	CBX4	chromobox homolog 4
51710	ZNF44	zinc finger protein 44
123036	TC2N	tandem C2 domains, nuclear
5742	PTGS1	prostaglandin-endoperoxide synthase 1 (prostaglandin G/H synthase and cyclooxygenase)
6815	STYX	serine/threonine/tyrosine interacting protein
51460	SFMBT1	Scm-like with four mbt domains 1
163859	C1orf55	chromosome 1 open reading frame 55
900	CCNG1	cyclin G1
93624	TADA2B	transcriptional adaptor 2B
1073	CFL2	cofilin 2 (muscle)
100128262	LOC100128262	hypothetical LOC100128262
117283	IP6K3	inositol hexakisphosphate kinase 3
126259	TMIGD2	transmembrane and immunoglobulin domain containing 2
401261	FLJ38717	FLJ38717 protein

9592	IER2	immediate early response 2
83481	EPPK1	epiplakin 1
6932	TCF7	transcription factor 7 (T-cell specific, HMG-box)
91120	ZNF682	zinc finger protein 682
51272	BET1L	blocked early in transport 1 homolog (S. cerevisiae)-like
729184	[No Symbol]	[No Name]
583	BBS2	Bardet-Biedl syndrome 2
9214	FAIM3	Fas apoptotic inhibitory molecule 3
94039	ZNF101	zinc finger protein 101
649299	RPL36AP51	ribosomal protein L36a pseudogene 51
64784	CRTC3	CREB regulated transcription coactivator 3
201626	PDE12	phosphodiesterase 12
100131944	[No Symbol]	[No Name]
7067	THRA	thyroid hormone receptor, alpha
25949	SYF2	SYF2 homolog, RNA splicing factor (S. cerevisiae)
22866	CNKSR2	connector enhancer of kinase suppressor of Ras 2
7343	UBTF	upstream binding transcription factor, RNA polymerase I
90321	ZNF766	zinc finger protein 766
57692	MAGEE1	melanoma antigen family E, 1
158293	FAM120AOS	family with sequence similarity 120A opposite strand
100131988	[No Symbol]	[No Name]
93035	PKHD1L1	polycystic kidney and hepatic disease 1 (autosomal recessive)-like 1
8445	DYRK2	dual-specificity tyrosine-(Y)-phosphorylation regulated kinase 2
152007	GLIPR2	GLI pathogenesis-related 2
7556	ZNF10	zinc finger protein 10
29083	GTPBP8	GTP-binding protein 8 (putative)
80024	SLC24A6	solute carrier family 24 (sodium/potassium/calcium exchanger), member 6
7533	YWHAH	tyrosine 3-monooxygenase/tryptophan 5-monooxygenase activation protein, eta polypeptide
1316	KLF6	Kruppel-like factor 6
643689	HNRNPA3P3	heterogeneous nuclear ribonucleoprotein A3 pseudogene 3
27284	SULT1B1	sulfotransferase family, cytosolic, 1B, member 1
55073	LRRC37A4	leucine rich repeat containing 37, member A4 (pseudogene)
84281	C2orf88	chromosome 2 open reading frame 88
79836	LONRF3	LON peptidase N-terminal domain and ring finger 3
4329	ALDH6A1	aldehyde dehydrogenase 6 family, member A1

78988	MRP63	mitochondrial ribosomal protein 63
5334	PLCL1	phospholipase C-like 1
27040	LAT	linker for activation of T cells
157489	SDAD1P1	SDA1 domain containing 1 pseudogene 1
80762	NDFIP1	Nedd4 family interacting protein 1
219749	ZNF25	zinc finger protein 25
10927	SPIN1	spindlin 1
5413	5-Sep	septin 5
80213	TM2D3	TM2 domain containing 3
50852	TRAT1	T cell receptor associated transmembrane adaptor 1
90007	MIDN	midnolin
3458	IFNG	interferon, gamma
146050	ZSCAN29	zinc finger and SCAN domain containing 29
10857	PGRMC1	progesterone receptor membrane component 1
254170	FBXO33	F-box protein 33
55251	PCMTD2	protein-L-isoaspartate (D-aspartate) O-methyltransferase domain containing 2
57134	MAN1C1	mannosidase, alpha, class 1C, member 1
84872	ZC3H10	zinc finger CCCH-type containing 10
100128269	[No Symbol]	[No Name]
145173	B3GALTL	beta 1,3-galactosyltransferase-like
54543	TOMM7	translocase of outer mitochondrial membrane 7 homolog (yeast)
5816	PVALB	parvalbumin
55340	GIMAP5	GTPase, IMAP family member 5
81606	LBH	limb bud and heart development homolog (mouse)
6903	TBCC	tubulin folding cofactor C
79956	ERMP1	endoplasmic reticulum metallopeptidase 1
100129956	LOC100129956	high mobility group nucleosomal binding domain 2 pseudogene
449645	SF3A3P2	splicing factor 3a, subunit 3 pseudogene 2
11168	PSIP1	PC4 and SFRS1 interacting protein 1
8345	HIST1H2BH	histone cluster 1, H2bh
22828	SCAF8	SR-related CTD-associated factor 8
440515	ZNF506	zinc finger protein 506
57708	MIER1	mesoderm induction early response 1 homolog (Xenopus laevis)
4118	MAL	mal, T-cell differentiation protein
283521	NCRNA00282	non-protein coding RNA 282

6232	RPS27	ribosomal protein S27
819	CAMLG	calcium modulating ligand
729627	PRR23A	proline rich 23A
64805	P2RY12	purinergic receptor P2Y, G-protein coupled, 12
7035	TFPI	tissue factor pathway inhibitor (lipoprotein-associated coagulation inhibitor)
58486	ZBED5	zinc finger, BED-type containing 5
5747	PTK2	PTK2 protein tyrosine kinase 2
2332	FMR1	fragile X mental retardation 1
55466	DNAJA4	DnaJ (Hsp40) homolog, subfamily A, member 4
26515	FXC1	fracture callus 1 homolog (rat)
100131308	[No Symbol]	[No Name]
4756	NEO1	neogenin 1
8365	HIST1H4H	histone cluster 1, H4h
338390	MRPS25P1	mitochondrial ribosomal protein S25 pseudogene 1
64393	ZMAT3	zinc finger, matrin-type 3
161436	EML5	echinoderm microtubule associated protein like 5
4674	NAP1L2	nucleosome assembly protein 1-like 2
1659	DHX8	DEAH (Asp-Glu-Ala-His) box polypeptide 8
145748	LYSMD4	LysM, putative peptidoglycan-binding, domain containing 4
51729	WBP11	WW domain binding protein 11
51132	RLIM	ring finger protein, LIM domain interacting
375287	RBM43	RNA binding motif protein 43
26292	MYCBP	c-myc binding protein
29080	CCDC59	coiled-coil domain containing 59
8988	HSPB3	heat shock 27kDa protein 3
9976	CLEC2B	C-type lectin domain family 2, member B
5504	PPP1R2	protein phosphatase 1, regulatory (inhibitor) subunit 2
5196	PF4	platelet factor 4
100131492	LOC100131492	PP12901
376940	ZC3H6	zinc finger CCCH-type containing 6
51440	HPCAL4	hippocalcin like 4
100132612	LOC100132612	hypothetical protein LOC100132612
5111	PCNA	proliferating cell nuclear antigen
441549	CDNF	cerebral dopamine neurotrophic factor
4040	LRP6	low density lipoprotein receptor-related protein 6

170575	GIMAP1	GTPase, IMAP family member 1
115024	NT5C3L	5'-nucleotidase, cytosolic III-like
23007	PLCH1	phospholipase C, eta 1
401718	OR5BK1P	olfactory receptor, family 5, subfamily BK, member 1 pseudogene
84224	NBPF3	neuroblastoma breakpoint family, member 3
3300	DNAJB2	DnaJ (Hsp40) homolog, subfamily B, member 2
2053	EPHX2	epoxide hydrolase 2, cytoplasmic
90121	TSR2	TSR2, 20S rRNA accumulation, homolog (S. cerevisiae)
5938	RBMS1P1	RNA binding motif, single stranded interacting protein 1 pseudogene 1
100131366	LOC100131366	hypothetical LOC100131366
84892	C3orf39	chromosome 3 open reading frame 39
79088	ZNF426	zinc finger protein 426
81894	SLC25A28	solute carrier family 25, member 28
1473	CST5	cystatin D
23507	LRRC8B	leucine rich repeat containing 8 family, member B
8436	SDPR	serum deprivation response
23484	LEPROTL1	leptin receptor overlapping transcript-like 1
643951	[No Symbol]	[No Name]
729687	HMGN2P25	high mobility group nucleosomal binding domain 2 pseudogene 25
84299	C17orf37	chromosome 17 open reading frame 37
147837	ZNF563	zinc finger protein 563
79144	PPDPF	pancreatic progenitor cell differentiation and proliferation factor homolog (zebrafish)
51237	MZB1	marginal zone B and B1 cell-specific protein
54557	SGTB	small glutamine-rich tetratricopeptide repeat (TPR)-containing, beta
26092	TOR1AIP1	torsin A interacting protein 1
100129973	LOC100129973	hypothetical LOC100129973
374868	ATP9B	ATPase, class II, type 9B
348158	ACSM2B	acyl-CoA synthetase medium-chain family member 2B
729076	[No Symbol]	[No Name]
6886	TAL1	T-cell acute lymphocytic leukemia 1
728914	LOC728914	histone H3.3-like
8722	CTSF	cathepsin F
28429	IGHV3-38	immunoglobulin heavy variable 3-38 (non-functional)
4601	MXI1	MAX interactor 1
345456	PFN3	profilin 3

22915	MMRN1	multimerin 1
440067	LOC440067	caspase-1-like
163259	DENND2C	DENN/MADD domain containing 2C
54414	SIAE	sialic acid acetylesterase
145259	RPSAP4	ribosomal protein SA pseudogene 4
100130880	LOC100130880	hypothetical protein LOC100130880
55363	HEMGN	hemogen
55070	DET1	de-etiolated homolog 1 (Arabidopsis)
29058	C20orf30	chromosome 20 open reading frame 30
26255	PTTG3P	pituitary tumor-transforming 3, pseudogene
54726	OTUD4	OTU domain containing 4
100129409	LOC100129409	hypothetical LOC100129409
1183	CLCN4	chloride channel 4
55623	THUMPD1	THUMP domain containing 1
91300	C19orf22	chromosome 19 open reading frame 22
1950	EGF	epidermal growth factor
55007	FAM118A	family with sequence similarity 118, member A
195828	ZNF367	zinc finger protein 367
5139	PDE3A	phosphodiesterase 3A, cGMP-inhibited
100132073	LOC100132073	cyclin B2 pseudogene
79842	ZBTB3	zinc finger and BTB domain containing 3
100131950	LOC100131950	methyltransferase like 5 pseudogene
117178	SSX2IP	synovial sarcoma, X breakpoint 2 interacting protein
219681	ARMC3	armadillo repeat containing 3
10322	SMYD5	SMYD family member 5
83744	ZNF484	zinc finger protein 484
116068	LYSMD3	LysM, putative peptidoglycan-binding, domain containing 3
406891	MIRLET7I	microRNA let-7i
3384	ICAM2	intercellular adhesion molecule 2
100130840	[No Symbol]	[No Name]
390190	OR5B2	olfactory receptor, family 5, subfamily B, member 2
10693	CCT6B	chaperonin containing TCP1, subunit 6B (zeta 2)
55793	FAM63A	family with sequence similarity 63, member A
647115	FLJ36848	hypothetical LOC647115
121551	BTBD11	BTB (POZ) domain containing 11

442017	LOC442017	interferon induced transmembrane protein pseudogene
51142	CHCHD2	coiled-coil-helix-coiled-coil-helix domain containing 2
83695	C12orf32	chromosome 12 open reading frame 32
9848	MFAP3L	microfibrillar-associated protein 3-like
5469	MED1	mediator complex subunit 1
285331	CCDC66	coiled-coil domain containing 66
100129472	PTP4A1P6	protein tyrosine phosphatase type IVA, member 1 pseudogene 6
440574	C1orf151	chromosome 1 open reading frame 151
51759	C9orf78	chromosome 9 open reading frame 78
93323	HAUS8	HAUS augmin-like complex, subunit 8
84033	OBSCN	obscurin, cytoskeletal calmodulin and titin-interacting RhoGEF
115752	DIS3L	DIS3 mitotic control homolog (S. cerevisiae)-like
643	CXCR5	chemokine (C-X-C motif) receptor 5
94121	SYTL4	synaptotagmin-like 4
23370	ARHGEF18	Rho/Rac guanine nucleotide exchange factor (GEF) 18
84779	NAA11	N(alpha)-acetyltransferase 11, NatA catalytic subunit
26258	PLDN	pallidin homolog (mouse)
11341	SCRG1	stimulator of chondrogenesis 1
83746	L3MBTL2	l(3)mbt-like 2 (Drosophila)
143888	KDELC2	KDEL (Lys-Asp-Glu-Leu) containing 2
92595	ZNF764	zinc finger protein 764
8349	HIST2H2BE	histone cluster 2, H2be
84886	C1orf198	chromosome 1 open reading frame 198
152006	RNF38	ring finger protein 38
254228	FAM26E	family with sequence similarity 26, member E
2273	FHL1	four and a half LIM domains 1
112487	C14orf126	chromosome 14 open reading frame 126
55151	TMEM38B	transmembrane protein 38B
79710	MORC4	MORC family CW-type zinc finger 4
6224	RPS20	ribosomal protein S20
285636	C5orf51	chromosome 5 open reading frame 51
10911	UTS2	urotensin 2
100128469	LOC100128469	small nuclear ribonucleoprotein polypeptide C pseudogene
51206	GP6	glycoprotein VI (platelet)
340542	BEX5	brain expressed, X-linked 5

5669	PSG1	pregnancy specific beta-1-glycoprotein 1
649446	FLJ35776	hypothetical LOC649446
8368	HIST1H4L	histone cluster 1, H4l
254427	C10orf47	chromosome 10 open reading frame 47
26049	FAM169A	family with sequence similarity 169, member A
2123	EVI2A	ecotropic viral integration site 2A
3017	HIST1H2BD	histone cluster 1, H2bd
126375	ZNF792	zinc finger protein 792
3945	LDHB	lactate dehydrogenase B
100131284	LOC100131284	proteasome (prosome, macropain) 26S subunit, non-ATPase, 14 pseudogene
27244	SESN1	sestrin 1
5252	PHF1	PHD finger protein 1
4747	NEFL	neurofilament, light polypeptide
7166	TPH1	tryptophan hydroxylase 1
100130231	LOC100130231	similar to hCG1814455
729013	LOC729013	hypothetical LOC729013
497661	C18orf32	chromosome 18 open reading frame 32
100129502	LOC100129502	hypothetical protein LOC100129502
50486	G0S2	G0/G1switch 2
1389	CREBL2	cAMP responsive element binding protein-like 2
27316	RBMX	RNA binding motif protein, X-linked
100128339	BCAS2P3	breast carcinoma amplified sequence 2 pseudogene 3
9743	ARHGAP32	Rho GTPase activating protein 32
54664	TMEM106B	transmembrane protein 106B
26995	TRUB2	TruB pseudouridine (psi) synthase homolog 2 (E. coli)
23189	KANK1	KN motif and ankyrin repeat domains 1
100130218	[No Symbol]	[No Name]
5619	PRM1	protamine 1
8821	INPP4B	inositol polyphosphate-4-phosphatase, type II, 105kDa
94274	PPP1R14A	protein phosphatase 1, regulatory (inhibitor) subunit 14A
84326	C16orf13	chromosome 16 open reading frame 13
728711	[No Symbol]	[No Name]
253039	LOC253039	hypothetical LOC253039
131118	DNAJC19	DnaJ (Hsp40) homolog, subfamily C, member 19
647209	[No Symbol]	[No Name]

84101	USP44	ubiquitin specific peptidase 44
29965	C16orf5	chromosome 16 open reading frame 5
163882	CNST	consortin, connexin sorting protein
9516	LITAF	lipopolysaccharide-induced TNF factor
1265	CNN2	calponin 2
2781	GNAZ	guanine nucleotide binding protein (G protein), alpha z polypeptide
10235	RASGRP2	RAS guanyl releasing protein 2 (calcium and DAG-regulated)
11102	RPP14	ribonuclease P/MRP 14kDa subunit
2841	GPR18	G protein-coupled receptor 18
84707	BEX2	brain expressed X-linked 2
81788	NUAK2	NUAK family, SNF1-like kinase, 2
267012	DAOA	D-amino acid oxidase activator
4700	NDUFA6	NADH dehydrogenase (ubiquinone) 1 alpha subcomplex, 6, 14kDa
8577	TMEFF1	transmembrane protein with EGF-like and two follistatin-like domains 1
23221	RHOBTB2	Rho-related BTB domain containing 2
140775	SMCR8	Smith-Magenis syndrome chromosome region, candidate 8
2959	GTF2B	general transcription factor IIB
10944	C11orf58	chromosome 11 open reading frame 58
10782	ZNF274	zinc finger protein 274
1816	DRD5	dopamine receptor D5
2354	FOSB	FBJ murine osteosarcoma viral oncogene homolog B
729451	LOC729451	hypothetical protein LOC729451
389023	LOC389023	hypothetical LOC389023
1983	EIF5	eukaryotic translation initiation factor 5
22877	MLXIP	MLX interacting protein
7572	ZNF24	zinc finger protein 24
84987	C12orf62	chromosome 12 open reading frame 62
8405	SPOP	speckle-type POZ protein
642280	ZNF876P	zinc finger protein 876, pseudogene
646268	LOC646268	hCG1654703
7048	TGFBR2	transforming growth factor, beta receptor II (70/80kDa)
51091	SEPSECS	Sep (O-phosphoserine) tRNA:Sec (selenocysteine) tRNA synthase
4956	ODF1	outer dense fiber of sperm tails 1
79758	DHRS12	dehydrogenase/reductase (SDR family) member 12
9093	DNAJA3	DnaJ (Hsp40) homolog, subfamily A, member 3

8029	CUBN	cubilin (intrinsic factor-cobalamin receptor)
93550	ANUBL1	AN1, ubiquitin-like, homolog (Xenopus laevis)
10455	ECI2	enoyl-CoA delta isomerase 2
57091	CASS4	Cas scaffolding protein family member 4
10472	ZNF238	zinc finger protein 238
441733	PRKXP1	protein kinase, X-linked, pseudogene 1
51554	CCRL1	chemokine (C-C motif) receptor-like 1
3575	IL7R	interleukin 7 receptor
100129412	[No Symbol]	[No Name]
2882	GPX7	glutathione peroxidase 7
55766	H2AFJ	H2A histone family, member J
3009	HIST1H1B	histone cluster 1, H1b
84460	ZMAT1	zinc finger, matrin-type 1
100129969	LOC100129969	transmembrane protein C9orf144B pseudogene
140735	DYNLL2	dynein, light chain, LC8-type 2
729314	LOC729314	putative POM121-like protein 1-like
121642	ALKBH2	alkB, alkylation repair homolog 2 (E. coli)
168620	BHLHA15	basic helix-loop-helix family, member a15
10038	PARP2	poly (ADP-ribose) polymerase 2
387990	TOMM20L	translocase of outer mitochondrial membrane 20 homolog (yeast)-like
64282	PAPD5	PAP associated domain containing 5
100113384	SNORD123	small nucleolar RNA, C/D box 123
1397	CRIP2	cysteine-rich protein 2
199777	ZNF626	zinc finger protein 626
100129353	[No Symbol]	[No Name]
10868	USP20	ubiquitin specific peptidase 20
6668	SP2	Sp2 transcription factor
731282	LOC731282	hypothetical protein LOC731282
8328	GFI1B	growth factor independent 1B transcription repressor
328	APEX1	APEX nuclease (multifunctional DNA repair enzyme) 1
25801	GCA	grancalcin, EF-hand calcium binding protein
55297	CCDC91	coiled-coil domain containing 91
64410	KLHL25	kelch-like 25 (Drosophila)
10054	UBA2	ubiquitin-like modifier activating enzyme 2
167	CRISP1	cysteine-rich secretory protein 1

389419	[No Symbol]	[No Name]
407025	MIR29B2	microRNA 29b-2
100131662	LOC100131662	hypothetical LOC100131662
23085	ERC1	ELKS/RAB6-interacting/CAST family member 1
29094	HSPC159	galectin-related protein
100131793	[No Symbol]	[No Name]
135293	PM20D2	peptidase M20 domain containing 2
374879	ZNF699	zinc finger protein 699
696	BTN1A1	butyrophilin, subfamily 1, member A1
161502	C15orf26	chromosome 15 open reading frame 26
79064	TMEM223	transmembrane protein 223
8347	HIST1H2BC	histone cluster 1, H2bc
1429	CRYZ	crystallin, zeta (quinone reductase)
84541	KBTBD8	kelch repeat and BTB (POZ) domain containing 8
85443	DCLK3	doublecortin-like kinase 3
22876	INPP5F	inositol polyphosphate-5-phosphatase F
57097	PARP11	poly (ADP-ribose) polymerase family, member 11
266812	NAP1L5	nucleosome assembly protein 1-like 5
123	PLIN2	perilipin 2
8330	HIST1H2AK	histone cluster 1, H2ak
23174	ZCCHC14	zinc finger, CCHC domain containing 14
201229	C17orf108	chromosome 17 open reading frame 108
6117	RPA1	replication protein A1, 70kDa
730651	LOC730651	zinc finger protein 709-like
100132345	[No Symbol]	[No Name]
57820	CCNB1IP1	cyclin B1 interacting protein 1, E3 ubiquitin protein ligase
10209	EIF1	eukaryotic translation initiation factor 1
729992	ST13P1	suppression of tumorigenicity 13 (colon carcinoma) (Hsp70 interacting protein) pseudogene 1
84132	USP42	ubiquitin specific peptidase 42
644459	LOC644459	BEN domain containing 3 pseudogene
644397	LOC644397	leucine-rich repeat-containing protein 37A2-like
79752	ZFAND1	zinc finger, AN1-type domain 1
729954	[No Symbol]	[No Name]
225	ABCD2	ATP-binding cassette, sub-family D (ALD), member 2
93164	HTR7P1	5-hydroxytryptamine (serotonin) receptor 7 pseudogene 1

643445	LOC643445	tripartite motif containing 43 pseudogene
4953	ODC1	ornithine decarboxylase 1
403274	OR5H15	olfactory receptor, family 5, subfamily H, member 15
22986	SORCS3	sortilin-related VPS10 domain containing receptor 3
57396	CLK4	CDC-like kinase 4
128312	HIST3H2BB	histone cluster 3, H2bb
7693	ZNF134	zinc finger protein 134
100131183	HMGN2P30	high mobility group nucleosomal binding domain 2 pseudogene 30
284546	C1orf185	chromosome 1 open reading frame 185
257160	RNF214	ring finger protein 214
165545	DQX1	DEAQ box RNA-dependent ATPase 1
100129032	GLYATL1P1	glycine-N-acyltransferase-like 1 pseudogene 1
353329	PSIP1P1	PC4 and SFRS1 interacting protein 1 pseudogene 1
5892	RAD51L3	RAD51-like 3 (S. cerevisiae)
401884	MGC57346	hypothetical LOC401884
387535	HCRP1	hepatocellular carcinoma-related HCRP1
158905	PHF10P1	PHD finger protein 10 pseudogene 1
83699	SH3BGRL2	SH3 domain binding glutamic acid-rich protein like 2
199834	LCE4A	late cornified envelope 4A
79066	METTL16	methyltransferase like 16
23111	SPG20	spastic paraplegia 20 (Troyer syndrome)
3301	DNAJA1	DnaJ (Hsp40) homolog, subfamily A, member 1
55906	ZC4H2	zinc finger, C4H2 domain containing
7316	UBC	ubiquitin C
387654	[No Symbol]	[No Name]
646934	LOC646934	putative golgin subfamily A member 6D-like
22869	ZNF510	zinc finger protein 510
4149	MAX	MYC associated factor X
11080	DNAJB4	DnaJ (Hsp40) homolog, subfamily B, member 4
55287	TMEM40	transmembrane protein 40
55619	DOCK10	dedicator of cytokinesis 10
23588	KLHDC2	kelch domain containing 2
894	CCND2	cyclin D2
58526	MID1IP1	MID1 interacting protein 1 (gastrulation specific G12 homolog (zebrafish))
9862	MED24	mediator complex subunit 24

79695	GALNT12	UDP-N-acetyl-alpha-D-galactosamine:polypeptide N-acetylgalactosaminyltransferase 12 (GalNAc-T12)
267	AMFR	autocrine motility factor receptor
9975	NR1D2	nuclear receptor subfamily 1, group D, member 2
11128	POLR3A	polymerase (RNA) III (DNA directed) polypeptide A, 155kDa
472	ATM	ataxia telangiectasia mutated
81325	OR4A18P	olfactory receptor, family 4, subfamily A, member 18 pseudogene
1964	EIF1AX	eukaryotic translation initiation factor 1A, X-linked
140461	ASB8	ankyrin repeat and SOCS box containing 8
22898	DENND3	DENN/MADD domain containing 3
4077	NBR1	neighbor of BRCA1 gene 1
58515	SELK	selenoprotein K
100133015	[No Symbol]	[No Name]
9360	PPIG	peptidylprolyl isomerase G (cyclophilin G)
3434	IFIT1	interferon-induced protein with tetratricopeptide repeats 1
8336	HIST1H2AM	histone cluster 1, H2am
814	CAMK4	calcium/calmodulin-dependent protein kinase IV
401152	C4orf3	chromosome 4 open reading frame 3
25793	FBXO7	F-box protein 7
55602	CDKN2AIP	CDKN2A interacting protein
64772	ENGASE	endo-beta-N-acetylglucosaminidase
11320	MGAT4A	mannosyl (alpha-1,3-)-glycoprotein beta-1,4-N-acetylglucosaminyltransferase, isozyme A
402096	LOC402096	uncharacterized protein C6orf106-like
27018	NGFRAP1	nerve growth factor receptor (TNFRSF16) associated protein 1
100130731	LOC100130731	proteasome (prosome, macropain) subunit, beta type, 1 pseudogene
10627	MYL12A	myosin, light chain 12A, regulatory, non-sarcomeric
1776	DNASE1L3	deoxyribonuclease I-like 3
728545	[No Symbol]	[No Name]
54496	PRMT7	protein arginine methyltransferase 7
728504	[No Symbol]	[No Name]
9252	RPS6KA5	ribosomal protein S6 kinase, 90kDa, polypeptide 5
201931	TMEM192	transmembrane protein 192
84919	PPP1R15B	protein phosphatase 1, regulatory (inhibitor) subunit 15B
4013	VWA5A	von Willebrand factor A domain containing 5A
55622	TTC27	tetratricopeptide repeat domain 27
9572	NR1D1	nuclear receptor subfamily 1, group D, member 1

5788	PTPRC	protein tyrosine phosphatase, receptor type, C
283537	SLC46A3	solute carrier family 46, member 3
9342	SNAP29	synaptosomal-associated protein, 29kDa
6124	RPL4	ribosomal protein L4
151457	LOC151457	developmentally regulated GTP binding protein 1 pseudogene
646344	LOC646344	similar to hCG2042391
619450	ATP6V1G1P2	ATPase, H+ transporting, lysosomal 13kDa, V1 subunit G1 pseudogene 2
83640	FAM103A1	family with sequence similarity 103, member A1
93556	EGFEM1P	EGF-like and EMI domain containing 1, pseudogene
56919	DHX33	DEAH (Asp-Glu-Ala-His) box polypeptide 33
100131049	[No Symbol]	[No Name]
26530	OR12D1P	olfactory receptor, family 12, subfamily D, member 1 pseudogene
8303	SNN	stannin
51065	RPS27L	ribosomal protein S27-like
9468	PCYT1B	phosphate cytidylyltransferase 1, choline, beta
9831	ZNF623	zinc finger protein 623
54386	TERF2IP	telomeric repeat binding factor 2, interacting protein
283876	FLJ39639	hypothetical protein FLJ39639
57622	LRFN1	leucine rich repeat and fibronectin type III domain containing 1
116444	GRIN3B	glutamate receptor, ionotropic, N-methyl-D-aspartate 3B
79269	DCAF10	DDB1 and CUL4 associated factor 10
219437	OR5L1	olfactory receptor, family 5, subfamily L, member 1
148003	LGALS16	beta-galactoside-binding lectin
203286	ANKS6	ankyrin repeat and sterile alpha motif domain containing 6
100128537	C1orf132	chromosome 1 open reading frame 132
10606	PAICS	phosphoribosylaminoimidazole carboxylase, phosphoribosylaminoimidazole succinocarboxamide synthetase
442215	LOC442215	eukaryotic translation elongation factor 1 alpha 2 pseudogene
58497	PRUNE	prune homolog (Drosophila)
59286	UBL5	ubiquitin-like 5
154881	KCTD7	potassium channel tetramerisation domain containing 7
51535	PPHLN1	periphilin 1
1742	DLG4	discs, large homolog 4 (Drosophila)
100128197	[No Symbol]	[No Name]
55690	PACS1	phosphofurin acidic cluster sorting protein 1
57463	AMIGO1	adhesion molecule with Ig-like domain 1

1123	CHN1	chimerin (chimaerin) 1
81110	OR11J2P	olfactory receptor, family 11, subfamily J, member 2 pseudogene
134510	UBLCP1	ubiquitin-like domain containing CTD phosphatase 1
646555	LOC646555	AT rich interactive domain 3B pseudogene
6990	DYNLT3	dynein, light chain, Tctex-type 3
7388	UQCRH	ubiquinol-cytochrome c reductase hinge protein
441666	LOC441666	zinc finger protein 91 pseudogene
644303	LOC644303	ATP-dependent RNA helicase DDX24-like
2113	ETS1	v-ets erythroblastosis virus E26 oncogene homolog 1 (avian)
26289	AK5	adenylate kinase 5
54970	TTC12	tetratricopeptide repeat domain 12
7259	TSPYL1	TSPY-like 1
8575	PRKRA	protein kinase, interferon-inducible double stranded RNA dependent activator
93621	MRFAP1	Morf4 family associated protein 1
10206	TRIM13	tripartite motif containing 13
79811	SLTM	SAFB-like, transcription modulator
10162	LPCAT3	lysophosphatidylcholine acyltransferase 3
284358	MAMSTR	MEF2 activating motif and SAP domain containing transcriptional regulator
54816	ZNF280D	zinc finger protein 280D
9751	SNPH	syntaphilin
648665	LOC648665	hypothetical LOC648665
80047	[No Symbol]	[No Name]
11059	WWP1	WW domain containing E3 ubiquitin protein ligase 1
10329	TMEM5	transmembrane protein 5
153364	MBLAC2	metallo-beta-lactamase domain containing 2
2947	GSTM3	glutathione S-transferase mu 3 (brain)
22984	PDCD11	programmed cell death 11
155370	SBDSP1	Shwachman-Bodian-Diamond syndrome pseudogene 1
100129482	ZNF37BP	zinc finger protein 37B, pseudogene
152118	C3orf79	chromosome 3 open reading frame 79
4064	CD180	CD180 molecule
51669	TMEM66	transmembrane protein 66
100132815	LOC100132815	hypothetical LOC100132815
23157	6-Sep	septin 6
6039	RNASE6	ribonuclease, RNase A family, k6

373	TRIM23	tripartite motif containing 23
842	CASP9	caspase 9, apoptosis-related cysteine peptidase
3007	HIST1H1D	histone cluster 1, H1d
23002	DAAM1	dishevelled associated activator of morphogenesis 1
1848	DUSP6	dual specificity phosphatase 6
92806	CENPBD1	CENPB DNA-binding domains containing 1
5603	MAPK13	mitogen-activated protein kinase 13
654369	RPL37P5	ribosomal protein L37 pseudogene 5
100126487	TRNAV-UAC	transfer RNA valine (anticodon UAC)
731223	LOC731223	hypothetical LOC731223
648154	[No Symbol]	[No Name]
2814	GP5	glycoprotein V (platelet)
79640	C22orf46	chromosome 22 open reading frame 46
1880	GPR183	G protein-coupled receptor 183
729678	LOC729678	hypothetical LOC729678
51411	BIN2	bridging integrator 2
135250	RAET1E	retinoic acid early transcript 1E
9550	ATP6V1G1	ATPase, H+ transporting, lysosomal 13kDa, V1 subunit G1
729614	FLJ37453	hypothetical LOC729614
8366	HIST1H4B	histone cluster 1, H4b
728279	KRTAP2-2	keratin associated protein 2-2
3303	HSPA1A	heat shock 70kDa protein 1A
100129203	LOC100129203	hypothetical LOC100129203
4673	NAP1L1	nucleosome assembly protein 1-like 1
259173	ALS2CL	ALS2 C-terminal like
342898	SYCN	syncollin
9221	NOLC1	nucleolar and coiled-body phosphoprotein 1
54737	MPHOSPH8	M-phase phosphoprotein 8
9046	DOK2	docking protein 2, 56kDa
280658	SSX7	synovial sarcoma, X breakpoint 7
199964	TMEM61	transmembrane protein 61
25865	PRKD2	protein kinase D2
51258	MRPL51	mitochondrial ribosomal protein L51
642852	LOC642852	hypothetical LOC642852
124274	GPR139	G protein-coupled receptor 139

85013	TMEM128	transmembrane protein 128
286336	FAM78A	family with sequence similarity 78, member A
11236	RNF139	ring finger protein 139
84146	ZNF644	zinc finger protein 644
9836	LCMT2	leucine carboxyl methyltransferase 2
85329	LGALS12	lectin, galactoside-binding, soluble, 12
6229	RPS24	ribosomal protein S24
100131041	LOC100131041	FK506 binding protein pseudogene
91603	ZNF830	zinc finger protein 830
4130	MAP1A	microtubule-associated protein 1A
79672	FN3KRP	fructosamine 3 kinase related protein
23658	LSM5	LSM5 homolog, U6 small nuclear RNA associated (S. cerevisiae)
8681	JMJD7-PLA2G4B	JMJD7-PLA2G4B readthrough
285704	RGMB	RGM domain family, member B
100129562	[No Symbol]	[No Name]
359804	MRPS29P1	mitochondrial ribosomal protein S29 pseudogene 1
2193	FARSA	phenylalanyl-tRNA synthetase, alpha subunit
9063	PIAS2	protein inhibitor of activated STAT, 2
391119	OR10J7P	olfactory receptor, family 10, subfamily J, member 7 pseudogene
283507	SUGT1P3	suppressor of G2 allele of SKP1 (S. cerevisiae) pseudogene 3
1486	CTBS	chitobiase, di-N-acetyl-
405754	ERVFRD-1	endogenous retrovirus group FRD, member 1
10210	TOPORS	topoisomerase I binding, arginine/serine-rich, E3 ubiquitin protein ligase
64773	FAM113A	family with sequence similarity 113, member A
400652	RPS3AP49	ribosomal protein S3a pseudogene 49
4306	NR3C2	nuclear receptor subfamily 3, group C, member 2
136895	C7orf31	chromosome 7 open reading frame 31
85865	GTPBP10	GTP-binding protein 10 (putative)
59348	ZNF350	zinc finger protein 350
11319	ECD	ecdysoneless homolog (Drosophila)
3111	HLA-DOA	major histocompatibility complex, class II, DO alpha
57447	NDRG2	NDRG family member 2
387647	LOC387647	patched domain containing 3 pseudogene
81794	ADAMTS10	ADAM metallopeptidase with thrombospondin type 1 motif, 10
79979	TRMT2B	TRM2 tRNA methyltransferase 2 homolog B (S. cerevisiae)

7090	TLE3	transducin-like enhancer of split 3 (E(sp1) homolog, Drosophila)
10365	KLF2	Kruppel-like factor 2 (lung)
26273	FBXO3	F-box protein 3
643067	LOC643067	HORMA domain containing 1 pseudogene
283953	TMEM114	transmembrane protein 114
8892	EIF2B2	eukaryotic translation initiation factor 2B, subunit 2 beta, 39kDa
100132049	RPS19P4	ribosomal protein S19 pseudogene 4
8313	AXIN2	axin 2
57587	KIAA1430	KIAA1430
585	BBS4	Bardet-Biedl syndrome 4
727789	RWDD1P3	RWD domain containing 1 pseudogene 3
84837	C14orf128	chromosome 14 open reading frame 128
5924	RASGRF2	Ras protein-specific guanine nucleotide-releasing factor 2
645446	LOC645446	hypothetical LOC645446
100130249	PP2672	hypothetical LOC100130249
2532	DARC	Duffy blood group, chemokine receptor
100133139	RPL12P41	ribosomal protein L12 pseudogene 41
10534	SSSCA1	Sjogren syndrome/scleroderma autoantigen 1
158983	H2BFWT	H2B histone family, member W, testis-specific
100129204	LOC100129204	hypothetical protein LOC100129204
8335	HIST1H2AB	histone cluster 1, H2ab
8434	RECK	reversion-inducing-cysteine-rich protein with kazal motifs
85444	LRRCC1	leucine rich repeat and coiled-coil domain containing 1
54942	C9orf6	chromosome 9 open reading frame 6
25845	PP7080	hypothetical LOC25845
84318	CCDC77	coiled-coil domain containing 77
56257	MEPCE	methylphosphate capping enzyme
117289	TAGAP	T-cell activation RhoGTPase activating protein
7536	SF1	splicing factor 1
11345	GABARAPL2	GABA(A) receptor-associated protein-like 2
54900	LAX1	lymphocyte transmembrane adaptor 1
51735	RAPGEF6	Rap guanine nucleotide exchange factor (GEF) 6
29079	MED4	mediator complex subunit 4
64167	ERAP2	endoplasmic reticulum aminopeptidase 2
284365	MGC45922	hypothetical LOC284365

55860	ACTR10	actin-related protein 10 homolog (S. cerevisiae)
23468	CBX5	chromobox homolog 5
84313	VPS25	vacuolar protein sorting 25 homolog (S. cerevisiae)
221830	TWISTNB	TWIST neighbor
729011	[No Symbol]	[No Name]
81440	OR6R1P	olfactory receptor, family 6, subfamily R, member 1 pseudogene
2483	FRG1	FSHD region gene 1
8678	BECN1	beclin 1, autophagy related
100130872	LOC100130872	hypothetical LOC100130872
22864	R3HDM2	R3H domain containing 2
116984	ARAP2	ArfGAP with RhoGAP domain, ankyrin repeat and PH domain 2
400539	FLJ27243	FLJ27243 protein
3953	LEPR	leptin receptor
100132010	[No Symbol]	[No Name]
158714	[No Symbol]	[No Name]
6750	SST	somatostatin
28976	ACAD9	acyl-CoA dehydrogenase family, member 9
7376	NR1H2	nuclear receptor subfamily 1, group H, member 2
23168	RTF1	Rtf1, Paf1/RNA polymerase II complex component, homolog (S. cerevisiae)
90673	PPP1R3E	protein phosphatase 1, regulatory (inhibitor) subunit 3E
8698	S1PR4	sphingosine-1-phosphate receptor 4
84692	CCDC54	coiled-coil domain containing 54
28951	TRIB2	tribbles homolog 2 (Drosophila)
231	AKR1B1	aldo-keto reductase family 1, member B1 (aldose reductase)
8302	KLRC4	killer cell lectin-like receptor subfamily C, member 4
5728	PTEN	phosphatase and tensin homolog
51103	NDUFAF1	NADH dehydrogenase (ubiquinone) 1 alpha subcomplex, assembly factor 1
29107	NXT1	NTF2-like export factor 1
387590	psiTPTE22	TPTE pseudogene
401251	C6orf26	chromosome 6 open reading frame 26
50964	SOST	sclerostin
8794	TNFRSF10C	tumor necrosis factor receptor superfamily, member 10c, decoy without an intracellular domain
100129854	LOC100129854	arginase, type II pseudogene
7336	UBE2V2	ubiquitin-conjugating enzyme E2 variant 2
55509	BATF3	basic leucine zipper transcription factor, ATF-like 3

145781	GCOM1	GRINL1A complex locus
643072	LOC643072	hypothetical LOC643072
90139	TSPAN18	tetraspanin 18
643977	FLJ32255	hypothetical LOC643977
391121	OR10J4	olfactory receptor, family 10, subfamily J, member 4 (gene/pseudogene)
5154	PDGFA	platelet-derived growth factor alpha polypeptide
391465	LOC391465	STIP1 homology and U-box containing protein 1 pseudogene
79614	[No Symbol]	[No Name]
121054	KRT18P20	keratin 18 pseudogene 20
339983	NAT8L	N-acetyltransferase 8-like (GCN5-related, putative)
27340	UTP20	UTP20, small subunit (SSU) processome component, homolog (yeast)
9792	SERTAD2	SERTA domain containing 2
126767	AADACL3	arylacetamide deacetylase-like 3
643008	C17orf109	chromosome 17 open reading frame 109
441046	GYPELOC441046	glucuronidase, beta pseudogene
3796	KIF2A	kinesin heavy chain member 2A
642635	LOC642635	tubulin, alpha 4a pseudogene
637	BID	BH3 interacting domain death agonist
6609	SMPD1	sphingomyelin phosphodiesterase 1, acid lysosomal
171568	POLR3H	polymerase (RNA) III (DNA directed) polypeptide H (22.9kD)
284	ANGPT1	angiopoietin 1
83719	YPEL3	yippee-like 3 (Drosophila)
1787	TRDMT1	tRNA aspartic acid methyltransferase 1
51526	C20orf111	chromosome 20 open reading frame 111
29078	NDUFAF4	NADH dehydrogenase (ubiquinone) 1 alpha subcomplex, assembly factor 4
339290	LOC339290	hypothetical LOC339290
641638	SNHG6	small nucleolar RNA host gene 6 (non-protein coding)
6588	SLN	sarcolipin
730079	[No Symbol]	[No Name]
729560	[No Symbol]	[No Name]
196415	C12orf77	chromosome 12 open reading frame 77
255231	MCOLN2	mucolipin 2
57599	WDR48	WD repeat domain 48
82500	PKP2P1	plakophilin 2 pseudogene 1
197370	NSMCE1	non-SMC element 1 homolog (S. cerevisiae)

9610	RIN1	Ras and Rab interactor 1
301	ANXA1	annexin A1
1493	CTLA4	cytotoxic T-lymphocyte-associated protein 4
79888	LPCAT1	lysophosphatidylcholine acyltransferase 1
55108	BSDC1	BSD domain containing 1
374443	LOC374443	CLR pseudogene
4656	MYOG	myogenin (myogenic factor 4)
23014	FBXO21	F-box protein 21
6289	SAA2	serum amyloid A2
503569	FLJ35946	hypothetical protein FLJ35946
3298	HSF2	heat shock transcription factor 2
84826	SFT2D3	SFT2 domain containing 3
91039	DPP9	dipeptidyl-peptidase 9
23131	GPATCH8	G patch domain containing 8
147912	SIX5	SIX homeobox 5
729141	LOC729141	chromosome 18 open reading frame 25 pseudogene
127018	LYPLAL1	lysophospholipase-like 1
139818	DOCK11	dedicator of cytokinesis 11
221178	SPATA13	spermatogenesis associated 13
8856	NR1I2	nuclear receptor subfamily 1, group I, member 2
4067	LYN	v-yes-1 Yamaguchi sarcoma viral related oncogene homolog
100131851	[No Symbol]	[No Name]
8732	RNGTT	RNA guanylyltransferase and 5'-phosphatase
57562	KIAA1377	KIAA1377
1203	CLN5	ceroid-lipofuscinosis, neuronal 5
10600	USP16	ubiquitin specific peptidase 16
2353	FOS	FBJ murine osteosarcoma viral oncogene homolog
57690	TNRC6C	trinucleotide repeat containing 6C
1939	EIF2D	eukaryotic translation initiation factor 2D
26205	GMEB2	glucocorticoid modulatory element binding protein 2
392371	KRT18P13	keratin 18 pseudogene 13
91574	C12orf65	chromosome 12 open reading frame 65
79857	FLJ13224	hypothetical LOC79857
7353	UFD1L	ubiquitin fusion degradation 1 like (yeast)
1107	CHD3	chromodomain helicase DNA binding protein 3

162993	ZNF846	zinc finger protein 846
27031	NPHP3	nephronophthisis 3 (adolescent)
51650	MRPS33	mitochondrial ribosomal protein S33
145389	SLC38A6	solute carrier family 38, member 6
394	ARHGAP5	Rho GTPase activating protein 5
100131135	LOC100131135	methylenetetrahydrofolate dehydrogenase (NADP+ dependent) 2, methenyltetrahydrofolate cyclohydrolase pseudogene
7507	XPA	xeroderma pigmentosum, complementation group A
7534	YWHAZ	tyrosine 3-monooxygenase/tryptophan 5-monooxygenase activation protein, zeta polypeptide
286042	FLJ10661	family with sequence similarity 86, member A pseudogene
100130240	RPS5P2	ribosomal protein S5 pseudogene 2
100131279	LOC100131279	chromosome 2 open reading frame 69 pseudogene
919	CD247	CD247 molecule
158866	ZDHHC15	zinc finger, DHHC-type containing 15
401207	C5orf63	chromosome 5 open reading frame 63
340481	ZDHHC21	zinc finger, DHHC-type containing 21
8021	NUP214	nucleoporin 214kDa
400359	C15orf53	chromosome 15 open reading frame 53
100130912	[No Symbol]	[No Name]
25901	CCDC28A	coiled-coil domain containing 28A
79037	PVRIG	poliovirus receptor related immunoglobulin domain containing
84647	PLA2G12B	phospholipase A2, group XIIB
150771	ITPRIPL1	inositol 1,4,5-trisphosphate receptor interacting protein-like 1
138649	ANKRD19P	ankyrin repeat domain 19, pseudogene
9472	AKAP6	A kinase (PRKA) anchor protein 6
100128465	IFIT6P	interferon-induced protein with tetratricopeptide repeats 6, pseudogene
1032	CDKN2D	cyclin-dependent kinase inhibitor 2D (p19, inhibits CDK4)
520	ATP5G2P1	ATP synthase, H+ transporting, mitochondrial Fo complex, subunit C2 (subunit 9) pseudogene 1
57125	PLXDC1	plexin domain containing 1
79314	OR4K6P	olfactory receptor, family 4, subfamily K, member 6 pseudogene
10794	ZNF460	zinc finger protein 460
539	ATP5O	ATP synthase, H+ transporting, mitochondrial F1 complex, O subunit
286101	ZNF252	zinc finger protein 252
4295	MLN	motilin
619208	C6orf225	chromosome 6 open reading frame 225
484	ATP1B3P1	ATPase, Na+/K+ transporting, beta 3 pseudogene

283431	GAS2L3	growth arrest-specific 2 like 3
3162	HMOX1	heme oxygenase (decycling) 1
56616	DIABLO	diablo, IAP-binding mitochondrial protein
50616	IL22	interleukin 22
10223	GPA33	glycoprotein A33 (transmembrane)
3687	ITGAX	integrin, alpha X (complement component 3 receptor 4 subunit)
57111	RAB25	RAB25, member RAS oncogene family
5557	PRIM1	primase, DNA, polypeptide 1 (49kDa)
1855	DVL1	dishevelled, dsh homolog 1 (Drosophila)
25915	NDUFAF3	NADH dehydrogenase (ubiquinone) 1 alpha subcomplex, assembly factor 3
286828	CSN1S2AP	casein alpha s2-like A, pseudogene
6647	SOD1	superoxide dismutase 1, soluble
28960	DCPS	decapping enzyme, scavenger
133619	PRRC1	proline-rich coiled-coil 1
51310	SLC22A17	solute carrier family 22, member 17
7023	TFAP4	transcription factor AP-4 (activating enhancer binding protein 4)
80095	ZNF606	zinc finger protein 606
6844	VAMP2	vesicle-associated membrane protein 2 (synaptobrevin 2)
57104	PNPLA2	patatin-like phospholipase domain containing 2
442210	LOC442210	tubulin beta chain-like
6596	HLTF	helicase-like transcription factor
51361	HOOK1	hook homolog 1 (Drosophila)
6424	SFRP4	secreted frizzled-related protein 4
56648	EIF5A2	eukaryotic translation initiation factor 5A2
440491	[No Symbol]	[No Name]
151313	FAHD2B	fumarylacetoacetate hydrolase domain containing 2B
392435	LOC392435	melanoma antigen family B, 3 pseudogene
9587	MAD2L1BP	MAD2L1 binding protein
100127967	LOC100127967	hypothetical LOC100127967
160857	CCDC122	coiled-coil domain containing 122
7597	ZBTB25	zinc finger and BTB domain containing 25
100132239	LOC100132239	calcium activated nucleotidase 1 pseudogene
401537	RPSAP49	ribosomal protein SA pseudogene 49
81565	NDEL1	nudE nuclear distribution gene E homolog (A. nidulans)-like 1
51430	C1orf9	chromosome 1 open reading frame 9

402220	YTHDF1P1	YTH domain family, member 1 pseudogene 1
5150	PDE7A	phosphodiesterase 7A
149076	ZNF362	zinc finger protein 362
79134	TMEM185B	transmembrane protein 185B
8969	HIST1H2AG	histone cluster 1, H2ag
84456	L3MBTL3	l(3)mbt-like 3 (Drosophila)
23552	CDK20	cyclin-dependent kinase 20
7745	ZNF192	zinc finger protein 192
116337	PANX3	pannexin 3
646324	LOC646324	hypothetical LOC646324
5591	PRKDC	protein kinase, DNA-activated, catalytic polypeptide
9020	MAP3K14	mitogen-activated protein kinase kinase kinase 14
219402	MTIF3	mitochondrial translational initiation factor 3
7485	WRB	tryptophan rich basic protein
343930	MSGN1	mesogenin 1
114836	SLAMF6	SLAM family member 6
9993	DGCR2	DiGeorge syndrome critical region gene 2
10992	SF3B2	splicing factor 3b, subunit 2, 145kDa
730217	[No Symbol]	[No Name]
516	ATP5G1	ATP synthase, H+ transporting, mitochondrial Fo complex, subunit C1 (subunit 9)
92960	PEX11G	peroxisomal biogenesis factor 11 gamma
7314	UBB	ubiquitin B
28989	METTL11A	methyltransferase like 11A
80700	UBXN6	UBX domain protein 6
196463	PLBD2	phospholipase B domain containing 2
146723	C17orf77	chromosome 17 open reading frame 77
51471	NAT8B	N-acetyltransferase 8B (GCN5-related, putative, gene/pseudogene)
4973	OLR1	oxidized low density lipoprotein (lectin-like) receptor 1
100128899	RPL7L1P9	ribosomal protein L7-like 1 pseudogene 9
10913	EDAR	ectodysplasin A receptor
80222	TARS2	threonyl-tRNA synthetase 2, mitochondrial (putative)
57594	HOMEZ	homeobox and leucine zipper encoding
55791	LRIF1	ligand dependent nuclear receptor interacting factor 1
440051	KRTAP5-11	keratin associated protein 5-11
54877	ZCCHC2	zinc finger, CCHC domain containing 2

124961	ZFP3	zinc finger protein 3 homolog (mouse)
51427	ZNF107	zinc finger protein 107
158219	TTC39B	tetratricopeptide repeat domain 39B
8813	DPM1	dolichyl-phosphate mannosyltransferase polypeptide 1, catalytic subunit
100128050	LOC100128050	WD repeat domain 77 pseudogene
64976	MRPL40	mitochondrial ribosomal protein L40
3049	HBQ1	hemoglobin, theta 1
5253	PHF2	PHD finger protein 2
221262	CCDC162P	coiled-coil domain containing 162, pseudogene
54570	UGT2B26P	UDP glucuronosyltransferase 2 family, polypeptide B26 pseudogene
7486	WRN	Werner syndrome, RecQ helicase-like
390933	LOC390933	protein associated with topoisomerase II homolog 1 (yeast) pseudogene
140576	S100A16	S100 calcium binding protein A16
2994	GYPB	glycophorin B (MNS blood group)
7174	TPP2	tripeptidyl peptidase II
403302	OR8A3P	olfactory receptor, family 8, subfamily A, member 3 pseudogene
81697	OR2B2	olfactory receptor, family 2, subfamily B, member 2
55128	TRIM68	tripartite motif containing 68
3573	IL6STP1	interleukin 6 signal transducer (gp130, oncostatin M receptor) pseudogene 1
220112	CTAGE3P	CTAGE family, member 3, pseudogene
387723	LOC387723	hypothetical LOC387723
11171	STRAP	serine/threonine kinase receptor associated protein
9935	MAFB	v-maf musculoaponeurotic fibrosarcoma oncogene homolog B (avian)
7874	USP7	ubiquitin specific peptidase 7 (herpes virus-associated)
3396	ICT1	immature colon carcinoma transcript 1
80223	RAB11FIP1	RAB11 family interacting protein 1 (class I)
284532	OR14A16	olfactory receptor, family 14, subfamily A, member 16
140738	TMEM37	transmembrane protein 37
79654	HECTD3	HECT domain containing 3
91947	ARRDC4	arrestin domain containing 4
6234	RPS28	ribosomal protein S28
2012	EMP1	epithelial membrane protein 1
3087	HHEX	hematopoietically expressed homeobox
79652	TMEM204	transmembrane protein 204
401981	FDPSP8	farnesyl diphosphate synthase pseudogene 8

5414	4-Sep	septin 4
127124	ATP6V1G3	ATPase, H+ transporting, lysosomal 13kDa, V1 subunit G3
10172	ZNF256	zinc finger protein 256
29949	IL19	interleukin 19
130733	TMEM178	transmembrane protein 178
100128135	GVINP2	GTPase, very large interferon inducible pseudogene 2
100132354	LOC100132354	hypothetical LOC100132354
90826	PRMT10	protein arginine methyltransferase 10 (putative)
727780	[No Symbol]	[No Name]
200035	NUDT17	nudix (nucleoside diphosphate linked moiety X)-type motif 17
100132060	[No Symbol]	[No Name]
124512	METTL23	methyltransferase like 23
55364	IMPACT	Impact homolog (mouse)
100130079	LOC100130079	ret finger protein-like 4A pseudogene
79723	SUV39H2	suppressor of variegation 3-9 homolog 2 (Drosophila)
23473	CAPN7	calpain 7
440026	TMEM41B	transmembrane protein 41B
100129060	LOC100129060	hypothetical LOC100129060
114907	FBXO32	F-box protein 32
100128104	[No Symbol]	[No Name]
54532	USP53	ubiquitin specific peptidase 53
2029	ENSA	endosulfine alpha
5734	PTGER4	prostaglandin E receptor 4 (subtype EP4)
54764	ZRANB1	zinc finger, RAN-binding domain containing 1
1896	EDA	ectodysplasin A
10169	SERF2	small EDRK-rich factor 2
158674	RPSAP3	ribosomal protein SA pseudogene 3
285550	FAM200B	family with sequence similarity 200, member B
346517	OR6V1	olfactory receptor, family 6, subfamily V, member 1
644159	LOC644159	solute carrier family 25 (mitochondrial carrier; citrate transporter), member 1 pseudogene
392132	OR2R1P	olfactory receptor, family 2, subfamily R, member 1, pseudogene
399	RHOH	ras homolog gene family, member H
51057	WDPCP	WD repeat containing planar cell polarity effector
6751	SSTR1	somatostatin receptor 1
10813	UTP14A	UTP14, U3 small nucleolar ribonucleoprotein, homolog A (yeast)

2530	FUT8	fucosyltransferase 8 (alpha (1,6) fucosyltransferase)
7168	TPM1	tropomyosin 1 (alpha)
286550	CYCSP46	cytochrome c, somatic pseudogene 46
81164	OR8L1P	olfactory receptor, family 8, subfamily L, member 1 pseudogene
100128709	[No Symbol]	[No Name]
7678	ZNF124	zinc finger protein 124
55000	TUG1	taurine upregulated 1 (non-protein coding)
23335	WDR7	WD repeat domain 7
388561	ZNF761	zinc finger protein 761
23218	NBEAL2	neurobeachin-like 2
51637	C14orf166	chromosome 14 open reading frame 166
130560	SPATA3	spermatogenesis associated 3
121504	HIST4H4	histone cluster 4, H4
23078	KIAA0564	KIAA0564
11108	PRDM4	PR domain containing 4
4111	MAGEA12	melanoma antigen family A, 12
116843	C6orf192	chromosome 6 open reading frame 192
143879	KBTBD3	kelch repeat and BTB (POZ) domain containing 3
5264	PHYH	phytanoyl-CoA 2-hydroxylase
79087	ALG12	asparagine-linked glycosylation 12, alpha-1,6-mannosyltransferase homolog (S. cerevisiae)
85453	TSPYL5	TSPY-like 5
8526	DGKE	diacylglycerol kinase, epsilon 64kDa
401337	FLJ45974	hypothetical LOC401337
343069	HNRNPCL1	heterogeneous nuclear ribonucleoprotein C-like 1
9218	VAPA	VAMP (vesicle-associated membrane protein)-associated protein A, 33kDa
401959	LOC401959	voltage-dependent anion channel 2 pseudogene
730091	LOC730091	hypothetical LOC730091
64777	RMND5B	required for meiotic nuclear division 5 homolog B (S. cerevisiae)
113402	SFT2D1	SFT2 domain containing 1
123103	KLHL33	kelch-like 33 (Drosophila)
84527	ZNF559	zinc finger protein 559
170959	ZNF431	zinc finger protein 431
100128172	LOC100128172	hypothetical protein LOC100128172
643637	GLYATL1P4	glycine-N-acyltransferase-like 1 pseudogene 4
407008	MIR223	microRNA 223

3996	LLGL1	lethal giant larvae homolog 1 (Drosophila)
5537	PPP6C	protein phosphatase 6, catalytic subunit
25775	C22orf24	chromosome 22 open reading frame 24
23324	MAN2B2	mannosidase, alpha, class 2B, member 2
1669	DEFA4	defensin, alpha 4, corticostatin
100129320	HMGB3P10	high mobility group box 3 pseudogene 10
6871	TADA2A	transcriptional adaptor 2A
7334	UBE2N	ubiquitin-conjugating enzyme E2N
3422	IDI1	isopentenyl-diphosphate delta isomerase 1
170958	ZNF525	zinc finger protein 525
553115	PEF1	penta-EF-hand domain containing 1
100129292	LOC100129292	hypothetical protein LOC100129292
139596	UPRT	uracil phosphoribosyltransferase (FUR1) homolog (S. cerevisiae)
390099	LOC390099	MAS-related GPR, member X3 pseudogene
1439	CSF2RB	colony stimulating factor 2 receptor, beta, low-affinity (granulocyte-macrophage)
140612	ZFP28	zinc finger protein 28 homolog (mouse)
1191	CLU	clusterin
83694	RPS6KL1	ribosomal protein S6 kinase-like 1
3070	HELLS	helicase, lymphoid-specific
55790	CSGALNACT1	chondroitin sulfate N-acetylgalactosaminyltransferase 1
153684	LOC153684	hypothetical LOC153684
5316	PKNOX1	PBX/knotted 1 homeobox 1
54458	PRR13	proline rich 13
51256	TBC1D7	TBC1 domain family, member 7
121274	ZNF641	zinc finger protein 641
83700	JAM3	junctional adhesion molecule 3
153527	ZMAT2	zinc finger, matrin-type 2
54529	ASNSD1	asparagine synthetase domain containing 1
8669	EIF3J	eukaryotic translation initiation factor 3, subunit J
81230	OR5AK1P	olfactory receptor, family 5, subfamily AK, member 1 pseudogene
6868	ADAM17	ADAM metallopeptidase domain 17
26148	C10orf12	chromosome 10 open reading frame 12
5318	PKP2	plakophilin 2
338094	FAM151A	family with sequence similarity 151, member A
647259	[No Symbol]	[No Name]

25981	DNAH1	dynein, axonemal, heavy chain 1
6617	SNAPC1	small nuclear RNA activating complex, polypeptide 1, 43kDa
100129175	LOC100129175	hypothetical protein LOC100129175
346085	GAPDHP72	glyceraldehyde-3-phosphate dehydrogenase pseudogene 72
8896	BUD31	BUD31 homolog (S. cerevisiae)
10174	SORBS3	sorbin and SH3 domain containing 3
152101	VENTXP4	VENT homeobox pseudogene 4
55520	ELAC1	elaC homolog 1 (E. coli)
92304	SCGB3A1	secretoglobin, family 3A, member 1
84961	FBXL20	F-box and leucine-rich repeat protein 20
340146	SLC35D3	solute carrier family 35, member D3
4772	NFATC1	nuclear factor of activated T-cells, cytoplasmic, calcineurin-dependent 1
6771	STARP1	steroidogenic acute regulatory protein pseudogene 1
6532	SLC6A4	solute carrier family 6 (neurotransmitter transporter, serotonin), member 4
9825	SPATA2	spermatogenesis associated 2
9849	ZNF518A	zinc finger protein 518A
2504	FTH1P12	ferritin, heavy polypeptide 1 pseudogene 12
1728	NQO1	NAD(P)H dehydrogenase, quinone 1
10847	SRCAP	Snf2-related CREBBP activator protein
118670	FAM24A	family with sequence similarity 24, member A
645218	[No Symbol]	[No Name]
727923	[No Symbol]	[No Name]
400064	RPS4XP1	ribosomal protein S4X pseudogene 1
90956	ADCK2	aarF domain containing kinase 2
152559	PAQR3	progestin and adipoQ receptor family member III
644022	RPL22P19	ribosomal protein L22 pseudogene 19
83480	PUS3	pseudouridylate synthase 3
211	ALAS1	aminolevulinate, delta-, synthase 1
51706	CYB5R1	cytochrome b5 reductase 1
80895	ILKAP	integrin-linked kinase-associated serine/threonine phosphatase
441108	C5orf56	chromosome 5 open reading frame 56
3312	HSPA8	heat shock 70kDa protein 8
80757	TMEM121	transmembrane protein 121
57542	KLHDC5	kelch domain containing 5
162967	ZNF320	zinc finger protein 320

347549	LOC347549	centromere protein V pseudogene
57232	ZNF630	zinc finger protein 630
8780	RIOK3	RIO kinase 3 (yeast)
55303	GIMAP4	GTPase, IMAP family member 4
81689	ISCA1	iron-sulfur cluster assembly 1 homolog (S. cerevisiae)
9465	AKAP7	A kinase (PRKA) anchor protein 7
55286	C4orf19	chromosome 4 open reading frame 19
221120	ALKBH3	alkB, alkylation repair homolog 3 (E. coli)
729665	C14orf38	chromosome 14 open reading frame 38
10294	DNAJA2	DnaJ (Hsp40) homolog, subfamily A, member 2
402716	RPL32P17	ribosomal protein L32 pseudogene 17
6515	SLC2A3	solute carrier family 2 (facilitated glucose transporter), member 3
23556	PIGN	phosphatidylinositol glycan anchor biosynthesis, class N
57194	ATP10A	ATPase, class V, type 10A
6192	RPS4Y1	ribosomal protein S4, Y-linked 1
51621	KLF13	Kruppel-like factor 13
51524	TMEM138	transmembrane protein 138
7627	ZNF75A	zinc finger protein 75a
85441	PRIC285	peroxisomal proliferator-activated receptor A interacting complex 285
54800	KLHL24	kelch-like 24 (Drosophila)
57653	BDAG1	LOC100499484-C9orf174 readthrough
6280	S100A9	S100 calcium binding protein A9
727941	LOC727941	similar to RIKEN cDNA 4930443G12
100128810	[No Symbol]	[No Name]
220213	OTUD1	OTU domain containing 1
84553	C6orf168	chromosome 6 open reading frame 168
6282	S100A11	S100 calcium binding protein A11
5885	RAD21	RAD21 homolog (S. pombe)
132	ADK	adenosine kinase
100128731	OST4	oligosaccharyltransferase 4 homolog (S. cerevisiae)
84124	ZNF394	zinc finger protein 394
114904	C1QTNF6	C1q and tumor necrosis factor related protein 6
442252	KRT18P22	keratin 18 pseudogene 22
388907	RPL5P34	ribosomal protein L5 pseudogene 34
8339	HIST1H2BG	histone cluster 1, H2bg

4338	MOCS2	molybdenum cofactor synthesis 2
55081	IFT57	intraflagellar transport 57 homolog (Chlamydomonas)
100130779	[No Symbol]	[No Name]
100130579	LOC100130579	hypothetical LOC100130579
136051	ZNF786	zinc finger protein 786
221718	C6orf218	chromosome 6 open reading frame 218
55223	TRIM62	tripartite motif containing 62
63874	ABHD4	abhydrolase domain containing 4
93654	ST7-AS2	ST7 antisense RNA 2 (non-protein coding)
79719	AAGAB	alpha- and gamma-adaptin binding protein
55776	C6orf64	chromosome 6 open reading frame 64
134505	LOC134505	eukaryotic translation initiation factor 3 subunit K pseudogene
56300	IL36G	interleukin 36, gamma
57120	GOPC	golgi-associated PDZ and coiled-coil motif containing
64283	RGNEF	190 kDa guanine nucleotide exchange factor
8736	MYOM1	myomesin 1, 185kDa
143098	MPP7	membrane protein, palmitoylated 7 (MAGUK p55 subfamily member 7)
28383	IGHV7-27	immunoglobulin heavy variable 7-27 (pseudogene)
9419	CRIPT	cysteine-rich PDZ-binding protein
56683	C21orf59	chromosome 21 open reading frame 59
5576	PRKAR2A	protein kinase, cAMP-dependent, regulatory, type II, alpha
341333	LOC341333	heterogeneous nuclear ribonucleoprotein A1 pseudogene
1446	CSN1S1	casein alpha s1
9194	SLC16A7	solute carrier family 16, member 7 (monocarboxylic acid transporter 2)
282808	RAB40AL	RAB40A, member RAS oncogene family-like
5430	POLR2A	polymerase (RNA) II (DNA directed) polypeptide A, 220kDa
10933	MORF4L1	mortality factor 4 like 1
7429	VIL1	villin 1
145567	TTC7B	tetratricopeptide repeat domain 7B
25851	TECPR1	tectonin beta-propeller repeat containing 1
65010	SLC26A6	solute carrier family 26, member 6
55843	ARHGAP15	Rho GTPase activating protein 15
100129026	[No Symbol]	[No Name]
3978	LIG1	ligase I, DNA, ATP-dependent
55088	C10orf118	chromosome 10 open reading frame 118

81887	LAS1L	LAS1-like (S. cerevisiae)
162137	MGC34800	hypothetical protein MGC34800
196264	MPZL3	myelin protein zero-like 3
1398	CRK	v-crk sarcoma virus CT10 oncogene homolog (avian)
729376	[No Symbol]	[No Name]
29117	BRD7	bromodomain containing 7
3937	LCP2	lymphocyte cytosolic protein 2 (SH2 domain containing leukocyte protein of 76kDa)
10207	INADL	InaD-like (Drosophila)
58533	SNX6	sorting nexin 6
146542	ZNF688	zinc finger protein 688
4598	MVK	mevalonate kinase
200186	CRTC2	CREB regulated transcription coactivator 2
100127897	[No Symbol]	[No Name]
5193	PEX12	peroxisomal biogenesis factor 12
130502	TTC32	tetratricopeptide repeat domain 32
85460	ZNF518B	zinc finger protein 518B
285605	DTWD2	DTW domain containing 2
253482	[No Symbol]	[No Name]
80230	RUFY1	RUN and FYVE domain containing 1
80264	ZNF430	zinc finger protein 430
374491	LOC374491	TPTE and PTEN homologous inositol lipid phosphatase pseudogene
6840	SVIL	supervillin
285025	CCDC141	coiled-coil domain containing 141
4900	NRGN	neurogranin (protein kinase C substrate, RC3)
10131	TRAP1	TNF receptor-associated protein 1
56987	BBX	bobby sox homolog (Drosophila)
4798	NFRKB	nuclear factor related to kappaB binding protein
10982	MAPRE2	microtubule-associated protein, RP/EB family, member 2
3146	HMGB1	high mobility group box 1
5682	PSMA1	proteasome (prosome, macropain) subunit, alpha type, 1
161145	TMEM229B	transmembrane protein 229B
9677	PPIP5K1	diphosphoinositol pentakisphosphate kinase 1
170690	ADAMTS16	ADAM metallopeptidase with thrombospondin type 1 motif, 16
57128	LYRM4	LYR motif containing 4
5010	CLDN11	claudin 11

7157	TP53	tumor protein p53
2794	GNL1	guanine nucleotide binding protein-like 1
23642	SNHG1	small nucleolar RNA host gene 1 (non-protein coding)
51056	LAP3	leucine aminopeptidase 3
158584	FAAH2	fatty acid amide hydrolase 2
8467	SMARCA5	SWI/SNF related, matrix associated, actin dependent regulator of chromatin, subfamily a, member 5
25948	KBTBD2	kelch repeat and BTB (POZ) domain containing 2
100130274	LOC100130274	coiled-coil domain containing 121-like
85313	PPIL4	peptidylprolyl isomerase (cyclophilin)-like 4
29103	DNAJC15	DnaJ (Hsp40) homolog, subfamily C, member 15
23232	TBC1D12	TBC1 domain family, member 12
90273	CEACAM21	carcinoembryonic antigen-related cell adhesion molecule 21
9045	RPL14	ribosomal protein L14
51227	PIGP	phosphatidylinositol glycan anchor biosynthesis, class P
132719	LOC132719	tubulin, beta 8 pseudogene
11147	HHLA3	HERV-H LTR-associating 3
261729	STEAP2	six transmembrane epithelial antigen of the prostate 2
10284	SAP18	Sin3A-associated protein, 18kDa
1997	ELF1	E74-like factor 1 (ets domain transcription factor)
219	ALDH1B1	aldehyde dehydrogenase 1 family, member B1
3781	KCNN2	potassium intermediate/small conductance calcium-activated channel, subfamily N, member 2
388818	KRTAP26-1	keratin associated protein 26-1
29798	C2orf27A	chromosome 2 open reading frame 27A
390431	OR4K2	olfactory receptor, family 4, subfamily K, member 2
439954	RPL7P37	ribosomal protein L7 pseudogene 37
5577	PRKAR2B	protein kinase, cAMP-dependent, regulatory, type II, beta
1723	DHODH	dihydroorotate dehydrogenase
54014	BRWD1	bromodomain and WD repeat domain containing 1
100128127	LOC100128127	hypothetical LOC100128127
29068	ZBTB44	zinc finger and BTB domain containing 44
54516	MTRF1L	mitochondrial translational release factor 1-like
391647	KRT18P25	keratin 18 pseudogene 25
79346	OR4C5	olfactory receptor, family 4, subfamily C, member 5
10128	LRPPRC	leucine-rich PPR-motif containing
85397	RGS8	regulator of G-protein signaling 8

654342	LOC654342	lymphocyte-specific protein 1 pseudogene
55038	CDCA4	cell division cycle associated 4
100128328	[No Symbol]	[No Name]
643613	[No Symbol]	[No Name]
8452	CUL3	cullin 3
5836	PYGL	phosphorylase, glycogen, liver
2802	GOLGA3	golgin A3
64101	LRRC4	leucine rich repeat containing 4
969	CD69	CD69 molecule
402415	XKRX	XK, Kell blood group complex subunit-related, X-linked
256006	ANKRD31	ankyrin repeat domain 31
6934	TCF7L2	transcription factor 7-like 2 (T-cell specific, HMG-box)
100131457	LOC100131457	solute carrier family 25 (mitochondrial carrier; citrate transporter), member 1 pseudogene
26984	SEC22A	SEC22 vesicle trafficking protein homolog A (S. cerevisiae)
10920	COPS8	COP9 constitutive photomorphogenic homolog subunit 8 (Arabidopsis)
283011	FLJ37201	tigger transposable element derived 2 pseudogene
282967	MAPK6PS6	mitogen-activated protein kinase 6 pseudogene 6
644941	[No Symbol]	[No Name]
7252	TSHB	thyroid stimulating hormone, beta
100129727	TMED10P2	transmembrane emp24-like trafficking protein 10 (yeast) pseudogene 2
6006	RHCE	Rh blood group, CcEe antigens
81027	TUBB1	tubulin, beta 1
389322	LOC389322	heterogeneous nuclear ribonucleoprotein K pseudogene
643641	ZNF862	zinc finger protein 862
100131977	LOC100131977	prickle homolog 1 (Drosophila) pseudogene
374500	THSD1P1	thrombospondin, type I, domain containing 1 pseudogene 1
100130396	ATP5LP4	ATP synthase, H+ transporting, mitochondrial Fo complex, subunit g, pseudogene 4
7846	TUBA1A	tubulin, alpha 1a
23338	PHF15	PHD finger protein 15
79786	KLHL36	kelch-like 36 (Drosophila)
6157	RPL27A	ribosomal protein L27a
79098	C1orf116	chromosome 1 open reading frame 116
55017	C14orf119	chromosome 14 open reading frame 119
118672	PSTK	phosphoseryl-tRNA kinase
7541	ZFP161	zinc finger protein 161 homolog (mouse)

1418	CRYGA	crystallin, gamma A
64327	LMBR1	limb region 1 homolog (mouse)
25992	SNED1	sushi, nidogen and EGF-like domains 1
2944	GSTM1	glutathione S-transferase mu 1
79893	GGNBP2	gametogenetin binding protein 2
4515	MTCP1	mature T-cell proliferation 1
100133321	KLF17P1	Kruppel-like factor 17 pseudogene 1
79363	RSG1	REM2 and RAB-like small GTPase 1
80201	HKDC1	hexokinase domain containing 1
7789	ZXDA	zinc finger, X-linked, duplicated A
131149	OTOL1	otolin 1
6884	TAF13	TAF13 RNA polymerase II, TATA box binding protein (TBP)-associated factor, 18kDa
57002	C7orf36	chromosome 7 open reading frame 36
5191	PEX7	peroxisomal biogenesis factor 7
84515	MCM8	minichromosome maintenance complex component 8
643836	ZFP62	zinc finger protein 62 homolog (mouse)
55170	PRMT6	protein arginine methyltransferase 6
57835	SLC4A5	solute carrier family 4, sodium bicarbonate cotransporter, member 5
56172	ANKH	ankylosis, progressive homolog (mouse)
51341	ZBTB7A	zinc finger and BTB domain containing 7A
9725	TMEM63A	transmembrane protein 63A
283130	SLC25A45	solute carrier family 25, member 45
23526	HMHA1	histocompatibility (minor) HA-1
10124	ARL4A	ADP-ribosylation factor-like 4A
55614	KIF16B	kinesin family member 16B
100093630	SNHG8	small nucleolar RNA host gene 8 (non-protein coding)
645668	TUFMP1	Tu translation elongation factor, mitochondrial pseudogene 1
51054	PLEKHA8P1	pleckstrin homology domain containing, family A member 8 pseudogene 1
389120	LOC389120	actin, beta pseudogene
1200	TPP1	tripeptidyl peptidase I
3500	IGHG1	immunoglobulin heavy constant gamma 1 (G1m marker)
7416	VDAC1	voltage-dependent anion channel 1
9804	TOMM20	translocase of outer mitochondrial membrane 20 homolog (yeast)
221468	TMEM217	transmembrane protein 217
55588	MED29	mediator complex subunit 29

54820	NDE1	nudE nuclear distribution gene E homolog 1 (A. nidulans)
26251	KCNG2	potassium voltage-gated channel, subfamily G, member 2
79831	JMJD5	jumonji domain containing 5
9	NAT1	N-acetyltransferase 1 (arylamine N-acetyltransferase)
3726	JUNB	jun B proto-oncogene
91975	ZNF300	zinc finger protein 300
6880	TAF9	TAF9 RNA polymerase II, TATA box binding protein (TBP)-associated factor, 32kDa
399512	SLC25A35	solute carrier family 25, member 35
8853	ASAP2	ArfGAP with SH3 domain, ankyrin repeat and PH domain 2
27036	SIGLEC7	sialic acid binding Ig-like lectin 7
25984	KRT23	keratin 23 (histone deacetylase inducible)
114798	SLITRK1	SLIT and NTRK-like family, member 1
3916	LAMP1	lysosomal-associated membrane protein 1
8649	LAMTOR3	late endosomal/lysosomal adaptor, MAPK and MTOR activator 3
6948	TCN2	transcobalamin II
55701	ARHGEF40	Rho guanine nucleotide exchange factor (GEF) 40
8863	PER3	period homolog 3 (Drosophila)
2180	ACSL1	acyl-CoA synthetase long-chain family member 1
143503	OR51E1	olfactory receptor, family 51, subfamily E, member 1
115416	C7orf30	chromosome 7 open reading frame 30
283948	NHLRC4	NHL repeat containing 4
5795	PTPRJ	protein tyrosine phosphatase, receptor type, J
6428	SRSF3	serine/arginine-rich splicing factor 3
442260	RPL23AP46	ribosomal protein L23a pseudogene 46
222894	FERD3L	Fer3-like (Drosophila)
100132857	LOC100132857	heat shock protein 90kDa alpha (cytosolic), class B member 1 pseudogene
6812	STXBP1	syntaxin binding protein 1
100129009	LOC100129009	WD repeats and SOF1 domain containing pseudogene
100131572	RPS27P9	ribosomal protein S27 pseudogene 9
100131593	LOC100131593	cytokine induced protein 29 kDa pseudogene
22859	LPHN1	latrophilin 1
6628	SNRPB	small nuclear ribonucleoprotein polypeptides B and B1
100129310	RPL31P14	ribosomal protein L31 pseudogene 14
166968	MIER3	mesoderm induction early response 1, family member 3
151556	GPR155	G protein-coupled receptor 155

255330	C3orf46	nucleoporin 210kDa pseudogene
10544	PROCR	protein C receptor, endothelial
55802	DCP1A	DCP1 decapping enzyme homolog A (S. cerevisiae)
23503	ZFYVE26	zinc finger, FYVE domain containing 26
195827	C9orf21	chromosome 9 open reading frame 21
5837	PYGM	phosphorylase, glycogen, muscle
142683	ITLN2	intelectin 2
55734	ZFP64	zinc finger protein 64 homolog (mouse)
4089	SMAD4	SMAD family member 4
100131311	[No Symbol]	[No Name]
10686	CLDN16	claudin 16
1649	DDIT3	DNA-damage-inducible transcript 3
54549	SDK2	sidekick homolog 2 (chicken)
285533	RNF175	ring finger protein 175
9021	SOCS3	suppressor of cytokine signaling 3
4599	MX1	myxovirus (influenza virus) resistance 1, interferon-inducible protein p78 (mouse)
729251	[No Symbol]	[No Name]
8582	OR8B1P	olfactory receptor, family 8, subfamily B, member 1 pseudogene
646672	RPL15P20	ribosomal protein L15 pseudogene 20
221833	SP8	Sp8 transcription factor
9683	N4BP1	NEDD4 binding protein 1
8971	H1FX	H1 histone family, member X
11264	PXMP4	peroxisomal membrane protein 4, 24kDa
441172	FLJ46906	hypothetical LOC441172
8718	TNFRSF25	tumor necrosis factor receptor superfamily, member 25
84843	MGC15705	hypothetical protein MGC15705
84064	HDHD2	haloacid dehalogenase-like hydrolase domain containing 2
6813	STXBP2	syntaxin binding protein 2
55454	CSGALNACT2	chondroitin sulfate N-acetylgalactosaminyltransferase 2
153241	CEP120	centrosomal protein 120kDa
389257	LRRC14B	leucine rich repeat containing 14B
10949	HNRNPA0	heterogeneous nuclear ribonucleoprotein A0
8778	SIGLEC5	sialic acid binding Ig-like lectin 5
30836	DNTTIP2	deoxynucleotidyltransferase, terminal, interacting protein 2
55365	TMEM176A	transmembrane protein 176A

139201	LOC139201	mitogen-activated protein kinase kinase 4 pseudogene
168741	PER4	period homolog 3 (Drosophila) pseudogene
342926	ZNF677	zinc finger protein 677
56675	NRIP3	nuclear receptor interacting protein 3
56474	CTPS2	CTP synthase II
442142	SOX30P1	SRY (sex determining region Y)-box 30 pseudogene 1
2854	GPR32	G protein-coupled receptor 32
1198	CLK3	CDC-like kinase 3
345462	ZNF879	zinc finger protein 879
199713	NLRP7	NLR family, pyrin domain containing 7
10717	AP4B1	adaptor-related protein complex 4, beta 1 subunit
7220	TRPC1	transient receptor potential cation channel, subfamily C, member 1
26168	SENP3	SUMO1/sentrin/SMT3 specific peptidase 3
90594	ZNF439	zinc finger protein 439
7132	TNFRSF1A	tumor necrosis factor receptor superfamily, member 1A
6936	C2orf3	chromosome 2 open reading frame 3
80342	TRAF3IP3	TRAF3 interacting protein 3
379025	FLJ31306	hypothetical LOC379025
18	ABAT	4-aminobutyrate aminotransferase
11060	WWP2	WW domain containing E3 ubiquitin protein ligase 2
54462	FAM190B	family with sequence similarity 190, member B
79818	ZNF552	zinc finger protein 552
2615	LRRC32	leucine rich repeat containing 32
100130847	[No Symbol]	[No Name]
7757	ZNF208	zinc finger protein 208
79016	DDA1	DET1 and DDB1 associated 1
100133044	BCRP8	breakpoint cluster region pseudogene 8
1327	COX4I1	cytochrome c oxidase subunit IV isoform 1
100127911	ST13P12	suppression of tumorigenicity 13 (colon carcinoma) (Hsp70 interacting protein) pseudogene 12
5813	PURA	purine-rich element binding protein A
647038	[No Symbol]	[No Name]
6433	SFSWAP	splicing factor, suppressor of white-apricot homolog (Drosophila)
54891	INO80D	INO80 complex subunit D
27253	PCDH17	protocadherin 17
220906	LOC220906	hypothetical LOC220906

8500	PPFIA1	protein tyrosine phosphatase, receptor type, f polypeptide (PTPRF), interacting protein (liprin), alpha 1
79809	TTC21B	tetratricopeptide repeat domain 21B
64921	CASD1	CAS1 domain containing 1
6208	RPS14	ribosomal protein S14
1870	E2F2	E2F transcription factor 2
9437	NCR1	natural cytotoxicity triggering receptor 1
3091	HIF1A	hypoxia inducible factor 1, alpha subunit (basic helix-loop-helix transcription factor)
55237	VRTN	vertebrae development homolog (pig)
11085	ADAM30	ADAM metallopeptidase domain 30
389634	LOC389634	hypothetical LOC389634
100133123	LOC100133123	similar to hCG1809003
541468	C1orf190	chromosome 1 open reading frame 190
22919	MAPRE1	microtubule-associated protein, RP/EB family, member 1
441058	MGC39584	hypothetical LOC441058
92346	C1orf105	chromosome 1 open reading frame 105
448834	KPRP	keratinocyte proline-rich protein
10370	CITED2	Cbp/p300-interacting transactivator, with Glu/Asp-rich carboxy-terminal domain, 2
79894	ZNF672	zinc finger protein 672
51531	C9orf156	chromosome 9 open reading frame 156
6142	RPL18A	ribosomal protein L18a
23613	ZMYND8	zinc finger, MYND-type containing 8
81115	OR7E106P	olfactory receptor, family 7, subfamily E, member 106 pseudogene
79574	EPS8L3	EPS8-like 3
359763	MRPS18BP2	mitochondrial ribosomal protein S18B pseudogene 2
55274	PHF10	PHD finger protein 10
646216	LOC646216	ribosomal protein S3a pseudogene
308	ANXA5	annexin A5
390082	OR52E5	olfactory receptor, family 52, subfamily E, member 5
10952	SEC61B	Sec61 beta subunit
8772	FADD	Fas (TNFRSF6)-associated via death domain
5476	CTSA	cathepsin A
26279	PLA2G2D	phospholipase A2, group IID
100132916	FAM159B	family with sequence similarity 159, member B
340527	NHSL2	NHS-like 2
114826	SMYD4	SET and MYND domain containing 4

158787	RIBC1	RIB43A domain with coiled-coils 1
389435	RPL27AP6	ribosomal protein L27a pseudogene 6
50802	IGK@	immunoglobulin kappa locus
54708	5-Mar	membrane-associated ring finger (C3HC4) 5
646719	LOC646719	hypothetical LOC646719
145482	PTGR2	prostaglandin reductase 2
9263	STK17A	serine/threonine kinase 17a
284467	FAM19A3	family with sequence similarity 19 (chemokine (C-C motif)-like), member A3
7364	UGT2B7	UDP glucuronosyltransferase 2 family, polypeptide B7
94241	TP53INP1	tumor protein p53 inducible nuclear protein 1
728509	RPS19P7	ribosomal protein S19 pseudogene 7
23271	CAMSAP1L1	calmodulin regulated spectrin-associated protein 1-like 1
650157	LOC650157	peptidyl-prolyl cis-trans isomerase A-like
51290	ERGIC2	ERGIC and golgi 2
103911	RPS27AP1	ribosomal protein S27a pseudogene 1
84287	ZDHHC16	zinc finger, DHHC-type containing 16
100127886	LOC100127886	hypothetical LOC100127886
23612	PHLDA3	pleckstrin homology-like domain, family A, member 3
2289	FKBP5	FK506 binding protein 5
100128327	BET3L	BET3 like (S. cerevisiae)
84830	C6orf105	chromosome 6 open reading frame 105
79324	OR51G1	olfactory receptor, family 51, subfamily G, member 1
100131789	VN1R46P	vomeronasal 1 receptor 46 pseudogene
10569	SLU7	SLU7 splicing factor homolog (S. cerevisiae)
119687	OR51A7	olfactory receptor, family 51, subfamily A, member 7
645397	LOC645397	methylenetetrahydrofolate dehydrogenase (NADP+ dependent) 1-like pseudogene
497049	FLJ25758	hypothetical locus FLJ25758
145853	C15orf61	chromosome 15 open reading frame 61
391106	VDAC1P9	voltage-dependent anion channel 1 pseudogene 9
1135	CHRNA2	cholinergic receptor, nicotinic, alpha 2 (neuronal)
391239	LOC391239	V-set domain containing T cell activation inhibitor 1 pseudogene
7634	ZNF80	zinc finger protein 80
85440	DOCK7	dedicator of cytokinesis 7
53371	NUP54	nucleoporin 54kDa
4809	NHP2L1	NHP2 non-histone chromosome protein 2-like 1 (S. cerevisiae)

391211	OR2G6	olfactory receptor, family 2, subfamily G, member 6
100129046	LOC100129046	hypothetical LOC100129046
3281	HSBP1	heat shock factor binding protein 1
10245	TIMM17B	translocase of inner mitochondrial membrane 17 homolog B (yeast)
26015	RPAP1	RNA polymerase II associated protein 1
146540	ZNF785	zinc finger protein 785
51659	GINS2	GINS complex subunit 2 (Psf2 homolog)
51696	HECA	headcase homolog (Drosophila)
57551	TAOK1	TAO kinase 1
10380	BPNT1	3'(2'), 5'-bisphosphate nucleotidase 1
3308	HSPA4	heat shock 70kDa protein 4
351	APP	amyloid beta (A4) precursor protein
22871	NLGN1	neuroligin 1
128977	C22orf39	chromosome 22 open reading frame 39
203259	C9orf25	chromosome 9 open reading frame 25
55775	TDP1	tyrosyl-DNA phosphodiesterase 1
2895	GRID2	glutamate receptor, ionotropic, delta 2
84630	TTBK1	tau tubulin kinase 1
83464	APH1B	anterior pharynx defective 1 homolog B (C. elegans)
6727	SRP14	signal recognition particle 14kDa (homologous Alu RNA binding protein)
100130321	LOC100130321	DNA fragmentation factor, 45kDa, alpha polypeptide pseudogene
729397	[No Symbol]	[No Name]
292	SLC25A5	solute carrier family 25 (mitochondrial carrier; adenine nucleotide translocator), member 5
79722	ANKRD55	ankyrin repeat domain 55
643605	LOC643605	CTD (carboxy-terminal domain, RNA polymerase II, polypeptide A) small phosphatase like 2 pseudogene
3437	IFIT3	interferon-induced protein with tetratricopeptide repeats 3
9147	NEMF	nuclear export mediator factor
4318	MMP9	matrix metallopeptidase 9 (gelatinase B, 92kDa gelatinase, 92kDa type IV collagenase)
9365	KL	klotho
51209	RAB9B	RAB9B, member RAS oncogene family
10797	MTHFD2	methylenetetrahydrofolate dehydrogenase (NADP+ dependent) 2, methenyltetrahydrofolate cyclohydrolase
55628	ZNF407	zinc finger protein 407
23398	PPWD1	peptidylprolyl isomerase domain and WD repeat containing 1
646913	SEPT7P3	septin 7 pseudogene 3
91056	DKFZp761E198	DKFZp761E198 protein

11282	MGAT4B	mannosyl (alpha-1,3-)-glycoprotein beta-1,4-N-acetylglucosaminyltransferase, isozyme B
55613	MTMR8	myotubularin related protein 8
2850	GPR27	G protein-coupled receptor 27
4691	NCL	nucleolin
359764	MRPS18CP3	mitochondrial ribosomal protein S18C pseudogene 3
847	CAT	catalase
100129761	LOC100129761	adenosylmethionine decarboxylase 1 pseudogene
10120	ACTR1B	ARP1 actin-related protein 1 homolog B, centractin beta (yeast)
9597	SMAD5-AS1	SMAD5 antisense RNA 1 (non-protein coding)
51027	BOLA1	bolA homolog 1 (E. coli)
337972	KRTAP19-5	keratin associated protein 19-5
5054	SERPINE1	serpin peptidase inhibitor, clade E (nexin, plasminogen activator inhibitor type 1), member 1
283600	SLC25A47	solute carrier family 25, member 47
79441	HAUS3	HAUS augmin-like complex, subunit 3
916	CD3E	CD3e molecule, epsilon (CD3-TCR complex)
440104	LOC440104	1110012D08Rik pseudogene
253959	RALGAPA1	Ral GTPase activating protein, alpha subunit 1 (catalytic)
393046	OR2A5	olfactory receptor, family 2, subfamily A, member 5
644186	C22orf41	chromosome 22 open reading frame 41
80067	DCAF17	DDB1 and CUL4 associated factor 17
7786	MAP3K12	mitogen-activated protein kinase kinase kinase 12
747	DAGLA	diacylglycerol lipase, alpha
3988	LIPA	lipase A, lysosomal acid, cholesterol esterase
2592	GALT	galactose-1-phosphate uridylyltransferase
56884	FSTL5	follistatin-like 5
100128310	LOC100128310	hypothetical LOC100128310
391205	[No Symbol]	[No Name]
55998	NXF5	nuclear RNA export factor 5
5834	PYGB	phosphorylase, glycogen; brain
4352	MPL	myeloproliferative leukemia virus oncogene
654480	LOC654480	ATP synthase, H+ transporting, mitochondrial F0 complex, subunit C2 (subunit 9) pseudogene
27314	RAB30	RAB30, member RAS oncogene family
100132214	[No Symbol]	[No Name]
6018	RLF	rearranged L-myc fusion
59343	SENP2	SUMO1/sentrin/SMT3 specific peptidase 2

80833	APOL3	apolipoprotein L, 3
170514	FTLP1	ferritin, light polypeptide pseudogene 1
3632	INPP5A	inositol polyphosphate-5-phosphatase, 40kDa
10227	MFSD10	major facilitator superfamily domain containing 10
440695	ETV3L	ets variant 3-like
65944	OR2B8P	olfactory receptor, family 2, subfamily B, member 8 pseudogene
359803	MRPS17P1	mitochondrial ribosomal protein S17 pseudogene 1
643353	LOC643353	C9orf140 pseudogene
100131213	NCRNA00245	non-protein coding RNA 245
441592	LOC441592	mas-related G-protein coupled receptor member X1-like
8490	RGS5	regulator of G-protein signaling 5
8737	RIPK1	receptor (TNFRSF)-interacting serine-threonine kinase 1
84699	CREB3L3	cAMP responsive element binding protein 3-like 3
222658	KCTD20	potassium channel tetramerisation domain containing 20
123862	LOC123862	interferon induced transmembrane protein pseudogene
23177	CEP68	centrosomal protein 68kDa
407044	MIR7-2	microRNA 7-2
100129476	LOC100129476	hypothetical protein LOC100129476
400223	[No Symbol]	[No Name]
148709	LOC148709	actin pseudogene
10085	EDIL3	EGF-like repeats and discoidin I-like domains 3
347404	LANCL3	LanC lantibiotic synthetase component C-like 3 (bacterial)
648927	LOC648927	histone acetyltransferase MYST2-like
254268	AKNAD1	AKNA domain containing 1
10347	ABCA7	ATP-binding cassette, sub-family A (ABC1), member 7
60558	GUF1	GUF1 GTPase homolog (S. cerevisiae)
11149	BVES	blood vessel epicardial substance
845	CASQ2	calsequestrin 2 (cardiac muscle)
92797	HELB	helicase (DNA) B
116534	MRGPRE	MAS-related GPR, member E
347734	SLC35B2	solute carrier family 35, member B2
717	C2	complement component 2
122942	FRDAP	Friedreich ataxia pseudogene
56911	C21orf7	chromosome 21 open reading frame 7
7581	ZNF33A	zinc finger protein 33A

672	BRCA1	breast cancer 1, early onset
5166	PDK4	pyruvate dehydrogenase kinase, isozyme 4
54981	C9orf95	chromosome 9 open reading frame 95
100131853	[No Symbol]	[No Name]
1838	DTNB	dystrobrevin, beta
727999	[No Symbol]	[No Name]
4694	NDUFA1	NADH dehydrogenase (ubiquinone) 1 alpha subcomplex, 1, 7.5kDa
359821	MRPL42P5	mitochondrial ribosomal protein L42 pseudogene 5
65117	RSRC2	arginine/serine-rich coiled-coil 2
100129588	[No Symbol]	[No Name]
55846	ITFG2	integrin alpha FG-GAP repeat containing 2
54921	CHTF8	CTF8, chromosome transmission fidelity factor 8 homolog (S. cerevisiae)
1606	DGKA	diacylglycerol kinase, alpha 80kDa
57062	DDX24	DEAD (Asp-Glu-Ala-Asp) box polypeptide 24
27086	FOXP1	forkhead box P1
25909	AHCTF1	AT hook containing transcription factor 1
100130298	LOC100130298	hCG1816373-like
9924	PAN2	PAN2 poly(A) specific ribonuclease subunit homolog (S. cerevisiae)
4296	MAP3K11	mitogen-activated protein kinase kinase kinase 11
944	TNFSF8	tumor necrosis factor (ligand) superfamily, member 8
257103	NCRNA00205	non-protein coding RNA 205
114793	FMNL2	formin-like 2
166379	BBS12	Bardet-Biedl syndrome 12
285154	CYP1B1-AS1	CYP1B1 antisense RNA 1 (non-protein coding)
7326	UBE2G1	ubiquitin-conjugating enzyme E2G 1
81930	KIF18A	kinesin family member 18A
79970	ZNF767	zinc finger family member 767
8411	EEA1	early endosome antigen 1
201232	SLC16A13	solute carrier family 16, member 13 (monocarboxylic acid transporter 13)
100126518	TRNAE-CUC	transfer RNA glutamic acid (anticodon CUC)
114899	C1QTNF3	C1q and tumor necrosis factor related protein 3
92285	ZNF585B	zinc finger protein 585B
337882	KRTAP19-1	keratin associated protein 19-1
55603	FAM46A	family with sequence similarity 46, member A
100129572	LOC100129572	hypothetical protein LOC100129572

441862	LOC441862	DUT pseudogene
100131484	[No Symbol]	[No Name]
644517	LOC644517	family with sequence similarity 86, member A pseudogene
29766	TMOD3	tropomodulin 3 (ubiquitous)
79741	C10orf68	chromosome 10 open reading frame 68
64225	ATL2	atlastin GTPase 2
7920	ABHD16A	abhydrolase domain containing 16A
402135	OR5K2	olfactory receptor, family 5, subfamily K, member 2
83896	KRTAP3-1	keratin associated protein 3-1
84660	CCDC62	coiled-coil domain containing 62
79810	PTCD2	pentatricopeptide repeat domain 2
63027	SLC22A23	solute carrier family 22, member 23
83895	KRTAP1-5	keratin associated protein 1-5
84752	B3GNT9	UDP-GlcNAc:betaGal beta-1,3-N-acetylglucosaminyltransferase 9
100132735	LOC100132735	hypothetical protein LOC100132735
56547	MMP26	matrix metallopeptidase 26
6881	TAF10	TAF10 RNA polymerase II, TATA box binding protein (TBP)-associated factor, 30kDa
9128	PRPF4	PRP4 pre-mRNA processing factor 4 homolog (yeast)
7277	TUBA4A	tubulin, alpha 4a
440077	ZNF705A	zinc finger protein 705A
348825	TPRXL	tetra-peptide repeat homeobox-like
4193	MDM2	Mdm2 p53 binding protein homolog (mouse)
54815	GATAD2A	GATA zinc finger domain containing 2A
27430	MAT2B	methionine adenosyltransferase II, beta
400836	LOC400836	SDA1 domain containing 1 pseudogene
6474	SHOX2	short stature homeobox 2
353274	ZNF445	zinc finger protein 445
128710	C20orf94	chromosome 20 open reading frame 94
6653	SORL1	sortilin-related receptor, L(DLR class) A repeats containing
5283	PIGH	phosphatidylinositol glycan anchor biosynthesis, class H
390054	OR52A5	olfactory receptor, family 52, subfamily A, member 5
9890	LPPR4	lipid phosphate phosphatase-related protein type 4
100128587	[No Symbol]	[No Name]
123688	AGPHD1	aminoglycoside phosphotransferase domain containing 1
374618	TEX9	testis expressed 9

124801	LSM12	LSM12 homolog (S. cerevisiae)
400798	C1orf220	chromosome 1 open reading frame 220
7454	WAS	Wiskott-Aldrich syndrome (eczema-thrombocytopenia)
342776	MRPS17P7	mitochondrial ribosomal protein S17 pseudogene 7
120775	OR2D3	olfactory receptor, family 2, subfamily D, member 3
8848	TSC22D1	TSC22 domain family, member 1
11026	LILRA3	leukocyte immunoglobulin-like receptor, subfamily A (without TM domain), member 3
64840	PORCN	porcupine homolog (Drosophila)
5046	PCSK6	proprotein convertase subtilisin/kexin type 6
51316	PLAC8	placenta-specific 8
11197	WIF1	WNT inhibitory factor 1
100131641	LOC100131641	RNA binding motif protein 22 pseudogene
55821	ALLC	allantoicase
81470	OR2G2	olfactory receptor, family 2, subfamily G, member 2
730105	[No Symbol]	[No Name]
4477	MSMB	microseminoprotein, beta-
648822	HMGN2P18	high mobility group nucleosomal binding domain 2 pseudogene 18
55013	CCDC109B	coiled-coil domain containing 109B
79145	CHCHD7	coiled-coil-helix-coiled-coil-helix domain containing 7
79862	ZNF669	zinc finger protein 669
170691	ADAMTS17	ADAM metallopeptidase with thrombospondin type 1 motif, 17
55911	APOBR	apolipoprotein B receptor
100130535	LOC100130535	vimentin pseudogene
151888	BTLA	B and T lymphocyte associated
11135	CDC42EP1	CDC42 effector protein (Rho GTPase binding) 1
83416	FCRL5	Fc receptor-like 5
148213	ZNF681	zinc finger protein 681
6869	TACR1	tachykinin receptor 1
100130158	LOC100130158	chromosome 16 open reading frame 88 pseudogene
23536	ADAT1	adenosine deaminase, tRNA-specific 1
22938	SNW1	SNW domain containing 1
100132636	[No Symbol]	[No Name]
56970	ATXN7L3	ataxin 7-like 3
6671	SP4	Sp4 transcription factor
285116	AHCTF1P1	AT hook containing transcription factor 1 pseudogene 1

4161	MC5R	melanocortin 5 receptor
729291	LOC729291	hypothetical protein LOC729291
283416	C12orf61	chromosome 12 open reading frame 61
23518	R3HDM1	R3H domain containing 1
7473	WNT3	wingless-type MMTV integration site family, member 3
91151	TIGD7	tigger transposable element derived 7
114195	SIGLEC22P	sialic acid binding Ig-like lectin 22, pseudogene
84858	ZNF503	zinc finger protein 503
142680	SLC34A3	solute carrier family 34 (sodium phosphate), member 3
119391	GSTO2	glutathione S-transferase omega 2
100131796	LOC100131796	LP2570
51631	LUC7L2	LUC7-like 2 (S. cerevisiae)
84926	SPRYD3	SPRY domain containing 3
178	AGL	amylo-alpha-1, 6-glucosidase, 4-alpha-glucanotransferase
100129202	LOC100129202	arginine/serine-rich coiled-coil 1 pseudogene
100129119	LOC100129119	hypothetical LOC100129119
100130798	LOC100130798	hypothetical LOC100130798
401074	LOC401074	hypothetical LOC401074
23233	EXOC6B	exocyst complex component 6B
255919	TMEM188	transmembrane protein 188
100129808	RPL24P6	ribosomal protein L24 pseudogene 6
140597	TCEAL2	transcription elongation factor A (SII)-like 2
114659	LRRC37B	leucine rich repeat containing 37B
29887	SNX10	sorting nexin 10
7580	ZNF32	zinc finger protein 32
441878	RPS4XP4	ribosomal protein S4X pseudogene 4
4223	MEOX2	mesenchyme homeobox 2
11138	TBC1D8	TBC1 domain family, member 8 (with GRAM domain)
285386	TPRG1	tumor protein p63 regulated 1
4715	NDUFB9	NADH dehydrogenase (ubiquinone) 1 beta subcomplex, 9, 22kDa
283683	LOC283683	hypothetical LOC283683
5544	PRB3	proline-rich protein BstNI subfamily 3
100130049	LOC100130049	ribosomal L1 domain-containing protein 1-like
10438	C1D	C1D nuclear receptor corepressor
116832	RPL39L	ribosomal protein L39-like

10242	KCNMB2	potassium large conductance calcium-activated channel, subfamily M, beta member 2
6188	RPS3	ribosomal protein S3
391739	CCT6P2	chaperonin containing TCP1, subunit 6 (zeta) pseudogene 2
81343	OR10V2P	olfactory receptor, family 10, subfamily V, member 2 pseudogene
100126476	TRNAL-CAA	transfer RNA leucine (anticodon CAA)
6389	SDHA	succinate dehydrogenase complex, subunit A, flavoprotein (Fp)
117247	SLC16A10	solute carrier family 16, member 10 (aromatic amino acid transporter)
2444	FRK	fyn-related kinase
8314	BAP1	BRCA1 associated protein-1 (ubiquitin carboxy-terminal hydrolase)
26524	LATS2	LATS, large tumor suppressor, homolog 2 (Drosophila)
152789	JAKMIP1	janus kinase and microtubule interacting protein 1
19	ABCA1	ATP-binding cassette, sub-family A (ABC1), member 1
1050	CEBPA	CCAAT/enhancer binding protein (C/EBP), alpha
441548	LOC441548	BTB (POZ) domain containing 7 pseudogene
650560	DDX3YP3	DEAD (Asp-Glu-Ala-Asp) box polypeptide 3, Y-linked pseudogene 3
8061	FOSL1	FOS-like antigen 1
391	RHOG	ras homolog gene family, member G (rho G)
134957	STXBP5	syntaxin binding protein 5 (tomosyn)
55234	SMU1	smu-1 suppressor of mec-8 and unc-52 homolog (C. elegans)
1043	CD52	CD52 molecule
100129750	[No Symbol]	[No Name]
729633	MRS2P2	MRS2 magnesium homeostasis factor homolog (S. cerevisiae) pseudogene 2
7056	THBD	thrombomodulin
91754	NEK9	NIMA (never in mitosis gene a)- related kinase 9
55196	C12orf35	chromosome 12 open reading frame 35
3397	ID1	inhibitor of DNA binding 1, dominant negative helix-loop-helix protein
401675	OR56B3P	olfactory receptor, family 56, subfamily B, member 3 pseudogene
84216	TMEM117	transmembrane protein 117
387707	CC2D2B	coiled-coil and C2 domain containing 2B
3964	LGALS8	lectin, galactoside-binding, soluble, 8
54809	SAMD9	sterile alpha motif domain containing 9
9724	UTP14C	UTP14, U3 small nucleolar ribonucleoprotein, homolog C (yeast)
7014	TERF2	telomeric repeat binding factor 2
57489	ODF2L	outer dense fiber of sperm tails 2-like
340286	FAM183B	acyloxyacyl hydrolase (neutrophil)

6477	SIAH1	seven in absentia homolog 1 (Drosophila)
6616	SNAP25	synaptosomal-associated protein, 25kDa
100129442	[No Symbol]	[No Name]
1656	DDX6	DEAD (Asp-Glu-Ala-Asp) box polypeptide 6
7594	ZNF43	zinc finger protein 43
442221	LOC442221	regulator of chromosome condensation 2 pseudogene
112476	PRRT2	proline-rich transmembrane protein 2
327659	HDHD1P2	haloacid dehalogenase-like hydrolase domain containing 1 pseudogene 2
727916	LOC727916	hypothetical protein LOC727916
83866	TTTY11	testis-specific transcript, Y-linked 11 (non-protein coding)
8208	CHAF1B	chromatin assembly factor 1, subunit B (p60)
9882	TBC1D4	TBC1 domain family, member 4
29063	ZCCHC4	zinc finger, CCHC domain containing 4
340665	CYP26C1	cytochrome P450, family 26, subfamily C, polypeptide 1
643596	RNF224	ring finger protein 224
100129464	CXorf28	chromosome X open reading frame 28
8580	OR8C1P	olfactory receptor, family 8, subfamily C, member 1 pseudogene
50943	FOXP3	forkhead box P3
642590	LOC642590	spermine synthase pseudogene
6453	ITSN1	intersectin 1 (SH3 domain protein)
594	BCKDHB	branched chain keto acid dehydrogenase E1, beta polypeptide
402214	RPL13AP13	ribosomal protein L13a pseudogene 13
7690	ZNF131	zinc finger protein 131
729259	RPL17P43	ribosomal protein L17 pseudogene 43
54472	TOLLIP	toll interacting protein
389768	LOC389768	potassium channel tetramerisation domain containing 1 pseudogene
3012	HIST1H2AE	histone cluster 1, H2ae
391189	OR11L1	olfactory receptor, family 11, subfamily L, member 1
100132835	[No Symbol]	[No Name]
9790	BMS1	BMS1 homolog, ribosome assembly protein (yeast)
928	CD9	CD9 molecule
400165	C13orf35	chromosome 13 open reading frame 35
284086	NEK8	NIMA (never in mitosis gene a)- related kinase 8
6625	SNRNP70	small nuclear ribonucleoprotein 70kDa (U1)
100131731	[No Symbol]	[No Name]

7743	ZNF189	zinc finger protein 189
2560	GABRB1	gamma-aminobutyric acid (GABA) A receptor, beta 1
339456	TMEM52	transmembrane protein 52
100130921	LOC100130921	hypothetical protein LOC100130921
5431	POLR2B	polymerase (RNA) II (DNA directed) polypeptide B, 140kDa
29930	PCDHB1	protocadherin beta 1
147711	ZNF285B	zinc finger protein 285B, pseudogene
386675	KRTAP10-7	keratin associated protein 10-7
761	CA3	carbonic anhydrase III, muscle specific
3423	IDS	iduronate 2-sulfatase
1054	CEBPG	CCAAT/enhancer binding protein (C/EBP), gamma
337879	KRTAP8-1	keratin associated protein 8-1
3394	IRF8	interferon regulatory factor 8
81224	OR5AN2P	olfactory receptor, family 5, subfamily AN, member 2 pseudogene
23558	WBP2	WW domain binding protein 2
161394	SAMD15	sterile alpha motif domain containing 15
27125	AFF4	AF4/FMR2 family, member 4
9830	TRIM14	tripartite motif containing 14
79089	TMUB2	transmembrane and ubiquitin-like domain containing 2
100132652	[No Symbol]	[No Name]
644790	RPS15AP11	ribosomal protein S15a pseudogene 11
27	ABL2	v-abl Abelson murine leukemia viral oncogene homolog 2
84913	ATOH8	atonal homolog 8 (Drosophila)
5540	PPYR1	pancreatic polypeptide receptor 1
54682	MANSC1	MANSC domain containing 1
574447	MIR146B	microRNA 146b
117584	RFFL	ring finger and FYVE-like domain containing 1
643167	LOC643167	RNA binding motif protein 39 pseudogene
400657	LOC400657	hypothetical LOC400657
643866	CBLN3	cerebellin 3 precursor
644335	LOC644335	Smg-5 homolog, nonsense mediated mRNA decay factor (C. elegans) pseudogene
92370	ACPL2	acid phosphatase-like 2
245937	DEFB124	defensin, beta 124
80022	MYO15B	myosin XVB pseudogene
646012	LOC646012	aurora kinase A interacting protein 1 pseudogene

2231	FDX1P1	ferredoxin 1 pseudogene 1
23436	CELA3B	chymotrypsin-like elastase family, member 3B
7976	FZD3	frizzled family receptor 3
100132765	[No Symbol]	[No Name]
137	ADORA2BP1	adenosine A2b receptor pseudogene 1
594841	WDR45P	WDR45 pseudogene
9704	DHX34	DEAH (Asp-Glu-Ala-His) box polypeptide 34
441617	LOC441617	hypothetical LOC441617
83932	C1orf124	chromosome 1 open reading frame 124
54487	DGCR8	DiGeorge syndrome critical region gene 8
55246	CCDC25	coiled-coil domain containing 25
64084	CLSTN2	calsyntenin 2
11167	FSTL1	follistatin-like 1
81282	OR51G2	olfactory receptor, family 51, subfamily G, member 2
554206	LOC554206	hypothetical LOC554206
391359	LOC391359	epithelial membrane protein 2 pseudogene
196074	METTL15	methyltransferase like 15
387869	LOC387869	microtubule-associated proteins 1A/1B light chain 3B-like
10681	GNB5	guanine nucleotide binding protein (G protein), beta 5
11009	IL24	interleukin 24
27145	FILIP1	filamin A interacting protein 1
51110	LACTB2	lactamase, beta 2
100130729	[No Symbol]	[No Name]
57101	ANO2	anoctamin 2
27107	ZBTB11	zinc finger and BTB domain containing 11
81140	OR9R1P	olfactory receptor, family 9, subfamily R, member 1 pseudogene
10314	LANCL1	LanC lantibiotic synthetase component C-like 1 (bacterial)
151903	CCDC12	coiled-coil domain containing 12
54098	C1QBPP	complement component 1, q subcomponent binding protein, pseudogene
100127974	LOC100127974	hypothetical LOC100127974
24148	PRPF6	PRP6 pre-mRNA processing factor 6 homolog (S. cerevisiae)
29101	SSU72	SSU72 RNA polymerase II CTD phosphatase homolog (S. cerevisiae)
2971	GTF3A	general transcription factor IIIA
342531	ALOX15P1	arachidonate 15-lipoxygenase pseudogene 1
9706	ULK2	unc-51-like kinase 2 (C. elegans)

100132006	LOC100132006	hypothetical protein LOC100132006
100130763	[No Symbol]	[No Name]
3925	STMN1	stathmin 1
84992	PIGY	phosphatidylinositol glycan anchor biosynthesis, class Y
390329	OR6U2P	olfactory receptor, family 6, subfamily U, member 2 pseudogene
100131885	[No Symbol]	[No Name]
4018	LPA	lipoprotein, Lp(a)
266724	HSPA9P1	heat shock 70kDa protein 9 pseudogene 1
9388	LIPG	lipase, endothelial
7700	ZNF141	zinc finger protein 141
22969	RPL41P3	ribosomal protein L41 pseudogene 3
85451	UNK	unkempt homolog (Drosophila)
729708	TPI1P1	triosephosphate isomerase 1 pseudogene 1
9863	MAGI2	membrane associated guanylate kinase, WW and PDZ domain containing 2
9180	OSMR	oncostatin M receptor
4210	MEFV	Mediterranean fever
151477	C2orf52	chromosome 2 open reading frame 52
1039	CDR2	cerebellar degeneration-related protein 2, 62kDa
26823	RNU12-2P	RNA, U12 small nuclear 2, pseudogene
401647	GOLGA7B	golgin A7 family, member B
65981	CAPRIN2	caprin family member 2
384	ARG2	arginase, type II
84861	KLHL22	kelch-like 22 (Drosophila)
8512	MBL1P	mannose-binding lectin (protein A) 1, pseudogene
2972	BRF1	BRF1 homolog, subunit of RNA polymerase III transcription initiation factor IIIB (S. cerevisiae)
129684	CNTNAP5	contactin associated protein-like 5
256227	STEAP1B	STEAP family member 1B
9667	SAFB2	scaffold attachment factor B2
2196	FAT2	FAT tumor suppressor homolog 2 (Drosophila)
728567	RPL23AP48	ribosomal protein L23a pseudogene 48
92736	OTOP2	otopetrin 2
728097	FAM8A2P	family with sequence similarity 8, member A1 pseudogene
4817	NIT1	nitrilase 1
84440	RAB11FIP4	RAB11 family interacting protein 4 (class II)
100129896	[No Symbol]	[No Name]

400506	C16orf88	chromosome 16 open reading frame 88
5525	PPP2R5A	protein phosphatase 2, regulatory subunit B', alpha
729926	[No Symbol]	[No Name]
216	ALDH1A1	aldehyde dehydrogenase 1 family, member A1
26002	MOXD1	monooxygenase, DBH-like 1
200504	GKN2	gastrokine 2
123720	WHAMM	WAS protein homolog associated with actin, golgi membranes and microtubules
66008	TRAK2	trafficking protein, kinesin binding 2
219982	OR5A1	olfactory receptor, family 5, subfamily A, member 1
51703	ACSL5	acyl-CoA synthetase long-chain family member 5
166979	CDC20B	cell division cycle 20 homolog B (S. cerevisiae)
253152	EPHX4	epoxide hydrolase 4
10285	SMNDC1	survival motor neuron domain containing 1
644915	METTL15P2	methyltransferase like 15 pseudogene 2
4909	NTF4	neurotrophin 4
30008	EFEMP2	EGF containing fibulin-like extracellular matrix protein 2
29940	DSE	dermatan sulfate epimerase
100130602	LOC100130602	regulation of nuclear pre-mRNA domain containing 1A pseudogene
90352	MAGOH3P	mago-nashi homolog 2, proliferation-associated (Drosophila), pseudogene
58476	TP53INP2	tumor protein p53 inducible nuclear protein 2
54902	TTC19	tetratricopeptide repeat domain 19
81532	MOB2	Mps one binder kinase activator-like 2
57657	HCN3	hyperpolarization activated cyclic nucleotide-gated potassium channel 3
84464	SLX4	SLX4 structure-specific endonuclease subunit homolog (S. cerevisiae)
88455	ANKRD13A	ankyrin repeat domain 13A
150946	FAM59B	family with sequence similarity 59, member B
100129458	LOC100129458	cell division control protein 42 homolog
9923	ZBTB40	zinc finger and BTB domain containing 40
9107	MTMR6	myotubularin related protein 6
100133210	COX6B1P7	cytochrome c oxidase subunit VIb polypeptide 1 (ubiquitous) pseudogene 7
27255	CNTN6	contactin 6
138716	C9orf23	chromosome 9 open reading frame 23
284129	SLC26A11	solute carrier family 26, member 11
8639	AOC3	amine oxidase, copper containing 3 (vascular adhesion protein 1)
9583	ENTPD4	ectonucleoside triphosphate diphosphohydrolase 4

644669	LOC644669	ankyrin repeat domain 30B pseudogene
360200	TMPRSS9	transmembrane protease, serine 9
100131211	TMEM194B	transmembrane protein 194B
83893	SPATA16	spermatogenesis associated 16
647034	RPS14P10	ribosomal protein S14 pseudogene 10
23326	USP22	ubiquitin specific peptidase 22
5443	POMC	proopiomelanocortin
23264	ZC3H7B	zinc finger CCCH-type containing 7B
10161	LPAR6	lysophosphatidic acid receptor 6
11052	CPSF6	cleavage and polyadenylation specific factor 6, 68kDa
163486	DENND1B	DENN/MADD domain containing 1B
1859	DYRK1A	dual-specificity tyrosine-(Y)-phosphorylation regulated kinase 1A
644943	RASSF10	Ras association (RalGDS/AF-6) domain family (N-terminal) member 10
7415	VCP	valosin containing protein
3178	HNRNPA1	heterogeneous nuclear ribonucleoprotein A1
81352	OR7M1P	olfactory receptor, family 7, subfamily M, member 1 pseudogene
49861	CLDN20	claudin 20
100128176	LOC100128176	hypothetical protein LOC100128176
729307	LOC729307	hypothetical protein LOC729307
79830	ZMYM1	zinc finger, MYM-type 1
221527	ZBTB12	zinc finger and BTB domain containing 12
10748	KLRAP1	killer cell lectin-like receptor subfamily A pseudogene 1
344807	CD200R1L	CD200 receptor 1-like
79731	NARS2	asparaginyl-tRNA synthetase 2, mitochondrial (putative)
255798	C3orf43	chromosome 3 open reading frame 43
81566	CSRNP2	cysteine-serine-rich nuclear protein 2
57472	CNOT6	CCR4-NOT transcription complex, subunit 6
282618	IL29	interleukin 29 (interferon, lambda 1)
28465	IGHV1-46	immunoglobulin heavy variable 1-46
965	CD58	CD58 molecule
10904	BLCAP	bladder cancer associated protein
8289	ARID1A	AT rich interactive domain 1A (SWI-like)
5930	RBBP6	retinoblastoma binding protein 6
3577	CXCR1	chemokine (C-X-C motif) receptor 1
162962	ZNF836	zinc finger protein 836

285706	LOC285706	cytokine receptor-like factor 3 pseudogene
8841	HDAC3	histone deacetylase 3
100129083	LOC100129083	similar to hCG2045285
56006	C19orf61	chromosome 19 open reading frame 61
27156	RTDR1	rhabdoid tumor deletion region gene 1
84959	UBASH3B	ubiquitin associated and SH3 domain containing B
51333	ZNF771	zinc finger protein 771
391632	OR7E35P	olfactory receptor, family 7, subfamily E, member 35 pseudogene
7879	RAB7A	RAB7A, member RAS oncogene family
28999	KLF15	Kruppel-like factor 15
284697	BTBD8	BTB (POZ) domain containing 8
27154	BRPF3	bromodomain and PHD finger containing, 3
80737	C6orf27	chromosome 6 open reading frame 27
136541	PRSS58	protease, serine, 58
100130292	[No Symbol]	[No Name]
55110	MAGOHB	mago-nashi homolog B (Drosophila)
260332	ECEL1P3	endothelin converting enzyme-like 1, pseudogene 3
171019	ADAMTS19	ADAM metallopeptidase with thrombospondin type 1 motif, 19
100131717	[No Symbol]	[No Name]
8503	PIK3R3	phosphoinositide-3-kinase, regulatory subunit 3 (gamma)
7762	ZNF215	zinc finger protein 215
3067	HDC	histidine decarboxylase
645685	[No Symbol]	[No Name]
23550	PSD4	pleckstrin and Sec7 domain containing 4
10444	ZER1	zer-1 homolog (C. elegans)
407033	MIR30D	microRNA 30d
7150	TOP1	topoisomerase (DNA) I
3169	FOXA1	forkhead box A1
54842	MFSD6	major facilitator superfamily domain containing 6
85302	FBF1	Fas (TNFRSF6) binding factor 1
79075	DSCC1	defective in sister chromatid cohesion 1 homolog (S. cerevisiae)
122622	ADSSL1	adenylosuccinate synthase like 1
402394	IMPDH1P2	IMP (inosine monophosphate) dehydrogenase 1 pseudogene 2
221491	C6orf1	chromosome 6 open reading frame 1
153129	SLC38A9	solute carrier family 38, member 9

8812	CCNK	cyclin K
55028	C17orf80	chromosome 17 open reading frame 80
375611	SLC26A5	solute carrier family 26, member 5 (prestin)
79339	OR51B4	olfactory receptor, family 51, subfamily B, member 4
6122	RPL3	ribosomal protein L3
23318	ZCCHC11	zinc finger, CCHC domain containing 11
8908	GYG2	glycogenin 2
23112	TNRC6B	trinucleotide repeat containing 6B
3606	IL18	interleukin 18 (interferon-gamma-inducing factor)
643954	RPSAP43	ribosomal protein SA pseudogene 43
9654	TTLL4	tubulin tyrosine ligase-like family, member 4
2856	GPR33	G protein-coupled receptor 33 (gene/pseudogene)
64925	CCDC71	coiled-coil domain containing 71
23269	MGA	MAX gene associated
389033	LOC389033	placenta-specific 9 pseudogene
7428	VHL	von Hippel-Lindau tumor suppressor
64764	CREB3L2	cAMP responsive element binding protein 3-like 2
219972	MPEG1	macrophage expressed 1
396	ARHGDIA	Rho GDP dissociation inhibitor (GDI) alpha
23334	KIAA0467	KIAA0467
100130246	[No Symbol]	[No Name]
402057	RPS17P16	ribosomal protein S17 pseudogene 16
151354	FAM84A	family with sequence similarity 84, member A
100131544	[No Symbol]	[No Name]
64834	ELOVL1	ELOVL fatty acid elongase 1
57337	SENP7	SUMO1/sentrin specific peptidase 7
60675	PROK2	prokineticin 2
29104	N6AMT1	N-6 adenine-specific DNA methyltransferase 1 (putative)
9778	KIAA0232	KIAA0232
389207	GRXCR1	glutaredoxin, cysteine rich 1
100129289	[No Symbol]	[No Name]
7351	UCP2	uncoupling protein 2 (mitochondrial, proton carrier)
148641	SLC35F3	solute carrier family 35, member F3
80273	GRPEL1	GrpE-like 1, mitochondrial (E. coli)
345930	ECT2L	epithelial cell transforming sequence 2 oncogene-like

6423	SFRP2	secreted frizzled-related protein 2
645317	CHCHD2P6	coiled-coil-helix-coiled-coil-helix domain containing 2 pseudogene 6
729643	[No Symbol]	[No Name]
30834	ZNRD1	zinc ribbon domain containing 1
2326	FMO1	flavin containing monooxygenase 1
285349	ZNF660	zinc finger protein 660
170954	KIAA1949	KIAA1949
2122	MECOM	MDS1 and EVI1 complex locus
285359	LOC285359	phosducin-like 3 pseudogene
8193	DPF1	D4, zinc and double PHD fingers family 1
100130015	LOC100130015	5-hydroxyisourate hydrolase pseudogene
60468	BACH2	BTB and CNC homology 1, basic leucine zipper transcription factor 2
6230	RPS25	ribosomal protein S25
326	AIRE	autoimmune regulator
80776	B9D2	B9 protein domain 2
644548	LOC644548	exocyst complex component 5 pseudogene
51192	CKLF	chemokine-like factor
390472	KRT8P2	keratin 8 pseudogene 2
54742	LY6K	lymphocyte antigen 6 complex, locus K
55198	APPL2	adaptor protein, phosphotyrosine interaction, PH domain and leucine zipper containing 2
4237	MFAP2	microfibrillar-associated protein 2
646658	TMEM90A	transmembrane protein 90A
10308	ZNF267	zinc finger protein 267
100130433	LOC100130433	hypothetical protein LOC100130433
728339	LOC728339	hypothetical protein LOC728339
53838	C11orf24	chromosome 11 open reading frame 24
56160	NDNL2	necdin-like 2
7464	CORO2A	coronin, actin binding protein, 2A
79991	OBFC1	oligonucleotide/oligosaccharide-binding fold containing 1
339665	SLC35E4	solute carrier family 35, member E4
84258	SYT3	synaptotagmin III
10893	MMP24	matrix metallopeptidase 24 (membrane-inserted)
3814	KISS1	KiSS-1 metastasis-suppressor
80781	COL18A1	collagen, type XVIII, alpha 1
149371	EXOC8	exocyst complex component 8

400765	FLJ35409	FLJ35409 protein
390203	LOC390203	probable polyprenol reductase-like
114785	MBD6	methyl-CpG binding domain protein 6
100131268	TPT1P1	tumor protein, translationally-controlled 1 pseudogene 1
3003	GZMK	granzyme K (granzyme 3; tryptase II)
54554	WDR5B	WD repeat domain 5B
91614	DEPDC7	DEP domain containing 7
83598	LUZP3P	leucine zipper protein 3, pseudogene
2132	EXT2	exostosin 2
3696	ITGB8	integrin, beta 8
26511	CHIC2	cysteine-rich hydrophobic domain 2
390078	OR52E6	olfactory receptor, family 52, subfamily E, member 6
100131533	LOC100131533	dpy-19-like 4 (C. elegans) pseudogene
136540	TRY3	trypsinogen-like pseudogene
343052	LOC343052	similar to hCG1996527
4638	MYLK	myosin light chain kinase
11184	MAP4K1	mitogen-activated protein kinase kinase kinase kinase 1
6155	RPL27	ribosomal protein L27
23331	TTC28	tetratricopeptide repeat domain 28
100128932	LOC100128932	hypothetical LOC100128932
51439	FAM8A1	family with sequence similarity 8, member A1
5277	PIGA	phosphatidylinositol glycan anchor biosynthesis, class A
647163	LOC647163	hypothetical protein LOC647163
100131604	LOC100131604	similar to hCG2045849
22865	SLITRK3	SLIT and NTRK-like family, member 3
9658	ZNF516	zinc finger protein 516
55779	WDR52	WD repeat domain 52
51228	GLTP	glycolipid transfer protein
142827	C10orf129	chromosome 10 open reading frame 129
5704	PSMC4	proteasome (prosome, macropain) 26S subunit, ATPase, 4
9814	SFI1	Sfi1 homolog, spindle assembly associated (yeast)
57661	PHRF1	PHD and ring finger domains 1
55841	WWC3	WWC family member 3
162466	PHOSPHO1	phosphatase, orphan 1
64081	PBLD	phenazine biosynthesis-like protein domain containing

57535	KIAA1324	KIAA1324
100130982	[No Symbol]	[No Name]
9236	CCPG1	cell cycle progression 1
100131807	[No Symbol]	[No Name]
91252	SLC39A13	solute carrier family 39 (zinc transporter), member 13
728217	[No Symbol]	[No Name]
100131813	[No Symbol]	[No Name]
8463	TEAD2	TEA domain family member 2
11120	BTN2A1	butyrophilin, subfamily 2, member A1
51315	KRCC1	lysine-rich coiled-coil 1
285175	UNC80	unc-80 homolog (C. elegans)
100132061	RPL9P14	ribosomal protein L9 pseudogene 14
57238	[No Symbol]	[No Name]
57862	ZNF410	zinc finger protein 410
5428	POLG	polymerase (DNA directed), gamma
360160	CYCSP8	cytochrome c, somatic pseudogene 8
100128944	YWHAQP3	YWHAQ pseudogene 3
57560	IFT80	intraflagellar transport 80 homolog (Chlamydomonas)
79993	ELOVL7	ELOVL fatty acid elongase 7
442183	OR2P1P	olfactory receptor, family 2, subfamily P, member 1 pseudogene
100129251	FTLP4	ferritin, light polypeptide pseudogene 4
8034	SLC25A16	solute carrier family 25 (mitochondrial carrier; Graves disease autoantigen), member 16
65084	TMEM135	transmembrane protein 135
28907	IGKV5-2	immunoglobulin kappa variable 5-2
128360	OR10T2	olfactory receptor, family 10, subfamily T, member 2
100129835	[No Symbol]	[No Name]
100132940	LOC100132940	protein slowmo homolog 2-like
5360	PLTP	phospholipid transfer protein
57048	PLSCR3	phospholipid scramblase 3
3163	HMOX2	heme oxygenase (decycling) 2
130355	C2orf76	chromosome 2 open reading frame 76
54904	WHSC1L1	Wolf-Hirschhorn syndrome candidate 1-like 1
5567	PRKACB	protein kinase, cAMP-dependent, catalytic, beta
221294	NT5DC1	5'-nucleotidase domain containing 1
23197	FAF2	Fas associated factor family member 2

100128266	RANP6	RAN, member RAS oncogene family pseudogene 6
112840	WDR89	WD repeat domain 89
29927	SEC61A1	Sec61 alpha 1 subunit (S. cerevisiae)
146862	UNC45B	unc-45 homolog B (C. elegans)
344758	GPR149	G protein-coupled receptor 149
79296	OR52E1	olfactory receptor, family 52, subfamily E, member 1 (gene/pseudogene)
729138	RPLP1P2	ribosomal protein, large, P1 pseudogene 2
100132993	RPS21P3	ribosomal protein S21 pseudogene 3
57105	CYSLTR2	cysteinyl leukotriene receptor 2
55700	MAP7D1	MAP7 domain containing 1
245972	ATP6V0D2	ATPase, H+ transporting, lysosomal 38kDa, V0 subunit d2
677824	SNORA43	small nucleolar RNA, H/ACA box 43
728655	HULC	highly up-regulated in liver cancer (non-protein coding)
151194	METTL21A	methyltransferase like 21A
645367	GGT8P	gamma-glutamyltransferase 8 pseudogene
5558	PRIM2	primase, DNA, polypeptide 2 (58kDa)
642702	[No Symbol]	[No Name]
54344	DPM3	dolichyl-phosphate mannosyltransferase polypeptide 3
100129536	[No Symbol]	[No Name]
2308	FOXO1	forkhead box O1
221016	CCDC7	coiled-coil domain containing 7
286076	BREA2	breast cancer estrogen-induced apoptosis 2
8720	MBTPS1	membrane-bound transcription factor peptidase, site 1
84154	RPF2	ribosome production factor 2 homolog (S. cerevisiae)
80003	PCNXL2	pecanex-like 2 (Drosophila)
388931	MFSD2B	major facilitator superfamily domain containing 2B
54205	CYCS	cytochrome c, somatic
114134	SLC2A13	solute carrier family 2 (facilitated glucose transporter), member 13
5931	RBBP7	retinoblastoma binding protein 7
9689	BZW1	basic leucine zipper and W2 domains 1
7535	ZAP70	zeta-chain (TCR) associated protein kinase 70kDa
5623	PSPN	persephin
100131890	LOC100131890	neugrin, neurite outgrowth associated pseudogene
729808	[No Symbol]	[No Name]
64866	CDCP1	CUB domain containing protein 1

10473	HMGN4	high mobility group nucleosomal binding domain 4
128770	EIF4E2P1	eukaryotic translation initiation factor 4E family member 2 pseudogene 1
57205	ATP10D	ATPase, class V, type 10D
5306	PITPNA	phosphatidylinositol transfer protein, alpha
126820	WDR63	WD repeat domain 63
646600	C3orf65	chromosome 3 open reading frame 65
26100	WIPI2	WD repeat domain, phosphoinositide interacting 2
5988	RFPL1	ret finger protein-like 1
100128021	[No Symbol]	[No Name]
8871	SYNJ2	synaptojanin 2
92815	HIST3H2A	histone cluster 3, H2a
56521	DNAJC12	DnaJ (Hsp40) homolog, subfamily C, member 12
80152	CENPT	centromere protein T
26099	C1orf144	chromosome 1 open reading frame 144
56252	YLPM1	YLP motif containing 1
23186	RCOR1	REST corepressor 1
100128208	[No Symbol]	[No Name]
114915	NCRNA00219	non-protein coding RNA 219
649489	LOC649489	protein phosphatase 1, regulatory (inhibitor) subunit 2 pseudogene
100132812	LOC100132812	nascent polypeptide-associated complex alpha subunit pseudogene
574452	MIR494	microRNA 494
26505	CNNM3	cyclin M3
132243	H1FOO	H1 histone family, member O, oocyte-specific
9698	PUM1	pumilio homolog 1 (Drosophila)
2778	GNAS	GNAS complex locus
6772	STAT1	signal transducer and activator of transcription 1, 91kDa
644638	[No Symbol]	[No Name]
149111	CNIH3	cornichon homolog 3 (Drosophila)
2288	FKBP4	FK506 binding protein 4, 59kDa
643449	[No Symbol]	[No Name]
100130564	SEPT7P7	septin 7 pseudogene 7
7030	TFE3	transcription factor binding to IGHM enhancer 3
399665	FAM102A	family with sequence similarity 102, member A
442429	EEF1DP2	eukaryotic translation elongation factor 1 delta pseudogene 2
6249	CLIP1	CAP-GLY domain containing linker protein 1

100128604	[No Symbol]	[No Name]
342615	[No Symbol]	[No Name]
63827	BCAN	brevican
337876	CHSY3	chondroitin sulfate synthase 3
968	CD68	CD68 molecule
162972	ZNF550	zinc finger protein 550
26093	CCDC9	coiled-coil domain containing 9
7266	DNAJC7	DnaJ (Hsp40) homolog, subfamily C, member 7
100129191	[No Symbol]	[No Name]
85486	RPS19P1	ribosomal protein S19 pseudogene 1
339843	LOC339843	WW domain containing E3 ubiquitin protein ligase 1 pseudogene
645513	LOC645513	hypothetical LOC645513
9013	TAF1C	TATA box binding protein (TBP)-associated factor, RNA polymerase I, C, 110kDa
100130837	[No Symbol]	[No Name]
153218	SPINK13	serine peptidase inhibitor, Kazal type 13 (putative)
256085	LOC256085	tryptophan rich basic protein pseudogene
6210	RPS15A	ribosomal protein S15a
286472	RAC1P4	ras-related C3 botulinum toxin substrate 1 pseudogene 4
5273	SERPINB10	serpin peptidase inhibitor, clade B (ovalbumin), member 10
81194	OR5F2P	olfactory receptor, family 5, subfamily F, member 2 pseudogene
100129367	LOC100129367	nucleolar and coiled-body phosphoprotein 1 pseudogene
402213	LOC402213	peptidyl-prolyl cis-trans isomerase A-like
100129683	LOC100129683	WEE1 homolog (S. pombe) pseudogene
6788	STK3	serine/threonine kinase 3
149840	C20orf196	chromosome 20 open reading frame 196
8295	TRRAP	transformation/transcription domain-associated protein
79663	HSPBAP1	HSPB (heat shock 27kDa) associated protein 1
360166	CYCSP10	cytochrome c, somatic pseudogene 10
10668	CGRRF1	cell growth regulator with ring finger domain 1
115111	SLC26A7	solute carrier family 26, member 7
9249	DHRS3	dehydrogenase/reductase (SDR family) member 3
199692	ZNF627	zinc finger protein 627
283588	LOC283588	hypothetical LOC283588
6829	SUPT5H	suppressor of Ty 5 homolog (S. cerevisiae)
23609	MKRN2	makorin ring finger protein 2

80345	ZSCAN16	zinc finger and SCAN domain containing 16
1813	DRD2	dopamine receptor D2
100131338	RPL15P13	ribosomal protein L15 pseudogene 13
27177	IL36B	interleukin 36, beta
3621	ING1	inhibitor of growth family, member 1
80014	WWC2	WW and C2 domain containing 2
286676	ILDR1	immunoglobulin-like domain containing receptor 1
100130512	[No Symbol]	[No Name]
81790	RNF170	ring finger protein 170
57185	NIPAL3	NIPA-like domain containing 3
100133205	NCRNA00240	non-protein coding RNA 240
27347	STK39	serine threonine kinase 39
55735	DNAJC11	DnaJ (Hsp40) homolog, subfamily C, member 11
26039	SS18L1	synovial sarcoma translocation gene on chromosome 18-like 1
7781	SLC30A3	solute carrier family 30 (zinc transporter), member 3
10471	PFDN6	prefoldin subunit 6
11025	LILRB3	leukocyte immunoglobulin-like receptor, subfamily B (with TM and ITIM domains), member 3
169355	IDO2	indoleamine 2,3-dioxygenase 2
84267	C9orf64	chromosome 9 open reading frame 64
494326	MIR377	microRNA 377
728869	[No Symbol]	[No Name]
10651	MTX2	metaxin 2
391707	LOC391707	chromatin accessibility complex protein 1-like
7079	TIMP4	TIMP metallopeptidase inhibitor 4
285095	LOC285095	hypothetical protein LOC285095
317705	VN1R5	vomeronasal 1 receptor 5 (gene/pseudogene)
23619	ZIM2	zinc finger, imprinted 2
6922	SKP1P1	S-phase kinase-associated protein 1 pseudogene 1
79071	ELOVL6	ELOVL fatty acid elongase 6
100128013	[No Symbol]	[No Name]
6605	SMARCE1	SWI/SNF related, matrix associated, actin dependent regulator of chromatin, subfamily e, member 1
11224	RPL35	ribosomal protein L35
286343	C9orf150	chromosome 9 open reading frame 150
392529	LOC392529	cordon-bleu homolog (mouse) pseudogene
23054	NCOA6	nuclear receptor coactivator 6

2057	EPOR	erythropoietin receptor
27091	CACNG5	calcium channel, voltage-dependent, gamma subunit 5
7620	ZNF69	zinc finger protein 69
23208	SYT11	synaptotagmin XI
51166	AADAT	aminoadipate aminotransferase
4627	MYH9	myosin, heavy chain 9, non-muscle
144811	C13orf31	chromosome 13 open reading frame 31
84632	AFAP1L2	actin filament associated protein 1-like 2
7975	MAFK	v-maf musculoaponeurotic fibrosarcoma oncogene homolog K (avian)
391804	RBMX2P5	RNA binding motif protein, X-linked 2 pseudogene 5
100131373	LOC100131373	hypothetical LOC100131373
10951	CBX1	chromobox homolog 1
148766	[No Symbol]	[No Name]
10388	SYCP2	synaptonemal complex protein 2
51278	IER5	immediate early response 5
81209	OR5BB1P	olfactory receptor, family 5, subfamily BB, member 1 pseudogene
55370	PPP4R1L	protein phosphatase 4, regulatory subunit 1-like
902	CCNH	cyclin H
100131614	RPS4XP22	ribosomal protein S4X pseudogene 22
25929	GEMIN5	gem (nuclear organelle) associated protein 5
55220	KLHDC8A	kelch domain containing 8A
340526	RGAG4	retrotransposon gag domain containing 4
80778	ZNF34	zinc finger protein 34
51070	NOSIP	nitric oxide synthase interacting protein
4738	NEDD8	neural precursor cell expressed, developmentally down-regulated 8
9878	TOX4	TOX high mobility group box family member 4
7341	SUMO1	SMT3 suppressor of mif two 3 homolog 1 (S. cerevisiae)
26291	FGF21	fibroblast growth factor 21
55506	H2AFY2	H2A histone family, member Y2
6529	SLC6A1	solute carrier family 6 (neurotransmitter transporter, GABA), member 1
5201	PFDN1	prefoldin subunit 1
80263	TRIM45	tripartite motif containing 45
729124	[No Symbol]	[No Name]
10089	KCNK7	potassium channel, subfamily K, member 7
482	ATP1B2	ATPase, Na+/K+ transporting, beta 2 polypeptide

55026	FAM70A	family with sequence similarity 70, member A
100130098	[No Symbol]	[No Name]
5329	PLAUR	plasminogen activator, urokinase receptor
79692	ZNF322A	zinc finger protein 322A
83888	FGFBP2	fibroblast growth factor binding protein 2
118426	LOH12CR1	loss of heterozygosity, 12, chromosomal region 1
151613	TTC14	tetratricopeptide repeat domain 14
56946	C11orf30	chromosome 11 open reading frame 30
3562	IL3	interleukin 3 (colony-stimulating factor, multiple)
3690	ITGB3	integrin, beta 3 (platelet glycoprotein IIIa, antigen CD61)
93517	SDR42E1	short chain dehydrogenase/reductase family 42E, member 1
727727	[No Symbol]	[No Name]
63898	SH2D4A	SH2 domain containing 4A
284443	ZNF493	zinc finger protein 493
54106	TLR9	toll-like receptor 9
23710	GABARAPL1	GABA(A) receptor-associated protein like 1
79973	ZNF442	zinc finger protein 442
388962	BOLA3	bolA homolog 3 (E. coli)
54807	ZNF586	zinc finger protein 586
57677	ZFP14	zinc finger protein 14 homolog (mouse)
79777	ACBD4	acyl-CoA binding domain containing 4
63970	TP53AIP1	tumor protein p53 regulated apoptosis inducing protein 1
6518	SLC2A5	solute carrier family 2 (facilitated glucose/fructose transporter), member 5
339318	ZNF181	zinc finger protein 181
540	ATP7B	ATPase, Cu++ transporting, beta polypeptide
100128898	LOC100128898	latexin pseudogene
55671	SMEK1	SMEK homolog 1, suppressor of mek1 (Dictyostelium)
391257	SUMO1P1	SUMO1 pseudogene 1
220359	TIGD3	tigger transposable element derived 3
729738	[No Symbol]	[No Name]
23401	FRAT2	frequently rearranged in advanced T-cell lymphomas 2
391077	GAPDHP58	glyceraldehyde 3 phosphate dehydrogenase pseudogene 58
57057	TBX20	T-box 20
643949	RPLP2P3	ribosomal protein, large, P2 pseudogene 3
100129847	LOC100129847	transforming protein RhoA-like

197322	ACSF3	acyl-CoA synthetase family member 3
27136	MORC1	MORC family CW-type zinc finger 1
391533	LOC391533	fms-related tyrosine kinase 1 (vascular endothelial growth factor/vascular permeability factor receptor) pseudogene
9651	PLCH2	phospholipase C, eta 2
5287	PIK3C2B	phosphoinositide-3-kinase, class 2, beta polypeptide
120	ADD3	adducin 3 (gamma)
55270	NUDT15	nudix (nucleoside diphosphate linked moiety X)-type motif 15
28462	IGHV1-68	immunoglobulin heavy variable 1-68 (pseudogene)
728152	[No Symbol]	[No Name]
349136	WDR86	WD repeat domain 86
3320	HSP90AA1	heat shock protein 90kDa alpha (cytosolic), class A member 1
8642	DCHS1	dachsous 1 (Drosophila)
89781	HPS4	Hermansky-Pudlak syndrome 4
10017	BCL2L10	BCL2-like 10 (apoptosis facilitator)
100128903	NPM1P4	nucleophosmin 1 (nucleolar phosphoprotein B23, numatrin) pseudogene 4
317701	VN1R2	vomeronasal 1 receptor 2
100130511	[No Symbol]	[No Name]
6406	SEMG1	semenogelin I
8631	SKAP1	src kinase associated phosphoprotein 1
2205	FCER1A	Fc fragment of IgE, high affinity I, receptor for; alpha polypeptide
3142	HLX	H2.0-like homeobox
64788	LMF1	lipase maturation factor 1
375449	MAST4	microtubule associated serine/threonine kinase family member 4
9097	USP14	ubiquitin specific peptidase 14 (tRNA-guanine transglycosylase)
644906	LOC644906	malate dehydrogenase 1, NAD (soluble) pseudogene
83444	INO80B	INO80 complex subunit B
51734	SEPX1	selenoprotein X, 1
100129525	[No Symbol]	[No Name]
728724	LOC728724	hCG1814486
4725	NDUFS5	NADH dehydrogenase (ubiquinone) Fe-S protein 5, 15kDa (NADH-coenzyme Q reductase)
100130192	RPS12P31	ribosomal protein S12 pseudogene 31
645120	LOC645120	apoptosis-inducing factor, mitochondrion-associated, 1 pseudogene
85300	ATCAY	ataxia, cerebellar, Cayman type
642460	ANKRD30BP1	ankyrin repeat domain 30B pseudogene 1
100128431	GEMIN8P1	gem (nuclear organelle) associated protein 8 pseudogene 1

7009	TMBIM6	transmembrane BAX inhibitor motif containing 6
26045	LRRTM2	leucine rich repeat transmembrane neuronal 2
81469	OR2G3	olfactory receptor, family 2, subfamily G, member 3
492307	C8orf22	chromosome 8 open reading frame 22
29114	TAGLN3	transgelin 3
57017	COQ9	coenzyme Q9 homolog (S. cerevisiae)
4885	NPTX2	neuronal pentraxin II
1371	CPOX	coproporphyrinogen oxidase
11227	GALNT5	UDP-N-acetyl-alpha-D-galactosamine:polypeptide N-acetylgalactosaminyltransferase 5 (GalNAc-T5)
65005	MRPL9	mitochondrial ribosomal protein L9
644076	GLYCAM1	glycosylation dependent cell adhesion molecule 1 (pseudogene)
23498	HAAO	3-hydroxyanthranilate 3,4-dioxygenase
57189	KIAA1147	KIAA1147
642776	LOC642776	hypothetical LOC642776
221060	C10orf111	chromosome 10 open reading frame 111
2637	GBX2	gastrulation brain homeobox 2
729740	[No Symbol]	[No Name]
10820	OR7E13P	olfactory receptor, family 7, subfamily E, member 13 pseudogene
9717	SEC14L5	SEC14-like 5 (S. cerevisiae)
5593	PRKG2	protein kinase, cGMP-dependent, type II
22989	MYH15	myosin, heavy chain 15
129790	C7orf13	chromosome 7 open reading frame 13
6191	RPS4X	ribosomal protein S4, X-linked
6014	RIT2	Ras-like without CAAX 2
4166	CHST6	carbohydrate (N-acetylglucosamine 6-O) sulfotransferase 6
2497	FTH1P2	ferritin, heavy polypeptide 1 pseudogene 2
2918	GRM8	glutamate receptor, metabotropic 8
51678	MPP6	membrane protein, palmitoylated 6 (MAGUK p55 subfamily member 6)
123606	NIPA1	non imprinted in Prader-Willi/Angelman syndrome 1
5621	PRNP	prion protein
116228	FAM36A	family with sequence similarity 36, member A
5020	OXT	oxytocin, prepropeptide
26065	LSM14A	LSM14A, SCD6 homolog A (S. cerevisiae)
65989	DLK2	delta-like 2 homolog (Drosophila)
128997	SLC9A3P2	solute carrier family 9 (sodium/hydrogen exchanger), member 3 pseudogene 2

205564	SENP5	SUMO1/sentrin specific peptidase 5
353144	LCE3C	late cornified envelope 3C
100131423	[No Symbol]	[No Name]
79140	CCDC28B	coiled-coil domain containing 28B
8676	STX11	syntaxin 11
2203	FBP1	fructose-1,6-bisphosphatase 1
10040	TOM1L1	target of myb1 (chicken)-like 1
100129291	LOC100129291	uncharacterized protein CXorf49-like
112464	PRKCDBP	protein kinase C, delta binding protein
284390	ZNF763	zinc finger protein 763
245806	VGLL2	vestigial like 2 (Drosophila)
9445	ITM2B	integral membrane protein 2B
6767	ST13	suppression of tumorigenicity 13 (colon carcinoma) (Hsp70 interacting protein)
728142	LOC728142	hypothetical LOC728142
149844	LOC149844	synaptotagmin binding, cytoplasmic RNA interacting protein pseudogene
649166	LOC649166	eukaryotic translation initiation factor 3, subunit J pseudogene
729257	RPS24P4	ribosomal protein S24 pseudogene 4
730021	LOC730021	eukaryotic translation initiation factor 3, subunit J pseudogene
51	ACOX1	acyl-CoA oxidase 1, palmitoyl
554225	STRCP1	stereocilin pseudogene 1
56681	SAR1A	SAR1 homolog A (S. cerevisiae)
1471	CST3	cystatin C
392516	KRT18P49	keratin 18 pseudogene 49
4683	NBN	nibrin
200373	PCDP1	primary ciliary dyskinesia protein 1
729142	[No Symbol]	[No Name]
100124402	LOC100124402	ubiquitin-fold modifier conjugating enzyme 1 pseudogene
100131471	LOC100131471	presenilins-associated rhomboid-like protein, mitochondrial-like
100128826	[No Symbol]	[No Name]
2475	MTOR	mechanistic target of rapamycin (serine/threonine kinase)
5467	PPARD	peroxisome proliferator-activated receptor delta
374403	TBC1D10C	TBC1 domain family, member 10C
54517	PUS7	pseudouridylate synthase 7 homolog (S. cerevisiae)
25885	POLR1A	polymerase (RNA) I polypeptide A, 194kDa
1448	CSN3	casein kappa

11200	CHEK2	CHK2 checkpoint homolog (S. pombe)
4693	NDP	Norrie disease (pseudoglioma)
402374	OR13D3P	olfactory receptor, family 13, subfamily D, member 3 pseudogene
221756	MGC39372	serpin peptidase inhibitor, clade B (ovalbumin), member 9 pseudogene
5726	TAS2R38	taste receptor, type 2, member 38
3305	HSPA1L	heat shock 70kDa protein 1-like
790	CAD	carbamoyl-phosphate synthetase 2, aspartate transcarbamylase, and dihydroorotase
100129103	[No Symbol]	[No Name]
8994	LIMD1	LIM domains containing 1
6940	ZNF354A	zinc finger protein 354A
5859	QARS	glutaminyl-tRNA synthetase
2533	FYB	FYN binding protein
92126	DSEL	dermatan sulfate epimerase-like
644129	LOC644129	hypothetical protein LOC644129
9853	RUSC2	RUN and SH3 domain containing 2
804	CALM1P2	calmodulin 1 (phosphorylase kinase, delta) pseudogene 2
100129362	[No Symbol]	[No Name]
56259	CTNNBL1	catenin, beta like 1
818	CAMK2G	calcium/calmodulin-dependent protein kinase II gamma
8534	CHST1	carbohydrate (keratan sulfate Gal-6) sulfotransferase 1
221504	ZBTB9	zinc finger and BTB domain containing 9
8618	CADPS	Ca++-dependent secretion activator
340602	CXorf67	chromosome X open reading frame 67
157956	CDC20P1	cell division cycle 20 homolog (S. cerevisiae) pseudogene 1
252839	TMEM9	transmembrane protein 9
11098	PRSS23	protease, serine, 23
100130147	[No Symbol]	[No Name]
58158	NEUROD4	neurogenic differentiation 4
100128422	[No Symbol]	[No Name]
167359	NIM1	serine/threonine-protein kinase NIM1
3061	HCRTR1	hypocretin (orexin) receptor 1
100130331	LOC100130331	actin, gamma-like
6697	SPR	sepiapterin reductase (7,8-dihydrobiopterin:NADP+ oxidoreductase)
3720	JARID2	jumonji, AT rich interactive domain 2
140469	MYO3B	myosin IIIB

7204	TRIO	triple functional domain (PTPRF interacting)
57505	AARS2	alanyl-tRNA synthetase 2, mitochondrial (putative)
100129427	LOC100129427	hypothetical LOC100129427
5495	PPM1B	protein phosphatase, Mg2+/Mn2+ dependent, 1B
994	CDC25B	cell division cycle 25 homolog B (S. pombe)
735301	SNHG9	small nucleolar RNA host gene 9 (non-protein coding)
55280	CWF19L1	CWF19-like 1, cell cycle control (S. pombe)
1647	GADD45A	growth arrest and DNA-damage-inducible, alpha
26225	ARL5A	ADP-ribosylation factor-like 5A
100129523	[No Symbol]	[No Name]
10772	SRSF10	serine/arginine-rich splicing factor 10
5256	PHKA2	phosphorylase kinase, alpha 2 (liver)
100130302	FAM48B1	family with sequence similarity 48, member B1
26164	GTPBP5	GTP binding protein 5 (putative)
7165	TPD52L2	tumor protein D52-like 2
390436	OR4K17	olfactory receptor, family 4, subfamily K, member 17
100129960	LOC100129960	syntenin-1-like
130540	ALS2CR12	amyotrophic lateral sclerosis 2 (juvenile) chromosome region, candidate 12
166929	SGMS2	sphingomyelin synthase 2
2020	EN2	engrailed homeobox 2
81407	OR2W4P	olfactory receptor, family 2, subfamily W, member 4 pseudogene
23603	CORO1C	coronin, actin binding protein, 1C
646255	RPL19P16	ribosomal protein L19 pseudogene 16
353174	ZACN	zinc activated ligand-gated ion channel
150	ADRA2A	adrenergic, alpha-2A-, receptor
5195	PEX14	peroxisomal biogenesis factor 14
215	ABCD1	ATP-binding cassette, sub-family D (ALD), member 1
54530	[No Symbol]	[No Name]
90637	ZFAND2A	zinc finger, AN1-type domain 2A
100131726	LOC100131726	HCC-related HCC-C11_v3
348980	HCN1	hyperpolarization activated cyclic nucleotide-gated potassium channel 1
4439	MSH5	mutS homolog 5 (E. coli)
1007	CDH9	cadherin 9, type 2 (T1-cadherin)
100129061	RPL7P25	ribosomal protein L7 pseudogene 25
139163	SALL1P1	sal-like 1 (Drosophila) pseudogene 1

2058	EPRS	glutamyl-prolyl-tRNA synthetase
645360	HMGB3P5	high mobility group box 3 pseudogene 5
100129658	LOC100129658	ubiquitin-conjugating enzyme E2 variant 1-like
9788	MTSS1	metastasis suppressor 1
80318	GKAP1	G kinase anchoring protein 1
65243	ZNF643	zinc finger protein 643
54986	ULK4	unc-51-like kinase 4 (C. elegans)
54931	RG9MTD1	RNA (guanine-9-) methyltransferase domain containing 1
344760	VN2R1P	vomeronasal 2 receptor 1 pseudogene
1522	CTSZ	cathepsin Z
5434	POLR2E	polymerase (RNA) II (DNA directed) polypeptide E, 25kDa
400129	SMARCE1P5	SWI/SNF related, matrix associated, actin dependent regulator of chromatin, subfamily e, member 1 pseudogene 5
1690	COCH	coagulation factor C homolog, cochlin (Limulus polyphemus)
6259	RYK	RYK receptor-like tyrosine kinase
54019	SLC6A6P1	solute carrier family 6, member 6 pseudogene 1
7918	GPANK1	G patch domain and ankyrin repeats 1
6854	SYN2	synapsin II
643875	CNN3P1	calponin 3, acidic pseudogene 1
442338	IRS3P	insulin receptor substrate 3, pseudogene
81619	TSPAN14	tetraspanin 14
286183	NKAIN3	Na+/K+ transporting ATPase interacting 3
81856	ZNF611	zinc finger protein 611
100132529	LOC100132529	hypothetical LOC100132529
10560	SLC19A2	solute carrier family 19 (thiamine transporter), member 2
3608	ILF2	interleukin enhancer binding factor 2, 45kDa
4045	LSAMP	limbic system-associated membrane protein
64403	CDH24	cadherin 24, type 2
643582	LOC643582	aldo-keto reductase family 1, member B10 (aldose reductase) pseudogene
791120	[No Symbol]	[No Name]
574080	LOC574080	uncharacterized hematopoietic stem/progenitor cells protein MDS029 pseudogene 3
23266	LPHN2	latrophilin 2
389203	C4orf52	chromosome 4 open reading frame 52
2145	EZH1	enhancer of zeste homolog 1 (Drosophila)
2590	GALNT2	UDP-N-acetyl-alpha-D-galactosamine:polypeptide N-acetylgalactosaminyltransferase 2 (GalNAc-T2)
7318	UBA7	ubiquitin-like modifier activating enzyme 7

127428	TCEANC2	transcription elongation factor A (SII) N-terminal and central domain containing 2
23766	GABARAPL3	GABA(A) receptors associated protein like 3, pseudogene
392454	LOC392454	proliferating cell nuclear antigen pseudogene
643684	[No Symbol]	[No Name]
55410	NCRNA00185	non-protein coding RNA 185
643264	RPL21P106	ribosomal protein L21 pseudogene 106
440031	LOC440031	hypothetical LOC440031
125875	CLDND2	claudin domain containing 2
58490	RPRD1B	regulation of nuclear pre-mRNA domain containing 1B
23248	RPRD2	regulation of nuclear pre-mRNA domain containing 2
51564	HDAC7	histone deacetylase 7
9994	CASP8AP2	caspase 8 associated protein 2
100127206	KIAA1024L	KIAA1024-like
8857	FCGBP	Fc fragment of IgG binding protein
10214	SSX3	synovial sarcoma, X breakpoint 3
399979	SNX19	sorting nexin 19
22987	SV2C	synaptic vesicle glycoprotein 2C
644780	[No Symbol]	[No Name]
55773	TBC1D23	TBC1 domain family, member 23
729717	[No Symbol]	[No Name]
389458	LOC389458	hypothetical LOC389458
100133235	RPL26P14	ribosomal protein L26 pseudogene 14
130813	C2orf50	chromosome 2 open reading frame 50
399687	MYO18A	myosin XVIIIA
79161	C7orf23	chromosome 7 open reading frame 23
83551	TAAR8	trace amine associated receptor 8
100132755	[No Symbol]	[No Name]
8578	SCARF1	scavenger receptor class F, member 1
399950	ST13P10	suppression of tumorigenicity 13 (colon carcinoma) (Hsp70 interacting protein) pseudogene 10
91937	TIMD4	T-cell immunoglobulin and mucin domain containing 4
126626	GABPB2	GA binding protein transcription factor, beta subunit 2
2395	FXN	frataxin
728665	[No Symbol]	[No Name]
728342	LOC728342	hypothetical protein LOC728342
84217	ZMYND12	zinc finger, MYND-type containing 12

128218	TMEM125	transmembrane protein 125
100128999	[No Symbol]	[No Name]
100131925	[No Symbol]	[No Name]
374872	C19orf35	chromosome 19 open reading frame 35
3208	HPCA	hippocalcin
10804	GJB6	gap junction protein, beta 6, 30kDa
128371	OR6K6	olfactory receptor, family 6, subfamily K, member 6
100128548	LOC100128548	60S ribosomal protein L17-like
100131983	HNRNPA1P25	heterogeneous nuclear ribonucleoprotein A1 pseudogene 25
26872	STEAP1	six transmembrane epithelial antigen of the prostate 1
728777	[No Symbol]	[No Name]
729537	[No Symbol]	[No Name]
80352	RNF39	ring finger protein 39
730255	RPL17P8	ribosomal protein L17 pseudogene 8
5488	PPIAP16	peptidylprolyl isomerase A (cyclophilin A) pseudogene 16
3096	HIVEP1	human immunodeficiency virus type I enhancer binding protein 1
100129388	LOC100129388	RWD domain-containing protein 1-like
55339	WDR33	WD repeat domain 33
27294	DHDH	dihydrodiol dehydrogenase (dimeric)
645996	NAP1L6	nucleosome assembly protein 1-like 6
6141	RPL18	ribosomal protein L18
1361	CPB2	carboxypeptidase B2 (plasma)
2764	GMFB	glia maturation factor, beta
6862	T	T, brachyury homolog (mouse)
7592	ZNF41	zinc finger protein 41
5888	RAD51	RAD51 homolog (S. cerevisiae)
801	CALM1	calmodulin 1 (phosphorylase kinase, delta)
728805	LOC728805	hypothetical protein LOC728805
147660	ZNF578	zinc finger protein 578
643246	MAP1LC3B2	microtubule-associated protein 1 light chain 3 beta 2
64324	NSD1	nuclear receptor binding SET domain protein 1
3043	HBB	hemoglobin, beta
7504	XK	X-linked Kx blood group (McLeod syndrome)
23270	TSPYL4	TSPY-like 4
100130251	LOC100130251	hypothetical LOC100130251

90665	TBL1Y	transducin (beta)-like 1, Y-linked
23336	SYNM	synemin, intermediate filament protein
1087	CEACAM7	carcinoembryonic antigen-related cell adhesion molecule 7
158833	AWAT1	acyl-CoA wax alcohol acyltransferase 1
54477	PLEKHA5	pleckstrin homology domain containing, family A member 5
650172	CCT4P1	chaperonin containing TCP1, subunit 4 (delta) pseudogene 1
3652	IPP	intracisternal A particle-promoted polypeptide
5007	OSBP	oxysterol binding protein
5168	ENPP2	ectonucleotide pyrophosphatase/phosphodiesterase 2
4313	MMP2	matrix metallopeptidase 2 (gelatinase A, 72kDa gelatinase, 72kDa type IV collagenase)
55644	OSGEP	O-sialoglycoprotein endopeptidase
100128454	LOC100128454	39S ribosomal protein L32, mitochondrial-like
646196	SMARCE1P6	SWI/SNF related, matrix associated, actin dependent regulator of chromatin, subfamily e, member 1 pseudogene 6
642791	LOC642791	elongation factor 1-alpha 2-like
6631	SNRPC	small nuclear ribonucleoprotein polypeptide C
6347	CCL2	chemokine (C-C motif) ligand 2
442211	ATP6V0CP3	ATPase, H+ transporting, lysosomal 16kDa, V0 subunit c pseudogene 3
83714	NRIP2	nuclear receptor interacting protein 2
51164	DCTN4	dynactin 4 (p62)
729009	FTH1P20	ferritin, heavy polypeptide 1 pseudogene 20
100132973	LOC100132973	transcription elongation factor B (SIII), polypeptide 1 (15kDa, elongin C) pseudogene
147199	SCGB1C1	secretoglobin, family 1C, member 1
100129256	LOC100129256	nicotinamide nucleotide adenylyltransferase 1 pseudogene
79869	CPSF7	cleavage and polyadenylation specific factor 7, 59kDa
54885	TBC1D8B	TBC1 domain family, member 8B (with GRAM domain)
22920	KIFAP3	kinesin-associated protein 3
131578	LRRC15	leucine rich repeat containing 15
285908	NCRNA00174	non-protein coding RNA 174
441938	[No Symbol]	[No Name]
255725	OR52B2	olfactory receptor, family 52, subfamily B, member 2
100128420	LOC100128420	hypothetical protein LOC100128420
494197	SPANXN5	SPANX family, member N5
100129377	RPL23P9	ribosomal protein L23 pseudogene 9
10572	SIVA1	SIVA1, apoptosis-inducing factor
2101	ESRRA	estrogen-related receptor alpha

4818	NKG7	natural killer cell group 7 sequence
1968	EIF2S3	eukaryotic translation initiation factor 2, subunit 3 gamma, 52kDa
222698	NKAPL	NFKB activating protein-like
27063	ANKRD1	ankyrin repeat domain 1 (cardiac muscle)
7363	UGT2B4	UDP glucuronosyltransferase 2 family, polypeptide B4
9334	B4GALT5	UDP-Gal:betaGlcNAc beta 1,4- galactosyltransferase, polypeptide 5
732156	LOC732156	mucin-3A-like
1164	CKS2	CDC28 protein kinase regulatory subunit 2
4802	NFYC	nuclear transcription factor Y, gamma
442213	C6orf138	chromosome 6 open reading frame 138
57717	PCDHB16	protocadherin beta 16
81487	OR10AA1P	olfactory receptor, family 10, subfamily AA, member 1 pseudogene
100131051	LOC100131051	mediator complex subunit 28 pseudogene
8567	MADD	MAP-kinase activating death domain
56288	PARD3	par-3 partitioning defective 3 homolog (C. elegans)
6898	TAT	tyrosine aminotransferase
85027	C5orf62	chromosome 5 open reading frame 62
6622	SNCA	synuclein, alpha (non A4 component of amyloid precursor)
100131935	GAPDHP41	glyceraldehyde 3 phosphate dehydrogenase pseudogene 41
27329	ANGPTL3	angiopoietin-like 3
25777	SUN2	Sad1 and UNC84 domain containing 2
79742	CXorf36	chromosome X open reading frame 36
339559	ZNF642	zinc finger protein 642
55140	ELP3	elongation protein 3 homolog (S. cerevisiae)
29950	SERTAD1	SERTA domain containing 1
29980	DONSON	downstream neighbor of SON
5676	PSG7	pregnancy specific beta-1-glycoprotein 7 (gene/pseudogene)
29925	GMPPB	GDP-mannose pyrophosphorylase B
9699	RIMS2	regulating synaptic membrane exocytosis 2
79613	TMCO7	transmembrane and coiled-coil domains 7
645053	[No Symbol]	[No Name]
348645	C22orf34	chromosome 22 open reading frame 34
4584	MUC3A	mucin 3A, cell surface associated
8443	GNPAT	glyceronephosphate O-acyltransferase
93611	FBXO44	F-box protein 44

407037	MIR320A	microRNA 320a
646720	[No Symbol]	[No Name]
729971	[No Symbol]	[No Name]
729082	LOC729082	hypothetical LOC729082
388942	LOC388942	hypothetical LOC388942
2741	GLRA1	glycine receptor, alpha 1
23639	LRRC6	leucine rich repeat containing 6
54469	ZFAND6	zinc finger, AN1-type domain 6
64321	SOX17	SRY (sex determining region Y)-box 17
4215	MAP3K3	mitogen-activated protein kinase kinase kinase 3
100132310	LOC100132310	FCF1 small subunit (SSU) processome component homolog (S. cerevisiae) pseudogene
2117	ETV3	ets variant 3
9674	KIAA0040	KIAA0040
2502	FTH1P10	ferritin, heavy polypeptide 1 pseudogene 10
100132739	[No Symbol]	[No Name]
390107	RPS24P15	ribosomal protein S24 pseudogene 15
51135	IRAK4	interleukin-1 receptor-associated kinase 4
80097	MZT2B	mitotic spindle organizing protein 2B
5875	RABGGTA	Rab geranylgeranyltransferase, alpha subunit
2059	EPS8	epidermal growth factor receptor pathway substrate 8
83696	TRAPPC9	trafficking protein particle complex 9
2070	EYA4	eyes absent homolog 4 (Drosophila)
11086	ADAM29	ADAM metallopeptidase domain 29
403315	FAM92A3	family with sequence similarity 92, member A3
651250	LOC651250	leucine rich repeat containing 37, member A3 pseudogene
392133	OR10AC1P	olfactory receptor, family 10, subfamily AC, member 1 pseudogene
57181	SLC39A10	solute carrier family 39 (zinc transporter), member 10
50650	ARHGEF3	Rho guanine nucleotide exchange factor (GEF) 3
51447	IP6K2	inositol hexakisphosphate kinase 2
81617	CAB39L	calcium binding protein 39-like
100129248	LOC100129248	polyprenyl pyrophosphate synthetase pseudogene
152405	C3orf30	chromosome 3 open reading frame 30
100133108	RPL13P10	ribosomal protein L13 pseudogene 10
131965	METTL6	methyltransferase like 6
56658	TRIM39	tripartite motif containing 39

10678	B3GNT2	UDP-GlcNAc:betaGal beta-1,3-N-acetylglucosaminyltransferase 2
2246	FGF1	fibroblast growth factor 1 (acidic)
8338	HIST2H2AC	histone cluster 2, H2ac
126826	TGIF2P1	TGFB-induced factor homeobox 2 pseudogene 1
92259	MRPS36	mitochondrial ribosomal protein S36
100101246	LOC100101246	interferon induced transmembrane protein pseudogene
115653	KIR3DL3	killer cell immunoglobulin-like receptor, three domains, long cytoplasmic tail, 3
1831	TSC22D3	TSC22 domain family, member 3
100129831	LOC100129831	EPWW6493
1263	PLK3	polo-like kinase 3
10870	HCST	hematopoietic cell signal transducer
4200	ME2	malic enzyme 2, NAD(+)-dependent, mitochondrial
90634	N4BP2L1	NEDD4 binding protein 2-like 1
646612	EEF1A1P25	eukaryotic translation elongation factor 1 alpha 1 pseudogene 25
64478	CSMD1	CUB and Sushi multiple domains 1
154860	LOC154860	hypothetical LOC154860
645874	[No Symbol]	[No Name]
643916	LOC643916	RAB1A, member RAS oncogene family pseudogene
100131289	LOC100131289	similar to hCG1820927
80212	CCDC92	coiled-coil domain containing 92
5199	CFP	complement factor properdin
339669	C22orf33	chromosome 22 open reading frame 33
92822	ZNF276	zinc finger protein 276
23180	RFTN1	raftlin, lipid raft linker 1
9921	RNF10	ring finger protein 10
91408	BTF3L4	basic transcription factor 3-like 4
119765	OR4B1	olfactory receptor, family 4, subfamily B, member 1
8470	SORBS2	sorbin and SH3 domain containing 2
100131568	LOC100131568	RNA binding motif protein 43 pseudogene
8209	C21orf33	chromosome 21 open reading frame 33
202122	RPL27P10	ribosomal protein L27 pseudogene 10
121727	PEX12P1	peroxisomal biogenesis factor 12 pseudogene 1
91526	ANKRD44	ankyrin repeat domain 44
406911	MIR125B1	microRNA 125b-1
2315	MLANA	melan-A

79533	OR52E3P	olfactory receptor, family 52, subfamily E, member 3 pseudogene
319144	RPL21P13	ribosomal protein L21 pseudogene 13
57520	HECW2	HECT, C2 and WW domain containing E3 ubiquitin protein ligase 2
5693	PSMB5	proteasome (prosome, macropain) subunit, beta type, 5
140831	ZSWIM3	zinc finger, SWIM-type containing 3
55743	CHFR	checkpoint with forkhead and ring finger domains
64711	HS3ST6	heparan sulfate (glucosamine) 3-O-sulfotransferase 6
647633	LOC647633	hypothetical LOC647633
6007	RHD	Rh blood group, D antigen
7564	ZNF16	zinc finger protein 16
5145	PDE6A	phosphodiesterase 6A, cGMP-specific, rod, alpha
1645	AKR1C1	aldo-keto reductase family 1, member C1 (dihydrodiol dehydrogenase 1; 20-alpha (3-alpha)-hydroxysteroid dehydrogenase)
286148	DPY19L4	dpy-19-like 4 (C. elegans)
128611	ZNF831	zinc finger protein 831
6627	SNRPA1	small nuclear ribonucleoprotein polypeptide A'
122876	GPHB5	glycoprotein hormone beta 5
2702	GJA5	gap junction protein, alpha 5, 40kDa
9620	CELSR1	cadherin, EGF LAG seven-pass G-type receptor 1 (flamingo homolog, Drosophila)
9967	THRAP3	thyroid hormone receptor associated protein 3
83723	FAM57B	family with sequence similarity 57, member B
4051	CYP4F3	cytochrome P450, family 4, subfamily F, polypeptide 3
51702	PADI3	peptidyl arginine deiminase, type III
160518	DENND5B	DENN/MADD domain containing 5B
729486	IL9RP3	interleukin 9 receptor pseudogene 3
27241	BBS9	Bardet-Biedl syndrome 9
64943	NT5DC2	5'-nucleotidase domain containing 2
1946	EFNA5	ephrin-A5
387129	NPSR1	neuropeptide S receptor 1
727969	TSPY16P	testis specific protein, Y-linked 16, pseudogene
50618	ITSN2	intersectin 2
100131476	[No Symbol]	[No Name]
646908	[No Symbol]	[No Name]
389432	SAMD5	sterile alpha motif domain containing 5
130026	ICA1L	islet cell autoantigen 1,69kDa-like
23434	C3orf27	chromosome 3 open reading frame 27

285647	LOC285647	suppressor of defective silencing 3 pseudogene
84240	ZCCHC9	zinc finger, CCHC domain containing 9
128668	RPL7AP12	ribosomal protein L7a pseudogene 12
731741	RPL32P12	ribosomal protein L32 pseudogene 12
9421	HAND1	heart and neural crest derivatives expressed 1
4151	MB	myoglobin
57165	GJC2	gap junction protein, gamma 2, 47kDa
644618	[No Symbol]	[No Name]
1717	DHCR7	7-dehydrocholesterol reductase
132864	CPEB2	cytoplasmic polyadenylation element binding protein 2
3875	KRT18	keratin 18
148867	SLC30A7	solute carrier family 30 (zinc transporter), member 7
285829	SUMO2P1	SMT3 suppressor of mif two 3 homolog 2 (S. cerevisiae) pseudogene 1
219790	RTKN2	rhotekin 2
109	ADCY3	adenylate cyclase 3
728599	CIAPIN1P	cytokine induced apoptosis inhibitor 1 pseudogene
946	SIGLEC6	sialic acid binding Ig-like lectin 6
345571	LOC345571	RUN and FYVE domain containing 3 pseudogene
7348	UPK1B	uroplakin 1B
128370	OR6K4P	olfactory receptor, family 6, subfamily K, member 4 pseudogene
153643	FAM81B	family with sequence similarity 81, member B
23601	CLEC5A	C-type lectin domain family 5, member A
157570	ESCO2	establishment of cohesion 1 homolog 2 (S. cerevisiae)
26577	PCOLCE2	procollagen C-endopeptidase enhancer 2
79865	TREML2	triggering receptor expressed on myeloid cells-like 2
100128169	[No Symbol]	[No Name]
127534	GJB4	gap junction protein, beta 4, 30.3kDa
2624	GATA2	GATA binding protein 2
144193	AMDHD1	amidohydrolase domain containing 1
23293	SMG6	Smg-6 homolog, nonsense mediated mRNA decay factor (C. elegans)
441720	[No Symbol]	[No Name]
79576	NKAP	NFKB activating protein
8301	PICALM	phosphatidylinositol binding clathrin assembly protein
90113	VWA5B2	von Willebrand factor A domain containing 5B2
677846	SNORA80	small nucleolar RNA, H/ACA box 80

390081	OR52E4	olfactory receptor, family 52, subfamily E, member 4
56203	LMOD3	leiomodin 3 (fetal)
10112	KIF20A	kinesin family member 20A
442184	OR2B3	olfactory receptor, family 2, subfamily B, member 3
100129497	LOC100129497	chromosome 14 open reading frame 138 pseudogene
2673	GFPT1	glutamine--fructose-6-phosphate transaminase 1
1977	EIF4E	eukaryotic translation initiation factor 4E
84894	LINGO1	leucine rich repeat and Ig domain containing 1
5127	CDK16	cyclin-dependent kinase 16
51429	SNX9	sorting nexin 9
51466	EVL	Enah/Vasp-like
389332	LOC389332	hypothetical LOC389332
2044	EPHA5	EPH receptor A5
9122	SLC16A4	solute carrier family 16, member 4 (monocarboxylic acid transporter 5)
2635	GBP3	guanylate binding protein 3
729454	LOC729454	destrin-like
100132989	[No Symbol]	[No Name]
392368	LOC392368	BEN domain containing 3 pseudogene
5270	SERPINE2	serpin peptidase inhibitor, clade E (nexin, plasminogen activator inhibitor type 1), member 2
653794	TRIM60P14	tripartite motif containing 60 pseudogene 14
286122	C8orf31	chromosome 8 open reading frame 31
5936	RBM4	RNA binding motif protein 4
80119	PIF1	PIF1 5'-to-3' DNA helicase homolog (S. cerevisiae)
7098	TLR3	toll-like receptor 3
100128119	MCART5P	mitochondrial carrier triple repeat 5 pseudogene
5739	PTGIR	prostaglandin I2 (prostacyclin) receptor (IP)
729626	LOC729626	hypothetical protein LOC729626
81569	ACTL8	actin-like 8
3664	IRF6	interferon regulatory factor 6
5348	FXYD1	FXYD domain containing ion transport regulator 1
442920	MIR196B	microRNA 196b
81695	OR2B7P	olfactory receptor, family 2, subfamily B, member 7 pseudogene
54901	CDKAL1	CDK5 regulatory subunit associated protein 1-like 1
9528	TMEM59	transmembrane protein 59
153768	PRELID2	PRELI domain containing 2

128338	DRAM2	DNA-damage regulated autophagy modulator 2
28387	IGHV5-78	immunoglobulin heavy variable 5-78 (pseudogene)
4867	NPHP1	nephronophthisis 1 (juvenile)
27178	IL37	interleukin 37
10670	RRAGA	Ras-related GTP binding A
26103	LRIT1	leucine-rich repeat, immunoglobulin-like and transmembrane domains 1
79800	ALS2CR8	amyotrophic lateral sclerosis 2 (juvenile) chromosome region, candidate 8
389384	C6orf222	chromosome 6 open reading frame 222
9963	SLC23A1	solute carrier family 23 (nucleobase transporters), member 1
54507	ADAMTSL4	ADAMTS-like 4
79609	METTL21D	methyltransferase like 21D
9474	ATG5	ATG5 autophagy related 5 homolog (S. cerevisiae)
100128437	LOC100128437	hypothetical protein LOC100128437
11019	LIAS	lipoic acid synthetase
100132937	[No Symbol]	[No Name]
51309	ARMCX1	armadillo repeat containing, X-linked 1
25898	RCHY1	ring finger and CHY zinc finger domain containing 1
493913	PAPPA-AS1	PAPPA antisense RNA 1 (non-protein coding)
503583	ARGFXP1	arginine-fifty homeobox pseudogene 1
4343	MOV10	Mov10, Moloney leukemia virus 10, homolog (mouse)
1298	COL9A2	collagen, type IX, alpha 2
167153	PAPD4	PAP associated domain containing 4
566	AZU1	azurocidin 1
121296	LOC121296	transmembrane protein 132B pseudogene
6430	SRSF5	serine/arginine-rich splicing factor 5
100129909	[No Symbol]	[No Name]
84307	ZNF397	zinc finger protein 397
55075	UACA	uveal autoantigen with coiled-coil domains and ankyrin repeats
150737	TTC30B	tetratricopeptide repeat domain 30B
83479	DDX59	DEAD (Asp-Glu-Ala-Asp) box polypeptide 59
1991	ELANE	elastase, neutrophil expressed
2499	FTH1P4	ferritin, heavy polypeptide 1 pseudogene 4
57121	LPAR5	lysophosphatidic acid receptor 5
8522	GAS7	growth arrest-specific 7
170960	ZNF721	zinc finger protein 721

150709	ANKAR	ankyrin and armadillo repeat containing
202020	FLJ39653	hypothetical FLJ39653
51816	CECR1	cat eye syndrome chromosome region, candidate 1
100131706	[No Symbol]	[No Name]
23383	MAU2	MAU2 chromatid cohesion factor homolog (C. elegans)
22906	TRAK1	trafficking protein, kinesin binding 1
80319	CXXC4	CXXC finger protein 4
26140	TTLL3	tubulin tyrosine ligase-like family, member 3
389136	VGLL3	vestigial like 3 (Drosophila)
9162	DGKI	diacylglycerol kinase, iota
387119	C6orf204	chromosome 6 open reading frame 204
4179	CD46	CD46 molecule, complement regulatory protein
10718	NRG3	neuregulin 3
9108	MTMR7	myotubularin related protein 7
55908	LOC55908	hepatocellular carcinoma-associated gene TD26
79699	ZYG11B	zyg-11 homolog B (C. elegans)
79673	ZNF329	zinc finger protein 329
302	ANXA2	annexin A2
4698	NDUFA5	NADH dehydrogenase (ubiquinone) 1 alpha subcomplex, 5, 13kDa
6611	SMS	spermine synthase
100132083	[No Symbol]	[No Name]
3910	LAMA4	laminin, alpha 4
9705	ST18	suppression of tumorigenicity 18 (breast carcinoma) (zinc finger protein)
642513	LOC642513	BTB/POZ domain-containing protein KCTD9-like
100132679	[No Symbol]	[No Name]
7791	ZYX	zyxin
5778	PTPN7	protein tyrosine phosphatase, non-receptor type 7
1014	CDH16	cadherin 16, KSP-cadherin
94015	TTYH2	tweety homolog 2 (Drosophila)
440044	SLC22A20	solute carrier family 22, member 20
9551	ATP5J2	ATP synthase, H+ transporting, mitochondrial Fo complex, subunit F2
29765	TMOD4	tropomodulin 4 (muscle)
220136	CCDC11	coiled-coil domain containing 11
100132116	LOC100132116	hypothetical LOC100132116
392382	RPL31P43	ribosomal protein L31 pseudogene 43

80196	RNF34	ring finger protein 34
90990	KIFC2	kinesin family member C2
441795	HMGB3P27	high mobility group box 3 pseudogene 27
9470	EIF4E2	eukaryotic translation initiation factor 4E family member 2
6535	SLC6A8	solute carrier family 6 (neurotransmitter transporter, creatine), member 8
392540	OR11N1P	olfactory receptor, family 11, subfamily N, member 1 pseudogene
120425	AMICA1	adhesion molecule, interacts with CXADR antigen 1
392288	LOC392288	microtubule-associated proteins 1A/1B light chain 3B-like
100129944	[No Symbol]	[No Name]
100129279	RPS27P14	ribosomal protein S27 pseudogene 14
54704	PDP1	pyruvate dehyrogenase phosphatase catalytic subunit 1
339210	C17orf67	chromosome 17 open reading frame 67
8435	SOAT2	sterol O-acyltransferase 2
100130095	[No Symbol]	[No Name]
89958	C9orf140	chromosome 9 open reading frame 140
404201	NCRNA00247	non-protein coding RNA 247
78991	PCYOX1L	prenylcysteine oxidase 1 like
23480	SEC61G	Sec61 gamma subunit
3141	HLCS	holocarboxylase synthetase (biotin-(proprionyl-CoA-carboxylase (ATP-hydrolysing)) ligase)
84848	MGC16121	hypothetical protein MGC16121
26115	TANC2	tetratricopeptide repeat, ankyrin repeat and coiled-coil containing 2
151531	UPP2	uridine phosphorylase 2
255220	TXNDC8	thioredoxin domain containing 8 (spermatozoa)
3872	KRT17	keratin 17
100130183	RPS24P19	ribosomal protein S24 pseudogene 19
7299	TYR	tyrosinase (oculocutaneous albinism IA)
28962	OSTM1	osteopetrosis associated transmembrane protein 1
64174	DPEP2	dipeptidase 2
27443	CECR2	cat eye syndrome chromosome region, candidate 2
51084	CRYL1	crystallin, lambda 1
11030	RBPMS	RNA binding protein with multiple splicing
339967	TMPRSS11A	transmembrane protease, serine 11A
768096	HAR1A	highly accelerated region 1A (non-protein coding)
645054	RPL6P4	ribosomal protein L6 pseudogene 4
4849	CNOT3	CCR4-NOT transcription complex, subunit 3

139422	MAGEB10	melanoma antigen family B, 10
30844	EHD4	EH-domain containing 4
11251	GPR44	G protein-coupled receptor 44
381	ARF5	ADP-ribosylation factor 5
26191	PTPN22	protein tyrosine phosphatase, non-receptor type 22 (lymphoid)
100129238	[No Symbol]	[No Name]
26062	HYALP1	hyaluronoglucosaminidase pseudogene 1
729126	LOC729126	GrpE-like 1, mitochondrial (E. coli) pseudogene
171024	SYNPO2	synaptopodin 2
642762	LOC642762	hCG2042779
1531	CYB5AP3	cytochrome b5 type A (microsomal) pseudogene 3
642496	LOC642496	UDP glucuronosyltransferase 2 family, polypeptide A3 pseudogene
84176	MYH16	myosin, heavy chain 16 pseudogene
83787	ARMC10	armadillo repeat containing 10
143279	HECTD2	HECT domain containing 2
51239	ANKRD39	ankyrin repeat domain 39
4482	MSRA	methionine sulfoxide reductase A
644672	CLDN25	claudin 25
100131595	[No Symbol]	[No Name]
29124	LGALS13	lectin, galactoside-binding, soluble, 13
6860	SYT4	synaptotagmin IV
57509	MTUS1	microtubule associated tumor suppressor 1
127011	LOC127011	ATPase, H+ transporting, lysosomal accessory protein 2 pseudogene
114781	BTBD9	BTB (POZ) domain containing 9
3189	HNRNPH3	heterogeneous nuclear ribonucleoprotein H3 (2H9)
115294	PCMTD1	protein-L-isoaspartate (D-aspartate) O-methyltransferase domain containing 1
171586	ABHD3	abhydrolase domain containing 3
6004	RGS16	regulator of G-protein signaling 16
10950	BTG3	BTG family, member 3
27180	SIGLEC9	sialic acid binding Ig-like lectin 9
993	CDC25A	cell division cycle 25 homolog A (S. pombe)
9886	RHOBTB1	Rho-related BTB domain containing 1
5803	PTPRZ1	protein tyrosine phosphatase, receptor-type, Z polypeptide 1
5782	PTPN12	protein tyrosine phosphatase, non-receptor type 12
64922	LRRC19	leucine rich repeat containing 19

100132537	LOC100132537	dedicator of cytokinesis protein 11-like
100129803	[No Symbol]	[No Name]
4607	MYBPC3	myosin binding protein C, cardiac
100132267	[No Symbol]	[No Name]
339896	GADL1	glutamate decarboxylase-like 1
7225	TRPC6	transient receptor potential cation channel, subfamily C, member 6
85236	HIST1H2BK	histone cluster 1, H2bk
374946	C1orf187	chromosome 1 open reading frame 187
81324	OR4A19P	olfactory receptor, family 4, subfamily A, member 19 pseudogene
135154	C6orf57	chromosome 6 open reading frame 57
1278	COL1A2	collagen, type I, alpha 2
55319	C4orf43	chromosome 4 open reading frame 43
100131251	[No Symbol]	[No Name]
23057	NMNAT2	nicotinamide nucleotide adenylyltransferase 2
54861	SNRK	SNF related kinase
24147	FJX1	four jointed box 1 (Drosophila)
147468	WBP2P1	WW domain binding protein 2 pseudogene 1
100131085	RPL7P12	ribosomal protein L7 pseudogene 12
84947	SERAC1	serine active site containing 1
549	AUH	AU RNA binding protein/enoyl-CoA hydratase
146853	C17orf50	chromosome 17 open reading frame 50
51673	TPPP3	tubulin polymerization-promoting protein family member 3
286103	C8orf77	chromosome 8 open reading frame 77
7089	TLE2	transducin-like enhancer of split 2 (E(sp1) homolog, Drosophila)
285381	DPH3	DPH3, KTI11 homolog (S. cerevisiae)
440955	TMEM89	transmembrane protein 89
391518	VENTXP7	VENT homeobox pseudogene 7
9749	PHACTR2	phosphatase and actin regulator 2
6337	SCNN1A	sodium channel, nonvoltage-gated 1 alpha
388407	C17orf82	chromosome 17 open reading frame 82
400891	LOC400891	chromosome 14 open reading frame 166B pseudogene
3428	IFI16	interferon, gamma-inducible protein 16
222553	SLC35F1	solute carrier family 35, member F1
10753	CAPN9	calpain 9
81616	ACSBG2	acyl-CoA synthetase bubblegum family member 2

127002	ATXN7L2	ataxin 7-like 2
283514	SIAH3	seven in absentia homolog 3 (Drosophila)
100132808	RBMX2P2	RNA binding motif protein, X-linked 2 pseudogene 2
1662	DDX10	DEAD (Asp-Glu-Ala-Asp) box polypeptide 10
5094	PCBP2	poly(rC) binding protein 2
11124	FAF1	Fas (TNFRSF6) associated factor 1
3150	HMGN1	high mobility group nucleosome binding domain 1
128153	SPATA17	spermatogenesis associated 17
154386	C6orf195	chromosome 6 open reading frame 195
1532	CYB5AP4	cytochrome b5 type A (microsomal) pseudogene 4
84734	FAM167B	family with sequence similarity 167, member B
54040	PCBP2P1	poly(rC) binding protein 2 pseudogene 1
2623	GATA1	GATA binding protein 1 (globin transcription factor 1)
401934	RNF223	ring finger protein 223
118856	MMP21	matrix metallopeptidase 21
57674	RNF213	ring finger protein 213
347333	KRT8P14	keratin 8 pseudogene 14
400617	FLJ36644	hypothetical LOC400617
6293	VPS52	vacuolar protein sorting 52 homolog (S. cerevisiae)
100131498	[No Symbol]	[No Name]
129560	LOC129560	adenosylhomocysteinase pseudogene
137107	RPL10AP3	ribosomal protein L10a pseudogene 3
148103	ZNF599	zinc finger protein 599
125228	C18orf19	chromosome 18 open reading frame 19
3566	IL4R	interleukin 4 receptor
727721	LOC727721	hypothetical LOC727721
2173	FABP7	fatty acid binding protein 7, brain
10265	IRX5	iroquois homeobox 5
5912	RAP2B	RAP2B, member of RAS oncogene family
4943	TBC1D25	TBC1 domain family, member 25
645922	S100A7L2	S100 calcium binding protein A7-like 2
55824	PAG1	phosphoprotein associated with glycosphingolipid microdomains 1
150244	ZDHHC8P1	zinc finger, DHHC-type containing 8 pseudogene 1
729832	LOC729832	glutathione S-transferase theta-1-like
407024	MIR29B1	microRNA 29b-1

553148	LOC553148	PAI-1 mRNA binding protein pseudogene
79094	CHAC1	ChaC, cation transport regulator homolog 1 (E. coli)
100131439	CD300LD	CD300 molecule-like family member d
81558	FAM117A	family with sequence similarity 117, member A
84265	POLR3GL	polymerase (RNA) III (DNA directed) polypeptide G (32kD)-like
80336	PABPC1L	poly(A) binding protein, cytoplasmic 1-like
140730	RIMS4	regulating synaptic membrane exocytosis 4
152519	NIPAL1	NIPA-like domain containing 1
124221	PRSS30P	protease, serine, 30 homolog (mouse), pseudogene
1496	CTNNA2	catenin (cadherin-associated protein), alpha 2
79864	C11orf63	chromosome 11 open reading frame 63
100131827	ZNF717	zinc finger protein 717
60509	AGBL5	ATP/GTP binding protein-like 5
100132303	[No Symbol]	[No Name]
151295	SLC23A3	solute carrier family 23 (nucleobase transporters), member 3
4692	NDN	necdin homolog (mouse)
6710	SPTB	spectrin, beta, erythrocytic
5798	PTPRN	protein tyrosine phosphatase, receptor type, N
55146	ZDHHC4	zinc finger, DHHC-type containing 4
4129	MAOB	monoamine oxidase B
11035	RIPK3	receptor-interacting serine-threonine kinase 3
401663	OR51H1P	olfactory receptor, family 51, subfamily H, member 1 pseudogene
9813	KIAA0494	KIAA0494
5770	PTPN1	protein tyrosine phosphatase, non-receptor type 1
3739	KCNA4	potassium voltage-gated channel, shaker-related subfamily, member 4
11022	TDRKH	tudor and KH domain containing
1143	CHRNB4	cholinergic receptor, nicotinic, beta 4
389043	LOC389043	hypothetical LOC389043
54868	TMEM104	transmembrane protein 104
9537	TP53I11	tumor protein p53 inducible protein 11
8648	NCOA1	nuclear receptor coactivator 1
1387	CREBBP	CREB binding protein
138802	OR13C8	olfactory receptor, family 13, subfamily C, member 8
9584	RBM39	RNA binding motif protein 39
3972	LHB	luteinizing hormone beta polypeptide

143	PARP4	poly (ADP-ribose) polymerase family, member 4
164237	WFDC13	WAP four-disulfide core domain 13
100132486	[No Symbol]	[No Name]
1629	DBT	dihydrolipoamide branched chain transacylase E2
2916	GRM6	glutamate receptor, metabotropic 6
100132707	LOC100132707	hypothetical LOC100132707
23113	CUL9	cullin 9
645805	LOC645805	deleted in azoospermia associated protein 2 pseudogene
728682	[No Symbol]	[No Name]
113730	KLHDC7B	kelch domain containing 7B
284648	LOC284648	hypothetical LOC284648
256356	GK5	glycerol kinase 5 (putative)
53353	LRP1B	low density lipoprotein receptor-related protein 1B
8820	HESX1	HESX homeobox 1
100130717	CECR5-AS1	CECR5 antisense RNA 1 (non-protein coding)
81832	NETO1	neuropilin (NRP) and tolloid (TLL)-like 1
57597	BAHCC1	BAH domain and coiled-coil containing 1
100129231	LOC100129231	heat shock 70kDa protein 8 pseudogene
283219	KCTD21	potassium channel tetramerisation domain containing 21
728780	ANKDD1B	ankyrin repeat and death domain containing 1B
56882	CDC42SE1	CDC42 small effector 1
642631	TSPY15P	testis specific protein, Y-linked 15, pseudogene
100128232	[No Symbol]	[No Name]
84532	ACSS1	acyl-CoA synthetase short-chain family member 1
27348	TOR1B	torsin family 1, member B (torsin B)
119	ADD2	adducin 2 (beta)
100128601	LOC100128601	zinc finger protein 770 pseudogene
400866	NCRNA00114	non-protein coding RNA 114
221400	TDRD6	tudor domain containing 6
1058	CENPA	centromere protein A
58472	SQRDL	sulfide quinone reductase-like (yeast)
100130613	CXorf64	chromosome X open reading frame 64
10415	SNAI1P1	snail homolog 1 (Drosophila) pseudogene 1
4899	NRF1	nuclear respiratory factor 1
401409	RAB19	RAB19, member RAS oncogene family

9423	NTN1	netrin 1
54544	CRCT1	cysteine-rich C-terminal 1
83891	SNX25	sorting nexin 25
83692	CD99L2	CD99 molecule-like 2
344022	NOTO	notochord homeobox
554210	MIR429	microRNA 429
25970	SH2B1	SH2B adaptor protein 1
51023	MRPS18C	mitochondrial ribosomal protein S18C
337979	KRTAP22-1	keratin associated protein 22-1
55201	MAP1S	microtubule-associated protein 1S
163126	EID2	EP300 interacting inhibitor of differentiation 2
100129163	[No Symbol]	[No Name]
60682	SMAP1	small ArfGAP 1
100133225	LOC100133225	zinc finger, DHHC-type containing 7 pseudogene
9223	MAGI1	membrane associated guanylate kinase, WW and PDZ domain containing 1
647109	[No Symbol]	[No Name]
453	ASS1P8	argininosuccinate synthetase 1 pseudogene 8
100130219	LOC100130219	hypothetical LOC100130219
1666	DECR1	2,4-dienoyl CoA reductase 1, mitochondrial
195977	ANTXRL	anthrax toxin receptor-like
114569	MAL2	mal, T-cell differentiation protein 2 (gene/pseudogene)
51642	MRPL48	mitochondrial ribosomal protein L48
4241	MFI2	antigen p97 (melanoma associated) identified by monoclonal antibodies 133.2 and 96.5
5118	PCOLCE	procollagen C-endopeptidase enhancer
4839	NOP2	NOP2 nucleolar protein homolog (yeast)
140886	PABPC5	poly(A) binding protein, cytoplasmic 5
57019	CIAPIN1	cytokine induced apoptosis inhibitor 1
8744	TNFSF9	tumor necrosis factor (ligand) superfamily, member 9
401242	LOC401242	hypothetical LOC401242
23566	LPAR3	lysophosphatidic acid receptor 3
11017	SNRNP27	small nuclear ribonucleoprotein 27kDa (U4/U6.U5)
100133097	LOC100133097	hypothetical LOC100133097
200403	VWA3B	von Willebrand factor A domain containing 3B
100131801	LOC100131801	hypothetical protein LOC100131801
26137	ZBTB20	zinc finger and BTB domain containing 20

149650	FLJ32154	hypothetical protein FLJ32154
221476	PI16	peptidase inhibitor 16
284998	LOC284998	hypothetical LOC284998
54503	ZDHHC13	zinc finger, DHHC-type containing 13
57451	ODZ2	odz, odd Oz/ten-m homolog 2 (Drosophila)
10244	RABEPK	Rab9 effector protein with kelch motifs
57795	FAM5B	family with sequence similarity 5, member B
23063	WAPAL	wings apart-like homolog (Drosophila)
5828	PEX2	peroxisomal biogenesis factor 2
51106	TFB1M	transcription factor B1, mitochondrial
100133095	[No Symbol]	[No Name]
284313	[No Symbol]	[No Name]
54536	EXOC6	exocyst complex component 6
78996	C7orf49	chromosome 7 open reading frame 49
391681	LOC391681	exocyst complex component 7 pseudogene
4047	LSS	lanosterol synthase (2,3-oxidosqualene-lanosterol cyclase)
441487	LOC441487	methyltransferase like 1 pseudogene
2357	FPR1	formyl peptide receptor 1
353116	RILPL1	Rab interacting lysosomal protein-like 1
100131240	[No Symbol]	[No Name]
1909	EDNRA	endothelin receptor type A
10485	C1orf61	chromosome 1 open reading frame 61
81159	OR9G3P	olfactory receptor, family 9, subfamily G, member 3 pseudogene
200317	FLJ23865	hypothetical protein FLJ23865
100129438	ZFRP1	zinc finger RNA binding protein pseudogene 1
133923	ZNF474	zinc finger protein 474
9350	CER1	cerberus 1, cysteine knot superfamily, homolog (Xenopus laevis)
2665	GDI2	GDP dissociation inhibitor 2
65999	LRRC61	leucine rich repeat containing 61
6998	TDGF3	teratocarcinoma-derived growth factor 3, pseudogene
10509	SEMA4B	sema domain, immunoglobulin domain (Ig), transmembrane domain (TM) and short cytoplasmic domain, (semaphorin) 4B
729794	[No Symbol]	[No Name]
100129716	LOC100129716	hypothetical LOC100129716
91948	LOC91948	hypothetical LOC91948
9167	COX7A2L	cytochrome c oxidase subunit VIIa polypeptide 2 like

100130315	[No Symbol]	[No Name]
10462	CLEC10A	C-type lectin domain family 10, member A
641522	[No Symbol]	[No Name]
7345	UCHL1	ubiquitin carboxyl-terminal esterase L1 (ubiquitin thiolesterase)
644578	LOC644578	hypothetical protein LOC644578
100128395	[No Symbol]	[No Name]
2052	EPHX1	epoxide hydrolase 1, microsomal (xenobiotic)
1445	CSK	c-src tyrosine kinase
285415	MRPL42P1	mitochondrial ribosomal protein L42 pseudogene 1
57583	TMEM181	transmembrane protein 181
64689	GORASP1	golgi reassembly stacking protein 1, 65kDa
9860	LRIG2	leucine-rich repeats and immunoglobulin-like domains 2
84324	SARNP	SAP domain containing ribonucleoprotein
6856	SYPL1	synaptophysin-like 1
116236	ABHD15	abhydrolase domain containing 15
81264	OR52E7P	olfactory receptor, family 52, subfamily E, member 7 pseudogene
10008	KCNE3	potassium voltage-gated channel, Isk-related family, member 3
203111	C8orf47	chromosome 8 open reading frame 47
2677	GGCX	gamma-glutamyl carboxylase
168002	DACT2	dapper, antagonist of beta-catenin, homolog 2 (Xenopus laevis)
284116	KRT42P	keratin 42 pseudogene
54476	RNF216	ring finger protein 216
100128081	LOC100128081	hypothetical LOC100128081
4154	MBNL1	muscleblind-like (Drosophila)
29085	PHPT1	phosphohistidine phosphatase 1
100131530	LOC100131530	hypothetical LOC100131530
266673	ZNF402P	zinc finger protein 402, pseudogene
100132126	LOC100132126	mitochondrial carrier homolog 2 pseudogene
8596	OR4A1P	olfactory receptor, family 4, subfamily A, member 1 pseudogene
100130155	LOC100130155	hypothetical LOC100130155
100129218	[No Symbol]	[No Name]
728927	ZNF736	zinc finger protein 736
643865	KRT126P	keratin 126 pseudogene
222643	UNC5CL	unc-5 homolog C (C. elegans)-like
286480	UBE2E4P	ubiquitin-conjugating enzyme E2E 4 pseudogene

131961	IMPDH1P8	IMP (inosine monophosphate) dehydrogenase 1 pseudogene 8
7321	UBE2D1	ubiquitin-conjugating enzyme E2D 1
100129753	[No Symbol]	[No Name]
643168	LOC643168	Rho GTPase activating protein 5 pseudogene
729566	LOC729566	protein archease-like
644681	LOC644681	synovial sarcoma, X breakpoint 2 interacting protein pseudogene
10234	LRRC17	leucine rich repeat containing 17
54769	DIRAS2	DIRAS family, GTP-binding RAS-like 2
55656	INTS8	integrator complex subunit 8
55346	TCP11L1	t-complex 11 (mouse)-like 1
6620	SNCB	synuclein, beta
100129876	[No Symbol]	[No Name]
727982	LOC727982	hypothetical LOC727982
100131740	LOC100131740	corepressor interacting with RBPJ, 1 pseudogene
5868	RAB5A	RAB5A, member RAS oncogene family
285877	POM121L12	POM121 membrane glycoprotein-like 12
6594	SMARCA1	SWI/SNF related, matrix associated, actin dependent regulator of chromatin, subfamily a, member 1
163688	CALML6	calmodulin-like 6
645478	[No Symbol]	[No Name]
204219	LASS3	LAG1 homolog, ceramide synthase 3
119392	MEIR5	MEI5 meiotic recombination protein homolog (S. cerevisiae)
128718	RPS11P1	ribosomal protein S11 pseudogene 1
391470	LOC391470	adenosylhomocysteinase pseudogene
3977	LIFR	leukemia inhibitory factor receptor alpha
100129583	FAM47E	family with sequence similarity 47, member E
449491	DEFA8P	defensin, alpha 8 pseudogene
93594	WDR67	WD repeat domain 67
407040	MIR34A	microRNA 34a
100131370	DDX18P6	DEAD (Asp-Glu-Ala-Asp) box polypeptide 18 pseudogene 6
84727	SPSB2	splA/ryanodine receptor domain and SOCS box containing 2
5650	KLK7	kallikrein-related peptidase 7
80100	[No Symbol]	[No Name]
8382	NME5	non-metastatic cells 5, protein expressed in (nucleoside-diphosphate kinase)
100128413	LOC100128413	X-linked inhibitor of apoptosis pseudogene
100129135	LOC100129135	selenocysteine insertion sequence-binding protein 2-like

84671	ZNF347	zinc finger protein 347
730184	LOC730184	hypothetical LOC730184
79753	SNIP1	Smad nuclear interacting protein 1
652411	RPSAP44	ribosomal protein SA pseudogene 44
359948	IRF2BP2	interferon regulatory factor 2 binding protein 2
1687	DFNA5	deafness, autosomal dominant 5
9453	GGPS1	geranylgeranyl diphosphate synthase 1
51617	HMP19	HMP19 protein
80146	UXS1	UDP-glucuronate decarboxylase 1
79009	DDX50	DEAD (Asp-Glu-Ala-Asp) box polypeptide 50
253017	TECRL	trans-2,3-enoyl-CoA reductase-like
400934	FLJ44385	hypothetical FLJ44385
100131073	[No Symbol]	[No Name]
390313	OR5BS1P	olfactory receptor, family 5, subfamily BS, member 1 pseudogene
8840	WISP1	WNT1 inducible signaling pathway protein 1
3833	KIFC1	kinesin family member C1
114192	SIGLEC20P	sialic acid binding Ig-like lectin 20, pseudogene
57619	SHROOM3	shroom family member 3
23616	SH3BP1	SH3-domain binding protein 1
11129	CLASRP	CLK4-associating serine/arginine rich protein
100130084	[No Symbol]	[No Name]
5062	PAK2	p21 protein (Cdc42/Rac)-activated kinase 2
1296	COL8A2	collagen, type VIII, alpha 2
441887	LOC441887	DnaJ (Hsp40) homolog, subfamily C, member 7 pseudogene
100131893	RPL3P11	ribosomal protein L3 pseudogene 11
5881	RAC3	ras-related C3 botulinum toxin substrate 3 (rho family, small GTP binding protein Rac3)
1555	CYP2B6	cytochrome P450, family 2, subfamily B, polypeptide 6
29035	C16orf72	chromosome 16 open reading frame 72
374986	FAM73A	family with sequence similarity 73, member A
126433	FBXO27	F-box protein 27
22999	RIMS1	regulating synaptic membrane exocytosis 1
391073	LOC391073	glyceraldehyde-3-phosphate dehydrogenase pseudogene
3857	KRT9	keratin 9
130802	DNMT3AP1	DNA methyltransferase 3A pseudogene 1
155435	RBM33	RNA binding motif protein 33

201633	TIGIT	T cell immunoreceptor with Ig and ITIM domains
55214	LEPREL1	leprecan-like 1
1141	CHRNB2	cholinergic receptor, nicotinic, beta 2 (neuronal)
219858	OR8B12	olfactory receptor, family 8, subfamily B, member 12
100128870	[No Symbol]	[No Name]
100129814	[No Symbol]	[No Name]
100129007	FMO8P	flavin containing monooxygenase 8 pseudogene
391117	OR10J2P	olfactory receptor, family 10, subfamily J, member 2 pseudogene
100133007	LOC100133007	DENN/MADD domain containing 1C pseudogene
6506	SLC1A2	solute carrier family 1 (glial high affinity glutamate transporter), member 2
9922	IQSEC1	IQ motif and Sec7 domain 1
100133252	LOC100133252	zinc finger protein 131 pseudogene
100129271	C1orf68	chromosome 1 open reading frame 68
730201	[No Symbol]	[No Name]
7545	ZIC1	Zic family member 1
2151	F2RL2	coagulation factor II (thrombin) receptor-like 2
284417	TMEM150B	transmembrane protein 150B
23262	PPIP5K2	diphosphoinositol pentakisphosphate kinase 2
83606	C22orf13	chromosome 22 open reading frame 13
3680	ITGA9	integrin, alpha 9
641765	[No Symbol]	[No Name]
84498	FAM120B	family with sequence similarity 120B
118813	ZFYVE27	zinc finger, FYVE domain containing 27
390327	OR6C70	olfactory receptor, family 6, subfamily C, member 70
100130776	LOC100130776	hypothetical LOC100130776
339500	ZNF678	zinc finger protein 678
389376	SFTA2	surfactant associated 2
5077	PAX3	paired box 3
3788	KCNS2	potassium voltage-gated channel, delayed-rectifier, subfamily S, member 2
7531	YWHAE	tyrosine 3-monooxygenase/tryptophan 5-monooxygenase activation protein, epsilon polypeptide
80139	ZNF703	zinc finger protein 703
4155	MBP	myelin basic protein
100129692	EIF4BP9	eukaryotic translation initiation factor 4B pseudogene 9
23746	AIPL1	aryl hydrocarbon receptor interacting protein-like 1
54569	UGT2B27P	UDP glucuronosyltransferase 2 family, polypeptide B27 pseudogene

9400	RECQL5	RecQ protein-like 5
642864	SRG7	spermatogenesis-related protein 7
2732	GLDCP1	glycine dehydrogenase (decarboxylase) pseudogene 1
401638	RPSAP10	ribosomal protein SA pseudogene 10
100128993	LOC100128993	similar to hCG2036572
100130968	RPL7L1P4	ribosomal protein L7-like 1 pseudogene 4
7115	TMSB4XP1	thymosin beta 4, X-linked pseudogene 1
29951	PDZRN4	PDZ domain containing ring finger 4
79300	OR51P1P	olfactory receptor, family 51, subfamily P, member 1 pseudogene
347732	CATSPER3	cation channel, sperm associated 3
100129053	LOC100129053	lysine (K)-specific demethylase 4D-like pseudogene
399947	C11orf87	chromosome 11 open reading frame 87
22834	ZNF652	zinc finger protein 652
100129773	[No Symbol]	[No Name]
730094	C16orf52	chromosome 16 open reading frame 52
8621	CDK13	cyclin-dependent kinase 13
9750	FAM65B	family with sequence similarity 65, member B
133688	UGT3A1	UDP glycosyltransferase 3 family, polypeptide A1
100132795	RPL12P32	ribosomal protein L12 pseudogene 32
4855	NOTCH4	notch 4
114876	OSBPL1A	oxysterol binding protein-like 1A
22885	ABLIM3	actin binding LIM protein family, member 3
64432	MRPS25	mitochondrial ribosomal protein S25
4838	NODAL	nodal homolog (mouse)
79550	OR4C4P	olfactory receptor, family 4, subfamily C, member 4 pseudogene
100128902	PRDX3P3	peroxiredoxin 3 pseudogene 3
9121	SLC16A5	solute carrier family 16, member 5 (monocarboxylic acid transporter 6)
286544	MXRA5P1	matrix-remodelling associated 5 pseudogene 1
64005	MYO1G	myosin IG
337976	KRTAP20-2	keratin associated protein 20-2
85378	TUBGCP6	tubulin, gamma complex associated protein 6
84938	ATG4C	ATG4 autophagy related 4 homolog C (S. cerevisiae)
441899	LOC441899	vacuolar protein sorting 25 homolog (S. cerevisiae) pseudogene
5573	PRKAR1A	protein kinase, cAMP-dependent, regulatory, type I, alpha (tissue specific extinguisher 1)
26493	OR8B8	olfactory receptor, family 8, subfamily B, member 8

100129348	LOC100129348	cell division cycle associated 4 pseudogene
9411	ARHGAP29	Rho GTPase activating protein 29
197358	NLRC3	NLR family, CARD domain containing 3
22872	SEC31A	SEC31 homolog A (S. cerevisiae)
283189	OR9G4	olfactory receptor, family 9, subfamily G, member 4
55268	ECHDC2	enoyl CoA hydratase domain containing 2
22926	ATF6	activating transcription factor 6
55626	AMBRA1	autophagy/beclin-1 regulator 1
80316	PPP1R2P9	protein phosphatase 1, regulatory (inhibitor) subunit 2 pseudogene 9
51742	ARID4B	AT rich interactive domain 4B (RBP1-like)
8440	NCK2	NCK adaptor protein 2
3065	HDAC1	histone deacetylase 1
3981	LIG4	ligase IV, DNA, ATP-dependent
729662	RPL22P3	ribosomal protein L22 pseudogene 3
5364	PLXNB1	plexin B1
9064	MAP3K6	mitogen-activated protein kinase kinase kinase 6
643941	PHKG1P1	phosphorylase kinase, gamma 1 pseudogene 1
80853	JHDM1D	jumonji C domain containing histone demethylase 1 homolog D (S. cerevisiae)
79940	C6orf155	chromosome 6 open reading frame 155
150696	PROM2	prominin 2
8106	PABPN1	poly(A) binding protein, nuclear 1
25973	PARS2	prolyl-tRNA synthetase 2, mitochondrial (putative)
57666	FBRSL1	fibrosin-like 1
58538	MPP4	membrane protein, palmitoylated 4 (MAGUK p55 subfamily member 4)
55716	LMBR1L	limb region 1 homolog (mouse)-like
84260	TCHP	trichoplein, keratin filament binding
2890	GRIA1	glutamate receptor, ionotropic, AMPA 1
100128153	[No Symbol]	[No Name]
10159	ATP6AP2	ATPase, H+ transporting, lysosomal accessory protein 2
84447	SYVN1	synovial apoptosis inhibitor 1, synoviolin
84765	ZNF577	zinc finger protein 577
79168	LILRA6	leukocyte immunoglobulin-like receptor, subfamily A (with TM domain), member 6
494329	MIR380	microRNA 380
729911	LOC729911	hypothetical LOC729911
100128554	LOC100128554	hypothetical LOC100128554

124872	B4GALNT2	beta-1,4-N-acetyl-galactosaminyl transferase 2
84168	ANTXR1	anthrax toxin receptor 1
219436	OR5D14	olfactory receptor, family 5, subfamily D, member 14
139425	DCAF8L1	DDB1 and CUL4 associated factor 8-like 1
9789	SPCS2	signal peptidase complex subunit 2 homolog (S. cerevisiae)
23557	SNAPIN	SNAP-associated protein
100133296	[No Symbol]	[No Name]
3324	HSP90AA2	heat shock protein 90kDa alpha (cytosolic), class A member 2
646187	[No Symbol]	[No Name]
79872	CBLL1	Cas-Br-M (murine) ecotropic retroviral transforming sequence-like 1
56994	CHPT1	choline phosphotransferase 1
145270	PRIMA1	proline rich membrane anchor 1
642819	ZNF487P	zinc finger protein 487, pseudogene
10237	SLC35B1	solute carrier family 35, member B1
100129856	RPL37AP5	ribosomal protein L37a pseudogene 5
23099	ZBTB43	zinc finger and BTB domain containing 43
100130142	[No Symbol]	[No Name]
2261	FGFR3	fibroblast growth factor receptor 3
1525	CXADR	coxsackie virus and adenovirus receptor
92482	BBIP1	BBSome interacting protein 1
2128	EVX1	even-skipped homeobox 1
55366	LGR4	leucine-rich repeat containing G protein-coupled receptor 4
28514	DLL1	delta-like 1 (Drosophila)
26523	EIF2C1	eukaryotic translation initiation factor 2C, 1
9245	GCNT3	glucosaminyl (N-acetyl) transferase 3, mucin type
5265	SERPINA1	serpin peptidase inhibitor, clade A (alpha-1 antiproteinase, antitrypsin), member 1
375056	MIA3	melanoma inhibitory activity family, member 3
337966	KRTAP6-1	keratin associated protein 6-1
389558	FAM180A	family with sequence similarity 180, member A
57492	ARID1B	AT rich interactive domain 1B (SWI1-like)
440072	NCRNA00167	non-protein coding RNA 167
23171	GPD1L	glycerol-3-phosphate dehydrogenase 1-like
6955	TRA@	T cell receptor alpha locus
100129955	LOC100129955	RNA polymerase I-specific transcription initiation factor RRN3-like
84418	C5orf32	chromosome 5 open reading frame 32

613227	HIGD1C	HIG1 hypoxia inducible domain family, member 1C
5688	PSMA7	proteasome (prosome, macropain) subunit, alpha type, 7
9232	PTTG1	pituitary tumor-transforming 1
1061	CENPC1P1	centromere protein C 1 pseudogene 1
10644	IGF2BP2	insulin-like growth factor 2 mRNA binding protein 2
100129456	LOC100129456	heat shock protein 90kDa alpha (cytosolic), class B member 1 pseudogene
6513	SLC2A1	solute carrier family 2 (facilitated glucose transporter), member 1
222584	FAM83B	family with sequence similarity 83, member B
100128613	[No Symbol]	[No Name]
57634	EP400	E1A binding protein p400
100133303	[No Symbol]	[No Name]
54541	DDIT4	DNA-damage-inducible transcript 4
728147	LOC728147	hypothetical protein LOC728147
146057	TTBK2	tau tubulin kinase 2
1374	CPT1A	carnitine palmitoyltransferase 1A (liver)
114827	FHAD1	forkhead-associated (FHA) phosphopeptide binding domain 1
646625	PRHOXNB	parahox cluster neighbor
91	ACVR1B	activin A receptor, type IB
147929	ZNF565	zinc finger protein 565
133083	LOC133083	peptidase (mitochondrial processing) alpha pseudogene
649095	[No Symbol]	[No Name]
55805	LRP2BP	LRP2 binding protein
100127978	LOC100127978	starch binding domain 1 pseudogene
399978	[No Symbol]	[No Name]
83597	RTP3	receptor (chemosensory) transporter protein 3
5104	SERPINA5	serpin peptidase inhibitor, clade A (alpha-1 antiproteinase, antitrypsin), member 5
119369	NUDT9P1	nudix (nucleoside diphosphate linked moiety X)-type motif 9 pseudogene 1
4091	SMAD6	SMAD family member 6
346562	GNAT3	guanine nucleotide binding protein, alpha transducing 3
10318	TNIP1	TNFAIP3 interacting protein 1
100130031	[No Symbol]	[No Name]
51465	UBE2J1	ubiquitin-conjugating enzyme E2, J1, U
5489	PPIAP7	peptidylprolyl isomerase A (cyclophilin A) pseudogene 7
142913	CFL1P1	cofilin 1 (non-muscle) pseudogene 1
245930	DEFB116	defensin, beta 116

5212	VIT	vitrin
8531	CSDA	cold shock domain protein A
4147	MATN2	matrilin 2
344892	RTP2	receptor (chemosensory) transporter protein 2
140565	[No Symbol]	[No Name]
100132779	[No Symbol]	[No Name]
127795	C1orf87	chromosome 1 open reading frame 87
23678	SGK3	serum/glucocorticoid regulated kinase family, member 3
120796	OR56A1	olfactory receptor, family 56, subfamily A, member 1
730173	[No Symbol]	[No Name]
100127949	RPL15P16	ribosomal protein L15 pseudogene 16
326337	SSXP4	SSX family pseudogene 4
117248	GALNTL2	UDP-N-acetyl-alpha-D-galactosamine:polypeptide N-acetylgalactosaminyltransferase-like 2
3280	HES1	hairy and enhancer of split 1, (Drosophila)
80011	FAM192A	family with sequence similarity 192, member A
5701	PSMC2	proteasome (prosome, macropain) 26S subunit, ATPase, 2
10324	KBTBD10	kelch repeat and BTB (POZ) domain containing 10
22824	HSPA4L	heat shock 70kDa protein 4-like
219473	OR8K3	olfactory receptor, family 8, subfamily K, member 3
11148	HHLA2	HERV-H LTR-associating 2
6015	RING1	ring finger protein 1
9690	UBE3C	ubiquitin protein ligase E3C
4990	SIX6	SIX homeobox 6
260340	TFAMP2	transcription factor A, mitochondrial pseudogene 2
613209	DEFB135	defensin, beta 135
100132992	HMGN1P30	high mobility group nucleosome binding domain 1 pseudogene 30
64710	NUCKS1	nuclear casein kinase and cyclin-dependent kinase substrate 1
245802	MS4A6E	membrane-spanning 4-domains, subfamily A, member 6E
81839	VANGL1	vang-like 1 (van gogh, Drosophila)
147699	PPM1N	protein phosphatase, Mg2+/Mn2+ dependent, 1N (putative)
63948	DMRTB1	DMRT-like family B with proline-rich C-terminal, 1
64968	MRPS6	mitochondrial ribosomal protein S6
646535	LOC646535	60S ribosomal protein L7a-like
2653	GCSH	glycine cleavage system protein H (aminomethyl carrier)
79958	DENND1C	DENN/MADD domain containing 1C

100128062	SLC2A3P1	solute carrier family 2 (facilitated glucose transporter), member 3 pseudogene 1
83939	EIF2A	eukaryotic translation initiation factor 2A, 65kDa
728294	D2HGDH	D-2-hydroxyglutarate dehydrogenase
79508	OR5M7P	olfactory receptor, family 5, subfamily M, member 7 pseudogene
57821	C1orf114	chromosome 1 open reading frame 114
810	CALML3	calmodulin-like 3
5458	POU4F2	POU class 4 homeobox 2
6720	SREBF1	sterol regulatory element binding transcription factor 1
4712	NDUFB6	NADH dehydrogenase (ubiquinone) 1 beta subcomplex, 6, 17kDa
140803	TRPM6	transient receptor potential cation channel, subfamily M, member 6
51011	FAHD2A	fumarylacetoacetate hydrolase domain containing 2A
643015	LOC643015	nucleolar protein 11 pseudogene
208	AKT2	v-akt murine thymoma viral oncogene homolog 2
2040	STOM	stomatin
23043	TNIK	TRAF2 and NCK interacting kinase
100128997	LOC100128997	hypothetical LOC100128997
401825	LOC401825	MKI67 (FHA domain) interacting nucleolar phosphoprotein pseudogene
440311	LOC440311	glioma tumor suppressor candidate region gene 2 pseudogene
7371	UCK2	uridine-cytidine kinase 2
199699	DAND5	DAN domain family, member 5
644584	LOC644584	Ewing sarcoma breakpoint region 1 pseudogene
55573	CDV3	CDV3 homolog (mouse)
730144	LOC730144	eukaryotic translation initiation factor 1 pseudogene
10466	COG5	component of oligomeric golgi complex 5
55215	FANCI	Fanconi anemia, complementation group I
283902	HTA	hypothetical LOC283902
2243	FGA	fibrinogen alpha chain
54873	PALMD	palmdelphin
284161	GDPD1	glycerophosphodiester phosphodiesterase domain containing 1
100126477	TRNAL-AAG	transfer RNA leucine (anticodon AAG)
51663	ZFR	zinc finger RNA binding protein
285489	DOK7	docking protein 7
81295	OR51A10P	olfactory receptor, family 51, subfamily A, member 10 pseudogene
100132424	[No Symbol]	[No Name]
84769	MPV17L2	MPV17 mitochondrial membrane protein-like 2

79676	OGFOD2	2-oxoglutarate and iron-dependent oxygenase domain containing 2
22848	AAK1	AP2 associated kinase 1
5049	PAFAH1B2	platelet-activating factor acetylhydrolase 1b, catalytic subunit 2 (30kDa)
100131036	HMGB3P28	high mobility group box 3 pseudogene 28
57052	[No Symbol]	[No Name]
79679	VTCN1	V-set domain containing T cell activation inhibitor 1
26005	C2CD3	C2 calcium-dependent domain containing 3
147429	CHST9-AS1	CHST9 antisense RNA 1 (non-protein coding)
285971	ZNF775	zinc finger protein 775
646962	HRCT1	histidine rich carboxyl terminus 1
10456	HAX1	HCLS1 associated protein X-1
100129816	[No Symbol]	[No Name]
50863	NTM	neurotrimin
10389	SCML2	sex comb on midleg-like 2 (Drosophila)
58528	RRAGD	Ras-related GTP binding D
91949	COG7	component of oligomeric golgi complex 7
5519	PPP2R1B	protein phosphatase 2, regulatory subunit A, beta
23205	ACSBG1	acyl-CoA synthetase bubblegum family member 1
51087	YBX2	Y box binding protein 2
1720	LOC1720	dihydrofolate reductase pseudogene
1758	DMP1	dentin matrix acidic phosphoprotein 1
283417	DPY19L2	dpy-19-like 2 (C. elegans)
326614	PEBP1P1	phosphatidylethanolamine binding protein 1 pseudogene 1
23732	C9orf4	chromosome 9 open reading frame 4
130576	LYPD6B	LY6/PLAUR domain containing 6B
645973	ATP5F1P2	ATP synthase, H+ transporting, mitochondrial Fo complex, subunit B1 pseudogene 2
51802	ACCN5	amiloride-sensitive cation channel 5, intestinal
806	CALM2P2	calmodulin 2 pseudogene 2
51205	ACP6	acid phosphatase 6, lysophosphatidic
100128335	LOC100128335	WD repeat domain 12 pseudogene
9629	CLCA3P	chloride channel accessory 3, pseudogene
3008	HIST1H1E	histone cluster 1, H1e
387758	FIBIN	fin bud initiation factor homolog (zebrafish)
150379	PNPLA5	patatin-like phospholipase domain containing 5
253820	[No Symbol]	[No Name]

339453	C1orf70	chromosome 1 open reading frame 70
79974	C7orf58	chromosome 7 open reading frame 58
387748	OR56B1	olfactory receptor, family 56, subfamily B, member 1
114800	CCDC85A	coiled-coil domain containing 85A
730495	LOC730495	hypothetical protein LOC730495
155185	AMZ1	archaelysin family metallopeptidase 1
388523	ZNF728	zinc finger protein 728
8214	DGCR6	DiGeorge syndrome critical region gene 6
391589	KRT18P34	keratin 18 pseudogene 34
391462	RPSAP24	ribosomal protein SA pseudogene 24
55742	PARVA	parvin, alpha
1266	CNN3	calponin 3, acidic
51277	DNAJC27	DnaJ (Hsp40) homolog, subfamily C, member 27
3791	KDR	kinase insert domain receptor (a type III receptor tyrosine kinase)
284723	SLC25A34	solute carrier family 25, member 34
715	C1R	complement component 1, r subcomponent
145645	C15orf43	chromosome 15 open reading frame 43
54490	UGT2B28	UDP glucuronosyltransferase 2 family, polypeptide B28
79971	WLS	wntless homolog (Drosophila)
729589	[No Symbol]	[No Name]
283450	C12orf51	chromosome 12 open reading frame 51
344595	LOC344595	hypothetical LOC344595
390294	LOC390294	heterogeneous nuclear ribonucleoprotein A/B pseudogene
286097	EFHA2	EF-hand domain family, member A2
164312	LRRN4	leucine rich repeat neuronal 4
10476	ATP5H	ATP synthase, H+ transporting, mitochondrial Fo complex, subunit d
100129461	LOC100129461	hypothetical LOC100129461
7652	ZNF99	zinc finger protein 99
7697	ZNF138	zinc finger protein 138
220974	OR6D1P	olfactory receptor, family 6, subfamily D, member 1 pseudogene
402422	LOC402422	tubulin, beta 4 pseudogene
166070	DDX50P2	DEAD/H (Asp-Glu-Ala-Asp/His) box polypeptide 50 pseudogene 2
777	CACNA1E	calcium channel, voltage-dependent, R type, alpha 1E subunit
5453	POU3F1	POU class 3 homeobox 1
7307	U2AF1	U2 small nuclear RNA auxiliary factor 1

84466	MEGF10	multiple EGF-like-domains 10
9238	TBRG4	transforming growth factor beta regulator 4
5335	PLCG1	phospholipase C, gamma 1
26980	PABPC1P1	poly(A) binding protein, cytoplasmic 1 pseudogene 1
100132440	[No Symbol]	[No Name]
78990	OTUB2	OTU domain, ubiquitin aldehyde binding 2
9941	EXOG	endo/exonuclease (5'-3'), endonuclease G-like
285643	KIF4B	kinesin family member 4B
57282	SLC4A10	solute carrier family 4, sodium bicarbonate transporter, member 10
246184	CDC26	cell division cycle 26 homolog (S. cerevisiae)
55195	C14orf105	chromosome 14 open reading frame 105
5783	PTPN13	protein tyrosine phosphatase, non-receptor type 13 (APO-1/CD95 (Fas)-associated phosphatase)
2185	PTK2B	PTK2B protein tyrosine kinase 2 beta
100131418	RPS27P22	ribosomal protein S27 pseudogene 22
90693	CCDC126	coiled-coil domain containing 126
5566	PRKACA	protein kinase, cAMP-dependent, catalytic, alpha
100132137	[No Symbol]	[No Name]
100131034	LOC100131034	hypothetical protein LOC100131034
284612	SYPL2	synaptophysin-like 2
389493	LOC389493	hypothetical protein LOC389493
4744	NEFH	neurofilament, heavy polypeptide
653588	LOC653588	Sjogren syndrome antigen B (autoantigen La) pseudogene
1454	CSNK1E	casein kinase 1, epsilon
100130618	LOC100130618	tetratricopeptide repeat domain 14 pseudogene
645474	S100A11P3	S100 calcium binding protein A11 pseudogene 3
10345	TRDN	triadin
220885	RPSAP15	ribosomal protein SA pseudogene 15
100128641	[No Symbol]	[No Name]
26232	FBXO2	F-box protein 2
673	BRAF	v-raf murine sarcoma viral oncogene homolog B1
228	ALDOAP2	aldolase A, fructose-bisphosphate pseudogene 2
340260	UNCX	UNC homeobox
6401	SELE	selectin E
26122	EPC2	enhancer of polycomb homolog 2 (Drosophila)
728716	LOC728716	hypothetical LOC728716

57617	VPS18	vacuolar protein sorting 18 homolog (S. cerevisiae)
100131056	[No Symbol]	[No Name]
85403	EAF1	ELL associated factor 1
100131772	[No Symbol]	[No Name]
730124	[No Symbol]	[No Name]
10236	HNRNPR	heterogeneous nuclear ribonucleoprotein R
124975	GGT6	gamma-glutamyltransferase 6
399717	FLJ45983	hypothetical LOC399717
26088	GGA1	golgi-associated, gamma adaptin ear containing, ARF binding protein 1
219875	OR4D5	olfactory receptor, family 4, subfamily D, member 5
64924	SLC30A5	solute carrier family 30 (zinc transporter), member 5
85026	C9orf37	chromosome 9 open reading frame 37
100131990	LOC100131990	similar to hCG1660378
2257	FGF12	fibroblast growth factor 12
25924	MYRIP	myosin VIIA and Rab interacting protein
2100	ESR2	estrogen receptor 2 (ER beta)
391467	IMPDH1P7	IMP (inosine monophosphate) dehydrogenase 1 pseudogene 7
93986	FOXP2	forkhead box P2
388630	LOC388630	UPF0632 protein A
3757	KCNH2	potassium voltage-gated channel, subfamily H (eag-related), member 2
100127986	LOC100127986	cation channel, sperm associated 2 pseudogene
286187	LRRC67	leucine rich repeat containing 67
10743	RAI1	retinoic acid induced 1
200726	LOC200726	hCG1657980
100131157	[No Symbol]	[No Name]
391012	LOC391012	dynein, cytoplasmic, light polypeptide 1 pseudogene
79310	OR5H2	olfactory receptor, family 5, subfamily H, member 2
222236	NAPEPLD	N-acyl phosphatidylethanolamine phospholipase D
8801	SUCLG2	succinate-CoA ligase, GDP-forming, beta subunit
5520	PPP2R2A	protein phosphatase 2, regulatory subunit B, alpha
83943	IMMP2L	IMP2 inner mitochondrial membrane peptidase-like (S. cerevisiae)
272	AMPD3	adenosine monophosphate deaminase 3
100128576	[No Symbol]	[No Name]
90019	SYT8	synaptotagmin VIII
100130431	[No Symbol]	[No Name]

641516	KC6	keratoconus gene 6
80092	[No Symbol]	[No Name]
100132805	[No Symbol]	[No Name]
644753	[No Symbol]	[No Name]
100130578	[No Symbol]	[No Name]
26986	PABPC1	poly(A) binding protein, cytoplasmic 1
144124	OR10A5	olfactory receptor, family 10, subfamily A, member 5
400986	LOC400986	ankyrin repeat domain-containing protein 36C
100132242	LOC100132242	exosome component 8 pseudogene
391587	LOC391587	peptidyl-prolyl cis-trans isomerase A-like
4888	NPY6R	neuropeptide Y receptor Y6 (pseudogene)
6519	SLC3A1	solute carrier family 3 (cystine, dibasic and neutral amino acid transporters, activator of cystine, dibasic and neutral amino acid transport), member 1
730034	[No Symbol]	[No Name]
9980	DOPEY2	dopey family member 2
23654	PLXNB2	plexin B2
55729	ATF7IP	activating transcription factor 7 interacting protein
123207	C15orf40	chromosome 15 open reading frame 40
442664	[No Symbol]	[No Name]
200014	CC2D1B	coiled-coil and C2 domain containing 1B
8686	KRT41P	keratin 41 pseudogene
23576	DDAH1	dimethylarginine dimethylaminohydrolase 1
152579	SCFD2	sec1 family domain containing 2
221264	AKD1	adenylate kinase domain containing 1
100132121	ETF1P3	eukaryotic translation termination factor 1 pseudogene 3
326593	MORF4L1P3	mortality factor 4 like 1 pseudogene 3
221458	KIF6	kinesin family member 6
100126493	TRNAP-AGG	transfer RNA proline (anticodon AGG)
79036	C19orf50	chromosome 19 open reading frame 50
57626	KLHL1	kelch-like 1 (Drosophila)
84343	HPS3	Hermansky-Pudlak syndrome 3
100125393	LOC100125393	TIMELESS interacting protein pseudogene
987	LRBA	LPS-responsive vesicle trafficking, beach and anchor containing
654463	FER1L6	fer-1-like 6 (C. elegans)
163720	CYP4Z2P	cytochrome P450, family 4, subfamily Z, polypeptide 2 pseudogene
83697	SLC4A9	solute carrier family 4, sodium bicarbonate cotransporter, member 9

100128955	[No Symbol]	[No Name]
80835	TAS1R1	taste receptor, type 1, member 1
100130462	HNF4GP1	hepatocyte nuclear factor 4, gamma pseudogene 1
56942	C16orf61	chromosome 16 open reading frame 61
151126	ZNF385B	zinc finger protein 385B
1299	COL9A3	collagen, type IX, alpha 3
140545	RNF32	ring finger protein 32
8739	HRK	harakiri, BCL2 interacting protein (contains only BH3 domain)
140832	WFDC10A	WAP four-disulfide core domain 10A
167410	LIX1	Lix1 homolog (chicken)
100131774	LOC100131774	hypothetical LOC100131774
100126509	TRNAQ-UUG	transfer RNA glutamine (anticodon UUG)
100128570	[No Symbol]	[No Name]
7871	SLMAP	sarcolemma associated protein
1767	DNAH5	dynein, axonemal, heavy chain 5
100131089	LOC100131089	hypothetical LOC100131089
3859	KRT12	keratin 12
56204	KIAA1370	KIAA1370
1469	CST1	cystatin SN
140885	SIRPA	signal-regulatory protein alpha
23620	NTSR2	neurotensin receptor 2
54798	DCHS2	dachsous 2 (Drosophila)
1805	DPT	dermatopontin
4783	NFIL3	nuclear factor, interleukin 3 regulated
646808	LOC646808	L antigen family member 3-like
219970	GLYATL2	glycine-N-acyltransferase-like 2
442086	RPL10P7	ribosomal protein L10 pseudogene 7
339879	LOC339879	asparagine-linked glycosylation 1 homolog pseudogene
677781	SCARNA16	small Cajal body-specific RNA 16
347097	LOC347097	dermatan sulfate epimerase pseudogene
124245	ZC3H18	zinc finger CCCH-type containing 18
100127998	[No Symbol]	[No Name]
6992	PPP1R11	protein phosphatase 1, regulatory (inhibitor) subunit 11
8975	USP13	ubiquitin specific peptidase 13 (isopeptidase T-3)
222255	ATXN7L1	ataxin 7-like 1

10320	IKZF1	IKAROS family zinc finger 1 (Ikaros)
100130363	[No Symbol]	[No Name]
7472	WNT2	wingless-type MMTV integration site family member 2
23192	ATG4B	ATG4 autophagy related 4 homolog B (S. cerevisiae)
6690	SPINK1	serine peptidase inhibitor, Kazal type 1
79577	CDC73	cell division cycle 73, Paf1/RNA polymerase II complex component, homolog (S. cerevisiae)
8745	ADAM23	ADAM metallopeptidase domain 23
92689	FAM114A1	family with sequence similarity 114, member A1
79802	HHIPL2	HHIP-like 2
284688	LOC284688	hypothetical LOC284688
55769	ZNF83	zinc finger protein 83
1791	DNTT	deoxynucleotidyltransferase, terminal
619518	C1orf191	chromosome 1 open reading frame 191
6332	SCN7A	sodium channel, voltage-gated, type VII, alpha
7159	TP53BP2	tumor protein p53 binding protein, 2
55300	PI4K2B	phosphatidylinositol 4-kinase type 2 beta
83661	MS4A8B	membrane-spanning 4-domains, subfamily A, member 8B
83953	FCAMR	Fc receptor, IgA, IgM, high affinity
55252	ASXL2	additional sex combs like 2 (Drosophila)
3097	HIVEP2	human immunodeficiency virus type I enhancer binding protein 2
346711	MRPL49P2	mitochondrial ribosomal protein L49 pseudogene 2
5706	PSMC6	proteasome (prosome, macropain) 26S subunit, ATPase, 6
7373	COL14A1	collagen, type XIV, alpha 1
83989	FAM172A	family with sequence similarity 172, member A
283303	C11orf36	chromosome 11 open reading frame 36
100133124	LOC100133124	cytochrome c oxidase subunit 7B, mitochondrial-like
326343	MT1DP	metallothionein 1D, pseudogene
145757	LOC145757	hypothetical LOC145757
342909	ZNF284	zinc finger protein 284
369	ARAF	v-raf murine sarcoma 3611 viral oncogene homolog
27190	IL17B	interleukin 17B
389337	ARHGEF37	Rho guanine nucleotide exchange factor (GEF) 37
2570	GABRR2	gamma-aminobutyric acid (GABA) receptor, rho 2
79740	ZBBX	zinc finger, B-box domain containing
56171	DNAH7	dynein, axonemal, heavy chain 7

5322	PLA2G5	phospholipase A2, group V
10671	DCTN6	dynactin 6
4188	MDFI	MyoD family inhibitor
112724	RDH13	retinol dehydrogenase 13 (all-trans/9-cis)
100129335	[No Symbol]	[No Name]
656	BMP8B	bone morphogenetic protein 8b
5095	PCCA	propionyl CoA carboxylase, alpha polypeptide
9427	ECEL1	endothelin converting enzyme-like 1
126006	PCP2	Purkinje cell protein 2
100133180	DDX3YP1	DEAD (Asp-Glu-Ala-Asp) box polypeptide 3, Y-linked pseudogene 1
159963	SLC5A12	solute carrier family 5 (sodium/glucose cotransporter), member 12
2492	FSHR	follicle stimulating hormone receptor
51088	KLHL5	kelch-like 5 (Drosophila)
8525	DGKZ	diacylglycerol kinase, zeta
10609	LEPREL4	leprecan-like 4
6811	STX5	syntaxin 5
7161	TP73	tumor protein p73
3620	IDO1	indoleamine 2,3-dioxygenase 1
57212	TP73-AS1	TP73 antisense RNA 1 (non-protein coding)
440675	NBPF12	neuroblastoma breakpoint family, member 12
11185	INMT	indolethylamine N-methyltransferase
343508	LOC343508	aconitate hydratase, mitochondrial-like
10232	MSLN	mesothelin
6368	CCL23	chemokine (C-C motif) ligand 23
55634	ZNF673	zinc finger family member 673
10815	CPLX1	complexin 1
729591	[No Symbol]	[No Name]
642574	LOC642574	IQ motif and ankyrin repeat domain-containing protein LOC642574
153571	C5orf38	chromosome 5 open reading frame 38
343505	NBPF7	neuroblastoma breakpoint family, member 7
4922	NTS	neurotensin
10533	ATG7	ATG7 autophagy related 7 homolog (S. cerevisiae)
89869	PLCZ1	phospholipase C, zeta 1
132205	[No Symbol]	[No Name]
100131039	[No Symbol]	[No Name]

645626	LOC645626	coiled-coil domain containing 29-like
54845	ESRP1	epithelial splicing regulatory protein 1
29964	PRICKLE4	prickle homolog 4 (Drosophila)
54467	ANKIB1	ankyrin repeat and IBR domain containing 1
146279	TEKT5	tektin 5
1611	DAP	death-associated protein
642319	LOC642319	transgelin 2 pseudogene
6786	STIM1	stromal interaction molecule 1
338699	ANKRD42	ankyrin repeat domain 42
1797	DOM3Z	dom-3 homolog Z (C. elegans)
5005	ORM2	orosomucoid 2
100129149	LOC100129149	hypothetical protein LOC100129149
59340	HRH4	histamine receptor H4
81579	PLA2G12A	phospholipase A2, group XIIA
202865	C7orf33	chromosome 7 open reading frame 33
100132448	[No Symbol]	[No Name]
391807	[No Symbol]	[No Name]
441869	LOC441869	hypothetical protein LOC441869
2158	F9	coagulation factor IX
442058	LOC442058	replication protein A 14 kDa subunit-like
139231	FAM199X	family with sequence similarity 199, X-linked
3467	IFNW1	interferon, omega 1
79845	RNF122	ring finger protein 122
100129381	LOC100129381	ribosomal protein S7 pseudogene
767598	SNORD114-20	small nucleolar RNA, C/D box 114-20
390079	OR52E8	olfactory receptor, family 52, subfamily E, member 8
6578	SLCO2A1	solute carrier organic anion transporter family, member 2A1
728763	LOC728763	rootletin-like
153222	C5orf41	chromosome 5 open reading frame 41
3748	KCNC3	potassium voltage-gated channel, Shaw-related subfamily, member 3
22925	PLA2R1	phospholipase A2 receptor 1, 180kDa
389174	[No Symbol]	[No Name]
728815	LOC728815	cytokine receptor-like factor 3 pseudogene
374907	B3GNT8	UDP-GlcNAc:betaGal beta-1,3-N-acetylglucosaminyltransferase 8
390211	KRT8P26	keratin 8 pseudogene 26

57403	RAB22A	RAB22A, member RAS oncogene family
78989	COLEC11	collectin sub-family member 11
10071	MUC12	mucin 12, cell surface associated
282890	ZNF311	zinc finger protein 311
200576	PIKFYVE	phosphoinositide kinase, FYVE finger containing
838	CASP5	caspase 5, apoptosis-related cysteine peptidase
2897	GRIK1	glutamate receptor, ionotropic, kainate 1
100128966	LOC100128966	similar to hCG2045131
79490	OR10G6	olfactory receptor, family 10, subfamily G, member 6
119678	OR52E2	olfactory receptor, family 52, subfamily E, member 2
54947	LPCAT2	lysophosphatidylcholine acyltransferase 2
143241	DYDC1	DPY30 domain containing 1
100130816	[No Symbol]	[No Name]
100128660	[No Symbol]	[No Name]
3663	IRF5	interferon regulatory factor 5
644042	SLC25A5P6	solute carrier family 25 (mitochondrial carrier; adenine nucleotide translocator), member 5 pseudogene 6
389369	[No Symbol]	[No Name]
89782	LMLN	leishmanolysin-like (metallopeptidase M8 family)
6092	ROBO2	roundabout, axon guidance receptor, homolog 2 (Drosophila)
388555	IGFL3	IGF-like family member 3
134147	CMBL	carboxymethylenebutenolidase homolog (Pseudomonas)
113540	CMTM1	CKLF-like MARVEL transmembrane domain containing 1
140101	RBMY2MP	RNA binding motif protein, Y-linked, family 2, member M pseudogene
57369	GJD2	gap junction protein, delta 2, 36kDa
1048	CEACAM5	carcinoembryonic antigen-related cell adhesion molecule 5
50674	NEUROG3	neurogenin 3
56062	KLHL4	kelch-like 4 (Drosophila)
84866	TMEM25	transmembrane protein 25
157530	ST13P6	suppression of tumorigenicity 13 (colon carcinoma) (Hsp70 interacting protein) pseudogene 6
84458	LCOR	ligand dependent nuclear receptor corepressor
652423	LOC652423	nucleophosmin (nucleolar phosphoprotein B23, numatrin) pseudogene
3834	KIF25	kinesin family member 25
5349	FXYD3	FXYD domain containing ion transport regulator 3
260293	CYP4X1	cytochrome P450, family 4, subfamily X, polypeptide 1
9378	NRXN1	neurexin 1

10261	IGSF6	immunoglobulin superfamily, member 6
3235	HOXD9	homeobox D9
79804	NBLA00301	Nbla00301
10716	TBR1	T-box, brain, 1
342510	CD300E	CD300e molecule
342527	SMTNL2	smoothelin-like 2
254950	KRTAP15-1	keratin associated protein 15-1
3567	IL5	interleukin 5 (colony-stimulating factor, eosinophil)
7414	VCL	vinculin
10282	BET1	blocked early in transport 1 homolog (S. cerevisiae)
23633	KPNA6	karyopherin alpha 6 (importin alpha 7)
7837	PXDN	peroxidasin homolog (Drosophila)
7752	ZNF200	zinc finger protein 200
123876	ACSM2A	acyl-CoA synthetase medium-chain family member 2A
55785	FGD6	FYVE, RhoGEF and PH domain containing 6
55225	RAVER2	ribonucleoprotein, PTB-binding 2
80086	TUBA4B	tubulin, alpha 4b (pseudogene)
100128161	[No Symbol]	[No Name]
359748	MRPL49P1	mitochondrial ribosomal protein L49 pseudogene 1
1080	CFTR	cystic fibrosis transmembrane conductance regulator (ATP-binding cassette sub-family C, member 7)
100133236	[No Symbol]	[No Name]
54795	TRPM4	transient receptor potential cation channel, subfamily M, member 4
100128790	LOC100128790	lysophospholipase II pseudogene
6326	SCN2A	sodium channel, voltage-gated, type II, alpha subunit
400058	MKRN9P	makorin ring finger protein 9, pseudogene
391578	LOC391578	MAF1 homolog (S. cerevisiae) pseudogene
116285	ACSM1	acyl-CoA synthetase medium-chain family member 1
956	ENTPD3	ectonucleoside triphosphate diphosphohydrolase 3
8226	HDHD1	haloacid dehalogenase-like hydrolase domain containing 1
780776	FAM18A	family with sequence similarity 18, member A
10060	ABCC9	ATP-binding cassette, sub-family C (CFTR/MRP), member 9
137682	C8orf38	chromosome 8 open reading frame 38
10846	PDE10A	phosphodiesterase 10A
8464	SUPT3H	suppressor of Ty 3 homolog (S. cerevisiae)
400793	C1orf226	chromosome 1 open reading frame 226

828	CAPS	calcyphosine
4690	NCK1	NCK adaptor protein 1
8324	FZD7	frizzled family receptor 7
642968	FAM163B	family with sequence similarity 163, member B
10331	B3GNT3	UDP-GlcNAc:betaGal beta-1,3-N-acetylglucosaminyltransferase 3
168417	ZNF679	zinc finger protein 679
7982	ST7	suppression of tumorigenicity 7
1089	CEACAM4	carcinoembryonic antigen-related cell adhesion molecule 4
54540	FAM193B	family with sequence similarity 193, member B
5122	PCSK1	proprotein convertase subtilisin/kexin type 1
100130226	[No Symbol]	[No Name]
1780	DYNC1I1	dynein, cytoplasmic 1, intermediate chain 1
574016	CLLU1OS	chronic lymphocytic leukemia up-regulated 1 opposite strand
390637	C15orf58	chromosome 15 open reading frame 58
7941	PLA2G7	phospholipase A2, group VII (platelet-activating factor acetylhydrolase, plasma)
10492	SYNCRIP	synaptotagmin binding, cytoplasmic RNA interacting protein
151825	KRT18P43	keratin 18 pseudogene 43
1303	COL12A1	collagen, type XII, alpha 1
10048	RANBP9	RAN binding protein 9
9991	ROD1	ROD1 regulator of differentiation 1 (S. pombe)
27123	DKK2	dickkopf homolog 2 (Xenopus laevis)
83957	FKSG43	FERM domain-containing protein 8 pseudogene
139378	GPR112	G protein-coupled receptor 112
89953	KLC4	kinesin light chain 4
10840	ALDH1L1	aldehyde dehydrogenase 1 family, member L1
26514	RPL34P1	ribosomal protein L34 pseudogene 1
1003	CDH5	cadherin 5, type 2 (vascular endothelium)
133308	NHEDC2	Na+/H+ exchanger domain containing 2
644232	[No Symbol]	[No Name]
402226	CCT5P1	chaperonin containing TCP1, subunit 5 (epsilon) pseudogene 1
391081	HSD3BP5	hydroxy-delta-5-steroid dehydrogenase, 3 beta, pseudogene 5
1363	CPE	carboxypeptidase E
7310	ZRSR1	zinc finger (CCCH type), RNA-binding motif and serine/arginine rich 1
100128916	[No Symbol]	[No Name]
6738	TROVE2	TROVE domain family, member 2

117155	CATSPER2	cation channel, sperm associated 2
55034	MOCOS	molybdenum cofactor sulfurase
728098	LOC728098	mitogen-activated protein kinase 1 interacting protein 1-like pseudogene
100131512	[No Symbol]	[No Name]
131920	TMEM207	transmembrane protein 207
100130557	LOC100130557	hypothetical LOC100130557
6277	S100A6	S100 calcium binding protein A6
100129520	LOC100129520	testis expressed sequence 13-like
728661	SLC35E2B	solute carrier family 35, member E2B
5786	PTPRA	protein tyrosine phosphatase, receptor type, A
100129212	[No Symbol]	[No Name]
22992	KDM2A	lysine (K)-specific demethylase 2A
10249	GLYAT	glycine-N-acyltransferase
139952	LOC139952	LIM domain kinase 2 pseudogene
6445	SGCG	sarcoglycan, gamma (35kDa dystrophin-associated glycoprotein)
54606	DDX56	DEAD (Asp-Glu-Ala-Asp) box polypeptide 56
7059	THBS3	thrombospondin 3
3683	ITGAL	integrin, alpha L (antigen CD11A (p180), lymphocyte function-associated antigen 1; alpha polypeptide)
146760	RTN4RL1	reticulon 4 receptor-like 1
26355	FAM162A	family with sequence similarity 162, member A
60592	SCOC	short coiled-coil protein
3706	ITPKA	inositol-trisphosphate 3-kinase A
100133307	CYP1D1	cytochrome P450, family 1, subfamily D pseudogene
23022	PALLD	palladin, cytoskeletal associated protein
100129848	[No Symbol]	[No Name]
100131060	LOC100131060	hypothetical LOC100131060
647001	GAPDHP35	glyceraldehyde 3 phosphate dehydrogenase pseudogene 35
125113	KRT222	keratin 222
10516	FBLN5	fibulin 5
100129730	[No Symbol]	[No Name]
3973	LHCGR	luteinizing hormone/choriogonadotropin receptor
127247	ASB17	ankyrin repeat and SOCS box containing 17
645301	[No Symbol]	[No Name]
340485	ACER2	alkaline ceramidase 2
730110	LOC730110	zinc finger protein 492 pseudogene

729085	FAM198A	family with sequence similarity 198, member A
80210	ARMC9	armadillo repeat containing 9
339761	CYP27C1	cytochrome P450, family 27, subfamily C, polypeptide 1
200150	PLD5	phospholipase D family, member 5
23554	TSPAN12	tetraspanin 12
337967	KRTAP6-2	keratin associated protein 6-2
652276	LOC652276	hypothetical LOC652276
79706	PRKRIP1	PRKR interacting protein 1 (IL11 inducible)
23581	CASP14	caspase 14, apoptosis-related cysteine peptidase
391844	[No Symbol]	[No Name]
56999	ADAMTS9	ADAM metallopeptidase with thrombospondin type 1 motif, 9
100130288	[No Symbol]	[No Name]
57216	VANGL2	vang-like 2 (van gogh, Drosophila)
160762	CCDC63	coiled-coil domain containing 63
22899	ARHGEF15	Rho guanine nucleotide exchange factor (GEF) 15
2186	BPTF	bromodomain PHD finger transcription factor
3326	HSP90AB1	heat shock protein 90kDa alpha (cytosolic), class B member 1
201305	SPNS3	spinster homolog 3 (Drosophila)
150921	TCF23	transcription factor 23
100130307	LOC100130307	speedy homolog E2 (Xenopus laevis) pseudogene
389676	LOC389676	hypothetical LOC389676
100132809	[No Symbol]	[No Name]
152189	CMTM8	CKLF-like MARVEL transmembrane domain containing 8
1415	CRYBB2	crystallin, beta B2
285362	SUMF1	sulfatase modifying factor 1
100131469	RPSAP62	ribosomal protein SA pseudogene 62
127731	VWA5B1	von Willebrand factor A domain containing 5B1
326627	RANBP20P	RAN binding protein 20 pseudogene
10594	PRPF8	PRP8 pre-mRNA processing factor 8 homolog (S. cerevisiae)
9320	TRIP12	thyroid hormone receptor interactor 12
26036	ZNF451	zinc finger protein 451
100128259	[No Symbol]	[No Name]
2160	F11	coagulation factor XI
54431	DNAJC10	DnaJ (Hsp40) homolog, subfamily C, member 10
3631	INPP4A	inositol polyphosphate-4-phosphatase, type I, 107kDa

5067	CNTN3	contactin 3 (plasmacytoma associated)
79955	PDZD7	PDZ domain containing 7
100128542	LOC100128542	hypothetical protein LOC100128542
23408	SIRT5	sirtuin 5
100130448	LOC100130448	MRS2 magnesium homeostasis factor homolog (S. cerevisiae) pseudogene
1716	DGUOK	deoxyguanosine kinase
7681	MKRN3	makorin ring finger protein 3
221806	VWDE	von Willebrand factor D and EGF domains
4585	MUC4	mucin 4, cell surface associated
730226	[No Symbol]	[No Name]
129881	C2orf77	chromosome 2 open reading frame 77
7903	ST8SIA4	ST8 alpha-N-acetyl-neuraminide alpha-2,8-sialyltransferase 4
8705	B3GALT4	UDP-Gal:betaGlcNAc beta 1,3-galactosyltransferase, polypeptide 4
283579	C14orf178	chromosome 14 open reading frame 178
100131348	LOC100131348	adaptor-related protein complex 2, beta 1 subunit pseudogene
2110	ETFDH	electron-transferring-flavoprotein dehydrogenase
339855	KY	kyphoscoliosis peptidase
5359	PLSCR1	phospholipid scramblase 1
202181	LOC202181	hypothetical LOC202181
8342	HIST1H2BM	histone cluster 1, H2bm
400454	UNQ9370	IFMQ9370
729179	[No Symbol]	[No Name]
441505	LOC441505	stress-induced-phosphoprotein 1 pseudogene
79582	SPAG16	sperm associated antigen 16
133558	HEATR7B2	HEAT repeat family member 7B2
6820	SULT2B1	sulfotransferase family, cytosolic, 2B, member 1
126755	LRRC38	leucine rich repeat containing 38
53833	IL20RB	interleukin 20 receptor beta
8995	TNFSF18	tumor necrosis factor (ligand) superfamily, member 18
9867	PJA2	praja ring finger 2
646560	[No Symbol]	[No Name]
5648	MASP1	mannan-binding lectin serine peptidase 1 (C4/C2 activating component of Ra-reactive factor)
55789	DEPDC1B	DEP domain containing 1B
5569	PKIA	protein kinase (cAMP-dependent, catalytic) inhibitor alpha
115	ADCY9	adenylate cyclase 9

266747	RGL4	ral guanine nucleotide dissociation stimulator-like 4
85456	TNKS1BP1	tankyrase 1 binding protein 1, 182kDa
84933	C8orf76	chromosome 8 open reading frame 76
81789	TIGD6	tigger transposable element derived 6
100131550	CNN2P12	calponin 2 pseudogene 12
7840	ALMS1	Alstrom syndrome 1
167465	ZNF366	zinc finger protein 366
100128438	[No Symbol]	[No Name]
441097	[No Symbol]	[No Name]
391123	VSIG8	V-set and immunoglobulin domain containing 8
9098	USP6	ubiquitin specific peptidase 6 (Tre-2 oncogene)
100130969	[No Symbol]	[No Name]
9202	ZMYM4	zinc finger, MYM-type 4
284912	LOC284912	hypothetical LOC284912
5581	PRKCE	protein kinase C, epsilon
149466	C1orf210	chromosome 1 open reading frame 210
646583	RPS4XP18	ribosomal protein S4X pseudogene 18
9022	CLIC3	chloride intracellular channel 3
3710	ITPR3	inositol 1,4,5-trisphosphate receptor, type 3
2135	EXTL2	exostoses (multiple)-like 2
93082	NEURL3	neuralized homolog 3 (Drosophila) pseudogene
339	APOBEC1	apolipoprotein B mRNA editing enzyme, catalytic polypeptide 1
6507	SLC1A3	solute carrier family 1 (glial high affinity glutamate transporter), member 3
442663	[No Symbol]	[No Name]
100128098	LOC100128098	hypothetical LOC100128098
100129726	LOC100129726	hypothetical LOC100129726
149069	DCDC2B	doublecortin domain containing 2B
60685	ZFAND3	zinc finger, AN1-type domain 3
22854	NTNG1	netrin G1
200959	GABRR3	gamma-aminobutyric acid (GABA) receptor, rho 3
84570	COL25A1	collagen, type XXV, alpha 1
55184	C20orf12	chromosome 20 open reading frame 12
3827	KNG1	kininogen 1
55696	RBM22	RNA binding motif protein 22
9100	USP10	ubiquitin specific peptidase 10

57154	SMURF1	SMAD specific E3 ubiquitin protein ligase 1
100132531	[No Symbol]	[No Name]
442382	LOC442382	ATPase, Ca++ transporting, plasma membrane 1 pseudogene
51201	ZDHHC2	zinc finger, DHHC-type containing 2
100131017	ZNF316	zinc finger protein 316
5259	PHKBP2	phosphorylase kinase, beta pseudogene 2
1956	EGFR	epidermal growth factor receptor
53632	PRKAG3	protein kinase, AMP-activated, gamma 3 non-catalytic subunit
57604	KIAA1456	KIAA1456
1118	CHIT1	chitinase 1 (chitotriosidase)
390174	OR9G1	olfactory receptor, family 9, subfamily G, member 1
57404	CYP20A1	cytochrome P450, family 20, subfamily A, polypeptide 1
643496	LOC643496	hCG1660379
10230	NBR2	neighbor of BRCA1 gene 2 (non-protein coding)
389158	PLSCR5	phospholipid scramblase family, member 5
6661	SOX5P	SRY (sex determining region Y)-box 5 pseudogene
4197	[No Symbol]	[No Name]
3780	KCNN1	potassium intermediate/small conductance calcium-activated channel, subfamily N, member 1
132203	SNTN	sentan, cilia apical structure protein
133482	SLCO6A1	solute carrier organic anion transporter family, member 6A1
100128238	LOC100128238	KIAA0895-like pseudogene
100130357	LOC100130357	hypothetical LOC100130357
146512	FLJ30679	hypothetical protein FLJ30679
8409	UXT	ubiquitously-expressed transcript
727936	GXYLT2	glucoside xylosyltransferase 2
136371	ASB10	ankyrin repeat and SOCS box containing 10
25862	USP49	ubiquitin specific peptidase 49
9390	SLC22A13	solute carrier family 22 (organic anion transporter), member 13
8761	PABPC4	poly(A) binding protein, cytoplasmic 4 (inducible form)
645638	LOC645638	WDNM1-like pseudogene
727	C5	complement component 5
2272	FHIT	fragile histidine triad gene
100131483	[No Symbol]	[No Name]
6414	SEPP1	selenoprotein P, plasma, 1
645852	LOC645852	splicing factor 3a, subunit 2, 66kDa pseudogene

352909	C19orf51	chromosome 19 open reading frame 51
6170	RPL39	ribosomal protein L39
163933	FAM43B	family with sequence similarity 43, member B
26150	RIBC2	RIB43A domain with coiled-coils 2
64599	GIGYF1	GRB10 interacting GYF protein 1
347252	IGFBPL1	insulin-like growth factor binding protein-like 1
113802	HENMT1	HEN1 methyltransferase homolog 1 (Arabidopsis)
353324	SPATA12	spermatogenesis associated 12
340351	AGBL3	ATP/GTP binding protein-like 3
400456	LOC400456	hypothetical LOC400456
390007	TOMM22P5	translocase of outer mitochondrial membrane 22 homolog (yeast) pseudogene 5
642448	KRT18P23	keratin 18 pseudogene 23
57159	TRIM54	tripartite motif containing 54
442233	SMARCE1P2	SWI/SNF related, matrix associated, actin dependent regulator of chromatin, subfamily e, member 1 pseudogene 2
9448	MAP4K4	mitogen-activated protein kinase kinase kinase kinase 4
10406	WFDC2	WAP four-disulfide core domain 2
56776	FMN2	formin 2
84561	SLC12A8	solute carrier family 12 (potassium/chloride transporters), member 8
79733	E2F8	E2F transcription factor 8
5378	PMS1	PMS1 postmeiotic segregation increased 1 (S. cerevisiae)
57826	RAP2C	RAP2C, member of RAS oncogene family
401546	C9orf152	chromosome 9 open reading frame 152
390924	EEF1A1P7	eukaryotic translation elongation factor 1 alpha 1 pseudogene 7
5606	MAP2K3	mitogen-activated protein kinase kinase 3
254187	TSGA10IP	testis specific, 10 interacting protein
100127936	LOC100127936	hypothetical LOC100127936
140701	ABHD16B	abhydrolase domain containing 16B
2066	ERBB4	v-erb-a erythroblastic leukemia viral oncogene homolog 4 (avian)
4856	NOV	nephroblastoma overexpressed gene
719	C3AR1	complement component 3a receptor 1
93010	B3GNT7	UDP-GlcNAc:betaGal beta-1,3-N-acetylglucosaminyltransferase 7
729234	LOC729234	fumarylacetoacetate hydrolase domain containing 2 pseudogene
386678	KRTAP10-11	keratin associated protein 10-11
7018	TF	transferrin
25960	GPR124	G protein-coupled receptor 124

7881	KCNAB1	potassium voltage-gated channel, shaker-related subfamily, beta member 1
5993	RFX5	regulatory factor X, 5 (influences HLA class II expression)
8869	ST3GAL5	ST3 beta-galactoside alpha-2,3-sialyltransferase 5
100128121	LOC100128121	60S ribosomal protein L9-like
644016	HSPD1P10	heat shock 60kDa protein 1 (chaperonin) pseudogene 10
10201	NME6	non-metastatic cells 6, protein expressed in (nucleoside-diphosphate kinase)
64326	RFWD2	ring finger and WD repeat domain 2
85366	MYLK2	myosin light chain kinase 2
81544	GDPD5	glycerophosphodiester phosphodiesterase domain containing 5
219578	ZNF804B	zinc finger protein 804B
100133135	[No Symbol]	[No Name]
375686	SPATC1	spermatogenesis and centriole associated 1
5568	PRKACG	protein kinase, cAMP-dependent, catalytic, gamma
100131989	[No Symbol]	[No Name]
57574	4-Mar	membrane-associated ring finger (C3HC4) 4
440335	LOC440335	hypothetical LOC440335
54870	QRICH1	glutamine-rich 1
261726	TIPRL	TIP41, TOR signaling pathway regulator-like (S. cerevisiae)
392442	LOC392442	BCL2-associated athanogene pseudogene
284252	KCTD1	potassium channel tetramerisation domain containing 1
201243	C17orf74	chromosome 17 open reading frame 74
5692	PSMB4	proteasome (prosome, macropain) subunit, beta type, 4
390423	ARF4P3	ADP-ribosylation factor 4 pseudogene 3
100129781	LOC100129781	hypothetical LOC100129781
1232	CCR3	chemokine (C-C motif) receptor 3
8614	STC2	stanniocalcin 2
319130	HMGB1P14	high mobility group box 1 pseudogene 14
286094	LOC286094	hypothetical LOC286094
140628	GATA5	GATA binding protein 5
3215	HOXB5	homeobox B5
203477	HMGA1P1	high mobility group AT-hook 1 pseudogene 1
8549	LGR5	leucine-rich repeat containing G protein-coupled receptor 5
442078	KRT8P18	keratin 8 pseudogene 18
392862	GRID2IP	glutamate receptor, ionotropic, delta 2 (Grid2) interacting protein
222865	TMEM130	transmembrane protein 130

27303	RBMS3	RNA binding motif, single stranded interacting protein 3
100132074	FOXO6	forkhead box O6
222537	HS3ST5	heparan sulfate (glucosamine) 3-O-sulfotransferase 5
4293	MAP3K9	mitogen-activated protein kinase kinase kinase 9
645726	[No Symbol]	[No Name]
648708	[No Symbol]	[No Name]
10586	MAB21L2	mab-21-like 2 (C. elegans)
100129416	[No Symbol]	[No Name]
643406	LOC643406	hypothetical LOC643406
403314	APOBEC4	apolipoprotein B mRNA editing enzyme, catalytic polypeptide-like 4 (putative)
84303	CHCHD6	coiled-coil-helix-coiled-coil-helix domain containing 6
23122	CLASP2	cytoplasmic linker associated protein 2
730978	CICP5	capicua homolog (Drosophila) pseudogene 5
389383	C6orf126	chromosome 6 open reading frame 126
732	C8B	complement component 8, beta polypeptide
55231	CCDC87	coiled-coil domain containing 87
100049716	LOC100049716	hypothetical LOC100049716
1488	CTBP2	C-terminal binding protein 2
9678	PHF14	PHD finger protein 14
23333	DPY19L1	dpy-19-like 1 (C. elegans)
29943	PADI1	peptidyl arginine deiminase, type I
391026	LOC391026	alpha-actinin-4-like
100133224	LOC100133224	hypothetical protein LOC100133224
51179	HAO2	hydroxyacid oxidase 2 (long chain)
3930	LBR	lamin B receptor
100130332	[No Symbol]	[No Name]
5628	PROSP	protein S pseudogene (beta)
647076	BRD7P2	bromodomain containing 7 pseudogene 2
116966	WDR17	WD repeat domain 17
23163	GGA3	golgi-associated, gamma adaptin ear containing, ARF binding protein 3
283298	OLFML1	olfactomedin-like 1
26687	OR4E1	olfactory receptor, family 4, subfamily E, member 1 (gene/pseudogene)
4168	MCF2	MCF.2 cell line derived transforming sequence
100132658	LOC100132658	NSA2 ribosome biogenesis homolog (S. cerevisiae) pseudogene
79675	FASTKD1	FAST kinase domains 1

339352	LOC339352	putative ATP-binding domain-containing protein 3-like protein-like
3756	KCNH1	potassium voltage-gated channel, subfamily H (eag-related), member 1
2561	GABRB2	gamma-aminobutyric acid (GABA) A receptor, beta 2
4493	MT1E	metallothionein 1E
57568	SIPA1L2	signal-induced proliferation-associated 1 like 2
100132056	LOC100132056	dihydrofolate reductase pseudogene
64983	MRPL32	mitochondrial ribosomal protein L32
51002	TPRKB	TP53RK binding protein
9260	PDLIM7	PDZ and LIM domain 7 (enigma)
100129263	[No Symbol]	[No Name]
6397	SEC14L1	SEC14-like 1 (S. cerevisiae)
1809	DPYSL3	dihydropyrimidinase-like 3
22821	RASA3	RAS p21 protein activator 3
84103	C4orf17	chromosome 4 open reading frame 17
359736	MRPL15P1	mitochondrial ribosomal protein L15 pseudogene 1
27124	INPP5J	inositol polyphosphate-5-phosphatase J
50937	CDON	Cdon homolog (mouse)
56605	ERO1LB	ERO1-like beta (S. cerevisiae)
6391	SDHC	succinate dehydrogenase complex, subunit C, integral membrane protein, 15kDa
347127	FAM75D5	FAM75-like protein FLJ46321 pseudogene
64420	SUSD1	sushi domain containing 1
827	CAPN6	calpain 6
340706	VWA2	von Willebrand factor A domain containing 2
9356	SLC22A6	solute carrier family 22 (organic anion transporter), member 6
284451	ODF3L2	outer dense fiber of sperm tails 3-like 2
150084	IGSF5	immunoglobulin superfamily, member 5
54537	FAM35A	family with sequence similarity 35, member A
8692	HYAL2	hyaluronoglucosaminidase 2
54937	SOHLH2	spermatogenesis and oogenesis specific basic helix-loop-helix 2
100132215	LOC100132215	hypothetical LOC100132215
23567	ZNF346	zinc finger protein 346
100130623	[No Symbol]	[No Name]
9733	SART3	squamous cell carcinoma antigen recognized by T cells 3
100129250	LOC100129250	hypothetical LOC100129250
646021	LOC646021	hypothetical protein LOC646021

762	CA4	carbonic anhydrase IV
103910	MYL12B	myosin, light chain 12B, regulatory
100132891	LOC100132891	hypothetical LOC100132891
11034	DSTN	destrin (actin depolymerizing factor)
55565	ZNF821	zinc finger protein 821
4807	NHLH1	nescient helix loop helix 1
139221	MUM1L1	melanoma associated antigen (mutated) 1-like 1
2259	FGF14	fibroblast growth factor 14
11222	MRPL3	mitochondrial ribosomal protein L3
7477	WNT7B	wingless-type MMTV integration site family, member 7B
120321	CSNK1A1P2	casein kinase 1, alpha 1 pseudogene 2
491	ATP2B2	ATPase, Ca++ transporting, plasma membrane 2
114188	SIGLEC29P	sialic acid binding Ig-like lectin 29, pseudogene
150519	LOC150519	hypothetical LOC150519
6357	CCL13	chemokine (C-C motif) ligand 13
6344	SCTR	secretin receptor
284611	FAM102B	family with sequence similarity 102, member B
221223	CES5A	carboxylesterase 5A
377007	KLHL30	kelch-like 30 (Drosophila)
9957	HS3ST1	heparan sulfate (glucosamine) 3-O-sulfotransferase 1
3704	ITPA	inosine triphosphatase (nucleoside triphosphate pyrophosphatase)
400508	NCRNA00169	non-protein coding RNA 169
728588	MS4A18	membrane-spanning 4-domains, subfamily A, member 18
2583	B4GALNT1	beta-1,4-N-acetyl-galactosaminyl transferase 1
2645	GCK	glucokinase (hexokinase 4)
400863	FLJ46020	FLJ46020 protein
55083	KIF26B	kinesin family member 26B
100132473	[No Symbol]	[No Name]
3032	HADHB	hydroxyacyl-CoA dehydrogenase/3-ketoacyl-CoA thiolase/enoyl-CoA hydratase (trifunctional protein), beta subunit
83871	RAB34	RAB34, member RAS oncogene family
5134	PDCD2	programmed cell death 2
81270	OR52B5P	olfactory receptor, family 52, subfamily B, member 5 pseudogene
647162	KRT18P33	keratin 18 pseudogene 33
4605	MYBL2	v-myb myeloblastosis viral oncogene homolog (avian)-like 2
8595	OR5D2P	olfactory receptor, family 5, subfamily D, member 2 pseudogene

100132348	LOC100132348	similar to hCG1817457
10200	MPHOSPH6	M-phase phosphoprotein 6
387826	[No Symbol]	[No Name]
4985	OPRD1	opioid receptor, delta 1
64094	SMOC2	SPARC related modular calcium binding 2
135886	WBSCR28	Williams-Beuren syndrome chromosome region 28
286006	C7orf53	chromosome 7 open reading frame 53
57829	ZP4	zona pellucida glycoprotein 4
4995	OR3A2	olfactory receptor, family 3, subfamily A, member 2
89876	C3orf15	chromosome 3 open reading frame 15
163059	ZNF433	zinc finger protein 433
25836	NIPBL	Nipped-B homolog (Drosophila)
84524	ZC3H8	zinc finger CCCH-type containing 8
730974	LOC730974	hypothetical LOC730974
114795	TMEM132B	transmembrane protein 132B
1729	DIAPH1	diaphanous homolog 1 (Drosophila)
254827	NAALADL2	N-acetylated alpha-linked acidic dipeptidase-like 2
100131661	FTLP5	ferritin, light polypeptide pseudogene 5
6900	CNTN2	contactin 2 (axonal)
51666	ASB4	ankyrin repeat and SOCS box containing 4
50940	PDE11A	phosphodiesterase 11A
91750	LIN52	lin-52 homolog (C. elegans)
79870	BAALC	brain and acute leukemia, cytoplasmic
1307	COL16A1	collagen, type XVI, alpha 1
100128296	[No Symbol]	[No Name]
260341	TFAMP1	transcription factor A, mitochondrial pseudogene 1
150554	LOC150554	tyrosine 3-monooxygenase/tryptophan 5-monooxygenase activation protein, zeta pseudogene
161	AP2A2	adaptor-related protein complex 2, alpha 2 subunit
8326	FZD9	frizzled family receptor 9
6251	RSU1	Ras suppressor protein 1
56246	MRAP	melanocortin 2 receptor accessory protein
10361	NPM2	nucleophosmin/nucleoplasmin 2
10625	IVNS1ABP	influenza virus NS1A binding protein
90293	KLHL13	kelch-like 13 (Drosophila)
123099	DEGS2	degenerative spermatocyte homolog 2, lipid desaturase (Drosophila)

266954	CABYRP1	calcium binding tyrosine-(Y)-phosphorylation regulated pseudogene 1
3897	L1CAM	L1 cell adhesion molecule
718	C3	complement component 3
390301	ST13P9	suppression of tumorigenicity 13 (colon carcinoma) (Hsp70 interacting protein) pseudogene 9
107	ADCY1	adenylate cyclase 1 (brain)
100128370	[No Symbol]	[No Name]
399980	[No Symbol]	[No Name]
6662	SOX9	SRY (sex determining region Y)-box 9
339201	C17orf65	chromosome 17 open reading frame 65
100131335	[No Symbol]	[No Name]
100131229	RPL6P17	ribosomal protein L6 pseudogene 17
79635	CCDC121	coiled-coil domain containing 121
347119	LOC347119	protein C15orf2-like
2588	GALNS	galactosamine (N-acetyl)-6-sulfate sulfatase
392617	ELFN1	extracellular leucine-rich repeat and fibronectin type III domain containing 1
155060	LOC155060	AI894139 pseudogene
56241	SUSD2	sushi domain containing 2
391819	KRT18P42	keratin 18 pseudogene 42
392485	LOC392485	ataxin 7-like 3 pseudogene
643797	LOC643797	AGVR6190
84624	FNDC1	fibronectin type III domain containing 1
51741	WWOX	WW domain containing oxidoreductase
100130023	[No Symbol]	[No Name]
23627	PRND	prion protein 2 (dublet)
391538	ALDOAP1	aldolase A, fructose-bisphosphate pseudogene 1
9783	RIMS3	regulating synaptic membrane exocytosis 3
7552	ZNF711	zinc finger protein 711
7500	XGPY	Xg pseudogene, Y-linked
285382	C3orf70	chromosome 3 open reading frame 70
79370	BCL2L14	BCL2-like 14 (apoptosis facilitator)
139420	SMEK3P	SMEK homolog 3, suppressor of mek1 (Dictyostelium) pseudogene
6290	SAA3P	serum amyloid A3 pseudogene
23198	PSME4	proteasome (prosome, macropain) activator subunit 4
154215	NKAIN2	Na+/K+ transporting ATPase interacting 2
730198	LOC730198	hypothetical protein LOC730198

5502	PPP1R1A	protein phosphatase 1, regulatory (inhibitor) subunit 1A
359724	RN18S2P	RNA, 18S ribosomal 2, pseudogene
257019	FRMD3	FERM domain containing 3
57608	KIAA1462	KIAA1462
7125	TNNC2	troponin C type 2 (fast)
649873	LOC649873	peptidyl-prolyl cis-trans isomerase A-like
388886	C22orf36	chromosome 22 open reading frame 36
100131130	HNRNPA1P13	heterogeneous nuclear ribonucleoprotein A1 pseudogene 13
1660	DHX9	DEAH (Asp-Glu-Ala-His) box polypeptide 9
127687	C1orf122	chromosome 1 open reading frame 122
441750	LOC441750	chromosome 12 open reading frame 49 pseudogene
3358	HTR2C	5-hydroxytryptamine (serotonin) receptor 2C
2672	GFI1	growth factor independent 1 transcription repressor
646576	LOC646576	hypothetical LOC646576
728361	OVOL3	ovo-like 3 (Drosophila)
643114	[No Symbol]	[No Name]
3535	IGL@	immunoglobulin lambda locus
140689	CBLN4	cerebellin 4 precursor
100129568	[No Symbol]	[No Name]
81849	ST6GALNAC5	ST6 (alpha-N-acetyl-neuraminyl-2,3-beta-galactosyl-1,3)-N-acetylgalactosaminide alpha-2,6-sialyltransferase 5
390816	THA1P	threonine aldolase 1, pseudogene
9436	NCR2	natural cytotoxicity triggering receptor 2
79017	GGCT	gamma-glutamylcyclotransferase
113612	CYP2U1	cytochrome P450, family 2, subfamily U, polypeptide 1
54905	CYP2W1	cytochrome P450, family 2, subfamily W, polypeptide 1
26057	ANKRD17	ankyrin repeat domain 17
65263	PYCRL	pyrroline-5-carboxylate reductase-like
54932	EXD3	exonuclease 3'-5' domain containing 3
497258	BDNF-AS1	BDNF antisense RNA 1 (non-protein coding)
23705	CADM1	cell adhesion molecule 1
51112	TTC15	tetratricopeptide repeat domain 15
23367	LARP1	La ribonucleoprotein domain family, member 1
53411	ATP5LP1	ATP synthase, H+ transporting, mitochondrial Fo complex, subunit g, pseudogene 1
391183	RSL24D1P4	ribosomal L24 domain containing 1 pseudogene 4
100129640	LOC100129640	DEAD (Asp-Glu-Ala-Asp) box polypeptide 55 pseudogene

10590	SCGN	secretagogin, EF-hand calcium binding protein
3381	IBSP	integrin-binding sialoprotein
6865	TACR2	tachykinin receptor 2
64766	S100PBP	S100P binding protein
130075	OR9A4	olfactory receptor, family 9, subfamily A, member 4
392490	FLJ44635	TPT1-like protein
203413	CXorf61	chromosome X open reading frame 61
140876	FAM65C	family with sequence similarity 65, member C
6426	SRSF1	serine/arginine-rich splicing factor 1
23284	LPHN3	latrophilin 3
1269	CNR2	cannabinoid receptor 2 (macrophage)
5172	SLC26A4	solute carrier family 26, member 4
8973	CHRNA6	cholinergic receptor, nicotinic, alpha 6
8572	PDLIM4	PDZ and LIM domain 4
100133156	[No Symbol]	[No Name]
9315	C5orf13	chromosome 5 open reading frame 13
2633	GBP1	guanylate binding protein 1, interferon-inducible
7173	TPO	thyroid peroxidase
317671	RFESD	Rieske (Fe-S) domain containing
100129015	[No Symbol]	[No Name]
144448	TSPAN19	tetraspanin 19
6533	SLC6A6	solute carrier family 6 (neurotransmitter transporter, taurine), member 6
646970	LOC646970	chromosome 5 open reading frame 15 pseudogene
8601	RGS20	regulator of G-protein signaling 20
11156	PTP4A3	protein tyrosine phosphatase type IVA, member 3
149281	METTL11B	methyltransferase like 11B
6733	SRPK2	SRSF protein kinase 2
93349	SP140L	SP140 nuclear body protein-like
10829	NPM1P18	nucleophosmin 1 (nucleolar phosphoprotein B23, numatrin) pseudogene 18
100133117	[No Symbol]	[No Name]
7682	MKRN4P	makorin ring finger protein 4, pseudogene
138882	OR1N2	olfactory receptor, family 1, subfamily N, member 2
162989	DEDD2	death effector domain containing 2
10418	SPON1	spondin 1, extracellular matrix protein
341356	RPL31P50	ribosomal protein L31 pseudogene 50

100130105	LOC100130105	tripartite motif-containing protein ENSP00000309378-like
439915	KRTAP5-5	keratin associated protein 5-5
283562	RPL21P9	ribosomal protein L21 pseudogene 9
641515	LOC641515	hypothetical LOC641515
83903	GSG2	germ cell associated 2 (haspin)
6209	RPS15	ribosomal protein S15
9175	MAP3K13	mitogen-activated protein kinase kinase kinase 13
56169	GSDMC	gasdermin C
100130206	[No Symbol]	[No Name]
25854	FAM149A	family with sequence similarity 149, member A
391051	UOX	urate oxidase, pseudogene
3295	HSD17B4	hydroxysteroid (17-beta) dehydrogenase 4
320	APBA1	amyloid beta (A4) precursor protein-binding, family A, member 1
129522	LOC129522	ralA binding protein 1 pseudogene
8387	OR1E1	olfactory receptor, family 1, subfamily E, member 1
219438	OR5D18	olfactory receptor, family 5, subfamily D, member 18
25837	RAB26	RAB26, member RAS oncogene family
3041	HBAP1	hemoglobin, alpha pseudogene 1
28421	IGHV3-52	immunoglobulin heavy variable 3-52 (pseudogene)
7579	ZSCAN20	zinc finger and SCAN domain containing 20
26831	RNU5A	RNA, U5A small nuclear
55890	GPRC5C	G protein-coupled receptor, family C, group 5, member C
100129810	[No Symbol]	[No Name]
57224	NHSL1	NHS-like 1
100129395	LOC100129395	NS5ATP13TP1
140870	WFDC6	WAP four-disulfide core domain 6
153328	SLC25A48	solute carrier family 25, member 48
100128522	HPRTP4	hypoxanthine phosphoribosyltransferase pseudogene 4
151887	CCDC80	coiled-coil domain containing 80
51684	SUFU	suppressor of fused homolog (Drosophila)
344887	LOC344887	NmrA-like family domain containing 1 pseudogene
100130946	[No Symbol]	[No Name]
100131805	RPL6P18	ribosomal protein L6 pseudogene 18
114112	TXNRD3	thioredoxin reductase 3
729059	LOC729059	hypothetical LOC729059

401286	LOC401286	hypothetical LOC401286
83698	CALN1	calneuron 1
85461	TANC1	tetratricopeptide repeat, ankyrin repeat and coiled-coil containing 1
55763	EXOC1	exocyst complex component 1
401677	LOC401677	eukaryotic translation elongation factor 1 alpha 2 pseudogene
5379	PMS2P1	postmeiotic segregation increased 2 pseudogene 1
285735	NCRNA00326	non-protein coding RNA 326
391179	KRT18P32	keratin 18 pseudogene 32
23414	ZFPM2	zinc finger protein, multitype 2
3737	KCNA2	potassium voltage-gated channel, shaker-related subfamily, member 2
6422	SFRP1	secreted frizzled-related protein 1
54492	NEURL1B	neuralized homolog 1B (Drosophila)
29998	GLTSCR1	glioma tumor suppressor candidate region gene 1
10858	CYP46A1	cytochrome P450, family 46, subfamily A, polypeptide 1
402643	TPM3P4	tropomyosin 3 pseudogene 4
84957	RELT	RELT tumor necrosis factor receptor
344371	SLC25A5P2	solute carrier family 25 (mitochondrial carrier; adenine nucleotide translocator), member 5 pseudogene 2
9413	FAM189A2	family with sequence similarity 189, member A2
2046	EPHA8	EPH receptor A8
51738	GHRL	ghrelin/obestatin prepropeptide
5396	PRRX1	paired related homeobox 1
100127962	LOC100127962	family with sequence similarity 190, member B pseudogene
344462	KRT18P39	keratin 18 pseudogene 39
846	CASR	calcium-sensing receptor
346524	OR2Q1P	olfactory receptor, family 2, subfamily Q, member 1 pseudogene
1149	CIDEA	cell death-inducing DFFA-like effector a
23255	CCDC165	coiled-coil domain containing 165
158506	ZNF645	zinc finger protein 645
4217	MAP3K5	mitogen-activated protein kinase kinase kinase 5
4968	OGG1	8-oxoguanine DNA glycosylase
23600	AMACR	alpha-methylacyl-CoA racemase
8651	SOCS1	suppressor of cytokine signaling 1
392443	[No Symbol]	[No Name]
5224	PGAM2	phosphoglycerate mutase 2 (muscle)
165082	GPR113	G protein-coupled receptor 113

55289	ACOXL	acyl-CoA oxidase-like
83445	GSG1	germ cell associated 1
326590	SSXP7	SSX family pseudogene 7
9819	TSC22D2	TSC22 domain family, member 2
653924	LOC653924	glycerol-3-phosphate acyltransferase 2, mitochondrial pseudogene
5294	PIK3CG	phosphoinositide-3-kinase, catalytic, gamma polypeptide
202559	KHDRBS2	KH domain containing, RNA binding, signal transduction associated 2
644661	LOC644661	importin subunit alpha-2-like
441893	GAPDHP29	glyceraldehyde 3 phosphate dehydrogenase pseudogene 29
5586	PKN2	protein kinase N2
121549	ASCL4	achaete-scute complex homolog 4 (Drosophila)
401975	RPS3AP10	ribosomal protein S3a pseudogene 10
6336	SCN10A	sodium channel, voltage-gated, type X, alpha subunit
9635	CLCA2	chloride channel accessory 2
3617	IMPG1	interphotoreceptor matrix proteoglycan 1
115584	SLC5A11	solute carrier family 5 (sodium/glucose cotransporter), member 11
115209	OMA1	OMA1 homolog, zinc metallopeptidase (S. cerevisiae)
55355	HJURP	Holliday junction recognition protein
2271	FH	fumarate hydratase
51108	METTL9	methyltransferase like 9
4159	MC3R	melanocortin 3 receptor
23397	NCAPH	non-SMC condensin I complex, subunit H
54967	CXorf48	chromosome X open reading frame 48
375298	CERKL	ceramide kinase-like
644651	HMGB1P25	high mobility group box 1 pseudogene 25
392262	RPSAP48	ribosomal protein SA pseudogene 48
147381	CBLN2	cerebellin 2 precursor
11278	KLF12	Kruppel-like factor 12
388813	LOC388813	uncharacterized protein ENSP00000383407-like
401036	ASB18	ankyrin repeat and SOCS box containing 18
89870	TRIM15	tripartite motif containing 15
284366	KLK9	kallikrein-related peptidase 9
57514	ARHGAP31	Rho GTPase activating protein 31
93109	TMEM44	transmembrane protein 44
7417	VDAC2	voltage-dependent anion channel 2

54661	PCDHB17	protocadherin beta 17 pseudogene
254048	UBN2	ubinuclein 2
285634	LOC285634	integrator complex subunit 6 pseudogene
84560	MT4	metallothionein 4
9375	TM9SF2	transmembrane 9 superfamily member 2
389180	5-HT3C2	5-HT3c2 serotonin receptor-like protein pseudogene
27252	KLHL20	kelch-like 20 (Drosophila)
155061	ZNF746	zinc finger protein 746
7873	MANF	mesencephalic astrocyte-derived neurotrophic factor
390937	LOC390937	Ets2 repressor factor-like
6563	SLC14A1	solute carrier family 14 (urea transporter), member 1 (Kidd blood group)
131368	ZPLD1	zona pellucida-like domain containing 1
3232	HOXD3	homeobox D3
79026	AHNAK	AHNAK nucleoprotein
79065	ATG9A	ATG9 autophagy related 9 homolog A (S. cerevisiae)
389765	LOC389765	kinesin family member 27 pseudogene
129642	MBOAT2	membrane bound O-acyltransferase domain containing 2
56158	TEX12	testis expressed 12
644739	WASF4P	WAS protein family, member 4, pseudogene
728947	LOC728947	zinc finger protein 680 pseudogene
4353	MPO	myeloperoxidase
286102	TMED10P1	transmembrane emp24-like trafficking protein 10 (yeast) pseudogene 1
728148	[No Symbol]	[No Name]
390649	OR4F15	olfactory receptor, family 4, subfamily F, member 15
1187	CLCNKA	chloride channel Ka
134	ADORA1	adenosine A1 receptor
729696	LOC729696	hypothetical protein LOC729696
340390	KIAA1875	KIAA1875
51725	FBXO40	F-box protein 40
10620	ARID3B	AT rich interactive domain 3B (BRIGHT-like)
2880	GPX5	glutathione peroxidase 5 (epididymal androgen-related protein)
132724	TMPRSS11B	transmembrane protease, serine 11B
79345	OR51B2	olfactory receptor, family 51, subfamily B, member 2
391112	OR6Y1	olfactory receptor, family 6, subfamily Y, member 1
100132953	[No Symbol]	[No Name]

6490	PMEL	premelanosome protein
9628	RGS6	regulator of G-protein signaling 6
1829	DSG2	desmoglein 2
27044	SND1	staphylococcal nuclease and tudor domain containing 1
3551	IKBKB	inhibitor of kappa light polypeptide gene enhancer in B-cells, kinase beta
84708	LNX1	ligand of numb-protein X 1
11040	PIM2	pim-2 oncogene
55362	TMEM63B	transmembrane protein 63B
287	ANK2	ankyrin 2, neuronal
84659	RNASE7	ribonuclease, RNase A family, 7
25823	TPSG1	tryptase gamma 1
55217	TMLHE	trimethyllysine hydroxylase, epsilon
1244	ABCC2	ATP-binding cassette, sub-family C (CFTR/MRP), member 2
387837	CLEC12B	C-type lectin domain family 12, member B
23604	DAPK2	death-associated protein kinase 2
345258	LOC345258	bridging integrator 2 pseudogene
249	ALPL	alkaline phosphatase, liver/bone/kidney
56995	TULP4	tubby like protein 4
55349	CHDH	choline dehydrogenase
100130943	[No Symbol]	[No Name]
89866	SEC16B	SEC16 homolog B (S. cerevisiae)
730512	[No Symbol]	[No Name]
100128822	LOC100128822	hypothetical LOC100128822
246135	TAF9BP1	TAF9B RNA polymerase II, TATA box binding protein (TBP)-associated factor, 31kDa pseudogene 1
200844	C3orf67	chromosome 3 open reading frame 67
149473	CCDC24	coiled-coil domain containing 24
100131236	PCBP2P3	poly(rC) binding protein 2 pseudogene 3
23647	ARFIP2	ADP-ribosylation factor interacting protein 2
26108	PYGO1	pygopus homolog 1 (Drosophila)
643965	TMEM88B	transmembrane protein 88B
127294	MYOM3	myomesin family, member 3
407023	MIR299	microRNA 299
399881	LOC399881	heterogeneous nuclear ribonucleoprotein K pseudogene
100131441	LOC100131441	coiled-coil domain containing 65 pseudogene
154197	PNLDC1	poly(A)-specific ribonuclease (PARN)-like domain containing 1

100128914	LOC100128914	hypothetical LOC100128914
92235	DUSP27	dual specificity phosphatase 27 (putative)
3651	PDX1	pancreatic and duodenal homeobox 1
646730	LOC646730	hypothetical protein LOC646730
100131193	LOC100131193	hypothetical LOC100131193
9159	PCSK7	proprotein convertase subtilisin/kexin type 7
112817	HOGA1	4-hydroxy-2-oxoglutarate aldolase 1
11082	ESM1	endothelial cell-specific molecule 1
6996	TDG	thymine-DNA glycosylase
5949	RBP3	retinol binding protein 3, interstitial
169841	ZNF169	zinc finger protein 169
391472	LOC391472	bicaudal D homolog 1 (Drosophila) pseudogene
57521	RPTOR	regulatory associated protein of MTOR, complex 1
10231	RCAN2	regulator of calcineurin 2
1264	CNN1	calponin 1, basic, smooth muscle
2798	GNRHR	gonadotropin-releasing hormone receptor
8613	PPAP2B	phosphatidic acid phosphatase type 2B
55068	ENOX1	ecto-NOX disulfide-thiol exchanger 1
388948	LOC388948	hypothetical LOC388948
54016	TUBAP	tubulin, alpha pseudogene
80206	FHOD3	formin homology 2 domain containing 3
1501	CTNND2	catenin (cadherin-associated protein), delta 2 (neural plakophilin-related arm-repeat protein)
220786	CDK7PS	cyclin-dependent kinase 7 pseudogene
100129517	RPL26P23	ribosomal protein L26 pseudogene 23
222068	TMED4	transmembrane emp24 protein transport domain containing 4
1979	EIF4EBP2	eukaryotic translation initiation factor 4E binding protein 2
91942	NDUFAF2	NADH dehydrogenase (ubiquinone) 1 alpha subcomplex, assembly factor 2
2358	FPR2	formyl peptide receptor 2
1890	TYMP	thymidine phosphorylase
91748	C14orf43	chromosome 14 open reading frame 43
8864	PER2	period homolog 2 (Drosophila)
4908	NTF3	neurotrophin 3
64284	RAB17	RAB17, member RAS oncogene family
4486	MST1R	macrophage stimulating 1 receptor (c-met-related tyrosine kinase)
158401	C9orf84	chromosome 9 open reading frame 84

374470	C12orf42	chromosome 12 open reading frame 42
128822	CST9	cystatin 9 (testatin)
116113	FOXP4	forkhead box P4
146439	CCDC64B	coiled-coil domain containing 64B
100130123	[No Symbol]	[No Name]
26701	OR2N1P	olfactory receptor, family 2, subfamily N, member 1 pseudogene
115019	SLC26A9	solute carrier family 26, member 9
2206	MS4A2	membrane-spanning 4-domains, subfamily A, member 2 (Fc fragment of IgE, high affinity I, receptor for; beta polypeptide)
390403	LOC390403	chromosome 9 open reading frame 6 pseudogene
441151	TMEM151B	transmembrane protein 151B
100132796	LOC100132796	MKI67 (FHA domain) interacting nucleolar phosphoprotein pseudogene
79258	MMEL1	membrane metallo-endopeptidase-like 1
7410	VAV2	vav 2 guanine nucleotide exchange factor
8587	OR7E2P	olfactory receptor, family 7, subfamily E, member 2 pseudogene
4436	MSH2	mutS homolog 2, colon cancer, nonpolyposis type 1 (E. coli)
7142	TNP2	transition protein 2 (during histone to protamine replacement)
400796	[No Symbol]	[No Name]
392456	LOC392456	catenin, beta like 1 pseudogene
81340	OR10Y1P	olfactory receptor, family 10, subfamily Y, member 1 pseudogene
285513	GPRIN3	GPRIN family member 3
26211	OR2F1	olfactory receptor, family 2, subfamily F, member 1
728376	LOC728376	zinc finger protein pseudogene
84690	SPATA22	spermatogenesis associated 22
133150	SULT1D1P	sulfotransferase family, cytosolic, 1D, member 1, pseudogene
23504	RIMBP2	RIMS binding protein 2
10905	MAN1A2	mannosidase, alpha, class 1A, member 2
100128988	LOC100128988	hypothetical LOC100128988
349391	CYCSP44	cytochrome c, somatic pseudogene 44
389144	OR7E130P	olfactory receptor, family 7, subfamily E, member 130 pseudogene
54715	RBFOX1	RNA binding protein, fox-1 homolog (C. elegans) 1
339168	TMEM95	transmembrane protein 95
55786	ZNF415	zinc finger protein 415
642006	LOC642006	glucuronidase, beta pseudogene
55161	TMEM33	transmembrane protein 33
339906	PRSS42	protease, serine, 42

386690	OFD1P6Y	OFD1 pseudogene 6, Y-linked
2649	NR6A1	nuclear receptor subfamily 6, group A, member 1
26507	CNNM1	cyclin M1
4017	LOXL2	lysyl oxidase-like 2
114787	GPRIN1	G protein regulated inducer of neurite outgrowth 1
577	BAI3	brain-specific angiogenesis inhibitor 3
84722	PSRC1	proline/serine-rich coiled-coil 1
51809	GALNT7	UDP-N-acetyl-alpha-D-galactosamine:polypeptide N-acetylgalactosaminyltransferase 7 (GalNAc-T7)
390101	LOC390101	mas-related G-protein coupled receptor member X3-like
6517	SLC2A4	solute carrier family 2 (facilitated glucose transporter), member 4
342184	FMN1	formin 1
58157	NGB	neuroglobin
2705	GJB1	gap junction protein, beta 1, 32kDa
729	C6	complement component 6
145447	ABHD12B	abhydrolase domain containing 12B
8787	RGS9	regulator of G-protein signaling 9
100130336	[No Symbol]	[No Name]
79698	ZMAT4	zinc finger, matrin-type 4
10447	FAM3C	family with sequence similarity 3, member C
85479	DNAJC5B	DnaJ (Hsp40) homolog, subfamily C, member 5 beta
5607	MAP2K5	mitogen-activated protein kinase kinase 5
100130490	LOC100130490	tumor protein p63 regulated 1-like pseudogene
100133169	[No Symbol]	[No Name]
5194	PEX13	peroxisomal biogenesis factor 13
284422	C19orf77	chromosome 19 open reading frame 77
71	ACTG1	actin, gamma 1
7399	USH2A	Usher syndrome 2A (autosomal recessive, mild)
2852	GPER	G protein-coupled estrogen receptor 1
197003	MGC15885	hypothetical protein MGC15885
643884	LOC643884	suppressor of cytokine signaling 5 pseudogene
64844	7-Mar	membrane-associated ring finger (C3HC4) 7
6323	SCN1A	sodium channel, voltage-gated, type I, alpha subunit
1138	CHRNA5	cholinergic receptor, nicotinic, alpha 5
642741	RPL3P7	ribosomal protein L3 pseudogene 7
118491	TTC18	tetratricopeptide repeat domain 18

100129090	RPS5P4	ribosomal protein S5 pseudogene 4
408029	C2orf27B	chromosome 2 open reading frame 27B
6581	SLC22A3	solute carrier family 22 (extraneuronal monoamine transporter), member 3
641367	LOC641367	cyclin Y-like pseudogene
145200	NCRNA00239	non-protein coding RNA 239
389123	IQCF2	IQ motif containing F2
283193	OR5AZ1P	olfactory receptor, family 5, subfamily AZ, member 1 pseudogene
1400	CRMP1	collapsin response mediator protein 1
375612	LHFPL3	lipoma HMGIC fusion partner-like 3
9312	KCNB2	potassium voltage-gated channel, Shab-related subfamily, member 2
57707	KIAA1609	KIAA1609
2547	XRCC6	X-ray repair complementing defective repair in Chinese hamster cells 6
138240	C9orf57	chromosome 9 open reading frame 57
130700	PSMB3P2	proteasome (prosome, macropain) subunit, beta type, 3 pseudogene 2
23478	SEC11A	SEC11 homolog A (S. cerevisiae)
124152	IQCK	IQ motif containing K
100129130	LOC100129130	THO complex 4 pseudogene
645687	C14orf34	chromosome 14 open reading frame 34
26973	CHORDC1	cysteine and histidine-rich domain (CHORD) containing 1
646480	FABP9	fatty acid binding protein 9, testis
390894	OR7A2P	olfactory receptor, family 7, subfamily A, member 2 pseudogene
7419	VDAC3	voltage-dependent anion channel 3
440419	LOC440419	TBC1 domain family, member 3H pseudogene
11083	DIDO1	death inducer-obliterator 1
57545	CC2D2A	coiled-coil and C2 domain containing 2A
57217	TTC7A	tetratricopeptide repeat domain 7A
55869	HDAC8	histone deacetylase 8
1281	COL3A1	collagen, type III, alpha 1
79961	DENND2D	DENN/MADD domain containing 2D
57491	AHRR	aryl-hydrocarbon receptor repressor
79908	BTNL8	butyrophilin-like 8
6238	RRBP1	ribosome binding protein 1 homolog 180kDa (dog)
100129936	LOC100129936	hypothetical LOC100129936
10647	SCGB1D2	secretoglobin, family 1D, member 2
1180	CLCN1	chloride channel 1, skeletal muscle

255239	ANKK1	ankyrin repeat and kinase domain containing 1
151534	LOC151534	hypothetical LOC151534
219771	CCNY	cyclin Y
729545	LOC729545	WBSCR19-like protein 5-like
401646	LOC401646	guanine nucleotide-binding protein G(i) subunit alpha-2-like
128344	C1orf88	chromosome 1 open reading frame 88
57449	PLEKHG5	pleckstrin homology domain containing, family G (with RhoGef domain) member 5
135112	NCOA7	nuclear receptor coactivator 7
7851	MALL	mal, T-cell differentiation protein-like
2695	GIP	gastric inhibitory polypeptide
124871	FLJ40194	hypothetical FLJ40194
54751	FBLIM1	filamin binding LIM protein 1
92345	NAF1	nuclear assembly factor 1 homolog (S. cerevisiae)
100130373	LOC100130373	hypothetical LOC100130373
4826	NNAT	neuronatin
79827	CLMP	CXADR-like membrane protein
7784	ZP3	zona pellucida glycoprotein 3 (sperm receptor)
79315	OR7E91P	olfactory receptor, family 7, subfamily E, member 91 pseudogene
91653	BOC	Boc homolog (mouse)
4301	MLLT4	myeloid/lymphoid or mixed-lineage leukemia (trithorax homolog, Drosophila); translocated to, 4
205272	CNN2P8	calponin 2 pseudogene 8
390988	[No Symbol]	[No Name]
118812	MORN4	MORN repeat containing 4
25875	LETMD1	LETM1 domain containing 1
647123	UQCRFS1P2	ubiquinol-cytochrome c reductase, Rieske iron-sulfur polypeptide 1 pseudogene 2
10113	PREB	prolactin regulatory element binding
55698	RADIL	Ras association and DIL domains
27075	TSPAN13	tetraspanin 13
26245	OR2M4	olfactory receptor, family 2, subfamily M, member 4
10379	IRF9	interferon regulatory factor 9
8694	DGAT1	diacylglycerol O-acyltransferase 1
100132621	LOC100132621	family with sequence similarity 54, member A pseudogene
5105	PCK1	phosphoenolpyruvate carboxykinase 1 (soluble)
441054	C4orf47	chromosome 4 open reading frame 47
441907	LOC441907	ribosomal protein L6 pseudogene

5225	PGC	progastricsin (pepsinogen C)
139189	DGKK	diacylglycerol kinase, kappa
59269	HIVEP3	human immunodeficiency virus type I enhancer binding protein 3
643031	LOC643031	mitochondrially encoded NADH dehydrogenase 5 pseudogene
8418	CMAHP	cytidine monophospho-N-acetylneuraminic acid hydroxylase, pseudogene
83878	USHBP1	Usher syndrome 1C binding protein 1
49860	CRNN	cornulin
240	ALOX5	arachidonate 5-lipoxygenase
353006	HCG2P6	HLA complex group 2 pseudogene 6
79930	DOK3	docking protein 3
728191	[No Symbol]	[No Name]
57477	SHROOM4	shroom family member 4
100128588	LOC100128588	PIN2/TERF1 interacting, telomerase inhibitor 1 pseudogene
55117	SLC6A15	solute carrier family 6 (neutral amino acid transporter), member 15
79925	SPEF2	sperm flagellar 2
774	CACNA1B	calcium channel, voltage-dependent, N type, alpha 1B subunit
401260	FLJ41649	hypothetical LOC401260
149233	IL23R	interleukin 23 receptor
27089	UQCRQ	ubiquinol-cytochrome c reductase, complex III subunit VII, 9.5kDa
494143	CHAC2	ChaC, cation transport regulator homolog 2 (E. coli)
4323	MMP14	matrix metallopeptidase 14 (membrane-inserted)
400943	LOC400943	hypothetical LOC400943
55222	LRRC20	leucine rich repeat containing 20
79805	VASH2	vasohibin 2
6770	STAR	steroidogenic acute regulatory protein
729650	METTL15P3	methyltransferase like 15 pseudogene 3
4072	EPCAM	epithelial cell adhesion molecule
85349	KRT121P	keratin 121 pseudogene
158809	MAGEB6	melanoma antigen family B, 6
100133311	LOC100133311	hypothetical LOC100133311
100128051	[No Symbol]	[No Name]
9547	CXCL14	chemokine (C-X-C motif) ligand 14
84517	ARPM1	actin related protein M1
53842	CLDN22	claudin 22
5697	PYY	peptide YY

553	AVPR1B	arginine vasopressin receptor 1B
100133208	[No Symbol]	[No Name]
100128546	LOC100128546	glutaredoxin 5 pseudogene
214	ALCAM	activated leukocyte cell adhesion molecule
100128987	LOC100128987	hypothetical LOC100128987
55259	CASC1	cancer susceptibility candidate 1
114786	XKR4	XK, Kell blood group complex subunit-related family, member 4
9206	RAD17P2	RAD17 homolog (S. pombe) pseudogene 2
643911	CRNDE	colorectal neoplasia differentially expressed (non-protein coding)
90203	SNX21	sorting nexin family member 21
10181	RBM5	RNA binding motif protein 5
84279	PRADC1	protease-associated domain containing 1
8993	PGLYRP1	peptidoglycan recognition protein 1
26740	OR1J2	olfactory receptor, family 1, subfamily J, member 2
83873	GPR61	G protein-coupled receptor 61
729894	ELMO2P1	engulfment and cell motility 2 pseudogene 1
5205	ATP8B1	ATPase, aminophospholipid transporter, class I, type 8B, member 1
54700	RRN3	RRN3 RNA polymerase I transcription factor homolog (S. cerevisiae)
5730	PTGDS	prostaglandin D2 synthase 21kDa (brain)
9846	GAB2	GRB2-associated binding protein 2
147872	CCDC155	coiled-coil domain containing 155
1282	COL4A1	collagen, type IV, alpha 1
79838	TMC5	transmembrane channel-like 5
4327	MMP19	matrix metallopeptidase 19
728250	[No Symbol]	[No Name]
401021	LOC401021	hypothetical LOC401021
375061	FAM89A	family with sequence similarity 89, member A
400026	LOC400026	protein-kinase, interferon-inducible double stranded RNA dependent inhibitor, repressor of (P58 repressor) pseudogene
50853	VILL	villin-like
3382	ICA1	islet cell autoantigen 1, 69kDa
100128043	LOC100128043	Fas associated factor family member 2 pseudogene
2104	ESRRG	estrogen-related receptor gamma
56000	NXF3	nuclear RNA export factor 3
100128834	LOC100128834	CDC-like kinase 3 pseudogene
1491	CTH	cystathionase (cystathionine gamma-lyase)

123041	SLC24A4	solute carrier family 24 (sodium/potassium/calcium exchanger), member 4
960	CD44	CD44 molecule (Indian blood group)
8910	SGCE	sarcoglycan, epsilon
284836	NCRNA00319	non-protein coding RNA 319
392221	CHCHD2P10	coiled-coil-helix-coiled-coil-helix domain containing 2 pseudogene 10
57575	PCDH10	protocadherin 10
25833	POU2F3	POU class 2 homeobox 3
100130353	LOC100130353	hippocampus abundant transcript-like 1 pseudogene
285313	IGSF10	immunoglobulin superfamily, member 10
100130666	[No Symbol]	[No Name]
5132	PDC	phosducin
642487	LOC642487	H1 histone family, member O, oocyte-specific pseudogene
386653	IL31	interleukin 31
169026	SLC30A8	solute carrier family 30 (zinc transporter), member 8
284680	C1orf111	chromosome 1 open reading frame 111
10926	DBF4	DBF4 homolog (S. cerevisiae)
51455	REV1	REV1 homolog (S. cerevisiae)
221301	FAM26D	family with sequence similarity 26, member D
388591	RNF207	ring finger protein 207
55770	EXOC2	exocyst complex component 2
9265	CYTH3	cytohesin 3
284325	C19orf54	chromosome 19 open reading frame 54
79906	MORN1	MORN repeat containing 1
1179	CLCA1	chloride channel accessory 1
11211	FZD10	frizzled family receptor 10
83729	INHBE	inhibin, beta E
63976	PRDM16	PR domain containing 16
5307	PITX1	paired-like homeodomain 1
9369	NRXN3	neurexin 3
59351	PBOV1	prostate and breast cancer overexpressed 1
9626	GUCA1C	guanylate cyclase activator 1C
51555	PEX5L	peroxisomal biogenesis factor 5-like
140836	BANF2	barrier to autointegration factor 2
387893	SETD8	SET domain containing (lysine methyltransferase) 8
100130405	HMGB3P26	high mobility group box 3 pseudogene 26

83594	NUDT12	nudix (nucleoside diphosphate linked moiety X)-type motif 12
643718	[No Symbol]	[No Name]
6792	CDKL5	cyclin-dependent kinase-like 5
84536	C21orf67	chromosome 21 open reading frame 67
255101	CCDC108	coiled-coil domain containing 108
7005	TEAD3	TEA domain family member 3
100128995	[No Symbol]	[No Name]
136853	SRCRB4D	scavenger receptor cysteine rich domain containing, group B (4 domains)
9931	HELZ	helicase with zinc finger
286023	FLJ40288	hypothetical FLJ40288
474345	GIMAP3P	GTPase, IMAP family member 3 pseudogene
642818	ST13P21	suppression of tumorigenicity 13 (colon carcinoma) (Hsp70 interacting protein) pseudogene 21
387103	CENPW	centromere protein W
56907	SPIRE1	spire homolog 1 (Drosophila)
286046	XKR6	XK, Kell blood group complex subunit-related family, member 6
10205	MPZL2	myelin protein zero-like 2
642614	LOC642614	chromosome 2 open reading frame 69 pseudogene
83869	TTTY14	testis-specific transcript, Y-linked 14 (non-protein coding)
339768	ESPNL	espin-like
255762	PDZD9	PDZ domain containing 9
23194	FBXL7	F-box and leucine-rich repeat protein 7
27112	FAM155B	family with sequence similarity 155, member B
1144	CHRND	cholinergic receptor, nicotinic, delta
3679	ITGA7	integrin, alpha 7
113675	SDSL	serine dehydratase-like
58155	PTBP2	polypyrimidine tract binding protein 2
148979	GLIS1	GLIS family zinc finger 1
152877	FAM53A	family with sequence similarity 53, member A
1294	COL7A1	collagen, type VII, alpha 1
100129405	MSTO2P	misato homolog 2 pseudogene
339766	HEATR7B1	HEAT repeat containing 7B1
4782	NFIC	nuclear factor I/C (CCAAT-binding transcription factor)
3161	HMMR	hyaluronan-mediated motility receptor (RHAMM)
317775	CHORDC2P	cysteine and histidine-rich domain (CHORD) containing 2 pseudogene
391657	LOC391657	dipeptidyl-peptidase 3 pseudogene

26508	HEYL	hairy/enhancer-of-split related with YRPW motif-like
145837	LOC145837	hypothetical LOC145837
100128705	AK4P4	adenylate kinase 4 pseudogene 4
2819	GPD1	glycerol-3-phosphate dehydrogenase 1 (soluble)
23247	KIAA0556	KIAA0556
83447	SLC25A31	solute carrier family 25 (mitochondrial carrier; adenine nucleotide translocator), member 31
100126531	TRNAG-GCC	transfer RNA glycine (anticodon GCC)
100130090	LOC100130090	chromosome 10 open reading frame 78 pseudogene
25943	C20orf194	chromosome 20 open reading frame 194
100133019	[No Symbol]	[No Name]
2779	GNAT1	guanine nucleotide binding protein (G protein), alpha transducing activity polypeptide 1
100128434	[No Symbol]	[No Name]
83543	AIF1L	allograft inflammatory factor 1-like
1286	COL4A4	collagen, type IV, alpha 4
374407	DNAJB13	DnaJ (Hsp40) homolog, subfamily B, member 13
54959	ODAM	odontogenic, ameloblast asssociated
80108	ZFP2	zinc finger protein 2 homolog (mouse)
400682	LOC400682	zinc finger protein 100-like
100131656	LOC100131656	syndecan binding protein (syntenin) pseudogene
80705	TSGA10	testis specific, 10
116085	SLC22A12	solute carrier family 22 (organic anion/urate transporter), member 12
5446	PON3	paraoxonase 3
29942	PURG	purine-rich element binding protein G
646813	LOC646813	DEAH (Asp-Glu-Ala-His) box polypeptide 9 pseudogene
389610	XKR5	XK, Kell blood group complex subunit-related family, member 5
645840	TXNRD3NB	thioredoxin reductase 3 neighbor
406984	MIR200B	microRNA 200b
440706	[No Symbol]	[No Name]
55930	MYO5C	myosin VC
79776	ZFHX4	zinc finger homeobox 4
100128725	LOC100128725	apolipoprotein O pseudogene
5671	PSG3	pregnancy specific beta-1-glycoprotein 3
89872	AQP10	aquaporin 10
460	ASTN1	astrotactin 1
28337	IGHVIV-44-1	immunoglobulin heavy variable (IV)-44-1 (pseudogene)

1571	CYP2E1	cytochrome P450, family 2, subfamily E, polypeptide 1
368	ABCC6	ATP-binding cassette, sub-family C (CFTR/MRP), member 6
260429	PRSS33	protease, serine, 33
100131818	LOC100131818	similar to hCG1644435
116369	SLC26A8	solute carrier family 26, member 8
91807	MYLK3	myosin light chain kinase 3
134548	ANKRD43	ankyrin repeat domain 43
23349	KIAA1045	KIAA1045
392255	GDF6	growth differentiation factor 6
5048	PAFAH1B1	platelet-activating factor acetylhydrolase 1b, regulatory subunit 1 (45kDa)
4828	NMB	neuromedin B
375057	C1orf95	chromosome 1 open reading frame 95
727827	[No Symbol]	[No Name]
51144	HSD17B12	hydroxysteroid (17-beta) dehydrogenase 12
83468	GLT8D2	glycosyltransferase 8 domain containing 2
100128596	LOC100128596	NADH dehydrogenase [ubiquinone] 1 beta subcomplex subunit 9-like
11216	AKAP10	A kinase (PRKA) anchor protein 10
9721	GPRIN2	G protein regulated inducer of neurite outgrowth 2
100130601	[No Symbol]	[No Name]
9499	MYOT	myotilin
26689	OR4D1	olfactory receptor, family 4, subfamily D, member 1
6396	SEC13	SEC13 homolog (S. cerevisiae)
6195	RPS6KA1	ribosomal protein S6 kinase, 90kDa, polypeptide 1
27445	PCLO	piccolo (presynaptic cytomatrix protein)
645634	ANO7L1	anoctamin 7-like 1
7444	VRK2	vaccinia related kinase 2
2199	FBLN2	fibulin 2
138046	RALYL	RALY RNA binding protein-like
80746	TSEN2	tRNA splicing endonuclease 2 homolog (S. cerevisiae)
659	BMPR2	bone morphogenetic protein receptor, type II (serine/threonine kinase)
83417	FCRL4	Fc receptor-like 4
150372	NFAM1	NFAT activating protein with ITAM motif 1
127733	UBXN10	UBX domain protein 10
644325	LOC644325	disintegrin and metalloprotease domain protein pseudogene
220929	ZNF438	zinc finger protein 438

28232	SLCO3A1	solute carrier organic anion transporter family, member 3A1
63978	PRDM14	PR domain containing 14
10490	VTI1B	vesicle transport through interaction with t-SNAREs homolog 1B (yeast)
843	CASP10	caspase 10, apoptosis-related cysteine peptidase
832	CAPZB	capping protein (actin filament) muscle Z-line, beta
100132328	[No Symbol]	[No Name]
151176	FAM132B	family with sequence similarity 132, member B
63923	TNN	tenascin N
80125	CCDC33	coiled-coil domain containing 33
100130138	[No Symbol]	[No Name]
55504	TNFRSF19	tumor necrosis factor receptor superfamily, member 19
54454	ATAD2B	ATPase family, AAA domain containing 2B
6204	RPS10	ribosomal protein S10
25806	VAX2	ventral anterior homeobox 2
644237	GAPDHP33	glyceraldehyde 3 phosphate dehydrogenase pseudogene 33
6002	RGS12	regulator of G-protein signaling 12
387715	ARMS2	age-related maculopathy susceptibility 2
727894	[No Symbol]	[No Name]
100133058	[No Symbol]	[No Name]
57628	DPP10	dipeptidyl-peptidase 10 (non-functional)
129656	LOC129656	CREB regulated transcription coactivator 1 pseudogene
25979	DHRS7B	dehydrogenase/reductase (SDR family) member 7B
9487	PIGL	phosphatidylinositol glycan anchor biosynthesis, class L
2033	EP300	E1A binding protein p300
2978	GUCA1A	guanylate cyclase activator 1A (retina)
7102	TSPAN7	tetraspanin 7
4589	MUC7	mucin 7, secreted
26011	ODZ4	odz, odd Oz/ten-m homolog 4 (Drosophila)
5552	SRGN	serglycin
83733	SLC25A18	solute carrier family 25 (mitochondrial carrier), member 18
442525	SLC25A5P5	solute carrier family 25 (mitochondrial carrier; adenine nucleotide translocator), member 5 pseudogene 5
729497	LOC729497	nuclear receptor coactivator 4 pseudogene
10332	CLEC4M	C-type lectin domain family 4, member M
344838	PAQR9	progestin and adipoQ receptor family member IX
647016	EIF2S2P7	eukaryotic translation initiation factor 2, subunit 2 beta pseudogene 7

100133062	LOC100133062	chromosome 6 open reading frame 146 pseudogene
728896	[No Symbol]	[No Name]
168975	CNBD1	cyclic nucleotide binding domain containing 1
9515	STXBP5L	syntaxin binding protein 5-like
100133268	LOC100133268	HAUS augmin-like complex subunit 6-like
79605	PGBD5	piggyBac transposable element derived 5
389599	STRADBP1	STE20-related kinase adaptor beta pseudogene 1
25976	TIPARP	TCDD-inducible poly(ADP-ribose) polymerase
2853	GPR31	G protein-coupled receptor 31
6159	RPL29	ribosomal protein L29
125488	TTC39C	tetratricopeptide repeat domain 39C
3268	AGFG2	ArfGAP with FG repeats 2
89765	RSPH1	radial spoke head 1 homolog (Chlamydomonas)
9271	PIWIL1	piwi-like 1 (Drosophila)
131831	FAM194A	family with sequence similarity 194, member A
647580	[No Symbol]	[No Name]
8664	EIF3D	eukaryotic translation initiation factor 3, subunit D
89932	PAPLN	papilin, proteoglycan-like sulfated glycoprotein
84701	COX4I2	cytochrome c oxidase subunit IV isoform 2 (lung)
11280	SCN11A	sodium channel, voltage-gated, type XI, alpha subunit
158248	TTC16	tetratricopeptide repeat domain 16
128861	C20orf71	chromosome 20 open reading frame 71
353299	RGSL1	regulator of G-protein signaling like 1
5992	RFX4	regulatory factor X, 4 (influences HLA class II expression)
360030	NANOGNB	NANOG neighbor homeobox
1129	CHRM2	cholinergic receptor, muscarinic 2
283551	C14orf182	chromosome 14 open reading frame 182
51493	C22orf28	chromosome 22 open reading frame 28
127623	OR2B11	olfactory receptor, family 2, subfamily B, member 11
359739	MRPL3P1	mitochondrial ribosomal protein L3 pseudogene 1
10505	SEMA4F	sema domain, immunoglobulin domain (Ig), transmembrane domain (TM) and short cytoplasmic domain, (semaphorin) 4F
387694	SH2D4B	SH2 domain containing 4B
130940	CCDC148	coiled-coil domain containing 148
56267	CCBL2	cysteine conjugate-beta lyase 2
28346	IGHVIII-38-1	immunoglobulin heavy variable (III)-38-1 (pseudogene)

100131347	LOC100131347	RAD52 motif 1 pseudogene
729707	LOC729707	transmembrane and tetratricopeptide repeat containing 4 pseudogene
56914	OTOR	otoraplin
644451	LOC644451	SHQ1 homolog (S. cerevisiae) pseudogene
5771	PTPN2	protein tyrosine phosphatase, non-receptor type 2
83439	TCF7L1	transcription factor 7-like 1 (T-cell specific, HMG-box)
8894	EIF2S2	eukaryotic translation initiation factor 2, subunit 2 beta, 38kDa
23148	NACAD	NAC alpha domain containing
3269	HRH1	histamine receptor H1
3615	IMPDH2	IMP (inosine 5'-monophosphate) dehydrogenase 2
116154	PHACTR3	phosphatase and actin regulator 3
1993	ELAVL2	ELAV (embryonic lethal, abnormal vision, Drosophila)-like 2 (Hu antigen B)
115123	3-Mar	membrane-associated ring finger (C3HC4) 3
282776	OR8V1P	olfactory receptor, family 8, subfamily V, member 1 pseudogene
121273	C12orf54	chromosome 12 open reading frame 54
376132	LRRC10	leucine rich repeat containing 10
8092	ALX1	ALX homeobox 1
3568	IL5RA	interleukin 5 receptor, alpha
647177	LOC647177	glutathione S-transferase A3-like
3339	HSPG2	heparan sulfate proteoglycan 2
729977	[No Symbol]	[No Name]
441027	TMEM150C	transmembrane protein 150C
22887	FOXJ3	forkhead box J3
100132318	[No Symbol]	[No Name]
10011	SRA1	steroid receptor RNA activator 1
645646	FAM32C	family with sequence similarity 32, member C (pseudogene)
1045	CDX2	caudal type homeobox 2
9563	H6PD	hexose-6-phosphate dehydrogenase (glucose 1-dehydrogenase)
100130044	LOC100130044	chromosome 7 open reading frame 63 pseudogene
138065	RNF183	ring finger protein 183
79033	ERI3	ERI1 exoribonuclease family member 3
80325	ABTB1	ankyrin repeat and BTB (POZ) domain containing 1
1506	CTRL	chymotrypsin-like
8789	FBP2	fructose-1,6-bisphosphatase 2
6699	SPRR1B	small proline-rich protein 1B

6262	RYR2	ryanodine receptor 2 (cardiac)
9649	RALGPS1	Ral GEF with PH domain and SH3 binding motif 1
100130801	LOC100130801	lupus La protein-like
124359	CDYL2	chromodomain protein, Y-like 2
167681	PRSS35	protease, serine, 35
9170	LPAR2	lysophosphatidic acid receptor 2
54857	GDPD2	glycerophosphodiester phosphodiesterase domain containing 2
392510	YWHAQP8	YWHAQ pseudogene 8
100132009	LOC100132009	Pentatricopeptide repeat domain 3 pseudogene
7554	ZNF8	zinc finger protein 8
404636	FAM45A	family with sequence similarity 45, member A
84791	C1orf97	chromosome 1 open reading frame 97
81706	PPP1R14C	protein phosphatase 1, regulatory (inhibitor) subunit 14C
138162	C9orf116	chromosome 9 open reading frame 116
286075	ZNF707	zinc finger protein 707
8653	DDX3Y	DEAD (Asp-Glu-Ala-Asp) box polypeptide 3, Y-linked
55809	TRERF1	transcriptional regulating factor 1
100130042	LOC100130042	methylenetetrahydrofolate dehydrogenase (NADP+ dependent) 2, methenyltetrahydrofolate cyclohydrolase pseudogene
388276	LOC388276	hCG2045437
1111	CHEK1	CHK1 checkpoint homolog (S. pombe)
57088	PLSCR4	phospholipid scramblase 4
100130718	RPL34P26	ribosomal protein L34 pseudogene 26
6586	SLIT3	slit homolog 3 (Drosophila)
10103	TSPAN1	tetraspanin 1
317727	LOC317727	ataxin 2 related protein pseudogene
729975	FLJ30403	hypothetical LOC729975
54954	FAM120C	family with sequence similarity 120C
149428	BNIPL	BCL2/adenovirus E1B 19kD interacting protein like
222950	C7orf51	chromosome 7 open reading frame 51
390597	LETM1P1	leucine zipper-EF-hand containing transmembrane protein 1, pseudogene 1
5740	PTGIS	prostaglandin I2 (prostacyclin) synthase
144699	FBXL14	F-box and leucine-rich repeat protein 14
677	ZFP36L1	zinc finger protein 36, C3H type-like 1
6283	S100A12	S100 calcium binding protein A12
26548	ITGB1BP2	integrin beta 1 binding protein (melusin) 2

11012	KLK11	kallikrein-related peptidase 11
149837	LOC149837	hypothetical LOC149837
78986	DUSP26	dual specificity phosphatase 26 (putative)
100129126	LOC100129126	hypothetical LOC100129126
79912	PYROXD1	pyridine nucleotide-disulphide oxidoreductase domain 1
127343	DMBX1	diencephalon/mesencephalon homeobox 1
222008	VSTM2A	V-set and transmembrane domain containing 2A
51300	C3orf1	chromosome 3 open reading frame 1
56269	IRGC	immunity-related GTPase family, cinema
728573	LOC728573	hypothetical protein LOC728573
727977	[No Symbol]	[No Name]
57482	KIAA1211	KIAA1211
93190	C1orf158	chromosome 1 open reading frame 158
56243	KIAA1217	KIAA1217
401541	CENPP	centromere protein P
1936	EEF1D	eukaryotic translation elongation factor 1 delta (guanine nucleotide exchange protein)
84230	LRRC8C	leucine rich repeat containing 8 family, member C
65061	CDK15	cyclin-dependent kinase 15
403284	OR6C68	olfactory receptor, family 6, subfamily C, member 68
23543	RBFOX2	RNA binding protein, fox-1 homolog (C. elegans) 2
285195	SLC9A9	solute carrier family 9 (sodium/hydrogen exchanger), member 9
9767	PHF16	PHD finger protein 16
9420	CYP7B1	cytochrome P450, family 7, subfamily B, polypeptide 1
345557	PLCXD3	phosphatidylinositol-specific phospholipase C, X domain containing 3
160065	PATE1	prostate and testis expressed 1
55917	CTTNBP2NL	CTTNBP2 N-terminal like
5898	RALA	v-ral simian leukemia viral oncogene homolog A (ras related)
5207	PFKFB1	6-phosphofructo-2-kinase/fructose-2,6-biphosphatase 1
146771	TCAM1P	testicular cell adhesion molecule 1 homolog (mouse), pseudogene
2168	FABP1	fatty acid binding protein 1, liver
255119	C4orf22	chromosome 4 open reading frame 22
221481	C6orf81	chromosome 6 open reading frame 81
8630	HSD17B6	hydroxysteroid (17-beta) dehydrogenase 6 homolog (mouse)
100131892	[No Symbol]	[No Name]
728621	CCDC30	coiled-coil domain containing 30

338674	OR5F1	olfactory receptor, family 5, subfamily F, member 1
100128977	LOC100128977	hypothetical LOC100128977
114792	KLHL32	kelch-like 32 (Drosophila)
391696	LOC391696	synaptic glycoprotein SC2-like
91695	RRP7B	ribosomal RNA processing 7 homolog B (S. cerevisiae)
406992	MIR210	microRNA 210
10279	PRSS16	protease, serine, 16 (thymus)
218	ALDH3A1	aldehyde dehydrogenase 3 family, member A1
3752	KCND3	potassium voltage-gated channel, Shal-related subfamily, member 3
100131195	LOC100131195	hypothetical protein LOC100131195
25859	PART1	prostate androgen-regulated transcript 1 (non-protein coding)
10216	PRG4	proteoglycan 4
261734	NPHP4	nephronophthisis 4
340273	ABCB5	ATP-binding cassette, sub-family B (MDR/TAP), member 5
10542	HBXIP	hepatitis B virus x interacting protein
401114	FLJ35816	FLJ35816 protein
100132146	LOC100132146	hypothetical LOC100132146
644154	RPL10AP8	ribosomal protein L10a pseudogene 8
6913	TBX15	T-box 15
5161	PDHA2	pyruvate dehydrogenase (lipoamide) alpha 2
1638	DCT	dopachrome tautomerase (dopachrome delta-isomerase, tyrosine-related protein 2)
392145	LOC392145	exosome component 6 pseudogene
54212	SNTG1	syntrophin, gamma 1
9068	ANGPTL1	angiopoietin-like 1
8675	STX16	syntaxin 16
91734	IDI2	isopentenyl-diphosphate delta isomerase 2
440518	LOC440518	golgin A2 pseudogene
786	CACNG1	calcium channel, voltage-dependent, gamma subunit 1
128434	VSTM2L	V-set and transmembrane domain containing 2 like
7099	TLR4	toll-like receptor 4
23521	RPL13A	ribosomal protein L13a
643342	LOC643342	ATM interactor pseudogene
143666	LOC143666	hypothetical LOC143666
27010	TPK1	thiamin pyrophosphokinase 1
54788	DNAJB12	DnaJ (Hsp40) homolog, subfamily B, member 12

10762	NUP50	nucleoporin 50kDa
653645	DHX40P1	DEAH (Asp-Glu-Ala-His) box polypeptide 40 pseudogene 1
2922	GRP	gastrin-releasing peptide
414899	BLID	BH3-like motif containing, cell death inducer
60482	SLC5A7	solute carrier family 5 (choline transporter), member 7
51078	THAP4	THAP domain containing 4
326297	RPL26P2	ribosomal protein L26 pseudogene 2
100130184	[No Symbol]	[No Name]
4544	MTNR1B	melatonin receptor 1B
64409	WBSCR17	Williams-Beuren syndrome chromosome region 17
643471	KRT18P12	keratin 18 pseudogene 12
84173	ELMOD3	ELMO/CED-12 domain containing 3
114088	TRIM9	tripartite motif containing 9
10642	IGF2BP1	insulin-like growth factor 2 mRNA binding protein 1
55277	FGGY	FGGY carbohydrate kinase domain containing
8543	LMO4	LIM domain only 4
401191	FLJ46010	FLJ46010 protein
647281	[No Symbol]	[No Name]
4595	MUTYH	mutY homolog (E. coli)
100128066	[No Symbol]	[No Name]
257062	TMEM146	transmembrane protein 146
6566	SLC16A1	solute carrier family 16, member 1 (monocarboxylic acid transporter 1)
1932	EEF1B2P1	eukaryotic translation elongation factor 1 beta 2 pseudogene 1
100128852	[No Symbol]	[No Name]
84310	C7orf50	chromosome 7 open reading frame 50
60313	GPBP1L1	GC-rich promoter binding protein 1-like 1
401498	TMEM215	transmembrane protein 215
340543	TCEAL5	transcription elongation factor A (SII)-like 5
390035	OR52K3P	olfactory receptor, family 52, subfamily K, member 3 pseudogene
253724	GNN	Grp94 neighboring nucleotidase pseudogene
64168	NECAB1	N-terminal EF-hand calcium binding protein 1
5710	PSMD4	proteasome (prosome, macropain) 26S subunit, non-ATPase, 4
344905	ATP13A5	ATPase type 13A5
9615	GDA	guanine deaminase
9865	TRIL	TLR4 interactor with leucine-rich repeats

10777	ARPP21	cAMP-regulated phosphoprotein, 21kDa
100126521	TRNAW-CCA	transfer RNA tryptophan (anticodon CCA)
400593	FLJ43944	FLJ43944 protein
131405	TRIM71	tripartite motif containing 71
3560	IL2RB	interleukin 2 receptor, beta
55714	ODZ3	odz, odd Oz/ten-m homolog 3 (Drosophila)
54052	EIF4A1P1	eukaryotic translation initiation factor 4A1 pseudogene 1
57157	PHTF2	putative homeodomain transcription factor 2
642337	FTLP17	ferritin, light polypeptide pseudogene 17
360203	GLT6D1	glycosyltransferase 6 domain containing 1
399783	LOC399783	zinc finger protein 532 pseudogene
1360	CPB1	carboxypeptidase B1 (tissue)
2915	GRM5	glutamate receptor, metabotropic 5
646956	LOC646956	heterogeneous nuclear ribonucleoprotein C (C1/C2) pseudogene
260425	MAGI3	membrane associated guanylate kinase, WW and PDZ domain containing 3
83605	CCM2	cerebral cavernous malformation 2
203102	ADAM32	ADAM metallopeptidase domain 32
116379	IL22RA2	interleukin 22 receptor, alpha 2
353497	POLN	polymerase (DNA directed) nu
642511	LOC642511	hypothetical protein LOC642511
400533	FLJ26245	hypothetical LOC400533
126206	NLRP5	NLR family, pyrin domain containing 5
220441	RNF152	ring finger protein 152
100126527	TRNAR-CCU	transfer RNA arginine (anticodon CCU)
647099	RPL23AP42	ribosomal protein L23a pseudogene 42
387914	SHISA2	shisa homolog 2 (Xenopus laevis)
100132656	LOC100132656	FK506 binding protein 4, 59kDa pseudogene
379007	RBMY2SP	RNA binding motif protein, Y-linked, family 2, member S pseudogene
221823	PRPS1L1	phosphoribosyl pyrophosphate synthetase 1-like 1
2984	GUCY2C	guanylate cyclase 2C (heat stable enterotoxin receptor)
10267	RAMP1	receptor (G protein-coupled) activity modifying protein 1
255967	PAN3	PAN3 poly(A) specific ribonuclease subunit homolog (S. cerevisiae)
10669	CGREF1	cell growth regulator with EF-hand domain 1
339483	MTMR9LP	myotubularin related protein 9-like, pseudogene
200810	ALG1L	asparagine-linked glycosylation 1-like

58189	WFDC1	WAP four-disulfide core domain 1
390928	PAPL	iron/zinc purple acid phosphatase-like protein
113146	AHNAK2	AHNAK nucleoprotein 2
441643	LOC441643	p53 and DNA damage-regulated protein 1-like
340460	KRT18P24	keratin 18 pseudogene 24
80019	UBTD1	ubiquitin domain containing 1
7224	TRPC5	transient receptor potential cation channel, subfamily C, member 5
114789	SLC25A25	solute carrier family 25 (mitochondrial carrier; phosphate carrier), member 25
389197	C4orf50	chromosome 4 open reading frame 50
81578	COL21A1	collagen, type XXI, alpha 1
6809	STX3	syntaxin 3
3763	KCNJ6	potassium inwardly-rectifying channel, subfamily J, member 6
5746	PTH2R	parathyroid hormone 2 receptor
199720	GGN	gametogenetin
10690	FUT9	fucosyltransferase 9 (alpha (1,3) fucosyltransferase)
646995	LOC646995	tripartite motif-containing protein 38-like
285759	FLJ34503	hypothetical FLJ34503
1119	CHKA	choline kinase alpha
57007	CXCR7	chemokine (C-X-C motif) receptor 7
441239	LOC441239	hypothetical protein LOC441239
391387	RPSAP28	ribosomal protein SA pseudogene 28
63876	PKNOX2	PBX/knotted 1 homeobox 2
132200	C3orf49	chromosome 3 open reading frame 49
126308	MOBKL2A	MOB1, Mps One Binder kinase activator-like 2A (yeast)
7448	VTN	vitronectin
2048	EPHB2	EPH receptor B2
407	ARR3	arrestin 3, retinal (X-arrestin)
2494	NR5A2	nuclear receptor subfamily 5, group A, member 2
108	ADCY2	adenylate cyclase 2 (brain)
10633	RASL10A	RAS-like, family 10, member A
7025	NR2F1	nuclear receptor subfamily 2, group F, member 1
3361	HTR5A	5-hydroxytryptamine (serotonin) receptor 5A
54952	TRNAU1AP	tRNA selenocysteine 1 associated protein 1
8829	NRP1	neuropilin 1
9351	SLC9A3R2	solute carrier family 9 (sodium/hydrogen exchanger), member 3 regulator 2

7080	NKX2-1	NK2 homeobox 1
100131576	[No Symbol]	[No Name]
255308	LOC255308	eukaryotic translation initiation factor 2, subunit 3 gamma, 52kDa pseudogene
57462	KIAA1161	KIAA1161
100128921	[No Symbol]	[No Name]
54810	GIPC2	GIPC PDZ domain containing family, member 2
100130502	LOC100130502	hypothetical LOC100130502
391053	CAPNS1P1	calpain, small subunit 1 pseudogene 1
100128547	[No Symbol]	[No Name]
51305	KCNK9	potassium channel, subfamily K, member 9
644743	LOC644743	keratin 8 pseudogene
121130	OR10P1	olfactory receptor, family 10, subfamily P, member 1
130589	GALM	galactose mutarotase (aldose 1-epimerase)
60673	C12orf44	chromosome 12 open reading frame 44
642554	LOC642554	leucine rich repeat containing 59 pseudogene
10178	ODZ1	odz, odd Oz/ten-m homolog 1 (Drosophila)
27128	CYTH4	cytohesin 4
92691	TMEM169	transmembrane protein 169
6369	CCL24	chemokine (C-C motif) ligand 24
26516	RPS5P1	ribosomal protein S5 pseudogene 1
391722	LOC391722	calcium-dependent protein kinase 7-like
285989	ZNF789	zinc finger protein 789
390892	OR7A10	olfactory receptor, family 7, subfamily A, member 10
4651	MYO10	myosin X
730036	[No Symbol]	[No Name]
93166	PRDM6	PR domain containing 6
401123	FLJ45721	hypothetical LOC401123
51251	NT5C3	5'-nucleotidase, cytosolic III
8515	ITGA10	integrin, alpha 10
100130316	LOC100130316	putative RNA-binding protein 15-like
6440	SFTPC	surfactant protein C
344191	EVX2	even-skipped homeobox 2
55584	CHRNA9	cholinergic receptor, nicotinic, alpha 9
643751	LOC643751	cell division cycle 42 pseudogene
5950	RBP4	retinol binding protein 4, plasma

200539	ANKRD23	ankyrin repeat domain 23
79308	OR4P1P	olfactory receptor, family 4, subfamily P, member 1 pseudogene
100130605	[No Symbol]	[No Name]
100129033	LOC100129033	QIQN5815
55315	SLC29A3	solute carrier family 29 (nucleoside transporters), member 3
1586	CYP17A1	cytochrome P450, family 17, subfamily A, polypeptide 1
81025	GJA9	gap junction protein, alpha 9, 59kDa
653381	SORD2	SORD pseudogene
619373	MBOAT4	membrane bound O-acyltransferase domain containing 4
90865	IL33	interleukin 33
441732	[No Symbol]	[No Name]
285600	KIAA0825	KIAA0825
7172	TPMT	thiopurine S-methyltransferase
283711	LOC283711	ubiquitin-conjugating enzyme E2C pseudogene
2609	GAPDHP67	glyceraldehyde 3 phosphate dehydrogenase pseudogene 67
100130329	LOC100130329	solute carrier family 41, member 2 pseudogene
100128017	TULP3P1	tubby like protein 3 pseudogene 1
140456	ASB11	ankyrin repeat and SOCS box containing 11
221091	LRRN4CL	LRRN4 C-terminal like
51327	AHSP	alpha hemoglobin stabilizing protein
401474	SAMD12	sterile alpha motif domain containing 12
145165	ST13P4	suppression of tumorigenicity 13 (colon carcinoma) (Hsp70 interacting protein) pseudogene 4
80256	KIAA1539	KIAA1539
7075	TIE1	tyrosine kinase with immunoglobulin-like and EGF-like domains 1
83690	CRISPLD1	cysteine-rich secretory protein LCCL domain containing 1
3205	HOXA9	homeobox A9
283212	KLHL35	kelch-like 35 (Drosophila)
51083	GAL	galanin prepropeptide
22955	SCMH1	sex comb on midleg homolog 1 (Drosophila)
1010	CDH12	cadherin 12, type 2 (N-cadherin 2)
11031	RAB31	RAB31, member RAS oncogene family
392225	LOC392225	phosducin-like 3 pseudogene
4025	LPO	lactoperoxidase
27159	CHIA	chitinase, acidic
123787	PRSS29P	protease, serine, 29 pseudogene

51130	ASB3	ankyrin repeat and SOCS box containing 3
885	CCK	cholecystokinin
645944	[No Symbol]	[No Name]
401973	OR10J6P	olfactory receptor, family 10, subfamily J, member 6 pseudogene
286053	NSMCE2	non-SMC element 2, MMS21 homolog (S. cerevisiae)
257106	ARHGAP30	Rho GTPase activating protein 30
114819	CROCCP3	ciliary rootlet coiled-coil, rootletin pseudogene 3
57453	DSCAML1	Down syndrome cell adhesion molecule like 1
127665	ZNF648	zinc finger protein 648
79083	MLPH	melanophilin
728392	LOC728392	hypothetical protein LOC728392
359710	C20orf185	chromosome 20 open reading frame 185
91746	YTHDC1	YTH domain containing 1
730043	LOC730043	hypothetical LOC730043
401705	[No Symbol]	[No Name]
79034	C7orf26	chromosome 7 open reading frame 26
10865	ARID5A	AT rich interactive domain 5A (MRF1-like)
130367	SGPP2	sphingosine-1-phosphate phosphatase 2
4137	MAPT	microtubule-associated protein tau
7455	ZAN	zonadhesin
1318	SLC31A2	solute carrier family 31 (copper transporters), member 2
2903	GRIN2A	glutamate receptor, ionotropic, N-methyl D-aspartate 2A
56961	SHD	Src homology 2 domain containing transforming protein D
136263	C7orf45	chromosome 7 open reading frame 45
158521	FMR1NB	fragile X mental retardation 1 neighbor
389328	[No Symbol]	[No Name]
100132822	LOC100132822	uncharacterized protein FLJ40521-like
11155	LDB3	LIM domain binding 3
23519	ANP32D	acidic (leucine-rich) nuclear phosphoprotein 32 family, member D
143379	C10orf82	chromosome 10 open reading frame 82
2867	FFAR2	free fatty acid receptor 2
5919	RARRES2	retinoic acid receptor responder (tazarotene induced) 2
65055	REEP1	receptor accessory protein 1
54073	[No Symbol]	[No Name]
8707	B3GALT2	UDP-Gal:betaGlcNAc beta 1,3-galactosyltransferase, polypeptide 2

1437	CSF2	colony stimulating factor 2 (granulocyte-macrophage)
26267	FBXO10	F-box protein 10
100132225	[No Symbol]	[No Name]
388588	LOC388588	hypothetical protein LOC388588
10058	ABCB6	ATP-binding cassette, sub-family B (MDR/TAP), member 6
57624	KIAA1486	KIAA1486
5126	PCSK2	proprotein convertase subtilisin/kexin type 2
6949	TCOF1	Treacher Collins-Franceschetti syndrome 1
140578	CHODL	chondrolectin
50506	DUOX2	dual oxidase 2
114196	SIGLEC24P	sialic acid binding Ig-like lectin 24, pseudogene
29062	WDR91	WD repeat domain 91
1046	CDX4	caudal type homeobox 4
642938	FAM196A	family with sequence similarity 196, member A
255104	TMCO4	transmembrane and coiled-coil domains 4
79677	SMC6	structural maintenance of chromosomes 6
100131617	LOC100131617	v-ets erythroblastosis virus E26 oncogene homolog 2 (avian) pseudogene
100131603	LOC100131603	zinc finger protein 93-like
100128272	[No Symbol]	[No Name]
388882	LOC388882	hypothetical LOC388882
343629	[No Symbol]	[No Name]
54437	SEMA5B	sema domain, seven thrombospondin repeats (type 1 and type 1-like), transmembrane domain (TM) and short cytoplasmic domain, (semaphorin) 5B
151242	PPP1R1C	protein phosphatase 1, regulatory (inhibitor) subunit 1C
3883	KRT33A	keratin 33A
113746	ODF3	outer dense fiber of sperm tails 3
266783	PSMD2P1	proteasome 26S subunit, non-ATPase, 2 pseudogene 1
1607	DGKB	diacylglycerol kinase, beta 90kDa
386726	FAM8A4P	family with sequence similarity 8, member A1 pseudogene
1953	MEGF6	multiple EGF-like-domains 6
152831	KLB	klotho beta
377047	PRSS45	protease, serine, 45
147646	LOC147646	hypothetical protein LOC147646
10458	BAIAP2	BAI1-associated protein 2
100132159	LOC100132159	hypothetical LOC100132159
1814	DRD3	dopamine receptor D3

191585	PLAC4	placenta-specific 4
400830	DEFB132	defensin, beta 132
440387	CTRB2	chymotrypsinogen B2
132014	IL17RE	interleukin 17 receptor E
326275	RPL12P7	ribosomal protein L12 pseudogene 7
131890	GRK7	G protein-coupled receptor kinase 7
4135	MAP6	microtubule-associated protein 6
122664	TPPP2	tubulin polymerization-promoting protein family member 2
10972	TMED10	transmembrane emp24-like trafficking protein 10 (yeast)
10022	INSL5	insulin-like 5
26281	FGF20	fibroblast growth factor 20
133748	RPL7AP32	ribosomal protein L7a pseudogene 32
442266	YAP1P1	Yes-associated protein 1 pseudogene 1
57758	SCUBE2	signal peptide, CUB domain, EGF-like 2
729229	[No Symbol]	[No Name]
3776	KCNK2	potassium channel, subfamily K, member 2
6557	SLC12A1	solute carrier family 12 (sodium/potassium/chloride transporters), member 1
285755	PPIL6	peptidylprolyl isomerase (cyclophilin)-like 6
100130647	[No Symbol]	[No Name]
220004	C11orf66	chromosome 11 open reading frame 66
648532	LOC648532	nuclear receptor binding factor 2 pseudogene
3938	LCT	lactase
26027	ACOT11	acyl-CoA thioesterase 11
100128661	[No Symbol]	[No Name]
100131700	[No Symbol]	[No Name]
153579	BTNL9	butyrophilin-like 9
440132	LOC440132	hCG1815504
84272	YIPF4	Yip1 domain family, member 4
80736	SLC44A4	solute carrier family 44, member 4
9125	RQCD1	RCD1 required for cell differentiation1 homolog (S. pombe)
6252	RTN1	reticulon 1
84083	ZRANB3	zinc finger, RAN-binding domain containing 3
648442	LOC648442	eukaryotic translation initiation factor 3, subunit I pseudogene
100128707	LOC100128707	MKI67 (FHA domain) interacting nucleolar phosphoprotein pseudogene
254050	LRRC43	leucine rich repeat containing 43

80267	EDEM3	ER degradation enhancer, mannosidase alpha-like 3
100132814	[No Symbol]	[No Name]
477	ATP1A2	ATPase, Na+/K+ transporting, alpha 2 polypeptide
503634	DUXAP5	double homeobox A pseudogene 5
176	ACAN	aggrecan
285220	EPHA6	EPH receptor A6
728318	KRTAP9-1	keratin associated protein 9-1
79644	SRD5A3	steroid 5 alpha-reductase 3
5530	PPP3CA	protein phosphatase 3, catalytic subunit, alpha isozyme
23765	IL17RA	interleukin 17 receptor A
283847	CCDC79	coiled-coil domain containing 79
79639	TMEM53	transmembrane protein 53
57595	PDZD4	PDZ domain containing 4
138009	DCAF4L2	DDB1 and CUL4 associated factor 4-like 2
57537	SORCS2	sortilin-related VPS10 domain containing receptor 2
54329	GPR85	G protein-coupled receptor 85
160492	IFLTD1	intermediate filament tail domain containing 1
81261	OR52H2P	olfactory receptor, family 52, subfamily H, member 2 pseudogene
2557	GABRA4	gamma-aminobutyric acid (GABA) A receptor, alpha 4
3336	HSPE1	heat shock 10kDa protein 1 (chaperonin 10)
3898	LAD1	ladinin 1
55112	WDR60	WD repeat domain 60
152100	CMC1	COX assembly mitochondrial protein homolog (S. cerevisiae)
64184	EDDM3B	epididymal protein 3B
643308	LOC643308	ribosomal protein L7 pseudogene
388007	SERPINA13	serpin peptidase inhibitor, clade A (alpha-1 antiproteinase, antitrypsin), member 13 (pseudogene)
5602	MAPK10	mitogen-activated protein kinase 10
100132919	LOC100132919	hypothetical protein LOC100132919
114821	SCAND3	SCAN domain containing 3
23371	TENC1	tensin like C1 domain containing phosphatase (tensin 2)
2125	EVPL	envoplakin
126859	AXDND1	axonemal dynein light chain domain containing 1
84539	MCHR2	melanin-concentrating hormone receptor 2
8000	PSCA	prostate stem cell antigen
158038	LINGO2	leucine rich repeat and Ig domain containing 2

4580	MTX1	metaxin 1
402152	RPL7L1P8	ribosomal protein L7-like 1 pseudogene 8
84976	DISP1	dispatched homolog 1 (Drosophila)
9794	MAML1	mastermind-like 1 (Drosophila)
100130391	YWHAQP5	YWHAQ pseudogene 5
134082	OR2AI1P	olfactory receptor, family 2, subfamily AI, member 1 pseudogene
6539	SLC6A12	solute carrier family 6 (neurotransmitter transporter, betaine/GABA), member 12
136227	EMID2	EMI domain containing 2
28513	CDH19	cadherin 19, type 2
646770	LOC646770	putative tripartite motif-containing protein 77-like
56981	PRDM11	PR domain containing 11
202309	GAPT	GRB2-binding adaptor protein, transmembrane
440330	LOC440330	hypothetical protein LOC440330
353135	LCE1E	late cornified envelope 1E
266722	HS6ST3	heparan sulfate 6-O-sulfotransferase 3
28316	CDH20	cadherin 20, type 2
100132762	LOC100132762	chromosome 17 open reading frame 80 pseudogene
131474	CHCHD4	coiled-coil-helix-coiled-coil-helix domain containing 4
56944	OLFML3	olfactomedin-like 3
25891	PAMR1	peptidase domain containing associated with muscle regeneration 1
9177	HTR3B	5-hydroxytryptamine (serotonin) receptor 3B
645251	CBX1P1	chromobox homolog 1 pseudogene 1
789	SLC25A20P1	solute carrier family 25 (carnitine/acylcarnitine translocase), member 20 pseudogene 1
341032	C11orf53	chromosome 11 open reading frame 53
166863	RBM46	RNA binding motif protein 46
143188	LOC143188	hypothetical LOC143188
442267	CCT7P1	chaperonin containing TCP1, subunit 7 (eta) pseudogene 1
9736	USP34	ubiquitin specific peptidase 34
728619	ASB9P1	ankyrin repeat and SOCS box containing 9 pseudogene 1
10529	NEBL	nebulette
100131093	[No Symbol]	[No Name]
344752	AADACL2	arylacetamide deacetylase-like 2
83868	TTTY13	testis-specific transcript, Y-linked 13 (non-protein coding)
57552	NCEH1	neutral cholesterol ester hydrolase 1
199731	CADM4	cell adhesion molecule 4

2742	GLRA2	glycine receptor, alpha 2
9687	GREB1	growth regulation by estrogen in breast cancer 1
80723	TMEM22	transmembrane protein 22
374860	ANKRD30B	ankyrin repeat domain 30B
84319	C3orf26	chromosome 3 open reading frame 26
6512	SLC1A7	solute carrier family 1 (glutamate transporter), member 7
100129514	[No Symbol]	[No Name]
3155	HMGCL	3-hydroxymethyl-3-methylglutaryl-CoA lyase
2845	GPR22	G protein-coupled receptor 22
134526	ACOT12	acyl-CoA thioesterase 12
431704	RGS21	regulator of G-protein signaling 21
128209	KLF17	Kruppel-like factor 17
375337	C3orf77	chromosome 3 open reading frame 77
345062	PRSS48	protease, serine, 48
100131909	[No Symbol]	[No Name]
1996	ELAVL4	ELAV (embryonic lethal, abnormal vision, Drosophila)-like 4 (Hu antigen D)
154288	C6orf221	chromosome 6 open reading frame 221
388324	INCA1	inhibitor of CDK, cyclin A1 interacting protein 1
9760	TOX	thymocyte selection-associated high mobility group box
442304	FTLP15	ferritin, light polypeptide pseudogene 15
3516	RBPJ	recombination signal binding protein for immunoglobulin kappa J region
121214	SDR9C7	short chain dehydrogenase/reductase family 9C, member 7
390206	RPS2P37	ribosomal protein S2 pseudogene 37
6538	SLC6A11	solute carrier family 6 (neurotransmitter transporter, GABA), member 11
56159	TEX11	testis expressed 11
50700	RDH8	retinol dehydrogenase 8 (all-trans)
728773	PABPC1P2	poly(A) binding protein, cytoplasmic 1 pseudogene 2
339209	[No Symbol]	[No Name]
5689	PSMB1	proteasome (prosome, macropain) subunit, beta type, 1
9133	CCNB2	cyclin B2
2566	GABRG2	gamma-aminobutyric acid (GABA) A receptor, gamma 2
525	ATP6V1B1	ATPase, H+ transporting, lysosomal 56/58kDa, V1 subunit B1
440125	HNRNPA1P30	heterogeneous nuclear ribonucleoprotein A1 pseudogene 30
84547	PGBD1	piggyBac transposable element derived 1
6707	SPRR3	small proline-rich protein 3

57818	G6PC2	glucose-6-phosphatase, catalytic, 2
348303	SELV	selenoprotein V
6511	SLC1A6	solute carrier family 1 (high affinity aspartate/glutamate transporter), member 6
4143	MAT1A	methionine adenosyltransferase I, alpha
375347	[No Symbol]	[No Name]
7060	THBS4	thrombospondin 4
5802	PTPRS	protein tyrosine phosphatase, receptor type, S
80851	SH3BP5L	SH3-binding domain protein 5-like
646804	LOC646804	alkylated DNA repair protein alkB homolog 8-like
199920	C1orf168	chromosome 1 open reading frame 168
100131394	[No Symbol]	[No Name]
4060	LUM	lumican
3240	HP	haptoglobin
100132788	[No Symbol]	[No Name]
9600	PITPNM1	phosphatidylinositol transfer protein, membrane-associated 1
646794	LOC646794	SNF8, ESCRT-II complex subunit, homolog (S. cerevisiae) pseudogene
100132592	[No Symbol]	[No Name]
91404	SESTD1	SEC14 and spectrin domains 1
100128111	[No Symbol]	[No Name]
1750	DLX6	distal-less homeobox 6
401044	FLJ40712	FLJ40712 protein
79290	OR13A1	olfactory receptor, family 13, subfamily A, member 1
730194	[No Symbol]	[No Name]
644714	LOC644714	hypothetical LOC644714
10077	TSPAN32	tetraspanin 32
4000	LMNA	lamin A/C
23426	GRIP1	glutamate receptor interacting protein 1
100129286	[No Symbol]	[No Name]
65268	WNK2	WNK lysine deficient protein kinase 2
220070	SHANK2-AS3	SHANK2 antisense RNA 3 (non-protein coding)
363	AQP6	aquaporin 6, kidney specific
9367	RAB9A	RAB9A, member RAS oncogene family
83850	ESYT3	extended synaptotagmin-like protein 3
100129153	[No Symbol]	[No Name]
195814	SDR16C5	short chain dehydrogenase/reductase family 16C, member 5

388946	LOC388946	transmembrane protein ENSP00000343375
647174	SERPINE3	serpin peptidase inhibitor, clade E (nexin, plasminogen activator inhibitor type 1), member 3
79155	TNIP2	TNFAIP3 interacting protein 2
392308	OR13E1P	olfactory receptor, family 13, subfamily E, member 1 pseudogene
56934	CA10	carbonic anhydrase X
5545	PRB4	proline-rich protein BstNI subfamily 4
729479	[No Symbol]	[No Name]
56287	GKN1	gastrokine 1
83876	MRO	maestro
100127992	[No Symbol]	[No Name]
7021	TFAP2B	transcription factor AP-2 beta (activating enhancer binding protein 2 beta)
201134	CEP112	centrosomal protein 112kDa
100129074	PRKRIRP5	protein-kinase, interferon-inducible double stranded RNA dependent inhibitor, repressor of (P58 repressor) pseudogene 5
9655	SOCS5	suppressor of cytokine signaling 5
219453	OR8K5	olfactory receptor, family 8, subfamily K, member 5
80028	FBXL18	F-box and leucine-rich repeat protein 18
282786	OR8G7P	olfactory receptor, family 8, subfamily G, member 7 pseudogene
79946	C10orf95	chromosome 10 open reading frame 95
100129767	LOC100129767	transmembrane protein 180-like
100129700	[No Symbol]	[No Name]
388121	TNFAIP8L3	tumor necrosis factor, alpha-induced protein 8-like 3
6442	SGCA	sarcoglycan, alpha (50kDa dystrophin-associated glycoprotein)
83734	ATG10	ATG10 autophagy related 10 homolog (S. cerevisiae)
122509	IFI27L1	interferon, alpha-inducible protein 27-like 1
729739	LOC729739	hypothetical LOC729739
3713	IVL	involucrin
56832	IFNK	interferon, kappa
2899	GRIK3	glutamate receptor, ionotropic, kainate 3
379034	FLJ10489	hypothetical protein FLJ10489
162966	ZNF600	zinc finger protein 600
676	BRDT	bromodomain, testis-specific
81126	OR4K16P	olfactory receptor, family 4, subfamily K, member 16 pseudogene
57144	PAK7	p21 protein (Cdc42/Rac)-activated kinase 7
11076	TPPP	tubulin polymerization promoting protein
146802	SLC47A2	solute carrier family 47, member 2

343990	C2orf55	chromosome 2 open reading frame 55
85455	DISP2	dispatched homolog 2 (Drosophila)
51421	AMOTL2	angiomotin like 2
26054	SENP6	SUMO1/sentrin specific peptidase 6
343641	TGM6	transglutaminase 6
646270	ELL2P1	elongation factor, RNA polymerase II, 2 pseudogene 1
9289	GPR56	G protein-coupled receptor 56
131909	FAM172BP	family with sequence similarity 172, member B pseudogene
131669	UROC1	urocanase domain containing 1
9076	CLDN1	claudin 1
100131431	[No Symbol]	[No Name]
114805	GALNT13	UDP-N-acetyl-alpha-D-galactosamine:polypeptide N-acetylgalactosaminyltransferase 13 (GalNAc-T13)
7100	TLR5	toll-like receptor 5
388336	SHISA6	shisa homolog 6 (Xenopus laevis)
146845	WDR16	WD repeat domain 16
23127	GLT25D2	glycosyltransferase 25 domain containing 2
400950	LOC400950	hypothetical LOC400950
728215	FAM155A	family with sequence similarity 155, member A
442181	RPSAP2	ribosomal protein SA pseudogene 2
151835	CPNE9	copine family member IX
2302	FOXJ1	forkhead box J1
401190	RGS7BP	regulator of G-protein signaling 7 binding protein
2564	GABRE	gamma-aminobutyric acid (GABA) A receptor, epsilon
119694	OR51F2	olfactory receptor, family 51, subfamily F, member 2
644604	EEF1A1P12	eukaryotic translation elongation factor 1 alpha 1 pseudogene 12
243	ALOX12P1	arachidonate 12-lipoxygenase pseudogene 1
2572	GAD2	glutamate decarboxylase 2 (pancreatic islets and brain, 65kDa)
116071	BATF2	basic leucine zipper transcription factor, ATF-like 2
55966	AJAP1	adherens junctions associated protein 1
1395	CRHR2	corticotropin releasing hormone receptor 2
3200	HOXA3	homeobox A3
400224	PLEKHD1	pleckstrin homology domain containing, family D (with coiled-coil domains) member 1
441631	TSPAN11	tetraspanin 11
100129386	[No Symbol]	[No Name]
1584	CYP11B1	cytochrome P450, family 11, subfamily B, polypeptide 1

55048	VPS37C	vacuolar protein sorting 37 homolog C (S. cerevisiae)
55084	SOBP	sine oculis binding protein homolog (Drosophila)
100128787	LOC100128787	similar to hCG1816414
23617	TSSK2	testis-specific serine kinase 2
285888	CNPY1	canopy 1 homolog (zebrafish)
646058	LOC646058	chromosome 4 open reading frame 27 pseudogene
441818	WBP11P1	WW domain binding protein 11 pseudogene 1
5506	PPP1R3A	protein phosphatase 1, regulatory (inhibitor) subunit 3A
7482	WNT2B	wingless-type MMTV integration site family, member 2B
399967	PATE2	prostate and testis expressed 2
51661	FKBP7	FK506 binding protein 7
390006	PPIAP19	peptidylprolyl isomerase A (cyclophilin A) pseudogene 19
26852	RNU2-5P	RNA, U2 small nuclear 5, pseudogene
144321	GLIPR1L2	GLI pathogenesis-related 1 like 2
64760	FAM160B2	family with sequence similarity 160, member B2
671	BPI	bactericidal/permeability-increasing protein
6334	SCN8A	sodium channel, voltage gated, type VIII, alpha subunit
374977	HEATR8	HEAT repeat containing 8
54991	C1orf159	chromosome 1 open reading frame 159
406906	MIR122	microRNA 122
100132713	LOC100132713	hypothetical protein LOC100132713
285676	ZNF454	zinc finger protein 454
4987	OPRL1	opiate receptor-like 1
100130986	[No Symbol]	[No Name]
53832	IL20RA	interleukin 20 receptor, alpha
64240	ABCG5	ATP-binding cassette, sub-family G (WHITE), member 5
100132978	ATP6V0CP1	ATPase, H+ transporting, lysosomal 16kDa, V0 subunit c pseudogene 1
8712	PAGE1	P antigen family, member 1 (prostate associated)
6236	RRAD	Ras-related associated with diabetes
58503	PROL1	proline rich, lacrimal 1
10487	CAP1	CAP, adenylate cyclase-associated protein 1 (yeast)
50604	IL20	interleukin 20
442153	PSMC1P11	proteasome (prosome, macropain) 26S subunit, ATPase, 1 pseudogene 11
66004	LYNX1	Ly6/neurotoxin 1
6100	RP9	retinitis pigmentosa 9 (autosomal dominant)

400451	FAM174B	family with sequence similarity 174, member B
9441	MED26	mediator complex subunit 26
136991	ASZ1	ankyrin repeat, SAM and basic leucine zipper domain containing 1
65267	WNK3	WNK lysine deficient protein kinase 3
22874	PLEKHA6	pleckstrin homology domain containing, family A member 6
391137	RPS15P3	ribosomal protein S15 pseudogene 3
2294	FOXF1	forkhead box F1
85379	KIAA1671	KIAA1671
7223	TRPC4	transient receptor potential cation channel, subfamily C, member 4
6340	SCNN1G	sodium channel, nonvoltage-gated 1, gamma
388135	C15orf59	chromosome 15 open reading frame 59
7325	UBE2E2	ubiquitin-conjugating enzyme E2E 2
2065	ERBB3	v-erb-b2 erythroblastic leukemia viral oncogene homolog 3 (avian)
729987	LOC729987	hypothetical protein LOC729987
401875	LOC401875	differentially expressed in FDCP 8 homolog (mouse) pseudogene
114818	KLHL29	kelch-like 29 (Drosophila)
1016	CDH18	cadherin 18, type 2
8120	AP3B2	adaptor-related protein complex 3, beta 2 subunit
388503	C3P1	complement component 3 precursor pseudogene
30818	KCNIP3	Kv channel interacting protein 3, calsenilin
379027	TSPY5P	testis specific protein, Y-linked 5, pseudogene
1828	DSG1	desmoglein 1
2813	GP2	glycoprotein 2 (zymogen granule membrane)
4004	LMO1	LIM domain only 1 (rhombotin 1)
401271	[No Symbol]	[No Name]
84643	KIF2B	kinesin family member 2B
6917	TCEA1	transcription elongation factor A (SII), 1
85449	KIAA1755	KIAA1755
6674	SPAG1	sperm associated antigen 1
84787	SUV420H2	suppressor of variegation 4-20 homolog 2 (Drosophila)
51196	PLCE1	phospholipase C, epsilon 1
80157	CWH43	cell wall biogenesis 43 C-terminal homolog (S. cerevisiae)
139334	IMPDH1P4	IMP (inosine monophosphate) dehydrogenase 1 pseudogene 4
727897	MUC5B	mucin 5B, oligomeric mucus/gel-forming
100128985	LOC100128985	nucleophosmin (nucleolar phosphoprotein B23, numatrin) pseudogene

25895	METTL21B	methyltransferase like 21B
6954	TCP11	t-complex 11 homolog (mouse)
400535	FLJ44674	FLJ44674 protein
2828	GPR4	G protein-coupled receptor 4
118424	UBE2J2	ubiquitin-conjugating enzyme E2, J2
94160	ABCC12	ATP-binding cassette, sub-family C (CFTR/MRP), member 12
399694	SHC4	SHC (Src homology 2 domain containing) family, member 4
280	AMY2B	amylase, alpha 2B (pancreatic)
84109	QRFPR	pyroglutamylated RFamide peptide receptor
442117	GALNTL6	UDP-N-acetyl-alpha-D-galactosamine:polypeptide N-acetylgalactosaminyltransferase-like 6
127833	SYT2	synaptotagmin II
644616	LOC644616	interleukin enhancer binding factor 2, 45kDa pseudogene
84812	PLCD4	phospholipase C, delta 4
285016	FAM150B	family with sequence similarity 150, member B
27185	DISC1	disrupted in schizophrenia 1
54806	AHI1	Abelson helper integration site 1
646174	C16orf90	chromosome 16 open reading frame 90
5792	PTPRF	protein tyrosine phosphatase, receptor type, F
728756	[No Symbol]	[No Name]
85407	NKD1	naked cuticle homolog 1 (Drosophila)
578	BAK1	BCL2-antagonist/killer 1
23527	ACAP2	ArfGAP with coiled-coil, ankyrin repeat and PH domains 2
3754	KCNF1	potassium voltage-gated channel, subfamily F, member 1
122449	RPS29P1	ribosomal protein S29 pseudogene 1
81242	OR52V1P	olfactory receptor, family 52, subfamily V, member 1 pseudogene
220164	DOK6	docking protein 6
11309	SLCO2B1	solute carrier organic anion transporter family, member 2B1
9700	ESPL1	extra spindle pole bodies homolog 1 (S. cerevisiae)
153562	MARVELD2	MARVEL domain containing 2
26468	LHX6	LIM homeobox 6
29070	CCDC113	coiled-coil domain containing 113
50939	IMPG2	interphotoreceptor matrix proteoglycan 2
285555	C4orf37	chromosome 4 open reading frame 37
5015	OTX2	orthodenticle homeobox 2
100130087	[No Symbol]	[No Name]

55258	THNSL2	threonine synthase-like 2 (S. cerevisiae)
646053	LOC646053	nuclear factor NF-kappa-B p100 subunit-like
146894	CD300LG	CD300 molecule-like family member g
100128442	LOC100128442	splicing factor, arginine/serine-rich 6 pseudogene
441609	YWHAZP9	tyrosine 3-monooxygenase/tryptophan 5-monooxygenase activation protein, zeta pseudogene 9
55691	FRMD4A	FERM domain containing 4A
4986	OPRK1	opioid receptor, kappa 1
100129702	LOC100129702	hypothetical protein LOC100129702
648947	LOC648947	aldo-keto reductase family 1, member C pseudogene
203447	NRK	Nik related kinase
100129620	LOC100129620	hypothetical LOC100129620
196385	DNAH10	dynein, axonemal, heavy chain 10
100129725	LOC100129725	PERP, TP53 apoptosis effector pseudogene
401052	LOC401052	hypothetical LOC401052
9479	MAPK8IP1	mitogen-activated protein kinase 8 interacting protein 1
403278	OR5K4	olfactory receptor, family 5, subfamily K, member 4
7092	TLL1	tolloid-like 1
154043	CNKSR3	CNKSR family member 3
248	ALPI	alkaline phosphatase, intestinal
283677	C15orf60	chromosome 15 open reading frame 60
196500	C12orf53	chromosome 12 open reading frame 53
100130522	LOC100130522	hypothetical LOC100130522
100132776	[No Symbol]	[No Name]
285613	RELL2	RELT-like 2
400941	FLJ42418	hypothetical LOC400941
5332	PLCB4	phospholipase C, beta 4
1	A1BG	alpha-1-B glycoprotein
2719	GPC3	glypican 3
100132641	BTF3P14	basic transcription factor 3 pseudogene 14
645940	LOC645940	heterogeneous nuclear ribonucleoprotein A1-like
57578	KIAA1409	KIAA1409
100129690	LOC100129690	RAD1 homolog (S. pombe) pseudogene
131096	KCNH8	potassium voltage-gated channel, subfamily H (eag-related), member 8
79712	GTDC1	glycosyltransferase-like domain containing 1
100129857	[No Symbol]	[No Name]

283310	OTOGL	otogelin-like
286499	FAM133A	family with sequence similarity 133, member A
353189	SLCO4C1	solute carrier organic anion transporter family, member 4C1
153	ADRB1	adrenergic, beta-1-, receptor
100131710	[No Symbol]	[No Name]
345016	LOC345016	pescadillo homolog 1, containing BRCT domain (zebrafish) pseudogene
85320	ABCC11	ATP-binding cassette, sub-family C (CFTR/MRP), member 11
642381	LOC642381	speckle-type POZ protein-like pseudogene
26533	OR10G3	olfactory receptor, family 10, subfamily G, member 3
158800	RHOXF1	Rhox homeobox family, member 1
80012	PHC3	polyhomeotic homolog 3 (Drosophila)
391075	GAPDHP23	glyceraldehyde 3 phosphate dehydrogenase pseudogene 23
50649	ARHGEF4	Rho guanine nucleotide exchange factor (GEF) 4
6928	HNF1B	HNF1 homeobox B
731940	[No Symbol]	[No Name]
55800	SCN3B	sodium channel, voltage-gated, type III, beta
338761	C1QL4	complement component 1, q subcomponent-like 4
55669	MFN1	mitofusin 1
100131117	LOC100131117	hypothetical LOC100131117
93622	LOC93622	hypothetical LOC93622
100130767	[No Symbol]	[No Name]
146212	KCTD19	potassium channel tetramerisation domain containing 19
2299	FOXI1	forkhead box I1
3764	KCNJ8	potassium inwardly-rectifying channel, subfamily J, member 8
2911	GRM1	glutamate receptor, metabotropic 1
255928	SYT14	synaptotagmin XIV
100128505	LOC100128505	hypothetical LOC100128505
3745	KCNB1	potassium voltage-gated channel, Shab-related subfamily, member 1
729406	LOC729406	protein phosphatase 1 regulatory subunit 14B-like
10376	TUBA1B	tubulin, alpha 1b
162333	10-Mar	membrane-associated ring finger (C3HC4) 10
1672	DEFB1	defensin, beta 1
83851	SYT16	synaptotagmin XVI
147658	ZNF534	zinc finger protein 534
100132631	[No Symbol]	[No Name]

100128764	PRKRIRP6	protein-kinase, interferon-inducible double stranded RNA dependent inhibitor, repressor of (P58 repressor) pseudogene 6
23245	ASTN2	astrotactin 2
4604	MYBPC1	myosin binding protein C, slow type
54829	ASPN	asporin
4224	MEP1A	meprin A, alpha (PABA peptide hydrolase)
646696	LOC646696	MAX-like protein X pseudogene
6242	RTKN	rhotekin
343515	ATP6V1E1P1	ATPase, H+ transporting, lysosomal 31kDa, V1 subunit E1 pseudogene 1
51412	ACTL6B	actin-like 6B
343450	KCNT2	potassium channel, subfamily T, member 2
100131031	LOC100131031	similar to hCG2041190
2641	GCG	glucagon
4850	CNOT4	CCR4-NOT transcription complex, subunit 4
284269	LOC284269	chromosome 9 open reading frame 86 pseudogene
148713	PTPRVP	protein tyrosine phosphatase, receptor type, V, pseudogene
51352	WT1-AS	WT1 antisense RNA (non-protein coding)
646430	LOC646430	amyloid P component, serum pseudogene
124817	CNTD1	cyclin N-terminal domain containing 1
729330	OC90	otoconin 90
29119	CTNNA3	catenin (cadherin-associated protein), alpha 3
131540	ZDHHC19	zinc finger, DHHC-type containing 19
117531	TMC1	transmembrane channel-like 1
90070	LACRT	lacritin
89	ACTN3	actinin, alpha 3
140739	UBE2F	ubiquitin-conjugating enzyme E2F (putative)
1290	COL5A2	collagen, type V, alpha 2
100129101	LOC100129101	hypothetical protein LOC100129101
285679	C5orf60	chromosome 5 open reading frame 60
390791	LOC390791	peptidyl-prolyl cis-trans isomerase A-like
9971	NR1H4	nuclear receptor subfamily 1, group H, member 4
414194	CCNYL2	cyclin Y-like 2
145957	NRG4	neuregulin 4
83938	C10orf11	chromosome 10 open reading frame 11
91862	MARVELD3	MARVEL domain containing 3
11285	B4GALT7	xylosylprotein beta 1,4-galactosyltransferase, polypeptide 7 (galactosyltransferase I)

192669	EIF2C3	eukaryotic translation initiation factor 2C, 3
84465	MEGF11	multiple EGF-like-domains 11
10814	CPLX2	complexin 2
326309	RPL7AP5	ribosomal protein L7a pseudogene 5
79785	RERGL	RERG/RAS-like
9590	AKAP12	A kinase (PRKA) anchor protein 12
56063	TMEM234	transmembrane protein 234
344653	LOC344653	ATP-binding cassette, sub-family F (GCN20), member 2 pseudogene
57188	ADAMTSL3	ADAMTS-like 3
130728	RPL7P13	ribosomal protein L7 pseudogene 13
56967	C14orf132	chromosome 14 open reading frame 132
387712	ENO4	enolase family member 4
404220	C6orf201	chromosome 6 open reading frame 201
3856	KRT8	keratin 8
100128282	[No Symbol]	[No Name]
246119	TTTY10	testis-specific transcript, Y-linked 10 (non-protein coding)
92292	GLYATL1	glycine-N-acyltransferase-like 1
3356	HTR2A	5-hydroxytryptamine (serotonin) receptor 2A
645357	LOC645357	kinesin family member pseudogene
22902	RUFY3	RUN and FYVE domain containing 3
130106	CIB4	calcium and integrin binding family member 4
203074	PRSS55	protease, serine, 55
1009	CDH11	cadherin 11, type 2, OB-cadherin (osteoblast)
8513	LIPF	lipase, gastric
2694	GIF	gastric intrinsic factor (vitamin B synthesis)
4640	MYO1A	myosin IA
284615	ANKRD34A	ankyrin repeat domain 34A
3850	KRT3	keratin 3
6376	CX3CL1	chemokine (C-X3-C motif) ligand 1
154313	C6orf165	chromosome 6 open reading frame 165
1091	CEACAMP2	carcinoembryonic antigen-related cell adhesion molecule pseudogene 2
11093	ADAMTS13	ADAM metallopeptidase with thrombospondin type 1 motif, 13
56156	TEX13B	testis expressed 13B
4653	MYOC	myocilin, trabecular meshwork inducible glucocorticoid response
100132358	EEF1A1P34	eukaryotic translation elongation factor 1 alpha 1 pseudogene 34

340156	MYLK4	myosin light chain kinase family, member 4
283446	MYO1H	myosin IH
100131598	[No Symbol]	[No Name]
93233	CCDC114	coiled-coil domain containing 114
84062	DTNBP1	dystrobrevin binding protein 1
100129644	[No Symbol]	[No Name]
100130275	LOC100130275	hypothetical LOC100130275
51121	RPL26L1	ribosomal protein L26-like 1
399965	LOC399965	hypothetical gene supported by AK128036
84448	ABLIM2	actin binding LIM protein family, member 2
3198	HOXA1	homeobox A1
56924	PAK6	p21 protein (Cdc42/Rac)-activated kinase 6
23114	NFASC	neurofascin
55321	C20orf46	chromosome 20 open reading frame 46
57214	KIAA1199	KIAA1199
9498	SLC4A8	solute carrier family 4, sodium bicarbonate cotransporter, member 8
6559	SLC12A3	solute carrier family 12 (sodium/chloride transporters), member 3
113691	MGC16703	tubulin, alpha pseudogene
2877	GPX2	glutathione peroxidase 2 (gastrointestinal)
55531	ELMOD1	ELMO/CED-12 domain containing 1
680	BRS3	bombesin-like receptor 3
6527	SLC5A4	solute carrier family 5 (low affinity glucose cotransporter), member 4
133874	C5orf58	chromosome 5 open reading frame 58
4319	MMP10	matrix metallopeptidase 10 (stromelysin 2)
6331	SCN5A	sodium channel, voltage-gated, type V, alpha subunit
5460	POU5F1	POU class 5 homeobox 1
10411	RAPGEF3	Rap guanine nucleotide exchange factor (GEF) 3
55282	LRRC36	leucine rich repeat containing 36
1620	DBC1	deleted in bladder cancer 1
8460	TPST1	tyrosylprotein sulfotransferase 1
100129287	[No Symbol]	[No Name]
645960	LOC645960	similar to hCG1644362
8796	SCEL	sciellin
100128676	LOC100128676	TBCC domain containing 1 pseudogene
374655	ZNF710	zinc finger protein 710

181	AGRP	agouti related protein homolog (mouse)
391428	SEPHS1P7	selenophosphate synthetase 1 pseudogene 7
8647	ABCB11	ATP-binding cassette, sub-family B (MDR/TAP), member 11
646543	LOC646543	hypothetical protein LOC646543
284338	PRR19	proline rich 19
100130995	LOC100130995	KRAB domain-containing protein ZNF321-like
65012	SLC26A10	solute carrier family 26, member 10
100129653	[No Symbol]	[No Name]
94233	OPN4	opsin 4
89884	LHX4	LIM homeobox 4
121256	TMEM132D	transmembrane protein 132D
10391	CORO2B	coronin, actin binding protein, 2B
91683	SYT12	synaptotagmin XII
647188	[No Symbol]	[No Name]
128674	PROKR2	prokineticin receptor 2
148066	ZNRF4	zinc and ring finger 4
283129	C11orf85	chromosome 11 open reading frame 85
10481	HOXB13	homeobox B13
100131552	[No Symbol]	[No Name]
10618	TGOLN2	trans-golgi network protein 2
100128134	[No Symbol]	[No Name]
8756	ADAM7	ADAM metallopeptidase domain 7
27115	PDE7B	phosphodiesterase 7B
284427	SLC25A41	solute carrier family 25, member 41
100131257	LOC100131257	zinc finger protein 655 pseudogene
728549	LOC728549	calcium binding protein P22 pseudogene
283208	P4HA3	prolyl 4-hydroxylase, alpha polypeptide III
57053	CHRNA10	cholinergic receptor, nicotinic, alpha 10
51560	RAB6B	RAB6B, member RAS oncogene family
183	AGT	angiotensinogen (serpin peptidase inhibitor, clade A, member 8)
143458	LDLRAD3	low density lipoprotein receptor class A domain containing 3
283652	SLC24A5	solute carrier family 24, member 5
23514	KIAA0146	KIAA0146
339403	RXFP4	relaxin/insulin-like family peptide receptor 4
130951	C2orf65	chromosome 2 open reading frame 65

100128076	LOC100128076	protein tyrosine phosphatase pseudogene
7288	TULP2	tubby like protein 2
114788	CSMD3	CUB and Sushi multiple domains 3
4883	NPR3	natriuretic peptide receptor C/guanylate cyclase C (atrionatriuretic peptide receptor C)
57554	LRRC7	leucine rich repeat containing 7
5794	PTPRH	protein tyrosine phosphatase, receptor type, H
150159	NHEDC1	Na+/H+ exchanger domain containing 1
100129633	[No Symbol]	[No Name]
285590	SH3PXD2B	SH3 and PX domains 2B
81833	SPACA1	sperm acrosome associated 1
221241	NCRNA00305	non-protein coding RNA 305
84189	SLITRK6	SLIT and NTRK-like family, member 6
65979	PHACTR4	phosphatase and actin regulator 4
10278	EFS	embryonal Fyn-associated substrate
645754	LOC645754	lectin, galactoside-binding, soluble, 9 pseudogene
10395	DLC1	deleted in liver cancer 1
219621	C10orf107	chromosome 10 open reading frame 107
57579	FAM135A	family with sequence similarity 135, member A
137902	PXDNL	peroxidasin homolog (Drosophila)-like
93649	MYOCD	myocardin
2327	FMO2	flavin containing monooxygenase 2 (non-functional)
23105	FSTL4	follistatin-like 4
1271	CNTFR	ciliary neurotrophic factor receptor
729417	[No Symbol]	[No Name]
83539	CHST9	carbohydrate (N-acetylgalactosamine 4-0) sulfotransferase 9
26280	IL1RAPL2	interleukin 1 receptor accessory protein-like 2
8749	ADAM18	ADAM metallopeptidase domain 18
4916	NTRK3	neurotrophic tyrosine kinase, receptor, type 3
100127920	[No Symbol]	[No Name]
92293	TMEM132C	transmembrane protein 132C
